# Downregulation of circLIFR exerts cancer-promoting effects on hepatocellular carcinoma *in vitro*

**DOI:** 10.3389/fgene.2022.986322

**Published:** 2022-09-12

**Authors:** Jingzhang Ji, Jialyu Tang, Ping Ren, Wenpin Cai, Meina Shen, Qiunan Wang, Xiaoyun Yang, Wei Chen

**Affiliations:** ^1^ Center for Laboratory Medicine, The First Affiliated Hospital of Xi’an Jiaotong University, Xi’an, China; ^2^ Zhejiang Provincial Key Laboratory of Medical Genetics, Key Laboratory of Laboratory Medicine, Ministry of Education, School of Laboratory Medicine and Life Sciences, Wenzhou Medical University, Wenzhou, China; ^3^ Department of Intervention, Wen Zhou Central Hospital, Wenzhou, China; ^4^ Department of Laboratory Medicine, Wen Zhou Traditional Chinese Medicine Hospital, Wenzhou, China; ^5^ Department of Laboratory Medicine, Ningbo Medical Center Lihuili Hospital, Ningbo, China

**Keywords:** hepatocellular carcinoma, circLIFR, metastasis, TBK1, CircRNAs

## Abstract

Hepatocellular carcinoma (HCC) is one of the most fatal malignant tumors worldwide. Circular RNAs (circRNAs) are a special type of RNA that lacks the 5′ and 3’ ends. The functional roles of circRNAs in HCC remain largely unknown. Using high-throughput sequencing, we found several differentially expressed circRNAs in HCC tissues compared with nearby normal tissues. Among them, circRNA derived from the LIFR gene, named circLIFR, was significantly downregulated in HCC. Intriguingly, circLIFR overexpression in SK-Hep-1 cells promoted cell growth and invasion. RNA pull-down and mass spectrometry detection revealed circLIFR interacting with TANK binding kinase 1 (TBK1). Anti-TBK1 RIP confirmed the interaction between circLIFR and TBK1. TBK1 is a serine/threonine kinase that regulates several signaling pathways, including the NF-κB pathway. TBK1 inhibitors inhibit NF-κB activation. Overexpression of circLIFR overcame the in-hibitory function of TBK1, resulting in the upregulation of several genes, including MMP13, MMP3, VEGF, and MAPK. This study shows that the downregulation of circLIFR in HCC has a can-cer-promoting effect by interacting with TBK1 to promote the activation of downstream NF-κB pathway genes related to cell proliferation, migration, and invasion. This novel finding reveals the diversity of circRNA functions in HCC and provides novel insights into the role of circRNAs.

## Introduction

The patocellular carcinoma (HCC) is the fifth most common malignancy world-wide, accounting for 70%–90% of all primary liver cancers (Li et al., 2020). The average survival times of patients with early liver cancer and advanced liver cancer receiving less spe-cialized treatment are 6–9 months and 1–2 months, respectively. More than 60% of pa-tients are diagnosed withadvanced liver cancer that has metastasized, resulting in an overall 5-year survival rate of <16% (Liu et al., 2020; Vibert et al., 2020). Few HCC biomarkers have sufficient diag-nostic performance for early HCC(Liu et al., 2020). Surgical resection of HCC has a 10-year recur-rence-free rate of approximately 22–25% compared with 50–70% following liver transplantation (Vibert et al., 2020).

Circular RNAs (circRNAs) are special closed RNAs that lack the 5′ and 3’ ends (Zhou and Yu, 2017). CircRNAs show higher stability than linear RNAs because of their single-stranded closed circular structures (Guarnerio et al., 2016; Sun et al., 2020). CircRNAs are usually generated by the back-splicing of mRNA gene exons. The function of circRNAs mainly includes but is not limited to: 1) acting as a sponge of miRNAs, 2) interacting with proteins, 3) regulating translation of proteins, and 4) regulating the transcription of linear RNAs. Some circRNAs play several roles (Li et al., 2019).

CircRNAs are closely linked to liver cancer and can influence the proliferation, invasion, and death of liver cells, being potential biomarkers and therapeutic targets. Previous research has shown that circRNAs in liver cancer are closely linked to the development of HCC(Cheng et al., 2020a; Cheng et al., 2020b; Zhang et al., 2020). CircRNAs are differentially expressed in this disease, such as circ-ITCH, circ-MALAT1, etc. (Chen et al., 2020; Wu et al., 2020). The specific functional mechanism of circRNAs in HCC is not yet clear.

A circRNA (circBase ID: hsa_circ_0072309) derived from the LIFR gene, named circLIFR, has been described to, when overexpressed, promote cell proliferation, inva-sion, and migration. CircLIFRs have been reported in several studies. Yan et al. re-ported that circLIFR inhibits the proliferation and invasion of breast cancer cells by targeting miR-492 (Yan et al., 2019). Chen et al. found that circLIFR inhibits cancer progression by sponging miR-100 in renal carcinoma cell lines (Chen et al., 2019). Yuan et al. reported that circLIFR inhibits proliferation and invasion of glioblastoma (Yuan et al., 2021). Zhao et al. revealed that cir-cLIFR promotes apoptosis in ischemic stroke by sponging miR-100 (Zhao et al., 2020). Zhang reported that circLIFRis downregulated in bladder cancer (Zhang et al., 2021).

In this study, we investigated the effects of circLIFRon HCC cells. We constructed plasmid vectors to investigate the function and effect ofcircLIFR on HCC at the cellular level. The interaction between circLIFR and TBK1 was also verified to further study the mechanism of circLIFR on HCC cells. Our results pro-vide novel insights into the role of circRNAs in this disease.

## Results

### CircRNA was differentially expressed inHuman normal and HCC samples

**FIGURE 1:**
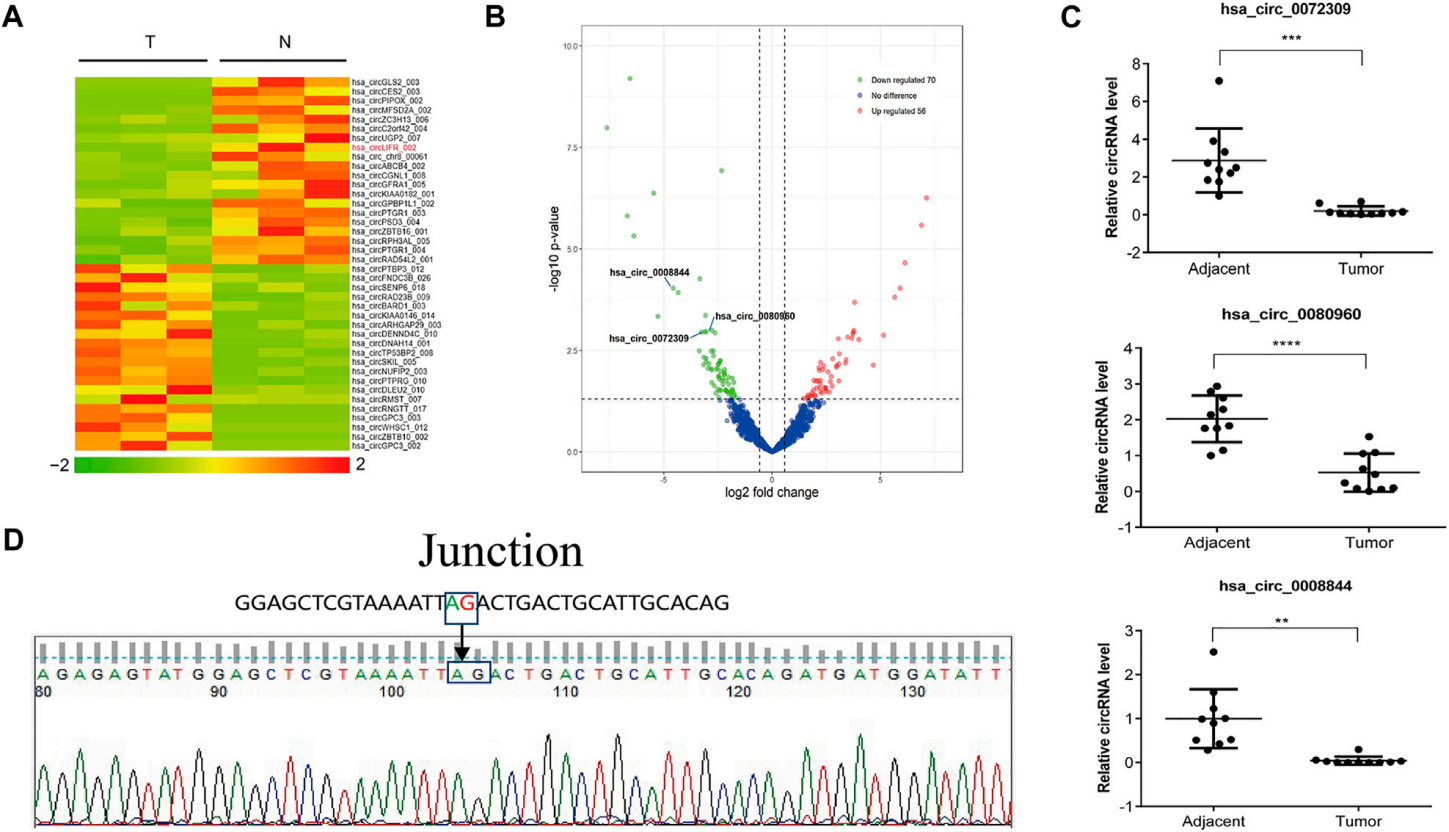
Identification of circLIFR by RNA-seq in liver cancer tissues.**(A)** Heat map of circRNA expression fold-change. Red indicates a higher fold-change; **(B)**Volcano plots of differentially expressed circRNAs in the three pairs of matched liver cancer tissues and adjacent normal tissues; **(C)** Expression levels of circRNA in paired HCC and adjacent tissues collected from 10 HCC patients were measured by qRT-PCR assay; **(D)** Sanger sequencing of circLIFR.

CircRNAs are expressed in atissue/disease-specific manner. To identify differen-tially expressed circRNAs in HCC, high-throughput RNA sequencing (RNA-seq) was per-formed on six tissue samples (three cancerous tissues and three paired normal tis-sues from adjacent sites). The results revealed that 56 circRNAs were upregulated and 70 circRNAs downregulated in cancerous tissues compared to normal tissues (Figures 1A,B). The top 10 significantly different circRNAs are shown (Table 1). Among these differen-tially expressed circRNAs, we selected three downregulated circRNAs and verified their expression in 10 other HCC and paired normal liver tissues. The results show that all these threecircRNAs were significantly downregulated in HCC tissues compared to their paired adjacent normal tissues (Figure 1C). In particular, circRNA from the LIFR gene (hsa_circ_0072309,circLIFR) was significantly downregulated in HCC samples and SK-hep-1 cells. Sanger sequencing revealed the interface position se-quence of cir-cLIFR (Figure 1D).

**TABLE 1:** Top 10 significantly differentially expressed circRNAs.

circRNA_ID	Hsa_circbase_ID	flodchange	PValue	Gene
chr12_97886238_97954825_+	Hsa_circ_0099634	25.43660308	0.007251484	RMST
chr1_223991875_223994649_-	Hsa_circ_0003914	13.17209606	0.001241027	TP53BP2
chr1_94667275_94697199_-	Hsa_circ_0013225	10.65415018	0.005344619	ARHGAP29
chr2_215632205_215661841_-	Hsa_circ_0058051	8.61555554	0.003816979	BARD1
chr10_126631025_126631876_+	Hsa_circ_0000268	5.155888499	0.007610163	ZRANB1
chr1_98144650_98165103_-	Hsa_circ_0004161	-5.4852015	0.015959556	DPYD
chr7_87068982_87069718_-	Hsa_circ_0080960	−8.296773227	0.001083509	ABCB4
chr5_38523520_38530768_-	Hsa_circ_0072309	−8.654777219	0.001098483	LIFR
chr2_70406663_70409129_-	Hsa_circ_0055113	−10.14342899	0.000054	C2orf42
chr1_40422758_40424497_+	Hsa_circ_0008844	−23.77657457	9.19E-05	MFSD2A

### Overexpression of circLIFR promotes cell growth and metastasis

LIFR acts as a tumor suppressor in several human cancers. To investigate the function of circLIFR in HCC, we constructed an overexpression vector for circLIFR and transfected Sk-Hep-1 HCC cells to evaluate cell growth and migration.

**FIGURE 2:**
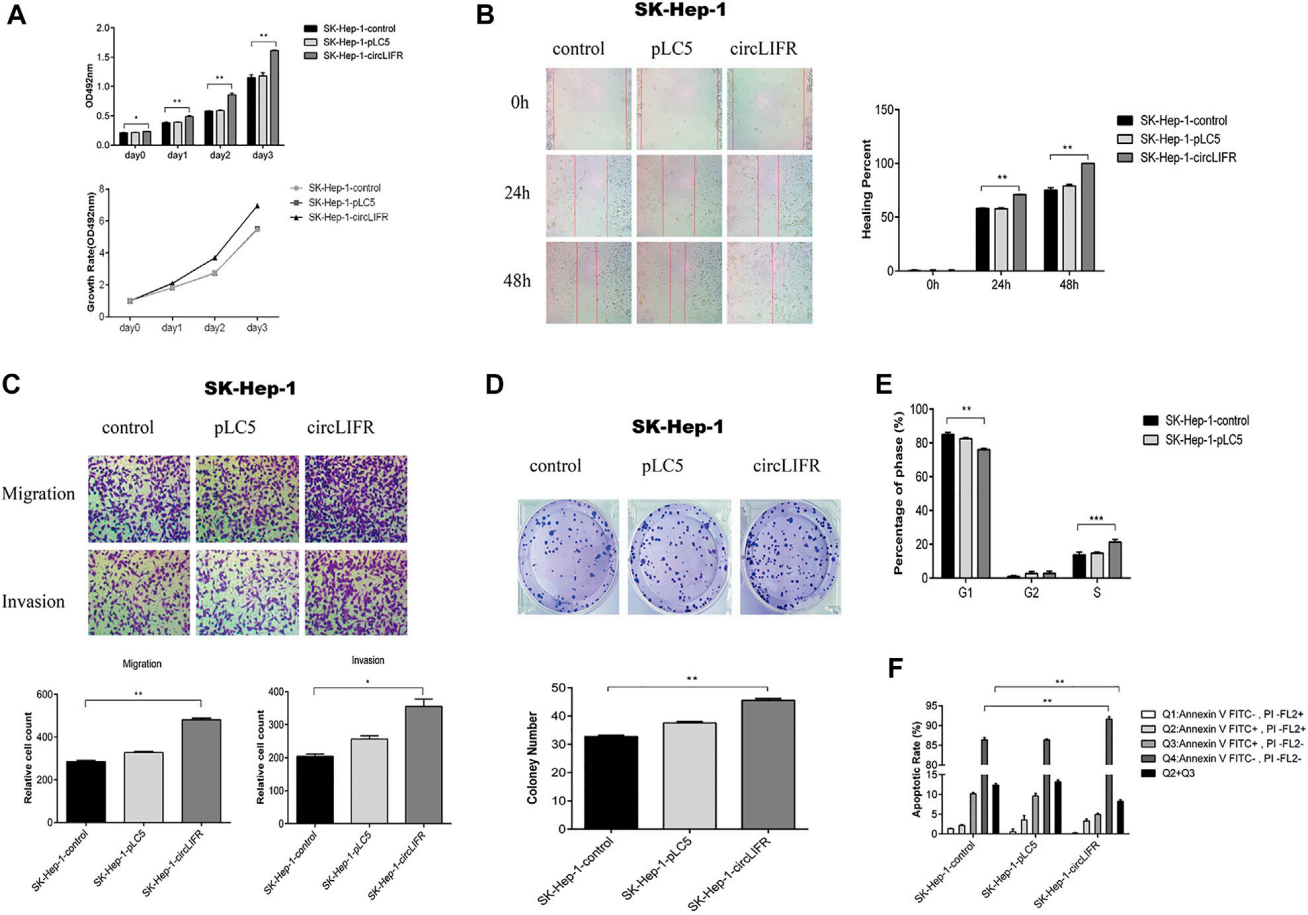
CircLIFR promotes proliferation, migration, and invasion in SK-Hep-1 cells.**(A)** The proliferation of SK-Hep-1 cells transfected with control overexpression circLIFR detected by CCK-8 assays; **(B)** Wound-healing assay for migration ability of SK-Hep-1 cells after overexpression of circLIFR; Original magnification: ×10. **p < 0.01 **(C)** Transwell assay shows that the overexpression circLIFR promoted metastasis and invasion of SK-Hep-1 cells. Original magnification: ×10. *p < 0.05; **p < 0.01; **(D)** Colony forming ability of SK-Hep-1 hepatoma cells transfected with circLIFR in the control group. *p < 0.05,**p < 0.01 (Student’s t-test); **(E)** Cell cycle assay for SK-Hep-1 cells transfected with control overexpression of circLIFR; **(F)** Flow cytometry to detect the effect of overexpression of circLIFR in the control group on apoptosis and renewal of SK-Hep-1 cells.

Intriguingly, overexpression of circLIFR promoted cell growth in Sk-Hep-1 (Figure 2A) and HepG2 cells (Supplementary Figure S1A). Wound-healing and transwell assays show that overexpression of circLIFR promoted migration and invasion of Sk-Hep-1 cells (Figures 2B,C) and HepG2 cells (Supplementary Figures S1B,C). Colony formation assays also show that circLIFRpromoted colony formation in Sk-Hep-1 cells (Figure 2D) and HepG2 cells (Supplementary Figure S1D). Cell cycle and apoptosis detec-tion also showsthat overexpression of circLIFR promoted cell progression through the cell cycle and reduced the apoptosis rate (Figures 2E,F) and HepG2 (Supplementary Figures S1E,F). The cell cycle and apoptosis analysis results are shown in Supplementary Figure S2.

### circLIFR bind to TBK1

**FIGURE 3:**
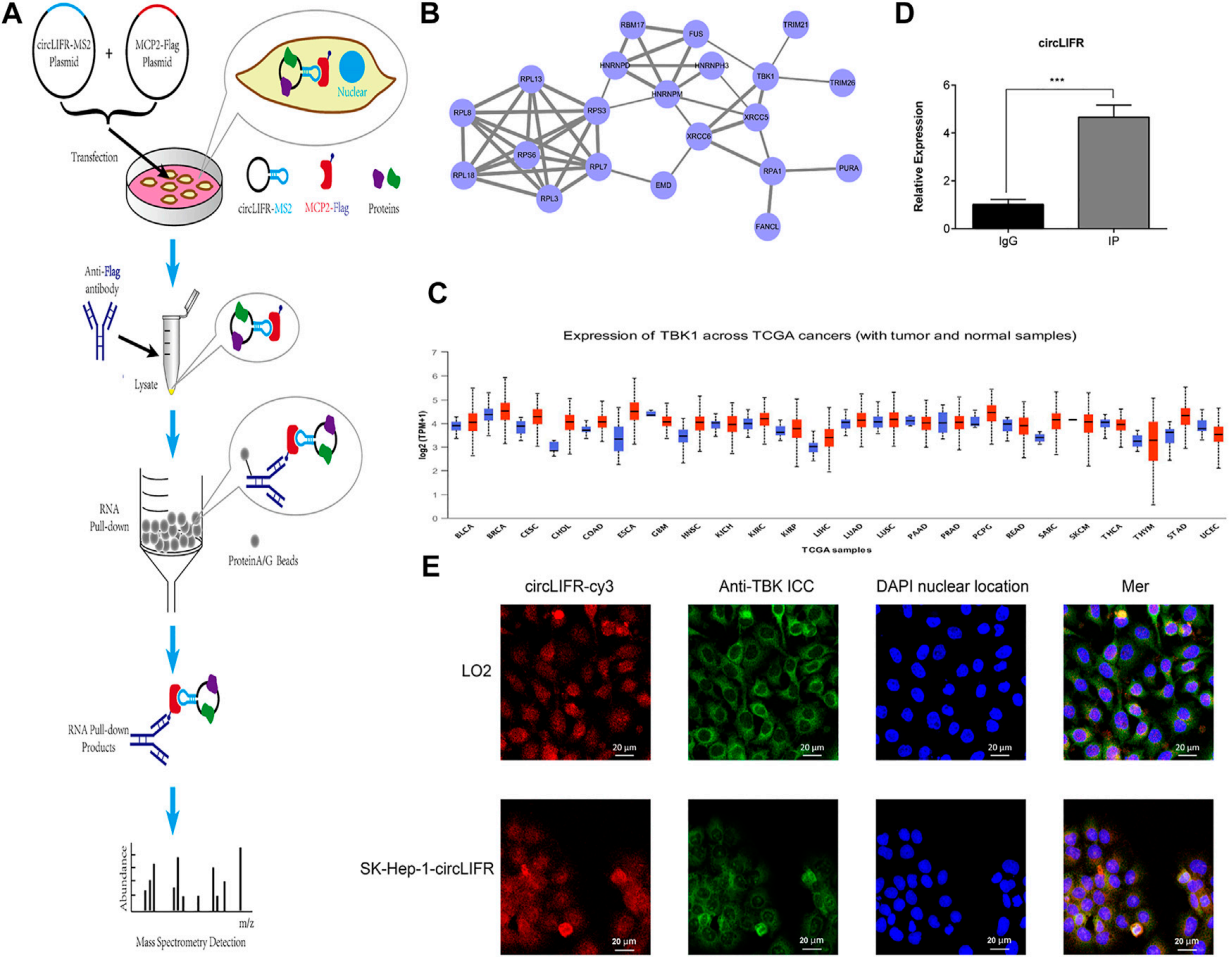
RNA pulldown for CircLIFR. **(A)** Schematic diagram for pulldown detection of the circLIFR gene; **(B)** PPI analysis of the detected proteins by mass spectrometry; **(C)** pan-cancer expression level of TBK1 in TCGA cohort, (obtained from the UALCAN website); **(D)** RIP test verified that overexpression of circLIFR significantly promoted specific binding between TBK1 proteins.*p < 0.05; **p < 0.01; (Student’s t-test); **(E)** FISH and IF detection for circLIFR and TBK1 in LO2 cells that enhanced expressing circLIFR.

All Functional analysis of the endogenous low-expression circLIFR shows that it had a tumor-promoting effect. CircRNAs are non-coding RNAs that often function by interacting with proteins. To explore the cancer-promoting mechanism of circLIFR, capture analysis of interacting proteins, including TBK1, was performed by RNA pull-down (Figure 3A). We identified 10 interacting proteins, including TBK1 (Table 2). The protein interaction network for these captured proteins was predicted by PPI, and the results show that TBK1 was the center of the protein interaction network (Figure 3B). TBK1 is often highly expressed in tumors, and TCGA data analysis shows that the ex-pression of TBK1 in most tumor types was higher than in paracancerous controls (Figure 3C). Therefore, we further verified and analyzed the interaction between circLIFR and TBK1 and determined whether this interaction mediates the tumor-promoting effect of circLIFR.

**TABLE 2:** Potential proteins that bind to circLIFR as predicted by pulldown assays.

prot_hit_num	prot_acc	prot_desc
11	sp|Q14764|MVP_HUMAN	Major vault protein OS = Homo sapiens OX = 9606 GN = MVP PE = 1 SV = 4
32	sp|Q96I25|SPF45_HUMAN	Splicing factor 45 OS = Homo sapiens OX = 9606 GN = RBM17 PE = 1 SV = 1
54	sp|P26373|RL13_HUMAN	60S ribosomal protein L13 OS = Homo sapiens OX = 9606 GN = RPL13 PE = 1 SV = 4
87	sp|A0A0C4DH55|KVD07_HUMAN	Immunoglobulin kappa variable 3D-7 OS = Homo sapiens OX = 9606 GN = IGKV3D-7 PE = 3 SV = 5
116	sp|Q5QNW6| _HUMAN	Histone H2B type 2-F OS = Homo sapiens OX = 9606 GN = HIST2H2BF PE = 1 SV = 3
48	sp|P1812 H2B2F 4|RL7_HUMAN	60S ribosomal protein L7 OS = Homo sapiens OX = 9606 GN = RPL7 PE = 1 SV = 1
34	sp|Q9UHD2|TBK1_HUMAN	Serine/threonine-protein kinase TBK1 OS = Homo sapiens OX = 9606 GN = TBK1 PE = 1 SV = 1
103	sp|P68871|HBB_HUMAN	Hemoglobin subunit beta OS = Homo sapiens OX = 9606 GN = HBB PE = 1 SV = 2
39	sp|P35637|FUS_HUMAN	RNA-binding protein FUS OS = Homo sapiens OX = 9606 GN = FUS PE = 1 SV = 1
47	sp|P19474|RO52_HUMAN	E3 ubiquitin-protein ligase TRIM21 OS = Homo sapiens OX = 9606 GN = TRIM21 PE = 1 SV = 1

The interaction between endogenous TBK1 and circLIFR was verified by An-ti-TBK1RIP assays (Figure 3D). FISH and IF colorization analyses also confirmed the colo-calization of circLIFR and TBK1 (Figure 3E). Through immunofluorescence fluorescence *in situ* hybridization (IF-FISH), we can perform co-localization and qualitative analysis of nucleic acid molecules and proteins in tissues and cells, which can be used to indi-cate the interaction between nucleic acids and proteins from the point of view ofco-localization. Consequently, we detected the interaction between circLIFR and TBK1 protein by FISHwith a red fluorescent cy3 probe, IF antigen localization with green fluorescent substance-labeled anti-TBK1 protein antibody, and nuclear locali-za-tion by DAPI staining. The results show that in LO2 cells and SK-Hep-1 cells over-ex-pressing circLIFR, the localization of circLIFR and TBK1 protein was basically the same, suggesting that there is an interaction between circLIFR and TBK1 protein.

### circLIFR-TBK1 inhibits NF-κB activity and promote downstream gene expression

The nuclear factor-κB (NF-κB) transcription factor family has been considered the central mediator of the inflammatory process and linked to the cancer development (DiDonato et al., 2012). Moreover, TBK1 also plays an importantrole in the NF-κB signaling pathway (Kim et al., 2010).

**FIGURE 4:**
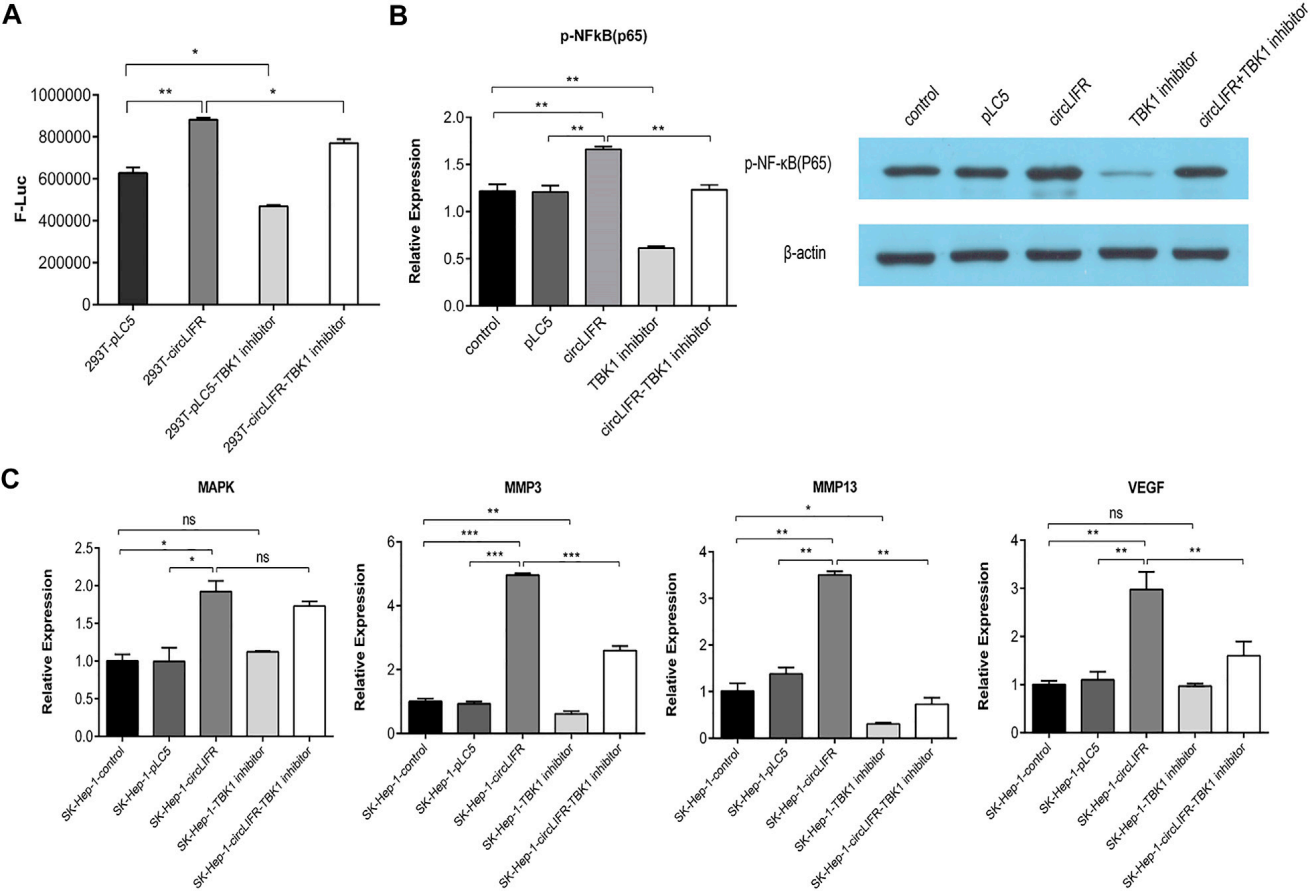
Interaction between circLIFR and TBK1 protein promotes NFκB signal pathway activation and regulates the expression of genes related to proliferation and migration. **(A)** NF-κB luciferase reporter system shows that overexpression of circLIFR counteract the inhibitory effect of TBK1 inhibitor and activated the NF-κB pathway to affect the activity of NF-κB by binding to TBK1; **(B)** Western blotting analysis suggests that circLIFR overexpression activated the NF-κB pathway; **(C)** qRT-PCR analysis shows that the overexpression of circLIFR interacted with TBK1 to affect the levels of oncogenes downstream of NF-κB.

NF-κB is an important downstream target of TBK1. To verify the function of cir-cLIFR binding to TBK1, a promoter-reporter gene that binds to NF-κB was investigated. circLIFRbinds to TBK1 and regulates the NF-κB pathway. The results show that over-expression of circLIFR promoted the expression of the NF-κB reporter gene, indicating that circLIFR promotes NF-κB activity (Figure 4A). The addition of TBK1 inhibitors re-duced the expression of the NF-κB reporter gene. When circLIFR was overexpressed and TBK1 was suppressed simultaneously, the effect of the NF-κB reporter gene was compensated (Figure 4A).

NF-κB subunit p65 is a known target of TBK1. To analyze the regulatory role of circLIFR-TBK1 in NF-κB, western blot analysis was used to detect the phosphorylation state of p65. The results show that circLIFR overexpression promotes p65phosphorylation, whereas the TBK1 inhibitor inhibits the phosphorylation of p65. Overexpression of circLIFR also reversed the effects of the TBK1 inhibitor on p65 phosphorylation. These results suggest that circLIFR-TBK1 regulates the activation of the NF-κB pathway, whereas circLIFR reverses the effect of the TBK1 inhibitor (Figure 4B).

NF-κB is an important transcription factor that mediates the expression of many genes involved in cell proliferation, migration, and invasion. MMP13, MMP3, VEGF, and MAPK were used as target molecules in qPCR detection to show the effect of cir-cLIFR-TBK1 on downstream genes. The results show that Overexpression of circLIFR enhanced the expression of several downstream genes, whereas TBK1 inhibitors downregulated these genes. Overexpression of circLIFR reversed the effect of the TBK1 inhibitor (Figure 4C).

## Discussion

CircRNAs play an important role in the metastasis and invasion of several cancer cells (Kristensen et al., 2018). A study reported that circFNDC3B is significantly downregulated in BCa tissues and is associated with pathological T staging, grading, lymphatic invasion, and overall patient survival (Liu et al., 2018). Other studies have reported that circ_0072309 inhibits the progression of NSCLC by blocking miR-580-3p (Yuan et al., 2021). Tao C. et al. reported that the circular RNA hsa-circ-0072309 plays an anti-tumor role by sponging miR-100 through deactivation of the PI3K/AKT and mTOR pathways in renal carcinoma cell lines (Chen et al., 2019). These findings indicate that circRNAs play an important regulatory role in tumor-igenesis. The present study found that circLIFR was downregulated in HCC. Our find-ings reveal that circLIFR functions as a tumor promoter in HCC by promoting cell pro-liferation, migration, and invasion, contrary to expectations from the sequences ob-tained. Our study findings are also contrary to an earlier study by Qin et al., which re-ported that LIFR functions as a metastasis suppressor in HCC by negatively regulating the phosphoinositide 3-kinase/AKT pathway (Luo et al., 2015).

The expression of cirLIFR *in vivo* in paracancerous tissues was higher than that in cancerous tissues. The regulatory mechanism of circLIFR in cell proliferation in para-cancerous tissues remains unclear. The LIFR gene is significantly underexpressed in liver cancer and has a significant tumor suppressor effect in liver cancer. The expres-sion of circLIFR decreases with a decrease in transcription factors.

TBK1 is often highly expressed in tumors, and our TCGA data analysis shows that the expression of TBK1 in most tumor types was higher than in paracancerous controls (Figure 3C). Anti-TBK1RIP assays were used to determine the interaction between endog-enous TBK1 and circLIFR. TBK1 regulates gene transcription and immune signaling pathways by regulating downstream target molecules such as NF-κB and IRF3. These results suggest that there is an interaction between circLIFR and TBK1, which may further regulate the NF-κB pathway. Abnormal activation of the NF-κBsignaling pathway is related to the occurrence and development of cancer and affects genes re-lated to cell proliferation, migration, and invasion (Sims et al., 2013; Bennett et al., 2017). This mechanism may ex-plain the increased cell proliferation and migration following circLIFR overexpression observed.

This study also found that downregulation of circLIFR in HCC promoted tumor proliferation, migration, and invasion. Further research in this area is required.

## Materials and methods

### Human normal and HCC samples

Thirteen pairs of human primary HCC and paired adjacent non-tumorous liver tissues were collected from the Wen Zhou Traditional Chinese Medicine Hospital. Human materials were obtained with informed consent, and the study was approved by the Wenzhou Hospital of Traditional Chinese Medicine Ethics Committee. Three paired HCC and non-tumorous liver tissues were used for high-throughput RNA se-quencing, and the other 10 paired HCC and non-tumorous liver tissues were used for QPCR detection of selected circRNAs.

### High throughput RNA-seq and sanger sequencing

Total RNA from three paired HCC and adjacent noncancerous liver tissues was extracted using TRIZOL (Life technologies). Ribosome-depleted RNA samples were fragmented and used for first- and second-strand complementary DNA (cDNA) syn-thesis with random hexamer primers. PCR was used for amplification. The purification and screening of a suitably sized library were carried out. Finally, the library was se-quenced using X. A standard of fold-change ≥ 1.5 or ≤ −1.5 was set as the cut-off value. Significant differential expression of circRNAs was observed.

### Quantitative real-time polymerase chain reaction (qRT-PCR)

Total RNA was extracted from tissues and cultured cells using TRIZOL reagent (Life Technologies) following the manufacturer’s instructions. Total RNA was reverse transcribed to cDNA using specific primers in line with the Geneseed® II First Strand cDNA Synthesis Kit (Geneseed). qRT-PCR was performed using the Geneseed® qPCR SYBR® Green Master Mix (Geneseed). The primers used were as follows:

GAPDH forward: AGA​AGG​CTG​GGG​CTC​ATT​TG;

Reverse: GCA​GGA​GGC​ATT​GCT​GAT​GAT;

circLIFR forward: 5′-GGA​GCT​CGT​AAA​ATT​AGA​CTG-3'.

Reverse: 5′-AAT​GTT​GAT​AAC​AGC​CAC​TGG​A-3'.

### Cell culture

HCC cell lines (SK-Hep-1, HepG2) and the human normal liver cell line (LO2) were purchased from ATCC and cultured in Roswell Park Memorial Institute-1640 medium (RPMI; Invitrogen, CA, United States).

### Vector construction

To recapitulate circRNA, the 3276bp sequence of LIFR was amplified using Pri-merSTAR Max DNA Polymerase Mix (Takara). The PCR products were inserted into the pCDNA3.1 (+)-IRES-EGFP vector.

### Cell transfection

Human HCC cell lines SK-hep-1 and HepG2 were inoculated into two six-well plates. Plasmids and Lipofectamine 2000 (3 µg plasmid in 4 μL) were mixed at a cell confluence of 70–90% and added to serum-free medium into cell samples. Cell trans-fection was verified using PCR. The extracted RNA was detected using NanoDropND2000 and 1% agarose gel electrophoresis.

### Wound-healing assay

Cells were seeded onto a six-well plate after 48 h of transfection. Pipette tips (10 μl) were used to make cell scratches perpendicular to plates. The old culture medium was removed, the cells were washed thrice with sterile PBS, damaged cells were re-moved, and a new culture medium was added. The cell scratch mobility was observed and calculated at 0, 24, and 48 h.

### Cell proliferation

Cells (1 × 10^4^) were seeded 48 h after transfection. The CCK-8 solution (10 μL) was added to each well at (24, 48, and 72 h) and incubated for 2 h. The absorbance was read at 450 nm using a microplate reader (BioTek). The following formulas were used for the calculation of:

Cell proliferation rate: Proliferation rate (same treatment sample) = Day NOD value/Day0 average OD value.

Cell inhibition rate formula: Inhibition rate (at the same time point) = (1-experimental group proliferation rate)/control group average proliferation rate.

### Transwell assay

After 48 h, the transfected cells were digested with trypsin-EDTA solution and resuspended in serum-free DMEM after centrifugation. Next, 100 µl of the cell suspen-sion was added to the upper part of the transwell chamber. The lower chamber was filled with 600 μm complete DMEM medium. After 24 h, the filters were fixed with 4% paraformaldehyde and stained with crystal violet. The cells that passed through the lower chamber were counted.

### Cell apoptosis assay

The apoptotic rate of the modulated cells was determined. The Annexin V-fluorescein isothiocyanate Apoptosis Detection Kit (KeyGen, Nanjing, China) was used to perform annexin V/propidium iodide staining. Flow cytometry was used to de-tect red fluorescence at 488 nm excitation wavelength. The results were analyzed using the cell cycle simulation software ModFit.

### Colony formation assay

Approximately 200 transfected cells were seeded per well in six-well plates, incu-bated for 10–15 days. Formed colonies were stained with 0.1% crystal violet solution and counted.

### RNA pull-down

MS2 was then inserted into circLIFR (circLIFR-MS2). circLIFR-MS2 was con-structed into the pCDNA3.1 (+)-IRES-EGFP vector. MS2 binding protein, mCP2 was cloned into the TetOn3G vector and fused with Flag and mCherry (Flag-mCP2-mCherry). Both circLIFR-MS2 and Flag-mCP2-mCherry were transfected into the Sk-Hep1 cells. The expression of circLIFR-MS2 and Flag-mCP2-mCherry was examined using a fluorescence microscope. An anti-FLAG antibody was used to purify the Flag-mCP2-mCherry/circLIFR-MS2 complex. Proteins interacting with cir-cLIFR-MS2 were extracted and used for mass spectrometry.

### Mass spectrometry

Peptides were dissolved in (0.1% formic acid and 2% acetonitrile) solution, cen-trifuged at 4°C, 13,200 rpm, for 20 min, and the supernatant was identified by mass spectrometry. The separated peptides were directly uploaded using a ThermoScientificQExactive mass spectrometer for online detection. The MMFileConversion software was used to convert the results to the MGF format file and then subjected to MASCOT (http://www.matrixscience.com/) to search the UniProt database. The comparison da-tabase for this experiment was Homosapien (https://www.uniprot.org/taxonomy/9606).

### circRNA *in vivo* precipitation

The cells were co-transfected with control MS2bs-Rluc, MS2bs-circLIFRmt, or MS2bs-circLIFR using Lipofectamine 2000. RIP was performed using a Magna RIP RNA-Binding Protein Immunoprecipitation Kit (Millipore, Germany) 48 h later. For the RIP assay on TBK1, RIP was performed with an anti-TBK1 antibody (Millipore, Billerica United States, MA) 48 h after transfection.

### Luciferase reporter assay

The 293T cells were cultured and inoculated in a 24-well plate. The cells were al-lowed to grow to 70% Mel 80% before being transfected into the hsa _circ_0072309 plasmid. The cells were treated with a TBK1 inhibitor for 1 and 24 h after transfection. The luciferase reporter gene was detected after complete lysis of the cells 48 h after transfection.

### Western blot assay

Proteins were separated using 10% SDS-PAGE and transferred to a PVDF mem-brane (Millipore). The film was then sealed in Tris-buffered saline at room temperature. Incubation was performed using primary antibodies. The membrane was then washed with TBST 3 times and incubated with the secondary antibody at room temperature for 2 h. Finally, chemiluminescence detection was performed.

### Immunofluorescence *in situ* hybridization

At harvest, cells were fixed with 4% paraformaldehyde and permeabilized with 0.5% Triton X-100. Then, the cells were incubated with denatured probe (hsa_circ_0072309) at 37°C overnight (12–18 h). The primary antibody was diluted with 3% bovine serum albumin (BSA) and incubated overnight with a fluorescent sec-ondary antibody (1:200) in the dark at RT for 1h. DAPI was used for nuclear staining. A TCS SP2 AOBS laser scanning confocal microscope was used for confocal microsco-py.

### Statistical analysis

All experimental data were analyzed by spss21.0 software. The results are ex-pressed as mean ± standard deviation (x ± s). The means of two independent samples were compared using t-tests. Data were compared between multiple groups using one-way ANOVA and the LSD method. Results were considered statistically signifi-cant at *p* < 0.05.

## Data Availability

The datasets presented in this study can be found in online repositories. The names of the repository/repositories and accession number(s) can be found in the article/Supplementary Material.
