# Supplementary material for: Downregulation of circLIFR exerts cancer-promoting effects on hepatocellular carcinoma in vitro
Source: Front Genet. 2022 Sep 12;13:986322. doi: 10.3389/fgene.2022.986322 (PMC9513674; doi:10.3389/fgene.2022.986322)
Supplement: Supplementary file 1 [file DataSheet3.ZIP › Original dataú¿1ú⌐/Mass spectrometric detection/Control group no.6/Peptide Summary Report (.._data_20191122_F024829.dat).html]

Peptide Summary Report (../data/20191122/F024829.dat)


# Mascot Search Results

```
User            : 
Email           : 
Search title    : 
MS data file    : GS_83_1.mgf
Database        : SP_human0806 human0806 (20432 sequences; 11380987 residues)
Timestamp       : 22 Nov 2019 at 01:34:05 GMT

|  |  |  |
| --- | --- | --- |
| Protein hits    : | sp|P04264|K2C1_HUMAN | Keratin, type II cytoskeletal 1 OS=Homo sapiens OX=9606 GN=KRT1 PE=1 SV=6 |
|  | sp|P13645|K1C10_HUMAN | Keratin, type I cytoskeletal 10 OS=Homo sapiens OX=9606 GN=KRT10 PE=1 SV=6 |
|  | sp|P35908|K22E_HUMAN | Keratin, type II cytoskeletal 2 epidermal OS=Homo sapiens OX=9606 GN=KRT2 PE=1 SV=2 |
|  | sp|P35527|K1C9_HUMAN | Keratin, type I cytoskeletal 9 OS=Homo sapiens OX=9606 GN=KRT9 PE=1 SV=3 |
|  | sp|P67809|YBOX1_HUMAN | Nuclease-sensitive element-binding protein 1 OS=Homo sapiens OX=9606 GN=YBX1 PE=1 SV=3 |
|  | sp|P02533|K1C14_HUMAN | Keratin, type I cytoskeletal 14 OS=Homo sapiens OX=9606 GN=KRT14 PE=1 SV=4 |
|  | sp|P13647|K2C5_HUMAN | Keratin, type II cytoskeletal 5 OS=Homo sapiens OX=9606 GN=KRT5 PE=1 SV=3 |
|  | sp|P02538|K2C6A_HUMAN | Keratin, type II cytoskeletal 6A OS=Homo sapiens OX=9606 GN=KRT6A PE=1 SV=3 |
|  | sp|P08779|K1C16_HUMAN | Keratin, type I cytoskeletal 16 OS=Homo sapiens OX=9606 GN=KRT16 PE=1 SV=4 |
|  | sp|P04259|K2C6B_HUMAN | Keratin, type II cytoskeletal 6B OS=Homo sapiens OX=9606 GN=KRT6B PE=1 SV=5 |
|  | sp|P05787|K2C8_HUMAN | Keratin, type II cytoskeletal 8 OS=Homo sapiens OX=9606 GN=KRT8 PE=1 SV=7 |
|  | sp|P08670|VIME_HUMAN | Vimentin OS=Homo sapiens OX=9606 GN=VIM PE=1 SV=4 |
|  | sp|P05783|K1C18_HUMAN | Keratin, type I cytoskeletal 18 OS=Homo sapiens OX=9606 GN=KRT18 PE=1 SV=2 |
|  | sp|P07437|TBB5_HUMAN | Tubulin beta chain OS=Homo sapiens OX=9606 GN=TUBB PE=1 SV=2 |
|  | sp|Q04695|K1C17_HUMAN | Keratin, type I cytoskeletal 17 OS=Homo sapiens OX=9606 GN=KRT17 PE=1 SV=2 |
|  | sp|P60709|ACTB_HUMAN | Actin, cytoplasmic 1 OS=Homo sapiens OX=9606 GN=ACTB PE=1 SV=1 |
|  | sp|P68371|TBB4B_HUMAN | Tubulin beta-4B chain OS=Homo sapiens OX=9606 GN=TUBB4B PE=1 SV=1 |
|  | sp|P16989|YBOX3_HUMAN | Y-box-binding protein 3 OS=Homo sapiens OX=9606 GN=YBX3 PE=1 SV=4 |
|  | sp|Q07666|KHDR1_HUMAN | KH domain-containing, RNA-binding, signal transduction-associated protein 1 OS=Homo sapiens OX=9606 GN=KHDRBS1 PE=1 SV=1 |
|  | sp|P08729|K2C7_HUMAN | Keratin, type II cytoskeletal 7 OS=Homo sapiens OX=9606 GN=KRT7 PE=1 SV=5 |
|  | sp|P13646|K1C13_HUMAN | Keratin, type I cytoskeletal 13 OS=Homo sapiens OX=9606 GN=KRT13 PE=1 SV=4 |
|  | sp|P19012|K1C15_HUMAN | Keratin, type I cytoskeletal 15 OS=Homo sapiens OX=9606 GN=KRT15 PE=1 SV=3 |
|  | sp|O95678|K2C75_HUMAN | Keratin, type II cytoskeletal 75 OS=Homo sapiens OX=9606 GN=KRT75 PE=1 SV=2 |
|  | sp|P68032|ACTC_HUMAN | Actin, alpha cardiac muscle 1 OS=Homo sapiens OX=9606 GN=ACTC1 PE=1 SV=1 |
|  | sp|P08727|K1C19_HUMAN | Keratin, type I cytoskeletal 19 OS=Homo sapiens OX=9606 GN=KRT19 PE=1 SV=4 |
|  | sp|Q7Z794|K2C1B_HUMAN | Keratin, type II cytoskeletal 1b OS=Homo sapiens OX=9606 GN=KRT77 PE=2 SV=3 |
|  | sp|Q6S8J3|POTEE_HUMAN | POTE ankyrin domain family member E OS=Homo sapiens OX=9606 GN=POTEE PE=2 SV=3 |
|  | sp|Q04837|SSBP_HUMAN | Single-stranded DNA-binding protein, mitochondrial OS=Homo sapiens OX=9606 GN=SSBP1 PE=1 SV=1 |
|  | sp|P52597|HNRPF_HUMAN | Heterogeneous nuclear ribonucleoprotein F OS=Homo sapiens OX=9606 GN=HNRNPF PE=1 SV=3 |
|  | sp|P02768|ALBU_HUMAN | Serum albumin OS=Homo sapiens OX=9606 GN=ALB PE=1 SV=2 |
|  | sp|Q01546|K22O_HUMAN | Keratin, type II cytoskeletal 2 oral OS=Homo sapiens OX=9606 GN=KRT76 PE=1 SV=2 |
|  | sp|Q5XKE5|K2C79_HUMAN | Keratin, type II cytoskeletal 79 OS=Homo sapiens OX=9606 GN=KRT79 PE=1 SV=2 |
|  | sp|P12035|K2C3_HUMAN | Keratin, type II cytoskeletal 3 OS=Homo sapiens OX=9606 GN=KRT3 PE=1 SV=3 |
|  | sp|P68363|TBA1B_HUMAN | Tubulin alpha-1B chain OS=Homo sapiens OX=9606 GN=TUBA1B PE=1 SV=1 |
|  | sp|Q14CN4|K2C72_HUMAN | Keratin, type II cytoskeletal 72 OS=Homo sapiens OX=9606 GN=KRT72 PE=1 SV=2 |
|  | sp|O00571|DDX3X_HUMAN | ATP-dependent RNA helicase DDX3X OS=Homo sapiens OX=9606 GN=DDX3X PE=1 SV=3 |
|  | sp|Q7RTS7|K2C74_HUMAN | Keratin, type II cytoskeletal 74 OS=Homo sapiens OX=9606 GN=KRT74 PE=1 SV=2 |
|  | sp|Q86Y46|K2C73_HUMAN | Keratin, type II cytoskeletal 73 OS=Homo sapiens OX=9606 GN=KRT73 PE=1 SV=1 |
|  | sp|Q86YZ3|HORN_HUMAN | Hornerin OS=Homo sapiens OX=9606 GN=HRNR PE=1 SV=2 |
|  | sp|P81605|DCD_HUMAN | Dermcidin OS=Homo sapiens OX=9606 GN=DCD PE=1 SV=2 |
|  | sp|Q7Z3Z0|K1C25_HUMAN | Keratin, type I cytoskeletal 25 OS=Homo sapiens OX=9606 GN=KRT25 PE=1 SV=1 |
|  | sp|Q92804|RBP56_HUMAN | TATA-binding protein-associated factor 2N OS=Homo sapiens OX=9606 GN=TAF15 PE=1 SV=1 |
|  | sp|O00622|CCN1_HUMAN | CCN family member 1 OS=Homo sapiens OX=9606 GN=CCN1 PE=1 SV=1 |
|  | sp|Q2M2I5|K1C24_HUMAN | Keratin, type I cytoskeletal 24 OS=Homo sapiens OX=9606 GN=KRT24 PE=1 SV=1 |
|  | sp|Q99456|K1C12_HUMAN | Keratin, type I cytoskeletal 12 OS=Homo sapiens OX=9606 GN=KRT12 PE=1 SV=1 |
|  | sp|P31943|HNRH1_HUMAN | Heterogeneous nuclear ribonucleoprotein H OS=Homo sapiens OX=9606 GN=HNRNPH1 PE=1 SV=4 |
|  | sp|P41219|PERI_HUMAN | Peripherin OS=Homo sapiens OX=9606 GN=PRPH PE=1 SV=2 |
|  | sp|Q562R1|ACTBL_HUMAN | Beta-actin-like protein 2 OS=Homo sapiens OX=9606 GN=ACTBL2 PE=1 SV=2 |
|  | sp|P17661|DESM_HUMAN | Desmin OS=Homo sapiens OX=9606 GN=DES PE=1 SV=3 |
|  | sp|P62805|H4_HUMAN | Histone H4 OS=Homo sapiens OX=9606 GN=HIST1H4A PE=1 SV=2 |
|  | sp|P04406|G3P_HUMAN | Glyceraldehyde-3-phosphate dehydrogenase OS=Homo sapiens OX=9606 GN=GAPDH PE=1 SV=3 |
|  | sp|Q02413|DSG1_HUMAN | Desmoglein-1 OS=Homo sapiens OX=9606 GN=DSG1 PE=1 SV=2 |
|  | sp|Q9NSB2|KRT84_HUMAN | Keratin, type II cuticular Hb4 OS=Homo sapiens OX=9606 GN=KRT84 PE=2 SV=2 |
|  | sp|Q96PK6|RBM14_HUMAN | RNA-binding protein 14 OS=Homo sapiens OX=9606 GN=RBM14 PE=1 SV=2 |
|  | sp|A6NMY6|AXA2L_HUMAN | Putative annexin A2-like protein OS=Homo sapiens OX=9606 GN=ANXA2P2 PE=5 SV=2 |
|  | sp|Q7Z3Y9|K1C26_HUMAN | Keratin, type I cytoskeletal 26 OS=Homo sapiens OX=9606 GN=KRT26 PE=1 SV=2 |
|  | sp|P35030|TRY3_HUMAN | Trypsin-3 OS=Homo sapiens OX=9606 GN=PRSS3 PE=1 SV=2 |
|  | sp|P62241|RS8_HUMAN | 40S ribosomal protein S8 OS=Homo sapiens OX=9606 GN=RPS8 PE=1 SV=2 |
|  | sp|P19013|K2C4_HUMAN | Keratin, type II cytoskeletal 4 OS=Homo sapiens OX=9606 GN=KRT4 PE=1 SV=4 |
|  | sp|Q9H4B7|TBB1_HUMAN | Tubulin beta-1 chain OS=Homo sapiens OX=9606 GN=TUBB1 PE=1 SV=1 |
|  | sp|P06702|S10A9_HUMAN | Protein S100-A9 OS=Homo sapiens OX=9606 GN=S100A9 PE=1 SV=1 |
|  | sp|P05109|S10A8_HUMAN | Protein S100-A8 OS=Homo sapiens OX=9606 GN=S100A8 PE=1 SV=1 |
|  | sp|P35900|K1C20_HUMAN | Keratin, type I cytoskeletal 20 OS=Homo sapiens OX=9606 GN=KRT20 PE=1 SV=1 |
|  | sp|Q5T749|KPRP_HUMAN | Keratinocyte proline-rich protein OS=Homo sapiens OX=9606 GN=KPRP PE=1 SV=1 |
|  | sp|Q8N1N4|K2C78_HUMAN | Keratin, type II cytoskeletal 78 OS=Homo sapiens OX=9606 GN=KRT78 PE=1 SV=2 |
|  | sp|P14923|PLAK_HUMAN | Junction plakoglobin OS=Homo sapiens OX=9606 GN=JUP PE=1 SV=3 |
|  | sp|Q92764|KRT35_HUMAN | Keratin, type I cuticular Ha5 OS=Homo sapiens OX=9606 GN=KRT35 PE=2 SV=5 |
|  | sp|P55795|HNRH2_HUMAN | Heterogeneous nuclear ribonucleoprotein H2 OS=Homo sapiens OX=9606 GN=HNRNPH2 PE=1 SV=1 |
|  | sp|Q8TF72|SHRM3_HUMAN | Protein Shroom3 OS=Homo sapiens OX=9606 GN=SHROOM3 PE=1 SV=2 |
|  | sp|Q5T750|XP32_HUMAN | Skin-specific protein 32 OS=Homo sapiens OX=9606 GN=XP32 PE=1 SV=1 |
|  | sp|P15924|DESP_HUMAN | Desmoplakin OS=Homo sapiens OX=9606 GN=DSP PE=1 SV=3 |
|  | sp|Q8NHM4|TRY6_HUMAN | Putative trypsin-6 OS=Homo sapiens OX=9606 GN=PRSS3P2 PE=5 SV=2 |
|  | sp|P0DOX5|IGG1_HUMAN | Immunoglobulin gamma-1 heavy chain OS=Homo sapiens OX=9606 PE=1 SV=2 |
|  | sp|Q9NQI0|DDX4_HUMAN | Probable ATP-dependent RNA helicase DDX4 OS=Homo sapiens OX=9606 GN=DDX4 PE=1 SV=2 |
|  | sp|P12236|ADT3_HUMAN | ADP/ATP translocase 3 OS=Homo sapiens OX=9606 GN=SLC25A6 PE=1 SV=4 |
|  | sp|P61978|HNRPK_HUMAN | Heterogeneous nuclear ribonucleoprotein K OS=Homo sapiens OX=9606 GN=HNRNPK PE=1 SV=1 |
|  | sp|P04003|C4BPA_HUMAN | C4b-binding protein alpha chain OS=Homo sapiens OX=9606 GN=C4BPA PE=1 SV=2 |
|  | sp|Q96P63|SPB12_HUMAN | Serpin B12 OS=Homo sapiens OX=9606 GN=SERPINB12 PE=1 SV=1 |
|  | sp|P34931|HS71L_HUMAN | Heat shock 70 kDa protein 1-like OS=Homo sapiens OX=9606 GN=HSPA1L PE=1 SV=2 |
|  | sp|P0C869|PA24B_HUMAN | Cytosolic phospholipase A2 beta OS=Homo sapiens OX=9606 GN=PLA2G4B PE=1 SV=2 |
|  | sp|Q00325|MPCP_HUMAN | Phosphate carrier protein, mitochondrial OS=Homo sapiens OX=9606 GN=SLC25A3 PE=1 SV=2 |
|  | sp|P31151|S10A7_HUMAN | Protein S100-A7 OS=Homo sapiens OX=9606 GN=S100A7 PE=1 SV=4 |
|  | sp|P83731|RL24_HUMAN | 60S ribosomal protein L24 OS=Homo sapiens OX=9606 GN=RPL24 PE=1 SV=1 |
|  | sp|O75525|KHDR3_HUMAN | KH domain-containing, RNA-binding, signal transduction-associated protein 3 OS=Homo sapiens OX=9606 GN=KHDRBS3 PE=1 SV=1 |
|  | sp|P61626|LYSC_HUMAN | Lysozyme C OS=Homo sapiens OX=9606 GN=LYZ PE=1 SV=1 |
|  | sp|Q6KB66|K2C80_HUMAN | Keratin, type II cytoskeletal 80 OS=Homo sapiens OX=9606 GN=KRT80 PE=1 SV=2 |
|  | sp|Q01844|EWS_HUMAN | RNA-binding protein EWS OS=Homo sapiens OX=9606 GN=EWSR1 PE=1 SV=1 |
|  | sp|O00338|ST1C2_HUMAN | Sulfotransferase 1C2 OS=Homo sapiens OX=9606 GN=SULT1C2 PE=1 SV=1 |
|  | sp|Q06830|PRDX1_HUMAN | Peroxiredoxin-1 OS=Homo sapiens OX=9606 GN=PRDX1 PE=1 SV=1 |
|  | sp|Q86U44|MTA70_HUMAN | N6-adenosine-methyltransferase catalytic subunit OS=Homo sapiens OX=9606 GN=METTL3 PE=1 SV=2 |
|  | sp|P62979|RS27A_HUMAN | Ubiquitin-40S ribosomal protein S27a OS=Homo sapiens OX=9606 GN=RPS27A PE=1 SV=2 |
|  | sp|Q9BYE4|SPR2G_HUMAN | Small proline-rich protein 2G OS=Homo sapiens OX=9606 GN=SPRR2G PE=3 SV=1 |
|  | sp|Q5D862|FILA2_HUMAN | Filaggrin-2 OS=Homo sapiens OX=9606 GN=FLG2 PE=1 SV=1 |
|  | sp|Q7RTW8|OTOAN_HUMAN | Otoancorin OS=Homo sapiens OX=9606 GN=OTOA PE=1 SV=1 |
|  | sp|Q9H2F5|EPC1_HUMAN | Enhancer of polycomb homolog 1 OS=Homo sapiens OX=9606 GN=EPC1 PE=1 SV=1 |
|  | sp|O00472|ELL2_HUMAN | RNA polymerase II elongation factor ELL2 OS=Homo sapiens OX=9606 GN=ELL2 PE=1 SV=2 |
|  | sp|O94868|FCSD2_HUMAN | F-BAR and double SH3 domains protein 2 OS=Homo sapiens OX=9606 GN=FCHSD2 PE=1 SV=3 |
|  | sp|O95715|CXL14_HUMAN | C-X-C motif chemokine 14 OS=Homo sapiens OX=9606 GN=CXCL14 PE=1 SV=2 |
|  | sp|P47914|RL29_HUMAN | 60S ribosomal protein L29 OS=Homo sapiens OX=9606 GN=RPL29 PE=1 SV=2 |
|  | sp|O75191|XYLB_HUMAN | Xylulose kinase OS=Homo sapiens OX=9606 GN=XYLB PE=1 SV=3 |
|  | sp|Q9P0W8|SPAT7_HUMAN | Spermatogenesis-associated protein 7 OS=Homo sapiens OX=9606 GN=SPATA7 PE=1 SV=3 |
|  | sp|Q6DN72|FCRL6_HUMAN | Fc receptor-like protein 6 OS=Homo sapiens OX=9606 GN=FCRL6 PE=1 SV=2 |
|  | sp|P0C0S5|H2AZ_HUMAN | Histone H2A.Z OS=Homo sapiens OX=9606 GN=H2AFZ PE=1 SV=2 |
|  | sp|Q96Q27|ASB2_HUMAN | Ankyrin repeat and SOCS box protein 2 OS=Homo sapiens OX=9606 GN=ASB2 PE=1 SV=1 |
|  | sp|P12036|NFH_HUMAN | Neurofilament heavy polypeptide OS=Homo sapiens OX=9606 GN=NEFH PE=1 SV=4 |
|  | sp|Q8TER0|SNED1_HUMAN | Sushi, nidogen and EGF-like domain-containing protein 1 OS=Homo sapiens OX=9606 GN=SNED1 PE=2 SV=2 |
|  | sp|P22079|PERL_HUMAN | Lactoperoxidase OS=Homo sapiens OX=9606 GN=LPO PE=1 SV=2 |
|  | sp|Q03989|ARI5A_HUMAN | AT-rich interactive domain-containing protein 5A OS=Homo sapiens OX=9606 GN=ARID5A PE=1 SV=2 |
|  | sp|P23490|LORI_HUMAN | Loricrin OS=Homo sapiens OX=9606 GN=LOR PE=1 SV=2 |
|  | sp|Q15517|CDSN_HUMAN | Corneodesmosin OS=Homo sapiens OX=9606 GN=CDSN PE=1 SV=3 |
|  | sp|Q08174|PCDH1_HUMAN | Protocadherin-1 OS=Homo sapiens OX=9606 GN=PCDH1 PE=1 SV=2 |
|  | sp|Q9HCF6|TRPM3_HUMAN | Transient receptor potential cation channel subfamily M member 3 OS=Homo sapiens OX=9606 GN=TRPM3 PE=2 SV=4 |
|  | sp|Q02880|TOP2B_HUMAN | DNA topoisomerase 2-beta OS=Homo sapiens OX=9606 GN=TOP2B PE=1 SV=3 |
|  | sp|Q5VX52|SPAT1_HUMAN | Spermatogenesis-associated protein 1 OS=Homo sapiens OX=9606 GN=SPATA1 PE=2 SV=4 |
|  | sp|P20674|COX5A_HUMAN | Cytochrome c oxidase subunit 5A, mitochondrial OS=Homo sapiens OX=9606 GN=COX5A PE=1 SV=2 |
|  | sp|Q3KNS1|PTHD3_HUMAN | Patched domain-containing protein 3 OS=Homo sapiens OX=9606 GN=PTCHD3 PE=1 SV=3 |
|  | sp|Q14192|FHL2_HUMAN | Four and a half LIM domains protein 2 OS=Homo sapiens OX=9606 GN=FHL2 PE=1 SV=3 |
|  | sp|Q8WXE9|STON2_HUMAN | Stonin-2 OS=Homo sapiens OX=9606 GN=STON2 PE=1 SV=1 |
|  | sp|P61313|RL15_HUMAN | 60S ribosomal protein L15 OS=Homo sapiens OX=9606 GN=RPL15 PE=1 SV=2 |
|  | sp|Q9BZF1|OSBL8_HUMAN | Oxysterol-binding protein-related protein 8 OS=Homo sapiens OX=9606 GN=OSBPL8 PE=1 SV=3 |
|  | sp|O95573|ACSL3_HUMAN | Long-chain-fatty-acid--CoA ligase 3 OS=Homo sapiens OX=9606 GN=ACSL3 PE=1 SV=3 |
|  | sp|Q76FK4|NOL8_HUMAN | Nucleolar protein 8 OS=Homo sapiens OX=9606 GN=NOL8 PE=1 SV=1 |
|  | sp|O43291|SPIT2_HUMAN | Kunitz-type protease inhibitor 2 OS=Homo sapiens OX=9606 GN=SPINT2 PE=1 SV=2 |
|  | sp|Q9BYB0|SHAN3_HUMAN | SH3 and multiple ankyrin repeat domains protein 3 OS=Homo sapiens OX=9606 GN=SHANK3 PE=1 SV=3 |
|  | sp|Q96P71|NECA3_HUMAN | N-terminal EF-hand calcium-binding protein 3 OS=Homo sapiens OX=9606 GN=NECAB3 PE=1 SV=2 |
|  | sp|Q08554|DSC1_HUMAN | Desmocollin-1 OS=Homo sapiens OX=9606 GN=DSC1 PE=1 SV=2 |
|  | sp|O94979|SC31A_HUMAN | Protein transport protein Sec31A OS=Homo sapiens OX=9606 GN=SEC31A PE=1 SV=3 |
|  | sp|Q96QD5|DEPD7_HUMAN | DEP domain-containing protein 7 OS=Homo sapiens OX=9606 GN=DEPDC7 PE=2 SV=1 |
|  | sp|Q9H5K3|SG196_HUMAN | Protein O-mannose kinase OS=Homo sapiens OX=9606 GN=POMK PE=1 SV=1 |
|  | sp|Q5H9J7|BEX5_HUMAN | Protein BEX5 OS=Homo sapiens OX=9606 GN=BEX5 PE=1 SV=1 |
|  | sp|P0C874|S31D3_HUMAN | Spermatogenesis-associated protein 31D3 OS=Homo sapiens OX=9606 GN=SPATA31D3 PE=2 SV=1 |
|  | sp|Q9UIF3|TEKT2_HUMAN | Tektin-2 OS=Homo sapiens OX=9606 GN=TEKT2 PE=1 SV=1 |
|  | sp|P27708|PYR1_HUMAN | CAD protein OS=Homo sapiens OX=9606 GN=CAD PE=1 SV=3 |
|  | sp|P35221|CTNA1_HUMAN | Catenin alpha-1 OS=Homo sapiens OX=9606 GN=CTNNA1 PE=1 SV=1 |
|  | sp|E7EW31|PROB1_HUMAN | Proline-rich basic protein 1 OS=Homo sapiens OX=9606 GN=PROB1 PE=2 SV=2 |
|  | sp|Q9UPW6|SATB2_HUMAN | DNA-binding protein SATB2 OS=Homo sapiens OX=9606 GN=SATB2 PE=1 SV=2 |
|  | sp|Q6UY18|LIGO4_HUMAN | Leucine-rich repeat and immunoglobulin-like domain-containing nogo receptor-interacting protein 4 OS=Homo sapiens OX=9606 GN=LINGO4 PE=2 SV=1 |
|  | sp|Q96QI5|HS3S6_HUMAN | Heparan sulfate glucosamine 3-O-sulfotransferase 6 OS=Homo sapiens OX=9606 GN=HS3ST6 PE=1 SV=2 |
|  | sp|P20930|FILA_HUMAN | Filaggrin OS=Homo sapiens OX=9606 GN=FLG PE=1 SV=3 |
|  | sp|Q15054|DPOD3_HUMAN | DNA polymerase delta subunit 3 OS=Homo sapiens OX=9606 GN=POLD3 PE=1 SV=2 |
|  | sp|Q14161|GIT2_HUMAN | ARF GTPase-activating protein GIT2 OS=Homo sapiens OX=9606 GN=GIT2 PE=1 SV=2 |
|  | sp|Q8N1G1|REXO1_HUMAN | RNA exonuclease 1 homolog OS=Homo sapiens OX=9606 GN=REXO1 PE=1 SV=3 |
|  | sp|O14640|DVL1_HUMAN | Segment polarity protein dishevelled homolog DVL-1 OS=Homo sapiens OX=9606 GN=DVL1 PE=1 SV=2 |
```

|  | SP\_human0806 | Decoy | False discovery rate |
| --- | --- | --- | --- |
| Peptide matches above identity threshold | 481 | 29 | 6.03 % |
| Peptide matches above homology or identity threshold | 523 | 47 | 8.99 % |

### Mascot Score Histogram

Ions score is -10\*Log(P), where P is the probability that the observed match is a random event.  
Individual ions scores > 22 indicate identity or extensive homology (p<0.05).  
Protein scores are derived from ions scores as a non-probabilistic basis for ranking protein hits.


### Peptide Summary Report

|  |  |  |  |
| --- | --- | --- | --- |
|  | Protein Family Summary Peptide Summary Select Summary (protein hits) Select Summary (unassigned) Export Search Results |  | Help |
|  | Significance threshold p< | Max. number of hits | Show Percolator scores |
|  | Standard scoring  MudPIT scoring | Ions score or expect cut-off | Show sub-sets |
|  | Show pop-ups  Suppress pop-ups | Sort unassigned  Decreasing Score Increasing query / Mr Decreasing Intensity | Require bold red |


  
  
  **Error tolerant**   

|  |  |
| --- | --- |
| **1.** | sp|P04264|K2C1\_HUMAN    **Mass:** 66170    **Score:** 2162   **Matches:** 70(62)  **Sequences:** 29(27)  **emPAI:** 5.01 |
|  | Keratin, type II cytoskeletal 1 OS=Homo sapiens OX=9606 GN=KRT1 PE=1 SV=6 |

|  |  |
| --- | --- |
|  | Check to include this hit in error tolerant search or archive report |
|  |  |

|  |  |  |  |  |  |  |  |  |  |  |  |
| --- | --- | --- | --- | --- | --- | --- | --- | --- | --- | --- | --- |
|  | **Query** | **Observed** | **Mr(expt)** | **Mr(calc)** | **ppm** | **Miss** | **Score** | **Expect** | **Rank** | **Unique** | **Peptide** |
|  | 270 | **416.7489** | **831.4832** | **831.4814** | **2.15** | **0** | **28** | **0.02** | **1** | **U** | **K.SISISVAR.G** |
|  | 271 | **416.7509** | **831.4871** | **831.4814** | **6.91** | **0** | **(24)** | **0.039** | **1** | **U** | **K.SISISVAR.G** |
|  | 559 | **448.2351** | **894.4556** | **894.4519** | **4.14** | **1** | **0** | **14** | **10** | **U** | **R.RTTSSSTR.R** |
|  | 841 | **487.2693** | **972.5241** | **972.5240** | **0.12** | **0** | **39** | **0.0024** | **1** |  | **K.IEISELNR.V** |
|  | 842 | **487.2694** | **972.5243** | **972.5240** | **0.30** | **0** | **(39)** | **0.0026** | **1** |  | **K.IEISELNR.V** |
|  | 943 | **500.2259** | **998.4372** | **998.4379** | **-0.73** | **0** | **26** | **0.016** | **1** | **U** | **K.DVDGAYMTK.V** |
|  | 944 | **500.2269** | **998.4392** | **998.4379** | **1.29** | **0** | **(23)** | **0.028** | **1** | **U** | **K.DVDGAYMTK.V** |
|  | 1079 | **517.2607** | **1032.5069** | **1032.5087** | **-1.74** | **0** | **(23)** | **0.11** | **1** | **U** | **R.TLLEGEESR.M** |
|  | 1080 | **517.2616** | **1032.5086** | **1032.5087** | **-0.08** | **0** | **(33)** | **0.0091** | **1** | **U** | **R.TLLEGEESR.M** |
|  | 1081 | **517.2621** | **1032.5096** | **1032.5087** | **0.85** | **0** | **40** | **0.0018** | **1** | **U** | **R.TLLEGEESR.M** |
|  | 1269 | **533.2640** | **1064.5135** | **1064.5138** | **-0.26** | **0** | **47** | **0.00031** | **1** | **U** | **K.AQYEDIAQK.S** |
|  | 1270 | **533.2701** | **1064.5256** | **1064.5138** | **11.1** | **0** | **(26)** | **0.037** | **1** | **U** | **K.AQYEDIAQK.S** |
|  | 1284 | **533.7620** | **1065.5094** | **1065.5090** | **0.30** | **1** | **10** | **1.6** | **1** |  | **K.YEDEINKR.T** |
|  | 1314 | **358.5362** | **1072.5867** | **1072.5876** | **-0.93** | **1** | **28** | **0.031** | **1** | **U** | **R.LRSEIDNVK.K** |
|  | 1315 | **537.3011** | **1072.5876** | **1072.5876** | **-0.02** | **1** | **(24)** | **0.082** | **1** | **U** | **R.LRSEIDNVK.K** |
|  | 1316 | **537.3011** | **1072.5876** | **1072.5876** | **-0.02** | **1** | **(24)** | **0.076** | **1** | **U** | **R.LRSEIDNVK.K** |
|  | 1556 | **563.2747** | **1124.5349** | **1124.5349** | **-0.03** | **0** | **51** | **9.5e-005** | **1** | **U** | **K.AEAESLYQSK.Y** |
|  | 1557 | **563.2748** | **1124.5350** | **1124.5349** | **0.07** | **0** | **(2)** | **8.7** | **10** | **U** | **K.AEAESLYQSK.Y** |
|  | 1636 | **571.2633** | **1140.5121** | **1140.5121** | **-0.03** | **0** | **40** | **0.00083** | **1** | **U** | **R.DYQELMNTK.L** |
|  | 1637 | **571.2634** | **1140.5122** | **1140.5121** | **0.08** | **0** | **(31)** | **0.007** | **1** | **U** | **R.DYQELMNTK.L** |
|  | 1683 | **579.2574** | **1156.5002** | **1156.5070** | **-5.87** | **0** | **(27)** | **0.01** | **1** | **U** | **R.DYQELMNTK.L + Oxidation (M)** |
|  | 1684 | **579.2604** | **1156.5063** | **1156.5070** | **-0.60** | **0** | **(33)** | **0.003** | **1** | **U** | **R.DYQELMNTK.L + Oxidation (M)** |
|  | 1780 | **590.3036** | **1178.5927** | **1178.5931** | **-0.32** | **0** | **(70)** | **1.7e-006** | **1** |  | **K.YEELQITAGR.H** |
|  | 1781 | **590.3037** | **1178.5929** | **1178.5931** | **-0.22** | **0** | **71** | **1.2e-006** | **1** |  | **K.YEELQITAGR.H** |
|  | 2126 | **633.3189** | **1264.6233** | **1264.6299** | **-5.25** | **0** | **(59)** | **2e-005** | **1** | **U** | **R.TNAENEFVTIK.K** |
|  | 2127 | **633.3210** | **1264.6274** | **1264.6299** | **-1.98** | **0** | **61** | **1.3e-005** | **1** | **U** | **R.TNAENEFVTIK.K** |
|  | 2128 | **633.3226** | **1264.6306** | **1264.6299** | **0.53** | **0** | **(27)** | **0.032** | **1** | **U** | **R.TNAENEFVTIK.K** |
|  | 2252 | **650.7679** | **1299.5213** | **1299.5224** | **-0.79** | **0** | **(58)** | **2.7e-006** | **1** | **U** | **K.NMQDMVEDYR.N** |
|  | 2253 | **650.7682** | **1299.5219** | **1299.5224** | **-0.31** | **0** | **62** | **1e-006** | **1** | **U** | **K.NMQDMVEDYR.N** |
|  | 2265 | **651.8536** | **1301.6927** | **1301.6939** | **-0.88** | **1** | **25** | **0.053** | **1** | **U** | **R.NSKIEISELNR.V** |
|  | 2267 | **651.8614** | **1301.7082** | **1301.7078** | **0.31** | **0** | **64** | **5.9e-006** | **1** | **U** | **R.SLDLDSIIAEVK.A** |
|  | 2317 | **658.7657** | **1315.5168** | **1315.5173** | **-0.34** | **0** | **(54)** | **4.9e-006** | **1** | **U** | **K.NMQDMVEDYR.N + Oxidation (M)** |
|  | 2318 | **658.7661** | **1315.5177** | **1315.5173** | **0.30** | **0** | **(55)** | **4.5e-006** | **1** | **U** | **K.NMQDMVEDYR.N + Oxidation (M)** |
|  | 2319 | **658.7667** | **1315.5188** | **1315.5173** | **1.14** | **0** | **(48)** | **2e-005** | **1** | **U** | **K.NMQDMVEDYR.N + Oxidation (M)** |
|  | 2373 | **666.7630** | **1331.5114** | **1331.5122** | **-0.56** | **0** | **(57)** | **2e-006** | **1** | **U** | **K.NMQDMVEDYR.N + 2 Oxidation (M)** |
|  | 2374 | **666.7631** | **1331.5116** | **1331.5122** | **-0.47** | **0** | **(57)** | **2.2e-006** | **1** | **U** | **K.NMQDMVEDYR.N + 2 Oxidation (M)** |
|  | 2399 | **670.8380** | **1339.6615** | **1339.6619** | **-0.33** | **1** | **77** | **2.6e-007** | **1** | **U** | **K.SKAEAESLYQSK.Y** |
|  | 2400 | **447.5611** | **1339.6616** | **1339.6619** | **-0.26** | **1** | **(32)** | **0.009** | **1** | **U** | **K.SKAEAESLYQSK.Y** |
|  | 2401 | **447.5612** | **1339.6619** | **1339.6619** | **0.01** | **1** | **(36)** | **0.0033** | **1** | **U** | **K.SKAEAESLYQSK.Y** |
|  | 2402 | **670.8383** | **1339.6620** | **1339.6619** | **0.04** | **1** | **(63)** | **6.3e-006** | **1** | **U** | **K.SKAEAESLYQSK.Y** |
|  | 2459 | **679.3514** | **1356.6882** | **1356.6885** | **-0.20** | **0** | **(76)** | **4.3e-007** | **1** | **U** | **K.LNDLEDALQQAK.E** |
|  | 2460 | **679.3514** | **1356.6883** | **1356.6885** | **-0.11** | **0** | **99** | **2e-009** | **1** | **U** | **K.LNDLEDALQQAK.E** |
|  | 2551 | **692.3460** | **1382.6773** | **1382.6830** | **-4.10** | **0** | **(47)** | **0.0003** | **1** | **U** | **K.SLNNQFASFIDK.V** |
|  | 2552 | **692.3485** | **1382.6825** | **1382.6830** | **-0.39** | **0** | **91** | **1.3e-008** | **1** | **U** | **K.SLNNQFASFIDK.V** |
|  | 2553 | **692.3487** | **1382.6828** | **1382.6830** | **-0.13** | **0** | **(12)** | **0.96** | **1** | **U** | **K.SLNNQFASFIDK.V** |
|  | 2599 | **697.3695** | **1392.7243** | **1392.7249** | **-0.37** | **1** | **(82)** | **9.4e-008** | **1** | **U** | **R.TNAENEFVTIKK.D** |
|  | 2600 | **465.2490** | **1392.7251** | **1392.7249** | **0.13** | **1** | **(56)** | **4.3e-005** | **1** | **U** | **R.TNAENEFVTIKK.D** |
|  | 2601 | **697.3699** | **1392.7252** | **1392.7249** | **0.23** | **1** | **90** | **1.6e-008** | **1** | **U** | **R.TNAENEFVTIKK.D** |
|  | 2602 | **465.2491** | **1392.7255** | **1392.7249** | **0.45** | **1** | **(11)** | **1.3** | **1** | **U** | **R.TNAENEFVTIKK.D** |
|  | 2870 | **738.3964** | **1474.7782** | **1474.7780** | **0.12** | **0** | **(49)** | **0.00019** | **1** |  | **R.FLEQQNQVLQTK.W** |
|  | 2871 | **738.3976** | **1474.7806** | **1474.7780** | **1.78** | **0** | **(64)** | **6e-006** | **1** |  | **R.FLEQQNQVLQTK.W** |
|  | 2872 | **738.3978** | **1474.7810** | **1474.7780** | **2.04** | **0** | **69** | **1.8e-006** | **1** |  | **R.FLEQQNQVLQTK.W** |
|  | 2991 | **762.3982** | **1522.7819** | **1522.7813** | **0.41** | **1** | **(29)** | **0.015** | **1** | **U** | **R.LLRDYQELMNTK.L** |
|  | 2992 | **508.6013** | **1522.7820** | **1522.7813** | **0.47** | **1** | **29** | **0.015** | **1** | **U** | **R.LLRDYQELMNTK.L** |
|  | 3168 | **533.9490** | **1598.8253** | **1598.8264** | **-0.66** | **1** | **(41)** | **0.0011** | **1** | **U** | **K.NKLNDLEDALQQAK.E** |
|  | 3169 | **800.4209** | **1598.8272** | **1598.8264** | **0.56** | **1** | **83** | **6.4e-008** | **1** | **U** | **K.NKLNDLEDALQQAK.E** |
|  | 3248 | **819.9351** | **1637.8556** | **1637.8525** | **1.85** | **1** | **31** | **0.0094** | **1** | **U** | **K.SLNNQFASFIDKVR.F** |
|  | 3278 | **829.3998** | **1656.7851** | **1656.7856** | **-0.28** | **0** | **(75)** | **4e-007** | **1** | **U** | **R.SGGGFSSGSAGIINYQR.R** |
|  | 3279 | **829.4005** | **1656.7865** | **1656.7856** | **0.52** | **0** | **96** | **2.8e-009** | **1** | **U** | **R.SGGGFSSGSAGIINYQR.R** |
|  | 3389 | **572.9550** | **1715.8432** | **1715.8438** | **-0.34** | **0** | **(44)** | **0.00056** | **1** | **U** | **K.QISNLQQSISDAEQR.G** |
|  | 3390 | **858.9291** | **1715.8436** | **1715.8438** | **-0.12** | **0** | **97** | **2.9e-009** | **1** | **U** | **K.QISNLQQSISDAEQR.G** |
|  | 3391 | **858.9295** | **1715.8444** | **1715.8438** | **0.37** | **0** | **(86)** | **3.4e-008** | **1** | **U** | **K.QISNLQQSISDAEQR.G** |
|  | 3392 | **572.9556** | **1715.8449** | **1715.8438** | **0.62** | **0** | **(55)** | **4.8e-005** | **1** | **U** | **K.QISNLQQSISDAEQR.G** |
|  | 3393 | **858.9308** | **1715.8471** | **1715.8438** | **1.94** | **0** | **(64)** | **6e-006** | **1** | **U** | **K.QISNLQQSISDAEQR.G** |
|  | 3509 | **883.3709** | **1764.7271** | **1764.7275** | **-0.20** | **0** | **116** | **8e-012** | **1** | **U** | **R.FSSCGGGGGSFGAGGGFGSR.S** |
|  | 3704 | **922.9792** | **1843.9439** | **1843.9388** | **2.81** | **1** | **126** | **3.3e-012** | **1** | **U** | **K.KQISNLQQSISDAEQR.G** |
|  | 3705 | **615.6556** | **1843.9449** | **1843.9388** | **3.33** | **1** | **(61)** | **1.1e-005** | **1** | **U** | **K.KQISNLQQSISDAEQR.G** |
|  | 3972 | **673.2993** | **2016.8761** | **2016.8768** | **-0.35** | **1** | **35** | **0.0012** | **1** | **U** | **R.LDSELKNMQDMVEDYR.N + 2 Oxidation (M)** |
|  | 3973 | **1009.4460** | **2016.8774** | **2016.8768** | **0.29** | **1** | **(33)** | **0.002** | **1** | **U** | **R.LDSELKNMQDMVEDYR.N + 2 Oxidation (M)** |
|  | 4320 | **1192.4812** | **2382.9478** | **2382.9447** | **1.34** | **0** | **80** | **1.1e-008** | **1** | **U** | **R.GGGGGGYGSGGSSYGSGGGSYGSGGGGGGGR.G** |

---

|  |  |
| --- | --- |
| **2.** | sp|P13645|K1C10\_HUMAN    **Mass:** 59020    **Score:** 1447   **Matches:** 62(54)  **Sequences:** 26(25)  **emPAI:** 5.67 |
|  | Keratin, type I cytoskeletal 10 OS=Homo sapiens OX=9606 GN=KRT10 PE=1 SV=6 |

|  |  |
| --- | --- |
|  | Check to include this hit in error tolerant search or archive report |
|  |  |

|  |  |  |  |  |  |  |  |  |  |  |  |
| --- | --- | --- | --- | --- | --- | --- | --- | --- | --- | --- | --- |
|  | **Query** | **Observed** | **Mr(expt)** | **Mr(calc)** | **ppm** | **Miss** | **Score** | **Expect** | **Rank** | **Unique** | **Peptide** |
|  | 189 | **404.2032** | **806.3918** | **806.3923** | **-0.61** | **0** | **43** | **0.0011** | **1** |  | **R.LAADDFR.L** |
|  | 190 | **404.2034** | **806.3923** | **806.3923** | **0.06** | **0** | **(31)** | **0.017** | **1** |  | **R.LAADDFR.L** |
|  | 192 | **405.2236** | **808.4326** | **808.4330** | **-0.56** | **0** | **(31)** | **0.012** | **1** |  | **R.LASYLDK.V** |
|  | 193 | **405.2237** | **808.4329** | **808.4330** | **-0.19** | **0** | **43** | **0.00066** | **1** |  | **R.LASYLDK.V** |
|  | 394 | **424.2292** | **846.4439** | **846.4447** | **-0.93** | **0** | **(27)** | **0.043** | **1** | **U** | **K.SEITELR.R** |
|  | 395 | **424.2293** | **846.4441** | **846.4447** | **-0.64** | **0** | **28** | **0.033** | **1** | **U** | **K.SEITELR.R** |
|  | 918 | **497.2536** | **992.4926** | **992.4927** | **-0.09** | **0** | **(38)** | **0.0027** | **1** | **U** | **K.YENEVALR.Q** |
|  | 919 | **497.2536** | **992.4927** | **992.4927** | **0.03** | **0** | **56** | **4.5e-005** | **1** | **U** | **K.YENEVALR.Q** |
|  | 927 | **498.2632** | **994.5119** | **994.5123** | **-0.48** | **1** | **34** | **0.0059** | **1** | **U** | **K.IKEWYEK.H** |
|  | 928 | **498.2638** | **994.5130** | **994.5123** | **0.63** | **1** | **(28)** | **0.022** | **1** | **U** | **K.IKEWYEK.H** |
|  | 953 | **502.2801** | **1002.5456** | **1002.5458** | **-0.15** | **1** | **16** | **0.44** | **1** | **U** | **K.SEITELRR.N** |
|  | 1073 | **516.3016** | **1030.5886** | **1030.5910** | **-2.36** | **0** | **41** | **0.00086** | **1** | **U** | **R.VLDELTLTK.A** |
|  | 1074 | **516.3024** | **1030.5902** | **1030.5910** | **-0.81** | **0** | **(26)** | **0.029** | **1** | **U** | **R.VLDELTLTK.A** |
|  | 1075 | **516.3027** | **1030.5909** | **1030.5910** | **-0.11** | **0** | **(33)** | **0.0051** | **1** | **U** | **R.VLDELTLTK.A** |
|  | 1267 | **355.5415** | **1063.6028** | **1063.6026** | **0.18** | **1** | **49** | **9.1e-005** | **1** |  | **R.LASYLDKVR.A** |
|  | 1268 | **532.8089** | **1063.6032** | **1063.6026** | **0.64** | **1** | **(45)** | **0.00026** | **1** |  | **R.LASYLDKVR.A** |
|  | 1392 | **545.7685** | **1089.5224** | **1089.5237** | **-1.15** | **0** | **(51)** | **0.00012** | **1** |  | **K.VTMQNLNDR.L** |
|  | 1393 | **545.7700** | **1089.5254** | **1089.5237** | **1.55** | **0** | **51** | **0.00012** | **1** |  | **K.VTMQNLNDR.L** |
|  | 1482 | **555.2471** | **1108.4796** | **1108.4825** | **-2.64** | **0** | **43** | **0.00033** | **1** |  | **K.DAEAWFNEK.S** |
|  | 1483 | **555.2486** | **1108.4826** | **1108.4825** | **0.12** | **0** | **(26)** | **0.017** | **1** |  | **K.DAEAWFNEK.S** |
|  | 1718 | **583.2947** | **1164.5748** | **1164.5775** | **-2.28** | **0** | **57** | **3e-005** | **1** | **U** | **R.LENEIQTYR.S** |
|  | 1814 | **594.3522** | **1186.6898** | **1186.6921** | **-1.97** | **1** | **(24)** | **0.03** | **1** | **U** | **R.RVLDELTLTK.A** |
|  | 1815 | **396.5710** | **1186.6913** | **1186.6921** | **-0.70** | **1** | **44** | **0.00028** | **1** | **U** | **R.RVLDELTLTK.A** |
|  | 1883 | **601.3116** | **1200.6087** | **1200.6098** | **-0.92** | **0** | **73** | **6.7e-007** | **1** |  | **R.QSVEADINGLR.R** |
|  | 2006 | **412.2310** | **1233.6711** | **1233.6717** | **-0.45** | **1** | **(28)** | **0.019** | **1** | **U** | **R.LKYENEVALR.Q** |
|  | 2007 | **617.8431** | **1233.6716** | **1233.6717** | **-0.07** | **1** | **(37)** | **0.0019** | **1** | **U** | **R.LKYENEVALR.Q** |
|  | 2008 | **617.8431** | **1233.6716** | **1233.6717** | **-0.07** | **1** | **60** | **1.1e-005** | **1** | **U** | **R.LKYENEVALR.Q** |
|  | 2015 | **619.2956** | **1236.5766** | **1236.5775** | **-0.68** | **1** | **66** | **2.7e-006** | **1** | **U** | **R.KDAEAWFNEK.S** |
|  | 2016 | **413.1997** | **1236.5771** | **1236.5775** | **-0.28** | **1** | **(49)** | **0.00015** | **1** | **U** | **R.KDAEAWFNEK.S** |
|  | 2118 | **631.8012** | **1261.5879** | **1261.5899** | **-1.58** | **0** | **(71)** | **8.9e-007** | **1** | **U** | **R.SLLEGEGSSGGGGR.G** |
|  | 2119 | **631.8012** | **1261.5879** | **1261.5899** | **-1.58** | **0** | **74** | **4.6e-007** | **1** | **U** | **R.SLLEGEGSSGGGGR.G** |
|  | 2120 | **631.8019** | **1261.5892** | **1261.5899** | **-0.52** | **0** | **(72)** | **7.1e-007** | **1** | **U** | **R.SLLEGEGSSGGGGR.G** |
|  | 2256 | **650.8010** | **1299.5875** | **1299.5877** | **-0.15** | **1** | **39** | **0.00082** | **1** | **U** | **K.NHEEEMKDLR.N** |
|  | 2257 | **434.2031** | **1299.5876** | **1299.5877** | **-0.11** | **1** | **(5)** | **2** | **1** | **U** | **K.NHEEEMKDLR.N** |
|  | 2258 | **434.2034** | **1299.5884** | **1299.5877** | **0.51** | **1** | **(28)** | **0.011** | **1** | **U** | **K.NHEEEMKDLR.N** |
|  | 2462 | **679.3623** | **1356.7100** | **1356.7110** | **-0.67** | **1** | **(12)** | **0.97** | **4** |  | **R.QSVEADINGLRR.V** |
|  | 2463 | **679.3630** | **1356.7114** | **1356.7110** | **0.34** | **1** | **(20)** | **0.17** | **1** |  | **R.QSVEADINGLRR.V** |
|  | 2464 | **453.2445** | **1356.7116** | **1356.7110** | **0.49** | **1** | **(12)** | **0.97** | **1** |  | **R.QSVEADINGLRR.V** |
|  | 2465 | **453.2445** | **1356.7118** | **1356.7110** | **0.62** | **1** | **(18)** | **0.26** | **2** |  | **R.QSVEADINGLRR.V** |
|  | 2466 | **679.3632** | **1356.7119** | **1356.7110** | **0.69** | **1** | **51** | **0.00013** | **1** |  | **R.QSVEADINGLRR.V** |
|  | 2467 | **453.2449** | **1356.7128** | **1356.7110** | **1.35** | **1** | **(47)** | **0.00032** | **1** |  | **R.QSVEADINGLRR.V** |
|  | 2501 | **683.3198** | **1364.6251** | **1364.6320** | **-5.08** | **0** | **(59)** | **1.5e-005** | **1** | **U** | **R.SQYEQLAEQNR.K** |
|  | 2502 | **683.3228** | **1364.6309** | **1364.6320** | **-0.78** | **0** | **66** | **3e-006** | **1** | **U** | **R.SQYEQLAEQNR.K** |
|  | 2503 | **455.8852** | **1364.6337** | **1364.6320** | **1.20** | **0** | **(29)** | **0.015** | **1** | **U** | **R.SQYEQLAEQNR.K** |
|  | 2543 | **691.3276** | **1380.6406** | **1380.6408** | **-0.17** | **0** | **87** | **2.1e-008** | **1** | **U** | **R.ALEESNYELEGK.I** |
|  | 2544 | **691.3281** | **1380.6416** | **1380.6408** | **0.53** | **0** | **(86)** | **2.5e-008** | **1** | **U** | **R.ALEESNYELEGK.I** |
|  | 2545 | **691.3284** | **1380.6423** | **1380.6408** | **1.06** | **0** | **(48)** | **0.00016** | **1** | **U** | **R.ALEESNYELEGK.I** |
|  | 2586 | **695.8438** | **1389.6729** | **1389.6736** | **-0.44** | **0** | **78** | **2.2e-007** | **1** | **U** | **K.QSLEASLAETEGR.Y** |
|  | 2587 | **695.8442** | **1389.6738** | **1389.6736** | **0.18** | **0** | **(60)** | **1.3e-005** | **1** | **U** | **K.QSLEASLAETEGR.Y** |
|  | 2588 | **464.2322** | **1389.6747** | **1389.6736** | **0.83** | **0** | **(35)** | **0.0044** | **1** | **U** | **K.QSLEASLAETEGR.Y** |
|  | 2758 | **717.8886** | **1433.7627** | **1433.7626** | **0.02** | **1** | **57** | **2.1e-005** | **1** | **U** | **K.IRLENEIQTYR.S** |
|  | 2759 | **478.9282** | **1433.7628** | **1433.7626** | **0.14** | **1** | **(36)** | **0.003** | **1** | **U** | **K.IRLENEIQTYR.S** |
|  | 2914 | **747.3702** | **1492.7259** | **1492.7270** | **-0.70** | **1** | **65** | **5.3e-006** | **1** | **U** | **R.SQYEQLAEQNRK.D** |
|  | 2915 | **747.3704** | **1492.7262** | **1492.7270** | **-0.54** | **1** | **(64)** | **6.1e-006** | **1** | **U** | **R.SQYEQLAEQNRK.D** |
|  | 2916 | **747.3705** | **1492.7264** | **1492.7270** | **-0.38** | **1** | **(20)** | **0.16** | **1** | **U** | **R.SQYEQLAEQNRK.D** |
|  | 2917 | **498.5828** | **1492.7265** | **1492.7270** | **-0.29** | **1** | **(31)** | **0.012** | **1** | **U** | **R.SQYEQLAEQNRK.D** |
|  | 2918 | **498.5829** | **1492.7269** | **1492.7270** | **-0.03** | **1** | **(41)** | **0.0012** | **1** | **U** | **R.SQYEQLAEQNRK.D** |
|  | 2919 | **498.5835** | **1492.7287** | **1492.7270** | **1.14** | **1** | **(15)** | **0.52** | **1** | **U** | **R.SQYEQLAEQNRK.D** |
|  | 3377 | **854.3895** | **1706.7644** | **1706.7649** | **-0.30** | **0** | **108** | **1.3e-010** | **1** | **U** | **K.GSLGGGFSSGGFSGGSFSR.G** |
|  | 3378 | **854.3898** | **1706.7651** | **1706.7649** | **0.12** | **0** | **(51)** | **6.6e-005** | **1** | **U** | **K.GSLGGGFSSGGFSGGSFSR.G** |
|  | 3934 | **998.9893** | **1995.9640** | **1995.9636** | **0.17** | **0** | **24** | **0.046** | **1** | **U** | **K.ELTTEIDNNIEQISSYK.S** |
|  | 4038 | **1041.9840** | **2081.9535** | **2081.9575** | **-1.95** | **0** | **41** | **0.00077** | **1** | **U** | **R.AETECQNTEYQQLLDIK.I** |

  


---

|  |  |
| --- | --- |
| **3.** | sp|P35908|K22E\_HUMAN    **Mass:** 65678    **Score:** 1296   **Matches:** 59(44)  **Sequences:** 31(23)  **emPAI:** 4.52 |
|  | Keratin, type II cytoskeletal 2 epidermal OS=Homo sapiens OX=9606 GN=KRT2 PE=1 SV=2 |

|  |  |
| --- | --- |
|  | Check to include this hit in error tolerant search or archive report |
|  |  |

|  |  |  |  |  |  |  |  |  |  |  |  |
| --- | --- | --- | --- | --- | --- | --- | --- | --- | --- | --- | --- |
|  | **Query** | **Observed** | **Mr(expt)** | **Mr(calc)** | **ppm** | **Miss** | **Score** | **Expect** | **Rank** | **Unique** | **Peptide** |
|  | 248 | **414.2183** | **826.4220** | **826.4225** | **-0.57** | **0** | **(29)** | **0.014** | **1** |  | **K.FASFIDK.V** |
|  | 249 | **414.2186** | **826.4226** | **826.4225** | **0.11** | **0** | **44** | **0.00045** | **1** |  | **K.FASFIDK.V** |
|  | 841 | 487.2693 | 972.5241 | 972.5240 | 0.12 | 0 | 39 | 0.0024 | 1 |  | K.IEISELNR.V |
|  | 842 | 487.2694 | 972.5243 | 972.5240 | 0.30 | 0 | (39) | 0.0026 | 1 |  | K.IEISELNR.V |
|  | 924 | **497.7877** | **993.5608** | **993.5607** | **0.05** | **0** | **18** | **0.17** | **1** | **U** | **R.LQGEIAHVK.K** |
|  | 965 | **503.2367** | **1004.4589** | **1004.4597** | **-0.78** | **0** | **22** | **0.08** | **1** |  | **K.LLEGEECR.M** |
|  | 1093 | **519.2668** | **1036.5191** | **1036.5189** | **0.23** | **0** | **46** | **0.00039** | **1** | **U** | **R.YLDGLTAER.T** |
|  | 1095 | **519.2669** | **1036.5193** | **1036.5189** | **0.35** | **0** | **(22)** | **0.093** | **1** | **U** | **R.YLDGLTAER.T** |
|  | 1284 | 533.7620 | 1065.5094 | 1065.5090 | 0.30 | 1 | 10 | 1.6 | 1 |  | K.YEDEINKR.T |
|  | 1289 | **534.7551** | **1067.4957** | **1067.4957** | **-0.02** | **0** | **40** | **0.001** | **1** | **U** | **K.DVDNAYMIK.V** |
|  | 1290 | **534.7557** | **1067.4968** | **1067.4957** | **1.01** | **0** | **(29)** | **0.011** | **1** | **U** | **K.DVDNAYMIK.V** |
|  | 1358 | **361.5378** | **1081.5917** | **1081.5920** | **-0.31** | **1** | **(31)** | **0.0083** | **1** |  | **K.FASFIDKVR.F** |
|  | 1359 | **541.8038** | **1081.5931** | **1081.5920** | **1.00** | **1** | **50** | **0.00011** | **1** |  | **K.FASFIDKVR.F** |
|  | 1372 | **542.7525** | **1083.4904** | **1083.4906** | **-0.18** | **0** | **(39)** | **0.00097** | **1** | **U** | **K.DVDNAYMIK.V + Oxidation (M)** |
|  | 1473 | **554.2658** | **1106.5171** | **1106.5356** | **-16.75** | **0** | **(1)** | **11** | **2** |  | **K.AQYEEIAQR.S** |
|  | 1475 | **554.2739** | **1106.5333** | **1106.5356** | **-2.07** | **0** | **42** | **0.00091** | **1** |  | **K.AQYEEIAQR.S** |
|  | 1591 | **566.2577** | **1130.5008** | **1130.5026** | **-1.59** | **0** | **(14)** | **0.24** | **1** | **U** | **R.STSSFSCLSR.H** |
|  | 1592 | **566.2586** | **1130.5027** | **1130.5026** | **0.04** | **0** | **31** | **0.0052** | **1** | **U** | **R.STSSFSCLSR.H** |
|  | 1604 | **567.2844** | **1132.5542** | **1132.5546** | **-0.40** | **1** | **20** | **0.17** | **1** |  | **R.KLLEGEECR.M** |
|  | 1605 | **378.5254** | **1132.5543** | **1132.5546** | **-0.33** | **1** | **(18)** | **0.29** | **1** |  | **R.KLLEGEECR.M** |
|  | 1625 | **570.2732** | **1138.5318** | **1138.5328** | **-0.89** | **0** | **29** | **0.015** | **1** |  | **R.DYQELMNVK.L** |
|  | 1680 | **578.2709** | **1154.5272** | **1154.5278** | **-0.49** | **0** | **(19)** | **0.12** | **1** |  | **R.DYQELMNVK.L + Oxidation (M)** |
|  | 1838 | **597.3115** | **1192.6084** | **1192.6088** | **-0.36** | **0** | **62** | **1.1e-005** | **1** | **U** | **K.YEELQVTVGR.H** |
|  | 1839 | **597.3119** | **1192.6092** | **1192.6088** | **0.36** | **0** | **(60)** | **1.8e-005** | **1** | **U** | **K.YEELQVTVGR.H** |
|  | 1860 | **598.8033** | **1195.5920** | **1195.5907** | **1.11** | **1** | **54** | **5.9e-005** | **1** | **U** | **K.KDVDNAYMIK.V** |
|  | 1916 | **604.8113** | **1207.6080** | **1207.6085** | **-0.37** | **0** | **(66)** | **4.2e-006** | **1** | **U** | **R.TAAENDFVTLK.K** |
|  | 1917 | **604.8114** | **1207.6082** | **1207.6085** | **-0.17** | **0** | **71** | **1.2e-006** | **1** | **U** | **R.TAAENDFVTLK.K** |
|  | 1934 | **606.8001** | **1211.5855** | **1211.5856** | **-0.04** | **1** | **(12)** | **0.73** | **1** | **U** | **K.KDVDNAYMIK.V + Oxidation (M)** |
|  | 2077 | **627.8060** | **1253.5975** | **1253.6001** | **-2.03** | **0** | **89** | **1.4e-008** | **1** | **U** | **R.GFSSGSAVVSGGSR.R** |
|  | 2078 | **627.8063** | **1253.5980** | **1253.6001** | **-1.65** | **0** | **(71)** | **1e-006** | **1** | **U** | **R.GFSSGSAVVSGGSR.R** |
|  | 2335 | **660.7931** | **1319.5716** | **1319.5756** | **-3.02** | **0** | **77** | **8.1e-008** | **1** | **U** | **R.HGGGGGGFGGGGFGSR.S** |
|  | 2336 | **440.8648** | **1319.5725** | **1319.5756** | **-2.35** | **0** | **(38)** | **0.00062** | **1** | **U** | **R.HGGGGGGFGGGGFGSR.S** |
|  | 2337 | **660.7944** | **1319.5743** | **1319.5756** | **-0.98** | **0** | **(29)** | **0.006** | **1** | **U** | **R.HGGGGGGFGGGGFGSR.S** |
|  | 2338 | **440.8655** | **1319.5748** | **1319.5756** | **-0.62** | **0** | **(34)** | **0.0018** | **1** | **U** | **R.HGGGGGGFGGGGFGSR.S** |
|  | 2362 | **665.3232** | **1328.6318** | **1328.6320** | **-0.17** | **0** | **(62)** | **8.2e-006** | **1** | **U** | **K.NVQDAIADAEQR.G** |
|  | 2363 | **665.3237** | **1328.6328** | **1328.6320** | **0.57** | **0** | **68** | **1.9e-006** | **1** | **U** | **K.NVQDAIADAEQR.G** |
|  | 2364 | **665.3669** | **1328.7193** | **1328.7187** | **0.46** | **0** | **23** | **0.087** | **1** |  | **R.NLDLDSIIAEVK.A** |
|  | 2383 | **668.8591** | **1335.7037** | **1335.7034** | **0.22** | **1** | **74** | **4.6e-007** | **1** | **U** | **R.TAAENDFVTLKK.D** |
|  | 2384 | **446.2419** | **1335.7037** | **1335.7034** | **0.23** | **1** | **(48)** | **0.0002** | **1** | **U** | **R.TAAENDFVTLKK.D** |
|  | 2413 | **448.5887** | **1342.7443** | **1342.7456** | **-0.95** | **1** | **(42)** | **0.00063** | **1** | **U** | **K.EIKIEISELNR.V** |
|  | 2414 | **672.3803** | **1342.7461** | **1342.7456** | **0.37** | **1** | **44** | **0.00039** | **1** | **U** | **K.EIKIEISELNR.V** |
|  | 2520 | **686.3588** | **1370.7030** | **1370.7041** | **-0.84** | **0** | **(66)** | **3.7e-006** | **1** | **U** | **K.LNDLEEALQQAK.E** |
|  | 2521 | **686.3596** | **1370.7047** | **1370.7041** | **0.42** | **0** | **71** | **1.2e-006** | **1** | **U** | **K.LNDLEEALQQAK.E** |
|  | 2592 | **696.3434** | **1390.6722** | **1390.6728** | **-0.43** | **1** | **77** | **3.1e-007** | **1** | **U** | **R.SKEEAEALYHSK.Y** |
|  | 2593 | **464.5648** | **1390.6726** | **1390.6728** | **-0.13** | **1** | **(15)** | **0.45** | **1** | **U** | **R.SKEEAEALYHSK.Y** |
|  | 2594 | **464.5649** | **1390.6729** | **1390.6728** | **0.07** | **1** | **(41)** | **0.0011** | **1** | **U** | **R.SKEEAEALYHSK.Y** |
|  | 2595 | **696.3438** | **1390.6729** | **1390.6728** | **0.10** | **1** | **(41)** | **0.0012** | **1** | **U** | **R.SKEEAEALYHSK.Y** |
|  | 2661 | **470.9077** | **1409.7013** | **1409.7012** | **0.10** | **1** | **13** | **0.72** | **1** | **U** | **R.GFSSGSAVVSGGSRR.S** |
|  | 2842 | **730.9031** | **1459.7917** | **1459.7922** | **-0.34** | **0** | **22** | **0.07** | **1** | **U** | **K.VDLLNQEIEFLK.V** |
|  | 2870 | 738.3964 | 1474.7782 | 1474.7780 | 0.12 | 0 | (49) | 0.00019 | 1 |  | R.FLEQQNQVLQTK.W |
|  | 2871 | 738.3976 | 1474.7806 | 1474.7780 | 1.78 | 0 | (64) | 6e-006 | 1 |  | R.FLEQQNQVLQTK.W |
|  | 2872 | 738.3978 | 1474.7810 | 1474.7780 | 2.04 | 0 | 69 | 1.8e-006 | 1 |  | R.FLEQQNQVLQTK.W |
|  | 2980 | **507.9410** | **1520.8011** | **1520.8021** | **-0.63** | **1** | **15** | **0.39** | **1** |  | **R.LLRDYQELMNVK.L** |
|  | 3192 | **807.4268** | **1612.8391** | **1612.8420** | **-1.80** | **1** | **57** | **2.3e-005** | **1** | **U** | **R.NKLNDLEEALQQAK.E** |
|  | 3193 | **538.6212** | **1612.8418** | **1612.8420** | **-0.10** | **1** | **(40)** | **0.0013** | **1** | **U** | **R.NKLNDLEEALQQAK.E** |
|  | 3445 | **871.3776** | **1740.7407** | **1740.7412** | **-0.31** | **0** | **24** | **0.012** | **1** | **U** | **R.GGSGGGGSISGGGYGSGGGSGGR.Y** |
|  | 4083 | **1072.4773** | **2142.9400** | **2142.9375** | **1.17** | **0** | **61** | **4.2e-006** | **1** | **U** | **R.TSQNSELNNMQDLVEDYK.K + Oxidation (M)** |
|  | 4207 | **752.6853** | **2255.0341** | **2255.0376** | **-1.54** | **1** | **60** | **9.4e-006** | **1** | **U** | **R.TSQNSELNNMQDLVEDYKK.K** |
|  | 4230 | **758.0184** | **2271.0335** | **2271.0325** | **0.44** | **1** | **(36)** | **0.0017** | **1** | **U** | **R.TSQNSELNNMQDLVEDYKK.K + Oxidation (M)** |

  


---

|  |  |
| --- | --- |
| **4.** | sp|P35527|K1C9\_HUMAN    **Mass:** 62255    **Score:** 819    **Matches:** 38(33)  **Sequences:** 17(16)  **emPAI:** 2.44 |
|  | Keratin, type I cytoskeletal 9 OS=Homo sapiens OX=9606 GN=KRT9 PE=1 SV=3 |

|  |  |
| --- | --- |
|  | Check to include this hit in error tolerant search or archive report |
|  |  |

|  |  |  |  |  |  |  |  |  |  |  |  |
| --- | --- | --- | --- | --- | --- | --- | --- | --- | --- | --- | --- |
|  | **Query** | **Observed** | **Mr(expt)** | **Mr(calc)** | **ppm** | **Miss** | **Score** | **Expect** | **Rank** | **Unique** | **Peptide** |
|  | 192 | 405.2236 | 808.4326 | 808.4330 | -0.56 | 0 | (31) | 0.012 | 1 |  | R.LASYLDK.V |
|  | 193 | 405.2237 | 808.4329 | 808.4330 | -0.19 | 0 | 43 | 0.00066 | 1 |  | R.LASYLDK.V |
|  | 561 | **449.2101** | **896.4057** | **896.4062** | **-0.53** | **0** | **40** | **0.00074** | **1** | **U** | **R.MTLDDFR.I** |
|  | 562 | **449.2104** | **896.4063** | **896.4062** | **0.09** | **0** | **(36)** | **0.0022** | **1** | **U** | **R.MTLDDFR.I** |
|  | 625 | **457.2076** | **912.4007** | **912.4011** | **-0.50** | **0** | **(27)** | **0.0084** | **1** | **U** | **R.MTLDDFR.I + Oxidation (M)** |
|  | 626 | **457.2084** | **912.4023** | **912.4011** | **1.32** | **0** | **(19)** | **0.052** | **1** | **U** | **R.MTLDDFR.I + Oxidation (M)** |
|  | 820 | **484.2299** | **966.4453** | **966.4447** | **0.62** | **0** | **33** | **0.0057** | **1** | **U** | **K.IQDWYDK.K** |
|  | 821 | **484.2302** | **966.4458** | **966.4447** | **1.12** | **0** | **(8)** | **1.7** | **1** | **U** | **K.IQDWYDK.K** |
|  | 1247 | **530.7851** | **1059.5556** | **1059.5560** | **-0.36** | **0** | **46** | **0.00074** | **1** | **U** | **K.TLLDIDNTR.M** |
|  | 1248 | **530.7852** | **1059.5559** | **1059.5560** | **-0.13** | **0** | **(39)** | **0.0031** | **1** | **U** | **K.TLLDIDNTR.M** |
|  | 1282 | **533.7532** | **1065.4919** | **1065.4913** | **0.57** | **0** | **45** | **0.00038** | **1** | **U** | **K.FEMEQNLR.Q** |
|  | 1355 | **541.7505** | **1081.4865** | **1081.4862** | **0.30** | **0** | **(36)** | **0.0025** | **1** | **U** | **K.FEMEQNLR.Q + Oxidation (M)** |
|  | 1423 | **365.8537** | **1094.5392** | **1094.5396** | **-0.42** | **1** | **(21)** | **0.12** | **1** | **U** | **K.IQDWYDKK.G** |
|  | 1424 | **548.2772** | **1094.5398** | **1094.5396** | **0.12** | **1** | **52** | **9.5e-005** | **1** | **U** | **K.IQDWYDKK.G** |
|  | 1540 | **561.2957** | **1120.5767** | **1120.5764** | **0.31** | **0** | **49** | **0.00023** | **1** | **U** | **R.QEYEQLIAK.N** |
|  | 1541 | **561.2963** | **1120.5780** | **1120.5764** | **1.40** | **0** | **(36)** | **0.0036** | **1** | **U** | **R.QEYEQLIAK.N** |
|  | 1685 | **579.2976** | **1156.5807** | **1156.5836** | **-2.58** | **0** | **(67)** | **2.5e-006** | **1** | **U** | **R.QGVDADINGLR.Q** |
|  | 1686 | **579.2987** | **1156.5829** | **1156.5836** | **-0.68** | **0** | **70** | **1.3e-006** | **1** | **U** | **R.QGVDADINGLR.Q** |
|  | 1822 | **595.8074** | **1189.6002** | **1189.6013** | **-0.90** | **0** | **(38)** | **0.0032** | **1** | **U** | **R.QVLDNLTMEK.S** |
|  | 1823 | **595.8080** | **1189.6015** | **1189.6013** | **0.23** | **0** | **(39)** | **0.0023** | **1** | **U** | **R.QVLDNLTMEK.S** |
|  | 1910 | **603.8062** | **1205.5977** | **1205.5962** | **1.30** | **0** | **(32)** | **0.011** | **1** | **U** | **R.QVLDNLTMEK.S + Oxidation (M)** |
|  | 1911 | **603.8066** | **1205.5986** | **1205.5962** | **2.02** | **0** | **44** | **0.00075** | **1** | **U** | **R.QVLDNLTMEK.S + Oxidation (M)** |
|  | 1995 | **616.8027** | **1231.5909** | **1231.5906** | **0.28** | **0** | **64** | **5.1e-006** | **1** | **U** | **R.SGGGGGGGLGSGGSIR.S** |
|  | 2009 | **618.2680** | **1234.5215** | **1234.5215** | **0.01** | **0** | **26** | **0.0086** | **1** | **U** | **R.FSSSSGYGGGSSR.V** |
|  | 2282 | **436.5640** | **1306.6701** | **1306.6703** | **-0.20** | **1** | **(20)** | **0.14** | **1** | **U** | **R.IKFEMEQNLR.Q** |
|  | 2283 | **654.3425** | **1306.6705** | **1306.6703** | **0.14** | **1** | **(44)** | **0.00059** | **1** | **U** | **R.IKFEMEQNLR.Q** |
|  | 2284 | **654.3426** | **1306.6706** | **1306.6703** | **0.24** | **1** | **(37)** | **0.0028** | **1** | **U** | **R.IKFEMEQNLR.Q** |
|  | 2312 | **658.3469** | **1314.6793** | **1314.6779** | **1.02** | **0** | **68** | **2.7e-006** | **1** | **U** | **K.DQIVDLTVGNNK.T** |
|  | 2349 | **662.3392** | **1322.6638** | **1322.6652** | **-1.10** | **1** | **(11)** | **1.1** | **2** | **U** | **R.IKFEMEQNLR.Q + Oxidation (M)** |
|  | 2350 | **662.3397** | **1322.6649** | **1322.6652** | **-0.26** | **1** | **(29)** | **0.017** | **1** | **U** | **R.IKFEMEQNLR.Q + Oxidation (M)** |
|  | 2351 | **441.8957** | **1322.6653** | **1322.6652** | **0.07** | **1** | **58** | **2.1e-005** | **1** | **U** | **R.IKFEMEQNLR.Q + Oxidation (M)** |
|  | 3140 | **793.8859** | **1585.7573** | **1585.7583** | **-0.65** | **0** | **(74)** | **4.9e-007** | **1** | **U** | **K.VQALEEANNDLENK.I** |
|  | 3141 | **529.5934** | **1585.7585** | **1585.7583** | **0.10** | **0** | **(40)** | **0.0013** | **1** | **U** | **K.VQALEEANNDLENK.I** |
|  | 3142 | **793.8873** | **1585.7601** | **1585.7583** | **1.12** | **0** | **83** | **6.6e-008** | **1** | **U** | **K.VQALEEANNDLENK.I** |
|  | 3569 | **896.3667** | **1790.7188** | **1790.7205** | **-0.92** | **0** | **8** | **0.21** | **1** | **U** | **R.GGSGGSYGGGGSGGGYGGGSGSR.G** |
|  | 3686 | **613.3246** | **1836.9519** | **1836.9581** | **-3.38** | **0** | **32** | **0.0079** | **1** | **U** | **R.HGVQELEIELQSQLSK.K** |
|  | 4374 | **1255.5685** | **2509.1224** | **2509.1245** | **-0.82** | **0** | **(56)** | **9.8e-006** | **1** | **U** | **K.EIETYHNLLEGGQEDFESSGAGK.I** |
|  | 4375 | **837.3822** | **2509.1248** | **2509.1245** | **0.12** | **0** | **84** | **1.8e-008** | **1** | **U** | **K.EIETYHNLLEGGQEDFESSGAGK.I** |

  


---

|  |  |
| --- | --- |
| **5.** | sp|P67809|YBOX1\_HUMAN    **Mass:** 35903    **Score:** 767    **Matches:** 36(22)  **Sequences:** 12(10)  **emPAI:** 3.50 |
|  | Nuclease-sensitive element-binding protein 1 OS=Homo sapiens OX=9606 GN=YBX1 PE=1 SV=3 |

|  |  |
| --- | --- |
|  | Check to include this hit in error tolerant search or archive report |
|  |  |

|  |  |  |  |  |  |  |  |  |  |  |  |
| --- | --- | --- | --- | --- | --- | --- | --- | --- | --- | --- | --- |
|  | **Query** | **Observed** | **Mr(expt)** | **Mr(calc)** | **ppm** | **Miss** | **Score** | **Expect** | **Rank** | **Unique** | **Peptide** |
|  | 23 | **361.1921** | **720.3697** | **720.3707** | **-1.49** | **0** | **23** | **0.12** | **1** |  | **K.WFNVR.N** |
|  | 24 | **361.1923** | **720.3700** | **720.3707** | **-0.99** | **0** | **(14)** | **0.87** | **1** |  | **K.WFNVR.N** |
|  | 734 | **470.7356** | **939.4566** | **939.4563** | **0.36** | **0** | **50** | **0.00013** | **1** |  | **R.NGYGFINR.N** |
|  | 834 | **487.2329** | **972.4512** | **972.4527** | **-1.58** | **0** | **(7)** | **1.6** | **7** | **U** | **R.FPPYYMR.R** |
|  | 836 | **487.2335** | **972.4525** | **972.4527** | **-0.26** | **0** | **(18)** | **0.12** | **1** | **U** | **R.FPPYYMR.R** |
|  | 837 | **487.2337** | **972.4528** | **972.4527** | **0.05** | **0** | **26** | **0.019** | **1** | **U** | **R.FPPYYMR.R** |
|  | 839 | **487.2338** | **972.4530** | **972.4527** | **0.31** | **0** | **(13)** | **0.4** | **2** | **U** | **R.FPPYYMR.R** |
|  | 840 | **487.2341** | **972.4536** | **972.4527** | **0.87** | **0** | **(13)** | **0.4** | **1** | **U** | **R.FPPYYMR.R** |
|  | 896 | **495.2318** | **988.4491** | **988.4477** | **1.48** | **0** | **(8)** | **1.4** | **2** | **U** | **R.FPPYYMR.R + Oxidation (M)** |
|  | 1579 | **377.1914** | **1128.5524** | **1128.5538** | **-1.28** | **1** | **(4)** | **4.9** | **2** | **U** | **R.RFPPYYMR.R** |
|  | 1580 | **565.2839** | **1128.5533** | **1128.5538** | **-0.46** | **1** | **12** | **0.77** | **1** | **U** | **R.RFPPYYMR.R** |
|  | 2211 | **643.8401** | **1285.6657** | **1285.6667** | **-0.71** | **0** | **45** | **0.00058** | **1** |  | **K.EDVFVHQTAIK.K** |
|  | 3345 | **848.4352** | **1694.8559** | **1694.8588** | **-1.68** | **0** | **91** | **1.1e-008** | **1** | **U** | **K.GAEAANVTGPGGVPVQGSK.Y** |
|  | 3346 | **848.4358** | **1694.8571** | **1694.8588** | **-0.96** | **0** | **(73)** | **7.4e-007** | **1** | **U** | **K.GAEAANVTGPGGVPVQGSK.Y** |
|  | 3347 | **565.9598** | **1694.8577** | **1694.8588** | **-0.64** | **0** | **(46)** | **0.00034** | **1** | **U** | **K.GAEAANVTGPGGVPVQGSK.Y** |
|  | 3457 | **582.3002** | **1743.8789** | **1743.8792** | **-0.17** | **1** | **(39)** | **0.0017** | **1** |  | **R.NDTKEDVFVHQTAIK.K** |
|  | 3458 | **582.3002** | **1743.8789** | **1743.8792** | **-0.17** | **1** | **(49)** | **0.00019** | **1** |  | **R.NDTKEDVFVHQTAIK.K** |
|  | 3459 | **872.9470** | **1743.8794** | **1743.8792** | **0.12** | **1** | **86** | **3.3e-008** | **1** |  | **R.NDTKEDVFVHQTAIK.K** |
|  | 3600 | **898.4061** | **1794.7976** | **1794.8160** | **-10.25** | **0** | **(1)** | **6.2** | **5** |  | **R.SVGDGETVEFDVVEGEK.G** |
|  | 3601 | **898.4174** | **1794.8203** | **1794.8160** | **2.40** | **0** | **40** | **0.00086** | **1** |  | **R.SVGDGETVEFDVVEGEK.G** |
|  | 3803 | **633.2725** | **1896.7956** | **1896.7973** | **-0.92** | **0** | **(28)** | **0.0044** | **1** | **U** | **K.AADPPAENSSAPEAEQGGAE.-** |
|  | 3804 | **949.4056** | **1896.7967** | **1896.7973** | **-0.30** | **0** | **53** | **1.5e-005** | **1** | **U** | **K.AADPPAENSSAPEAEQGGAE.-** |
|  | 3805 | **949.4061** | **1896.7977** | **1896.7973** | **0.21** | **0** | **(43)** | **0.00013** | **1** | **U** | **K.AADPPAENSSAPEAEQGGAE.-** |
|  | 4407 | **876.7055** | **2627.0947** | **2627.0968** | **-0.78** | **1** | **(26)** | **0.004** | **1** | **U** | **R.EDGNEEDKENQGDETQGQQPPQR.R** |
|  | 4408 | **1314.5547** | **2627.0948** | **2627.0968** | **-0.73** | **1** | **(7)** | **0.27** | **2** | **U** | **R.EDGNEEDKENQGDETQGQQPPQR.R** |
|  | 4409 | **876.7056** | **2627.0951** | **2627.0968** | **-0.64** | **1** | **78** | **2.5e-008** | **1** | **U** | **R.EDGNEEDKENQGDETQGQQPPQR.R** |
|  | 4410 | **657.7812** | **2627.0957** | **2627.0968** | **-0.42** | **1** | **(20)** | **0.015** | **1** | **U** | **R.EDGNEEDKENQGDETQGQQPPQR.R** |
|  | 4493 | **806.6378** | **3222.5219** | **3222.5225** | **-0.16** | **0** | **(58)** | **8.6e-006** | **1** | **U** | **R.RPQYSNPPVQGEVMEGADNQGAGEQGRPVR.Q** |
|  | 4494 | **1075.1816** | **3222.5231** | **3222.5225** | **0.20** | **0** | **(16)** | **0.14** | **1** | **U** | **R.RPQYSNPPVQGEVMEGADNQGAGEQGRPVR.Q** |
|  | 4495 | **806.6381** | **3222.5231** | **3222.5225** | **0.21** | **0** | **(53)** | **3e-005** | **1** | **U** | **R.RPQYSNPPVQGEVMEGADNQGAGEQGRPVR.Q** |
|  | 4496 | **1075.1824** | **3222.5253** | **3222.5225** | **0.88** | **0** | **(23)** | **0.026** | **1** | **U** | **R.RPQYSNPPVQGEVMEGADNQGAGEQGRPVR.Q** |
|  | 4498 | **1080.5065** | **3238.4977** | **3238.5174** | **-6.07** | **0** | **(19)** | **0.062** | **1** | **U** | **R.RPQYSNPPVQGEVMEGADNQGAGEQGRPVR.Q + Oxidation (M)** |
|  | 4499 | **810.6343** | **3238.5083** | **3238.5174** | **-2.82** | **0** | **78** | **9.9e-008** | **1** | **U** | **R.RPQYSNPPVQGEVMEGADNQGAGEQGRPVR.Q + Oxidation (M)** |
|  | 4501 | **1086.4703** | **3256.3892** | **3256.3889** | **0.10** | **1** | **116** | **4.5e-012** | **1** | **U** | **R.NYQQNYQNSESGEKNEGSESAPEGQAQQR.R** |
|  | 4502 | **1086.4727** | **3256.3962** | **3256.3889** | **2.24** | **1** | **(31)** | **0.0016** | **1** | **U** | **R.NYQQNYQNSESGEKNEGSESAPEGQAQQR.R** |
|  | 4503 | **815.1063** | **3256.3962** | **3256.3889** | **2.24** | **1** | **(8)** | **0.27** | **1** | **U** | **R.NYQQNYQNSESGEKNEGSESAPEGQAQQR.R** |

  


---

|  |  |
| --- | --- |
| **6.** | sp|P02533|K1C14\_HUMAN    **Mass:** 51872    **Score:** 688    **Matches:** 37(25)  **Sequences:** 25(18)  **emPAI:** 2.43 |
|  | Keratin, type I cytoskeletal 14 OS=Homo sapiens OX=9606 GN=KRT14 PE=1 SV=4 |

|  |  |
| --- | --- |
|  | Check to include this hit in error tolerant search or archive report |
|  |  |

|  |  |  |  |  |  |  |  |  |  |  |  |
| --- | --- | --- | --- | --- | --- | --- | --- | --- | --- | --- | --- |
|  | **Query** | **Observed** | **Mr(expt)** | **Mr(calc)** | **ppm** | **Miss** | **Score** | **Expect** | **Rank** | **Unique** | **Peptide** |
|  | 1 | **350.7339** | **699.4532** | **699.4531** | **0.15** | **0** | **40** | **0.00068** | **1** |  | **K.ILLDVK.T** |
|  | 134 | **388.2085** | **774.4024** | **774.4058** | **-4.30** | **0** | **19** | **0.44** | **2** |  | **R.ILNEMR.D** |
|  | 189 | 404.2032 | 806.3918 | 806.3923 | -0.61 | 0 | 43 | 0.0011 | 1 |  | R.LAADDFR.T |
|  | 190 | 404.2034 | 806.3923 | 806.3923 | 0.06 | 0 | (31) | 0.017 | 1 |  | R.LAADDFR.T |
|  | 192 | 405.2236 | 808.4326 | 808.4330 | -0.56 | 0 | (31) | 0.012 | 1 |  | R.LASYLDK.V |
|  | 193 | 405.2237 | 808.4329 | 808.4330 | -0.19 | 0 | 43 | 0.00066 | 1 |  | R.LASYLDK.V |
|  | 654 | **460.2129** | **918.4113** | **918.4123** | **-1.13** | **0** | **8** | **1.2** | **1** |  | **K.DYSPYFK.T** |
|  | 1066 | **515.3005** | **1028.5865** | **1028.5866** | **-0.06** | **0** | **43** | **0.00066** | **1** |  | **R.VLDELTLAR.A** |
|  | 1067 | **515.3011** | **1028.5876** | **1028.5866** | **1.01** | **0** | **(23)** | **0.063** | **1** |  | **R.VLDELTLAR.A** |
|  | 1092 | **518.7702** | **1035.5258** | **1035.5250** | **0.82** | **1** | **17** | **0.35** | **1** |  | **K.IRDWYQR.Q** |
|  | 1093 | 519.2668 | 1036.5191 | 1036.5189 | 0.25 | 0 | (2) | 11 | 5 | U | K.YETELNLR.M |
|  | 1094 | **519.2668** | **1036.5191** | **1036.5189** | **0.25** | **0** | **36** | **0.0038** | **1** | **U** | **K.YETELNLR.M** |
|  | 1095 | 519.2669 | 1036.5193 | 1036.5189 | 0.36 | 0 | (1) | 11 | 7 | U | K.YETELNLR.M |
|  | 1267 | 355.5415 | 1063.6028 | 1063.6026 | 0.18 | 1 | 49 | 9.1e-005 | 1 |  | R.LASYLDKVR.A |
|  | 1268 | 532.8089 | 1063.6032 | 1063.6026 | 0.64 | 1 | (45) | 0.00026 | 1 |  | R.LASYLDKVR.A |
|  | 1392 | 545.7685 | 1089.5224 | 1089.5237 | -1.15 | 0 | (51) | 0.00012 | 1 |  | K.VTMQNLNDR.L |
|  | 1393 | 545.7700 | 1089.5254 | 1089.5237 | 1.55 | 0 | 51 | 0.00012 | 1 |  | K.VTMQNLNDR.L |
|  | 1467 | **553.7848** | **1105.5550** | **1105.5550** | **0.03** | **0** | **59** | **2.6e-005** | **1** |  | **R.ISSVLAGGSCR.A** |
|  | 1723 | **584.3278** | **1166.6410** | **1166.6408** | **0.16** | **0** | **36** | **0.0026** | **1** | **U** | **K.VVSTHEQVLR.T** |
|  | 1753 | **586.7665** | **1171.5185** | **1171.5186** | **-0.05** | **0** | **44** | **0.00028** | **1** | **U** | **K.DAEEWFFTK.T** |
|  | 1809 | **395.9031** | **1184.6873** | **1184.6877** | **-0.32** | **1** | **2** | **6.4** | **4** |  | **R.RVLDELTLAR.A** |
|  | 1810 | **395.9031** | **1184.6873** | **1184.6877** | **-0.32** | **1** | **(1)** | **7.2** | **3** |  | **R.RVLDELTLAR.A** |
|  | 1907 | **602.8027** | **1203.5909** | **1203.5918** | **-0.70** | **0** | **45** | **0.00053** | **1** |  | **R.MSVEADINGLR.R** |
|  | 1952 | **610.8038** | **1219.5931** | **1219.5932** | **-0.05** | **0** | **56** | **4.8e-005** | **1** |  | **K.ASLENSLEETK.G** |
|  | 2038 | **621.7800** | **1241.5454** | **1241.5458** | **-0.36** | **0** | **(25)** | **0.018** | **1** |  | **K.NHEEEMNALR.G** |
|  | 2039 | **621.7800** | **1241.5455** | **1241.5458** | **-0.26** | **0** | **47** | **0.00011** | **1** |  | **K.NHEEEMNALR.G** |
|  | 2040 | **414.8558** | **1241.5457** | **1241.5458** | **-0.14** | **0** | **(41)** | **0.00046** | **1** |  | **K.NHEEEMNALR.G** |
|  | 2174 | **639.7949** | **1277.5753** | **1277.5783** | **-2.34** | **0** | **32** | **0.0071** | **1** |  | **K.GSCGIGGGIGGGSSR.I** |
|  | 2264 | **651.3348** | **1300.6550** | **1300.6510** | **3.07** | **0** | **80** | **1.6e-007** | **1** |  | **R.ALEEANADLEVK.I** |
|  | 2484 | **680.8541** | **1359.6936** | **1359.6929** | **0.52** | **1** | **11** | **1.5** | **2** |  | **R.MSVEADINGLRR.V** |
|  | 2489 | **681.3486** | **1360.6827** | **1360.6834** | **-0.52** | **0** | **(77)** | **3.8e-007** | **1** |  | **R.EVATNSELVQSGK.S** |
|  | 2490 | **681.3488** | **1360.6829** | **1360.6834** | **-0.34** | **0** | **84** | **7.8e-008** | **1** |  | **R.EVATNSELVQSGK.S** |
|  | 2518 | **457.5541** | **1369.6406** | **1369.6408** | **-0.15** | **1** | **8** | **1.8** | **1** |  | **K.KNHEEEMNALR.G** |
|  | 2539 | **460.5807** | **1378.7202** | **1378.7204** | **-0.21** | **1** | **57** | **3.4e-005** | **1** |  | **K.TRLEQEIATYR.R** |
|  | 2724 | **713.3514** | **1424.6883** | **1424.6896** | **-0.88** | **0** | **64** | **5.8e-006** | **1** | **U** | **R.APSTYGGGLSVSSSR.F** |
|  | 2781 | **480.2378** | **1437.6917** | **1437.6922** | **-0.31** | **1** | **(11)** | **1.1** | **1** |  | **R.ILNEMRDQYEK.M** |
|  | 2824 | **727.8499** | **1453.6853** | **1453.6871** | **-1.25** | **1** | **14** | **0.48** | **1** |  | **R.ILNEMRDQYEK.M + Oxidation (M)** |

  


---

|  |  |
| --- | --- |
| **7.** | sp|P13647|K2C5\_HUMAN    **Mass:** 62568    **Score:** 679    **Matches:** 42(30)  **Sequences:** 30(23)  **emPAI:** 3.64 |
|  | Keratin, type II cytoskeletal 5 OS=Homo sapiens OX=9606 GN=KRT5 PE=1 SV=3 |

|  |  |
| --- | --- |
|  | Check to include this hit in error tolerant search or archive report |
|  |  |

|  |  |  |  |  |  |  |  |  |  |  |  |
| --- | --- | --- | --- | --- | --- | --- | --- | --- | --- | --- | --- |
|  | **Query** | **Observed** | **Mr(expt)** | **Mr(calc)** | **ppm** | **Miss** | **Score** | **Expect** | **Rank** | **Unique** | **Peptide** |
|  | 248 | 414.2183 | 826.4220 | 826.4225 | -0.57 | 0 | (29) | 0.014 | 1 |  | K.FASFIDK.V |
|  | 249 | 414.2186 | 826.4226 | 826.4225 | 0.11 | 0 | 44 | 0.00045 | 1 |  | K.FASFIDK.V |
|  | 637 | **458.7585** | **915.5025** | **915.5025** | **0.01** | **1** | **2** | **15** | **6** | **U** | **R.AEIDNVKK.Q** |
|  | 730 | **469.7523** | **937.4900** | **937.4869** | **3.30** | **0** | **31** | **0.0079** | **1** | **U** | **R.SLYNLGGSK.R** |
|  | 747 | **473.2593** | **944.5039** | **944.5039** | **0.03** | **1** | **34** | **0.0099** | **1** |  | **R.GRLDSELR.N** |
|  | 965 | 503.2367 | 1004.4589 | 1004.4597 | -0.78 | 0 | 22 | 0.08 | 1 |  | K.LLEGEECR.L |
|  | 1006 | **508.7732** | **1015.5318** | **1015.5298** | **1.98** | **0** | **46** | **0.00052** | **1** |  | **R.QLDSIVGER.G** |
|  | 1007 | **508.7740** | **1015.5334** | **1015.5298** | **3.50** | **0** | **(39)** | **0.0022** | **1** |  | **R.QLDSIVGER.G** |
|  | 1044 | **513.7310** | **1025.4475** | **1025.4488** | **-1.26** | **0** | **31** | **0.0057** | **1** |  | **K.DVDAAYMNK.V** |
|  | 1234 | **529.3038** | **1056.5931** | **1056.5927** | **0.36** | **1** | **33** | **0.0068** | **1** | **U** | **R.LRAEIDNVK.K** |
|  | 1284 | 533.7620 | 1065.5094 | 1065.5090 | 0.30 | 1 | 10 | 1.6 | 1 |  | K.YEDEINKR.T |
|  | 1358 | 361.5378 | 1081.5917 | 1081.5920 | -0.31 | 1 | (31) | 0.0083 | 1 |  | K.FASFIDKVR.F |
|  | 1359 | 541.8038 | 1081.5931 | 1081.5920 | 1.00 | 1 | 50 | 0.00011 | 1 |  | K.FASFIDKVR.F |
|  | 1504 | **556.2908** | **1110.5670** | **1110.5669** | **0.04** | **0** | **63** | **6.3e-006** | **1** | **U** | **R.ISISTSGGSFR.N** |
|  | 1604 | 567.2844 | 1132.5542 | 1132.5546 | -0.40 | 1 | 20 | 0.17 | 1 |  | R.KLLEGEECR.L |
|  | 1605 | 378.5254 | 1132.5543 | 1132.5546 | -0.33 | 1 | (18) | 0.29 | 1 |  | R.KLLEGEECR.L |
|  | 1646 | **572.3210** | **1142.6275** | **1142.6183** | **8.11** | **0** | **6** | **4.3** | **4** | **U** | **K.LAELEEALQK.A** |
|  | 1681 | **578.2714** | **1154.5283** | **1154.5277** | **0.47** | **0** | **41** | **0.00077** | **1** | **U** | **R.EYQELMNTK.L** |
|  | 1756 | **586.8228** | **1171.6311** | **1171.6309** | **0.13** | **1** | **28** | **0.025** | **1** |  | **R.RQLDSIVGER.G** |
|  | 1843 | **597.7907** | **1193.5669** | **1193.5676** | **-0.65** | **0** | **(48)** | **0.00024** | **1** | **U** | **K.YEELQQTAGR.H** |
|  | 1844 | **597.7909** | **1193.5672** | **1193.5676** | **-0.35** | **0** | **62** | **8.1e-006** | **1** | **U** | **K.YEELQQTAGR.H** |
|  | 1901 | **602.3210** | **1202.6275** | **1202.6295** | **-1.67** | **0** | **(11)** | **1.6** | **1** |  | **K.WTLLQEQGTK.T** |
|  | 1902 | **602.3215** | **1202.6284** | **1202.6295** | **-0.95** | **0** | **51** | **0.00015** | **1** |  | **K.WTLLQEQGTK.T** |
|  | 2018 | **619.7897** | **1237.5648** | **1237.5649** | **-0.07** | **0** | **(22)** | **0.059** | **1** | **U** | **R.NMQDLVEDFK.N** |
|  | 2041 | **621.7855** | **1241.5565** | **1241.5564** | **0.06** | **0** | **36** | **0.0017** | **1** | **U** | **R.TEAESWYQTK.Y** |
|  | 2075 | **627.7867** | **1253.5589** | **1253.5598** | **-0.69** | **0** | **44** | **0.00031** | **1** | **U** | **R.NMQDLVEDFK.N + Oxidation (M)** |
|  | 2186 | **641.8229** | **1281.6312** | **1281.6275** | **2.92** | **0** | **59** | **1.8e-005** | **1** | **U** | **R.TTAENEFVMLK.K** |
|  | 2243 | **649.8186** | **1297.6226** | **1297.6224** | **0.20** | **0** | **(56)** | **3.3e-005** | **1** | **U** | **R.TTAENEFVMLK.K + Oxidation (M)** |
|  | 2364 | 665.3669 | 1328.7193 | 1328.7187 | 0.46 | 0 | 23 | 0.087 | 1 |  | R.NLDLDSIIAEVK.A |
|  | 2660 | **705.8448** | **1409.6750** | **1409.6722** | **2.03** | **0** | **77** | **2.6e-007** | **1** | **U** | **R.VSLAGACGVGGYGSR.S** |
|  | 2662 | **705.8649** | **1409.7152** | **1409.7151** | **0.09** | **0** | **(19)** | **0.15** | **1** | **U** | **R.SFSTASAITPSVSR.T** |
|  | 2663 | **705.8652** | **1409.7158** | **1409.7151** | **0.52** | **0** | **54** | **5.7e-005** | **1** | **U** | **R.SFSTASAITPSVSR.T** |
|  | 2664 | **470.9150** | **1409.7232** | **1409.7224** | **0.55** | **1** | **(19)** | **0.16** | **1** | **U** | **R.TTAENEFVMLKK.D** |
|  | 2665 | **705.8692** | **1409.7238** | **1409.7224** | **1.01** | **1** | **51** | **9.4e-005** | **1** | **U** | **R.TTAENEFVMLKK.D** |
|  | 2723 | **713.3415** | **1424.6685** | **1424.6680** | **0.42** | **0** | **(25)** | **0.037** | **1** | **U** | **K.VDALMDEINFMK.M** |
|  | 2782 | **720.3594** | **1438.7043** | **1438.7053** | **-0.65** | **0** | **48** | **0.00026** | **1** | **U** | **R.GLGVGFGSGGGSSSSVK.F** |
|  | 2833 | **729.3361** | **1456.6576** | **1456.6578** | **-0.15** | **0** | **45** | **0.00026** | **1** | **U** | **K.VDALMDEINFMK.M + 2 Oxidation (M)** |
|  | 2891 | **743.3520** | **1484.6894** | **1484.6895** | **-0.08** | **1** | **23** | **0.051** | **1** | **U** | **R.SRTEAESWYQTK.Y** |
|  | 3091 | **780.3774** | **1558.7403** | **1558.7344** | **3.81** | **1** | **0** | **11** | **5** | **U** | **K.HEISEMNRMIQR.L + Oxidation (M)** |
|  | 3342 | **848.4156** | **1694.8166** | **1694.8185** | **-1.10** | **1** | **79** | **1.7e-007** | **1** |  | **K.DVDAAYMNKVELEAK.V** |
|  | 3344 | **565.9470** | **1694.8192** | **1694.8185** | **0.43** | **1** | **(31)** | **0.012** | **1** |  | **K.DVDAAYMNKVELEAK.V** |
|  | 3361 | **851.4023** | **1700.7901** | **1700.7900** | **0.08** | **0** | **36** | **0.002** | **1** | **U** | **K.QCANLQNAIADAEQR.G** |

  


---

|  |  |
| --- | --- |
| **8.** | sp|P02538|K2C6A\_HUMAN    **Mass:** 60293    **Score:** 632    **Matches:** 36(28)  **Sequences:** 26(22)  **emPAI:** 3.43 |
|  | Keratin, type II cytoskeletal 6A OS=Homo sapiens OX=9606 GN=KRT6A PE=1 SV=3 |

|  |  |
| --- | --- |
|  | Check to include this hit in error tolerant search or archive report |
|  |  |

|  |  |  |  |  |  |  |  |  |  |  |  |
| --- | --- | --- | --- | --- | --- | --- | --- | --- | --- | --- | --- |
|  | **Query** | **Observed** | **Mr(expt)** | **Mr(calc)** | **ppm** | **Miss** | **Score** | **Expect** | **Rank** | **Unique** | **Peptide** |
|  | 248 | 414.2183 | 826.4220 | 826.4225 | -0.57 | 0 | (29) | 0.014 | 1 |  | K.FASFIDK.V |
|  | 249 | 414.2186 | 826.4226 | 826.4225 | 0.11 | 0 | 44 | 0.00045 | 1 |  | K.FASFIDK.V |
|  | 523 | **441.2439** | **880.4733** | **880.4654** | **8.95** | **0** | **35** | **0.0035** | **1** |  | **R.SLYGLGGSK.R** |
|  | 747 | 473.2593 | 944.5039 | 944.5039 | 0.03 | 1 | 34 | 0.0099 | 1 |  | R.GRLDSELR.G |
|  | 830 | **486.7591** | **971.5036** | **971.5036** | **-0.00** | **0** | **54** | **5.7e-005** | **1** |  | **K.QEIAEINR.M** |
|  | 965 | 503.2367 | 1004.4589 | 1004.4597 | -0.78 | 0 | 22 | 0.08 | 1 |  | K.LLEGEECR.L |
|  | 988 | **506.7556** | **1011.4966** | **1011.4985** | **-1.91** | **0** | **43** | **0.00051** | **1** | **U** | **R.SGFSSVSVSR.S** |
|  | 1006 | 508.7732 | 1015.5318 | 1015.5298 | 1.98 | 0 | 46 | 0.00052 | 1 |  | R.QLDSIVGER.G |
|  | 1007 | 508.7740 | 1015.5334 | 1015.5298 | 3.50 | 0 | (39) | 0.0022 | 1 |  | R.QLDSIVGER.G |
|  | 1044 | 513.7310 | 1025.4475 | 1025.4488 | -1.26 | 0 | 31 | 0.0057 | 1 |  | K.DVDAAYMNK.V |
|  | 1284 | 533.7620 | 1065.5094 | 1065.5090 | 0.30 | 1 | 10 | 1.6 | 1 |  | K.YEDEINKR.T |
|  | 1358 | 361.5378 | 1081.5917 | 1081.5920 | -0.31 | 1 | (31) | 0.0083 | 1 |  | K.FASFIDKVR.F |
|  | 1359 | 541.8038 | 1081.5931 | 1081.5920 | 1.00 | 1 | 50 | 0.00011 | 1 |  | K.FASFIDKVR.F |
|  | 1473 | 554.2658 | 1106.5171 | 1106.5356 | -16.75 | 0 | (1) | 11 | 2 |  | K.AQYEEIAQR.S |
|  | 1475 | 554.2739 | 1106.5333 | 1106.5356 | -2.07 | 0 | 42 | 0.00091 | 1 |  | K.AQYEEIAQR.S |
|  | 1604 | 567.2844 | 1132.5542 | 1132.5546 | -0.40 | 1 | 20 | 0.17 | 1 |  | R.KLLEGEECR.L |
|  | 1605 | 378.5254 | 1132.5543 | 1132.5546 | -0.33 | 1 | (18) | 0.29 | 1 |  | R.KLLEGEECR.L |
|  | 1671 | **577.2816** | **1152.5487** | **1152.5485** | **0.18** | **0** | **34** | **0.0061** | **1** |  | **K.EYQELMNVK.L** |
|  | 1719 | **583.2961** | **1164.5777** | **1164.5775** | **0.21** | **0** | **50** | **0.00016** | **1** |  | **K.YEELQVTAGR.H** |
|  | 1741 | **585.2788** | **1168.5431** | **1168.5434** | **-0.28** | **0** | **(30)** | **0.012** | **1** |  | **K.EYQELMNVK.L + Oxidation (M)** |
|  | 1756 | 586.8228 | 1171.6311 | 1171.6309 | 0.13 | 1 | 28 | 0.025 | 1 |  | R.RQLDSIVGER.G |
|  | 1901 | 602.3210 | 1202.6275 | 1202.6295 | -1.67 | 0 | (11) | 1.6 | 1 |  | K.WTLLQEQGTK.T |
|  | 1902 | 602.3215 | 1202.6284 | 1202.6295 | -0.95 | 0 | 51 | 0.00015 | 1 |  | K.WTLLQEQGTK.T |
|  | 1933 | **606.7818** | **1211.5490** | **1211.5458** | **2.65** | **0** | **25** | **0.028** | **1** |  | **R.AEAESWYQTK.Y** |
|  | 1956 | **611.8193** | **1221.6241** | **1221.6241** | **0.02** | **0** | **50** | **0.00016** | **1** |  | **R.TAAENEFVTLK.K** |
|  | 1957 | **611.8248** | **1221.6351** | **1221.6241** | **9.01** | **0** | **(2)** | **10** | **6** |  | **R.TAAENEFVTLK.K** |
|  | 2364 | 665.3669 | 1328.7193 | 1328.7187 | 0.46 | 0 | 23 | 0.087 | 1 |  | R.NLDLDSIIAEVK.A |
|  | 2468 | **453.2496** | **1356.7269** | **1356.7249** | **1.49** | **1** | **(36)** | **0.0042** | **1** |  | **K.NKLEGLEDALQK.A** |
|  | 2469 | **679.3712** | **1356.7279** | **1356.7249** | **2.24** | **1** | **70** | **1.7e-006** | **1** |  | **K.NKLEGLEDALQK.A** |
|  | 2656 | **704.3592** | **1406.7038** | **1406.7041** | **-0.23** | **0** | **43** | **0.00066** | **1** |  | **K.ADTLTDEINFLR.A** |
|  | 2712 | **712.8199** | **1423.6252** | **1423.6263** | **-0.75** | **0** | **56** | **1.5e-005** | **1** |  | **R.GSGGLGGACGGAGFGSR.S** |
|  | 2802 | **724.3918** | **1446.7690** | **1446.7678** | **0.81** | **0** | **67** | **2.4e-006** | **1** | **U** | **R.AIGGGLSSVGGGSSTIK.Y** |
|  | 2827 | **728.3465** | **1454.6784** | **1454.6790** | **-0.36** | **1** | **(25)** | **0.038** | **1** |  | **R.SRAEAESWYQTK.Y** |
|  | 2828 | **485.9006** | **1454.6800** | **1454.6790** | **0.69** | **1** | **33** | **0.0061** | **1** |  | **R.SRAEAESWYQTK.Y** |
|  | 3163 | **799.8834** | **1597.7523** | **1597.7519** | **0.27** | **0** | **120** | **1e-011** | **1** |  | **R.ISIGGGSCAISGGYGSR.A** |
|  | 3341 | **847.9249** | **1693.8353** | **1693.8345** | **0.48** | **1** | **25** | **0.043** | **1** |  | **K.DVDAAYMNKVELQAK.A** |

  


---

|  |  |
| --- | --- |
| **9.** | sp|P08779|K1C16\_HUMAN    **Mass:** 51578    **Score:** 605    **Matches:** 31(22)  **Sequences:** 23(18)  **emPAI:** 2.24 |
|  | Keratin, type I cytoskeletal 16 OS=Homo sapiens OX=9606 GN=KRT16 PE=1 SV=4 |

|  |  |
| --- | --- |
|  | Check to include this hit in error tolerant search or archive report |
|  |  |

|  |  |  |  |  |  |  |  |  |  |  |  |
| --- | --- | --- | --- | --- | --- | --- | --- | --- | --- | --- | --- |
|  | **Query** | **Observed** | **Mr(expt)** | **Mr(calc)** | **ppm** | **Miss** | **Score** | **Expect** | **Rank** | **Unique** | **Peptide** |
|  | 134 | 388.2085 | 774.4024 | 774.4058 | -4.30 | 0 | 19 | 0.44 | 2 |  | R.ILNEMR.D |
|  | 189 | 404.2032 | 806.3918 | 806.3923 | -0.61 | 0 | 43 | 0.0011 | 1 |  | R.LAADDFR.T |
|  | 190 | 404.2034 | 806.3923 | 806.3923 | 0.06 | 0 | (31) | 0.017 | 1 |  | R.LAADDFR.T |
|  | 192 | 405.2236 | 808.4326 | 808.4330 | -0.56 | 0 | (31) | 0.012 | 1 |  | R.LASYLDK.V |
|  | 193 | 405.2237 | 808.4329 | 808.4330 | -0.19 | 0 | 43 | 0.00066 | 1 |  | R.LASYLDK.V |
|  | 654 | 460.2129 | 918.4113 | 918.4123 | -1.13 | 0 | 8 | 1.2 | 1 |  | K.DYSPYFK.T |
|  | 1066 | 515.3005 | 1028.5865 | 1028.5866 | -0.06 | 0 | 43 | 0.00066 | 1 |  | R.VLDELTLAR.T |
|  | 1067 | 515.3011 | 1028.5876 | 1028.5866 | 1.01 | 0 | (23) | 0.063 | 1 |  | R.VLDELTLAR.T |
|  | 1092 | 518.7702 | 1035.5258 | 1035.5250 | 0.82 | 1 | 17 | 0.35 | 1 |  | K.IRDWYQR.Q |
|  | 1267 | 355.5415 | 1063.6028 | 1063.6026 | 0.18 | 1 | 49 | 9.1e-005 | 1 |  | R.LASYLDKVR.A |
|  | 1268 | 532.8089 | 1063.6032 | 1063.6026 | 0.64 | 1 | (45) | 0.00026 | 1 |  | R.LASYLDKVR.A |
|  | 1392 | 545.7685 | 1089.5224 | 1089.5237 | -1.15 | 0 | (51) | 0.00012 | 1 |  | K.VTMQNLNDR.L |
|  | 1393 | 545.7700 | 1089.5254 | 1089.5237 | 1.55 | 0 | 51 | 0.00012 | 1 |  | K.VTMQNLNDR.L |
|  | 1431 | **548.7690** | **1095.5234** | **1095.5237** | **-0.24** | **0** | **36** | **0.0035** | **1** | **U** | **R.DAETWFLSK.T** |
|  | 1467 | 553.7848 | 1105.5550 | 1105.5550 | 0.03 | 0 | 59 | 2.6e-005 | 1 |  | R.ISSVLAGGSCR.A |
|  | 1634 | **571.2447** | **1140.4748** | **1140.4757** | **-0.77** | **0** | **40** | **0.00038** | **1** | **U** | **R.DQYEQMAEK.N** |
|  | 1809 | 395.9031 | 1184.6873 | 1184.6877 | -0.32 | 1 | 2 | 6.4 | 4 |  | R.RVLDELTLAR.T |
|  | 1810 | 395.9031 | 1184.6873 | 1184.6877 | -0.32 | 1 | (1) | 7.2 | 3 |  | R.RVLDELTLAR.T |
|  | 1883 | 601.3116 | 1200.6087 | 1200.6099 | -0.93 | 0 | 8 | 2.1 | 3 | U | R.QTVEADVNGLR.R |
|  | 1952 | 610.8038 | 1219.5931 | 1219.5932 | -0.05 | 0 | 56 | 4.8e-005 | 1 |  | K.ASLENSLEETK.G |
|  | 2030 | **621.3003** | **1240.5861** | **1240.5870** | **-0.67** | **0** | **30** | **0.0091** | **1** | **U** | **K.NHEEEMLALR.G** |
|  | 2113 | **420.5626** | **1258.6659** | **1258.6669** | **-0.80** | **1** | **37** | **0.0031** | **1** | **U** | **R.TKYEHELALR.Q** |
|  | 2174 | 639.7949 | 1277.5753 | 1277.5783 | -2.34 | 0 | 32 | 0.0071 | 1 |  | K.GSCGIGGGIGGGSSR.I |
|  | 2264 | 651.3348 | 1300.6550 | 1300.6510 | 3.07 | 0 | 80 | 1.6e-007 | 1 |  | R.ALEEANADLEVK.I |
|  | 2387 | **669.8362** | **1337.6579** | **1337.6575** | **0.28** | **0** | **63** | **7.8e-006** | **1** | **U** | **R.APSTYGGGLSVSSR.F** |
|  | 2465 | 453.2445 | 1356.7118 | 1356.7110 | 0.61 | 1 | 46 | 0.00045 | 1 | U | R.QTVEADVNGLRR.V |
|  | 2466 | 679.3632 | 1356.7119 | 1356.7110 | 0.68 | 1 | (11) | 1.3 | 4 | U | R.QTVEADVNGLRR.V |
|  | 2467 | 453.2449 | 1356.7128 | 1356.7110 | 1.34 | 1 | (3) | 7.9 | 7 | U | R.QTVEADVNGLRR.V |
|  | 2539 | 460.5807 | 1378.7202 | 1378.7204 | -0.21 | 1 | 57 | 3.4e-005 | 1 |  | K.TRLEQEIATYR.R |
|  | 2651 | **703.3472** | **1404.6799** | **1404.6845** | **-3.24** | **0** | **87** | **2.7e-008** | **1** | **U** | **K.EVASNSELVQSSR.S** |
|  | 4306 | **784.0349** | **2349.0827** | **2349.0833** | **-0.23** | **0** | **66** | **1.8e-006** | **1** | **U** | **R.LLEGEDAHLSSQQASGQSYSSR.E** |

  


---

|  |  |
| --- | --- |
| **10.** | sp|P04259|K2C6B\_HUMAN    **Mass:** 60315    **Score:** 602    **Matches:** 33(25)  **Sequences:** 23(19)  **emPAI:** 2.77 |
|  | Keratin, type II cytoskeletal 6B OS=Homo sapiens OX=9606 GN=KRT6B PE=1 SV=5 |

|  |  |
| --- | --- |
|  | Check to include this hit in error tolerant search or archive report |
|  |  |

|  |  |  |  |  |  |  |  |  |  |  |  |
| --- | --- | --- | --- | --- | --- | --- | --- | --- | --- | --- | --- |
|  | **Query** | **Observed** | **Mr(expt)** | **Mr(calc)** | **ppm** | **Miss** | **Score** | **Expect** | **Rank** | **Unique** | **Peptide** |
|  | 248 | 414.2183 | 826.4220 | 826.4225 | -0.57 | 0 | (29) | 0.014 | 1 |  | K.FASFIDK.V |
|  | 249 | 414.2186 | 826.4226 | 826.4225 | 0.11 | 0 | 44 | 0.00045 | 1 |  | K.FASFIDK.V |
|  | 523 | 441.2439 | 880.4733 | 880.4654 | 8.95 | 0 | 35 | 0.0035 | 1 |  | R.SLYGLGGSK.R |
|  | 747 | 473.2593 | 944.5039 | 944.5039 | 0.03 | 1 | 34 | 0.0099 | 1 |  | R.GRLDSELR.N |
|  | 830 | 486.7591 | 971.5036 | 971.5036 | -0.00 | 0 | 54 | 5.7e-005 | 1 |  | K.QEIAEINR.M |
|  | 965 | 503.2367 | 1004.4589 | 1004.4597 | -0.78 | 0 | 22 | 0.08 | 1 |  | K.LLEGEECR.L |
|  | 1044 | 513.7310 | 1025.4475 | 1025.4488 | -1.26 | 0 | 31 | 0.0057 | 1 |  | K.DVDAAYMNK.V |
|  | 1284 | 533.7620 | 1065.5094 | 1065.5090 | 0.30 | 1 | 10 | 1.6 | 1 |  | K.YEDEINKR.T |
|  | 1358 | 361.5378 | 1081.5917 | 1081.5920 | -0.31 | 1 | (31) | 0.0083 | 1 |  | K.FASFIDKVR.F |
|  | 1359 | 541.8038 | 1081.5931 | 1081.5920 | 1.00 | 1 | 50 | 0.00011 | 1 |  | K.FASFIDKVR.F |
|  | 1473 | 554.2658 | 1106.5171 | 1106.5356 | -16.75 | 0 | (1) | 11 | 2 |  | K.AQYEEIAQR.S |
|  | 1475 | 554.2739 | 1106.5333 | 1106.5356 | -2.07 | 0 | 42 | 0.00091 | 1 |  | K.AQYEEIAQR.S |
|  | 1604 | 567.2844 | 1132.5542 | 1132.5546 | -0.40 | 1 | 20 | 0.17 | 1 |  | R.KLLEGEECR.L |
|  | 1605 | 378.5254 | 1132.5543 | 1132.5546 | -0.33 | 1 | (18) | 0.29 | 1 |  | R.KLLEGEECR.L |
|  | 1671 | 577.2816 | 1152.5487 | 1152.5485 | 0.18 | 0 | 34 | 0.0061 | 1 |  | K.EYQELMNVK.L |
|  | 1741 | 585.2788 | 1168.5431 | 1168.5434 | -0.28 | 0 | (30) | 0.012 | 1 |  | K.EYQELMNVK.L + Oxidation (M) |
|  | 1780 | 590.3036 | 1178.5927 | 1178.5931 | -0.32 | 0 | (70) | 1.7e-006 | 1 |  | K.YEELQITAGR.H |
|  | 1781 | 590.3037 | 1178.5929 | 1178.5931 | -0.22 | 0 | 71 | 1.2e-006 | 1 |  | K.YEELQITAGR.H |
|  | 1901 | 602.3210 | 1202.6275 | 1202.6295 | -1.67 | 0 | (11) | 1.6 | 1 |  | K.WTLLQEQGTK.T |
|  | 1902 | 602.3215 | 1202.6284 | 1202.6295 | -0.95 | 0 | 51 | 0.00015 | 1 |  | K.WTLLQEQGTK.T |
|  | 1933 | 606.7818 | 1211.5490 | 1211.5458 | 2.65 | 0 | 25 | 0.028 | 1 |  | R.AEAESWYQTK.Y |
|  | 1956 | 611.8193 | 1221.6241 | 1221.6241 | 0.02 | 0 | 50 | 0.00016 | 1 |  | R.TAAENEFVTLK.K |
|  | 1957 | 611.8248 | 1221.6351 | 1221.6241 | 9.01 | 0 | (2) | 10 | 6 |  | R.TAAENEFVTLK.K |
|  | 2364 | 665.3669 | 1328.7193 | 1328.7187 | 0.46 | 0 | 23 | 0.087 | 1 |  | R.NLDLDSIIAEVK.A |
|  | 2468 | 453.2496 | 1356.7269 | 1356.7249 | 1.49 | 1 | (36) | 0.0042 | 1 |  | K.NKLEGLEDALQK.A |
|  | 2469 | 679.3712 | 1356.7279 | 1356.7249 | 2.24 | 1 | 70 | 1.7e-006 | 1 |  | K.NKLEGLEDALQK.A |
|  | 2656 | 704.3592 | 1406.7038 | 1406.7041 | -0.23 | 0 | 43 | 0.00066 | 1 |  | K.ADTLTDEINFLR.A |
|  | 2712 | 712.8199 | 1423.6252 | 1423.6263 | -0.75 | 0 | 56 | 1.5e-005 | 1 |  | R.GSGGLGGACGGAGFGSR.S |
|  | 2763 | **718.3730** | **1434.7314** | **1434.7315** | **-0.02** | **0** | **37** | **0.0028** | **1** | **U** | **R.ATGGGLSSVGGGSSTIK.Y** |
|  | 2827 | 728.3465 | 1454.6784 | 1454.6790 | -0.36 | 1 | (25) | 0.038 | 1 |  | R.SRAEAESWYQTK.Y |
|  | 2828 | 485.9006 | 1454.6800 | 1454.6790 | 0.69 | 1 | 33 | 0.0061 | 1 |  | R.SRAEAESWYQTK.Y |
|  | 3163 | 799.8834 | 1597.7523 | 1597.7519 | 0.27 | 0 | 120 | 1e-011 | 1 |  | R.ISIGGGSCAISGGYGSR.A |
|  | 3341 | 847.9249 | 1693.8353 | 1693.8345 | 0.48 | 1 | 25 | 0.043 | 1 |  | K.DVDAAYMNKVELQAK.A |

  


---

|  |  |
| --- | --- |
| **11.** | sp|P05787|K2C8\_HUMAN    **Mass:** 53671    **Score:** 496    **Matches:** 29(19)  **Sequences:** 17(14)  **emPAI:** 1.75 |
|  | Keratin, type II cytoskeletal 8 OS=Homo sapiens OX=9606 GN=KRT8 PE=1 SV=7 |

|  |  |
| --- | --- |
|  | Check to include this hit in error tolerant search or archive report |
|  |  |

|  |  |  |  |  |  |  |  |  |  |  |  |
| --- | --- | --- | --- | --- | --- | --- | --- | --- | --- | --- | --- |
|  | **Query** | **Observed** | **Mr(expt)** | **Mr(calc)** | **ppm** | **Miss** | **Score** | **Expect** | **Rank** | **Unique** | **Peptide** |
|  | 248 | 414.2183 | 826.4220 | 826.4225 | -0.57 | 0 | (29) | 0.014 | 1 |  | K.FASFIDK.V |
|  | 249 | 414.2186 | 826.4226 | 826.4225 | 0.11 | 0 | 44 | 0.00045 | 1 |  | K.FASFIDK.V |
|  | 948 | **500.7871** | **999.5595** | **999.5600** | **-0.48** | **0** | **53** | **7.8e-005** | **1** | **U** | **R.LQAEIEGLK.G** |
|  | 1284 | 533.7620 | 1065.5094 | 1065.5090 | 0.30 | 1 | 10 | 1.6 | 1 |  | K.YEDEINKR.T |
|  | 1358 | 361.5378 | 1081.5917 | 1081.5920 | -0.31 | 1 | (31) | 0.0083 | 1 |  | K.FASFIDKVR.F |
|  | 1359 | 541.8038 | 1081.5931 | 1081.5920 | 1.00 | 1 | 50 | 0.00011 | 1 |  | K.FASFIDKVR.F |
|  | 1583 | **565.3142** | **1128.6139** | **1128.6138** | **0.02** | **0** | **46** | **0.00047** | **1** | **U** | **K.LSELEAALQR.A** |
|  | 1671 | 577.2816 | 1152.5487 | 1152.5485 | 0.18 | 0 | 34 | 0.0061 | 1 |  | R.EYQELMNVK.L |
|  | 1741 | 585.2788 | 1168.5431 | 1168.5434 | -0.28 | 0 | (30) | 0.012 | 1 |  | R.EYQELMNVK.L + Oxidation (M) |
|  | 1758 | **587.3215** | **1172.6284** | **1172.6289** | **-0.40** | **0** | **40** | **0.0018** | **1** | **U** | **K.LVSESSDVLPK.-** |
|  | 1759 | **587.3225** | **1172.6303** | **1172.6289** | **1.27** | **0** | **(11)** | **1.5** | **1** | **U** | **K.LVSESSDVLPK.-** |
|  | 2342 | **660.8394** | **1319.6642** | **1319.6642** | **-0.06** | **0** | **(12)** | **1.2** | **1** | **U** | **R.SLDMDSIIAEVK.A** |
|  | 2382 | **668.8409** | **1335.6673** | **1335.6592** | **6.11** | **0** | **50** | **0.00016** | **1** | **U** | **R.SLDMDSIIAEVK.A + Oxidation (M)** |
|  | 2408 | **671.3773** | **1340.7400** | **1340.7412** | **-0.90** | **1** | **7** | **2** | **2** | **U** | **R.LQAEIEGLKGQR.A** |
|  | 2421 | **672.8403** | **1343.6660** | **1343.6681** | **-1.54** | **0** | **92** | **8.2e-009** | **1** | **U** | **R.ASLEAAIADAEQR.G** |
|  | 2440 | **676.8444** | **1351.6743** | **1351.6693** | **3.67** | **0** | **34** | **0.0072** | **1** | **U** | **R.TEMENEFVLIK.K** |
|  | 2669 | **706.8453** | **1411.6761** | **1411.6765** | **-0.27** | **1** | **43** | **0.00069** | **1** | **U** | **R.SRAEAESMYQIK.Y** |
|  | 2670 | **471.5662** | **1411.6767** | **1411.6765** | **0.16** | **1** | **(32)** | **0.0078** | **1** | **U** | **R.SRAEAESMYQIK.Y** |
|  | 2690 | **710.3771** | **1418.7397** | **1418.7405** | **-0.56** | **0** | **49** | **0.00021** | **1** | **U** | **R.LEGLTDEINFLR.Q** |
|  | 2691 | **710.3778** | **1418.7411** | **1418.7405** | **0.38** | **0** | **(26)** | **0.037** | **1** | **U** | **R.LEGLTDEINFLR.Q** |
|  | 2735 | **476.8978** | **1427.6717** | **1427.6714** | **0.16** | **1** | **(3)** | **5.2** | **5** | **U** | **R.SRAEAESMYQIK.Y + Oxidation (M)** |
|  | 2869 | **738.3518** | **1474.6891** | **1474.6908** | **-1.18** | **0** | **68** | **1.7e-006** | **1** | **U** | **R.LESGMQNMSIHTK.T** |
|  | 2885 | **740.8893** | **1479.7640** | **1479.7643** | **-0.19** | **1** | **70** | **1.3e-006** | **1** | **U** | **R.TEMENEFVLIKK.D** |
|  | 2902 | **746.3497** | **1490.6848** | **1490.6857** | **-0.63** | **0** | **(4)** | **3.2** | **2** | **U** | **R.LESGMQNMSIHTK.T + Oxidation (M)** |
|  | 2903 | **497.9025** | **1490.6857** | **1490.6857** | **-0.04** | **0** | **(2)** | **5.1** | **5** | **U** | **R.LESGMQNMSIHTK.T + Oxidation (M)** |
|  | 2904 | **746.3503** | **1490.6860** | **1490.6857** | **0.19** | **0** | **(12)** | **0.63** | **1** | **U** | **R.LESGMQNMSIHTK.T + Oxidation (M)** |
|  | 2928 | **748.8876** | **1495.7607** | **1495.7592** | **1.01** | **1** | **(16)** | **0.29** | **1** | **U** | **R.TEMENEFVLIKK.D + Oxidation (M)** |
|  | 3603 | **899.4197** | **1796.8249** | **1796.8250** | **-0.06** | **1** | **79** | **1.2e-007** | **1** | **U** | **K.DVDEAYMNKVELESR.L** |
|  | 3777 | **470.7095** | **1878.8090** | **1878.7876** | **11.4** | **0** | **6** | **1.2** | **1** | **U** | **R.SNMDNMFESYINNLR.R + 2 Oxidation (M)** |

  


---

|  |  |
| --- | --- |
| **12.** | sp|P08670|VIME\_HUMAN    **Mass:** 53676    **Score:** 471    **Matches:** 17(14)  **Sequences:** 16(14)  **emPAI:** 1.30 |
|  | Vimentin OS=Homo sapiens OX=9606 GN=VIM PE=1 SV=4 |

|  |  |
| --- | --- |
|  | Check to include this hit in error tolerant search or archive report |
|  |  |

|  |  |  |  |  |  |  |  |  |  |  |  |
| --- | --- | --- | --- | --- | --- | --- | --- | --- | --- | --- | --- |
|  | **Query** | **Observed** | **Mr(expt)** | **Mr(calc)** | **ppm** | **Miss** | **Score** | **Expect** | **Rank** | **Unique** | **Peptide** |
|  | 1325 | **538.7557** | **1075.4968** | **1075.4968** | **0.03** | **0** | **34** | **0.0037** | **1** | **U** | **R.DNLAEDIMR.L** |
|  | 1412 | **547.2694** | **1092.5243** | **1092.5200** | **3.95** | **0** | **54** | **5.9e-005** | **1** | **U** | **K.FADLSEAANR.N** |
|  | 1518 | **558.2894** | **1114.5643** | **1114.5618** | **2.22** | **0** | **43** | **0.00071** | **1** |  | **K.VELQELNDR.F** |
|  | 1743 | **585.3608** | **1168.7071** | **1168.7067** | **0.38** | **0** | **33** | **0.0019** | **1** | **U** | **K.ILLAELEQLK.G** |
|  | 2076 | **627.7869** | **1253.5592** | **1253.5598** | **-0.46** | **0** | **71** | **6.6e-007** | **1** | **U** | **R.LGDLYEEEMR.E** |
|  | 2293 | **655.3064** | **1308.5982** | **1308.5986** | **-0.26** | **0** | **59** | **1.1e-005** | **1** |  | **K.NLQEAEEWYK.S** |
|  | 2300 | **656.3341** | **1310.6535** | **1310.6540** | **-0.35** | **0** | **60** | **1.4e-005** | **1** |  | **K.MALDIEIATYR.K + Oxidation (M)** |
|  | 2348 | **662.3122** | **1322.6098** | **1322.6102** | **-0.30** | **0** | **60** | **1.1e-005** | **1** | **U** | **R.EEAENTLQSFR.Q** |
|  | 2741 | **714.8619** | **1427.7093** | **1427.7045** | **3.39** | **0** | **58** | **2.2e-005** | **1** | **U** | **R.SLYASSPGGVYATR.S** |
|  | 2901 | **745.8796** | **1489.7447** | **1489.7446** | **0.06** | **0** | **79** | **1.9e-007** | **1** | **U** | **R.QVQSLTCEVDALK.G** |
|  | 2927 | **499.2675** | **1494.7806** | **1494.7790** | **1.07** | **0** | **18** | **0.23** | **1** | **U** | **R.TYSLGSALRPSTSR.S** |
|  | 3029 | **513.9761** | **1538.9066** | **1538.9032** | **2.24** | **1** | **37** | **0.00061** | **1** | **U** | **K.ILLAELEQLKGQGK.S** |
|  | 3147 | **529.9376** | **1586.7909** | **1586.7900** | **0.55** | **1** | **(22)** | **0.1** | **1** |  | **R.TNEKVELQELNDR.F** |
|  | 3148 | **794.4036** | **1586.7926** | **1586.7900** | **1.63** | **1** | **77** | **3.4e-007** | **1** |  | **R.TNEKVELQELNDR.F** |
|  | 3298 | **556.9529** | **1667.8368** | **1667.8366** | **0.11** | **0** | **12** | **0.74** | **1** | **U** | **R.ETNLDSLPLVDTHSK.R** |
|  | 3434 | **578.9433** | **1733.8081** | **1733.8076** | **0.27** | **1** | **44** | **0.0004** | **1** | **U** | **R.LQDEIQNMKEEMAR.H** |
|  | 3683 | **918.9012** | **1835.7878** | **1835.7922** | **-2.40** | **0** | **38** | **0.00085** | **1** | **U** | **R.DGQVINETSQHHDDLE.-** |

  


---

|  |  |
| --- | --- |
| **13.** | sp|P05783|K1C18\_HUMAN    **Mass:** 48029    **Score:** 450    **Matches:** 23(18)  **Sequences:** 17(15)  **emPAI:** 1.71 |
|  | Keratin, type I cytoskeletal 18 OS=Homo sapiens OX=9606 GN=KRT18 PE=1 SV=2 |

|  |  |
| --- | --- |
|  | Check to include this hit in error tolerant search or archive report |
|  |  |

|  |  |  |  |  |  |  |  |  |  |  |  |
| --- | --- | --- | --- | --- | --- | --- | --- | --- | --- | --- | --- |
|  | **Query** | **Observed** | **Mr(expt)** | **Mr(calc)** | **ppm** | **Miss** | **Score** | **Expect** | **Rank** | **Unique** | **Peptide** |
|  | 18 | **359.6990** | **717.3835** | **717.3843** | **-1.11** | **0** | **18** | **0.38** | **1** | **U** | **K.IMADIR.A** |
|  | 96 | **379.7237** | **757.4328** | **757.4334** | **-0.73** | **0** | **32** | **0.02** | **1** | **U** | **K.IIEDLR.A** |
|  | 189 | 404.2032 | 806.3918 | 806.3923 | -0.61 | 0 | 43 | 0.0011 | 1 |  | R.LAADDFR.V |
|  | 190 | 404.2034 | 806.3923 | 806.3923 | 0.06 | 0 | (31) | 0.017 | 1 |  | R.LAADDFR.V |
|  | 815 | **483.2376** | **964.4607** | **964.4614** | **-0.74** | **0** | **29** | **0.017** | **1** | **U** | **R.AQYDELAR.K** |
|  | 851 | **488.2282** | **974.4417** | **974.4458** | **-4.11** | **0** | **26** | **0.026** | **1** | **U** | **R.STFSTNYR.S** |
|  | 872 | **491.7248** | **981.4351** | **981.4345** | **0.64** | **0** | **23** | **0.032** | **1** | **U** | **R.DWSHYFK.I** |
|  | 1102 | **521.3063** | **1040.5981** | **1040.5978** | **0.29** | **0** | **50** | **7.2e-005** | **1** |  | **R.IVLQIDNAR.L** |
|  | 1272 | **533.2825** | **1064.5505** | **1064.5502** | **0.30** | **0** | **45** | **0.00054** | **1** | **U** | **K.LEAEIATYR.R** |
|  | 1763 | **587.8247** | **1173.6349** | **1173.6353** | **-0.41** | **1** | **25** | **0.052** | **1** | **U** | **R.KVIDDTNITR.L** |
|  | 1764 | **587.8317** | **1173.6488** | **1173.6353** | **11.5** | **1** | **(20)** | **0.13** | **1** | **U** | **R.KVIDDTNITR.L** |
|  | 2024 | **620.3238** | **1238.6330** | **1238.6329** | **0.12** | **1** | **38** | **0.0021** | **1** | **U** | **R.VKYETELAMR.Q** |
|  | 2135 | **634.3229** | **1266.6313** | **1266.6317** | **-0.26** | **0** | **95** | **4.1e-009** | **1** | **U** | **R.QSVENDIHGLR.K** |
|  | 2227 | **646.8640** | **1291.7135** | **1291.7136** | **-0.07** | **1** | **49** | **0.00013** | **1** | **U** | **K.VKLEAEIATYR.R** |
|  | 2228 | **646.8641** | **1291.7136** | **1291.7136** | **0.02** | **1** | **(37)** | **0.0019** | **1** | **U** | **K.VKLEAEIATYR.R** |
|  | 2327 | **660.3377** | **1318.6607** | **1318.6629** | **-1.67** | **0** | **(62)** | **1.2e-005** | **1** | **U** | **R.AQIFANTVDNAR.I** |
|  | 2328 | **660.3390** | **1318.6634** | **1318.6629** | **0.36** | **0** | **87** | **3.3e-008** | **1** | **U** | **R.AQIFANTVDNAR.I** |
|  | 2611 | **698.3697** | **1394.7248** | **1394.7266** | **-1.28** | **1** | **26** | **0.03** | **1** | **U** | **R.QSVENDIHGLRK.V** |
|  | 2612 | **465.9157** | **1394.7252** | **1394.7266** | **-1.00** | **1** | **(21)** | **0.11** | **1** | **U** | **R.QSVENDIHGLRK.V** |
|  | 2613 | **465.9161** | **1394.7265** | **1394.7266** | **-0.08** | **1** | **(1)** | **8.5** | **2** | **U** | **R.QSVENDIHGLRK.V** |
|  | 2692 | **710.3783** | **1418.7420** | **1418.7405** | **1.10** | **0** | **61** | **1.4e-005** | **1** | **U** | **R.QAQEYEALLNIK.V** |
|  | 2981 | **761.8741** | **1521.7337** | **1521.7345** | **-0.47** | **0** | **67** | **2.2e-006** | **1** | **U** | **R.TVQSLEIDLDSMR.N + Oxidation (M)** |
|  | 4211 | **1131.0553** | **2260.0960** | **2260.0940** | **0.90** | **0** | **17** | **0.19** | **1** | **U** | **R.GGMGSGGLATGIAGGLAGMGGIQNEK.E** |

  


---

|  |  |
| --- | --- |
| **14.** | sp|P07437|TBB5\_HUMAN    **Mass:** 50095    **Score:** 427    **Matches:** 17(12)  **Sequences:** 13(9)  **emPAI:** 1.15 |
|  | Tubulin beta chain OS=Homo sapiens OX=9606 GN=TUBB PE=1 SV=2 |

|  |  |
| --- | --- |
|  | Check to include this hit in error tolerant search or archive report |
|  |  |

|  |  |  |  |  |  |  |  |  |  |  |  |
| --- | --- | --- | --- | --- | --- | --- | --- | --- | --- | --- | --- |
|  | **Query** | **Observed** | **Mr(expt)** | **Mr(calc)** | **ppm** | **Miss** | **Score** | **Expect** | **Rank** | **Unique** | **Peptide** |
|  | 1061 | **514.7636** | **1027.5127** | **1027.5121** | **0.58** | **0** | **40** | **0.0013** | **1** |  | **K.TAVCDIPPR.G** |
|  | 1098 | **520.3007** | **1038.5868** | **1038.5862** | **0.54** | **0** | **18** | **0.13** | **1** |  | **R.YLTVAAVFR.G** |
|  | 1586 | **565.8026** | **1129.5907** | **1129.5880** | **2.36** | **0** | **47** | **0.00043** | **1** |  | **R.FPGQLNADLR.K** |
|  | 1587 | **565.8045** | **1129.5944** | **1129.5880** | **5.71** | **0** | **(16)** | **0.57** | **1** |  | **R.FPGQLNADLR.K** |
|  | 1646 | 572.3210 | 1142.6275 | 1142.6270 | 0.44 | 0 | 47 | 0.00034 | 1 |  | K.LAVNMVPFPR.L |
|  | 1983 | **615.3021** | **1228.5896** | **1228.5910** | **-1.19** | **0** | **73** | **5.6e-007** | **1** |  | **R.ISEQFTAMFR.R** |
|  | 2263 | **651.3232** | **1300.6319** | **1300.6299** | **1.55** | **0** | **46** | **0.00032** | **1** | **U** | **R.ISVYYNEATGGK.Y** |
|  | 2329 | **660.3538** | **1318.6931** | **1318.6955** | **-1.84** | **0** | **(52)** | **0.00011** | **1** |  | **R.IMNTFSVVPSPK.V** |
|  | 2330 | **660.3572** | **1318.6998** | **1318.6955** | **3.26** | **0** | **78** | **2.8e-007** | **1** |  | **R.IMNTFSVVPSPK.V** |
|  | 2798 | **723.8500** | **1445.6854** | **1445.6820** | **2.34** | **0** | **47** | **0.00028** | **1** |  | **K.EVDEQMLNVQNK.N** |
|  | 3194 | **808.4233** | **1614.8321** | **1614.8287** | **2.12** | **0** | **57** | **2.8e-005** | **1** | **U** | **R.AILVDLEPGTMDSVR.S** |
|  | 3225 | **816.4196** | **1630.8246** | **1630.8236** | **0.58** | **0** | **(48)** | **0.00023** | **1** | **U** | **R.AILVDLEPGTMDSVR.S + Oxidation (M)** |
|  | 3281 | **830.4515** | **1658.8885** | **1658.8879** | **0.36** | **0** | **11** | **0.89** | **1** | **U** | **R.ALTVPELTQQVFDAK.N** |
|  | 3348 | **848.9194** | **1695.8243** | **1695.8257** | **-0.79** | **0** | **5** | **5** | **1** |  | **K.NSSYFVEWIPNNVK.T** |
|  | 3655 | **608.3128** | **1821.9166** | **1821.9156** | **0.57** | **0** | **(69)** | **1.7e-006** | **1** | **U** | **R.EIVHIQAGQCGNQIGAK.F** |
|  | 3656 | **911.9659** | **1821.9173** | **1821.9156** | **0.97** | **0** | **80** | **1.3e-007** | **1** | **U** | **R.EIVHIQAGQCGNQIGAK.F** |
|  | 4310 | **786.0702** | **2355.1889** | **2355.1583** | **13.0** | **1** | **7** | **2.1** | **1** |  | **R.LHFFMPGFAPLTSRGSQQYR.A + Oxidation (M)** |

  


---

|  |  |
| --- | --- |
| **15.** | sp|Q04695|K1C17\_HUMAN    **Mass:** 48361    **Score:** 420    **Matches:** 29(17)  **Sequences:** 19(11)  **emPAI:** 1.36 |
|  | Keratin, type I cytoskeletal 17 OS=Homo sapiens OX=9606 GN=KRT17 PE=1 SV=2 |

|  |  |
| --- | --- |
|  | Check to include this hit in error tolerant search or archive report |
|  |  |

|  |  |  |  |  |  |  |  |  |  |  |  |
| --- | --- | --- | --- | --- | --- | --- | --- | --- | --- | --- | --- |
|  | **Query** | **Observed** | **Mr(expt)** | **Mr(calc)** | **ppm** | **Miss** | **Score** | **Expect** | **Rank** | **Unique** | **Peptide** |
|  | 1 | 350.7339 | 699.4532 | 699.4531 | 0.15 | 0 | 40 | 0.00068 | 1 |  | K.ILLDVK.T |
|  | 134 | 388.2085 | 774.4024 | 774.4058 | -4.30 | 0 | 19 | 0.44 | 2 |  | R.ILNEMR.D |
|  | 189 | 404.2032 | 806.3918 | 806.3923 | -0.61 | 0 | 43 | 0.0011 | 1 |  | R.LAADDFR.T |
|  | 190 | 404.2034 | 806.3923 | 806.3923 | 0.06 | 0 | (31) | 0.017 | 1 |  | R.LAADDFR.T |
|  | 192 | 405.2236 | 808.4326 | 808.4330 | -0.56 | 0 | (31) | 0.012 | 1 |  | R.LASYLDK.V |
|  | 193 | 405.2237 | 808.4329 | 808.4330 | -0.19 | 0 | 43 | 0.00066 | 1 |  | R.LASYLDK.V |
|  | 919 | 497.2536 | 992.4927 | 992.4927 | 0.02 | 0 | 10 | 2 | 4 |  | K.FETEQALR.L |
|  | 1066 | 515.3005 | 1028.5865 | 1028.5866 | -0.06 | 0 | 43 | 0.00066 | 1 |  | R.VLDELTLAR.A |
|  | 1067 | 515.3011 | 1028.5876 | 1028.5866 | 1.01 | 0 | (23) | 0.063 | 1 |  | R.VLDELTLAR.A |
|  | 1092 | 518.7702 | 1035.5258 | 1035.5250 | 0.82 | 1 | 17 | 0.35 | 1 |  | K.IRDWYQR.Q |
|  | 1267 | 355.5415 | 1063.6028 | 1063.6026 | 0.18 | 1 | 49 | 9.1e-005 | 1 |  | R.LASYLDKVR.A |
|  | 1268 | 532.8089 | 1063.6032 | 1063.6026 | 0.64 | 1 | (45) | 0.00026 | 1 |  | R.LASYLDKVR.A |
|  | 1522 | **559.2910** | **1116.5675** | **1116.5663** | **1.09** | **0** | **40** | **0.0017** | **1** | **U** | **R.TIVEEVQDGK.V** |
|  | 1647 | **572.7507** | **1143.4869** | **1143.4873** | **-0.33** | **0** | **52** | **3.5e-005** | **1** | **U** | **K.DAEDWFFSK.T** |
|  | 1809 | 395.9031 | 1184.6873 | 1184.6877 | -0.32 | 1 | 2 | 6.4 | 4 |  | R.RVLDELTLAR.A |
|  | 1810 | 395.9031 | 1184.6873 | 1184.6877 | -0.32 | 1 | (1) | 7.2 | 3 |  | R.RVLDELTLAR.A |
|  | 1956 | 611.8193 | 1221.6241 | 1221.6353 | -9.17 | 1 | (5) | 5.6 | 4 |  | R.TKFETEQALR.L |
|  | 1957 | 611.8248 | 1221.6351 | 1221.6353 | -0.18 | 1 | 25 | 0.05 | 1 |  | R.TKFETEQALR.L |
|  | 2038 | 621.7800 | 1241.5454 | 1241.5458 | -0.36 | 0 | (25) | 0.018 | 1 |  | K.NHEEEMNALR.G |
|  | 2039 | 621.7800 | 1241.5455 | 1241.5458 | -0.26 | 0 | 47 | 0.00011 | 1 |  | K.NHEEEMNALR.G |
|  | 2040 | 414.8558 | 1241.5457 | 1241.5458 | -0.14 | 0 | (41) | 0.00046 | 1 |  | K.NHEEEMNALR.G |
|  | 2411 | **671.8755** | **1341.7364** | **1341.7364** | **-0.01** | **1** | **14** | **0.43** | **1** | **U** | **R.LSVEADINGLRR.V** |
|  | 2489 | 681.3486 | 1360.6827 | 1360.6834 | -0.52 | 0 | (77) | 3.8e-007 | 1 |  | R.EVATNSELVQSGK.S |
|  | 2490 | 681.3488 | 1360.6829 | 1360.6834 | -0.34 | 0 | 84 | 7.8e-008 | 1 |  | R.EVATNSELVQSGK.S |
|  | 2518 | 457.5541 | 1369.6406 | 1369.6408 | -0.15 | 1 | 8 | 1.8 | 1 |  | K.KNHEEEMNALR.G |
|  | 2539 | 460.5807 | 1378.7202 | 1378.7204 | -0.21 | 1 | 57 | 3.4e-005 | 1 |  | K.TRLEQEIATYR.R |
|  | 2781 | 480.2378 | 1437.6917 | 1437.6922 | -0.31 | 1 | (11) | 1.1 | 1 |  | R.ILNEMRDQYEK.M |
|  | 2824 | 727.8499 | 1453.6853 | 1453.6871 | -1.25 | 1 | 14 | 0.48 | 1 |  | R.ILNEMRDQYEK.M + Oxidation (M) |
|  | 4078 | **1065.5187** | **2129.0228** | **2129.0059** | **7.94** | **0** | **0** | **10** | **7** | **U** | **R.GQVGGEINVEMDAAPGVDLSR.I + Oxidation (M)** |

  


---

|  |  |
| --- | --- |
| **16.** | sp|P60709|ACTB\_HUMAN    **Mass:** 42052    **Score:** 376    **Matches:** 26(18)  **Sequences:** 12(10)  **emPAI:** 2.11 |
|  | Actin, cytoplasmic 1 OS=Homo sapiens OX=9606 GN=ACTB PE=1 SV=1 |

|  |  |
| --- | --- |
|  | Check to include this hit in error tolerant search or archive report |
|  |  |

|  |  |  |  |  |  |  |  |  |  |  |  |
| --- | --- | --- | --- | --- | --- | --- | --- | --- | --- | --- | --- |
|  | **Query** | **Observed** | **Mr(expt)** | **Mr(calc)** | **ppm** | **Miss** | **Score** | **Expect** | **Rank** | **Unique** | **Peptide** |
|  | 162 | **398.2395** | **794.4644** | **794.4650** | **-0.71** | **0** | **28** | **0.013** | **1** |  | **K.IIAPPER.K** |
|  | 854 | **488.7276** | **975.4406** | **975.4410** | **-0.37** | **0** | **41** | **0.00083** | **1** |  | **K.AGFAGDDAPR.A** |
|  | 939 | **499.7468** | **997.4790** | **997.4790** | **0.03** | **0** | **(18)** | **0.18** | **1** |  | **R.DLTDYLMK.I** |
|  | 940 | **499.7468** | **997.4790** | **997.4790** | **0.03** | **0** | **(28)** | **0.02** | **1** |  | **R.DLTDYLMK.I** |
|  | 995 | **507.7447** | **1013.4748** | **1013.4739** | **0.88** | **0** | **30** | **0.0079** | **1** |  | **R.DLTDYLMK.I + Oxidation (M)** |
|  | 1597 | **566.7665** | **1131.5184** | **1131.5197** | **-1.10** | **0** | **26** | **0.025** | **1** | **U** | **R.GYSFTTTAER.E** |
|  | 1703 | **581.3121** | **1160.6097** | **1160.6111** | **-1.19** | **0** | **(20)** | **0.2** | **1** |  | **K.EITALAPSTMK.I** |
|  | 1704 | **581.3134** | **1160.6123** | **1160.6111** | **1.04** | **0** | **(30)** | **0.018** | **1** |  | **K.EITALAPSTMK.I** |
|  | 1751 | **586.2892** | **1170.5638** | **1170.5638** | **0.01** | **0** | **(10)** | **1.2** | **1** |  | **R.HQGVMVGMGQK.D** |
|  | 1752 | **586.2892** | **1170.5638** | **1170.5638** | **0.01** | **0** | **66** | **3e-006** | **1** |  | **R.HQGVMVGMGQK.D** |
|  | 1772 | **589.3107** | **1176.6068** | **1176.6060** | **0.67** | **0** | **36** | **0.0046** | **1** |  | **K.EITALAPSTMK.I + Oxidation (M)** |
|  | 1870 | **599.7648** | **1197.5150** | **1197.5150** | **0.03** | **0** | **2** | **3.4** | **1** |  | **K.DSYVGDEAQSK.R** |
|  | 1874 | **400.2401** | **1197.6986** | **1197.6982** | **0.30** | **0** | **28** | **0.011** | **1** |  | **R.AVFPSIVGRPR.H** |
|  | 2959 | **379.6922** | **1514.7397** | **1514.7419** | **-1.43** | **0** | **(15)** | **0.41** | **1** |  | **K.IWHHTFYNELR.V** |
|  | 2960 | **758.3784** | **1514.7423** | **1514.7419** | **0.28** | **0** | **(35)** | **0.0039** | **1** |  | **K.IWHHTFYNELR.V** |
|  | 2963 | **505.9216** | **1514.7430** | **1514.7419** | **0.73** | **0** | **(2)** | **8.8** | **2** |  | **K.IWHHTFYNELR.V** |
|  | 2965 | **505.9216** | **1514.7431** | **1514.7419** | **0.79** | **0** | **36** | **0.0029** | **1** |  | **K.IWHHTFYNELR.V** |
|  | 2966 | **505.9217** | **1514.7433** | **1514.7419** | **0.97** | **0** | **(9)** | **1.6** | **2** |  | **K.IWHHTFYNELR.V** |
|  | 2970 | **506.2375** | **1515.6906** | **1515.6954** | **-3.12** | **0** | **(55)** | **2.6e-005** | **1** |  | **K.QEYDESGPSIVHR.K** |
|  | 2971 | **758.8532** | **1515.6919** | **1515.6954** | **-2.31** | **0** | **72** | **5.6e-007** | **1** |  | **K.QEYDESGPSIVHR.K** |
|  | 2972 | **506.2394** | **1515.6963** | **1515.6954** | **0.62** | **0** | **(37)** | **0.0017** | **1** |  | **K.QEYDESGPSIVHR.K** |
|  | 3901 | **652.0258** | **1953.0555** | **1953.0571** | **-0.84** | **0** | **(33)** | **0.0034** | **1** | **U** | **R.VAPEEHPVLLTEAPLNPK.A** |
|  | 3902 | **977.5354** | **1953.0562** | **1953.0571** | **-0.44** | **0** | **(50)** | **7e-005** | **1** | **U** | **R.VAPEEHPVLLTEAPLNPK.A** |
|  | 3903 | **652.0264** | **1953.0573** | **1953.0571** | **0.09** | **0** | **(34)** | **0.0028** | **1** | **U** | **R.VAPEEHPVLLTEAPLNPK.A** |
|  | 3904 | **977.5361** | **1953.0576** | **1953.0571** | **0.25** | **0** | **59** | **8.4e-006** | **1** | **U** | **R.VAPEEHPVLLTEAPLNPK.A** |
|  | 4452 | **562.2686** | **2806.3067** | **2806.3041** | **0.95** | **1** | **0** | **8** | **1** |  | **K.EKLCYVALDFEQEMATAASSSSLEK.S** |

  

|  |  |
| --- | --- |
|  | |
|  | **Proteins matching the same set of peptides:** |

|  |  |
| --- | --- |
|  | sp|P63261|ACTG\_HUMAN    **Mass:** 42108    **Score:** 376    **Matches:** 26(18)  **Sequences:** 12(10) |
|  | Actin, cytoplasmic 2 OS=Homo sapiens OX=9606 GN=ACTG1 PE=1 SV=1 |

---

|  |  |
| --- | --- |
| **17.** | sp|P68371|TBB4B\_HUMAN    **Mass:** 50255    **Score:** 346    **Matches:** 13(9)  **Sequences:** 10(7)  **emPAI:** 0.77 |
|  | Tubulin beta-4B chain OS=Homo sapiens OX=9606 GN=TUBB4B PE=1 SV=1 |

|  |  |
| --- | --- |
|  | Check to include this hit in error tolerant search or archive report |
|  |  |

|  |  |  |  |  |  |  |  |  |  |  |  |
| --- | --- | --- | --- | --- | --- | --- | --- | --- | --- | --- | --- |
|  | **Query** | **Observed** | **Mr(expt)** | **Mr(calc)** | **ppm** | **Miss** | **Score** | **Expect** | **Rank** | **Unique** | **Peptide** |
|  | 1061 | 514.7636 | 1027.5127 | 1027.5121 | 0.58 | 0 | 40 | 0.0013 | 1 |  | K.TAVCDIPPR.G |
|  | 1098 | 520.3007 | 1038.5868 | 1038.5862 | 0.54 | 0 | 18 | 0.13 | 1 |  | R.YLTVAAVFR.G |
|  | 1586 | 565.8026 | 1129.5907 | 1129.5880 | 2.36 | 0 | 47 | 0.00043 | 1 |  | R.FPGQLNADLR.K |
|  | 1587 | 565.8045 | 1129.5944 | 1129.5880 | 5.71 | 0 | (16) | 0.57 | 1 |  | R.FPGQLNADLR.K |
|  | 1646 | 572.3210 | 1142.6275 | 1142.6270 | 0.44 | 0 | 47 | 0.00034 | 1 |  | K.LAVNMVPFPR.L |
|  | 1983 | 615.3021 | 1228.5896 | 1228.5910 | -1.19 | 0 | 73 | 5.6e-007 | 1 |  | R.ISEQFTAMFR.R |
|  | 2329 | 660.3538 | 1318.6931 | 1318.6955 | -1.84 | 0 | (52) | 0.00011 | 1 |  | R.IMNTFSVVPSPK.V |
|  | 2330 | 660.3572 | 1318.6998 | 1318.6955 | 3.26 | 0 | 78 | 2.8e-007 | 1 |  | R.IMNTFSVVPSPK.V |
|  | 2798 | 723.8500 | 1445.6854 | 1445.6820 | 2.34 | 0 | 47 | 0.00028 | 1 |  | K.EVDEQMLNVQNK.N |
|  | 3348 | 848.9194 | 1695.8243 | 1695.8257 | -0.79 | 0 | 5 | 5 | 1 |  | K.NSSYFVEWIPNNVK.T |
|  | 3655 | 608.3128 | 1821.9166 | 1821.9156 | 0.57 | 0 | (69) | 1.7e-006 | 1 | U | R.EIVHLQAGQCGNQIGAK.F |
|  | 3656 | 911.9659 | 1821.9173 | 1821.9156 | 0.97 | 0 | 80 | 1.3e-007 | 1 | U | R.EIVHLQAGQCGNQIGAK.F |
|  | 4310 | 786.0702 | 2355.1889 | 2355.1583 | 13.0 | 1 | 7 | 2.1 | 1 |  | R.LHFFMPGFAPLTSRGSQQYR.A + Oxidation (M) |

  


---

|  |  |
| --- | --- |
| **18.** | sp|P16989|YBOX3\_HUMAN    **Mass:** 40066    **Score:** 288    **Matches:** 11(8)  **Sequences:** 7(6)  **emPAI:** 0.74 |
|  | Y-box-binding protein 3 OS=Homo sapiens OX=9606 GN=YBX3 PE=1 SV=4 |

|  |  |
| --- | --- |
|  | Check to include this hit in error tolerant search or archive report |
|  |  |

|  |  |  |  |  |  |  |  |  |  |  |  |
| --- | --- | --- | --- | --- | --- | --- | --- | --- | --- | --- | --- |
|  | **Query** | **Observed** | **Mr(expt)** | **Mr(calc)** | **ppm** | **Miss** | **Score** | **Expect** | **Rank** | **Unique** | **Peptide** |
|  | 23 | 361.1921 | 720.3697 | 720.3707 | -1.49 | 0 | 23 | 0.12 | 1 |  | K.WFNVR.N |
|  | 24 | 361.1923 | 720.3700 | 720.3707 | -0.99 | 0 | (14) | 0.87 | 1 |  | K.WFNVR.N |
|  | 734 | 470.7356 | 939.4566 | 939.4563 | 0.36 | 0 | 50 | 0.00013 | 1 |  | R.NGYGFINR.N |
|  | 2211 | 643.8401 | 1285.6657 | 1285.6667 | -0.71 | 0 | 45 | 0.00058 | 1 |  | K.EDVFVHQTAIK.K |
|  | 3092 | **780.3989** | **1558.7832** | **1558.7852** | **-1.31** | **0** | **65** | **5.3e-006** | **1** | **U** | **K.DGVPEGAQLQGPVHR.N** |
|  | 3457 | 582.3002 | 1743.8789 | 1743.8792 | -0.17 | 1 | (39) | 0.0017 | 1 |  | R.NDTKEDVFVHQTAIK.K |
|  | 3458 | 582.3002 | 1743.8789 | 1743.8792 | -0.17 | 1 | (49) | 0.00019 | 1 |  | R.NDTKEDVFVHQTAIK.K |
|  | 3459 | 872.9470 | 1743.8794 | 1743.8792 | 0.12 | 1 | 86 | 3.3e-008 | 1 |  | R.NDTKEDVFVHQTAIK.K |
|  | 3555 | **891.9351** | **1781.8556** | **1781.8544** | **0.65** | **0** | **71** | **9.7e-007** | **1** | **U** | **K.GAEAANVTGPDGVPVEGSR.Y** |
|  | 3600 | 898.4061 | 1794.7976 | 1794.8160 | -10.25 | 0 | (1) | 6.2 | 5 |  | R.SVGDGETVEFDVVEGEK.G |
|  | 3601 | 898.4174 | 1794.8203 | 1794.8160 | 2.40 | 0 | 40 | 0.00086 | 1 |  | R.SVGDGETVEFDVVEGEK.G |

  


---

|  |  |
| --- | --- |
| **19.** | sp|Q07666|KHDR1\_HUMAN    **Mass:** 48311    **Score:** 287    **Matches:** 17(10)  **Sequences:** 8(6)  **emPAI:** 0.94 |
|  | KH domain-containing, RNA-binding, signal transduction-associated protein 1 OS=Homo sapiens OX=9606 GN=KHDRBS1 PE=1 SV=1 |

|  |  |
| --- | --- |
|  | Check to include this hit in error tolerant search or archive report |
|  |  |

|  |  |  |  |  |  |  |  |  |  |  |  |
| --- | --- | --- | --- | --- | --- | --- | --- | --- | --- | --- | --- |
|  | **Query** | **Observed** | **Mr(expt)** | **Mr(calc)** | **ppm** | **Miss** | **Score** | **Expect** | **Rank** | **Unique** | **Peptide** |
|  | 10 | **356.1946** | **710.3746** | **710.3752** | **-0.79** | **0** | **26** | **0.021** | **1** |  | **K.FNFVGK.I** |
|  | 162 | 398.2395 | 794.4644 | 794.4511 | 16.8 | 1 | 6 | 2 | 4 | U | R.GAPAPRAR.T |
|  | 1100 | **520.8084** | **1039.6023** | **1039.6026** | **-0.30** | **0** | **(6)** | **1.3** | **1** | **U** | **K.ILGPQGNTIK.R** |
|  | 1101 | **520.8087** | **1039.6029** | **1039.6026** | **0.29** | **0** | **24** | **0.018** | **1** | **U** | **K.ILGPQGNTIK.R** |
|  | 1415 | **547.2841** | **1092.5536** | **1092.5525** | **0.99** | **0** | **30** | **0.015** | **1** |  | **K.YLPELMAEK.D** |
|  | 1416 | **547.2849** | **1092.5553** | **1092.5525** | **2.54** | **0** | **(18)** | **0.26** | **1** |  | **K.YLPELMAEK.D** |
|  | 1488 | **555.2813** | **1108.5479** | **1108.5474** | **0.49** | **0** | **(13)** | **0.74** | **1** |  | **K.YLPELMAEK.D + Oxidation (M)** |
|  | 1489 | **555.2819** | **1108.5492** | **1108.5474** | **1.59** | **0** | **(22)** | **0.088** | **1** |  | **K.YLPELMAEK.D + Oxidation (M)** |
|  | 1862 | **399.5752** | **1195.7037** | **1195.7037** | **-0.03** | **1** | **(16)** | **0.11** | **1** | **U** | **K.ILGPQGNTIKR.L** |
|  | 1863 | **598.8592** | **1195.7038** | **1195.7037** | **0.12** | **1** | **18** | **0.061** | **1** | **U** | **K.ILGPQGNTIKR.L** |
|  | 2556 | **692.8170** | **1383.6194** | **1383.6201** | **-0.54** | **0** | **(48)** | **0.00012** | **1** | **U** | **R.SGSMDPSGAHPSVR.Q** |
|  | 2557 | **462.2138** | **1383.6194** | **1383.6201** | **-0.50** | **0** | **(35)** | **0.0026** | **1** | **U** | **R.SGSMDPSGAHPSVR.Q** |
|  | 2558 | **692.8171** | **1383.6197** | **1383.6201** | **-0.28** | **0** | **65** | **2.4e-006** | **1** | **U** | **R.SGSMDPSGAHPSVR.Q** |
|  | 3209 | **812.8598** | **1623.7050** | **1623.7053** | **-0.13** | **0** | **52** | **3.4e-005** | **1** | **U** | **K.DDEENYLDLFSHK.N** |
|  | 3478 | **876.9061** | **1751.7976** | **1751.8002** | **-1.50** | **1** | **91** | **7.8e-009** | **1** | **U** | **K.KDDEENYLDLFSHK.N** |
|  | 3479 | **584.9406** | **1751.8000** | **1751.8002** | **-0.12** | **1** | **(44)** | **0.00034** | **1** | **U** | **K.KDDEENYLDLFSHK.N** |
|  | 3480 | **438.9574** | **1751.8004** | **1751.8002** | **0.09** | **1** | **(39)** | **0.0012** | **1** | **U** | **K.KDDEENYLDLFSHK.N** |

  


---

|  |  |
| --- | --- |
| **20.** | sp|P08729|K2C7\_HUMAN    **Mass:** 51411    **Score:** 260    **Matches:** 18(11)  **Sequences:** 15(9)  **emPAI:** 0.86 |
|  | Keratin, type II cytoskeletal 7 OS=Homo sapiens OX=9606 GN=KRT7 PE=1 SV=5 |

|  |  |
| --- | --- |
|  | Check to include this hit in error tolerant search or archive report |
|  |  |

|  |  |  |  |  |  |  |  |  |  |  |  |
| --- | --- | --- | --- | --- | --- | --- | --- | --- | --- | --- | --- |
|  | **Query** | **Observed** | **Mr(expt)** | **Mr(calc)** | **ppm** | **Miss** | **Score** | **Expect** | **Rank** | **Unique** | **Peptide** |
|  | 248 | 414.2183 | 826.4220 | 826.4225 | -0.57 | 0 | (29) | 0.014 | 1 |  | K.FASFIDK.V |
|  | 249 | 414.2186 | 826.4226 | 826.4225 | 0.11 | 0 | 44 | 0.00045 | 1 |  | K.FASFIDK.V |
|  | 944 | 500.2269 | 998.4392 | 998.4379 | 1.31 | 0 | (3) | 2.9 | 2 | U | K.DVDAAYMSK.V |
|  | 945 | **500.2274** | **998.4402** | **998.4379** | **2.29** | **0** | **24** | **0.021** | **1** | **U** | **K.DVDAAYMSK.V** |
|  | 1172 | **523.2868** | **1044.5590** | **1044.5604** | **-1.27** | **0** | **10** | **1.8** | **1** | **U** | **K.WTLLQEQK.S** |
|  | 1358 | 361.5378 | 1081.5917 | 1081.5920 | -0.31 | 1 | (31) | 0.0083 | 1 |  | K.FASFIDKVR.F |
|  | 1359 | 541.8038 | 1081.5931 | 1081.5920 | 1.00 | 1 | 50 | 0.00011 | 1 |  | K.FASFIDKVR.F |
|  | 1404 | **546.7890** | **1091.5634** | **1091.5611** | **2.14** | **0** | **32** | **0.012** | **1** | **U** | **K.FETLQAQAGK.H** |
|  | 1463 | **552.7938** | **1103.5731** | **1103.5724** | **0.66** | **0** | **52** | **0.00012** | **1** | **U** | **R.SAYGGPVGAGIR.E** |
|  | 1560 | **563.7762** | **1125.5378** | **1125.5376** | **0.20** | **0** | **15** | **0.42** | **1** |  | **R.EYQELMSVK.L** |
|  | 1858 | **598.7859** | **1195.5573** | **1195.5509** | **5.38** | **0** | **36** | **0.0035** | **1** |  | **R.AEAEAWYQTK.F** |
|  | 2043 | **621.8265** | **1241.6385** | **1241.6364** | **1.70** | **0** | **81** | **1.1e-007** | **1** | **U** | **R.GQLEALQVDGGR.L** |
|  | 2323 | **439.8773** | **1316.6100** | **1316.6109** | **-0.64** | **1** | **1** | **8.7** | **9** | **U** | **K.NKYEDEINHR.T** |
|  | 2433 | **674.8779** | **1347.7413** | **1347.7398** | **1.12** | **1** | **15** | **0.42** | **1** |  | **R.TAAENEFVVLKK.D** |
|  | 2499 | **455.5709** | **1363.6908** | **1363.6885** | **1.75** | **0** | **4** | **6.2** | **5** | **U** | **M.SIHFSSPVFTSR.S** |
|  | 2686 | **709.8669** | **1417.7192** | **1417.7201** | **-0.65** | **0** | **65** | **6.2e-006** | **1** | **U** | **K.VDALNDEINFLR.T** |
|  | 3643 | **604.9946** | **1811.9621** | **1811.9628** | **-0.42** | **1** | **37** | **0.0018** | **1** | **U** | **K.LEAAIAEAEERGELALK.D** |
|  | 4184 | **747.0359** | **2238.0858** | **2238.1089** | **-10.31** | **1** | **2** | **8.2** | **2** | **U** | **R.SLDLDGIIAEVKAQYEEMAK.C + Oxidation (M)** |

  


---

|  |  |
| --- | --- |
| **21.** | sp|P13646|K1C13\_HUMAN    **Mass:** 49900    **Score:** 239    **Matches:** 13(7)  **Sequences:** 7(5)  **emPAI:** 0.47 |
|  | Keratin, type I cytoskeletal 13 OS=Homo sapiens OX=9606 GN=KRT13 PE=1 SV=4 |

|  |  |
| --- | --- |
|  | Check to include this hit in error tolerant search or archive report |
|  |  |

|  |  |  |  |  |  |  |  |  |  |  |  |
| --- | --- | --- | --- | --- | --- | --- | --- | --- | --- | --- | --- |
|  | **Query** | **Observed** | **Mr(expt)** | **Mr(calc)** | **ppm** | **Miss** | **Score** | **Expect** | **Rank** | **Unique** | **Peptide** |
|  | 69 | **374.7170** | **747.4194** | **747.4200** | **-0.85** | **0** | **6** | **2.9** | **6** |  | **K.MLLDIK.T + Oxidation (M)** |
|  | 189 | 404.2032 | 806.3918 | 806.3923 | -0.61 | 0 | 43 | 0.0011 | 1 |  | R.LAADDFR.L |
|  | 190 | 404.2034 | 806.3923 | 806.3923 | 0.06 | 0 | (31) | 0.017 | 1 |  | R.LAADDFR.L |
|  | 1883 | 601.3116 | 1200.6087 | 1200.6098 | -0.92 | 0 | 73 | 6.7e-007 | 1 |  | R.QSVEADINGLR.R |
|  | 2264 | 651.3348 | 1300.6550 | 1300.6510 | 3.07 | 0 | 80 | 1.6e-007 | 1 |  | R.ALEEANADLEVK.I |
|  | 2462 | 679.3623 | 1356.7100 | 1356.7110 | -0.67 | 1 | (12) | 0.97 | 4 |  | R.QSVEADINGLRR.V |
|  | 2463 | 679.3630 | 1356.7114 | 1356.7110 | 0.34 | 1 | (20) | 0.17 | 1 |  | R.QSVEADINGLRR.V |
|  | 2464 | 453.2445 | 1356.7116 | 1356.7110 | 0.49 | 1 | (12) | 0.97 | 1 |  | R.QSVEADINGLRR.V |
|  | 2465 | 453.2445 | 1356.7118 | 1356.7110 | 0.62 | 1 | (18) | 0.26 | 2 |  | R.QSVEADINGLRR.V |
|  | 2466 | 679.3632 | 1356.7119 | 1356.7110 | 0.69 | 1 | 51 | 0.00013 | 1 |  | R.QSVEADINGLRR.V |
|  | 2467 | 453.2449 | 1356.7128 | 1356.7110 | 1.35 | 1 | (47) | 0.00032 | 1 |  | R.QSVEADINGLRR.V |
|  | 2539 | 460.5807 | 1378.7202 | 1378.7204 | -0.21 | 1 | 57 | 3.4e-005 | 1 |  | K.TRLEQEIATYR.S |
|  | 2596 | **696.8434** | **1391.6722** | **1391.6867** | **-10.44** | **0** | **1** | **10** | **3** | **U** | **K.MIGFPSSAGSVSPR.S** |

  


---

|  |  |
| --- | --- |
| **22.** | sp|P19012|K1C15\_HUMAN    **Mass:** 49409    **Score:** 239    **Matches:** 14(9)  **Sequences:** 9(6)  **emPAI:** 0.57 |
|  | Keratin, type I cytoskeletal 15 OS=Homo sapiens OX=9606 GN=KRT15 PE=1 SV=3 |

|  |  |
| --- | --- |
|  | Check to include this hit in error tolerant search or archive report |
|  |  |

|  |  |  |  |  |  |  |  |  |  |  |  |
| --- | --- | --- | --- | --- | --- | --- | --- | --- | --- | --- | --- |
|  | **Query** | **Observed** | **Mr(expt)** | **Mr(calc)** | **ppm** | **Miss** | **Score** | **Expect** | **Rank** | **Unique** | **Peptide** |
|  | 69 | 374.7170 | 747.4194 | 747.4200 | -0.85 | 0 | 6 | 2.9 | 6 |  | K.MLLDIK.T + Oxidation (M) |
|  | 189 | 404.2032 | 806.3918 | 806.3923 | -0.61 | 0 | 43 | 0.0011 | 1 |  | R.LAADDFR.L |
|  | 190 | 404.2034 | 806.3923 | 806.3923 | 0.06 | 0 | (31) | 0.017 | 1 |  | R.LAADDFR.L |
|  | 192 | 405.2236 | 808.4326 | 808.4330 | -0.56 | 0 | (31) | 0.012 | 1 |  | R.LASYLDK.V |
|  | 193 | 405.2237 | 808.4329 | 808.4330 | -0.19 | 0 | 43 | 0.00066 | 1 |  | R.LASYLDK.V |
|  | 1066 | 515.3005 | 1028.5865 | 1028.5866 | -0.06 | 0 | 43 | 0.00066 | 1 |  | R.VLDELTLAR.T |
|  | 1067 | 515.3011 | 1028.5876 | 1028.5866 | 1.01 | 0 | (23) | 0.063 | 1 |  | R.VLDELTLAR.T |
|  | 1267 | 355.5415 | 1063.6028 | 1063.6026 | 0.18 | 1 | 49 | 9.1e-005 | 1 |  | R.LASYLDKVR.A |
|  | 1268 | 532.8089 | 1063.6032 | 1063.6026 | 0.64 | 1 | (45) | 0.00026 | 1 |  | R.LASYLDKVR.A |
|  | 1809 | 395.9031 | 1184.6873 | 1184.6877 | -0.32 | 1 | 2 | 6.4 | 4 |  | R.RVLDELTLAR.T |
|  | 1810 | 395.9031 | 1184.6873 | 1184.6877 | -0.32 | 1 | (1) | 7.2 | 3 |  | R.RVLDELTLAR.T |
|  | 2264 | 651.3348 | 1300.6550 | 1300.6510 | 3.07 | 0 | 80 | 1.6e-007 | 1 |  | R.ALEEANADLEVK.I |
|  | 2539 | 460.5807 | 1378.7202 | 1378.7204 | -0.21 | 1 | 57 | 3.4e-005 | 1 |  | K.TRLEQEIATYR.S |
|  | 4235 | **761.3872** | **2281.1398** | **2281.1107** | **12.7** | **1** | **5** | **3.6** | **3** | **U** | **K.EVASNTEMIQTSKTEITDLR.R + Oxidation (M)** |

  


---

|  |  |
| --- | --- |
| **23.** | sp|O95678|K2C75\_HUMAN    **Mass:** 59809    **Score:** 222    **Matches:** 16(11)  **Sequences:** 11(7)  **emPAI:** 0.90 |
|  | Keratin, type II cytoskeletal 75 OS=Homo sapiens OX=9606 GN=KRT75 PE=1 SV=2 |

|  |  |
| --- | --- |
|  | Check to include this hit in error tolerant search or archive report |
|  |  |

|  |  |  |  |  |  |  |  |  |  |  |  |
| --- | --- | --- | --- | --- | --- | --- | --- | --- | --- | --- | --- |
|  | **Query** | **Observed** | **Mr(expt)** | **Mr(calc)** | **ppm** | **Miss** | **Score** | **Expect** | **Rank** | **Unique** | **Peptide** |
|  | 248 | 414.2183 | 826.4220 | 826.4225 | -0.57 | 0 | (29) | 0.014 | 1 |  | K.FASFIDK.V |
|  | 249 | 414.2186 | 826.4226 | 826.4225 | 0.11 | 0 | 44 | 0.00045 | 1 |  | K.FASFIDK.V |
|  | 965 | 503.2367 | 1004.4589 | 1004.4597 | -0.78 | 0 | 22 | 0.08 | 1 |  | K.LLEGEECR.L |
|  | 1044 | 513.7310 | 1025.4475 | 1025.4488 | -1.26 | 0 | 31 | 0.0057 | 1 |  | K.DVDAAYMNK.V |
|  | 1284 | 533.7620 | 1065.5094 | 1065.5090 | 0.30 | 1 | 10 | 1.6 | 1 |  | R.YEDEINKR.T |
|  | 1358 | 361.5378 | 1081.5917 | 1081.5920 | -0.31 | 1 | (31) | 0.0083 | 1 |  | K.FASFIDKVR.F |
|  | 1359 | 541.8038 | 1081.5931 | 1081.5920 | 1.00 | 1 | 50 | 0.00011 | 1 |  | K.FASFIDKVR.F |
|  | 1604 | 567.2844 | 1132.5542 | 1132.5546 | -0.40 | 1 | 20 | 0.17 | 1 |  | R.KLLEGEECR.L |
|  | 1605 | 378.5254 | 1132.5543 | 1132.5546 | -0.33 | 1 | (18) | 0.29 | 1 |  | R.KLLEGEECR.L |
|  | 1719 | 583.2961 | 1164.5777 | 1164.5775 | 0.21 | 0 | 50 | 0.00016 | 1 |  | K.YEELQVTAGR.H |
|  | 1933 | 606.7818 | 1211.5490 | 1211.5458 | 2.65 | 0 | 25 | 0.028 | 1 |  | R.AEAESWYQTK.Y |
|  | 2364 | 665.3669 | 1328.7193 | 1328.7187 | 0.46 | 0 | 23 | 0.087 | 1 |  | R.NLDLDSIIAEVK.A |
|  | 2827 | 728.3465 | 1454.6784 | 1454.6790 | -0.36 | 1 | (25) | 0.038 | 1 |  | R.SRAEAESWYQTK.Y |
|  | 2828 | 485.9006 | 1454.6800 | 1454.6790 | 0.69 | 1 | 33 | 0.0061 | 1 |  | R.SRAEAESWYQTK.Y |
|  | 3342 | 848.4156 | 1694.8166 | 1694.8185 | -1.10 | 1 | 79 | 1.7e-007 | 1 |  | K.DVDAAYMNKVELEAK.V |
|  | 3344 | 565.9470 | 1694.8192 | 1694.8185 | 0.43 | 1 | (31) | 0.012 | 1 |  | K.DVDAAYMNKVELEAK.V |

  


---

|  |  |
| --- | --- |
| **24.** | sp|P68032|ACTC\_HUMAN    **Mass:** 42334    **Score:** 206    **Matches:** 21(13)  **Sequences:** 9(8)  **emPAI:** 1.66 |
|  | Actin, alpha cardiac muscle 1 OS=Homo sapiens OX=9606 GN=ACTC1 PE=1 SV=1 |

|  |  |
| --- | --- |
|  | Check to include this hit in error tolerant search or archive report |
|  |  |

|  |  |  |  |  |  |  |  |  |  |  |  |
| --- | --- | --- | --- | --- | --- | --- | --- | --- | --- | --- | --- |
|  | **Query** | **Observed** | **Mr(expt)** | **Mr(calc)** | **ppm** | **Miss** | **Score** | **Expect** | **Rank** | **Unique** | **Peptide** |
|  | 162 | 398.2395 | 794.4644 | 794.4650 | -0.71 | 0 | 28 | 0.013 | 1 |  | K.IIAPPER.K |
|  | 854 | 488.7276 | 975.4406 | 975.4410 | -0.37 | 0 | 41 | 0.00083 | 1 |  | K.AGFAGDDAPR.A |
|  | 939 | 499.7468 | 997.4790 | 997.4790 | 0.03 | 0 | (18) | 0.18 | 1 |  | R.DLTDYLMK.I |
|  | 940 | 499.7468 | 997.4790 | 997.4790 | 0.03 | 0 | (28) | 0.02 | 1 |  | R.DLTDYLMK.I |
|  | 995 | 507.7447 | 1013.4748 | 1013.4739 | 0.88 | 0 | 30 | 0.0079 | 1 |  | R.DLTDYLMK.I + Oxidation (M) |
|  | 1703 | 581.3121 | 1160.6097 | 1160.6111 | -1.19 | 0 | (20) | 0.2 | 1 |  | K.EITALAPSTMK.I |
|  | 1704 | 581.3134 | 1160.6123 | 1160.6111 | 1.04 | 0 | (30) | 0.018 | 1 |  | K.EITALAPSTMK.I |
|  | 1751 | 586.2892 | 1170.5638 | 1170.5638 | 0.01 | 0 | (10) | 1.2 | 1 |  | R.HQGVMVGMGQK.D |
|  | 1752 | 586.2892 | 1170.5638 | 1170.5638 | 0.01 | 0 | 66 | 3e-006 | 1 |  | R.HQGVMVGMGQK.D |
|  | 1772 | 589.3107 | 1176.6068 | 1176.6060 | 0.67 | 0 | 36 | 0.0046 | 1 |  | K.EITALAPSTMK.I + Oxidation (M) |
|  | 1870 | 599.7648 | 1197.5150 | 1197.5150 | 0.03 | 0 | 2 | 3.4 | 1 |  | K.DSYVGDEAQSK.R |
|  | 1874 | 400.2401 | 1197.6986 | 1197.6982 | 0.30 | 0 | 28 | 0.011 | 1 |  | R.AVFPSIVGRPR.H |
|  | 2959 | 379.6922 | 1514.7397 | 1514.7419 | -1.43 | 0 | (15) | 0.41 | 1 |  | K.IWHHTFYNELR.V |
|  | 2960 | 758.3784 | 1514.7423 | 1514.7419 | 0.28 | 0 | (35) | 0.0039 | 1 |  | K.IWHHTFYNELR.V |
|  | 2963 | 505.9216 | 1514.7430 | 1514.7419 | 0.73 | 0 | (2) | 8.8 | 2 |  | K.IWHHTFYNELR.V |
|  | 2965 | 505.9216 | 1514.7431 | 1514.7419 | 0.79 | 0 | 36 | 0.0029 | 1 |  | K.IWHHTFYNELR.V |
|  | 2966 | 505.9217 | 1514.7433 | 1514.7419 | 0.97 | 0 | (9) | 1.6 | 2 |  | K.IWHHTFYNELR.V |
|  | 3912 | **980.9569** | **1959.8993** | **1959.9036** | **-2.23** | **0** | **35** | **0.0024** | **1** | **U** | **K.YPIEHGIITNWDDMEK.I** |
|  | 3913 | **654.3076** | **1959.9009** | **1959.9036** | **-1.42** | **0** | **(32)** | **0.0048** | **1** | **U** | **K.YPIEHGIITNWDDMEK.I** |
|  | 3923 | **659.6396** | **1975.8969** | **1975.8986** | **-0.82** | **0** | **(19)** | **0.1** | **1** | **U** | **K.YPIEHGIITNWDDMEK.I + Oxidation (M)** |
|  | 3924 | **988.9562** | **1975.8978** | **1975.8986** | **-0.38** | **0** | **(29)** | **0.011** | **1** | **U** | **K.YPIEHGIITNWDDMEK.I + Oxidation (M)** |

  

|  |  |
| --- | --- |
|  | |
|  | **Proteins matching the same set of peptides:** |

|  |  |
| --- | --- |
|  | sp|P68133|ACTS\_HUMAN    **Mass:** 42366    **Score:** 206    **Matches:** 21(13)  **Sequences:** 9(8) |
|  | Actin, alpha skeletal muscle OS=Homo sapiens OX=9606 GN=ACTA1 PE=1 SV=1 |

---

|  |  |
| --- | --- |
| **25.** | sp|P08727|K1C19\_HUMAN    **Mass:** 44079    **Score:** 205    **Matches:** 17(10)  **Sequences:** 11(7)  **emPAI:** 0.78 |
|  | Keratin, type I cytoskeletal 19 OS=Homo sapiens OX=9606 GN=KRT19 PE=1 SV=4 |

|  |  |
| --- | --- |
|  | Check to include this hit in error tolerant search or archive report |
|  |  |

|  |  |  |  |  |  |  |  |  |  |  |  |
| --- | --- | --- | --- | --- | --- | --- | --- | --- | --- | --- | --- |
|  | **Query** | **Observed** | **Mr(expt)** | **Mr(calc)** | **ppm** | **Miss** | **Score** | **Expect** | **Rank** | **Unique** | **Peptide** |
|  | 189 | 404.2032 | 806.3918 | 806.3923 | -0.61 | 0 | 43 | 0.0011 | 1 |  | R.LAADDFR.T |
|  | 190 | 404.2034 | 806.3923 | 806.3923 | 0.06 | 0 | (31) | 0.017 | 1 |  | R.LAADDFR.T |
|  | 192 | 405.2236 | 808.4326 | 808.4330 | -0.56 | 0 | (31) | 0.012 | 1 |  | R.LASYLDK.V |
|  | 193 | 405.2237 | 808.4329 | 808.4330 | -0.19 | 0 | 43 | 0.00066 | 1 |  | R.LASYLDK.V |
|  | 919 | 497.2536 | 992.4927 | 992.4927 | 0.02 | 0 | 10 | 2 | 4 |  | K.FETEQALR.M |
|  | 926 | **498.2599** | **994.5052** | **994.4944** | **10.8** | **0** | **3** | **6.6** | **4** | **U** | **R.APSIHGGSGGR.G** |
|  | 1066 | 515.3005 | 1028.5865 | 1028.5866 | -0.06 | 0 | 43 | 0.00066 | 1 |  | R.VLDELTLAR.T |
|  | 1067 | 515.3011 | 1028.5876 | 1028.5866 | 1.01 | 0 | (23) | 0.063 | 1 |  | R.VLDELTLAR.T |
|  | 1102 | 521.3063 | 1040.5981 | 1040.5978 | 0.29 | 0 | 50 | 7.2e-005 | 1 |  | R.IVLQIDNAR.L |
|  | 1267 | 355.5415 | 1063.6028 | 1063.6026 | 0.18 | 1 | 49 | 9.1e-005 | 1 |  | R.LASYLDKVR.A |
|  | 1268 | 532.8089 | 1063.6032 | 1063.6026 | 0.64 | 1 | (45) | 0.00026 | 1 |  | R.LASYLDKVR.A |
|  | 1809 | 395.9031 | 1184.6873 | 1184.6877 | -0.32 | 1 | 2 | 6.4 | 4 |  | R.RVLDELTLAR.T |
|  | 1810 | 395.9031 | 1184.6873 | 1184.6877 | -0.32 | 1 | (1) | 7.2 | 3 |  | R.RVLDELTLAR.T |
|  | 1907 | 602.8027 | 1203.5909 | 1203.5918 | -0.70 | 0 | 45 | 0.00053 | 1 |  | R.MSVEADINGLR.R |
|  | 1956 | 611.8193 | 1221.6241 | 1221.6353 | -9.17 | 1 | (5) | 5.6 | 4 |  | R.TKFETEQALR.M |
|  | 1957 | 611.8248 | 1221.6351 | 1221.6353 | -0.18 | 1 | 25 | 0.05 | 1 |  | R.TKFETEQALR.M |
|  | 2484 | 680.8541 | 1359.6936 | 1359.6929 | 0.52 | 1 | 11 | 1.5 | 2 |  | R.MSVEADINGLRR.V |

  


---

|  |  |
| --- | --- |
| **26.** | sp|Q7Z794|K2C1B\_HUMAN    **Mass:** 62149    **Score:** 205    **Matches:** 17(9)  **Sequences:** 13(5)  **emPAI:** 0.36 |
|  | Keratin, type II cytoskeletal 1b OS=Homo sapiens OX=9606 GN=KRT77 PE=2 SV=3 |

|  |  |
| --- | --- |
|  | Check to include this hit in error tolerant search or archive report |
|  |  |

|  |  |  |  |  |  |  |  |  |  |  |  |
| --- | --- | --- | --- | --- | --- | --- | --- | --- | --- | --- | --- |
|  | **Query** | **Observed** | **Mr(expt)** | **Mr(calc)** | **ppm** | **Miss** | **Score** | **Expect** | **Rank** | **Unique** | **Peptide** |
|  | 248 | 414.2183 | 826.4220 | 826.4225 | -0.57 | 0 | (29) | 0.014 | 1 |  | K.FASFIDK.V |
|  | 249 | 414.2186 | 826.4226 | 826.4225 | 0.11 | 0 | 44 | 0.00045 | 1 |  | K.FASFIDK.V |
|  | 1284 | 533.7620 | 1065.5094 | 1065.5090 | 0.30 | 1 | 10 | 1.6 | 1 |  | K.YEDEINKR.T |
|  | 1358 | 361.5378 | 1081.5917 | 1081.5920 | -0.31 | 1 | (31) | 0.0083 | 1 |  | K.FASFIDKVR.F |
|  | 1359 | 541.8038 | 1081.5931 | 1081.5920 | 1.00 | 1 | 50 | 0.00011 | 1 |  | K.FASFIDKVR.F |
|  | 1383 | **544.7913** | **1087.5681** | **1087.5695** | **-1.34** | **0** | **10** | **2.1** | **2** | **U** | **R.EQIMVLNNK.F** |
|  | 1542 | **561.3007** | **1120.5869** | **1120.5876** | **-0.68** | **0** | **4** | **6.6** | **1** | **U** | **R.TQYELIAQR.S** |
|  | 1776 | **589.8080** | **1177.6014** | **1177.6091** | **-6.55** | **0** | **17** | **0.34** | **1** | **U** | **K.YQELQITAGR.H** |
|  | 1936 | **607.2738** | **1212.5330** | **1212.5332** | **-0.17** | **0** | **26** | **0.014** | **1** | **U** | **R.SMQDVVEDYK.S** |
|  | 2162 | **637.3137** | **1272.6129** | **1272.6098** | **2.39** | **0** | **2** | **9** | **5** | **U** | **R.GEQALQDAWQK.L** |
|  | 2381 | **668.3617** | **1334.7088** | **1334.7082** | **0.48** | **0** | **14** | **0.53** | **1** | **U** | **R.VDTLTGEVNFLK.Y** |
|  | 2384 | 446.2419 | 1335.7037 | 1335.7034 | 0.22 | 1 | 6 | 3.1 | 4 | U | R.TGSENDFVVLKK.D |
|  | 2499 | 455.5709 | 1363.6908 | 1363.7096 | -13.74 | 1 | 3 | 8.9 | 10 | U | K.RTGSENDFVVLK.K |
|  | 2870 | 738.3964 | 1474.7782 | 1474.7780 | 0.12 | 0 | (49) | 0.00019 | 1 |  | R.FLEQQNQVLQTK.W |
|  | 2871 | 738.3976 | 1474.7806 | 1474.7780 | 1.78 | 0 | (64) | 6e-006 | 1 |  | R.FLEQQNQVLQTK.W |
|  | 2872 | 738.3978 | 1474.7810 | 1474.7780 | 2.04 | 0 | 69 | 1.8e-006 | 1 |  | R.FLEQQNQVLQTK.W |
|  | 3260 | **822.8621** | **1643.7096** | **1643.7111** | **-0.94** | **0** | **20** | **0.041** | **1** | **U** | **R.STSGFCQGGGVGGFGGGR.G** |

  


---

|  |  |
| --- | --- |
| **27.** | sp|Q6S8J3|POTEE\_HUMAN    **Mass:** 122882   **Score:** 184    **Matches:** 15(7)  **Sequences:** 8(4)  **emPAI:** 0.17 |
|  | POTE ankyrin domain family member E OS=Homo sapiens OX=9606 GN=POTEE PE=2 SV=3 |

|  |  |
| --- | --- |
|  | Check to include this hit in error tolerant search or archive report |
|  |  |

|  |  |  |  |  |  |  |  |  |  |  |  |
| --- | --- | --- | --- | --- | --- | --- | --- | --- | --- | --- | --- |
|  | **Query** | **Observed** | **Mr(expt)** | **Mr(calc)** | **ppm** | **Miss** | **Score** | **Expect** | **Rank** | **Unique** | **Peptide** |
|  | 114 | **384.7054** | **767.3961** | **767.4038** | **-10.00** | **0** | **7** | **2** | **1** | **U** | **K.HQSQLR.E** |
|  | 854 | 488.7276 | 975.4406 | 975.4410 | -0.37 | 0 | 41 | 0.00083 | 1 |  | K.AGFAGDDAPR.A |
|  | 1473 | 554.2658 | 1106.5171 | 1106.5277 | -9.64 | 0 | (0) | 13 | 5 | U | K.MSQELEINK.D + Oxidation (M) |
|  | 1475 | 554.2739 | 1106.5333 | 1106.5277 | 5.03 | 0 | 6 | 4 | 2 | U | K.MSQELEINK.D + Oxidation (M) |
|  | 1874 | 400.2401 | 1197.6986 | 1197.6982 | 0.30 | 0 | 28 | 0.011 | 1 |  | R.AVFPSIVGRPR.Q |
|  | 2959 | 379.6922 | 1514.7397 | 1514.7419 | -1.43 | 0 | (15) | 0.41 | 1 |  | K.IWHHTFYNELR.V |
|  | 2960 | 758.3784 | 1514.7423 | 1514.7419 | 0.28 | 0 | (35) | 0.0039 | 1 |  | K.IWHHTFYNELR.V |
|  | 2963 | 505.9216 | 1514.7430 | 1514.7419 | 0.73 | 0 | (2) | 8.8 | 2 |  | K.IWHHTFYNELR.V |
|  | 2965 | 505.9216 | 1514.7431 | 1514.7419 | 0.79 | 0 | 36 | 0.0029 | 1 |  | K.IWHHTFYNELR.V |
|  | 2966 | 505.9217 | 1514.7433 | 1514.7419 | 0.97 | 0 | (9) | 1.6 | 2 |  | K.IWHHTFYNELR.V |
|  | 2970 | 506.2375 | 1515.6906 | 1515.6954 | -3.12 | 0 | (55) | 2.6e-005 | 1 |  | K.QEYDESGPSIVHR.K |
|  | 2971 | 758.8532 | 1515.6919 | 1515.6954 | -2.31 | 0 | 72 | 5.6e-007 | 1 |  | K.QEYDESGPSIVHR.K |
|  | 2972 | 506.2394 | 1515.6963 | 1515.6954 | 0.62 | 0 | (37) | 0.0017 | 1 |  | K.QEYDESGPSIVHR.K |
|  | 3894 | **486.4757** | **1941.8739** | **1941.8448** | **15.0** | **1** | **1** | **5.6** | **7** | **U** | **K.DGDRELENFMAIEEMK.K + Oxidation (M)** |
|  | 4452 | 562.2686 | 2806.3067 | 2806.3041 | 0.95 | 1 | 0 | 8 | 1 |  | K.EKLCYVALDFEQEMATAASSSSLEK.S |

  


---

|  |  |
| --- | --- |
| **28.** | sp|Q04837|SSBP\_HUMAN    **Mass:** 17249    **Score:** 151    **Matches:** 6(5)  **Sequences:** 4(3)  **emPAI:** 1.05 |
|  | Single-stranded DNA-binding protein, mitochondrial OS=Homo sapiens OX=9606 GN=SSBP1 PE=1 SV=1 |

|  |  |
| --- | --- |
|  | Check to include this hit in error tolerant search or archive report |
|  |  |

|  |  |  |  |  |  |  |  |  |  |  |  |
| --- | --- | --- | --- | --- | --- | --- | --- | --- | --- | --- | --- |
|  | **Query** | **Observed** | **Mr(expt)** | **Mr(calc)** | **ppm** | **Miss** | **Score** | **Expect** | **Rank** | **Unique** | **Peptide** |
|  | 533 | **442.2501** | **882.4856** | **882.4923** | **-7.66** | **0** | **55** | **2.9e-005** | **1** | **U** | **R.VGQDPVLR.Q** |
|  | 885 | **493.2532** | **984.4918** | **984.4916** | **0.21** | **0** | **13** | **0.47** | **1** | **U** | **R.DVAYQYVK.K** |
|  | 1600 | **567.2451** | **1132.4757** | **1132.4747** | **0.91** | **0** | **31** | **0.0027** | **1** | **U** | **K.IDYGEYMDK.N** |
|  | 1660 | **575.2424** | **1148.4702** | **1148.4696** | **0.53** | **0** | **(20)** | **0.026** | **1** | **U** | **K.IDYGEYMDK.N + Oxidation (M)** |
|  | 3190 | **806.3782** | **1610.7419** | **1610.7424** | **-0.31** | **0** | **(49)** | **0.00012** | **1** | **U** | **R.SGDSEVYQLGDVSQK.T** |
|  | 3191 | **806.3785** | **1610.7424** | **1610.7424** | **0.00** | **0** | **75** | **3.3e-007** | **1** | **U** | **R.SGDSEVYQLGDVSQK.T** |

  


---

|  |  |
| --- | --- |
| **29.** | sp|P52597|HNRPF\_HUMAN    **Mass:** 45985    **Score:** 150    **Matches:** 9(6)  **Sequences:** 6(4)  **emPAI:** 0.52 |
|  | Heterogeneous nuclear ribonucleoprotein F OS=Homo sapiens OX=9606 GN=HNRNPF PE=1 SV=3 |

|  |  |
| --- | --- |
|  | Check to include this hit in error tolerant search or archive report |
|  |  |

|  |  |  |  |  |  |  |  |  |  |  |  |
| --- | --- | --- | --- | --- | --- | --- | --- | --- | --- | --- | --- |
|  | **Query** | **Observed** | **Mr(expt)** | **Mr(calc)** | **ppm** | **Miss** | **Score** | **Expect** | **Rank** | **Unique** | **Peptide** |
|  | 167 | **399.7232** | **797.4319** | **797.4323** | **-0.50** | **0** | **25** | **0.021** | **1** | **U** | **R.YIEVFK.S** |
|  | 519 | **440.2232** | **878.4319** | **878.4320** | **-0.19** | **0** | **14** | **0.55** | **1** | **U** | **R.GLPFGCTK.E** |
|  | 1019 | **511.2551** | **1020.4956** | **1020.4950** | **0.57** | **0** | **19** | **0.22** | **1** | **U** | **R.TEMDWVLK.H** |
|  | 1405 | **364.8644** | **1091.5714** | **1091.5724** | **-0.87** | **0** | **38** | **0.0027** | **1** |  | **R.VHIEIGPDGR.V** |
|  | 1406 | **546.7938** | **1091.5730** | **1091.5724** | **0.56** | **0** | **(33)** | **0.0082** | **1** |  | **R.VHIEIGPDGR.V** |
|  | 3222 | **815.8636** | **1629.7126** | **1629.7132** | **-0.33** | **0** | **61** | **3.1e-006** | **1** | **U** | **K.HSGPNSADSANDGFVR.L** |
|  | 3223 | **544.2449** | **1629.7128** | **1629.7132** | **-0.24** | **0** | **(57)** | **7.8e-006** | **1** | **U** | **K.HSGPNSADSANDGFVR.L** |
|  | 3879 | **645.6599** | **1933.9577** | **1933.9581** | **-0.19** | **0** | **32** | **0.0075** | **1** | **U** | **K.FMSVQRPGPYDRPGTAR.R** |
|  | 3897 | **488.4952** | **1949.9515** | **1949.9530** | **-0.78** | **0** | **(1)** | **9.8** | **5** | **U** | **K.FMSVQRPGPYDRPGTAR.R + Oxidation (M)** |

  


---

|  |  |
| --- | --- |
| **30.** | sp|P02768|ALBU\_HUMAN    **Mass:** 71317    **Score:** 142    **Matches:** 11(5)  **Sequences:** 9(3)  **emPAI:** 0.31 |
|  | Serum albumin OS=Homo sapiens OX=9606 GN=ALB PE=1 SV=2 |

|  |  |
| --- | --- |
|  | Check to include this hit in error tolerant search or archive report |
|  |  |

|  |  |  |  |  |  |  |  |  |  |  |  |
| --- | --- | --- | --- | --- | --- | --- | --- | --- | --- | --- | --- |
|  | **Query** | **Observed** | **Mr(expt)** | **Mr(calc)** | **ppm** | **Miss** | **Score** | **Expect** | **Rank** | **Unique** | **Peptide** |
|  | 156 | **395.2394** | **788.4643** | **788.4644** | **-0.05** | **0** | **39** | **0.0015** | **1** | **U** | **K.LVTDLTK.V** |
|  | 520 | **440.7329** | **879.4512** | **879.4338** | **19.8** | **0** | **1** | **14** | **1** | **U** | **K.AEFAEVSK.L** |
|  | 673 | **464.2504** | **926.4863** | **926.4861** | **0.19** | **0** | **23** | **0.065** | **1** | **U** | **K.YLYEIAR.R** |
|  | 734 | 470.7356 | 939.4566 | 939.4410 | 16.6 | 0 | 4 | 5 | 5 | U | K.DDNPNLPR.L |
|  | 794 | **480.7848** | **959.5550** | **959.5552** | **-0.28** | **0** | **19** | **0.12** | **1** | **U** | **K.FQNALLVR.Y** |
|  | 949 | **500.8056** | **999.5966** | **999.5964** | **0.15** | **0** | **13** | **0.56** | **1** | **U** | **K.QTALVELVK.H** |
|  | 1622 | **569.7524** | **1137.4902** | **1137.4907** | **-0.41** | **0** | **38** | **0.00077** | **1** | **U** | **K.CCTESLVNR.R** |
|  | 2792 | **722.3237** | **1442.6328** | **1442.6347** | **-1.35** | **0** | **12** | **0.39** | **1** | **U** | **K.YICENQDSISSK.L** |
|  | 3251 | **820.4715** | **1638.9284** | **1638.9305** | **-1.24** | **1** | **(31)** | **0.0028** | **1** | **U** | **K.KVPQVSTPTLVEVSR.N** |
|  | 3252 | **547.3173** | **1638.9301** | **1638.9305** | **-0.21** | **1** | **(49)** | **4.4e-005** | **1** | **U** | **K.KVPQVSTPTLVEVSR.N** |
|  | 3253 | **547.3174** | **1638.9303** | **1638.9305** | **-0.10** | **1** | **58** | **6.2e-006** | **1** | **U** | **K.KVPQVSTPTLVEVSR.N** |

  


---

|  |  |
| --- | --- |
| **31.** | sp|Q01546|K22O\_HUMAN    **Mass:** 66370    **Score:** 126    **Matches:** 17(6)  **Sequences:** 11(4)  **emPAI:** 0.40 |
|  | Keratin, type II cytoskeletal 2 oral OS=Homo sapiens OX=9606 GN=KRT76 PE=1 SV=2 |

|  |  |
| --- | --- |
|  | Check to include this hit in error tolerant search or archive report |
|  |  |

|  |  |  |  |  |  |  |  |  |  |  |  |
| --- | --- | --- | --- | --- | --- | --- | --- | --- | --- | --- | --- |
|  | **Query** | **Observed** | **Mr(expt)** | **Mr(calc)** | **ppm** | **Miss** | **Score** | **Expect** | **Rank** | **Unique** | **Peptide** |
|  | 248 | 414.2183 | 826.4220 | 826.4225 | -0.57 | 0 | (29) | 0.014 | 1 |  | K.FASFIDK.V |
|  | 249 | 414.2186 | 826.4226 | 826.4225 | 0.11 | 0 | 44 | 0.00045 | 1 |  | K.FASFIDK.V |
|  | 965 | 503.2367 | 1004.4589 | 1004.4597 | -0.78 | 0 | 22 | 0.08 | 1 |  | K.LLEGEECR.M |
|  | 1044 | 513.7310 | 1025.4475 | 1025.4488 | -1.27 | 0 | 16 | 0.16 | 2 |  | K.DVDAAFMNK.V + Oxidation (M) |
|  | 1149 | **523.2856** | **1044.5567** | **1044.5564** | **0.35** | **0** | **(0)** | **19** | **2** |  | **K.LGELQTTAGR.H** |
|  | 1168 | **523.2861** | **1044.5577** | **1044.5564** | **1.28** | **0** | **1** | **15** | **4** |  | **K.LGELQTTAGR.H** |
|  | 1284 | 533.7620 | 1065.5094 | 1065.5090 | 0.30 | 1 | 10 | 1.6 | 1 |  | K.YEDEINKR.T |
|  | 1358 | 361.5378 | 1081.5917 | 1081.5920 | -0.31 | 1 | (31) | 0.0083 | 1 |  | K.FASFIDKVR.F |
|  | 1359 | 541.8038 | 1081.5931 | 1081.5920 | 1.00 | 1 | 50 | 0.00011 | 1 |  | K.FASFIDKVR.F |
|  | 1473 | 554.2658 | 1106.5171 | 1106.5356 | -16.75 | 0 | (1) | 11 | 2 |  | R.AQYEEIAQR.S |
|  | 1475 | 554.2739 | 1106.5333 | 1106.5356 | -2.07 | 0 | 42 | 0.00091 | 1 |  | R.AQYEEIAQR.S |
|  | 1604 | 567.2844 | 1132.5542 | 1132.5546 | -0.40 | 1 | 20 | 0.17 | 1 |  | R.KLLEGEECR.M |
|  | 1605 | 378.5254 | 1132.5543 | 1132.5546 | -0.33 | 1 | (18) | 0.29 | 1 |  | R.KLLEGEECR.M |
|  | 1625 | 570.2732 | 1138.5318 | 1138.5328 | -0.89 | 0 | 29 | 0.015 | 1 |  | R.DYQELMNVK.L |
|  | 1680 | 578.2709 | 1154.5272 | 1154.5278 | -0.49 | 0 | (19) | 0.12 | 1 |  | R.DYQELMNVK.L + Oxidation (M) |
|  | 2980 | 507.9410 | 1520.8011 | 1520.8021 | -0.63 | 1 | 15 | 0.39 | 1 |  | R.LLRDYQELMNVK.L |
|  | 3341 | 847.9249 | 1693.8353 | 1693.8345 | 0.48 | 1 | 17 | 0.26 | 2 | U | K.DVDAAFMNKVELQAK.V + Oxidation (M) |

  


---

|  |  |
| --- | --- |
| **32.** | sp|Q5XKE5|K2C79\_HUMAN    **Mass:** 58085    **Score:** 125    **Matches:** 10(7)  **Sequences:** 7(5)  **emPAI:** 0.39 |
|  | Keratin, type II cytoskeletal 79 OS=Homo sapiens OX=9606 GN=KRT79 PE=1 SV=2 |

|  |  |
| --- | --- |
|  | Check to include this hit in error tolerant search or archive report |
|  |  |

|  |  |  |  |  |  |  |  |  |  |  |  |
| --- | --- | --- | --- | --- | --- | --- | --- | --- | --- | --- | --- |
|  | **Query** | **Observed** | **Mr(expt)** | **Mr(calc)** | **ppm** | **Miss** | **Score** | **Expect** | **Rank** | **Unique** | **Peptide** |
|  | 248 | 414.2183 | 826.4220 | 826.4225 | -0.57 | 0 | (29) | 0.014 | 1 |  | K.FASFIDK.V |
|  | 249 | 414.2186 | 826.4226 | 826.4225 | 0.11 | 0 | 44 | 0.00045 | 1 |  | K.FASFIDK.V |
|  | 747 | 473.2593 | 944.5039 | 944.5039 | 0.03 | 1 | 34 | 0.0099 | 1 |  | R.GRLDSELR.N |
|  | 1358 | 361.5378 | 1081.5917 | 1081.5920 | -0.31 | 1 | (31) | 0.0083 | 1 |  | K.FASFIDKVR.F |
|  | 1359 | 541.8038 | 1081.5931 | 1081.5920 | 1.00 | 1 | 50 | 0.00011 | 1 |  | K.FASFIDKVR.F |
|  | 1625 | 570.2732 | 1138.5318 | 1138.5328 | -0.89 | 0 | 29 | 0.015 | 1 |  | R.DYQELMNVK.L |
|  | 1680 | 578.2709 | 1154.5272 | 1154.5278 | -0.49 | 0 | (19) | 0.12 | 1 |  | R.DYQELMNVK.L + Oxidation (M) |
|  | 1858 | 598.7859 | 1195.5573 | 1195.5509 | 5.38 | 0 | 36 | 0.0035 | 1 |  | R.AEAEAWYQTK.Y |
|  | 2364 | 665.3669 | 1328.7193 | 1328.7187 | 0.46 | 0 | 23 | 0.087 | 1 |  | R.NLDLDSIIAEVK.A |
|  | 2980 | 507.9410 | 1520.8011 | 1520.8021 | -0.63 | 1 | 15 | 0.39 | 1 |  | R.LLRDYQELMNVK.L |

  


---

|  |  |
| --- | --- |
| **33.** | sp|P12035|K2C3\_HUMAN    **Mass:** 64549    **Score:** 124    **Matches:** 14(6)  **Sequences:** 9(4)  **emPAI:** 0.28 |
|  | Keratin, type II cytoskeletal 3 OS=Homo sapiens OX=9606 GN=KRT3 PE=1 SV=3 |

|  |  |
| --- | --- |
|  | Check to include this hit in error tolerant search or archive report |
|  |  |

|  |  |  |  |  |  |  |  |  |  |  |  |
| --- | --- | --- | --- | --- | --- | --- | --- | --- | --- | --- | --- |
|  | **Query** | **Observed** | **Mr(expt)** | **Mr(calc)** | **ppm** | **Miss** | **Score** | **Expect** | **Rank** | **Unique** | **Peptide** |
|  | 248 | 414.2183 | 826.4220 | 826.4225 | -0.57 | 0 | (29) | 0.014 | 1 |  | K.FASFIDK.V |
|  | 249 | 414.2186 | 826.4226 | 826.4225 | 0.11 | 0 | 44 | 0.00045 | 1 |  | K.FASFIDK.V |
|  | 841 | 487.2693 | 972.5241 | 972.5240 | 0.12 | 0 | 4 | 8.5 | 7 | U | K.SEIIELNR.M |
|  | 1149 | 523.2856 | 1044.5567 | 1044.5564 | 0.35 | 0 | (0) | 19 | 2 |  | K.LGELQTTAGR.H |
|  | 1168 | 523.2861 | 1044.5577 | 1044.5564 | 1.28 | 0 | 1 | 15 | 4 |  | K.LGELQTTAGR.H |
|  | 1284 | 533.7620 | 1065.5094 | 1065.5090 | 0.30 | 1 | 10 | 1.6 | 1 |  | K.YEDEINKR.T |
|  | 1358 | 361.5378 | 1081.5917 | 1081.5920 | -0.31 | 1 | (31) | 0.0083 | 1 |  | K.FASFIDKVR.F |
|  | 1359 | 541.8038 | 1081.5931 | 1081.5920 | 1.00 | 1 | 50 | 0.00011 | 1 |  | K.FASFIDKVR.F |
|  | 1625 | 570.2732 | 1138.5318 | 1138.5328 | -0.89 | 0 | 29 | 0.015 | 1 |  | R.DYQELMNVK.L |
|  | 1680 | 578.2709 | 1154.5272 | 1154.5278 | -0.49 | 0 | (19) | 0.12 | 1 |  | R.DYQELMNVK.L + Oxidation (M) |
|  | 1956 | 611.8193 | 1221.6241 | 1221.6241 | 0.02 | 0 | 50 | 0.00016 | 1 |  | R.TAAENEFVTLK.K |
|  | 1957 | 611.8248 | 1221.6351 | 1221.6241 | 9.01 | 0 | (2) | 10 | 6 |  | R.TAAENEFVTLK.K |
|  | 2686 | 709.8669 | 1417.7192 | 1417.7453 | -18.39 | 0 | 25 | 0.058 | 2 | U | K.VDALIDEIDFLR.T |
|  | 2980 | 507.9410 | 1520.8011 | 1520.8021 | -0.63 | 1 | 15 | 0.39 | 1 |  | R.LLRDYQELMNVK.L |

  


---

|  |  |
| --- | --- |
| **34.** | sp|P68363|TBA1B\_HUMAN    **Mass:** 50804    **Score:** 119    **Matches:** 10(6)  **Sequences:** 8(5)  **emPAI:** 0.46 |
|  | Tubulin alpha-1B chain OS=Homo sapiens OX=9606 GN=TUBA1B PE=1 SV=1 |

|  |  |
| --- | --- |
|  | Check to include this hit in error tolerant search or archive report |
|  |  |

|  |  |  |  |  |  |  |  |  |  |  |  |
| --- | --- | --- | --- | --- | --- | --- | --- | --- | --- | --- | --- |
|  | **Query** | **Observed** | **Mr(expt)** | **Mr(calc)** | **ppm** | **Miss** | **Score** | **Expect** | **Rank** | **Unique** | **Peptide** |
|  | 1003 | **508.2931** | **1014.5717** | **1014.5709** | **0.74** | **0** | **42** | **0.0012** | **1** | **U** | **K.DVNAAIATIK.T** |
|  | 1374 | **543.3130** | **1084.6114** | **1084.6128** | **-1.27** | **0** | **(42)** | **0.00072** | **1** | **U** | **K.EIIDLVLDR.I** |
|  | 1375 | **543.3136** | **1084.6126** | **1084.6128** | **-0.15** | **0** | **47** | **0.00024** | **1** | **U** | **K.EIIDLVLDR.I** |
|  | 2057 | **625.2802** | **1248.5459** | **1248.5453** | **0.43** | **0** | **29** | **0.0071** | **1** | **U** | **K.YMACCLLYR.G** |
|  | 3135 | **792.8790** | **1583.7434** | **1583.7443** | **-0.56** | **0** | **8** | **1.8** | **1** | **U** | **R.SIQFVDWCPTGFK.V** |
|  | 3363 | **851.4568** | **1700.8990** | **1700.8985** | **0.30** | **0** | **14** | **0.47** | **1** | **U** | **R.AVFVDLEPTVIDEVR.T** |
|  | 3397 | **859.9445** | **1717.8745** | **1717.8747** | **-0.13** | **0** | **28** | **0.026** | **1** | **U** | **R.NLDIERPTYTNLNR.L** |
|  | 3398 | **573.6323** | **1717.8751** | **1717.8747** | **0.25** | **0** | **(3)** | **6.9** | **1** | **U** | **R.NLDIERPTYTNLNR.L** |
|  | 3486 | **878.9855** | **1755.9565** | **1755.9559** | **0.33** | **0** | **21** | **0.071** | **1** | **U** | **R.IHFPLATYAPVISAEK.A** |
|  | 3942 | **1004.4482** | **2006.8819** | **2006.8858** | **-1.93** | **0** | **42** | **0.00037** | **1** | **U** | **K.TIGGGDDSFNTFFSETGAGK.H** |

  


---

|  |  |
| --- | --- |
| **35.** | sp|Q14CN4|K2C72\_HUMAN    **Mass:** 56470    **Score:** 115    **Matches:** 11(8)  **Sequences:** 7(5)  **emPAI:** 0.40 |
|  | Keratin, type II cytoskeletal 72 OS=Homo sapiens OX=9606 GN=KRT72 PE=1 SV=2 |

|  |  |
| --- | --- |
|  | Check to include this hit in error tolerant search or archive report |
|  |  |

|  |  |  |  |  |  |  |  |  |  |  |  |
| --- | --- | --- | --- | --- | --- | --- | --- | --- | --- | --- | --- |
|  | **Query** | **Observed** | **Mr(expt)** | **Mr(calc)** | **ppm** | **Miss** | **Score** | **Expect** | **Rank** | **Unique** | **Peptide** |
|  | 248 | 414.2183 | 826.4220 | 826.4225 | -0.57 | 0 | (29) | 0.014 | 1 |  | K.FASFIDK.V |
|  | 249 | 414.2186 | 826.4226 | 826.4225 | 0.11 | 0 | 44 | 0.00045 | 1 |  | K.FASFIDK.V |
|  | 1044 | 513.7310 | 1025.4475 | 1025.4488 | -1.26 | 0 | 31 | 0.0057 | 1 |  | K.DVDAAYMNK.V |
|  | 1358 | 361.5378 | 1081.5917 | 1081.5920 | -0.31 | 1 | (31) | 0.0083 | 1 |  | K.FASFIDKVR.F |
|  | 1359 | 541.8038 | 1081.5931 | 1081.5920 | 1.00 | 1 | 50 | 0.00011 | 1 |  | K.FASFIDKVR.F |
|  | 2236 | **648.3363** | **1294.6580** | **1294.6591** | **-0.80** | **0** | **(1)** | **11** | **9** |  | **K.LALDMEIATYR.K** |
|  | 2300 | 656.3341 | 1310.6535 | 1310.6540 | -0.35 | 0 | 13 | 0.76 | 2 |  | K.LALDMEIATYR.K + Oxidation (M) |
|  | 2433 | 674.8779 | 1347.7413 | 1347.7398 | 1.12 | 1 | 15 | 0.42 | 1 |  | R.TAAENEFVVLKK.D |
|  | 2874 | **738.8894** | **1475.7642** | **1475.7620** | **1.53** | **0** | **42** | **0.0011** | **1** |  | **R.FLEQQNQVLETK.W** |
|  | 2875 | **738.8913** | **1475.7680** | **1475.7620** | **4.10** | **0** | **(24)** | **0.058** | **1** |  | **R.FLEQQNQVLETK.W** |
|  | 3341 | 847.9249 | 1693.8353 | 1693.8345 | 0.48 | 1 | 25 | 0.043 | 1 |  | K.DVDAAYMNKVELQAK.V |

  


---

|  |  |
| --- | --- |
| **36.** | sp|O00571|DDX3X\_HUMAN    **Mass:** 73597    **Score:** 112    **Matches:** 7(6)  **Sequences:** 7(6)  **emPAI:** 0.36 |
|  | ATP-dependent RNA helicase DDX3X OS=Homo sapiens OX=9606 GN=DDX3X PE=1 SV=3 |

|  |  |
| --- | --- |
|  | Check to include this hit in error tolerant search or archive report |
|  |  |

|  |  |  |  |  |  |  |  |  |  |  |  |
| --- | --- | --- | --- | --- | --- | --- | --- | --- | --- | --- | --- |
|  | **Query** | **Observed** | **Mr(expt)** | **Mr(calc)** | **ppm** | **Miss** | **Score** | **Expect** | **Rank** | **Unique** | **Peptide** |
|  | 159 | **396.2598** | **790.5050** | **790.5065** | **-1.89** | **0** | **31** | **0.0014** | **1** | **U** | **K.HAIPIIK.E** |
|  | 403 | **426.7176** | **851.4207** | **851.4211** | **-0.45** | **0** | **24** | **0.053** | **1** | **U** | **K.IGLDFCK.Y** |
|  | 1734 | **584.8561** | **1167.6977** | **1167.6975** | **0.16** | **0** | **30** | **0.0034** | **1** | **U** | **K.SPILVATAVAAR.G** |
|  | 1783 | **590.8165** | **1179.6185** | **1179.6183** | **0.20** | **0** | **27** | **0.031** | **1** | **U** | **R.GCHLLVATPGR.L** |
|  | 2017 | **619.7784** | **1237.5422** | **1237.5431** | **-0.73** | **0** | **44** | **0.00027** | **1** |  | **R.DLMACAQTGSGK.T** |
|  | 2260 | **434.2182** | **1299.6327** | **1299.6320** | **0.55** | **1** | **18** | **0.21** | **1** | **U** | **R.DREEALHQFR.S** |
|  | 3788 | **630.0005** | **1886.9796** | **1886.9785** | **0.60** | **0** | **58** | **2e-005** | **1** | **U** | **R.VRPCVVYGGADIGQQIR.D** |

  

|  |  |
| --- | --- |
|  | |
|  | **Proteins matching the same set of peptides:** |

|  |  |
| --- | --- |
|  | sp|O15523|DDX3Y\_HUMAN    **Mass:** 73564    **Score:** 112    **Matches:** 7(6)  **Sequences:** 7(6) |
|  | ATP-dependent RNA helicase DDX3Y OS=Homo sapiens OX=9606 GN=DDX3Y PE=1 SV=2 |

---

|  |  |
| --- | --- |
| **37.** | sp|Q7RTS7|K2C74\_HUMAN    **Mass:** 58229    **Score:** 112    **Matches:** 11(6)  **Sequences:** 6(3)  **emPAI:** 0.39 |
|  | Keratin, type II cytoskeletal 74 OS=Homo sapiens OX=9606 GN=KRT74 PE=1 SV=2 |

|  |  |
| --- | --- |
|  | Check to include this hit in error tolerant search or archive report |
|  |  |

|  |  |  |  |  |  |  |  |  |  |  |  |
| --- | --- | --- | --- | --- | --- | --- | --- | --- | --- | --- | --- |
|  | **Query** | **Observed** | **Mr(expt)** | **Mr(calc)** | **ppm** | **Miss** | **Score** | **Expect** | **Rank** | **Unique** | **Peptide** |
|  | 248 | 414.2183 | 826.4220 | 826.4225 | -0.57 | 0 | (29) | 0.014 | 1 |  | K.FASFIDK.V |
|  | 249 | 414.2186 | 826.4226 | 826.4225 | 0.11 | 0 | 44 | 0.00045 | 1 |  | K.FASFIDK.V |
|  | 965 | 503.2367 | 1004.4589 | 1004.4597 | -0.78 | 0 | 22 | 0.08 | 1 |  | K.LLEGEECR.M |
|  | 1358 | 361.5378 | 1081.5917 | 1081.5920 | -0.31 | 1 | (31) | 0.0083 | 1 |  | K.FASFIDKVR.F |
|  | 1359 | 541.8038 | 1081.5931 | 1081.5920 | 1.00 | 1 | 50 | 0.00011 | 1 |  | K.FASFIDKVR.F |
|  | 1604 | 567.2844 | 1132.5542 | 1132.5546 | -0.40 | 1 | 20 | 0.17 | 1 |  | R.KLLEGEECR.M |
|  | 1605 | 378.5254 | 1132.5543 | 1132.5546 | -0.33 | 1 | (18) | 0.29 | 1 |  | R.KLLEGEECR.M |
|  | 2236 | 648.3363 | 1294.6580 | 1294.6591 | -0.80 | 0 | (1) | 11 | 9 |  | K.LALDMEIATYR.K |
|  | 2300 | 656.3341 | 1310.6535 | 1310.6540 | -0.35 | 0 | 13 | 0.76 | 2 |  | K.LALDMEIATYR.K + Oxidation (M) |
|  | 2874 | 738.8894 | 1475.7642 | 1475.7620 | 1.53 | 0 | 42 | 0.0011 | 1 |  | R.FLEQQNQVLETK.W |
|  | 2875 | 738.8913 | 1475.7680 | 1475.7620 | 4.10 | 0 | (24) | 0.058 | 1 |  | R.FLEQQNQVLETK.W |

  


---

|  |  |
| --- | --- |
| **38.** | sp|Q86Y46|K2C73\_HUMAN    **Mass:** 59457    **Score:** 112    **Matches:** 11(6)  **Sequences:** 6(3)  **emPAI:** 0.38 |
|  | Keratin, type II cytoskeletal 73 OS=Homo sapiens OX=9606 GN=KRT73 PE=1 SV=1 |

|  |  |
| --- | --- |
|  | Check to include this hit in error tolerant search or archive report |
|  |  |

|  |  |  |  |  |  |  |  |  |  |  |  |
| --- | --- | --- | --- | --- | --- | --- | --- | --- | --- | --- | --- |
|  | **Query** | **Observed** | **Mr(expt)** | **Mr(calc)** | **ppm** | **Miss** | **Score** | **Expect** | **Rank** | **Unique** | **Peptide** |
|  | 248 | 414.2183 | 826.4220 | 826.4225 | -0.57 | 0 | (29) | 0.014 | 1 |  | K.FASFIDK.V |
|  | 249 | 414.2186 | 826.4226 | 826.4225 | 0.11 | 0 | 44 | 0.00045 | 1 |  | K.FASFIDK.V |
|  | 965 | 503.2367 | 1004.4589 | 1004.4597 | -0.78 | 0 | 22 | 0.08 | 1 |  | K.LLEGEECR.M |
|  | 1323 | **538.7539** | **1075.4931** | **1075.5121** | **-17.58** | **1** | **(0)** | **8.9** | **3** | **U** | **-.MSRQFTYK.S + Oxidation (M)** |
|  | 1326 | **538.7740** | **1075.5334** | **1075.5121** | **19.9** | **1** | **1** | **14** | **3** | **U** | **-.MSRQFTYK.S + Oxidation (M)** |
|  | 1358 | 361.5378 | 1081.5917 | 1081.5920 | -0.31 | 1 | (31) | 0.0083 | 1 |  | K.FASFIDKVR.F |
|  | 1359 | 541.8038 | 1081.5931 | 1081.5920 | 1.00 | 1 | 50 | 0.00011 | 1 |  | K.FASFIDKVR.F |
|  | 1604 | 567.2844 | 1132.5542 | 1132.5546 | -0.40 | 1 | 20 | 0.17 | 1 |  | R.KLLEGEECR.M |
|  | 1605 | 378.5254 | 1132.5543 | 1132.5546 | -0.33 | 1 | (18) | 0.29 | 1 |  | R.KLLEGEECR.M |
|  | 2874 | 738.8894 | 1475.7642 | 1475.7620 | 1.53 | 0 | 42 | 0.0011 | 1 |  | R.FLEQQNQVLETK.W |
|  | 2875 | 738.8913 | 1475.7680 | 1475.7620 | 4.10 | 0 | (24) | 0.058 | 1 |  | R.FLEQQNQVLETK.W |

  


---

|  |  |
| --- | --- |
| **39.** | sp|Q86YZ3|HORN\_HUMAN    **Mass:** 283140   **Score:** 112    **Matches:** 6(2)  **Sequences:** 5(2)  **emPAI:** 0.02 |
|  | Hornerin OS=Homo sapiens OX=9606 GN=HRNR PE=1 SV=2 |

|  |  |
| --- | --- |
|  | Check to include this hit in error tolerant search or archive report |
|  |  |

|  |  |  |  |  |  |  |  |  |  |  |  |
| --- | --- | --- | --- | --- | --- | --- | --- | --- | --- | --- | --- |
|  | **Query** | **Observed** | **Mr(expt)** | **Mr(calc)** | **ppm** | **Miss** | **Score** | **Expect** | **Rank** | **Unique** | **Peptide** |
|  | 1558 | **563.2786** | **1124.5426** | **1124.5210** | **19.2** | **1** | **7** | **2.9** | **1** | **U** | **R.SSSRGPYESR.S** |
|  | 2609 | **698.3240** | **1394.6335** | **1394.6175** | **11.5** | **0** | **5** | **2.5** | **5** | **U** | **R.YGQQGSGSGQSPSR.G** |
|  | 2610 | **465.8855** | **1394.6347** | **1394.6175** | **12.3** | **0** | **(1)** | **7** | **4** | **U** | **R.YGQQGSGSGQSPSR.G** |
|  | 3908 | **978.9521** | **1955.8896** | **1955.8834** | **3.18** | **1** | **2** | **3.9** | **1** | **U** | **R.GPYESGSGHSSGLGHRESR.S** |
|  | 4304 | **783.6636** | **2347.9689** | **2347.9690** | **-0.05** | **0** | **68** | **2.4e-007** | **1** | **U** | **R.HGSGSGQSSSYSPYGSGSGWSSSR.G** |
|  | 4363 | **620.0103** | **2476.0119** | **2476.0137** | **-0.72** | **0** | **57** | **1.9e-006** | **1** | **U** | **R.HGSGSGHSSSYGQHGSGSGWSSSSGR.H** |

  


---

|  |  |
| --- | --- |
| **40.** | sp|P81605|DCD\_HUMAN    **Mass:** 11391    **Score:** 112    **Matches:** 5(4)  **Sequences:** 3(2)  **emPAI:** 1.20 |
|  | Dermcidin OS=Homo sapiens OX=9606 GN=DCD PE=1 SV=2 |

|  |  |
| --- | --- |
|  | Check to include this hit in error tolerant search or archive report |
|  |  |

|  |  |  |  |  |  |  |  |  |  |  |  |
| --- | --- | --- | --- | --- | --- | --- | --- | --- | --- | --- | --- |
|  | **Query** | **Observed** | **Mr(expt)** | **Mr(calc)** | **ppm** | **Miss** | **Score** | **Expect** | **Rank** | **Unique** | **Peptide** |
|  | 1571 | **564.7676** | **1127.5207** | **1127.5207** | **0.03** | **0** | **9** | **0.94** | **1** | **U** | **K.ENAGEDPGLAR.Q** |
|  | 1701 | **581.2844** | **1160.5542** | **1160.5561** | **-1.66** | **0** | **(50)** | **0.00014** | **1** | **U** | **K.DAVEDLESVGK.G** |
|  | 1702 | **581.2850** | **1160.5554** | **1160.5561** | **-0.61** | **0** | **59** | **2e-005** | **1** | **U** | **K.DAVEDLESVGK.G** |
|  | 2838 | **487.2596** | **1458.7570** | **1458.7566** | **0.29** | **1** | **(31)** | **0.011** | **1** | **U** | **K.LGKDAVEDLESVGK.G** |
|  | 2839 | **730.3866** | **1458.7586** | **1458.7566** | **1.42** | **1** | **44** | **0.00071** | **1** | **U** | **K.LGKDAVEDLESVGK.G** |

  


---

|  |  |
| --- | --- |
| **41.** | sp|Q7Z3Z0|K1C25\_HUMAN    **Mass:** 49858    **Score:** 111    **Matches:** 7(4)  **Sequences:** 5(2)  **emPAI:** 0.14 |
|  | Keratin, type I cytoskeletal 25 OS=Homo sapiens OX=9606 GN=KRT25 PE=1 SV=1 |

|  |  |
| --- | --- |
|  | Check to include this hit in error tolerant search or archive report |
|  |  |

|  |  |  |  |  |  |  |  |  |  |  |  |
| --- | --- | --- | --- | --- | --- | --- | --- | --- | --- | --- | --- |
|  | **Query** | **Observed** | **Mr(expt)** | **Mr(calc)** | **ppm** | **Miss** | **Score** | **Expect** | **Rank** | **Unique** | **Peptide** |
|  | 1392 | 545.7685 | 1089.5224 | 1089.5237 | -1.15 | 0 | (51) | 0.00012 | 1 |  | K.VTMQNLNDR.L |
|  | 1393 | 545.7700 | 1089.5254 | 1089.5237 | 1.55 | 0 | 51 | 0.00012 | 1 |  | K.VTMQNLNDR.L |
|  | 1482 | 555.2471 | 1108.4796 | 1108.4825 | -2.64 | 0 | 43 | 0.00033 | 1 |  | R.DAEAWFNEK.S |
|  | 1483 | 555.2486 | 1108.4826 | 1108.4825 | 0.12 | 0 | (26) | 0.017 | 1 |  | R.DAEAWFNEK.S |
|  | 1546 | **562.2948** | **1122.5750** | **1122.5815** | **-5.76** | **1** | **11** | **1.1** | **2** | **U** | **-.MSLRLSSASR.R + Oxidation (M)** |
|  | 1898 | **601.8214** | **1201.6283** | **1201.6302** | **-1.65** | **1** | **2** | **11** | **8** | **U** | **K.VLEEVDQRSK.I** |
|  | 3407 | **862.3850** | **1722.7553** | **1722.7533** | **1.21** | **1** | **1** | **3.8** | **5** | **U** | **K.FGPGSCRGLDHDYSR.Y** |

  


---

|  |  |
| --- | --- |
| **42.** | sp|Q92804|RBP56\_HUMAN    **Mass:** 62021    **Score:** 109    **Matches:** 7(4)  **Sequences:** 4(3)  **emPAI:** 0.17 |
|  | TATA-binding protein-associated factor 2N OS=Homo sapiens OX=9606 GN=TAF15 PE=1 SV=1 |

|  |  |
| --- | --- |
|  | Check to include this hit in error tolerant search or archive report |
|  |  |

|  |  |  |  |  |  |  |  |  |  |  |  |
| --- | --- | --- | --- | --- | --- | --- | --- | --- | --- | --- | --- |
|  | **Query** | **Observed** | **Mr(expt)** | **Mr(calc)** | **ppm** | **Miss** | **Score** | **Expect** | **Rank** | **Unique** | **Peptide** |
|  | 1032 | **511.7508** | **1021.4870** | **1021.4869** | **0.13** | **0** | **54** | **5.3e-005** | **1** | **U** | **K.AAIDWFDGK.E** |
|  | 2694 | **710.8331** | **1419.6516** | **1419.6518** | **-0.15** | **0** | **45** | **0.00033** | **1** | **U** | **K.GEATVSFDDPPSAK.A** |
|  | 2695 | **710.8345** | **1419.6545** | **1419.6518** | **1.91** | **0** | **(23)** | **0.049** | **1** | **U** | **K.GEATVSFDDPPSAK.A** |
|  | 3940 | **502.5183** | **2006.0440** | **2006.0506** | **-3.30** | **1** | **52** | **6.8e-005** | **1** | **U** | **K.TGKPMINLYTDKDTGKPK.G** |
|  | 3976 | **675.0223** | **2022.0450** | **2022.0456** | **-0.27** | **1** | **(3)** | **4.5** | **1** | **U** | **K.TGKPMINLYTDKDTGKPK.G + Oxidation (M)** |
|  | 3977 | **506.5186** | **2022.0453** | **2022.0456** | **-0.15** | **1** | **(7)** | **2** | **1** | **U** | **K.TGKPMINLYTDKDTGKPK.G + Oxidation (M)** |
|  | 4477 | **992.4225** | **2974.2456** | **2974.2496** | **-1.33** | **0** | **2** | **1.1** | **2** | **U** | **K.QSSYSQQPYNNQGQQQNMESSGSQGGR.A** |

  


---

|  |  |
| --- | --- |
| **43.** | sp|O00622|CCN1\_HUMAN    **Mass:** 44165    **Score:** 108    **Matches:** 4(3)  **Sequences:** 4(3)  **emPAI:** 0.24 |
|  | CCN family member 1 OS=Homo sapiens OX=9606 GN=CCN1 PE=1 SV=1 |

|  |  |
| --- | --- |
|  | Check to include this hit in error tolerant search or archive report |
|  |  |

|  |  |  |  |  |  |  |  |  |  |  |  |
| --- | --- | --- | --- | --- | --- | --- | --- | --- | --- | --- | --- |
|  | **Query** | **Observed** | **Mr(expt)** | **Mr(calc)** | **ppm** | **Miss** | **Score** | **Expect** | **Rank** | **Unique** | **Peptide** |
|  | 1760 | **587.3358** | **1172.6571** | **1172.6553** | **1.50** | **0** | **27** | **0.029** | **1** | **U** | **R.ILYNPLQGQK.C** |
|  | 1885 | **601.3303** | **1200.6461** | **1200.6437** | **1.96** | **1** | **6** | **3.7** | **2** | **U** | **K.RLPVFGMEPR.I** |
|  | 2826 | **727.8508** | **1453.6871** | **1453.6871** | **-0.01** | **0** | **55** | **3.8e-005** | **1** | **U** | **K.GLECNFGASSTALK.G** |
|  | 2852 | **733.3614** | **1464.7082** | **1464.7096** | **-0.95** | **0** | **71** | **1e-006** | **1** | **U** | **K.ELGFDASEVELTR.N** |

  


---

|  |  |
| --- | --- |
| **44.** | sp|Q2M2I5|K1C24\_HUMAN    **Mass:** 55567    **Score:** 102    **Matches:** 4(3)  **Sequences:** 3(2)  **emPAI:** 0.12 |
|  | Keratin, type I cytoskeletal 24 OS=Homo sapiens OX=9606 GN=KRT24 PE=1 SV=1 |

|  |  |
| --- | --- |
|  | Check to include this hit in error tolerant search or archive report |
|  |  |

|  |  |  |  |  |  |  |  |  |  |  |  |
| --- | --- | --- | --- | --- | --- | --- | --- | --- | --- | --- | --- |
|  | **Query** | **Observed** | **Mr(expt)** | **Mr(calc)** | **ppm** | **Miss** | **Score** | **Expect** | **Rank** | **Unique** | **Peptide** |
|  | 189 | 404.2032 | 806.3918 | 806.3923 | -0.61 | 0 | 43 | 0.0011 | 1 |  | R.LAADDFR.L |
|  | 190 | 404.2034 | 806.3923 | 806.3923 | 0.06 | 0 | (31) | 0.017 | 1 |  | R.LAADDFR.L |
|  | 1367 | **362.1681** | **1083.4824** | **1083.4727** | **8.99** | **1** | **7** | **1.8** | **6** | **U** | **M.SCSSRASSSR.A** |
|  | 1883 | 601.3116 | 1200.6087 | 1200.6098 | -0.92 | 0 | 73 | 6.7e-007 | 1 |  | R.QSVEADINGLR.K |

  


---

|  |  |
| --- | --- |
| **45.** | sp|Q99456|K1C12\_HUMAN    **Mass:** 53592    **Score:** 101    **Matches:** 6(4)  **Sequences:** 4(2)  **emPAI:** 0.20 |
|  | Keratin, type I cytoskeletal 12 OS=Homo sapiens OX=9606 GN=KRT12 PE=1 SV=1 |

|  |  |
| --- | --- |
|  | Check to include this hit in error tolerant search or archive report |
|  |  |

|  |  |  |  |  |  |  |  |  |  |  |  |
| --- | --- | --- | --- | --- | --- | --- | --- | --- | --- | --- | --- |
|  | **Query** | **Observed** | **Mr(expt)** | **Mr(calc)** | **ppm** | **Miss** | **Score** | **Expect** | **Rank** | **Unique** | **Peptide** |
|  | 192 | 405.2236 | 808.4326 | 808.4330 | -0.56 | 0 | (31) | 0.012 | 1 |  | R.LASYLDK.V |
|  | 193 | 405.2237 | 808.4329 | 808.4330 | -0.19 | 0 | 43 | 0.00066 | 1 |  | R.LASYLDK.V |
|  | 1267 | 355.5415 | 1063.6028 | 1063.6026 | 0.18 | 1 | 49 | 9.1e-005 | 1 |  | R.LASYLDKVR.A |
|  | 1268 | 532.8089 | 1063.6032 | 1063.6026 | 0.64 | 1 | (45) | 0.00026 | 1 |  | R.LASYLDKVR.A |
|  | 2779 | **719.8444** | **1437.6743** | **1437.6709** | **2.37** | **1** | **10** | **1.2** | **1** | **U** | **R.ADAERQNVDHQR.L** |
|  | 2857 | **734.3505** | **1466.6865** | **1466.6857** | **0.52** | **0** | **0** | **9.4** | **7** | **U** | **-.MDLSNNTMSLSVR.T** |

  


---

|  |  |
| --- | --- |
| **46.** | sp|P31943|HNRH1\_HUMAN    **Mass:** 49484    **Score:** 100    **Matches:** 6(3)  **Sequences:** 5(2)  **emPAI:** 0.21 |
|  | Heterogeneous nuclear ribonucleoprotein H OS=Homo sapiens OX=9606 GN=HNRNPH1 PE=1 SV=4 |

|  |  |
| --- | --- |
|  | Check to include this hit in error tolerant search or archive report |
|  |  |

|  |  |  |  |  |  |  |  |  |  |  |  |
| --- | --- | --- | --- | --- | --- | --- | --- | --- | --- | --- | --- |
|  | **Query** | **Observed** | **Mr(expt)** | **Mr(calc)** | **ppm** | **Miss** | **Score** | **Expect** | **Rank** | **Unique** | **Peptide** |
|  | 139 | **392.7157** | **783.4168** | **783.4167** | **0.21** | **0** | **9** | **0.95** | **1** |  | **R.YVEVFK.S** |
|  | 200 | **406.7307** | **811.4469** | **811.4480** | **-1.28** | **0** | **14** | **0.32** | **1** |  | **R.YIEIFK.S** |
|  | 1405 | 364.8644 | 1091.5714 | 1091.5724 | -0.87 | 0 | 38 | 0.0027 | 1 |  | R.VHIEIGPDGR.V |
|  | 1406 | 546.7938 | 1091.5730 | 1091.5724 | 0.56 | 0 | (33) | 0.0082 | 1 |  | R.VHIEIGPDGR.V |
|  | 2376 | **667.8235** | **1333.6324** | **1333.6336** | **-0.89** | **0** | **9** | **2.1** | **1** | **U** | **K.SNNVEMDWVLK.H** |
|  | 2939 | **752.8460** | **1503.6775** | **1503.6776** | **-0.11** | **0** | **70** | **8e-007** | **1** | **U** | **R.GLPWSCSADEVQR.F** |

  


---

|  |  |
| --- | --- |
| **47.** | sp|P41219|PERI\_HUMAN    **Mass:** 53732    **Score:** 99     **Matches:** 3(2)  **Sequences:** 3(2)  **emPAI:** 0.13 |
|  | Peripherin OS=Homo sapiens OX=9606 GN=PRPH PE=1 SV=2 |

|  |  |
| --- | --- |
|  | Check to include this hit in error tolerant search or archive report |
|  |  |

|  |  |  |  |  |  |  |  |  |  |  |  |
| --- | --- | --- | --- | --- | --- | --- | --- | --- | --- | --- | --- |
|  | **Query** | **Observed** | **Mr(expt)** | **Mr(calc)** | **ppm** | **Miss** | **Score** | **Expect** | **Rank** | **Unique** | **Peptide** |
|  | 49 | **364.7243** | **727.4341** | **727.4341** | **0.08** | **0** | **15** | **0.39** | **1** | **U** | **R.AGAGALLR.L** |
|  | 2293 | 655.3064 | 1308.5982 | 1308.5986 | -0.26 | 0 | 59 | 1.1e-005 | 1 |  | K.NLQEAEEWYK.S |
|  | 2300 | 656.3341 | 1310.6535 | 1310.6540 | -0.35 | 0 | 60 | 1.4e-005 | 1 |  | K.MALDIEIATYR.K + Oxidation (M) |

  


---

|  |  |
| --- | --- |
| **48.** | sp|Q562R1|ACTBL\_HUMAN    **Mass:** 42318    **Score:** 97     **Matches:** 12(7)  **Sequences:** 6(4)  **emPAI:** 0.57 |
|  | Beta-actin-like protein 2 OS=Homo sapiens OX=9606 GN=ACTBL2 PE=1 SV=2 |

|  |  |
| --- | --- |
|  | Check to include this hit in error tolerant search or archive report |
|  |  |

|  |  |  |  |  |  |  |  |  |  |  |  |
| --- | --- | --- | --- | --- | --- | --- | --- | --- | --- | --- | --- |
|  | **Query** | **Observed** | **Mr(expt)** | **Mr(calc)** | **ppm** | **Miss** | **Score** | **Expect** | **Rank** | **Unique** | **Peptide** |
|  | 162 | 398.2395 | 794.4644 | 794.4650 | -0.71 | 0 | 28 | 0.013 | 1 |  | K.IIAPPER.K |
|  | 939 | 499.7468 | 997.4790 | 997.4790 | 0.03 | 0 | (18) | 0.18 | 1 |  | R.DLTDYLMK.I |
|  | 940 | 499.7468 | 997.4790 | 997.4790 | 0.03 | 0 | (28) | 0.02 | 1 |  | R.DLTDYLMK.I |
|  | 995 | 507.7447 | 1013.4748 | 1013.4739 | 0.88 | 0 | 30 | 0.0079 | 1 |  | R.DLTDYLMK.I + Oxidation (M) |
|  | 1751 | 586.2892 | 1170.5638 | 1170.5638 | 0.01 | 0 | (10) | 1.2 | 1 |  | R.HQGVMVGMGQK.D |
|  | 1752 | 586.2892 | 1170.5638 | 1170.5638 | 0.01 | 0 | 66 | 3e-006 | 1 |  | R.HQGVMVGMGQK.D |
|  | 1989 | **615.8405** | **1229.6665** | **1229.6703** | **-3.09** | **0** | **2** | **9.1** | **2** | **U** | **R.AVFPSMIGRPR.H** |
|  | 3901 | 652.0258 | 1953.0555 | 1953.0571 | -0.84 | 0 | (22) | 0.048 | 2 | U | R.VAPDEHPILLTEAPLNPK.I |
|  | 3902 | 977.5354 | 1953.0562 | 1953.0571 | -0.44 | 0 | (20) | 0.068 | 2 | U | R.VAPDEHPILLTEAPLNPK.I |
|  | 3903 | 652.0264 | 1953.0573 | 1953.0571 | 0.09 | 0 | (24) | 0.027 | 2 | U | R.VAPDEHPILLTEAPLNPK.I |
|  | 3904 | 977.5361 | 1953.0576 | 1953.0571 | 0.25 | 0 | 26 | 0.016 | 2 | U | R.VAPDEHPILLTEAPLNPK.I |
|  | 4033 | **691.6460** | **2071.9162** | **2071.8860** | **14.5** | **0** | **2** | **2.6** | **5** | **U** | **-.MTDNELSALVVDNGSGMCK.A + 2 Oxidation (M)** |

  


---

|  |  |
| --- | --- |
| **49.** | sp|P17661|DESM\_HUMAN    **Mass:** 53560    **Score:** 96     **Matches:** 6(2)  **Sequences:** 4(2)  **emPAI:** 0.13 |
|  | Desmin OS=Homo sapiens OX=9606 GN=DES PE=1 SV=3 |

|  |  |
| --- | --- |
|  | Check to include this hit in error tolerant search or archive report |
|  |  |

|  |  |  |  |  |  |  |  |  |  |  |  |
| --- | --- | --- | --- | --- | --- | --- | --- | --- | --- | --- | --- |
|  | **Query** | **Observed** | **Mr(expt)** | **Mr(calc)** | **ppm** | **Miss** | **Score** | **Expect** | **Rank** | **Unique** | **Peptide** |
|  | 948 | 500.7871 | 999.5595 | 999.5600 | -0.48 | 0 | 7 | 3.2 | 5 | U | K.LQEEIQLK.E |
|  | 1518 | 558.2894 | 1114.5643 | 1114.5618 | 2.22 | 0 | 43 | 0.00071 | 1 |  | K.VELQELNDR.F |
|  | 2425 | **673.8528** | **1345.6910** | **1345.7062** | **-11.31** | **1** | **(2)** | **11** | **8** | **U** | **R.TSGGAGGLGSLRASR.L** |
|  | 2426 | **673.8531** | **1345.6916** | **1345.7062** | **-10.85** | **1** | **3** | **10** | **4** | **U** | **R.TSGGAGGLGSLRASR.L** |
|  | 3147 | 529.9376 | 1586.7909 | 1586.7900 | 0.55 | 1 | (22) | 0.1 | 1 |  | R.TNEKVELQELNDR.F |
|  | 3148 | 794.4036 | 1586.7926 | 1586.7900 | 1.63 | 1 | 77 | 3.4e-007 | 1 |  | R.TNEKVELQELNDR.F |

  


---

|  |  |
| --- | --- |
| **50.** | sp|P62805|H4\_HUMAN    **Mass:** 11360    **Score:** 96     **Matches:** 4(3)  **Sequences:** 4(3)  **emPAI:** 1.22 |
|  | Histone H4 OS=Homo sapiens OX=9606 GN=HIST1H4A PE=1 SV=2 |

|  |  |
| --- | --- |
|  | Check to include this hit in error tolerant search or archive report |
|  |  |

|  |  |  |  |  |  |  |  |  |  |  |  |
| --- | --- | --- | --- | --- | --- | --- | --- | --- | --- | --- | --- |
|  | **Query** | **Observed** | **Mr(expt)** | **Mr(calc)** | **ppm** | **Miss** | **Score** | **Expect** | **Rank** | **Unique** | **Peptide** |
|  | 900 | **495.2928** | **988.5710** | **988.5706** | **0.42** | **0** | **26** | **0.021** | **1** | **U** | **K.VFLENVIR.D** |
|  | 1782 | **590.8144** | **1179.6143** | **1179.6135** | **0.70** | **0** | **55** | **4.7e-005** | **1** | **U** | **R.ISGLIYEETR.G** |
|  | 2299 | **655.8552** | **1309.6959** | **1309.6952** | **0.54** | **0** | **1** | **10** | **6** | **U** | **K.TVTAMDVVYALK.R** |
|  | 2358 | **663.3806** | **1324.7467** | **1324.7463** | **0.31** | **0** | **53** | **4e-005** | **1** | **U** | **R.DNIQGITKPAIR.R** |

  


---

|  |  |
| --- | --- |
| **51.** | sp|P04406|G3P\_HUMAN    **Mass:** 36201    **Score:** 94     **Matches:** 4(1)  **Sequences:** 4(1)  **emPAI:** 0.09 |
|  | Glyceraldehyde-3-phosphate dehydrogenase OS=Homo sapiens OX=9606 GN=GAPDH PE=1 SV=3 |

|  |  |
| --- | --- |
|  | Check to include this hit in error tolerant search or archive report |
|  |  |

|  |  |  |  |  |  |  |  |  |  |  |  |
| --- | --- | --- | --- | --- | --- | --- | --- | --- | --- | --- | --- |
|  | **Query** | **Observed** | **Mr(expt)** | **Mr(calc)** | **ppm** | **Miss** | **Score** | **Expect** | **Rank** | **Unique** | **Peptide** |
|  | 161 | **398.2129** | **794.4113** | **794.4109** | **0.58** | **0** | **19** | **0.14** | **1** | **U** | **K.LTGMAFR.V** |
|  | 1595 | **377.8874** | **1130.6403** | **1130.6309** | **8.36** | **1** | **9** | **1.3** | **3** | **U** | **K.VGVNGFGRIGR.L** |
|  | 2667 | **706.4020** | **1410.7895** | **1410.7831** | **4.59** | **0** | **16** | **0.21** | **1** | **U** | **R.GALQNIIPASTGAAK.A** |
|  | 3001 | **765.9009** | **1529.7873** | **1529.7872** | **0.08** | **0** | **94** | **5.6e-009** | **1** | **U** | **R.VPTANVSVVDLTCR.L** |

  


---

|  |  |
| --- | --- |
| **52.** | sp|Q02413|DSG1\_HUMAN    **Mass:** 114702   **Score:** 89     **Matches:** 7(4)  **Sequences:** 5(4)  **emPAI:** 0.12 |
|  | Desmoglein-1 OS=Homo sapiens OX=9606 GN=DSG1 PE=1 SV=2 |

|  |  |
| --- | --- |
|  | Check to include this hit in error tolerant search or archive report |
|  |  |

|  |  |  |  |  |  |  |  |  |  |  |  |
| --- | --- | --- | --- | --- | --- | --- | --- | --- | --- | --- | --- |
|  | **Query** | **Observed** | **Mr(expt)** | **Mr(calc)** | **ppm** | **Miss** | **Score** | **Expect** | **Rank** | **Unique** | **Peptide** |
|  | 2242 | **649.3293** | **1296.6440** | **1296.6462** | **-1.71** | **0** | **48** | **0.00021** | **1** | **U** | **R.EQYGQYALAVR.G** |
|  | 3058 | **775.3693** | **1548.7241** | **1548.7242** | **-0.09** | **0** | **43** | **0.00053** | **1** | **U** | **R.QEPSDSPMFIINR.N + Oxidation (M)** |
|  | 3059 | **775.3715** | **1548.7284** | **1548.7242** | **2.67** | **0** | **(1)** | **9.3** | **3** | **U** | **R.QEPSDSPMFIINR.N + Oxidation (M)** |
|  | 3236 | **818.4198** | **1634.8250** | **1634.8264** | **-0.82** | **0** | **16** | **0.41** | **1** | **U** | **K.YQGTILSIDDNLQR.T** |
|  | 3286 | **554.9265** | **1661.7575** | **1661.7580** | **-0.29** | **0** | **27** | **0.015** | **1** | **U** | **K.IHSDCAANQQVTYR.I** |
|  | 3731 | **929.4760** | **1856.9373** | **1856.9414** | **-2.19** | **0** | **(14)** | **0.46** | **2** | **U** | **R.ALNSMGQDLERPLELR.V + Oxidation (M)** |
|  | 3732 | **619.9879** | **1856.9419** | **1856.9414** | **0.28** | **0** | **25** | **0.043** | **1** | **U** | **R.ALNSMGQDLERPLELR.V + Oxidation (M)** |

  


---

|  |  |
| --- | --- |
| **53.** | sp|Q9NSB2|KRT84\_HUMAN    **Mass:** 65942    **Score:** 86     **Matches:** 9(4)  **Sequences:** 6(2)  **emPAI:** 0.16 |
|  | Keratin, type II cuticular Hb4 OS=Homo sapiens OX=9606 GN=KRT84 PE=2 SV=2 |

|  |  |
| --- | --- |
|  | Check to include this hit in error tolerant search or archive report |
|  |  |

|  |  |  |  |  |  |  |  |  |  |  |  |
| --- | --- | --- | --- | --- | --- | --- | --- | --- | --- | --- | --- |
|  | **Query** | **Observed** | **Mr(expt)** | **Mr(calc)** | **ppm** | **Miss** | **Score** | **Expect** | **Rank** | **Unique** | **Peptide** |
|  | 248 | 414.2183 | 826.4220 | 826.4225 | -0.57 | 0 | (29) | 0.014 | 1 |  | K.FASFIDK.V |
|  | 249 | 414.2186 | 826.4226 | 826.4225 | 0.11 | 0 | 44 | 0.00045 | 1 |  | K.FASFIDK.V |
|  | 815 | 483.2376 | 964.4607 | 964.4614 | -0.74 | 0 | 8 | 2.3 | 3 | U | K.AQYEEVAR.R |
|  | 1044 | 513.7310 | 1025.4475 | 1025.4488 | -1.27 | 0 | 16 | 0.16 | 2 |  | K.DVDAAFMNK.S + Oxidation (M) |
|  | 1358 | 361.5378 | 1081.5917 | 1081.5920 | -0.31 | 1 | (31) | 0.0083 | 1 |  | K.FASFIDKVR.F |
|  | 1359 | 541.8038 | 1081.5931 | 1081.5920 | 1.00 | 1 | 50 | 0.00011 | 1 |  | K.FASFIDKVR.F |
|  | 1381 | **544.7878** | **1087.5611** | **1087.5621** | **-0.94** | **1** | **6** | **6.1** | **3** | **U** | **R.RLLEGEESR.L** |
|  | 1382 | **544.7883** | **1087.5621** | **1087.5621** | **-0.04** | **1** | **(2)** | **12** | **2** | **U** | **R.RLLEGEESR.L** |
|  | 4306 | 784.0349 | 2349.0827 | 2349.0590 | 10.1 | 1 | 1 | 5.6 | 2 | U | K.YEEMQVTAGQHCDNLRNIR.N + Oxidation (M) |

  


---

|  |  |
| --- | --- |
| **54.** | sp|Q96PK6|RBM14\_HUMAN    **Mass:** 69620    **Score:** 86     **Matches:** 5(2)  **Sequences:** 4(2)  **emPAI:** 0.10 |
|  | RNA-binding protein 14 OS=Homo sapiens OX=9606 GN=RBM14 PE=1 SV=2 |

|  |  |
| --- | --- |
|  | Check to include this hit in error tolerant search or archive report |
|  |  |

|  |  |  |  |  |  |  |  |  |  |  |  |
| --- | --- | --- | --- | --- | --- | --- | --- | --- | --- | --- | --- |
|  | **Query** | **Observed** | **Mr(expt)** | **Mr(calc)** | **ppm** | **Miss** | **Score** | **Expect** | **Rank** | **Unique** | **Peptide** |
|  | 2051 | **623.3331** | **1244.6517** | **1244.6513** | **0.31** | **0** | **41** | **0.0015** | **1** | **U** | **R.AQPSVSLGAPYR.G** |
|  | 2259 | **434.2181** | **1299.6326** | **1299.6320** | **0.48** | **1** | **15** | **0.41** | **1** | **U** | **R.RLPDAHSDYAR.Y** |
|  | 2261 | **650.8237** | **1299.6328** | **1299.6320** | **0.63** | **1** | **(5)** | **4** | **6** | **U** | **R.RLPDAHSDYAR.Y** |
|  | 3184 | **804.9221** | **1607.8297** | **1607.8307** | **-0.65** | **0** | **63** | **7.4e-006** | **1** | **U** | **R.ASYVAPLTAQPATYR.A** |
|  | 3471 | **876.4411** | **1750.8676** | **1750.8672** | **0.25** | **0** | **6** | **2.8** | **1** | **U** | **K.IFVGNVSAACTSQELR.S** |

  


---

|  |  |
| --- | --- |
| **55.** | sp|A6NMY6|AXA2L\_HUMAN    **Mass:** 38806    **Score:** 85     **Matches:** 3(3)  **Sequences:** 3(3)  **emPAI:** 0.28 |
|  | Putative annexin A2-like protein OS=Homo sapiens OX=9606 GN=ANXA2P2 PE=5 SV=2 |

|  |  |
| --- | --- |
|  | Check to include this hit in error tolerant search or archive report |
|  |  |

|  |  |  |  |  |  |  |  |  |  |  |  |
| --- | --- | --- | --- | --- | --- | --- | --- | --- | --- | --- | --- |
|  | **Query** | **Observed** | **Mr(expt)** | **Mr(calc)** | **ppm** | **Miss** | **Score** | **Expect** | **Rank** | **Unique** | **Peptide** |
|  | 1380 | **544.3022** | **1086.5898** | **1086.5921** | **-2.05** | **0** | **37** | **0.0039** | **1** | **U** | **R.DALNIETAIK.T** |
|  | 1954 | **611.8014** | **1221.5882** | **1221.5877** | **0.43** | **0** | **40** | **0.0014** | **1** | **U** | **K.TPAQYDASELK.A** |
|  | 2048 | **622.8134** | **1243.6121** | **1243.6156** | **-2.81** | **0** | **52** | **0.0001** | **1** | **U** | **R.TNQELQEINR.V** |

  

|  |  |
| --- | --- |
|  | |
|  | **Proteins matching the same set of peptides:** |

|  |  |
| --- | --- |
|  | sp|P07355|ANXA2\_HUMAN    **Mass:** 38808    **Score:** 85     **Matches:** 3(3)  **Sequences:** 3(3) |
|  | Annexin A2 OS=Homo sapiens OX=9606 GN=ANXA2 PE=1 SV=2 |

---

|  |  |
| --- | --- |
| **56.** | sp|Q7Z3Y9|K1C26\_HUMAN    **Mass:** 52620    **Score:** 83     **Matches:** 3(2)  **Sequences:** 2(1)  **emPAI:** 0.06 |
|  | Keratin, type I cytoskeletal 26 OS=Homo sapiens OX=9606 GN=KRT26 PE=1 SV=2 |

|  |  |
| --- | --- |
|  | Check to include this hit in error tolerant search or archive report |
|  |  |

|  |  |  |  |  |  |  |  |  |  |  |  |
| --- | --- | --- | --- | --- | --- | --- | --- | --- | --- | --- | --- |
|  | **Query** | **Observed** | **Mr(expt)** | **Mr(calc)** | **ppm** | **Miss** | **Score** | **Expect** | **Rank** | **Unique** | **Peptide** |
|  | 1019 | 511.2551 | 1020.4956 | 1020.4975 | -1.89 | 0 | 7 | 3.4 | 4 | U | K.DSTEETIVK.T |
|  | 1392 | 545.7685 | 1089.5224 | 1089.5237 | -1.15 | 0 | (51) | 0.00012 | 1 |  | K.VTMQNLNDR.L |
|  | 1393 | 545.7700 | 1089.5254 | 1089.5237 | 1.55 | 0 | 51 | 0.00012 | 1 |  | K.VTMQNLNDR.L |

  


---

|  |  |
| --- | --- |
| **57.** | sp|P35030|TRY3\_HUMAN    **Mass:** 33306    **Score:** 81     **Matches:** 2(1)  **Sequences:** 1(1)  **emPAI:** 0.10 |
|  | Trypsin-3 OS=Homo sapiens OX=9606 GN=PRSS3 PE=1 SV=2 |

|  |  |
| --- | --- |
|  | Check to include this hit in error tolerant search or archive report |
|  |  |

|  |  |  |  |  |  |  |  |  |  |  |  |
| --- | --- | --- | --- | --- | --- | --- | --- | --- | --- | --- | --- |
|  | **Query** | **Observed** | **Mr(expt)** | **Mr(calc)** | **ppm** | **Miss** | **Score** | **Expect** | **Rank** | **Unique** | **Peptide** |
|  | 2755 | **716.8719** | **1431.7292** | **1431.7358** | **-4.57** | **0** | **(18)** | **0.27** | **1** | **U** | **K.VLEGNEQFINAAK.I** |
|  | 2756 | **716.8749** | **1431.7352** | **1431.7358** | **-0.39** | **0** | **81** | **1.3e-007** | **1** | **U** | **K.VLEGNEQFINAAK.I** |

  


---

|  |  |
| --- | --- |
| **58.** | sp|P62241|RS8\_HUMAN    **Mass:** 24475    **Score:** 72     **Matches:** 3(2)  **Sequences:** 3(2)  **emPAI:** 0.29 |
|  | 40S ribosomal protein S8 OS=Homo sapiens OX=9606 GN=RPS8 PE=1 SV=2 |

|  |  |
| --- | --- |
|  | Check to include this hit in error tolerant search or archive report |
|  |  |

|  |  |  |  |  |  |  |  |  |  |  |  |
| --- | --- | --- | --- | --- | --- | --- | --- | --- | --- | --- | --- |
|  | **Query** | **Observed** | **Mr(expt)** | **Mr(calc)** | **ppm** | **Miss** | **Score** | **Expect** | **Rank** | **Unique** | **Peptide** |
|  | 826 | **485.2556** | **968.4965** | **968.4967** | **-0.16** | **0** | **17** | **0.24** | **1** | **U** | **K.ELEFYLR.K** |
|  | 2307 | **657.8429** | **1313.6712** | **1313.6714** | **-0.13** | **0** | **44** | **0.00054** | **1** | **U** | **K.LTPEEEEILNK.K** |
|  | 2947 | **753.8945** | **1505.7744** | **1505.7726** | **1.22** | **0** | **49** | **0.00016** | **1** | **U** | **K.ISSLLEEQFQQGK.L** |

  


---

|  |  |
| --- | --- |
| **59.** | sp|P19013|K2C4\_HUMAN    **Mass:** 57649    **Score:** 71     **Matches:** 5(3)  **Sequences:** 3(2)  **emPAI:** 0.12 |
|  | Keratin, type II cytoskeletal 4 OS=Homo sapiens OX=9606 GN=KRT4 PE=1 SV=4 |

|  |  |
| --- | --- |
|  | Check to include this hit in error tolerant search or archive report |
|  |  |

|  |  |  |  |  |  |  |  |  |  |  |  |
| --- | --- | --- | --- | --- | --- | --- | --- | --- | --- | --- | --- |
|  | **Query** | **Observed** | **Mr(expt)** | **Mr(calc)** | **ppm** | **Miss** | **Score** | **Expect** | **Rank** | **Unique** | **Peptide** |
|  | 248 | 414.2183 | 826.4220 | 826.4225 | -0.57 | 0 | (29) | 0.014 | 1 |  | K.FASFIDK.V |
|  | 249 | 414.2186 | 826.4226 | 826.4225 | 0.11 | 0 | 44 | 0.00045 | 1 |  | K.FASFIDK.V |
|  | 1473 | 554.2658 | 1106.5171 | 1106.5356 | -16.75 | 0 | (1) | 11 | 2 |  | R.AQYEEIAQR.S |
|  | 1475 | 554.2739 | 1106.5333 | 1106.5356 | -2.07 | 0 | 42 | 0.00091 | 1 |  | R.AQYEEIAQR.S |
|  | 1560 | 563.7762 | 1125.5378 | 1125.5376 | 0.20 | 0 | 15 | 0.42 | 1 |  | R.EYQELMSVK.L |

  


---

|  |  |
| --- | --- |
| **60.** | sp|Q9H4B7|TBB1\_HUMAN    **Mass:** 50865    **Score:** 69     **Matches:** 6(2)  **Sequences:** 3(2)  **emPAI:** 0.13 |
|  | Tubulin beta-1 chain OS=Homo sapiens OX=9606 GN=TUBB1 PE=1 SV=1 |

|  |  |
| --- | --- |
|  | Check to include this hit in error tolerant search or archive report |
|  |  |

|  |  |  |  |  |  |  |  |  |  |  |  |
| --- | --- | --- | --- | --- | --- | --- | --- | --- | --- | --- | --- |
|  | **Query** | **Observed** | **Mr(expt)** | **Mr(calc)** | **ppm** | **Miss** | **Score** | **Expect** | **Rank** | **Unique** | **Peptide** |
|  | 1586 | 565.8026 | 1129.5907 | 1129.5880 | 2.36 | 0 | 47 | 0.00043 | 1 |  | R.FPGQLNADLR.K |
|  | 1587 | 565.8045 | 1129.5944 | 1129.5880 | 5.71 | 0 | (16) | 0.57 | 1 |  | R.FPGQLNADLR.K |
|  | 1646 | 572.3210 | 1142.6275 | 1142.6270 | 0.44 | 0 | 47 | 0.00034 | 1 |  | K.LAVNMVPFPR.L |
|  | 4219 | **1134.5652** | **2267.1158** | **2267.1368** | **-9.26** | **0** | **3** | **4.8** | **1** | **U** | **R.GLSMAATFIGNNTAIQEIFNR.V** |
|  | 4220 | **756.7129** | **2267.1168** | **2267.1368** | **-8.81** | **0** | **(1)** | **8.7** | **3** | **U** | **R.GLSMAATFIGNNTAIQEIFNR.V** |
|  | 4222 | **1134.5676** | **2267.1207** | **2267.1368** | **-7.11** | **0** | **(1)** | **8.3** | **1** | **U** | **R.GLSMAATFIGNNTAIQEIFNR.V** |

  


---

|  |  |
| --- | --- |
| **61.** | sp|P06702|S10A9\_HUMAN    **Mass:** 13291    **Score:** 60     **Matches:** 6(3)  **Sequences:** 5(2)  **emPAI:** 0.98 |
|  | Protein S100-A9 OS=Homo sapiens OX=9606 GN=S100A9 PE=1 SV=1 |

|  |  |
| --- | --- |
|  | Check to include this hit in error tolerant search or archive report |
|  |  |

|  |  |  |  |  |  |  |  |  |  |  |  |
| --- | --- | --- | --- | --- | --- | --- | --- | --- | --- | --- | --- |
|  | **Query** | **Observed** | **Mr(expt)** | **Mr(calc)** | **ppm** | **Miss** | **Score** | **Expect** | **Rank** | **Unique** | **Peptide** |
|  | 516 | **439.2424** | **876.4703** | **876.4705** | **-0.22** | **0** | **23** | **0.091** | **1** | **U** | **K.DLQNFLK.K** |
|  | 966 | **503.2905** | **1004.5665** | **1004.5655** | **1.04** | **1** | **21** | **0.083** | **1** | **U** | **R.KDLQNFLK.K** |
|  | 2829 | **485.9124** | **1454.7153** | **1454.7154** | **-0.06** | **0** | **(27)** | **0.031** | **1** | **U** | **K.LGHPDTLNQGEFK.E** |
|  | 2830 | **728.3661** | **1454.7177** | **1454.7154** | **1.62** | **0** | **43** | **0.00064** | **1** | **U** | **K.LGHPDTLNQGEFK.E** |
|  | 3454 | **871.9167** | **1741.8189** | **1741.8192** | **-0.17** | **0** | **31** | **0.0087** | **1** | **U** | **K.VIEHIMEDLDTNADK.Q** |
|  | 4078 | 1065.5187 | 2129.0228 | 2128.9946 | 13.2 | 1 | 5 | 3.2 | 1 | U | K.NEKVIEHIMEDLDTNADK.Q + Oxidation (M) |

  


---

|  |  |
| --- | --- |
| **62.** | sp|P05109|S10A8\_HUMAN    **Mass:** 10885    **Score:** 59     **Matches:** 3(2)  **Sequences:** 2(1)  **emPAI:** 1.29 |
|  | Protein S100-A8 OS=Homo sapiens OX=9606 GN=S100A8 PE=1 SV=1 |

|  |  |
| --- | --- |
|  | Check to include this hit in error tolerant search or archive report |
|  |  |

|  |  |  |  |  |  |  |  |  |  |  |  |
| --- | --- | --- | --- | --- | --- | --- | --- | --- | --- | --- | --- |
|  | **Query** | **Observed** | **Mr(expt)** | **Mr(calc)** | **ppm** | **Miss** | **Score** | **Expect** | **Rank** | **Unique** | **Peptide** |
|  | 222 | **411.7111** | **821.4076** | **821.4072** | **0.45** | **0** | **21** | **0.1** | **1** | **U** | **K.GADVWFK.E** |
|  | 2157 | **636.8510** | **1271.6875** | **1271.6874** | **0.08** | **0** | **54** | **6.3e-005** | **1** | **U** | **K.ALNSIIDVYHK.Y** |
|  | 2158 | **424.9041** | **1271.6904** | **1271.6874** | **2.40** | **0** | **(29)** | **0.017** | **1** | **U** | **K.ALNSIIDVYHK.Y** |

  


---

|  |  |
| --- | --- |
| **63.** | sp|P35900|K1C20\_HUMAN    **Mass:** 48514    **Score:** 57     **Matches:** 2(1)  **Sequences:** 2(1)  **emPAI:** 0.07 |
|  | Keratin, type I cytoskeletal 20 OS=Homo sapiens OX=9606 GN=KRT20 PE=1 SV=1 |

|  |  |
| --- | --- |
|  | Check to include this hit in error tolerant search or archive report |
|  |  |

|  |  |  |  |  |  |  |  |  |  |  |  |
| --- | --- | --- | --- | --- | --- | --- | --- | --- | --- | --- | --- |
|  | **Query** | **Observed** | **Mr(expt)** | **Mr(calc)** | **ppm** | **Miss** | **Score** | **Expect** | **Rank** | **Unique** | **Peptide** |
|  | 2539 | 460.5807 | 1378.7202 | 1378.7204 | -0.21 | 1 | 57 | 3.4e-005 | 1 |  | K.TRLEQEIATYR.R |
|  | 2807 | **724.8547** | **1447.6949** | **1447.6956** | **-0.49** | **1** | **2** | **9** | **5** | **U** | **K.QWYETNAPRAGR.D** |

  


---

|  |  |
| --- | --- |
| **64.** | sp|Q5T749|KPRP\_HUMAN    **Mass:** 67172    **Score:** 54     **Matches:** 6(3)  **Sequences:** 5(2)  **emPAI:** 0.10 |
|  | Keratinocyte proline-rich protein OS=Homo sapiens OX=9606 GN=KPRP PE=1 SV=1 |

|  |  |
| --- | --- |
|  | Check to include this hit in error tolerant search or archive report |
|  |  |

|  |  |  |  |  |  |  |  |  |  |  |  |
| --- | --- | --- | --- | --- | --- | --- | --- | --- | --- | --- | --- |
|  | **Query** | **Observed** | **Mr(expt)** | **Mr(calc)** | **ppm** | **Miss** | **Score** | **Expect** | **Rank** | **Unique** | **Peptide** |
|  | 127 | **387.2366** | **772.4587** | **772.4595** | **-1.09** | **0** | **(34)** | **0.011** | **1** | **U** | **R.LQLFPR.S** |
|  | 128 | **387.2369** | **772.4592** | **772.4595** | **-0.44** | **0** | **37** | **0.0051** | **1** | **U** | **R.LQLFPR.S** |
|  | 684 | **465.2894** | **928.5642** | **928.5607** | **3.81** | **1** | **4** | **2.8** | **3** | **U** | **R.RLQLFPR.S** |
|  | 1347 | **540.7972** | **1079.5799** | **1079.5797** | **0.17** | **0** | **32** | **0.0089** | **1** | **U** | **R.CPVEIPPIR.R** |
|  | 2932 | **749.8718** | **1497.7291** | **1497.7358** | **-4.46** | **1** | **18** | **0.18** | **1** | **U** | **R.RLDQCPESPLQR.C** |
|  | 3891 | **971.9440** | **1941.8735** | **1941.8929** | **-10.00** | **1** | **7** | **1.3** | **2** | **U** | **R.GQDGHGDQGNAFAGVKGEAK.S** |

  


---

|  |  |
| --- | --- |
| **65.** | sp|Q8N1N4|K2C78\_HUMAN    **Mass:** 57629    **Score:** 54     **Matches:** 8(2)  **Sequences:** 8(2)  **emPAI:** 0.18 |
|  | Keratin, type II cytoskeletal 78 OS=Homo sapiens OX=9606 GN=KRT78 PE=1 SV=2 |

|  |  |
| --- | --- |
|  | Check to include this hit in error tolerant search or archive report |
|  |  |

|  |  |  |  |  |  |  |  |  |  |  |  |
| --- | --- | --- | --- | --- | --- | --- | --- | --- | --- | --- | --- |
|  | **Query** | **Observed** | **Mr(expt)** | **Mr(calc)** | **ppm** | **Miss** | **Score** | **Expect** | **Rank** | **Unique** | **Peptide** |
|  | 852 | **488.2635** | **974.5124** | **974.5113** | **1.10** | **0** | **9** | **2.8** | **1** | **U** | **R.EYLYFLK.H** |
|  | 965 | 503.2367 | 1004.4589 | 1004.4597 | -0.78 | 0 | 22 | 0.08 | 1 |  | R.LLEGEECR.M |
|  | 1000 | **508.2747** | **1014.5348** | **1014.5345** | **0.22** | **0** | **46** | **0.00045** | **1** | **U** | **K.VDELEAALR.M** |
|  | 2056 | **624.8456** | **1247.6766** | **1247.6761** | **0.37** | **0** | **13** | **0.81** | **1** | **U** | **R.ATLENDFVVLK.K** |
|  | 2412 | **672.3701** | **1342.7256** | **1342.7245** | **0.79** | **0** | **30** | **0.014** | **1** | **U** | **K.IEIDPQFQVVR.T** |
|  | 2490 | 681.3488 | 1360.6829 | 1360.7020 | -14.01 | 1 | 3 | 11 | 7 | U | K.VDELEAALRMAK.Q + Oxidation (M) |
|  | 2624 | **699.3571** | **1396.6997** | **1396.6987** | **0.73** | **0** | **16** | **0.41** | **1** | **U** | **R.TLNNQFASFIDK.V** |
|  | 2880 | **493.6070** | **1477.7991** | **1477.8001** | **-0.72** | **0** | **12** | **0.57** | **1** | **U** | **K.VQISQLHQEIQR.L** |

  


---

|  |  |
| --- | --- |
| **66.** | sp|P14923|PLAK\_HUMAN    **Mass:** 82434    **Score:** 52     **Matches:** 13(3)  **Sequences:** 10(3)  **emPAI:** 0.17 |
|  | Junction plakoglobin OS=Homo sapiens OX=9606 GN=JUP PE=1 SV=3 |

|  |  |
| --- | --- |
|  | Check to include this hit in error tolerant search or archive report |
|  |  |

|  |  |  |  |  |  |  |  |  |  |  |  |
| --- | --- | --- | --- | --- | --- | --- | --- | --- | --- | --- | --- |
|  | **Query** | **Observed** | **Mr(expt)** | **Mr(calc)** | **ppm** | **Miss** | **Score** | **Expect** | **Rank** | **Unique** | **Peptide** |
|  | 67 | **372.2412** | **742.4679** | **742.4701** | **-2.94** | **0** | **29** | **0.014** | **1** | **U** | **K.ATIGLIR.N** |
|  | 205 | **406.7835** | **811.5525** | **811.5531** | **-0.80** | **0** | **24** | **0.0067** | **1** | **U** | **R.LVQLLVK.A** |
|  | 515 | **438.7509** | **875.4873** | **875.4865** | **0.97** | **0** | **3** | **8.1** | **1** | **U** | **K.LLWTTSR.V** |
|  | 952 | **501.7771** | **1001.5396** | **1001.5393** | **0.34** | **0** | **18** | **0.37** | **1** | **U** | **K.QEGLESVLK.I** |
|  | 1887 | **401.2347** | **1200.6822** | **1200.6649** | **14.5** | **1** | **(3)** | **5.1** | **7** | **U** | **K.MAVRLADGLQK.M** |
|  | 1888 | **601.3484** | **1200.6823** | **1200.6649** | **14.6** | **1** | **10** | **1.2** | **8** | **U** | **K.MAVRLADGLQK.M** |
|  | 2439 | **676.8085** | **1351.6025** | **1351.6038** | **-0.95** | **0** | **32** | **0.0046** | **1** | **U** | **R.TMQNTSDLDTAR.C** |
|  | 2668 | **706.4079** | **1410.8012** | **1410.8017** | **-0.32** | **0** | **21** | **0.06** | **1** | **U** | **R.ALMGSPQLVAAVVR.T** |
|  | 3167 | **800.3972** | **1598.7799** | **1598.7801** | **-0.16** | **0** | **1** | **10** | **3** | **U** | **R.HVAAGTQQPYTDGVR.M** |
|  | 3426 | **865.9229** | **1729.8313** | **1729.8305** | **0.45** | **1** | **10** | **1** | **1** | **U** | **R.AGDKDDITEPAVCALR.H** |
|  | 3818 | **633.9528** | **1898.8365** | **1898.8502** | **-7.25** | **1** | **(1)** | **4.1** | **2** | **U** | **K.GIMEEDEACGRQYTLK.K** |
|  | 3819 | **950.4257** | **1898.8368** | **1898.8502** | **-7.08** | **1** | **(1)** | **3.8** | **2** | **U** | **K.GIMEEDEACGRQYTLK.K** |
|  | 3821 | **950.4263** | **1898.8381** | **1898.8502** | **-6.38** | **1** | **1** | **3.9** | **5** | **U** | **K.GIMEEDEACGRQYTLK.K** |

  


---

|  |  |
| --- | --- |
| **67.** | sp|Q92764|KRT35\_HUMAN    **Mass:** 51640    **Score:** 52     **Matches:** 3(2)  **Sequences:** 2(1)  **emPAI:** 0.06 |
|  | Keratin, type I cuticular Ha5 OS=Homo sapiens OX=9606 GN=KRT35 PE=2 SV=5 |

|  |  |
| --- | --- |
|  | Check to include this hit in error tolerant search or archive report |
|  |  |

|  |  |  |  |  |  |  |  |  |  |  |  |
| --- | --- | --- | --- | --- | --- | --- | --- | --- | --- | --- | --- |
|  | **Query** | **Observed** | **Mr(expt)** | **Mr(calc)** | **ppm** | **Miss** | **Score** | **Expect** | **Rank** | **Unique** | **Peptide** |
|  | 189 | 404.2032 | 806.3918 | 806.3923 | -0.61 | 0 | 43 | 0.0011 | 1 |  | K.LAADDFR.T |
|  | 190 | 404.2034 | 806.3923 | 806.3923 | 0.06 | 0 | (31) | 0.017 | 1 |  | K.LAADDFR.T |
|  | 948 | 500.7871 | 999.5595 | 999.5600 | -0.50 | 0 | 4 | 6.4 | 10 | U | R.LVVEIDNAK.L |

  


---

|  |  |
| --- | --- |
| **68.** | sp|P55795|HNRH2\_HUMAN    **Mass:** 49517    **Score:** 51     **Matches:** 5(2)  **Sequences:** 4(1)  **emPAI:** 0.14 |
|  | Heterogeneous nuclear ribonucleoprotein H2 OS=Homo sapiens OX=9606 GN=HNRNPH2 PE=1 SV=1 |

|  |  |
| --- | --- |
|  | Check to include this hit in error tolerant search or archive report |
|  |  |

|  |  |  |  |  |  |  |  |  |  |  |  |
| --- | --- | --- | --- | --- | --- | --- | --- | --- | --- | --- | --- |
|  | **Query** | **Observed** | **Mr(expt)** | **Mr(calc)** | **ppm** | **Miss** | **Score** | **Expect** | **Rank** | **Unique** | **Peptide** |
|  | 139 | 392.7157 | 783.4168 | 783.4167 | 0.21 | 0 | 9 | 0.95 | 1 |  | R.YVEVFK.S |
|  | 200 | 406.7307 | 811.4469 | 811.4480 | -1.28 | 0 | 14 | 0.32 | 1 |  | R.YIEIFK.S |
|  | 1405 | 364.8644 | 1091.5714 | 1091.5724 | -0.87 | 0 | 38 | 0.0027 | 1 |  | R.VHIEIGPDGR.V |
|  | 1406 | 546.7938 | 1091.5730 | 1091.5724 | 0.56 | 0 | (33) | 0.0082 | 1 |  | R.VHIEIGPDGR.V |
|  | 3133 | **792.3889** | **1582.7631** | **1582.7847** | **-13.63** | **1** | **1** | **8.5** | **2** | **U** | **-.MMLSTEGREGFVVK.V** |

  


---

|  |  |
| --- | --- |
| **69.** | sp|Q8TF72|SHRM3\_HUMAN    **Mass:** 218321   **Score:** 49     **Matches:** 2(2)  **Sequences:** 1(1)  **emPAI:** 0.01 |
|  | Protein Shroom3 OS=Homo sapiens OX=9606 GN=SHROOM3 PE=1 SV=2 |

|  |  |
| --- | --- |
|  | Check to include this hit in error tolerant search or archive report |
|  |  |

|  |  |  |  |  |  |  |  |  |  |  |  |
| --- | --- | --- | --- | --- | --- | --- | --- | --- | --- | --- | --- |
|  | **Query** | **Observed** | **Mr(expt)** | **Mr(calc)** | **ppm** | **Miss** | **Score** | **Expect** | **Rank** | **Unique** | **Peptide** |
|  | 694 | **466.7433** | **931.4721** | **931.4723** | **-0.22** | **1** | **39** | **0.0027** | **1** | **U** | **R.SSPATADKR.Q** |
|  | 695 | **466.7440** | **931.4734** | **931.4723** | **1.16** | **1** | **(36)** | **0.0059** | **1** | **U** | **R.SSPATADKR.Q** |

  


---

|  |  |
| --- | --- |
| **70.** | sp|Q5T750|XP32\_HUMAN    **Mass:** 28557    **Score:** 47     **Matches:** 2(1)  **Sequences:** 2(1)  **emPAI:** 0.12 |
|  | Skin-specific protein 32 OS=Homo sapiens OX=9606 GN=XP32 PE=1 SV=1 |

|  |  |
| --- | --- |
|  | Check to include this hit in error tolerant search or archive report |
|  |  |

|  |  |  |  |  |  |  |  |  |  |  |  |
| --- | --- | --- | --- | --- | --- | --- | --- | --- | --- | --- | --- |
|  | **Query** | **Observed** | **Mr(expt)** | **Mr(calc)** | **ppm** | **Miss** | **Score** | **Expect** | **Rank** | **Unique** | **Peptide** |
|  | 514 | **438.7502** | **875.4857** | **875.4865** | **-0.87** | **0** | **47** | **0.00031** | **1** | **U** | **R.TFGVSPLR.R** |
|  | 2095 | **628.8021** | **1255.5896** | **1255.5907** | **-0.91** | **0** | **2** | **7.7** | **1** | **U** | **K.YPTPCQTYVK.C** |

  


---

|  |  |
| --- | --- |
| **71.** | sp|P15924|DESP\_HUMAN    **Mass:** 334021   **Score:** 46     **Matches:** 8(1)  **Sequences:** 8(1)  **emPAI:** 0.01 |
|  | Desmoplakin OS=Homo sapiens OX=9606 GN=DSP PE=1 SV=3 |

|  |  |
| --- | --- |
|  | Check to include this hit in error tolerant search or archive report |
|  |  |

|  |  |  |  |  |  |  |  |  |  |  |  |
| --- | --- | --- | --- | --- | --- | --- | --- | --- | --- | --- | --- |
|  | **Query** | **Observed** | **Mr(expt)** | **Mr(calc)** | **ppm** | **Miss** | **Score** | **Expect** | **Rank** | **Unique** | **Peptide** |
|  | 222 | 411.7111 | 821.4076 | 821.3953 | 15.0 | 0 | 6 | 3.3 | 2 | U | K.MSAAEAVK.E + Oxidation (M) |
|  | 1884 | **601.3301** | **1200.6456** | **1200.6350** | **8.84** | **0** | **8** | **2.6** | **1** | **U** | **R.QLLQEQESVK.Q** |
|  | 2151 | **636.3585** | **1270.7024** | **1270.6993** | **2.39** | **0** | **46** | **0.00031** | **1** | **U** | **R.QLQNIIQATSR.E** |
|  | 2442 | **676.8591** | **1351.7037** | **1351.6983** | **4.00** | **1** | **7** | **2.7** | **2** | **U** | **R.NKYETEINITK.T** |
|  | 2677 | **707.8566** | **1413.6986** | **1413.7001** | **-1.04** | **0** | **16** | **0.35** | **1** | **U** | **K.QQIQNDLNQWK.T** |
|  | 2840 | **730.8750** | **1459.7354** | **1459.7518** | **-11.20** | **1** | **7** | **3.5** | **2** | **U** | **K.QSLEEAAKTIQDK.N** |
|  | 2975 | **759.3613** | **1516.7080** | **1516.7126** | **-3.05** | **1** | **0** | **10** | **10** | **U** | **R.QIEHCEGRMTLK.N + Oxidation (M)** |
|  | 3858 | **641.9742** | **1922.9009** | **1922.8905** | **5.43** | **1** | **2** | **6.6** | **2** | **U** | **K.GLPSPYNMSSAPGSRSGSR.S + Oxidation (M)** |

  


---

|  |  |
| --- | --- |
| **72.** | sp|Q8NHM4|TRY6\_HUMAN    **Mass:** 27090    **Score:** 45     **Matches:** 4(2)  **Sequences:** 1(1)  **emPAI:** 0.26 |
|  | Putative trypsin-6 OS=Homo sapiens OX=9606 GN=PRSS3P2 PE=5 SV=2 |

|  |  |
| --- | --- |
|  | Check to include this hit in error tolerant search or archive report |
|  |  |

|  |  |  |  |  |  |  |  |  |  |  |  |
| --- | --- | --- | --- | --- | --- | --- | --- | --- | --- | --- | --- |
|  | **Query** | **Observed** | **Mr(expt)** | **Mr(calc)** | **ppm** | **Miss** | **Score** | **Expect** | **Rank** | **Unique** | **Peptide** |
|  | 4162 | **742.3669** | **2224.0788** | **2224.1124** | **-15.09** | **0** | **(14)** | **0.4** | **1** | **U** | **R.LGEHNIEVLEGNEQFINAAK.I** |
|  | 4163 | **742.3769** | **2224.1088** | **2224.1124** | **-1.58** | **0** | **41** | **0.00075** | **1** | **U** | **R.LGEHNIEVLEGNEQFINAAK.I** |
|  | 4164 | **1113.0623** | **2224.1100** | **2224.1124** | **-1.08** | **0** | **(23)** | **0.047** | **1** | **U** | **R.LGEHNIEVLEGNEQFINAAK.I** |
|  | 4166 | **1113.0636** | **2224.1126** | **2224.1124** | **0.13** | **0** | **(3)** | **5.8** | **1** | **U** | **R.LGEHNIEVLEGNEQFINAAK.I** |

  

|  |  |
| --- | --- |
|  | |
|  | **Proteins matching the same set of peptides:** |

|  |  |
| --- | --- |
|  | sp|P07477|TRY1\_HUMAN    **Mass:** 27111    **Score:** 45     **Matches:** 4(2)  **Sequences:** 1(1) |
|  | Trypsin-1 OS=Homo sapiens OX=9606 GN=PRSS1 PE=1 SV=1 |

|  |  |
| --- | --- |
|  | sp|P07478|TRY2\_HUMAN    **Mass:** 26927    **Score:** 45     **Matches:** 4(2)  **Sequences:** 1(1) |
|  | Trypsin-2 OS=Homo sapiens OX=9606 GN=PRSS2 PE=1 SV=1 |

---

|  |  |
| --- | --- |
| **73.** | sp|P0DOX5|IGG1\_HUMAN    **Mass:** 49925    **Score:** 45     **Matches:** 5(5)  **Sequences:** 2(2)  **emPAI:** 0.21 |
|  | Immunoglobulin gamma-1 heavy chain OS=Homo sapiens OX=9606 PE=1 SV=2 |

|  |  |
| --- | --- |
|  | Check to include this hit in error tolerant search or archive report |
|  |  |

|  |  |  |  |  |  |  |  |  |  |  |  |
| --- | --- | --- | --- | --- | --- | --- | --- | --- | --- | --- | --- |
|  | **Query** | **Observed** | **Mr(expt)** | **Mr(calc)** | **ppm** | **Miss** | **Score** | **Expect** | **Rank** | **Unique** | **Peptide** |
|  | 277 | **418.2215** | **834.4284** | **834.4269** | **1.75** | **0** | **(29)** | **0.024** | **1** | **U** | **K.DTLMISR.T** |
|  | 279 | **419.7549** | **837.4953** | **837.4960** | **-0.77** | **0** | **(22)** | **0.034** | **1** | **U** | **K.ALPAPIEK.T** |
|  | 280 | **419.7552** | **837.4958** | **837.4960** | **-0.24** | **0** | **23** | **0.032** | **1** | **U** | **K.ALPAPIEK.T** |
|  | 401 | **426.2177** | **850.4208** | **850.4218** | **-1.22** | **0** | **(28)** | **0.023** | **1** | **U** | **K.DTLMISR.T + Oxidation (M)** |
|  | 402 | **426.2182** | **850.4218** | **850.4218** | **-0.07** | **0** | **31** | **0.011** | **1** | **U** | **K.DTLMISR.T + Oxidation (M)** |

  

|  |  |
| --- | --- |
|  | |
|  | **Proteins matching the same set of peptides:** |

|  |  |
| --- | --- |
|  | sp|P01857|IGHG1\_HUMAN    **Mass:** 36596    **Score:** 45     **Matches:** 5(5)  **Sequences:** 2(2) |
|  | Immunoglobulin heavy constant gamma 1 OS=Homo sapiens OX=9606 GN=IGHG1 PE=1 SV=1 |

|  |  |
| --- | --- |
|  | sp|P01860|IGHG3\_HUMAN    **Mass:** 42287    **Score:** 45     **Matches:** 5(5)  **Sequences:** 2(2) |
|  | Immunoglobulin heavy constant gamma 3 OS=Homo sapiens OX=9606 GN=IGHG3 PE=1 SV=2 |

---

|  |  |
| --- | --- |
| **74.** | sp|Q9NQI0|DDX4\_HUMAN    **Mass:** 80113    **Score:** 44     **Matches:** 2(1)  **Sequences:** 2(1)  **emPAI:** 0.04 |
|  | Probable ATP-dependent RNA helicase DDX4 OS=Homo sapiens OX=9606 GN=DDX4 PE=1 SV=2 |

|  |  |
| --- | --- |
|  | Check to include this hit in error tolerant search or archive report |
|  |  |

|  |  |  |  |  |  |  |  |  |  |  |  |
| --- | --- | --- | --- | --- | --- | --- | --- | --- | --- | --- | --- |
|  | **Query** | **Observed** | **Mr(expt)** | **Mr(calc)** | **ppm** | **Miss** | **Score** | **Expect** | **Rank** | **Unique** | **Peptide** |
|  | 1271 | **533.2773** | **1064.5401** | **1064.5251** | **14.1** | **0** | **1** | **12** | **3** | **U** | **R.GNVFASVDTR.K** |
|  | 2017 | 619.7784 | 1237.5422 | 1237.5431 | -0.73 | 0 | 44 | 0.00027 | 1 |  | R.DLMACAQTGSGK.T |

  


---

|  |  |
| --- | --- |
| **75.** | sp|P12236|ADT3\_HUMAN    **Mass:** 33073    **Score:** 44     **Matches:** 5(1)  **Sequences:** 5(1)  **emPAI:** 0.21 |
|  | ADP/ATP translocase 3 OS=Homo sapiens OX=9606 GN=SLC25A6 PE=1 SV=4 |

|  |  |
| --- | --- |
|  | Check to include this hit in error tolerant search or archive report |
|  |  |

|  |  |  |  |  |  |  |  |  |  |  |  |
| --- | --- | --- | --- | --- | --- | --- | --- | --- | --- | --- | --- |
|  | **Query** | **Observed** | **Mr(expt)** | **Mr(calc)** | **ppm** | **Miss** | **Score** | **Expect** | **Rank** | **Unique** | **Peptide** |
|  | 431 | **431.2297** | **860.4449** | **860.4426** | **2.64** | **0** | **16** | **0.54** | **1** | **U** | **R.GLGDCLVK.I** |
|  | 588 | **451.7461** | **901.4776** | **901.4770** | **0.64** | **0** | **44** | **0.0011** | **1** | **U** | **K.GAWSNVLR.G** |
|  | 1539 | **561.2913** | **1120.5681** | **1120.5665** | **1.38** | **0** | **21** | **0.14** | **1** | **U** | **K.EQGVLSFWR.G** |
|  | 1611 | **568.8426** | **1135.6706** | **1135.6713** | **-0.61** | **0** | **8** | **0.57** | **1** | **U** | **K.LLLQVQHASK.Q** |
|  | 4286 | **780.7385** | **2339.1936** | **2339.2195** | **-11.08** | **1** | **0** | **9.9** | **6** | **U** | **-.MTEQAISFAKDFLAGGIAAAISK.T** |

  


---

|  |  |
| --- | --- |
| **76.** | sp|P61978|HNRPK\_HUMAN    **Mass:** 51230    **Score:** 44     **Matches:** 4(1)  **Sequences:** 4(1)  **emPAI:** 0.06 |
|  | Heterogeneous nuclear ribonucleoprotein K OS=Homo sapiens OX=9606 GN=HNRNPK PE=1 SV=1 |

|  |  |
| --- | --- |
|  | Check to include this hit in error tolerant search or archive report |
|  |  |

|  |  |  |  |  |  |  |  |  |  |  |  |
| --- | --- | --- | --- | --- | --- | --- | --- | --- | --- | --- | --- |
|  | **Query** | **Observed** | **Mr(expt)** | **Mr(calc)** | **ppm** | **Miss** | **Score** | **Expect** | **Rank** | **Unique** | **Peptide** |
|  | 254 | **414.7117** | **827.4089** | **827.4137** | **-5.81** | **0** | **2** | **7.4** | **7** | **U** | **R.HESGASIK.I** |
|  | 2110 | **630.2911** | **1258.5676** | **1258.5677** | **-0.08** | **0** | **44** | **0.00028** | **1** | **U** | **K.IDEPLEGSEDR.I** |
|  | 3470 | **876.4044** | **1750.7942** | **1750.7944** | **-0.13** | **1** | **3** | **4.9** | **3** | **U** | **K.RPAEDMEEEQAFKR.S + Oxidation (M)** |
|  | 3540 | **594.2712** | **1779.7919** | **1779.7911** | **0.42** | **0** | **2** | **3.9** | **4** | **U** | **R.TDYNASVSVPDSSGPER.I** |

  


---

|  |  |
| --- | --- |
| **77.** | sp|P04003|C4BPA\_HUMAN    **Mass:** 69042    **Score:** 43     **Matches:** 8(1)  **Sequences:** 8(1)  **emPAI:** 0.05 |
|  | C4b-binding protein alpha chain OS=Homo sapiens OX=9606 GN=C4BPA PE=1 SV=2 |

|  |  |
| --- | --- |
|  | Check to include this hit in error tolerant search or archive report |
|  |  |

|  |  |  |  |  |  |  |  |  |  |  |  |
| --- | --- | --- | --- | --- | --- | --- | --- | --- | --- | --- | --- |
|  | **Query** | **Observed** | **Mr(expt)** | **Mr(calc)** | **ppm** | **Miss** | **Score** | **Expect** | **Rank** | **Unique** | **Peptide** |
|  | 149 | **394.2186** | **786.4227** | **786.4236** | **-1.07** | **0** | **2** | **13** | **6** | **U** | **R.NGQVEIK.T** |
|  | 1013 | **510.2612** | **1018.5078** | **1018.5124** | **-4.50** | **0** | **9** | **2.1** | **3** | **U** | **R.TWYPEVPK.C** |
|  | 1489 | 555.2819 | 1108.5492 | 1108.5488 | 0.36 | 1 | 6 | 3.7 | 2 | U | R.KMAAWPFSR.L + Oxidation (M) |
|  | 1573 | **564.7790** | **1127.5434** | **1127.5434** | **0.06** | **0** | **20** | **0.13** | **1** | **U** | **K.YTCLPGYVR.S** |
|  | 2061 | **625.3429** | **1248.6712** | **1248.6714** | **-0.14** | **0** | **43** | **0.00063** | **1** | **U** | **K.EDVYVVGTVLR.Y** |
|  | 2793 | **722.3527** | **1442.6909** | **1442.6898** | **0.78** | **0** | **16** | **0.25** | **1** | **U** | **R.LMQCLPNPEDVK.M** |
|  | 3132 | **791.3568** | **1580.6989** | **1580.7042** | **-3.32** | **0** | **9** | **0.67** | **1** | **U** | **R.FSAICQGDGTWSPR.T** |
|  | 3375 | **569.6275** | **1705.8607** | **1705.8610** | **-0.21** | **0** | **14** | **0.66** | **1** | **U** | **R.GVGWSHPLPQCEIVK.C** |

  


---

|  |  |
| --- | --- |
| **78.** | sp|Q96P63|SPB12\_HUMAN    **Mass:** 46646    **Score:** 43     **Matches:** 4(1)  **Sequences:** 3(1)  **emPAI:** 0.07 |
|  | Serpin B12 OS=Homo sapiens OX=9606 GN=SERPINB12 PE=1 SV=1 |

|  |  |
| --- | --- |
|  | Check to include this hit in error tolerant search or archive report |
|  |  |

|  |  |  |  |  |  |  |  |  |  |  |  |
| --- | --- | --- | --- | --- | --- | --- | --- | --- | --- | --- | --- |
|  | **Query** | **Observed** | **Mr(expt)** | **Mr(calc)** | **ppm** | **Miss** | **Score** | **Expect** | **Rank** | **Unique** | **Peptide** |
|  | 712 | **467.7666** | **933.5186** | **933.5171** | **1.64** | **0** | **43** | **0.00067** | **1** | **U** | **R.IGFIEEVK.A** |
|  | 841 | 487.2693 | 972.5241 | 972.5240 | 0.12 | 1 | 13 | 1.1 | 4 | U | K.GLEELERK.I |
|  | 842 | 487.2694 | 972.5243 | 972.5240 | 0.30 | 1 | (4) | 7.4 | 8 | U | K.GLEELERK.I |
|  | 4321 | **796.7009** | **2387.0810** | **2387.0773** | **1.52** | **0** | **1** | **4.8** | **4** | **U** | **K.MVAWSSSENMSEESVVLSFPR.F + Oxidation (M)** |

  


---

|  |  |
| --- | --- |
| **79.** | sp|P34931|HS71L\_HUMAN    **Mass:** 70730    **Score:** 41     **Matches:** 3(1)  **Sequences:** 3(1)  **emPAI:** 0.05 |
|  | Heat shock 70 kDa protein 1-like OS=Homo sapiens OX=9606 GN=HSPA1L PE=1 SV=2 |

|  |  |
| --- | --- |
|  | Check to include this hit in error tolerant search or archive report |
|  |  |

|  |  |  |  |  |  |  |  |  |  |  |  |
| --- | --- | --- | --- | --- | --- | --- | --- | --- | --- | --- | --- |
|  | **Query** | **Observed** | **Mr(expt)** | **Mr(calc)** | **ppm** | **Miss** | **Score** | **Expect** | **Rank** | **Unique** | **Peptide** |
|  | 1864 | **599.3506** | **1196.6867** | **1196.6877** | **-0.81** | **0** | **15** | **0.21** | **1** | **U** | **K.DAGVIAGLNVLR.I** |
|  | 2894 | **744.3541** | **1486.6936** | **1486.6940** | **-0.29** | **0** | **41** | **0.00075** | **1** | **U** | **R.TTPSYVAFTDTER.L** |
|  | 2903 | 497.9025 | 1490.6857 | 1490.7109 | -16.91 | 0 | 1 | 7.7 | 7 | U | K.ELEQMCNPIITK.L + Oxidation (M) |

  


---

|  |  |
| --- | --- |
| **80.** | sp|P0C869|PA24B\_HUMAN    **Score:** 41     **Matches:** 1(1)  **Sequences:** 1(1)  **emPAI:** 0.04 |
|  | Cytosolic phospholipase A2 beta OS=Homo sapiens OX=9606 GN=PLA2G4B PE=1 SV=2 |

|  |  |
| --- | --- |
|  | Check to include this hit in error tolerant search or archive report |
|  |  |

|  |  |  |  |  |  |  |  |  |  |  |  |
| --- | --- | --- | --- | --- | --- | --- | --- | --- | --- | --- | --- |
|  | **Query** | **Observed** | **Mr(expt)** | **Mr(calc)** | **ppm** | **Miss** | **Score** | **Expect** | **Rank** | **Unique** | **Peptide** |
|  | 830 | 486.7591 | 971.5036 | 971.5148 | -11.56 | 1 | 41 | 0.0013 | 2 | U | R.QELAERAR.L |

  


---

|  |  |
| --- | --- |
| **81.** | sp|Q00325|MPCP\_HUMAN    **Mass:** 40525    **Score:** 40     **Matches:** 8(1)  **Sequences:** 4(1)  **emPAI:** 0.08 |
|  | Phosphate carrier protein, mitochondrial OS=Homo sapiens OX=9606 GN=SLC25A3 PE=1 SV=2 |

|  |  |
| --- | --- |
|  | Check to include this hit in error tolerant search or archive report |
|  |  |

|  |  |  |  |  |  |  |  |  |  |  |  |
| --- | --- | --- | --- | --- | --- | --- | --- | --- | --- | --- | --- |
|  | **Query** | **Observed** | **Mr(expt)** | **Mr(calc)** | **ppm** | **Miss** | **Score** | **Expect** | **Rank** | **Unique** | **Peptide** |
|  | 223 | **412.2314** | **822.4483** | **822.4487** | **-0.53** | **0** | **2** | **6.6** | **6** | **U** | **R.TVEALYK.F** |
|  | 295 | **421.7580** | **841.5014** | **841.5174** | **-19.04** | **0** | **(3)** | **5.4** | **9** | **U** | **K.FVVPKPR.S** |
|  | 321 | **421.7582** | **841.5017** | **841.5174** | **-18.61** | **0** | **(2)** | **6.7** | **9** | **U** | **K.FVVPKPR.S** |
|  | 369 | **421.7585** | **841.5025** | **841.5174** | **-17.68** | **0** | **(3)** | **5.1** | **10** | **U** | **K.FVVPKPR.S** |
|  | 372 | **421.7588** | **841.5031** | **841.5174** | **-17.02** | **0** | **4** | **4** | **3** | **U** | **K.FVVPKPR.S** |
|  | 375 | **421.7591** | **841.5036** | **841.5174** | **-16.38** | **0** | **(3)** | **5.3** | **7** | **U** | **K.FVVPKPR.S** |
|  | 679 | **465.2560** | **928.4974** | **928.4953** | **2.29** | **0** | **19** | **0.26** | **2** | **U** | **K.GVAPLWMR.Q** |
|  | 2491 | **681.3619** | **1360.7092** | **1360.7099** | **-0.51** | **0** | **40** | **0.0021** | **1** | **U** | **R.IQTQPGYANTLR.D** |

  


---

|  |  |
| --- | --- |
| **82.** | sp|P31151|S10A7\_HUMAN    **Mass:** 11578    **Score:** 39     **Matches:** 1(1)  **Sequences:** 1(1)  **emPAI:** 0.30 |
|  | Protein S100-A7 OS=Homo sapiens OX=9606 GN=S100A7 PE=1 SV=4 |

|  |  |
| --- | --- |
|  | Check to include this hit in error tolerant search or archive report |
|  |  |

|  |  |  |  |  |  |  |  |  |  |  |  |
| --- | --- | --- | --- | --- | --- | --- | --- | --- | --- | --- | --- |
|  | **Query** | **Observed** | **Mr(expt)** | **Mr(calc)** | **ppm** | **Miss** | **Score** | **Expect** | **Rank** | **Unique** | **Peptide** |
|  | 2096 | **628.8119** | **1255.6092** | **1255.6085** | **0.61** | **0** | **39** | **0.0017** | **1** | **U** | **K.GTNYLADVFEK.K** |

  


---

|  |  |
| --- | --- |
| **83.** | sp|P83731|RL24\_HUMAN    **Mass:** 17882    **Score:** 38     **Matches:** 3(1)  **Sequences:** 3(1)  **emPAI:** 0.19 |
|  | 60S ribosomal protein L24 OS=Homo sapiens OX=9606 GN=RPL24 PE=1 SV=1 |

|  |  |
| --- | --- |
|  | Check to include this hit in error tolerant search or archive report |
|  |  |

|  |  |  |  |  |  |  |  |  |  |  |  |
| --- | --- | --- | --- | --- | --- | --- | --- | --- | --- | --- | --- |
|  | **Query** | **Observed** | **Mr(expt)** | **Mr(calc)** | **ppm** | **Miss** | **Score** | **Expect** | **Rank** | **Unique** | **Peptide** |
|  | 817 | **483.7744** | **965.5342** | **965.5334** | **0.79** | **0** | **8** | **1.4** | **1** | **U** | **K.VFQFLNAK.C** |
|  | 1836 | **596.8389** | **1191.6633** | **1191.6400** | **19.5** | **0** | **0** | **9.7** | **2** | **U** | **R.QINWTVLYR.R** |
|  | 2117 | **631.3451** | **1260.6757** | **1260.6747** | **0.80** | **0** | **38** | **0.0019** | **1** | **U** | **R.AITGASLADIMAK.R** |

  


---

|  |  |
| --- | --- |
| **84.** | sp|O75525|KHDR3\_HUMAN    **Mass:** 38776    **Score:** 38     **Matches:** 9(2)  **Sequences:** 5(2)  **emPAI:** 0.28 |
|  | KH domain-containing, RNA-binding, signal transduction-associated protein 3 OS=Homo sapiens OX=9606 GN=KHDRBS3 PE=1 SV=1 |

|  |  |
| --- | --- |
|  | Check to include this hit in error tolerant search or archive report |
|  |  |

|  |  |  |  |  |  |  |  |  |  |  |  |
| --- | --- | --- | --- | --- | --- | --- | --- | --- | --- | --- | --- |
|  | **Query** | **Observed** | **Mr(expt)** | **Mr(calc)** | **ppm** | **Miss** | **Score** | **Expect** | **Rank** | **Unique** | **Peptide** |
|  | 10 | 356.1946 | 710.3746 | 710.3752 | -0.79 | 0 | 26 | 0.021 | 1 |  | K.FNFVGK.L |
|  | 525 | **441.2634** | **880.5123** | **880.5130** | **-0.84** | **0** | **1** | **4.2** | **3** | **U** | **R.GVPAPAITR.G** |
|  | 1415 | 547.2841 | 1092.5536 | 1092.5525 | 0.99 | 0 | 30 | 0.015 | 1 |  | K.YLPELMAEK.D |
|  | 1416 | 547.2849 | 1092.5553 | 1092.5525 | 2.54 | 0 | (18) | 0.26 | 1 |  | K.YLPELMAEK.D |
|  | 1488 | 555.2813 | 1108.5479 | 1108.5474 | 0.49 | 0 | (13) | 0.74 | 1 |  | K.YLPELMAEK.D + Oxidation (M) |
|  | 1489 | 555.2819 | 1108.5492 | 1108.5474 | 1.59 | 0 | (22) | 0.088 | 1 |  | K.YLPELMAEK.D + Oxidation (M) |
|  | 1503 | **556.2901** | **1110.5656** | **1110.5525** | **11.8** | **1** | **1** | **9.8** | **1** | **U** | **K.MSILGKGSMR.D + 2 Oxidation (M)** |
|  | 1504 | 556.2908 | 1110.5670 | 1110.5525 | 13.0 | 1 | (0) | 13 | 4 | U | K.MSILGKGSMR.D + 2 Oxidation (M) |
|  | 2851 | **732.8922** | **1463.7697** | **1463.7507** | **13.0** | **1** | **2** | **8.1** | **7** | **U** | **K.DEEKYIDVVINK.N** |

  


---

|  |  |
| --- | --- |
| **85.** | sp|P61626|LYSC\_HUMAN    **Mass:** 16982    **Score:** 37     **Matches:** 1(1)  **Sequences:** 1(1)  **emPAI:** 0.20 |
|  | Lysozyme C OS=Homo sapiens OX=9606 GN=LYZ PE=1 SV=1 |

|  |  |
| --- | --- |
|  | Check to include this hit in error tolerant search or archive report |
|  |  |

|  |  |  |  |  |  |  |  |  |  |  |  |
| --- | --- | --- | --- | --- | --- | --- | --- | --- | --- | --- | --- |
|  | **Query** | **Observed** | **Mr(expt)** | **Mr(calc)** | **ppm** | **Miss** | **Score** | **Expect** | **Rank** | **Unique** | **Peptide** |
|  | 2630 | **700.8423** | **1399.6700** | **1399.6732** | **-2.26** | **0** | **37** | **0.0021** | **1** | **U** | **R.STDYGIFQINSR.Y** |

  


---

|  |  |
| --- | --- |
| **86.** | sp|Q6KB66|K2C80\_HUMAN    **Mass:** 51007    **Score:** 37     **Matches:** 7(1)  **Sequences:** 5(1)  **emPAI:** 0.06 |
|  | Keratin, type II cytoskeletal 80 OS=Homo sapiens OX=9606 GN=KRT80 PE=1 SV=2 |

|  |  |
| --- | --- |
|  | Check to include this hit in error tolerant search or archive report |
|  |  |

|  |  |  |  |  |  |  |  |  |  |  |  |
| --- | --- | --- | --- | --- | --- | --- | --- | --- | --- | --- | --- |
|  | **Query** | **Observed** | **Mr(expt)** | **Mr(calc)** | **ppm** | **Miss** | **Score** | **Expect** | **Rank** | **Unique** | **Peptide** |
|  | 1005 | **508.7728** | **1015.5311** | **1015.5298** | **1.29** | **0** | **25** | **0.061** | **1** | **U** | **R.SEIADLNVR.I** |
|  | 1006 | 508.7732 | 1015.5318 | 1015.5298 | 2.00 | 1 | 11 | 1.6 | 3 | U | R.KLVEGEEGR.M |
|  | 1007 | 508.7740 | 1015.5334 | 1015.5298 | 3.51 | 1 | (10) | 2.1 | 3 | U | R.KLVEGEEGR.M |
|  | 2168 | **638.8644** | **1275.7143** | **1275.7187** | **-3.39** | **1** | **0** | **7.9** | **3** | **U** | **K.ALNDKFASLIGK.V** |
|  | 2193 | **642.3569** | **1282.6992** | **1282.7245** | **-19.70** | **0** | **(1)** | **8.8** | **6** | **U** | **K.LAQLEAALQQAK.Q** |
|  | 2194 | **642.3729** | **1282.7313** | **1282.7245** | **5.33** | **0** | **4** | **2.3** | **2** | **U** | **K.LAQLEAALQQAK.Q** |
|  | 3020 | **768.8621** | **1535.7096** | **1535.7103** | **-0.50** | **0** | **37** | **0.0021** | **1** | **U** | **K.TAEEQGELAFQDAK.T** |

  


---

|  |  |
| --- | --- |
| **87.** | sp|Q01844|EWS\_HUMAN    **Mass:** 68721    **Score:** 36     **Matches:** 3(1)  **Sequences:** 2(1)  **emPAI:** 0.05 |
|  | RNA-binding protein EWS OS=Homo sapiens OX=9606 GN=EWSR1 PE=1 SV=1 |

|  |  |
| --- | --- |
|  | Check to include this hit in error tolerant search or archive report |
|  |  |

|  |  |  |  |  |  |  |  |  |  |  |  |
| --- | --- | --- | --- | --- | --- | --- | --- | --- | --- | --- | --- |
|  | **Query** | **Observed** | **Mr(expt)** | **Mr(calc)** | **ppm** | **Miss** | **Score** | **Expect** | **Rank** | **Unique** | **Peptide** |
|  | 1032 | 511.7508 | 1021.4870 | 1021.4869 | 0.13 | 0 | 36 | 0.0036 | 2 | U | K.AAVEWFDGK.D |
|  | 1461 | **552.7825** | **1103.5505** | **1103.5579** | **-6.73** | **1** | **12** | **1.2** | **1** | **U** | **R.KKPPMNSMR.G + Oxidation (M)** |
|  | 1462 | **552.7854** | **1103.5562** | **1103.5579** | **-1.53** | **1** | **(5)** | **6.2** | **4** | **U** | **R.KKPPMNSMR.G + Oxidation (M)** |

  


---

|  |  |
| --- | --- |
| **88.** | sp|O00338|ST1C2\_HUMAN    **Score:** 35     **Matches:** 1(1)  **Sequences:** 1(1)  **emPAI:** 0.10 |
|  | Sulfotransferase 1C2 OS=Homo sapiens OX=9606 GN=SULT1C2 PE=1 SV=1 |

|  |  |
| --- | --- |
|  | Check to include this hit in error tolerant search or archive report |
|  |  |

|  |  |  |  |  |  |  |  |  |  |  |  |
| --- | --- | --- | --- | --- | --- | --- | --- | --- | --- | --- | --- |
|  | **Query** | **Observed** | **Mr(expt)** | **Mr(calc)** | **ppm** | **Miss** | **Score** | **Expect** | **Rank** | **Unique** | **Peptide** |
|  | 2552 | 692.3485 | 1382.6825 | 1382.6864 | -2.83 | 0 | 35 | 0.005 | 2 | U | K.SILDQSISSFMR.K |

  


---

|  |  |
| --- | --- |
| **89.** | sp|Q06830|PRDX1\_HUMAN    **Mass:** 22324    **Score:** 35     **Matches:** 2(1)  **Sequences:** 2(1)  **emPAI:** 0.15 |
|  | Peroxiredoxin-1 OS=Homo sapiens OX=9606 GN=PRDX1 PE=1 SV=1 |

|  |  |
| --- | --- |
|  | Check to include this hit in error tolerant search or archive report |
|  |  |

|  |  |  |  |  |  |  |  |  |  |  |  |
| --- | --- | --- | --- | --- | --- | --- | --- | --- | --- | --- | --- |
|  | **Query** | **Observed** | **Mr(expt)** | **Mr(calc)** | **ppm** | **Miss** | **Score** | **Expect** | **Rank** | **Unique** | **Peptide** |
|  | 1926 | **606.3403** | **1210.6661** | **1210.6670** | **-0.73** | **0** | **35** | **0.0035** | **1** | **U** | **R.QITVNDLPVGR.S** |
|  | 3246 | **819.9326** | **1637.8507** | **1637.8447** | **3.66** | **0** | **1** | **9.8** | **5** | **U** | **K.QGGLGPMNIPLVSDPK.R + Oxidation (M)** |

  


---

|  |  |
| --- | --- |
| **90.** | sp|Q86U44|MTA70\_HUMAN    **Score:** 34     **Matches:** 3(2)  **Sequences:** 2(1)  **emPAI:** 0.05 |
|  | N6-adenosine-methyltransferase catalytic subunit OS=Homo sapiens OX=9606 GN=METTL3 PE=1 SV=2 |

|  |  |
| --- | --- |
|  | Check to include this hit in error tolerant search or archive report |
|  |  |

|  |  |  |  |  |  |  |  |  |  |  |  |
| --- | --- | --- | --- | --- | --- | --- | --- | --- | --- | --- | --- |
|  | **Query** | **Observed** | **Mr(expt)** | **Mr(calc)** | **ppm** | **Miss** | **Score** | **Expect** | **Rank** | **Unique** | **Peptide** |
|  | 1006 | 508.7732 | 1015.5318 | 1015.5410 | -9.06 | 1 | 32 | 0.012 | 2 | U | K.QLDSLRER.L |
|  | 1007 | 508.7740 | 1015.5334 | 1015.5410 | -7.55 | 1 | (28) | 0.033 | 2 | U | K.QLDSLRER.L |
|  | 1626 | **570.2830** | **1138.5515** | **1138.5362** | **13.4** | **0** | **8** | **2.2** | **5** | **U** | **K.LSAMMGAVAEK.K + 2 Oxidation (M)** |

  


---

|  |  |
| --- | --- |
| **91.** | sp|P62979|RS27A\_HUMAN    **Mass:** 18296    **Score:** 34     **Matches:** 4(3)  **Sequences:** 3(3)  **emPAI:** 0.66 |
|  | Ubiquitin-40S ribosomal protein S27a OS=Homo sapiens OX=9606 GN=RPS27A PE=1 SV=2 |

|  |  |
| --- | --- |
|  | Check to include this hit in error tolerant search or archive report |
|  |  |

|  |  |  |  |  |  |  |  |  |  |  |  |
| --- | --- | --- | --- | --- | --- | --- | --- | --- | --- | --- | --- |
|  | **Query** | **Observed** | **Mr(expt)** | **Mr(calc)** | **ppm** | **Miss** | **Score** | **Expect** | **Rank** | **Unique** | **Peptide** |
|  | 105 | **383.2194** | **764.4242** | **764.4255** | **-1.71** | **0** | **27** | **0.027** | **1** | **U** | **-.MQIFVK.T** |
|  | 1350 | **541.2802** | **1080.5459** | **1080.5451** | **0.69** | **0** | **24** | **0.059** | **1** | **U** | **R.TLSDYNIQK.E** |
|  | 2985 | **762.3937** | **1522.7729** | **1522.7740** | **-0.68** | **1** | **(3)** | **6.5** | **2** | **U** | **K.IQDKEGIPPDQQR.L** |
|  | 2988 | **508.5989** | **1522.7748** | **1522.7740** | **0.56** | **1** | **27** | **0.025** | **1** | **U** | **K.IQDKEGIPPDQQR.L** |

  

|  |  |
| --- | --- |
|  | |
|  | **Proteins matching the same set of peptides:** |

|  |  |
| --- | --- |
|  | sp|P62987|RL40\_HUMAN    **Mass:** 15004    **Score:** 34     **Matches:** 4(3)  **Sequences:** 3(3) |
|  | Ubiquitin-60S ribosomal protein L40 OS=Homo sapiens OX=9606 GN=UBA52 PE=1 SV=2 |

|  |  |
| --- | --- |
|  | sp|P0CG47|UBB\_HUMAN    **Mass:** 25803    **Score:** 34     **Matches:** 4(3)  **Sequences:** 3(3) |
|  | Polyubiquitin-B OS=Homo sapiens OX=9606 GN=UBB PE=1 SV=1 |

|  |  |
| --- | --- |
|  | sp|P0CG48|UBC\_HUMAN    **Mass:** 76992    **Score:** 34     **Matches:** 4(3)  **Sequences:** 3(3) |
|  | Polyubiquitin-C OS=Homo sapiens OX=9606 GN=UBC PE=1 SV=3 |

---

|  |  |
| --- | --- |
| **92.** | sp|Q9BYE4|SPR2G\_HUMAN    **Mass:** 8779     **Score:** 34     **Matches:** 1(1)  **Sequences:** 1(1)  **emPAI:** 0.40 |
|  | Small proline-rich protein 2G OS=Homo sapiens OX=9606 GN=SPRR2G PE=3 SV=1 |

|  |  |
| --- | --- |
|  | Check to include this hit in error tolerant search or archive report |
|  |  |

|  |  |  |  |  |  |  |  |  |  |  |  |
| --- | --- | --- | --- | --- | --- | --- | --- | --- | --- | --- | --- |
|  | **Query** | **Observed** | **Mr(expt)** | **Mr(calc)** | **ppm** | **Miss** | **Score** | **Expect** | **Rank** | **Unique** | **Peptide** |
|  | 2942 | **753.3652** | **1504.7158** | **1504.7167** | **-0.59** | **0** | **34** | **0.0058** | **1** | **U** | **K.QPCQPPPVCPTPK.C** |

  

|  |  |
| --- | --- |
|  | |
|  | **Proteins matching the same set of peptides:** |

|  |  |
| --- | --- |
|  | sp|P22531|SPR2E\_HUMAN    **Mass:** 8591     **Score:** 34     **Matches:** 1(1)  **Sequences:** 1(1) |
|  | Small proline-rich protein 2E OS=Homo sapiens OX=9606 GN=SPRR2E PE=2 SV=2 |

|  |  |
| --- | --- |
|  | sp|P22532|SPR2D\_HUMAN    **Mass:** 8584     **Score:** 34     **Matches:** 1(1)  **Sequences:** 1(1) |
|  | Small proline-rich protein 2D OS=Homo sapiens OX=9606 GN=SPRR2D PE=2 SV=2 |

|  |  |
| --- | --- |
|  | sp|P35325|SPR2B\_HUMAN    **Mass:** 8597     **Score:** 34     **Matches:** 1(1)  **Sequences:** 1(1) |
|  | Small proline-rich protein 2B OS=Homo sapiens OX=9606 GN=SPRR2B PE=2 SV=1 |

|  |  |
| --- | --- |
|  | sp|P35326|SPR2A\_HUMAN    **Mass:** 8587     **Score:** 34     **Matches:** 1(1)  **Sequences:** 1(1) |
|  | Small proline-rich protein 2A OS=Homo sapiens OX=9606 GN=SPRR2A PE=1 SV=1 |

---

|  |  |
| --- | --- |
| **93.** | sp|Q5D862|FILA2\_HUMAN    **Mass:** 249296   **Score:** 33     **Matches:** 3(1)  **Sequences:** 3(1)  **emPAI:** 0.01 |
|  | Filaggrin-2 OS=Homo sapiens OX=9606 GN=FLG2 PE=1 SV=1 |

|  |  |
| --- | --- |
|  | Check to include this hit in error tolerant search or archive report |
|  |  |

|  |  |  |  |  |  |  |  |  |  |  |  |
| --- | --- | --- | --- | --- | --- | --- | --- | --- | --- | --- | --- |
|  | **Query** | **Observed** | **Mr(expt)** | **Mr(calc)** | **ppm** | **Miss** | **Score** | **Expect** | **Rank** | **Unique** | **Peptide** |
|  | 2144 | **635.3579** | **1268.7013** | **1268.7017** | **-0.31** | **0** | **33** | **0.0044** | **1** | **U** | **R.SVVTVIDVFYK.Y** |
|  | 2334 | **660.7878** | **1319.5611** | **1319.5630** | **-1.39** | **0** | **14** | **0.14** | **1** | **U** | **R.FSNSSSSNEFSK.C** |
|  | 4307 | **784.6672** | **2350.9797** | **2350.9799** | **-0.08** | **0** | **9** | **0.21** | **1** | **U** | **R.QSSYGQHGSGSSQSSGYGQYGSR.E** |

  


---

|  |  |
| --- | --- |
| **94.** | sp|Q7RTW8|OTOAN\_HUMAN    **Mass:** 129534   **Score:** 32     **Matches:** 3(1)  **Sequences:** 3(1)  **emPAI:** 0.03 |
|  | Otoancorin OS=Homo sapiens OX=9606 GN=OTOA PE=1 SV=1 |

|  |  |
| --- | --- |
|  | Check to include this hit in error tolerant search or archive report |
|  |  |

|  |  |  |  |  |  |  |  |  |  |  |  |
| --- | --- | --- | --- | --- | --- | --- | --- | --- | --- | --- | --- |
|  | **Query** | **Observed** | **Mr(expt)** | **Mr(calc)** | **ppm** | **Miss** | **Score** | **Expect** | **Rank** | **Unique** | **Peptide** |
|  | 96 | 379.7237 | 757.4328 | 757.4334 | -0.73 | 0 | 32 | 0.02 | 1 | U | K.LLEDLR.K |
|  | 2785 | **720.4099** | **1438.8053** | **1438.8143** | **-6.31** | **0** | **4** | **3.1** | **4** | **U** | **R.RPEELLSAGQLVK.G** |
|  | 4270 | **770.3876** | **2308.1409** | **2308.1741** | **-14.41** | **1** | **0** | **8.5** | **7** | **U** | **R.CFQMLNSLECVEILGKVLR.G** |

  


---

|  |  |
| --- | --- |
| **95.** | sp|Q9H2F5|EPC1\_HUMAN    **Mass:** 93975    **Score:** 31     **Matches:** 7(2)  **Sequences:** 2(1)  **emPAI:** 0.03 |
|  | Enhancer of polycomb homolog 1 OS=Homo sapiens OX=9606 GN=EPC1 PE=1 SV=1 |

|  |  |
| --- | --- |
|  | Check to include this hit in error tolerant search or archive report |
|  |  |

|  |  |  |  |  |  |  |  |  |  |  |  |
| --- | --- | --- | --- | --- | --- | --- | --- | --- | --- | --- | --- |
|  | **Query** | **Observed** | **Mr(expt)** | **Mr(calc)** | **ppm** | **Miss** | **Score** | **Expect** | **Rank** | **Unique** | **Peptide** |
|  | 833 | **487.2326** | **972.4506** | **972.4335** | **17.6** | **0** | **(16)** | **0.22** | **1** | **U** | **K.HQEAMDVK.E + Oxidation (M)** |
|  | 834 | 487.2329 | 972.4512 | 972.4335 | 18.2 | 0 | (12) | 0.54 | 1 | U | K.HQEAMDVK.E + Oxidation (M) |
|  | 835 | **487.2335** | **972.4524** | **972.4335** | **19.4** | **0** | **(25)** | **0.026** | **1** | **U** | **K.HQEAMDVK.E + Oxidation (M)** |
|  | 836 | 487.2335 | 972.4525 | 972.4335 | 19.6 | 0 | (10) | 0.77 | 3 | U | K.HQEAMDVK.E + Oxidation (M) |
|  | 837 | 487.2337 | 972.4528 | 972.4335 | 19.9 | 0 | (10) | 0.77 | 3 | U | K.HQEAMDVK.E + Oxidation (M) |
|  | 838 | **487.2337** | **972.4528** | **972.4335** | **19.9** | **0** | **28** | **0.014** | **1** | **U** | **K.HQEAMDVK.E + Oxidation (M)** |
|  | 1008 | **509.2955** | **1016.5764** | **1016.5940** | **-17.24** | **0** | **2** | **9.3** | **2** | **U** | **R.AVTILEMIK.R** |

  


---

|  |  |
| --- | --- |
| **96.** | sp|O00472|ELL2\_HUMAN    **Mass:** 72736    **Score:** 30     **Matches:** 1(1)  **Sequences:** 1(1)  **emPAI:** 0.05 |
|  | RNA polymerase II elongation factor ELL2 OS=Homo sapiens OX=9606 GN=ELL2 PE=1 SV=2 |

|  |  |
| --- | --- |
|  | Check to include this hit in error tolerant search or archive report |
|  |  |

|  |  |  |  |  |  |  |  |  |  |  |  |
| --- | --- | --- | --- | --- | --- | --- | --- | --- | --- | --- | --- |
|  | **Query** | **Observed** | **Mr(expt)** | **Mr(calc)** | **ppm** | **Miss** | **Score** | **Expect** | **Rank** | **Unique** | **Peptide** |
|  | 2461 | **679.3621** | **1356.7097** | **1356.6932** | **12.2** | **1** | **30** | **0.017** | **1** | **U** | **K.RSTPMNPANTIR.K** |

  


---

|  |  |
| --- | --- |
| **97.** | sp|O94868|FCSD2\_HUMAN    **Score:** 29     **Matches:** 4(1)  **Sequences:** 4(1)  **emPAI:** 0.04 |
|  | F-BAR and double SH3 domains protein 2 OS=Homo sapiens OX=9606 GN=FCHSD2 PE=1 SV=3 |

|  |  |
| --- | --- |
|  | Check to include this hit in error tolerant search or archive report |
|  |  |

|  |  |  |  |  |  |  |  |  |  |  |  |
| --- | --- | --- | --- | --- | --- | --- | --- | --- | --- | --- | --- |
|  | **Query** | **Observed** | **Mr(expt)** | **Mr(calc)** | **ppm** | **Miss** | **Score** | **Expect** | **Rank** | **Unique** | **Peptide** |
|  | 696 | **467.2364** | **932.4582** | **932.4716** | **-14.36** | **0** | **6** | **4.4** | **9** | **U** | **K.NFISEPAR.T** |
|  | 2264 | 651.3348 | 1300.6550 | 1300.6622 | -5.57 | 1 | 29 | 0.021 | 2 | U | R.AELEQKIDEAR.E |
|  | 3381 | **856.3821** | **1710.7496** | **1710.7189** | **18.0** | **0** | **1** | **5.1** | **3** | **U** | **K.SAMNQVMEELENER.W + 2 Oxidation (M)** |
|  | 3528 | **887.4381** | **1772.8617** | **1772.8767** | **-8.47** | **1** | **0** | **10** | **4** | **U** | **R.EYAQGMQKLASQYLK.R + Oxidation (M)** |

  


---

|  |  |
| --- | --- |
| **98.** | sp|O95715|CXL14\_HUMAN    **Mass:** 13297    **Score:** 28     **Matches:** 2(1)  **Sequences:** 1(1)  **emPAI:** 0.26 |
|  | C-X-C motif chemokine 14 OS=Homo sapiens OX=9606 GN=CXCL14 PE=1 SV=2 |

|  |  |
| --- | --- |
|  | Check to include this hit in error tolerant search or archive report |
|  |  |

|  |  |  |  |  |  |  |  |  |  |  |  |
| --- | --- | --- | --- | --- | --- | --- | --- | --- | --- | --- | --- |
|  | **Query** | **Observed** | **Mr(expt)** | **Mr(calc)** | **ppm** | **Miss** | **Score** | **Expect** | **Rank** | **Unique** | **Peptide** |
|  | 628 | **457.2640** | **912.5135** | **912.4963** | **18.8** | **1** | **(4)** | **4.7** | **2** | **U** | **R.RAPPVSMR.L** |
|  | 679 | 465.2560 | 928.4974 | 928.4913 | 6.62 | 1 | 28 | 0.032 | 1 | U | R.RAPPVSMR.L + Oxidation (M) |

  


---

|  |  |
| --- | --- |
| **99.** | sp|P47914|RL29\_HUMAN    **Mass:** 17798    **Score:** 28     **Matches:** 3(1)  **Sequences:** 2(1)  **emPAI:** 0.19 |
|  | 60S ribosomal protein L29 OS=Homo sapiens OX=9606 GN=RPL29 PE=1 SV=2 |

|  |  |
| --- | --- |
|  | Check to include this hit in error tolerant search or archive report |
|  |  |

|  |  |  |  |  |  |  |  |  |  |  |  |
| --- | --- | --- | --- | --- | --- | --- | --- | --- | --- | --- | --- |
|  | **Query** | **Observed** | **Mr(expt)** | **Mr(calc)** | **ppm** | **Miss** | **Score** | **Expect** | **Rank** | **Unique** | **Peptide** |
|  | 202 | **406.7524** | **811.4903** | **811.5028** | **-15.43** | **1** | **(1)** | **4.4** | **9** | **U** | **R.NGIKKPR.S** |
|  | 203 | **406.7539** | **811.4933** | **811.5028** | **-11.74** | **1** | **7** | **1.2** | **4** | **U** | **R.NGIKKPR.S** |
|  | 2536 | **689.3764** | **1376.7382** | **1376.7412** | **-2.13** | **0** | **28** | **0.024** | **1** | **U** | **K.AQAAAPASVPAQAPK.R** |

  


---

|  |  |
| --- | --- |
| **100.** | sp|O75191|XYLB\_HUMAN    **Score:** 27     **Matches:** 1(1)  **Sequences:** 1(1)  **emPAI:** 0.06 |
|  | Xylulose kinase OS=Homo sapiens OX=9606 GN=XYLB PE=1 SV=3 |

|  |  |
| --- | --- |
|  | Check to include this hit in error tolerant search or archive report |
|  |  |

|  |  |  |  |  |  |  |  |  |  |  |  |
| --- | --- | --- | --- | --- | --- | --- | --- | --- | --- | --- | --- |
|  | **Query** | **Observed** | **Mr(expt)** | **Mr(calc)** | **ppm** | **Miss** | **Score** | **Expect** | **Rank** | **Unique** | **Peptide** |
|  | 2358 | 663.3806 | 1324.7467 | 1324.7211 | 19.3 | 1 | 27 | 0.015 | 2 | U | K.TKILATGGASHNR.E |

  


---

|  |  |
| --- | --- |
| **101.** | sp|Q9P0W8|SPAT7\_HUMAN    **Mass:** 68190    **Score:** 27     **Matches:** 4(1)  **Sequences:** 3(1)  **emPAI:** 0.05 |
|  | Spermatogenesis-associated protein 7 OS=Homo sapiens OX=9606 GN=SPATA7 PE=1 SV=3 |

|  |  |
| --- | --- |
|  | Check to include this hit in error tolerant search or archive report |
|  |  |

|  |  |  |  |  |  |  |  |  |  |  |  |
| --- | --- | --- | --- | --- | --- | --- | --- | --- | --- | --- | --- |
|  | **Query** | **Observed** | **Mr(expt)** | **Mr(calc)** | **ppm** | **Miss** | **Score** | **Expect** | **Rank** | **Unique** | **Peptide** |
|  | 65 | **372.2240** | **742.4334** | **742.4337** | **-0.46** | **0** | **27** | **0.045** | **1** | **U** | **R.ATSVLPR.Y** |
|  | 66 | **372.2240** | **742.4334** | **742.4337** | **-0.38** | **0** | **(11)** | **1.4** | **1** | **U** | **R.ATSVLPR.Y** |
|  | 2449 | **677.8455** | **1353.6765** | **1353.7014** | **-18.40** | **1** | **2** | **10** | **4** | **U** | **R.RPRSTFPNSHR.F** |
|  | 4077 | **1065.4791** | **2128.9437** | **2128.9847** | **-19.27** | **1** | **0** | **4.6** | **10** | **U** | **K.EEMNGFSSFARSLVPSSER.L** |

  


---

|  |  |
| --- | --- |
| **102.** | sp|Q6DN72|FCRL6\_HUMAN    **Mass:** 48515    **Score:** 27     **Matches:** 3(1)  **Sequences:** 1(1)  **emPAI:** 0.07 |
|  | Fc receptor-like protein 6 OS=Homo sapiens OX=9606 GN=FCRL6 PE=1 SV=2 |

|  |  |
| --- | --- |
|  | Check to include this hit in error tolerant search or archive report |
|  |  |

|  |  |  |  |  |  |  |  |  |  |  |  |
| --- | --- | --- | --- | --- | --- | --- | --- | --- | --- | --- | --- |
|  | **Query** | **Observed** | **Mr(expt)** | **Mr(calc)** | **ppm** | **Miss** | **Score** | **Expect** | **Rank** | **Unique** | **Peptide** |
|  | 736 | **471.2267** | **940.4389** | **940.4363** | **2.79** | **0** | **(14)** | **0.35** | **1** | **U** | **K.DGHTLQDR.G** |
|  | 737 | **471.2270** | **940.4394** | **940.4363** | **3.36** | **0** | **(9)** | **1** | **1** | **U** | **K.DGHTLQDR.G** |
|  | 738 | **471.2271** | **940.4395** | **940.4363** | **3.49** | **0** | **27** | **0.019** | **1** | **U** | **K.DGHTLQDR.G** |

  


---

|  |  |
| --- | --- |
| **103.** | sp|P0C0S5|H2AZ\_HUMAN    **Mass:** 13545    **Score:** 26     **Matches:** 2(1)  **Sequences:** 1(1)  **emPAI:** 0.25 |
|  | Histone H2A.Z OS=Homo sapiens OX=9606 GN=H2AFZ PE=1 SV=2 |

|  |  |
| --- | --- |
|  | Check to include this hit in error tolerant search or archive report |
|  |  |

|  |  |  |  |  |  |  |  |  |  |  |  |
| --- | --- | --- | --- | --- | --- | --- | --- | --- | --- | --- | --- |
|  | **Query** | **Observed** | **Mr(expt)** | **Mr(calc)** | **ppm** | **Miss** | **Score** | **Expect** | **Rank** | **Unique** | **Peptide** |
|  | 745 | **472.7695** | **943.5245** | **943.5240** | **0.59** | **0** | **26** | **0.054** | **1** | **U** | **R.AGLQFPVGR.I** |
|  | 746 | **472.7698** | **943.5251** | **943.5240** | **1.18** | **0** | **(15)** | **0.7** | **1** | **U** | **R.AGLQFPVGR.I** |

  

|  |  |
| --- | --- |
|  | |
|  | **Proteins matching the same set of peptides:** |

|  |  |
| --- | --- |
|  | sp|P0C0S8|H2A1\_HUMAN    **Mass:** 14083    **Score:** 26     **Matches:** 2(1)  **Sequences:** 1(1) |
|  | Histone H2A type 1 OS=Homo sapiens OX=9606 GN=HIST1H2AG PE=1 SV=2 |

|  |  |
| --- | --- |
|  | sp|Q6FI13|H2A2A\_HUMAN    **Mass:** 14087    **Score:** 26     **Matches:** 2(1)  **Sequences:** 1(1) |
|  | Histone H2A type 2-A OS=Homo sapiens OX=9606 GN=HIST2H2AA3 PE=1 SV=3 |

|  |  |
| --- | --- |
|  | sp|Q7L7L0|H2A3\_HUMAN    **Mass:** 14113    **Score:** 26     **Matches:** 2(1)  **Sequences:** 1(1) |
|  | Histone H2A type 3 OS=Homo sapiens OX=9606 GN=HIST3H2A PE=1 SV=3 |

|  |  |
| --- | --- |
|  | sp|Q8IUE6|H2A2B\_HUMAN    **Mass:** 13987    **Score:** 26     **Matches:** 2(1)  **Sequences:** 1(1) |
|  | Histone H2A type 2-B OS=Homo sapiens OX=9606 GN=HIST2H2AB PE=1 SV=3 |

|  |  |
| --- | --- |
|  | sp|Q9BTM1|H2AJ\_HUMAN    **Mass:** 14011    **Score:** 26     **Matches:** 2(1)  **Sequences:** 1(1) |
|  | Histone H2A.J OS=Homo sapiens OX=9606 GN=H2AFJ PE=1 SV=1 |

|  |  |
| --- | --- |
|  | sp|Q71UI9|H2AV\_HUMAN    **Mass:** 13501    **Score:** 26     **Matches:** 2(1)  **Sequences:** 1(1) |
|  | Histone H2A.V OS=Homo sapiens OX=9606 GN=H2AFV PE=1 SV=3 |

|  |  |
| --- | --- |
|  | sp|Q96KK5|H2A1H\_HUMAN    **Mass:** 13898    **Score:** 26     **Matches:** 2(1)  **Sequences:** 1(1) |
|  | Histone H2A type 1-H OS=Homo sapiens OX=9606 GN=HIST1H2AH PE=1 SV=3 |

|  |  |
| --- | --- |
|  | sp|Q96QV6|H2A1A\_HUMAN    **Mass:** 14225    **Score:** 26     **Matches:** 2(1)  **Sequences:** 1(1) |
|  | Histone H2A type 1-A OS=Homo sapiens OX=9606 GN=HIST1H2AA PE=1 SV=3 |

|  |  |
| --- | --- |
|  | sp|P04908|H2A1B\_HUMAN    **Mass:** 14127    **Score:** 26     **Matches:** 2(1)  **Sequences:** 1(1) |
|  | Histone H2A type 1-B/E OS=Homo sapiens OX=9606 GN=HIST1H2AB PE=1 SV=2 |

|  |  |
| --- | --- |
|  | sp|P16104|H2AX\_HUMAN    **Mass:** 15135    **Score:** 26     **Matches:** 2(1)  **Sequences:** 1(1) |
|  | Histone H2AX OS=Homo sapiens OX=9606 GN=H2AFX PE=1 SV=2 |

|  |  |
| --- | --- |
|  | sp|Q16777|H2A2C\_HUMAN    **Mass:** 13980    **Score:** 26     **Matches:** 2(1)  **Sequences:** 1(1) |
|  | Histone H2A type 2-C OS=Homo sapiens OX=9606 GN=HIST2H2AC PE=1 SV=4 |

|  |  |
| --- | --- |
|  | sp|P20671|H2A1D\_HUMAN    **Mass:** 14099    **Score:** 26     **Matches:** 2(1)  **Sequences:** 1(1) |
|  | Histone H2A type 1-D OS=Homo sapiens OX=9606 GN=HIST1H2AD PE=1 SV=2 |

|  |  |
| --- | --- |
|  | sp|Q93077|H2A1C\_HUMAN    **Mass:** 14097    **Score:** 26     **Matches:** 2(1)  **Sequences:** 1(1) |
|  | Histone H2A type 1-C OS=Homo sapiens OX=9606 GN=HIST1H2AC PE=1 SV=3 |

|  |  |
| --- | --- |
|  | sp|Q99878|H2A1J\_HUMAN    **Mass:** 13928    **Score:** 26     **Matches:** 2(1)  **Sequences:** 1(1) |
|  | Histone H2A type 1-J OS=Homo sapiens OX=9606 GN=HIST1H2AJ PE=1 SV=3 |

---

|  |  |
| --- | --- |
| **104.** | sp|Q96Q27|ASB2\_HUMAN    **Score:** 26     **Matches:** 3(1)  **Sequences:** 1(1)  **emPAI:** 0.05 |
|  | Ankyrin repeat and SOCS box protein 2 OS=Homo sapiens OX=9606 GN=ASB2 PE=1 SV=1 |

|  |  |
| --- | --- |
|  | Check to include this hit in error tolerant search or archive report |
|  |  |

|  |  |  |  |  |  |  |  |  |  |  |  |
| --- | --- | --- | --- | --- | --- | --- | --- | --- | --- | --- | --- |
|  | **Query** | **Observed** | **Mr(expt)** | **Mr(calc)** | **ppm** | **Miss** | **Score** | **Expect** | **Rank** | **Unique** | **Peptide** |
|  | 2501 | 683.3198 | 1364.6251 | 1364.6493 | -17.76 | 1 | (14) | 0.5 | 2 | U | K.DGDEEALKTMIK.E + Oxidation (M) |
|  | 2502 | 683.3228 | 1364.6309 | 1364.6493 | -13.46 | 1 | 26 | 0.031 | 2 | U | K.DGDEEALKTMIK.E + Oxidation (M) |
|  | 2503 | 455.8852 | 1364.6337 | 1364.6493 | -11.48 | 1 | (10) | 1.3 | 4 | U | K.DGDEEALKTMIK.E + Oxidation (M) |

  


---

|  |  |
| --- | --- |
| **105.** | sp|P12036|NFH\_HUMAN    **Mass:** 112639   **Score:** 26     **Matches:** 5(0)  **Sequences:** 4(0)  **emPAI:** 0.06 |
|  | Neurofilament heavy polypeptide OS=Homo sapiens OX=9606 GN=NEFH PE=1 SV=4 |

|  |  |
| --- | --- |
|  | Check to include this hit in error tolerant search or archive report |
|  |  |

|  |  |  |  |  |  |  |  |  |  |  |  |
| --- | --- | --- | --- | --- | --- | --- | --- | --- | --- | --- | --- |
|  | **Query** | **Observed** | **Mr(expt)** | **Mr(calc)** | **ppm** | **Miss** | **Score** | **Expect** | **Rank** | **Unique** | **Peptide** |
|  | 965 | 503.2367 | 1004.4589 | 1004.4597 | -0.78 | 0 | 22 | 0.08 | 1 |  | K.LLEGEECR.I |
|  | 1005 | 508.7728 | 1015.5311 | 1015.5298 | 1.30 | 0 | 4 | 7.6 | 5 | U | R.SLEGEAAALR.Q |
|  | 1516 | **557.8273** | **1113.6400** | **1113.6393** | **0.58** | **1** | **5** | **3.5** | **4** | **U** | **K.TLSKEPSKPK.A** |
|  | 1604 | 567.2844 | 1132.5542 | 1132.5546 | -0.40 | 1 | 20 | 0.17 | 1 |  | R.KLLEGEECR.I |
|  | 1605 | 378.5254 | 1132.5543 | 1132.5546 | -0.33 | 1 | (18) | 0.29 | 1 |  | R.KLLEGEECR.I |

  


---

|  |  |
| --- | --- |
| **106.** | sp|Q8TER0|SNED1\_HUMAN    **Mass:** 158206   **Score:** 25     **Matches:** 4(1)  **Sequences:** 3(1)  **emPAI:** 0.02 |
|  | Sushi, nidogen and EGF-like domain-containing protein 1 OS=Homo sapiens OX=9606 GN=SNED1 PE=2 SV=2 |

|  |  |
| --- | --- |
|  | Check to include this hit in error tolerant search or archive report |
|  |  |

|  |  |  |  |  |  |  |  |  |  |  |  |
| --- | --- | --- | --- | --- | --- | --- | --- | --- | --- | --- | --- |
|  | **Query** | **Observed** | **Mr(expt)** | **Mr(calc)** | **ppm** | **Miss** | **Score** | **Expect** | **Rank** | **Unique** | **Peptide** |
|  | 192 | 405.2236 | 808.4326 | 808.4443 | -14.47 | 0 | (13) | 0.69 | 3 | U | K.LASYTVR.D |
|  | 193 | 405.2237 | 808.4329 | 808.4443 | -14.10 | 0 | 25 | 0.042 | 2 | U | K.LASYTVR.D |
|  | 2193 | 642.3569 | 1282.6992 | 1282.7034 | -3.25 | 1 | 0 | 10 | 10 | U | R.AYNISVFSVKR.N |
|  | 3700 | **922.4251** | **1842.8357** | **1842.8570** | **-11.59** | **1** | **11** | **0.84** | **1** | **U** | **R.AGDVYYREATDPAMLR.R + Oxidation (M)** |

  


---

|  |  |
| --- | --- |
| **107.** | sp|P22079|PERL\_HUMAN    **Mass:** 81149    **Score:** 25     **Matches:** 2(1)  **Sequences:** 2(1)  **emPAI:** 0.04 |
|  | Lactoperoxidase OS=Homo sapiens OX=9606 GN=LPO PE=1 SV=2 |

|  |  |
| --- | --- |
|  | Check to include this hit in error tolerant search or archive report |
|  |  |

|  |  |  |  |  |  |  |  |  |  |  |  |
| --- | --- | --- | --- | --- | --- | --- | --- | --- | --- | --- | --- |
|  | **Query** | **Observed** | **Mr(expt)** | **Mr(calc)** | **ppm** | **Miss** | **Score** | **Expect** | **Rank** | **Unique** | **Peptide** |
|  | 1937 | **405.5325** | **1213.5757** | **1213.5761** | **-0.36** | **1** | **0** | **9.6** | **4** | **U** | **K.DSLQKMSFSR.L + Oxidation (M)** |
|  | 2716 | **712.8428** | **1423.6710** | **1423.6977** | **-18.75** | **1** | **25** | **0.045** | **1** | **U** | **R.LKTAMSSETPTSR.Q + Oxidation (M)** |

  


---

|  |  |
| --- | --- |
| **108.** | sp|Q03989|ARI5A\_HUMAN    **Mass:** 64718    **Score:** 24     **Matches:** 4(2)  **Sequences:** 3(1)  **emPAI:** 0.05 |
|  | AT-rich interactive domain-containing protein 5A OS=Homo sapiens OX=9606 GN=ARID5A PE=1 SV=2 |

|  |  |
| --- | --- |
|  | Check to include this hit in error tolerant search or archive report |
|  |  |

|  |  |  |  |  |  |  |  |  |  |  |  |
| --- | --- | --- | --- | --- | --- | --- | --- | --- | --- | --- | --- |
|  | **Query** | **Observed** | **Mr(expt)** | **Mr(calc)** | **ppm** | **Miss** | **Score** | **Expect** | **Rank** | **Unique** | **Peptide** |
|  | 1870 | 599.7648 | 1197.5150 | 1197.5192 | -3.51 | 1 | 1 | 4.7 | 2 | U | R.MDQMMPGKTK.A + 2 Oxidation (M) |
|  | 2007 | 617.8431 | 1233.6716 | 1233.6830 | -9.20 | 1 | (24) | 0.046 | 2 | U | K.LGAYELVTGRR.L |
|  | 2008 | 617.8431 | 1233.6716 | 1233.6830 | -9.20 | 1 | 24 | 0.046 | 2 | U | K.LGAYELVTGRR.L |
|  | 3737 | **622.6339** | **1864.8797** | **1864.9167** | **-19.80** | **0** | **17** | **0.25** | **1** | **U** | **K.QSTEGDALDPPASPKPAGK.Q** |

  


---

|  |  |
| --- | --- |
| **109.** | sp|P23490|LORI\_HUMAN    **Mass:** 26828    **Score:** 24     **Matches:** 2(1)  **Sequences:** 2(1)  **emPAI:** 0.12 |
|  | Loricrin OS=Homo sapiens OX=9606 GN=LOR PE=1 SV=2 |

|  |  |
| --- | --- |
|  | Check to include this hit in error tolerant search or archive report |
|  |  |

|  |  |  |  |  |  |  |  |  |  |  |  |
| --- | --- | --- | --- | --- | --- | --- | --- | --- | --- | --- | --- |
|  | **Query** | **Observed** | **Mr(expt)** | **Mr(calc)** | **ppm** | **Miss** | **Score** | **Expect** | **Rank** | **Unique** | **Peptide** |
|  | 631 | **457.7402** | **913.4658** | **913.4658** | **0.10** | **0** | **24** | **0.046** | **1** | **U** | **K.QAPTWPSK.-** |
|  | 2505 | **455.8967** | **1364.6682** | **1364.6759** | **-5.62** | **0** | **2** | **11** | **10** | **U** | **K.QPTPQPPVDCVK.T** |

  


---

|  |  |
| --- | --- |
| **110.** | sp|Q15517|CDSN\_HUMAN    **Mass:** 52288    **Score:** 24     **Matches:** 1(1)  **Sequences:** 1(1)  **emPAI:** 0.06 |
|  | Corneodesmosin OS=Homo sapiens OX=9606 GN=CDSN PE=1 SV=3 |

|  |  |
| --- | --- |
|  | Check to include this hit in error tolerant search or archive report |
|  |  |

|  |  |  |  |  |  |  |  |  |  |  |  |
| --- | --- | --- | --- | --- | --- | --- | --- | --- | --- | --- | --- |
|  | **Query** | **Observed** | **Mr(expt)** | **Mr(calc)** | **ppm** | **Miss** | **Score** | **Expect** | **Rank** | **Unique** | **Peptide** |
|  | 3271 | **827.9233** | **1653.8321** | **1653.8362** | **-2.49** | **0** | **24** | **0.051** | **1** | **U** | **K.GSPGVPSFAAGPPISEGK.Y** |

  


---

|  |  |
| --- | --- |
| **111.** | sp|Q08174|PCDH1\_HUMAN    **Mass:** 115015   **Score:** 24     **Matches:** 2(0)  **Sequences:** 2(0)  **emPAI:** 0.03 |
|  | Protocadherin-1 OS=Homo sapiens OX=9606 GN=PCDH1 PE=1 SV=2 |

|  |  |
| --- | --- |
|  | Check to include this hit in error tolerant search or archive report |
|  |  |

|  |  |  |  |  |  |  |  |  |  |  |  |
| --- | --- | --- | --- | --- | --- | --- | --- | --- | --- | --- | --- |
|  | **Query** | **Observed** | **Mr(expt)** | **Mr(calc)** | **ppm** | **Miss** | **Score** | **Expect** | **Rank** | **Unique** | **Peptide** |
|  | 2539 | 460.5807 | 1378.7202 | 1378.7092 | 7.93 | 1 | 2 | 11 | 5 | U | R.ESYELKVVAADR.G |
|  | 2757 | **717.3919** | **1432.7693** | **1432.7562** | **9.15** | **1** | **24** | **0.055** | **1** | **U** | **K.ETKDLYAPKPSGK.A** |

  


---

|  |  |
| --- | --- |
| **112.** | sp|Q9HCF6|TRPM3\_HUMAN    **Score:** 22     **Matches:** 2(1)  **Sequences:** 2(1)  **emPAI:** 0.02 |
|  | Transient receptor potential cation channel subfamily M member 3 OS=Homo sapiens OX=9606 GN=TRPM3 PE=2 SV=4 |

|  |  |
| --- | --- |
|  | Check to include this hit in error tolerant search or archive report |
|  |  |

|  |  |  |  |  |  |  |  |  |  |  |  |
| --- | --- | --- | --- | --- | --- | --- | --- | --- | --- | --- | --- |
|  | **Query** | **Observed** | **Mr(expt)** | **Mr(calc)** | **ppm** | **Miss** | **Score** | **Expect** | **Rank** | **Unique** | **Peptide** |
|  | 1592 | 566.2586 | 1130.5027 | 1130.5244 | -19.22 | 0 | 22 | 0.039 | 2 | U | R.TSAFQSFESK.H |
|  | 3673 | **914.4072** | **1826.7999** | **1826.8120** | **-6.62** | **0** | **0** | **4.4** | **3** | **U** | **K.MALFFWQHGEEAMAK.A + 2 Oxidation (M)** |

  


---

|  |  |
| --- | --- |
| **113.** | sp|Q02880|TOP2B\_HUMAN    **Mass:** 184122   **Score:** 22     **Matches:** 3(1)  **Sequences:** 3(1)  **emPAI:** 0.02 |
|  | DNA topoisomerase 2-beta OS=Homo sapiens OX=9606 GN=TOP2B PE=1 SV=3 |

|  |  |
| --- | --- |
|  | Check to include this hit in error tolerant search or archive report |
|  |  |

|  |  |  |  |  |  |  |  |  |  |  |  |
| --- | --- | --- | --- | --- | --- | --- | --- | --- | --- | --- | --- |
|  | **Query** | **Observed** | **Mr(expt)** | **Mr(calc)** | **ppm** | **Miss** | **Score** | **Expect** | **Rank** | **Unique** | **Peptide** |
|  | 1 | 350.7339 | 699.4532 | 699.4531 | 0.15 | 0 | 22 | 0.041 | 2 | U | K.LIEVVK.K |
|  | 653 | **459.7558** | **917.4970** | **917.5004** | **-3.68** | **0** | **2** | **11** | **4** | **U** | **K.DIVALMTR.R** |
|  | 1696 | **580.3505** | **1158.6865** | **1158.6761** | **8.96** | **0** | **0** | **2.8** | **1** | **U** | **K.AGVSVKPFQVK.N** |

  


---

|  |  |
| --- | --- |
| **114.** | sp|Q5VX52|SPAT1\_HUMAN    **Score:** 22     **Matches:** 1(1)  **Sequences:** 1(1)  **emPAI:** 0.06 |
|  | Spermatogenesis-associated protein 1 OS=Homo sapiens OX=9606 GN=SPATA1 PE=2 SV=4 |

|  |  |
| --- | --- |
|  | Check to include this hit in error tolerant search or archive report |
|  |  |

|  |  |  |  |  |  |  |  |  |  |  |  |
| --- | --- | --- | --- | --- | --- | --- | --- | --- | --- | --- | --- |
|  | **Query** | **Observed** | **Mr(expt)** | **Mr(calc)** | **ppm** | **Miss** | **Score** | **Expect** | **Rank** | **Unique** | **Peptide** |
|  | 1 | 350.7339 | 699.4532 | 699.4531 | 0.15 | 0 | 22 | 0.041 | 2 | U | K.LIVEVK.M |

  


---

|  |  |
| --- | --- |
| **115.** | sp|P20674|COX5A\_HUMAN    **Score:** 22     **Matches:** 1(1)  **Sequences:** 1(1)  **emPAI:** 0.20 |
|  | Cytochrome c oxidase subunit 5A, mitochondrial OS=Homo sapiens OX=9606 GN=COX5A PE=1 SV=2 |

|  |  |
| --- | --- |
|  | Check to include this hit in error tolerant search or archive report |
|  |  |

|  |  |  |  |  |  |  |  |  |  |  |  |
| --- | --- | --- | --- | --- | --- | --- | --- | --- | --- | --- | --- |
|  | **Query** | **Observed** | **Mr(expt)** | **Mr(calc)** | **ppm** | **Miss** | **Score** | **Expect** | **Rank** | **Unique** | **Peptide** |
|  | 1 | 350.7339 | 699.4532 | 699.4531 | 0.15 | 0 | 22 | 0.041 | 2 | U | R.ILEVVK.D |

  


---

|  |  |
| --- | --- |
| **116.** | sp|Q3KNS1|PTHD3\_HUMAN    **Mass:** 87842    **Score:** 22     **Matches:** 5(0)  **Sequences:** 2(0)  **emPAI:** 0.04 |
|  | Patched domain-containing protein 3 OS=Homo sapiens OX=9606 GN=PTCHD3 PE=1 SV=3 |

|  |  |
| --- | --- |
|  | Check to include this hit in error tolerant search or archive report |
|  |  |

|  |  |  |  |  |  |  |  |  |  |  |  |
| --- | --- | --- | --- | --- | --- | --- | --- | --- | --- | --- | --- |
|  | **Query** | **Observed** | **Mr(expt)** | **Mr(calc)** | **ppm** | **Miss** | **Score** | **Expect** | **Rank** | **Unique** | **Peptide** |
|  | 702 | **467.3012** | **932.5877** | **932.5729** | **15.9** | **1** | **7** | **0.61** | **1** | **U** | **R.VMVIVTKK.V + Oxidation (M)** |
|  | 1891 | **601.8041** | **1201.5936** | **1201.6051** | **-9.55** | **1** | **(20)** | **0.14** | **1** | **U** | **K.TNLADDIRER.M** |
|  | 1892 | **601.8041** | **1201.5936** | **1201.6051** | **-9.55** | **1** | **22** | **0.095** | **1** | **U** | **K.TNLADDIRER.M** |
|  | 1893 | **601.8049** | **1201.5952** | **1201.6051** | **-8.24** | **1** | **(14)** | **0.66** | **1** | **U** | **K.TNLADDIRER.M** |
|  | 1895 | **601.8052** | **1201.5959** | **1201.6051** | **-7.62** | **1** | **(16)** | **0.36** | **1** | **U** | **K.TNLADDIRER.M** |

  


---

|  |  |
| --- | --- |
| **117.** | sp|Q14192|FHL2\_HUMAN    **Mass:** 34166    **Score:** 21     **Matches:** 1(0)  **Sequences:** 1(0)  **emPAI:** 0.10 |
|  | Four and a half LIM domains protein 2 OS=Homo sapiens OX=9606 GN=FHL2 PE=1 SV=3 |

|  |  |
| --- | --- |
|  | Check to include this hit in error tolerant search or archive report |
|  |  |

|  |  |  |  |  |  |  |  |  |  |  |  |
| --- | --- | --- | --- | --- | --- | --- | --- | --- | --- | --- | --- |
|  | **Query** | **Observed** | **Mr(expt)** | **Mr(calc)** | **ppm** | **Miss** | **Score** | **Expect** | **Rank** | **Unique** | **Peptide** |
|  | 1836 | 596.8389 | 1191.6633 | 1191.6612 | 1.79 | 0 | 21 | 0.074 | 1 | U | K.KPITTGGVTYR.E |

  


---

|  |  |
| --- | --- |
| **118.** | sp|Q8WXE9|STON2\_HUMAN    **Mass:** 101843   **Score:** 21     **Matches:** 2(1)  **Sequences:** 2(1)  **emPAI:** 0.03 |
|  | Stonin-2 OS=Homo sapiens OX=9606 GN=STON2 PE=1 SV=1 |

|  |  |
| --- | --- |
|  | Check to include this hit in error tolerant search or archive report |
|  |  |

|  |  |  |  |  |  |  |  |  |  |  |  |
| --- | --- | --- | --- | --- | --- | --- | --- | --- | --- | --- | --- |
|  | **Query** | **Observed** | **Mr(expt)** | **Mr(calc)** | **ppm** | **Miss** | **Score** | **Expect** | **Rank** | **Unique** | **Peptide** |
|  | 401 | 426.2177 | 850.4208 | 850.4331 | -14.41 | 1 | 4 | 6.8 | 9 | U | K.KNIMSSR.H + Oxidation (M) |
|  | 884 | **492.8078** | **983.6011** | **983.6015** | **-0.42** | **0** | **21** | **0.057** | **1** | **U** | **R.LGLNDILVK.G** |

  


---

|  |  |
| --- | --- |
| **119.** | sp|P61313|RL15\_HUMAN    **Mass:** 24245    **Score:** 21     **Matches:** 1(0)  **Sequences:** 1(0)  **emPAI:** 0.14 |
|  | 60S ribosomal protein L15 OS=Homo sapiens OX=9606 GN=RPL15 PE=1 SV=2 |

|  |  |
| --- | --- |
|  | Check to include this hit in error tolerant search or archive report |
|  |  |

|  |  |  |  |  |  |  |  |  |  |  |  |
| --- | --- | --- | --- | --- | --- | --- | --- | --- | --- | --- | --- |
|  | **Query** | **Observed** | **Mr(expt)** | **Mr(calc)** | **ppm** | **Miss** | **Score** | **Expect** | **Rank** | **Unique** | **Peptide** |
|  | 1010 | **509.7618** | **1017.5090** | **1017.5091** | **-0.06** | **0** | **21** | **0.16** | **1** | **U** | **R.SLQSVAEER.A** |

  


---

|  |  |
| --- | --- |
| **120.** | sp|Q9BZF1|OSBL8\_HUMAN    **Mass:** 101759   **Score:** 21     **Matches:** 4(0)  **Sequences:** 2(0)  **emPAI:** 0.03 |
|  | Oxysterol-binding protein-related protein 8 OS=Homo sapiens OX=9606 GN=OSBPL8 PE=1 SV=3 |

|  |  |
| --- | --- |
|  | Check to include this hit in error tolerant search or archive report |
|  |  |

|  |  |  |  |  |  |  |  |  |  |  |  |
| --- | --- | --- | --- | --- | --- | --- | --- | --- | --- | --- | --- |
|  | **Query** | **Observed** | **Mr(expt)** | **Mr(calc)** | **ppm** | **Miss** | **Score** | **Expect** | **Rank** | **Unique** | **Peptide** |
|  | 2611 | 698.3697 | 1394.7248 | 1394.7228 | 1.47 | 1 | (7) | 2.8 | 3 | U | R.KDGFCLSGSILAK.S |
|  | 2612 | 465.9157 | 1394.7252 | 1394.7228 | 1.75 | 1 | (4) | 4.9 | 5 | U | R.KDGFCLSGSILAK.S |
|  | 2613 | 465.9161 | 1394.7265 | 1394.7228 | 2.67 | 1 | 21 | 0.098 | 1 | U | R.KDGFCLSGSILAK.S |
|  | 3430 | **434.1883** | **1732.7239** | **1732.6895** | **19.9** | **0** | **2** | **1.3** | **2** | **U** | **R.GEDYVMTMPYAHCK.G + 2 Oxidation (M)** |

  


---

|  |  |
| --- | --- |
| **121.** | sp|O95573|ACSL3\_HUMAN    **Mass:** 81338    **Score:** 21     **Matches:** 5(1)  **Sequences:** 2(1)  **emPAI:** 0.04 |
|  | Long-chain-fatty-acid--CoA ligase 3 OS=Homo sapiens OX=9606 GN=ACSL3 PE=1 SV=3 |

|  |  |
| --- | --- |
|  | Check to include this hit in error tolerant search or archive report |
|  |  |

|  |  |  |  |  |  |  |  |  |  |  |  |
| --- | --- | --- | --- | --- | --- | --- | --- | --- | --- | --- | --- |
|  | **Query** | **Observed** | **Mr(expt)** | **Mr(calc)** | **ppm** | **Miss** | **Score** | **Expect** | **Rank** | **Unique** | **Peptide** |
|  | 567 | **449.7719** | **897.5293** | **897.5284** | **1.00** | **0** | **(1)** | **5** | **4** | **U** | **K.DIVSLVPR.L** |
|  | 568 | **449.7769** | **897.5393** | **897.5284** | **12.2** | **0** | **(12)** | **0.35** | **2** | **U** | **K.DIVSLVPR.L** |
|  | 569 | **449.7770** | **897.5395** | **897.5284** | **12.4** | **0** | **(11)** | **0.53** | **2** | **U** | **K.DIVSLVPR.L** |
|  | 570 | **449.7770** | **897.5395** | **897.5284** | **12.4** | **0** | **21** | **0.053** | **1** | **U** | **K.DIVSLVPR.L** |
|  | 1714 | **582.8174** | **1163.6202** | **1163.6186** | **1.36** | **0** | **4** | **4.8** | **1** |  | **K.LQAGEYVSLGK.V** |

  


---

|  |  |
| --- | --- |
| **122.** | sp|Q76FK4|NOL8\_HUMAN    **Mass:** 132448   **Score:** 20     **Matches:** 5(0)  **Sequences:** 5(0)  **emPAI:** 0.02 |
|  | Nucleolar protein 8 OS=Homo sapiens OX=9606 GN=NOL8 PE=1 SV=1 |

|  |  |
| --- | --- |
|  | Check to include this hit in error tolerant search or archive report |
|  |  |

|  |  |  |  |  |  |  |  |  |  |  |  |
| --- | --- | --- | --- | --- | --- | --- | --- | --- | --- | --- | --- |
|  | **Query** | **Observed** | **Mr(expt)** | **Mr(calc)** | **ppm** | **Miss** | **Score** | **Expect** | **Rank** | **Unique** | **Peptide** |
|  | 276 | **417.2506** | **832.4866** | **832.5018** | **-18.23** | **1** | **1** | **6** | **7** | **U** | **K.KSLSLSAK.T** |
|  | 926 | 498.2599 | 994.5052 | 994.4940 | 11.3 | 1 | 1 | 13 | 9 | U | K.KCMSVLNK.T + Oxidation (M) |
|  | 2103 | **629.8236** | **1257.6327** | **1257.6452** | **-9.97** | **1** | **3** | **8** | **3** | **U** | **R.EEAEKLPEVSK.E** |
|  | 3171 | **801.8321** | **1601.6497** | **1601.6767** | **-16.81** | **0** | **4** | **0.79** | **1** | **U** | **R.EYDSGDTDEIIAMK.K + Oxidation (M)** |
|  | 3185 | **805.8778** | **1609.7411** | **1609.7366** | **2.78** | **0** | **20** | **0.1** | **1** | **U** | **K.DESSTGSLAMSTRPR.R + Oxidation (M)** |

  


---

|  |  |
| --- | --- |
| **123.** | sp|O43291|SPIT2\_HUMAN    **Mass:** 28951    **Score:** 20     **Matches:** 1(1)  **Sequences:** 1(1)  **emPAI:** 0.11 |
|  | Kunitz-type protease inhibitor 2 OS=Homo sapiens OX=9606 GN=SPINT2 PE=1 SV=2 |

|  |  |
| --- | --- |
|  | Check to include this hit in error tolerant search or archive report |
|  |  |

|  |  |  |  |  |  |  |  |  |  |  |  |
| --- | --- | --- | --- | --- | --- | --- | --- | --- | --- | --- | --- |
|  | **Query** | **Observed** | **Mr(expt)** | **Mr(calc)** | **ppm** | **Miss** | **Score** | **Expect** | **Rank** | **Unique** | **Peptide** |
|  | 3002 | **766.3204** | **1530.6263** | **1530.6555** | **-19.05** | **1** | **20** | **0.017** | **1** | **U** | **K.NSYRSEEACMLR.C + Oxidation (M)** |

  


---

|  |  |
| --- | --- |
| **124.** | sp|Q9BYB0|SHAN3\_HUMAN    **Mass:** 185125   **Score:** 20     **Matches:** 6(0)  **Sequences:** 4(0)  **emPAI:** 0.02 |
|  | SH3 and multiple ankyrin repeat domains protein 3 OS=Homo sapiens OX=9606 GN=SHANK3 PE=1 SV=3 |

|  |  |
| --- | --- |
|  | Check to include this hit in error tolerant search or archive report |
|  |  |

|  |  |  |  |  |  |  |  |  |  |  |  |
| --- | --- | --- | --- | --- | --- | --- | --- | --- | --- | --- | --- |
|  | **Query** | **Observed** | **Mr(expt)** | **Mr(calc)** | **ppm** | **Miss** | **Score** | **Expect** | **Rank** | **Unique** | **Peptide** |
|  | 1056 | **514.2802** | **1026.5457** | **1026.5458** | **-0.07** | **0** | **1** | **8.4** | **2** | **U** | **M.DGPGASAVVVR.V** |
|  | 1059 | **514.2805** | **1026.5465** | **1026.5458** | **0.65** | **0** | **(1)** | **8.6** | **7** | **U** | **M.DGPGASAVVVR.V** |
|  | 1240 | **529.7756** | **1057.5366** | **1057.5193** | **16.4** | **0** | **(18)** | **0.4** | **1** | **U** | **K.LWGDPVESR.G** |
|  | 1241 | **529.7771** | **1057.5396** | **1057.5193** | **19.3** | **0** | **20** | **0.26** | **1** | **U** | **K.LWGDPVESR.G** |
|  | 1764 | 587.8317 | 1173.6488 | 1173.6717 | -19.54 | 1 | 0 | 13 | 8 | U | R.APSTTLTLRSK.S |
|  | 3204 | **541.2728** | **1620.7965** | **1620.8141** | **-10.85** | **1** | **4** | **5.1** | **1** | **U** | **K.SMTAELEELASIRR.R + Oxidation (M)** |

  


---

|  |  |
| --- | --- |
| **125.** | sp|Q96P71|NECA3\_HUMAN    **Mass:** 44778    **Score:** 19     **Matches:** 2(0)  **Sequences:** 1(0)  **emPAI:** 0.07 |
|  | N-terminal EF-hand calcium-binding protein 3 OS=Homo sapiens OX=9606 GN=NECAB3 PE=1 SV=2 |

|  |  |
| --- | --- |
|  | Check to include this hit in error tolerant search or archive report |
|  |  |

|  |  |  |  |  |  |  |  |  |  |  |  |
| --- | --- | --- | --- | --- | --- | --- | --- | --- | --- | --- | --- |
|  | **Query** | **Observed** | **Mr(expt)** | **Mr(calc)** | **ppm** | **Miss** | **Score** | **Expect** | **Rank** | **Unique** | **Peptide** |
|  | 902 | **495.7659** | **989.5172** | **989.5215** | **-4.34** | **0** | **(12)** | **1.5** | **1** | **U** | **R.AVLAAMDATK.L** |
|  | 903 | **495.7664** | **989.5182** | **989.5215** | **-3.41** | **0** | **19** | **0.25** | **1** | **U** | **R.AVLAAMDATK.L** |

  


---

|  |  |
| --- | --- |
| **126.** | sp|Q08554|DSC1\_HUMAN    **Mass:** 101406   **Score:** 19     **Matches:** 2(0)  **Sequences:** 1(0)  **emPAI:** 0.03 |
|  | Desmocollin-1 OS=Homo sapiens OX=9606 GN=DSC1 PE=1 SV=2 |

|  |  |
| --- | --- |
|  | Check to include this hit in error tolerant search or archive report |
|  |  |

|  |  |  |  |  |  |  |  |  |  |  |  |
| --- | --- | --- | --- | --- | --- | --- | --- | --- | --- | --- | --- |
|  | **Query** | **Observed** | **Mr(expt)** | **Mr(calc)** | **ppm** | **Miss** | **Score** | **Expect** | **Rank** | **Unique** | **Peptide** |
|  | 2877 | **739.8644** | **1477.7143** | **1477.7161** | **-1.22** | **0** | **(13)** | **0.7** | **1** | **U** | **K.VQDQDLPNTPHSK.A** |
|  | 2878 | **493.5802** | **1477.7188** | **1477.7161** | **1.79** | **0** | **19** | **0.18** | **1** | **U** | **K.VQDQDLPNTPHSK.A** |

  


---

|  |  |
| --- | --- |
| **127.** | sp|O94979|SC31A\_HUMAN    **Mass:** 133900   **Score:** 19     **Matches:** 2(1)  **Sequences:** 2(1)  **emPAI:** 0.02 |
|  | Protein transport protein Sec31A OS=Homo sapiens OX=9606 GN=SEC31A PE=1 SV=3 |

|  |  |
| --- | --- |
|  | Check to include this hit in error tolerant search or archive report |
|  |  |

|  |  |  |  |  |  |  |  |  |  |  |  |
| --- | --- | --- | --- | --- | --- | --- | --- | --- | --- | --- | --- |
|  | **Query** | **Observed** | **Mr(expt)** | **Mr(calc)** | **ppm** | **Miss** | **Score** | **Expect** | **Rank** | **Unique** | **Peptide** |
|  | 2324 | **659.8892** | **1317.7639** | **1317.7479** | **12.2** | **1** | **19** | **0.052** | **1** | **U** | **R.LITAVVMKNWK.E + Oxidation (M)** |
|  | 4095 | **721.0424** | **2160.1053** | **2160.0959** | **4.34** | **0** | **1** | **8.2** | **7** | **U** | **R.GILAIAWSMADPELLLSCGK.D + Oxidation (M)** |

  


---

|  |  |
| --- | --- |
| **128.** | sp|Q96QD5|DEPD7\_HUMAN    **Mass:** 58615    **Score:** 19     **Matches:** 3(0)  **Sequences:** 1(0)  **emPAI:** 0.06 |
|  | DEP domain-containing protein 7 OS=Homo sapiens OX=9606 GN=DEPDC7 PE=2 SV=1 |

|  |  |
| --- | --- |
|  | Check to include this hit in error tolerant search or archive report |
|  |  |

|  |  |  |  |  |  |  |  |  |  |  |  |
| --- | --- | --- | --- | --- | --- | --- | --- | --- | --- | --- | --- |
|  | **Query** | **Observed** | **Mr(expt)** | **Mr(calc)** | **ppm** | **Miss** | **Score** | **Expect** | **Rank** | **Unique** | **Peptide** |
|  | 1327 | **539.2640** | **1076.5134** | **1076.5284** | **-13.95** | **1** | **(1)** | **13** | **1** | **U** | **K.ESDNRMVVK.R** |
|  | 1328 | **539.2642** | **1076.5138** | **1076.5284** | **-13.62** | **1** | **19** | **0.22** | **1** | **U** | **K.ESDNRMVVK.R** |
|  | 1329 | **539.2821** | **1076.5496** | **1076.5284** | **19.7** | **1** | **(7)** | **3.5** | **1** | **U** | **K.ESDNRMVVK.R** |

  


---

|  |  |
| --- | --- |
| **129.** | sp|Q9H5K3|SG196\_HUMAN    **Mass:** 40480    **Score:** 18     **Matches:** 2(0)  **Sequences:** 1(0)  **emPAI:** 0.08 |
|  | Protein O-mannose kinase OS=Homo sapiens OX=9606 GN=POMK PE=1 SV=1 |

|  |  |
| --- | --- |
|  | Check to include this hit in error tolerant search or archive report |
|  |  |

|  |  |  |  |  |  |  |  |  |  |  |  |
| --- | --- | --- | --- | --- | --- | --- | --- | --- | --- | --- | --- |
|  | **Query** | **Observed** | **Mr(expt)** | **Mr(calc)** | **ppm** | **Miss** | **Score** | **Expect** | **Rank** | **Unique** | **Peptide** |
|  | 2379 | **668.3577** | **1334.7008** | **1334.7115** | **-8.05** | **0** | **18** | **0.24** | **1** | **U** | **K.VALSQLTSLEMK.D + Oxidation (M)** |
|  | 2380 | **668.3581** | **1334.7016** | **1334.7115** | **-7.42** | **0** | **(14)** | **0.6** | **1** | **U** | **K.VALSQLTSLEMK.D + Oxidation (M)** |

  


---

|  |  |
| --- | --- |
| **130.** | sp|Q5H9J7|BEX5\_HUMAN    **Mass:** 12651    **Score:** 18     **Matches:** 3(0)  **Sequences:** 1(0)  **emPAI:** 0.27 |
|  | Protein BEX5 OS=Homo sapiens OX=9606 GN=BEX5 PE=1 SV=1 |

|  |  |
| --- | --- |
|  | Check to include this hit in error tolerant search or archive report |
|  |  |

|  |  |  |  |  |  |  |  |  |  |  |  |
| --- | --- | --- | --- | --- | --- | --- | --- | --- | --- | --- | --- |
|  | **Query** | **Observed** | **Mr(expt)** | **Mr(calc)** | **ppm** | **Miss** | **Score** | **Expect** | **Rank** | **Unique** | **Peptide** |
|  | 4233 | **761.3857** | **2281.1354** | **2281.0975** | **16.6** | **0** | **(7)** | **2.5** | **1** | **U** | **K.APVQNEAPALGGGEYQEPGGNVK.G** |
|  | 4234 | **761.3867** | **2281.1383** | **2281.0975** | **17.9** | **0** | **18** | **0.19** | **1** | **U** | **K.APVQNEAPALGGGEYQEPGGNVK.G** |
|  | 4235 | 761.3872 | 2281.1398 | 2281.0975 | 18.6 | 0 | (2) | 6.5 | 5 | U | K.APVQNEAPALGGGEYQEPGGNVK.G |

  


---

|  |  |
| --- | --- |
| **131.** | sp|P0C874|S31D3\_HUMAN    **Mass:** 103380   **Score:** 18     **Matches:** 8(0)  **Sequences:** 1(0)  **emPAI:** 0.03 |
|  | Spermatogenesis-associated protein 31D3 OS=Homo sapiens OX=9606 GN=SPATA31D3 PE=2 SV=1 |

|  |  |
| --- | --- |
|  | Check to include this hit in error tolerant search or archive report |
|  |  |

|  |  |  |  |  |  |  |  |  |  |  |  |
| --- | --- | --- | --- | --- | --- | --- | --- | --- | --- | --- | --- |
|  | **Query** | **Observed** | **Mr(expt)** | **Mr(calc)** | **ppm** | **Miss** | **Score** | **Expect** | **Rank** | **Unique** | **Peptide** |
|  | 3665 | **913.9052** | **1825.7959** | **1825.8200** | **-13.20** | **0** | **18** | **0.088** | **1** | **U** | **R.DLGTHMMHLSGNDSGVR.L** |
|  | 3666 | **913.9058** | **1825.7970** | **1825.8200** | **-12.59** | **0** | **(3)** | **2.8** | **1** | **U** | **R.DLGTHMMHLSGNDSGVR.L** |
|  | 3667 | **913.9061** | **1825.7976** | **1825.8200** | **-12.25** | **0** | **(9)** | **0.69** | **1** | **U** | **R.DLGTHMMHLSGNDSGVR.L** |
|  | 3668 | **913.9063** | **1825.7979** | **1825.8200** | **-12.06** | **0** | **(6)** | **1.4** | **1** | **U** | **R.DLGTHMMHLSGNDSGVR.L** |
|  | 3669 | **913.9064** | **1825.7983** | **1825.8200** | **-11.86** | **0** | **(13)** | **0.26** | **1** | **U** | **R.DLGTHMMHLSGNDSGVR.L** |
|  | 3672 | **913.9075** | **1825.8005** | **1825.8200** | **-10.65** | **0** | **(17)** | **0.11** | **1** | **U** | **R.DLGTHMMHLSGNDSGVR.L** |
|  | 3689 | **921.9025** | **1841.7905** | **1841.8149** | **-13.23** | **0** | **(2)** | **2.9** | **3** | **U** | **R.DLGTHMMHLSGNDSGVR.L + Oxidation (M)** |
|  | 3692 | **921.9037** | **1841.7928** | **1841.8149** | **-11.97** | **0** | **(12)** | **0.26** | **1** | **U** | **R.DLGTHMMHLSGNDSGVR.L + Oxidation (M)** |

  

|  |  |
| --- | --- |
|  | |
|  | **Proteins matching the same set of peptides:** |

|  |  |
| --- | --- |
|  | sp|Q6ZUB0|S31D4\_HUMAN    **Mass:** 103267   **Score:** 18     **Matches:** 8(0)  **Sequences:** 1(0) |
|  | Spermatogenesis-associated protein 31D4 OS=Homo sapiens OX=9606 GN=SPATA31D4 PE=2 SV=2 |

---

|  |  |
| --- | --- |
| **132.** | sp|Q9UIF3|TEKT2\_HUMAN    **Mass:** 50155    **Score:** 17     **Matches:** 2(0)  **Sequences:** 1(0)  **emPAI:** 0.07 |
|  | Tektin-2 OS=Homo sapiens OX=9606 GN=TEKT2 PE=1 SV=1 |

|  |  |
| --- | --- |
|  | Check to include this hit in error tolerant search or archive report |
|  |  |

|  |  |  |  |  |  |  |  |  |  |  |  |
| --- | --- | --- | --- | --- | --- | --- | --- | --- | --- | --- | --- |
|  | **Query** | **Observed** | **Mr(expt)** | **Mr(calc)** | **ppm** | **Miss** | **Score** | **Expect** | **Rank** | **Unique** | **Peptide** |
|  | 629 | **457.2770** | **912.5393** | **912.5392** | **0.11** | **1** | **17** | **0.21** | **1** | **U** | **R.KLTVPAER.F** |
|  | 630 | **457.2770** | **912.5395** | **912.5392** | **0.31** | **1** | **(9)** | **1.3** | **1** | **U** | **R.KLTVPAER.F** |

  


---

|  |  |
| --- | --- |
| **133.** | sp|P27708|PYR1\_HUMAN    **Mass:** 245167   **Score:** 17     **Matches:** 4(0)  **Sequences:** 3(0)  **emPAI:** 0.01 |
|  | CAD protein OS=Homo sapiens OX=9606 GN=CAD PE=1 SV=3 |

|  |  |
| --- | --- |
|  | Check to include this hit in error tolerant search or archive report |
|  |  |

|  |  |  |  |  |  |  |  |  |  |  |  |
| --- | --- | --- | --- | --- | --- | --- | --- | --- | --- | --- | --- |
|  | **Query** | **Observed** | **Mr(expt)** | **Mr(calc)** | **ppm** | **Miss** | **Score** | **Expect** | **Rank** | **Unique** | **Peptide** |
|  | 844 | **487.2936** | **972.5727** | **972.5604** | **12.7** | **1** | **5** | **3.6** | **1** | **U** | **K.ERSLDILK.G** |
|  | 2379 | 668.3577 | 1334.7008 | 1334.7016 | -0.63 | 0 | 17 | 0.29 | 2 | U | K.NFELVINLSMR.G |
|  | 2380 | 668.3581 | 1334.7016 | 1334.7016 | -0.00 | 0 | (14) | 0.64 | 2 | U | K.NFELVINLSMR.G |
|  | 2656 | 704.3592 | 1406.7038 | 1406.7194 | -11.06 | 1 | 2 | 9.6 | 4 | U | K.GWKEIEYEVVR.D |

  


---

|  |  |
| --- | --- |
| **134.** | sp|P35221|CTNA1\_HUMAN    **Mass:** 100693   **Score:** 17     **Matches:** 3(1)  **Sequences:** 3(1)  **emPAI:** 0.03 |
|  | Catenin alpha-1 OS=Homo sapiens OX=9606 GN=CTNNA1 PE=1 SV=1 |

|  |  |
| --- | --- |
|  | Check to include this hit in error tolerant search or archive report |
|  |  |

|  |  |  |  |  |  |  |  |  |  |  |  |
| --- | --- | --- | --- | --- | --- | --- | --- | --- | --- | --- | --- |
|  | **Query** | **Observed** | **Mr(expt)** | **Mr(calc)** | **ppm** | **Miss** | **Score** | **Expect** | **Rank** | **Unique** | **Peptide** |
|  | 61 | **370.7548** | **739.4951** | **739.5068** | **-15.78** | **1** | **17** | **0.021** | **1** | **U** | **K.KPLVKR.E** |
|  | 2476 | **679.8536** | **1357.6926** | **1357.6950** | **-1.76** | **1** | **0** | **14** | **10** | **U** | **K.DVDGLDRTAGAIR.G** |
|  | 3007 | **511.5985** | **1531.7736** | **1531.7882** | **-9.53** | **0** | **6** | **3.8** | **5** | **U** | **K.QIIVDPLSFSEER.F** |

  


---

|  |  |
| --- | --- |
| **135.** | sp|E7EW31|PROB1\_HUMAN    **Mass:** 107308   **Score:** 17     **Matches:** 2(0)  **Sequences:** 2(0)  **emPAI:** 0.03 |
|  | Proline-rich basic protein 1 OS=Homo sapiens OX=9606 GN=PROB1 PE=2 SV=2 |

|  |  |
| --- | --- |
|  | Check to include this hit in error tolerant search or archive report |
|  |  |

|  |  |  |  |  |  |  |  |  |  |  |  |
| --- | --- | --- | --- | --- | --- | --- | --- | --- | --- | --- | --- |
|  | **Query** | **Observed** | **Mr(expt)** | **Mr(calc)** | **ppm** | **Miss** | **Score** | **Expect** | **Rank** | **Unique** | **Peptide** |
|  | 617 | **456.2325** | **910.4505** | **910.4410** | **10.4** | **0** | **17** | **0.26** | **1** | **U** | **R.HGPGSGFPR.G** |
|  | 3424 | **433.2543** | **1728.9882** | **1728.9859** | **1.32** | **1** | **9** | **0.38** | **1** | **U** | **K.RQIELRPRPQSPPR.A** |

  


---

|  |  |
| --- | --- |
| **136.** | sp|Q9UPW6|SATB2\_HUMAN    **Mass:** 83017    **Score:** 17     **Matches:** 5(0)  **Sequences:** 2(0)  **emPAI:** 0.04 |
|  | DNA-binding protein SATB2 OS=Homo sapiens OX=9606 GN=SATB2 PE=1 SV=2 |

|  |  |
| --- | --- |
|  | Check to include this hit in error tolerant search or archive report |
|  |  |

|  |  |  |  |  |  |  |  |  |  |  |  |
| --- | --- | --- | --- | --- | --- | --- | --- | --- | --- | --- | --- |
|  | **Query** | **Observed** | **Mr(expt)** | **Mr(calc)** | **ppm** | **Miss** | **Score** | **Expect** | **Rank** | **Unique** | **Peptide** |
|  | 2782 | 720.3594 | 1438.7043 | 1438.7164 | -8.41 | 1 | 1 | 13 | 4 | U | R.QQSQPAKESSPPR.E |
|  | 3948 | **672.3104** | **2013.9094** | **2013.8732** | **18.0** | **0** | **(12)** | **0.52** | **1** | **U** | **R.SMNPNVSMVSSASSSPSSSR.T + Oxidation (M)** |
|  | 3949 | **504.4849** | **2013.9106** | **2013.8732** | **18.6** | **0** | **17** | **0.16** | **1** | **U** | **R.SMNPNVSMVSSASSSPSSSR.T + Oxidation (M)** |
|  | 3950 | **672.3108** | **2013.9107** | **2013.8732** | **18.7** | **0** | **(9)** | **0.91** | **1** | **U** | **R.SMNPNVSMVSSASSSPSSSR.T + Oxidation (M)** |
|  | 3951 | **504.4850** | **2013.9110** | **2013.8732** | **18.8** | **0** | **(10)** | **0.74** | **1** | **U** | **R.SMNPNVSMVSSASSSPSSSR.T + Oxidation (M)** |

  


---

|  |  |
| --- | --- |
| **137.** | sp|Q6UY18|LIGO4\_HUMAN    **Mass:** 64532    **Score:** 16     **Matches:** 1(0)  **Sequences:** 1(0)  **emPAI:** 0.05 |
|  | Leucine-rich repeat and immunoglobulin-like domain-containing nogo receptor-interacting protein 4 OS=Homo sapiens OX=9606 GN=LINGO4 PE=2 SV=1 |

|  |  |
| --- | --- |
|  | Check to include this hit in error tolerant search or archive report |
|  |  |

|  |  |  |  |  |  |  |  |  |  |  |  |
| --- | --- | --- | --- | --- | --- | --- | --- | --- | --- | --- | --- |
|  | **Query** | **Observed** | **Mr(expt)** | **Mr(calc)** | **ppm** | **Miss** | **Score** | **Expect** | **Rank** | **Unique** | **Peptide** |
|  | 3729 | **929.4476** | **1856.8807** | **1856.8628** | **9.63** | **0** | **16** | **0.29** | **1** | **U** | **K.HHMTFDFVAPRPSGDK.N + Oxidation (M)** |

  


---

|  |  |
| --- | --- |
| **138.** | sp|Q96QI5|HS3S6\_HUMAN    **Mass:** 37505    **Score:** 15     **Matches:** 5(0)  **Sequences:** 3(0)  **emPAI:** 0.09 |
|  | Heparan sulfate glucosamine 3-O-sulfotransferase 6 OS=Homo sapiens OX=9606 GN=HS3ST6 PE=1 SV=2 |

|  |  |
| --- | --- |
|  | Check to include this hit in error tolerant search or archive report |
|  |  |

|  |  |  |  |  |  |  |  |  |  |  |  |
| --- | --- | --- | --- | --- | --- | --- | --- | --- | --- | --- | --- |
|  | **Query** | **Observed** | **Mr(expt)** | **Mr(calc)** | **ppm** | **Miss** | **Score** | **Expect** | **Rank** | **Unique** | **Peptide** |
|  | 786 | **478.7799** | **955.5453** | **955.5311** | **14.8** | **1** | **(0)** | **11** | **10** | **U** | **K.KAQGGSRPR.C** |
|  | 787 | **478.7813** | **955.5480** | **955.5311** | **17.6** | **1** | **10** | **1** | **2** | **U** | **K.KAQGGSRPR.C** |
|  | 1307 | **536.3243** | **1070.6340** | **1070.6488** | **-13.83** | **0** | **(1)** | **6.9** | **5** | **U** | **R.FPQALIVGVK.K** |
|  | 1308 | **536.3245** | **1070.6345** | **1070.6488** | **-13.39** | **0** | **15** | **0.27** | **1** | **U** | **R.FPQALIVGVK.K** |
|  | 4311 | **786.3968** | **2356.1687** | **2356.1414** | **11.6** | **1** | **2** | **5.7** | **1** | **U** | **-.MAGSGGLGGGAGGGQGAGAGQGAALRASR.A** |

  


---

|  |  |
| --- | --- |
| **139.** | sp|P20930|FILA\_HUMAN    **Mass:** 435036   **Score:** 14     **Matches:** 7(0)  **Sequences:** 6(0)  **emPAI:** 0.01 |
|  | Filaggrin OS=Homo sapiens OX=9606 GN=FLG PE=1 SV=3 |

|  |  |
| --- | --- |
|  | Check to include this hit in error tolerant search or archive report |
|  |  |

|  |  |  |  |  |  |  |  |  |  |  |  |
| --- | --- | --- | --- | --- | --- | --- | --- | --- | --- | --- | --- |
|  | **Query** | **Observed** | **Mr(expt)** | **Mr(calc)** | **ppm** | **Miss** | **Score** | **Expect** | **Rank** | **Unique** | **Peptide** |
|  | 1603 | **567.2839** | **1132.5532** | **1132.5585** | **-4.68** | **1** | **14** | **0.57** | **1** | **U** | **R.GQASSAVRDSR.H** |
|  | 1636 | 571.2633 | 1140.5121 | 1140.5272 | -13.25 | 0 | 1 | 6.5 | 3 | U | R.GQAASSHEQAR.S |
|  | 2409 | **671.7839** | **1341.5533** | **1341.5797** | **-19.64** | **1** | **8** | **0.29** | **1** | **U** | **K.TYDKEQSGDGSR.H** |
|  | 3185 | 805.8778 | 1609.7411 | 1609.7305 | 6.54 | 1 | 4 | 4.4 | 3 | U | R.AGHGHSADSSRQSGTR.H |
|  | 3692 | 921.9037 | 1841.7928 | 1841.7902 | 1.44 | 1 | (1) | 3.4 | 6 | U | R.SHHEDRAGHGHSADSSR.K |
|  | 3693 | **921.9038** | **1841.7931** | **1841.7902** | **1.57** | **1** | **2** | **2.9** | **2** | **U** | **R.SHHEDRAGHGHSADSSR.K** |
|  | 3965 | **672.6393** | **2014.8960** | **2014.9358** | **-19.73** | **1** | **2** | **3.5** | **1** | **U** | **R.WSGSASRNHLGSAWEQSR.D** |

  


---

|  |  |
| --- | --- |
| **140.** | sp|Q15054|DPOD3\_HUMAN    **Mass:** 51653    **Score:** 14     **Matches:** 2(0)  **Sequences:** 2(0)  **emPAI:** 0.06 |
|  | DNA polymerase delta subunit 3 OS=Homo sapiens OX=9606 GN=POLD3 PE=1 SV=2 |

|  |  |
| --- | --- |
|  | Check to include this hit in error tolerant search or archive report |
|  |  |

|  |  |  |  |  |  |  |  |  |  |  |  |
| --- | --- | --- | --- | --- | --- | --- | --- | --- | --- | --- | --- |
|  | **Query** | **Observed** | **Mr(expt)** | **Mr(calc)** | **ppm** | **Miss** | **Score** | **Expect** | **Rank** | **Unique** | **Peptide** |
|  | 835 | 487.2335 | 972.4524 | 972.4409 | 11.8 | 0 | 4 | 3.7 | 8 | U | K.GIMGMFASK.A + 2 Oxidation (M) |
|  | 1603 | 567.2839 | 1132.5532 | 1132.5724 | -16.97 | 1 | 14 | 0.57 | 1 | U | K.RVALSDDETK.E |

  


---

|  |  |
| --- | --- |
| **141.** | sp|Q14161|GIT2\_HUMAN    **Mass:** 85117    **Score:** 14     **Matches:** 1(0)  **Sequences:** 1(0)  **emPAI:** 0.04 |
|  | ARF GTPase-activating protein GIT2 OS=Homo sapiens OX=9606 GN=GIT2 PE=1 SV=2 |

|  |  |
| --- | --- |
|  | Check to include this hit in error tolerant search or archive report |
|  |  |

|  |  |  |  |  |  |  |  |  |  |  |  |
| --- | --- | --- | --- | --- | --- | --- | --- | --- | --- | --- | --- |
|  | **Query** | **Observed** | **Mr(expt)** | **Mr(calc)** | **ppm** | **Miss** | **Score** | **Expect** | **Rank** | **Unique** | **Peptide** |
|  | 1603 | 567.2839 | 1132.5532 | 1132.5374 | 14.0 | 0 | 14 | 0.57 | 1 | U | R.QGGHHELAER.L |

  


---

|  |  |
| --- | --- |
| **142.** | sp|Q8N1G1|REXO1\_HUMAN    **Mass:** 132740   **Score:** 14     **Matches:** 6(0)  **Sequences:** 5(0)  **emPAI:** 0.02 |
|  | RNA exonuclease 1 homolog OS=Homo sapiens OX=9606 GN=REXO1 PE=1 SV=3 |

|  |  |
| --- | --- |
|  | Check to include this hit in error tolerant search or archive report |
|  |  |

|  |  |  |  |  |  |  |  |  |  |  |  |
| --- | --- | --- | --- | --- | --- | --- | --- | --- | --- | --- | --- |
|  | **Query** | **Observed** | **Mr(expt)** | **Mr(calc)** | **ppm** | **Miss** | **Score** | **Expect** | **Rank** | **Unique** | **Peptide** |
|  | 206 | **407.2401** | **812.4656** | **812.4617** | **4.85** | **1** | **14** | **0.39** | **1** | **U** | **K.AVSQPRR.H** |
|  | 207 | **407.2405** | **812.4665** | **812.4617** | **5.98** | **1** | **(1)** | **7.7** | **1** | **U** | **K.AVSQPRR.H** |
|  | 673 | 464.2504 | 926.4863 | 926.4934 | -7.61 | 1 | 5 | 3.7 | 3 | U | K.KGPQASSPR.R |
|  | 782 | **478.2878** | **954.5611** | **954.5610** | **0.10** | **1** | **1** | **4.9** | **5** | **U** | **R.LAAAPTGAKR.T** |
|  | 2346 | **441.5614** | **1321.6625** | **1321.6738** | **-8.61** | **1** | **12** | **1** | **1** | **U** | **K.YSLASLDRGQGR.G** |
|  | 2648 | **702.8782** | **1403.7419** | **1403.7269** | **10.7** | **1** | **5** | **4.9** | **1** | **U** | **R.EHRSAEAPALAPR.G** |

  


---

|  |  |
| --- | --- |
| **143.** | sp|O14640|DVL1\_HUMAN    **Mass:** 75539    **Score:** 14     **Matches:** 2(0)  **Sequences:** 2(0)  **emPAI:** 0.04 |
|  | Segment polarity protein dishevelled homolog DVL-1 OS=Homo sapiens OX=9606 GN=DVL1 PE=1 SV=2 |

|  |  |
| --- | --- |
|  | Check to include this hit in error tolerant search or archive report |
|  |  |

|  |  |  |  |  |  |  |  |  |  |  |  |
| --- | --- | --- | --- | --- | --- | --- | --- | --- | --- | --- | --- |
|  | **Query** | **Observed** | **Mr(expt)** | **Mr(calc)** | **ppm** | **Miss** | **Score** | **Expect** | **Rank** | **Unique** | **Peptide** |
|  | 1556 | 563.2747 | 1124.5349 | 1124.5172 | 15.7 | 0 | 2 | 7.9 | 4 | U | K.SMDQDFGVVK.E |
|  | 4429 | **680.8257** | **2719.2736** | **2719.3044** | **-11.34** | **0** | **14** | **0.32** | **1** | **U** | **R.ASSFSSITDSTMSLNIVTVTLNMER.H + Oxidation (M)** |

  


---

**Peptide matches not assigned to protein hits:** (no details means no match)  
  

|  |  |  |  |  |  |  |  |  |  |  |  |
| --- | --- | --- | --- | --- | --- | --- | --- | --- | --- | --- | --- |
|  | **Query** | **Observed** | **Mr(expt)** | **Mr(calc)** | **ppm** | **Miss** | **Score** | **Expect** | **Rank** | **Unique** | **Peptide** |
|  | 2288 | **436.8893** | **1307.6461** | **1307.6656** | **-14.91** | **1** | **23** | **0.077** | **1** |  | **MLRADGDFLVR + Oxidation (M)** |
|  | 53 | **367.1973** | **732.3801** | **732.3878** | **-10.55** | **1** | **23** | **0.11** | **1** |  | **SRLDSR** |
|  | 2290 | **436.8897** | **1307.6471** | **1307.6656** | **-14.13** | **1** | **23** | **0.08** | **1** |  | **MLRADGDFLVR + Oxidation (M)** |
|  | 184 | **401.7426** | **801.4707** | **801.4709** | **-0.23** | **0** | **23** | **0.11** | **1** |  | **LGSAVVTR** |
|  | 3697 | **615.2855** | **1842.8347** | **1842.8683** | **-18.20** | **0** | **22** | **0.065** | **1** |  | **AQGMPESGVALAEGFHAR + Oxidation (M)** |
|  | 2004 | **412.2263** | **1233.6570** | **1233.6428** | **11.6** | **0** | **22** | **0.099** | **1** |  | **EVFLVMPTGGGK** |
|  | 2547 | **691.8613** | **1381.7081** | **1381.7097** | **-1.19** | **1** | **21** | **0.12** | **1** |  | **GAMMSIAFKEGLK** |
|  | 2680 | **472.9130** | **1415.7172** | **1415.7078** | **6.63** | **1** | **21** | **0.11** | **1** |  | **MDPETRGQEIIK** |
|  | 748 | **473.2594** | **944.5042** | **944.5225** | **-19.43** | **1** | **21** | **0.19** | **1** |  | **RMQELLR** |
|  | 839 | 487.2338 | 972.4530 | 972.4698 | -17.28 | 0 | 21 | 0.064 | 1 |  | MPPSLQER + Oxidation (M) |
|  | 997 | **507.8133** | **1013.6120** | **1013.6121** | **-0.05** | **1** | **21** | **0.054** | **1** |  | **VDAKVEIIK** |
|  | 2431 | **674.3442** | **1346.6739** | **1346.6751** | **-0.90** | **0** | **21** | **0.14** | **1** |  | **IMELDSNDGLLK** |
|  | 2537 | **689.8111** | **1377.6076** | **1377.6082** | **-0.38** | **1** | **20** | **0.078** | **1** |  | **RVEIMEEESEQ** |
|  | 1509 | **557.2795** | **1112.5445** | **1112.5574** | **-11.60** | **0** | **20** | **0.13** | **1** |  | **NQGPQESVVR** |
|  | 3926 | **660.9848** | **1979.9326** | **1979.9602** | **-13.96** | **1** | **20** | **0.11** | **1** |  | **FREAHGQPPGPFDEQIR** |
|  | 1506 | **557.2779** | **1112.5412** | **1112.5574** | **-14.57** | **0** | **20** | **0.13** | **1** |  | **NQGPQESVVR** |
|  | 2215 | **430.2371** | **1287.6896** | **1287.7147** | **-19.44** | **0** | **20** | **0.16** | **1** |  | **LSTLSVSGQQLR** |
|  | 1834 | **596.8095** | **1191.6045** | **1191.6248** | **-17.04** | **1** | **20** | **0.18** | **1** |  | **IGDSLFGAREK** |
|  | 134 | 388.2085 | 774.4024 | 774.4058 | -4.32 | 0 | 19 | 0.38 | 1 |  | LLQDMR |
|  | 3104 | **784.3665** | **1566.7185** | **1566.7130** | **3.49** | **1** | **19** | **0.11** | **1** |  | **GGDMSKNVSQSQMAK** |
|  | 1595 | 377.8874 | 1130.6403 | 1130.6196 | 18.3 | 1 | 19 | 0.13 | 1 |  | KSSSFLIHGR |
|  | 1888 | 601.3484 | 1200.6823 | 1200.6714 | 9.14 | 1 | 19 | 0.13 | 1 |  | KNTEGALDLLK |
|  | 3730 | **929.4691** | **1856.9236** | **1856.9091** | **7.81** | **1** | **19** | **0.14** | **1** |  | **LGVFSEMEANFKNLSR + Oxidation (M)** |
|  | 2060 | **417.2116** | **1248.6131** | **1248.6350** | **-17.54** | **0** | **19** | **0.2** | **1** |  | **SLFLNGNDIEK** |
|  | 1013 | 510.2612 | 1018.5078 | 1018.5043 | 3.41 | 0 | 19 | 0.24 | 1 |  | AGSSSPSSALR |
|  | 436 | **432.7502** | **863.4858** | **863.4753** | **12.2** | **0** | **18** | **0.19** | **1** |  | **AVGVEYVK** |
|  | 1507 | **557.2784** | **1112.5422** | **1112.5574** | **-13.69** | **0** | **18** | **0.18** | **1** |  | **NQGPQESVVR** |
|  | 1706 | **582.2873** | **1162.5601** | **1162.5618** | **-1.45** | **0** | **18** | **0.24** | **1** |  | **NFLVEEEQR** |
|  | 1906 | **602.8019** | **1203.5892** | **1203.5852** | **3.30** | **1** | **18** | **0.24** | **1** |  | **EMIGRIPCGR + Oxidation (M)** |
|  | 3641 | **604.6184** | **1810.8334** | **1810.8068** | **14.7** | **0** | **18** | **0.14** | **1** |  | **GTNSSDSEELSAGESITK** |
|  | 51 | **366.7198** | **731.4250** | **731.4177** | **9.98** | **0** | **18** | **0.27** | **1** |  | **INSSAIK** |
|  | 1661 | **575.7900** | **1149.5654** | **1149.5488** | **14.4** | **0** | **18** | **0.23** | **1** |  | **SLDFGIMPDR** |
|  | 4130 | **737.7057** | **2210.0954** | **2210.1259** | **-13.80** | **1** | **18** | **0.18** | **1** |  | **QLQPSVVWIEDTEKTFYK** |
|  | 1832 | **596.3181** | **1190.6217** | **1190.6216** | **0.05** | **1** | **18** | **0.25** | **1** |  | **KTEAELEMLK** |
|  | 3276 | **828.9452** | **1655.8759** | **1655.8743** | **0.98** | **0** | **18** | **0.18** | **1** |  | **AGPQSPSPGAPPAAKPAR** |
|  | 2163 | **637.3188** | **1272.6230** | **1272.6422** | **-15.05** | **1** | **18** | **0.23** | **1** |  | **IEEARSQANQK** |
|  | 1249 | **530.7908** | **1059.5670** | **1059.5560** | **10.3** | **0** | **18** | **0.33** | **1** |  | **VSSDLPTSVR** |
|  | 1606 | **567.2961** | **1132.5777** | **1132.5699** | **6.92** | **0** | **18** | **0.29** | **1** |  | **MAEQLAWLR + Oxidation (M)** |
|  | 1386 | **544.8019** | **1087.5893** | **1087.6026** | **-12.18** | **1** | **18** | **0.33** | **1** |  | **SWAGLELGKK** |
|  | 2289 | **654.8307** | **1307.6468** | **1307.6656** | **-14.36** | **1** | **18** | **0.26** | **1** |  | **MLRADGDFLVR + Oxidation (M)** |
|  | 2171 | **639.3578** | **1276.7011** | **1276.6809** | **15.9** | **1** | **18** | **0.2** | **1** |  | **AIMEKSNVTLR + Oxidation (M)** |
|  | 2549 | **691.8956** | **1381.7767** | **1381.7752** | **1.12** | **0** | **17** | **0.13** | **1** |  | **LATQLTGPVMPVR** |
|  | 55 | **369.1898** | **736.3651** | **736.3538** | **15.4** | **1** | **17** | **0.33** | **1** |  | **TKMGER + Oxidation (M)** |
|  | 766 | **475.7758** | **949.5371** | **949.5206** | **17.4** | **1** | **17** | **0.16** | **1** |  | **RDGPPRPR** |
|  | 4066 | **1057.5189** | **2113.0233** | **2113.0613** | **-17.99** | **0** | **17** | **0.2** | **1** |  | **TTSDLFLEVTSATSLQICK** |
|  | 3871 | **965.4272** | **1928.8398** | **1928.8365** | **1.71** | **1** | **17** | **0.089** | **1** |  | **FTCACPDGMLLARDMR + Oxidation (M)** |
|  | 2311 | **438.9296** | **1313.7670** | **1313.7554** | **8.77** | **0** | **17** | **0.11** | **1** |  | **EAGLLAAVTLTQK** |
|  | 3698 | **615.2857** | **1842.8353** | **1842.8683** | **-17.89** | **0** | **17** | **0.2** | **1** |  | **AQGMPESGVALAEGFHAR + Oxidation (M)** |
|  | 792 | **479.2773** | **956.5401** | **956.5291** | **11.5** | **0** | **17** | **0.31** | **1** |  | **TIGISVDPR** |
|  | 2223 | **431.2385** | **1290.6938** | **1290.6728** | **16.3** | **1** | **17** | **0.27** | **1** |  | **RVHGGHQMTIR** |
|  | 2484 | 680.8541 | 1359.6936 | 1359.7180 | -17.99 | 0 | 17 | 0.41 | 1 |  | LQSAMTEGGVVLR |
|  | 1816 | **396.8875** | **1187.6405** | **1187.6550** | **-12.21** | **0** | **17** | **0.33** | **1** |  | **QAGVFEPTIVK** |
|  | 3006 | **383.9506** | **1531.7731** | **1531.7552** | **11.7** | **0** | **17** | **0.33** | **1** |  | **EQMVAVTEANEALK** |
|  | 1766 | **588.3204** | **1174.6263** | **1174.6380** | **-9.93** | **0** | **17** | **0.37** | **1** |  | **SGIQNTAMLLK** |
|  | 471 | **435.7740** | **869.5334** | **869.5447** | **-13.01** | **1** | **17** | **0.15** | **1** |  | **VVVERLR** |
|  | 1367 | 362.1681 | 1083.4824 | 1083.4833 | -0.76 | 0 | 17 | 0.17 | 1 |  | ISQDDYTSR |
|  | 2130 | **633.8138** | **1265.6130** | **1265.6139** | **-0.73** | **0** | **17** | **0.3** | **1** |  | **TGETNDFELLK** |
|  | 4034 | **691.6461** | **2071.9164** | **2071.9179** | **-0.76** | **0** | **17** | **0.084** | **1** |  | **GPPFGSPMGHPGPMPPHGMR + 2 Oxidation (M)** |
|  | 2462 | 679.3623 | 1356.7100 | 1356.7262 | -11.91 | 1 | 17 | 0.36 | 1 |  | GARESAVWNVLR |
|  | 2178 | **640.8384** | **1279.6622** | **1279.6707** | **-6.61** | **1** | **17** | **0.3** | **1** |  | **SIKFLSQCAAR** |
|  | 2423 | **673.8037** | **1345.5929** | **1345.5681** | **18.4** | **0** | **17** | **0.13** | **1** |  | **AGGSCQAAQPEDR** |
|  | 1588 | **565.8122** | **1129.6098** | **1129.5880** | **19.3** | **0** | **16** | **0.43** | **1** |  | **GAAEPFLAQAR** |
|  | 2642 | **702.3622** | **1402.7098** | **1402.7092** | **0.42** | **1** | **16** | **0.37** | **1** |  | **ELSWKQQDEIK** |
|  | 1508 | **557.2787** | **1112.5428** | **1112.5574** | **-13.13** | **0** | **16** | **0.31** | **1** |  | **NQGPQESVVR** |
|  | 3499 | **588.6154** | **1762.8244** | **1762.8248** | **-0.20** | **0** | **16** | **0.3** | **1** |  | **FGQGVHHAAGQAGNEAGR** |
|  | 150 | **394.2366** | **786.4587** | **786.4487** | **12.7** | **0** | **16** | **0.56** | **1** |  | **EAVDILK** |
|  | 1742 | **585.3293** | **1168.6441** | **1168.6241** | **17.2** | **1** | **16** | **0.2** | **1** |  | **KVDVYGFVSR** |
|  | 4072 | **707.3175** | **2118.9307** | **2118.9537** | **-10.86** | **1** | **16** | **0.11** | **1** |  | **TMMGYWSTQGCKLVDTNK** |
|  | 2261 | 650.8237 | 1299.6328 | 1299.6167 | 12.3 | 0 | 16 | 0.35 | 1 |  | DLQSSQGGQQPR |
|  | 1150 | **523.2856** | **1044.5567** | **1044.5386** | **17.3** | **0** | **16** | **0.49** | **1** |  | **LGVNDCVLR** |
|  | 3266 | **550.9249** | **1649.7530** | **1649.7818** | **-17.46** | **0** | **16** | **0.23** | **1** |  | **EAAASEIEAVSVAMEK + Oxidation (M)** |
|  | 2849 | **732.3623** | **1462.7100** | **1462.7353** | **-17.25** | **0** | **16** | **0.39** | **1** |  | **AMFFSMGFIVAVK + Oxidation (M)** |
|  | 1889 | **601.3488** | **1200.6831** | **1200.6900** | **-5.76** | **1** | **16** | **0.27** | **1** |  | **KDLIQMVLNK** |
|  | 3503 | **882.8870** | **1763.7595** | **1763.7308** | **16.3** | **0** | **16** | **0.13** | **1** |  | **EEYSQYATSDTAMPR + Oxidation (M)** |
|  | 1511 | **557.2805** | **1112.5464** | **1112.5574** | **-9.95** | **0** | **16** | **0.36** | **1** |  | **NQGPQESVVR** |
|  | 3739 | **622.9810** | **1865.9211** | **1865.9232** | **-1.13** | **0** | **16** | **0.38** | **1** |  | **AAQGSQSPTGHEGGTIQLK** |
|  | 1466 | **553.7665** | **1105.5184** | **1105.5114** | **6.37** | **0** | **16** | **0.41** | **1** |  | **LMTAYNSYK + Oxidation (M)** |
|  | 3664 | **913.9046** | **1825.7946** | **1825.8013** | **-3.65** | **1** | **16** | **0.14** | **1** |  | **KEQSHEDQGHIMDTR + Oxidation (M)** |
|  | 2548 | **691.8634** | **1381.7122** | **1381.7097** | **1.81** | **1** | **16** | **0.43** | **1** |  | **GAMMSIAFKEGLK** |
|  | 981 | **505.7681** | **1009.5216** | **1009.5192** | **2.38** | **0** | **16** | **0.38** | **1** |  | **STNPELPPR** |
|  | 2742 | **714.8704** | **1427.7262** | **1427.7481** | **-15.35** | **1** | **16** | **0.38** | **1** |  | **LSGAAARGDVQEVR** |
|  | 577 | **450.2695** | **898.5245** | **898.5097** | **16.5** | **1** | **16** | **0.29** | **1** |  | **AQGAQRLR** |
|  | 2770 | **718.8854** | **1435.7563** | **1435.7493** | **4.89** | **1** | **16** | **0.33** | **1** |  | **TSIPIKAICDYR** |
|  | 52 | **366.7199** | **731.4251** | **731.4177** | **10.1** | **0** | **16** | **0.48** | **1** |  | **SNIGTLK** |
|  | 2735 | 476.8978 | 1427.6717 | 1427.6802 | -5.98 | 1 | 15 | 0.3 | 1 |  | EKHFHCLLCGK |
|  | 4244 | **762.0598** | **2283.1576** | **2283.1894** | **-13.94** | **0** | **15** | **0.29** | **1** |  | **EEVIDFSKPFMSLGISIMIK** |
|  | 991 | **506.8056** | **1011.5966** | **1011.5937** | **2.88** | **1** | **15** | **0.2** | **1** |  | **AVAGAIARQR** |
|  | 565 | **449.7589** | **897.5033** | **897.4854** | **19.9** | **0** | **15** | **0.28** | **1** |  | **VMNILHR + Oxidation (M)** |
|  | 2273 | **652.8459** | **1303.6772** | **1303.6772** | **0.04** | **0** | **15** | **0.51** | **1** |  | **LENLFEALNNK** |
|  | 791 | **479.2481** | **956.4817** | **956.4927** | **-11.46** | **0** | **15** | **0.32** | **1** |  | **AVSLEGEPR** |
|  | 576 | **450.2692** | **898.5238** | **898.5097** | **15.7** | **1** | **15** | **0.32** | **1** |  | **AQGAQRLR** |
|  | 2865 | **736.3484** | **1470.6822** | **1470.6878** | **-3.79** | **0** | **15** | **0.28** | **1** |  | **EELGELSEQFYK** |
|  | 223 | 412.2314 | 822.4483 | 822.4460 | 2.74 | 0 | 15 | 0.33 | 1 |  | ATHNRPK |
|  | 725 | **469.2428** | **936.4711** | **936.4811** | **-10.66** | **1** | **15** | **0.42** | **1** |  | **MSSSVVRR + Oxidation (M)** |
|  | 4242 | **762.0583** | **2283.1530** | **2283.1313** | **9.53** | **0** | **15** | **0.31** | **1** |  | **ALLFVMMDLTGEVSNGAVAMAK + Oxidation (M)** |
|  | 3577 | **897.9091** | **1793.8037** | **1793.8028** | **0.51** | **0** | **15** | **0.25** | **1** |  | **TGTTGQSGAESGTTEPSAR** |
|  | 3731 | 929.4760 | 1856.9373 | 1856.9051 | 17.4 | 0 | 15 | 0.37 | 1 |  | QVPNSTGPDNATLQMLR + Oxidation (M) |
|  | 1811 | **593.3511** | **1184.6876** | **1184.6957** | **-6.88** | **1** | **15** | **0.29** | **1** |  | **KYFFLLNLK** |
|  | 2903 | 497.9025 | 1490.6857 | 1490.7035 | -11.94 | 1 | 15 | 0.28 | 1 |  | AGEDLEREVAQMK + Oxidation (M) |
|  | 1663 | **575.7911** | **1149.5677** | **1149.5560** | **10.2** | **1** | **15** | **0.51** | **1** |  | **SQVQSGMRNK + Oxidation (M)** |
|  | 2700 | **711.3229** | **1420.6312** | **1420.6194** | **8.34** | **0** | **15** | **0.2** | **1** |  | **NYMTHAHYDAAK** |
|  | 4336 | **807.4330** | **2419.2771** | **2419.3066** | **-12.19** | **1** | **15** | **0.17** | **1** |  | **MAITLTLQTAEMQEGLLAVKVK + 2 Oxidation (M)** |
|  | 641 | **458.7876** | **915.5606** | **915.5575** | **3.39** | **0** | **15** | **0.11** | **1** |  | **VLLTMIAR** |
|  | 1835 | **596.8107** | **1191.6069** | **1191.6248** | **-14.99** | **1** | **15** | **0.53** | **1** |  | **IGDSLFGAREK** |
|  | 890 | **493.7930** | **985.5714** | **985.5668** | **4.68** | **1** | **15** | **0.49** | **1** |  | **SRAEALLAR** |
|  | 920 | **497.2721** | **992.5297** | **992.5212** | **8.58** | **1** | **15** | **0.5** | **1** |  | **MEKIISEK + Oxidation (M)** |
|  | 4383 | **846.0548** | **2535.1426** | **2535.1920** | **-19.47** | **1** | **15** | **0.21** | **1** |  | **KSANLVISASQLGDSAMYFCAMR + Oxidation (M)** |
|  | 761 | **474.7619** | **947.5092** | **947.5222** | **-13.75** | **1** | **15** | **0.54** | **1** |  | **QKMSALVR + Oxidation (M)** |
|  | 1401 | **546.2875** | **1090.5605** | **1090.5441** | **15.1** | **0** | **15** | **0.73** | **1** |  | **VGTVTMGPSAR + Oxidation (M)** |
|  | 1546 | 562.2948 | 1122.5750 | 1122.5669 | 7.24 | 0 | 15 | 0.53 | 1 |  | AVSEGLHPDAK |
|  | 1898 | 601.8214 | 1201.6283 | 1201.6415 | -10.98 | 1 | 15 | 0.67 | 1 |  | ASRVSAENLQK |
|  | 1524 | **559.2960** | **1116.5774** | **1116.5887** | **-10.15** | **1** | **15** | **0.67** | **1** |  | **ASLEERAVSR** |
|  | 165 | **399.2412** | **796.4679** | **796.4807** | **-15.99** | **0** | **15** | **0.21** | **1** |  | **AAKPSVPK** |
|  | 2678 | **707.8610** | **1413.7074** | **1413.7108** | **-2.46** | **1** | **15** | **0.49** | **1** |  | **TLCIRMTTGAFK + Oxidation (M)** |
|  | 2212 | **644.3127** | **1286.6109** | **1286.6143** | **-2.62** | **0** | **14** | **0.39** | **1** |  | **FSGSGAGTDFTLK** |
|  | 3947 | **672.0092** | **2013.0058** | **2012.9837** | **11.0** | **0** | **14** | **0.48** | **1** |  | **DLQEDEVPVVGIMATGGGAR** |
|  | 1173 | **523.2873** | **1044.5601** | **1044.5788** | **-17.87** | **1** | **14** | **0.64** | **1** |  | **SISRPRSSR** |
|  | 2059 | **625.3120** | **1248.6095** | **1248.6251** | **-12.52** | **0** | **14** | **0.56** | **1** |  | **FTFRPPNNEK** |
|  | 696 | 467.2364 | 932.4582 | 932.4716 | -14.39 | 0 | 14 | 0.66 | 1 |  | SSGFPVSPR |
|  | 201 | **406.7345** | **811.4544** | **811.4439** | **12.9** | **0** | **14** | **0.29** | **1** |  | **LPASAEPK** |
|  | 3056 | **774.8687** | **1547.7227** | **1547.7177** | **3.24** | **0** | **14** | **0.4** | **1** |  | **EIYYITEDMSIR + Oxidation (M)** |
|  | 1126 | **523.2849** | **1044.5551** | **1044.5716** | **-15.76** | **0** | **14** | **0.76** | **1** |  | **LSSAHVYLR** |
|  | 2214 | **644.8518** | **1287.6891** | **1287.7147** | **-19.87** | **0** | **14** | **0.56** | **1** |  | **LSTLSVSGQQLR** |
|  | 892 | **494.2952** | **986.5758** | **986.5648** | **11.1** | **0** | **14** | **0.45** | **1** |  | **LLETPISSK** |
|  | 2606 | **465.5769** | **1393.7090** | **1393.7275** | **-13.33** | **1** | **14** | **0.58** | **1** |  | **LFTSPLCDVSKK** |
|  | 575 | **450.2690** | **898.5235** | **898.5097** | **15.4** | **1** | **14** | **0.41** | **1** |  | **AQGAQRLR** |
|  | 921 | **497.2904** | **992.5663** | **992.5655** | **0.87** | **0** | **14** | **0.27** | **1** |  | **LLIYGASTR** |
|  | 3986 | **507.9891** | **2027.9272** | **2027.8928** | **16.9** | **0** | **14** | **0.27** | **1** |  | **MPAISDQDMSAYLAEQSR + Oxidation (M)** |
|  | 3516 | **883.8946** | **1765.7747** | **1765.7723** | **1.37** | **1** | **14** | **0.2** | **1** |  | **DAVQNCCGISKTEER** |
|  | 7 | **353.6976** | **705.3806** | **705.3843** | **-5.32** | **0** | **14** | **0.68** | **1** |  | **TMLAVR + Oxidation (M)** |
|  | 2744 | **714.8758** | **1427.7370** | **1427.7117** | **17.7** | **0** | **14** | **0.53** | **1** |  | **AGGGAGGSGGSGPSAILR** |
|  | 3500 | **441.7135** | **1762.8250** | **1762.8248** | **0.13** | **0** | **14** | **0.52** | **1** |  | **FGQGVHHAAGQAGNEAGR** |
|  | 525 | 441.2634 | 880.5123 | 880.5243 | -13.61 | 1 | 14 | 0.2 | 1 |  | GVPRTPVR |
|  | 3007 | 511.5985 | 1531.7736 | 1531.7630 | 6.91 | 1 | 14 | 0.61 | 1 |  | LENQWETSIREK |
|  | 1584 | **565.3380** | **1128.6615** | **1128.6615** | **-0.03** | **1** | **14** | **0.36** | **1** |  | **QVSLTAVKQR** |
|  | 209 | **408.2339** | **814.4533** | **814.4548** | **-1.92** | **0** | **14** | **0.96** | **1** |  | **ELIIGDR** |
|  | 992 | **506.8056** | **1011.5967** | **1011.5825** | **14.0** | **1** | **14** | **0.28** | **1** |  | **AEATRVLPR** |
|  | 366 | **421.7584** | **841.5023** | **841.5021** | **0.25** | **1** | **14** | **0.4** | **1** |  | **VAGAATPKK** |
|  | 568 | 449.7769 | 897.5393 | 897.5396 | -0.33 | 1 | 14 | 0.24 | 1 |  | AKITNVPR |
|  | 603 | **453.7184** | **905.4222** | **905.4389** | **-18.44** | **0** | **14** | **0.53** | **1** |  | **GAGTAVQMR + Oxidation (M)** |
|  | 445 | **434.7663** | **867.5180** | **867.5079** | **11.6** | **0** | **14** | **0.19** | **1** |  | **VPRPFPR** |
|  | 518 | **439.7710** | **877.5274** | **877.5134** | **16.0** | **0** | **14** | **0.14** | **1** |  | **HVSPLIGR** |
|  | 1141 | **523.2855** | **1044.5565** | **1044.5716** | **-14.48** | **0** | **14** | **0.78** | **1** |  | **LSSAHVYLR** |
|  | 2029 | **620.8859** | **1239.7573** | **1239.7373** | **16.1** | **1** | **14** | **0.12** | **1** |  | **VTPALQMKKPK** |
|  | 2738 | **714.8485** | **1427.6825** | **1427.6980** | **-10.85** | **1** | **14** | **0.46** | **1** |  | **KAQQHIETCWK** |
|  | 2955 | **505.2488** | **1512.7247** | **1512.7534** | **-18.98** | **0** | **14** | **0.5** | **1** |  | **VLSSPAEFFELMK + Oxidation (M)** |
|  | 2340 | **660.8320** | **1319.6495** | **1319.6470** | **1.93** | **0** | **14** | **0.71** | **1** |  | **AQGISAEPQTYR** |
|  | 3179 | **804.4082** | **1606.8018** | **1606.8315** | **-18.43** | **1** | **14** | **0.63** | **1** |  | **LGYSIPSRSGASGLDK** |
|  | 1448 | **368.1888** | **1101.5444** | **1101.5567** | **-11.15** | **1** | **14** | **0.63** | **1** |  | **TPVNWKDSR** |
|  | 102 | **382.2083** | **762.4021** | **762.4058** | **-4.86** | **1** | **14** | **1.1** | **1** |  | **LKMAER + Oxidation (M)** |
|  | 451 | **435.7559** | **869.4972** | **869.4971** | **0.16** | **0** | **14** | **0.33** | **1** |  | **VVDVSVPR** |
|  | 605 | **453.7499** | **905.4853** | **905.4971** | **-12.95** | **1** | **14** | **0.66** | **1** |  | **TAFPASGKK** |
|  | 4190 | **747.3784** | **2239.1134** | **2239.1347** | **-9.50** | **0** | **14** | **0.44** | **1** |  | **GSLSSYAYTLMVLYFLQQR** |
|  | 4312 | **787.6884** | **2360.0434** | **2360.0325** | **4.61** | **0** | **14** | **0.23** | **1** |  | **TLEEVVMAEEEDEGTDRPGSPA** |
|  | 2137 | **634.8321** | **1267.6497** | **1267.6642** | **-11.36** | **1** | **14** | **0.58** | **1** |  | **LVRMHAICPR + Oxidation (M)** |
|  | 3933 | **998.0352** | **1994.0559** | **1994.0585** | **-1.32** | **0** | **14** | **0.37** | **1** |  | **APTLPDTLQGSLLWAAGQR** |
|  | 1143 | **523.2856** | **1044.5566** | **1044.5716** | **-14.36** | **0** | **14** | **0.85** | **1** |  | **LSSAHVYLR** |
|  | 3428 | **866.9523** | **1731.8900** | **1731.8904** | **-0.23** | **1** | **14** | **0.57** | **1** |  | **FEQEAVAVDSNLRVR** |
|  | 4360 | **825.0702** | **2472.1887** | **2472.1561** | **13.2** | **0** | **13** | **0.36** | **1** |  | **YLQMVGMYASSYMILAMTLDR + Oxidation (M)** |
|  | 2165 | **425.5378** | **1273.5915** | **1273.6150** | **-18.43** | **0** | **13** | **0.42** | **1** |  | **ESGPSGIETELR** |
|  | 1145 | **523.2856** | **1044.5566** | **1044.5716** | **-14.36** | **0** | **13** | **0.86** | **1** |  | **LSSAHVYLR** |
|  | 2655 | **703.8767** | **1405.7389** | **1405.7540** | **-10.78** | **0** | **13** | **0.66** | **1** |  | **FQCIVFLNIPR** |
|  | 2425 | 673.8528 | 1345.6910 | 1345.6837 | 5.40 | 0 | 13 | 0.88 | 1 |  | LGLGDSNNQSTLK |
|  | 2958 | **505.9201** | **1514.7384** | **1514.7623** | **-15.82** | **1** | **13** | **0.57** | **1** |  | **LNQPGRTEGQLMR + Oxidation (M)** |
|  | 2730 | **714.3990** | **1426.7835** | **1426.7892** | **-3.96** | **1** | **13** | **0.61** | **1** |  | **LKLQSNSNTGIPR** |
|  | 690 | **465.7785** | **929.5425** | **929.5294** | **14.1** | **0** | **13** | **0.64** | **1** |  | **LVSSVAAQR** |
|  | 783 | **478.7462** | **955.4777** | **955.4835** | **-6.06** | **0** | **13** | **0.54** | **1** |  | **QAQATGAGPR** |
|  | 1383 | 544.7913 | 1087.5681 | 1087.5873 | -17.69 | 1 | 13 | 1 | 1 |  | LGLVNDKDSK |
|  | 3895 | **486.4758** | **1941.8740** | **1941.9089** | **-17.96** | **0** | **13** | **0.31** | **1** |  | **DMATETDASLSTLLTETK + Oxidation (M)** |
|  | 1896 | **601.8119** | **1201.6092** | **1201.6125** | **-2.73** | **0** | **13** | **0.9** | **1** |  | **LSVSNMVHTAK + Oxidation (M)** |
|  | 2432 | **674.3455** | **1346.6764** | **1346.6904** | **-10.42** | **0** | **13** | **0.81** | **1** |  | **TWLEEQGMILK** |
|  | 4065 | **1057.5176** | **2113.0206** | **2112.9937** | **12.7** | **0** | **13** | **0.51** | **1** |  | **GAPGPDGNNGAQGPPGPQGVQGGK** |
|  | 4265 | **767.3917** | **2299.1533** | **2299.1597** | **-2.76** | **0** | **13** | **0.49** | **1** |  | **VSWRPPADDGGADILGYILER** |
|  | 775 | **477.8027** | **953.5908** | **953.6022** | **-11.97** | **1** | **13** | **0.19** | **1** |  | **GKVVSVLPR** |
|  | 2535 | **689.3438** | **1376.6731** | **1376.6653** | **5.67** | **1** | **13** | **0.7** | **1** |  | **MAAQRGMPSSAVR + Oxidation (M)** |
|  | 3715 | **617.9504** | **1850.8293** | **1850.8534** | **-13.01** | **0** | **13** | **0.32** | **1** |  | **SGEGQEDAGELDFSGLLK** |
|  | 898 | **495.2731** | **988.5317** | **988.5276** | **4.12** | **1** | **13** | **0.96** | **1** |  | **AISFHMKR** |
|  | 2604 | **697.3935** | **1392.7724** | **1392.7586** | **9.96** | **1** | **13** | **0.37** | **1** |  | **RPRSLQESPAPR** |
|  | 2607 | **697.8622** | **1393.7098** | **1393.6945** | **11.0** | **1** | **13** | **0.75** | **1** |  | **NTMALMDLDVKK + Oxidation (M)** |
|  | 2213 | **644.8518** | **1287.6891** | **1287.6670** | **17.1** | **0** | **13** | **0.76** | **1** |  | **AQSIIDANDTLK** |
|  | 5 | **353.6973** | **705.3801** | **705.3843** | **-6.00** | **0** | **13** | **0.92** | **1** |  | **TMLAVR + Oxidation (M)** |
|  | 3894 | 486.4757 | 1941.8739 | 1941.9089 | -18.03 | 0 | 13 | 0.33 | 1 |  | DMATETDASLSTLLTETK + Oxidation (M) |
|  | 3620 | **902.4619** | **1802.9093** | **1802.8985** | **5.97** | **1** | **13** | **0.65** | **1** |  | **KQVIYMDAPAPENGVR + Oxidation (M)** |
|  | 452 | **435.7559** | **869.4973** | **869.4971** | **0.34** | **0** | **13** | **0.39** | **1** |  | **SIVAVEPR** |
|  | 2862 | **735.8566** | **1469.6986** | **1469.6933** | **3.63** | **1** | **13** | **0.53** | **1** |  | **MSADSRLGYEVSR** |
|  | 3470 | 876.4044 | 1750.7942 | 1750.7767 | 9.98 | 1 | 13 | 0.46 | 1 |  | GWDQGLLGMCEGEKR + Oxidation (M) |
|  | 3703 | **922.9119** | **1843.8093** | **1843.8015** | **4.22** | **1** | **13** | **0.23** | **1** |  | **CIAFGMKQDSVQCER + Oxidation (M)** |
|  | 1425 | **548.2915** | **1094.5684** | **1094.5760** | **-6.95** | **0** | **13** | **0.69** | **1** |  | **QVIQEFFGK** |
|  | 4274 | **772.3383** | **2313.9931** | **2314.0205** | **-11.84** | **1** | **13** | **0.16** | **1** |  | **DPQRSGDLYTQAAEAAMEAMK + 2 Oxidation (M)** |
|  | 832 | **486.7992** | **971.5838** | **971.5763** | **7.71** | **0** | **13** | **0.6** | **1** |  | **AAAIASSLIR** |
|  | 3483 | **877.9040** | **1753.7935** | **1753.7809** | **7.24** | **0** | **13** | **0.47** | **1** |  | **YDFVDVYNGHANGQR** |
|  | 3112 | **525.2708** | **1572.7906** | **1572.7678** | **14.5** | **1** | **13** | **0.84** | **1** |  | **AQMLREAAEAGAEAR** |
|  | 998 | **508.2244** | **1014.4342** | **1014.4151** | **18.9** | **0** | **13** | **0.19** | **1** |  | **ADMLDFCK + Oxidation (M)** |
|  | 2427 | **673.8817** | **1345.7489** | **1345.7275** | **15.9** | **0** | **13** | **0.58** | **1** |  | **NGTILVDNMLIK + Oxidation (M)** |
|  | 1478 | **554.7797** | **1107.5449** | **1107.5457** | **-0.71** | **0** | **13** | **0.73** | **1** |  | **LGMMPLWTK + 2 Oxidation (M)** |
|  | 2794 | **482.2794** | **1443.8163** | **1443.8198** | **-2.43** | **0** | **13** | **0.36** | **1** |  | **LSGYIERPQLIR** |
|  | 2444 | **451.6177** | **1351.8311** | **1351.8299** | **0.88** | **1** | **13** | **0.074** | **1** |  | **AGAELRAVLALLR** |
|  | 1641 | **571.7549** | **1141.4953** | **1141.4856** | **8.54** | **1** | **13** | **0.23** | **1** |  | **MRAGGMSDSSK + Oxidation (M)** |
|  | 2676 | **472.2176** | **1413.6309** | **1413.6121** | **13.4** | **0** | **13** | **0.32** | **1** |  | **GTEEGPGTSHTEGR** |
|  | 1235 | **529.3040** | **1056.5935** | **1056.5927** | **0.72** | **1** | **13** | **0.67** | **1** |  | **GILNEIKDR** |
|  | 1825 | **595.8212** | **1189.6279** | **1189.6204** | **6.33** | **1** | **13** | **0.95** | **1** |  | **QRYVPGDTVR** |
|  | 4070 | **706.0184** | **2115.0335** | **2115.0484** | **-7.06** | **1** | **13** | **0.64** | **1** |  | **TQDEILFSNSTRLSFETK** |
|  | 413 | **428.7660** | **855.5174** | **855.5178** | **-0.48** | **0** | **13** | **0.54** | **1** |  | **SPLSLIAR** |
|  | 2737 | **357.9277** | **1427.6816** | **1427.6645** | **12.0** | **0** | **13** | **0.63** | **1** |  | **TMMLMLCDLLR + 2 Oxidation (M)** |
|  | 2857 | 734.3505 | 1466.6865 | 1466.7010 | -9.89 | 1 | 13 | 0.55 | 1 |  | YSGMMETVHIRK + Oxidation (M) |
|  | 2272 | **652.8254** | **1303.6363** | **1303.6368** | **-0.35** | **1** | **13** | **0.87** | **1** |  | **GETLSQEEQRK** |
|  | 1418 | **547.3159** | **1092.6172** | **1092.6152** | **1.80** | **1** | **13** | **0.36** | **1** |  | **APTLATAHRR** |
|  | 2101 | **629.3329** | **1256.6513** | **1256.6612** | **-7.87** | **0** | **13** | **0.88** | **1** |  | **TQALTEVDVPGK** |
|  | 1746 | **585.7901** | **1169.5656** | **1169.5564** | **7.90** | **0** | **13** | **0.61** | **1** |  | **EPDSEQQPLK** |
|  | 2370 | **665.8213** | **1329.6280** | **1329.6273** | **0.53** | **0** | **13** | **0.67** | **1** |  | **SSPGSVAASPSGGGGR** |
|  | 2170 | **639.3405** | **1276.6665** | **1276.6445** | **17.2** | **0** | **13** | **0.89** | **1** |  | **TGTINGSLLCNK** |
|  | 638 | **458.7640** | **915.5135** | **915.5025** | **11.9** | **0** | **13** | **1.1** | **1** |  | **LVLTEGER** |
|  | 4084 | **1074.0709** | **2146.1273** | **2146.0881** | **18.2** | **1** | **13** | **0.5** | **1** |  | **GFGKMFVSSSGLPPSPVPSPR + Oxidation (M)** |
|  | 612 | **454.7289** | **907.4433** | **907.4399** | **3.70** | **0** | **13** | **0.81** | **1** |  | **AEADAYIR** |
|  | 111 | **384.2355** | **766.4564** | **766.4701** | **-17.82** | **0** | **13** | **0.45** | **1** |  | **ALSHLVK** |
|  | 322 | **421.7582** | **841.5017** | **841.5021** | **-0.46** | **1** | **13** | **0.57** | **1** |  | **VAGAATPKK** |
|  | 285 | **421.7576** | **841.5006** | **841.5022** | **-1.79** | **0** | **13** | **0.61** | **1** |  | **AVSVTPIR** |
|  | 4526 | **1142.1574** | **3423.4502** | **3423.3869** | **18.5** | **1** | **12** | **0.082** | **1** |  | **ESFCRGFPMSGCELETSCCVCHSTALGER** |
|  | 1765 | **588.3203** | **1174.6261** | **1174.6194** | **5.70** | **0** | **12** | **1** | **1** |  | **TSAGLGLSLDGGK** |
|  | 503 | **436.7751** | **871.5356** | **871.5239** | **13.4** | **1** | **12** | **0.56** | **1** |  | **QLIKSQR** |
|  | 2560 | **693.3929** | **1384.7712** | **1384.7966** | **-18.33** | **1** | **12** | **0.59** | **1** |  | **EPSVLFEVPKLK** |
|  | 1949 | **406.5546** | **1216.6420** | **1216.6234** | **15.3** | **1** | **12** | **1** | **1** |  | **MEDNKQLALR** |
|  | 183 | **401.7425** | **801.4704** | **801.4709** | **-0.61** | **0** | **12** | **1.2** | **1** |  | **LGSAVVTR** |
|  | 203 | 406.7539 | 811.4933 | 811.4804 | 15.9 | 0 | 12 | 0.32 | 1 |  | AVTPVPTK |
|  | 1414 | **547.2802** | **1092.5457** | **1092.5597** | **-12.79** | **0** | **12** | **1** | **1** |  | **MQSTLISAAR + Oxidation (M)** |
|  | 2608 | **697.8628** | **1393.7110** | **1393.6945** | **11.9** | **1** | **12** | **0.94** | **1** |  | **NTMALMDLDVKK + Oxidation (M)** |
|  | 893 | **494.2962** | **986.5778** | **986.5648** | **13.2** | **0** | **12** | **0.73** | **1** |  | **LLETPISSK** |
|  | 2114 | **420.5627** | **1258.6662** | **1258.6670** | **-0.62** | **0** | **12** | **0.88** | **1** |  | **IPLQPHGPGLGSS** |
|  | 2341 | **660.8390** | **1319.6634** | **1319.6622** | **0.89** | **0** | **12** | **1.1** | **1** |  | **GVVFPYQSPNGR** |
|  | 2561 | **693.3942** | **1384.7738** | **1384.7966** | **-16.46** | **1** | **12** | **0.6** | **1** |  | **EPSVLFEVPKLK** |
|  | 269 | **416.7463** | **831.4781** | **831.4637** | **17.4** | **0** | **12** | **0.92** | **1** |  | **VAIMVSGR** |
|  | 1121 | **523.2840** | **1044.5534** | **1044.5386** | **14.2** | **0** | **12** | **1.1** | **1** |  | **LGVNDCVLR** |
|  | 299 | **421.7580** | **841.5015** | **841.5022** | **-0.76** | **0** | **12** | **0.61** | **1** |  | **AVSVTPIR** |
|  | 721 | **468.7346** | **935.4546** | **935.4494** | **5.54** | **1** | **12** | **0.89** | **1** |  | **LRMDTER + Oxidation (M)** |
|  | 3044 | **771.8769** | **1541.7392** | **1541.7395** | **-0.21** | **0** | **12** | **0.68** | **1** |  | **DVPNSQPEMVEAVK** |
|  | 3743 | **934.4210** | **1866.8275** | **1866.8101** | **9.31** | **0** | **12** | **0.37** | **1** |  | **NIHTGEKPHSCNECGK** |
|  | 906 | **496.2427** | **990.4709** | **990.4804** | **-9.63** | **0** | **12** | **0.92** | **1** |  | **GEASLEVMR** |
|  | 3514 | **883.8946** | **1765.7746** | **1765.7433** | **17.7** | **1** | **12** | **0.33** | **1** |  | **EECDRLGPGMADICK + Oxidation (M)** |
|  | 3864 | **642.6310** | **1924.8713** | **1924.9097** | **-19.93** | **1** | **12** | **0.42** | **1** |  | **GPMWSVLLNTEEMKMK + 2 Oxidation (M)** |
|  | 3898 | **650.9913** | **1949.9520** | **1949.9378** | **7.29** | **0** | **12** | **0.71** | **1** |  | **GNTGIIHGACLTLTDHDR** |
|  | 396 | **424.2404** | **846.4663** | **846.4811** | **-17.47** | **0** | **12** | **1.1** | **1** |  | **SAVASSVVK** |
|  | 573 | **450.2688** | **898.5230** | **898.5097** | **14.9** | **1** | **12** | **0.68** | **1** |  | **AQGAQRLR** |
|  | 3540 | 594.2712 | 1779.7919 | 1779.8136 | -12.20 | 0 | 12 | 0.37 | 1 |  | QDVQGPRPGSPGDAEDR |
|  | 2107 | **420.2419** | **1257.7038** | **1257.6969** | **5.52** | **0** | **12** | **0.68** | **1** |  | **VATYLPAPEGLK** |
|  | 3360 | **851.3925** | **1700.7704** | **1700.7724** | **-1.22** | **0** | **12** | **0.38** | **1** |  | **MFMGDSCPYLAGKPK** |
|  | 336 | **421.7582** | **841.5019** | **841.5022** | **-0.34** | **0** | **12** | **0.63** | **1** |  | **AVSVTPIR** |
|  | 1948 | **609.2803** | **1216.5461** | **1216.5394** | **5.53** | **0** | **12** | **0.38** | **1** |  | **DMLDSHLENK + Oxidation (M)** |
|  | 2162 | 637.3137 | 1272.6129 | 1272.6098 | 2.39 | 0 | 12 | 0.82 | 1 |  | SLAASSSFYGQR |
|  | 607 | **453.7557** | **905.4967** | **905.4793** | **19.3** | **0** | **12** | **0.92** | **1** |  | **KPMLFDR** |
|  | 347 | **421.7582** | **841.5019** | **841.5022** | **-0.27** | **0** | **12** | **0.64** | **1** |  | **AVSVTPIR** |
|  | 1120 | **523.2839** | **1044.5532** | **1044.5716** | **-17.63** | **0** | **12** | **1.2** | **1** |  | **LSSAHVYLR** |
|  | 3039 | **771.4411** | **1540.8676** | **1540.8548** | **8.34** | **0** | **12** | **0.4** | **1** |  | **RPPGDIMFLLVQR** |
|  | 319 | **421.7582** | **841.5017** | **841.5022** | **-0.48** | **0** | **12** | **0.65** | **1** |  | **AVSVTPIR** |
|  | 855 | **489.2476** | **976.4806** | **976.4872** | **-6.82** | **1** | **12** | **1.2** | **1** |  | **VASMNQRR + Oxidation (M)** |
|  | 2232 | **647.7830** | **1293.5514** | **1293.5442** | **5.56** | **1** | **12** | **0.31** | **1** |  | **KQIDQQNCTC** |
|  | 2638 | **701.9118** | **1401.8090** | **1401.7953** | **9.81** | **1** | **12** | **0.33** | **1** |  | **ERHRPLVVPGSR** |
|  | 574 | **450.2690** | **898.5234** | **898.5348** | **-12.72** | **1** | **12** | **0.72** | **1** |  | **RDVQLIR** |
|  | 313 | **421.7581** | **841.5017** | **841.5022** | **-0.55** | **0** | **12** | **0.66** | **1** |  | **AVSVTPIR** |
|  | 1532 | **560.7859** | **1119.5572** | **1119.5594** | **-1.94** | **0** | **12** | **1.2** | **1** |  | **ISSDMPVSLR + Oxidation (M)** |
|  | 2042 | **621.8081** | **1241.6017** | **1241.5863** | **12.4** | **0** | **12** | **0.85** | **1** |  | **AEMFLSQFNR** |
|  | 4067 | **705.3630** | **2113.0673** | **2113.0666** | **0.33** | **1** | **12** | **0.81** | **1** |  | **EIYIMAANYLQSLDWRK** |
|  | 2106 | **629.8541** | **1257.6936** | **1257.7115** | **-14.25** | **0** | **12** | **0.84** | **1** |  | **MILLTLTGQPR + Oxidation (M)** |
|  | 453 | **435.7560** | **869.4975** | **869.5083** | **-12.44** | **0** | **12** | **0.51** | **1** |  | **VVQANALR** |
|  | 1050 | **514.2599** | **1026.5052** | **1026.5134** | **-8.00** | **0** | **12** | **0.78** | **1** |  | **AGELSFFTR** |
|  | 2786 | **720.8159** | **1439.6173** | **1439.6277** | **-7.21** | **1** | **12** | **0.24** | **1** |  | **SSSYSSYSSRSSR** |
|  | 3118 | **788.8868** | **1575.7590** | **1575.7450** | **8.88** | **0** | **12** | **0.82** | **1** |  | **QEALMVLEEEDVR + Oxidation (M)** |
|  | 3167 | 800.3972 | 1598.7799 | 1598.7684 | 7.19 | 1 | 12 | 0.77 | 1 |  | ECIEFLKEASVMK + Oxidation (M) |
|  | 4051 | **700.0109** | **2097.0108** | **2097.0491** | **-18.27** | **0** | **12** | **0.72** | **1** |  | **ATYIQTIEEGINTHTHAAK** |
|  | 3590 | **598.9430** | **1793.8073** | **1793.7787** | **16.0** | **0** | **12** | **0.5** | **1** |  | **FMVDIACGMEYLSSR + Oxidation (M)** |
|  | 541 | **443.2615** | **884.5084** | **884.5080** | **0.50** | **0** | **12** | **0.72** | **1** |  | **AALSATVPR** |
|  | 2347 | **662.3112** | **1322.6079** | **1322.6024** | **4.16** | **1** | **12** | **0.7** | **1** |  | **MPSDSDDSLTKK** |
|  | 3809 | **633.6245** | **1897.8515** | **1897.8853** | **-17.81** | **1** | **12** | **0.45** | **1** |  | **APRHEGMTNGDSGFLPGR** |
|  | 3861 | **481.9787** | **1923.8858** | **1923.8706** | **7.91** | **1** | **12** | **0.61** | **1** |  | **AEMYSEYLSTCSKLAR + Oxidation (M)** |
|  | 569 | 449.7770 | 897.5395 | 897.5396 | -0.13 | 1 | 12 | 0.4 | 1 |  | AKITNVPR |
|  | 2804 | **483.5713** | **1447.6921** | **1447.7195** | **-18.88** | **0** | **12** | **0.94** | **1** |  | **QGILSFDLSDPEK** |
|  | 289 | **421.7579** | **841.5012** | **841.5021** | **-1.11** | **1** | **12** | **0.72** | **1** |  | **ELKSIPR** |
|  | 3003 | **766.3441** | **1530.6737** | **1530.6798** | **-3.98** | **1** | **12** | **0.34** | **1** |  | **KENADQSDPEDVGK** |
|  | 3111 | **394.2045** | **1572.7890** | **1572.7896** | **-0.38** | **1** | **12** | **1.1** | **1** |  | **LSEGFSIHTRDSPK** |
|  | 2479 | **679.8546** | **1357.6947** | **1357.6950** | **-0.23** | **1** | **12** | **1.1** | **1** |  | **EVEQSRQEVVR** |
|  | 393 | **424.1972** | **846.3799** | **846.3719** | **9.46** | **0** | **12** | **0.56** | **1** |  | **EASGGDSPK** |
|  | 2405 | **671.3412** | **1340.6678** | **1340.6733** | **-4.09** | **0** | **12** | **0.99** | **1** |  | **RPMNAFMLFAK + Oxidation (M)** |
|  | 437 | **433.7342** | **865.4539** | **865.4658** | **-13.65** | **0** | **12** | **1** | **1** |  | **GIYGTISR** |
|  | 2108 | **629.8592** | **1257.7038** | **1257.7016** | **1.78** | **1** | **12** | **0.75** | **1** |  | **QLMVHAFIKR + Oxidation (M)** |
|  | 291 | **421.7579** | **841.5013** | **841.5022** | **-1.00** | **0** | **12** | **0.68** | **1** |  | **AVSVTPIR** |
|  | 1451 | **551.7949** | **1101.5753** | **1101.5891** | **-12.49** | **1** | **12** | **1.4** | **1** |  | **AVRVSTEANR** |
|  | 363 | **421.7584** | **841.5022** | **841.5021** | **0.04** | **1** | **12** | **0.68** | **1** |  | **VAGAATPKK** |
|  | 160 | **396.7343** | **791.4540** | **791.4575** | **-4.40** | **1** | **12** | **0.75** | **1** |  | **IGSMKIK + Oxidation (M)** |
|  | 300 | **421.7580** | **841.5015** | **841.5022** | **-0.76** | **0** | **12** | **0.69** | **1** |  | **AVSVTPIR** |
|  | 1754 | **586.7802** | **1171.5459** | **1171.5258** | **17.1** | **0** | **12** | **0.6** | **1** |  | **ETSFNQAYGR** |
|  | 2339 | **440.8901** | **1319.6484** | **1319.6470** | **1.10** | **0** | **12** | **1.1** | **1** |  | **TNSNAITSWAQK** |
|  | 2442 | 676.8591 | 1351.7037 | 1351.7096 | -4.34 | 0 | 12 | 0.89 | 1 |  | GANILLTDHGDVK |
|  | 296 | **421.7580** | **841.5014** | **841.5022** | **-0.91** | **0** | **12** | **0.69** | **1** |  | **AVSVTPIR** |
|  | 581 | **450.7599** | **899.5052** | **899.5076** | **-2.74** | **0** | **12** | **1.1** | **1** |  | **EVDAVVLR** |
|  | 1389 | **544.8130** | **1087.6115** | **1087.6172** | **-5.18** | **1** | **12** | **0.88** | **1** |  | **KICTLGLQR** |
|  | 364 | **421.7584** | **841.5022** | **841.5021** | **0.11** | **1** | **12** | **0.69** | **1** |  | **VAGAATPKK** |
|  | 697 | **467.2364** | **932.4582** | **932.4498** | **9.06** | **1** | **12** | **1.3** | **1** |  | **MADRTAPR + Oxidation (M)** |
|  | 1349 | **540.8149** | **1079.6153** | **1079.6128** | **2.38** | **1** | **12** | **0.52** | **1** |  | **VPLGTKFYR** |
|  | 1817 | **594.8283** | **1187.6419** | **1187.6220** | **16.8** | **0** | **12** | **1.1** | **1** |  | **QILMENAELK** |
|  | 3995 | **677.6388** | **2029.8945** | **2029.8941** | **0.22** | **1** | **12** | **0.36** | **1** |  | **MSLQMKMDCQEQQLTK + 2 Oxidation (M)** |
|  | 4373 | **836.6840** | **2507.0302** | **2507.0320** | **-0.72** | **1** | **12** | **0.079** | **1** |  | **FDALKDDDSGDHDQNEENSTQK** |
|  | 338 | **421.7582** | **841.5019** | **841.5022** | **-0.34** | **0** | **12** | **0.71** | **1** |  | **AVSVTPIR** |
|  | 2291 | **436.8898** | **1307.6475** | **1307.6689** | **-16.41** | **1** | **12** | **1.1** | **1** |  | **LRIIMEQSMR + 2 Oxidation (M)** |
|  | 369 | **421.7585** | **841.5025** | **841.5022** | **0.45** | **0** | **12** | **0.71** | **1** |  | **AVSVTPIR** |
|  | 3037 | **514.6293** | **1540.8660** | **1540.8460** | **13.0** | **0** | **12** | **0.46** | **1** |  | **EAALSQEQIQILAK** |
|  | 3771 | **938.9004** | **1875.7863** | **1875.7979** | **-6.16** | **0** | **12** | **0.14** | **1** |  | **SSSLDTEGCFQTGLCSK** |
|  | 1199 | **526.2640** | **1050.5135** | **1050.5202** | **-6.32** | **0** | **12** | **1.1** | **1** |  | **ITGNMGLAMK + Oxidation (M)** |
|  | 145 | **393.2468** | **784.4790** | **784.4919** | **-16.38** | **1** | **12** | **0.89** | **1** |  | **ILAAKGGR** |
|  | 1810 | 395.9031 | 1184.6873 | 1184.6666 | 17.5 | 1 | 12 | 0.67 | 1 |  | LGSLFVKHER |
|  | 3170 | **800.9128** | **1599.8110** | **1599.8216** | **-6.64** | **1** | **12** | **0.87** | **1** |  | **IELSEEGAEGRVVGR** |
|  | 644 | **458.7878** | **915.5609** | **915.5575** | **3.72** | **0** | **12** | **0.25** | **1** |  | **VLLTMIAR** |
|  | 1356 | **541.7509** | **1081.4871** | **1081.5082** | **-19.50** | **1** | **12** | **0.67** | **1** |  | **AVVCKACMK + Oxidation (M)** |
|  | 1585 | **565.7687** | **1129.5228** | **1129.5363** | **-11.99** | **1** | **12** | **0.65** | **1** |  | **KEDQEATGPR** |
|  | 3183 | **804.9011** | **1607.7876** | **1607.7712** | **10.2** | **1** | **12** | **1.2** | **1** |  | **ESMAVLEKQDISDK + Oxidation (M)** |
|  | 329 | **421.7582** | **841.5018** | **841.5134** | **-13.74** | **1** | **12** | **0.72** | **1** |  | **AVDLIRR** |
|  | 6 | **353.6974** | **705.3802** | **705.3843** | **-5.83** | **0** | **12** | **1.3** | **1** |  | **TMLAVR + Oxidation (M)** |
|  | 469 | **435.7740** | **869.5334** | **869.5487** | **-17.64** | **1** | **11** | **0.5** | **1** |  | **VVKFLHK** |
|  | 1402 | **546.2902** | **1090.5659** | **1090.5553** | **9.67** | **1** | **11** | **1.4** | **1** |  | **VGICGRTGSGK** |
|  | 1569 | **564.3189** | **1126.6231** | **1126.6346** | **-10.18** | **0** | **11** | **0.6** | **1** |  | **TQPTVINNLK** |
|  | 2349 | 662.3392 | 1322.6638 | 1322.6500 | 10.4 | 1 | 11 | 1 | 1 |  | EMSNSKELTLR + Oxidation (M) |
|  | 776 | **478.2451** | **954.4757** | **954.4631** | **13.2** | **1** | **11** | **0.87** | **1** |  | **HETGERAR** |
|  | 1222 | **528.2773** | **1054.5401** | **1054.5560** | **-15.02** | **0** | **11** | **0.91** | **1** |  | **LWGIPDQAR** |
|  | 1299 | **535.2635** | **1068.5124** | **1068.5087** | **3.45** | **0** | **11** | **0.75** | **1** |  | **ELTSVYDSR** |
|  | 3432 | **867.9089** | **1733.8032** | **1733.7791** | **13.9** | **1** | **11** | **0.65** | **1** |  | **HSMREEDFIQPSSR + Oxidation (M)** |
|  | 2817 | **725.8821** | **1449.7496** | **1449.7497** | **-0.08** | **1** | **11** | **1.2** | **1** |  | **KSVSTPVSAQMTSK** |
|  | 3046 | **771.9334** | **1541.8521** | **1541.8314** | **13.4** | **1** | **11** | **0.76** | **1** |  | **VLGDLIFNQPDRR** |
|  | 2961 | **505.9214** | **1514.7423** | **1514.7260** | **10.8** | **1** | **11** | **0.9** | **1** |  | **ESVSRMPVSSQHR + Oxidation (M)** |
|  | 664 | **462.2452** | **922.4759** | **922.4616** | **15.5** | **0** | **11** | **1** | **1** |  | **MSVACVLK + Oxidation (M)** |
|  | 2644 | **468.5783** | **1402.7132** | **1402.6940** | **13.7** | **0** | **11** | **1.2** | **1** |  | **QTPTVVESAETNK** |
|  | 2800 | **724.3548** | **1446.6950** | **1446.7150** | **-13.79** | **1** | **11** | **0.92** | **1** |  | **AFSRLSSLCNHR** |
|  | 3407 | 862.3850 | 1722.7553 | 1722.7238 | 18.3 | 0 | 11 | 0.33 | 1 |  | FEEMCGLVMGMFTR + Oxidation (M) |
|  | 492 | **435.7750** | **869.5355** | **869.5487** | **-15.20** | **1** | **11** | **0.52** | **1** |  | **VVKFLHK** |
|  | 2064 | **625.8245** | **1249.6344** | **1249.6197** | **11.7** | **1** | **11** | **1.1** | **1** |  | **TMTGGRAQSIGR + Oxidation (M)** |
|  | 1459 | **552.2881** | **1102.5617** | **1102.5618** | **-0.07** | **1** | **11** | **1.5** | **1** |  | **EKSQDEVLR** |
|  | 1291 | **534.7673** | **1067.5201** | **1067.4996** | **19.2** | **0** | **11** | **0.94** | **1** |  | **SVFSQSGNSR** |
|  | 4331 | **803.9937** | **2408.9591** | **2408.9966** | **-15.57** | **1** | **11** | **0.073** | **1** |  | **SPDTYSRGHDSSSQSDHYGGGGR** |
|  | 3149 | **796.8690** | **1591.7235** | **1591.7413** | **-11.17** | **1** | **11** | **0.52** | **1** |  | **RAPEPSAGTFQEMR + Oxidation (M)** |
|  | 3401 | **573.9742** | **1718.9009** | **1718.8736** | **15.9** | **1** | **11** | **0.88** | **1** |  | **TPPPMAPKTPPPMTPK + 2 Oxidation (M)** |
|  | 346 | **421.7582** | **841.5019** | **841.5021** | **-0.25** | **1** | **11** | **0.75** | **1** |  | **VAGAATPKK** |
|  | 2907 | **746.8855** | **1491.7564** | **1491.7430** | **9.01** | **1** | **11** | **1.2** | **1** |  | **GPGQKTGPDPASAPGR** |
|  | 3873 | **644.6245** | **1930.8515** | **1930.8189** | **16.9** | **1** | **11** | **0.39** | **1** |  | **SFHSEKIYQCTECDK** |
|  | 740 | **471.2927** | **940.5709** | **940.5818** | **-11.59** | **1** | **11** | **0.47** | **1** |  | **VVVRNLNK** |
|  | 2912 | **498.5667** | **1492.6783** | **1492.6715** | **4.56** | **1** | **11** | **0.79** | **1** |  | **KMEESDEEAVQAK** |
|  | 373 | **421.7589** | **841.5032** | **841.5021** | **1.27** | **1** | **11** | **0.75** | **1** |  | **VAGAATPKK** |
|  | 4157 | **738.3624** | **2212.0655** | **2212.0980** | **-14.70** | **1** | **11** | **0.75** | **1** |  | **VHSSMGAPEIRMSKPLEAEK + Oxidation (M)** |
|  | 610 | **454.2316** | **906.4486** | **906.4559** | **-8.09** | **1** | **11** | **1.4** | **1** |  | **RDSSAFPK** |
|  | 290 | **421.7579** | **841.5012** | **841.5021** | **-1.04** | **1** | **11** | **0.76** | **1** |  | **ELKSIPR** |
|  | 3594 | **897.9114** | **1793.8083** | **1793.8195** | **-6.24** | **0** | **11** | **0.59** | **1** |  | **LETMYNHWTWELR + Oxidation (M)** |
|  | 3176 | **803.4460** | **1604.8775** | **1604.8774** | **0.10** | **0** | **11** | **0.63** | **1** |  | **QLLQQLVTSYPSTK** |
|  | 314 | **421.7581** | **841.5017** | **841.5021** | **-0.51** | **1** | **11** | **0.77** | **1** |  | **ELKSIPR** |
|  | 2122 | **631.8432** | **1261.6718** | **1261.6886** | **-13.29** | **1** | **11** | **1.1** | **1** |  | **KMVNILMANTK** |
|  | 375 | 421.7591 | 841.5036 | 841.5021 | 1.77 | 1 | 11 | 0.76 | 1 |  | VAGAATPKK |
|  | 1819 | **595.3139** | **1188.6132** | **1188.6285** | **-12.80** | **1** | **11** | **1.5** | **1** |  | **KLLGMQQESR** |
|  | 1243 | **530.2576** | **1058.5007** | **1058.4992** | **1.39** | **0** | **11** | **0.77** | **1** |  | **GLSNGEETPR** |
|  | 4143 | **737.7063** | **2210.0971** | **2210.0637** | **15.1** | **1** | **11** | **0.86** | **1** |  | **DDPDGKQEAKPQQAAGMLSPK** |
|  | 164 | **399.2408** | **796.4671** | **796.4807** | **-17.07** | **0** | **11** | **0.46** | **1** |  | **AAKPSVPK** |
|  | 2840 | 730.8750 | 1459.7354 | 1459.7419 | -4.44 | 1 | 11 | 1.2 | 1 |  | SKNTIHEDTIFR |
|  | 292 | **421.7579** | **841.5013** | **841.5021** | **-0.97** | **1** | **11** | **0.78** | **1** |  | **ELKSIPR** |
|  | 883 | **492.7902** | **983.5659** | **983.5624** | **3.54** | **1** | **11** | **0.65** | **1** |  | **AAAAAARLNR** |
|  | 2815 | **484.2552** | **1449.7437** | **1449.7650** | **-14.70** | **1** | **11** | **1.2** | **1** |  | **CSLFLVEGAAAGKK** |
|  | 115 | **384.7324** | **767.4502** | **767.4402** | **13.1** | **1** | **11** | **0.51** | **1** |  | **HKSIQR** |
|  | 1769 | **588.8124** | **1175.6102** | **1175.6154** | **-4.44** | **1** | **11** | **1.5** | **1** |  | **NAEMARMLLK** |
|  | 3561 | **894.0070** | **1785.9994** | **1786.0141** | **-8.26** | **0** | **11** | **0.38** | **1** |  | **WLALGSLFALAGLLEGR** |
|  | 1699 | **580.7958** | **1159.5771** | **1159.5986** | **-18.48** | **0** | **11** | **1.4** | **1** |  | **GIDSWINLSR** |
|  | 4289 | **781.0661** | **2340.1765** | **2340.1644** | **5.14** | **1** | **11** | **0.81** | **1** |  | **QARLVLQPPSNMHETLDGYR + Oxidation (M)** |
|  | 3676 | **611.5914** | **1831.7523** | **1831.7842** | **-17.45** | **1** | **11** | **0.14** | **1** |  | **GDFRAQENVPSCCHR** |
|  | 4514 | **827.4374** | **3305.7207** | **3305.7190** | **0.49** | **1** | **11** | **0.31** | **1** |  | **SYDEAILHLVVAGLGSKPLISMRGHEDGGIR + Oxidation (M)** |
|  | 1914 | **603.8204** | **1205.6263** | **1205.6252** | **0.95** | **1** | **11** | **1.4** | **1** |  | **SSDSKPGVSSKK** |
|  | 1122 | **523.2841** | **1044.5536** | **1044.5386** | **14.3** | **0** | **11** | **1.5** | **1** |  | **LGVNDCVLR** |
|  | 2295 | **437.2172** | **1308.6299** | **1308.6198** | **7.77** | **0** | **11** | **0.97** | **1** |  | **QEGTATLYDSPK** |
|  | 1711 | **582.7983** | **1163.5821** | **1163.5903** | **-7.04** | **1** | **11** | **1.5** | **1** |  | **MRTVNAMAVR + Oxidation (M)** |
|  | 2103 | 629.8236 | 1257.6327 | 1257.6538 | -16.79 | 1 | 11 | 1.2 | 1 |  | ATSGRQTRPER |
|  | 609 | **454.2312** | **906.4478** | **906.4633** | **-17.12** | **0** | **11** | **1.5** | **1** |  | **MNISLWK + Oxidation (M)** |
|  | 989 | **506.7936** | **1011.5726** | **1011.5825** | **-9.80** | **1** | **11** | **0.68** | **1** |  | **VSPTSPRLR** |
|  | 2506 | **455.8968** | **1364.6685** | **1364.6837** | **-11.12** | **0** | **11** | **1.3** | **1** |  | **GDFPSPIHVSGPR** |
|  | 1286 | **534.2757** | **1066.5368** | **1066.5519** | **-14.15** | **0** | **11** | **1.2** | **1** |  | **AQQQDKPPR** |
|  | 657 | **460.7449** | **919.4753** | **919.4797** | **-4.77** | **0** | **11** | **1.3** | **1** |  | **AVSSQMIGK** |
|  | 362 | **421.7583** | **841.5021** | **841.5021** | **-0.02** | **1** | **11** | **0.81** | **1** |  | **ELKSIPR** |
|  | 374 | **421.7591** | **841.5036** | **841.5021** | **1.70** | **1** | **11** | **0.81** | **1** |  | **VAGAATPKK** |
|  | 642 | **458.7877** | **915.5609** | **915.5575** | **3.66** | **0** | **11** | **0.28** | **1** |  | **VLLTMIAR** |
|  | 1421 | **547.7781** | **1093.5417** | **1093.5226** | **17.5** | **0** | **11** | **1.2** | **1** |  | **GYLPGTAEMR** |
|  | 360 | **421.7583** | **841.5021** | **841.5021** | **-0.03** | **1** | **11** | **0.81** | **1** |  | **VAGAATPKK** |
|  | 787 | 478.7813 | 955.5480 | 955.5603 | -12.90 | 1 | 11 | 0.9 | 1 |  | KVTPAWVR |
|  | 1125 | **523.2845** | **1044.5544** | **1044.5386** | **15.1** | **0** | **11** | **1.7** | **1** |  | **LGVNDCVLR** |
|  | 3299 | **557.0001** | **1667.9784** | **1667.9570** | **12.8** | **1** | **11** | **0.21** | **1** |  | **TTLIKALTGDAAIQPR** |
|  | 1581 | **565.2843** | **1128.5540** | **1128.5636** | **-8.44** | **1** | **11** | **0.86** | **1** |  | **GASRSPSPQSR** |
|  | 356 | **421.7583** | **841.5020** | **841.5021** | **-0.16** | **1** | **11** | **0.82** | **1** |  | **ELKSIPR** |
|  | 365 | **421.7584** | **841.5022** | **841.5022** | **0.09** | **0** | **11** | **0.82** | **1** |  | **AVSVTPIR** |
|  | 2013 | **618.8201** | **1235.6257** | **1235.6398** | **-11.37** | **1** | **11** | **1.3** | **1** |  | **KVSITAYDPDK** |
|  | 2597 | **696.8465** | **1391.6784** | **1391.7045** | **-18.70** | **0** | **11** | **1.2** | **1** |  | **QLSESEYVGKPR** |
|  | 77 | **378.2654** | **754.5163** | **754.5065** | **13.0** | **0** | **11** | **0.14** | **1** |  | **ILAGLLR** |
|  | 2320 | **439.5704** | **1315.6893** | **1315.6806** | **6.63** | **0** | **11** | **1.5** | **1** |  | **LNGLADLEACIK** |
|  | 4094 | **719.6854** | **2156.0343** | **2156.0168** | **8.10** | **1** | **11** | **0.96** | **1** |  | **APPDAAPGLVASNCKSGSADSGK** |
|  | 1568 | **376.5479** | **1126.6220** | **1126.6207** | **1.16** | **1** | **11** | **0.72** | **1** |  | **NSGLRSIQPR** |
|  | 2393 | **446.9453** | **1337.8141** | **1337.8104** | **2.74** | **0** | **11** | **0.14** | **1** |  | **LPNALLMLVNLK** |
|  | 2184 | **641.8084** | **1281.6023** | **1281.6214** | **-14.95** | **0** | **11** | **0.92** | **1** |  | **NQEQWHVVSR** |
|  | 639 | **458.7873** | **915.5600** | **915.5575** | **2.72** | **0** | **11** | **0.29** | **1** |  | **VLLTMIAR** |
|  | 768 | **476.2854** | **950.5563** | **950.5410** | **16.1** | **1** | **11** | **0.56** | **1** |  | **AHARLDLR** |
|  | 2714 | **712.8401** | **1423.6656** | **1423.6427** | **16.1** | **0** | **11** | **1** | **1** |  | **SSDSDVSDVSAISR** |
|  | 4490 | **634.0927** | **3165.4272** | **3165.4819** | **-17.29** | **0** | **11** | **0.32** | **1** |  | **ETGEVVDCHLSDMLQQLHSVNASKPSER** |
|  | 1457 | **552.2873** | **1102.5601** | **1102.5618** | **-1.51** | **1** | **11** | **1.7** | **1** |  | **KELENLDSR** |
|  | 2082 | **418.9015** | **1253.6828** | **1253.6802** | **2.11** | **0** | **11** | **0.75** | **1** |  | **VNLVALETMHK** |
|  | 647 | **458.7882** | **915.5619** | **915.5575** | **4.79** | **0** | **11** | **0.3** | **1** |  | **VLLTMIAR** |
|  | 1146 | **523.2856** | **1044.5566** | **1044.5716** | **-14.36** | **0** | **11** | **1.6** | **1** |  | **LSSAHVYLR** |
|  | 2019 | **619.8089** | **1237.6032** | **1237.6125** | **-7.48** | **0** | **11** | **1.2** | **1** |  | **AVSIMGNEVFR + Oxidation (M)** |
|  | 2197 | **642.8462** | **1283.6778** | **1283.6721** | **4.44** | **0** | **11** | **1** | **1** |  | **VSPAGGTLDDKPK** |
|  | 2767 | **718.3811** | **1434.7476** | **1434.7653** | **-12.31** | **1** | **11** | **1.2** | **1** |  | **QDLGIKQFSLMR** |
|  | 2538 | **689.8550** | **1377.6954** | **1377.7139** | **-13.45** | **1** | **11** | **1.3** | **1** |  | **EEAQKLLEQYK** |
|  | 4105 | **1086.5198** | **2171.0250** | **2171.0218** | **1.47** | **0** | **11** | **0.78** | **1** |  | **IFQVAQELSGEDMHQFHR** |
|  | 4126 | **737.7054** | **2210.0943** | **2210.1259** | **-14.31** | **1** | **11** | **0.96** | **1** |  | **QLQPSVVWIEDTEKTFYK** |
|  | 349 | **421.7582** | **841.5019** | **841.5022** | **-0.27** | **0** | **11** | **0.85** | **1** |  | **AVSVTPIR** |
|  | 3327 | **564.6292** | **1690.8658** | **1690.8642** | **0.93** | **0** | **11** | **1.1** | **1** |  | **MLPMGLIIMPGMISR + 2 Oxidation (M)** |
|  | 1645 | **572.2856** | **1142.5566** | **1142.5760** | **-17.02** | **0** | **11** | **1** | **1** |  | **SDPFLVFYR** |
|  | 1903 | **602.3356** | **1202.6567** | **1202.6441** | **10.5** | **1** | **11** | **1.3** | **1** |  | **SLKDINNMIR** |
|  | 3687 | **919.9224** | **1837.8302** | **1837.7935** | **20.0** | **0** | **11** | **0.72** | **1** |  | **NLTNCSSVMLPSNDDR + Oxidation (M)** |
|  | 1062 | **514.7703** | **1027.5260** | **1027.5410** | **-14.67** | **1** | **11** | **1.1** | **1** |  | **GGSSKHNTIK** |
|  | 3402 | **861.4832** | **1720.9519** | **1720.9472** | **2.74** | **1** | **11** | **0.52** | **1** |  | **AIVHQTDNVIDQLKK** |
|  | 1133 | **523.2853** | **1044.5560** | **1044.5716** | **-14.94** | **0** | **11** | **1.8** | **1** |  | **LSSAHVYLR** |
|  | 456 | **435.7736** | **869.5326** | **869.5447** | **-13.84** | **1** | **11** | **0.65** | **1** |  | **AKAKPVTR** |
|  | 69 | 374.7170 | 747.4194 | 747.4200 | -0.85 | 0 | 11 | 1.1 | 1 |  | LDIMLK + Oxidation (M) |
|  | 2623 | **466.5688** | **1396.6846** | **1396.6769** | **5.54** | **1** | **11** | **1.2** | **1** |  | **RDNVAVMATGYGK + Oxidation (M)** |
|  | 2430 | **674.3440** | **1346.6734** | **1346.6751** | **-1.27** | **0** | **11** | **1.5** | **1** |  | **IMELDSNDGLLK** |
|  | 2406 | **671.3417** | **1340.6688** | **1340.6507** | **13.5** | **0** | **11** | **1.3** | **1** |  | **NPPAMSPAGQLSR + Oxidation (M)** |
|  | 536 | **442.7632** | **883.5119** | **883.5127** | **-0.87** | **0** | **11** | **0.7** | **1** |  | **LSLPADIR** |
|  | 926 | 498.2599 | 994.5052 | 994.5057 | -0.47 | 1 | 11 | 1.3 | 1 |  | GGGARGGPPGGR |
|  | 571 | **449.7824** | **897.5503** | **897.5396** | **11.9** | **1** | **11** | **0.45** | **1** |  | **AKITNVPR** |
|  | 771 | **477.3053** | **952.5961** | **952.5957** | **0.44** | **0** | **11** | **0.24** | **1** |  | **LLLPGELAK** |
|  | 2121 | **631.8202** | **1261.6259** | **1261.6165** | **7.49** | **1** | **11** | **1.5** | **1** |  | **FEKLMEYFR** |
|  | 3125 | **790.8870** | **1579.7594** | **1579.7525** | **4.34** | **1** | **11** | **1.2** | **1** |  | **RVDHLTEQCSAHK** |
|  | 1827 | **595.8223** | **1189.6301** | **1189.6203** | **8.21** | **1** | **11** | **1.6** | **1** |  | **TYAPPARASTR** |
|  | 2739 | **476.9016** | **1427.6829** | **1427.6676** | **10.7** | **0** | **11** | **0.98** | **1** |  | **IPAYEMVMESVK + 2 Oxidation (M)** |
|  | 3326 | **846.4373** | **1690.8600** | **1690.8712** | **-6.66** | **0** | **10** | **1.2** | **1** |  | **LYANCEDVGVAILVR** |
|  | 2308 | **438.9292** | **1313.7659** | **1313.7554** | **7.93** | **0** | **10** | **0.48** | **1** |  | **EAGLLAAVTLTQK** |
|  | 3610 | **600.2682** | **1797.7829** | **1797.7628** | **11.2** | **1** | **10** | **0.38** | **1** |  | **YDDTNHIMDKDYPR + Oxidation (M)** |
|  | 1771 | **589.2976** | **1176.5807** | **1176.5775** | **2.72** | **1** | **10** | **1.5** | **1** |  | **EFEKDEKPR** |
|  | 1142 | **523.2855** | **1044.5565** | **1044.5716** | **-14.48** | **0** | **10** | **1.7** | **1** |  | **LSSAHVYLR** |
|  | 3309 | **839.8774** | **1677.7402** | **1677.7457** | **-3.28** | **0** | **10** | **0.52** | **1** |  | **SGPQYMWDPDTLPR + Oxidation (M)** |
|  | 4350 | **817.0446** | **2448.1119** | **2448.0904** | **8.77** | **0** | **10** | **0.53** | **1** |  | **SPTPDELPTCYPGDDWSGVSLR** |
|  | 1144 | **523.2856** | **1044.5566** | **1044.5716** | **-14.36** | **0** | **10** | **1.7** | **1** |  | **LSSAHVYLR** |
|  | 4031 | **1036.5582** | **2071.1019** | **2071.1248** | **-11.06** | **0** | **10** | **0.72** | **1** |  | **MASPVSITWAVRPLTLSSR** |
|  | 2635 | **701.9013** | **1401.7881** | **1401.7868** | **0.93** | **0** | **10** | **0.76** | **1** |  | **VVPIASLTPYQSK** |
|  | 2641 | **702.2966** | **1402.5787** | **1402.5931** | **-10.25** | **0** | **10** | **0.18** | **1** |  | **MMESDYIVMPR + 2 Oxidation (M)** |
|  | 2768 | **718.3817** | **1434.7489** | **1434.7514** | **-1.78** | **0** | **10** | **1.3** | **1** |  | **MAPVGGGGRPVGGPAR** |
|  | 417 | **428.7666** | **855.5186** | **855.5178** | **0.95** | **0** | **10** | **0.94** | **1** |  | **SPLSLIAR** |
|  | 1510 | **557.2804** | **1112.5462** | **1112.5574** | **-10.07** | **0** | **10** | **1.2** | **1** |  | **TTGGTAQPPGAR** |
|  | 1644 | **572.2845** | **1142.5544** | **1142.5760** | **-18.94** | **0** | **10** | **1.1** | **1** |  | **SDPFLVFYR** |
|  | 2102 | **629.7949** | **1257.5753** | **1257.5659** | **7.44** | **0** | **10** | **0.77** | **1** |  | **VSHMGEDLENK** |
|  | 3491 | **587.2933** | **1758.8582** | **1758.8896** | **-17.85** | **0** | **10** | **1.2** | **1** |  | **SMAVEESISMKPLPPK + Oxidation (M)** |
|  | 1768 | **588.3209** | **1174.6272** | **1174.6346** | **-6.34** | **1** | **10** | **1.7** | **1** |  | **DVNVYVNPKK** |
|  | 3249 | **819.9513** | **1637.8880** | **1637.9042** | **-9.86** | **1** | **10** | **0.71** | **1** |  | **LPWWRESSPLVLR** |
|  | 2191 | **642.3403** | **1282.6661** | **1282.6768** | **-8.36** | **0** | **10** | **1.2** | **1** |  | **VNPAAALEELEK** |
|  | 1014 | **510.2726** | **1018.5307** | **1018.5342** | **-3.39** | **1** | **10** | **2.1** | **1** |  | **ATMGLAGRAR + Oxidation (M)** |
|  | 2493 | **681.8383** | **1361.6620** | **1361.6497** | **9.03** | **1** | **10** | **1.4** | **1** |  | **DLGDPSMDKAAVK + Oxidation (M)** |
|  | 3317 | **560.2789** | **1677.8150** | **1677.7859** | **17.3** | **0** | **10** | **1.3** | **1** |  | **TSEPHGNGVWSSPAPR** |
|  | 1440 | **550.8317** | **1099.6488** | **1099.6349** | **12.6** | **1** | **10** | **0.76** | **1** |  | **ITRGIEAVGGK** |
|  | 4054 | **1052.4974** | **2102.9803** | **2102.9397** | **19.3** | **0** | **10** | **0.86** | **1** |  | **EQMCLMVADMLEYVPVK + 3 Oxidation (M)** |
|  | 3048 | **771.9353** | **1541.8560** | **1541.8276** | **18.5** | **0** | **10** | **0.92** | **1** |  | **MPVVWPTLLDLSR + Oxidation (M)** |
|  | 2201 | **643.2813** | **1284.5481** | **1284.5730** | **-19.42** | **0** | **10** | **0.38** | **1** |  | **MSPFPLTSMDK + 2 Oxidation (M)** |
|  | 3372 | **569.6157** | **1705.8253** | **1705.8536** | **-16.58** | **1** | **10** | **1.4** | **1** |  | **ILTGDWFQEARSQR** |
|  | 1959 | **612.8027** | **1223.5909** | **1223.6047** | **-11.29** | **0** | **10** | **1.2** | **1** |  | **EGLGPHGPSFAR** |
|  | 3808 | **949.9328** | **1897.8510** | **1897.8878** | **-19.37** | **1** | **10** | **0.64** | **1** |  | **EHTSQNNEGTPTQKTAR** |
|  | 1826 | **595.8223** | **1189.6300** | **1189.6203** | **8.11** | **1** | **10** | **1.7** | **1** |  | **TYAPPARASTR** |
|  | 1208 | **526.7740** | **1051.5335** | **1051.5339** | **-0.30** | **0** | **10** | **1.4** | **1** |  | **VATPFGGFEK** |
|  | 567 | 449.7719 | 897.5293 | 897.5283 | 1.04 | 0 | 10 | 0.68 | 1 |  | LLAESLPR |
|  | 4181 | **746.3761** | **2236.1065** | **2236.1014** | **2.27** | **1** | **10** | **1.1** | **1** |  | **AIVNPMDMQTSGALLRTCVK + 2 Oxidation (M)** |
|  | 473 | **435.7740** | **869.5334** | **869.5195** | **15.9** | **1** | **10** | **0.7** | **1** |  | **AVRQALGR** |
|  | 1913 | **603.8098** | **1205.6051** | **1205.5942** | **9.05** | **1** | **10** | **1.8** | **1** |  | **QAVPNGGRFNF** |
|  | 246 | **413.2882** | **824.5618** | **824.5484** | **16.3** | **0** | **10** | **0.099** | **1** |  | **VKPQLLK** |
|  | 1563 | **563.7958** | **1125.5771** | **1125.5931** | **-14.18** | **1** | **10** | **1.4** | **1** |  | **GNKSFGFTLR** |
|  | 391 | **423.7186** | **845.4227** | **845.4130** | **11.4** | **0** | **10** | **2** | **1** |  | **AIESSPDK** |
|  | 2816 | **725.8799** | **1449.7453** | **1449.7537** | **-5.78** | **1** | **10** | **1.6** | **1** |  | **EMTQAPYLEIKK** |
|  | 1127 | **523.2850** | **1044.5555** | **1044.5386** | **16.2** | **0** | **10** | **2.1** | **1** |  | **LGVNDCVLR** |
|  | 2562 | **693.3944** | **1384.7743** | **1384.7966** | **-16.12** | **1** | **10** | **0.93** | **1** |  | **EPSVLFEVPKLK** |
|  | 2740 | **476.9016** | **1427.6830** | **1427.6892** | **-4.36** | **0** | **10** | **1.1** | **1** |  | **ETDGDHLSSLLNK** |
|  | 4146 | **737.7065** | **2210.0976** | **2210.1188** | **-9.58** | **0** | **10** | **1.1** | **1** |  | **VGVTVAQTTMEPHLLEACVR** |
|  | 1069 | **515.7802** | **1029.5459** | **1029.5641** | **-17.70** | **0** | **10** | **1.8** | **1** |  | **IGLINDMVR** |
|  | 1476 | **554.2883** | **1106.5621** | **1106.5754** | **-12.02** | **1** | **10** | **1.9** | **1** |  | **DTKVVSMVGR + Oxidation (M)** |
|  | 2576 | **693.3952** | **1384.7758** | **1384.7966** | **-14.97** | **1** | **10** | **0.94** | **1** |  | **EPSVLFEVPKLK** |
|  | 1233 | **529.3034** | **1056.5923** | **1056.5750** | **16.4** | **0** | **10** | **1.4** | **1** |  | **VGLNAPSMLR** |
|  | 2054 | **624.8085** | **1247.6025** | **1247.6067** | **-3.39** | **0** | **10** | **1.4** | **1** |  | **VLGESGEMDALK** |
|  | 983 | **506.2533** | **1010.4920** | **1010.5080** | **-15.77** | **0** | **10** | **1.2** | **1** |  | **HVLQCQAR** |
|  | 1456 | **552.2852** | **1102.5559** | **1102.5618** | **-5.37** | **1** | **10** | **2.1** | **1** |  | **KELENLDSR** |
|  | 2933 | **750.3411** | **1498.6677** | **1498.6973** | **-19.77** | **0** | **10** | **0.76** | **1** |  | **MEELETSLFQTR + Oxidation (M)** |
|  | 185 | **402.2470** | **802.4794** | **802.4701** | **11.6** | **0** | **10** | **0.87** | **1** |  | **VVPLYGR** |
|  | 1096 | **519.7410** | **1037.4675** | **1037.4852** | **-17.02** | **0** | **10** | **0.75** | **1** |  | **DFSNPMALK + Oxidation (M)** |
|  | 1123 | **523.2842** | **1044.5539** | **1044.5716** | **-16.93** | **0** | **10** | **2** | **1** |  | **LSSAHVYLR** |
|  | 191 | **404.2322** | **806.4499** | **806.4572** | **-8.98** | **0** | **10** | **1.1** | **1** |  | **LMTSLVK + Oxidation (M)** |
|  | 4050 | **1049.5122** | **2097.0099** | **2097.0491** | **-18.69** | **0** | **10** | **1.1** | **1** |  | **ATYIQTIEEGINTHTHAAK** |
|  | 868 | **490.7338** | **979.4530** | **979.4644** | **-11.62** | **0** | **10** | **1.2** | **1** |  | **TLLSEDMR + Oxidation (M)** |
|  | 2574 | **693.3950** | **1384.7754** | **1384.7966** | **-15.32** | **1** | **10** | **0.97** | **1** |  | **EPSVLFEVPKLK** |
|  | 303 | **421.7580** | **841.5015** | **841.5021** | **-0.73** | **1** | **10** | **1.1** | **1** |  | **ELKSIPR** |
|  | 2386 | **669.3505** | **1336.6865** | **1336.6987** | **-9.11** | **0** | **10** | **1.5** | **1** |  | **ATAAFSNVGTAISK** |
|  | 3600 | 898.4061 | 1794.7976 | 1794.8306 | -18.37 | 0 | 10 | 0.75 | 1 |  | SLDEIAMDLTETGTQR + Oxidation (M) |
|  | 4243 | **762.0591** | **2283.1554** | **2283.1894** | **-14.90** | **0** | **10** | **1.1** | **1** |  | **EEVIDFSKPFMSLGISIMIK** |
|  | 587 | **451.7307** | **901.4467** | **901.4617** | **-16.62** | **0** | **10** | **1.8** | **1** |  | **GLTGQAEAR** |
|  | 157 | **395.7494** | **789.4842** | **789.4861** | **-2.45** | **1** | **10** | **0.56** | **1** |  | **IFKLGGR** |
|  | 2785 | 720.4099 | 1438.8053 | 1438.7892 | 11.1 | 1 | 10 | 0.79 | 1 |  | GVDLATGPSRTPLR |
|  | 3353 | **849.9542** | **1697.8939** | **1697.8696** | **14.3** | **1** | **10** | **1.2** | **1** |  | **AQAIQRQLEEVEER** |
|  | 2180 | **641.2982** | **1280.5818** | **1280.5745** | **5.66** | **0** | **10** | **0.94** | **1** |  | **QSNNSHSPLPSN** |
|  | 3482 | **585.6037** | **1753.7893** | **1753.8101** | **-11.85** | **1** | **10** | **0.92** | **1** |  | **HCQAGAPADVACSVRR** |
|  | 2132 | **633.8249** | **1265.6353** | **1265.6227** | **10.0** | **0** | **10** | **1.6** | **1** |  | **YPAYLSIHMR + Oxidation (M)** |
|  | 348 | **421.7582** | **841.5019** | **841.5021** | **-0.25** | **1** | **10** | **1.1** | **1** |  | **VAGAATPKK** |
|  | 1697 | **580.7542** | **1159.4939** | **1159.4815** | **10.6** | **0** | **10** | **0.33** | **1** |  | **SYSPTEMSSR + Oxidation (M)** |
|  | 2998 | **763.8799** | **1525.7452** | **1525.7420** | **2.13** | **1** | **10** | **1.3** | **1** |  | **QPGALTQGHSCSRK** |
|  | 158 | **396.2185** | **790.4225** | **790.4371** | **-18.42** | **1** | **10** | **2.3** | **1** |  | **MKVLER + Oxidation (M)** |
|  | 2072 | **627.3537** | **1252.6928** | **1252.7027** | **-7.86** | **1** | **10** | **0.86** | **1** |  | **GLVPTKLDPEGK** |
|  | 2450 | **678.8466** | **1355.6787** | **1355.6946** | **-11.73** | **0** | **10** | **1.3** | **1** |  | **APHPSHTSQPVAK** |
|  | 2569 | **693.3948** | **1384.7750** | **1384.7966** | **-15.58** | **1** | **10** | **1** | **1** |  | **EPSVLFEVPKLK** |
|  | 3019 | **768.8324** | **1535.6502** | **1535.6708** | **-13.40** | **0** | **10** | **0.35** | **1** |  | **ENGMPMDIVNTSGR + Oxidation (M)** |
|  | 438 | **433.7376** | **865.4605** | **865.4545** | **6.95** | **0** | **10** | **1.7** | **1** |  | **AVFDLSSK** |
|  | 643 | **458.7878** | **915.5609** | **915.5575** | **3.72** | **0** | **10** | **0.38** | **1** |  | **VLLTMIAR** |
|  | 2956 | **757.3704** | **1512.7262** | **1512.7130** | **8.73** | **1** | **10** | **1.3** | **1** |  | **SDMDYLSNALEKK** |
|  | 2710 | **712.3556** | **1422.6967** | **1422.6991** | **-1.63** | **1** | **10** | **1.5** | **1** |  | **DGVELTREETFK** |
|  | 3508 | **589.2388** | **1764.6945** | **1764.7270** | **-18.41** | **0** | **10** | **0.11** | **1** |  | **YQAMLSVDDCFGMGR + Oxidation (M)** |
|  | 199 | **406.7183** | **811.4220** | **811.4374** | **-19.07** | **0** | **10** | **1** | **1** |  | **LPGCLPR** |
|  | 221 | **411.2191** | **820.4236** | **820.4113** | **15.0** | **0** | **10** | **1.8** | **1** |  | **AAGAGMISK + Oxidation (M)** |
|  | 3650 | **908.9006** | **1815.7866** | **1815.7821** | **2.45** | **0** | **10** | **0.43** | **1** |  | **VGTYAVCWCSWNTGR** |
|  | 97 | **380.2025** | **758.3905** | **758.3922** | **-2.23** | **0** | **10** | **2.1** | **1** |  | **APSEISR** |
|  | 463 | **435.7738** | **869.5331** | **869.5446** | **-13.27** | **1** | **10** | **0.77** | **1** |  | **RLALLER** |
|  | 1887 | 401.2347 | 1200.6822 | 1200.6714 | 9.04 | 1 | 10 | 1.1 | 1 |  | IIENQEKSLK |
|  | 3314 | **420.4608** | **1677.8143** | **1677.8362** | **-13.06** | **1** | **10** | **1.5** | **1** |  | **YLEYKQVPSSDPPR** |
|  | 2476 | **679.8536** | **1357.6926** | **1357.6950** | **-1.74** | **1** | **10** | **1.7** | **1** |  | **EVEQSRQEVVR** |
|  | 632 | **457.7683** | **913.5220** | **913.5345** | **-13.67** | **0** | **10** | **1.5** | **1** |  | **VAAISVAQR** |
|  | 3036 | **514.6284** | **1540.8633** | **1540.8573** | **3.86** | **0** | **10** | **0.73** | **1** |  | **NQLTSVPSLISGLGR** |
|  | 1397 | **546.2588** | **1090.5030** | **1090.5189** | **-14.56** | **1** | **10** | **1.2** | **1** |  | **EALNSCNKR** |
|  | 2297 | **655.3227** | **1308.6308** | **1308.6310** | **-0.12** | **1** | **10** | **1.4** | **1** |  | **TAEDLSFRAGDK** |
|  | 4459 | **710.5814** | **2838.2963** | **2838.3251** | **-10.15** | **1** | **10** | **0.7** | **1** |  | **MPWTSPSCQNSCELGIQNLSFKVR** |
|  | 633 | **457.7686** | **913.5227** | **913.5167** | **6.53** | **1** | **10** | **1.6** | **1** |  | **MPEIRLR** |
|  | 3578 | **897.9091** | **1793.8037** | **1793.8028** | **0.51** | **0** | **10** | **0.92** | **1** |  | **TGTTGQSGAESGTTEPSAR** |
|  | 539 | **442.7637** | **883.5128** | **883.5127** | **0.09** | **0** | **10** | **0.84** | **1** |  | **ATVVEIPR** |
|  | 583 | **450.7609** | **899.5072** | **899.5076** | **-0.51** | **0** | **10** | **1.7** | **1** |  | **DISLVPTR** |
|  | 1643 | **571.8604** | **1141.7061** | **1141.6893** | **14.8** | **1** | **10** | **0.19** | **1** |  | **SPKCLALLLK** |
|  | 1171 | **523.2866** | **1044.5587** | **1044.5716** | **-12.37** | **0** | **10** | **2.2** | **1** |  | **LSSAHVYLR** |
|  | 372 | 421.7588 | 841.5031 | 841.5134 | -12.21 | 1 | 10 | 1.1 | 1 |  | RDILLGR |
|  | 468 | **435.7739** | **869.5333** | **869.5334** | **-0.14** | **1** | **9** | **0.8** | **1** |  | **QALSPKVK** |
|  | 2123 | **421.8947** | **1262.6623** | **1262.6693** | **-5.54** | **1** | **9** | **1.7** | **1** |  | **GTVDGILKMWK + Oxidation (M)** |
|  | 2529 | **687.8683** | **1373.7221** | **1373.7263** | **-3.00** | **1** | **9** | **1.7** | **1** |  | **QLSGAGSETLKQR** |
|  | 3145 | **794.3789** | **1586.7431** | **1586.7246** | **11.7** | **1** | **9** | **1.3** | **1** |  | **KMSGGQSGYELSEAK + Oxidation (M)** |
|  | 295 | 421.7580 | 841.5014 | 841.5021 | -0.87 | 1 | 9 | 1.2 | 1 |  | ELKSIPR |
|  | 2654 | **469.5861** | **1405.7364** | **1405.7201** | **11.6** | **1** | **9** | **1.7** | **1** |  | **DLFIDGQSKDIR** |
|  | 1670 | **576.3244** | **1150.6342** | **1150.6458** | **-10.08** | **0** | **9** | **1.2** | **1** |  | **APQLPGVSNIR** |
|  | 1304 | **536.2932** | **1070.5719** | **1070.5682** | **3.46** | **0** | **9** | **1.7** | **1** |  | **LSLMYSITK + Oxidation (M)** |
|  | 1285 | **534.2737** | **1066.5329** | **1066.5263** | **6.19** | **1** | **9** | **1.9** | **1** |  | **MVQKTSMSR** |
|  | 1001 | **508.2783** | **1014.5421** | **1014.5247** | **17.2** | **1** | **9** | **2.2** | **1** |  | **AVTRSYYR** |
|  | 4027 | **1036.0103** | **2070.0059** | **2070.0060** | **-0.05** | **1** | **9** | **1.6** | **1** |  | **AYCVLVTGPNMGGKSTLMR + Oxidation (M)** |
|  | 444 | **434.7662** | **867.5179** | **867.5079** | **11.6** | **0** | **9** | **0.54** | **1** |  | **VPRPFPR** |
|  | 578 | **450.2705** | **898.5265** | **898.5097** | **18.7** | **1** | **9** | **1.2** | **1** |  | **AQGAQRLR** |
|  | 582 | **450.7608** | **899.5071** | **899.5076** | **-0.58** | **0** | **9** | **1.8** | **1** |  | **IDVTLSPR** |
|  | 1516 | 557.8273 | 1113.6400 | 1113.6506 | -9.52 | 1 | 9 | 1.3 | 1 |  | VSDATKLRPK |
|  | 1824 | **595.8120** | **1189.6095** | **1189.5914** | **15.2** | **1** | **9** | **2.1** | **1** |  | **WVGEGEKMVR** |
|  | 3202 | **811.3977** | **1620.7809** | **1620.8042** | **-14.40** | **0** | **9** | **1.4** | **1** |  | **LCQELIIHTSDHR** |
|  | 1264 | **532.2670** | **1062.5194** | **1062.5305** | **-10.49** | **1** | **9** | **2.3** | **1** |  | **VETNKNSSGK** |
|  | 3295 | **556.3019** | **1665.8838** | **1665.9103** | **-15.91** | **1** | **9** | **1.1** | **1** |  | **EFIRFHQIPNPLR** |
|  | 1310 | **536.7900** | **1071.5655** | **1071.5673** | **-1.62** | **0** | **9** | **4.2** | **1** |  | **LGAAAADAVTGR** |
|  | 2488 | **681.3450** | **1360.6754** | **1360.6769** | **-1.11** | **0** | **9** | **2.1** | **1** |  | **VVATTQMQAADAR** |
|  | 2966 | 505.9217 | 1514.7433 | 1514.7576 | -9.44 | 0 | 9 | 1.5 | 1 |  | EQGVDENETLLLR |
|  | 2994 | **763.8769** | **1525.7393** | **1525.7420** | **-1.71** | **1** | **9** | **1.3** | **1** |  | **QPGALTQGHSCSRK** |
|  | 2323 | 439.8773 | 1316.6100 | 1316.5894 | 15.7 | 0 | 9 | 1.3 | 1 |  | CPVCSFVYGTK |
|  | 482 | **435.7740** | **869.5335** | **869.5334** | **0.12** | **1** | **9** | **0.84** | **1** |  | **TVVPKAQK** |
|  | 318 | **421.7582** | **841.5017** | **841.5021** | **-0.46** | **1** | **9** | **1.2** | **1** |  | **VAGAATPKK** |
|  | 1452 | **551.7959** | **1101.5772** | **1101.5891** | **-10.71** | **1** | **9** | **2.4** | **1** |  | **AVRVSTEANR** |
|  | 2573 | **462.5991** | **1384.7754** | **1384.7714** | **2.84** | **1** | **9** | **1.1** | **1** |  | **WGDIKINIIGEK** |
|  | 3591 | **897.9109** | **1793.8073** | **1793.7787** | **16.0** | **0** | **9** | **0.92** | **1** |  | **FMVDIACGMEYLSSR + Oxidation (M)** |
|  | 2046 | **622.3378** | **1242.6610** | **1242.6754** | **-11.62** | **1** | **9** | **1.8** | **1** |  | **KVSMGKPDPLR + Oxidation (M)** |
|  | 39 | **363.2493** | **724.4840** | **724.4959** | **-16.48** | **1** | **9** | **0.31** | **1** |  | **KIPIVR** |
|  | 2392 | **446.9453** | **1337.8140** | **1337.8031** | **8.18** | **1** | **9** | **0.2** | **1** |  | **DRLSLVLVPAQK** |
|  | 3573 | **896.9030** | **1791.7915** | **1791.8097** | **-10.19** | **0** | **9** | **0.86** | **1** |  | **HEMTEGVTAYEEKPR + Oxidation (M)** |
|  | 1712 | **582.7985** | **1163.5824** | **1163.5856** | **-2.79** | **0** | **9** | **2.3** | **1** |  | **VATMTQQLEK + Oxidation (M)** |
|  | 2647 | **702.8328** | **1403.6511** | **1403.6568** | **-4.10** | **0** | **9** | **1.3** | **1** |  | **SPSPEPIYNSEGK** |
|  | 711 | **467.7536** | **933.4926** | **933.4814** | **12.0** | **1** | **9** | **2.4** | **1** |  | **TRIQNMR + Oxidation (M)** |
|  | 3027 | **769.3913** | **1536.7680** | **1536.7647** | **2.20** | **0** | **9** | **1.8** | **1** |  | **VGGGWMALDEFLVK + Oxidation (M)** |
|  | 1609 | **568.2946** | **1134.5747** | **1134.5894** | **-12.94** | **1** | **9** | **2.2** | **1** |  | **RIHAGENPNK** |
|  | 646 | **458.7879** | **915.5612** | **915.5575** | **4.05** | **0** | **9** | **0.44** | **1** |  | **VLLTMIAR** |
|  | 415 | **428.7662** | **855.5179** | **855.5039** | **16.4** | **1** | **9** | **1.2** | **1** |  | **LAGAVRNR** |
|  | 4226 | **757.0424** | **2268.1053** | **2268.1341** | **-12.72** | **1** | **9** | **1.3** | **1** |  | **GGYHDGHLVIRWFWAAVER** |
|  | 990 | **506.8055** | **1011.5964** | **1011.5937** | **2.64** | **1** | **9** | **0.88** | **1** |  | **AVAGAIARQR** |
|  | 1515 | **557.8270** | **1113.6394** | **1113.6506** | **-10.06** | **1** | **9** | **1.4** | **1** |  | **VSDATKLRPK** |
|  | 3629 | **905.4163** | **1808.8180** | **1808.8332** | **-8.40** | **1** | **9** | **1.1** | **1** |  | **MDAMLLRQCPTQGTR + 2 Oxidation (M)** |
|  | 2371 | **666.3877** | **1330.7608** | **1330.7642** | **-2.55** | **1** | **9** | **0.88** | **1** |  | **VAKSVEMLILGR + Oxidation (M)** |
|  | 1837 | **597.2372** | **1192.4598** | **1192.4778** | **-15.12** | **1** | **9** | **0.12** | **1** |  | **GEAERDCDNK** |
|  | 3927 | **495.9907** | **1979.9335** | **1979.9371** | **-1.79** | **1** | **9** | **1.3** | **1** |  | **FTSAVKLSEGGPGSGMENGR** |
|  | 764 | **475.7176** | **949.4206** | **949.4175** | **3.25** | **0** | **9** | **1.1** | **1** |  | **ATQADMGEK** |
|  | 1160 | **523.2859** | **1044.5572** | **1044.5716** | **-13.77** | **0** | **9** | **2.4** | **1** |  | **LSSAHVYLR** |
|  | 3238 | **546.2517** | **1635.7333** | **1635.7596** | **-16.10** | **1** | **9** | **0.91** | **1** |  | **CGDGGKAVEATLMSPK + Oxidation (M)** |
|  | 312 | **421.7581** | **841.5017** | **841.5021** | **-0.53** | **1** | **9** | **1.3** | **1** |  | **VAGAATPKK** |
|  | 359 | **421.7583** | **841.5021** | **841.5021** | **-0.03** | **1** | **9** | **1.3** | **1** |  | **VAGAATPKK** |
|  | 2105 | **629.8251** | **1257.6357** | **1257.6248** | **8.67** | **1** | **9** | **1.8** | **1** |  | **PDRACAVGSVAR** |
|  | 1157 | **523.2857** | **1044.5569** | **1044.5716** | **-14.11** | **0** | **9** | **2.4** | **1** |  | **LSSAHVYLR** |
|  | 458 | **435.7737** | **869.5329** | **869.5334** | **-0.60** | **0** | **9** | **0.95** | **1** |  | **VLTAPTLR** |
|  | 4481 | **1011.4301** | **3031.2685** | **3031.3071** | **-12.75** | **0** | **9** | **0.21** | **1** |  | **DNFQETMEAMHIMGFSHEEILSMLK + 4 Oxidation (M)** |
|  | 559 | **448.2351** | **894.4556** | **894.4382** | **19.5** | **0** | **9** | **1.7** | **1** |  | **ATFCALGR** |
|  | 2224 | **646.3542** | **1290.6938** | **1290.7044** | **-8.20** | **1** | **9** | **1.7** | **1** |  | **AFSRQLAIETR** |
|  | 130 | **387.2442** | **772.4738** | **772.4807** | **-8.89** | **1** | **9** | **1.1** | **1** |  | **LSLKNAK** |
|  | 390 | **423.7177** | **845.4208** | **845.4243** | **-4.11** | **0** | **9** | **2.3** | **1** |  | **SPAIDSTR** |
|  | 2079 | **627.8208** | **1253.6270** | **1253.6438** | **-13.36** | **0** | **9** | **1.9** | **1** |  | **VATPLPDPMASR** |
|  | 2931 | **749.3874** | **1496.7602** | **1496.7367** | **15.7** | **0** | **9** | **1.8** | **1** |  | **AGYLMGLNSAEMLK** |
|  | 3040 | **514.6304** | **1540.8693** | **1540.8573** | **7.78** | **1** | **9** | **0.81** | **1** |  | **LQETGKQNVAVQVK** |
|  | 136 | **390.7326** | **779.4506** | **779.4653** | **-18.93** | **0** | **9** | **0.67** | **1** |  | **ILAAQHK** |
|  | 1521 | **559.2772** | **1116.5398** | **1116.5312** | **7.67** | **0** | **9** | **1.8** | **1** |  | **NGNPVAENFR** |
|  | 1223 | **528.3140** | **1054.6135** | **1054.6069** | **6.22** | **0** | **9** | **0.74** | **1** |  | **LIQLRPCR** |
|  | 1904 | **602.7977** | **1203.5808** | **1203.5805** | **0.24** | **0** | **9** | **1.9** | **1** |  | **MAEAELVQEGK** |
|  | 370 | **421.7586** | **841.5026** | **841.5021** | **0.56** | **1** | **9** | **1.3** | **1** |  | **VAGAATPKK** |
|  | 951 | **501.2763** | **1000.5381** | **1000.5553** | **-17.15** | **0** | **9** | **2.9** | **1** |  | **AGLLTEEIR** |
|  | 3589 | **897.9107** | **1793.8069** | **1793.8400** | **-18.46** | **1** | **9** | **1.1** | **1** |  | **VTAQQMAMERAELER + 2 Oxidation (M)** |
|  | 3785 | **628.3616** | **1882.0629** | **1882.0636** | **-0.38** | **1** | **9** | **0.49** | **1** |  | **ALTSSRGVLQQLAPSVQK** |
|  | 1427 | **548.2933** | **1094.5721** | **1094.5832** | **-10.14** | **1** | **9** | **1.7** | **1** |  | **KAHEAALEAR** |
|  | 4368 | **828.4108** | **2482.2107** | **2482.2129** | **-0.89** | **1** | **9** | **1.4** | **1** |  | **DPGSPGPFDYVGAPPAESIPQKTR** |
|  | 2190 | **642.2570** | **1282.4995** | **1282.5023** | **-2.22** | **0** | **9** | **0.21** | **1** |  | **DMDDEESWIK + Oxidation (M)** |
|  | 1156 | **523.2857** | **1044.5569** | **1044.5716** | **-14.11** | **0** | **9** | **2.5** | **1** |  | **LSSAHVYLR** |
|  | 1713 | **582.7986** | **1163.5827** | **1163.5903** | **-6.50** | **1** | **9** | **2.4** | **1** |  | **MRTVNAMAVR + Oxidation (M)** |
|  | 2570 | **462.5990** | **1384.7752** | **1384.7973** | **-15.95** | **1** | **9** | **1.2** | **1** |  | **MRLLGAAAVAALGR + Oxidation (M)** |
|  | 3938 | **1002.9981** | **2003.9817** | **2003.9623** | **9.67** | **0** | **9** | **1.6** | **1** |  | **TVDSQGPTPVCTPTFLER** |
|  | 422 | **429.7376** | **857.4605** | **857.4606** | **-0.12** | **0** | **9** | **2.6** | **1** |  | **LGAEEALR** |
|  | 2567 | **462.5989** | **1384.7749** | **1384.7973** | **-16.14** | **1** | **9** | **1.2** | **1** |  | **MRLLGAAAVAALGR + Oxidation (M)** |
|  | 337 | **421.7582** | **841.5019** | **841.4882** | **16.2** | **1** | **9** | **1.3** | **1** |  | **AGRTRPGK** |
|  | 1138 | **523.2854** | **1044.5562** | **1044.5716** | **-14.71** | **0** | **9** | **2.5** | **1** |  | **LSSAHVYLR** |
|  | 4000 | **679.6779** | **2036.0119** | **2035.9997** | **6.02** | **1** | **9** | **1.7** | **1** |  | **SVEDLVRMDGINAAFLDR + Oxidation (M)** |
|  | 19 | **360.1848** | **718.3550** | **718.3609** | **-8.27** | **1** | **9** | **3.5** | **1** |  | **KTAEDR** |
|  | 1747 | **585.7905** | **1169.5664** | **1169.5685** | **-1.82** | **1** | **9** | **1.4** | **1** |  | **AMYKAGMVQR + Oxidation (M)** |
|  | 658 | **460.7463** | **919.4780** | **919.4797** | **-1.83** | **0** | **9** | **2.2** | **1** |  | **AVSSQMIGK** |
|  | 2727 | **475.9211** | **1424.7416** | **1424.7372** | **3.08** | **1** | **9** | **1.8** | **1** |  | **FSGGSLLTTGSSRR** |
|  | 3175 | **803.4451** | **1604.8757** | **1604.8774** | **-1.03** | **0** | **9** | **1.1** | **1** |  | **QLLQQLVTSYPSTK** |
|  | 3598 | **898.4033** | **1794.7921** | **1794.8206** | **-15.91** | **0** | **9** | **0.87** | **1** |  | **AQEMLQNQFIGEDTR + Oxidation (M)** |
|  | 3630 | **905.4166** | **1808.8186** | **1808.8332** | **-8.07** | **1** | **9** | **1.1** | **1** |  | **MDAMLLRQCPTQGTR + 2 Oxidation (M)** |
|  | 1334 | **540.2777** | **1078.5409** | **1078.5553** | **-13.39** | **1** | **9** | **2.3** | **1** |  | **TLTRQSMSR** |
|  | 2115 | **630.8576** | **1259.7006** | **1259.7020** | **-1.06** | **1** | **9** | **1.6** | **1** |  | **MQLTVKALQGR + Oxidation (M)** |
|  | 3004 | **511.2325** | **1530.6756** | **1530.6919** | **-10.65** | **0** | **9** | **0.66** | **1** |  | **QSPPGTPVSSCQMR** |
|  | 3207 | **812.3847** | **1622.7548** | **1622.7862** | **-19.32** | **0** | **9** | **1.4** | **1** |  | **FVGTDPASDEVVLMK + Oxidation (M)** |
|  | 2131 | **633.8149** | **1265.6153** | **1265.5995** | **12.5** | **0** | **9** | **1.9** | **1** |  | **NTMALMDLDVK + Oxidation (M)** |
|  | 2572 | **462.5991** | **1384.7754** | **1384.7973** | **-15.82** | **1** | **9** | **1.3** | **1** |  | **MRLLGAAAVAALGR + Oxidation (M)** |
|  | 3531 | **887.9486** | **1773.8827** | **1773.8760** | **3.78** | **1** | **9** | **1.8** | **1** |  | **QWMEYFKASINTLK + Oxidation (M)** |
|  | 3599 | **599.2722** | **1794.7947** | **1794.8206** | **-14.48** | **0** | **9** | **0.92** | **1** |  | **AQEMLQNQFIGEDTR + Oxidation (M)** |
|  | 1136 | **523.2854** | **1044.5562** | **1044.5386** | **16.9** | **0** | **9** | **2.6** | **1** |  | **LGVNDCVLR** |
|  | 282 | **421.2423** | **840.4700** | **840.4817** | **-13.95** | **0** | **9** | **1.4** | **1** |  | **AVQALPSR** |
|  | 3419 | **576.5894** | **1726.7463** | **1726.7217** | **14.2** | **0** | **9** | **0.51** | **1** |  | **ALEEQCDEDHQTPR** |
|  | 307 | **421.7581** | **841.5016** | **841.5021** | **-0.67** | **1** | **9** | **1.4** | **1** |  | **VAGAATPKK** |
|  | 3399 | **860.4329** | **1718.8513** | **1718.8522** | **-0.56** | **0** | **9** | **1.6** | **1** |  | **SPPSAGPLVVHCSAGAGR** |
|  | 621 | **456.7790** | **911.5434** | **911.5301** | **14.6** | **1** | **9** | **0.66** | **1** |  | **GRQVTVPR** |
|  | 1908 | **602.8310** | **1203.6474** | **1203.6611** | **-11.40** | **1** | **9** | **2.1** | **1** |  | **KQGLEQFINK** |
|  | 3066 | **517.5902** | **1549.7488** | **1549.7294** | **12.5** | **0** | **9** | **1.9** | **1** |  | **TSPSQEDLLMQSSK** |
|  | 1635 | **571.2617** | **1140.5089** | **1140.5159** | **-6.19** | **0** | **9** | **1.2** | **1** |  | **EAAEGLGSHDR** |
|  | 4408 | 1314.5547 | 2627.0948 | 2627.1416 | -17.80 | 1 | 9 | 0.2 | 1 |  | DSNMPMDMREFVLLFMTFDEK + 2 Oxidation (M) |
|  | 3371 | **853.8986** | **1705.7826** | **1705.7651** | **10.2** | **1** | **9** | **1.2** | **1** |  | **TTDATCTSLSKFCSK** |
|  | 636 | **458.7299** | **915.4453** | **915.4484** | **-3.34** | **0** | **9** | **1.6** | **1** |  | **IVMGDTHK + Oxidation (M)** |
|  | 2403 | **670.8496** | **1339.6845** | **1339.7109** | **-19.66** | **1** | **9** | **1.8** | **1** |  | **AAGAGAGSAPRWLR** |
|  | 2480 | **453.5727** | **1357.6962** | **1357.7201** | **-17.66** | **0** | **9** | **2.4** | **1** |  | **ELSDALGISIAGGR** |
|  | 4011 | **1026.4550** | **2050.8955** | **2050.9154** | **-9.69** | **0** | **9** | **0.51** | **1** |  | **TDDITSGYMTDGGLGLYTR + Oxidation (M)** |
|  | 1078 | **516.7319** | **1031.4493** | **1031.4519** | **-2.55** | **0** | **9** | **0.53** | **1** |  | **TSEPENNNK** |
|  | 433 | **432.7146** | **863.4146** | **863.4058** | **10.2** | **0** | **9** | **2.2** | **1** |  | **ALEMQEK + Oxidation (M)** |
|  | 1064 | **514.7713** | **1027.5280** | **1027.5410** | **-12.65** | **1** | **9** | **1.9** | **1** |  | **GGSSKHNTIK** |
|  | 1439 | **550.8311** | **1099.6475** | **1099.6601** | **-11.41** | **1** | **9** | **1.1** | **1** |  | **LGSATPKIVSK** |
|  | 2407 | **671.3445** | **1340.6745** | **1340.6502** | **18.2** | **1** | **9** | **2.1** | **1** |  | **MKQMTLLDMAK + 2 Oxidation (M)** |
|  | 2477 | **679.8543** | **1357.6941** | **1357.6911** | **2.17** | **0** | **9** | **2.4** | **1** |  | **EMEELIPLVNR + Oxidation (M)** |
|  | 3414 | **863.9493** | **1725.8841** | **1725.8621** | **12.8** | **1** | **9** | **1.6** | **1** |  | **TGQMYPVPANHLDKR** |
|  | 3028 | **769.8796** | **1537.7447** | **1537.7269** | **11.6** | **0** | **9** | **1.7** | **1** |  | **VTGTQPITCTWMK + Oxidation (M)** |
|  | 3592 | **897.9112** | **1793.8078** | **1793.7787** | **16.3** | **0** | **9** | **1.1** | **1** |  | **FMVDIACGMEYLSSR + Oxidation (M)** |
|  | 68 | **373.2139** | **744.4132** | **744.4242** | **-14.75** | **1** | **9** | **4.9** | **1** |  | **AVGGARSK** |
|  | 439 | **433.7377** | **865.4608** | **865.4545** | **7.23** | **0** | **9** | **2.2** | **1** |  | **AVFDLSSK** |
|  | 618 | **456.2689** | **910.5233** | **910.5348** | **-12.67** | **1** | **9** | **0.96** | **1** |  | **RGAEILPR** |
|  | 2963 | 505.9216 | 1514.7430 | 1514.7592 | -10.69 | 0 | 9 | 1.8 | 1 |  | YCLAGLPVGYQFK |
|  | 3237 | **545.9665** | **1634.8778** | **1634.8549** | **14.0** | **0** | **9** | **1.4** | **1** |  | **LLASTASSSPASVMVAK + Oxidation (M)** |
|  | 3690 | **921.9034** | **1841.7923** | **1841.7957** | **-1.84** | **1** | **9** | **0.61** | **1** |  | **ERELALMEQEMMER + 3 Oxidation (M)** |
|  | 1161 | **523.2859** | **1044.5573** | **1044.5386** | **17.9** | **0** | **9** | **2.7** | **1** |  | **LGVNDCVLR** |
|  | 320 | **421.7582** | **841.5017** | **841.5021** | **-0.46** | **1** | **9** | **1.4** | **1** |  | **VAGAATPKK** |
|  | 3101 | **783.8735** | **1565.7325** | **1565.7606** | **-17.95** | **1** | **9** | **1.4** | **1** |  | **MSEKSVEAAAELSAK + Oxidation (M)** |
|  | 3897 | 488.4952 | 1949.9515 | 1949.9490 | 1.29 | 1 | 9 | 1.7 | 1 |  | HRSTLTQASSMTPHSGPR |
|  | 1626 | 570.2830 | 1138.5515 | 1138.5593 | -6.88 | 1 | 8 | 1.9 | 1 |  | QYKHYCIK |
|  | 1177 | **523.7763** | **1045.5381** | **1045.5192** | **18.0** | **0** | **8** | **3** | **1** |  | **IWQEEVSR** |
|  | 304 | **421.7580** | **841.5015** | **841.5021** | **-0.75** | **1** | **8** | **1.4** | **1** |  | **VAGAATPKK** |
|  | 780 | **478.2762** | **954.5378** | **954.5359** | **2.01** | **1** | **8** | **1.3** | **1** |  | **AVQRAASPR** |
|  | 1139 | **523.2855** | **1044.5564** | **1044.5716** | **-14.59** | **0** | **8** | **2.7** | **1** |  | **LSSAHVYLR** |
|  | 1175 | **523.2931** | **1044.5717** | **1044.5829** | **-10.65** | **1** | **8** | **2.2** | **1** |  | **GVGSRGFPLR** |
|  | 2310 | **657.8908** | **1313.7669** | **1313.7415** | **19.4** | **1** | **8** | **0.78** | **1** |  | **NLTRLDLSLNR** |
|  | 3032 | **770.8882** | **1539.7619** | **1539.7656** | **-2.41** | **0** | **8** | **2.3** | **1** |  | **HNTVLMAWPASWK** |
|  | 3396 | **859.9443** | **1717.8740** | **1717.8595** | **8.44** | **0** | **8** | **2.1** | **1** |  | **NGSQTVSVSVSQALDAR** |
|  | 4236 | **761.6909** | **2282.0508** | **2282.0130** | **16.5** | **0** | **8** | **1.2** | **1** |  | **DGINMPTVCTSGCLSFPSAPR + Oxidation (M)** |
|  | 4235 | 761.3872 | 2281.1398 | 2281.1637 | -10.47 | 1 | 8 | 1.6 | 1 |  | NLDLENDILYMRAHQALPR |
|  | 481 | **435.7740** | **869.5335** | **869.5447** | **-12.80** | **1** | **8** | **1** | **1** |  | **VVVERLR** |
|  | 2566 | **462.5988** | **1384.7746** | **1384.7714** | **2.32** | **1** | **8** | **1.3** | **1** |  | **WGDIKINIIGEK** |
|  | 2685 | **709.8537** | **1417.6928** | **1417.6772** | **11.0** | **1** | **8** | **2.4** | **1** |  | **SFANDDRHVMVK** |
|  | 2609 | 698.3240 | 1394.6335 | 1394.6170 | 11.9 | 1 | 8 | 1.2 | 1 |  | DGAVKAMEEMNGK + Oxidation (M) |
|  | 3765 | **469.0007** | **1871.9736** | **1871.9714** | **1.14** | **1** | **8** | **1.6** | **1** |  | **QTGAAADIPQPAVRHQGR** |
|  | 773 | **477.7643** | **953.5141** | **953.5294** | **-16.04** | **0** | **8** | **1.4** | **1** |  | **IVDPNGLAR** |
|  | 2216 | **645.3415** | **1288.6684** | **1288.6623** | **4.77** | **1** | **8** | **2.9** | **1** |  | **VDATAETDLAKR** |
|  | 2220 | **431.2139** | **1290.6198** | **1290.6238** | **-3.10** | **0** | **8** | **2.2** | **1** |  | **CQSLDSALLER** |
|  | 3254 | **820.4751** | **1638.9356** | **1638.9093** | **16.1** | **0** | **8** | **0.53** | **1** |  | **LLAGAYHAVVLLDER** |
|  | 212 | **408.2510** | **814.4874** | **814.4912** | **-4.67** | **1** | **8** | **2** | **1** |  | **EIANKIK** |
|  | 420 | **428.7673** | **855.5200** | **855.5178** | **2.57** | **0** | **8** | **1.5** | **1** |  | **VTIASLPR** |
|  | 1134 | **523.2853** | **1044.5560** | **1044.5716** | **-14.94** | **0** | **8** | **3.1** | **1** |  | **LSSAHVYLR** |
|  | 2897 | **744.8790** | **1487.7435** | **1487.7369** | **4.47** | **1** | **8** | **2.3** | **1** |  | **RTDLTQDDFHLK** |
|  | 254 | 414.7117 | 827.4089 | 827.4184 | -11.50 | 1 | 8 | 1.6 | 1 |  | HTRQMR |
|  | 4138 | **737.7061** | **2210.0965** | **2210.1188** | **-10.06** | **0** | **8** | **1.7** | **1** |  | **VGVTVAQTTMEPHLLEACVR** |
|  | 999 | **508.2675** | **1014.5205** | **1014.5094** | **10.9** | **1** | **8** | **2.4** | **1** |  | **AEPKAEAGSR** |
|  | 1124 | **523.2842** | **1044.5539** | **1044.5716** | **-16.93** | **0** | **8** | **2.8** | **1** |  | **LSSAHVYLR** |
|  | 4085 | **1074.5636** | **2147.1126** | **2147.1078** | **2.24** | **1** | **8** | **1.3** | **1** |  | **GDLNINMTSPMGTKSILLSR** |
|  | 446 | **434.7663** | **867.5180** | **867.5079** | **11.6** | **0** | **8** | **0.68** | **1** |  | **VPRPFPR** |
|  | 1119 | **523.2835** | **1044.5525** | **1044.5564** | **-3.72** | **1** | **8** | **2.9** | **1** |  | **ESAVIARDGK** |
|  | 831 | **486.7990** | **971.5834** | **971.5763** | **7.27** | **0** | **8** | **1.8** | **1** |  | **AAAIASSLIR** |
|  | 2541 | **690.8441** | **1379.6736** | **1379.6656** | **5.77** | **0** | **8** | **2.2** | **1** |  | **FISMTSAAPHFR + Oxidation (M)** |
|  | 3057 | **774.8688** | **1547.7230** | **1547.7177** | **3.40** | **0** | **8** | **1.6** | **1** |  | **EIYYITEDMSIR + Oxidation (M)** |
|  | 1450 | **551.7939** | **1101.5732** | **1101.5527** | **18.6** | **1** | **8** | **2.9** | **1** |  | **AVRADTGQER** |
|  | 1244 | **530.2936** | **1058.5726** | **1058.5608** | **11.2** | **0** | **8** | **2.7** | **1** |  | **VAELNDVTAK** |
|  | 202 | 406.7524 | 811.4903 | 811.4804 | 12.2 | 0 | 8 | 0.77 | 1 |  | AVTPVPTK |
|  | 1394 | **545.7717** | **1089.5288** | **1089.5124** | **15.0** | **0** | **8** | **2.8** | **1** |  | **LEEGEVAMGR** |
|  | 1578 | **564.8350** | **1127.6555** | **1127.6662** | **-9.54** | **1** | **8** | **1.5** | **1** |  | **TRISNLPTVK** |
|  | 2266 | **651.8606** | **1301.7066** | **1301.7033** | **2.57** | **1** | **8** | **2.2** | **1** |  | **ATARWWWVVK** |
|  | 3364 | **852.9042** | **1703.7939** | **1703.8090** | **-8.83** | **0** | **8** | **1.5** | **1** |  | **CPAPSFSWTGAALSPR** |
|  | 2344 | **661.8328** | **1321.6510** | **1321.6361** | **11.2** | **0** | **8** | **2.1** | **1** |  | **ASLESTSDLGASGK** |
|  | 3374 | **427.4663** | **1705.8361** | **1705.8370** | **-0.49** | **0** | **8** | **2.3** | **1** |  | **AEQSIAELSSSENTLK** |
|  | 4276 | **1163.0259** | **2324.0372** | **2324.0309** | **2.70** | **1** | **8** | **0.98** | **1** |  | **LMVSMLDRDMSGTMGFNEFK + Oxidation (M)** |
|  | 2055 | **624.8144** | **1247.6142** | **1247.6180** | **-3.01** | **1** | **8** | **2.5** | **1** |  | **CVEQKTDALGK** |
|  | 3858 | 641.9742 | 1922.9009 | 1922.8680 | 17.1 | 0 | 8 | 1.4 | 1 |  | EDPLAADGVYTASQMPSR + Oxidation (M) |
|  | 1303 | **357.5061** | **1069.4966** | **1069.4941** | **2.31** | **0** | **8** | **1.4** | **1** |  | **WIHQEDSR** |
|  | 2065 | **626.3453** | **1250.6761** | **1250.6731** | **2.41** | **1** | **8** | **1.7** | **1** |  | **LYRSLTAGSQR** |
|  | 50 | **365.2167** | **728.4188** | **728.4181** | **0.98** | **0** | **8** | **3.2** | **1** |  | **GISSLPR** |
|  | 1200 | **526.2933** | **1050.5721** | **1050.5644** | **7.30** | **1** | **8** | **1.7** | **1** |  | **GLFGVAGKMR + Oxidation (M)** |
|  | 2510 | **456.2892** | **1365.8459** | **1365.8344** | **8.47** | **0** | **8** | **0.15** | **1** |  | **AQLGLGEIILAIR** |
|  | 2881 | **739.9135** | **1477.8125** | **1477.8042** | **5.63** | **1** | **8** | **1.3** | **1** |  | **VTQLIRGFGSSWK** |
|  | 1446 | **551.3042** | **1100.5938** | **1100.5801** | **12.5** | **0** | **8** | **2.5** | **1** |  | **TPFMSHILR** |
|  | 1602 | **567.2502** | **1132.4859** | **1132.4859** | **0.02** | **0** | **8** | **0.68** | **1** |  | **YSDMSPVYR + Oxidation (M)** |
|  | 3067 | **517.5903** | **1549.7492** | **1549.7332** | **10.3** | **1** | **8** | **2.2** | **1** |  | **ESGSTAASEEARSLR** |
|  | 3870 | **643.6203** | **1927.8391** | **1927.8703** | **-16.19** | **1** | **8** | **0.78** | **1** |  | **GPEGAMGLPGMRGPSGPGCK + Oxidation (M)** |
|  | 645 | **458.7878** | **915.5611** | **915.5575** | **3.92** | **0** | **8** | **0.56** | **1** |  | **VLLTMIAR** |
|  | 1557 | **563.2748** | **1124.5350** | **1124.5397** | **-4.16** | **1** | **8** | **2.1** | **1** |  | **NGVRGCFSTK** |
|  | 2496 | **682.8490** | **1363.6834** | **1363.6732** | **7.52** | **0** | **8** | **2.6** | **1** |  | **AGSLDLNFTSPSR** |
|  | 3493 | **881.3609** | **1760.7072** | **1760.7233** | **-9.11** | **1** | **8** | **0.17** | **1** |  | **NDKYAGEEGMIEDMK + 2 Oxidation (M)** |
|  | 4397 | **870.7023** | **2609.0850** | **2609.1288** | **-16.81** | **1** | **8** | **0.23** | **1** |  | **YWCHECGKGFSQSSNLQTHQR** |
|  | 151 | **394.2427** | **786.4708** | **786.4712** | **-0.47** | **1** | **8** | **3.1** | **1** |  | **VKSGAIGR** |
|  | 4071 | **1059.0217** | **2116.0289** | **2116.0145** | **6.83** | **1** | **8** | **2** | **1** |  | **NSTHQNSLEAQKSSDTLTR** |
|  | 367 | **421.7585** | **841.5024** | **841.5021** | **0.32** | **1** | **8** | **1.6** | **1** |  | **VAGAATPKK** |
|  | 1395 | **545.7961** | **1089.5777** | **1089.5852** | **-6.86** | **1** | **8** | **3.2** | **1** |  | **MLSGQALKDK** |
|  | 1302 | **357.5051** | **1069.4935** | **1069.4862** | **6.76** | **0** | **8** | **1.5** | **1** |  | **GLTMYQGER + Oxidation (M)** |
|  | 2568 | **462.5989** | **1384.7749** | **1384.7973** | **-16.14** | **1** | **8** | **1.5** | **1** |  | **MRLLGAAAVAALGR + Oxidation (M)** |
|  | 99 | **380.2028** | **758.3910** | **758.3922** | **-1.60** | **0** | **8** | **3.1** | **1** |  | **APSEISR** |
|  | 2853 | **733.3851** | **1464.7556** | **1464.7613** | **-3.87** | **0** | **8** | **2.3** | **1** |  | **TLYNNQPIDFLK** |
|  | 4128 | **737.7056** | **2210.0951** | **2210.0637** | **14.2** | **1** | **8** | **1.8** | **1** |  | **DDPDGKQEAKPQQAAGMLSPK** |
|  | 1918 | **604.8199** | **1207.6252** | **1207.6118** | **11.1** | **1** | **8** | **2.5** | **1** |  | **TMDVSKLSAEK** |
|  | 2270 | **652.2950** | **1302.5755** | **1302.5800** | **-3.43** | **0** | **8** | **0.84** | **1** |  | **QSNQDAQDIER** |
|  | 2715 | **712.8425** | **1423.6704** | **1423.6732** | **-1.97** | **0** | **8** | **2** | **1** |  | **FTASLDEWTAGAR** |
|  | 118 | **385.2554** | **768.4962** | **768.4858** | **13.6** | **1** | **8** | **0.53** | **1** |  | **GLVQPKK** |
|  | 554 | **447.7403** | **893.4660** | **893.4640** | **2.16** | **0** | **8** | **2.5** | **1** |  | **LSLATMSR + Oxidation (M)** |
|  | 1164 | **523.2859** | **1044.5573** | **1044.5716** | **-13.65** | **0** | **8** | **3** | **1** |  | **LSSAHVYLR** |
|  | 1107 | **522.7697** | **1043.5249** | **1043.5359** | **-10.60** | **1** | **8** | **2.3** | **1** |  | **RINDEIER** |
|  | 3891 | 971.9440 | 1941.8735 | 1941.9003 | -13.80 | 1 | 8 | 1 | 1 |  | QSFNMHDSSSVASKVFR + Oxidation (M) |
|  | 2940 | **752.8678** | **1503.7210** | **1503.7100** | **7.37** | **1** | **8** | **2** | **1** |  | **MRSSNSASASAGPPGK** |
|  | 4255 | **767.0625** | **2298.1657** | **2298.1790** | **-5.81** | **1** | **8** | **1.6** | **1** |  | **AFTESARFGGIAGSSALVMLAAR + Oxidation (M)** |
|  | 955 | **502.2932** | **1002.5718** | **1002.5532** | **18.6** | **1** | **8** | **1.8** | **1** |  | **AVCGLDKLK** |
|  | 1833 | **596.3182** | **1190.6219** | **1190.6216** | **0.25** | **1** | **8** | **2.5** | **1** |  | **KTEAELEMLK** |
|  | 3918 | **656.9642** | **1967.8707** | **1967.8928** | **-11.25** | **0** | **8** | **0.75** | **1** |  | **SNNMATNGCILLGETTEK + Oxidation (M)** |
|  | 613 | **454.7295** | **907.4444** | **907.4433** | **1.23** | **0** | **8** | **2.3** | **1** |  | **SMSTLTPR + Oxidation (M)** |
|  | 2217 | **645.3418** | **1288.6690** | **1288.6696** | **-0.46** | **1** | **8** | **3.2** | **1** |  | **KEILLEGCAEK** |
|  | 2345 | **661.8334** | **1321.6522** | **1321.6435** | **6.58** | **1** | **8** | **2.3** | **1** |  | **TLEESKEMDIK** |
|  | 2923 | **747.8663** | **1493.7181** | **1493.7474** | **-19.61** | **0** | **8** | **2.1** | **1** |  | **TTFNSSQLQALER** |
|  | 3587 | **897.9103** | **1793.8061** | **1793.7787** | **15.3** | **0** | **8** | **1.4** | **1** |  | **FMVDIACGMEYLSSR + Oxidation (M)** |
|  | 611 | **454.2318** | **906.4491** | **906.4481** | **1.18** | **0** | **8** | **3.2** | **1** |  | **IINGDTMK + Oxidation (M)** |
|  | 1254 | **531.2838** | **1060.5531** | **1060.5513** | **1.70** | **1** | **8** | **3.2** | **1** |  | **ISSERSGTPK** |
|  | 3065 | **775.8817** | **1549.7487** | **1549.7705** | **-14.01** | **1** | **8** | **2.3** | **1** |  | **QSTCEAVMILRSR** |
|  | 1147 | **523.2856** | **1044.5566** | **1044.5716** | **-14.36** | **0** | **8** | **3.1** | **1** |  | **LSSAHVYLR** |
|  | 1813 | **594.2868** | **1186.5590** | **1186.5804** | **-18.03** | **1** | **8** | **1.7** | **1** |  | **KLYANMFER + Oxidation (M)** |
|  | 2653 | **703.8563** | **1405.6981** | **1405.7215** | **-16.64** | **1** | **8** | **2.6** | **1** |  | **QRIQGPFSNFGR** |
|  | 4240 | **1142.0945** | **2282.1744** | **2282.1365** | **16.6** | **1** | **8** | **1.6** | **1** |  | **NNGFFQKLNVTEGAMQDLLK + Oxidation (M)** |
|  | 2950 | **754.3731** | **1506.7315** | **1506.7453** | **-9.16** | **0** | **8** | **2.3** | **1** |  | **FAELVDELDLTDK** |
|  | 1059 | 514.2805 | 1026.5465 | 1026.5346 | 11.6 | 0 | 8 | 1.9 | 1 |  | GLSPSPSPASK |
|  | 2478 | **453.5720** | **1357.6942** | **1357.7089** | **-10.81** | **1** | **8** | **2.8** | **1** |  | **QSLDPTVDEVKK** |
|  | 3613 | **600.2749** | **1797.8029** | **1797.8138** | **-6.08** | **0** | **8** | **0.94** | **1** |  | **DGYMGIGLSAQGVNMNR + Oxidation (M)** |
|  | 447 | **434.7663** | **867.5181** | **867.5079** | **11.8** | **0** | **8** | **0.76** | **1** |  | **VPRPFPR** |
|  | 2449 | 677.8455 | 1353.6765 | 1353.6749 | 1.16 | 1 | 8 | 2.5 | 1 |  | AGPTERGAQGSPAR |
|  | 2732 | **714.4330** | **1426.8514** | **1426.8296** | **15.3** | **1** | **8** | **0.36** | **1** |  | **LNPIVVKEVFNR** |
|  | 98 | **380.2027** | **758.3909** | **758.3922** | **-1.76** | **0** | **8** | **3.3** | **1** |  | **APSEISR** |
|  | 464 | **435.7738** | **869.5331** | **869.5446** | **-13.27** | **1** | **8** | **1.2** | **1** |  | **RLALLER** |
|  | 276 | 417.2506 | 832.4866 | 832.4919 | -6.35 | 1 | 8 | 1.2 | 1 |  | LRFELR |
|  | 1974 | **614.3379** | **1226.6612** | **1226.6731** | **-9.66** | **1** | **8** | **2.2** | **1** |  | **LEAARAAEQLR** |
|  | 2719 | **712.8787** | **1423.7428** | **1423.7493** | **-4.59** | **1** | **8** | **2** | **1** |  | **SVMSLAEAGKLYR** |
|  | 572 | **450.2678** | **898.5210** | **898.5348** | **-15.37** | **1** | **8** | **1.9** | **1** |  | **VATISPRR** |
|  | 1002 | **508.2790** | **1014.5434** | **1014.5458** | **-2.37** | **0** | **8** | **3.2** | **1** |  | **LQLAQESAR** |
|  | 1575 | **564.8012** | **1127.5877** | **1127.6087** | **-18.60** | **0** | **8** | **2.5** | **1** |  | **GNIWEQILR** |
|  | 1662 | **575.7905** | **1149.5664** | **1149.5601** | **5.49** | **1** | **8** | **2.7** | **1** |  | **EWTTLCKGR** |
|  | 2124 | **632.3502** | **1262.6859** | **1262.6718** | **11.2** | **1** | **8** | **2.3** | **1** |  | **TTEVGSVSEVKK** |
|  | 847 | **487.7902** | **973.5658** | **973.5821** | **-16.74** | **1** | **8** | **1.6** | **1** |  | **GNLLRVFR** |
|  | 2474 | **679.8413** | **1357.6681** | **1357.6950** | **-19.82** | **1** | **8** | **2.6** | **1** |  | **VQATDADAGLNRK** |
|  | 3086 | **777.8953** | **1553.7761** | **1553.7719** | **2.70** | **1** | **8** | **2.5** | **1** |  | **SSSMASLRSPSTSIK + Oxidation (M)** |
|  | 149 | 394.2186 | 786.4227 | 786.4137 | 11.5 | 0 | 8 | 3.9 | 1 |  | LGTFHGR |
|  | 2026 | **620.3805** | **1238.7464** | **1238.7459** | **0.45** | **1** | **8** | **0.47** | **1** |  | **RLAVQEILAAR** |
|  | 4012 | **513.7503** | **2050.9721** | **2051.0099** | **-18.41** | **0** | **8** | **1.8** | **1** |  | **NVIFEISPTEEVGDFEVK** |
|  | 2863 | **490.9074** | **1469.7002** | **1469.7124** | **-8.27** | **1** | **8** | **1.8** | **1** |  | **SNFNKHQTTHTR** |
|  | 4217 | **756.6651** | **2266.9735** | **2266.9607** | **5.62** | **0** | **8** | **0.47** | **1** |  | **SEAAEAGNGAETMAAEAESAQTR + Oxidation (M)** |
|  | 3319 | **841.4479** | **1680.8813** | **1680.8545** | **15.9** | **0** | **8** | **2.1** | **1** |  | **LCFDGIEIDILFAR** |
|  | 4309 | **786.0643** | **2355.1712** | **2355.1488** | **9.49** | **1** | **8** | **1.9** | **1** |  | **NKPPMELDLNSSSEDNKPGKR** |
|  | 719 | **468.7244** | **935.4342** | **935.4382** | **-4.29** | **1** | **8** | **2.1** | **1** |  | **AEQDMKAK + Oxidation (M)** |
|  | 1166 | **523.2861** | **1044.5576** | **1044.5386** | **18.2** | **0** | **8** | **3.3** | **1** |  | **LGVNDCVLR** |
|  | 3030 | **770.8868** | **1539.7590** | **1539.7656** | **-4.31** | **0** | **8** | **2.7** | **1** |  | **HNTVLMAWPASWK** |
|  | 1174 | **523.2877** | **1044.5609** | **1044.5788** | **-17.16** | **1** | **8** | **3.3** | **1** |  | **SISRPRSSR** |
|  | 3523 | **589.9728** | **1766.8965** | **1766.9026** | **-3.42** | **0** | **8** | **2** | **1** |  | **VPGNTPLLFSTYVMGR + Oxidation (M)** |
|  | 4195 | **749.4065** | **2245.1976** | **2245.1776** | **8.93** | **1** | **8** | **1.1** | **1** |  | **ALANESGLNFLAIKGPELMNK + Oxidation (M)** |
|  | 4212 | **755.3388** | **2262.9944** | **2262.9674** | **11.9** | **1** | **8** | **0.72** | **1** |  | **SASNHSEMFFADQVDKCYK** |
|  | 1117 | **523.2692** | **1044.5239** | **1044.5352** | **-10.83** | **1** | **8** | **2.8** | **1** |  | **WKQAEDIR** |
|  | 2063 | **625.8096** | **1249.6046** | **1249.6197** | **-12.11** | **1** | **8** | **2.4** | **1** |  | **TMTGGRAQSIGR + Oxidation (M)** |
|  | 2313 | **658.3826** | **1314.7506** | **1314.7660** | **-11.69** | **1** | **8** | **1.4** | **1** |  | **KLIDAVGFSPLR** |
|  | 2408 | 671.3773 | 1340.7400 | 1340.7300 | 7.46 | 0 | 8 | 1.8 | 1 |  | QLLDQVEQIQK |
|  | 2996 | **763.8793** | **1525.7440** | **1525.7420** | **1.33** | **1** | **8** | **2.1** | **1** |  | **QPGALTQGHSCSRK** |
|  | 786 | **478.7799** | **955.5453** | **955.5451** | **0.28** | **1** | **8** | **2.1** | **1** |  | **AKVSSLSHK** |
|  | 1953 | **611.3086** | **1220.6026** | **1220.6010** | **1.35** | **1** | **8** | **3.1** | **1** |  | **ASAYNNRAQAR** |
|  | 3585 | **897.9103** | **1793.8060** | **1793.7787** | **15.2** | **0** | **8** | **1.5** | **1** |  | **FMVDIACGMEYLSSR + Oxidation (M)** |
|  | 4187 | **747.0497** | **2238.1274** | **2238.1136** | **6.14** | **1** | **8** | **1.9** | **1** |  | **ELGLIRKPASFMTSICDER + Oxidation (M)** |
|  | 3277 | **829.3686** | **1656.7226** | **1656.7487** | **-15.74** | **1** | **8** | **0.83** | **1** |  | **MAMIQEEEQNFKK + 2 Oxidation (M)** |
|  | 896 | 495.2318 | 988.4491 | 988.4470 | 2.12 | 0 | 8 | 1.4 | 1 |  | MPVDVCPR + Oxidation (M) |
|  | 2014 | **618.8222** | **1235.6298** | **1235.6510** | **-17.09** | **1** | **8** | **3** | **1** |  | **AKLDSFLSEAR** |
|  | 361 | **421.7583** | **841.5021** | **841.5134** | **-13.36** | **1** | **8** | **1.8** | **1** |  | **AAANKLVR** |
|  | 566 | **449.7718** | **897.5290** | **897.5283** | **0.77** | **0** | **8** | **1.2** | **1** |  | **LLAESLPR** |
|  | 1905 | **402.2031** | **1203.5876** | **1203.5893** | **-1.41** | **1** | **8** | **2.7** | **1** |  | **LCGKPKSHFM** |
|  | 4376 | **837.7530** | **2510.2371** | **2510.2549** | **-7.07** | **1** | **8** | **1.5** | **1** |  | **ASVLLLGPEPGMAWDETQPPKMK + Oxidation (M)** |
|  | 490 | **435.7747** | **869.5349** | **869.5447** | **-11.24** | **1** | **7** | **1.3** | **1** |  | **AKAKPVTR** |
|  | 1462 | 552.7854 | 1103.5562 | 1103.5611 | -4.39 | 0 | 7 | 3.7 | 1 |  | SWQENISLK |
|  | 3178 | **804.4066** | **1606.7987** | **1606.7881** | **6.61** | **1** | **7** | **2.6** | **1** |  | **SMPKDAQMMAQILK + Oxidation (M)** |
|  | 3539 | **890.9028** | **1779.7910** | **1779.8171** | **-14.68** | **1** | **7** | **1.4** | **1** |  | **NTYTNCSLIKYMEK + Oxidation (M)** |
|  | 3699 | **461.7161** | **1842.8354** | **1842.8505** | **-8.18** | **1** | **7** | **1.8** | **1** |  | **MMHYFSQRTSEAAIR + Oxidation (M)** |
|  | 3842 | **955.9404** | **1909.8663** | **1909.8815** | **-7.95** | **0** | **7** | **1.5** | **1** |  | **ISCFGSGSCPHFLAAATK** |
|  | 459 | **435.7738** | **869.5330** | **869.5447** | **-13.44** | **1** | **7** | **1.4** | **1** |  | **VVVERLR** |
|  | 4372 | **834.4221** | **2500.2444** | **2500.2025** | **16.7** | **0** | **7** | **1.8** | **1** |  | **QSLTMFVLIMNGCHIEIDAHR + Oxidation (M)** |
|  | 722 | **468.7504** | **935.4863** | **935.4937** | **-7.88** | **1** | **7** | **2.6** | **1** |  | **AAQPHREK** |
|  | 870 | **491.2721** | **980.5297** | **980.5264** | **3.38** | **1** | **7** | **2.1** | **1** |  | **RGLSHGAQR** |
|  | 2296 | **655.3227** | **1308.6308** | **1308.6357** | **-3.71** | **1** | **7** | **2.3** | **1** |  | **NQSMNLFQRR + Oxidation (M)** |
|  | 4388 | **861.4381** | **2581.2925** | **2581.3031** | **-4.11** | **1** | **7** | **1.7** | **1** |  | **TIHEVNVQGTRNVIEACVQTGTR** |
|  | 404 | **426.7343** | **851.4541** | **851.4575** | **-3.95** | **0** | **7** | **2.3** | **1** |  | **SIMFNLK** |
|  | 3575 | **896.9698** | **1791.9250** | **1791.9223** | **1.54** | **0** | **7** | **2** | **1** |  | **ILDIANMLGLSNTVMR + 2 Oxidation (M)** |
|  | 1016 | **510.7718** | **1019.5290** | **1019.5321** | **-3.02** | **1** | **7** | **3.3** | **1** |  | **MLKVEQEK + Oxidation (M)** |
|  | 3939 | **1003.0240** | **2004.0334** | **2004.0415** | **-4.02** | **1** | **7** | **1.8** | **1** |  | **SEISPEGEKYKPLITGEK** |
|  | 1413 | **547.2787** | **1092.5429** | **1092.5386** | **3.96** | **0** | **7** | **3.1** | **1** |  | **SFGPAVVMNR + Oxidation (M)** |
|  | 543 | **443.7713** | **885.5280** | **885.5284** | **-0.34** | **0** | **7** | **2.7** | **1** |  | **LSEIVGLR** |
|  | 550 | **446.7654** | **891.5163** | **891.5178** | **-1.64** | **0** | **7** | **1.7** | **1** |  | **VTAVIYAR** |
|  | 1135 | **523.2853** | **1044.5561** | **1044.5716** | **-14.82** | **0** | **7** | **3.6** | **1** |  | **LSSAHVYLR** |
|  | 2743 | **476.9195** | **1427.7368** | **1427.7117** | **17.5** | **0** | **7** | **2.6** | **1** |  | **AGGGAGGSGGSGPSAILR** |
|  | 392 | **423.7190** | **845.4234** | **845.4355** | **-14.37** | **1** | **7** | **3.7** | **1** |  | **QKNDVSR** |
|  | 3519 | **883.8954** | **1765.7763** | **1765.7723** | **2.28** | **1** | **7** | **0.95** | **1** |  | **DAVQNCCGISKTEER** |
|  | 3631 | **603.9469** | **1808.8189** | **1808.8332** | **-7.90** | **1** | **7** | **1.5** | **1** |  | **MDAMLLRQCPTQGTR + 2 Oxidation (M)** |
|  | 825 | **485.2553** | **968.4961** | **968.4815** | **15.1** | **0** | **7** | **2.2** | **1** |  | **IGDTSVSYK** |
|  | 3181 | **804.8991** | **1607.7837** | **1607.7831** | **0.34** | **0** | **7** | **3.2** | **1** |  | **SAPGSPDQAYDFLLK** |
|  | 3518 | **883.8954** | **1765.7762** | **1765.7723** | **2.21** | **1** | **7** | **0.95** | **1** |  | **DAVQNCCGISKTEER** |
|  | 3526 | **885.8925** | **1769.7705** | **1769.7674** | **1.73** | **1** | **7** | **0.75** | **1** |  | **MTGFMKGLYTDAEMK + 3 Oxidation (M)** |
|  | 1698 | **580.7545** | **1159.4944** | **1159.4815** | **11.1** | **0** | **7** | **0.57** | **1** |  | **SYSPTEMSSR + Oxidation (M)** |
|  | 1750 | **586.2778** | **1170.5410** | **1170.5227** | **15.6** | **0** | **7** | **1.7** | **1** |  | **MSSGGYTDPLK + Oxidation (M)** |
|  | 4541 | **1393.7462** | **4178.2168** | **4178.1618** | **13.2** | **1** | **7** | **0.39** | **1** |  | **QLVMTMGVADSQLFFYPQLLPIHTLDVKSTMLPAAVR + 3 Oxidation (M)** |
|  | 1155 | **523.2857** | **1044.5569** | **1044.5716** | **-14.11** | **0** | **7** | **3.5** | **1** |  | **LSSAHVYLR** |
|  | 1607 | **567.7933** | **1133.5721** | **1133.5750** | **-2.59** | **1** | **7** | **3.8** | **1** |  | **GETIKDALCK** |
|  | 3155 | **797.8892** | **1593.7638** | **1593.7920** | **-17.69** | **1** | **7** | **2.2** | **1** |  | **ISKTLNMTTSPEEK + Oxidation (M)** |
|  | 1153 | **523.2856** | **1044.5567** | **1044.5424** | **13.7** | **1** | **7** | **3.5** | **1** |  | **TASVNQRNR** |
|  | 368 | **421.7585** | **841.5025** | **841.5021** | **0.39** | **1** | **7** | **1.9** | **1** |  | **VAGAATPKK** |
|  | 749 | **473.2792** | **944.5439** | **944.5291** | **15.7** | **0** | **7** | **2.7** | **1** |  | **GLGIISSNGK** |
|  | 1894 | **601.8049** | **1201.5953** | **1201.5802** | **12.6** | **0** | **7** | **2.9** | **1** |  | **LFMDSFTLGR + Oxidation (M)** |
|  | 2309 | **657.8903** | **1313.7661** | **1313.7529** | **10.0** | **0** | **7** | **1** | **1** |  | **ILALQLCGWIK** |
|  | 152 | **394.2501** | **786.4856** | **786.4712** | **18.3** | **1** | **7** | **1.9** | **1** |  | **LGSVQRK** |
|  | 3268 | **826.9007** | **1651.7868** | **1651.8100** | **-14.02** | **1** | **7** | **2.7** | **1** |  | **SRHCELEQGLQAPK** |
|  | 3649 | **908.9002** | **1815.7857** | **1815.7821** | **1.99** | **0** | **7** | **0.77** | **1** |  | **VGTYAVCWCSWNTGR** |
|  | 1455 | **552.2685** | **1102.5224** | **1102.5367** | **-12.93** | **1** | **7** | **2.9** | **1** |  | **ALGAEDTDRR** |
|  | 2864 | **736.3483** | **1470.6821** | **1470.7024** | **-13.81** | **1** | **7** | **1.8** | **1** |  | **EDPSGAAVPEMPKK + Oxidation (M)** |
|  | 3208 | **812.4069** | **1622.7993** | **1622.7862** | **8.09** | **0** | **7** | **2.4** | **1** |  | **AISSYFVSTMSSSIK + Oxidation (M)** |
|  | 1400 | **546.2720** | **1090.5294** | **1090.5441** | **-13.46** | **1** | **7** | **3.3** | **1** |  | **EKLMTQADR** |
|  | 3247 | **546.9580** | **1637.8522** | **1637.8334** | **11.5** | **1** | **7** | **2.2** | **1** |  | **SILNAYPSEKEMLK + Oxidation (M)** |
|  | 3708 | **925.4119** | **1848.8093** | **1848.8359** | **-14.40** | **1** | **7** | **1.4** | **1** |  | **VMERQGWAEGQGLGCR + Oxidation (M)** |
|  | 3753 | **623.6901** | **1868.0485** | **1868.0665** | **-9.64** | **1** | **7** | **0.78** | **1** |  | **LQAMLQELLVSAQRLR** |
|  | 2049 | **622.8533** | **1243.6921** | **1243.6707** | **17.2** | **1** | **7** | **2.4** | **1** |  | **QCALAALRDVK** |
|  | 623 | **456.7800** | **911.5454** | **911.5301** | **16.8** | **1** | **7** | **0.94** | **1** |  | **GRQVTVPR** |
|  | 1523 | **559.2948** | **1116.5750** | **1116.5676** | **6.67** | **1** | **7** | **3.7** | **1** |  | **SLSFKQDHR** |
|  | 2778 | **719.8436** | **1437.6726** | **1437.6592** | **9.34** | **0** | **7** | **2** | **1** |  | **LAEAEQMALMGSR + 2 Oxidation (M)** |
|  | 2780 | **719.8514** | **1437.6882** | **1437.6823** | **4.08** | **1** | **7** | **2.4** | **1** |  | **DCPGFVFTPRSR** |
|  | 2922 | **747.8658** | **1493.7171** | **1493.6933** | **16.0** | **0** | **7** | **2.3** | **1** |  | **WMEVIQGASSSAGR + Oxidation (M)** |
|  | 538 | **442.7636** | **883.5126** | **883.5127** | **-0.12** | **0** | **7** | **1.5** | **1** |  | **NLNTPVVK** |
|  | 1857 | **598.7836** | **1195.5526** | **1195.5391** | **11.3** | **0** | **7** | **2.3** | **1** |  | **TAGDSSPMSISK + Oxidation (M)** |
|  | 2002 | **617.7944** | **1233.5742** | **1233.5837** | **-7.71** | **0** | **7** | **2.1** | **1** |  | **TQSELRPSETS** |
|  | 1428 | **548.3450** | **1094.6754** | **1094.6560** | **17.7** | **1** | **7** | **0.3** | **1** |  | **RSLSPILPGR** |
|  | 3837 | **637.3051** | **1908.8933** | **1908.9040** | **-5.58** | **1** | **7** | **1.9** | **1** |  | **EEDFRMYHEILGQVK + Oxidation (M)** |
|  | 288 | **421.7579** | **841.5012** | **841.5021** | **-1.13** | **1** | **7** | **2.1** | **1** |  | **VAGAATPKK** |
|  | 3097 | **782.8519** | **1563.6892** | **1563.6873** | **1.18** | **0** | **7** | **1.2** | **1** |  | **GASLHSSSGGGSSGSSSR** |
|  | 3367 | **853.4511** | **1704.8877** | **1704.8696** | **10.6** | **1** | **7** | **2.3** | **1** |  | **DTKPGVPHIREWDR** |
|  | 3520 | **883.8962** | **1765.7778** | **1765.7723** | **3.11** | **1** | **7** | **1.1** | **1** |  | **DAVQNCCGISKTEER** |
|  | 4033 | 691.6460 | 2071.9162 | 2071.9179 | -0.85 | 0 | 7 | 0.75 | 1 |  | GPPFGSPMGHPGPMPPHGMR + 2 Oxidation (M) |
|  | 4313 | **789.0742** | **2364.2007** | **2364.1744** | **11.1** | **1** | **7** | **1.8** | **1** |  | **QATVGDINTERPGMLDFTGKAK + Oxidation (M)** |
|  | 147 | **393.2595** | **784.5045** | **784.5058** | **-1.69** | **0** | **7** | **1.7** | **1** |  | **LIAVLEK** |
|  | 1323 | 538.7539 | 1075.4931 | 1075.4968 | -3.41 | 0 | 7 | 1.8 | 1 |  | GVASLCQLDN |
|  | 2858 | **735.3637** | **1468.7129** | **1468.7344** | **-14.66** | **0** | **7** | **2.4** | **1** |  | **GPVLPEADQQMLR + Oxidation (M)** |
|  | 1596 | **566.7551** | **1131.4957** | **1131.4913** | **3.85** | **0** | **7** | **1.2** | **1** |  | **MVGHSNATCR** |
|  | 2554 | **692.4009** | **1382.7872** | **1382.7670** | **14.6** | **0** | **7** | **1.3** | **1** |  | **AAAGALEGVWLAVR** |
|  | 1057 | **514.2803** | **1026.5460** | **1026.5498** | **-3.73** | **0** | **7** | **2.2** | **1** |  | **TGSLALFYR** |
|  | 1118 | **523.2695** | **1044.5245** | **1044.5386** | **-13.51** | **0** | **7** | **3.6** | **1** |  | **LGVNDCVLR** |
|  | 3242 | **819.4004** | **1636.7863** | **1636.7879** | **-0.94** | **1** | **7** | **2.7** | **1** |  | **ERGEIEVDIQFMR + Oxidation (M)** |
|  | 1653 | **574.2790** | **1146.5435** | **1146.5453** | **-1.56** | **0** | **7** | **2.5** | **1** |  | **MMLEFFGIK + 2 Oxidation (M)** |
|  | 2176 | **639.8754** | **1277.7363** | **1277.7343** | **1.55** | **1** | **7** | **0.91** | **1** |  | **FARLVTTTELK** |
|  | 12 | **357.2117** | **712.4089** | **712.4119** | **-4.24** | **0** | **7** | **1.9** | **1** |  | **GLDIPAK** |
|  | 1179 | **523.7773** | **1045.5400** | **1045.5265** | **13.0** | **1** | **7** | **4.6** | **1** |  | **SDSAGGRAGLR** |
|  | 1306 | **536.2933** | **1070.5720** | **1070.5682** | **3.57** | **0** | **7** | **2.9** | **1** |  | **LSLMYSITK + Oxidation (M)** |
|  | 3296 | **833.9497** | **1665.8847** | **1665.9137** | **-17.39** | **1** | **7** | **1.8** | **1** |  | **HTMIRLFVHEVLR + Oxidation (M)** |
|  | 1176 | **523.3037** | **1044.5929** | **1044.5927** | **0.13** | **0** | **7** | **2.1** | **1** |  | **LSAALSSLQR** |
|  | 1966 | **613.3719** | **1224.7292** | **1224.7190** | **8.36** | **0** | **7** | **0.79** | **1** |  | **NIQQLIELVR** |
|  | 2666 | **706.3719** | **1410.7292** | **1410.7064** | **16.2** | **0** | **7** | **2.5** | **1** |  | **TQLALEMYEVAK + Oxidation (M)** |
|  | 2936 | **751.3620** | **1500.7094** | **1500.7144** | **-3.27** | **0** | **7** | **2.1** | **1** |  | **EEIALGGGGFCVHR** |
|  | 3096 | **782.8512** | **1563.6878** | **1563.6922** | **-2.79** | **0** | **7** | **1.1** | **1** |  | **EYQPCVRPCEAR** |
|  | 3781 | **941.4252** | **1880.8359** | **1880.8541** | **-9.66** | **0** | **7** | **1.2** | **1** |  | **AVSFEHLSFGSQDDSAGK** |
|  | 4133 | **737.7059** | **2210.0958** | **2210.1080** | **-5.54** | **1** | **7** | **2.2** | **1** |  | **APVTSGRGPPSEDGPGVPPPSPR** |
|  | 3272 | **827.9406** | **1653.8665** | **1653.8369** | **17.9** | **1** | **7** | **2.1** | **1** |  | **RGSPGILQGPNVCGSR** |
|  | 235 | **412.7530** | **823.4915** | **823.5028** | **-13.67** | **1** | **7** | **0.8** | **1** |  | **RLHTAVK** |
|  | 4004 | **1021.0062** | **2039.9978** | **2040.0316** | **-16.60** | **1** | **7** | **2.2** | **1** |  | **VWIPDPDEVWRSAELTK** |
|  | 1980 | **410.2349** | **1227.6828** | **1227.6683** | **11.7** | **1** | **7** | **3.9** | **1** |  | **SLPRPSKSNSR** |
|  | 4112 | **1094.5400** | **2187.0655** | **2187.1088** | **-19.77** | **1** | **7** | **2.3** | **1** |  | **LFEWLIAPMPPDHFYRR** |
|  | 1792 | **591.8439** | **1181.6732** | **1181.6517** | **18.2** | **0** | **7** | **1.5** | **1** |  | **QVILGNTAGGPR** |
|  | 78 | **378.2655** | **754.5164** | **754.5065** | **13.2** | **0** | **7** | **0.35** | **1** |  | **ILQILR** |
|  | 421 | **428.7676** | **855.5207** | **855.5290** | **-9.75** | **1** | **7** | **2** | **1** |  | **IAQKQIR** |
|  | 3055 | **516.5849** | **1546.7329** | **1546.7528** | **-12.91** | **1** | **7** | **2.2** | **1** |  | **KGPQGYGFNLHSDK** |
|  | 4144 | **737.7064** | **2210.0974** | **2210.1080** | **-4.79** | **1** | **7** | **2.2** | **1** |  | **APVTSGRGPPSEDGPGVPPPSPR** |
|  | 2731 | **476.6239** | **1426.8499** | **1426.8759** | **-18.25** | **1** | **7** | **0.46** | **1** |  | **TVTLVEAIKQVVK** |
|  | 1162 | **523.2859** | **1044.5573** | **1044.5716** | **-13.65** | **0** | **7** | **3.8** | **1** |  | **LSSAHVYLR** |
|  | 3612 | **899.9085** | **1797.8025** | **1797.8138** | **-6.30** | **0** | **7** | **1.1** | **1** |  | **DGYMGIGLSAQGVNMNR + Oxidation (M)** |
|  | 409 | **427.7764** | **853.5383** | **853.5273** | **12.9** | **0** | **7** | **0.62** | **1** |  | **GLLDVLPK** |
|  | 443 | **434.7658** | **867.5169** | **867.5079** | **10.4** | **0** | **7** | **0.97** | **1** |  | **VPRPFPR** |
|  | 1209 | **526.7996** | **1051.5847** | **1051.5914** | **-6.34** | **1** | **7** | **1.9** | **1** |  | **ELKTVTFSK** |
|  | 1294 | **356.8934** | **1067.6583** | **1067.6451** | **12.4** | **0** | **7** | **0.67** | **1** |  | **GLLARPLNSK** |
|  | 2962 | **505.9216** | **1514.7429** | **1514.7511** | **-5.43** | **1** | **7** | **2.6** | **1** |  | **SMLSKDNTVHDLR** |
|  | 3832 | **636.3398** | **1905.9975** | **1906.0272** | **-15.59** | **1** | **7** | **2** | **1** |  | **DPGHVVEQLSRSLQTLK** |
|  | 1885 | 601.3303 | 1200.6461 | 1200.6285 | 14.7 | 1 | 7 | 3.1 | 1 |  | GLAGPMGEKGLR + Oxidation (M) |
|  | 3438 | **868.4606** | **1734.9067** | **1734.8828** | **13.8** | **0** | **7** | **2.3** | **1** |  | **ENIIAFEEIIEPYR** |
|  | 1806 | **593.2907** | **1184.5669** | **1184.5608** | **5.13** | **1** | **7** | **2.5** | **1** |  | **GEKGAMGEPGPR** |
|  | 3584 | **897.9103** | **1793.8060** | **1793.7787** | **15.2** | **0** | **7** | **1.8** | **1** |  | **FMVDIACGMEYLSSR + Oxidation (M)** |
|  | 4214 | **756.0554** | **2265.1443** | **2265.1675** | **-10.25** | **1** | **7** | **2** | **1** |  | **SAMKTLALLFVGATGAEAEAGGGK + Oxidation (M)** |
|  | 2498 | **682.8527** | **1363.6908** | **1363.7030** | **-8.99** | **1** | **7** | **3.3** | **1** |  | **EQMAISGGFIRR** |
|  | 4111 | **730.0281** | **2187.0624** | **2187.0749** | **-5.71** | **1** | **7** | **2.3** | **1** |  | **GFHPDPEALKGFHPDPDALK** |
|  | 3333 | **846.8858** | **1691.7570** | **1691.7751** | **-10.66** | **0** | **7** | **1.5** | **1** |  | **YELLQEPGGGGSGGESR** |
|  | 3357 | **850.4137** | **1698.8128** | **1698.8222** | **-5.50** | **1** | **7** | **2.4** | **1** |  | **MAATFFGEVVKAPCR + Oxidation (M)** |
|  | 412 | **428.2741** | **854.5336** | **854.5338** | **-0.17** | **1** | **7** | **0.84** | **1** |  | **LAAKSPIR** |
|  | 3073 | **776.8872** | **1551.7597** | **1551.7684** | **-5.56** | **1** | **7** | **2.7** | **1** |  | **CLRCTSLMLGLGR + Oxidation (M)** |
|  | 3313 | **839.9139** | **1677.8133** | **1677.7933** | **11.9** | **1** | **7** | **2.8** | **1** |  | **MFLRFYSDNSVQR + Oxidation (M)** |
|  | 2168 | 638.8644 | 1275.7143 | 1275.6936 | 16.3 | 0 | 7 | 1.7 | 1 |  | GVQGPPGPTGKPGK |
|  | 2175 | **639.8254** | **1277.6362** | **1277.6252** | **8.65** | **1** | **7** | **3.2** | **1** |  | **KEAESSPFVER** |
|  | 2679 | **707.8624** | **1413.7103** | **1413.7286** | **-12.91** | **0** | **7** | **3** | **1** |  | **MQELALSEGALPR** |
|  | 1387 | **544.8020** | **1087.5894** | **1087.5986** | **-8.38** | **1** | **7** | **4.1** | **1** |  | **EAQVLGKTSR** |
|  | 3618 | **902.0060** | **1801.9974** | **1801.9720** | **14.1** | **1** | **7** | **0.91** | **1** |  | **LTMRPLVLQKEESSR + Oxidation (M)** |
|  | 1851 | **598.2731** | **1194.5316** | **1194.5485** | **-14.16** | **1** | **7** | **1.5** | **1** |  | **VANMMADSGKR + Oxidation (M)** |
|  | 2280 | **654.3420** | **1306.6695** | **1306.6881** | **-14.22** | **0** | **7** | **3.2** | **1** |  | **QNITETSFVLR** |
|  | 379 | **423.2262** | **844.4378** | **844.4515** | **-16.21** | **1** | **7** | **4.3** | **1** |  | **AIRENSR** |
|  | 899 | **495.2732** | **988.5319** | **988.5454** | **-13.65** | **1** | **7** | **4** | **1** |  | **RNYQIPAK** |
|  | 1382 | 544.7883 | 1087.5621 | 1087.5509 | 10.3 | 0 | 7 | 4.4 | 1 |  | EQLQTVQDK |
|  | 3421 | **864.3808** | **1726.7470** | **1726.7435** | **2.08** | **0** | **7** | **0.78** | **1** |  | **YDSTSDDSNFLNPPR** |
|  | 1700 | **580.8199** | **1159.6253** | **1159.6277** | **-2.05** | **0** | **7** | **3.3** | **1** |  | **IIWEDIFPK** |
|  | 4510 | **820.8926** | **3279.5415** | **3279.5369** | **1.38** | **0** | **7** | **1.4** | **1** |  | **YPSTGMLVLFFALHVCDEVNVYGFGADSR + Oxidation (M)** |
|  | 2287 | **654.8303** | **1307.6461** | **1307.6656** | **-14.92** | **1** | **7** | **3.2** | **1** |  | **MLRADGDFLVR + Oxidation (M)** |
|  | 3597 | **897.9133** | **1793.8120** | **1793.7899** | **12.3** | **0** | **7** | **1.6** | **1** |  | **TCDWLPKPNMSASCK** |
|  | 414 | **428.7662** | **855.5178** | **855.5178** | **0.08** | **0** | **7** | **2.1** | **1** |  | **SPLSLIAR** |
|  | 2807 | 724.8547 | 1447.6949 | 1447.6912 | 2.59 | 0 | 7 | 3 | 1 |  | QVSLACVCAVGER |
|  | 1128 | **523.2851** | **1044.5556** | **1044.5352** | **19.6** | **1** | **7** | **4.4** | **1** |  | **AEEKGWGIR** |
|  | 1210 | **526.7997** | **1051.5849** | **1051.5914** | **-6.11** | **0** | **7** | **2** | **1** |  | **VATTASVIYK** |
|  | 2394 | **446.9454** | **1337.8143** | **1337.8031** | **8.38** | **1** | **7** | **0.34** | **1** |  | **DRLSLVLVPAQK** |
|  | 2791 | **721.9071** | **1441.7996** | **1441.8140** | **-9.97** | **0** | **7** | **2.2** | **1** |  | **SVLAVENLLTLDR** |
|  | 4540 | **1393.7449** | **4178.2128** | **4178.1995** | **3.17** | **0** | **7** | **0.44** | **1** |  | **GLFLPSCPGCGHPELLQAVGIVGAIIMPHNIYLHSALVK** |
|  | 1211 | **526.7999** | **1051.5853** | **1051.5662** | **18.2** | **0** | **7** | **2** | **1** |  | **SPPSLTPTPR** |
|  | 4168 | **1113.5569** | **2225.0992** | **2225.1224** | **-10.44** | **0** | **7** | **2.3** | **1** |  | **LLNVIDMAIFDFLIGNMDR + Oxidation (M)** |
|  | 4210 | **1129.4924** | **2256.9703** | **2256.9595** | **4.77** | **0** | **7** | **0.71** | **1** |  | **ACGLTSDCCEALSLALSCNR** |
|  | 1610 | **568.3034** | **1134.5923** | **1134.5815** | **9.47** | **1** | **7** | **3.9** | **1** |  | **AVTDAIMSRR + Oxidation (M)** |
|  | 1691 | **579.7928** | **1157.5710** | **1157.5941** | **-19.97** | **1** | **7** | **3.2** | **1** |  | **VQGDWIRER** |
|  | 1779 | **590.3022** | **1178.5899** | **1178.5866** | **2.82** | **0** | **7** | **3.7** | **1** |  | **FQLMQQQTR** |
|  | 3625 | **904.4058** | **1806.7971** | **1806.7664** | **17.0** | **0** | **7** | **1.8** | **1** |  | **MANGGGGGGGSSGGGGGGGGSSLR** |
|  | 252 | **414.2297** | **826.4448** | **826.4337** | **13.4** | **0** | **7** | **2** | **1** |  | **AYASLFR** |
|  | 1633 | **571.2433** | **1140.4721** | **1140.4903** | **-15.94** | **1** | **7** | **0.81** | **1** |  | **ADADTMKAMR + 2 Oxidation (M)** |
|  | 3494 | **881.3619** | **1760.7092** | **1760.7094** | **-0.11** | **1** | **7** | **0.23** | **1** |  | **RNDPECDLCGGDPEK** |
|  | 3572 | **896.9021** | **1791.7896** | **1791.8097** | **-11.21** | **0** | **7** | **1.5** | **1** |  | **HEMTEGVTAYEEKPR + Oxidation (M)** |
|  | 3262 | **824.4050** | **1646.7955** | **1646.8125** | **-10.31** | **1** | **7** | **3.5** | **1** |  | **QNKQVHASHTPGTDK** |
|  | 3338 | **847.8746** | **1693.7346** | **1693.7155** | **11.3** | **0** | **7** | **1** | **1** |  | **YHEDAHMLDTQYR + Oxidation (M)** |
|  | 3919 | **984.9432** | **1967.8719** | **1967.8652** | **3.42** | **1** | **7** | **1** | **1** |  | **YGQVPMCDAGEQCAVRK** |
|  | 2426 | 673.8531 | 1345.6916 | 1345.6725 | 14.2 | 1 | 7 | 4 | 1 |  | NEKEQELDTLK |
|  | 3394 | **859.4226** | **1716.8305** | **1716.8431** | **-7.32** | **1** | **7** | **2.5** | **1** |  | **KDSGVQESSHFLQGAK** |
|  | 1065 | **514.7724** | **1027.5302** | **1027.5484** | **-17.70** | **0** | **7** | **2.7** | **1** |  | **MDVNIAPLR** |
|  | 2301 | **656.3583** | **1310.7021** | **1310.6803** | **16.6** | **1** | **7** | **2.4** | **1** |  | **LARGRPDGEGAGR** |
|  | 2361 | **664.2920** | **1326.5694** | **1326.5656** | **2.89** | **0** | **7** | **1.2** | **1** |  | **NISSMSNMNSSR** |
|  | 637 | 458.7585 | 915.5025 | 915.4848 | 19.4 | 0 | 7 | 4.5 | 1 |  | SLMPSIPR + Oxidation (M) |
|  | 1370 | **542.7505** | **1083.4864** | **1083.4945** | **-7.43** | **0** | **7** | **1.9** | **1** |  | **HLEAQEDSR** |
|  | 1989 | 615.8405 | 1229.6665 | 1229.6616 | 4.01 | 0 | 7 | 3.4 | 1 |  | IGTTVIDLENR |
|  | 3524 | **884.4573** | **1766.9000** | **1766.9026** | **-1.44** | **0** | **7** | **2.4** | **1** |  | **VPGNTPLLFSTYVMGR + Oxidation (M)** |
|  | 1923 | **605.8013** | **1209.5880** | **1209.5919** | **-3.27** | **1** | **7** | **3.2** | **1** |  | **MMGLGLMAKDK + Oxidation (M)** |
|  | 3042 | **514.6307** | **1540.8702** | **1540.8725** | **-1.51** | **0** | **7** | **1.4** | **1** |  | **LIAEQPPHLTPGIR** |
|  | 2957 | **758.3622** | **1514.7099** | **1514.6858** | **16.0** | **1** | **7** | **2** | **1** |  | **VFTKMCDIGDASR + Oxidation (M)** |
|  | 4279 | **775.7208** | **2324.1407** | **2324.1801** | **-16.96** | **1** | **7** | **2.4** | **1** |  | **LKYEVDTSGIYHINQEIFR** |
|  | 3430 | 434.1883 | 1732.7239 | 1732.7219 | 1.19 | 0 | 7 | 0.44 | 1 |  | CPSGSCVMNQYLSSK + Oxidation (M) |
|  | 3100 | **783.8733** | **1565.7320** | **1565.7541** | **-14.11** | **1** | **7** | **2.1** | **1** |  | **LAEAEQMALMGSRK + 2 Oxidation (M)** |
|  | 36 | **363.2182** | **724.4218** | **724.4344** | **-17.39** | **1** | **7** | **1.5** | **1** |  | **SPRLPR** |
|  | 1331 | **539.8326** | **1077.6507** | **1077.6335** | **16.0** | **1** | **7** | **0.66** | **1** |  | **SIYLRLWK** |
|  | 2801 | **724.3552** | **1446.6959** | **1446.6926** | **2.30** | **0** | **7** | **2.8** | **1** |  | **SSGHVTFTMDPIR** |
|  | 1650 | **573.8102** | **1145.6058** | **1145.5863** | **17.0** | **1** | **7** | **4.1** | **1** |  | **VTVGEPCTRK** |
|  | 3510 | **883.8939** | **1765.7732** | **1765.7723** | **0.48** | **1** | **7** | **1.2** | **1** |  | **DAVQNCCGISKTEER** |
|  | 3943 | **1004.9518** | **2007.8891** | **2007.9095** | **-10.15** | **0** | **7** | **1.3** | **1** |  | **SSNDSSLMAGIIYYSQEK + Oxidation (M)** |
|  | 1129 | **523.2852** | **1044.5559** | **1044.5716** | **-15.05** | **0** | **7** | **4.6** | **1** |  | **LSSAHVYLR** |
|  | 2868 | **492.2479** | **1473.7218** | **1473.7470** | **-17.09** | **1** | **7** | **3.3** | **1** |  | **MSGGDTRAAIARPR + Oxidation (M)** |
|  | 3159 | **399.7425** | **1594.9411** | **1594.9406** | **0.30** | **1** | **7** | **0.56** | **1** |  | **LRETKPEVGLVNIK** |
|  | 1033 | **511.8214** | **1021.6283** | **1021.6284** | **-0.15** | **0** | **7** | **0.55** | **1** |  | **AVLGPVPLTR** |
|  | 2385 | **669.3502** | **1336.6858** | **1336.6623** | **17.6** | **1** | **7** | **3.3** | **1** |  | **KDTWGVVSSGSSK** |
|  | 3091 | 780.3774 | 1558.7403 | 1558.7151 | 16.2 | 0 | 7 | 2.5 | 1 |  | TYGGDTGSPEISFTK |
|  | 2813 | **725.3835** | **1448.7524** | **1448.7367** | **10.8** | **1** | **7** | **3.2** | **1** |  | **VPLTKMMEVDVR + 2 Oxidation (M)** |
|  | 476 | **435.7740** | **869.5335** | **869.5447** | **-12.87** | **1** | **7** | **1.6** | **1** |  | **VVVERLR** |
|  | 548 | **446.7470** | **891.4795** | **891.4848** | **-5.90** | **0** | **7** | **3.8** | **1** |  | **GMVSTLLR + Oxidation (M)** |
|  | 1803 | **592.8128** | **1183.6111** | **1183.6197** | **-7.27** | **0** | **7** | **2.8** | **1** |  | **AAAASVPNADGLK** |
|  | 1194 | **350.8623** | **1049.5652** | **1049.5692** | **-3.79** | **0** | **7** | **3** | **1** |  | **QIMGAYLVR** |
|  | 1312 | **537.2826** | **1072.5506** | **1072.5400** | **9.87** | **1** | **7** | **4.3** | **1** |  | **KEGPGEITDK** |
|  | 133 | **388.1813** | **774.3481** | **774.3620** | **-17.95** | **1** | **7** | **1.5** | **1** |  | **DQDGGRK** |
|  | 4180 | **559.7963** | **2235.1559** | **2235.1648** | **-3.96** | **0** | **7** | **2.1** | **1** |  | **GGLEVALQVQVLDTPGYPHSR** |
|  | 208 | **407.7540** | **813.4934** | **813.5072** | **-17.00** | **1** | **7** | **3.6** | **1** |  | **VLRIEGK** |
|  | 1574 | **564.7899** | **1127.5653** | **1127.5757** | **-9.25** | **1** | **7** | **3.2** | **1** |  | **KQPIMADGPR + Oxidation (M)** |
|  | 2298 | **655.3352** | **1308.6559** | **1308.6615** | **-4.29** | **1** | **7** | **3.2** | **1** |  | **KNWSHYSFLK** |
|  | 4093 | **719.6814** | **2156.0224** | **2156.0459** | **-10.93** | **1** | **7** | **2.6** | **1** |  | **QMEAVWKTEVASSSYAIEK** |
|  | 1434 | **548.7910** | **1095.5675** | **1095.5672** | **0.22** | **0** | **6** | **3** | **1** |  | **NHNEIIQTK** |
|  | 2475 | **453.5714** | **1357.6924** | **1357.7024** | **-7.34** | **1** | **6** | **3.6** | **1** |  | **CIKPNEDKVAGK** |
[truncated: 2,209,430 more chars]
