# Supplementary material for: Downregulation of circLIFR exerts cancer-promoting effects on hepatocellular carcinoma in vitro
Source: Front Genet. 2022 Sep 12;13:986322. doi: 10.3389/fgene.2022.986322 (PMC9513674; doi:10.3389/fgene.2022.986322)
Supplement: Supplementary file 1 [file DataSheet3.ZIP › Original dataú¿1ú⌐/Mass spectrometric detection/Enrichment group no. 5/Peptide Summary Report (.._data_20191122_F024828.dat).html]

Peptide Summary Report (../data/20191122/F024828.dat)


# Mascot Search Results

```
User            : 
Email           : 
Search title    : 
MS data file    : GS_82.mgf
Database        : SP_human0806 human0806 (20432 sequences; 11380987 residues)
Timestamp       : 22 Nov 2019 at 01:33:31 GMT

|  |  |  |
| --- | --- | --- |
| Protein hits    : | sp|P04264|K2C1_HUMAN | Keratin, type II cytoskeletal 1 OS=Homo sapiens OX=9606 GN=KRT1 PE=1 SV=6 |
|  | sp|P67809|YBOX1_HUMAN | Nuclease-sensitive element-binding protein 1 OS=Homo sapiens OX=9606 GN=YBX1 PE=1 SV=3 |
|  | sp|P13645|K1C10_HUMAN | Keratin, type I cytoskeletal 10 OS=Homo sapiens OX=9606 GN=KRT10 PE=1 SV=6 |
|  | sp|P35908|K22E_HUMAN | Keratin, type II cytoskeletal 2 epidermal OS=Homo sapiens OX=9606 GN=KRT2 PE=1 SV=2 |
|  | sp|P35527|K1C9_HUMAN | Keratin, type I cytoskeletal 9 OS=Homo sapiens OX=9606 GN=KRT9 PE=1 SV=3 |
|  | sp|P02538|K2C6A_HUMAN | Keratin, type II cytoskeletal 6A OS=Homo sapiens OX=9606 GN=KRT6A PE=1 SV=3 |
|  | sp|P08779|K1C16_HUMAN | Keratin, type I cytoskeletal 16 OS=Homo sapiens OX=9606 GN=KRT16 PE=1 SV=4 |
|  | sp|P02533|K1C14_HUMAN | Keratin, type I cytoskeletal 14 OS=Homo sapiens OX=9606 GN=KRT14 PE=1 SV=4 |
|  | sp|P04259|K2C6B_HUMAN | Keratin, type II cytoskeletal 6B OS=Homo sapiens OX=9606 GN=KRT6B PE=1 SV=5 |
|  | sp|P13647|K2C5_HUMAN | Keratin, type II cytoskeletal 5 OS=Homo sapiens OX=9606 GN=KRT5 PE=1 SV=3 |
|  | sp|P48668|K2C6C_HUMAN | Keratin, type II cytoskeletal 6C OS=Homo sapiens OX=9606 GN=KRT6C PE=1 SV=3 |
|  | sp|Q14764|MVP_HUMAN | Major vault protein OS=Homo sapiens OX=9606 GN=MVP PE=1 SV=4 |
|  | sp|P07437|TBB5_HUMAN | Tubulin beta chain OS=Homo sapiens OX=9606 GN=TUBB PE=1 SV=2 |
|  | sp|P60709|ACTB_HUMAN | Actin, cytoplasmic 1 OS=Homo sapiens OX=9606 GN=ACTB PE=1 SV=1 |
|  | sp|P68371|TBB4B_HUMAN | Tubulin beta-4B chain OS=Homo sapiens OX=9606 GN=TUBB4B PE=1 SV=1 |
|  | sp|Q04695|K1C17_HUMAN | Keratin, type I cytoskeletal 17 OS=Homo sapiens OX=9606 GN=KRT17 PE=1 SV=2 |
|  | sp|Q86YZ3|HORN_HUMAN | Hornerin OS=Homo sapiens OX=9606 GN=HRNR PE=1 SV=2 |
|  | sp|P16989|YBOX3_HUMAN | Y-box-binding protein 3 OS=Homo sapiens OX=9606 GN=YBX3 PE=1 SV=4 |
|  | sp|Q07666|KHDR1_HUMAN | KH domain-containing, RNA-binding, signal transduction-associated protein 1 OS=Homo sapiens OX=9606 GN=KHDRBS1 PE=1 SV=1 |
|  | sp|P05787|K2C8_HUMAN | Keratin, type II cytoskeletal 8 OS=Homo sapiens OX=9606 GN=KRT8 PE=1 SV=7 |
|  | sp|P04350|TBB4A_HUMAN | Tubulin beta-4A chain OS=Homo sapiens OX=9606 GN=TUBB4A PE=1 SV=2 |
|  | sp|O00571|DDX3X_HUMAN | ATP-dependent RNA helicase DDX3X OS=Homo sapiens OX=9606 GN=DDX3X PE=1 SV=3 |
|  | sp|P05783|K1C18_HUMAN | Keratin, type I cytoskeletal 18 OS=Homo sapiens OX=9606 GN=KRT18 PE=1 SV=2 |
|  | sp|Q92804|RBP56_HUMAN | TATA-binding protein-associated factor 2N OS=Homo sapiens OX=9606 GN=TAF15 PE=1 SV=1 |
|  | sp|P31943|HNRH1_HUMAN | Heterogeneous nuclear ribonucleoprotein H OS=Homo sapiens OX=9606 GN=HNRNPH1 PE=1 SV=4 |
|  | sp|P08670|VIME_HUMAN | Vimentin OS=Homo sapiens OX=9606 GN=VIM PE=1 SV=4 |
|  | sp|P02768|ALBU_HUMAN | Serum albumin OS=Homo sapiens OX=9606 GN=ALB PE=1 SV=2 |
|  | sp|P19012|K1C15_HUMAN | Keratin, type I cytoskeletal 15 OS=Homo sapiens OX=9606 GN=KRT15 PE=1 SV=3 |
|  | sp|Q96PK6|RBM14_HUMAN | RNA-binding protein 14 OS=Homo sapiens OX=9606 GN=RBM14 PE=1 SV=2 |
|  | sp|P13646|K1C13_HUMAN | Keratin, type I cytoskeletal 13 OS=Homo sapiens OX=9606 GN=KRT13 PE=1 SV=4 |
|  | sp|P08729|K2C7_HUMAN | Keratin, type II cytoskeletal 7 OS=Homo sapiens OX=9606 GN=KRT7 PE=1 SV=5 |
|  | sp|P68032|ACTC_HUMAN | Actin, alpha cardiac muscle 1 OS=Homo sapiens OX=9606 GN=ACTC1 PE=1 SV=1 |
|  | sp|P52597|HNRPF_HUMAN | Heterogeneous nuclear ribonucleoprotein F OS=Homo sapiens OX=9606 GN=HNRNPF PE=1 SV=3 |
|  | sp|Q02413|DSG1_HUMAN | Desmoglein-1 OS=Homo sapiens OX=9606 GN=DSG1 PE=1 SV=2 |
|  | sp|Q8N1N4|K2C78_HUMAN | Keratin, type II cytoskeletal 78 OS=Homo sapiens OX=9606 GN=KRT78 PE=1 SV=2 |
|  | sp|P08727|K1C19_HUMAN | Keratin, type I cytoskeletal 19 OS=Homo sapiens OX=9606 GN=KRT19 PE=1 SV=4 |
|  | sp|O95678|K2C75_HUMAN | Keratin, type II cytoskeletal 75 OS=Homo sapiens OX=9606 GN=KRT75 PE=1 SV=2 |
|  | sp|Q7Z794|K2C1B_HUMAN | Keratin, type II cytoskeletal 1b OS=Homo sapiens OX=9606 GN=KRT77 PE=2 SV=3 |
|  | sp|P55795|HNRH2_HUMAN | Heterogeneous nuclear ribonucleoprotein H2 OS=Homo sapiens OX=9606 GN=HNRNPH2 PE=1 SV=1 |
|  | sp|Q96I25|SPF45_HUMAN | Splicing factor 45 OS=Homo sapiens OX=9606 GN=RBM17 PE=1 SV=1 |
|  | sp|Q6S8J3|POTEE_HUMAN | POTE ankyrin domain family member E OS=Homo sapiens OX=9606 GN=POTEE PE=2 SV=3 |
|  | sp|P12035|K2C3_HUMAN | Keratin, type II cytoskeletal 3 OS=Homo sapiens OX=9606 GN=KRT3 PE=1 SV=3 |
|  | sp|Q5XKE5|K2C79_HUMAN | Keratin, type II cytoskeletal 79 OS=Homo sapiens OX=9606 GN=KRT79 PE=1 SV=2 |
|  | sp|Q04837|SSBP_HUMAN | Single-stranded DNA-binding protein, mitochondrial OS=Homo sapiens OX=9606 GN=SSBP1 PE=1 SV=1 |
|  | sp|Q01546|K22O_HUMAN | Keratin, type II cytoskeletal 2 oral OS=Homo sapiens OX=9606 GN=KRT76 PE=1 SV=2 |
|  | sp|Q9UHD2|TBK1_HUMAN | Serine/threonine-protein kinase TBK1 OS=Homo sapiens OX=9606 GN=TBK1 PE=1 SV=1 |
|  | sp|Q9BQE3|TBA1C_HUMAN | Tubulin alpha-1C chain OS=Homo sapiens OX=9606 GN=TUBA1C PE=1 SV=1 |
|  | sp|P81605|DCD_HUMAN | Dermcidin OS=Homo sapiens OX=9606 GN=DCD PE=1 SV=2 |
|  | sp|P61978|HNRPK_HUMAN | Heterogeneous nuclear ribonucleoprotein K OS=Homo sapiens OX=9606 GN=HNRNPK PE=1 SV=1 |
|  | sp|Q9H4B7|TBB1_HUMAN | Tubulin beta-1 chain OS=Homo sapiens OX=9606 GN=TUBB1 PE=1 SV=1 |
|  | sp|Q14CN4|K2C72_HUMAN | Keratin, type II cytoskeletal 72 OS=Homo sapiens OX=9606 GN=KRT72 PE=1 SV=2 |
|  | sp|Q7RTS7|K2C74_HUMAN | Keratin, type II cytoskeletal 74 OS=Homo sapiens OX=9606 GN=KRT74 PE=1 SV=2 |
|  | sp|P17661|DESM_HUMAN | Desmin OS=Homo sapiens OX=9606 GN=DES PE=1 SV=3 |
|  | sp|P35637|FUS_HUMAN | RNA-binding protein FUS OS=Homo sapiens OX=9606 GN=FUS PE=1 SV=1 |
|  | sp|Q562R1|ACTBL_HUMAN | Beta-actin-like protein 2 OS=Homo sapiens OX=9606 GN=ACTBL2 PE=1 SV=2 |
|  | sp|O00622|CCN1_HUMAN | CCN family member 1 OS=Homo sapiens OX=9606 GN=CCN1 PE=1 SV=1 |
|  | sp|P35900|K1C20_HUMAN | Keratin, type I cytoskeletal 20 OS=Homo sapiens OX=9606 GN=KRT20 PE=1 SV=1 |
|  | sp|Q07954|LRP1_HUMAN | Prolow-density lipoprotein receptor-related protein 1 OS=Homo sapiens OX=9606 GN=LRP1 PE=1 SV=2 |
|  | sp|Q5T749|KPRP_HUMAN | Keratinocyte proline-rich protein OS=Homo sapiens OX=9606 GN=KPRP PE=1 SV=1 |
|  | sp|P15924|DESP_HUMAN | Desmoplakin OS=Homo sapiens OX=9606 GN=DSP PE=1 SV=3 |
|  | sp|P19013|K2C4_HUMAN | Keratin, type II cytoskeletal 4 OS=Homo sapiens OX=9606 GN=KRT4 PE=1 SV=4 |
|  | sp|P34931|HS71L_HUMAN | Heat shock 70 kDa protein 1-like OS=Homo sapiens OX=9606 GN=HSPA1L PE=1 SV=2 |
|  | sp|P07355|ANXA2_HUMAN | Annexin A2 OS=Homo sapiens OX=9606 GN=ANXA2 PE=1 SV=2 |
|  | sp|Q9NSB2|KRT84_HUMAN | Keratin, type II cuticular Hb4 OS=Homo sapiens OX=9606 GN=KRT84 PE=2 SV=2 |
|  | sp|P04406|G3P_HUMAN | Glyceraldehyde-3-phosphate dehydrogenase OS=Homo sapiens OX=9606 GN=GAPDH PE=1 SV=3 |
|  | sp|P11142|HSP7C_HUMAN | Heat shock cognate 71 kDa protein OS=Homo sapiens OX=9606 GN=HSPA8 PE=1 SV=1 |
|  | sp|P17066|HSP76_HUMAN | Heat shock 70 kDa protein 6 OS=Homo sapiens OX=9606 GN=HSPA6 PE=1 SV=2 |
|  | sp|P19474|RO52_HUMAN | E3 ubiquitin-protein ligase TRIM21 OS=Homo sapiens OX=9606 GN=TRIM21 PE=1 SV=1 |
|  | sp|Q2M2I5|K1C24_HUMAN | Keratin, type I cytoskeletal 24 OS=Homo sapiens OX=9606 GN=KRT24 PE=1 SV=1 |
|  | sp|P18124|RL7_HUMAN | 60S ribosomal protein L7 OS=Homo sapiens OX=9606 GN=RPL7 PE=1 SV=1 |
|  | sp|P27694|RFA1_HUMAN | Replication protein A 70 kDa DNA-binding subunit OS=Homo sapiens OX=9606 GN=RPA1 PE=1 SV=2 |
|  | sp|P14923|PLAK_HUMAN | Junction plakoglobin OS=Homo sapiens OX=9606 GN=JUP PE=1 SV=3 |
|  | sp|O75955|FLOT1_HUMAN | Flotillin-1 OS=Homo sapiens OX=9606 GN=FLOT1 PE=1 SV=3 |
|  | sp|P05141|ADT2_HUMAN | ADP/ATP translocase 2 OS=Homo sapiens OX=9606 GN=SLC25A5 PE=1 SV=7 |
|  | sp|Q9BQ70|TCF25_HUMAN | Transcription factor 25 OS=Homo sapiens OX=9606 GN=TCF25 PE=1 SV=1 |
|  | sp|P62241|RS8_HUMAN | 40S ribosomal protein S8 OS=Homo sapiens OX=9606 GN=RPS8 PE=1 SV=2 |
|  | sp|Q6PEY2|TBA3E_HUMAN | Tubulin alpha-3E chain OS=Homo sapiens OX=9606 GN=TUBA3E PE=1 SV=2 |
|  | sp|P26373|RL13_HUMAN | 60S ribosomal protein L13 OS=Homo sapiens OX=9606 GN=RPL13 PE=1 SV=4 |
|  | sp|P12236|ADT3_HUMAN | ADP/ATP translocase 3 OS=Homo sapiens OX=9606 GN=SLC25A6 PE=1 SV=4 |
|  | sp|Q08188|TGM3_HUMAN | Protein-glutamine gamma-glutamyltransferase E OS=Homo sapiens OX=9606 GN=TGM3 PE=1 SV=4 |
|  | sp|Q92841|DDX17_HUMAN | Probable ATP-dependent RNA helicase DDX17 OS=Homo sapiens OX=9606 GN=DDX17 PE=1 SV=2 |
|  | sp|Q7Z3Y9|K1C26_HUMAN | Keratin, type I cytoskeletal 26 OS=Homo sapiens OX=9606 GN=KRT26 PE=1 SV=2 |
|  | sp|P35030|TRY3_HUMAN | Trypsin-3 OS=Homo sapiens OX=9606 GN=PRSS3 PE=1 SV=2 |
|  | sp|P0DOX5|IGG1_HUMAN | Immunoglobulin gamma-1 heavy chain OS=Homo sapiens OX=9606 PE=1 SV=2 |
|  | sp|Q5VTE0|EF1A3_HUMAN | Putative elongation factor 1-alpha-like 3 OS=Homo sapiens OX=9606 GN=EEF1A1P5 PE=5 SV=1 |
|  | sp|Q07020|RL18_HUMAN | 60S ribosomal protein L18 OS=Homo sapiens OX=9606 GN=RPL18 PE=1 SV=2 |
|  | sp|P83731|RL24_HUMAN | 60S ribosomal protein L24 OS=Homo sapiens OX=9606 GN=RPL24 PE=1 SV=1 |
|  | sp|P62805|H4_HUMAN | Histone H4 OS=Homo sapiens OX=9606 GN=HIST1H4A PE=1 SV=2 |
|  | sp|P05109|S10A8_HUMAN | Protein S100-A8 OS=Homo sapiens OX=9606 GN=S100A8 PE=1 SV=1 |
|  | sp|Q9Y5X1|SNX9_HUMAN | Sorting nexin-9 OS=Homo sapiens OX=9606 GN=SNX9 PE=1 SV=1 |
|  | sp|P31942|HNRH3_HUMAN | Heterogeneous nuclear ribonucleoprotein H3 OS=Homo sapiens OX=9606 GN=HNRNPH3 PE=1 SV=2 |
|  | sp|P61313|RL15_HUMAN | 60S ribosomal protein L15 OS=Homo sapiens OX=9606 GN=RPL15 PE=1 SV=2 |
|  | sp|P00738|HPT_HUMAN | Haptoglobin OS=Homo sapiens OX=9606 GN=HP PE=1 SV=1 |
|  | sp|Q00325|MPCP_HUMAN | Phosphate carrier protein, mitochondrial OS=Homo sapiens OX=9606 GN=SLC25A3 PE=1 SV=2 |
|  | sp|Q5D862|FILA2_HUMAN | Filaggrin-2 OS=Homo sapiens OX=9606 GN=FLG2 PE=1 SV=1 |
|  | sp|P62917|RL8_HUMAN | 60S ribosomal protein L8 OS=Homo sapiens OX=9606 GN=RPL8 PE=1 SV=2 |
|  | sp|P62753|RS6_HUMAN | 40S ribosomal protein S6 OS=Homo sapiens OX=9606 GN=RPS6 PE=1 SV=1 |
|  | sp|O75525|KHDR3_HUMAN | KH domain-containing, RNA-binding, signal transduction-associated protein 3 OS=Homo sapiens OX=9606 GN=KHDRBS3 PE=1 SV=1 |
|  | sp|Q6KB66|K2C80_HUMAN | Keratin, type II cytoskeletal 80 OS=Homo sapiens OX=9606 GN=KRT80 PE=1 SV=2 |
|  | sp|O76013|KRT36_HUMAN | Keratin, type I cuticular Ha6 OS=Homo sapiens OX=9606 GN=KRT36 PE=2 SV=1 |
|  | sp|Q92764|KRT35_HUMAN | Keratin, type I cuticular Ha5 OS=Homo sapiens OX=9606 GN=KRT35 PE=2 SV=5 |
|  | sp|Q14525|KT33B_HUMAN | Keratin, type I cuticular Ha3-II OS=Homo sapiens OX=9606 GN=KRT33B PE=1 SV=3 |
|  | sp|P47914|RL29_HUMAN | 60S ribosomal protein L29 OS=Homo sapiens OX=9606 GN=RPL29 PE=1 SV=2 |
|  | sp|Q8TF72|SHRM3_HUMAN | Protein Shroom3 OS=Homo sapiens OX=9606 GN=SHROOM3 PE=1 SV=2 |
|  | sp|Q8NHM4|TRY6_HUMAN | Putative trypsin-6 OS=Homo sapiens OX=9606 GN=PRSS3P2 PE=5 SV=2 |
|  | sp|O00712|NFIB_HUMAN | Nuclear factor 1 B-type OS=Homo sapiens OX=9606 GN=NFIB PE=1 SV=2 |
|  | sp|Q9NQI0|DDX4_HUMAN | Probable ATP-dependent RNA helicase DDX4 OS=Homo sapiens OX=9606 GN=DDX4 PE=1 SV=2 |
|  | sp|Q86U44|MTA70_HUMAN | N6-adenosine-methyltransferase catalytic subunit OS=Homo sapiens OX=9606 GN=METTL3 PE=1 SV=2 |
|  | sp|Q96P63|SPB12_HUMAN | Serpin B12 OS=Homo sapiens OX=9606 GN=SERPINB12 PE=1 SV=1 |
|  | sp|P23396|RS3_HUMAN | 40S ribosomal protein S3 OS=Homo sapiens OX=9606 GN=RPS3 PE=1 SV=2 |
|  | sp|P07996|TSP1_HUMAN | Thrombospondin-1 OS=Homo sapiens OX=9606 GN=THBS1 PE=1 SV=2 |
|  | sp|Q96NE9|FRMD6_HUMAN | FERM domain-containing protein 6 OS=Homo sapiens OX=9606 GN=FRMD6 PE=1 SV=1 |
|  | sp|P04003|C4BPA_HUMAN | C4b-binding protein alpha chain OS=Homo sapiens OX=9606 GN=C4BPA PE=1 SV=2 |
|  | sp|Q15517|CDSN_HUMAN | Corneodesmosin OS=Homo sapiens OX=9606 GN=CDSN PE=1 SV=3 |
|  | sp|Q08554|DSC1_HUMAN | Desmocollin-1 OS=Homo sapiens OX=9606 GN=DSC1 PE=1 SV=2 |
|  | sp|Q9BYE4|SPR2G_HUMAN | Small proline-rich protein 2G OS=Homo sapiens OX=9606 GN=SPRR2G PE=3 SV=1 |
|  | sp|Q15654|TRIP6_HUMAN | Thyroid receptor-interacting protein 6 OS=Homo sapiens OX=9606 GN=TRIP6 PE=1 SV=3 |
|  | sp|Q06830|PRDX1_HUMAN | Peroxiredoxin-1 OS=Homo sapiens OX=9606 GN=PRDX1 PE=1 SV=1 |
|  | sp|A0A0C4DH55|KVD07_HUMAN | Immunoglobulin kappa variable 3D-7 OS=Homo sapiens OX=9606 GN=IGKV3D-7 PE=3 SV=5 |
|  | sp|P62979|RS27A_HUMAN | Ubiquitin-40S ribosomal protein S27a OS=Homo sapiens OX=9606 GN=RPS27A PE=1 SV=2 |
|  | sp|P0CG47|UBB_HUMAN | Polyubiquitin-B OS=Homo sapiens OX=9606 GN=UBB PE=1 SV=1 |
|  | sp|Q9NRL3|STRN4_HUMAN | Striatin-4 OS=Homo sapiens OX=9606 GN=STRN4 PE=1 SV=2 |
|  | sp|P0C869|PA24B_HUMAN | Cytosolic phospholipase A2 beta OS=Homo sapiens OX=9606 GN=PLA2G4B PE=1 SV=2 |
|  | sp|P06702|S10A9_HUMAN | Protein S100-A9 OS=Homo sapiens OX=9606 GN=S100A9 PE=1 SV=1 |
|  | sp|Q01844|EWS_HUMAN | RNA-binding protein EWS OS=Homo sapiens OX=9606 GN=EWSR1 PE=1 SV=1 |
|  | sp|P39023|RL3_HUMAN | 60S ribosomal protein L3 OS=Homo sapiens OX=9606 GN=RPL3 PE=1 SV=2 |
|  | sp|P14784|IL2RB_HUMAN | Interleukin-2 receptor subunit beta OS=Homo sapiens OX=9606 GN=IL2RB PE=1 SV=1 |
|  | sp|Q14103|HNRPD_HUMAN | Heterogeneous nuclear ribonucleoprotein D0 OS=Homo sapiens OX=9606 GN=HNRNPD PE=1 SV=1 |
|  | sp|P78559|MAP1A_HUMAN | Microtubule-associated protein 1A OS=Homo sapiens OX=9606 GN=MAP1A PE=1 SV=6 |
|  | sp|P46821|MAP1B_HUMAN | Microtubule-associated protein 1B OS=Homo sapiens OX=9606 GN=MAP1B PE=1 SV=2 |
|  | sp|P12956|XRCC6_HUMAN | X-ray repair cross-complementing protein 6 OS=Homo sapiens OX=9606 GN=XRCC6 PE=1 SV=2 |
|  | sp|Q08380|LG3BP_HUMAN | Galectin-3-binding protein OS=Homo sapiens OX=9606 GN=LGALS3BP PE=1 SV=1 |
|  | sp|Q5T750|XP32_HUMAN | Skin-specific protein 32 OS=Homo sapiens OX=9606 GN=XP32 PE=1 SV=1 |
|  | sp|P07196|NFL_HUMAN | Neurofilament light polypeptide OS=Homo sapiens OX=9606 GN=NEFL PE=1 SV=3 |
|  | sp|P07197|NFM_HUMAN | Neurofilament medium polypeptide OS=Homo sapiens OX=9606 GN=NEFM PE=1 SV=3 |
|  | sp|Q16352|AINX_HUMAN | Alpha-internexin OS=Homo sapiens OX=9606 GN=INA PE=1 SV=2 |
|  | sp|Q13162|PRDX4_HUMAN | Peroxiredoxin-4 OS=Homo sapiens OX=9606 GN=PRDX4 PE=1 SV=1 |
|  | sp|Q00577|PURA_HUMAN | Transcriptional activator protein Pur-alpha OS=Homo sapiens OX=9606 GN=PURA PE=1 SV=2 |
|  | sp|Q6ZR08|DYH12_HUMAN | Dynein heavy chain 12, axonemal OS=Homo sapiens OX=9606 GN=DNAH12 PE=2 SV=2 |
|  | sp|O00338|ST1C2_HUMAN | Sulfotransferase 1C2 OS=Homo sapiens OX=9606 GN=SULT1C2 PE=1 SV=1 |
|  | sp|Q9P0W8|SPAT7_HUMAN | Spermatogenesis-associated protein 7 OS=Homo sapiens OX=9606 GN=SPATA7 PE=1 SV=3 |
|  | sp|P20930|FILA_HUMAN | Filaggrin OS=Homo sapiens OX=9606 GN=FLG PE=1 SV=3 |
|  | sp|Q9NW38|FANCL_HUMAN | E3 ubiquitin-protein ligase FANCL OS=Homo sapiens OX=9606 GN=FANCL PE=1 SV=2 |
|  | sp|P68871|HBB_HUMAN | Hemoglobin subunit beta OS=Homo sapiens OX=9606 GN=HBB PE=1 SV=2 |
|  | sp|Q6UWP8|SBSN_HUMAN | Suprabasin OS=Homo sapiens OX=9606 GN=SBSN PE=1 SV=2 |
|  | sp|P50402|EMD_HUMAN | Emerin OS=Homo sapiens OX=9606 GN=EMD PE=1 SV=1 |
|  | sp|P31151|S10A7_HUMAN | Protein S100-A7 OS=Homo sapiens OX=9606 GN=S100A7 PE=1 SV=4 |
|  | sp|P13010|XRCC5_HUMAN | X-ray repair cross-complementing protein 5 OS=Homo sapiens OX=9606 GN=XRCC5 PE=1 SV=3 |
|  | sp|Q96Q27|ASB2_HUMAN | Ankyrin repeat and SOCS box protein 2 OS=Homo sapiens OX=9606 GN=ASB2 PE=1 SV=1 |
|  | sp|P52272|HNRPM_HUMAN | Heterogeneous nuclear ribonucleoprotein M OS=Homo sapiens OX=9606 GN=HNRNPM PE=1 SV=3 |
|  | sp|Q12899|TRI26_HUMAN | Tripartite motif-containing protein 26 OS=Homo sapiens OX=9606 GN=TRIM26 PE=1 SV=1 |
|  | sp|Q7Z6J0|SH3R1_HUMAN | E3 ubiquitin-protein ligase SH3RF1 OS=Homo sapiens OX=9606 GN=SH3RF1 PE=1 SV=2 |
|  | sp|Q9NYY1|IL20_HUMAN | Interleukin-20 OS=Homo sapiens OX=9606 GN=IL20 PE=1 SV=2 |
|  | sp|Q6PJT7|ZC3HE_HUMAN | Zinc finger CCCH domain-containing protein 14 OS=Homo sapiens OX=9606 GN=ZC3H14 PE=1 SV=1 |
|  | sp|P0DOX8|IGL1_HUMAN | Immunoglobulin lambda-1 light chain OS=Homo sapiens OX=9606 PE=1 SV=1 |
|  | sp|Q96IF1|AJUBA_HUMAN | LIM domain-containing protein ajuba OS=Homo sapiens OX=9606 GN=AJUBA PE=1 SV=1 |
|  | sp|Q03001|DYST_HUMAN | Dystonin OS=Homo sapiens OX=9606 GN=DST PE=1 SV=4 |
|  | sp|O75628|REM1_HUMAN | GTP-binding protein REM 1 OS=Homo sapiens OX=9606 GN=REM1 PE=1 SV=2 |
|  | sp|Q9UJ98|STAG3_HUMAN | Cohesin subunit SA-3 OS=Homo sapiens OX=9606 GN=STAG3 PE=1 SV=2 |
|  | sp|Q96JQ0|PCD16_HUMAN | Protocadherin-16 OS=Homo sapiens OX=9606 GN=DCHS1 PE=1 SV=1 |
|  | sp|P82279|CRUM1_HUMAN | Protein crumbs homolog 1 OS=Homo sapiens OX=9606 GN=CRB1 PE=1 SV=2 |
|  | sp|P04792|HSPB1_HUMAN | Heat shock protein beta-1 OS=Homo sapiens OX=9606 GN=HSPB1 PE=1 SV=2 |
|  | sp|Q9HCF6|TRPM3_HUMAN | Transient receptor potential cation channel subfamily M member 3 OS=Homo sapiens OX=9606 GN=TRPM3 PE=2 SV=4 |
|  | sp|Q9Y6V0|PCLO_HUMAN | Protein piccolo OS=Homo sapiens OX=9606 GN=PCLO PE=1 SV=5 |
|  | sp|Q86UW8|HPLN4_HUMAN | Hyaluronan and proteoglycan link protein 4 OS=Homo sapiens OX=9606 GN=HAPLN4 PE=2 SV=1 |
|  | sp|Q96BA8|CR3L1_HUMAN | Cyclic AMP-responsive element-binding protein 3-like protein 1 OS=Homo sapiens OX=9606 GN=CREB3L1 PE=1 SV=1 |
|  | sp|Q9UNX3|RL26L_HUMAN | 60S ribosomal protein L26-like 1 OS=Homo sapiens OX=9606 GN=RPL26L1 PE=1 SV=1 |
|  | sp|Q8WV41|SNX33_HUMAN | Sorting nexin-33 OS=Homo sapiens OX=9606 GN=SNX33 PE=1 SV=1 |
|  | sp|O60573|IF4E2_HUMAN | Eukaryotic translation initiation factor 4E type 2 OS=Homo sapiens OX=9606 GN=EIF4E2 PE=1 SV=1 |
|  | sp|O43663|PRC1_HUMAN | Protein regulator of cytokinesis 1 OS=Homo sapiens OX=9606 GN=PRC1 PE=1 SV=2 |
|  | sp|Q96PH1|NOX5_HUMAN | NADPH oxidase 5 OS=Homo sapiens OX=9606 GN=NOX5 PE=1 SV=1 |
|  | sp|A8MX80|YM017_HUMAN | Putative UPF0607 protein ENSP00000383144 OS=Homo sapiens OX=9606 PE=3 SV=2 |
|  | sp|Q5SNV9|CA167_HUMAN | Uncharacterized protein C1orf167 OS=Homo sapiens OX=9606 GN=C1orf167 PE=2 SV=2 |
|  | sp|Q86UK0|ABCAC_HUMAN | ATP-binding cassette sub-family A member 12 OS=Homo sapiens OX=9606 GN=ABCA12 PE=1 SV=3 |
|  | sp|Q8N257|H2B3B_HUMAN | Histone H2B type 3-B OS=Homo sapiens OX=9606 GN=HIST3H2BB PE=1 SV=3 |
|  | sp|Q14393|GAS6_HUMAN | Growth arrest-specific protein 6 OS=Homo sapiens OX=9606 GN=GAS6 PE=1 SV=3 |
|  | sp|O60437|PEPL_HUMAN | Periplakin OS=Homo sapiens OX=9606 GN=PPL PE=1 SV=4 |
|  | sp|Q9UM47|NOTC3_HUMAN | Neurogenic locus notch homolog protein 3 OS=Homo sapiens OX=9606 GN=NOTCH3 PE=1 SV=2 |
|  | sp|O60858|TRI13_HUMAN | E3 ubiquitin-protein ligase TRIM13 OS=Homo sapiens OX=9606 GN=TRIM13 PE=1 SV=2 |
|  | sp|Q5VV41|ARHGG_HUMAN | Rho guanine nucleotide exchange factor 16 OS=Homo sapiens OX=9606 GN=ARHGEF16 PE=1 SV=1 |
|  | sp|Q9UFH2|DYH17_HUMAN | Dynein heavy chain 17, axonemal OS=Homo sapiens OX=9606 GN=DNAH17 PE=1 SV=3 |
|  | sp|Q99536|VAT1_HUMAN | Synaptic vesicle membrane protein VAT-1 homolog OS=Homo sapiens OX=9606 GN=VAT1 PE=1 SV=2 |
|  | sp|Q96QD5|DEPD7_HUMAN | DEP domain-containing protein 7 OS=Homo sapiens OX=9606 GN=DEPDC7 PE=2 SV=1 |
|  | sp|E7EW31|PROB1_HUMAN | Proline-rich basic protein 1 OS=Homo sapiens OX=9606 GN=PROB1 PE=2 SV=2 |
|  | sp|Q96FV9|THOC1_HUMAN | THO complex subunit 1 OS=Homo sapiens OX=9606 GN=THOC1 PE=1 SV=1 |
|  | sp|Q5SYB0|FRPD1_HUMAN | FERM and PDZ domain-containing protein 1 OS=Homo sapiens OX=9606 GN=FRMPD1 PE=1 SV=1 |
```

|  | SP\_human0806 | Decoy | False discovery rate |
| --- | --- | --- | --- |
| Peptide matches above identity threshold | 691 | 32 | 4.63 % |
| Peptide matches above homology or identity threshold | 740 | 46 | 6.22 % |

### Mascot Score Histogram

Ions score is -10\*Log(P), where P is the probability that the observed match is a random event.  
Individual ions scores > 22 indicate identity or extensive homology (p<0.05).  
Protein scores are derived from ions scores as a non-probabilistic basis for ranking protein hits.


### Peptide Summary Report

|  |  |  |  |
| --- | --- | --- | --- |
|  | Protein Family Summary Peptide Summary Select Summary (protein hits) Select Summary (unassigned) Export Search Results |  | Help |
|  | Significance threshold p< | Max. number of hits | Show Percolator scores |
|  | Standard scoring  MudPIT scoring | Ions score or expect cut-off | Show sub-sets |
|  | Show pop-ups  Suppress pop-ups | Sort unassigned  Decreasing Score Increasing query / Mr Decreasing Intensity | Require bold red |


  
  
  **Error tolerant**   

|  |  |
| --- | --- |
| **1.** | sp|P04264|K2C1\_HUMAN    **Mass:** 66170    **Score:** 2292   **Matches:** 70(62)  **Sequences:** 26(25)  **emPAI:** 4.73 |
|  | Keratin, type II cytoskeletal 1 OS=Homo sapiens OX=9606 GN=KRT1 PE=1 SV=6 |

|  |  |
| --- | --- |
|  | Check to include this hit in error tolerant search or archive report |
|  |  |

|  |  |  |  |  |  |  |  |  |  |  |  |
| --- | --- | --- | --- | --- | --- | --- | --- | --- | --- | --- | --- |
|  | **Query** | **Observed** | **Mr(expt)** | **Mr(calc)** | **ppm** | **Miss** | **Score** | **Expect** | **Rank** | **Unique** | **Peptide** |
|  | 256 | **416.7485** | **831.4824** | **831.4814** | **1.19** | **0** | **(20)** | **0.15** | **1** | **U** | **K.SISISVAR.G** |
|  | 257 | **416.7499** | **831.4853** | **831.4814** | **4.65** | **0** | **(20)** | **0.16** | **1** | **U** | **K.SISISVAR.G** |
|  | 258 | **416.7507** | **831.4869** | **831.4814** | **6.62** | **0** | **34** | **0.0044** | **1** | **U** | **K.SISISVAR.G** |
|  | 469 | **437.7532** | **873.4918** | **873.4920** | **-0.21** | **0** | **38** | **0.0033** | **1** | **U** | **R.SLVNLGGSK.S** |
|  | 791 | **487.2693** | **972.5241** | **972.5240** | **0.18** | **0** | **(46)** | **0.00058** | **1** |  | **K.IEISELNR.V** |
|  | 792 | **487.2693** | **972.5241** | **972.5240** | **0.18** | **0** | **(51)** | **0.00017** | **1** |  | **K.IEISELNR.V** |
|  | 793 | **487.2695** | **972.5245** | **972.5240** | **0.55** | **0** | **52** | **0.00012** | **1** |  | **K.IEISELNR.V** |
|  | 892 | **500.2261** | **998.4377** | **998.4379** | **-0.17** | **0** | **(21)** | **0.045** | **1** | **U** | **K.DVDGAYMTK.V** |
|  | 894 | **500.2269** | **998.4392** | **998.4379** | **1.29** | **0** | **28** | **0.0088** | **1** | **U** | **K.DVDGAYMTK.V** |
|  | 1024 | **517.2607** | **1032.5069** | **1032.5087** | **-1.74** | **0** | **37** | **0.0038** | **1** | **U** | **R.TLLEGEESR.M** |
|  | 1025 | **517.2617** | **1032.5089** | **1032.5087** | **0.15** | **0** | **(35)** | **0.0058** | **1** | **U** | **R.TLLEGEESR.M** |
|  | 1026 | **517.2624** | **1032.5102** | **1032.5087** | **1.45** | **0** | **(20)** | **0.21** | **1** | **U** | **R.TLLEGEESR.M** |
|  | 1199 | **533.2642** | **1064.5139** | **1064.5138** | **0.07** | **0** | **(33)** | **0.0069** | **1** | **U** | **K.AQYEDIAQK.S** |
|  | 1200 | **533.2662** | **1064.5178** | **1064.5138** | **3.74** | **0** | **34** | **0.0065** | **1** | **U** | **K.AQYEDIAQK.S** |
|  | 1241 | **537.3012** | **1072.5879** | **1072.5876** | **0.21** | **1** | **28** | **0.031** | **1** | **U** | **R.LRSEIDNVK.K** |
|  | 1567 | **571.2630** | **1140.5114** | **1140.5121** | **-0.57** | **0** | **(35)** | **0.0025** | **1** | **U** | **R.DYQELMNTK.L** |
|  | 1568 | **571.2636** | **1140.5127** | **1140.5121** | **0.50** | **0** | **42** | **0.00053** | **1** | **U** | **R.DYQELMNTK.L** |
|  | 1569 | **571.2639** | **1140.5131** | **1140.5121** | **0.92** | **0** | **(28)** | **0.014** | **1** | **U** | **R.DYQELMNTK.L** |
|  | 1630 | **579.2592** | **1156.5039** | **1156.5070** | **-2.71** | **0** | **(37)** | **0.0013** | **1** | **U** | **R.DYQELMNTK.L + Oxidation (M)** |
|  | 1631 | **579.2598** | **1156.5050** | **1156.5070** | **-1.76** | **0** | **(23)** | **0.029** | **1** | **U** | **R.DYQELMNTK.L + Oxidation (M)** |
|  | 1734 | **590.3039** | **1178.5932** | **1178.5931** | **0.09** | **0** | **(53)** | **9.2e-005** | **1** |  | **K.YEELQITAGR.H** |
|  | 1735 | **590.3040** | **1178.5934** | **1178.5931** | **0.21** | **0** | **61** | **1.2e-005** | **1** |  | **K.YEELQITAGR.H** |
|  | 2110 | **633.3218** | **1264.6290** | **1264.6299** | **-0.72** | **0** | **81** | **1.5e-007** | **1** | **U** | **R.TNAENEFVTIK.K** |
|  | 2111 | **633.3225** | **1264.6305** | **1264.6299** | **0.44** | **0** | **(57)** | **3.7e-005** | **1** | **U** | **R.TNAENEFVTIK.K** |
|  | 2112 | **633.3227** | **1264.6308** | **1264.6299** | **0.72** | **0** | **(55)** | **5.3e-005** | **1** | **U** | **R.TNAENEFVTIK.K** |
|  | 2244 | **650.7681** | **1299.5216** | **1299.5224** | **-0.59** | **0** | **(62)** | **1.1e-006** | **1** | **U** | **K.NMQDMVEDYR.N** |
|  | 2245 | **650.7682** | **1299.5218** | **1299.5224** | **-0.40** | **0** | **63** | **8.8e-007** | **1** | **U** | **K.NMQDMVEDYR.N** |
|  | 2257 | **651.8611** | **1301.7076** | **1301.7078** | **-0.17** | **0** | **59** | **2e-005** | **1** | **U** | **R.SLDLDSIIAEVK.A** |
|  | 2326 | **658.7657** | **1315.5168** | **1315.5173** | **-0.34** | **0** | **(55)** | **3.9e-006** | **1** | **U** | **K.NMQDMVEDYR.N + Oxidation (M)** |
|  | 2327 | **658.7662** | **1315.5178** | **1315.5173** | **0.39** | **0** | **(55)** | **4.1e-006** | **1** | **U** | **K.NMQDMVEDYR.N + Oxidation (M)** |
|  | 2328 | **658.7663** | **1315.5180** | **1315.5173** | **0.59** | **0** | **(55)** | **4.4e-006** | **1** | **U** | **K.NMQDMVEDYR.N + Oxidation (M)** |
|  | 2381 | **666.7629** | **1331.5113** | **1331.5122** | **-0.65** | **0** | **(57)** | **2e-006** | **1** | **U** | **K.NMQDMVEDYR.N + 2 Oxidation (M)** |
|  | 2382 | **666.7632** | **1331.5119** | **1331.5122** | **-0.20** | **0** | **(56)** | **2.3e-006** | **1** | **U** | **K.NMQDMVEDYR.N + 2 Oxidation (M)** |
|  | 2411 | **670.8380** | **1339.6615** | **1339.6619** | **-0.33** | **1** | **(57)** | **2.6e-005** | **1** | **U** | **K.SKAEAESLYQSK.Y** |
|  | 2412 | **447.5611** | **1339.6616** | **1339.6619** | **-0.26** | **1** | **(32)** | **0.0092** | **1** | **U** | **K.SKAEAESLYQSK.Y** |
|  | 2413 | **670.8384** | **1339.6622** | **1339.6619** | **0.22** | **1** | **60** | **1.5e-005** | **1** | **U** | **K.SKAEAESLYQSK.Y** |
|  | 2474 | **679.3513** | **1356.6881** | **1356.6885** | **-0.29** | **0** | **(80)** | **1.6e-007** | **1** | **U** | **K.LNDLEDALQQAK.E** |
|  | 2475 | **679.3515** | **1356.6884** | **1356.6885** | **-0.02** | **0** | **85** | **4.7e-008** | **1** | **U** | **K.LNDLEDALQQAK.E** |
|  | 2583 | **692.3456** | **1382.6767** | **1382.6830** | **-4.55** | **0** | **(87)** | **2.9e-008** | **1** | **U** | **K.SLNNQFASFIDK.V** |
|  | 2584 | **692.3477** | **1382.6809** | **1382.6830** | **-1.54** | **0** | **(40)** | **0.0016** | **1** | **U** | **K.SLNNQFASFIDK.V** |
|  | 2585 | **692.3482** | **1382.6819** | **1382.6830** | **-0.83** | **0** | **(15)** | **0.49** | **1** | **U** | **K.SLNNQFASFIDK.V** |
|  | 2586 | **692.3483** | **1382.6821** | **1382.6830** | **-0.66** | **0** | **95** | **5.3e-009** | **1** | **U** | **K.SLNNQFASFIDK.V** |
|  | 2643 | **465.2468** | **1392.7186** | **1392.7249** | **-4.50** | **1** | **(11)** | **1.3** | **1** | **U** | **R.TNAENEFVTIKK.D** |
|  | 2644 | **697.3696** | **1392.7247** | **1392.7249** | **-0.12** | **1** | **88** | **2.8e-008** | **1** | **U** | **R.TNAENEFVTIKK.D** |
|  | 2645 | **697.3697** | **1392.7248** | **1392.7249** | **-0.03** | **1** | **(78)** | **2.4e-007** | **1** | **U** | **R.TNAENEFVTIKK.D** |
|  | 2646 | **465.2490** | **1392.7252** | **1392.7249** | **0.26** | **1** | **(54)** | **6.1e-005** | **1** | **U** | **R.TNAENEFVTIKK.D** |
|  | 2648 | **465.2491** | **1392.7253** | **1392.7249** | **0.33** | **1** | **(54)** | **5.6e-005** | **1** | **U** | **R.TNAENEFVTIKK.D** |
|  | 2954 | **738.3951** | **1474.7757** | **1474.7780** | **-1.53** | **0** | **(11)** | **1.1** | **1** |  | **R.FLEQQNQVLQTK.W** |
|  | 2955 | **738.3966** | **1474.7785** | **1474.7780** | **0.38** | **0** | **74** | **6.1e-007** | **1** |  | **R.FLEQQNQVLQTK.W** |
|  | 2956 | **492.6006** | **1474.7799** | **1474.7780** | **1.27** | **0** | **(48)** | **0.00024** | **1** |  | **R.FLEQQNQVLQTK.W** |
|  | 2957 | **738.3979** | **1474.7812** | **1474.7780** | **2.20** | **0** | **(67)** | **3.3e-006** | **1** |  | **R.FLEQQNQVLQTK.W** |
|  | 3087 | **508.6012** | **1522.7818** | **1522.7813** | **0.29** | **1** | **30** | **0.014** | **1** | **U** | **R.LLRDYQELMNTK.L** |
|  | 3088 | **762.3982** | **1522.7818** | **1522.7813** | **0.33** | **1** | **(29)** | **0.017** | **1** | **U** | **R.LLRDYQELMNTK.L** |
|  | 3135 | **513.9318** | **1538.7735** | **1538.7762** | **-1.82** | **1** | **(1)** | **9.2** | **1** | **U** | **R.LLRDYQELMNTK.L + Oxidation (M)** |
|  | 3276 | **533.9496** | **1598.8269** | **1598.8264** | **0.35** | **1** | **(47)** | **0.00027** | **1** | **U** | **K.NKLNDLEDALQQAK.E** |
|  | 3277 | **800.4210** | **1598.8275** | **1598.8264** | **0.71** | **1** | **77** | **2.4e-007** | **1** | **U** | **K.NKLNDLEDALQQAK.E** |
|  | 3357 | **819.9340** | **1637.8534** | **1637.8525** | **0.51** | **1** | **21** | **0.092** | **1** | **U** | **K.SLNNQFASFIDKVR.F** |
|  | 3392 | **829.3992** | **1656.7839** | **1656.7856** | **-1.02** | **0** | **(35)** | **0.0035** | **1** | **U** | **R.SGGGFSSGSAGIINYQR.R** |
|  | 3393 | **829.4000** | **1656.7854** | **1656.7856** | **-0.14** | **0** | **105** | **3.8e-010** | **1** | **U** | **R.SGGGFSSGSAGIINYQR.R** |
|  | 3509 | **858.9283** | **1715.8421** | **1715.8438** | **-0.98** | **0** | **(77)** | **3.1e-007** | **1** | **U** | **K.QISNLQQSISDAEQR.G** |
|  | 3510 | **858.9289** | **1715.8432** | **1715.8438** | **-0.34** | **0** | **(82)** | **8.4e-008** | **1** | **U** | **K.QISNLQQSISDAEQR.G** |
|  | 3511 | **858.9294** | **1715.8443** | **1715.8438** | **0.30** | **0** | **85** | **3.9e-008** | **1** | **U** | **K.QISNLQQSISDAEQR.G** |
|  | 3512 | **572.9554** | **1715.8445** | **1715.8438** | **0.40** | **0** | **(65)** | **4.9e-006** | **1** | **U** | **K.QISNLQQSISDAEQR.G** |
|  | 3513 | **858.9296** | **1715.8446** | **1715.8438** | **0.45** | **0** | **(65)** | **4.3e-006** | **1** | **U** | **K.QISNLQQSISDAEQR.G** |
|  | 3824 | **922.9794** | **1843.9443** | **1843.9388** | **3.01** | **1** | **105** | **3.9e-010** | **1** | **U** | **K.KQISNLQQSISDAEQR.G** |
|  | 3825 | **615.6556** | **1843.9449** | **1843.9388** | **3.33** | **1** | **(57)** | **2.6e-005** | **1** | **U** | **K.KQISNLQQSISDAEQR.G** |
|  | 4123 | **1009.4453** | **2016.8759** | **2016.8768** | **-0.44** | **1** | **(40)** | **0.00042** | **1** | **U** | **R.LDSELKNMQDMVEDYR.N + 2 Oxidation (M)** |
|  | 4124 | **673.2995** | **2016.8767** | **2016.8768** | **-0.08** | **1** | **46** | **9.5e-005** | **1** | **U** | **R.LDSELKNMQDMVEDYR.N + 2 Oxidation (M)** |
|  | 4460 | **1192.4807** | **2382.9469** | **2382.9447** | **0.93** | **0** | **135** | **3.5e-014** | **1** | **U** | **R.GGGGGGYGSGGSSYGSGGGSYGSGGGGGGGR.G** |
|  | 4739 | **1104.7766** | **3311.3080** | **3311.3009** | **2.14** | **0** | **115** | **3.3e-012** | **1** | **U** | **R.GSYGSGGSSYGSGGGSYGSGGGGGGHGSYGSGSSSGGYR.G** |

---

|  |  |
| --- | --- |
| **2.** | sp|P67809|YBOX1\_HUMAN    **Mass:** 35903    **Score:** 1357   **Matches:** 59(37)  **Sequences:** 13(12)  **emPAI:** 6.64 |
|  | Nuclease-sensitive element-binding protein 1 OS=Homo sapiens OX=9606 GN=YBX1 PE=1 SV=3 |

|  |  |
| --- | --- |
|  | Check to include this hit in error tolerant search or archive report |
|  |  |

|  |  |  |  |  |  |  |  |  |  |  |  |
| --- | --- | --- | --- | --- | --- | --- | --- | --- | --- | --- | --- |
|  | **Query** | **Observed** | **Mr(expt)** | **Mr(calc)** | **ppm** | **Miss** | **Score** | **Expect** | **Rank** | **Unique** | **Peptide** |
|  | 23 | **361.1922** | **720.3698** | **720.3707** | **-1.33** | **0** | **22** | **0.13** | **1** |  | **K.WFNVR.N** |
|  | 24 | **361.1923** | **720.3700** | **720.3707** | **-0.99** | **0** | **(12)** | **1.4** | **1** |  | **K.WFNVR.N** |
|  | 677 | **470.7357** | **939.4568** | **939.4563** | **0.61** | **0** | **49** | **0.00017** | **1** |  | **R.NGYGFINR.N** |
|  | 782 | **487.2336** | **972.4527** | **972.4527** | **-0.08** | **0** | **(20)** | **0.087** | **2** | **U** | **R.FPPYYMR.R** |
|  | 783 | **487.2336** | **972.4527** | **972.4527** | **-0.02** | **0** | **(13)** | **0.41** | **3** | **U** | **R.FPPYYMR.R** |
|  | 784 | **487.2336** | **972.4527** | **972.4527** | **-0.02** | **0** | **(15)** | **0.29** | **1** | **U** | **R.FPPYYMR.R** |
|  | 785 | **487.2337** | **972.4528** | **972.4527** | **0.11** | **0** | **(28)** | **0.012** | **1** | **U** | **R.FPPYYMR.R** |
|  | 786 | **487.2338** | **972.4530** | **972.4527** | **0.31** | **0** | **35** | **0.0025** | **1** | **U** | **R.FPPYYMR.R** |
|  | 787 | **487.2339** | **972.4532** | **972.4527** | **0.50** | **0** | **(6)** | **2** | **6** | **U** | **R.FPPYYMR.R** |
|  | 788 | **487.2339** | **972.4533** | **972.4527** | **0.56** | **0** | **(15)** | **0.24** | **3** | **U** | **R.FPPYYMR.R** |
|  | 789 | **487.2341** | **972.4536** | **972.4527** | **0.87** | **0** | **(8)** | **1.3** | **3** | **U** | **R.FPPYYMR.R** |
|  | 790 | **487.2346** | **972.4547** | **972.4527** | **2.06** | **0** | **(8)** | **1.5** | **5** | **U** | **R.FPPYYMR.R** |
|  | 843 | **495.2295** | **988.4444** | **988.4477** | **-3.27** | **0** | **(19)** | **0.087** | **1** | **U** | **R.FPPYYMR.R + Oxidation (M)** |
|  | 844 | **495.2303** | **988.4461** | **988.4477** | **-1.59** | **0** | **(5)** | **2.4** | **3** | **U** | **R.FPPYYMR.R + Oxidation (M)** |
|  | 845 | **495.2309** | **988.4473** | **988.4477** | **-0.36** | **0** | **(12)** | **0.51** | **3** | **U** | **R.FPPYYMR.R + Oxidation (M)** |
|  | 846 | **495.2312** | **988.4479** | **988.4477** | **0.25** | **0** | **(13)** | **0.37** | **1** | **U** | **R.FPPYYMR.R + Oxidation (M)** |
|  | 847 | **495.2314** | **988.4483** | **988.4477** | **0.61** | **0** | **(23)** | **0.034** | **1** | **U** | **R.FPPYYMR.R + Oxidation (M)** |
|  | 848 | **495.2319** | **988.4493** | **988.4477** | **1.67** | **0** | **(24)** | **0.03** | **1** | **U** | **R.FPPYYMR.R + Oxidation (M)** |
|  | 1501 | **377.1917** | **1128.5532** | **1128.5538** | **-0.56** | **1** | **28** | **0.016** | **1** | **U** | **R.RFPPYYMR.R** |
|  | 1502 | **565.2842** | **1128.5539** | **1128.5538** | **0.07** | **1** | **(16)** | **0.26** | **1** | **U** | **R.RFPPYYMR.R** |
|  | 1503 | **565.2842** | **1128.5539** | **1128.5538** | **0.07** | **1** | **(12)** | **0.71** | **1** | **U** | **R.RFPPYYMR.R** |
|  | 1504 | **565.2843** | **1128.5540** | **1128.5538** | **0.18** | **1** | **(24)** | **0.041** | **1** | **U** | **R.RFPPYYMR.R** |
|  | 1505 | **565.2843** | **1128.5540** | **1128.5538** | **0.18** | **1** | **(16)** | **0.3** | **1** | **U** | **R.RFPPYYMR.R** |
|  | 1589 | **382.5232** | **1144.5478** | **1144.5488** | **-0.82** | **1** | **(28)** | **0.019** | **1** | **U** | **R.RFPPYYMR.R + Oxidation (M)** |
|  | 1590 | **573.2820** | **1144.5495** | **1144.5488** | **0.66** | **1** | **(15)** | **0.4** | **1** | **U** | **R.RFPPYYMR.R + Oxidation (M)** |
|  | 1591 | **573.2821** | **1144.5496** | **1144.5488** | **0.77** | **1** | **(17)** | **0.26** | **1** | **U** | **R.RFPPYYMR.R + Oxidation (M)** |
|  | 2195 | **429.5627** | **1285.6664** | **1285.6667** | **-0.21** | **0** | **(22)** | **0.12** | **1** |  | **K.EDVFVHQTAIK.K** |
|  | 2196 | **643.8409** | **1285.6673** | **1285.6667** | **0.52** | **0** | **34** | **0.0068** | **1** |  | **K.EDVFVHQTAIK.K** |
|  | 2722 | **707.8874** | **1413.7602** | **1413.7616** | **-0.98** | **1** | **76** | **3.3e-007** | **1** |  | **K.EDVFVHQTAIKK.N** |
|  | 2723 | **472.2618** | **1413.7634** | **1413.7616** | **1.28** | **1** | **(26)** | **0.036** | **1** |  | **K.EDVFVHQTAIKK.N** |
|  | 3466 | **848.4350** | **1694.8554** | **1694.8588** | **-1.97** | **0** | **88** | **2.1e-008** | **1** | **U** | **K.GAEAANVTGPGGVPVQGSK.Y** |
|  | 3467 | **848.4357** | **1694.8569** | **1694.8588** | **-1.10** | **0** | **(83)** | **6.6e-008** | **1** | **U** | **K.GAEAANVTGPGGVPVQGSK.Y** |
|  | 3468 | **565.9598** | **1694.8575** | **1694.8588** | **-0.75** | **0** | **(49)** | **0.0002** | **1** | **U** | **K.GAEAANVTGPGGVPVQGSK.Y** |
|  | 3469 | **565.9600** | **1694.8582** | **1694.8588** | **-0.32** | **0** | **(48)** | **0.00021** | **1** | **U** | **K.GAEAANVTGPGGVPVQGSK.Y** |
|  | 3470 | **848.4371** | **1694.8596** | **1694.8588** | **0.48** | **0** | **(77)** | **2.8e-007** | **1** | **U** | **K.GAEAANVTGPGGVPVQGSK.Y** |
|  | 3574 | **582.3003** | **1743.8792** | **1743.8792** | **0.03** | **1** | **(72)** | **7.6e-007** | **1** |  | **R.NDTKEDVFVHQTAIK.K** |
|  | 3575 | **872.9469** | **1743.8792** | **1743.8792** | **0.05** | **1** | **94** | **4.9e-009** | **1** |  | **R.NDTKEDVFVHQTAIK.K** |
|  | 3576 | **872.9469** | **1743.8792** | **1743.8792** | **0.05** | **1** | **(94)** | **5e-009** | **1** |  | **R.NDTKEDVFVHQTAIK.K** |
|  | 3577 | **436.9772** | **1743.8796** | **1743.8792** | **0.24** | **1** | **(40)** | **0.0015** | **1** |  | **R.NDTKEDVFVHQTAIK.K** |
|  | 3722 | **898.4153** | **1794.8161** | **1794.8160** | **0.08** | **0** | **39** | **0.0011** | **1** |  | **R.SVGDGETVEFDVVEGEK.G** |
|  | 3930 | **949.4058** | **1896.7970** | **1896.7973** | **-0.18** | **0** | **65** | **9.3e-007** | **1** | **U** | **K.AADPPAENSSAPEAEQGGAE.-** |
|  | 3931 | **949.4064** | **1896.7983** | **1896.7973** | **0.53** | **0** | **(42)** | **0.00019** | **1** | **U** | **K.AADPPAENSSAPEAEQGGAE.-** |
|  | 3932 | **949.4066** | **1896.7987** | **1896.7973** | **0.73** | **0** | **(51)** | **2.1e-005** | **1** | **U** | **K.AADPPAENSSAPEAEQGGAE.-** |
|  | 4556 | **876.7056** | **2627.0949** | **2627.0968** | **-0.71** | **1** | **(48)** | **2.2e-005** | **1** | **U** | **R.EDGNEEDKENQGDETQGQQPPQR.R** |
|  | 4557 | **876.7059** | **2627.0960** | **2627.0968** | **-0.30** | **1** | **(75)** | **5.3e-008** | **1** | **U** | **R.EDGNEEDKENQGDETQGQQPPQR.R** |
|  | 4558 | **876.7062** | **2627.0967** | **2627.0968** | **-0.02** | **1** | **(54)** | **5.9e-006** | **1** | **U** | **R.EDGNEEDKENQGDETQGQQPPQR.R** |
|  | 4559 | **876.7064** | **2627.0973** | **2627.0968** | **0.19** | **1** | **(7)** | **0.35** | **1** | **U** | **R.EDGNEEDKENQGDETQGQQPPQR.R** |
|  | 4560 | **876.7065** | **2627.0976** | **2627.0968** | **0.33** | **1** | **(42)** | **9.9e-005** | **1** | **U** | **R.EDGNEEDKENQGDETQGQQPPQR.R** |
|  | 4561 | **1314.5569** | **2627.0992** | **2627.0968** | **0.93** | **1** | **(34)** | **0.0007** | **1** | **U** | **R.EDGNEEDKENQGDETQGQQPPQR.R** |
|  | 4562 | **1314.5573** | **2627.0999** | **2627.0968** | **1.22** | **1** | **84** | **7.3e-009** | **1** | **U** | **R.EDGNEEDKENQGDETQGQQPPQR.R** |
|  | 4564 | **876.7075** | **2627.1007** | **2627.0968** | **1.51** | **1** | **(27)** | **0.0035** | **1** | **U** | **R.EDGNEEDKENQGDETQGQQPPQR.R** |
|  | 4705 | **806.6377** | **3222.5217** | **3222.5225** | **-0.24** | **0** | **(59)** | **7.6e-006** | **1** | **U** | **R.RPQYSNPPVQGEVMEGADNQGAGEQGRPVR.Q** |
|  | 4706 | **806.6380** | **3222.5229** | **3222.5225** | **0.13** | **0** | **82** | **3.1e-008** | **1** | **U** | **R.RPQYSNPPVQGEVMEGADNQGAGEQGRPVR.Q** |
|  | 4707 | **1075.1816** | **3222.5231** | **3222.5225** | **0.20** | **0** | **(53)** | **2.6e-005** | **1** | **U** | **R.RPQYSNPPVQGEVMEGADNQGAGEQGRPVR.Q** |
|  | 4709 | **810.6346** | **3238.5092** | **3238.5174** | **-2.52** | **0** | **(78)** | **8e-008** | **1** | **U** | **R.RPQYSNPPVQGEVMEGADNQGAGEQGRPVR.Q + Oxidation (M)** |
|  | 4710 | **1080.5120** | **3238.5141** | **3238.5174** | **-1.03** | **0** | **(16)** | **0.15** | **1** | **U** | **R.RPQYSNPPVQGEVMEGADNQGAGEQGRPVR.Q + Oxidation (M)** |
|  | 4711 | **815.1038** | **3256.3859** | **3256.3889** | **-0.90** | **1** | **(9)** | **0.22** | **1** | **U** | **R.NYQQNYQNSESGEKNEGSESAPEGQAQQR.R** |
|  | 4712 | **1086.4705** | **3256.3896** | **3256.3889** | **0.21** | **1** | **109** | **2e-011** | **1** | **U** | **R.NYQQNYQNSESGEKNEGSESAPEGQAQQR.R** |
|  | 4713 | **1086.4721** | **3256.3943** | **3256.3889** | **1.67** | **1** | **(96)** | **4.8e-010** | **1** | **U** | **R.NYQQNYQNSESGEKNEGSESAPEGQAQQR.R** |

  


---

|  |  |
| --- | --- |
| **3.** | sp|P13645|K1C10\_HUMAN    **Mass:** 59020    **Score:** 1330   **Matches:** 59(51)  **Sequences:** 24(23)  **emPAI:** 4.09 |
|  | Keratin, type I cytoskeletal 10 OS=Homo sapiens OX=9606 GN=KRT10 PE=1 SV=6 |

|  |  |
| --- | --- |
|  | Check to include this hit in error tolerant search or archive report |
|  |  |

|  |  |  |  |  |  |  |  |  |  |  |  |
| --- | --- | --- | --- | --- | --- | --- | --- | --- | --- | --- | --- |
|  | **Query** | **Observed** | **Mr(expt)** | **Mr(calc)** | **ppm** | **Miss** | **Score** | **Expect** | **Rank** | **Unique** | **Peptide** |
|  | 179 | **404.2029** | **806.3913** | **806.3923** | **-1.23** | **0** | **41** | **0.0016** | **1** |  | **R.LAADDFR.L** |
|  | 180 | **404.2037** | **806.3928** | **806.3923** | **0.73** | **0** | **(26)** | **0.044** | **1** |  | **R.LAADDFR.L** |
|  | 182 | **405.2236** | **808.4326** | **808.4330** | **-0.49** | **0** | **(30)** | **0.013** | **1** |  | **R.LASYLDK.V** |
|  | 183 | **405.2238** | **808.4329** | **808.4330** | **-0.12** | **0** | **40** | **0.0016** | **1** |  | **R.LASYLDK.V** |
|  | 362 | **424.2290** | **846.4434** | **846.4447** | **-1.45** | **0** | **(1)** | **20** | **5** | **U** | **K.SEITELR.R** |
|  | 363 | **424.2291** | **846.4436** | **846.4447** | **-1.21** | **0** | **28** | **0.035** | **1** | **U** | **K.SEITELR.R** |
|  | 866 | **497.2538** | **992.4930** | **992.4927** | **0.33** | **0** | **51** | **0.00013** | **1** | **U** | **K.YENEVALR.Q** |
|  | 867 | **497.2541** | **992.4936** | **992.4927** | **0.96** | **0** | **(44)** | **0.00078** | **1** | **U** | **K.YENEVALR.Q** |
|  | 875 | **498.2632** | **994.5119** | **994.5123** | **-0.48** | **1** | **(23)** | **0.081** | **1** | **U** | **K.IKEWYEK.H** |
|  | 876 | **498.2633** | **994.5121** | **994.5123** | **-0.28** | **1** | **40** | **0.0014** | **1** | **U** | **K.IKEWYEK.H** |
|  | 904 | **502.2800** | **1002.5454** | **1002.5458** | **-0.33** | **1** | **(19)** | **0.25** | **1** | **U** | **K.SEITELRR.N** |
|  | 905 | **502.2804** | **1002.5462** | **1002.5458** | **0.47** | **1** | **21** | **0.16** | **1** | **U** | **K.SEITELRR.N** |
|  | 1014 | **516.3004** | **1030.5863** | **1030.5910** | **-4.59** | **0** | **49** | **0.00021** | **1** | **U** | **R.VLDELTLTK.A** |
|  | 1015 | **516.3027** | **1030.5908** | **1030.5910** | **-0.22** | **0** | **(8)** | **1.6** | **2** | **U** | **R.VLDELTLTK.A** |
|  | 1017 | **516.3028** | **1030.5910** | **1030.5910** | **0.03** | **0** | **(33)** | **0.006** | **1** | **U** | **R.VLDELTLTK.A** |
|  | 1197 | **355.5417** | **1063.6032** | **1063.6026** | **0.63** | **1** | **(41)** | **0.00067** | **1** |  | **R.LASYLDKVR.A** |
|  | 1198 | **532.8090** | **1063.6034** | **1063.6026** | **0.75** | **1** | **47** | **0.00017** | **1** |  | **R.LASYLDKVR.A** |
|  | 1312 | **545.7676** | **1089.5207** | **1089.5237** | **-2.71** | **0** | **55** | **4.7e-005** | **1** |  | **K.VTMQNLNDR.L** |
|  | 1313 | **545.7702** | **1089.5258** | **1089.5237** | **1.99** | **0** | **(31)** | **0.013** | **1** |  | **K.VTMQNLNDR.L** |
|  | 1402 | **555.2486** | **1108.4826** | **1108.4825** | **0.12** | **0** | **(44)** | **0.00024** | **1** | **U** | **K.DAEAWFNEK.S** |
|  | 1403 | **555.2488** | **1108.4830** | **1108.4825** | **0.45** | **0** | **48** | **0.00012** | **1** | **U** | **K.DAEAWFNEK.S** |
|  | 1675 | **583.2955** | **1164.5765** | **1164.5775** | **-0.83** | **0** | **60** | **1.7e-005** | **1** | **U** | **R.LENEIQTYR.S** |
|  | 1676 | **583.2958** | **1164.5770** | **1164.5775** | **-0.40** | **0** | **(56)** | **3.7e-005** | **1** | **U** | **R.LENEIQTYR.S** |
|  | 1780 | **396.5712** | **1186.6919** | **1186.6921** | **-0.22** | **1** | **36** | **0.002** | **1** | **U** | **R.RVLDELTLTK.A** |
|  | 1781 | **594.3533** | **1186.6920** | **1186.6921** | **-0.11** | **1** | **(34)** | **0.003** | **1** | **U** | **R.RVLDELTLTK.A** |
|  | 1849 | **601.3110** | **1200.6075** | **1200.6098** | **-1.93** | **0** | **(4)** | **5.7** | **5** |  | **R.QSVEADINGLR.R** |
|  | 1850 | **601.3121** | **1200.6096** | **1200.6098** | **-0.22** | **0** | **58** | **2.6e-005** | **1** |  | **R.QSVEADINGLR.R** |
|  | 1986 | **617.8428** | **1233.6711** | **1233.6717** | **-0.47** | **1** | **47** | **0.00023** | **1** | **U** | **R.LKYENEVALR.Q** |
|  | 1987 | **617.8429** | **1233.6712** | **1233.6717** | **-0.36** | **1** | **(37)** | **0.002** | **1** | **U** | **R.LKYENEVALR.Q** |
|  | 1988 | **412.2313** | **1233.6720** | **1233.6717** | **0.21** | **1** | **(37)** | **0.0021** | **1** | **U** | **R.LKYENEVALR.Q** |
|  | 1998 | **619.2956** | **1236.5766** | **1236.5775** | **-0.68** | **1** | **52** | **6.9e-005** | **1** | **U** | **R.KDAEAWFNEK.S** |
|  | 1999 | **413.1997** | **1236.5773** | **1236.5775** | **-0.14** | **1** | **(38)** | **0.0016** | **1** | **U** | **R.KDAEAWFNEK.S** |
|  | 2101 | **631.8013** | **1261.5881** | **1261.5899** | **-1.39** | **0** | **82** | **6.8e-008** | **1** | **U** | **R.SLLEGEGSSGGGGR.G** |
|  | 2102 | **631.8019** | **1261.5893** | **1261.5899** | **-0.42** | **0** | **(74)** | **4.9e-007** | **1** | **U** | **R.SLLEGEGSSGGGGR.G** |
|  | 2248 | **434.2031** | **1299.5874** | **1299.5877** | **-0.20** | **1** | **25** | **0.019** | **1** | **U** | **K.NHEEEMKDLR.N** |
|  | 2476 | **453.2444** | **1356.7112** | **1356.7110** | **0.20** | **1** | **(7)** | **3.1** | **8** |  | **R.QSVEADINGLRR.V** |
|  | 2477 | **453.2444** | **1356.7113** | **1356.7110** | **0.26** | **1** | **(28)** | **0.027** | **1** |  | **R.QSVEADINGLRR.V** |
|  | 2479 | **679.3632** | **1356.7118** | **1356.7110** | **0.60** | **1** | **(31)** | **0.012** | **1** |  | **R.QSVEADINGLRR.V** |
|  | 2480 | **453.2449** | **1356.7128** | **1356.7110** | **1.35** | **1** | **(35)** | **0.0053** | **1** |  | **R.QSVEADINGLRR.V** |
|  | 2481 | **453.2451** | **1356.7135** | **1356.7110** | **1.90** | **1** | **(9)** | **2.3** | **2** |  | **R.QSVEADINGLRR.V** |
|  | 2482 | **679.3646** | **1356.7147** | **1356.7110** | **2.75** | **1** | **57** | **3.1e-005** | **1** |  | **R.QSVEADINGLRR.V** |
|  | 2521 | **683.3199** | **1364.6252** | **1364.6320** | **-4.98** | **0** | **(52)** | **7.8e-005** | **1** | **U** | **R.SQYEQLAEQNR.K** |
|  | 2522 | **683.3226** | **1364.6306** | **1364.6320** | **-1.05** | **0** | **56** | **2.7e-005** | **1** | **U** | **R.SQYEQLAEQNR.K** |
|  | 2573 | **691.3259** | **1380.6372** | **1380.6408** | **-2.64** | **0** | **(47)** | **0.00024** | **1** | **U** | **R.ALEESNYELEGK.I** |
|  | 2574 | **691.3278** | **1380.6410** | **1380.6408** | **0.09** | **0** | **87** | **2.1e-008** | **1** | **U** | **R.ALEESNYELEGK.I** |
|  | 2575 | **691.3278** | **1380.6410** | **1380.6408** | **0.09** | **0** | **(86)** | **2.5e-008** | **1** | **U** | **R.ALEESNYELEGK.I** |
|  | 2624 | **695.8440** | **1389.6734** | **1389.6736** | **-0.09** | **0** | **(47)** | **0.00027** | **1** | **U** | **K.QSLEASLAETEGR.Y** |
|  | 2625 | **695.8441** | **1389.6736** | **1389.6736** | **0.01** | **0** | **(69)** | **1.9e-006** | **1** | **U** | **K.QSLEASLAETEGR.Y** |
|  | 2626 | **695.8441** | **1389.6737** | **1389.6736** | **0.10** | **0** | **86** | **3.6e-008** | **1** | **U** | **K.QSLEASLAETEGR.Y** |
|  | 2627 | **695.8451** | **1389.6757** | **1389.6736** | **1.58** | **0** | **(25)** | **0.044** | **1** | **U** | **K.QSLEASLAETEGR.Y** |
|  | 2817 | **717.8883** | **1433.7621** | **1433.7626** | **-0.39** | **1** | **50** | **0.00012** | **1** | **U** | **K.IRLENEIQTYR.S** |
|  | 2818 | **717.8887** | **1433.7629** | **1433.7626** | **0.19** | **1** | **(43)** | **0.00056** | **1** | **U** | **K.IRLENEIQTYR.S** |
|  | 2819 | **478.9285** | **1433.7636** | **1433.7626** | **0.66** | **1** | **(35)** | **0.0036** | **1** | **U** | **K.IRLENEIQTYR.S** |
|  | 3011 | **747.3699** | **1492.7253** | **1492.7270** | **-1.12** | **1** | **(32)** | **0.0098** | **1** | **U** | **R.SQYEQLAEQNRK.D** |
|  | 3012 | **747.3703** | **1492.7260** | **1492.7270** | **-0.62** | **1** | **(64)** | **5.9e-006** | **1** | **U** | **R.SQYEQLAEQNRK.D** |
|  | 3013 | **747.3704** | **1492.7263** | **1492.7270** | **-0.46** | **1** | **65** | **5e-006** | **1** | **U** | **R.SQYEQLAEQNRK.D** |
|  | 3014 | **498.5829** | **1492.7268** | **1492.7270** | **-0.09** | **1** | **(31)** | **0.013** | **1** | **U** | **R.SQYEQLAEQNRK.D** |
|  | 3015 | **498.5830** | **1492.7273** | **1492.7270** | **0.21** | **1** | **(35)** | **0.0053** | **1** | **U** | **R.SQYEQLAEQNRK.D** |
|  | 4201 | **1041.9841** | **2081.9537** | **2081.9575** | **-1.83** | **0** | **92** | **6.4e-009** | **1** | **U** | **R.AETECQNTEYQQLLDIK.I** |

  


---

|  |  |
| --- | --- |
| **4.** | sp|P35908|K22E\_HUMAN    **Mass:** 65678    **Score:** 1266   **Matches:** 54(47)  **Sequences:** 25(22)  **emPAI:** 4.00 |
|  | Keratin, type II cytoskeletal 2 epidermal OS=Homo sapiens OX=9606 GN=KRT2 PE=1 SV=2 |

|  |  |
| --- | --- |
|  | Check to include this hit in error tolerant search or archive report |
|  |  |

|  |  |  |  |  |  |  |  |  |  |  |  |
| --- | --- | --- | --- | --- | --- | --- | --- | --- | --- | --- | --- |
|  | **Query** | **Observed** | **Mr(expt)** | **Mr(calc)** | **ppm** | **Miss** | **Score** | **Expect** | **Rank** | **Unique** | **Peptide** |
|  | 236 | **414.2182** | **826.4219** | **826.4225** | **-0.72** | **0** | **40** | **0.0012** | **1** |  | **K.FASFIDK.V** |
|  | 237 | **414.2184** | **826.4223** | **826.4225** | **-0.28** | **0** | **(38)** | **0.0019** | **1** |  | **K.FASFIDK.V** |
|  | 791 | 487.2693 | 972.5241 | 972.5240 | 0.18 | 0 | (46) | 0.00058 | 1 |  | K.IEISELNR.V |
|  | 792 | 487.2693 | 972.5241 | 972.5240 | 0.18 | 0 | (51) | 0.00017 | 1 |  | K.IEISELNR.V |
|  | 793 | 487.2695 | 972.5245 | 972.5240 | 0.55 | 0 | 52 | 0.00012 | 1 |  | K.IEISELNR.V |
|  | 872 | **497.7877** | **993.5608** | **993.5607** | **0.05** | **0** | **6** | **2.8** | **3** | **U** | **R.LQGEIAHVK.K** |
|  | 1040 | **519.2669** | **1036.5193** | **1036.5189** | **0.35** | **0** | **34** | **0.0067** | **1** | **U** | **R.YLDGLTAER.T** |
|  | 1041 | **519.2672** | **1036.5197** | **1036.5189** | **0.81** | **0** | **(15)** | **0.45** | **1** | **U** | **R.YLDGLTAER.T** |
|  | 1221 | **534.7553** | **1067.4959** | **1067.4957** | **0.21** | **0** | **39** | **0.0012** | **1** | **U** | **K.DVDNAYMIK.V** |
|  | 1276 | **361.5378** | **1081.5916** | **1081.5920** | **-0.39** | **1** | **(29)** | **0.014** | **1** |  | **K.FASFIDKVR.F** |
|  | 1277 | **541.8041** | **1081.5937** | **1081.5920** | **1.57** | **1** | **44** | **0.00038** | **1** |  | **K.FASFIDKVR.F** |
|  | 1290 | **542.7524** | **1083.4902** | **1083.4906** | **-0.40** | **0** | **(29)** | **0.012** | **1** | **U** | **K.DVDNAYMIK.V + Oxidation (M)** |
|  | 1396 | **554.2670** | **1106.5194** | **1106.5356** | **-14.65** | **0** | **(3)** | **6.8** | **2** |  | **K.AQYEEIAQR.S** |
|  | 1398 | **554.2739** | **1106.5333** | **1106.5356** | **-2.07** | **0** | **40** | **0.0016** | **1** |  | **K.AQYEEIAQR.S** |
|  | 1515 | **566.2587** | **1130.5028** | **1130.5026** | **0.14** | **0** | **38** | **0.0011** | **1** | **U** | **R.STSSFSCLSR.H** |
|  | 1516 | **566.2589** | **1130.5031** | **1130.5026** | **0.46** | **0** | **(30)** | **0.0057** | **1** | **U** | **R.STSSFSCLSR.H** |
|  | 1555 | **570.2736** | **1138.5327** | **1138.5328** | **-0.13** | **0** | **37** | **0.0026** | **1** |  | **R.DYQELMNVK.L** |
|  | 1625 | **578.2707** | **1154.5268** | **1154.5278** | **-0.80** | **0** | **(23)** | **0.044** | **1** |  | **R.DYQELMNVK.L + Oxidation (M)** |
|  | 1805 | **597.3118** | **1192.6090** | **1192.6088** | **0.16** | **0** | **55** | **5.6e-005** | **1** | **U** | **K.YEELQVTVGR.H** |
|  | 1806 | **597.3118** | **1192.6091** | **1192.6088** | **0.26** | **0** | **(53)** | **9e-005** | **1** | **U** | **K.YEELQVTVGR.H** |
|  | 1825 | **399.5371** | **1195.5896** | **1195.5907** | **-0.91** | **1** | **(34)** | **0.0054** | **1** | **U** | **K.KDVDNAYMIK.V** |
|  | 1826 | **598.8027** | **1195.5909** | **1195.5907** | **0.19** | **1** | **48** | **0.00026** | **1** | **U** | **K.KDVDNAYMIK.V** |
|  | 1891 | **604.8113** | **1207.6081** | **1207.6085** | **-0.27** | **0** | **61** | **1.4e-005** | **1** | **U** | **R.TAAENDFVTLK.K** |
|  | 1892 | **604.8115** | **1207.6085** | **1207.6085** | **0.02** | **0** | **(44)** | **0.00074** | **1** | **U** | **R.TAAENDFVTLK.K** |
|  | 2062 | **627.8047** | **1253.5948** | **1253.6001** | **-4.17** | **0** | **(55)** | **3.8e-005** | **1** | **U** | **R.GFSSGSAVVSGGSR.R** |
|  | 2063 | **627.8064** | **1253.5982** | **1253.6001** | **-1.44** | **0** | **71** | **9.5e-007** | **1** | **U** | **R.GFSSGSAVVSGGSR.R** |
|  | 2064 | **627.8066** | **1253.5986** | **1253.6001** | **-1.15** | **0** | **(55)** | **3.7e-005** | **1** | **U** | **R.GFSSGSAVVSGGSR.R** |
|  | 2343 | **660.7938** | **1319.5731** | **1319.5756** | **-1.91** | **0** | **91** | **3.7e-009** | **1** | **U** | **R.HGGGGGGFGGGGFGSR.S** |
|  | 2344 | **660.7943** | **1319.5739** | **1319.5756** | **-1.26** | **0** | **(77)** | **9.6e-008** | **1** | **U** | **R.HGGGGGGFGGGGFGSR.S** |
|  | 2345 | **440.8654** | **1319.5743** | **1319.5756** | **-0.96** | **0** | **(46)** | **0.00013** | **1** | **U** | **R.HGGGGGGFGGGGFGSR.S** |
|  | 2346 | **440.8657** | **1319.5753** | **1319.5756** | **-0.21** | **0** | **(31)** | **0.0034** | **1** | **U** | **R.HGGGGGGFGGGGFGSR.S** |
|  | 2373 | **665.3231** | **1328.6316** | **1328.6320** | **-0.35** | **0** | **68** | **1.9e-006** | **1** | **U** | **K.NVQDAIADAEQR.G** |
|  | 2374 | **665.3234** | **1328.6323** | **1328.6320** | **0.21** | **0** | **(63)** | **7e-006** | **1** | **U** | **K.NVQDAIADAEQR.G** |
|  | 2393 | **446.2417** | **1335.7033** | **1335.7034** | **-0.11** | **1** | **(51)** | **0.0001** | **1** | **U** | **R.TAAENDFVTLKK.D** |
|  | 2394 | **668.8591** | **1335.7037** | **1335.7034** | **0.22** | **1** | **(61)** | **9.1e-006** | **1** | **U** | **R.TAAENDFVTLKK.D** |
|  | 2395 | **668.8591** | **1335.7037** | **1335.7034** | **0.22** | **1** | **64** | **4.6e-006** | **1** | **U** | **R.TAAENDFVTLKK.D** |
|  | 2396 | **446.2434** | **1335.7085** | **1335.7034** | **3.80** | **1** | **(50)** | **0.00012** | **1** | **U** | **R.TAAENDFVTLKK.D** |
|  | 2427 | **448.5888** | **1342.7446** | **1342.7456** | **-0.72** | **1** | **(34)** | **0.0041** | **1** | **U** | **K.EIKIEISELNR.V** |
|  | 2428 | **672.3808** | **1342.7470** | **1342.7456** | **1.10** | **1** | **45** | **0.00033** | **1** | **U** | **K.EIKIEISELNR.V** |
|  | 2545 | **686.3587** | **1370.7028** | **1370.7041** | **-0.93** | **0** | **(64)** | **5.8e-006** | **1** | **U** | **K.LNDLEEALQQAK.E** |
|  | 2546 | **686.3601** | **1370.7057** | **1370.7041** | **1.13** | **0** | **70** | **1.4e-006** | **1** | **U** | **K.LNDLEEALQQAK.E** |
|  | 2631 | **696.3431** | **1390.6717** | **1390.6728** | **-0.78** | **1** | **54** | **6.1e-005** | **1** | **U** | **R.SKEEAEALYHSK.Y** |
|  | 2632 | **464.5648** | **1390.6727** | **1390.6728** | **-0.06** | **1** | **(13)** | **0.81** | **2** | **U** | **R.SKEEAEALYHSK.Y** |
|  | 2633 | **464.5651** | **1390.6734** | **1390.6728** | **0.39** | **1** | **(30)** | **0.015** | **1** | **U** | **R.SKEEAEALYHSK.Y** |
|  | 2920 | **730.9032** | **1459.7918** | **1459.7922** | **-0.26** | **0** | **24** | **0.046** | **1** | **U** | **K.VDLLNQEIEFLK.V** |
|  | 2954 | 738.3951 | 1474.7757 | 1474.7780 | -1.53 | 0 | (11) | 1.1 | 1 |  | R.FLEQQNQVLQTK.W |
|  | 2955 | 738.3966 | 1474.7785 | 1474.7780 | 0.38 | 0 | 74 | 6.1e-007 | 1 |  | R.FLEQQNQVLQTK.W |
|  | 2956 | 492.6006 | 1474.7799 | 1474.7780 | 1.27 | 0 | (48) | 0.00024 | 1 |  | R.FLEQQNQVLQTK.W |
|  | 2957 | 738.3979 | 1474.7812 | 1474.7780 | 2.20 | 0 | (67) | 3.3e-006 | 1 |  | R.FLEQQNQVLQTK.W |
|  | 3298 | **538.6199** | **1612.8380** | **1612.8420** | **-2.50** | **1** | **20** | **0.11** | **1** | **U** | **R.NKLNDLEEALQQAK.E** |
|  | 4060 | **655.6649** | **1963.9728** | **1963.9711** | **0.83** | **1** | **39** | **0.0014** | **1** | **U** | **K.NVQDAIADAEQRGEHALK.D** |
|  | 4260 | **1072.4772** | **2142.9398** | **2142.9375** | **1.06** | **0** | **14** | **0.19** | **1** | **U** | **R.TSQNSELNNMQDLVEDYK.K + Oxidation (M)** |
|  | 4376 | **752.6854** | **2255.0343** | **2255.0376** | **-1.46** | **1** | **62** | **6e-006** | **1** | **U** | **R.TSQNSELNNMQDLVEDYKK.K** |
|  | 4377 | **1128.5269** | **2255.0392** | **2255.0376** | **0.72** | **1** | **(42)** | **0.00052** | **1** | **U** | **R.TSQNSELNNMQDLVEDYKK.K** |

  


---

|  |  |
| --- | --- |
| **5.** | sp|P35527|K1C9\_HUMAN    **Mass:** 62255    **Score:** 1193   **Matches:** 49(43)  **Sequences:** 19(17)  **emPAI:** 2.62 |
|  | Keratin, type I cytoskeletal 9 OS=Homo sapiens OX=9606 GN=KRT9 PE=1 SV=3 |

|  |  |
| --- | --- |
|  | Check to include this hit in error tolerant search or archive report |
|  |  |

|  |  |  |  |  |  |  |  |  |  |  |  |
| --- | --- | --- | --- | --- | --- | --- | --- | --- | --- | --- | --- |
|  | **Query** | **Observed** | **Mr(expt)** | **Mr(calc)** | **ppm** | **Miss** | **Score** | **Expect** | **Rank** | **Unique** | **Peptide** |
|  | 182 | 405.2236 | 808.4326 | 808.4330 | -0.49 | 0 | (30) | 0.013 | 1 |  | R.LASYLDK.V |
|  | 183 | 405.2238 | 808.4329 | 808.4330 | -0.12 | 0 | 40 | 0.0016 | 1 |  | R.LASYLDK.V |
|  | 529 | **449.2099** | **896.4053** | **896.4062** | **-1.00** | **0** | **41** | **0.00069** | **1** | **U** | **R.MTLDDFR.I** |
|  | 530 | **449.2101** | **896.4057** | **896.4062** | **-0.60** | **0** | **(25)** | **0.023** | **1** | **U** | **R.MTLDDFR.I** |
|  | 531 | **449.2104** | **896.4062** | **896.4062** | **-0.04** | **0** | **(36)** | **0.0022** | **1** | **U** | **R.MTLDDFR.I** |
|  | 584 | **457.2080** | **912.4015** | **912.4011** | **0.44** | **0** | **(23)** | **0.025** | **1** | **U** | **R.MTLDDFR.I + Oxidation (M)** |
|  | 585 | **457.2083** | **912.4021** | **912.4011** | **1.04** | **0** | **(19)** | **0.055** | **1** | **U** | **R.MTLDDFR.I + Oxidation (M)** |
|  | 586 | **457.2086** | **912.4027** | **912.4011** | **1.78** | **0** | **(38)** | **0.00079** | **1** | **U** | **R.MTLDDFR.I + Oxidation (M)** |
|  | 770 | **484.2297** | **966.4449** | **966.4447** | **0.19** | **0** | **32** | **0.0074** | **1** | **U** | **K.IQDWYDK.K** |
|  | 771 | **484.2301** | **966.4456** | **966.4447** | **0.93** | **0** | **(26)** | **0.025** | **1** | **U** | **K.IQDWYDK.K** |
|  | 1177 | **530.7846** | **1059.5547** | **1059.5560** | **-1.29** | **0** | **(46)** | **0.00072** | **1** | **U** | **K.TLLDIDNTR.M** |
|  | 1178 | **530.7853** | **1059.5560** | **1059.5560** | **-0.02** | **0** | **52** | **0.00018** | **1** | **U** | **K.TLLDIDNTR.M** |
|  | 1179 | **530.7866** | **1059.5586** | **1059.5560** | **2.40** | **0** | **(20)** | **0.22** | **1** | **U** | **K.TLLDIDNTR.M** |
|  | 1211 | **533.7534** | **1065.4923** | **1065.4913** | **0.93** | **0** | **52** | **8.1e-005** | **1** | **U** | **K.FEMEQNLR.Q** |
|  | 1212 | **533.7534** | **1065.4923** | **1065.4913** | **0.93** | **0** | **(41)** | **0.0011** | **1** | **U** | **K.FEMEQNLR.Q** |
|  | 1242 | **537.7665** | **1073.5184** | **1073.5142** | **3.96** | **0** | **33** | **0.0072** | **1** | **U** | **R.QFSSSYLSR.S** |
|  | 1243 | **537.7678** | **1073.5211** | **1073.5142** | **6.45** | **0** | **(26)** | **0.04** | **1** | **U** | **R.QFSSSYLSR.S** |
|  | 1272 | **541.7505** | **1081.4864** | **1081.4862** | **0.19** | **0** | **(49)** | **0.00012** | **1** | **U** | **K.FEMEQNLR.Q + Oxidation (M)** |
|  | 1345 | **548.2766** | **1094.5385** | **1094.5396** | **-1.00** | **1** | **(37)** | **0.0025** | **1** | **U** | **K.IQDWYDKK.G** |
|  | 1346 | **365.8538** | **1094.5395** | **1094.5396** | **-0.17** | **1** | **(20)** | **0.15** | **1** | **U** | **K.IQDWYDKK.G** |
|  | 1347 | **548.2772** | **1094.5399** | **1094.5396** | **0.22** | **1** | **49** | **0.00018** | **1** | **U** | **K.IQDWYDKK.G** |
|  | 1467 | **561.2951** | **1120.5756** | **1120.5764** | **-0.67** | **0** | **(46)** | **0.00042** | **1** | **U** | **R.QEYEQLIAK.N** |
|  | 1469 | **561.2972** | **1120.5799** | **1120.5764** | **3.15** | **0** | **51** | **0.00012** | **1** | **U** | **R.QEYEQLIAK.N** |
|  | 1470 | **561.2989** | **1120.5832** | **1120.5764** | **6.09** | **0** | **(6)** | **3.7** | **2** | **U** | **R.QEYEQLIAK.N** |
|  | 1632 | **579.2990** | **1156.5835** | **1156.5836** | **-0.16** | **0** | **(59)** | **1.7e-005** | **1** | **U** | **R.QGVDADINGLR.Q** |
|  | 1633 | **579.2992** | **1156.5838** | **1156.5836** | **0.15** | **0** | **72** | **8e-007** | **1** | **U** | **R.QGVDADINGLR.Q** |
|  | 1791 | **595.8053** | **1189.5960** | **1189.6013** | **-4.38** | **0** | **(5)** | **6.1** | **2** | **U** | **R.QVLDNLTMEK.S** |
|  | 1792 | **595.8079** | **1189.6012** | **1189.6013** | **-0.07** | **0** | **(39)** | **0.0022** | **1** | **U** | **R.QVLDNLTMEK.S** |
|  | 1793 | **595.8083** | **1189.6020** | **1189.6013** | **0.65** | **0** | **42** | **0.0013** | **1** | **U** | **R.QVLDNLTMEK.S** |
|  | 1877 | **603.8068** | **1205.5990** | **1205.5962** | **2.32** | **0** | **(30)** | **0.017** | **1** | **U** | **R.QVLDNLTMEK.S + Oxidation (M)** |
|  | 1978 | **616.8022** | **1231.5898** | **1231.5906** | **-0.60** | **0** | **84** | **5.8e-008** | **1** | **U** | **R.SGGGGGGGLGSGGSIR.S** |
|  | 1989 | **618.2682** | **1234.5219** | **1234.5215** | **0.40** | **0** | **48** | **5.7e-005** | **1** | **U** | **R.FSSSSGYGGGSSR.V** |
|  | 2278 | **654.3424** | **1306.6703** | **1306.6703** | **-0.04** | **1** | **58** | **2.1e-005** | **1** | **U** | **R.IKFEMEQNLR.Q** |
|  | 2279 | **436.5641** | **1306.6705** | **1306.6703** | **0.16** | **1** | **(46)** | **0.00036** | **1** | **U** | **R.IKFEMEQNLR.Q** |
|  | 2280 | **654.3428** | **1306.6710** | **1306.6703** | **0.51** | **1** | **(25)** | **0.042** | **1** | **U** | **R.IKFEMEQNLR.Q** |
|  | 2318 | **658.3467** | **1314.6789** | **1314.6779** | **0.75** | **0** | **50** | **0.0002** | **1** | **U** | **K.DQIVDLTVGNNK.T** |
|  | 2355 | **662.3396** | **1322.6646** | **1322.6652** | **-0.45** | **1** | **(28)** | **0.021** | **1** | **U** | **R.IKFEMEQNLR.Q + Oxidation (M)** |
|  | 2356 | **441.8956** | **1322.6649** | **1322.6652** | **-0.27** | **1** | **(51)** | **0.00012** | **1** | **U** | **R.IKFEMEQNLR.Q + Oxidation (M)** |
|  | 2357 | **662.3398** | **1322.6651** | **1322.6652** | **-0.08** | **1** | **(30)** | **0.014** | **1** | **U** | **R.IKFEMEQNLR.Q + Oxidation (M)** |
|  | 2358 | **441.8958** | **1322.6656** | **1322.6652** | **0.28** | **1** | **(43)** | **0.00065** | **1** | **U** | **R.IKFEMEQNLR.Q + Oxidation (M)** |
|  | 3254 | **793.8849** | **1585.7552** | **1585.7583** | **-1.96** | **0** | **(88)** | **2e-008** | **1** | **U** | **K.VQALEEANNDLENK.I** |
|  | 3255 | **793.8862** | **1585.7578** | **1585.7583** | **-0.34** | **0** | **102** | **7.9e-010** | **1** | **U** | **K.VQALEEANNDLENK.I** |
|  | 3256 | **529.5942** | **1585.7609** | **1585.7583** | **1.62** | **0** | **(67)** | **2.8e-006** | **1** | **U** | **K.VQALEEANNDLENK.I** |
|  | 3807 | **613.3259** | **1836.9560** | **1836.9581** | **-1.17** | **0** | **16** | **0.3** | **1** | **U** | **R.HGVQELEIELQSQLSK.K** |
|  | 3844 | **617.9802** | **1850.9188** | **1850.9196** | **-0.42** | **1** | **4** | **5.1** | **9** | **U** | **K.TLNDMRQEYEQLIAK.N** |
|  | 4515 | **1255.5686** | **2509.1226** | **2509.1245** | **-0.72** | **0** | **98** | **7.2e-010** | **1** | **U** | **K.EIETYHNLLEGGQEDFESSGAGK.I** |
|  | 4516 | **837.3820** | **2509.1241** | **2509.1245** | **-0.16** | **0** | **(84)** | **1.9e-008** | **1** | **U** | **K.EIETYHNLLEGGQEDFESSGAGK.I** |
|  | 4517 | **837.3824** | **2509.1253** | **2509.1245** | **0.35** | **0** | **(70)** | **4.4e-007** | **1** | **U** | **K.EIETYHNLLEGGQEDFESSGAGK.I** |
|  | 4704 | **1075.0983** | **3222.2730** | **3222.2744** | **-0.44** | **0** | **116** | **2.5e-012** | **1** | **U** | **R.GGSGGSHGGGSGFGGESGGSYGGGEEASGSGGGYGGGSGK.S** |

  


---

|  |  |
| --- | --- |
| **6.** | sp|P02538|K2C6A\_HUMAN    **Mass:** 60293    **Score:** 870    **Matches:** 40(34)  **Sequences:** 26(25)  **emPAI:** 4.19 |
|  | Keratin, type II cytoskeletal 6A OS=Homo sapiens OX=9606 GN=KRT6A PE=1 SV=3 |

|  |  |
| --- | --- |
|  | Check to include this hit in error tolerant search or archive report |
|  |  |

|  |  |  |  |  |  |  |  |  |  |  |  |
| --- | --- | --- | --- | --- | --- | --- | --- | --- | --- | --- | --- |
|  | **Query** | **Observed** | **Mr(expt)** | **Mr(calc)** | **ppm** | **Miss** | **Score** | **Expect** | **Rank** | **Unique** | **Peptide** |
|  | 236 | 414.2182 | 826.4219 | 826.4225 | -0.72 | 0 | 40 | 0.0012 | 1 |  | K.FASFIDK.V |
|  | 237 | 414.2184 | 826.4223 | 826.4225 | -0.28 | 0 | (38) | 0.0019 | 1 |  | K.FASFIDK.V |
|  | 489 | **441.2384** | **880.4622** | **880.4654** | **-3.68** | **0** | **(0)** | **11** | **1** |  | **R.SLYGLGGSK.R** |
|  | 490 | **441.2427** | **880.4708** | **880.4654** | **6.11** | **0** | **41** | **0.00099** | **1** |  | **R.SLYGLGGSK.R** |
|  | 694 | **473.2593** | **944.5039** | **944.5039** | **0.03** | **1** | **37** | **0.0045** | **1** |  | **R.GRLDSELR.G** |
|  | 779 | **486.7606** | **971.5066** | **971.5036** | **3.08** | **0** | **48** | **0.00022** | **1** |  | **K.QEIAEINR.M** |
|  | 933 | **506.7563** | **1011.4980** | **1011.4985** | **-0.56** | **0** | **50** | **0.00011** | **1** | **U** | **R.SGFSSVSVSR.S** |
|  | 948 | **508.7734** | **1015.5323** | **1015.5298** | **2.41** | **0** | **(39)** | **0.0027** | **1** |  | **R.QLDSIVGER.G** |
|  | 949 | **508.7739** | **1015.5333** | **1015.5298** | **3.44** | **0** | **54** | **8.3e-005** | **1** |  | **R.QLDSIVGER.G** |
|  | 983 | **513.7317** | **1025.4488** | **1025.4488** | **0.05** | **0** | **31** | **0.0054** | **1** |  | **K.DVDAAYMNK.V** |
|  | 1276 | 361.5378 | 1081.5916 | 1081.5920 | -0.39 | 1 | (29) | 0.014 | 1 |  | K.FASFIDKVR.F |
|  | 1277 | 541.8041 | 1081.5937 | 1081.5920 | 1.57 | 1 | 44 | 0.00038 | 1 |  | K.FASFIDKVR.F |
|  | 1396 | 554.2670 | 1106.5194 | 1106.5356 | -14.65 | 0 | (3) | 6.8 | 2 |  | K.AQYEEIAQR.S |
|  | 1398 | 554.2739 | 1106.5333 | 1106.5356 | -2.07 | 0 | 40 | 0.0016 | 1 |  | K.AQYEEIAQR.S |
|  | 1616 | **577.2816** | **1152.5487** | **1152.5485** | **0.18** | **0** | **36** | **0.0034** | **1** |  | **K.EYQELMNVK.L** |
|  | 1677 | **583.2967** | **1164.5788** | **1164.5775** | **1.15** | **0** | **57** | **2.9e-005** | **1** |  | **K.YEELQVTAGR.H** |
|  | 1693 | **585.2791** | **1168.5437** | **1168.5434** | **0.23** | **0** | **(23)** | **0.066** | **1** |  | **K.EYQELMNVK.L + Oxidation (M)** |
|  | 1708 | **586.8227** | **1171.6308** | **1171.6309** | **-0.08** | **1** | **27** | **0.027** | **1** |  | **R.RQLDSIVGER.G** |
|  | 1750 | **591.2783** | **1180.5421** | **1180.5434** | **-1.13** | **0** | **(56)** | **2.5e-005** | **1** | **U** | **R.GMQDLVEDFK.N** |
|  | 1831 | **599.2771** | **1196.5396** | **1196.5383** | **1.09** | **0** | **60** | **9.4e-006** | **1** | **U** | **R.GMQDLVEDFK.N + Oxidation (M)** |
|  | 1865 | **602.3213** | **1202.6280** | **1202.6295** | **-1.25** | **0** | **(49)** | **0.00025** | **1** |  | **K.WTLLQEQGTK.T** |
|  | 1866 | **602.3218** | **1202.6291** | **1202.6295** | **-0.34** | **0** | **50** | **0.00017** | **1** |  | **K.WTLLQEQGTK.T** |
|  | 1906 | **606.7822** | **1211.5499** | **1211.5458** | **3.36** | **0** | **42** | **0.0006** | **1** |  | **R.AEAESWYQTK.Y** |
|  | 1928 | **611.8197** | **1221.6248** | **1221.6241** | **0.61** | **0** | **66** | **4.3e-006** | **1** |  | **R.TAAENEFVTLK.K** |
|  | 1929 | **611.8249** | **1221.6353** | **1221.6241** | **9.21** | **0** | **(2)** | **10** | **9** |  | **R.TAAENEFVTLK.K** |
|  | 1930 | **408.2196** | **1221.6370** | **1221.6241** | **10.5** | **0** | **(8)** | **2.5** | **4** |  | **R.TAAENEFVTLK.K** |
|  | 1965 | **615.3251** | **1228.6357** | **1228.6524** | **-13.58** | **1** | **4** | **7.6** | **10** |  | **R.QLDSIVGERGR.L** |
|  | 2319 | **439.2370** | **1314.6891** | **1314.6891** | **-0.04** | **1** | **36** | **0.0044** | **1** |  | **R.NTKQEIAEINR.M** |
|  | 2320 | **658.3520** | **1314.6895** | **1314.6891** | **0.32** | **1** | **(30)** | **0.019** | **1** |  | **R.NTKQEIAEINR.M** |
|  | 2484 | **453.2494** | **1356.7264** | **1356.7249** | **1.16** | **1** | **(29)** | **0.023** | **1** |  | **K.NKLEGLEDALQK.A** |
|  | 2485 | **679.3720** | **1356.7295** | **1356.7249** | **3.40** | **1** | **49** | **0.00021** | **1** |  | **K.NKLEGLEDALQK.A** |
|  | 2696 | **704.3595** | **1406.7044** | **1406.7041** | **0.21** | **0** | **56** | **3.9e-005** | **1** |  | **K.ADTLTDEINFLR.A** |
|  | 2763 | **712.8204** | **1423.6263** | **1423.6263** | **0.01** | **0** | **64** | **2.3e-006** | **1** |  | **R.GSGGLGGACGGAGFGSR.S** |
|  | 2877 | **724.3926** | **1446.7707** | **1446.7678** | **2.00** | **0** | **78** | **2.2e-007** | **1** |  | **R.AIGGGLSSVGGGSSTIK.Y** |
|  | 2907 | **728.3460** | **1454.6775** | **1454.6790** | **-1.03** | **1** | **54** | **4.4e-005** | **1** |  | **R.SRAEAESWYQTK.Y** |
|  | 2908 | **485.9004** | **1454.6793** | **1454.6790** | **0.19** | **1** | **(30)** | **0.012** | **1** |  | **R.SRAEAESWYQTK.Y** |
|  | 2962 | **738.8926** | **1475.7706** | **1475.7984** | **-18.81** | **1** | **(34)** | **0.0064** | **1** |  | **R.FLEQQNKVLETK.W** |
|  | 2963 | **738.8928** | **1475.7711** | **1475.7984** | **-18.49** | **1** | **58** | **2.3e-005** | **1** |  | **R.FLEQQNKVLETK.W** |
|  | 3274 | **799.8833** | **1597.7520** | **1597.7519** | **0.12** | **0** | **104** | **4.6e-010** | **1** |  | **R.ISIGGGSCAISGGYGSR.A** |
|  | 3395 | **829.8987** | **1657.7829** | **1657.7842** | **-0.76** | **0** | **68** | **1.7e-006** | **1** |  | **K.QCANLQAAIADAEQR.G** |

  


---

|  |  |
| --- | --- |
| **7.** | sp|P08779|K1C16\_HUMAN    **Mass:** 51578    **Score:** 842    **Matches:** 41(32)  **Sequences:** 23(20)  **emPAI:** 3.70 |
|  | Keratin, type I cytoskeletal 16 OS=Homo sapiens OX=9606 GN=KRT16 PE=1 SV=4 |

|  |  |
| --- | --- |
|  | Check to include this hit in error tolerant search or archive report |
|  |  |

|  |  |  |  |  |  |  |  |  |  |  |  |
| --- | --- | --- | --- | --- | --- | --- | --- | --- | --- | --- | --- |
|  | **Query** | **Observed** | **Mr(expt)** | **Mr(calc)** | **ppm** | **Miss** | **Score** | **Expect** | **Rank** | **Unique** | **Peptide** |
|  | 179 | 404.2029 | 806.3913 | 806.3923 | -1.23 | 0 | 41 | 0.0016 | 1 |  | R.LAADDFR.T |
|  | 180 | 404.2037 | 806.3928 | 806.3923 | 0.73 | 0 | (26) | 0.044 | 1 |  | R.LAADDFR.T |
|  | 182 | 405.2236 | 808.4326 | 808.4330 | -0.49 | 0 | (30) | 0.013 | 1 |  | R.LASYLDK.V |
|  | 183 | 405.2238 | 808.4329 | 808.4330 | -0.12 | 0 | 40 | 0.0016 | 1 |  | R.LASYLDK.V |
|  | 602 | **460.2132** | **918.4118** | **918.4123** | **-0.52** | **0** | **21** | **0.059** | **1** |  | **K.DYSPYFK.T** |
|  | 850 | **495.2728** | **988.5311** | **988.5301** | **0.98** | **1** | **14** | **0.85** | **2** | **U** | **R.SEVTELRR.V** |
|  | 1006 | **515.3003** | **1028.5860** | **1028.5866** | **-0.55** | **0** | **44** | **0.00051** | **1** |  | **R.VLDELTLAR.T** |
|  | 1007 | **515.3008** | **1028.5871** | **1028.5866** | **0.52** | **0** | **(33)** | **0.0058** | **1** |  | **R.VLDELTLAR.T** |
|  | 1197 | 355.5417 | 1063.6032 | 1063.6026 | 0.63 | 1 | (41) | 0.00067 | 1 |  | R.LASYLDKVR.A |
|  | 1198 | 532.8090 | 1063.6034 | 1063.6026 | 0.75 | 1 | 47 | 0.00017 | 1 |  | R.LASYLDKVR.A |
|  | 1312 | 545.7676 | 1089.5207 | 1089.5237 | -2.71 | 0 | 55 | 4.7e-005 | 1 |  | K.VTMQNLNDR.L |
|  | 1313 | 545.7702 | 1089.5258 | 1089.5237 | 1.99 | 0 | (31) | 0.013 | 1 |  | K.VTMQNLNDR.L |
|  | 1353 | **548.7691** | **1095.5236** | **1095.5237** | **-0.02** | **0** | **(36)** | **0.0037** | **1** | **U** | **R.DAETWFLSK.T** |
|  | 1354 | **548.7694** | **1095.5241** | **1095.5237** | **0.44** | **0** | **49** | **0.00019** | **1** | **U** | **R.DAETWFLSK.T** |
|  | 1473 | **561.7929** | **1121.5713** | **1121.5717** | **-0.35** | **0** | **45** | **0.00066** | **1** |  | **R.LEQEIATYR.R** |
|  | 1566 | **571.2450** | **1140.4754** | **1140.4757** | **-0.23** | **0** | **17** | **0.087** | **1** | **U** | **R.DQYEQMAEK.N** |
|  | 1774 | **395.9029** | **1184.6868** | **1184.6877** | **-0.72** | **1** | **(2)** | **6** | **2** |  | **R.RVLDELTLAR.T** |
|  | 1775 | **395.9031** | **1184.6873** | **1184.6877** | **-0.32** | **1** | **4** | **3.6** | **2** |  | **R.RVLDELTLAR.T** |
|  | 1849 | 601.3110 | 1200.6075 | 1200.6099 | -1.94 | 0 | 45 | 0.00052 | 1 | U | R.QTVEADVNGLR.R |
|  | 1850 | 601.3121 | 1200.6096 | 1200.6099 | -0.23 | 0 | (3) | 6.7 | 9 | U | R.QTVEADVNGLR.R |
|  | 1924 | **610.8045** | **1219.5944** | **1219.5932** | **1.05** | **0** | **54** | **6.6e-005** | **1** |  | **K.ASLENSLEETK.G** |
|  | 1925 | **610.8046** | **1219.5947** | **1219.5932** | **1.26** | **0** | **(37)** | **0.0031** | **1** |  | **K.ASLENSLEETK.G** |
|  | 2012 | **621.3008** | **1240.5871** | **1240.5870** | **0.12** | **0** | **31** | **0.0085** | **1** | **U** | **K.NHEEEMLALR.G** |
|  | 2095 | **630.3395** | **1258.6645** | **1258.6669** | **-1.92** | **1** | **52** | **0.0001** | **1** | **U** | **R.TKYEHELALR.Q** |
|  | 2096 | **420.5627** | **1258.6661** | **1258.6669** | **-0.65** | **1** | **(49)** | **0.00017** | **1** | **U** | **R.TKYEHELALR.Q** |
|  | 2155 | **639.7947** | **1277.5749** | **1277.5783** | **-2.62** | **0** | **53** | **5.3e-005** | **1** |  | **K.GSCGIGGGIGGGSSR.I** |
|  | 2255 | **651.3339** | **1300.6532** | **1300.6510** | **1.65** | **0** | **73** | **8.3e-007** | **1** |  | **R.ALEEANADLEVK.I** |
|  | 2401 | **669.8327** | **1337.6508** | **1337.6575** | **-5.01** | **0** | **(50)** | **0.00017** | **1** | **U** | **R.APSTYGGGLSVSSR.F** |
|  | 2402 | **669.8357** | **1337.6568** | **1337.6575** | **-0.54** | **0** | **73** | **6.8e-007** | **1** | **U** | **R.APSTYGGGLSVSSR.F** |
|  | 2476 | 453.2444 | 1356.7112 | 1356.7110 | 0.19 | 1 | 48 | 0.00024 | 1 | U | R.QTVEADVNGLRR.V |
|  | 2477 | 453.2444 | 1356.7113 | 1356.7110 | 0.25 | 1 | (2) | 11 | 8 | U | R.QTVEADVNGLRR.V |
|  | 2478 | **679.3630** | **1356.7114** | **1356.7110** | **0.32** | **1** | **(42)** | **0.001** | **1** | **U** | **R.QTVEADVNGLRR.V** |
|  | 2479 | 679.3632 | 1356.7118 | 1356.7110 | 0.59 | 1 | (14) | 0.72 | 5 | U | R.QTVEADVNGLRR.V |
|  | 2480 | 453.2449 | 1356.7128 | 1356.7110 | 1.34 | 1 | (2) | 10 | 6 | U | R.QTVEADVNGLRR.V |
|  | 2482 | 679.3646 | 1356.7147 | 1356.7110 | 2.74 | 1 | (15) | 0.48 | 2 | U | R.QTVEADVNGLRR.V |
|  | 2566 | **460.5807** | **1378.7202** | **1378.7204** | **-0.21** | **1** | **60** | **1.7e-005** | **1** |  | **K.TRLEQEIATYR.R** |
|  | 2567 | **690.3676** | **1378.7207** | **1378.7204** | **0.16** | **1** | **(51)** | **0.00011** | **1** |  | **K.TRLEQEIATYR.R** |
|  | 2690 | **703.3477** | **1404.6809** | **1404.6845** | **-2.55** | **0** | **72** | **9.3e-007** | **1** | **U** | **K.EVASNSELVQSSR.S** |
|  | 2814 | **717.3652** | **1432.7159** | **1432.7157** | **0.11** | **1** | **78** | **2.8e-007** | **1** |  | **K.ASLENSLEETKGR.Y** |
|  | 2815 | **478.5793** | **1432.7162** | **1432.7157** | **0.33** | **1** | **(61)** | **1.1e-005** | **1** |  | **K.ASLENSLEETKGR.Y** |
|  | 4445 | **784.0352** | **2349.0837** | **2349.0833** | **0.16** | **0** | **81** | **5.2e-008** | **1** | **U** | **R.LLEGEDAHLSSQQASGQSYSSR.E** |

  


---

|  |  |
| --- | --- |
| **8.** | sp|P02533|K1C14\_HUMAN    **Mass:** 51872    **Score:** 777    **Matches:** 44(32)  **Sequences:** 25(22)  **emPAI:** 3.67 |
|  | Keratin, type I cytoskeletal 14 OS=Homo sapiens OX=9606 GN=KRT14 PE=1 SV=4 |

|  |  |
| --- | --- |
|  | Check to include this hit in error tolerant search or archive report |
|  |  |

|  |  |  |  |  |  |  |  |  |  |  |  |
| --- | --- | --- | --- | --- | --- | --- | --- | --- | --- | --- | --- |
|  | **Query** | **Observed** | **Mr(expt)** | **Mr(calc)** | **ppm** | **Miss** | **Score** | **Expect** | **Rank** | **Unique** | **Peptide** |
|  | 2 | **350.7336** | **699.4526** | **699.4531** | **-0.65** | **0** | **35** | **0.0033** | **1** |  | **K.ILLDVK.T** |
|  | 179 | 404.2029 | 806.3913 | 806.3923 | -1.23 | 0 | 41 | 0.0016 | 1 |  | R.LAADDFR.T |
|  | 180 | 404.2037 | 806.3928 | 806.3923 | 0.73 | 0 | (26) | 0.044 | 1 |  | R.LAADDFR.T |
|  | 182 | 405.2236 | 808.4326 | 808.4330 | -0.49 | 0 | (30) | 0.013 | 1 |  | R.LASYLDK.V |
|  | 183 | 405.2238 | 808.4329 | 808.4330 | -0.12 | 0 | 40 | 0.0016 | 1 |  | R.LASYLDK.V |
|  | 602 | 460.2132 | 918.4118 | 918.4123 | -0.52 | 0 | 21 | 0.059 | 1 |  | K.DYSPYFK.T |
|  | 850 | 495.2728 | 988.5311 | 988.5301 | 1.00 | 1 | 24 | 0.08 | 1 |  | K.SEISELRR.T |
|  | 1006 | 515.3003 | 1028.5860 | 1028.5866 | -0.55 | 0 | 44 | 0.00051 | 1 |  | R.VLDELTLAR.A |
|  | 1007 | 515.3008 | 1028.5871 | 1028.5866 | 0.52 | 0 | (33) | 0.0058 | 1 |  | R.VLDELTLAR.A |
|  | 1039 | **519.2667** | **1036.5188** | **1036.5189** | **-0.12** | **0** | **33** | **0.0078** | **1** | **U** | **K.YETELNLR.M** |
|  | 1040 | 519.2669 | 1036.5193 | 1036.5189 | 0.36 | 0 | (2) | 10 | 5 | U | K.YETELNLR.M |
|  | 1197 | 355.5417 | 1063.6032 | 1063.6026 | 0.63 | 1 | (41) | 0.00067 | 1 |  | R.LASYLDKVR.A |
|  | 1198 | 532.8090 | 1063.6034 | 1063.6026 | 0.75 | 1 | 47 | 0.00017 | 1 |  | R.LASYLDKVR.A |
|  | 1312 | 545.7676 | 1089.5207 | 1089.5237 | -2.71 | 0 | 55 | 4.7e-005 | 1 |  | K.VTMQNLNDR.L |
|  | 1313 | 545.7702 | 1089.5258 | 1089.5237 | 1.99 | 0 | (31) | 0.013 | 1 |  | K.VTMQNLNDR.L |
|  | 1473 | 561.7929 | 1121.5713 | 1121.5717 | -0.35 | 0 | 45 | 0.00066 | 1 |  | R.LEQEIATYR.R |
|  | 1705 | **586.7661** | **1171.5177** | **1171.5186** | **-0.78** | **0** | **(2)** | **3.8** | **1** | **U** | **K.DAEEWFFTK.T** |
|  | 1706 | **586.7665** | **1171.5185** | **1171.5186** | **-0.05** | **0** | **51** | **4.8e-005** | **1** | **U** | **K.DAEEWFFTK.T** |
|  | 1774 | 395.9029 | 1184.6868 | 1184.6877 | -0.72 | 1 | (2) | 6 | 2 |  | R.RVLDELTLAR.A |
|  | 1775 | 395.9031 | 1184.6873 | 1184.6877 | -0.32 | 1 | 4 | 3.6 | 2 |  | R.RVLDELTLAR.A |
|  | 1924 | 610.8045 | 1219.5944 | 1219.5932 | 1.05 | 0 | 54 | 6.6e-005 | 1 |  | K.ASLENSLEETK.G |
|  | 1925 | 610.8046 | 1219.5947 | 1219.5932 | 1.26 | 0 | (37) | 0.0031 | 1 |  | K.ASLENSLEETK.G |
|  | 2021 | **621.7800** | **1241.5454** | **1241.5458** | **-0.36** | **0** | **35** | **0.0018** | **1** |  | **K.NHEEEMNALR.G** |
|  | 2116 | **633.8387** | **1265.6628** | **1265.6615** | **1.01** | **1** | **38** | **0.002** | **1** | **U** | **R.TKYETELNLR.M** |
|  | 2155 | 639.7947 | 1277.5749 | 1277.5783 | -2.62 | 0 | 53 | 5.3e-005 | 1 |  | K.GSCGIGGGIGGGSSR.I |
|  | 2255 | 651.3339 | 1300.6532 | 1300.6510 | 1.65 | 0 | 73 | 8.3e-007 | 1 |  | R.ALEEANADLEVK.I |
|  | 2505 | **454.2378** | **1359.6917** | **1359.6929** | **-0.83** | **1** | **32** | **0.011** | **1** |  | **R.MSVEADINGLRR.V** |
|  | 2506 | **680.8541** | **1359.6936** | **1359.6929** | **0.52** | **1** | **(14)** | **0.85** | **1** |  | **R.MSVEADINGLRR.V** |
|  | 2509 | **681.3486** | **1360.6826** | **1360.6834** | **-0.60** | **0** | **85** | **6.1e-008** | **1** |  | **R.EVATNSELVQSGK.S** |
|  | 2510 | **681.3487** | **1360.6828** | **1360.6834** | **-0.43** | **0** | **(72)** | **1.1e-006** | **1** |  | **R.EVATNSELVQSGK.S** |
|  | 2542 | **457.5541** | **1369.6405** | **1369.6408** | **-0.21** | **1** | **28** | **0.017** | **1** |  | **K.KNHEEEMNALR.G** |
|  | 2566 | 460.5807 | 1378.7202 | 1378.7204 | -0.21 | 1 | 60 | 1.7e-005 | 1 |  | K.TRLEQEIATYR.R |
|  | 2567 | 690.3676 | 1378.7207 | 1378.7204 | 0.16 | 1 | (51) | 0.00011 | 1 |  | K.TRLEQEIATYR.R |
|  | 2776 | **713.3516** | **1424.6887** | **1424.6896** | **-0.62** | **0** | **82** | **1.1e-007** | **1** | **U** | **R.APSTYGGGLSVSSSR.F** |
|  | 2777 | **713.3523** | **1424.6900** | **1424.6896** | **0.32** | **0** | **(24)** | **0.052** | **1** | **U** | **R.APSTYGGGLSVSSSR.F** |
|  | 2814 | 717.3652 | 1432.7159 | 1432.7157 | 0.11 | 1 | 78 | 2.8e-007 | 1 |  | K.ASLENSLEETKGR.Y |
|  | 2815 | 478.5793 | 1432.7162 | 1432.7157 | 0.33 | 1 | (61) | 1.1e-005 | 1 |  | K.ASLENSLEETKGR.Y |
|  | 2844 | **480.2376** | **1437.6911** | **1437.6922** | **-0.75** | **1** | **25** | **0.042** | **1** |  | **R.ILNEMRDQYEK.M** |
|  | 2845 | **719.8533** | **1437.6920** | **1437.6922** | **-0.13** | **1** | **(23)** | **0.068** | **1** |  | **R.ILNEMRDQYEK.M** |
|  | 2902 | **485.5682** | **1453.6826** | **1453.6871** | **-3.07** | **1** | **(8)** | **1.7** | **1** |  | **R.ILNEMRDQYEK.M + Oxidation (M)** |
|  | 2903 | **485.5695** | **1453.6866** | **1453.6871** | **-0.37** | **1** | **(4)** | **4.2** | **2** |  | **R.ILNEMRDQYEK.M + Oxidation (M)** |
|  | 2904 | **727.8509** | **1453.6872** | **1453.6871** | **0.10** | **1** | **(6)** | **2.7** | **1** |  | **R.ILNEMRDQYEK.M + Oxidation (M)** |
|  | 4205 | **695.9897** | **2084.9472** | **2084.9797** | **-15.57** | **0** | **3** | **3.8** | **3** | **U** | **R.GQVGGDVNVEMDAAPGVDLSR.I** |
|  | 4206 | **695.9899** | **2084.9478** | **2084.9797** | **-15.31** | **0** | **(1)** | **6.3** | **1** | **U** | **R.GQVGGDVNVEMDAAPGVDLSR.I** |

  


---

|  |  |
| --- | --- |
| **9.** | sp|P04259|K2C6B\_HUMAN    **Mass:** 60315    **Score:** 764    **Matches:** 35(28)  **Sequences:** 22(21)  **emPAI:** 3.20 |
|  | Keratin, type II cytoskeletal 6B OS=Homo sapiens OX=9606 GN=KRT6B PE=1 SV=5 |

|  |  |
| --- | --- |
|  | Check to include this hit in error tolerant search or archive report |
|  |  |

|  |  |  |  |  |  |  |  |  |  |  |  |
| --- | --- | --- | --- | --- | --- | --- | --- | --- | --- | --- | --- |
|  | **Query** | **Observed** | **Mr(expt)** | **Mr(calc)** | **ppm** | **Miss** | **Score** | **Expect** | **Rank** | **Unique** | **Peptide** |
|  | 236 | 414.2182 | 826.4219 | 826.4225 | -0.72 | 0 | 40 | 0.0012 | 1 |  | K.FASFIDK.V |
|  | 237 | 414.2184 | 826.4223 | 826.4225 | -0.28 | 0 | (38) | 0.0019 | 1 |  | K.FASFIDK.V |
|  | 489 | 441.2384 | 880.4622 | 880.4654 | -3.68 | 0 | (0) | 11 | 1 |  | R.SLYGLGGSK.R |
|  | 490 | 441.2427 | 880.4708 | 880.4654 | 6.11 | 0 | 41 | 0.00099 | 1 |  | R.SLYGLGGSK.R |
|  | 694 | 473.2593 | 944.5039 | 944.5039 | 0.03 | 1 | 37 | 0.0045 | 1 |  | R.GRLDSELR.N |
|  | 779 | 486.7606 | 971.5066 | 971.5036 | 3.08 | 0 | 48 | 0.00022 | 1 |  | K.QEIAEINR.M |
|  | 983 | 513.7317 | 1025.4488 | 1025.4488 | 0.05 | 0 | 31 | 0.0054 | 1 |  | K.DVDAAYMNK.V |
|  | 985 | **513.7642** | **1025.5139** | **1025.5142** | **-0.28** | **0** | **(0)** | **12** | **2** |  | **R.SGFSSISVSR.S** |
|  | 987 | **513.7656** | **1025.5167** | **1025.5142** | **2.47** | **0** | **56** | **3.3e-005** | **1** |  | **R.SGFSSISVSR.S** |
|  | 1276 | 361.5378 | 1081.5916 | 1081.5920 | -0.39 | 1 | (29) | 0.014 | 1 |  | K.FASFIDKVR.F |
|  | 1277 | 541.8041 | 1081.5937 | 1081.5920 | 1.57 | 1 | 44 | 0.00038 | 1 |  | K.FASFIDKVR.F |
|  | 1396 | 554.2670 | 1106.5194 | 1106.5356 | -14.65 | 0 | (3) | 6.8 | 2 |  | K.AQYEEIAQR.S |
|  | 1398 | 554.2739 | 1106.5333 | 1106.5356 | -2.07 | 0 | 40 | 0.0016 | 1 |  | K.AQYEEIAQR.S |
|  | 1616 | 577.2816 | 1152.5487 | 1152.5485 | 0.18 | 0 | 36 | 0.0034 | 1 |  | K.EYQELMNVK.L |
|  | 1693 | 585.2791 | 1168.5437 | 1168.5434 | 0.23 | 0 | (23) | 0.066 | 1 |  | K.EYQELMNVK.L + Oxidation (M) |
|  | 1734 | 590.3039 | 1178.5932 | 1178.5931 | 0.09 | 0 | (53) | 9.2e-005 | 1 |  | K.YEELQITAGR.H |
|  | 1735 | 590.3040 | 1178.5934 | 1178.5931 | 0.21 | 0 | 61 | 1.2e-005 | 1 |  | K.YEELQITAGR.H |
|  | 1865 | 602.3213 | 1202.6280 | 1202.6295 | -1.25 | 0 | (49) | 0.00025 | 1 |  | K.WTLLQEQGTK.T |
|  | 1866 | 602.3218 | 1202.6291 | 1202.6295 | -0.34 | 0 | 50 | 0.00017 | 1 |  | K.WTLLQEQGTK.T |
|  | 1869 | **602.7974** | **1203.5803** | **1203.5805** | **-0.19** | **0** | **20** | **0.13** | **1** |  | **R.NMQDLVEDLK.N** |
|  | 1906 | 606.7822 | 1211.5499 | 1211.5458 | 3.36 | 0 | 42 | 0.0006 | 1 |  | R.AEAESWYQTK.Y |
|  | 1928 | 611.8197 | 1221.6248 | 1221.6241 | 0.61 | 0 | 66 | 4.3e-006 | 1 |  | R.TAAENEFVTLK.K |
|  | 1929 | 611.8249 | 1221.6353 | 1221.6241 | 9.21 | 0 | (2) | 10 | 9 |  | R.TAAENEFVTLK.K |
|  | 1930 | 408.2196 | 1221.6370 | 1221.6241 | 10.5 | 0 | (8) | 2.5 | 4 |  | R.TAAENEFVTLK.K |
|  | 2319 | 439.2370 | 1314.6891 | 1314.6891 | -0.04 | 1 | 36 | 0.0044 | 1 |  | R.NTKQEIAEINR.M |
|  | 2320 | 658.3520 | 1314.6895 | 1314.6891 | 0.32 | 1 | (30) | 0.019 | 1 |  | R.NTKQEIAEINR.M |
|  | 2484 | 453.2494 | 1356.7264 | 1356.7249 | 1.16 | 1 | (29) | 0.023 | 1 |  | K.NKLEGLEDALQK.A |
|  | 2485 | 679.3720 | 1356.7295 | 1356.7249 | 3.40 | 1 | 49 | 0.00021 | 1 |  | K.NKLEGLEDALQK.A |
|  | 2696 | 704.3595 | 1406.7044 | 1406.7041 | 0.21 | 0 | 56 | 3.9e-005 | 1 |  | K.ADTLTDEINFLR.A |
|  | 2763 | 712.8204 | 1423.6263 | 1423.6263 | 0.01 | 0 | 64 | 2.3e-006 | 1 |  | R.GSGGLGGACGGAGFGSR.S |
|  | 2823 | **718.3715** | **1434.7284** | **1434.7315** | **-2.16** | **0** | **98** | **2.4e-009** | **1** | **U** | **R.ATGGGLSSVGGGSSTIK.Y** |
|  | 2907 | 728.3460 | 1454.6775 | 1454.6790 | -1.03 | 1 | 54 | 4.4e-005 | 1 |  | R.SRAEAESWYQTK.Y |
|  | 2908 | 485.9004 | 1454.6793 | 1454.6790 | 0.19 | 1 | (30) | 0.012 | 1 |  | R.SRAEAESWYQTK.Y |
|  | 3274 | 799.8833 | 1597.7520 | 1597.7519 | 0.12 | 0 | 104 | 4.6e-010 | 1 |  | R.ISIGGGSCAISGGYGSR.A |
|  | 3395 | 829.8987 | 1657.7829 | 1657.7842 | -0.76 | 0 | 68 | 1.7e-006 | 1 |  | K.QCANLQAAIADAEQR.G |

  


---

|  |  |
| --- | --- |
| **10.** | sp|P13647|K2C5\_HUMAN    **Mass:** 62568    **Score:** 716    **Matches:** 38(31)  **Sequences:** 24(22)  **emPAI:** 3.19 |
|  | Keratin, type II cytoskeletal 5 OS=Homo sapiens OX=9606 GN=KRT5 PE=1 SV=3 |

|  |  |
| --- | --- |
|  | Check to include this hit in error tolerant search or archive report |
|  |  |

|  |  |  |  |  |  |  |  |  |  |  |  |
| --- | --- | --- | --- | --- | --- | --- | --- | --- | --- | --- | --- |
|  | **Query** | **Observed** | **Mr(expt)** | **Mr(calc)** | **ppm** | **Miss** | **Score** | **Expect** | **Rank** | **Unique** | **Peptide** |
|  | 236 | 414.2182 | 826.4219 | 826.4225 | -0.72 | 0 | 40 | 0.0012 | 1 |  | K.FASFIDK.V |
|  | 237 | 414.2184 | 826.4223 | 826.4225 | -0.28 | 0 | (38) | 0.0019 | 1 |  | K.FASFIDK.V |
|  | 671 | **469.7516** | **937.4887** | **937.4869** | **2.00** | **0** | **(20)** | **0.1** | **1** | **U** | **R.SLYNLGGSK.R** |
|  | 672 | **469.7521** | **937.4896** | **937.4869** | **2.91** | **0** | **25** | **0.033** | **1** | **U** | **R.SLYNLGGSK.R** |
|  | 694 | 473.2593 | 944.5039 | 944.5039 | 0.03 | 1 | 37 | 0.0045 | 1 |  | R.GRLDSELR.N |
|  | 948 | 508.7734 | 1015.5323 | 1015.5298 | 2.41 | 0 | (39) | 0.0027 | 1 |  | R.QLDSIVGER.G |
|  | 949 | 508.7739 | 1015.5333 | 1015.5298 | 3.44 | 0 | 54 | 8.3e-005 | 1 |  | R.QLDSIVGER.G |
|  | 983 | 513.7317 | 1025.4488 | 1025.4488 | 0.05 | 0 | 31 | 0.0054 | 1 |  | K.DVDAAYMNK.V |
|  | 1164 | **529.3037** | **1056.5929** | **1056.5927** | **0.13** | **1** | **39** | **0.0015** | **1** | **U** | **R.LRAEIDNVK.K** |
|  | 1276 | 361.5378 | 1081.5916 | 1081.5920 | -0.39 | 1 | (29) | 0.014 | 1 |  | K.FASFIDKVR.F |
|  | 1277 | 541.8041 | 1081.5937 | 1081.5920 | 1.57 | 1 | 44 | 0.00038 | 1 |  | K.FASFIDKVR.F |
|  | 1422 | **556.2908** | **1110.5670** | **1110.5669** | **0.04** | **0** | **46** | **0.00032** | **1** | **U** | **R.ISISTSGGSFR.N** |
|  | 1582 | **572.3162** | **1142.6178** | **1142.6183** | **-0.43** | **0** | **67** | **3.5e-006** | **1** | **U** | **K.LAELEEALQK.A** |
|  | 1583 | **572.3204** | **1142.6263** | **1142.6183** | **7.05** | **0** | **(6)** | **4.2** | **5** | **U** | **K.LAELEEALQK.A** |
|  | 1626 | **578.2716** | **1154.5285** | **1154.5277** | **0.70** | **0** | **30** | **0.0082** | **1** | **U** | **R.EYQELMNTK.L** |
|  | 1708 | 586.8227 | 1171.6308 | 1171.6309 | -0.08 | 1 | 27 | 0.027 | 1 |  | R.RQLDSIVGER.G |
|  | 1811 | **597.7911** | **1193.5676** | **1193.5676** | **-0.03** | **0** | **55** | **4.4e-005** | **1** | **U** | **K.YEELQQTAGR.H** |
|  | 1865 | 602.3213 | 1202.6280 | 1202.6295 | -1.25 | 0 | (49) | 0.00025 | 1 |  | K.WTLLQEQGTK.T |
|  | 1866 | 602.3218 | 1202.6291 | 1202.6295 | -0.34 | 0 | 50 | 0.00017 | 1 |  | K.WTLLQEQGTK.T |
|  | 1965 | 615.3251 | 1228.6357 | 1228.6524 | -13.58 | 1 | 4 | 7.6 | 10 |  | R.QLDSIVGERGR.L |
|  | 2000 | **619.7894** | **1237.5643** | **1237.5649** | **-0.46** | **0** | **24** | **0.039** | **1** | **U** | **R.NMQDLVEDFK.N** |
|  | 2022 | **621.7859** | **1241.5572** | **1241.5564** | **0.66** | **0** | **49** | **7.4e-005** | **1** | **U** | **R.TEAESWYQTK.Y** |
|  | 2165 | **641.8218** | **1281.6291** | **1281.6275** | **1.29** | **0** | **53** | **5.9e-005** | **1** | **U** | **R.TTAENEFVMLK.K** |
|  | 2702 | **705.8356** | **1409.6567** | **1409.6722** | **-10.96** | **0** | **(7)** | **1.9** | **2** | **U** | **R.VSLAGACGVGGYGSR.S** |
|  | 2704 | **705.8450** | **1409.6755** | **1409.6722** | **2.37** | **0** | **77** | **2.7e-007** | **1** | **U** | **R.VSLAGACGVGGYGSR.S** |
|  | 2705 | **705.8646** | **1409.7146** | **1409.7151** | **-0.35** | **0** | **(41)** | **0.0011** | **1** | **U** | **R.SFSTASAITPSVSR.T** |
|  | 2706 | **705.8658** | **1409.7171** | **1409.7151** | **1.47** | **0** | **51** | **0.00011** | **1** | **U** | **R.SFSTASAITPSVSR.T** |
|  | 2707 | **470.9148** | **1409.7225** | **1409.7224** | **0.02** | **1** | **(47)** | **0.00023** | **1** | **U** | **R.TTAENEFVMLKK.D** |
|  | 2708 | **705.8688** | **1409.7230** | **1409.7224** | **0.40** | **1** | **72** | **7.4e-007** | **1** | **U** | **R.TTAENEFVMLKK.D** |
|  | 2775 | **713.3407** | **1424.6668** | **1424.6680** | **-0.78** | **0** | **(19)** | **0.14** | **1** | **U** | **K.VDALMDEINFMK.M** |
|  | 2780 | **713.8666** | **1425.7186** | **1425.7173** | **0.89** | **1** | **(46)** | **0.00033** | **1** | **U** | **R.TTAENEFVMLKK.D + Oxidation (M)** |
|  | 2781 | **476.2477** | **1425.7212** | **1425.7173** | **2.71** | **1** | **(38)** | **0.0022** | **1** | **U** | **R.TTAENEFVMLKK.D + Oxidation (M)** |
|  | 2846 | **720.3596** | **1438.7046** | **1438.7053** | **-0.48** | **0** | **39** | **0.002** | **1** | **U** | **R.GLGVGFGSGGGSSSSVK.F** |
|  | 2855 | **721.3383** | **1440.6620** | **1440.6629** | **-0.63** | **0** | **(55)** | **3e-005** | **1** | **U** | **K.VDALMDEINFMK.M + Oxidation (M)** |
|  | 2915 | **729.3364** | **1456.6583** | **1456.6578** | **0.35** | **0** | **55** | **2.6e-005** | **1** | **U** | **K.VDALMDEINFMK.M + 2 Oxidation (M)** |
|  | 2982 | **743.3517** | **1484.6888** | **1484.6895** | **-0.50** | **1** | **37** | **0.002** | **1** | **U** | **R.SRTEAESWYQTK.Y** |
|  | 2983 | **495.9036** | **1484.6889** | **1484.6895** | **-0.47** | **1** | **(14)** | **0.41** | **1** | **U** | **R.SRTEAESWYQTK.Y** |
|  | 3485 | **851.3931** | **1700.7717** | **1700.7900** | **-10.75** | **0** | **7** | **1.4** | **1** | **U** | **K.QCANLQNAIADAEQR.G** |

  


---

|  |  |
| --- | --- |
| **11.** | sp|P48668|K2C6C\_HUMAN    **Mass:** 60273    **Score:** 714    **Matches:** 37(29)  **Sequences:** 24(22)  **emPAI:** 3.43 |
|  | Keratin, type II cytoskeletal 6C OS=Homo sapiens OX=9606 GN=KRT6C PE=1 SV=3 |

|  |  |
| --- | --- |
|  | Check to include this hit in error tolerant search or archive report |
|  |  |

|  |  |  |  |  |  |  |  |  |  |  |  |
| --- | --- | --- | --- | --- | --- | --- | --- | --- | --- | --- | --- |
|  | **Query** | **Observed** | **Mr(expt)** | **Mr(calc)** | **ppm** | **Miss** | **Score** | **Expect** | **Rank** | **Unique** | **Peptide** |
|  | 236 | 414.2182 | 826.4219 | 826.4225 | -0.72 | 0 | 40 | 0.0012 | 1 |  | K.FASFIDK.V |
|  | 237 | 414.2184 | 826.4223 | 826.4225 | -0.28 | 0 | (38) | 0.0019 | 1 |  | K.FASFIDK.V |
|  | 489 | 441.2384 | 880.4622 | 880.4654 | -3.68 | 0 | (0) | 11 | 1 |  | R.SLYGLGGSK.R |
|  | 490 | 441.2427 | 880.4708 | 880.4654 | 6.11 | 0 | 41 | 0.00099 | 1 |  | R.SLYGLGGSK.R |
|  | 694 | 473.2593 | 944.5039 | 944.5039 | 0.03 | 1 | 37 | 0.0045 | 1 |  | R.GRLDSELR.N |
|  | 779 | 486.7606 | 971.5066 | 971.5036 | 3.08 | 0 | 48 | 0.00022 | 1 |  | K.QEIAEINR.M |
|  | 948 | 508.7734 | 1015.5323 | 1015.5298 | 2.41 | 0 | (39) | 0.0027 | 1 |  | R.QLDSIVGER.G |
|  | 949 | 508.7739 | 1015.5333 | 1015.5298 | 3.44 | 0 | 54 | 8.3e-005 | 1 |  | R.QLDSIVGER.G |
|  | 983 | 513.7317 | 1025.4488 | 1025.4488 | 0.05 | 0 | 31 | 0.0054 | 1 |  | K.DVDAAYMNK.V |
|  | 985 | 513.7642 | 1025.5139 | 1025.5142 | -0.28 | 0 | (0) | 12 | 2 |  | R.SGFSSISVSR.S |
|  | 987 | 513.7656 | 1025.5167 | 1025.5142 | 2.47 | 0 | 56 | 3.3e-005 | 1 |  | R.SGFSSISVSR.S |
|  | 1276 | 361.5378 | 1081.5916 | 1081.5920 | -0.39 | 1 | (29) | 0.014 | 1 |  | K.FASFIDKVR.F |
|  | 1277 | 541.8041 | 1081.5937 | 1081.5920 | 1.57 | 1 | 44 | 0.00038 | 1 |  | K.FASFIDKVR.F |
|  | 1396 | 554.2670 | 1106.5194 | 1106.5356 | -14.65 | 0 | (3) | 6.8 | 2 |  | K.AQYEEIAQR.S |
|  | 1398 | 554.2739 | 1106.5333 | 1106.5356 | -2.07 | 0 | 40 | 0.0016 | 1 |  | K.AQYEEIAQR.S |
|  | 1616 | 577.2816 | 1152.5487 | 1152.5485 | 0.18 | 0 | 36 | 0.0034 | 1 |  | K.EYQELMNVK.L |
|  | 1677 | 583.2967 | 1164.5788 | 1164.5775 | 1.15 | 0 | 57 | 2.9e-005 | 1 |  | K.YEELQVTAGR.H |
|  | 1693 | 585.2791 | 1168.5437 | 1168.5434 | 0.23 | 0 | (23) | 0.066 | 1 |  | K.EYQELMNVK.L + Oxidation (M) |
|  | 1708 | 586.8227 | 1171.6308 | 1171.6309 | -0.08 | 1 | 27 | 0.027 | 1 |  | R.RQLDSIVGER.G |
|  | 1865 | 602.3213 | 1202.6280 | 1202.6295 | -1.25 | 0 | (49) | 0.00025 | 1 |  | K.WTLLQEQGTK.T |
|  | 1866 | 602.3218 | 1202.6291 | 1202.6295 | -0.34 | 0 | 50 | 0.00017 | 1 |  | K.WTLLQEQGTK.T |
|  | 1869 | 602.7974 | 1203.5803 | 1203.5805 | -0.19 | 0 | 20 | 0.13 | 1 |  | R.NMQDLVEDLK.N |
|  | 1906 | 606.7822 | 1211.5499 | 1211.5458 | 3.36 | 0 | 42 | 0.0006 | 1 |  | R.AEAESWYQTK.Y |
|  | 1928 | 611.8197 | 1221.6248 | 1221.6241 | 0.61 | 0 | 66 | 4.3e-006 | 1 |  | R.TAAENEFVTLK.K |
|  | 1929 | 611.8249 | 1221.6353 | 1221.6241 | 9.21 | 0 | (2) | 10 | 9 |  | R.TAAENEFVTLK.K |
|  | 1930 | 408.2196 | 1221.6370 | 1221.6241 | 10.5 | 0 | (8) | 2.5 | 4 |  | R.TAAENEFVTLK.K |
|  | 1965 | 615.3251 | 1228.6357 | 1228.6524 | -13.58 | 1 | 4 | 7.6 | 10 |  | R.QLDSIVGERGR.L |
|  | 2319 | 439.2370 | 1314.6891 | 1314.6891 | -0.04 | 1 | 36 | 0.0044 | 1 |  | R.NTKQEIAEINR.M |
|  | 2320 | 658.3520 | 1314.6895 | 1314.6891 | 0.32 | 1 | (30) | 0.019 | 1 |  | R.NTKQEIAEINR.M |
|  | 2484 | 453.2494 | 1356.7264 | 1356.7249 | 1.16 | 1 | (29) | 0.023 | 1 |  | K.NKLEGLEDALQK.A |
|  | 2485 | 679.3720 | 1356.7295 | 1356.7249 | 3.40 | 1 | 49 | 0.00021 | 1 |  | K.NKLEGLEDALQK.A |
|  | 2696 | 704.3595 | 1406.7044 | 1406.7041 | 0.21 | 0 | 56 | 3.9e-005 | 1 |  | K.ADTLTDEINFLR.A |
|  | 2763 | 712.8204 | 1423.6263 | 1423.6263 | 0.01 | 0 | 64 | 2.3e-006 | 1 |  | R.GSGGLGGACGGAGFGSR.S |
|  | 2877 | 724.3926 | 1446.7707 | 1446.7678 | 2.00 | 0 | 78 | 2.2e-007 | 1 |  | R.AIGGGLSSVGGGSSTIK.Y |
|  | 2907 | 728.3460 | 1454.6775 | 1454.6790 | -1.03 | 1 | 54 | 4.4e-005 | 1 |  | R.SRAEAESWYQTK.Y |
|  | 2908 | 485.9004 | 1454.6793 | 1454.6790 | 0.19 | 1 | (30) | 0.012 | 1 |  | R.SRAEAESWYQTK.Y |
|  | 3274 | 799.8833 | 1597.7520 | 1597.7519 | 0.12 | 0 | 104 | 4.6e-010 | 1 |  | R.ISIGGGSCAISGGYGSR.A |

  


---

|  |  |
| --- | --- |
| **12.** | sp|Q14764|MVP\_HUMAN    **Mass:** 99551    **Score:** 555    **Matches:** 35(24)  **Sequences:** 24(19)  **emPAI:** 1.10 |
|  | Major vault protein OS=Homo sapiens OX=9606 GN=MVP PE=1 SV=4 |

|  |  |
| --- | --- |
|  | Check to include this hit in error tolerant search or archive report |
|  |  |

|  |  |  |  |  |  |  |  |  |  |  |  |
| --- | --- | --- | --- | --- | --- | --- | --- | --- | --- | --- | --- |
|  | **Query** | **Observed** | **Mr(expt)** | **Mr(calc)** | **ppm** | **Miss** | **Score** | **Expect** | **Rank** | **Unique** | **Peptide** |
|  | 261 | **417.2387** | **832.4629** | **832.4629** | **-0.06** | **0** | **(24)** | **0.063** | **1** | **U** | **R.VLFAPMR.M** |
|  | 366 | **425.2362** | **848.4578** | **848.4578** | **-0.04** | **0** | **25** | **0.043** | **1** | **U** | **R.VLFAPMR.M + Oxidation (M)** |
|  | 456 | **436.2858** | **870.5571** | **870.5538** | **3.76** | **0** | **25** | **0.02** | **1** | **U** | **K.LLQSLGLK.S** |
|  | 492 | **441.2637** | **880.5128** | **880.5130** | **-0.28** | **0** | **39** | **0.00066** | **1** | **U** | **K.SLQPLAPR.N** |
|  | 493 | **441.2642** | **880.5138** | **880.5130** | **0.84** | **0** | **(2)** | **3.1** | **5** | **U** | **K.SLQPLAPR.N** |
|  | 712 | **475.7453** | **949.4761** | **949.4756** | **0.49** | **0** | **22** | **0.095** | **1** | **U** | **K.ALLDFEDK.D** |
|  | 865 | **496.7739** | **991.5332** | **991.5338** | **-0.57** | **0** | **35** | **0.0041** | **1** | **U** | **R.ELELVYAR.A** |
|  | 950 | **508.7852** | **1015.5558** | **1015.5549** | **0.81** | **0** | **38** | **0.003** | **1** | **U** | **R.AQLELEVSK.A** |
|  | 1180 | **530.7873** | **1059.5600** | **1059.5600** | **-0.01** | **0** | **(51)** | **0.00015** | **1** | **U** | **R.GPLEYVPSAK.V** |
|  | 1181 | **530.7875** | **1059.5604** | **1059.5600** | **0.34** | **0** | **63** | **9.9e-006** | **1** | **U** | **R.GPLEYVPSAK.V** |
|  | 1438 | **557.8086** | **1113.6026** | **1113.6030** | **-0.30** | **0** | **43** | **0.00072** | **1** | **U** | **K.AQQLAEVEVK.K** |
|  | 1511 | **565.8062** | **1129.5977** | **1129.5979** | **-0.12** | **0** | **63** | **1.1e-005** | **1** | **U** | **R.IEGEGSVLQAK.L** |
|  | 1512 | **565.8065** | **1129.5985** | **1129.5979** | **0.53** | **0** | **(30)** | **0.022** | **1** | **U** | **R.IEGEGSVLQAK.L** |
|  | 1757 | **394.8747** | **1181.6022** | **1181.5975** | **3.92** | **0** | **11** | **1.1** | **1** | **U** | **K.GPDGMALPRPR.D + Oxidation (M)** |
|  | 2025 | **414.9066** | **1241.6979** | **1241.6979** | **-0.06** | **1** | **(31)** | **0.0078** | **1** | **U** | **K.AQQLAEVEVKK.F** |
|  | 2026 | **621.8564** | **1241.6982** | **1241.6979** | **0.23** | **1** | **39** | **0.0013** | **1** | **U** | **K.AQQLAEVEVKK.F** |
|  | 2083 | **629.3283** | **1256.6421** | **1256.6435** | **-1.11** | **0** | **(18)** | **0.26** | **1** | **U** | **R.DLAVAGPEMQVK.L** |
|  | 2084 | **629.3286** | **1256.6427** | **1256.6435** | **-0.63** | **0** | **33** | **0.0071** | **1** | **U** | **R.DLAVAGPEMQVK.L** |
|  | 2146 | **637.3270** | **1272.6394** | **1272.6384** | **0.79** | **0** | **(28)** | **0.027** | **1** | **U** | **R.DLAVAGPEMQVK.L + Oxidation (M)** |
|  | 2193 | **643.8156** | **1285.6165** | **1285.6190** | **-1.93** | **0** | **(22)** | **0.075** | **1** | **U** | **R.TAVFGFETSEAK.G** |
|  | 2194 | **643.8184** | **1285.6222** | **1285.6190** | **2.44** | **0** | **49** | **0.00019** | **1** | **U** | **R.TAVFGFETSEAK.G** |
|  | 2263 | **652.3143** | **1302.6141** | **1302.6139** | **0.15** | **0** | **62** | **7.9e-006** | **1** | **U** | **R.HYCTVANPVSR.D** |
|  | 2337 | **660.3334** | **1318.6522** | **1318.6551** | **-2.20** | **0** | **4** | **7.7** | **1** | **U** | **K.QMTEAIGPSTIR.D + Oxidation (M)** |
|  | 2399 | **669.3670** | **1336.7194** | **1336.7238** | **-3.26** | **0** | **23** | **0.052** | **1** | **U** | **K.ELPPGVEELLNK.G** |
|  | 2400 | **669.3687** | **1336.7227** | **1336.7238** | **-0.79** | **0** | **(12)** | **0.66** | **1** | **U** | **K.ELPPGVEELLNK.G** |
|  | 2523 | **455.8889** | **1364.6448** | **1364.6460** | **-0.82** | **1** | **11** | **1.1** | **1** | **U** | **K.ALLDFEDKDGDK.V** |
|  | 2641 | **465.2303** | **1392.6692** | **1392.6674** | **1.31** | **0** | **(3)** | **7.3** | **6** | **U** | **R.GAVASVTFDDFHK.N** |
|  | 2642 | **697.3427** | **1392.6707** | **1392.6674** | **2.40** | **0** | **51** | **0.00012** | **1** | **U** | **R.GAVASVTFDDFHK.N** |
|  | 2906 | **727.8524** | **1453.6902** | **1453.6912** | **-0.69** | **0** | **27** | **0.024** | **1** | **U** | **K.LFSVPDFVGDACK.A** |
|  | 3106 | **766.3850** | **1530.7555** | **1530.7579** | **-1.60** | **0** | **58** | **2.1e-005** | **1** | **U** | **R.VPHNAAVQVYDYR.E** |
|  | 3107 | **511.2604** | **1530.7594** | **1530.7579** | **0.97** | **0** | **(35)** | **0.0049** | **1** | **U** | **R.VPHNAAVQVYDYR.E** |
|  | 3155 | **771.9103** | **1541.8061** | **1541.8049** | **0.81** | **0** | **80** | **1.4e-007** | **1** | **U** | **K.AQALAIETEAELQR.V** |
|  | 3764 | **908.4804** | **1814.9463** | **1814.9454** | **0.46** | **0** | **25** | **0.031** | **1** | **U** | **R.LAQDPFPLYPGEVLEK.D** |
|  | 4007 | **965.9914** | **1929.9682** | **1929.9684** | **-0.06** | **0** | **74** | **4.9e-007** | **1** | **U** | **R.QAIPLDENEGIYVQDVK.T** |
|  | 4265 | **718.0353** | **2151.0842** | **2151.0861** | **-0.90** | **0** | **23** | **0.064** | **1** | **U** | **R.IPPYHYIHVLDQNSNVSR.V** |

  


---

|  |  |
| --- | --- |
| **13.** | sp|P07437|TBB5\_HUMAN    **Mass:** 50095    **Score:** 504    **Matches:** 26(19)  **Sequences:** 13(10)  **emPAI:** 1.44 |
|  | Tubulin beta chain OS=Homo sapiens OX=9606 GN=TUBB PE=1 SV=2 |

|  |  |
| --- | --- |
|  | Check to include this hit in error tolerant search or archive report |
|  |  |

|  |  |  |  |  |  |  |  |  |  |  |  |
| --- | --- | --- | --- | --- | --- | --- | --- | --- | --- | --- | --- |
|  | **Query** | **Observed** | **Mr(expt)** | **Mr(calc)** | **ppm** | **Miss** | **Score** | **Expect** | **Rank** | **Unique** | **Peptide** |
|  | 997 | **514.7636** | **1027.5125** | **1027.5121** | **0.47** | **0** | **37** | **0.0027** | **1** |  | **K.TAVCDIPPR.G** |
|  | 998 | **514.7717** | **1027.5289** | **1027.5121** | **16.4** | **0** | **(6)** | **3.9** | **3** |  | **K.TAVCDIPPR.G** |
|  | 1000 | **514.7734** | **1027.5323** | **1027.5121** | **19.7** | **0** | **(15)** | **0.41** | **1** |  | **K.TAVCDIPPR.G** |
|  | 1045 | **520.2997** | **1038.5849** | **1038.5862** | **-1.24** | **0** | **21** | **0.061** | **1** |  | **R.YLTVAAVFR.G** |
|  | 1046 | **520.3008** | **1038.5870** | **1038.5862** | **0.77** | **0** | **(12)** | **0.54** | **1** |  | **R.YLTVAAVFR.G** |
|  | 1509 | **565.8023** | **1129.5901** | **1129.5880** | **1.83** | **0** | **53** | **8.7e-005** | **1** |  | **R.FPGQLNADLR.K** |
|  | 1510 | **565.8048** | **1129.5949** | **1129.5880** | **6.15** | **0** | **(38)** | **0.0036** | **1** |  | **R.FPGQLNADLR.K** |
|  | 1583 | 572.3204 | 1142.6263 | 1142.6270 | -0.63 | 0 | 51 | 0.00014 | 1 |  | K.LAVNMVPFPR.L |
|  | 1584 | **572.3210** | **1142.6275** | **1142.6270** | **0.44** | **0** | **(30)** | **0.018** | **1** |  | **K.LAVNMVPFPR.L** |
|  | 1641 | **580.3174** | **1158.6203** | **1158.6219** | **-1.40** | **0** | **(25)** | **0.065** | **1** |  | **K.LAVNMVPFPR.L + Oxidation (M)** |
|  | 1642 | **580.3181** | **1158.6217** | **1158.6219** | **-0.22** | **0** | **(29)** | **0.029** | **1** |  | **K.LAVNMVPFPR.L + Oxidation (M)** |
|  | 1964 | **615.3022** | **1228.5899** | **1228.5910** | **-0.88** | **0** | **43** | **0.00057** | **1** |  | **R.ISEQFTAMFR.R** |
|  | 2133 | **636.3674** | **1270.7203** | **1270.7220** | **-1.32** | **1** | **50** | **9.7e-005** | **1** |  | **R.KLAVNMVPFPR.L** |
|  | 2254 | **651.3233** | **1300.6320** | **1300.6299** | **1.64** | **0** | **53** | **6.8e-005** | **1** | **U** | **R.ISVYYNEATGGK.Y** |
|  | 2339 | **660.3550** | **1318.6955** | **1318.6955** | **0.01** | **0** | **(52)** | **0.0001** | **1** |  | **R.IMNTFSVVPSPK.V** |
|  | 2340 | **660.3560** | **1318.6974** | **1318.6955** | **1.41** | **0** | **(45)** | **0.00051** | **1** |  | **R.IMNTFSVVPSPK.V** |
|  | 2389 | **668.3522** | **1334.6899** | **1334.6904** | **-0.39** | **0** | **56** | **4.1e-005** | **1** |  | **R.IMNTFSVVPSPK.V + Oxidation (M)** |
|  | 2390 | **668.3546** | **1334.6945** | **1334.6904** | **3.09** | **0** | **(34)** | **0.0061** | **1** |  | **R.IMNTFSVVPSPK.V + Oxidation (M)** |
|  | 2874 | **723.8502** | **1445.6858** | **1445.6820** | **2.59** | **0** | **56** | **2.9e-005** | **1** |  | **K.EVDEQMLNVQNK.N** |
|  | 3301 | **808.4214** | **1614.8282** | **1614.8287** | **-0.30** | **0** | **48** | **0.00023** | **1** | **U** | **R.AILVDLEPGTMDSVR.S** |
|  | 3331 | **816.4196** | **1630.8247** | **1630.8236** | **0.65** | **0** | **(26)** | **0.044** | **1** | **U** | **R.AILVDLEPGTMDSVR.S + Oxidation (M)** |
|  | 3332 | **816.4199** | **1630.8252** | **1630.8236** | **0.95** | **0** | **(33)** | **0.0077** | **1** | **U** | **R.AILVDLEPGTMDSVR.S + Oxidation (M)** |
|  | 3396 | **830.4515** | **1658.8884** | **1658.8879** | **0.28** | **0** | **14** | **0.44** | **1** | **U** | **R.ALTVPELTQQVFDAK.N** |
|  | 3471 | **848.9187** | **1695.8228** | **1695.8257** | **-1.65** | **0** | **4** | **6** | **2** |  | **K.NSSYFVEWIPNNVK.T** |
|  | 3778 | **911.9653** | **1821.9160** | **1821.9156** | **0.24** | **0** | **80** | **1.4e-007** | **1** | **U** | **R.EIVHIQAGQCGNQIGAK.F** |
|  | 3779 | **608.3131** | **1821.9175** | **1821.9156** | **1.07** | **0** | **(68)** | **2e-006** | **1** | **U** | **R.EIVHIQAGQCGNQIGAK.F** |

  


---

|  |  |
| --- | --- |
| **14.** | sp|P60709|ACTB\_HUMAN    **Mass:** 42052    **Score:** 466    **Matches:** 22(17)  **Sequences:** 11(10)  **emPAI:** 2.11 |
|  | Actin, cytoplasmic 1 OS=Homo sapiens OX=9606 GN=ACTB PE=1 SV=1 |

|  |  |
| --- | --- |
|  | Check to include this hit in error tolerant search or archive report |
|  |  |

|  |  |  |  |  |  |  |  |  |  |  |  |
| --- | --- | --- | --- | --- | --- | --- | --- | --- | --- | --- | --- |
|  | **Query** | **Observed** | **Mr(expt)** | **Mr(calc)** | **ppm** | **Miss** | **Score** | **Expect** | **Rank** | **Unique** | **Peptide** |
|  | 142 | **398.2396** | **794.4647** | **794.4650** | **-0.41** | **0** | **27** | **0.016** | **1** |  | **K.IIAPPER.K** |
|  | 802 | **488.7278** | **975.4409** | **975.4410** | **-0.06** | **0** | **63** | **4.7e-006** | **1** |  | **K.AGFAGDDAPR.A** |
|  | 888 | **499.7467** | **997.4789** | **997.4790** | **-0.11** | **0** | **27** | **0.025** | **1** |  | **R.DLTDYLMK.I** |
|  | 889 | **499.7469** | **997.4793** | **997.4790** | **0.27** | **0** | **(19)** | **0.13** | **1** |  | **R.DLTDYLMK.I** |
|  | 938 | **507.7443** | **1013.4740** | **1013.4739** | **0.09** | **0** | **(26)** | **0.024** | **1** |  | **R.DLTDYLMK.I + Oxidation (M)** |
|  | 939 | **507.7447** | **1013.4748** | **1013.4739** | **0.82** | **0** | **(5)** | **2.9** | **1** |  | **R.DLTDYLMK.I + Oxidation (M)** |
|  | 1520 | **566.7672** | **1131.5199** | **1131.5197** | **0.19** | **0** | **45** | **0.00032** | **1** | **U** | **R.GYSFTTTAER.E** |
|  | 1653 | **581.3132** | **1160.6119** | **1160.6111** | **0.71** | **0** | **47** | **0.00042** | **1** |  | **K.EITALAPSTMK.I** |
|  | 1703 | **586.2890** | **1170.5634** | **1170.5638** | **-0.30** | **0** | **71** | **9.3e-007** | **1** |  | **R.HQGVMVGMGQK.D** |
|  | 1729 | **589.3102** | **1176.6058** | **1176.6060** | **-0.17** | **0** | **(44)** | **0.00076** | **1** |  | **K.EITALAPSTMK.I + Oxidation (M)** |
|  | 1842 | **400.2400** | **1197.6981** | **1197.6982** | **-0.07** | **0** | **31** | **0.006** | **1** |  | **R.AVFPSIVGRPR.H** |
|  | 2677 | **468.2770** | **1401.8090** | **1401.7901** | **13.5** | **1** | **2** | **3.5** | **2** |  | **K.EITALAPSTMKIK.I** |
|  | 3063 | **379.6925** | **1514.7408** | **1514.7419** | **-0.72** | **0** | **(17)** | **0.24** | **2** |  | **K.IWHHTFYNELR.V** |
|  | 3064 | **505.9215** | **1514.7426** | **1514.7419** | **0.49** | **0** | **(28)** | **0.022** | **1** |  | **K.IWHHTFYNELR.V** |
|  | 3065 | **758.3787** | **1514.7428** | **1514.7419** | **0.59** | **0** | **40** | **0.0013** | **1** |  | **K.IWHHTFYNELR.V** |
|  | 3071 | **758.8526** | **1515.6906** | **1515.6954** | **-3.12** | **0** | **86** | **2.4e-008** | **1** |  | **K.QEYDESGPSIVHR.K** |
|  | 3072 | **506.2388** | **1515.6947** | **1515.6954** | **-0.47** | **0** | **(51)** | **7.2e-005** | **1** |  | **K.QEYDESGPSIVHR.K** |
|  | 3073 | **506.2394** | **1515.6965** | **1515.6954** | **0.74** | **0** | **(9)** | **1.1** | **1** |  | **K.QEYDESGPSIVHR.K** |
|  | 4040 | **652.0263** | **1953.0571** | **1953.0571** | **0.00** | **0** | **(34)** | **0.0027** | **1** | **U** | **R.VAPEEHPVLLTEAPLNPK.A** |
|  | 4041 | **652.0264** | **1953.0573** | **1953.0571** | **0.09** | **0** | **(33)** | **0.0034** | **1** | **U** | **R.VAPEEHPVLLTEAPLNPK.A** |
|  | 4042 | **977.5362** | **1953.0578** | **1953.0571** | **0.37** | **0** | **(76)** | **1.8e-007** | **1** | **U** | **R.VAPEEHPVLLTEAPLNPK.A** |
|  | 4043 | **977.5367** | **1953.0588** | **1953.0571** | **0.88** | **0** | **76** | **1.6e-007** | **1** | **U** | **R.VAPEEHPVLLTEAPLNPK.A** |

  

|  |  |
| --- | --- |
|  | |
|  | **Proteins matching the same set of peptides:** |

|  |  |
| --- | --- |
|  | sp|P63261|ACTG\_HUMAN    **Mass:** 42108    **Score:** 466    **Matches:** 22(17)  **Sequences:** 11(10) |
|  | Actin, cytoplasmic 2 OS=Homo sapiens OX=9606 GN=ACTG1 PE=1 SV=1 |

---

|  |  |
| --- | --- |
| **15.** | sp|P68371|TBB4B\_HUMAN    **Mass:** 50255    **Score:** 462    **Matches:** 22(16)  **Sequences:** 11(9)  **emPAI:** 1.14 |
|  | Tubulin beta-4B chain OS=Homo sapiens OX=9606 GN=TUBB4B PE=1 SV=1 |

|  |  |
| --- | --- |
|  | Check to include this hit in error tolerant search or archive report |
|  |  |

|  |  |  |  |  |  |  |  |  |  |  |  |
| --- | --- | --- | --- | --- | --- | --- | --- | --- | --- | --- | --- |
|  | **Query** | **Observed** | **Mr(expt)** | **Mr(calc)** | **ppm** | **Miss** | **Score** | **Expect** | **Rank** | **Unique** | **Peptide** |
|  | 997 | 514.7636 | 1027.5125 | 1027.5121 | 0.47 | 0 | 37 | 0.0027 | 1 |  | K.TAVCDIPPR.G |
|  | 998 | 514.7717 | 1027.5289 | 1027.5121 | 16.4 | 0 | (6) | 3.9 | 3 |  | K.TAVCDIPPR.G |
|  | 1000 | 514.7734 | 1027.5323 | 1027.5121 | 19.7 | 0 | (15) | 0.41 | 1 |  | K.TAVCDIPPR.G |
|  | 1045 | 520.2997 | 1038.5849 | 1038.5862 | -1.24 | 0 | 21 | 0.061 | 1 |  | R.YLTVAAVFR.G |
|  | 1046 | 520.3008 | 1038.5870 | 1038.5862 | 0.77 | 0 | (12) | 0.54 | 1 |  | R.YLTVAAVFR.G |
|  | 1509 | 565.8023 | 1129.5901 | 1129.5880 | 1.83 | 0 | 53 | 8.7e-005 | 1 |  | R.FPGQLNADLR.K |
|  | 1510 | 565.8048 | 1129.5949 | 1129.5880 | 6.15 | 0 | (38) | 0.0036 | 1 |  | R.FPGQLNADLR.K |
|  | 1583 | 572.3204 | 1142.6263 | 1142.6270 | -0.63 | 0 | 51 | 0.00014 | 1 |  | K.LAVNMVPFPR.L |
|  | 1584 | 572.3210 | 1142.6275 | 1142.6270 | 0.44 | 0 | (30) | 0.018 | 1 |  | K.LAVNMVPFPR.L |
|  | 1641 | 580.3174 | 1158.6203 | 1158.6219 | -1.40 | 0 | (25) | 0.065 | 1 |  | K.LAVNMVPFPR.L + Oxidation (M) |
|  | 1642 | 580.3181 | 1158.6217 | 1158.6219 | -0.22 | 0 | (29) | 0.029 | 1 |  | K.LAVNMVPFPR.L + Oxidation (M) |
|  | 1964 | 615.3022 | 1228.5899 | 1228.5910 | -0.88 | 0 | 43 | 0.00057 | 1 |  | R.ISEQFTAMFR.R |
|  | 2133 | 636.3674 | 1270.7203 | 1270.7220 | -1.32 | 1 | 50 | 9.7e-005 | 1 |  | R.KLAVNMVPFPR.L |
|  | 2339 | 660.3550 | 1318.6955 | 1318.6955 | 0.01 | 0 | (52) | 0.0001 | 1 |  | R.IMNTFSVVPSPK.V |
|  | 2340 | 660.3560 | 1318.6974 | 1318.6955 | 1.41 | 0 | (45) | 0.00051 | 1 |  | R.IMNTFSVVPSPK.V |
|  | 2371 | **664.8287** | **1327.6428** | **1327.6408** | **1.49** | **0** | **58** | **1.8e-005** | **1** | **U** | **R.INVYYNEATGGK.Y** |
|  | 2389 | 668.3522 | 1334.6899 | 1334.6904 | -0.39 | 0 | 56 | 4.1e-005 | 1 |  | R.IMNTFSVVPSPK.V + Oxidation (M) |
|  | 2390 | 668.3546 | 1334.6945 | 1334.6904 | 3.09 | 0 | (34) | 0.0061 | 1 |  | R.IMNTFSVVPSPK.V + Oxidation (M) |
|  | 2874 | 723.8502 | 1445.6858 | 1445.6820 | 2.59 | 0 | 56 | 2.9e-005 | 1 |  | K.EVDEQMLNVQNK.N |
|  | 3471 | 848.9187 | 1695.8228 | 1695.8257 | -1.65 | 0 | 4 | 6 | 2 |  | K.NSSYFVEWIPNNVK.T |
|  | 3778 | 911.9653 | 1821.9160 | 1821.9156 | 0.24 | 0 | 80 | 1.4e-007 | 1 |  | R.EIVHLQAGQCGNQIGAK.F |
|  | 3779 | 608.3131 | 1821.9175 | 1821.9156 | 1.07 | 0 | (68) | 2e-006 | 1 |  | R.EIVHLQAGQCGNQIGAK.F |

  


---

|  |  |
| --- | --- |
| **16.** | sp|Q04695|K1C17\_HUMAN    **Mass:** 48361    **Score:** 457    **Matches:** 34(21)  **Sequences:** 18(14)  **emPAI:** 2.07 |
|  | Keratin, type I cytoskeletal 17 OS=Homo sapiens OX=9606 GN=KRT17 PE=1 SV=2 |

|  |  |
| --- | --- |
|  | Check to include this hit in error tolerant search or archive report |
|  |  |

|  |  |  |  |  |  |  |  |  |  |  |  |
| --- | --- | --- | --- | --- | --- | --- | --- | --- | --- | --- | --- |
|  | **Query** | **Observed** | **Mr(expt)** | **Mr(calc)** | **ppm** | **Miss** | **Score** | **Expect** | **Rank** | **Unique** | **Peptide** |
|  | 2 | 350.7336 | 699.4526 | 699.4531 | -0.65 | 0 | 35 | 0.0033 | 1 |  | K.ILLDVK.T |
|  | 179 | 404.2029 | 806.3913 | 806.3923 | -1.23 | 0 | 41 | 0.0016 | 1 |  | R.LAADDFR.T |
|  | 180 | 404.2037 | 806.3928 | 806.3923 | 0.73 | 0 | (26) | 0.044 | 1 |  | R.LAADDFR.T |
|  | 182 | 405.2236 | 808.4326 | 808.4330 | -0.49 | 0 | (30) | 0.013 | 1 |  | R.LASYLDK.V |
|  | 183 | 405.2238 | 808.4329 | 808.4330 | -0.12 | 0 | 40 | 0.0016 | 1 |  | R.LASYLDK.V |
|  | 850 | 495.2728 | 988.5311 | 988.5301 | 1.00 | 1 | 24 | 0.08 | 1 |  | K.SEISELRR.T |
|  | 866 | 497.2538 | 992.4930 | 992.4927 | 0.32 | 0 | 8 | 2.9 | 6 |  | K.FETEQALR.L |
|  | 867 | 497.2541 | 992.4936 | 992.4927 | 0.94 | 0 | (4) | 6.6 | 8 |  | K.FETEQALR.L |
|  | 1006 | 515.3003 | 1028.5860 | 1028.5866 | -0.55 | 0 | 44 | 0.00051 | 1 |  | R.VLDELTLAR.A |
|  | 1007 | 515.3008 | 1028.5871 | 1028.5866 | 0.52 | 0 | (33) | 0.0058 | 1 |  | R.VLDELTLAR.A |
|  | 1197 | 355.5417 | 1063.6032 | 1063.6026 | 0.63 | 1 | (41) | 0.00067 | 1 |  | R.LASYLDKVR.A |
|  | 1198 | 532.8090 | 1063.6034 | 1063.6026 | 0.75 | 1 | 47 | 0.00017 | 1 |  | R.LASYLDKVR.A |
|  | 1447 | **559.2907** | **1116.5669** | **1116.5663** | **0.54** | **0** | **56** | **5.2e-005** | **1** | **U** | **R.TIVEEVQDGK.V** |
|  | 1450 | **559.2963** | **1116.5780** | **1116.5663** | **10.5** | **0** | **(0)** | **18** | **3** | **U** | **R.TIVEEVQDGK.V** |
|  | 1473 | 561.7929 | 1121.5713 | 1121.5717 | -0.35 | 0 | 45 | 0.00066 | 1 |  | R.LEQEIATYR.R |
|  | 1586 | **572.7509** | **1143.4871** | **1143.4873** | **-0.12** | **0** | **46** | **0.00013** | **1** | **U** | **K.DAEDWFFSK.T** |
|  | 1774 | 395.9029 | 1184.6868 | 1184.6877 | -0.72 | 1 | (2) | 6 | 2 |  | R.RVLDELTLAR.A |
|  | 1775 | 395.9031 | 1184.6873 | 1184.6877 | -0.32 | 1 | 4 | 3.6 | 2 |  | R.RVLDELTLAR.A |
|  | 1928 | 611.8197 | 1221.6248 | 1221.6353 | -8.58 | 1 | (6) | 4.4 | 5 |  | R.TKFETEQALR.L |
|  | 1929 | 611.8249 | 1221.6353 | 1221.6353 | 0.01 | 1 | (26) | 0.035 | 1 |  | R.TKFETEQALR.L |
|  | 1930 | 408.2196 | 1221.6370 | 1221.6353 | 1.34 | 1 | 40 | 0.0016 | 1 |  | R.TKFETEQALR.L |
|  | 2021 | 621.7800 | 1241.5454 | 1241.5458 | -0.36 | 0 | 35 | 0.0018 | 1 |  | K.NHEEEMNALR.G |
|  | 2425 | **448.2523** | **1341.7350** | **1341.7364** | **-1.11** | **1** | **15** | **0.39** | **1** | **U** | **R.LSVEADINGLRR.V** |
|  | 2426 | **671.8769** | **1341.7392** | **1341.7364** | **2.08** | **1** | **(2)** | **6.6** | **3** | **U** | **R.LSVEADINGLRR.V** |
|  | 2509 | 681.3486 | 1360.6826 | 1360.6834 | -0.60 | 0 | 85 | 6.1e-008 | 1 |  | R.EVATNSELVQSGK.S |
|  | 2510 | 681.3487 | 1360.6828 | 1360.6834 | -0.43 | 0 | (72) | 1.1e-006 | 1 |  | R.EVATNSELVQSGK.S |
|  | 2542 | 457.5541 | 1369.6405 | 1369.6408 | -0.21 | 1 | 28 | 0.017 | 1 |  | K.KNHEEEMNALR.G |
|  | 2566 | 460.5807 | 1378.7202 | 1378.7204 | -0.21 | 1 | 60 | 1.7e-005 | 1 |  | K.TRLEQEIATYR.R |
|  | 2567 | 690.3676 | 1378.7207 | 1378.7204 | 0.16 | 1 | (51) | 0.00011 | 1 |  | K.TRLEQEIATYR.R |
|  | 2844 | 480.2376 | 1437.6911 | 1437.6922 | -0.75 | 1 | 25 | 0.042 | 1 |  | R.ILNEMRDQYEK.M |
|  | 2845 | 719.8533 | 1437.6920 | 1437.6922 | -0.13 | 1 | (23) | 0.068 | 1 |  | R.ILNEMRDQYEK.M |
|  | 2902 | 485.5682 | 1453.6826 | 1453.6871 | -3.07 | 1 | (8) | 1.7 | 1 |  | R.ILNEMRDQYEK.M + Oxidation (M) |
|  | 2903 | 485.5695 | 1453.6866 | 1453.6871 | -0.37 | 1 | (4) | 4.2 | 2 |  | R.ILNEMRDQYEK.M + Oxidation (M) |
|  | 2904 | 727.8509 | 1453.6872 | 1453.6871 | 0.10 | 1 | (6) | 2.7 | 1 |  | R.ILNEMRDQYEK.M + Oxidation (M) |

  


---

|  |  |
| --- | --- |
| **17.** | sp|Q86YZ3|HORN\_HUMAN    **Mass:** 283140   **Score:** 454    **Matches:** 15(9)  **Sequences:** 8(4)  **emPAI:** 0.08 |
|  | Hornerin OS=Homo sapiens OX=9606 GN=HRNR PE=1 SV=2 |

|  |  |
| --- | --- |
|  | Check to include this hit in error tolerant search or archive report |
|  |  |

|  |  |  |  |  |  |  |  |  |  |  |  |
| --- | --- | --- | --- | --- | --- | --- | --- | --- | --- | --- | --- |
|  | **Query** | **Observed** | **Mr(expt)** | **Mr(calc)** | **ppm** | **Miss** | **Score** | **Expect** | **Rank** | **Unique** | **Peptide** |
|  | 1490 | **563.2775** | **1124.5405** | **1124.5210** | **17.3** | **1** | **2** | **9.7** | **2** | **U** | **R.SSSRGPYESR.S** |
|  | 2656 | **698.3229** | **1394.6313** | **1394.6175** | **9.93** | **0** | **1** | **6.6** | **8** | **U** | **R.YGQQGSGSGQSPSR.G** |
|  | 4424 | **773.6603** | **2317.9590** | **2317.9585** | **0.24** | **0** | **89** | **2.8e-009** | **1** | **U** | **R.HGSGSGQSSSYGPYGSGSGWSSSR.G** |
|  | 4442 | **1174.9917** | **2347.9688** | **2347.9690** | **-0.07** | **0** | **115** | **5.2e-012** | **1** | **U** | **R.HGSGSGQSSSYSPYGSGSGWSSSR.G** |
|  | 4443 | **783.6637** | **2347.9693** | **2347.9690** | **0.11** | **0** | **(74)** | **6.7e-008** | **1** | **U** | **R.HGSGSGQSSSYSPYGSGSGWSSSR.G** |
|  | 4444 | **783.6646** | **2347.9720** | **2347.9690** | **1.27** | **0** | **(92)** | **1.1e-009** | **1** | **U** | **R.HGSGSGQSSSYSPYGSGSGWSSSR.G** |
|  | 4502 | **826.3445** | **2476.0118** | **2476.0137** | **-0.77** | **0** | **(10)** | **0.1** | **1** | **U** | **R.HGSGSGHSSSYGQHGSGSGWSSSSGR.H** |
|  | 4503 | **826.3448** | **2476.0127** | **2476.0137** | **-0.39** | **0** | **(6)** | **0.26** | **1** | **U** | **R.HGSGSGHSSSYGQHGSGSGWSSSSGR.H** |
|  | 4504 | **620.0106** | **2476.0131** | **2476.0137** | **-0.22** | **0** | **74** | **4.5e-008** | **1** | **U** | **R.HGSGSGHSSSYGQHGSGSGWSSSSGR.H** |
|  | 4505 | **620.0108** | **2476.0141** | **2476.0137** | **0.17** | **0** | **(29)** | **0.0015** | **1** | **U** | **R.HGSGSGHSSSYGQHGSGSGWSSSSGR.H** |
|  | 4506 | **496.2102** | **2476.0147** | **2476.0137** | **0.40** | **0** | **(30)** | **0.0013** | **1** | **U** | **R.HGSGSGHSSSYGQHGSGSGWSSSSGR.H** |
|  | 4624 | **930.7256** | **2789.1551** | **2789.1550** | **0.05** | **0** | **(22)** | **0.011** | **1** | **U** | **R.GHYESGSGQTSGFGQHESGSGQSSGYSK.H** |
|  | 4625 | **698.2964** | **2789.1565** | **2789.1550** | **0.53** | **0** | **46** | **4e-005** | **1** | **U** | **R.GHYESGSGQTSGFGQHESGSGQSSGYSK.H** |
|  | 4639 | **574.6502** | **2868.2144** | **2868.2156** | **-0.44** | **0** | **14** | **0.075** | **1** | **U** | **R.SEQHGSSSGLSSSYGQHGSGSHQSSGHGR.Q** |
|  | 4790 | **950.5975** | **4747.9512** | **4747.9514** | **-0.05** | **0** | **10** | **0.1** | **1** | **U** | **R.SSSSGQHGSGLGESSGFGHHESSSGQSSSYSQHGSGSGHSSGYGQHGSR.S** |

  


---

|  |  |
| --- | --- |
| **18.** | sp|P16989|YBOX3\_HUMAN    **Mass:** 40066    **Score:** 436    **Matches:** 15(11)  **Sequences:** 9(7)  **emPAI:** 1.21 |
|  | Y-box-binding protein 3 OS=Homo sapiens OX=9606 GN=YBX3 PE=1 SV=4 |

|  |  |
| --- | --- |
|  | Check to include this hit in error tolerant search or archive report |
|  |  |

|  |  |  |  |  |  |  |  |  |  |  |  |
| --- | --- | --- | --- | --- | --- | --- | --- | --- | --- | --- | --- |
|  | **Query** | **Observed** | **Mr(expt)** | **Mr(calc)** | **ppm** | **Miss** | **Score** | **Expect** | **Rank** | **Unique** | **Peptide** |
|  | 23 | 361.1922 | 720.3698 | 720.3707 | -1.33 | 0 | 22 | 0.13 | 1 |  | K.WFNVR.N |
|  | 24 | 361.1923 | 720.3700 | 720.3707 | -0.99 | 0 | (12) | 1.4 | 1 |  | K.WFNVR.N |
|  | 677 | 470.7357 | 939.4568 | 939.4563 | 0.61 | 0 | 49 | 0.00017 | 1 |  | R.NGYGFINR.N |
|  | 2105 | **421.8951** | **1262.6636** | **1262.6632** | **0.30** | **0** | **3** | **8.4** | **8** | **U** | **R.RPQYRPQYR.Q** |
|  | 2195 | 429.5627 | 1285.6664 | 1285.6667 | -0.21 | 0 | (22) | 0.12 | 1 |  | K.EDVFVHQTAIK.K |
|  | 2196 | 643.8409 | 1285.6673 | 1285.6667 | 0.52 | 0 | 34 | 0.0068 | 1 |  | K.EDVFVHQTAIK.K |
|  | 2722 | 707.8874 | 1413.7602 | 1413.7616 | -0.98 | 1 | 76 | 3.3e-007 | 1 |  | K.EDVFVHQTAIKK.N |
|  | 2723 | 472.2618 | 1413.7634 | 1413.7616 | 1.28 | 1 | (26) | 0.036 | 1 |  | K.EDVFVHQTAIKK.N |
|  | 3200 | **780.4001** | **1558.7856** | **1558.7852** | **0.26** | **0** | **56** | **3.6e-005** | **1** | **U** | **K.DGVPEGAQLQGPVHR.N** |
|  | 3574 | 582.3003 | 1743.8792 | 1743.8792 | 0.03 | 1 | (72) | 7.6e-007 | 1 |  | R.NDTKEDVFVHQTAIK.K |
|  | 3575 | 872.9469 | 1743.8792 | 1743.8792 | 0.05 | 1 | 94 | 4.9e-009 | 1 |  | R.NDTKEDVFVHQTAIK.K |
|  | 3576 | 872.9469 | 1743.8792 | 1743.8792 | 0.05 | 1 | (94) | 5e-009 | 1 |  | R.NDTKEDVFVHQTAIK.K |
|  | 3577 | 436.9772 | 1743.8796 | 1743.8792 | 0.24 | 1 | (40) | 0.0015 | 1 |  | R.NDTKEDVFVHQTAIK.K |
|  | 3675 | **891.9335** | **1781.8525** | **1781.8544** | **-1.07** | **0** | **87** | **3e-008** | **1** | **U** | **K.GAEAANVTGPDGVPVEGSR.Y** |
|  | 3722 | 898.4153 | 1794.8161 | 1794.8160 | 0.08 | 0 | 39 | 0.0011 | 1 |  | R.SVGDGETVEFDVVEGEK.G |

  


---

|  |  |
| --- | --- |
| **19.** | sp|Q07666|KHDR1\_HUMAN    **Mass:** 48311    **Score:** 420    **Matches:** 23(16)  **Sequences:** 9(8)  **emPAI:** 1.36 |
|  | KH domain-containing, RNA-binding, signal transduction-associated protein 1 OS=Homo sapiens OX=9606 GN=KHDRBS1 PE=1 SV=1 |

|  |  |
| --- | --- |
|  | Check to include this hit in error tolerant search or archive report |
|  |  |

|  |  |  |  |  |  |  |  |  |  |  |  |
| --- | --- | --- | --- | --- | --- | --- | --- | --- | --- | --- | --- |
|  | **Query** | **Observed** | **Mr(expt)** | **Mr(calc)** | **ppm** | **Miss** | **Score** | **Expect** | **Rank** | **Unique** | **Peptide** |
|  | 11 | **356.1945** | **710.3744** | **710.3752** | **-1.04** | **0** | **(27)** | **0.017** | **1** |  | **K.FNFVGK.I** |
|  | 12 | **356.1948** | **710.3750** | **710.3752** | **-0.28** | **0** | **27** | **0.017** | **1** |  | **K.FNFVGK.I** |
|  | 142 | 398.2396 | 794.4647 | 794.4511 | 17.1 | 1 | 7 | 1.6 | 3 | U | R.GAPAPRAR.T |
|  | 603 | **460.2724** | **918.5302** | **918.5287** | **1.67** | **0** | **35** | **0.0031** | **1** | **U** | **R.GVPPPPTVR.G** |
|  | 1048 | **520.8086** | **1039.6027** | **1039.6026** | **0.16** | **0** | **43** | **0.00026** | **1** | **U** | **K.ILGPQGNTIK.R** |
|  | 1049 | **520.8088** | **1039.6031** | **1039.6026** | **0.52** | **0** | **(1)** | **3.6** | **3** | **U** | **K.ILGPQGNTIK.R** |
|  | 1050 | **520.8093** | **1039.6041** | **1039.6026** | **1.46** | **0** | **(2)** | **3.4** | **1** | **U** | **K.ILGPQGNTIK.R** |
|  | 1337 | **547.2842** | **1092.5538** | **1092.5525** | **1.21** | **0** | **29** | **0.019** | **1** |  | **K.YLPELMAEK.D** |
|  | 1338 | **547.2843** | **1092.5540** | **1092.5525** | **1.43** | **0** | **(16)** | **0.39** | **1** |  | **K.YLPELMAEK.D** |
|  | 1407 | **555.2810** | **1108.5475** | **1108.5474** | **0.05** | **0** | **(8)** | **2.2** | **1** |  | **K.YLPELMAEK.D + Oxidation (M)** |
|  | 1408 | **555.2812** | **1108.5478** | **1108.5474** | **0.38** | **0** | **(22)** | **0.084** | **1** |  | **K.YLPELMAEK.D + Oxidation (M)** |
|  | 1828 | **399.5750** | **1195.7032** | **1195.7037** | **-0.41** | **1** | **39** | **0.00051** | **1** | **U** | **K.ILGPQGNTIKR.L** |
|  | 1829 | **598.8591** | **1195.7037** | **1195.7037** | **0.02** | **1** | **(39)** | **0.00057** | **1** | **U** | **K.ILGPQGNTIKR.L** |
|  | 1830 | **399.5753** | **1195.7041** | **1195.7037** | **0.37** | **1** | **(21)** | **0.032** | **1** | **U** | **K.ILGPQGNTIKR.L** |
|  | 2589 | **462.2139** | **1383.6198** | **1383.6201** | **-0.24** | **0** | **(51)** | **6.5e-005** | **1** | **U** | **R.SGSMDPSGAHPSVR.Q** |
|  | 2590 | **692.8172** | **1383.6198** | **1383.6201** | **-0.19** | **0** | **(49)** | **0.0001** | **1** | **U** | **R.SGSMDPSGAHPSVR.Q** |
|  | 2591 | **462.2139** | **1383.6199** | **1383.6201** | **-0.18** | **0** | **(31)** | **0.0061** | **1** | **U** | **R.SGSMDPSGAHPSVR.Q** |
|  | 2592 | **692.8174** | **1383.6202** | **1383.6201** | **0.07** | **0** | **77** | **1.6e-007** | **1** | **U** | **R.SGSMDPSGAHPSVR.Q** |
|  | 3320 | **542.2426** | **1623.7058** | **1623.7053** | **0.35** | **0** | **(10)** | **0.55** | **1** | **U** | **K.DDEENYLDLFSHK.N** |
|  | 3321 | **812.8612** | **1623.7079** | **1623.7053** | **1.61** | **0** | **58** | **8.9e-006** | **1** | **U** | **K.DDEENYLDLFSHK.N** |
|  | 3599 | **876.9072** | **1751.7998** | **1751.8002** | **-0.24** | **1** | **96** | **2.4e-009** | **1** | **U** | **K.KDDEENYLDLFSHK.N** |
|  | 3600 | **438.9573** | **1751.8001** | **1751.8002** | **-0.05** | **1** | **(46)** | **0.00021** | **1** | **U** | **K.KDDEENYLDLFSHK.N** |
|  | 3601 | **584.9407** | **1751.8004** | **1751.8002** | **0.08** | **1** | **(51)** | **7e-005** | **1** | **U** | **K.KDDEENYLDLFSHK.N** |

  


---

|  |  |
| --- | --- |
| **20.** | sp|P05787|K2C8\_HUMAN    **Mass:** 53671    **Score:** 410    **Matches:** 26(18)  **Sequences:** 16(13)  **emPAI:** 2.10 |
|  | Keratin, type II cytoskeletal 8 OS=Homo sapiens OX=9606 GN=KRT8 PE=1 SV=7 |

|  |  |
| --- | --- |
|  | Check to include this hit in error tolerant search or archive report |
|  |  |

|  |  |  |  |  |  |  |  |  |  |  |  |
| --- | --- | --- | --- | --- | --- | --- | --- | --- | --- | --- | --- |
|  | **Query** | **Observed** | **Mr(expt)** | **Mr(calc)** | **ppm** | **Miss** | **Score** | **Expect** | **Rank** | **Unique** | **Peptide** |
|  | 236 | 414.2182 | 826.4219 | 826.4225 | -0.72 | 0 | 40 | 0.0012 | 1 |  | K.FASFIDK.V |
|  | 237 | 414.2184 | 826.4223 | 826.4225 | -0.28 | 0 | (38) | 0.0019 | 1 |  | K.FASFIDK.V |
|  | 898 | **500.7870** | **999.5594** | **999.5600** | **-0.60** | **0** | **45** | **0.00049** | **1** | **U** | **R.LQAEIEGLK.G** |
|  | 1257 | **540.2725** | **1078.5304** | **1078.5294** | **0.85** | **0** | **38** | **0.003** | **1** | **U** | **R.QLYEEEIR.E** |
|  | 1276 | 361.5378 | 1081.5916 | 1081.5920 | -0.39 | 1 | (29) | 0.014 | 1 |  | K.FASFIDKVR.F |
|  | 1277 | 541.8041 | 1081.5937 | 1081.5920 | 1.57 | 1 | 44 | 0.00038 | 1 |  | K.FASFIDKVR.F |
|  | 1507 | **565.3143** | **1128.6140** | **1128.6138** | **0.13** | **0** | **51** | **0.00016** | **1** | **U** | **K.LSELEAALQR.A** |
|  | 1616 | 577.2816 | 1152.5487 | 1152.5485 | 0.18 | 0 | 36 | 0.0034 | 1 |  | R.EYQELMNVK.L |
|  | 1693 | 585.2791 | 1168.5437 | 1168.5434 | 0.23 | 0 | (23) | 0.066 | 1 |  | R.EYQELMNVK.L + Oxidation (M) |
|  | 1713 | **587.3226** | **1172.6306** | **1172.6289** | **1.47** | **0** | **45** | **0.00059** | **1** | **U** | **K.LVSESSDVLPK.-** |
|  | 1714 | **587.3271** | **1172.6397** | **1172.6289** | **9.29** | **0** | **(5)** | **5.9** | **2** | **U** | **K.LVSESSDVLPK.-** |
|  | 1715 | **587.3272** | **1172.6399** | **1172.6289** | **9.39** | **0** | **(2)** | **11** | **3** | **U** | **K.LVSESSDVLPK.-** |
|  | 2348 | **660.8391** | **1319.6637** | **1319.6642** | **-0.44** | **0** | **(32)** | **0.011** | **1** | **U** | **R.SLDMDSIIAEVK.A** |
|  | 2392 | **668.8392** | **1335.6639** | **1335.6592** | **3.55** | **0** | **52** | **8.7e-005** | **1** | **U** | **R.SLDMDSIIAEVK.A + Oxidation (M)** |
|  | 2437 | **672.8413** | **1343.6681** | **1343.6681** | **0.01** | **0** | **70** | **1.4e-006** | **1** | **U** | **R.ASLEAAIADAEQR.G** |
|  | 2458 | **676.8442** | **1351.6738** | **1351.6693** | **3.32** | **0** | **32** | **0.011** | **1** | **U** | **R.TEMENEFVLIK.K** |
|  | 2502 | **679.8686** | **1357.7226** | **1357.7466** | **-17.67** | **1** | **3** | **6.6** | **8** | **U** | **K.WSLLQQQKTAR.S** |
|  | 2711 | **706.8456** | **1411.6766** | **1411.6765** | **0.07** | **1** | **38** | **0.0019** | **1** | **U** | **R.SRAEAESMYQIK.Y** |
|  | 2742 | **710.3787** | **1418.7428** | **1418.7405** | **1.58** | **0** | **22** | **0.1** | **1** | **U** | **R.LEGLTDEINFLR.Q** |
|  | 2789 | **476.8982** | **1427.6727** | **1427.6714** | **0.85** | **1** | **(18)** | **0.16** | **1** | **U** | **R.SRAEAESMYQIK.Y + Oxidation (M)** |
|  | 2952 | **738.3517** | **1474.6888** | **1474.6908** | **-1.36** | **0** | **74** | **3.7e-007** | **1** | **U** | **R.LESGMQNMSIHTK.T** |
|  | 2953 | **492.5712** | **1474.6919** | **1474.6908** | **0.71** | **0** | **(34)** | **0.0043** | **1** | **U** | **R.LESGMQNMSIHTK.T** |
|  | 2976 | **740.8892** | **1479.7639** | **1479.7643** | **-0.27** | **1** | **38** | **0.0022** | **1** | **U** | **R.TEMENEFVLIKK.D** |
|  | 2995 | **497.9021** | **1490.6845** | **1490.6857** | **-0.84** | **0** | **(39)** | **0.0011** | **1** | **U** | **R.LESGMQNMSIHTK.T + Oxidation (M)** |
|  | 2997 | **746.3511** | **1490.6877** | **1490.6857** | **1.34** | **0** | **(6)** | **2.3** | **2** | **U** | **R.LESGMQNMSIHTK.T + Oxidation (M)** |
|  | 3906 | **470.7101** | **1878.8113** | **1878.7876** | **12.6** | **0** | **10** | **0.46** | **1** | **U** | **R.SNMDNMFESYINNLR.R + 2 Oxidation (M)** |

  


---

|  |  |
| --- | --- |
| **21.** | sp|P04350|TBB4A\_HUMAN    **Mass:** 50010    **Score:** 391    **Matches:** 21(14)  **Sequences:** 10(7)  **emPAI:** 0.89 |
|  | Tubulin beta-4A chain OS=Homo sapiens OX=9606 GN=TUBB4A PE=1 SV=2 |

|  |  |
| --- | --- |
|  | Check to include this hit in error tolerant search or archive report |
|  |  |

|  |  |  |  |  |  |  |  |  |  |  |  |
| --- | --- | --- | --- | --- | --- | --- | --- | --- | --- | --- | --- |
|  | **Query** | **Observed** | **Mr(expt)** | **Mr(calc)** | **ppm** | **Miss** | **Score** | **Expect** | **Rank** | **Unique** | **Peptide** |
|  | 997 | 514.7636 | 1027.5125 | 1027.5121 | 0.47 | 0 | 37 | 0.0027 | 1 |  | K.TAVCDIPPR.G |
|  | 998 | 514.7717 | 1027.5289 | 1027.5121 | 16.4 | 0 | (6) | 3.9 | 3 |  | K.TAVCDIPPR.G |
|  | 1000 | 514.7734 | 1027.5323 | 1027.5121 | 19.7 | 0 | (15) | 0.41 | 1 |  | K.TAVCDIPPR.G |
|  | 1045 | 520.2997 | 1038.5849 | 1038.5862 | -1.24 | 0 | 21 | 0.061 | 1 |  | R.YLTVAAVFR.G |
|  | 1046 | 520.3008 | 1038.5870 | 1038.5862 | 0.77 | 0 | (12) | 0.54 | 1 |  | R.YLTVAAVFR.G |
|  | 1509 | 565.8023 | 1129.5901 | 1129.5880 | 1.83 | 0 | 53 | 8.7e-005 | 1 |  | R.FPGQLNADLR.K |
|  | 1510 | 565.8048 | 1129.5949 | 1129.5880 | 6.15 | 0 | (38) | 0.0036 | 1 |  | R.FPGQLNADLR.K |
|  | 1583 | 572.3204 | 1142.6263 | 1142.6270 | -0.63 | 0 | 51 | 0.00014 | 1 |  | K.LAVNMVPFPR.L |
|  | 1584 | 572.3210 | 1142.6275 | 1142.6270 | 0.44 | 0 | (30) | 0.018 | 1 |  | K.LAVNMVPFPR.L |
|  | 1641 | 580.3174 | 1158.6203 | 1158.6219 | -1.40 | 0 | (25) | 0.065 | 1 |  | K.LAVNMVPFPR.L + Oxidation (M) |
|  | 1642 | 580.3181 | 1158.6217 | 1158.6219 | -0.22 | 0 | (29) | 0.029 | 1 |  | K.LAVNMVPFPR.L + Oxidation (M) |
|  | 1964 | 615.3022 | 1228.5899 | 1228.5910 | -0.88 | 0 | 43 | 0.00057 | 1 |  | R.ISEQFTAMFR.R |
|  | 2133 | 636.3674 | 1270.7203 | 1270.7220 | -1.32 | 1 | 50 | 9.7e-005 | 1 |  | R.KLAVNMVPFPR.L |
|  | 2339 | 660.3550 | 1318.6955 | 1318.6955 | 0.01 | 0 | (52) | 0.0001 | 1 |  | R.IMNTFSVVPSPK.V |
|  | 2340 | 660.3560 | 1318.6974 | 1318.6955 | 1.41 | 0 | (45) | 0.00051 | 1 |  | R.IMNTFSVVPSPK.V |
|  | 2389 | 668.3522 | 1334.6899 | 1334.6904 | -0.39 | 0 | 56 | 4.1e-005 | 1 |  | R.IMNTFSVVPSPK.V + Oxidation (M) |
|  | 2390 | 668.3546 | 1334.6945 | 1334.6904 | 3.09 | 0 | (34) | 0.0061 | 1 |  | R.IMNTFSVVPSPK.V + Oxidation (M) |
|  | 3471 | 848.9187 | 1695.8228 | 1695.8257 | -1.65 | 0 | 4 | 6 | 2 |  | K.NSSYFVEWIPNNVK.T |
|  | 3778 | 911.9653 | 1821.9160 | 1821.9156 | 0.24 | 0 | 80 | 1.4e-007 | 1 |  | R.EIVHLQAGQCGNQIGAK.F |
|  | 3779 | 608.3131 | 1821.9175 | 1821.9156 | 1.07 | 0 | (68) | 2e-006 | 1 |  | R.EIVHLQAGQCGNQIGAK.F |
|  | 3952 | **951.4263** | **1900.8381** | **1900.8580** | **-10.47** | **1** | **0** | **5.8** | **2** | **U** | **R.MSMKEVDEQMLSVQSK.N + 2 Oxidation (M)** |

  


---

|  |  |
| --- | --- |
| **22.** | sp|O00571|DDX3X\_HUMAN    **Mass:** 73597    **Score:** 371    **Matches:** 20(15)  **Sequences:** 15(10)  **emPAI:** 0.92 |
|  | ATP-dependent RNA helicase DDX3X OS=Homo sapiens OX=9606 GN=DDX3X PE=1 SV=3 |

|  |  |
| --- | --- |
|  | Check to include this hit in error tolerant search or archive report |
|  |  |

|  |  |  |  |  |  |  |  |  |  |  |  |
| --- | --- | --- | --- | --- | --- | --- | --- | --- | --- | --- | --- |
|  | **Query** | **Observed** | **Mr(expt)** | **Mr(calc)** | **ppm** | **Miss** | **Score** | **Expect** | **Rank** | **Unique** | **Peptide** |
|  | 140 | **396.2605** | **790.5064** | **790.5065** | **-0.05** | **0** | **31** | **0.0013** | **1** | **U** | **K.HAIPIIK.E** |
|  | 145 | **399.6980** | **797.3815** | **797.3821** | **-0.68** | **0** | **19** | **0.1** | **1** | **U** | **R.FSGGFGAR.D** |
|  | 375 | **426.7178** | **851.4211** | **851.4211** | **-0.02** | **0** | **33** | **0.0076** | **1** | **U** | **K.IGLDFCK.Y** |
|  | 1334 | **547.2789** | **1092.5433** | **1092.5451** | **-1.66** | **0** | **42** | **0.0011** | **1** |  | **K.YLVLDEADR.M** |
|  | 1687 | **584.8560** | **1167.6975** | **1167.6975** | **-0.04** | **0** | **(48)** | **5.9e-005** | **1** | **U** | **K.SPILVATAVAAR.G** |
|  | 1688 | **584.8568** | **1167.6989** | **1167.6975** | **1.21** | **0** | **49** | **4.1e-005** | **1** | **U** | **K.SPILVATAVAAR.G** |
|  | 1738 | **394.2133** | **1179.6181** | **1179.6183** | **-0.12** | **0** | **60** | **1.5e-005** | **1** | **U** | **R.GCHLLVATPGR.L** |
|  | 1739 | **590.8165** | **1179.6185** | **1179.6183** | **0.20** | **0** | **(49)** | **0.00021** | **1** | **U** | **R.GCHLLVATPGR.L** |
|  | 2034 | **416.2183** | **1245.6330** | **1245.6353** | **-1.85** | **1** | **22** | **0.13** | **1** | **U** | **K.VVWVEESDKR.S** |
|  | 2208 | **431.2384** | **1290.6933** | **1290.7183** | **-19.41** | **0** | **8** | **2.1** | **7** | **U** | **R.SFLLDLLNATGK.D** |
|  | 2231 | **649.3072** | **1296.5999** | **1296.5995** | **0.34** | **0** | **24** | **0.04** | **1** | **U** | **R.HTMMFSATFPK.E** |
|  | 2249 | **650.8233** | **1299.6320** | **1299.6320** | **0.06** | **1** | **50** | **0.00015** | **1** | **U** | **R.DREEALHQFR.S** |
|  | 2250 | **434.2180** | **1299.6322** | **1299.6320** | **0.20** | **1** | **(32)** | **0.0079** | **1** | **U** | **R.DREEALHQFR.S** |
|  | 2391 | **668.8235** | **1335.6325** | **1335.6315** | **0.77** | **0** | **44** | **0.0005** | **1** |  | **R.MLDMGFEPQIR.R** |
|  | 2457 | **676.8214** | **1351.6281** | **1351.6264** | **1.27** | **0** | **(38)** | **0.0017** | **1** |  | **R.MLDMGFEPQIR.R + Oxidation (M)** |
|  | 2486 | **679.3950** | **1356.7754** | **1356.7765** | **-0.85** | **0** | **71** | **5.1e-007** | **1** | **U** | **K.QYPISLVLAPTR.E** |
|  | 2986 | **495.9644** | **1484.8713** | **1484.8715** | **-0.15** | **1** | **17** | **0.072** | **1** | **U** | **R.KQYPISLVLAPTR.E** |
|  | 3090 | **762.8901** | **1523.7656** | **1523.7732** | **-5.01** | **0** | **15** | **0.45** | **1** | **U** | **R.VGNLGLATSFFNER.N** |
|  | 3920 | **944.4960** | **1886.9774** | **1886.9785** | **-0.60** | **0** | **(23)** | **0.057** | **1** | **U** | **R.VRPCVVYGGADIGQQIR.D** |
|  | 3921 | **629.9998** | **1886.9776** | **1886.9785** | **-0.47** | **0** | **60** | **1.3e-005** | **1** | **U** | **R.VRPCVVYGGADIGQQIR.D** |

  


---

|  |  |
| --- | --- |
| **23.** | sp|P05783|K1C18\_HUMAN    **Mass:** 48029    **Score:** 351    **Matches:** 17(14)  **Sequences:** 14(11)  **emPAI:** 1.22 |
|  | Keratin, type I cytoskeletal 18 OS=Homo sapiens OX=9606 GN=KRT18 PE=1 SV=2 |

|  |  |
| --- | --- |
|  | Check to include this hit in error tolerant search or archive report |
|  |  |

|  |  |  |  |  |  |  |  |  |  |  |  |
| --- | --- | --- | --- | --- | --- | --- | --- | --- | --- | --- | --- |
|  | **Query** | **Observed** | **Mr(expt)** | **Mr(calc)** | **ppm** | **Miss** | **Score** | **Expect** | **Rank** | **Unique** | **Peptide** |
|  | 19 | **359.6992** | **717.3838** | **717.3843** | **-0.69** | **0** | **15** | **0.73** | **1** | **U** | **K.IMADIR.A** |
|  | 179 | 404.2029 | 806.3913 | 806.3923 | -1.23 | 0 | 41 | 0.0016 | 1 |  | R.LAADDFR.V |
|  | 180 | 404.2037 | 806.3928 | 806.3923 | 0.73 | 0 | (26) | 0.044 | 1 |  | R.LAADDFR.V |
|  | 765 | **483.2379** | **964.4613** | **964.4614** | **-0.10** | **0** | **29** | **0.019** | **1** | **U** | **R.AQYDELAR.K** |
|  | 799 | **488.2298** | **974.4450** | **974.4458** | **-0.79** | **0** | **34** | **0.0041** | **1** | **U** | **R.STFSTNYR.S** |
|  | 819 | **491.7247** | **981.4349** | **981.4345** | **0.45** | **0** | **20** | **0.075** | **1** | **U** | **R.DWSHYFK.I** |
|  | 1051 | **521.3065** | **1040.5984** | **1040.5978** | **0.52** | **0** | **50** | **7.3e-005** | **1** |  | **R.IVLQIDNAR.L** |
|  | 1201 | **533.2848** | **1064.5550** | **1064.5502** | **4.55** | **0** | **50** | **0.00017** | **1** | **U** | **K.LEAEIATYR.R** |
|  | 1718 | **587.8248** | **1173.6350** | **1173.6353** | **-0.30** | **1** | **6** | **4.7** | **3** | **U** | **R.KVIDDTNITR.L** |
|  | 2006 | **620.3242** | **1238.6338** | **1238.6329** | **0.72** | **1** | **36** | **0.0037** | **1** | **U** | **R.VKYETELAMR.Q** |
|  | 2117 | **423.2173** | **1266.6300** | **1266.6317** | **-1.27** | **0** | **41** | **0.0011** | **1** | **U** | **R.QSVENDIHGLR.K** |
|  | 2212 | **646.8642** | **1291.7138** | **1291.7136** | **0.22** | **1** | **(44)** | **0.00046** | **1** | **U** | **K.VKLEAEIATYR.R** |
|  | 2213 | **646.8645** | **1291.7144** | **1291.7136** | **0.69** | **1** | **48** | **0.00013** | **1** | **U** | **K.VKLEAEIATYR.R** |
|  | 2338 | **660.3385** | **1318.6624** | **1318.6629** | **-0.38** | **0** | **60** | **1.7e-005** | **1** | **U** | **R.AQIFANTVDNAR.I** |
|  | 2658 | **698.3694** | **1394.7242** | **1394.7266** | **-1.72** | **1** | **68** | **2.3e-006** | **1** | **U** | **R.QSVENDIHGLRK.V** |
|  | 2659 | **465.9164** | **1394.7273** | **1394.7266** | **0.50** | **1** | **(46)** | **0.00032** | **1** | **U** | **R.QSVENDIHGLRK.V** |
|  | 2741 | **710.3768** | **1418.7391** | **1418.7405** | **-0.98** | **0** | **64** | **6.4e-006** | **1** | **U** | **R.QAQEYEALLNIK.V** |

  


---

|  |  |
| --- | --- |
| **24.** | sp|Q92804|RBP56\_HUMAN    **Mass:** 62021    **Score:** 346    **Matches:** 16(14)  **Sequences:** 6(6)  **emPAI:** 0.86 |
|  | TATA-binding protein-associated factor 2N OS=Homo sapiens OX=9606 GN=TAF15 PE=1 SV=1 |

|  |  |
| --- | --- |
|  | Check to include this hit in error tolerant search or archive report |
|  |  |

|  |  |  |  |  |  |  |  |  |  |  |  |
| --- | --- | --- | --- | --- | --- | --- | --- | --- | --- | --- | --- |
|  | **Query** | **Observed** | **Mr(expt)** | **Mr(calc)** | **ppm** | **Miss** | **Score** | **Expect** | **Rank** | **Unique** | **Peptide** |
|  | 733 | **479.2613** | **956.5080** | **956.5080** | **0.02** | **0** | **24** | **0.054** | **1** | **U** | **K.EFHGNIIK.V** |
|  | 972 | **511.7507** | **1021.4868** | **1021.4869** | **-0.04** | **0** | **45** | **0.00039** | **1** |  | **K.AAIDWFDGK.E** |
|  | 973 | **511.7511** | **1021.4876** | **1021.4869** | **0.74** | **0** | **(42)** | **0.00094** | **1** |  | **K.AAIDWFDGK.E** |
|  | 2744 | **710.8338** | **1419.6530** | **1419.6518** | **0.88** | **0** | **53** | **4.5e-005** | **1** |  | **K.GEATVSFDDPPSAK.A** |
|  | 2745 | **710.8339** | **1419.6533** | **1419.6518** | **1.05** | **0** | **(51)** | **7.1e-005** | **1** |  | **K.GEATVSFDDPPSAK.A** |
|  | 4087 | **502.5186** | **2006.0455** | **2006.0506** | **-2.57** | **1** | **67** | **2e-006** | **1** | **U** | **K.TGKPMINLYTDKDTGKPK.G** |
|  | 4088 | **669.6899** | **2006.0478** | **2006.0506** | **-1.41** | **1** | **(44)** | **0.00036** | **1** | **U** | **K.TGKPMINLYTDKDTGKPK.G** |
|  | 4089 | **402.2174** | **2006.0505** | **2006.0506** | **-0.06** | **1** | **(56)** | **2.2e-005** | **1** | **U** | **K.TGKPMINLYTDKDTGKPK.G** |
|  | 4131 | **405.4160** | **2022.0439** | **2022.0456** | **-0.84** | **1** | **(52)** | **5.5e-005** | **1** | **U** | **K.TGKPMINLYTDKDTGKPK.G + Oxidation (M)** |
|  | 4132 | **506.5184** | **2022.0446** | **2022.0456** | **-0.47** | **1** | **(22)** | **0.062** | **1** | **U** | **K.TGKPMINLYTDKDTGKPK.G + Oxidation (M)** |
|  | 4133 | **675.0222** | **2022.0447** | **2022.0456** | **-0.45** | **1** | **(28)** | **0.015** | **1** | **U** | **K.TGKPMINLYTDKDTGKPK.G + Oxidation (M)** |
|  | 4134 | **506.5189** | **2022.0465** | **2022.0456** | **0.44** | **1** | **(34)** | **0.0041** | **1** | **U** | **K.TGKPMINLYTDKDTGKPK.G + Oxidation (M)** |
|  | 4192 | **1034.9238** | **2067.8331** | **2067.8350** | **-0.90** | **0** | **20** | **0.013** | **1** | **U** | **K.SGDWVCPNPSCGNMNFAR.R** |
|  | 4203 | **1042.9232** | **2083.8319** | **2083.8299** | **0.96** | **0** | **(20)** | **0.013** | **1** | **U** | **K.SGDWVCPNPSCGNMNFAR.R + Oxidation (M)** |
|  | 4659 | **992.4247** | **2974.2524** | **2974.2496** | **0.94** | **0** | **65** | **5.3e-007** | **1** | **U** | **K.QSSYSQQPYNNQGQQQNMESSGSQGGR.A** |
|  | 4663 | **997.7554** | **2990.2443** | **2990.2445** | **-0.08** | **0** | **(3)** | **0.54** | **2** | **U** | **K.QSSYSQQPYNNQGQQQNMESSGSQGGR.A + Oxidation (M)** |

  


---

|  |  |
| --- | --- |
| **25.** | sp|P31943|HNRH1\_HUMAN    **Mass:** 49484    **Score:** 332    **Matches:** 15(10)  **Sequences:** 11(7)  **emPAI:** 0.67 |
|  | Heterogeneous nuclear ribonucleoprotein H OS=Homo sapiens OX=9606 GN=HNRNPH1 PE=1 SV=4 |

|  |  |
| --- | --- |
|  | Check to include this hit in error tolerant search or archive report |
|  |  |

|  |  |  |  |  |  |  |  |  |  |  |  |
| --- | --- | --- | --- | --- | --- | --- | --- | --- | --- | --- | --- |
|  | **Query** | **Observed** | **Mr(expt)** | **Mr(calc)** | **ppm** | **Miss** | **Score** | **Expect** | **Rank** | **Unique** | **Peptide** |
|  | 118 | **392.7156** | **783.4167** | **783.4167** | **0.05** | **0** | **13** | **0.35** | **1** |  | **R.YVEVFK.S** |
|  | 190 | **406.7313** | **811.4480** | **811.4480** | **0.07** | **0** | **13** | **0.36** | **1** |  | **R.YIEIFK.S** |
|  | 408 | **433.2155** | **864.4164** | **864.4164** | **0.03** | **0** | **13** | **0.64** | **1** |  | **R.GLPFGCSK.E** |
|  | 1321 | **546.7930** | **1091.5714** | **1091.5724** | **-0.89** | **0** | **(41)** | **0.0013** | **1** |  | **R.VHIEIGPDGR.V** |
|  | 1322 | **546.7931** | **1091.5717** | **1091.5724** | **-0.56** | **0** | **(38)** | **0.0025** | **1** |  | **R.VHIEIGPDGR.V** |
|  | 1323 | **364.8646** | **1091.5719** | **1091.5724** | **-0.46** | **0** | **49** | **0.00021** | **1** |  | **R.VHIEIGPDGR.V** |
|  | 1324 | **364.8646** | **1091.5721** | **1091.5724** | **-0.22** | **0** | **(40)** | **0.0014** | **1** |  | **R.VHIEIGPDGR.V** |
|  | 2385 | **667.8237** | **1333.6329** | **1333.6336** | **-0.53** | **0** | **54** | **6.4e-005** | **1** | **U** | **K.SNNVEMDWVLK.H** |
|  | 3041 | **752.8461** | **1503.6777** | **1503.6776** | **0.05** | **0** | **64** | **3e-006** | **1** | **U** | **R.GLPWSCSADEVQR.F** |
|  | 3280 | **801.3275** | **1600.6405** | **1600.6399** | **0.38** | **0** | **61** | **1.5e-006** | **1** |  | **R.DLNYCFSGMSDHR.Y** |
|  | 3442 | **562.2592** | **1683.7557** | **1683.7601** | **-2.64** | **0** | **64** | **2.9e-006** | **1** |  | **K.HTGPNSPDTANDGFVR.L** |
|  | 3443 | **842.8865** | **1683.7585** | **1683.7601** | **-0.95** | **0** | **(14)** | **0.3** | **1** |  | **K.HTGPNSPDTANDGFVR.L** |
|  | 3571 | **581.3025** | **1740.8856** | **1740.8539** | **18.2** | **1** | **5** | **4.1** | **1** | **U** | **-.MMLGTEGGEGFVVKVR.G + 2 Oxidation (M)** |
|  | 3809 | **921.4487** | **1840.8828** | **1840.8843** | **-0.82** | **0** | **29** | **0.014** | **1** |  | **R.STGEAFVQFASQEIAEK.A** |
|  | 4223 | **699.9661** | **2096.8764** | **2096.8793** | **-1.41** | **0** | **74** | **8.2e-008** | **1** | **U** | **R.YGDGGSTFQSTTGHCVHMR.G** |

  


---

|  |  |
| --- | --- |
| **26.** | sp|P08670|VIME\_HUMAN    **Mass:** 53676    **Score:** 326    **Matches:** 14(12)  **Sequences:** 13(11)  **emPAI:** 1.04 |
|  | Vimentin OS=Homo sapiens OX=9606 GN=VIM PE=1 SV=4 |

|  |  |
| --- | --- |
|  | Check to include this hit in error tolerant search or archive report |
|  |  |

|  |  |  |  |  |  |  |  |  |  |  |  |
| --- | --- | --- | --- | --- | --- | --- | --- | --- | --- | --- | --- |
|  | **Query** | **Observed** | **Mr(expt)** | **Mr(calc)** | **ppm** | **Miss** | **Score** | **Expect** | **Rank** | **Unique** | **Peptide** |
|  | 420 | **435.7233** | **869.4321** | **869.4283** | **4.38** | **0** | **4** | **4.5** | **3** | **U** | **R.FANYIDK.V** |
|  | 1250 | **538.7557** | **1075.4968** | **1075.4968** | **0.03** | **0** | **35** | **0.0031** | **1** | **U** | **R.DNLAEDIMR.L** |
|  | 1333 | **547.2691** | **1092.5236** | **1092.5200** | **3.38** | **0** | **49** | **0.0002** | **1** | **U** | **K.FADLSEAANR.N** |
|  | 1444 | **558.2887** | **1114.5628** | **1114.5618** | **0.91** | **0** | **45** | **0.00044** | **1** |  | **K.VELQELNDR.F** |
|  | 1468 | **561.2958** | **1120.5770** | **1120.5764** | **0.53** | **0** | **30** | **0.016** | **1** |  | **R.EYQDLLNVK.M** |
|  | 1694 | **585.3607** | **1168.7068** | **1168.7067** | **0.08** | **0** | **28** | **0.0058** | **1** | **U** | **K.ILLAELEQLK.G** |
|  | 2061 | **627.7864** | **1253.5583** | **1253.5598** | **-1.15** | **0** | **54** | **3.7e-005** | **1** | **U** | **R.LGDLYEEEMR.E** |
|  | 2286 | **655.3060** | **1308.5974** | **1308.5986** | **-0.92** | **0** | **40** | **0.00096** | **1** | **U** | **K.NLQEAEEWYK.S** |
|  | 2354 | **662.3119** | **1322.6092** | **1322.6102** | **-0.76** | **0** | **61** | **8e-006** | **1** | **U** | **R.EEAENTLQSFR.Q** |
|  | 2794 | **714.8631** | **1427.7116** | **1427.7045** | **5.02** | **0** | **58** | **1.9e-005** | **1** | **U** | **R.SLYASSPGGVYATR.S** |
|  | 2994 | **745.8801** | **1489.7457** | **1489.7446** | **0.71** | **0** | **50** | **0.00017** | **1** | **U** | **R.QVQSLTCEVDALK.G** |
|  | 3023 | **499.2670** | **1494.7793** | **1494.7790** | **0.15** | **0** | **11** | **1.1** | **1** | **U** | **R.TYSLGSALRPSTSR.S** |
|  | 3262 | **529.9375** | **1586.7907** | **1586.7900** | **0.44** | **1** | **(59)** | **1.9e-005** | **1** |  | **R.TNEKVELQELNDR.F** |
|  | 3263 | **794.4044** | **1586.7943** | **1586.7900** | **2.72** | **1** | **67** | **3.1e-006** | **1** |  | **R.TNEKVELQELNDR.F** |

  


---

|  |  |
| --- | --- |
| **27.** | sp|P02768|ALBU\_HUMAN    **Mass:** 71317    **Score:** 302    **Matches:** 17(9)  **Sequences:** 12(6)  **emPAI:** 0.57 |
|  | Serum albumin OS=Homo sapiens OX=9606 GN=ALB PE=1 SV=2 |

|  |  |
| --- | --- |
|  | Check to include this hit in error tolerant search or archive report |
|  |  |

|  |  |  |  |  |  |  |  |  |  |  |  |
| --- | --- | --- | --- | --- | --- | --- | --- | --- | --- | --- | --- |
|  | **Query** | **Observed** | **Mr(expt)** | **Mr(calc)** | **ppm** | **Miss** | **Score** | **Expect** | **Rank** | **Unique** | **Peptide** |
|  | 136 | **395.2394** | **788.4643** | **788.4644** | **-0.05** | **0** | **32** | **0.0069** | **1** | **U** | **K.LVTDLTK.V** |
|  | 486 | **440.7329** | **879.4513** | **879.4338** | **20.0** | **0** | **0** | **15** | **1** | **U** | **K.AEFAEVSK.L** |
|  | 617 | **464.2504** | **926.4862** | **926.4861** | **0.06** | **0** | **20** | **0.12** | **1** | **U** | **K.YLYEIAR.R** |
|  | 618 | **464.2508** | **926.4870** | **926.4861** | **0.90** | **0** | **(7)** | **2.6** | **1** | **U** | **K.YLYEIAR.R** |
|  | 677 | 470.7357 | 939.4568 | 939.4410 | 16.9 | 0 | 6 | 3.4 | 3 | U | K.DDNPNLPR.L |
|  | 735 | **480.7848** | **959.5550** | **959.5552** | **-0.22** | **0** | **20** | **0.11** | **1** | **U** | **K.FQNALLVR.Y** |
|  | 899 | **500.8054** | **999.5962** | **999.5964** | **-0.21** | **0** | **38** | **0.0017** | **1** | **U** | **K.QTALVELVK.H** |
|  | 937 | **507.3029** | **1012.5912** | **1012.5917** | **-0.44** | **0** | **13** | **0.44** | **1** | **U** | **K.LVAASQAALGL.-** |
|  | 1549 | **569.7526** | **1137.4906** | **1137.4907** | **-0.10** | **0** | **59** | **6.9e-006** | **1** | **U** | **K.CCTESLVNR.R** |
|  | 1601 | **575.3115** | **1148.6084** | **1148.6077** | **0.56** | **0** | **52** | **0.00011** | **1** | **U** | **K.LVNEVTEFAK.T** |
|  | 2869 | **722.3230** | **1442.6314** | **1442.6347** | **-2.28** | **0** | **(12)** | **0.36** | **1** | **U** | **K.YICENQDSISSK.L** |
|  | 2870 | **722.3245** | **1442.6344** | **1442.6347** | **-0.25** | **0** | **20** | **0.064** | **1** | **U** | **K.YICENQDSISSK.L** |
|  | 3057 | **756.4261** | **1510.8376** | **1510.8355** | **1.39** | **0** | **40** | **0.00084** | **1** | **U** | **K.VPQVSTPTLVEVSR.N** |
|  | 3359 | **547.3175** | **1638.9307** | **1638.9305** | **0.12** | **1** | **(51)** | **2.8e-005** | **1** | **U** | **K.KVPQVSTPTLVEVSR.N** |
|  | 3360 | **547.3176** | **1638.9309** | **1638.9305** | **0.24** | **1** | **66** | **9.1e-007** | **1** | **U** | **K.KVPQVSTPTLVEVSR.N** |
|  | 3361 | **820.4728** | **1638.9310** | **1638.9305** | **0.32** | **1** | **(63)** | **1.8e-006** | **1** | **U** | **K.KVPQVSTPTLVEVSR.N** |
|  | 3362 | **820.4739** | **1638.9333** | **1638.9305** | **1.74** | **1** | **(56)** | **1e-005** | **1** | **U** | **K.KVPQVSTPTLVEVSR.N** |

  


---

|  |  |
| --- | --- |
| **28.** | sp|P19012|K1C15\_HUMAN    **Mass:** 49409    **Score:** 286    **Matches:** 15(12)  **Sequences:** 9(7)  **emPAI:** 0.79 |
|  | Keratin, type I cytoskeletal 15 OS=Homo sapiens OX=9606 GN=KRT15 PE=1 SV=3 |

|  |  |
| --- | --- |
|  | Check to include this hit in error tolerant search or archive report |
|  |  |

|  |  |  |  |  |  |  |  |  |  |  |  |
| --- | --- | --- | --- | --- | --- | --- | --- | --- | --- | --- | --- |
|  | **Query** | **Observed** | **Mr(expt)** | **Mr(calc)** | **ppm** | **Miss** | **Score** | **Expect** | **Rank** | **Unique** | **Peptide** |
|  | 179 | 404.2029 | 806.3913 | 806.3923 | -1.23 | 0 | 41 | 0.0016 | 1 |  | R.LAADDFR.L |
|  | 180 | 404.2037 | 806.3928 | 806.3923 | 0.73 | 0 | (26) | 0.044 | 1 |  | R.LAADDFR.L |
|  | 182 | 405.2236 | 808.4326 | 808.4330 | -0.49 | 0 | (30) | 0.013 | 1 |  | R.LASYLDK.V |
|  | 183 | 405.2238 | 808.4329 | 808.4330 | -0.12 | 0 | 40 | 0.0016 | 1 |  | R.LASYLDK.V |
|  | 1006 | 515.3003 | 1028.5860 | 1028.5866 | -0.55 | 0 | 44 | 0.00051 | 1 |  | R.VLDELTLAR.T |
|  | 1007 | 515.3008 | 1028.5871 | 1028.5866 | 0.52 | 0 | (33) | 0.0058 | 1 |  | R.VLDELTLAR.T |
|  | 1197 | 355.5417 | 1063.6032 | 1063.6026 | 0.63 | 1 | (41) | 0.00067 | 1 |  | R.LASYLDKVR.A |
|  | 1198 | 532.8090 | 1063.6034 | 1063.6026 | 0.75 | 1 | 47 | 0.00017 | 1 |  | R.LASYLDKVR.A |
|  | 1384 | **552.7781** | **1103.5416** | **1103.5393** | **2.07** | **0** | **7** | **3.6** | **1** |  | **K.ITMQNLNDR.L** |
|  | 1473 | 561.7929 | 1121.5713 | 1121.5717 | -0.35 | 0 | 45 | 0.00066 | 1 |  | R.LEQEIATYR.S |
|  | 1774 | 395.9029 | 1184.6868 | 1184.6877 | -0.72 | 1 | (2) | 6 | 2 |  | R.RVLDELTLAR.T |
|  | 1775 | 395.9031 | 1184.6873 | 1184.6877 | -0.32 | 1 | 4 | 3.6 | 2 |  | R.RVLDELTLAR.T |
|  | 2255 | 651.3339 | 1300.6532 | 1300.6510 | 1.65 | 0 | 73 | 8.3e-007 | 1 |  | R.ALEEANADLEVK.I |
|  | 2566 | 460.5807 | 1378.7202 | 1378.7204 | -0.21 | 1 | 60 | 1.7e-005 | 1 |  | K.TRLEQEIATYR.S |
|  | 2567 | 690.3676 | 1378.7207 | 1378.7204 | 0.16 | 1 | (51) | 0.00011 | 1 |  | K.TRLEQEIATYR.S |

  


---

|  |  |
| --- | --- |
| **29.** | sp|Q96PK6|RBM14\_HUMAN    **Mass:** 69620    **Score:** 275    **Matches:** 17(12)  **Sequences:** 16(11)  **emPAI:** 0.91 |
|  | RNA-binding protein 14 OS=Homo sapiens OX=9606 GN=RBM14 PE=1 SV=2 |

|  |  |
| --- | --- |
|  | Check to include this hit in error tolerant search or archive report |
|  |  |

|  |  |  |  |  |  |  |  |  |  |  |  |
| --- | --- | --- | --- | --- | --- | --- | --- | --- | --- | --- | --- |
|  | **Query** | **Observed** | **Mr(expt)** | **Mr(calc)** | **ppm** | **Miss** | **Score** | **Expect** | **Rank** | **Unique** | **Peptide** |
|  | 558 | **452.2611** | **902.5077** | **902.5073** | **0.43** | **0** | **38** | **0.0023** | **1** | **U** | **R.INVELSTK.G** |
|  | 748 | **481.2552** | **960.4958** | **960.4950** | **0.83** | **0** | **24** | **0.096** | **1** | **U** | **R.VIECDVVK.D** |
|  | 767 | **483.7499** | **965.4853** | **965.4818** | **3.61** | **0** | **34** | **0.0062** | **1** | **U** | **R.LAELSDYR.R** |
|  | 1032 | **518.2629** | **1034.5113** | **1034.5120** | **-0.66** | **0** | **20** | **0.15** | **1** | **U** | **K.QFAFVHMR.E** |
|  | 1116 | **523.7726** | **1045.5306** | **1045.5345** | **-3.75** | **0** | **31** | **0.016** | **1** | **U** | **R.QPTPPFFGR.D** |
|  | 1215 | **533.7803** | **1065.5460** | **1065.5455** | **0.50** | **0** | **47** | **0.00027** | **1** | **U** | **R.LSESQLSFR.R** |
|  | 1474 | **374.8679** | **1121.5819** | **1121.5829** | **-0.85** | **1** | **(30)** | **0.018** | **1** | **U** | **R.LAELSDYRR.L** |
|  | 1475 | **561.7990** | **1121.5835** | **1121.5829** | **0.52** | **1** | **30** | **0.017** | **1** | **U** | **R.LAELSDYRR.L** |
|  | 1554 | **570.2637** | **1138.5129** | **1138.5117** | **1.04** | **0** | **22** | **0.055** | **1** | **U** | **K.DYAFVHMEK.E** |
|  | 1587 | **382.1841** | **1143.5303** | **1143.5309** | **-0.48** | **0** | **8** | **1.4** | **1** | **U** | **R.LPDAHSDYAR.Y** |
|  | 1873 | **603.3180** | **1204.6215** | **1204.6200** | **1.26** | **0** | **40** | **0.0019** | **1** | **U** | **R.AQPSASLGVGYR.T** |
|  | 1922 | **610.3289** | **1218.6433** | **1218.6357** | **6.25** | **0** | **41** | **0.0014** | **1** | **U** | **R.AQPSVSLGAAYR.A** |
|  | 2033 | **623.3329** | **1244.6512** | **1244.6513** | **-0.07** | **0** | **46** | **0.00045** | **1** | **U** | **R.AQPSVSLGAPYR.G** |
|  | 2251 | **434.2181** | **1299.6325** | **1299.6320** | **0.41** | **1** | **20** | **0.14** | **1** | **U** | **R.RLPDAHSDYAR.Y** |
|  | 2361 | **662.8187** | **1323.6229** | **1323.6241** | **-0.91** | **0** | **12** | **0.76** | **1** | **U** | **R.TQPMTAQAASYR.A** |
|  | 3289 | **804.9225** | **1607.8304** | **1607.8307** | **-0.19** | **0** | **43** | **0.00078** | **1** | **U** | **R.ASYVAPLTAQPATYR.A** |
|  | 3591 | **876.4431** | **1750.8717** | **1750.8672** | **2.55** | **0** | **95** | **3.7e-009** | **1** | **U** | **K.IFVGNVSAACTSQELR.S** |

  


---

|  |  |
| --- | --- |
| **30.** | sp|P13646|K1C13\_HUMAN    **Mass:** 49900    **Score:** 271    **Matches:** 16(11)  **Sequences:** 8(6)  **emPAI:** 0.67 |
|  | Keratin, type I cytoskeletal 13 OS=Homo sapiens OX=9606 GN=KRT13 PE=1 SV=4 |

|  |  |
| --- | --- |
|  | Check to include this hit in error tolerant search or archive report |
|  |  |

|  |  |  |  |  |  |  |  |  |  |  |  |
| --- | --- | --- | --- | --- | --- | --- | --- | --- | --- | --- | --- |
|  | **Query** | **Observed** | **Mr(expt)** | **Mr(calc)** | **ppm** | **Miss** | **Score** | **Expect** | **Rank** | **Unique** | **Peptide** |
|  | 179 | 404.2029 | 806.3913 | 806.3923 | -1.23 | 0 | 41 | 0.0016 | 1 |  | R.LAADDFR.L |
|  | 180 | 404.2037 | 806.3928 | 806.3923 | 0.73 | 0 | (26) | 0.044 | 1 |  | R.LAADDFR.L |
|  | 1384 | 552.7781 | 1103.5416 | 1103.5393 | 2.07 | 0 | 7 | 3.6 | 1 |  | K.ITMQNLNDR.L |
|  | 1473 | 561.7929 | 1121.5713 | 1121.5717 | -0.35 | 0 | 45 | 0.00066 | 1 |  | R.LEQEIATYR.S |
|  | 1849 | 601.3110 | 1200.6075 | 1200.6098 | -1.93 | 0 | (4) | 5.7 | 5 |  | R.QSVEADINGLR.R |
|  | 1850 | 601.3121 | 1200.6096 | 1200.6098 | -0.22 | 0 | 58 | 2.6e-005 | 1 |  | R.QSVEADINGLR.R |
|  | 2255 | 651.3339 | 1300.6532 | 1300.6510 | 1.65 | 0 | 73 | 8.3e-007 | 1 |  | R.ALEEANADLEVK.I |
|  | 2476 | 453.2444 | 1356.7112 | 1356.7110 | 0.20 | 1 | (7) | 3.1 | 8 |  | R.QSVEADINGLRR.V |
|  | 2477 | 453.2444 | 1356.7113 | 1356.7110 | 0.26 | 1 | (28) | 0.027 | 1 |  | R.QSVEADINGLRR.V |
|  | 2479 | 679.3632 | 1356.7118 | 1356.7110 | 0.60 | 1 | (31) | 0.012 | 1 |  | R.QSVEADINGLRR.V |
|  | 2480 | 453.2449 | 1356.7128 | 1356.7110 | 1.35 | 1 | (35) | 0.0053 | 1 |  | R.QSVEADINGLRR.V |
|  | 2481 | 453.2451 | 1356.7135 | 1356.7110 | 1.90 | 1 | (9) | 2.3 | 2 |  | R.QSVEADINGLRR.V |
|  | 2482 | 679.3646 | 1356.7147 | 1356.7110 | 2.75 | 1 | 57 | 3.1e-005 | 1 |  | R.QSVEADINGLRR.V |
|  | 2566 | 460.5807 | 1378.7202 | 1378.7204 | -0.21 | 1 | 60 | 1.7e-005 | 1 |  | K.TRLEQEIATYR.S |
|  | 2567 | 690.3676 | 1378.7207 | 1378.7204 | 0.16 | 1 | (51) | 0.00011 | 1 |  | K.TRLEQEIATYR.S |
|  | 2636 | **696.8470** | **1391.6794** | **1391.6867** | **-5.27** | **0** | **0** | **13** | **5** | **U** | **K.MIGFPSSAGSVSPR.S** |

  


---

|  |  |
| --- | --- |
| **31.** | sp|P08729|K2C7\_HUMAN    **Mass:** 51411    **Score:** 266    **Matches:** 19(12)  **Sequences:** 14(10)  **emPAI:** 0.98 |
|  | Keratin, type II cytoskeletal 7 OS=Homo sapiens OX=9606 GN=KRT7 PE=1 SV=5 |

|  |  |
| --- | --- |
|  | Check to include this hit in error tolerant search or archive report |
|  |  |

|  |  |  |  |  |  |  |  |  |  |  |  |
| --- | --- | --- | --- | --- | --- | --- | --- | --- | --- | --- | --- |
|  | **Query** | **Observed** | **Mr(expt)** | **Mr(calc)** | **ppm** | **Miss** | **Score** | **Expect** | **Rank** | **Unique** | **Peptide** |
|  | 236 | 414.2182 | 826.4219 | 826.4225 | -0.72 | 0 | 40 | 0.0012 | 1 |  | K.FASFIDK.V |
|  | 237 | 414.2184 | 826.4223 | 826.4225 | -0.28 | 0 | (38) | 0.0019 | 1 |  | K.FASFIDK.V |
|  | 893 | **500.2261** | **998.4377** | **998.4379** | **-0.15** | **0** | **23** | **0.029** | **1** | **U** | **K.DVDAAYMSK.V** |
|  | 894 | 500.2269 | 998.4392 | 998.4379 | 1.31 | 0 | (2) | 3.3 | 2 | U | K.DVDAAYMSK.V |
|  | 940 | **507.7629** | **1013.5112** | **1013.5142** | **-2.91** | **0** | **17** | **0.28** | **1** | **U** | **R.LDADPSLQR.V** |
|  | 1094 | **523.2856** | **1044.5566** | **1044.5604** | **-3.61** | **0** | **(1)** | **14** | **5** | **U** | **K.WTLLQEQK.S** |
|  | 1100 | **523.2857** | **1044.5569** | **1044.5604** | **-3.36** | **0** | **(3)** | **10** | **2** | **U** | **K.WTLLQEQK.S** |
|  | 1114 | **523.2869** | **1044.5593** | **1044.5604** | **-1.03** | **0** | **16** | **0.47** | **1** | **U** | **K.WTLLQEQK.S** |
|  | 1276 | 361.5378 | 1081.5916 | 1081.5920 | -0.39 | 1 | (29) | 0.014 | 1 |  | K.FASFIDKVR.F |
|  | 1277 | 541.8041 | 1081.5937 | 1081.5920 | 1.57 | 1 | 44 | 0.00038 | 1 |  | K.FASFIDKVR.F |
|  | 1386 | **552.7948** | **1103.5750** | **1103.5724** | **2.44** | **0** | **34** | **0.0079** | **1** | **U** | **R.SAYGGPVGAGIR.E** |
|  | 1492 | **563.7764** | **1125.5383** | **1125.5376** | **0.65** | **0** | **29** | **0.017** | **1** |  | **R.EYQELMSVK.L** |
|  | 1823 | **598.7844** | **1195.5542** | **1195.5509** | **2.72** | **0** | **43** | **0.00061** | **1** |  | **R.AEAEAWYQTK.F** |
|  | 1831 | 599.2771 | 1196.5396 | 1196.5383 | 1.09 | 0 | 12 | 0.49 | 2 | U | R.SMQDVVEDFK.N |
|  | 2024 | **621.8261** | **1241.6377** | **1241.6364** | **1.01** | **0** | **80** | **1.2e-007** | **1** | **U** | **R.GQLEALQVDGGR.L** |
|  | 2596 | **693.3732** | **1384.7318** | **1384.7310** | **0.58** | **1** | **29** | **0.016** | **1** | **U** | **R.AKQEELEAALQR.G** |
|  | 2735 | **709.8659** | **1417.7173** | **1417.7201** | **-2.01** | **0** | **50** | **0.00016** | **1** | **U** | **K.VDALNDEINFLR.T** |
|  | 2900 | **727.4230** | **1452.8315** | **1452.8300** | **1.04** | **0** | **64** | **2.1e-006** | **1** | **U** | **R.EVTINQSLLAPLR.L** |
|  | 4359 | **1120.0533** | **2238.0921** | **2238.1089** | **-7.50** | **1** | **2** | **7.8** | **3** | **U** | **R.SLDLDGIIAEVKAQYEEMAK.C + Oxidation (M)** |

  


---

|  |  |
| --- | --- |
| **32.** | sp|P68032|ACTC\_HUMAN    **Mass:** 42334    **Score:** 249    **Matches:** 17(13)  **Sequences:** 9(8)  **emPAI:** 1.66 |
|  | Actin, alpha cardiac muscle 1 OS=Homo sapiens OX=9606 GN=ACTC1 PE=1 SV=1 |

|  |  |
| --- | --- |
|  | Check to include this hit in error tolerant search or archive report |
|  |  |

|  |  |  |  |  |  |  |  |  |  |  |  |
| --- | --- | --- | --- | --- | --- | --- | --- | --- | --- | --- | --- |
|  | **Query** | **Observed** | **Mr(expt)** | **Mr(calc)** | **ppm** | **Miss** | **Score** | **Expect** | **Rank** | **Unique** | **Peptide** |
|  | 142 | 398.2396 | 794.4647 | 794.4650 | -0.41 | 0 | 27 | 0.016 | 1 |  | K.IIAPPER.K |
|  | 802 | 488.7278 | 975.4409 | 975.4410 | -0.06 | 0 | 63 | 4.7e-006 | 1 |  | K.AGFAGDDAPR.A |
|  | 888 | 499.7467 | 997.4789 | 997.4790 | -0.11 | 0 | 27 | 0.025 | 1 |  | R.DLTDYLMK.I |
|  | 889 | 499.7469 | 997.4793 | 997.4790 | 0.27 | 0 | (19) | 0.13 | 1 |  | R.DLTDYLMK.I |
|  | 938 | 507.7443 | 1013.4740 | 1013.4739 | 0.09 | 0 | (26) | 0.024 | 1 |  | R.DLTDYLMK.I + Oxidation (M) |
|  | 939 | 507.7447 | 1013.4748 | 1013.4739 | 0.82 | 0 | (5) | 2.9 | 1 |  | R.DLTDYLMK.I + Oxidation (M) |
|  | 1653 | 581.3132 | 1160.6119 | 1160.6111 | 0.71 | 0 | 47 | 0.00042 | 1 |  | K.EITALAPSTMK.I |
|  | 1703 | 586.2890 | 1170.5634 | 1170.5638 | -0.30 | 0 | 71 | 9.3e-007 | 1 |  | R.HQGVMVGMGQK.D |
|  | 1729 | 589.3102 | 1176.6058 | 1176.6060 | -0.17 | 0 | (44) | 0.00076 | 1 |  | K.EITALAPSTMK.I + Oxidation (M) |
|  | 1842 | 400.2400 | 1197.6981 | 1197.6982 | -0.07 | 0 | 31 | 0.006 | 1 |  | R.AVFPSIVGRPR.H |
|  | 2677 | 468.2770 | 1401.8090 | 1401.7901 | 13.5 | 1 | 2 | 3.5 | 2 |  | K.EITALAPSTMKIK.I |
|  | 3063 | 379.6925 | 1514.7408 | 1514.7419 | -0.72 | 0 | (17) | 0.24 | 2 |  | K.IWHHTFYNELR.V |
|  | 3064 | 505.9215 | 1514.7426 | 1514.7419 | 0.49 | 0 | (28) | 0.022 | 1 |  | K.IWHHTFYNELR.V |
|  | 3065 | 758.3787 | 1514.7428 | 1514.7419 | 0.59 | 0 | 40 | 0.0013 | 1 |  | K.IWHHTFYNELR.V |
|  | 4057 | **654.3077** | **1959.9014** | **1959.9036** | **-1.15** | **0** | **37** | **0.0016** | **1** | **U** | **K.YPIEHGIITNWDDMEK.I** |
|  | 4073 | **659.6399** | **1975.8978** | **1975.8986** | **-0.36** | **0** | **(24)** | **0.034** | **1** | **U** | **K.YPIEHGIITNWDDMEK.I + Oxidation (M)** |
|  | 4074 | **988.9577** | **1975.9008** | **1975.8986** | **1.16** | **0** | **(29)** | **0.0098** | **1** | **U** | **K.YPIEHGIITNWDDMEK.I + Oxidation (M)** |

  

|  |  |
| --- | --- |
|  | |
|  | **Proteins matching the same set of peptides:** |

|  |  |
| --- | --- |
|  | sp|P68133|ACTS\_HUMAN    **Mass:** 42366    **Score:** 249    **Matches:** 17(13)  **Sequences:** 9(8) |
|  | Actin, alpha skeletal muscle OS=Homo sapiens OX=9606 GN=ACTA1 PE=1 SV=1 |

---

|  |  |
| --- | --- |
| **33.** | sp|P52597|HNRPF\_HUMAN    **Mass:** 45985    **Score:** 244    **Matches:** 13(9)  **Sequences:** 7(5)  **emPAI:** 0.63 |
|  | Heterogeneous nuclear ribonucleoprotein F OS=Homo sapiens OX=9606 GN=HNRNPF PE=1 SV=3 |

|  |  |
| --- | --- |
|  | Check to include this hit in error tolerant search or archive report |
|  |  |

|  |  |  |  |  |  |  |  |  |  |  |  |
| --- | --- | --- | --- | --- | --- | --- | --- | --- | --- | --- | --- |
|  | **Query** | **Observed** | **Mr(expt)** | **Mr(calc)** | **ppm** | **Miss** | **Score** | **Expect** | **Rank** | **Unique** | **Peptide** |
|  | 146 | **399.7232** | **797.4319** | **797.4323** | **-0.58** | **0** | **(13)** | **0.32** | **1** | **U** | **R.YIEVFK.S** |
|  | 147 | **399.7233** | **797.4320** | **797.4323** | **-0.43** | **0** | **25** | **0.021** | **1** | **U** | **R.YIEVFK.S** |
|  | 482 | **440.2233** | **878.4320** | **878.4320** | **-0.06** | **0** | **15** | **0.5** | **1** | **U** | **R.GLPFGCTK.E** |
|  | 962 | **511.2545** | **1020.4945** | **1020.4950** | **-0.51** | **0** | **23** | **0.077** | **1** | **U** | **R.TEMDWVLK.H** |
|  | 1321 | 546.7930 | 1091.5714 | 1091.5724 | -0.89 | 0 | (41) | 0.0013 | 1 |  | R.VHIEIGPDGR.V |
|  | 1322 | 546.7931 | 1091.5717 | 1091.5724 | -0.56 | 0 | (38) | 0.0025 | 1 |  | R.VHIEIGPDGR.V |
|  | 1323 | 364.8646 | 1091.5719 | 1091.5724 | -0.46 | 0 | 49 | 0.00021 | 1 |  | R.VHIEIGPDGR.V |
|  | 1324 | 364.8646 | 1091.5721 | 1091.5724 | -0.22 | 0 | (40) | 0.0014 | 1 |  | R.VHIEIGPDGR.V |
|  | 3302 | **808.8435** | **1615.6725** | **1615.6759** | **-2.13** | **0** | **32** | **0.0013** | **1** | **U** | **R.DLSYCLSGMYDHR.Y** |
|  | 3328 | **544.2447** | **1629.7124** | **1629.7132** | **-0.46** | **0** | **(74)** | **1.8e-007** | **1** | **U** | **K.HSGPNSADSANDGFVR.L** |
|  | 3329 | **815.8641** | **1629.7136** | **1629.7132** | **0.26** | **0** | **77** | **9.2e-008** | **1** | **U** | **K.HSGPNSADSANDGFVR.L** |
|  | 4012 | **484.4970** | **1933.9587** | **1933.9581** | **0.31** | **0** | **(10)** | **1.3** | **1** | **U** | **K.FMSVQRPGPYDRPGTAR.R** |
|  | 4013 | **645.6605** | **1933.9597** | **1933.9581** | **0.84** | **0** | **28** | **0.021** | **1** | **U** | **K.FMSVQRPGPYDRPGTAR.R** |

  


---

|  |  |
| --- | --- |
| **34.** | sp|Q02413|DSG1\_HUMAN    **Mass:** 114702   **Score:** 240    **Matches:** 9(5)  **Sequences:** 6(5)  **emPAI:** 0.15 |
|  | Desmoglein-1 OS=Homo sapiens OX=9606 GN=DSG1 PE=1 SV=2 |

|  |  |
| --- | --- |
|  | Check to include this hit in error tolerant search or archive report |
|  |  |

|  |  |  |  |  |  |  |  |  |  |  |  |
| --- | --- | --- | --- | --- | --- | --- | --- | --- | --- | --- | --- |
|  | **Query** | **Observed** | **Mr(expt)** | **Mr(calc)** | **ppm** | **Miss** | **Score** | **Expect** | **Rank** | **Unique** | **Peptide** |
|  | 206 | **408.7450** | **815.4755** | **815.4752** | **0.32** | **0** | **10** | **2.4** | **1** | **U** | **K.LADISLGK.E** |
|  | 1899 | **606.3014** | **1210.5882** | **1210.5904** | **-1.78** | **0** | **(14)** | **0.41** | **1** | **U** | **R.MTGFELTEGVK.T** |
|  | 1953 | **614.3003** | **1226.5860** | **1226.5853** | **0.60** | **0** | **52** | **6.2e-005** | **1** | **U** | **R.MTGFELTEGVK.T + Oxidation (M)** |
|  | 2232 | **649.3293** | **1296.6440** | **1296.6462** | **-1.71** | **0** | **48** | **0.00022** | **1** | **U** | **R.EQYGQYALAVR.G** |
|  | 3169 | **775.3689** | **1548.7232** | **1548.7242** | **-0.63** | **0** | **58** | **1.8e-005** | **1** | **U** | **R.QEPSDSPMFIINR.N + Oxidation (M)** |
|  | 3170 | **775.3713** | **1548.7280** | **1548.7242** | **2.44** | **0** | **(15)** | **0.39** | **1** | **U** | **R.QEPSDSPMFIINR.N + Oxidation (M)** |
|  | 3401 | **831.8857** | **1661.7569** | **1661.7580** | **-0.64** | **0** | **(1)** | **6.1** | **2** | **U** | **K.IHSDCAANQQVTYR.I** |
|  | 3402 | **554.9266** | **1661.7579** | **1661.7580** | **-0.07** | **0** | **55** | **2.9e-005** | **1** | **U** | **K.IHSDCAANQQVTYR.I** |
|  | 3589 | **875.9257** | **1749.8368** | **1749.8356** | **0.68** | **0** | **115** | **3.6e-011** | **1** | **U** | **R.YVMGNNPADLLAVDSR.T + Oxidation (M)** |

  


---

|  |  |
| --- | --- |
| **35.** | sp|Q8N1N4|K2C78\_HUMAN    **Mass:** 57629    **Score:** 237    **Matches:** 12(9)  **Sequences:** 10(8)  **emPAI:** 0.56 |
|  | Keratin, type II cytoskeletal 78 OS=Homo sapiens OX=9606 GN=KRT78 PE=1 SV=2 |

|  |  |
| --- | --- |
|  | Check to include this hit in error tolerant search or archive report |
|  |  |

|  |  |  |  |  |  |  |  |  |  |  |  |
| --- | --- | --- | --- | --- | --- | --- | --- | --- | --- | --- | --- |
|  | **Query** | **Observed** | **Mr(expt)** | **Mr(calc)** | **ppm** | **Miss** | **Score** | **Expect** | **Rank** | **Unique** | **Peptide** |
|  | 800 | **488.2625** | **974.5105** | **974.5113** | **-0.78** | **0** | **10** | **2.2** | **1** | **U** | **R.EYLYFLK.H** |
|  | 815 | **490.2590** | **978.5034** | **978.5022** | **1.19** | **0** | **12** | **0.91** | **1** | **U** | **K.DVDGVFLSK.M** |
|  | 945 | **508.2746** | **1014.5346** | **1014.5345** | **0.10** | **0** | **52** | **0.00011** | **1** | **U** | **K.VDELEAALR.M** |
|  | 1477 | **562.2861** | **1122.5577** | **1122.5557** | **1.82** | **0** | **31** | **0.011** | **1** |  | **K.AEAEALYQTK.Y** |
|  | 2041 | **624.8448** | **1247.6751** | **1247.6761** | **-0.80** | **0** | **28** | **0.025** | **1** | **U** | **R.ATLENDFVVLK.K** |
|  | 2582 | **692.3206** | **1382.6266** | **1382.6249** | **1.23** | **0** | **43** | **0.0005** | **1** | **U** | **R.SLNSFGGCLEGSR.G** |
|  | 2663 | **699.3562** | **1396.6978** | **1396.6987** | **-0.59** | **0** | **57** | **3.1e-005** | **1** | **U** | **R.TLNNQFASFIDK.V** |
|  | 2962 | 738.8926 | 1475.7706 | 1475.7984 | -18.81 | 1 | (34) | 0.0064 | 1 |  | R.FLEQQNKVLETK.W |
|  | 2963 | 738.8928 | 1475.7711 | 1475.7984 | -18.49 | 1 | 58 | 2.3e-005 | 1 |  | R.FLEQQNKVLETK.W |
|  | 2968 | **739.9067** | **1477.7989** | **1477.8001** | **-0.80** | **0** | **63** | **4.6e-006** | **1** | **U** | **K.VQISQLHQEIQR.L** |
|  | 2969 | **493.6078** | **1477.8016** | **1477.8001** | **1.03** | **0** | **(12)** | **0.57** | **1** | **U** | **K.VQISQLHQEIQR.L** |
|  | 2981 | **742.8674** | **1483.7203** | **1483.7228** | **-1.70** | **0** | **44** | **0.0005** | **1** | **U** | **R.LLCEYQELTSTK.L** |

  


---

|  |  |
| --- | --- |
| **36.** | sp|P08727|K1C19\_HUMAN    **Mass:** 44079    **Score:** 228    **Matches:** 20(13)  **Sequences:** 11(8)  **emPAI:** 1.06 |
|  | Keratin, type I cytoskeletal 19 OS=Homo sapiens OX=9606 GN=KRT19 PE=1 SV=4 |

|  |  |
| --- | --- |
|  | Check to include this hit in error tolerant search or archive report |
|  |  |

|  |  |  |  |  |  |  |  |  |  |  |  |
| --- | --- | --- | --- | --- | --- | --- | --- | --- | --- | --- | --- |
|  | **Query** | **Observed** | **Mr(expt)** | **Mr(calc)** | **ppm** | **Miss** | **Score** | **Expect** | **Rank** | **Unique** | **Peptide** |
|  | 179 | 404.2029 | 806.3913 | 806.3923 | -1.23 | 0 | 41 | 0.0016 | 1 |  | R.LAADDFR.T |
|  | 180 | 404.2037 | 806.3928 | 806.3923 | 0.73 | 0 | (26) | 0.044 | 1 |  | R.LAADDFR.T |
|  | 182 | 405.2236 | 808.4326 | 808.4330 | -0.49 | 0 | (30) | 0.013 | 1 |  | R.LASYLDK.V |
|  | 183 | 405.2238 | 808.4329 | 808.4330 | -0.12 | 0 | 40 | 0.0016 | 1 |  | R.LASYLDK.V |
|  | 866 | 497.2538 | 992.4930 | 992.4927 | 0.32 | 0 | 8 | 2.9 | 6 |  | K.FETEQALR.M |
|  | 867 | 497.2541 | 992.4936 | 992.4927 | 0.94 | 0 | (4) | 6.6 | 8 |  | K.FETEQALR.M |
|  | 1006 | 515.3003 | 1028.5860 | 1028.5866 | -0.55 | 0 | 44 | 0.00051 | 1 |  | R.VLDELTLAR.T |
|  | 1007 | 515.3008 | 1028.5871 | 1028.5866 | 0.52 | 0 | (33) | 0.0058 | 1 |  | R.VLDELTLAR.T |
|  | 1051 | 521.3065 | 1040.5984 | 1040.5978 | 0.52 | 0 | 50 | 7.3e-005 | 1 |  | R.IVLQIDNAR.L |
|  | 1197 | 355.5417 | 1063.6032 | 1063.6026 | 0.63 | 1 | (41) | 0.00067 | 1 |  | R.LASYLDKVR.A |
|  | 1198 | 532.8090 | 1063.6034 | 1063.6026 | 0.75 | 1 | 47 | 0.00017 | 1 |  | R.LASYLDKVR.A |
|  | 1384 | 552.7781 | 1103.5416 | 1103.5393 | 2.07 | 0 | 7 | 3.6 | 1 | U | K.LTMQNLNDR.L |
|  | 1473 | 561.7929 | 1121.5713 | 1121.5717 | -0.35 | 0 | 45 | 0.00066 | 1 |  | R.LEQEIATYR.S |
|  | 1774 | 395.9029 | 1184.6868 | 1184.6877 | -0.72 | 1 | (2) | 6 | 2 |  | R.RVLDELTLAR.T |
|  | 1775 | 395.9031 | 1184.6873 | 1184.6877 | -0.32 | 1 | 4 | 3.6 | 2 |  | R.RVLDELTLAR.T |
|  | 1928 | 611.8197 | 1221.6248 | 1221.6353 | -8.58 | 1 | (6) | 4.4 | 5 |  | R.TKFETEQALR.M |
|  | 1929 | 611.8249 | 1221.6353 | 1221.6353 | 0.01 | 1 | (26) | 0.035 | 1 |  | R.TKFETEQALR.M |
|  | 1930 | 408.2196 | 1221.6370 | 1221.6353 | 1.34 | 1 | 40 | 0.0016 | 1 |  | R.TKFETEQALR.M |
|  | 2505 | 454.2378 | 1359.6917 | 1359.6929 | -0.83 | 1 | 32 | 0.011 | 1 |  | R.MSVEADINGLRR.V |
|  | 2506 | 680.8541 | 1359.6936 | 1359.6929 | 0.52 | 1 | (14) | 0.85 | 1 |  | R.MSVEADINGLRR.V |

  


---

|  |  |
| --- | --- |
| **37.** | sp|O95678|K2C75\_HUMAN    **Mass:** 59809    **Score:** 227    **Matches:** 13(11)  **Sequences:** 9(7)  **emPAI:** 0.62 |
|  | Keratin, type II cytoskeletal 75 OS=Homo sapiens OX=9606 GN=KRT75 PE=1 SV=2 |

|  |  |
| --- | --- |
|  | Check to include this hit in error tolerant search or archive report |
|  |  |

|  |  |  |  |  |  |  |  |  |  |  |  |
| --- | --- | --- | --- | --- | --- | --- | --- | --- | --- | --- | --- |
|  | **Query** | **Observed** | **Mr(expt)** | **Mr(calc)** | **ppm** | **Miss** | **Score** | **Expect** | **Rank** | **Unique** | **Peptide** |
|  | 236 | 414.2182 | 826.4219 | 826.4225 | -0.72 | 0 | 40 | 0.0012 | 1 |  | K.FASFIDK.V |
|  | 237 | 414.2184 | 826.4223 | 826.4225 | -0.28 | 0 | (38) | 0.0019 | 1 |  | K.FASFIDK.V |
|  | 971 | **511.7238** | **1021.4329** | **1021.4498** | **-16.53** | **0** | **10** | **0.47** | **1** | **U** | **K.QEISEMNR.M + Oxidation (M)** |
|  | 983 | 513.7317 | 1025.4488 | 1025.4488 | 0.05 | 0 | 31 | 0.0054 | 1 |  | K.DVDAAYMNK.V |
|  | 1276 | 361.5378 | 1081.5916 | 1081.5920 | -0.39 | 1 | (29) | 0.014 | 1 |  | K.FASFIDKVR.F |
|  | 1277 | 541.8041 | 1081.5937 | 1081.5920 | 1.57 | 1 | 44 | 0.00038 | 1 |  | K.FASFIDKVR.F |
|  | 1677 | 583.2967 | 1164.5788 | 1164.5775 | 1.15 | 0 | 57 | 2.9e-005 | 1 |  | K.YEELQVTAGR.H |
|  | 1802 | **596.8079** | **1191.6012** | **1191.6135** | **-10.37** | **0** | **1** | **15** | **8** | **U** | **R.TAAENEFVALK.K** |
|  | 1906 | 606.7822 | 1211.5499 | 1211.5458 | 3.36 | 0 | 42 | 0.0006 | 1 |  | R.AEAESWYQTK.Y |
|  | 2907 | 728.3460 | 1454.6775 | 1454.6790 | -1.03 | 1 | 54 | 4.4e-005 | 1 |  | R.SRAEAESWYQTK.Y |
|  | 2908 | 485.9004 | 1454.6793 | 1454.6790 | 0.19 | 1 | (30) | 0.012 | 1 |  | R.SRAEAESWYQTK.Y |
|  | 2962 | 738.8926 | 1475.7706 | 1475.7984 | -18.81 | 1 | (34) | 0.0064 | 1 |  | R.FLEQQNKVLETK.W |
|  | 2963 | 738.8928 | 1475.7711 | 1475.7984 | -18.49 | 1 | 58 | 2.3e-005 | 1 |  | R.FLEQQNKVLETK.W |

  


---

|  |  |
| --- | --- |
| **38.** | sp|Q7Z794|K2C1B\_HUMAN    **Mass:** 62149    **Score:** 202    **Matches:** 10(7)  **Sequences:** 5(3)  **emPAI:** 0.29 |
|  | Keratin, type II cytoskeletal 1b OS=Homo sapiens OX=9606 GN=KRT77 PE=2 SV=3 |

|  |  |
| --- | --- |
|  | Check to include this hit in error tolerant search or archive report |
|  |  |

|  |  |  |  |  |  |  |  |  |  |  |  |
| --- | --- | --- | --- | --- | --- | --- | --- | --- | --- | --- | --- |
|  | **Query** | **Observed** | **Mr(expt)** | **Mr(calc)** | **ppm** | **Miss** | **Score** | **Expect** | **Rank** | **Unique** | **Peptide** |
|  | 236 | 414.2182 | 826.4219 | 826.4225 | -0.72 | 0 | 40 | 0.0012 | 1 |  | K.FASFIDK.V |
|  | 237 | 414.2184 | 826.4223 | 826.4225 | -0.28 | 0 | (38) | 0.0019 | 1 |  | K.FASFIDK.V |
|  | 1276 | 361.5378 | 1081.5916 | 1081.5920 | -0.39 | 1 | (29) | 0.014 | 1 |  | K.FASFIDKVR.F |
|  | 1277 | 541.8041 | 1081.5937 | 1081.5920 | 1.57 | 1 | 44 | 0.00038 | 1 |  | K.FASFIDKVR.F |
|  | 1470 | 561.2989 | 1120.5832 | 1120.5876 | -3.94 | 0 | 8 | 2.8 | 1 | U | R.TQYELIAQR.S |
|  | 2396 | 446.2434 | 1335.7085 | 1335.7034 | 3.79 | 1 | 9 | 1.4 | 2 | U | R.TGSENDFVVLKK.D |
|  | 2954 | 738.3951 | 1474.7757 | 1474.7780 | -1.53 | 0 | (11) | 1.1 | 1 |  | R.FLEQQNQVLQTK.W |
|  | 2955 | 738.3966 | 1474.7785 | 1474.7780 | 0.38 | 0 | 74 | 6.1e-007 | 1 |  | R.FLEQQNQVLQTK.W |
|  | 2956 | 492.6006 | 1474.7799 | 1474.7780 | 1.27 | 0 | (48) | 0.00024 | 1 |  | R.FLEQQNQVLQTK.W |
|  | 2957 | 738.3979 | 1474.7812 | 1474.7780 | 2.20 | 0 | (67) | 3.3e-006 | 1 |  | R.FLEQQNQVLQTK.W |

  


---

|  |  |
| --- | --- |
| **39.** | sp|P55795|HNRH2\_HUMAN    **Mass:** 49517    **Score:** 202    **Matches:** 12(7)  **Sequences:** 8(4)  **emPAI:** 0.38 |
|  | Heterogeneous nuclear ribonucleoprotein H2 OS=Homo sapiens OX=9606 GN=HNRNPH2 PE=1 SV=1 |

|  |  |
| --- | --- |
|  | Check to include this hit in error tolerant search or archive report |
|  |  |

|  |  |  |  |  |  |  |  |  |  |  |  |
| --- | --- | --- | --- | --- | --- | --- | --- | --- | --- | --- | --- |
|  | **Query** | **Observed** | **Mr(expt)** | **Mr(calc)** | **ppm** | **Miss** | **Score** | **Expect** | **Rank** | **Unique** | **Peptide** |
|  | 118 | 392.7156 | 783.4167 | 783.4167 | 0.05 | 0 | 13 | 0.35 | 1 |  | R.YVEVFK.S |
|  | 190 | 406.7313 | 811.4480 | 811.4480 | 0.07 | 0 | 13 | 0.36 | 1 |  | R.YIEIFK.S |
|  | 408 | 433.2155 | 864.4164 | 864.4164 | 0.03 | 0 | 13 | 0.64 | 1 |  | R.GLPFGCSK.E |
|  | 1321 | 546.7930 | 1091.5714 | 1091.5724 | -0.89 | 0 | (41) | 0.0013 | 1 |  | R.VHIEIGPDGR.V |
|  | 1322 | 546.7931 | 1091.5717 | 1091.5724 | -0.56 | 0 | (38) | 0.0025 | 1 |  | R.VHIEIGPDGR.V |
|  | 1323 | 364.8646 | 1091.5719 | 1091.5724 | -0.46 | 0 | 49 | 0.00021 | 1 |  | R.VHIEIGPDGR.V |
|  | 1324 | 364.8646 | 1091.5721 | 1091.5724 | -0.22 | 0 | (40) | 0.0014 | 1 |  | R.VHIEIGPDGR.V |
|  | 2276 | **654.3179** | **1306.6212** | **1306.6227** | **-1.18** | **0** | **1** | **10** | **3** | **U** | **K.SNSVEMDWVLK.H** |
|  | 3280 | 801.3275 | 1600.6405 | 1600.6399 | 0.38 | 0 | 61 | 1.5e-006 | 1 |  | R.DLNYCFSGMSDHR.Y |
|  | 3442 | 562.2592 | 1683.7557 | 1683.7601 | -2.64 | 0 | 64 | 2.9e-006 | 1 |  | K.HTGPNSPDTANDGFVR.L |
|  | 3443 | 842.8865 | 1683.7585 | 1683.7601 | -0.95 | 0 | (14) | 0.3 | 1 |  | K.HTGPNSPDTANDGFVR.L |
|  | 3809 | 921.4487 | 1840.8828 | 1840.8843 | -0.82 | 0 | 29 | 0.014 | 1 |  | R.STGEAFVQFASQEIAEK.A |

  


---

|  |  |
| --- | --- |
| **40.** | sp|Q96I25|SPF45\_HUMAN    **Mass:** 45162    **Score:** 196    **Matches:** 15(9)  **Sequences:** 13(8)  **emPAI:** 1.17 |
|  | Splicing factor 45 OS=Homo sapiens OX=9606 GN=RBM17 PE=1 SV=1 |

|  |  |
| --- | --- |
|  | Check to include this hit in error tolerant search or archive report |
|  |  |

|  |  |  |  |  |  |  |  |  |  |  |  |
| --- | --- | --- | --- | --- | --- | --- | --- | --- | --- | --- | --- |
|  | **Query** | **Observed** | **Mr(expt)** | **Mr(calc)** | **ppm** | **Miss** | **Score** | **Expect** | **Rank** | **Unique** | **Peptide** |
|  | 695 | **473.2667** | **944.5188** | **944.5179** | **1.03** | **0** | **11** | **2** | **1** | **U** | **K.IIVGDATEK.D** |
|  | 717 | **477.2582** | **952.5018** | **952.5018** | **0.01** | **0** | **19** | **0.15** | **1** | **U** | **R.IFLEFER.V** |
|  | 1158 | **528.8245** | **1055.6344** | **1055.6339** | **0.46** | **0** | **26** | **0.017** | **1** | **U** | **K.LLQSQLQVK.K** |
|  | 1589 | 382.5232 | 1144.5478 | 1144.5374 | 9.15 | 1 | 9 | 1.5 | 6 | U | K.DRHEASGFAR.R |
|  | 2174 | **642.3823** | **1282.7501** | **1282.7496** | **0.34** | **0** | **48** | **0.0001** | **1** | **U** | **K.QSTVLAPVIDLK.R** |
|  | 2438 | **672.8724** | **1343.7302** | **1343.7296** | **0.44** | **1** | **30** | **0.015** | **1** | **U** | **K.KSDSNPLTEILK.C** |
|  | 2443 | **673.8623** | **1345.7100** | **1345.7089** | **0.86** | **1** | **15** | **0.61** | **1** | **U** | **K.IIVGDATEKDASK.K** |
|  | 2667 | **699.8649** | **1397.7153** | **1397.7150** | **0.19** | **0** | **85** | **3.9e-008** | **1** | **U** | **K.HEQGLSTALSVEK.T** |
|  | 2851 | **720.4324** | **1438.8503** | **1438.8508** | **-0.31** | **1** | **31** | **0.0028** | **1** | **U** | **K.QSTVLAPVIDLKR.G** |
|  | 3102 | **764.9052** | **1527.7957** | **1527.7967** | **-0.60** | **0** | **26** | **0.037** | **1** | **U** | **R.SMGGAAIAPPTSLVEK.D** |
|  | 3156 | **772.9037** | **1543.7929** | **1543.7916** | **0.89** | **0** | **(23)** | **0.081** | **1** | **U** | **R.SMGGAAIAPPTSLVEK.D + Oxidation (M)** |
|  | 3557 | **869.3442** | **1736.6739** | **1736.6761** | **-1.28** | **0** | **(24)** | **0.0043** | **1** | **U** | **R.RPDPDSDEDEDYER.E** |
|  | 3558 | **579.8997** | **1736.6772** | **1736.6761** | **0.58** | **0** | **34** | **0.00037** | **1** | **U** | **R.RPDPDSDEDEDYER.E** |
|  | 3559 | **579.9689** | **1736.8850** | **1736.8846** | **0.24** | **0** | **22** | **0.073** | **1** | **U** | **K.AAIPPPVYEEQDRPR.S** |
|  | 4129 | **674.9482** | **2021.8229** | **2021.8198** | **1.51** | **1** | **33** | **0.00048** | **1** | **U** | **R.RPDPDSDEDEDYERER.R** |

  


---

|  |  |
| --- | --- |
| **41.** | sp|Q6S8J3|POTEE\_HUMAN    **Mass:** 122882   **Score:** 196    **Matches:** 13(6)  **Sequences:** 9(4)  **emPAI:** 0.17 |
|  | POTE ankyrin domain family member E OS=Homo sapiens OX=9606 GN=POTEE PE=2 SV=3 |

|  |  |
| --- | --- |
|  | Check to include this hit in error tolerant search or archive report |
|  |  |

|  |  |  |  |  |  |  |  |  |  |  |  |
| --- | --- | --- | --- | --- | --- | --- | --- | --- | --- | --- | --- |
|  | **Query** | **Observed** | **Mr(expt)** | **Mr(calc)** | **ppm** | **Miss** | **Score** | **Expect** | **Rank** | **Unique** | **Peptide** |
|  | 103 | **384.7143** | **767.4140** | **767.4038** | **13.2** | **0** | **0** | **9.3** | **2** | **U** | **K.HQSQLR.E** |
|  | 802 | 488.7278 | 975.4409 | 975.4410 | -0.06 | 0 | 63 | 4.7e-006 | 1 |  | K.AGFAGDDAPR.A |
|  | 838 | **494.2949** | **986.5753** | **986.5947** | **-19.56** | **1** | **4** | **4.8** | **2** |  | **R.KDLIVMLR.D** |
|  | 1398 | 554.2739 | 1106.5333 | 1106.5277 | 5.03 | 0 | 6 | 4.1 | 2 | U | K.MSQELEINK.D + Oxidation (M) |
|  | 1842 | 400.2400 | 1197.6981 | 1197.6982 | -0.07 | 0 | 31 | 0.006 | 1 |  | R.AVFPSIVGRPR.Q |
|  | 3063 | 379.6925 | 1514.7408 | 1514.7419 | -0.72 | 0 | (17) | 0.24 | 2 |  | K.IWHHTFYNELR.V |
|  | 3064 | 505.9215 | 1514.7426 | 1514.7419 | 0.49 | 0 | (28) | 0.022 | 1 |  | K.IWHHTFYNELR.V |
|  | 3065 | 758.3787 | 1514.7428 | 1514.7419 | 0.59 | 0 | 40 | 0.0013 | 1 |  | K.IWHHTFYNELR.V |
|  | 3071 | 758.8526 | 1515.6906 | 1515.6954 | -3.12 | 0 | 86 | 2.4e-008 | 1 |  | K.QEYDESGPSIVHR.K |
|  | 3072 | 506.2388 | 1515.6947 | 1515.6954 | -0.47 | 0 | (51) | 7.2e-005 | 1 |  | K.QEYDESGPSIVHR.K |
|  | 3073 | 506.2394 | 1515.6965 | 1515.6954 | 0.74 | 0 | (9) | 1.1 | 1 |  | K.QEYDESGPSIVHR.K |
|  | 3240 | **790.9051** | **1579.7956** | **1579.7772** | **11.7** | **1** | **4** | **5.4** | **1** | **U** | **R.MQKEIAALAPSMMK.I + 2 Oxidation (M)** |
|  | 3299 | **807.9205** | **1613.8264** | **1613.8195** | **4.26** | **1** | **8** | **2.2** | **1** | **U** | **R.LELDTMKHQSQLR.E + Oxidation (M)** |

  


---

|  |  |
| --- | --- |
| **42.** | sp|P12035|K2C3\_HUMAN    **Mass:** 64549    **Score:** 191    **Matches:** 14(10)  **Sequences:** 8(6)  **emPAI:** 0.49 |
|  | Keratin, type II cytoskeletal 3 OS=Homo sapiens OX=9606 GN=KRT3 PE=1 SV=3 |

|  |  |
| --- | --- |
|  | Check to include this hit in error tolerant search or archive report |
|  |  |

|  |  |  |  |  |  |  |  |  |  |  |  |
| --- | --- | --- | --- | --- | --- | --- | --- | --- | --- | --- | --- |
|  | **Query** | **Observed** | **Mr(expt)** | **Mr(calc)** | **ppm** | **Miss** | **Score** | **Expect** | **Rank** | **Unique** | **Peptide** |
|  | 236 | 414.2182 | 826.4219 | 826.4225 | -0.72 | 0 | 40 | 0.0012 | 1 |  | K.FASFIDK.V |
|  | 237 | 414.2184 | 826.4223 | 826.4225 | -0.28 | 0 | (38) | 0.0019 | 1 |  | K.FASFIDK.V |
|  | 792 | 487.2693 | 972.5241 | 972.5240 | 0.18 | 0 | 6 | 4.9 | 7 | U | K.SEIIELNR.M |
|  | 1276 | 361.5378 | 1081.5916 | 1081.5920 | -0.39 | 1 | (29) | 0.014 | 1 |  | K.FASFIDKVR.F |
|  | 1277 | 541.8041 | 1081.5937 | 1081.5920 | 1.57 | 1 | 44 | 0.00038 | 1 |  | K.FASFIDKVR.F |
|  | 1477 | 562.2861 | 1122.5577 | 1122.5557 | 1.82 | 0 | 31 | 0.011 | 1 |  | K.AEAEALYQTK.L |
|  | 1555 | 570.2736 | 1138.5327 | 1138.5328 | -0.13 | 0 | 37 | 0.0026 | 1 |  | R.DYQELMNVK.L |
|  | 1625 | 578.2707 | 1154.5268 | 1154.5278 | -0.80 | 0 | (23) | 0.044 | 1 |  | R.DYQELMNVK.L + Oxidation (M) |
|  | 1928 | 611.8197 | 1221.6248 | 1221.6241 | 0.61 | 0 | 66 | 4.3e-006 | 1 |  | R.TAAENEFVTLK.K |
|  | 1929 | 611.8249 | 1221.6353 | 1221.6241 | 9.21 | 0 | (2) | 10 | 9 |  | R.TAAENEFVTLK.K |
|  | 1930 | 408.2196 | 1221.6370 | 1221.6241 | 10.5 | 0 | (8) | 2.5 | 4 |  | R.TAAENEFVTLK.K |
|  | 2735 | 709.8659 | 1417.7173 | 1417.7453 | -19.76 | 0 | 14 | 0.67 | 2 | U | K.VDALIDEIDFLR.T |
|  | 2962 | 738.8926 | 1475.7706 | 1475.7984 | -18.81 | 1 | (34) | 0.0064 | 1 |  | R.FLEQQNKVLETK.W |
|  | 2963 | 738.8928 | 1475.7711 | 1475.7984 | -18.49 | 1 | 58 | 2.3e-005 | 1 |  | R.FLEQQNKVLETK.W |

  


---

|  |  |
| --- | --- |
| **43.** | sp|Q5XKE5|K2C79\_HUMAN    **Mass:** 58085    **Score:** 173    **Matches:** 12(10)  **Sequences:** 7(6)  **emPAI:** 0.55 |
|  | Keratin, type II cytoskeletal 79 OS=Homo sapiens OX=9606 GN=KRT79 PE=1 SV=2 |

|  |  |
| --- | --- |
|  | Check to include this hit in error tolerant search or archive report |
|  |  |

|  |  |  |  |  |  |  |  |  |  |  |  |
| --- | --- | --- | --- | --- | --- | --- | --- | --- | --- | --- | --- |
|  | **Query** | **Observed** | **Mr(expt)** | **Mr(calc)** | **ppm** | **Miss** | **Score** | **Expect** | **Rank** | **Unique** | **Peptide** |
|  | 236 | 414.2182 | 826.4219 | 826.4225 | -0.72 | 0 | 40 | 0.0012 | 1 |  | K.FASFIDK.V |
|  | 237 | 414.2184 | 826.4223 | 826.4225 | -0.28 | 0 | (38) | 0.0019 | 1 |  | K.FASFIDK.V |
|  | 694 | 473.2593 | 944.5039 | 944.5039 | 0.03 | 1 | 37 | 0.0045 | 1 |  | R.GRLDSELR.N |
|  | 1276 | 361.5378 | 1081.5916 | 1081.5920 | -0.39 | 1 | (29) | 0.014 | 1 |  | K.FASFIDKVR.F |
|  | 1277 | 541.8041 | 1081.5937 | 1081.5920 | 1.57 | 1 | 44 | 0.00038 | 1 |  | K.FASFIDKVR.F |
|  | 1555 | 570.2736 | 1138.5327 | 1138.5328 | -0.13 | 0 | 37 | 0.0026 | 1 |  | R.DYQELMNVK.L |
|  | 1625 | 578.2707 | 1154.5268 | 1154.5278 | -0.80 | 0 | (23) | 0.044 | 1 |  | R.DYQELMNVK.L + Oxidation (M) |
|  | 1823 | 598.7844 | 1195.5542 | 1195.5509 | 2.72 | 0 | 43 | 0.00061 | 1 |  | R.AEAEAWYQTK.Y |
|  | 2962 | 738.8926 | 1475.7706 | 1475.7984 | -18.81 | 1 | (34) | 0.0064 | 1 |  | R.FLEQQNKVLETK.W |
|  | 2963 | 738.8928 | 1475.7711 | 1475.7984 | -18.49 | 1 | 58 | 2.3e-005 | 1 |  | R.FLEQQNKVLETK.W |
|  | 3424 | **839.8777** | **1677.7408** | **1677.7603** | **-11.61** | **1** | **2** | **3.6** | **1** | **U** | **K.DVDAAYMGRMDLHGK.V** |
|  | 3459 | **847.8746** | **1693.7346** | **1693.7552** | **-12.18** | **1** | **(0)** | **4.8** | **3** | **U** | **K.DVDAAYMGRMDLHGK.V + Oxidation (M)** |

  


---

|  |  |
| --- | --- |
| **44.** | sp|Q04837|SSBP\_HUMAN    **Mass:** 17249    **Score:** 165    **Matches:** 7(6)  **Sequences:** 5(4)  **emPAI:** 1.45 |
|  | Single-stranded DNA-binding protein, mitochondrial OS=Homo sapiens OX=9606 GN=SSBP1 PE=1 SV=1 |

|  |  |
| --- | --- |
|  | Check to include this hit in error tolerant search or archive report |
|  |  |

|  |  |  |  |  |  |  |  |  |  |  |  |
| --- | --- | --- | --- | --- | --- | --- | --- | --- | --- | --- | --- |
|  | **Query** | **Observed** | **Mr(expt)** | **Mr(calc)** | **ppm** | **Miss** | **Score** | **Expect** | **Rank** | **Unique** | **Peptide** |
|  | 500 | **442.2515** | **882.4885** | **882.4923** | **-4.33** | **0** | **59** | **1.1e-005** | **1** | **U** | **R.VGQDPVLR.Q** |
|  | 829 | **493.2533** | **984.4920** | **984.4916** | **0.42** | **0** | **15** | **0.35** | **1** | **U** | **R.DVAYQYVK.K** |
|  | 1525 | **567.2448** | **1132.4751** | **1132.4747** | **0.36** | **0** | **30** | **0.0032** | **1** | **U** | **K.IDYGEYMDK.N** |
|  | 1600 | **575.2424** | **1148.4702** | **1148.4696** | **0.53** | **0** | **(20)** | **0.025** | **1** | **U** | **K.IDYGEYMDK.N + Oxidation (M)** |
|  | 3295 | **806.3777** | **1610.7409** | **1610.7424** | **-0.90** | **0** | **(42)** | **0.00059** | **1** | **U** | **R.SGDSEVYQLGDVSQK.T** |
|  | 3296 | **806.3789** | **1610.7433** | **1610.7424** | **0.54** | **0** | **73** | **4.3e-007** | **1** | **U** | **R.SGDSEVYQLGDVSQK.T** |
|  | 3333 | **816.8662** | **1631.7179** | **1631.7249** | **-4.34** | **1** | **40** | **0.00051** | **1** | **U** | **K.IDYGEYMDKNNVR.R + Oxidation (M)** |

  


---

|  |  |
| --- | --- |
| **45.** | sp|Q01546|K22O\_HUMAN    **Mass:** 66370    **Score:** 161    **Matches:** 11(9)  **Sequences:** 6(5)  **emPAI:** 0.40 |
|  | Keratin, type II cytoskeletal 2 oral OS=Homo sapiens OX=9606 GN=KRT76 PE=1 SV=2 |

|  |  |
| --- | --- |
|  | Check to include this hit in error tolerant search or archive report |
|  |  |

|  |  |  |  |  |  |  |  |  |  |  |  |
| --- | --- | --- | --- | --- | --- | --- | --- | --- | --- | --- | --- |
|  | **Query** | **Observed** | **Mr(expt)** | **Mr(calc)** | **ppm** | **Miss** | **Score** | **Expect** | **Rank** | **Unique** | **Peptide** |
|  | 236 | 414.2182 | 826.4219 | 826.4225 | -0.72 | 0 | 40 | 0.0012 | 1 |  | K.FASFIDK.V |
|  | 237 | 414.2184 | 826.4223 | 826.4225 | -0.28 | 0 | (38) | 0.0019 | 1 |  | K.FASFIDK.V |
|  | 983 | 513.7317 | 1025.4488 | 1025.4488 | 0.04 | 0 | 17 | 0.12 | 2 |  | K.DVDAAFMNK.V + Oxidation (M) |
|  | 1276 | 361.5378 | 1081.5916 | 1081.5920 | -0.39 | 1 | (29) | 0.014 | 1 |  | K.FASFIDKVR.F |
|  | 1277 | 541.8041 | 1081.5937 | 1081.5920 | 1.57 | 1 | 44 | 0.00038 | 1 |  | K.FASFIDKVR.F |
|  | 1396 | 554.2670 | 1106.5194 | 1106.5356 | -14.65 | 0 | (3) | 6.8 | 2 |  | R.AQYEEIAQR.S |
|  | 1398 | 554.2739 | 1106.5333 | 1106.5356 | -2.07 | 0 | 40 | 0.0016 | 1 |  | R.AQYEEIAQR.S |
|  | 1555 | 570.2736 | 1138.5327 | 1138.5328 | -0.13 | 0 | 37 | 0.0026 | 1 |  | R.DYQELMNVK.L |
|  | 1625 | 578.2707 | 1154.5268 | 1154.5278 | -0.80 | 0 | (23) | 0.044 | 1 |  | R.DYQELMNVK.L + Oxidation (M) |
|  | 2962 | 738.8926 | 1475.7706 | 1475.7984 | -18.81 | 1 | (34) | 0.0064 | 1 |  | R.FLEQQNKVLETK.W |
|  | 2963 | 738.8928 | 1475.7711 | 1475.7984 | -18.49 | 1 | 58 | 2.3e-005 | 1 |  | R.FLEQQNKVLETK.W |

  


---

|  |  |
| --- | --- |
| **46.** | sp|Q9UHD2|TBK1\_HUMAN    **Mass:** 84216    **Score:** 160    **Matches:** 7(5)  **Sequences:** 6(5)  **emPAI:** 0.21 |
|  | Serine/threonine-protein kinase TBK1 OS=Homo sapiens OX=9606 GN=TBK1 PE=1 SV=1 |

|  |  |
| --- | --- |
|  | Check to include this hit in error tolerant search or archive report |
|  |  |

|  |  |  |  |  |  |  |  |  |  |  |  |
| --- | --- | --- | --- | --- | --- | --- | --- | --- | --- | --- | --- |
|  | **Query** | **Observed** | **Mr(expt)** | **Mr(calc)** | **ppm** | **Miss** | **Score** | **Expect** | **Rank** | **Unique** | **Peptide** |
|  | 934 | **506.7764** | **1011.5382** | **1011.5389** | **-0.64** | **1** | **14** | **0.46** | **1** | **U** | **K.KYEAFLNK.S** |
|  | 1813 | **597.8043** | **1193.5940** | **1193.5928** | **0.96** | **0** | **40** | **0.0015** | **1** | **U** | **R.VIGEDGQSVYK.L** |
|  | 2292 | **655.3353** | **1308.6560** | **1308.6561** | **-0.11** | **0** | **54** | **5.3e-005** | **1** | **U** | **K.LFAIEEETTTR.H** |
|  | 2634 | **696.3604** | **1390.7061** | **1390.7092** | **-2.23** | **0** | **74** | **5.6e-007** | **1** | **U** | **K.TTEENPIFVVSR.E** |
|  | 2910 | **728.4288** | **1454.8430** | **1454.8457** | **-1.85** | **0** | **(5)** | **1.3** | **2** | **U** | **K.IITGKPSGAISGVQK.A** |
|  | 2911 | **485.9563** | **1454.8470** | **1454.8457** | **0.90** | **0** | **27** | **0.0089** | **1** | **U** | **K.IITGKPSGAISGVQK.A** |
|  | 3077 | **761.3997** | **1520.7848** | **1520.7834** | **0.88** | **0** | **49** | **0.00021** | **1** | **U** | **K.IISSNQELIYEGR.R** |

  


---

|  |  |
| --- | --- |
| **47.** | sp|Q9BQE3|TBA1C\_HUMAN    **Mass:** 50548    **Score:** 158    **Matches:** 9(8)  **Sequences:** 7(7)  **emPAI:** 0.56 |
|  | Tubulin alpha-1C chain OS=Homo sapiens OX=9606 GN=TUBA1C PE=1 SV=1 |

|  |  |
| --- | --- |
|  | Check to include this hit in error tolerant search or archive report |
|  |  |

|  |  |  |  |  |  |  |  |  |  |  |  |
| --- | --- | --- | --- | --- | --- | --- | --- | --- | --- | --- | --- |
|  | **Query** | **Observed** | **Mr(expt)** | **Mr(calc)** | **ppm** | **Miss** | **Score** | **Expect** | **Rank** | **Unique** | **Peptide** |
|  | 946 | **508.2933** | **1014.5720** | **1014.5709** | **1.09** | **0** | **35** | **0.0061** | **1** |  | **K.DVNAAIATIK.T** |
|  | 1294 | **543.3131** | **1084.6117** | **1084.6128** | **-1.05** | **0** | **34** | **0.0053** | **1** | **U** | **K.EIIDLVLDR.I** |
|  | 1295 | **543.3138** | **1084.6130** | **1084.6128** | **0.18** | **0** | **(33)** | **0.0063** | **1** | **U** | **K.EIIDLVLDR.I** |
|  | 2042 | **625.2817** | **1248.5489** | **1248.5453** | **2.88** | **0** | **52** | **4.4e-005** | **1** | **U** | **K.YMACCLLYR.G** |
|  | 2572 | **460.9039** | **1379.6900** | **1379.6907** | **-0.56** | **1** | **36** | **0.0036** | **1** | **U** | **R.LDHKFDLMYAK.R** |
|  | 3487 | **851.4566** | **1700.8986** | **1700.8985** | **0.08** | **0** | **34** | **0.0047** | **1** | **U** | **R.AVFVDLEPTVIDEVR.T** |
|  | 3516 | **859.9440** | **1717.8734** | **1717.8747** | **-0.77** | **0** | **26** | **0.04** | **1** |  | **R.NLDIERPTYTNLNR.L** |
|  | 3517 | **573.6319** | **1717.8739** | **1717.8747** | **-0.49** | **0** | **(2)** | **8.8** | **3** |  | **R.NLDIERPTYTNLNR.L** |
|  | 4090 | **1004.4489** | **2006.8833** | **2006.8858** | **-1.26** | **0** | **49** | **7.1e-005** | **1** |  | **K.TIGGGDDSFNTFFSETGAGK.H** |

  

|  |  |
| --- | --- |
|  | |
|  | **Proteins matching the same set of peptides:** |

|  |  |
| --- | --- |
|  | sp|Q71U36|TBA1A\_HUMAN    **Mass:** 50788    **Score:** 158    **Matches:** 9(8)  **Sequences:** 7(7) |
|  | Tubulin alpha-1A chain OS=Homo sapiens OX=9606 GN=TUBA1A PE=1 SV=1 |

|  |  |
| --- | --- |
|  | sp|P68363|TBA1B\_HUMAN    **Mass:** 50804    **Score:** 158    **Matches:** 9(8)  **Sequences:** 7(7) |
|  | Tubulin alpha-1B chain OS=Homo sapiens OX=9606 GN=TUBA1B PE=1 SV=1 |

---

|  |  |
| --- | --- |
| **48.** | sp|P81605|DCD\_HUMAN    **Mass:** 11391    **Score:** 155    **Matches:** 4(4)  **Sequences:** 2(2)  **emPAI:** 1.20 |
|  | Dermcidin OS=Homo sapiens OX=9606 GN=DCD PE=1 SV=2 |

|  |  |
| --- | --- |
|  | Check to include this hit in error tolerant search or archive report |
|  |  |

|  |  |  |  |  |  |  |  |  |  |  |  |
| --- | --- | --- | --- | --- | --- | --- | --- | --- | --- | --- | --- |
|  | **Query** | **Observed** | **Mr(expt)** | **Mr(calc)** | **ppm** | **Miss** | **Score** | **Expect** | **Rank** | **Unique** | **Peptide** |
|  | 1651 | **581.2849** | **1160.5553** | **1160.5561** | **-0.71** | **0** | **(44)** | **0.00059** | **1** | **U** | **K.DAVEDLESVGK.G** |
|  | 1652 | **581.2851** | **1160.5556** | **1160.5561** | **-0.38** | **0** | **55** | **4.9e-005** | **1** | **U** | **K.DAVEDLESVGK.G** |
|  | 2918 | **730.3853** | **1458.7559** | **1458.7566** | **-0.43** | **1** | **80** | **1.6e-007** | **1** | **U** | **K.LGKDAVEDLESVGK.G** |
|  | 2919 | **487.2594** | **1458.7564** | **1458.7566** | **-0.14** | **1** | **(46)** | **0.00035** | **1** | **U** | **K.LGKDAVEDLESVGK.G** |

  


---

|  |  |
| --- | --- |
| **49.** | sp|P61978|HNRPK\_HUMAN    **Mass:** 51230    **Score:** 148    **Matches:** 5(4)  **Sequences:** 5(4)  **emPAI:** 0.28 |
|  | Heterogeneous nuclear ribonucleoprotein K OS=Homo sapiens OX=9606 GN=HNRNPK PE=1 SV=1 |

|  |  |
| --- | --- |
|  | Check to include this hit in error tolerant search or archive report |
|  |  |

|  |  |  |  |  |  |  |  |  |  |  |  |
| --- | --- | --- | --- | --- | --- | --- | --- | --- | --- | --- | --- |
|  | **Query** | **Observed** | **Mr(expt)** | **Mr(calc)** | **ppm** | **Miss** | **Score** | **Expect** | **Rank** | **Unique** | **Peptide** |
|  | 1151 | **351.8852** | **1052.6337** | **1052.6342** | **-0.55** | **0** | **40** | **0.00029** | **1** | **U** | **R.VVLIGGKPDR.V** |
|  | 1356 | **549.7305** | **1097.4464** | **1097.4448** | **1.47** | **0** | **45** | **6.9e-005** | **1** | **U** | **K.GSDFDCELR.L** |
|  | 2092 | **630.2911** | **1258.5676** | **1258.5677** | **-0.08** | **0** | **91** | **5.4e-009** | **1** | **U** | **K.IDEPLEGSEDR.I** |
|  | 3590 | **584.6044** | **1750.7913** | **1750.7944** | **-1.78** | **1** | **25** | **0.026** | **1** | **U** | **K.RPAEDMEEEQAFKR.S + Oxidation (M)** |
|  | 3662 | **890.9013** | **1779.7881** | **1779.7911** | **-1.72** | **0** | **7** | **1.6** | **1** | **U** | **R.TDYNASVSVPDSSGPER.I** |

  


---

|  |  |
| --- | --- |
| **50.** | sp|Q9H4B7|TBB1\_HUMAN    **Mass:** 50865    **Score:** 148    **Matches:** 9(6)  **Sequences:** 4(3)  **emPAI:** 0.29 |
|  | Tubulin beta-1 chain OS=Homo sapiens OX=9606 GN=TUBB1 PE=1 SV=1 |

|  |  |
| --- | --- |
|  | Check to include this hit in error tolerant search or archive report |
|  |  |

|  |  |  |  |  |  |  |  |  |  |  |  |
| --- | --- | --- | --- | --- | --- | --- | --- | --- | --- | --- | --- |
|  | **Query** | **Observed** | **Mr(expt)** | **Mr(calc)** | **ppm** | **Miss** | **Score** | **Expect** | **Rank** | **Unique** | **Peptide** |
|  | 1509 | 565.8023 | 1129.5901 | 1129.5880 | 1.83 | 0 | 53 | 8.7e-005 | 1 |  | R.FPGQLNADLR.K |
|  | 1510 | 565.8048 | 1129.5949 | 1129.5880 | 6.15 | 0 | (38) | 0.0036 | 1 |  | R.FPGQLNADLR.K |
|  | 1583 | 572.3204 | 1142.6263 | 1142.6270 | -0.63 | 0 | 51 | 0.00014 | 1 |  | K.LAVNMVPFPR.L |
|  | 1584 | 572.3210 | 1142.6275 | 1142.6270 | 0.44 | 0 | (30) | 0.018 | 1 |  | K.LAVNMVPFPR.L |
|  | 1641 | 580.3174 | 1158.6203 | 1158.6219 | -1.40 | 0 | (25) | 0.065 | 1 |  | K.LAVNMVPFPR.L + Oxidation (M) |
|  | 1642 | 580.3181 | 1158.6217 | 1158.6219 | -0.22 | 0 | (29) | 0.029 | 1 |  | K.LAVNMVPFPR.L + Oxidation (M) |
|  | 2133 | 636.3674 | 1270.7203 | 1270.7220 | -1.32 | 1 | 50 | 9.7e-005 | 1 |  | R.KLAVNMVPFPR.L |
|  | 4386 | **756.7139** | **2267.1198** | **2267.1368** | **-7.52** | **0** | **(2)** | **6.1** | **1** | **U** | **R.GLSMAATFIGNNTAIQEIFNR.V** |
|  | 4388 | **1134.5681** | **2267.1217** | **2267.1368** | **-6.68** | **0** | **3** | **5.5** | **1** | **U** | **R.GLSMAATFIGNNTAIQEIFNR.V** |

  


---

|  |  |
| --- | --- |
| **51.** | sp|Q14CN4|K2C72\_HUMAN    **Mass:** 56470    **Score:** 148    **Matches:** 9(8)  **Sequences:** 5(4)  **emPAI:** 0.33 |
|  | Keratin, type II cytoskeletal 72 OS=Homo sapiens OX=9606 GN=KRT72 PE=1 SV=2 |

|  |  |
| --- | --- |
|  | Check to include this hit in error tolerant search or archive report |
|  |  |

|  |  |  |  |  |  |  |  |  |  |  |  |
| --- | --- | --- | --- | --- | --- | --- | --- | --- | --- | --- | --- |
|  | **Query** | **Observed** | **Mr(expt)** | **Mr(calc)** | **ppm** | **Miss** | **Score** | **Expect** | **Rank** | **Unique** | **Peptide** |
|  | 236 | 414.2182 | 826.4219 | 826.4225 | -0.72 | 0 | 40 | 0.0012 | 1 |  | K.FASFIDK.V |
|  | 237 | 414.2184 | 826.4223 | 826.4225 | -0.28 | 0 | (38) | 0.0019 | 1 |  | K.FASFIDK.V |
|  | 983 | 513.7317 | 1025.4488 | 1025.4488 | 0.05 | 0 | 31 | 0.0054 | 1 |  | K.DVDAAYMNK.V |
|  | 1028 | **517.2855** | **1032.5564** | **1032.5386** | **17.2** | **1** | **3** | **8.2** | **7** |  | **K.EELARMLR.E + Oxidation (M)** |
|  | 1276 | 361.5378 | 1081.5916 | 1081.5920 | -0.39 | 1 | (29) | 0.014 | 1 |  | K.FASFIDKVR.F |
|  | 1277 | 541.8041 | 1081.5937 | 1081.5920 | 1.57 | 1 | 44 | 0.00038 | 1 |  | K.FASFIDKVR.F |
|  | 2961 | **738.8900** | **1475.7653** | **1475.7620** | **2.27** | **0** | **(35)** | **0.0053** | **1** |  | **R.FLEQQNQVLETK.W** |
|  | 2962 | 738.8926 | 1475.7706 | 1475.7620 | 5.84 | 0 | (34) | 0.0064 | 1 |  | R.FLEQQNQVLETK.W |
|  | 2963 | 738.8928 | 1475.7711 | 1475.7620 | 6.16 | 0 | 58 | 2.3e-005 | 1 |  | R.FLEQQNQVLETK.W |

  


---

|  |  |
| --- | --- |
| **52.** | sp|Q7RTS7|K2C74\_HUMAN    **Mass:** 58229    **Score:** 145    **Matches:** 10(8)  **Sequences:** 6(4)  **emPAI:** 0.32 |
|  | Keratin, type II cytoskeletal 74 OS=Homo sapiens OX=9606 GN=KRT74 PE=1 SV=2 |

|  |  |
| --- | --- |
|  | Check to include this hit in error tolerant search or archive report |
|  |  |

|  |  |  |  |  |  |  |  |  |  |  |  |
| --- | --- | --- | --- | --- | --- | --- | --- | --- | --- | --- | --- |
|  | **Query** | **Observed** | **Mr(expt)** | **Mr(calc)** | **ppm** | **Miss** | **Score** | **Expect** | **Rank** | **Unique** | **Peptide** |
|  | 236 | 414.2182 | 826.4219 | 826.4225 | -0.72 | 0 | 40 | 0.0012 | 1 |  | K.FASFIDK.V |
|  | 237 | 414.2184 | 826.4223 | 826.4225 | -0.28 | 0 | (38) | 0.0019 | 1 |  | K.FASFIDK.V |
|  | 1028 | 517.2855 | 1032.5564 | 1032.5386 | 17.2 | 1 | 3 | 8.2 | 7 |  | K.EELARMLR.E + Oxidation (M) |
|  | 1276 | 361.5378 | 1081.5916 | 1081.5920 | -0.39 | 1 | (29) | 0.014 | 1 |  | K.FASFIDKVR.F |
|  | 1277 | 541.8041 | 1081.5937 | 1081.5920 | 1.57 | 1 | 44 | 0.00038 | 1 |  | K.FASFIDKVR.F |
|  | 1475 | 561.7990 | 1121.5835 | 1121.5941 | -9.51 | 1 | 2 | 11 | 10 | U | R.SLYSLGGNRR.I |
|  | 1477 | 562.2861 | 1122.5577 | 1122.5557 | 1.82 | 0 | 31 | 0.011 | 1 |  | K.AEAEALYQTK.I |
|  | 2961 | 738.8900 | 1475.7653 | 1475.7620 | 2.27 | 0 | (35) | 0.0053 | 1 |  | R.FLEQQNQVLETK.W |
|  | 2962 | 738.8926 | 1475.7706 | 1475.7620 | 5.84 | 0 | (34) | 0.0064 | 1 |  | R.FLEQQNQVLETK.W |
|  | 2963 | 738.8928 | 1475.7711 | 1475.7620 | 6.16 | 0 | 58 | 2.3e-005 | 1 |  | R.FLEQQNQVLETK.W |

  


---

|  |  |
| --- | --- |
| **53.** | sp|P17661|DESM\_HUMAN    **Mass:** 53560    **Score:** 128    **Matches:** 7(4)  **Sequences:** 6(3)  **emPAI:** 0.27 |
|  | Desmin OS=Homo sapiens OX=9606 GN=DES PE=1 SV=3 |

|  |  |
| --- | --- |
|  | Check to include this hit in error tolerant search or archive report |
|  |  |

|  |  |  |  |  |  |  |  |  |  |  |  |
| --- | --- | --- | --- | --- | --- | --- | --- | --- | --- | --- | --- |
|  | **Query** | **Observed** | **Mr(expt)** | **Mr(calc)** | **ppm** | **Miss** | **Score** | **Expect** | **Rank** | **Unique** | **Peptide** |
|  | 898 | 500.7870 | 999.5594 | 999.5600 | -0.60 | 0 | 6 | 4.1 | 5 | U | K.LQEEIQLK.E |
|  | 1444 | 558.2887 | 1114.5628 | 1114.5618 | 0.91 | 0 | 45 | 0.00044 | 1 |  | K.VELQELNDR.F |
|  | 1468 | 561.2958 | 1120.5770 | 1120.5764 | 0.53 | 0 | 30 | 0.016 | 1 |  | R.EYQDLLNVK.M |
|  | 2099 | **631.3443** | **1260.6740** | **1260.6674** | **5.31** | **1** | **3** | **7.9** | **7** | **U** | **K.SKVSDLTQAANK.N** |
|  | 3262 | 529.9375 | 1586.7907 | 1586.7900 | 0.44 | 1 | (59) | 1.9e-005 | 1 |  | R.TNEKVELQELNDR.F |
|  | 3263 | 794.4044 | 1586.7943 | 1586.7900 | 2.72 | 1 | 67 | 3.1e-006 | 1 |  | R.TNEKVELQELNDR.F |
|  | 3544 | **867.4427** | **1732.8708** | **1732.8631** | **4.44** | **1** | **7** | **3.1** | **3** | **U** | **R.EPTRVAELYEEELR.E** |

  


---

|  |  |
| --- | --- |
| **54.** | sp|P35637|FUS\_HUMAN    **Mass:** 53622    **Score:** 125    **Matches:** 5(5)  **Sequences:** 3(3)  **emPAI:** 0.20 |
|  | RNA-binding protein FUS OS=Homo sapiens OX=9606 GN=FUS PE=1 SV=1 |

|  |  |
| --- | --- |
|  | Check to include this hit in error tolerant search or archive report |
|  |  |

|  |  |  |  |  |  |  |  |  |  |  |  |
| --- | --- | --- | --- | --- | --- | --- | --- | --- | --- | --- | --- |
|  | **Query** | **Observed** | **Mr(expt)** | **Mr(calc)** | **ppm** | **Miss** | **Score** | **Expect** | **Rank** | **Unique** | **Peptide** |
|  | 972 | 511.7507 | 1021.4868 | 1021.4869 | -0.04 | 0 | 45 | 0.00039 | 1 |  | K.AAIDWFDGK.E |
|  | 973 | 511.7511 | 1021.4876 | 1021.4869 | 0.74 | 0 | (42) | 0.00094 | 1 |  | K.AAIDWFDGK.E |
|  | 2744 | 710.8338 | 1419.6530 | 1419.6518 | 0.88 | 0 | 53 | 4.5e-005 | 1 |  | K.GEATVSFDDPPSAK.A |
|  | 2745 | 710.8339 | 1419.6533 | 1419.6518 | 1.05 | 0 | (51) | 7.1e-005 | 1 |  | K.GEATVSFDDPPSAK.A |
|  | 4375 | **563.9987** | **2251.9658** | **2251.9665** | **-0.33** | **0** | **19** | **0.034** | **1** | **U** | **K.APKPDGPGGGPGGSHMGGNYGDDR.R** |

  


---

|  |  |
| --- | --- |
| **55.** | sp|Q562R1|ACTBL\_HUMAN    **Mass:** 42318    **Score:** 118    **Matches:** 10(8)  **Sequences:** 4(4)  **emPAI:** 0.57 |
|  | Beta-actin-like protein 2 OS=Homo sapiens OX=9606 GN=ACTBL2 PE=1 SV=2 |

|  |  |
| --- | --- |
|  | Check to include this hit in error tolerant search or archive report |
|  |  |

|  |  |  |  |  |  |  |  |  |  |  |  |
| --- | --- | --- | --- | --- | --- | --- | --- | --- | --- | --- | --- |
|  | **Query** | **Observed** | **Mr(expt)** | **Mr(calc)** | **ppm** | **Miss** | **Score** | **Expect** | **Rank** | **Unique** | **Peptide** |
|  | 142 | 398.2396 | 794.4647 | 794.4650 | -0.41 | 0 | 27 | 0.016 | 1 |  | K.IIAPPER.K |
|  | 888 | 499.7467 | 997.4789 | 997.4790 | -0.11 | 0 | 27 | 0.025 | 1 |  | R.DLTDYLMK.I |
|  | 889 | 499.7469 | 997.4793 | 997.4790 | 0.27 | 0 | (19) | 0.13 | 1 |  | R.DLTDYLMK.I |
|  | 938 | 507.7443 | 1013.4740 | 1013.4739 | 0.09 | 0 | (26) | 0.024 | 1 |  | R.DLTDYLMK.I + Oxidation (M) |
|  | 939 | 507.7447 | 1013.4748 | 1013.4739 | 0.82 | 0 | (5) | 2.9 | 1 |  | R.DLTDYLMK.I + Oxidation (M) |
|  | 1703 | 586.2890 | 1170.5634 | 1170.5638 | -0.30 | 0 | 71 | 9.3e-007 | 1 |  | R.HQGVMVGMGQK.D |
|  | 4040 | 652.0263 | 1953.0571 | 1953.0571 | 0.00 | 0 | (25) | 0.021 | 2 | U | R.VAPDEHPILLTEAPLNPK.I |
|  | 4041 | 652.0264 | 1953.0573 | 1953.0571 | 0.09 | 0 | (22) | 0.043 | 2 | U | R.VAPDEHPILLTEAPLNPK.I |
|  | 4042 | 977.5362 | 1953.0578 | 1953.0571 | 0.37 | 0 | (32) | 0.0048 | 2 | U | R.VAPDEHPILLTEAPLNPK.I |
|  | 4043 | 977.5367 | 1953.0588 | 1953.0571 | 0.88 | 0 | 35 | 0.0021 | 2 | U | R.VAPDEHPILLTEAPLNPK.I |

  


---

|  |  |
| --- | --- |
| **56.** | sp|O00622|CCN1\_HUMAN    **Mass:** 44165    **Score:** 114    **Matches:** 5(2)  **Sequences:** 5(2)  **emPAI:** 0.24 |
|  | CCN family member 1 OS=Homo sapiens OX=9606 GN=CCN1 PE=1 SV=1 |

|  |  |
| --- | --- |
|  | Check to include this hit in error tolerant search or archive report |
|  |  |

|  |  |  |  |  |  |  |  |  |  |  |  |
| --- | --- | --- | --- | --- | --- | --- | --- | --- | --- | --- | --- |
|  | **Query** | **Observed** | **Mr(expt)** | **Mr(calc)** | **ppm** | **Miss** | **Score** | **Expect** | **Rank** | **Unique** | **Peptide** |
|  | 623 | **464.7548** | **927.4951** | **927.4960** | **-1.02** | **0** | **9** | **1.5** | **2** | **U** | **K.CAPGVGLVR.D** |
|  | 1716 | **587.3348** | **1172.6550** | **1172.6553** | **-0.28** | **0** | **21** | **0.1** | **1** | **U** | **R.ILYNPLQGQK.C** |
|  | 1851 | **601.3290** | **1200.6435** | **1200.6437** | **-0.17** | **1** | **17** | **0.3** | **1** | **U** | **K.RLPVFGMEPR.I** |
|  | 2905 | **727.8511** | **1453.6877** | **1453.6871** | **0.42** | **0** | **69** | **1.4e-006** | **1** | **U** | **K.GLECNFGASSTALK.G** |
|  | 2931 | **733.3633** | **1464.7121** | **1464.7096** | **1.71** | **0** | **67** | **2.8e-006** | **1** | **U** | **K.ELGFDASEVELTR.N** |

  


---

|  |  |
| --- | --- |
| **57.** | sp|P35900|K1C20\_HUMAN    **Mass:** 48514    **Score:** 113    **Matches:** 4(3)  **Sequences:** 3(2)  **emPAI:** 0.22 |
|  | Keratin, type I cytoskeletal 20 OS=Homo sapiens OX=9606 GN=KRT20 PE=1 SV=1 |

|  |  |
| --- | --- |
|  | Check to include this hit in error tolerant search or archive report |
|  |  |

|  |  |  |  |  |  |  |  |  |  |  |  |
| --- | --- | --- | --- | --- | --- | --- | --- | --- | --- | --- | --- |
|  | **Query** | **Observed** | **Mr(expt)** | **Mr(calc)** | **ppm** | **Miss** | **Score** | **Expect** | **Rank** | **Unique** | **Peptide** |
|  | 1473 | 561.7929 | 1121.5713 | 1121.5717 | -0.35 | 0 | 45 | 0.00066 | 1 |  | R.LEQEIATYR.R |
|  | 2566 | 460.5807 | 1378.7202 | 1378.7204 | -0.21 | 1 | 60 | 1.7e-005 | 1 |  | K.TRLEQEIATYR.R |
|  | 2567 | 690.3676 | 1378.7207 | 1378.7204 | 0.16 | 1 | (51) | 0.00011 | 1 |  | K.TRLEQEIATYR.R |
|  | 2881 | **483.5779** | **1447.7120** | **1447.6956** | **11.3** | **1** | **11** | **1.4** | **1** | **U** | **K.QWYETNAPRAGR.D** |

  


---

|  |  |
| --- | --- |
| **58.** | sp|Q07954|LRP1\_HUMAN    **Mass:** 523150   **Score:** 99     **Matches:** 11(4)  **Sequences:** 8(4)  **emPAI:** 0.03 |
|  | Prolow-density lipoprotein receptor-related protein 1 OS=Homo sapiens OX=9606 GN=LRP1 PE=1 SV=2 |

|  |  |
| --- | --- |
|  | Check to include this hit in error tolerant search or archive report |
|  |  |

|  |  |  |  |  |  |  |  |  |  |  |  |
| --- | --- | --- | --- | --- | --- | --- | --- | --- | --- | --- | --- |
|  | **Query** | **Observed** | **Mr(expt)** | **Mr(calc)** | **ppm** | **Miss** | **Score** | **Expect** | **Rank** | **Unique** | **Peptide** |
|  | 1021 | **516.7737** | **1031.5329** | **1031.5321** | **0.78** | **0** | **29** | **0.025** | **1** | **U** | **R.DVIEVAQMK.G** |
|  | 1029 | **517.7584** | **1033.5022** | **1033.5015** | **0.64** | **0** | **13** | **0.87** | **1** | **U** | **R.CLPGFLGDR.C** |
|  | 1551 | **380.2242** | **1137.6508** | **1137.6506** | **0.17** | **0** | **35** | **0.0019** | **1** | **U** | **R.GVTHLNISGLK.M** |
|  | 1552 | **569.8329** | **1137.6513** | **1137.6506** | **0.65** | **0** | **(11)** | **0.45** | **1** | **U** | **R.GVTHLNISGLK.M** |
|  | 1728 | **589.2989** | **1176.5833** | **1176.5809** | **2.12** | **0** | **(12)** | **1.2** | **1** | **U** | **K.IETAAMDGTLR.E** |
|  | 1804 | **597.2947** | **1192.5749** | **1192.5758** | **-0.71** | **0** | **53** | **7e-005** | **1** | **U** | **K.IETAAMDGTLR.E + Oxidation (M)** |
|  | 2421 | **671.7857** | **1341.5569** | **1341.5619** | **-3.75** | **0** | **0** | **2.3** | **5** | **U** | **K.SDEKPSYCNSR.R** |
|  | 3235 | **789.8433** | **1577.6721** | **1577.6722** | **-0.04** | **0** | **45** | **0.00014** | **1** | **U** | **R.GTMYWSDWGNHPK.I** |
|  | 4502 | 826.3445 | 2476.0118 | 2475.9690 | 17.3 | 1 | 2 | 0.69 | 3 | U | K.DDGRTCADVDECSTTFPCSQR.C |
|  | 4679 | **779.8409** | **3115.3347** | **3115.3943** | **-19.16** | **0** | **(0)** | **1.6** | **2** | **U** | **R.EVVLSSNNMDMFSVSVFEDFIYWSDR.T** |
|  | 4681 | **1039.4536** | **3115.3390** | **3115.3943** | **-17.76** | **0** | **0** | **1.6** | **3** | **U** | **R.EVVLSSNNMDMFSVSVFEDFIYWSDR.T** |

  


---

|  |  |
| --- | --- |
| **59.** | sp|Q5T749|KPRP\_HUMAN    **Mass:** 67172    **Score:** 96     **Matches:** 9(4)  **Sequences:** 6(3)  **emPAI:** 0.27 |
|  | Keratinocyte proline-rich protein OS=Homo sapiens OX=9606 GN=KPRP PE=1 SV=1 |

|  |  |
| --- | --- |
|  | Check to include this hit in error tolerant search or archive report |
|  |  |

|  |  |  |  |  |  |  |  |  |  |  |  |
| --- | --- | --- | --- | --- | --- | --- | --- | --- | --- | --- | --- |
|  | **Query** | **Observed** | **Mr(expt)** | **Mr(calc)** | **ppm** | **Miss** | **Score** | **Expect** | **Rank** | **Unique** | **Peptide** |
|  | 113 | **387.2368** | **772.4590** | **772.4595** | **-0.75** | **0** | **(33)** | **0.015** | **1** | **U** | **R.LQLFPR.S** |
|  | 114 | **387.2369** | **772.4591** | **772.4595** | **-0.52** | **0** | **33** | **0.013** | **1** | **U** | **R.LQLFPR.S** |
|  | 1265 | **540.7974** | **1079.5802** | **1079.5797** | **0.39** | **0** | **54** | **5.3e-005** | **1** | **U** | **R.CPVEIPPIR.R** |
|  | 2423 | **671.8241** | **1341.6336** | **1341.6347** | **-0.78** | **0** | **23** | **0.059** | **1** | **U** | **R.LDQCPESPLQR.C** |
|  | 3028 | **500.2499** | **1497.7279** | **1497.7358** | **-5.27** | **1** | **(21)** | **0.097** | **1** | **U** | **R.RLDQCPESPLQR.C** |
|  | 3029 | **749.8740** | **1497.7335** | **1497.7358** | **-1.54** | **1** | **45** | **0.00035** | **1** | **U** | **R.RLDQCPESPLQR.C** |
|  | 4030 | **648.2983** | **1941.8732** | **1941.8929** | **-10.16** | **1** | **(1)** | **5.1** | **3** | **U** | **R.GQDGHGDQGNAFAGVKGEAK.S** |
|  | 4034 | **971.9448** | **1941.8751** | **1941.8929** | **-9.19** | **1** | **3** | **3.5** | **1** | **U** | **R.GQDGHGDQGNAFAGVKGEAK.S** |
|  | 4669 | **1011.4642** | **3031.3709** | **3031.3301** | **13.4** | **1** | **1** | **3.8** | **6** | **U** | **R.TSFSPCVPQCQTQGSYGSFTEQHRSR.S** |

  


---

|  |  |
| --- | --- |
| **60.** | sp|P15924|DESP\_HUMAN    **Mass:** 334021   **Score:** 96     **Matches:** 18(3)  **Sequences:** 14(3)  **emPAI:** 0.04 |
|  | Desmoplakin OS=Homo sapiens OX=9606 GN=DSP PE=1 SV=3 |

|  |  |
| --- | --- |
|  | Check to include this hit in error tolerant search or archive report |
|  |  |

|  |  |  |  |  |  |  |  |  |  |  |  |
| --- | --- | --- | --- | --- | --- | --- | --- | --- | --- | --- | --- |
|  | **Query** | **Observed** | **Mr(expt)** | **Mr(calc)** | **ppm** | **Miss** | **Score** | **Expect** | **Rank** | **Unique** | **Peptide** |
|  | 171 | **402.2186** | **802.4226** | **802.4225** | **0.13** | **0** | **20** | **0.31** | **1** | **U** | **R.LWDLEK.Q** |
|  | 215 | **411.7112** | **821.4078** | **821.3953** | **15.3** | **0** | **0** | **14** | **3** | **U** | **K.MSAAEAVK.E + Oxidation (M)** |
|  | 403 | **431.7554** | **861.4962** | **861.4960** | **0.22** | **0** | **15** | **0.31** | **1** | **U** | **R.GLVGIEFK.E** |
|  | 628 | **465.2744** | **928.5342** | **928.5341** | **0.06** | **0** | **4** | **5.6** | **1** | **U** | **K.NATILELR.S** |
|  | 912 | **503.2720** | **1004.5295** | **1004.5291** | **0.42** | **0** | **6** | **4.5** | **3** | **U** | **K.FLDQNLQK.Y** |
|  | 1088 | **523.2854** | **1044.5562** | **1044.5564** | **-0.13** | **0** | **(0)** | **18** | **4** | **U** | **R.GIVDSITGQR.L** |
|  | 1089 | **523.2855** | **1044.5564** | **1044.5564** | **-0.01** | **0** | **2** | **11** | **4** | **U** | **R.GIVDSITGQR.L** |
|  | 1115 | **523.2911** | **1044.5677** | **1044.5564** | **10.9** | **0** | **(1)** | **13** | **8** | **U** | **R.GIVDSITGQR.L** |
|  | 1387 | **368.8728** | **1103.5967** | **1103.5975** | **-0.73** | **1** | **38** | **0.003** | **1** | **U** | **R.LPVEEAYKR.G** |
|  | 2132 | **636.3570** | **1270.6994** | **1270.6993** | **0.08** | **0** | **40** | **0.0016** | **1** | **U** | **R.QLQNIIQATSR.E** |
|  | 2133 | 636.3674 | 1270.7203 | 1270.6993 | 16.5 | 0 | (0) | 10 | 6 | U | R.QLQNIIQATSR.E |
|  | 2208 | 431.2384 | 1290.6933 | 1290.6680 | 19.6 | 1 | 7 | 2.5 | 10 | U | K.FQKQALEASNR.I |
|  | 2223 | **648.3284** | **1294.6422** | **1294.6405** | **1.34** | **1** | **18** | **0.2** | **1** | **U** | **R.LTYEIEDEKR.R** |
|  | 2718 | **707.8562** | **1413.6978** | **1413.7001** | **-1.56** | **0** | **65** | **4.8e-006** | **1** | **U** | **K.QQIQNDLNQWK.T** |
|  | 3125 | **768.8699** | **1535.7253** | **1535.7038** | **14.0** | **0** | **11** | **0.89** | **1** | **U** | **K.GLPSPYNMSSAPGSR.S + Oxidation (M)** |
|  | 3182 | **776.9294** | **1551.8443** | **1551.8218** | **14.5** | **1** | **4** | **3.6** | **5** | **U** | **R.FLSEMLKSLEDLK.L** |
|  | 3985 | **641.9742** | **1922.9007** | **1922.8905** | **5.33** | **1** | **(2)** | **6.5** | **2** | **U** | **K.GLPSPYNMSSAPGSRSGSR.S + Oxidation (M)** |
|  | 3986 | **641.9746** | **1922.9020** | **1922.8905** | **6.00** | **1** | **2** | **5.6** | **2** | **U** | **K.GLPSPYNMSSAPGSRSGSR.S + Oxidation (M)** |

  


---

|  |  |
| --- | --- |
| **61.** | sp|P19013|K2C4\_HUMAN    **Mass:** 57649    **Score:** 89     **Matches:** 7(5)  **Sequences:** 5(4)  **emPAI:** 0.25 |
|  | Keratin, type II cytoskeletal 4 OS=Homo sapiens OX=9606 GN=KRT4 PE=1 SV=4 |

|  |  |
| --- | --- |
|  | Check to include this hit in error tolerant search or archive report |
|  |  |

|  |  |  |  |  |  |  |  |  |  |  |  |
| --- | --- | --- | --- | --- | --- | --- | --- | --- | --- | --- | --- |
|  | **Query** | **Observed** | **Mr(expt)** | **Mr(calc)** | **ppm** | **Miss** | **Score** | **Expect** | **Rank** | **Unique** | **Peptide** |
|  | 236 | 414.2182 | 826.4219 | 826.4225 | -0.72 | 0 | 40 | 0.0012 | 1 |  | K.FASFIDK.V |
|  | 237 | 414.2184 | 826.4223 | 826.4225 | -0.28 | 0 | (38) | 0.0019 | 1 |  | K.FASFIDK.V |
|  | 1028 | 517.2855 | 1032.5564 | 1032.5386 | 17.2 | 1 | 3 | 8.2 | 7 |  | K.EELARMLR.E + Oxidation (M) |
|  | 1396 | 554.2670 | 1106.5194 | 1106.5356 | -14.65 | 0 | (3) | 6.8 | 2 |  | R.AQYEEIAQR.S |
|  | 1398 | 554.2739 | 1106.5333 | 1106.5356 | -2.07 | 0 | 40 | 0.0016 | 1 |  | R.AQYEEIAQR.S |
|  | 1477 | 562.2861 | 1122.5577 | 1122.5557 | 1.82 | 0 | 31 | 0.011 | 1 |  | K.AEAEALYQTK.V |
|  | 1492 | 563.7764 | 1125.5383 | 1125.5376 | 0.65 | 0 | 29 | 0.017 | 1 |  | R.EYQELMSVK.L |

  


---

|  |  |
| --- | --- |
| **62.** | sp|P34931|HS71L\_HUMAN    **Mass:** 70730    **Score:** 83     **Matches:** 4(3)  **Sequences:** 4(3)  **emPAI:** 0.15 |
|  | Heat shock 70 kDa protein 1-like OS=Homo sapiens OX=9606 GN=HSPA1L PE=1 SV=2 |

|  |  |
| --- | --- |
|  | Check to include this hit in error tolerant search or archive report |
|  |  |

|  |  |  |  |  |  |  |  |  |  |  |  |
| --- | --- | --- | --- | --- | --- | --- | --- | --- | --- | --- | --- |
|  | **Query** | **Observed** | **Mr(expt)** | **Mr(calc)** | **ppm** | **Miss** | **Score** | **Expect** | **Rank** | **Unique** | **Peptide** |
|  | 1832 | **599.3517** | **1196.6888** | **1196.6877** | **0.91** | **0** | **22** | **0.035** | **1** | **U** | **K.DAGVIAGLNVLR.I** |
|  | 1959 | **614.8171** | **1227.6197** | **1227.6207** | **-0.83** | **0** | **50** | **0.00018** | **1** |  | **K.VEIIANDQGNR.T** |
|  | 2987 | **744.3546** | **1486.6945** | **1486.6940** | **0.37** | **0** | **49** | **0.00013** | **1** |  | **R.TTPSYVAFTDTER.L** |
|  | 2997 | 746.3511 | 1490.6877 | 1490.7109 | -15.53 | 0 | 0 | 7.8 | 7 | U | K.ELEQMCNPIITK.L + Oxidation (M) |

  


---

|  |  |
| --- | --- |
| **63.** | sp|P07355|ANXA2\_HUMAN    **Mass:** 38808    **Score:** 83     **Matches:** 6(3)  **Sequences:** 5(3)  **emPAI:** 0.28 |
|  | Annexin A2 OS=Homo sapiens OX=9606 GN=ANXA2 PE=1 SV=2 |

|  |  |
| --- | --- |
|  | Check to include this hit in error tolerant search or archive report |
|  |  |

|  |  |  |  |  |  |  |  |  |  |  |  |
| --- | --- | --- | --- | --- | --- | --- | --- | --- | --- | --- | --- |
|  | **Query** | **Observed** | **Mr(expt)** | **Mr(calc)** | **ppm** | **Miss** | **Score** | **Expect** | **Rank** | **Unique** | **Peptide** |
|  | 484 | **440.7321** | **879.4496** | **879.4338** | **18.0** | **0** | **7** | **3.1** | **2** | **U** | **R.DLYDAGVK.R** |
|  | 1138 | **526.2676** | **1050.5206** | **1050.5168** | **3.62** | **0** | **6** | **3.5** | **1** | **U** | **K.WISIMTER.S + Oxidation (M)** |
|  | 1301 | **544.3027** | **1086.5909** | **1086.5921** | **-1.06** | **0** | **42** | **0.0013** | **1** | **U** | **R.DALNIETAIK.T** |
|  | 1302 | **544.3090** | **1086.6035** | **1086.5921** | **10.5** | **0** | **(9)** | **2.5** | **3** | **U** | **R.DALNIETAIK.T** |
|  | 1926 | **611.8009** | **1221.5872** | **1221.5877** | **-0.38** | **0** | **32** | **0.0084** | **1** | **U** | **K.TPAQYDASELK.A** |
|  | 2030 | **622.8148** | **1243.6151** | **1243.6156** | **-0.45** | **0** | **57** | **3.2e-005** | **1** | **U** | **R.TNQELQEINR.V** |

  


---

|  |  |
| --- | --- |
| **64.** | sp|Q9NSB2|KRT84\_HUMAN    **Mass:** 65942    **Score:** 83     **Matches:** 7(4)  **Sequences:** 5(2)  **emPAI:** 0.16 |
|  | Keratin, type II cuticular Hb4 OS=Homo sapiens OX=9606 GN=KRT84 PE=2 SV=2 |

|  |  |
| --- | --- |
|  | Check to include this hit in error tolerant search or archive report |
|  |  |

|  |  |  |  |  |  |  |  |  |  |  |  |
| --- | --- | --- | --- | --- | --- | --- | --- | --- | --- | --- | --- |
|  | **Query** | **Observed** | **Mr(expt)** | **Mr(calc)** | **ppm** | **Miss** | **Score** | **Expect** | **Rank** | **Unique** | **Peptide** |
|  | 236 | 414.2182 | 826.4219 | 826.4225 | -0.72 | 0 | 40 | 0.0012 | 1 |  | K.FASFIDK.V |
|  | 237 | 414.2184 | 826.4223 | 826.4225 | -0.28 | 0 | (38) | 0.0019 | 1 |  | K.FASFIDK.V |
|  | 765 | 483.2379 | 964.4613 | 964.4614 | -0.10 | 0 | 6 | 4 | 5 | U | K.AQYEEVAR.R |
|  | 983 | 513.7317 | 1025.4488 | 1025.4488 | 0.04 | 0 | 17 | 0.12 | 2 |  | K.DVDAAFMNK.S + Oxidation (M) |
|  | 1276 | 361.5378 | 1081.5916 | 1081.5920 | -0.39 | 1 | (29) | 0.014 | 1 |  | K.FASFIDKVR.F |
|  | 1277 | 541.8041 | 1081.5937 | 1081.5920 | 1.57 | 1 | 44 | 0.00038 | 1 |  | K.FASFIDKVR.F |
|  | 2775 | 713.3407 | 1424.6668 | 1424.6684 | -1.10 | 1 | 2 | 7.6 | 4 | U | R.SRADAEAWYQTK.Y |

  


---

|  |  |
| --- | --- |
| **65.** | sp|P04406|G3P\_HUMAN    **Mass:** 36201    **Score:** 82     **Matches:** 2(2)  **Sequences:** 2(2)  **emPAI:** 0.19 |
|  | Glyceraldehyde-3-phosphate dehydrogenase OS=Homo sapiens OX=9606 GN=GAPDH PE=1 SV=3 |

|  |  |
| --- | --- |
|  | Check to include this hit in error tolerant search or archive report |
|  |  |

|  |  |  |  |  |  |  |  |  |  |  |  |
| --- | --- | --- | --- | --- | --- | --- | --- | --- | --- | --- | --- |
|  | **Query** | **Observed** | **Mr(expt)** | **Mr(calc)** | **ppm** | **Miss** | **Score** | **Expect** | **Rank** | **Unique** | **Peptide** |
|  | 2709 | **706.4030** | **1410.7915** | **1410.7831** | **5.98** | **0** | **41** | **0.00069** | **1** | **U** | **R.GALQNIIPASTGAAK.A** |
|  | 3104 | **765.9009** | **1529.7872** | **1529.7872** | **0.00** | **0** | **62** | **8.3e-006** | **1** | **U** | **R.VPTANVSVVDLTCR.L** |

  


---

|  |  |
| --- | --- |
| **66.** | sp|P11142|HSP7C\_HUMAN    **Mass:** 71082    **Score:** 80     **Matches:** 3(2)  **Sequences:** 3(2)  **emPAI:** 0.09 |
|  | Heat shock cognate 71 kDa protein OS=Homo sapiens OX=9606 GN=HSPA8 PE=1 SV=1 |

|  |  |
| --- | --- |
|  | Check to include this hit in error tolerant search or archive report |
|  |  |

|  |  |  |  |  |  |  |  |  |  |  |  |
| --- | --- | --- | --- | --- | --- | --- | --- | --- | --- | --- | --- |
|  | **Query** | **Observed** | **Mr(expt)** | **Mr(calc)** | **ppm** | **Miss** | **Score** | **Expect** | **Rank** | **Unique** | **Peptide** |
|  | 850 | 495.2728 | 988.5311 | 988.5189 | 12.4 | 1 | 5 | 6.8 | 10 | U | R.LSKEDIER.M |
|  | 1959 | 614.8171 | 1227.6197 | 1227.6207 | -0.83 | 0 | 50 | 0.00018 | 1 |  | K.VEIIANDQGNR.T |
|  | 2987 | 744.3546 | 1486.6945 | 1486.6940 | 0.37 | 0 | 49 | 0.00013 | 1 |  | R.TTPSYVAFTDTER.L |

  


---

|  |  |
| --- | --- |
| **67.** | sp|P17066|HSP76\_HUMAN    **Mass:** 71440    **Score:** 80     **Matches:** 2(2)  **Sequences:** 2(2)  **emPAI:** 0.09 |
|  | Heat shock 70 kDa protein 6 OS=Homo sapiens OX=9606 GN=HSPA6 PE=1 SV=2 |

|  |  |
| --- | --- |
|  | Check to include this hit in error tolerant search or archive report |
|  |  |

|  |  |  |  |  |  |  |  |  |  |  |  |
| --- | --- | --- | --- | --- | --- | --- | --- | --- | --- | --- | --- |
|  | **Query** | **Observed** | **Mr(expt)** | **Mr(calc)** | **ppm** | **Miss** | **Score** | **Expect** | **Rank** | **Unique** | **Peptide** |
|  | 1959 | 614.8171 | 1227.6197 | 1227.6207 | -0.83 | 0 | 50 | 0.00018 | 1 | U | R.VEILANDQGNR.T |
|  | 2987 | 744.3546 | 1486.6945 | 1486.6940 | 0.37 | 0 | 49 | 0.00013 | 1 |  | R.TTPSYVAFTDTER.L |

  

|  |  |
| --- | --- |
|  | |
|  | **Proteins matching the same set of peptides:** |

|  |  |
| --- | --- |
|  | sp|P48741|HSP77\_HUMAN    **Mass:** 40448    **Score:** 80     **Matches:** 2(2)  **Sequences:** 2(2) |
|  | Putative heat shock 70 kDa protein 7 OS=Homo sapiens OX=9606 GN=HSPA7 PE=5 SV=2 |

---

|  |  |
| --- | --- |
| **68.** | sp|P19474|RO52\_HUMAN    **Mass:** 55162    **Score:** 80     **Matches:** 7(3)  **Sequences:** 7(3)  **emPAI:** 0.19 |
|  | E3 ubiquitin-protein ligase TRIM21 OS=Homo sapiens OX=9606 GN=TRIM21 PE=1 SV=1 |

|  |  |
| --- | --- |
|  | Check to include this hit in error tolerant search or archive report |
|  |  |

|  |  |  |  |  |  |  |  |  |  |  |  |
| --- | --- | --- | --- | --- | --- | --- | --- | --- | --- | --- | --- |
|  | **Query** | **Observed** | **Mr(expt)** | **Mr(calc)** | **ppm** | **Miss** | **Score** | **Expect** | **Rank** | **Unique** | **Peptide** |
|  | 700 | **473.7450** | **945.4755** | **945.4742** | **1.37** | **0** | **16** | **0.48** | **1** | **U** | **R.LHLFCEK.D** |
|  | 890 | **499.8032** | **997.5918** | **997.5920** | **-0.18** | **0** | **55** | **2.5e-005** | **1** | **U** | **K.LQVALGELR.R** |
|  | 1388 | **553.2585** | **1104.5024** | **1104.5022** | **0.16** | **0** | **27** | **0.022** | **1** | **U** | **K.EAWDLGVCR.D** |
|  | 1485 | **375.5463** | **1123.6170** | **1123.6172** | **-0.22** | **1** | **2** | **9** | **2** | **U** | **R.SVCHVPGLKK.M** |
|  | 1637 | **579.8038** | **1157.5931** | **1157.5928** | **0.26** | **0** | **13** | **0.87** | **1** | **U** | **K.DLDITSPELR.S** |
|  | 1657 | **582.2878** | **1162.5611** | **1162.5618** | **-0.60** | **0** | **38** | **0.0028** | **1** | **U** | **K.NFLVEEEQR.Q** |
|  | 4687 | **631.0934** | **3150.4305** | **3150.4658** | **-11.20** | **1** | **1** | **3** | **2** | **U** | **R.FDSYPMVLGAQHFHSGKHYWEVDVTGK.E + Oxidation (M)** |

  


---

|  |  |
| --- | --- |
| **69.** | sp|Q2M2I5|K1C24\_HUMAN    **Mass:** 55567    **Score:** 79     **Matches:** 6(3)  **Sequences:** 3(2)  **emPAI:** 0.12 |
|  | Keratin, type I cytoskeletal 24 OS=Homo sapiens OX=9606 GN=KRT24 PE=1 SV=1 |

|  |  |
| --- | --- |
|  | Check to include this hit in error tolerant search or archive report |
|  |  |

|  |  |  |  |  |  |  |  |  |  |  |  |
| --- | --- | --- | --- | --- | --- | --- | --- | --- | --- | --- | --- |
|  | **Query** | **Observed** | **Mr(expt)** | **Mr(calc)** | **ppm** | **Miss** | **Score** | **Expect** | **Rank** | **Unique** | **Peptide** |
|  | 179 | 404.2029 | 806.3913 | 806.3923 | -1.23 | 0 | 41 | 0.0016 | 1 |  | R.LAADDFR.L |
|  | 180 | 404.2037 | 806.3928 | 806.3923 | 0.73 | 0 | (26) | 0.044 | 1 |  | R.LAADDFR.L |
|  | 1285 | **362.1682** | **1083.4826** | **1083.4727** | **9.16** | **1** | **5** | **2.5** | **6** | **U** | **M.SCSSRASSSR.A** |
|  | 1286 | **362.1685** | **1083.4836** | **1083.4727** | **10.1** | **1** | **(3)** | **4** | **4** | **U** | **M.SCSSRASSSR.A** |
|  | 1849 | 601.3110 | 1200.6075 | 1200.6098 | -1.93 | 0 | (4) | 5.7 | 5 |  | R.QSVEADINGLR.K |
|  | 1850 | 601.3121 | 1200.6096 | 1200.6098 | -0.22 | 0 | 58 | 2.6e-005 | 1 |  | R.QSVEADINGLR.K |

  


---

|  |  |
| --- | --- |
| **70.** | sp|P18124|RL7\_HUMAN    **Mass:** 29264    **Score:** 77     **Matches:** 5(2)  **Sequences:** 5(2)  **emPAI:** 0.24 |
|  | 60S ribosomal protein L7 OS=Homo sapiens OX=9606 GN=RPL7 PE=1 SV=1 |

|  |  |
| --- | --- |
|  | Check to include this hit in error tolerant search or archive report |
|  |  |

|  |  |  |  |  |  |  |  |  |  |  |  |
| --- | --- | --- | --- | --- | --- | --- | --- | --- | --- | --- | --- |
|  | **Query** | **Observed** | **Mr(expt)** | **Mr(calc)** | **ppm** | **Miss** | **Score** | **Expect** | **Rank** | **Unique** | **Peptide** |
|  | 24 | 361.1923 | 720.3700 | 720.3806 | -14.70 | 0 | 8 | 3.5 | 5 | U | R.NFAELK.I |
|  | 1278 | **541.8102** | **1081.6059** | **1081.6019** | **3.71** | **0** | **12** | **0.53** | **1** | **U** | **K.EVPAVPETLK.K** |
|  | 1701 | **585.8455** | **1169.6764** | **1169.6768** | **-0.36** | **0** | **48** | **0.00011** | **1** | **U** | **R.IALTDNALIAR.S** |
|  | 1802 | 596.8079 | 1191.6012 | 1191.5924 | 7.35 | 0 | 2 | 11 | 3 | U | K.AGNFYVPAEPK.L |
|  | 4243 | **529.7507** | **2114.9738** | **2114.9730** | **0.40** | **1** | **49** | **9.2e-005** | **1** | **U** | **K.TTHFVEGGDAGNREDQINR.L** |

  


---

|  |  |
| --- | --- |
| **71.** | sp|P27694|RFA1\_HUMAN    **Mass:** 68723    **Score:** 75     **Matches:** 8(2)  **Sequences:** 7(2)  **emPAI:** 0.15 |
|  | Replication protein A 70 kDa DNA-binding subunit OS=Homo sapiens OX=9606 GN=RPA1 PE=1 SV=2 |

|  |  |
| --- | --- |
|  | Check to include this hit in error tolerant search or archive report |
|  |  |

|  |  |  |  |  |  |  |  |  |  |  |  |
| --- | --- | --- | --- | --- | --- | --- | --- | --- | --- | --- | --- |
|  | **Query** | **Observed** | **Mr(expt)** | **Mr(calc)** | **ppm** | **Miss** | **Score** | **Expect** | **Rank** | **Unique** | **Peptide** |
|  | 176 | **403.7028** | **805.3911** | **805.3905** | **0.74** | **0** | **13** | **0.81** | **1** | **U** | **K.WTICAR.V** |
|  | 264 | **417.7582** | **833.5018** | **833.5011** | **0.87** | **0** | **31** | **0.0052** | **1** | **U** | **R.FIVNTLK.D** |
|  | 1392 | **553.8159** | **1105.6172** | **1105.6172** | **-0.00** | **0** | **12** | **0.75** | **1** | **U** | **K.FFPLIEVNK.V** |
|  | 1393 | **553.8159** | **1105.6172** | **1105.6172** | **-0.00** | **0** | **(9)** | **1.3** | **1** | **U** | **K.FFPLIEVNK.V** |
|  | 1872 | **603.3174** | **1204.6203** | **1204.6200** | **0.25** | **0** | **25** | **0.06** | **1** | **U** | **K.VIDQQNGLYR.C** |
|  | 1981 | **616.8313** | **1231.6480** | **1231.6482** | **-0.14** | **0** | **12** | **1.1** | **1** | **U** | **K.DSLVDIIGICK.S** |
|  | 2676 | **701.9004** | **1401.7862** | **1401.7868** | **-0.38** | **0** | **58** | **1.2e-005** | **1** | **U** | **K.VVPIASLTPYQSK.W** |
|  | 3431 | **560.2790** | **1677.8151** | **1677.8430** | **-16.58** | **0** | **8** | **2** | **1** | **U** | **-.MVGQLSEGAIAAIMQK.G + 2 Oxidation (M)** |

  


---

|  |  |
| --- | --- |
| **72.** | sp|P14923|PLAK\_HUMAN    **Mass:** 82434    **Score:** 73     **Matches:** 9(5)  **Sequences:** 9(5)  **emPAI:** 0.22 |
|  | Junction plakoglobin OS=Homo sapiens OX=9606 GN=JUP PE=1 SV=3 |

|  |  |
| --- | --- |
|  | Check to include this hit in error tolerant search or archive report |
|  |  |

|  |  |  |  |  |  |  |  |  |  |  |  |
| --- | --- | --- | --- | --- | --- | --- | --- | --- | --- | --- | --- |
|  | **Query** | **Observed** | **Mr(expt)** | **Mr(calc)** | **ppm** | **Miss** | **Score** | **Expect** | **Rank** | **Unique** | **Peptide** |
|  | 62 | **372.2418** | **742.4690** | **742.4701** | **-1.54** | **0** | **18** | **0.18** | **1** | **U** | **K.ATIGLIR.N** |
|  | 196 | **406.7837** | **811.5528** | **811.5531** | **-0.43** | **0** | **25** | **0.0053** | **1** | **U** | **R.LVQLLVK.A** |
|  | 477 | **438.7505** | **875.4864** | **875.4865** | **-0.08** | **0** | **17** | **0.32** | **1** |  | **K.LLWTTSR.V** |
|  | 902 | **501.7769** | **1001.5392** | **1001.5393** | **-0.08** | **0** | **28** | **0.034** | **1** | **U** | **K.QEGLESVLK.I** |
|  | 1303 | **544.3287** | **1086.6428** | **1086.6437** | **-0.85** | **0** | **11** | **0.65** | **1** | **U** | **R.LNYGIPAIVK.L** |
|  | 1996 | **618.8448** | **1235.6750** | **1235.6761** | **-0.91** | **0** | **34** | **0.0052** | **1** | **U** | **R.VSVELTNSLFK.H** |
|  | 2710 | **706.4095** | **1410.8044** | **1410.8017** | **1.92** | **0** | **30** | **0.0075** | **1** | **U** | **R.ALMGSPQLVAAVVR.T** |
|  | 3275 | **533.9340** | **1598.7801** | **1598.7801** | **-0.05** | **0** | **34** | **0.0047** | **1** | **U** | **R.HVAAGTQQPYTDGVR.M** |
|  | 3942 | **633.9528** | **1898.8366** | **1898.8502** | **-7.16** | **1** | **0** | **5.1** | **4** | **U** | **K.GIMEEDEACGRQYTLK.K** |

  


---

|  |  |
| --- | --- |
| **73.** | sp|O75955|FLOT1\_HUMAN    **Mass:** 47554    **Score:** 71     **Matches:** 1(1)  **Sequences:** 1(1)  **emPAI:** 0.07 |
|  | Flotillin-1 OS=Homo sapiens OX=9606 GN=FLOT1 PE=1 SV=3 |

|  |  |
| --- | --- |
|  | Check to include this hit in error tolerant search or archive report |
|  |  |

|  |  |  |  |  |  |  |  |  |  |  |  |
| --- | --- | --- | --- | --- | --- | --- | --- | --- | --- | --- | --- |
|  | **Query** | **Observed** | **Mr(expt)** | **Mr(calc)** | **ppm** | **Miss** | **Score** | **Expect** | **Rank** | **Unique** | **Peptide** |
|  | 2940 | **735.3887** | **1468.7628** | **1468.7634** | **-0.40** | **0** | **71** | **8.8e-007** | **1** | **U** | **R.AQQVAVQEQEIAR.R** |

  


---

|  |  |
| --- | --- |
| **74.** | sp|P05141|ADT2\_HUMAN    **Mass:** 33059    **Score:** 71     **Matches:** 7(5)  **Sequences:** 5(3)  **emPAI:** 0.61 |
|  | ADP/ATP translocase 2 OS=Homo sapiens OX=9606 GN=SLC25A5 PE=1 SV=7 |

|  |  |
| --- | --- |
|  | Check to include this hit in error tolerant search or archive report |
|  |  |

|  |  |  |  |  |  |  |  |  |  |  |  |
| --- | --- | --- | --- | --- | --- | --- | --- | --- | --- | --- | --- |
|  | **Query** | **Observed** | **Mr(expt)** | **Mr(calc)** | **ppm** | **Miss** | **Score** | **Expect** | **Rank** | **Unique** | **Peptide** |
|  | 401 | **431.2294** | **860.4442** | **860.4426** | **1.85** | **0** | **15** | **0.66** | **1** |  | **R.GLGDCLVK.I** |
|  | 555 | **451.7459** | **901.4773** | **901.4770** | **0.31** | **0** | **44** | **0.001** | **1** |  | **K.GAWSNVLR.G** |
|  | 1466 | **561.2916** | **1120.5687** | **1120.5665** | **1.93** | **0** | **19** | **0.26** | **1** |  | **K.EQGVLSFWR.G** |
|  | 1521 | **378.2213** | **1131.6422** | **1131.6400** | **1.90** | **1** | **(22)** | **0.052** | **1** | **U** | **K.QIFLGGVDKR.T** |
|  | 1522 | **566.8301** | **1131.6456** | **1131.6400** | **4.92** | **1** | **26** | **0.022** | **1** | **U** | **K.QIFLGGVDKR.T** |
|  | 1535 | **379.5640** | **1135.6701** | **1135.6713** | **-1.12** | **0** | **(24)** | **0.012** | **1** |  | **K.LLLQVQHASK.Q** |
|  | 1536 | **568.8431** | **1135.6716** | **1135.6713** | **0.25** | **0** | **35** | **0.00093** | **1** |  | **K.LLLQVQHASK.Q** |

  


---

|  |  |
| --- | --- |
| **75.** | sp|Q9BQ70|TCF25\_HUMAN    **Mass:** 77246    **Score:** 71     **Matches:** 7(3)  **Sequences:** 6(3)  **emPAI:** 0.18 |
|  | Transcription factor 25 OS=Homo sapiens OX=9606 GN=TCF25 PE=1 SV=1 |

|  |  |
| --- | --- |
|  | Check to include this hit in error tolerant search or archive report |
|  |  |

|  |  |  |  |  |  |  |  |  |  |  |  |
| --- | --- | --- | --- | --- | --- | --- | --- | --- | --- | --- | --- |
|  | **Query** | **Observed** | **Mr(expt)** | **Mr(calc)** | **ppm** | **Miss** | **Score** | **Expect** | **Rank** | **Unique** | **Peptide** |
|  | 86 | **379.7105** | **757.4065** | **757.4195** | **-17.16** | **1** | **1** | **13** | **1** | **U** | **R.LRGEQR.G** |
|  | 209 | **409.2216** | **816.4287** | **816.4283** | **0.53** | **0** | **20** | **0.19** | **1** | **U** | **R.SHFLWK.E** |
|  | 674 | **469.7874** | **937.5603** | **937.5596** | **0.75** | **0** | **30** | **0.004** | **1** | **U** | **R.HVILSEIK.E** |
|  | 915 | **503.7536** | **1005.4926** | **1005.4954** | **-2.70** | **0** | **4** | **6** | **1** | **U** | **K.CTWLTTPK.S** |
|  | 1931 | **408.2229** | **1221.6469** | **1221.6466** | **0.25** | **1** | **(20)** | **0.15** | **1** | **U** | **R.HLNPDTELKR.Y** |
|  | 1932 | **408.2230** | **1221.6471** | **1221.6466** | **0.48** | **1** | **27** | **0.029** | **1** | **U** | **R.HLNPDTELKR.Y** |
|  | 3868 | **622.9938** | **1865.9597** | **1865.9595** | **0.09** | **0** | **55** | **3.6e-005** | **1** | **U** | **R.IEDSTGLNRPGPAPLSSR.K** |

  


---

|  |  |
| --- | --- |
| **76.** | sp|P62241|RS8\_HUMAN    **Mass:** 24475    **Score:** 70     **Matches:** 5(2)  **Sequences:** 5(2)  **emPAI:** 0.47 |
|  | 40S ribosomal protein S8 OS=Homo sapiens OX=9606 GN=RPS8 PE=1 SV=2 |

|  |  |
| --- | --- |
|  | Check to include this hit in error tolerant search or archive report |
|  |  |

|  |  |  |  |  |  |  |  |  |  |  |  |
| --- | --- | --- | --- | --- | --- | --- | --- | --- | --- | --- | --- |
|  | **Query** | **Observed** | **Mr(expt)** | **Mr(calc)** | **ppm** | **Miss** | **Score** | **Expect** | **Rank** | **Unique** | **Peptide** |
|  | 775 | **485.2558** | **968.4970** | **968.4967** | **0.29** | **0** | **20** | **0.13** | **1** | **U** | **K.ELEFYLR.K** |
|  | 2312 | **657.8437** | **1313.6728** | **1313.6714** | **1.07** | **0** | **43** | **0.00073** | **1** | **U** | **K.LTPEEEEILNK.K** |
|  | 2450 | **449.9182** | **1346.7326** | **1346.7306** | **1.50** | **1** | **12** | **0.66** | **1** | **U** | **R.KYELGRPAANTK.I** |
|  | 3049 | **753.8918** | **1505.7691** | **1505.7726** | **-2.26** | **0** | **48** | **0.00024** | **1** | **U** | **K.ISSLLEEQFQQGK.L** |
|  | 3517 | 573.6319 | 1717.8739 | 1717.8999 | -15.13 | 0 | 3 | 7.1 | 1 | U | R.IIDVVYNASNNELVR.T |

  


---

|  |  |
| --- | --- |
| **77.** | sp|Q6PEY2|TBA3E\_HUMAN    **Mass:** 50568    **Score:** 70     **Matches:** 5(3)  **Sequences:** 4(3)  **emPAI:** 0.21 |
|  | Tubulin alpha-3E chain OS=Homo sapiens OX=9606 GN=TUBA3E PE=1 SV=2 |

|  |  |
| --- | --- |
|  | Check to include this hit in error tolerant search or archive report |
|  |  |

|  |  |  |  |  |  |  |  |  |  |  |  |
| --- | --- | --- | --- | --- | --- | --- | --- | --- | --- | --- | --- |
|  | **Query** | **Observed** | **Mr(expt)** | **Mr(calc)** | **ppm** | **Miss** | **Score** | **Expect** | **Rank** | **Unique** | **Peptide** |
|  | 946 | 508.2933 | 1014.5720 | 1014.5709 | 1.09 | 0 | 35 | 0.0061 | 1 |  | K.DVNAAIATIK.T |
|  | 3516 | 859.9440 | 1717.8734 | 1717.8747 | -0.77 | 0 | 26 | 0.04 | 1 |  | R.NLDIERPTYTNLNR.L |
|  | 3517 | 573.6319 | 1717.8739 | 1717.8747 | -0.49 | 0 | (2) | 8.8 | 3 |  | R.NLDIERPTYTNLNR.L |
|  | 4090 | 1004.4489 | 2006.8833 | 2006.8858 | -1.26 | 0 | 49 | 7.1e-005 | 1 |  | K.TIGGGDDSFNTFFSETGAGK.H |
|  | 4521 | **633.7864** | **2531.1167** | **2531.0852** | **12.4** | **0** | **1** | **3.1** | **3** | **U** | **K.WAFVHWYVGEGMEEGEFSEAR.E + Oxidation (M)** |

  


---

|  |  |
| --- | --- |
| **78.** | sp|P26373|RL13\_HUMAN    **Mass:** 24304    **Score:** 69     **Matches:** 3(3)  **Sequences:** 3(3)  **emPAI:** 0.47 |
|  | 60S ribosomal protein L13 OS=Homo sapiens OX=9606 GN=RPL13 PE=1 SV=4 |

|  |  |
| --- | --- |
|  | Check to include this hit in error tolerant search or archive report |
|  |  |

|  |  |  |  |  |  |  |  |  |  |  |  |
| --- | --- | --- | --- | --- | --- | --- | --- | --- | --- | --- | --- |
|  | **Query** | **Observed** | **Mr(expt)** | **Mr(calc)** | **ppm** | **Miss** | **Score** | **Expect** | **Rank** | **Unique** | **Peptide** |
|  | 713 | **475.7509** | **949.4873** | **949.4869** | **0.43** | **0** | **30** | **0.017** | **1** | **U** | **R.GFSLEELR.V** |
|  | 1788 | **595.3094** | **1188.6043** | **1188.6040** | **0.30** | **0** | **46** | **0.00049** | **1** | **U** | **R.VATWFNQPAR.K** |
|  | 2580 | **691.8953** | **1381.7760** | **1381.7752** | **0.58** | **0** | **33** | **0.0033** | **1** | **U** | **K.LATQLTGPVMPVR.N** |

  


---

|  |  |
| --- | --- |
| **79.** | sp|P12236|ADT3\_HUMAN    **Mass:** 33073    **Score:** 65     **Matches:** 7(3)  **Sequences:** 6(2)  **emPAI:** 0.47 |
|  | ADP/ATP translocase 3 OS=Homo sapiens OX=9606 GN=SLC25A6 PE=1 SV=4 |

|  |  |
| --- | --- |
|  | Check to include this hit in error tolerant search or archive report |
|  |  |

|  |  |  |  |  |  |  |  |  |  |  |  |
| --- | --- | --- | --- | --- | --- | --- | --- | --- | --- | --- | --- |
|  | **Query** | **Observed** | **Mr(expt)** | **Mr(calc)** | **ppm** | **Miss** | **Score** | **Expect** | **Rank** | **Unique** | **Peptide** |
|  | 401 | 431.2294 | 860.4442 | 860.4426 | 1.85 | 0 | 15 | 0.66 | 1 |  | R.GLGDCLVK.I |
|  | 513 | **445.2602** | **888.5058** | **888.5029** | **3.26** | **1** | **4** | **6.9** | **6** | **U** | **K.ITKSDGIR.G** |
|  | 555 | 451.7459 | 901.4773 | 901.4770 | 0.31 | 0 | 44 | 0.001 | 1 |  | K.GAWSNVLR.G |
|  | 1466 | 561.2916 | 1120.5687 | 1120.5665 | 1.93 | 0 | 19 | 0.26 | 1 |  | K.EQGVLSFWR.G |
|  | 1535 | 379.5640 | 1135.6701 | 1135.6713 | -1.12 | 0 | (24) | 0.012 | 1 |  | K.LLLQVQHASK.Q |
|  | 1536 | 568.8431 | 1135.6716 | 1135.6713 | 0.25 | 0 | 35 | 0.00093 | 1 |  | K.LLLQVQHASK.Q |
|  | 1871 | **603.2957** | **1204.5769** | **1204.5764** | **0.38** | **0** | **21** | **0.13** | **1** | **U** | **R.AAYFGVYDTAK.G** |

  


---

|  |  |
| --- | --- |
| **80.** | sp|Q08188|TGM3\_HUMAN    **Mass:** 76926    **Score:** 64     **Matches:** 4(2)  **Sequences:** 4(2)  **emPAI:** 0.09 |
|  | Protein-glutamine gamma-glutamyltransferase E OS=Homo sapiens OX=9606 GN=TGM3 PE=1 SV=4 |

|  |  |
| --- | --- |
|  | Check to include this hit in error tolerant search or archive report |
|  |  |

|  |  |  |  |  |  |  |  |  |  |  |  |
| --- | --- | --- | --- | --- | --- | --- | --- | --- | --- | --- | --- |
|  | **Query** | **Observed** | **Mr(expt)** | **Mr(calc)** | **ppm** | **Miss** | **Score** | **Expect** | **Rank** | **Unique** | **Peptide** |
|  | 2082 | **629.3194** | **1256.6242** | **1256.6248** | **-0.48** | **0** | **38** | **0.002** | **1** | **U** | **K.VPDESEVVVER.D** |
|  | 2993 | **497.2389** | **1488.6948** | **1488.6957** | **-0.65** | **0** | **44** | **0.00043** | **1** | **U** | **R.VITNFNSAHDTDR.N** |
|  | 3659 | **888.4607** | **1774.9068** | **1774.9115** | **-2.61** | **0** | **8** | **2** | **2** | **U** | **M.AALGVQSINWQTAFNR.Q** |
|  | 4136 | **1013.9998** | **2025.9851** | **2025.9643** | **10.2** | **0** | **3** | **5.1** | **5** | **U** | **R.EEYVQEDAGIIFVGSTNR.I** |

  


---

|  |  |
| --- | --- |
| **81.** | sp|Q92841|DDX17\_HUMAN    **Mass:** 80906    **Score:** 64     **Matches:** 5(2)  **Sequences:** 3(1)  **emPAI:** 0.08 |
|  | Probable ATP-dependent RNA helicase DDX17 OS=Homo sapiens OX=9606 GN=DDX17 PE=1 SV=2 |

|  |  |
| --- | --- |
|  | Check to include this hit in error tolerant search or archive report |
|  |  |

|  |  |  |  |  |  |  |  |  |  |  |  |
| --- | --- | --- | --- | --- | --- | --- | --- | --- | --- | --- | --- |
|  | **Query** | **Observed** | **Mr(expt)** | **Mr(calc)** | **ppm** | **Miss** | **Score** | **Expect** | **Rank** | **Unique** | **Peptide** |
|  | 1104 | **523.2858** | **1044.5571** | **1044.5465** | **10.2** | **1** | **(0)** | **18** | **5** | **U** | **K.FGNPGERLR.K** |
|  | 1113 | **523.2867** | **1044.5589** | **1044.5465** | **11.9** | **1** | **1** | **15** | **2** | **U** | **K.FGNPGERLR.K** |
|  | 2391 | 668.8235 | 1335.6325 | 1335.6315 | 0.77 | 0 | 44 | 0.0005 | 1 |  | R.MLDMGFEPQIR.K |
|  | 2457 | 676.8214 | 1351.6281 | 1351.6264 | 1.27 | 0 | (38) | 0.0017 | 1 |  | R.MLDMGFEPQIR.K + Oxidation (M) |
|  | 2907 | 728.3460 | 1454.6775 | 1454.6750 | 1.70 | 0 | 17 | 0.23 | 2 | U | R.SSQSSSQQFSGIGR.S |

  


---

|  |  |
| --- | --- |
| **82.** | sp|Q7Z3Y9|K1C26\_HUMAN    **Mass:** 52620    **Score:** 64     **Matches:** 3(2)  **Sequences:** 2(1)  **emPAI:** 0.06 |
|  | Keratin, type I cytoskeletal 26 OS=Homo sapiens OX=9606 GN=KRT26 PE=1 SV=2 |

|  |  |
| --- | --- |
|  | Check to include this hit in error tolerant search or archive report |
|  |  |

|  |  |  |  |  |  |  |  |  |  |  |  |
| --- | --- | --- | --- | --- | --- | --- | --- | --- | --- | --- | --- |
|  | **Query** | **Observed** | **Mr(expt)** | **Mr(calc)** | **ppm** | **Miss** | **Score** | **Expect** | **Rank** | **Unique** | **Peptide** |
|  | 962 | 511.2545 | 1020.4945 | 1020.4975 | -2.96 | 0 | 9 | 2.1 | 3 | U | K.DSTEETIVK.T |
|  | 1312 | 545.7676 | 1089.5207 | 1089.5237 | -2.71 | 0 | 55 | 4.7e-005 | 1 |  | K.VTMQNLNDR.L |
|  | 1313 | 545.7702 | 1089.5258 | 1089.5237 | 1.99 | 0 | (31) | 0.013 | 1 |  | K.VTMQNLNDR.L |

  


---

|  |  |
| --- | --- |
| **83.** | sp|P35030|TRY3\_HUMAN    **Mass:** 33306    **Score:** 63     **Matches:** 1(1)  **Sequences:** 1(1)  **emPAI:** 0.10 |
|  | Trypsin-3 OS=Homo sapiens OX=9606 GN=PRSS3 PE=1 SV=2 |

|  |  |
| --- | --- |
|  | Check to include this hit in error tolerant search or archive report |
|  |  |

|  |  |  |  |  |  |  |  |  |  |  |  |
| --- | --- | --- | --- | --- | --- | --- | --- | --- | --- | --- | --- |
|  | **Query** | **Observed** | **Mr(expt)** | **Mr(calc)** | **ppm** | **Miss** | **Score** | **Expect** | **Rank** | **Unique** | **Peptide** |
|  | 2813 | **716.8755** | **1431.7365** | **1431.7358** | **0.55** | **0** | **63** | **8.5e-006** | **1** | **U** | **K.VLEGNEQFINAAK.I** |

  


---

|  |  |
| --- | --- |
| **84.** | sp|P0DOX5|IGG1\_HUMAN    **Mass:** 49925    **Score:** 61     **Matches:** 8(6)  **Sequences:** 2(2)  **emPAI:** 0.21 |
|  | Immunoglobulin gamma-1 heavy chain OS=Homo sapiens OX=9606 PE=1 SV=2 |

|  |  |
| --- | --- |
|  | Check to include this hit in error tolerant search or archive report |
|  |  |

|  |  |  |  |  |  |  |  |  |  |  |  |
| --- | --- | --- | --- | --- | --- | --- | --- | --- | --- | --- | --- |
|  | **Query** | **Observed** | **Mr(expt)** | **Mr(calc)** | **ppm** | **Miss** | **Score** | **Expect** | **Rank** | **Unique** | **Peptide** |
|  | 265 | **418.2211** | **834.4276** | **834.4269** | **0.79** | **0** | **(26)** | **0.045** | **1** | **U** | **K.DTLMISR.T** |
|  | 266 | **418.2212** | **834.4278** | **834.4269** | **1.08** | **0** | **(24)** | **0.076** | **1** | **U** | **K.DTLMISR.T** |
|  | 267 | **418.2215** | **834.4285** | **834.4269** | **1.90** | **0** | **(28)** | **0.032** | **1** | **U** | **K.DTLMISR.T** |
|  | 268 | **418.2224** | **834.4302** | **834.4269** | **3.93** | **0** | **(24)** | **0.085** | **1** | **U** | **K.DTLMISR.T** |
|  | 271 | **419.7549** | **837.4953** | **837.4960** | **-0.84** | **0** | **(29)** | **0.0069** | **1** | **U** | **K.ALPAPIEK.T** |
|  | 272 | **419.7552** | **837.4958** | **837.4960** | **-0.17** | **0** | **30** | **0.0058** | **1** | **U** | **K.ALPAPIEK.T** |
|  | 372 | **426.2178** | **850.4210** | **850.4218** | **-1.01** | **0** | **31** | **0.012** | **1** | **U** | **K.DTLMISR.T + Oxidation (M)** |
|  | 373 | **426.2181** | **850.4217** | **850.4218** | **-0.21** | **0** | **(27)** | **0.029** | **1** | **U** | **K.DTLMISR.T + Oxidation (M)** |

  

|  |  |
| --- | --- |
|  | |
|  | **Proteins matching the same set of peptides:** |

|  |  |
| --- | --- |
|  | sp|P01857|IGHG1\_HUMAN    **Mass:** 36596    **Score:** 61     **Matches:** 8(6)  **Sequences:** 2(2) |
|  | Immunoglobulin heavy constant gamma 1 OS=Homo sapiens OX=9606 GN=IGHG1 PE=1 SV=1 |

|  |  |
| --- | --- |
|  | sp|P01860|IGHG3\_HUMAN    **Mass:** 42287    **Score:** 61     **Matches:** 8(6)  **Sequences:** 2(2) |
|  | Immunoglobulin heavy constant gamma 3 OS=Homo sapiens OX=9606 GN=IGHG3 PE=1 SV=2 |

---

|  |  |
| --- | --- |
| **85.** | sp|Q5VTE0|EF1A3\_HUMAN    **Mass:** 50495    **Score:** 58     **Matches:** 2(1)  **Sequences:** 2(1)  **emPAI:** 0.13 |
|  | Putative elongation factor 1-alpha-like 3 OS=Homo sapiens OX=9606 GN=EEF1A1P5 PE=5 SV=1 |

|  |  |
| --- | --- |
|  | Check to include this hit in error tolerant search or archive report |
|  |  |

|  |  |  |  |  |  |  |  |  |  |  |  |
| --- | --- | --- | --- | --- | --- | --- | --- | --- | --- | --- | --- |
|  | **Query** | **Observed** | **Mr(expt)** | **Mr(calc)** | **ppm** | **Miss** | **Score** | **Expect** | **Rank** | **Unique** | **Peptide** |
|  | 801 | **488.2804** | **974.5462** | **974.5437** | **2.64** | **0** | **19** | **0.18** | **1** | **U** | **R.LPLQDVYK.I** |
|  | 2685 | **702.8681** | **1403.7216** | **1403.7197** | **1.36** | **0** | **55** | **4.3e-005** | **1** | **U** | **K.YYVTIIDAPGHR.D** |

  

|  |  |
| --- | --- |
|  | |
|  | **Proteins matching the same set of peptides:** |

|  |  |
| --- | --- |
|  | sp|P68104|EF1A1\_HUMAN    **Mass:** 50451    **Score:** 58     **Matches:** 2(1)  **Sequences:** 2(1) |
|  | Elongation factor 1-alpha 1 OS=Homo sapiens OX=9606 GN=EEF1A1 PE=1 SV=1 |

---

|  |  |
| --- | --- |
| **86.** | sp|Q07020|RL18\_HUMAN    **Mass:** 21735    **Score:** 58     **Matches:** 2(1)  **Sequences:** 2(1)  **emPAI:** 0.15 |
|  | 60S ribosomal protein L18 OS=Homo sapiens OX=9606 GN=RPL18 PE=1 SV=2 |

|  |  |
| --- | --- |
|  | Check to include this hit in error tolerant search or archive report |
|  |  |

|  |  |  |  |  |  |  |  |  |  |  |  |
| --- | --- | --- | --- | --- | --- | --- | --- | --- | --- | --- | --- |
|  | **Query** | **Observed** | **Mr(expt)** | **Mr(calc)** | **ppm** | **Miss** | **Score** | **Expect** | **Rank** | **Unique** | **Peptide** |
|  | 2439 | **673.3691** | **1344.7236** | **1344.7249** | **-0.98** | **0** | **58** | **2.5e-005** | **1** | **U** | **K.TAVVVGTITDDVR.V** |
|  | 2920 | 730.9032 | 1459.7918 | 1459.7922 | -0.27 | 0 | 1 | 8.9 | 9 | U | K.ILTFDQLALDSPK.G |

  


---

|  |  |
| --- | --- |
| **87.** | sp|P83731|RL24\_HUMAN    **Mass:** 17882    **Score:** 56     **Matches:** 2(2)  **Sequences:** 2(2)  **emPAI:** 0.41 |
|  | 60S ribosomal protein L24 OS=Homo sapiens OX=9606 GN=RPL24 PE=1 SV=1 |

|  |  |
| --- | --- |
|  | Check to include this hit in error tolerant search or archive report |
|  |  |

|  |  |  |  |  |  |  |  |  |  |  |  |
| --- | --- | --- | --- | --- | --- | --- | --- | --- | --- | --- | --- |
|  | **Query** | **Observed** | **Mr(expt)** | **Mr(calc)** | **ppm** | **Miss** | **Score** | **Expect** | **Rank** | **Unique** | **Peptide** |
|  | 768 | **483.7744** | **965.5341** | **965.5334** | **0.73** | **0** | **39** | **0.0011** | **1** | **U** | **K.VFQFLNAK.C** |
|  | 2100 | **631.3464** | **1260.6783** | **1260.6747** | **2.84** | **0** | **39** | **0.0016** | **1** | **U** | **R.AITGASLADIMAK.R** |

  


---

|  |  |
| --- | --- |
| **88.** | sp|P62805|H4\_HUMAN    **Mass:** 11360    **Score:** 55     **Matches:** 2(2)  **Sequences:** 2(2)  **emPAI:** 0.70 |
|  | Histone H4 OS=Homo sapiens OX=9606 GN=HIST1H4A PE=1 SV=2 |

|  |  |
| --- | --- |
|  | Check to include this hit in error tolerant search or archive report |
|  |  |

|  |  |  |  |  |  |  |  |  |  |  |  |
| --- | --- | --- | --- | --- | --- | --- | --- | --- | --- | --- | --- |
|  | **Query** | **Observed** | **Mr(expt)** | **Mr(calc)** | **ppm** | **Miss** | **Score** | **Expect** | **Rank** | **Unique** | **Peptide** |
|  | 851 | **495.2927** | **988.5708** | **988.5706** | **0.27** | **0** | **34** | **0.0036** | **1** | **U** | **K.VFLENVIR.D** |
|  | 2366 | **663.3800** | **1324.7454** | **1324.7463** | **-0.63** | **0** | **41** | **0.00063** | **1** | **U** | **R.DNIQGITKPAIR.R** |

  


---

|  |  |
| --- | --- |
| **89.** | sp|P05109|S10A8\_HUMAN    **Mass:** 10885    **Score:** 54     **Matches:** 2(2)  **Sequences:** 1(1)  **emPAI:** 0.74 |
|  | Protein S100-A8 OS=Homo sapiens OX=9606 GN=S100A8 PE=1 SV=1 |

|  |  |
| --- | --- |
|  | Check to include this hit in error tolerant search or archive report |
|  |  |

|  |  |  |  |  |  |  |  |  |  |  |  |
| --- | --- | --- | --- | --- | --- | --- | --- | --- | --- | --- | --- |
|  | **Query** | **Observed** | **Mr(expt)** | **Mr(calc)** | **ppm** | **Miss** | **Score** | **Expect** | **Rank** | **Unique** | **Peptide** |
|  | 2139 | **636.8517** | **1271.6888** | **1271.6874** | **1.14** | **0** | **51** | **0.00011** | **1** | **U** | **K.ALNSIIDVYHK.Y** |
|  | 2140 | **424.9040** | **1271.6903** | **1271.6874** | **2.33** | **0** | **(27)** | **0.03** | **1** | **U** | **K.ALNSIIDVYHK.Y** |

  


---

|  |  |
| --- | --- |
| **90.** | sp|Q9Y5X1|SNX9\_HUMAN    **Mass:** 66949    **Score:** 54     **Matches:** 3(1)  **Sequences:** 3(1)  **emPAI:** 0.15 |
|  | Sorting nexin-9 OS=Homo sapiens OX=9606 GN=SNX9 PE=1 SV=1 |

|  |  |
| --- | --- |
|  | Check to include this hit in error tolerant search or archive report |
|  |  |

|  |  |  |  |  |  |  |  |  |  |  |  |
| --- | --- | --- | --- | --- | --- | --- | --- | --- | --- | --- | --- |
|  | **Query** | **Observed** | **Mr(expt)** | **Mr(calc)** | **ppm** | **Miss** | **Score** | **Expect** | **Rank** | **Unique** | **Peptide** |
|  | 565 | **453.2366** | **904.4586** | **904.4589** | **-0.27** | **0** | **25** | **0.07** | **1** | **U** | **R.LQAWMTR.M** |
|  | 2420 | **671.3733** | **1340.7321** | **1340.7340** | **-1.38** | **0** | **17** | **0.21** | **1** | **U** | **K.FGSAIPIPSLPDK.Q** |
|  | 3481 | **850.4183** | **1698.8221** | **1698.8213** | **0.48** | **0** | **47** | **0.00022** | **1** | **U** | **K.SYIEYQLTPTNTNR.S** |

  


---

|  |  |
| --- | --- |
| **91.** | sp|P31942|HNRH3\_HUMAN    **Mass:** 36960    **Score:** 54     **Matches:** 5(2)  **Sequences:** 5(2)  **emPAI:** 0.19 |
|  | Heterogeneous nuclear ribonucleoprotein H3 OS=Homo sapiens OX=9606 GN=HNRNPH3 PE=1 SV=2 |

|  |  |
| --- | --- |
|  | Check to include this hit in error tolerant search or archive report |
|  |  |

|  |  |  |  |  |  |  |  |  |  |  |  |
| --- | --- | --- | --- | --- | --- | --- | --- | --- | --- | --- | --- |
|  | **Query** | **Observed** | **Mr(expt)** | **Mr(calc)** | **ppm** | **Miss** | **Score** | **Expect** | **Rank** | **Unique** | **Peptide** |
|  | 273 | **420.7341** | **839.4536** | **839.4541** | **-0.56** | **0** | **18** | **0.22** | **1** | **U** | **R.YIEIFR.S** |
|  | 408 | 433.2155 | 864.4164 | 864.4164 | 0.03 | 0 | 13 | 0.64 | 1 |  | R.GLPFGCSK.E |
|  | 1147 | **526.7776** | **1051.5407** | **1051.5411** | **-0.30** | **0** | **28** | **0.022** | **1** | **U** | **R.VHIDIGADGR.A** |
|  | 2130 | **636.3170** | **1270.6194** | **1270.6194** | **-0.00** | **0** | **46** | **0.00028** | **1** | **U** | **R.STGEAFVQFASK.E** |
|  | 3739 | **602.2495** | **1803.7265** | **1803.7384** | **-6.57** | **0** | **1** | **1** | **1** | **U** | **R.GGGGSGGYYGQGGMSGGGWR.G** |

  


---

|  |  |
| --- | --- |
| **92.** | sp|P61313|RL15\_HUMAN    **Mass:** 24245    **Score:** 53     **Matches:** 4(2)  **Sequences:** 4(2)  **emPAI:** 0.30 |
|  | 60S ribosomal protein L15 OS=Homo sapiens OX=9606 GN=RPL15 PE=1 SV=2 |

|  |  |
| --- | --- |
|  | Check to include this hit in error tolerant search or archive report |
|  |  |

|  |  |  |  |  |  |  |  |  |  |  |  |
| --- | --- | --- | --- | --- | --- | --- | --- | --- | --- | --- | --- |
|  | **Query** | **Observed** | **Mr(expt)** | **Mr(calc)** | **ppm** | **Miss** | **Score** | **Expect** | **Rank** | **Unique** | **Peptide** |
|  | 921 | **504.2696** | **1006.5247** | **1006.5236** | **1.09** | **0** | **16** | **0.59** | **2** | **U** | **K.YIQELWR.K** |
|  | 952 | **509.7620** | **1017.5095** | **1017.5091** | **0.42** | **0** | **46** | **0.00054** | **1** | **U** | **R.SLQSVAEER.A** |
|  | 1334 | 547.2789 | 1092.5433 | 1092.5346 | 7.99 | 1 | 5 | 5.4 | 5 | U | R.EMRGLTSAGR.K + Oxidation (M) |
|  | 3490 | **569.3094** | **1704.9065** | **1704.9060** | **0.31** | **0** | **29** | **0.012** | **1** | **U** | **K.GATYGKPVHHGVNQLK.F** |

  


---

|  |  |
| --- | --- |
| **93.** | sp|P00738|HPT\_HUMAN    **Mass:** 45861    **Score:** 50     **Matches:** 1(1)  **Sequences:** 1(1)  **emPAI:** 0.07 |
|  | Haptoglobin OS=Homo sapiens OX=9606 GN=HP PE=1 SV=1 |

|  |  |
| --- | --- |
|  | Check to include this hit in error tolerant search or archive report |
|  |  |

|  |  |  |  |  |  |  |  |  |  |  |  |
| --- | --- | --- | --- | --- | --- | --- | --- | --- | --- | --- | --- |
|  | **Query** | **Observed** | **Mr(expt)** | **Mr(calc)** | **ppm** | **Miss** | **Score** | **Expect** | **Rank** | **Unique** | **Peptide** |
|  | 2203 | **645.8686** | **1289.7226** | **1289.7231** | **-0.37** | **0** | **50** | **9.3e-005** | **1** | **U** | **K.DIAPTLTLYVGK.K** |

  

|  |  |
| --- | --- |
|  | |
|  | **Proteins matching the same set of peptides:** |

|  |  |
| --- | --- |
|  | sp|P00739|HPTR\_HUMAN    **Mass:** 39518    **Score:** 50     **Matches:** 1(1)  **Sequences:** 1(1) |
|  | Haptoglobin-related protein OS=Homo sapiens OX=9606 GN=HPR PE=2 SV=2 |

---

|  |  |
| --- | --- |
| **94.** | sp|Q00325|MPCP\_HUMAN    **Mass:** 40525    **Score:** 49     **Matches:** 9(1)  **Sequences:** 3(1)  **emPAI:** 0.08 |
|  | Phosphate carrier protein, mitochondrial OS=Homo sapiens OX=9606 GN=SLC25A3 PE=1 SV=2 |

|  |  |
| --- | --- |
|  | Check to include this hit in error tolerant search or archive report |
|  |  |

|  |  |  |  |  |  |  |  |  |  |  |  |
| --- | --- | --- | --- | --- | --- | --- | --- | --- | --- | --- | --- |
|  | **Query** | **Observed** | **Mr(expt)** | **Mr(calc)** | **ppm** | **Miss** | **Score** | **Expect** | **Rank** | **Unique** | **Peptide** |
|  | 293 | **421.7581** | **841.5016** | **841.5174** | **-18.83** | **0** | **(2)** | **6.8** | **7** | **U** | **K.FVVPKPR.S** |
|  | 304 | **421.7581** | **841.5017** | **841.5174** | **-18.68** | **0** | **(1)** | **9** | **5** | **U** | **K.FVVPKPR.S** |
|  | 307 | **421.7582** | **841.5017** | **841.5174** | **-18.61** | **0** | **(3)** | **5.1** | **10** | **U** | **K.FVVPKPR.S** |
|  | 319 | **421.7582** | **841.5019** | **841.5174** | **-18.47** | **0** | **(2)** | **6.2** | **8** | **U** | **K.FVVPKPR.S** |
|  | 320 | **421.7582** | **841.5019** | **841.5174** | **-18.47** | **0** | **(1)** | **8.6** | **10** | **U** | **K.FVVPKPR.S** |
|  | 323 | **421.7582** | **841.5019** | **841.5174** | **-18.47** | **0** | **5** | **3.1** | **5** | **U** | **K.FVVPKPR.S** |
|  | 331 | **421.7583** | **841.5020** | **841.5174** | **-18.33** | **0** | **(4)** | **4.5** | **9** | **U** | **K.FVVPKPR.S** |
|  | 626 | **465.2551** | **928.4956** | **928.4953** | **0.39** | **0** | **21** | **0.18** | **2** | **U** | **K.GVAPLWMR.Q** |
|  | 2511 | **681.3629** | **1360.7113** | **1360.7099** | **1.02** | **0** | **49** | **0.00024** | **1** | **U** | **R.IQTQPGYANTLR.D** |

  


---

|  |  |
| --- | --- |
| **95.** | sp|Q5D862|FILA2\_HUMAN    **Mass:** 249296   **Score:** 49     **Matches:** 3(2)  **Sequences:** 3(2)  **emPAI:** 0.03 |
|  | Filaggrin-2 OS=Homo sapiens OX=9606 GN=FLG2 PE=1 SV=1 |

|  |  |
| --- | --- |
|  | Check to include this hit in error tolerant search or archive report |
|  |  |

|  |  |  |  |  |  |  |  |  |  |  |  |
| --- | --- | --- | --- | --- | --- | --- | --- | --- | --- | --- | --- |
|  | **Query** | **Observed** | **Mr(expt)** | **Mr(calc)** | **ppm** | **Miss** | **Score** | **Expect** | **Rank** | **Unique** | **Peptide** |
|  | 2023 | **621.8076** | **1241.6006** | **1241.6113** | **-8.60** | **1** | **14** | **0.48** | **1** | **U** | **K.DRHGSSSVELR.E** |
|  | 4446 | **784.6674** | **2350.9803** | **2350.9799** | **0.15** | **0** | **48** | **2.6e-005** | **1** | **U** | **R.QSSYGQHGSGSSQSSGYGQYGSR.E** |
|  | 4638 | **714.3058** | **2853.1943** | **2853.1935** | **0.27** | **0** | **15** | **0.038** | **1** | **U** | **R.SGSGQSSGFGQHGSGSGQSSGFGQHESGSGK.S** |

  


---

|  |  |
| --- | --- |
| **96.** | sp|P62917|RL8\_HUMAN    **Mass:** 28235    **Score:** 49     **Matches:** 4(1)  **Sequences:** 4(1)  **emPAI:** 0.12 |
|  | 60S ribosomal protein L8 OS=Homo sapiens OX=9606 GN=RPL8 PE=1 SV=2 |

|  |  |
| --- | --- |
|  | Check to include this hit in error tolerant search or archive report |
|  |  |

|  |  |  |  |  |  |  |  |  |  |  |  |
| --- | --- | --- | --- | --- | --- | --- | --- | --- | --- | --- | --- |
|  | **Query** | **Observed** | **Mr(expt)** | **Mr(calc)** | **ppm** | **Miss** | **Score** | **Expect** | **Rank** | **Unique** | **Peptide** |
|  | 180 | 404.2037 | 806.3928 | 806.3923 | 0.73 | 0 | 4 | 8.6 | 9 | U | R.AVDFAER.H |
|  | 609 | **461.7349** | **921.4553** | **921.4668** | **-12.52** | **0** | **3** | **6.8** | **4** | **U** | **K.DIIHDPGR.G** |
|  | 682 | **471.2778** | **940.5410** | **940.5454** | **-4.73** | **0** | **49** | **0.00012** | **1** | **U** | **R.AVVGVVAGGGR.I** |
|  | 2342 | **660.3829** | **1318.7512** | **1318.7357** | **11.7** | **1** | **2** | **3.7** | **4** | **U** | **K.GIVKDIIHDPGR.G** |

  


---

|  |  |
| --- | --- |
| **97.** | sp|P62753|RS6\_HUMAN    **Mass:** 28834    **Score:** 48     **Matches:** 2(1)  **Sequences:** 2(1)  **emPAI:** 0.12 |
|  | 40S ribosomal protein S6 OS=Homo sapiens OX=9606 GN=RPS6 PE=1 SV=1 |

|  |  |
| --- | --- |
|  | Check to include this hit in error tolerant search or archive report |
|  |  |

|  |  |  |  |  |  |  |  |  |  |  |  |
| --- | --- | --- | --- | --- | --- | --- | --- | --- | --- | --- | --- |
|  | **Query** | **Observed** | **Mr(expt)** | **Mr(calc)** | **ppm** | **Miss** | **Score** | **Expect** | **Rank** | **Unique** | **Peptide** |
|  | 2176 | **642.8433** | **1283.6721** | **1283.6722** | **-0.05** | **0** | **48** | **0.0002** | **1** | **U** | **K.DIPGLTDTTVPR.R** |
|  | 2388 | **668.3388** | **1334.6631** | **1334.6653** | **-1.65** | **0** | **7** | **2.8** | **1** | **U** | **K.LNISFPATGCQK.L** |

  


---

|  |  |
| --- | --- |
| **98.** | sp|O75525|KHDR3\_HUMAN    **Mass:** 38776    **Score:** 48     **Matches:** 10(3)  **Sequences:** 5(2)  **emPAI:** 0.28 |
|  | KH domain-containing, RNA-binding, signal transduction-associated protein 3 OS=Homo sapiens OX=9606 GN=KHDRBS3 PE=1 SV=1 |

|  |  |
| --- | --- |
|  | Check to include this hit in error tolerant search or archive report |
|  |  |

|  |  |  |  |  |  |  |  |  |  |  |  |
| --- | --- | --- | --- | --- | --- | --- | --- | --- | --- | --- | --- |
|  | **Query** | **Observed** | **Mr(expt)** | **Mr(calc)** | **ppm** | **Miss** | **Score** | **Expect** | **Rank** | **Unique** | **Peptide** |
|  | 11 | 356.1945 | 710.3744 | 710.3752 | -1.04 | 0 | (27) | 0.017 | 1 |  | K.FNFVGK.L |
|  | 12 | 356.1948 | 710.3750 | 710.3752 | -0.28 | 0 | 27 | 0.017 | 1 |  | K.FNFVGK.L |
|  | 491 | **441.2632** | **880.5117** | **880.5130** | **-1.47** | **0** | **(1)** | **3.8** | **3** | **U** | **R.GVPAPAITR.G** |
|  | 494 | **441.2698** | **880.5250** | **880.5130** | **13.6** | **0** | **3** | **2.4** | **4** | **U** | **R.GVPAPAITR.G** |
|  | 1337 | 547.2842 | 1092.5538 | 1092.5525 | 1.21 | 0 | 29 | 0.019 | 1 |  | K.YLPELMAEK.D |
|  | 1338 | 547.2843 | 1092.5540 | 1092.5525 | 1.43 | 0 | (16) | 0.39 | 1 |  | K.YLPELMAEK.D |
|  | 1407 | 555.2810 | 1108.5475 | 1108.5474 | 0.05 | 0 | (8) | 2.2 | 1 |  | K.YLPELMAEK.D + Oxidation (M) |
|  | 1408 | 555.2812 | 1108.5478 | 1108.5474 | 0.38 | 0 | (22) | 0.084 | 1 |  | K.YLPELMAEK.D + Oxidation (M) |
|  | 1422 | 556.2908 | 1110.5670 | 1110.5525 | 13.0 | 1 | 0 | 13 | 2 | U | K.MSILGKGSMR.D + 2 Oxidation (M) |
|  | 1551 | 380.2242 | 1137.6508 | 1137.6618 | -9.70 | 1 | 9 | 0.79 | 2 | U | R.TRGVPAPAITR.G |

  


---

|  |  |
| --- | --- |
| **99.** | sp|Q6KB66|K2C80\_HUMAN    **Mass:** 51007    **Score:** 45     **Matches:** 4(1)  **Sequences:** 3(1)  **emPAI:** 0.06 |
|  | Keratin, type II cytoskeletal 80 OS=Homo sapiens OX=9606 GN=KRT80 PE=1 SV=2 |

|  |  |
| --- | --- |
|  | Check to include this hit in error tolerant search or archive report |
|  |  |

|  |  |  |  |  |  |  |  |  |  |  |  |
| --- | --- | --- | --- | --- | --- | --- | --- | --- | --- | --- | --- |
|  | **Query** | **Observed** | **Mr(expt)** | **Mr(calc)** | **ppm** | **Miss** | **Score** | **Expect** | **Rank** | **Unique** | **Peptide** |
|  | 948 | 508.7734 | 1015.5323 | 1015.5298 | 2.43 | 1 | (9) | 2.6 | 3 | U | R.KLVEGEEGR.M |
|  | 949 | 508.7739 | 1015.5333 | 1015.5298 | 3.45 | 1 | 16 | 0.53 | 3 | U | R.KLVEGEEGR.M |
|  | 2172 | **642.3588** | **1282.7030** | **1282.7245** | **-16.75** | **0** | **1** | **8.5** | **10** | **U** | **K.LAQLEAALQQAK.Q** |
|  | 3124 | **768.8645** | **1535.7144** | **1535.7103** | **2.67** | **0** | **45** | **0.00029** | **1** | **U** | **K.TAEEQGELAFQDAK.T** |

  


---

|  |  |
| --- | --- |
| **100.** | sp|O76013|KRT36\_HUMAN    **Mass:** 53354    **Score:** 45     **Matches:** 4(2)  **Sequences:** 3(1)  **emPAI:** 0.06 |
|  | Keratin, type I cuticular Ha6 OS=Homo sapiens OX=9606 GN=KRT36 PE=2 SV=1 |

|  |  |
| --- | --- |
|  | Check to include this hit in error tolerant search or archive report |
|  |  |

|  |  |  |  |  |  |  |  |  |  |  |  |
| --- | --- | --- | --- | --- | --- | --- | --- | --- | --- | --- | --- |
|  | **Query** | **Observed** | **Mr(expt)** | **Mr(calc)** | **ppm** | **Miss** | **Score** | **Expect** | **Rank** | **Unique** | **Peptide** |
|  | 179 | 404.2029 | 806.3913 | 806.3923 | -1.23 | 0 | 41 | 0.0016 | 1 |  | K.LAADDFR.T |
|  | 180 | 404.2037 | 806.3928 | 806.3923 | 0.73 | 0 | (26) | 0.044 | 1 |  | K.LAADDFR.T |
|  | 1138 | 526.2676 | 1050.5206 | 1050.5345 | -13.27 | 0 | 2 | 8.6 | 2 | U | R.LEGEIATYR.H |
|  | 2587 | **692.3835** | **1382.7524** | **1382.7630** | **-7.65** | **1** | **9** | **1.2** | **3** | **U** | **R.QLVEADINGLRR.I** |

  


---

|  |  |
| --- | --- |
| **101.** | sp|Q92764|KRT35\_HUMAN    **Mass:** 51640    **Score:** 45     **Matches:** 4(2)  **Sequences:** 3(1)  **emPAI:** 0.06 |
|  | Keratin, type I cuticular Ha5 OS=Homo sapiens OX=9606 GN=KRT35 PE=2 SV=5 |

|  |  |
| --- | --- |
|  | Check to include this hit in error tolerant search or archive report |
|  |  |

|  |  |  |  |  |  |  |  |  |  |  |  |
| --- | --- | --- | --- | --- | --- | --- | --- | --- | --- | --- | --- |
|  | **Query** | **Observed** | **Mr(expt)** | **Mr(calc)** | **ppm** | **Miss** | **Score** | **Expect** | **Rank** | **Unique** | **Peptide** |
|  | 179 | 404.2029 | 806.3913 | 806.3923 | -1.23 | 0 | 41 | 0.0016 | 1 |  | K.LAADDFR.T |
|  | 180 | 404.2037 | 806.3928 | 806.3923 | 0.73 | 0 | (26) | 0.044 | 1 |  | K.LAADDFR.T |
|  | 898 | 500.7870 | 999.5594 | 999.5600 | -0.62 | 0 | 6 | 4.1 | 6 | U | R.LVVEIDNAK.L |
|  | 2463 | **452.2321** | **1353.6744** | **1353.6637** | **7.97** | **1** | **2** | **9.7** | **1** | **U** | **K.KNHEEEVNSLR.C** |

  


---

|  |  |
| --- | --- |
| **102.** | sp|Q14525|KT33B\_HUMAN    **Mass:** 47325    **Score:** 45     **Matches:** 3(2)  **Sequences:** 2(1)  **emPAI:** 0.07 |
|  | Keratin, type I cuticular Ha3-II OS=Homo sapiens OX=9606 GN=KRT33B PE=1 SV=3 |

|  |  |
| --- | --- |
|  | Check to include this hit in error tolerant search or archive report |
|  |  |

|  |  |  |  |  |  |  |  |  |  |  |  |
| --- | --- | --- | --- | --- | --- | --- | --- | --- | --- | --- | --- |
|  | **Query** | **Observed** | **Mr(expt)** | **Mr(calc)** | **ppm** | **Miss** | **Score** | **Expect** | **Rank** | **Unique** | **Peptide** |
|  | 179 | 404.2029 | 806.3913 | 806.3923 | -1.23 | 0 | 41 | 0.0016 | 1 |  | K.LAADDFR.T |
|  | 180 | 404.2037 | 806.3928 | 806.3923 | 0.73 | 0 | (26) | 0.044 | 1 |  | K.LAADDFR.T |
|  | 3842 | **617.9615** | **1850.8626** | **1850.8686** | **-3.24** | **0** | **1** | **7.3** | **4** | **U** | **R.EVEQWFATQTEELNK.Q** |

  


---

|  |  |
| --- | --- |
| **103.** | sp|P47914|RL29\_HUMAN    **Mass:** 17798    **Score:** 44     **Matches:** 4(1)  **Sequences:** 2(1)  **emPAI:** 0.19 |
|  | 60S ribosomal protein L29 OS=Homo sapiens OX=9606 GN=RPL29 PE=1 SV=2 |

|  |  |
| --- | --- |
|  | Check to include this hit in error tolerant search or archive report |
|  |  |

|  |  |  |  |  |  |  |  |  |  |  |  |
| --- | --- | --- | --- | --- | --- | --- | --- | --- | --- | --- | --- |
|  | **Query** | **Observed** | **Mr(expt)** | **Mr(calc)** | **ppm** | **Miss** | **Score** | **Expect** | **Rank** | **Unique** | **Peptide** |
|  | 192 | **406.7525** | **811.4905** | **811.5028** | **-15.14** | **1** | **(0)** | **4.8** | **7** | **U** | **R.NGIKKPR.S** |
|  | 193 | **406.7529** | **811.4912** | **811.5028** | **-14.30** | **1** | **(1)** | **4.9** | **6** | **U** | **R.NGIKKPR.S** |
|  | 194 | **406.7531** | **811.4916** | **811.5028** | **-13.78** | **1** | **1** | **4.7** | **7** | **U** | **R.NGIKKPR.S** |
|  | 2562 | **689.3766** | **1376.7386** | **1376.7412** | **-1.85** | **0** | **44** | **0.0005** | **1** | **U** | **K.AQAAAPASVPAQAPK.R** |

  


---

|  |  |
| --- | --- |
| **104.** | sp|Q8TF72|SHRM3\_HUMAN    **Mass:** 218321   **Score:** 43     **Matches:** 2(2)  **Sequences:** 1(1)  **emPAI:** 0.01 |
|  | Protein Shroom3 OS=Homo sapiens OX=9606 GN=SHROOM3 PE=1 SV=2 |

|  |  |
| --- | --- |
|  | Check to include this hit in error tolerant search or archive report |
|  |  |

|  |  |  |  |  |  |  |  |  |  |  |  |
| --- | --- | --- | --- | --- | --- | --- | --- | --- | --- | --- | --- |
|  | **Query** | **Observed** | **Mr(expt)** | **Mr(calc)** | **ppm** | **Miss** | **Score** | **Expect** | **Rank** | **Unique** | **Peptide** |
|  | 637 | **466.7446** | **931.4746** | **931.4723** | **2.47** | **1** | **(33)** | **0.01** | **1** | **U** | **R.SSPATADKR.Q** |
|  | 638 | **466.7448** | **931.4750** | **931.4723** | **2.92** | **1** | **36** | **0.0059** | **1** | **U** | **R.SSPATADKR.Q** |

  


---

|  |  |
| --- | --- |
| **105.** | sp|Q8NHM4|TRY6\_HUMAN    **Mass:** 27090    **Score:** 43     **Matches:** 3(1)  **Sequences:** 1(1)  **emPAI:** 0.12 |
|  | Putative trypsin-6 OS=Homo sapiens OX=9606 GN=PRSS3P2 PE=5 SV=2 |

|  |  |
| --- | --- |
|  | Check to include this hit in error tolerant search or archive report |
|  |  |

|  |  |  |  |  |  |  |  |  |  |  |  |
| --- | --- | --- | --- | --- | --- | --- | --- | --- | --- | --- | --- |
|  | **Query** | **Observed** | **Mr(expt)** | **Mr(calc)** | **ppm** | **Miss** | **Score** | **Expect** | **Rank** | **Unique** | **Peptide** |
|  | 4346 | **1113.0629** | **2224.1112** | **2224.1124** | **-0.53** | **0** | **(3)** | **5.8** | **1** | **U** | **R.LGEHNIEVLEGNEQFINAAK.I** |
|  | 4347 | **742.3777** | **2224.1112** | **2224.1124** | **-0.51** | **0** | **43** | **0.00051** | **1** | **U** | **R.LGEHNIEVLEGNEQFINAAK.I** |
|  | 4348 | **1113.0631** | **2224.1117** | **2224.1124** | **-0.31** | **0** | **(6)** | **2.6** | **1** | **U** | **R.LGEHNIEVLEGNEQFINAAK.I** |

  

|  |  |
| --- | --- |
|  | |
|  | **Proteins matching the same set of peptides:** |

|  |  |
| --- | --- |
|  | sp|P07477|TRY1\_HUMAN    **Mass:** 27111    **Score:** 43     **Matches:** 3(1)  **Sequences:** 1(1) |
|  | Trypsin-1 OS=Homo sapiens OX=9606 GN=PRSS1 PE=1 SV=1 |

|  |  |
| --- | --- |
|  | sp|P07478|TRY2\_HUMAN    **Mass:** 26927    **Score:** 43     **Matches:** 3(1)  **Sequences:** 1(1) |
|  | Trypsin-2 OS=Homo sapiens OX=9606 GN=PRSS2 PE=1 SV=1 |

---

|  |  |
| --- | --- |
| **106.** | sp|O00712|NFIB\_HUMAN    **Mass:** 47754    **Score:** 42     **Matches:** 3(1)  **Sequences:** 2(1)  **emPAI:** 0.07 |
|  | Nuclear factor 1 B-type OS=Homo sapiens OX=9606 GN=NFIB PE=1 SV=2 |

|  |  |
| --- | --- |
|  | Check to include this hit in error tolerant search or archive report |
|  |  |

|  |  |  |  |  |  |  |  |  |  |  |  |
| --- | --- | --- | --- | --- | --- | --- | --- | --- | --- | --- | --- |
|  | **Query** | **Observed** | **Mr(expt)** | **Mr(calc)** | **ppm** | **Miss** | **Score** | **Expect** | **Rank** | **Unique** | **Peptide** |
|  | 555 | 451.7459 | 901.4773 | 901.4770 | 0.31 | 1 | 16 | 0.61 | 5 | U | R.QADKVWR.L |
|  | 1710 | **587.2905** | **1172.5665** | **1172.5673** | **-0.69** | **0** | **42** | **0.00081** | **1** | **U** | **K.GIPLESTDGER.L** |
|  | 1712 | **587.2945** | **1172.5744** | **1172.5673** | **6.06** | **0** | **(2)** | **9.3** | **9** | **U** | **K.GIPLESTDGER.L** |

  

|  |  |
| --- | --- |
|  | |
|  | **Proteins matching the same set of peptides:** |

|  |  |
| --- | --- |
|  | sp|P08651|NFIC\_HUMAN    **Mass:** 56096    **Score:** 42     **Matches:** 3(1)  **Sequences:** 2(1) |
|  | Nuclear factor 1 C-type OS=Homo sapiens OX=9606 GN=NFIC PE=1 SV=2 |

|  |  |
| --- | --- |
|  | sp|Q12857|NFIA\_HUMAN    **Mass:** 56308    **Score:** 42     **Matches:** 3(1)  **Sequences:** 2(1) |
|  | Nuclear factor 1 A-type OS=Homo sapiens OX=9606 GN=NFIA PE=1 SV=2 |

|  |  |
| --- | --- |
|  | sp|Q14938|NFIX\_HUMAN    **Mass:** 55577    **Score:** 42     **Matches:** 3(1)  **Sequences:** 2(1) |
|  | Nuclear factor 1 X-type OS=Homo sapiens OX=9606 GN=NFIX PE=1 SV=2 |

---

|  |  |
| --- | --- |
| **107.** | sp|Q9NQI0|DDX4\_HUMAN    **Mass:** 80113    **Score:** 42     **Matches:** 2(1)  **Sequences:** 2(1)  **emPAI:** 0.04 |
|  | Probable ATP-dependent RNA helicase DDX4 OS=Homo sapiens OX=9606 GN=DDX4 PE=1 SV=2 |

|  |  |
| --- | --- |
|  | Check to include this hit in error tolerant search or archive report |
|  |  |

|  |  |  |  |  |  |  |  |  |  |  |  |
| --- | --- | --- | --- | --- | --- | --- | --- | --- | --- | --- | --- |
|  | **Query** | **Observed** | **Mr(expt)** | **Mr(calc)** | **ppm** | **Miss** | **Score** | **Expect** | **Rank** | **Unique** | **Peptide** |
|  | 785 | 487.2337 | 972.4528 | 972.4488 | 4.20 | 0 | 2 | 5.3 | 8 | U | K.FSFGTCVR.A |
|  | 1334 | 547.2789 | 1092.5433 | 1092.5451 | -1.66 | 0 | 42 | 0.0011 | 1 |  | K.YLVLDEADR.M |

  


---

|  |  |
| --- | --- |
| **108.** | sp|Q86U44|MTA70\_HUMAN    **Score:** 40     **Matches:** 2(2)  **Sequences:** 1(1)  **emPAI:** 0.05 |
|  | N6-adenosine-methyltransferase catalytic subunit OS=Homo sapiens OX=9606 GN=METTL3 PE=1 SV=2 |

|  |  |
| --- | --- |
|  | Check to include this hit in error tolerant search or archive report |
|  |  |

|  |  |  |  |  |  |  |  |  |  |  |  |
| --- | --- | --- | --- | --- | --- | --- | --- | --- | --- | --- | --- |
|  | **Query** | **Observed** | **Mr(expt)** | **Mr(calc)** | **ppm** | **Miss** | **Score** | **Expect** | **Rank** | **Unique** | **Peptide** |
|  | 948 | 508.7734 | 1015.5323 | 1015.5410 | -8.63 | 1 | (27) | 0.041 | 2 | U | K.QLDSLRER.L |
|  | 949 | 508.7739 | 1015.5333 | 1015.5410 | -7.60 | 1 | 39 | 0.0025 | 2 | U | K.QLDSLRER.L |

  


---

|  |  |
| --- | --- |
| **109.** | sp|Q96P63|SPB12\_HUMAN    **Mass:** 46646    **Score:** 40     **Matches:** 5(1)  **Sequences:** 5(1)  **emPAI:** 0.07 |
|  | Serpin B12 OS=Homo sapiens OX=9606 GN=SERPINB12 PE=1 SV=1 |

|  |  |
| --- | --- |
|  | Check to include this hit in error tolerant search or archive report |
|  |  |

|  |  |  |  |  |  |  |  |  |  |  |  |
| --- | --- | --- | --- | --- | --- | --- | --- | --- | --- | --- | --- |
|  | **Query** | **Observed** | **Mr(expt)** | **Mr(calc)** | **ppm** | **Miss** | **Score** | **Expect** | **Rank** | **Unique** | **Peptide** |
|  | 399 | **430.7376** | **859.4607** | **859.4585** | **2.47** | **0** | **16** | **0.72** | **1** | **U** | **K.AQILEMR.Y** |
|  | 656 | **467.7667** | **933.5188** | **933.5171** | **1.77** | **0** | **40** | **0.0012** | **1** | **U** | **R.IGFIEEVK.A** |
|  | 791 | 487.2693 | 972.5241 | 972.5240 | 0.18 | 1 | 13 | 1.1 | 4 | U | K.GLEELERK.I |
|  | 2320 | 658.3520 | 1314.6895 | 1314.6779 | 8.86 | 1 | 1 | 17 | 7 | U | K.DNLKGLEELER.K |
|  | 3602 | **876.9079** | **1751.8012** | **1751.7937** | **4.30** | **0** | **9** | **1.2** | **1** | **U** | **R.QEINFWVECQSQGK.I** |

  


---

|  |  |
| --- | --- |
| **110.** | sp|P23396|RS3\_HUMAN    **Mass:** 26842    **Score:** 39     **Matches:** 1(1)  **Sequences:** 1(1)  **emPAI:** 0.12 |
|  | 40S ribosomal protein S3 OS=Homo sapiens OX=9606 GN=RPS3 PE=1 SV=2 |

|  |  |
| --- | --- |
|  | Check to include this hit in error tolerant search or archive report |
|  |  |

|  |  |  |  |  |  |  |  |  |  |  |  |
| --- | --- | --- | --- | --- | --- | --- | --- | --- | --- | --- | --- |
|  | **Query** | **Observed** | **Mr(expt)** | **Mr(calc)** | **ppm** | **Miss** | **Score** | **Expect** | **Rank** | **Unique** | **Peptide** |
|  | 1008 | **515.3189** | **1028.6231** | **1028.6230** | **0.17** | **0** | **39** | **0.00058** | **1** | **U** | **R.TEIIILATR.T** |

  


---

|  |  |
| --- | --- |
| **111.** | sp|P07996|TSP1\_HUMAN    **Mass:** 133291   **Score:** 38     **Matches:** 2(1)  **Sequences:** 2(1)  **emPAI:** 0.05 |
|  | Thrombospondin-1 OS=Homo sapiens OX=9606 GN=THBS1 PE=1 SV=2 |

|  |  |
| --- | --- |
|  | Check to include this hit in error tolerant search or archive report |
|  |  |

|  |  |  |  |  |  |  |  |  |  |  |  |
| --- | --- | --- | --- | --- | --- | --- | --- | --- | --- | --- | --- |
|  | **Query** | **Observed** | **Mr(expt)** | **Mr(calc)** | **ppm** | **Miss** | **Score** | **Expect** | **Rank** | **Unique** | **Peptide** |
|  | 926 | **505.2695** | **1008.5244** | **1008.5240** | **0.38** | **0** | **23** | **0.064** | **1** | **U** | **R.AQGYSGLSVK.V** |
|  | 1882 | **604.3197** | **1206.6248** | **1206.6245** | **0.33** | **0** | **38** | **0.0026** | **1** | **U** | **K.SITLFVQEDR.A** |

  


---

|  |  |
| --- | --- |
| **112.** | sp|Q96NE9|FRMD6\_HUMAN    **Mass:** 72853    **Score:** 38     **Matches:** 2(1)  **Sequences:** 2(1)  **emPAI:** 0.05 |
|  | FERM domain-containing protein 6 OS=Homo sapiens OX=9606 GN=FRMD6 PE=1 SV=1 |

|  |  |
| --- | --- |
|  | Check to include this hit in error tolerant search or archive report |
|  |  |

|  |  |  |  |  |  |  |  |  |  |  |  |
| --- | --- | --- | --- | --- | --- | --- | --- | --- | --- | --- | --- |
|  | **Query** | **Observed** | **Mr(expt)** | **Mr(calc)** | **ppm** | **Miss** | **Score** | **Expect** | **Rank** | **Unique** | **Peptide** |
|  | 1156 | **528.2784** | **1054.5422** | **1054.5407** | **1.42** | **0** | **38** | **0.0021** | **1** | **U** | **R.HSLSLDDIR.L** |
|  | 2006 | 620.3242 | 1238.6338 | 1238.6554 | -17.45 | 0 | 3 | 8 | 5 | U | K.QVLHSQCVLR.E |

  


---

|  |  |
| --- | --- |
| **113.** | sp|P04003|C4BPA\_HUMAN    **Mass:** 69042    **Score:** 37     **Matches:** 2(2)  **Sequences:** 2(2)  **emPAI:** 0.10 |
|  | C4b-binding protein alpha chain OS=Homo sapiens OX=9606 GN=C4BPA PE=1 SV=2 |

|  |  |
| --- | --- |
|  | Check to include this hit in error tolerant search or archive report |
|  |  |

|  |  |  |  |  |  |  |  |  |  |  |  |
| --- | --- | --- | --- | --- | --- | --- | --- | --- | --- | --- | --- |
|  | **Query** | **Observed** | **Mr(expt)** | **Mr(calc)** | **ppm** | **Miss** | **Score** | **Expect** | **Rank** | **Unique** | **Peptide** |
|  | 2047 | **625.3420** | **1248.6694** | **1248.6714** | **-1.61** | **0** | **38** | **0.002** | **1** | **U** | **K.EDVYVVGTVLR.Y** |
|  | 3244 | **791.3577** | **1580.7008** | **1580.7042** | **-2.15** | **0** | **20** | **0.056** | **1** | **U** | **R.FSAICQGDGTWSPR.T** |

  


---

|  |  |
| --- | --- |
| **114.** | sp|Q15517|CDSN\_HUMAN    **Mass:** 52288    **Score:** 37     **Matches:** 3(1)  **Sequences:** 2(1)  **emPAI:** 0.06 |
|  | Corneodesmosin OS=Homo sapiens OX=9606 GN=CDSN PE=1 SV=3 |

|  |  |
| --- | --- |
|  | Check to include this hit in error tolerant search or archive report |
|  |  |

|  |  |  |  |  |  |  |  |  |  |  |  |
| --- | --- | --- | --- | --- | --- | --- | --- | --- | --- | --- | --- |
|  | **Query** | **Observed** | **Mr(expt)** | **Mr(calc)** | **ppm** | **Miss** | **Score** | **Expect** | **Rank** | **Unique** | **Peptide** |
|  | 1299 | **544.2946** | **1086.5747** | **1086.5750** | **-0.27** | **0** | **9** | **3.1** | **1** | **U** | **K.IYPVGYFTK.E** |
|  | 1300 | **544.2959** | **1086.5772** | **1086.5750** | **2.09** | **0** | **(2)** | **13** | **2** | **U** | **K.IYPVGYFTK.E** |
|  | 3383 | **827.9254** | **1653.8363** | **1653.8362** | **0.03** | **0** | **37** | **0.0024** | **1** | **U** | **K.GSPGVPSFAAGPPISEGK.Y** |

  


---

|  |  |
| --- | --- |
| **115.** | sp|Q08554|DSC1\_HUMAN    **Mass:** 101406   **Score:** 37     **Matches:** 5(2)  **Sequences:** 4(1)  **emPAI:** 0.07 |
|  | Desmocollin-1 OS=Homo sapiens OX=9606 GN=DSC1 PE=1 SV=2 |

|  |  |
| --- | --- |
|  | Check to include this hit in error tolerant search or archive report |
|  |  |

|  |  |  |  |  |  |  |  |  |  |  |  |
| --- | --- | --- | --- | --- | --- | --- | --- | --- | --- | --- | --- |
|  | **Query** | **Observed** | **Mr(expt)** | **Mr(calc)** | **ppm** | **Miss** | **Score** | **Expect** | **Rank** | **Unique** | **Peptide** |
|  | 1446 | **558.8309** | **1115.6473** | **1115.6372** | **9.03** | **1** | **1** | **6.8** | **6** | **U** | **R.IDVEILRMK.V** |
|  | 1785 | **594.8385** | **1187.6624** | **1187.6662** | **-3.20** | **0** | **(29)** | **0.017** | **1** | **U** | **K.ILQQIPDHPK.H** |
|  | 1786 | **396.8950** | **1187.6631** | **1187.6662** | **-2.69** | **0** | **31** | **0.0088** | **1** | **U** | **K.ILQQIPDHPK.H** |
|  | 2967 | **493.5793** | **1477.7160** | **1477.7161** | **-0.08** | **0** | **11** | **1.3** | **1** | **U** | **K.VQDQDLPNTPHSK.A** |
|  | 3605 | **585.2890** | **1752.8452** | **1752.8465** | **-0.75** | **1** | **16** | **0.27** | **1** | **U** | **R.MKVQDQDLPNTPHSK.A + Oxidation (M)** |

  


---

|  |  |
| --- | --- |
| **116.** | sp|Q9BYE4|SPR2G\_HUMAN    **Mass:** 8779     **Score:** 37     **Matches:** 1(1)  **Sequences:** 1(1)  **emPAI:** 0.40 |
|  | Small proline-rich protein 2G OS=Homo sapiens OX=9606 GN=SPRR2G PE=3 SV=1 |

|  |  |
| --- | --- |
|  | Check to include this hit in error tolerant search or archive report |
|  |  |

|  |  |  |  |  |  |  |  |  |  |  |  |
| --- | --- | --- | --- | --- | --- | --- | --- | --- | --- | --- | --- |
|  | **Query** | **Observed** | **Mr(expt)** | **Mr(calc)** | **ppm** | **Miss** | **Score** | **Expect** | **Rank** | **Unique** | **Peptide** |
|  | 3044 | **753.3661** | **1504.7176** | **1504.7167** | **0.63** | **0** | **37** | **0.003** | **1** | **U** | **K.QPCQPPPVCPTPK.C** |

  

|  |  |
| --- | --- |
|  | |
|  | **Proteins matching the same set of peptides:** |

|  |  |
| --- | --- |
|  | sp|P22531|SPR2E\_HUMAN    **Mass:** 8591     **Score:** 37     **Matches:** 1(1)  **Sequences:** 1(1) |
|  | Small proline-rich protein 2E OS=Homo sapiens OX=9606 GN=SPRR2E PE=2 SV=2 |

|  |  |
| --- | --- |
|  | sp|P22532|SPR2D\_HUMAN    **Mass:** 8584     **Score:** 37     **Matches:** 1(1)  **Sequences:** 1(1) |
|  | Small proline-rich protein 2D OS=Homo sapiens OX=9606 GN=SPRR2D PE=2 SV=2 |

|  |  |
| --- | --- |
|  | sp|P35325|SPR2B\_HUMAN    **Mass:** 8597     **Score:** 37     **Matches:** 1(1)  **Sequences:** 1(1) |
|  | Small proline-rich protein 2B OS=Homo sapiens OX=9606 GN=SPRR2B PE=2 SV=1 |

|  |  |
| --- | --- |
|  | sp|P35326|SPR2A\_HUMAN    **Mass:** 8587     **Score:** 37     **Matches:** 1(1)  **Sequences:** 1(1) |
|  | Small proline-rich protein 2A OS=Homo sapiens OX=9606 GN=SPRR2A PE=1 SV=1 |

---

|  |  |
| --- | --- |
| **117.** | sp|Q15654|TRIP6\_HUMAN    **Mass:** 51738    **Score:** 36     **Matches:** 2(1)  **Sequences:** 2(1)  **emPAI:** 0.06 |
|  | Thyroid receptor-interacting protein 6 OS=Homo sapiens OX=9606 GN=TRIP6 PE=1 SV=3 |

|  |  |
| --- | --- |
|  | Check to include this hit in error tolerant search or archive report |
|  |  |

|  |  |  |  |  |  |  |  |  |  |  |  |
| --- | --- | --- | --- | --- | --- | --- | --- | --- | --- | --- | --- |
|  | **Query** | **Observed** | **Mr(expt)** | **Mr(calc)** | **ppm** | **Miss** | **Score** | **Expect** | **Rank** | **Unique** | **Peptide** |
|  | 3616 | **586.6447** | **1756.9123** | **1756.9121** | **0.09** | **0** | **36** | **0.003** | **1** | **U** | **R.GTPGPPPAHGAALQPHPR.V** |
|  | 4278 | **542.4840** | **2165.9069** | **2165.9380** | **-14.34** | **1** | **4** | **0.57** | **1** | **U** | **R.AMGKAYHPGCFTCVVCHR.G + Oxidation (M)** |

  


---

|  |  |
| --- | --- |
| **118.** | sp|Q06830|PRDX1\_HUMAN    **Mass:** 22324    **Score:** 35     **Matches:** 1(1)  **Sequences:** 1(1)  **emPAI:** 0.15 |
|  | Peroxiredoxin-1 OS=Homo sapiens OX=9606 GN=PRDX1 PE=1 SV=1 |

|  |  |
| --- | --- |
|  | Check to include this hit in error tolerant search or archive report |
|  |  |

|  |  |  |  |  |  |  |  |  |  |  |  |
| --- | --- | --- | --- | --- | --- | --- | --- | --- | --- | --- | --- |
|  | **Query** | **Observed** | **Mr(expt)** | **Mr(calc)** | **ppm** | **Miss** | **Score** | **Expect** | **Rank** | **Unique** | **Peptide** |
|  | 1900 | **606.3409** | **1210.6673** | **1210.6670** | **0.28** | **0** | **35** | **0.0029** | **1** | **U** | **R.QITVNDLPVGR.S** |

  

|  |  |
| --- | --- |
|  | |
|  | **Proteins matching the same set of peptides:** |

|  |  |
| --- | --- |
|  | sp|P32119|PRDX2\_HUMAN    **Mass:** 22049    **Score:** 35     **Matches:** 1(1)  **Sequences:** 1(1) |
|  | Peroxiredoxin-2 OS=Homo sapiens OX=9606 GN=PRDX2 PE=1 SV=5 |

---

|  |  |
| --- | --- |
| **119.** | sp|A0A0C4DH55|KVD07\_HUMAN    **Mass:** 13254    **Score:** 35     **Matches:** 1(1)  **Sequences:** 1(1)  **emPAI:** 0.26 |
|  | Immunoglobulin kappa variable 3D-7 OS=Homo sapiens OX=9606 GN=IGKV3D-7 PE=3 SV=5 |

|  |  |
| --- | --- |
|  | Check to include this hit in error tolerant search or archive report |
|  |  |

|  |  |  |  |  |  |  |  |  |  |  |  |
| --- | --- | --- | --- | --- | --- | --- | --- | --- | --- | --- | --- |
|  | **Query** | **Observed** | **Mr(expt)** | **Mr(calc)** | **ppm** | **Miss** | **Score** | **Expect** | **Rank** | **Unique** | **Peptide** |
|  | 871 | **497.2909** | **992.5672** | **992.5655** | **1.78** | **0** | **35** | **0.0023** | **1** | **U** | **R.LLIYGASTR.A** |

  

|  |  |
| --- | --- |
|  | |
|  | **Proteins matching the same set of peptides:** |

|  |  |
| --- | --- |
|  | sp|P01624|KV315\_HUMAN    **Mass:** 12602    **Score:** 35     **Matches:** 1(1)  **Sequences:** 1(1) |
|  | Immunoglobulin kappa variable 3-15 OS=Homo sapiens OX=9606 GN=IGKV3-15 PE=1 SV=2 |

---

|  |  |
| --- | --- |
| **120.** | sp|P62979|RS27A\_HUMAN    **Mass:** 18296    **Score:** 35     **Matches:** 4(2)  **Sequences:** 3(2)  **emPAI:** 0.66 |
|  | Ubiquitin-40S ribosomal protein S27a OS=Homo sapiens OX=9606 GN=RPS27A PE=1 SV=2 |

|  |  |
| --- | --- |
|  | Check to include this hit in error tolerant search or archive report |
|  |  |

|  |  |  |  |  |  |  |  |  |  |  |  |
| --- | --- | --- | --- | --- | --- | --- | --- | --- | --- | --- | --- |
|  | **Query** | **Observed** | **Mr(expt)** | **Mr(calc)** | **ppm** | **Miss** | **Score** | **Expect** | **Rank** | **Unique** | **Peptide** |
|  | 96 | **383.2195** | **764.4245** | **764.4255** | **-1.24** | **0** | **29** | **0.017** | **1** |  | **-.MQIFVK.T** |
|  | 1266 | **541.2803** | **1080.5460** | **1080.5451** | **0.80** | **0** | **18** | **0.23** | **1** |  | **R.TLSDYNIQK.E** |
|  | 3081 | **762.3939** | **1522.7732** | **1522.7740** | **-0.52** | **1** | **(15)** | **0.44** | **1** |  | **K.IQDKEGIPPDQQR.L** |
|  | 3084 | **508.5989** | **1522.7747** | **1522.7740** | **0.50** | **1** | **28** | **0.024** | **1** |  | **K.IQDKEGIPPDQQR.L** |

  

|  |  |
| --- | --- |
|  | |
|  | **Proteins matching the same set of peptides:** |

|  |  |
| --- | --- |
|  | sp|P62987|RL40\_HUMAN    **Mass:** 15004    **Score:** 35     **Matches:** 4(2)  **Sequences:** 3(2) |
|  | Ubiquitin-60S ribosomal protein L40 OS=Homo sapiens OX=9606 GN=UBA52 PE=1 SV=2 |

---

|  |  |
| --- | --- |
| **121.** | sp|P0CG47|UBB\_HUMAN    **Mass:** 25803    **Score:** 35     **Matches:** 5(2)  **Sequences:** 4(2)  **emPAI:** 0.44 |
|  | Polyubiquitin-B OS=Homo sapiens OX=9606 GN=UBB PE=1 SV=1 |

|  |  |
| --- | --- |
|  | Check to include this hit in error tolerant search or archive report |
|  |  |

|  |  |  |  |  |  |  |  |  |  |  |  |
| --- | --- | --- | --- | --- | --- | --- | --- | --- | --- | --- | --- |
|  | **Query** | **Observed** | **Mr(expt)** | **Mr(calc)** | **ppm** | **Miss** | **Score** | **Expect** | **Rank** | **Unique** | **Peptide** |
|  | 96 | 383.2195 | 764.4245 | 764.4255 | -1.24 | 0 | 29 | 0.017 | 1 |  | -.MQIFVK.T |
|  | 1266 | 541.2803 | 1080.5460 | 1080.5451 | 0.80 | 0 | 18 | 0.23 | 1 |  | R.TLSDYNIQK.E |
|  | 2568 | **690.3931** | **1378.7717** | **1378.7643** | **5.40** | **1** | **0** | **6.9** | **6** | **U** | **R.GGMQIFVKTLTGK.T** |
|  | 3081 | 762.3939 | 1522.7732 | 1522.7740 | -0.52 | 1 | (15) | 0.44 | 1 |  | K.IQDKEGIPPDQQR.L |
|  | 3084 | 508.5989 | 1522.7747 | 1522.7740 | 0.50 | 1 | 28 | 0.024 | 1 |  | K.IQDKEGIPPDQQR.L |

  

|  |  |
| --- | --- |
|  | |
|  | **Proteins matching the same set of peptides:** |

|  |  |
| --- | --- |
|  | sp|P0CG48|UBC\_HUMAN    **Mass:** 76992    **Score:** 35     **Matches:** 5(2)  **Sequences:** 4(2) |
|  | Polyubiquitin-C OS=Homo sapiens OX=9606 GN=UBC PE=1 SV=3 |

---

|  |  |
| --- | --- |
| **122.** | sp|Q9NRL3|STRN4\_HUMAN    **Score:** 35     **Matches:** 1(1)  **Sequences:** 1(1)  **emPAI:** 0.04 |
|  | Striatin-4 OS=Homo sapiens OX=9606 GN=STRN4 PE=1 SV=2 |

|  |  |
| --- | --- |
|  | Check to include this hit in error tolerant search or archive report |
|  |  |

|  |  |  |  |  |  |  |  |  |  |  |  |
| --- | --- | --- | --- | --- | --- | --- | --- | --- | --- | --- | --- |
|  | **Query** | **Observed** | **Mr(expt)** | **Mr(calc)** | **ppm** | **Miss** | **Score** | **Expect** | **Rank** | **Unique** | **Peptide** |
|  | 890 | 499.8032 | 997.5918 | 997.5920 | -0.18 | 0 | 35 | 0.0027 | 2 | U | K.LQGILADLR.D |

  


---

|  |  |
| --- | --- |
| **123.** | sp|P0C869|PA24B\_HUMAN    **Score:** 35     **Matches:** 1(1)  **Sequences:** 1(1)  **emPAI:** 0.04 |
|  | Cytosolic phospholipase A2 beta OS=Homo sapiens OX=9606 GN=PLA2G4B PE=1 SV=2 |

|  |  |
| --- | --- |
|  | Check to include this hit in error tolerant search or archive report |
|  |  |

|  |  |  |  |  |  |  |  |  |  |  |  |
| --- | --- | --- | --- | --- | --- | --- | --- | --- | --- | --- | --- |
|  | **Query** | **Observed** | **Mr(expt)** | **Mr(calc)** | **ppm** | **Miss** | **Score** | **Expect** | **Rank** | **Unique** | **Peptide** |
|  | 779 | 486.7606 | 971.5066 | 971.5148 | -8.47 | 1 | 35 | 0.0051 | 2 | U | R.QELAERAR.L |

  


---

|  |  |
| --- | --- |
| **124.** | sp|P06702|S10A9\_HUMAN    **Mass:** 13291    **Score:** 34     **Matches:** 2(1)  **Sequences:** 2(1)  **emPAI:** 0.26 |
|  | Protein S100-A9 OS=Homo sapiens OX=9606 GN=S100A9 PE=1 SV=1 |

|  |  |
| --- | --- |
|  | Check to include this hit in error tolerant search or archive report |
|  |  |

|  |  |  |  |  |  |  |  |  |  |  |  |
| --- | --- | --- | --- | --- | --- | --- | --- | --- | --- | --- | --- |
|  | **Query** | **Observed** | **Mr(expt)** | **Mr(calc)** | **ppm** | **Miss** | **Score** | **Expect** | **Rank** | **Unique** | **Peptide** |
|  | 480 | **439.2421** | **876.4696** | **876.4705** | **-0.98** | **0** | **9** | **2.4** | **4** | **U** | **K.DLQNFLK.K** |
|  | 2909 | **728.3669** | **1454.7193** | **1454.7154** | **2.70** | **0** | **34** | **0.0059** | **1** | **U** | **K.LGHPDTLNQGEFK.E** |

  


---

|  |  |
| --- | --- |
| **125.** | sp|Q01844|EWS\_HUMAN    **Score:** 34     **Matches:** 2(2)  **Sequences:** 1(1)  **emPAI:** 0.05 |
|  | RNA-binding protein EWS OS=Homo sapiens OX=9606 GN=EWSR1 PE=1 SV=1 |

|  |  |
| --- | --- |
|  | Check to include this hit in error tolerant search or archive report |
|  |  |

|  |  |  |  |  |  |  |  |  |  |  |  |
| --- | --- | --- | --- | --- | --- | --- | --- | --- | --- | --- | --- |
|  | **Query** | **Observed** | **Mr(expt)** | **Mr(calc)** | **ppm** | **Miss** | **Score** | **Expect** | **Rank** | **Unique** | **Peptide** |
|  | 972 | 511.7507 | 1021.4868 | 1021.4869 | -0.04 | 0 | 29 | 0.016 | 2 | U | K.AAVEWFDGK.D |
|  | 973 | 511.7511 | 1021.4876 | 1021.4869 | 0.74 | 0 | (29) | 0.02 | 2 | U | K.AAVEWFDGK.D |

  


---

|  |  |
| --- | --- |
| **126.** | sp|P39023|RL3\_HUMAN    **Mass:** 46365    **Score:** 33     **Matches:** 1(1)  **Sequences:** 1(1)  **emPAI:** 0.07 |
|  | 60S ribosomal protein L3 OS=Homo sapiens OX=9606 GN=RPL3 PE=1 SV=2 |

|  |  |
| --- | --- |
|  | Check to include this hit in error tolerant search or archive report |
|  |  |

|  |  |  |  |  |  |  |  |  |  |  |  |
| --- | --- | --- | --- | --- | --- | --- | --- | --- | --- | --- | --- |
|  | **Query** | **Observed** | **Mr(expt)** | **Mr(calc)** | **ppm** | **Miss** | **Score** | **Expect** | **Rank** | **Unique** | **Peptide** |
|  | 823 | **492.2748** | **982.5350** | **982.5349** | **0.16** | **0** | **33** | **0.0062** | **1** | **U** | **R.HGSLGFLPR.K** |

  


---

|  |  |
| --- | --- |
| **127.** | sp|P14784|IL2RB\_HUMAN    **Mass:** 61820    **Score:** 32     **Matches:** 10(4)  **Sequences:** 2(1)  **emPAI:** 0.05 |
|  | Interleukin-2 receptor subunit beta OS=Homo sapiens OX=9606 GN=IL2RB PE=1 SV=1 |

|  |  |
| --- | --- |
|  | Check to include this hit in error tolerant search or archive report |
|  |  |

|  |  |  |  |  |  |  |  |  |  |  |  |
| --- | --- | --- | --- | --- | --- | --- | --- | --- | --- | --- | --- |
|  | **Query** | **Observed** | **Mr(expt)** | **Mr(calc)** | **ppm** | **Miss** | **Score** | **Expect** | **Rank** | **Unique** | **Peptide** |
|  | 782 | 487.2336 | 972.4527 | 972.4698 | -17.67 | 0 | 26 | 0.021 | 1 | U | R.MPPSLQER.V + Oxidation (M) |
|  | 783 | 487.2336 | 972.4527 | 972.4698 | -17.61 | 0 | (22) | 0.055 | 1 | U | R.MPPSLQER.V + Oxidation (M) |
|  | 784 | 487.2336 | 972.4527 | 972.4698 | -17.61 | 0 | (14) | 0.34 | 2 | U | R.MPPSLQER.V + Oxidation (M) |
|  | 785 | 487.2337 | 972.4528 | 972.4698 | -17.48 | 0 | (25) | 0.029 | 2 | U | R.MPPSLQER.V + Oxidation (M) |
|  | 786 | 487.2338 | 972.4530 | 972.4698 | -17.28 | 0 | (22) | 0.045 | 2 | U | R.MPPSLQER.V + Oxidation (M) |
|  | 787 | 487.2339 | 972.4532 | 972.4698 | -17.09 | 0 | (14) | 0.34 | 2 | U | R.MPPSLQER.V + Oxidation (M) |
|  | 788 | 487.2339 | 972.4533 | 972.4698 | -17.03 | 0 | (25) | 0.025 | 1 | U | R.MPPSLQER.V + Oxidation (M) |
|  | 789 | 487.2341 | 972.4536 | 972.4698 | -16.72 | 0 | (18) | 0.13 | 1 | U | R.MPPSLQER.V + Oxidation (M) |
|  | 790 | 487.2346 | 972.4547 | 972.4698 | -15.53 | 0 | (18) | 0.14 | 1 | U | R.MPPSLQER.V + Oxidation (M) |
|  | 4691 | **632.2871** | **3156.3989** | **3156.4254** | **-8.41** | **1** | **8** | **0.48** | **1** | **U** | **R.ANISCVWSQDGALQDTSCQVHAWPDRR.R** |

  


---

|  |  |
| --- | --- |
| **128.** | sp|Q14103|HNRPD\_HUMAN    **Mass:** 38581    **Score:** 32     **Matches:** 1(1)  **Sequences:** 1(1)  **emPAI:** 0.09 |
|  | Heterogeneous nuclear ribonucleoprotein D0 OS=Homo sapiens OX=9606 GN=HNRNPD PE=1 SV=1 |

|  |  |
| --- | --- |
|  | Check to include this hit in error tolerant search or archive report |
|  |  |

|  |  |  |  |  |  |  |  |  |  |  |  |
| --- | --- | --- | --- | --- | --- | --- | --- | --- | --- | --- | --- |
|  | **Query** | **Observed** | **Mr(expt)** | **Mr(calc)** | **ppm** | **Miss** | **Score** | **Expect** | **Rank** | **Unique** | **Peptide** |
|  | 2989 | **744.8790** | **1487.7434** | **1487.7508** | **-4.97** | **0** | **32** | **0.0099** | **1** | **U** | **K.IFVGGLSPDTPEEK.I** |

  


---

|  |  |
| --- | --- |
| **129.** | sp|P78559|MAP1A\_HUMAN    **Mass:** 306781   **Score:** 31     **Matches:** 3(1)  **Sequences:** 2(1)  **emPAI:** 0.01 |
|  | Microtubule-associated protein 1A OS=Homo sapiens OX=9606 GN=MAP1A PE=1 SV=6 |

|  |  |
| --- | --- |
|  | Check to include this hit in error tolerant search or archive report |
|  |  |

|  |  |  |  |  |  |  |  |  |  |  |  |
| --- | --- | --- | --- | --- | --- | --- | --- | --- | --- | --- | --- |
|  | **Query** | **Observed** | **Mr(expt)** | **Mr(calc)** | **ppm** | **Miss** | **Score** | **Expect** | **Rank** | **Unique** | **Peptide** |
|  | 535 | **449.7770** | **897.5393** | **897.5284** | **12.2** | **0** | **4** | **2.4** | **5** | **U** | **K.TGIVLPNGK.E** |
|  | 536 | **449.7770** | **897.5394** | **897.5284** | **12.3** | **0** | **(0)** | **5.4** | **9** | **U** | **K.TGIVLPNGK.E** |
|  | 1002 | **514.8034** | **1027.5923** | **1027.5913** | **0.90** | **0** | **31** | **0.0088** | **1** |  | **R.AVLDALLEGK.A** |

  


---

|  |  |
| --- | --- |
| **130.** | sp|P46821|MAP1B\_HUMAN    **Mass:** 271665   **Score:** 31     **Matches:** 4(1)  **Sequences:** 4(1)  **emPAI:** 0.01 |
|  | Microtubule-associated protein 1B OS=Homo sapiens OX=9606 GN=MAP1B PE=1 SV=2 |

|  |  |
| --- | --- |
|  | Check to include this hit in error tolerant search or archive report |
|  |  |

|  |  |  |  |  |  |  |  |  |  |  |  |
| --- | --- | --- | --- | --- | --- | --- | --- | --- | --- | --- | --- |
|  | **Query** | **Observed** | **Mr(expt)** | **Mr(calc)** | **ppm** | **Miss** | **Score** | **Expect** | **Rank** | **Unique** | **Peptide** |
|  | 1002 | 514.8034 | 1027.5923 | 1027.5913 | 0.90 | 0 | 31 | 0.0088 | 1 |  | R.AVLDALLEGK.A |
|  | 1455 | **560.2976** | **1118.5805** | **1118.5679** | **11.3** | **1** | **3** | **10** | **2** | **U** | **K.NAANASASKSAK.T** |
|  | 3782 | **912.4067** | **1822.7988** | **1822.7655** | **18.3** | **0** | **0** | **6.6** | **2** | **U** | **K.EMQYFMQQWTGTNK.D + 2 Oxidation (M)** |
|  | 4009 | **966.9313** | **1931.8481** | **1931.8748** | **-13.83** | **1** | **2** | **3.3** | **6** | **U** | **K.TTRTPEEGGYSYDISEK.T** |

  


---

|  |  |
| --- | --- |
| **131.** | sp|P12956|XRCC6\_HUMAN    **Mass:** 70084    **Score:** 31     **Matches:** 4(2)  **Sequences:** 4(2)  **emPAI:** 0.10 |
|  | X-ray repair cross-complementing protein 6 OS=Homo sapiens OX=9606 GN=XRCC6 PE=1 SV=2 |

|  |  |
| --- | --- |
|  | Check to include this hit in error tolerant search or archive report |
|  |  |

|  |  |  |  |  |  |  |  |  |  |  |  |
| --- | --- | --- | --- | --- | --- | --- | --- | --- | --- | --- | --- |
|  | **Query** | **Observed** | **Mr(expt)** | **Mr(calc)** | **ppm** | **Miss** | **Score** | **Expect** | **Rank** | **Unique** | **Peptide** |
|  | 913 | **503.2846** | **1004.5547** | **1004.5542** | **0.44** | **0** | **29** | **0.015** | **1** | **U** | **R.ILELDQFK.G** |
|  | 921 | 504.2696 | 1006.5247 | 1006.5335 | -8.76 | 0 | 22 | 0.12 | 1 | U | R.LGSLVDEFK.E |
|  | 1240 | **537.2860** | **1072.5575** | **1072.5587** | **-1.12** | **0** | **13** | **1** | **1** | **U** | **K.IMATPEQVGK.M** |
|  | 1709 | **586.8483** | **1171.6820** | **1171.6812** | **0.67** | **1** | **23** | **0.039** | **1** | **U** | **K.KQELLEALTK.H** |

  


---

|  |  |
| --- | --- |
| **132.** | sp|Q08380|LG3BP\_HUMAN    **Mass:** 66202    **Score:** 31     **Matches:** 3(1)  **Sequences:** 3(1)  **emPAI:** 0.10 |
|  | Galectin-3-binding protein OS=Homo sapiens OX=9606 GN=LGALS3BP PE=1 SV=1 |

|  |  |
| --- | --- |
|  | Check to include this hit in error tolerant search or archive report |
|  |  |

|  |  |  |  |  |  |  |  |  |  |  |  |
| --- | --- | --- | --- | --- | --- | --- | --- | --- | --- | --- | --- |
|  | **Query** | **Observed** | **Mr(expt)** | **Mr(calc)** | **ppm** | **Miss** | **Score** | **Expect** | **Rank** | **Unique** | **Peptide** |
|  | 1518 | **566.3407** | **1130.6668** | **1130.6659** | **0.83** | **1** | **18** | **0.081** | **1** | **U** | **R.RIDITLSSVK.C** |
|  | 1876 | **603.7804** | **1205.5462** | **1205.5465** | **-0.24** | **0** | **6** | **2** | **2** | **U** | **K.AVDTWSWGER.A** |
|  | 2367 | **663.8311** | **1325.6475** | **1325.6463** | **0.95** | **0** | **29** | **0.019** | **1** | **U** | **R.ASHEEVEGLVEK.I** |

  


---

|  |  |
| --- | --- |
| **133.** | sp|Q5T750|XP32\_HUMAN    **Mass:** 28557    **Score:** 31     **Matches:** 1(1)  **Sequences:** 1(1)  **emPAI:** 0.12 |
|  | Skin-specific protein 32 OS=Homo sapiens OX=9606 GN=XP32 PE=1 SV=1 |

|  |  |
| --- | --- |
|  | Check to include this hit in error tolerant search or archive report |
|  |  |

|  |  |  |  |  |  |  |  |  |  |  |  |
| --- | --- | --- | --- | --- | --- | --- | --- | --- | --- | --- | --- |
|  | **Query** | **Observed** | **Mr(expt)** | **Mr(calc)** | **ppm** | **Miss** | **Score** | **Expect** | **Rank** | **Unique** | **Peptide** |
|  | 478 | **438.7505** | **875.4865** | **875.4865** | **0.04** | **0** | **31** | **0.014** | **1** | **U** | **R.TFGVSPLR.R** |

  


---

|  |  |
| --- | --- |
| **134.** | sp|P07196|NFL\_HUMAN    **Mass:** 61536    **Score:** 30     **Matches:** 3(1)  **Sequences:** 3(1)  **emPAI:** 0.05 |
|  | Neurofilament light polypeptide OS=Homo sapiens OX=9606 GN=NEFL PE=1 SV=3 |

|  |  |
| --- | --- |
|  | Check to include this hit in error tolerant search or archive report |
|  |  |

|  |  |  |  |  |  |  |  |  |  |  |  |
| --- | --- | --- | --- | --- | --- | --- | --- | --- | --- | --- | --- |
|  | **Query** | **Observed** | **Mr(expt)** | **Mr(calc)** | **ppm** | **Miss** | **Score** | **Expect** | **Rank** | **Unique** | **Peptide** |
|  | 1317 | **546.2595** | **1090.5044** | **1090.5254** | **-19.32** | **1** | **7** | **2.4** | **3** | **U** | **R.AAKDEVSESR.R** |
|  | 1468 | 561.2958 | 1120.5770 | 1120.5764 | 0.53 | 0 | 30 | 0.016 | 1 |  | K.EYQDLLNVK.M |
|  | 3208 | **782.8524** | **1563.6902** | **1563.7199** | **-18.99** | **0** | **1** | **4.5** | **3** | **U** | **K.QNADISAMQDTINK.L + Oxidation (M)** |

  


---

|  |  |
| --- | --- |
| **135.** | sp|P07197|NFM\_HUMAN    **Mass:** 102468   **Score:** 30     **Matches:** 3(1)  **Sequences:** 2(1)  **emPAI:** 0.03 |
|  | Neurofilament medium polypeptide OS=Homo sapiens OX=9606 GN=NEFM PE=1 SV=3 |

|  |  |
| --- | --- |
|  | Check to include this hit in error tolerant search or archive report |
|  |  |

|  |  |  |  |  |  |  |  |  |  |  |  |
| --- | --- | --- | --- | --- | --- | --- | --- | --- | --- | --- | --- |
|  | **Query** | **Observed** | **Mr(expt)** | **Mr(calc)** | **ppm** | **Miss** | **Score** | **Expect** | **Rank** | **Unique** | **Peptide** |
|  | 236 | 414.2182 | 826.4219 | 826.4225 | -0.70 | 0 | 2 | 7.1 | 10 | U | R.FAGYIEK.V |
|  | 237 | 414.2184 | 826.4223 | 826.4225 | -0.26 | 0 | (1) | 9.2 | 10 | U | R.FAGYIEK.V |
|  | 1468 | 561.2958 | 1120.5770 | 1120.5764 | 0.53 | 0 | 30 | 0.016 | 1 |  | R.EYQDLLNVK.M |

  


---

|  |  |
| --- | --- |
| **136.** | sp|Q16352|AINX\_HUMAN    **Mass:** 55528    **Score:** 30     **Matches:** 2(1)  **Sequences:** 2(1)  **emPAI:** 0.06 |
|  | Alpha-internexin OS=Homo sapiens OX=9606 GN=INA PE=1 SV=2 |

|  |  |
| --- | --- |
|  | Check to include this hit in error tolerant search or archive report |
|  |  |

|  |  |  |  |  |  |  |  |  |  |  |  |
| --- | --- | --- | --- | --- | --- | --- | --- | --- | --- | --- | --- |
|  | **Query** | **Observed** | **Mr(expt)** | **Mr(calc)** | **ppm** | **Miss** | **Score** | **Expect** | **Rank** | **Unique** | **Peptide** |
|  | 1468 | 561.2958 | 1120.5770 | 1120.5764 | 0.53 | 0 | 30 | 0.016 | 1 |  | R.EYQDLLNVK.M |
|  | 1483 | **562.7855** | **1123.5564** | **1123.5734** | **-15.17** | **0** | **1** | **11** | **2** | **U** | **K.VHQLETQNR.A** |

  


---

|  |  |
| --- | --- |
| **137.** | sp|Q13162|PRDX4\_HUMAN    **Mass:** 30749    **Score:** 30     **Matches:** 1(1)  **Sequences:** 1(1)  **emPAI:** 0.11 |
|  | Peroxiredoxin-4 OS=Homo sapiens OX=9606 GN=PRDX4 PE=1 SV=1 |

|  |  |
| --- | --- |
|  | Check to include this hit in error tolerant search or archive report |
|  |  |

|  |  |  |  |  |  |  |  |  |  |  |  |
| --- | --- | --- | --- | --- | --- | --- | --- | --- | --- | --- | --- |
|  | **Query** | **Observed** | **Mr(expt)** | **Mr(calc)** | **ppm** | **Miss** | **Score** | **Expect** | **Rank** | **Unique** | **Peptide** |
|  | 1907 | **606.8161** | **1211.6176** | **1211.6186** | **-0.82** | **0** | **30** | **0.011** | **1** | **U** | **R.LVQAFQYTDK.H** |

  


---

|  |  |
| --- | --- |
| **138.** | sp|Q00577|PURA\_HUMAN    **Mass:** 35003    **Score:** 29     **Matches:** 1(1)  **Sequences:** 1(1)  **emPAI:** 0.09 |
|  | Transcriptional activator protein Pur-alpha OS=Homo sapiens OX=9606 GN=PURA PE=1 SV=2 |

|  |  |
| --- | --- |
|  | Check to include this hit in error tolerant search or archive report |
|  |  |

|  |  |  |  |  |  |  |  |  |  |  |  |
| --- | --- | --- | --- | --- | --- | --- | --- | --- | --- | --- | --- |
|  | **Query** | **Observed** | **Mr(expt)** | **Mr(calc)** | **ppm** | **Miss** | **Score** | **Expect** | **Rank** | **Unique** | **Peptide** |
|  | 1175 | **530.7586** | **1059.5027** | **1059.5026** | **0.10** | **0** | **29** | **0.016** | **1** | **U** | **R.FFFDVGSNK.Y** |

  


---

|  |  |
| --- | --- |
| **139.** | sp|Q6ZR08|DYH12\_HUMAN    **Mass:** 359792   **Score:** 29     **Matches:** 5(1)  **Sequences:** 4(1)  **emPAI:** 0.01 |
|  | Dynein heavy chain 12, axonemal OS=Homo sapiens OX=9606 GN=DNAH12 PE=2 SV=2 |

|  |  |
| --- | --- |
|  | Check to include this hit in error tolerant search or archive report |
|  |  |

|  |  |  |  |  |  |  |  |  |  |  |  |
| --- | --- | --- | --- | --- | --- | --- | --- | --- | --- | --- | --- |
|  | **Query** | **Observed** | **Mr(expt)** | **Mr(calc)** | **ppm** | **Miss** | **Score** | **Expect** | **Rank** | **Unique** | **Peptide** |
|  | 48 | **366.7200** | **731.4255** | **731.4290** | **-4.73** | **1** | **5** | **5.2** | **6** | **U** | **K.LSKQTR.T** |
|  | 1417 | **555.7401** | **1109.4655** | **1109.4488** | **15.1** | **0** | **29** | **0.0047** | **1** | **U** | **K.WECPFDEK.G** |
|  | 3413 | **833.9497** | **1665.8847** | **1665.9137** | **-17.39** | **1** | **6** | **2.1** | **1** | **U** | **K.HTMIRLFVHEVLR.V + Oxidation (M)** |
|  | 4434 | **586.0512** | **2340.1755** | **2340.2114** | **-15.33** | **1** | **2** | **6.7** | **1** | **U** | **K.YAYEYLGNSPRLVITPLTDR.C** |
|  | 4438 | **586.0522** | **2340.1799** | **2340.2114** | **-13.45** | **1** | **(0)** | **9.4** | **4** | **U** | **K.YAYEYLGNSPRLVITPLTDR.C** |

  


---

|  |  |
| --- | --- |
| **140.** | sp|O00338|ST1C2\_HUMAN    **Score:** 29     **Matches:** 3(2)  **Sequences:** 1(1)  **emPAI:** 0.10 |
|  | Sulfotransferase 1C2 OS=Homo sapiens OX=9606 GN=SULT1C2 PE=1 SV=1 |

|  |  |
| --- | --- |
|  | Check to include this hit in error tolerant search or archive report |
|  |  |

|  |  |  |  |  |  |  |  |  |  |  |  |
| --- | --- | --- | --- | --- | --- | --- | --- | --- | --- | --- | --- |
|  | **Query** | **Observed** | **Mr(expt)** | **Mr(calc)** | **ppm** | **Miss** | **Score** | **Expect** | **Rank** | **Unique** | **Peptide** |
|  | 2583 | 692.3456 | 1382.6767 | 1382.6864 | -6.98 | 0 | (25) | 0.049 | 2 | U | K.SILDQSISSFMR.K |
|  | 2584 | 692.3477 | 1382.6809 | 1382.6864 | -3.98 | 0 | (3) | 7.5 | 3 | U | K.SILDQSISSFMR.K |
|  | 2586 | 692.3483 | 1382.6821 | 1382.6864 | -3.09 | 0 | 28 | 0.026 | 2 | U | K.SILDQSISSFMR.K |

  


---

|  |  |
| --- | --- |
| **141.** | sp|Q9P0W8|SPAT7\_HUMAN    **Mass:** 68190    **Score:** 28     **Matches:** 3(1)  **Sequences:** 3(1)  **emPAI:** 0.05 |
|  | Spermatogenesis-associated protein 7 OS=Homo sapiens OX=9606 GN=SPATA7 PE=1 SV=3 |

|  |  |
| --- | --- |
|  | Check to include this hit in error tolerant search or archive report |
|  |  |

|  |  |  |  |  |  |  |  |  |  |  |  |
| --- | --- | --- | --- | --- | --- | --- | --- | --- | --- | --- | --- |
|  | **Query** | **Observed** | **Mr(expt)** | **Mr(calc)** | **ppm** | **Miss** | **Score** | **Expect** | **Rank** | **Unique** | **Peptide** |
|  | 61 | **372.2241** | **742.4336** | **742.4337** | **-0.14** | **0** | **28** | **0.029** | **1** | **U** | **R.ATSVLPR.Y** |
|  | 1655 | **581.8577** | **1161.7008** | **1161.6805** | **17.5** | **1** | **1** | **1.9** | **3** | **U** | **K.MRHLLHVLK.V + Oxidation (M)** |
|  | 4251 | **1065.4811** | **2128.9476** | **2128.9847** | **-17.44** | **1** | **0** | **4.6** | **6** | **U** | **K.EEMNGFSSFARSLVPSSER.L** |

  


---

|  |  |
| --- | --- |
| **142.** | sp|P20930|FILA\_HUMAN    **Mass:** 435036   **Score:** 28     **Matches:** 3(1)  **Sequences:** 3(1)  **emPAI:** 0.01 |
|  | Filaggrin OS=Homo sapiens OX=9606 GN=FLG PE=1 SV=3 |

|  |  |
| --- | --- |
|  | Check to include this hit in error tolerant search or archive report |
|  |  |

|  |  |  |  |  |  |  |  |  |  |  |  |
| --- | --- | --- | --- | --- | --- | --- | --- | --- | --- | --- | --- |
|  | **Query** | **Observed** | **Mr(expt)** | **Mr(calc)** | **ppm** | **Miss** | **Score** | **Expect** | **Rank** | **Unique** | **Peptide** |
|  | 1848 | **601.2964** | **1200.5782** | **1200.5775** | **0.63** | **0** | **28** | **0.023** | **1** | **U** | **K.LAQAYYESTR.K** |
|  | 2229 | **648.8137** | **1295.6129** | **1295.5967** | **12.5** | **0** | **2** | **7.4** | **8** | **U** | **R.HSQSGQGQSAGPR.T** |
|  | 2421 | 671.7857 | 1341.5569 | 1341.5797 | -17.00 | 1 | 0 | 2.3 | 4 | U | K.TYDKEQSGDGSR.H |

  


---

|  |  |
| --- | --- |
| **143.** | sp|Q9NW38|FANCL\_HUMAN    **Mass:** 43561    **Score:** 27     **Matches:** 1(1)  **Sequences:** 1(1)  **emPAI:** 0.08 |
|  | E3 ubiquitin-protein ligase FANCL OS=Homo sapiens OX=9606 GN=FANCL PE=1 SV=2 |

|  |  |
| --- | --- |
|  | Check to include this hit in error tolerant search or archive report |
|  |  |

|  |  |  |  |  |  |  |  |  |  |  |  |
| --- | --- | --- | --- | --- | --- | --- | --- | --- | --- | --- | --- |
|  | **Query** | **Observed** | **Mr(expt)** | **Mr(calc)** | **ppm** | **Miss** | **Score** | **Expect** | **Rank** | **Unique** | **Peptide** |
|  | 734 | **480.2701** | **958.5255** | **958.5447** | **-20.01** | **0** | **27** | **0.04** | **1** | **U** | **M.AVTEASLLR.Q** |

  


---

|  |  |
| --- | --- |
| **144.** | sp|P68871|HBB\_HUMAN    **Mass:** 16102    **Score:** 26     **Matches:** 1(1)  **Sequences:** 1(1)  **emPAI:** 0.21 |
|  | Hemoglobin subunit beta OS=Homo sapiens OX=9606 GN=HBB PE=1 SV=2 |

|  |  |
| --- | --- |
|  | Check to include this hit in error tolerant search or archive report |
|  |  |

|  |  |  |  |  |  |  |  |  |  |  |  |
| --- | --- | --- | --- | --- | --- | --- | --- | --- | --- | --- | --- |
|  | **Query** | **Observed** | **Mr(expt)** | **Mr(calc)** | **ppm** | **Miss** | **Score** | **Expect** | **Rank** | **Unique** | **Peptide** |
|  | 2310 | **657.8377** | **1313.6609** | **1313.6575** | **2.53** | **0** | **26** | **0.03** | **1** | **U** | **K.VNVDEVGGEALGR.L** |

  


---

|  |  |
| --- | --- |
| **145.** | sp|Q6UWP8|SBSN\_HUMAN    **Mass:** 60562    **Score:** 26     **Matches:** 2(1)  **Sequences:** 1(1)  **emPAI:** 0.05 |
|  | Suprabasin OS=Homo sapiens OX=9606 GN=SBSN PE=1 SV=2 |

|  |  |
| --- | --- |
|  | Check to include this hit in error tolerant search or archive report |
|  |  |

|  |  |  |  |  |  |  |  |  |  |  |  |
| --- | --- | --- | --- | --- | --- | --- | --- | --- | --- | --- | --- |
|  | **Query** | **Observed** | **Mr(expt)** | **Mr(calc)** | **ppm** | **Miss** | **Score** | **Expect** | **Rank** | **Unique** | **Peptide** |
|  | 3628 | **441.7137** | **1762.8256** | **1762.8248** | **0.47** | **0** | **(15)** | **0.41** | **1** | **U** | **R.FGQGVHHAAGQAGNEAGR.F** |
|  | 3629 | **588.6159** | **1762.8259** | **1762.8248** | **0.63** | **0** | **26** | **0.032** | **1** | **U** | **R.FGQGVHHAAGQAGNEAGR.F** |

  


---

|  |  |
| --- | --- |
| **146.** | sp|P50402|EMD\_HUMAN    **Mass:** 29033    **Score:** 26     **Matches:** 2(1)  **Sequences:** 2(1)  **emPAI:** 0.11 |
|  | Emerin OS=Homo sapiens OX=9606 GN=EMD PE=1 SV=1 |

|  |  |
| --- | --- |
|  | Check to include this hit in error tolerant search or archive report |
|  |  |

|  |  |  |  |  |  |  |  |  |  |  |  |
| --- | --- | --- | --- | --- | --- | --- | --- | --- | --- | --- | --- |
|  | **Query** | **Observed** | **Mr(expt)** | **Mr(calc)** | **ppm** | **Miss** | **Score** | **Expect** | **Rank** | **Unique** | **Peptide** |
|  | 1814 | **597.8205** | **1193.6264** | **1193.6292** | **-2.28** | **1** | **26** | **0.039** | **1** | **U** | **K.KEDALLYQSK.G** |
|  | 2048 | **625.7865** | **1249.5584** | **1249.5575** | **0.77** | **0** | **3** | **5.1** | **1** | **U** | **R.TYGEPESAGPSR.A** |

  


---

|  |  |
| --- | --- |
| **147.** | sp|P31151|S10A7\_HUMAN    **Mass:** 11578    **Score:** 25     **Matches:** 1(1)  **Sequences:** 1(1)  **emPAI:** 0.30 |
|  | Protein S100-A7 OS=Homo sapiens OX=9606 GN=S100A7 PE=1 SV=4 |

|  |  |
| --- | --- |
|  | Check to include this hit in error tolerant search or archive report |
|  |  |

|  |  |  |  |  |  |  |  |  |  |  |  |
| --- | --- | --- | --- | --- | --- | --- | --- | --- | --- | --- | --- |
|  | **Query** | **Observed** | **Mr(expt)** | **Mr(calc)** | **ppm** | **Miss** | **Score** | **Expect** | **Rank** | **Unique** | **Peptide** |
|  | 2079 | **628.8119** | **1255.6092** | **1255.6085** | **0.61** | **0** | **25** | **0.041** | **1** | **U** | **K.GTNYLADVFEK.K** |

  


---

|  |  |
| --- | --- |
| **148.** | sp|P13010|XRCC5\_HUMAN    **Mass:** 83222    **Score:** 25     **Matches:** 3(1)  **Sequences:** 2(1)  **emPAI:** 0.04 |
|  | X-ray repair cross-complementing protein 5 OS=Homo sapiens OX=9606 GN=XRCC5 PE=1 SV=3 |

|  |  |
| --- | --- |
|  | Check to include this hit in error tolerant search or archive report |
|  |  |

|  |  |  |  |  |  |  |  |  |  |  |  |
| --- | --- | --- | --- | --- | --- | --- | --- | --- | --- | --- | --- |
|  | **Query** | **Observed** | **Mr(expt)** | **Mr(calc)** | **ppm** | **Miss** | **Score** | **Expect** | **Rank** | **Unique** | **Peptide** |
|  | 1017 | 516.3028 | 1030.5910 | 1030.6063 | -14.76 | 0 | (1) | 8.3 | 2 | U | K.TLFPLIEAK.K |
|  | 1018 | **516.3104** | **1030.6063** | **1030.6063** | **0.03** | **0** | **25** | **0.017** | **1** | **U** | **K.TLFPLIEAK.K** |
|  | 2570 | **690.8471** | **1379.6797** | **1379.6820** | **-1.72** | **0** | **12** | **1** | **1** | **U** | **K.TDTLEDLFPTTK.I** |

  


---

|  |  |
| --- | --- |
| **149.** | sp|Q96Q27|ASB2\_HUMAN    **Score:** 25     **Matches:** 4(1)  **Sequences:** 3(1)  **emPAI:** 0.05 |
|  | Ankyrin repeat and SOCS box protein 2 OS=Homo sapiens OX=9606 GN=ASB2 PE=1 SV=1 |

|  |  |
| --- | --- |
|  | Check to include this hit in error tolerant search or archive report |
|  |  |

|  |  |  |  |  |  |  |  |  |  |  |  |
| --- | --- | --- | --- | --- | --- | --- | --- | --- | --- | --- | --- |
|  | **Query** | **Observed** | **Mr(expt)** | **Mr(calc)** | **ppm** | **Miss** | **Score** | **Expect** | **Rank** | **Unique** | **Peptide** |
|  | 609 | 461.7349 | 921.4553 | 921.4668 | -12.49 | 1 | 8 | 2.2 | 3 | U | R.ARLYEDR.R |
|  | 2521 | 683.3199 | 1364.6252 | 1364.6493 | -17.66 | 1 | (12) | 0.73 | 2 | U | K.DGDEEALKTMIK.E + Oxidation (M) |
|  | 2522 | 683.3226 | 1364.6306 | 1364.6493 | -13.73 | 1 | 25 | 0.036 | 2 | U | K.DGDEEALKTMIK.E + Oxidation (M) |
|  | 2934 | **733.8925** | **1465.7705** | **1465.7525** | **12.3** | **0** | **2** | **8** | **4** | **U** | **R.NHDEVLEALLSAR.F** |

  


---

|  |  |
| --- | --- |
| **150.** | sp|P52272|HNRPM\_HUMAN    **Mass:** 77749    **Score:** 25     **Matches:** 6(1)  **Sequences:** 5(1)  **emPAI:** 0.04 |
|  | Heterogeneous nuclear ribonucleoprotein M OS=Homo sapiens OX=9606 GN=HNRNPM PE=1 SV=3 |

|  |  |
| --- | --- |
|  | Check to include this hit in error tolerant search or archive report |
|  |  |

|  |  |  |  |  |  |  |  |  |  |  |  |
| --- | --- | --- | --- | --- | --- | --- | --- | --- | --- | --- | --- |
|  | **Query** | **Observed** | **Mr(expt)** | **Mr(calc)** | **ppm** | **Miss** | **Score** | **Expect** | **Rank** | **Unique** | **Peptide** |
|  | 778 | **486.6989** | **971.3833** | **971.3953** | **-12.35** | **0** | **2** | **0.71** | **2** | **U** | **R.FGSGMNMGR.I + Oxidation (M)** |
|  | 1211 | 533.7534 | 1065.4923 | 1065.5131 | -19.52 | 0 | (9) | 1.9 | 3 | U | R.FEPYANPTK.R |
|  | 1212 | 533.7534 | 1065.4923 | 1065.5131 | -19.52 | 0 | 10 | 1.3 | 2 | U | R.FEPYANPTK.R |
|  | 1929 | 611.8249 | 1221.6353 | 1221.6142 | 17.3 | 1 | 2 | 9.5 | 8 | U | R.FEPYANPTKR.Y |
|  | 2108 | **632.8497** | **1263.6848** | **1263.6863** | **-1.22** | **0** | **2** | **6.6** | **2** | **U** | **R.AFITNIPFDVK.W** |
|  | 2782 | **714.3613** | **1426.7080** | **1426.7061** | **1.34** | **0** | **25** | **0.051** | **1** | **U** | **R.MGPAMGPALGAGIER.M** |

  


---

|  |  |
| --- | --- |
| **151.** | sp|Q12899|TRI26\_HUMAN    **Mass:** 62925    **Score:** 25     **Matches:** 2(1)  **Sequences:** 2(1)  **emPAI:** 0.05 |
|  | Tripartite motif-containing protein 26 OS=Homo sapiens OX=9606 GN=TRIM26 PE=1 SV=1 |

|  |  |
| --- | --- |
|  | Check to include this hit in error tolerant search or archive report |
|  |  |

|  |  |  |  |  |  |  |  |  |  |  |  |
| --- | --- | --- | --- | --- | --- | --- | --- | --- | --- | --- | --- |
|  | **Query** | **Observed** | **Mr(expt)** | **Mr(calc)** | **ppm** | **Miss** | **Score** | **Expect** | **Rank** | **Unique** | **Peptide** |
|  | 898 | 500.7870 | 999.5594 | 999.5600 | -0.60 | 0 | 10 | 1.6 | 3 | U | K.GEADILAALK.K |
|  | 1704 | **586.3497** | **1170.6849** | **1170.6860** | **-0.90** | **0** | **25** | **0.025** | **1** | **U** | **R.LALVISELEGK.A** |

  


---

|  |  |
| --- | --- |
| **152.** | sp|Q7Z6J0|SH3R1\_HUMAN    **Score:** 25     **Matches:** 1(1)  **Sequences:** 1(1)  **emPAI:** 0.03 |
|  | E3 ubiquitin-protein ligase SH3RF1 OS=Homo sapiens OX=9606 GN=SH3RF1 PE=1 SV=2 |

|  |  |
| --- | --- |
|  | Check to include this hit in error tolerant search or archive report |
|  |  |

|  |  |  |  |  |  |  |  |  |  |  |  |
| --- | --- | --- | --- | --- | --- | --- | --- | --- | --- | --- | --- |
|  | **Query** | **Observed** | **Mr(expt)** | **Mr(calc)** | **ppm** | **Miss** | **Score** | **Expect** | **Rank** | **Unique** | **Peptide** |
|  | 2249 | 650.8233 | 1299.6320 | 1299.6167 | 11.8 | 0 | 25 | 0.049 | 2 | U | K.DLQSSQGGQQPR.V |

  


---

|  |  |
| --- | --- |
| **153.** | sp|Q9NYY1|IL20\_HUMAN    **Mass:** 20402    **Score:** 24     **Matches:** 1(1)  **Sequences:** 1(1)  **emPAI:** 0.16 |
|  | Interleukin-20 OS=Homo sapiens OX=9606 GN=IL20 PE=1 SV=2 |

|  |  |
| --- | --- |
|  | Check to include this hit in error tolerant search or archive report |
|  |  |

|  |  |  |  |  |  |  |  |  |  |  |  |
| --- | --- | --- | --- | --- | --- | --- | --- | --- | --- | --- | --- |
|  | **Query** | **Observed** | **Mr(expt)** | **Mr(calc)** | **ppm** | **Miss** | **Score** | **Expect** | **Rank** | **Unique** | **Peptide** |
|  | 720 | **477.7929** | **953.5712** | **953.5546** | **17.5** | **0** | **24** | **0.019** | **1** | **U** | **K.LEPQAAVVK.A** |

  


---

|  |  |
| --- | --- |
| **154.** | sp|Q6PJT7|ZC3HE\_HUMAN    **Mass:** 83793    **Score:** 23     **Matches:** 5(1)  **Sequences:** 4(1)  **emPAI:** 0.04 |
|  | Zinc finger CCCH domain-containing protein 14 OS=Homo sapiens OX=9606 GN=ZC3H14 PE=1 SV=1 |

|  |  |
| --- | --- |
|  | Check to include this hit in error tolerant search or archive report |
|  |  |

|  |  |  |  |  |  |  |  |  |  |  |  |
| --- | --- | --- | --- | --- | --- | --- | --- | --- | --- | --- | --- |
|  | **Query** | **Observed** | **Mr(expt)** | **Mr(calc)** | **ppm** | **Miss** | **Score** | **Expect** | **Rank** | **Unique** | **Peptide** |
|  | 491 | 441.2632 | 880.5117 | 880.5130 | -1.47 | 0 | (1) | 4 | 4 | U | K.QTLPVAPR.T |
|  | 493 | 441.2642 | 880.5138 | 880.5130 | 0.82 | 0 | 23 | 0.024 | 1 | U | K.QTLPVAPR.T |
|  | 3322 | **812.8755** | **1623.7364** | **1623.7376** | **-0.75** | **0** | **18** | **0.15** | **1** | **U** | **K.SSDTNIFDSNVPSNK.S** |
|  | 3678 | **892.8850** | **1783.7553** | **1783.7406** | **8.25** | **0** | **1** | **2** | **1** | **U** | **K.NGDECAYHHPISPCK.A** |
|  | 4761 | **867.1251** | **3464.4714** | **3464.4880** | **-4.81** | **0** | **1** | **1.3** | **1** | **U** | **R.LQIDPVMAETLQMSQDYYDMESMVHADTR.S + 3 Oxidation (M)** |

  


---

|  |  |
| --- | --- |
| **155.** | sp|P0DOX8|IGL1\_HUMAN    **Mass:** 23101    **Score:** 23     **Matches:** 1(1)  **Sequences:** 1(1)  **emPAI:** 0.15 |
|  | Immunoglobulin lambda-1 light chain OS=Homo sapiens OX=9606 PE=1 SV=1 |

|  |  |
| --- | --- |
|  | Check to include this hit in error tolerant search or archive report |
|  |  |

|  |  |  |  |  |  |  |  |  |  |  |  |
| --- | --- | --- | --- | --- | --- | --- | --- | --- | --- | --- | --- |
|  | **Query** | **Observed** | **Mr(expt)** | **Mr(calc)** | **ppm** | **Miss** | **Score** | **Expect** | **Rank** | **Unique** | **Peptide** |
|  | 275 | **421.2601** | **840.5056** | **840.5069** | **-1.50** | **0** | **23** | **0.036** | **1** | **U** | **K.VTVLGQPK.A** |

  

|  |  |
| --- | --- |
|  | |
|  | **Proteins matching the same set of peptides:** |

|  |  |
| --- | --- |
|  | sp|B9A064|IGLL5\_HUMAN    **Mass:** 23391    **Score:** 23     **Matches:** 1(1)  **Sequences:** 1(1) |
|  | Immunoglobulin lambda-like polypeptide 5 OS=Homo sapiens OX=9606 GN=IGLL5 PE=2 SV=2 |

---

|  |  |
| --- | --- |
| **156.** | sp|Q96IF1|AJUBA\_HUMAN    **Mass:** 58722    **Score:** 23     **Matches:** 3(1)  **Sequences:** 1(1)  **emPAI:** 0.06 |
|  | LIM domain-containing protein ajuba OS=Homo sapiens OX=9606 GN=AJUBA PE=1 SV=1 |

|  |  |
| --- | --- |
|  | Check to include this hit in error tolerant search or archive report |
|  |  |

|  |  |  |  |  |  |  |  |  |  |  |  |
| --- | --- | --- | --- | --- | --- | --- | --- | --- | --- | --- | --- |
|  | **Query** | **Observed** | **Mr(expt)** | **Mr(calc)** | **ppm** | **Miss** | **Score** | **Expect** | **Rank** | **Unique** | **Peptide** |
|  | 3608 | **585.9326** | **1754.7759** | **1754.8071** | **-17.81** | **0** | **(2)** | **3.7** | **1** | **U** | **R.SSFASSSASDASKPSSPR.G** |
|  | 3609 | **585.9327** | **1754.7762** | **1754.8071** | **-17.60** | **0** | **(5)** | **1.9** | **1** | **U** | **R.SSFASSSASDASKPSSPR.G** |
|  | 3611 | **585.9437** | **1754.8092** | **1754.8071** | **1.17** | **0** | **23** | **0.046** | **1** | **U** | **R.SSFASSSASDASKPSSPR.G** |

  


---

|  |  |
| --- | --- |
| **157.** | sp|Q03001|DYST\_HUMAN    **Mass:** 865259   **Score:** 23     **Matches:** 15(0)  **Sequences:** 13(0)  **emPAI:** 0.00 |
|  | Dystonin OS=Homo sapiens OX=9606 GN=DST PE=1 SV=4 |

|  |  |
| --- | --- |
|  | Check to include this hit in error tolerant search or archive report |
|  |  |

|  |  |  |  |  |  |  |  |  |  |  |  |
| --- | --- | --- | --- | --- | --- | --- | --- | --- | --- | --- | --- |
|  | **Query** | **Observed** | **Mr(expt)** | **Mr(calc)** | **ppm** | **Miss** | **Score** | **Expect** | **Rank** | **Unique** | **Peptide** |
|  | 876 | 498.2633 | 994.5121 | 994.4971 | 15.0 | 0 | 8 | 2.7 | 4 | U | K.LLADGYSEK.I |
|  | 1165 | **529.3038** | **1056.5931** | **1056.6040** | **-10.27** | **1** | **0** | **12** | **6** | **U** | **K.QTRLEAALR.Q** |
|  | 1267 | **361.1905** | **1080.5498** | **1080.5638** | **-12.90** | **0** | **1** | **12** | **10** | **U** | **K.ITGVFELMR.E + Oxidation (M)** |
|  | 1446 | 558.8309 | 1115.6473 | 1115.6550 | -6.88 | 1 | 0 | 7.8 | 8 | U | K.IAELNTKLSK.L |
|  | 2043 | **417.2111** | **1248.6115** | **1248.5887** | **18.3** | **0** | **9** | **2** | **6** | **U** | **K.FQPSTVHDYR.Q** |
|  | 2084 | 629.3286 | 1256.6427 | 1256.6612 | -14.78 | 0 | (2) | 9.2 | 4 | U | K.TQALTEVDVPGK.D |
|  | 2085 | **629.3329** | **1256.6512** | **1256.6612** | **-7.97** | **0** | **9** | **2.1** | **1** | **U** | **K.TQALTEVDVPGK.D** |
|  | 2338 | 660.3385 | 1318.6624 | 1318.6365 | 19.7 | 0 | 2 | 12 | 4 | U | K.QLQESQTSGDVK.V |
|  | 2484 | 453.2494 | 1356.7264 | 1356.7150 | 8.44 | 1 | 4 | 7.2 | 7 | U | K.TQLAQHKEFQK.S |
|  | 2831 | **718.8854** | **1435.7563** | **1435.7493** | **4.89** | **1** | **23** | **0.061** | **1** | **U** | **K.TSIPIKAICDYR.Q** |
|  | 2918 | 730.3853 | 1458.7559 | 1458.7566 | -0.44 | 0 | (6) | 4.1 | 2 | U | K.QTTGDEVLIIQDK.L |
|  | 2919 | 487.2594 | 1458.7564 | 1458.7566 | -0.15 | 0 | 9 | 2 | 2 | U | K.QTTGDEVLIIQDK.L |
|  | 3398 | **554.6013** | **1660.7820** | **1660.7991** | **-10.34** | **1** | **2** | **6.9** | **5** | **U** | **R.AAHEGLIDRETMFR.L + Oxidation (M)** |
|  | 3893 | **469.0009** | **1871.9746** | **1871.9451** | **15.7** | **0** | **1** | **8.6** | **5** | **U** | **K.NIQNFPSDLIENPIMK.S** |
|  | 4476 | **807.6812** | **2420.0218** | **2420.0301** | **-3.41** | **0** | **4** | **1.1** | **1** | **U** | **R.EMFSQFAEFDDELDSMAPVGR.D** |

  


---

|  |  |
| --- | --- |
| **158.** | sp|O75628|REM1\_HUMAN    **Score:** 23     **Matches:** 1(1)  **Sequences:** 1(1)  **emPAI:** 0.10 |
|  | GTP-binding protein REM 1 OS=Homo sapiens OX=9606 GN=REM1 PE=1 SV=2 |

|  |  |
| --- | --- |
|  | Check to include this hit in error tolerant search or archive report |
|  |  |

|  |  |  |  |  |  |  |  |  |  |  |  |
| --- | --- | --- | --- | --- | --- | --- | --- | --- | --- | --- | --- |
|  | **Query** | **Observed** | **Mr(expt)** | **Mr(calc)** | **ppm** | **Miss** | **Score** | **Expect** | **Rank** | **Unique** | **Peptide** |
|  | 1151 | 351.8852 | 1052.6337 | 1052.6230 | 10.1 | 0 | 23 | 0.013 | 2 | U | R.VVLLGDPGVGK.T |

  


---

|  |  |
| --- | --- |
| **159.** | sp|Q9UJ98|STAG3\_HUMAN    **Mass:** 140257   **Score:** 22     **Matches:** 5(0)  **Sequences:** 3(0)  **emPAI:** 0.02 |
|  | Cohesin subunit SA-3 OS=Homo sapiens OX=9606 GN=STAG3 PE=1 SV=2 |

|  |  |
| --- | --- |
|  | Check to include this hit in error tolerant search or archive report |
|  |  |

|  |  |  |  |  |  |  |  |  |  |  |  |
| --- | --- | --- | --- | --- | --- | --- | --- | --- | --- | --- | --- |
|  | **Query** | **Observed** | **Mr(expt)** | **Mr(calc)** | **ppm** | **Miss** | **Score** | **Expect** | **Rank** | **Unique** | **Peptide** |
|  | 816 | **490.7322** | **979.4498** | **979.4328** | **17.4** | **1** | **22** | **0.058** | **1** | **U** | **R.MMGGREQR.Q + Oxidation (M)** |
|  | 1039 | 519.2667 | 1036.5188 | 1036.5263 | -7.29 | 0 | (2) | 11 | 3 | U | K.GIVTPEMFK.K + Oxidation (M) |
|  | 1040 | 519.2669 | 1036.5193 | 1036.5263 | -6.80 | 0 | (0) | 15 | 8 | U | K.GIVTPEMFK.K + Oxidation (M) |
|  | 1041 | 519.2672 | 1036.5197 | 1036.5263 | -6.34 | 0 | 3 | 8 | 3 | U | K.GIVTPEMFK.K + Oxidation (M) |
|  | 3522 | **860.4572** | **1718.8999** | **1718.8951** | **2.75** | **1** | **5** | **4.3** | **2** | **U** | **R.ADFARSQLVDLLTDR.F** |

  


---

|  |  |
| --- | --- |
| **160.** | sp|Q96JQ0|PCD16\_HUMAN    **Mass:** 346712   **Score:** 22     **Matches:** 7(0)  **Sequences:** 4(0)  **emPAI:** 0.01 |
|  | Protocadherin-16 OS=Homo sapiens OX=9606 GN=DCHS1 PE=1 SV=1 |

|  |  |
| --- | --- |
|  | Check to include this hit in error tolerant search or archive report |
|  |  |

|  |  |  |  |  |  |  |  |  |  |  |  |
| --- | --- | --- | --- | --- | --- | --- | --- | --- | --- | --- | --- |
|  | **Query** | **Observed** | **Mr(expt)** | **Mr(calc)** | **ppm** | **Miss** | **Score** | **Expect** | **Rank** | **Unique** | **Peptide** |
|  | 1015 | 516.3027 | 1030.5908 | 1030.5771 | 13.3 | 0 | 22 | 0.063 | 1 | U | R.VASVASSLAAR.G |
|  | 1016 | **516.3027** | **1030.5909** | **1030.5771** | **13.4** | **0** | **(3)** | **5.9** | **1** | **U** | **R.VASVASSLAAR.G** |
|  | 1017 | 516.3028 | 1030.5910 | 1030.5771 | 13.5 | 0 | (1) | 8.5 | 4 | U | R.VASVASSLAAR.G |
|  | 3312 | **811.4030** | **1620.7915** | **1620.8219** | **-18.79** | **1** | **3** | **7** | **2** | **U** | **R.EAFALNSSTGELRAR.V** |
|  | 3342 | **546.2526** | **1635.7359** | **1635.7563** | **-12.49** | **0** | **(0)** | **7.6** | **2** | **U** | **R.VSLTGPLDFEQCDR.Y** |
|  | 3344 | **546.2534** | **1635.7383** | **1635.7563** | **-11.03** | **0** | **2** | **4.7** | **4** | **U** | **R.VSLTGPLDFEQCDR.Y** |
|  | 4017 | **646.6465** | **1936.9176** | **1936.9391** | **-11.11** | **0** | **0** | **9.9** | **5** | **U** | **R.VQVQVQDENEHAPAFAR.D** |

  


---

|  |  |
| --- | --- |
| **161.** | sp|P82279|CRUM1\_HUMAN    **Mass:** 161094   **Score:** 22     **Matches:** 3(1)  **Sequences:** 2(1)  **emPAI:** 0.02 |
|  | Protein crumbs homolog 1 OS=Homo sapiens OX=9606 GN=CRB1 PE=1 SV=2 |

|  |  |
| --- | --- |
|  | Check to include this hit in error tolerant search or archive report |
|  |  |

|  |  |  |  |  |  |  |  |  |  |  |  |
| --- | --- | --- | --- | --- | --- | --- | --- | --- | --- | --- | --- |
|  | **Query** | **Observed** | **Mr(expt)** | **Mr(calc)** | **ppm** | **Miss** | **Score** | **Expect** | **Rank** | **Unique** | **Peptide** |
|  | 1 | **350.6723** | **699.3300** | **699.3340** | **-5.69** | **0** | **22** | **0.032** | **1** | **U** | **R.TFYGGR.D** |
|  | 4275 | **721.3182** | **2160.9327** | **2160.9754** | **-19.77** | **1** | **12** | **0.24** | **1** | **U** | **K.EGSRVEMWNLMPPPAMER.L + 2 Oxidation (M)** |
|  | 4276 | **541.2408** | **2160.9343** | **2160.9754** | **-19.05** | **1** | **(1)** | **2.6** | **1** | **U** | **K.EGSRVEMWNLMPPPAMER.L + 2 Oxidation (M)** |

  


---

|  |  |
| --- | --- |
| **162.** | sp|P04792|HSPB1\_HUMAN    **Mass:** 22826    **Score:** 22     **Matches:** 1(0)  **Sequences:** 1(0)  **emPAI:** 0.15 |
|  | Heat shock protein beta-1 OS=Homo sapiens OX=9606 GN=HSPB1 PE=1 SV=2 |

|  |  |
| --- | --- |
|  | Check to include this hit in error tolerant search or archive report |
|  |  |

|  |  |  |  |  |  |  |  |  |  |  |  |
| --- | --- | --- | --- | --- | --- | --- | --- | --- | --- | --- | --- |
|  | **Query** | **Observed** | **Mr(expt)** | **Mr(calc)** | **ppm** | **Miss** | **Score** | **Expect** | **Rank** | **Unique** | **Peptide** |
|  | 1658 | **582.3146** | **1162.6146** | **1162.6135** | **0.96** | **0** | **22** | **0.096** | **1** | **U** | **R.LFDQAFGLPR.L** |

  


---

|  |  |
| --- | --- |
| **163.** | sp|Q9HCF6|TRPM3\_HUMAN    **Score:** 22     **Matches:** 5(1)  **Sequences:** 4(1)  **emPAI:** 0.02 |
|  | Transient receptor potential cation channel subfamily M member 3 OS=Homo sapiens OX=9606 GN=TRPM3 PE=2 SV=4 |

|  |  |
| --- | --- |
|  | Check to include this hit in error tolerant search or archive report |
|  |  |

|  |  |  |  |  |  |  |  |  |  |  |  |
| --- | --- | --- | --- | --- | --- | --- | --- | --- | --- | --- | --- |
|  | **Query** | **Observed** | **Mr(expt)** | **Mr(calc)** | **ppm** | **Miss** | **Score** | **Expect** | **Rank** | **Unique** | **Peptide** |
|  | 1515 | 566.2587 | 1130.5028 | 1130.5244 | -19.12 | 0 | (18) | 0.09 | 2 | U | R.TSAFQSFESK.H |
|  | 1516 | 566.2589 | 1130.5031 | 1130.5244 | -18.80 | 0 | 22 | 0.04 | 2 | U | R.TSAFQSFESK.H |
|  | 2902 | 485.5682 | 1453.6826 | 1453.6653 | 11.9 | 1 | 4 | 4.2 | 6 |  | R.VTSERVENMSMR.L + Oxidation (M) |
|  | 3194 | **778.3286** | **1554.6427** | **1554.6541** | **-7.38** | **0** | **0** | **4.2** | **3** | **U** | **K.EEEDMELTAMLGR.N + 2 Oxidation (M)** |
|  | 4131 | 405.4160 | 2022.0439 | 2022.0357 | 4.04 | 0 | 8 | 1.5 | 2 | U | K.AAMTTGAWIFTGGVNTGVIR.H |

  


---

|  |  |
| --- | --- |
| **164.** | sp|Q9Y6V0|PCLO\_HUMAN    **Mass:** 562179   **Score:** 22     **Matches:** 6(1)  **Sequences:** 6(1)  **emPAI:** 0.01 |
|  | Protein piccolo OS=Homo sapiens OX=9606 GN=PCLO PE=1 SV=5 |

|  |  |
| --- | --- |
|  | Check to include this hit in error tolerant search or archive report |
|  |  |

|  |  |  |  |  |  |  |  |  |  |  |  |
| --- | --- | --- | --- | --- | --- | --- | --- | --- | --- | --- | --- |
|  | **Query** | **Observed** | **Mr(expt)** | **Mr(calc)** | **ppm** | **Miss** | **Score** | **Expect** | **Rank** | **Unique** | **Peptide** |
|  | 488 | **440.7376** | **879.4607** | **879.4450** | **17.8** | **0** | **3** | **7.4** | **8** | **U** | **K.YNSLDLR.I** |
|  | 874 | **498.2602** | **994.5059** | **994.4906** | **15.5** | **0** | **7** | **3** | **5** | **U** | **R.YLEMGINR.R** |
|  | 944 | **507.8132** | **1013.6118** | **1013.6121** | **-0.28** | **1** | **22** | **0.045** | **1** | **U** | **R.VDAKVEIIK.H** |
|  | 982 | **513.2668** | **1024.5190** | **1024.5375** | **-18.09** | **1** | **0** | **14** | **3** | **U** | **R.KMPPAPSGPK.A + Oxidation (M)** |
|  | 1545 | **569.2578** | **1136.5011** | **1136.4946** | **5.73** | **0** | **0** | **7.1** | **5** | **U** | **K.SSTSIDEDAGR.R** |
|  | 3055 | **755.4346** | **1508.8546** | **1508.8423** | **8.14** | **1** | **0** | **5.1** | **1** | **U** | **K.LPLQRDASRPQTK.Q** |

  


---

|  |  |
| --- | --- |
| **165.** | sp|Q86UW8|HPLN4\_HUMAN    **Mass:** 43402    **Score:** 21     **Matches:** 1(0)  **Sequences:** 1(0)  **emPAI:** 0.08 |
|  | Hyaluronan and proteoglycan link protein 4 OS=Homo sapiens OX=9606 GN=HAPLN4 PE=2 SV=1 |

|  |  |
| --- | --- |
|  | Check to include this hit in error tolerant search or archive report |
|  |  |

|  |  |  |  |  |  |  |  |  |  |  |  |
| --- | --- | --- | --- | --- | --- | --- | --- | --- | --- | --- | --- |
|  | **Query** | **Observed** | **Mr(expt)** | **Mr(calc)** | **ppm** | **Miss** | **Score** | **Expect** | **Rank** | **Unique** | **Peptide** |
|  | 3757 | **604.6594** | **1810.9564** | **1810.9479** | **4.73** | **1** | **21** | **0.07** | **1** | **U** | **K.LDLEGVVFPYHPRGGR.Y** |

  


---

|  |  |
| --- | --- |
| **166.** | sp|Q96BA8|CR3L1\_HUMAN    **Mass:** 57255    **Score:** 21     **Matches:** 3(0)  **Sequences:** 1(0)  **emPAI:** 0.06 |
|  | Cyclic AMP-responsive element-binding protein 3-like protein 1 OS=Homo sapiens OX=9606 GN=CREB3L1 PE=1 SV=1 |

|  |  |
| --- | --- |
|  | Check to include this hit in error tolerant search or archive report |
|  |  |

|  |  |  |  |  |  |  |  |  |  |  |  |
| --- | --- | --- | --- | --- | --- | --- | --- | --- | --- | --- | --- |
|  | **Query** | **Observed** | **Mr(expt)** | **Mr(calc)** | **ppm** | **Miss** | **Score** | **Expect** | **Rank** | **Unique** | **Peptide** |
|  | 3985 | 641.9742 | 1922.9007 | 1922.8680 | 17.0 | 0 | 21 | 0.081 | 1 | U | K.EDPLAADGVYTASQMPSR.S + Oxidation (M) |
|  | 3986 | 641.9746 | 1922.9020 | 1922.8680 | 17.7 | 0 | (5) | 3.1 | 1 | U | K.EDPLAADGVYTASQMPSR.S + Oxidation (M) |
|  | 3987 | **481.7329** | **1922.9027** | **1922.8680** | **18.0** | **0** | **(0)** | **8.7** | **2** | **U** | **K.EDPLAADGVYTASQMPSR.S + Oxidation (M)** |

  


---

|  |  |
| --- | --- |
| **167.** | sp|Q9UNX3|RL26L\_HUMAN    **Mass:** 17246    **Score:** 21     **Matches:** 1(0)  **Sequences:** 1(0)  **emPAI:** 0.20 |
|  | 60S ribosomal protein L26-like 1 OS=Homo sapiens OX=9606 GN=RPL26L1 PE=1 SV=1 |

|  |  |
| --- | --- |
|  | Check to include this hit in error tolerant search or archive report |
|  |  |

|  |  |  |  |  |  |  |  |  |  |  |  |
| --- | --- | --- | --- | --- | --- | --- | --- | --- | --- | --- | --- |
|  | **Query** | **Observed** | **Mr(expt)** | **Mr(calc)** | **ppm** | **Miss** | **Score** | **Expect** | **Rank** | **Unique** | **Peptide** |
|  | 1273 | **541.7649** | **1081.5152** | **1081.5193** | **-3.74** | **0** | **21** | **0.12** | **1** | **U** | **K.FNPFVTSDR.S** |

  

|  |  |
| --- | --- |
|  | |
|  | **Proteins matching the same set of peptides:** |

|  |  |
| --- | --- |
|  | sp|P61254|RL26\_HUMAN    **Mass:** 17248    **Score:** 21     **Matches:** 1(0)  **Sequences:** 1(0) |
|  | 60S ribosomal protein L26 OS=Homo sapiens OX=9606 GN=RPL26 PE=1 SV=1 |

---

|  |  |
| --- | --- |
| **168.** | sp|Q8WV41|SNX33\_HUMAN    **Mass:** 65565    **Score:** 20     **Matches:** 1(0)  **Sequences:** 1(0)  **emPAI:** 0.05 |
|  | Sorting nexin-33 OS=Homo sapiens OX=9606 GN=SNX33 PE=1 SV=1 |

|  |  |
| --- | --- |
|  | Check to include this hit in error tolerant search or archive report |
|  |  |

|  |  |  |  |  |  |  |  |  |  |  |  |
| --- | --- | --- | --- | --- | --- | --- | --- | --- | --- | --- | --- |
|  | **Query** | **Observed** | **Mr(expt)** | **Mr(calc)** | **ppm** | **Miss** | **Score** | **Expect** | **Rank** | **Unique** | **Peptide** |
|  | 2298 | **438.2391** | **1311.6956** | **1311.6935** | **1.57** | **0** | **20** | **0.13** | **1** | **U** | **K.LTPTHAASPVYR.R** |

  


---

|  |  |
| --- | --- |
| **169.** | sp|O60573|IF4E2\_HUMAN    **Mass:** 28458    **Score:** 20     **Matches:** 2(0)  **Sequences:** 2(0)  **emPAI:** 0.12 |
|  | Eukaryotic translation initiation factor 4E type 2 OS=Homo sapiens OX=9606 GN=EIF4E2 PE=1 SV=1 |

|  |  |
| --- | --- |
|  | Check to include this hit in error tolerant search or archive report |
|  |  |

|  |  |  |  |  |  |  |  |  |  |  |  |
| --- | --- | --- | --- | --- | --- | --- | --- | --- | --- | --- | --- |
|  | **Query** | **Observed** | **Mr(expt)** | **Mr(calc)** | **ppm** | **Miss** | **Score** | **Expect** | **Rank** | **Unique** | **Peptide** |
|  | 3692 | **598.2993** | **1791.8761** | **1791.8751** | **0.56** | **0** | **20** | **0.12** | **1** | **U** | **R.TPGRPTSSQSYEQNIK.Q** |
|  | 4036 | **488.0019** | **1947.9785** | **1947.9762** | **1.14** | **1** | **15** | **0.35** | **1** | **U** | **R.RTPGRPTSSQSYEQNIK.Q** |

  


---

|  |  |
| --- | --- |
| **170.** | sp|O43663|PRC1\_HUMAN    **Mass:** 72246    **Score:** 19     **Matches:** 1(0)  **Sequences:** 1(0)  **emPAI:** 0.05 |
|  | Protein regulator of cytokinesis 1 OS=Homo sapiens OX=9606 GN=PRC1 PE=1 SV=2 |

|  |  |
| --- | --- |
|  | Check to include this hit in error tolerant search or archive report |
|  |  |

|  |  |  |  |  |  |  |  |  |  |  |  |
| --- | --- | --- | --- | --- | --- | --- | --- | --- | --- | --- | --- |
|  | **Query** | **Observed** | **Mr(expt)** | **Mr(calc)** | **ppm** | **Miss** | **Score** | **Expect** | **Rank** | **Unique** | **Peptide** |
|  | 717 | 477.2582 | 952.5018 | 952.5018 | 0.01 | 0 | 19 | 0.15 | 1 | U | R.LFLEFER.K |

  


---

|  |  |
| --- | --- |
| **171.** | sp|Q96PH1|NOX5\_HUMAN    **Mass:** 87353    **Score:** 19     **Matches:** 3(0)  **Sequences:** 3(0)  **emPAI:** 0.04 |
|  | NADPH oxidase 5 OS=Homo sapiens OX=9606 GN=NOX5 PE=1 SV=1 |

|  |  |
| --- | --- |
|  | Check to include this hit in error tolerant search or archive report |
|  |  |

|  |  |  |  |  |  |  |  |  |  |  |  |
| --- | --- | --- | --- | --- | --- | --- | --- | --- | --- | --- | --- |
|  | **Query** | **Observed** | **Mr(expt)** | **Mr(calc)** | **ppm** | **Miss** | **Score** | **Expect** | **Rank** | **Unique** | **Peptide** |
|  | 916 | **503.7638** | **1005.5131** | **1005.5165** | **-3.31** | **0** | **19** | **0.18** | **1** | **U** | **R.DLGASVMVAK.G + Oxidation (M)** |
|  | 1797 | **595.8425** | **1189.6705** | **1189.6554** | **12.7** | **1** | **4** | **4** | **3** | **U** | **R.SSKGSEILLEK.H** |
|  | 4546 | **1305.5530** | **2609.0914** | **2609.0718** | **7.51** | **1** | **1** | **1.3** | **1** | **U** | **M.NTSGDPAQTGPEGCRGTMSAEEDAR.W + Oxidation (M)** |

  


---

|  |  |
| --- | --- |
| **172.** | sp|A8MX80|YM017\_HUMAN    **Mass:** 37896    **Score:** 18     **Matches:** 1(0)  **Sequences:** 1(0)  **emPAI:** 0.09 |
|  | Putative UPF0607 protein ENSP00000383144 OS=Homo sapiens OX=9606 PE=3 SV=2 |

|  |  |
| --- | --- |
|  | Check to include this hit in error tolerant search or archive report |
|  |  |

|  |  |  |  |  |  |  |  |  |  |  |  |
| --- | --- | --- | --- | --- | --- | --- | --- | --- | --- | --- | --- |
|  | **Query** | **Observed** | **Mr(expt)** | **Mr(calc)** | **ppm** | **Miss** | **Score** | **Expect** | **Rank** | **Unique** | **Peptide** |
|  | 3464 | **848.3898** | **1694.7650** | **1694.7729** | **-4.68** | **1** | **18** | **0.12** | **1** | **U** | **R.EGHLQQGNMHKNMR.V + Oxidation (M)** |

  


---

|  |  |
| --- | --- |
| **173.** | sp|Q5SNV9|CA167\_HUMAN    **Mass:** 164602   **Score:** 18     **Matches:** 2(0)  **Sequences:** 2(0)  **emPAI:** 0.02 |
|  | Uncharacterized protein C1orf167 OS=Homo sapiens OX=9606 GN=C1orf167 PE=2 SV=2 |

|  |  |
| --- | --- |
|  | Check to include this hit in error tolerant search or archive report |
|  |  |

|  |  |  |  |  |  |  |  |  |  |  |  |
| --- | --- | --- | --- | --- | --- | --- | --- | --- | --- | --- | --- |
|  | **Query** | **Observed** | **Mr(expt)** | **Mr(calc)** | **ppm** | **Miss** | **Score** | **Expect** | **Rank** | **Unique** | **Peptide** |
|  | 199 | **407.7559** | **813.4973** | **813.4861** | **13.8** | **0** | **2** | **11** | **4** | **U** | **R.AVLGLWR.Q** |
|  | 4081 | **998.0327** | **1994.0509** | **1994.0585** | **-3.84** | **0** | **18** | **0.14** | **1** | **U** | **R.APTLPDTLQGSLLWAAGQR.Q** |

  


---

|  |  |
| --- | --- |
| **174.** | sp|Q86UK0|ABCAC\_HUMAN    **Mass:** 295387   **Score:** 18     **Matches:** 4(0)  **Sequences:** 3(0)  **emPAI:** 0.01 |
|  | ATP-binding cassette sub-family A member 12 OS=Homo sapiens OX=9606 GN=ABCA12 PE=1 SV=3 |

|  |  |
| --- | --- |
|  | Check to include this hit in error tolerant search or archive report |
|  |  |

|  |  |  |  |  |  |  |  |  |  |  |  |
| --- | --- | --- | --- | --- | --- | --- | --- | --- | --- | --- | --- |
|  | **Query** | **Observed** | **Mr(expt)** | **Mr(calc)** | **ppm** | **Miss** | **Score** | **Expect** | **Rank** | **Unique** | **Peptide** |
|  | 671 | 469.7516 | 937.4887 | 937.4790 | 10.4 | 0 | 6 | 2.6 | 3 | U | K.VTMETLTK.F + Oxidation (M) |
|  | 672 | 469.7521 | 937.4896 | 937.4790 | 11.3 | 0 | (6) | 2.8 | 2 | U | K.VTMETLTK.F + Oxidation (M) |
|  | 2153 | **639.3575** | **1276.7005** | **1276.6809** | **15.4** | **1** | **18** | **0.2** | **1** | **U** | **K.AIMEKSNVTLR.Q + Oxidation (M)** |
|  | 4293 | **725.9902** | **2174.9489** | **2174.9799** | **-14.27** | **0** | **0** | **3.8** | **3** | **U** | **K.NMGVCMQHDVLFSYLTTK.E + 2 Oxidation (M)** |

  


---

|  |  |
| --- | --- |
| **175.** | sp|Q8N257|H2B3B\_HUMAN    **Mass:** 13900    **Score:** 18     **Matches:** 4(1)  **Sequences:** 4(1)  **emPAI:** 0.25 |
|  | Histone H2B type 3-B OS=Homo sapiens OX=9606 GN=HIST3H2BB PE=1 SV=3 |

|  |  |
| --- | --- |
|  | Check to include this hit in error tolerant search or archive report |
|  |  |

|  |  |  |  |  |  |  |  |  |  |  |  |
| --- | --- | --- | --- | --- | --- | --- | --- | --- | --- | --- | --- |
|  | **Query** | **Observed** | **Mr(expt)** | **Mr(calc)** | **ppm** | **Miss** | **Score** | **Expect** | **Rank** | **Unique** | **Peptide** |
|  | 242 | **414.7133** | **827.4120** | **827.4137** | **-2.05** | **0** | **1** | **8.8** | **4** | **U** | **K.HAVSEGTK.A** |
|  | 718 | **477.3054** | **952.5963** | **952.5957** | **0.63** | **0** | **18** | **0.046** | **1** | **U** | **R.LLLPGELAK.H** |
|  | 2919 | 487.2594 | 1458.7564 | 1458.7579 | -1.03 | 1 | 5 | 5.4 | 7 | U | R.IASEASRLAHYNK.R |
|  | 3657 | **888.4084** | **1774.8022** | **1774.8018** | **0.22** | **0** | **5** | **3.1** | **1** | **U** | **K.AMGIMNSFVNDIFER.I + 2 Oxidation (M)** |

  

|  |  |
| --- | --- |
|  | |
|  | **Proteins matching the same set of peptides:** |

|  |  |
| --- | --- |
|  | sp|Q99880|H2B1L\_HUMAN    **Mass:** 13944    **Score:** 18     **Matches:** 4(1)  **Sequences:** 4(1) |
|  | Histone H2B type 1-L OS=Homo sapiens OX=9606 GN=HIST1H2BL PE=1 SV=3 |

---

|  |  |
| --- | --- |
| **176.** | sp|Q14393|GAS6\_HUMAN    **Mass:** 76873    **Score:** 18     **Matches:** 1(0)  **Sequences:** 1(0)  **emPAI:** 0.04 |
|  | Growth arrest-specific protein 6 OS=Homo sapiens OX=9606 GN=GAS6 PE=1 SV=3 |

|  |  |
| --- | --- |
|  | Check to include this hit in error tolerant search or archive report |
|  |  |

|  |  |  |  |  |  |  |  |  |  |  |  |
| --- | --- | --- | --- | --- | --- | --- | --- | --- | --- | --- | --- |
|  | **Query** | **Observed** | **Mr(expt)** | **Mr(calc)** | **ppm** | **Miss** | **Score** | **Expect** | **Rank** | **Unique** | **Peptide** |
|  | 2321 | **658.3531** | **1314.6916** | **1314.6932** | **-1.19** | **0** | **18** | **0.3** | **1** | **U** | **K.IAVAGDLFQPER.G** |

  


---

|  |  |
| --- | --- |
| **177.** | sp|O60437|PEPL\_HUMAN    **Mass:** 205193   **Score:** 18     **Matches:** 12(0)  **Sequences:** 5(0)  **emPAI:** 0.02 |
|  | Periplakin OS=Homo sapiens OX=9606 GN=PPL PE=1 SV=4 |

|  |  |
| --- | --- |
|  | Check to include this hit in error tolerant search or archive report |
|  |  |

|  |  |  |  |  |  |  |  |  |  |  |  |
| --- | --- | --- | --- | --- | --- | --- | --- | --- | --- | --- | --- |
|  | **Query** | **Observed** | **Mr(expt)** | **Mr(calc)** | **ppm** | **Miss** | **Score** | **Expect** | **Rank** | **Unique** | **Peptide** |
|  | 359 | **423.7175** | **845.4205** | **845.4243** | **-4.45** | **0** | **2** | **10** | **5** | **U** | **R.ALEQETR.D** |
|  | 360 | **423.7186** | **845.4226** | **845.4243** | **-1.99** | **0** | **(1)** | **13** | **7** | **U** | **R.ALEQETR.D** |
|  | 1427 | **557.2784** | **1112.5422** | **1112.5574** | **-13.69** | **0** | **(9)** | **1.5** | **1** | **U** | **R.NQGPQESVVR.K** |
|  | 1428 | **557.2795** | **1112.5444** | **1112.5574** | **-11.71** | **0** | **(18)** | **0.19** | **1** | **U** | **R.NQGPQESVVR.K** |
|  | 1429 | **557.2795** | **1112.5445** | **1112.5574** | **-11.60** | **0** | **19** | **0.17** | **1** | **U** | **R.NQGPQESVVR.K** |
|  | 1430 | **557.2797** | **1112.5449** | **1112.5574** | **-11.28** | **0** | **(18)** | **0.22** | **1** | **U** | **R.NQGPQESVVR.K** |
|  | 1431 | **557.2802** | **1112.5457** | **1112.5574** | **-10.50** | **0** | **(16)** | **0.32** | **1** | **U** | **R.NQGPQESVVR.K** |
|  | 1432 | **557.2804** | **1112.5462** | **1112.5574** | **-10.05** | **0** | **(15)** | **0.4** | **1** | **U** | **R.NQGPQESVVR.K** |
|  | 1433 | **557.2804** | **1112.5462** | **1112.5574** | **-10.05** | **0** | **(5)** | **4.6** | **1** | **U** | **R.NQGPQESVVR.K** |
|  | 1584 | 572.3210 | 1142.6275 | 1142.6295 | -1.73 | 1 | 5 | 5.1 | 2 | U | R.LELVEQERK.Y |
|  | 1730 | **589.8046** | **1177.5947** | **1177.5979** | **-2.71** | **0** | **3** | **9** | **3** | **U** | **K.YEDVVQGLQK.R** |
|  | 1863 | **601.8121** | **1201.6097** | **1201.5979** | **9.86** | **0** | **2** | **12** | **3** | **U** | **K.IWALEEENAK.V** |

  


---

|  |  |
| --- | --- |
| **178.** | sp|Q9UM47|NOTC3\_HUMAN    **Mass:** 256640   **Score:** 17     **Matches:** 3(0)  **Sequences:** 2(0)  **emPAI:** 0.01 |
|  | Neurogenic locus notch homolog protein 3 OS=Homo sapiens OX=9606 GN=NOTCH3 PE=1 SV=2 |

|  |  |
| --- | --- |
|  | Check to include this hit in error tolerant search or archive report |
|  |  |

|  |  |  |  |  |  |  |  |  |  |  |  |
| --- | --- | --- | --- | --- | --- | --- | --- | --- | --- | --- | --- |
|  | **Query** | **Observed** | **Mr(expt)** | **Mr(calc)** | **ppm** | **Miss** | **Score** | **Expect** | **Rank** | **Unique** | **Peptide** |
|  | 956 | **510.7713** | **1019.5280** | **1019.5440** | **-15.65** | **0** | **17** | **0.32** | **1** | **U** | **R.LDFPYPLR.D** |
|  | 958 | **510.7721** | **1019.5296** | **1019.5440** | **-14.10** | **0** | **(9)** | **2.1** | **2** | **U** | **R.LDFPYPLR.D** |
|  | 2520 | **455.5727** | **1363.6962** | **1363.7208** | **-18.07** | **1** | **1** | **15** | **9** | **U** | **R.EITDHLDRLPR.D** |

  


---

|  |  |
| --- | --- |
| **179.** | sp|O60858|TRI13\_HUMAN    **Mass:** 47812    **Score:** 17     **Matches:** 1(0)  **Sequences:** 1(0)  **emPAI:** 0.07 |
|  | E3 ubiquitin-protein ligase TRIM13 OS=Homo sapiens OX=9606 GN=TRIM13 PE=1 SV=2 |

|  |  |
| --- | --- |
|  | Check to include this hit in error tolerant search or archive report |
|  |  |

|  |  |  |  |  |  |  |  |  |  |  |  |
| --- | --- | --- | --- | --- | --- | --- | --- | --- | --- | --- | --- |
|  | **Query** | **Observed** | **Mr(expt)** | **Mr(calc)** | **ppm** | **Miss** | **Score** | **Expect** | **Rank** | **Unique** | **Peptide** |
|  | 2999 | **497.9219** | **1490.7438** | **1490.7439** | **-0.05** | **0** | **17** | **0.3** | **1** | **U** | **K.LAVMQAYDPEINK.L** |

  


---

|  |  |
| --- | --- |
| **180.** | sp|Q5VV41|ARHGG\_HUMAN    **Mass:** 80340    **Score:** 17     **Matches:** 1(1)  **Sequences:** 1(1)  **emPAI:** 0.04 |
|  | Rho guanine nucleotide exchange factor 16 OS=Homo sapiens OX=9606 GN=ARHGEF16 PE=1 SV=1 |

|  |  |
| --- | --- |
|  | Check to include this hit in error tolerant search or archive report |
|  |  |

|  |  |  |  |  |  |  |  |  |  |  |  |
| --- | --- | --- | --- | --- | --- | --- | --- | --- | --- | --- | --- |
|  | **Query** | **Observed** | **Mr(expt)** | **Mr(calc)** | **ppm** | **Miss** | **Score** | **Expect** | **Rank** | **Unique** | **Peptide** |
|  | 2679 | **702.2988** | **1402.5831** | **1402.5830** | **0.08** | **1** | **17** | **0.047** | **1** | **U** | **R.QCNEGAHRMER.M + Oxidation (M)** |

  


---

|  |  |
| --- | --- |
| **181.** | sp|Q9UFH2|DYH17\_HUMAN    **Mass:** 512751   **Score:** 16     **Matches:** 10(0)  **Sequences:** 9(0)  **emPAI:** 0.01 |
|  | Dynein heavy chain 17, axonemal OS=Homo sapiens OX=9606 GN=DNAH17 PE=1 SV=3 |

|  |  |
| --- | --- |
|  | Check to include this hit in error tolerant search or archive report |
|  |  |

|  |  |  |  |  |  |  |  |  |  |  |  |
| --- | --- | --- | --- | --- | --- | --- | --- | --- | --- | --- | --- |
|  | **Query** | **Observed** | **Mr(expt)** | **Mr(calc)** | **ppm** | **Miss** | **Score** | **Expect** | **Rank** | **Unique** | **Peptide** |
|  | 51 | **367.1971** | **732.3797** | **732.3806** | **-1.23** | **0** | **5** | **9** | **10** |  | **R.DFNIPK.I** |
|  | 127 | **393.2460** | **784.4774** | **784.4919** | **-18.42** | **1** | **4** | **5.4** | **1** | **U** | **K.AIRLGQK.S** |
|  | 946 | 508.2933 | 1014.5720 | 1014.5597 | 12.2 | 0 | 5 | 5.8 | 5 | U | K.AVLDDILEK.I |
|  | 957 | **510.7719** | **1019.5293** | **1019.5247** | **4.45** | **1** | **10** | **1.7** | **1** |  | **K.TETTKDLGR.A** |
|  | 985 | 513.7642 | 1025.5139 | 1025.4964 | 17.0 | 1 | 16 | 0.3 | 1 | U | K.MGSKFVEGR.S + Oxidation (M) |
|  | 1111 | **523.2863** | **1044.5581** | **1044.5451** | **12.4** | **0** | **1** | **15** | **4** | **U** | **K.ELEDSLLAR.L** |
|  | 3088 | 762.3982 | 1522.7818 | 1522.8038 | -14.42 | 1 | 4 | 5.3 | 4 | U | R.EILHNLQNRMQK.A |
|  | 3093 | **763.8790** | **1525.7435** | **1525.7156** | **18.3** | **0** | **(0)** | **11** | **3** | **U** | **K.IPETFNMAEIMAK.A + 2 Oxidation (M)** |
|  | 3096 | **763.8792** | **1525.7439** | **1525.7156** | **18.5** | **0** | **1** | **9.1** | **5** | **U** | **K.IPETFNMAEIMAK.A + 2 Oxidation (M)** |
|  | 3271 | **399.7425** | **1594.9410** | **1594.9155** | **16.0** | **1** | **1** | **1.8** | **4** | **U** | **K.VLTLASNERIPLNR.T** |

  


---

|  |  |
| --- | --- |
| **182.** | sp|Q99536|VAT1\_HUMAN    **Mass:** 42122    **Score:** 16     **Matches:** 2(0)  **Sequences:** 2(0)  **emPAI:** 0.08 |
|  | Synaptic vesicle membrane protein VAT-1 homolog OS=Homo sapiens OX=9606 GN=VAT1 PE=1 SV=2 |

|  |  |
| --- | --- |
|  | Check to include this hit in error tolerant search or archive report |
|  |  |

|  |  |  |  |  |  |  |  |  |  |  |  |
| --- | --- | --- | --- | --- | --- | --- | --- | --- | --- | --- | --- |
|  | **Query** | **Observed** | **Mr(expt)** | **Mr(calc)** | **ppm** | **Miss** | **Score** | **Expect** | **Rank** | **Unique** | **Peptide** |
|  | 1509 | 565.8023 | 1129.5901 | 1129.6026 | -11.09 | 1 | 5 | 6.5 | 8 | U | K.AGDRVMVLNR.S |
|  | 2974 | **740.4016** | **1478.7885** | **1478.7803** | **5.57** | **0** | **16** | **0.27** | **1** | **U** | **K.VVTYGMANLLTGPK.R + Oxidation (M)** |

  


---

|  |  |
| --- | --- |
| **183.** | sp|Q96QD5|DEPD7\_HUMAN    **Mass:** 58615    **Score:** 16     **Matches:** 2(0)  **Sequences:** 1(0)  **emPAI:** 0.06 |
|  | DEP domain-containing protein 7 OS=Homo sapiens OX=9606 GN=DEPDC7 PE=2 SV=1 |

|  |  |
| --- | --- |
|  | Check to include this hit in error tolerant search or archive report |
|  |  |

|  |  |  |  |  |  |  |  |  |  |  |  |
| --- | --- | --- | --- | --- | --- | --- | --- | --- | --- | --- | --- |
|  | **Query** | **Observed** | **Mr(expt)** | **Mr(calc)** | **ppm** | **Miss** | **Score** | **Expect** | **Rank** | **Unique** | **Peptide** |
|  | 1251 | **539.2639** | **1076.5133** | **1076.5284** | **-14.06** | **1** | **16** | **0.38** | **1** | **U** | **K.ESDNRMVVK.R** |
|  | 1252 | **539.2817** | **1076.5489** | **1076.5284** | **19.0** | **1** | **(12)** | **1.2** | **1** | **U** | **K.ESDNRMVVK.R** |

  


---

|  |  |
| --- | --- |
| **184.** | sp|E7EW31|PROB1\_HUMAN    **Mass:** 107308   **Score:** 16     **Matches:** 4(0)  **Sequences:** 2(0)  **emPAI:** 0.03 |
|  | Proline-rich basic protein 1 OS=Homo sapiens OX=9606 GN=PROB1 PE=2 SV=2 |

|  |  |
| --- | --- |
|  | Check to include this hit in error tolerant search or archive report |
|  |  |

|  |  |  |  |  |  |  |  |  |  |  |  |
| --- | --- | --- | --- | --- | --- | --- | --- | --- | --- | --- | --- |
|  | **Query** | **Observed** | **Mr(expt)** | **Mr(calc)** | **ppm** | **Miss** | **Score** | **Expect** | **Rank** | **Unique** | **Peptide** |
|  | 578 | **456.2322** | **910.4499** | **910.4410** | **9.85** | **0** | **16** | **0.31** | **1** | **U** | **R.HGPGSGFPR.G** |
|  | 579 | **456.2333** | **910.4521** | **910.4410** | **12.3** | **0** | **(15)** | **0.41** | **1** | **U** | **R.HGPGSGFPR.G** |
|  | 580 | **456.2334** | **910.4522** | **910.4410** | **12.4** | **0** | **(15)** | **0.37** | **1** | **U** | **R.HGPGSGFPR.G** |
|  | 2912 | **728.8339** | **1455.6532** | **1455.6565** | **-2.28** | **0** | **0** | **6.8** | **1** | **U** | **R.CPSPQNLSPWDR.T** |

  


---

|  |  |
| --- | --- |
| **185.** | sp|Q96FV9|THOC1\_HUMAN    **Mass:** 76360    **Score:** 16     **Matches:** 3(1)  **Sequences:** 3(1)  **emPAI:** 0.04 |
|  | THO complex subunit 1 OS=Homo sapiens OX=9606 GN=THOC1 PE=1 SV=1 |

|  |  |
| --- | --- |
|  | Check to include this hit in error tolerant search or archive report |
|  |  |

|  |  |  |  |  |  |  |  |  |  |  |  |
| --- | --- | --- | --- | --- | --- | --- | --- | --- | --- | --- | --- |
|  | **Query** | **Observed** | **Mr(expt)** | **Mr(calc)** | **ppm** | **Miss** | **Score** | **Expect** | **Rank** | **Unique** | **Peptide** |
|  | 196 | 406.7837 | 811.5528 | 811.5392 | 16.8 | 1 | 16 | 0.042 | 2 | U | R.ALRLLAR.R |
|  | 1794 | **595.8129** | **1189.6112** | **1189.6051** | **5.12** | **1** | **2** | **11** | **5** | **U** | **K.ERTSDTKPTR.I** |
|  | 4762 | **867.1259** | **3464.4743** | **3464.4218** | **15.2** | **0** | **1** | **1.4** | **1** | **U** | **R.EEGMDVEEGEMGDEEAPTTCSIPIDYNLYR.K + Oxidation (M)** |

  


---

|  |  |
| --- | --- |
| **186.** | sp|Q5SYB0|FRPD1\_HUMAN    **Mass:** 175723   **Score:** 13     **Matches:** 2(0)  **Sequences:** 2(0)  **emPAI:** 0.02 |
|  | FERM and PDZ domain-containing protein 1 OS=Homo sapiens OX=9606 GN=FRMPD1 PE=1 SV=1 |

|  |  |
| --- | --- |
|  | Check to include this hit in error tolerant search or archive report |
|  |  |

|  |  |  |  |  |  |  |  |  |  |  |  |
| --- | --- | --- | --- | --- | --- | --- | --- | --- | --- | --- | --- |
|  | **Query** | **Observed** | **Mr(expt)** | **Mr(calc)** | **ppm** | **Miss** | **Score** | **Expect** | **Rank** | **Unique** | **Peptide** |
|  | 161 | **401.2619** | **800.5093** | **800.5007** | **10.7** | **0** | **3** | **2.2** | **3** | **U** | **K.DIILTVK.E** |
|  | 3031 | **750.3423** | **1498.6700** | **1498.6973** | **-18.23** | **0** | **13** | **0.37** | **1** | **U** | **-.MEELETSLFQTR.K + Oxidation (M)** |

  


---

**Peptide matches not assigned to protein hits:** (no details means no match)  
  

|  |  |  |  |  |  |  |  |  |  |  |  |
| --- | --- | --- | --- | --- | --- | --- | --- | --- | --- | --- | --- |
|  | **Query** | **Observed** | **Mr(expt)** | **Mr(calc)** | **ppm** | **Miss** | **Score** | **Expect** | **Rank** | **Unique** | **Peptide** |
|  | 626 | 465.2551 | 928.4956 | 928.4913 | 4.73 | 1 | 25 | 0.06 | 1 |  | RAPPVSMR + Oxidation (M) |
|  | 2372 | **664.8555** | **1327.6964** | **1327.6745** | **16.5** | **1** | **24** | **0.062** | **1** |  | **SAAEAGGVFHRAR** |
|  | 691 | **472.7695** | **943.5245** | **943.5240** | **0.59** | **0** | **23** | **0.11** | **1** |  | **AGLQFPVGR** |
|  | 3018 | **747.8658** | **1493.7170** | **1493.7144** | **1.76** | **1** | **23** | **0.062** | **1** |  | **KEMSANTVLDSQR + Oxidation (M)** |
|  | 168 | **401.7427** | **801.4707** | **801.4709** | **-0.13** | **0** | **23** | **0.1** | **1** |  | **LGSAVVTR** |
|  | 2282 | **436.8898** | **1307.6477** | **1307.6656** | **-13.72** | **1** | **22** | **0.088** | **1** |  | **MLRADGDFLVR + Oxidation (M)** |
|  | 169 | **401.7428** | **801.4711** | **801.4709** | **0.31** | **0** | **22** | **0.12** | **1** |  | **LGSAVVTR** |
|  | 571 | **453.7558** | **905.4971** | **905.4793** | **19.7** | **0** | **22** | **0.094** | **1** |  | **KPMLFDR** |
|  | 7 | **353.6977** | **705.3808** | **705.3843** | **-5.06** | **0** | **22** | **0.12** | **1** |  | **TMLAVR + Oxidation (M)** |
|  | 1302 | 544.3090 | 1086.6035 | 1086.5921 | 10.5 | 0 | 21 | 0.14 | 1 |  | GEEVTILAQK |
|  | 1784 | **396.8879** | **1187.6420** | **1187.6411** | **0.74** | **0** | **21** | **0.12** | **1** |  | **GAFGKPQGTVAR** |
|  | 2783 | **714.3976** | **1426.7806** | **1426.7681** | **8.78** | **0** | **21** | **0.1** | **1** |  | **IQNNPFGIQIQR** |
|  | 51 | **367.1971** | **732.3797** | **732.3878** | **-11.07** | **1** | **21** | **0.22** | **1** |  | **RSLDSR** |
|  | 2151 | **639.3093** | **1276.6041** | **1276.6220** | **-14.05** | **0** | **21** | **0.11** | **1** |  | **LDAILTDMEEK** |
|  | 3398 | 554.6013 | 1660.7820 | 1660.7805 | 0.86 | 0 | 21 | 0.1 | 1 |  | HSSTPDHTSTLEPPR |
|  | 1860 | **601.8054** | **1201.5962** | **1201.6051** | **-7.42** | **1** | **20** | **0.14** | **1** |  | **TNLADDIRER** |
|  | 3115 | **766.9075** | **1531.8005** | **1531.7916** | **5.84** | **0** | **20** | **0.13** | **1** |  | **MLQEQLAEVAVSAK + Oxidation (M)** |
|  | 690 | **472.7693** | **943.5241** | **943.5240** | **0.14** | **0** | **20** | **0.21** | **1** |  | **AGLQFPVGR** |
|  | 1096 | **523.2856** | **1044.5566** | **1044.5716** | **-14.36** | **0** | **20** | **0.18** | **1** |  | **LSSAHVYLR** |
|  | 4407 | **766.0571** | **2295.1494** | **2295.1616** | **-5.34** | **1** | **20** | **0.097** | **1** |  | **AFLRHLEGGCSVPVAVHTAMK + Oxidation (M)** |
|  | 88 | **379.7149** | **757.4152** | **757.4082** | **9.16** | **0** | **20** | **0.2** | **1** |  | **QDIVAGR** |
|  | 6 | **353.6974** | **705.3803** | **705.3843** | **-5.74** | **0** | **20** | **0.2** | **1** |  | **TMLAVR + Oxidation (M)** |
|  | 3109 | **511.5986** | **1531.7739** | **1531.7630** | **7.08** | **1** | **19** | **0.18** | **1** |  | **LENQWETSIREK** |
|  | 3649 | **589.9728** | **1766.8965** | **1766.9026** | **-3.42** | **0** | **19** | **0.14** | **1** |  | **VPGNTPLLFSTYVMGR + Oxidation (M)** |
|  | 167 | **401.7424** | **801.4703** | **801.4709** | **-0.68** | **0** | **19** | **0.24** | **1** |  | **LGSAVVTR** |
|  | 54 | **369.1898** | **736.3651** | **736.3538** | **15.5** | **1** | **19** | **0.19** | **1** |  | **TKMGER + Oxidation (M)** |
|  | 2578 | **691.8616** | **1381.7086** | **1381.7131** | **-3.27** | **1** | **19** | **0.2** | **1** |  | **KMALNSLMSLMK + Oxidation (M)** |
|  | 2500 | **679.8550** | **1357.6955** | **1357.6950** | **0.41** | **1** | **19** | **0.22** | **1** |  | **EVEQSRQEVVR** |
|  | 544 | **450.2697** | **898.5249** | **898.5348** | **-11.09** | **1** | **19** | **0.14** | **1** |  | **VATISPRR** |
|  | 3353 | **819.9324** | **1637.8503** | **1637.8195** | **18.8** | **1** | **19** | **0.15** | **1** |  | **MRAVSANYSTGSPAVK** |
|  | 2271 | **653.7962** | **1305.5778** | **1305.5870** | **-7.04** | **1** | **19** | **0.071** | **1** |  | **GEPSEEKNAMAK + Oxidation (M)** |
|  | 2790 | **714.8481** | **1427.6816** | **1427.6645** | **12.0** | **0** | **19** | **0.16** | **1** |  | **TMMLMLCDLLR + 2 Oxidation (M)** |
|  | 709 | **475.2426** | **948.4707** | **948.4698** | **0.86** | **0** | **18** | **0.26** | **1** |  | **MINTDLSR** |
|  | 387 | **428.7659** | **855.5172** | **855.5290** | **-13.81** | **1** | **18** | **0.15** | **1** |  | **RDILALR** |
|  | 472 | **438.2565** | **874.4984** | **874.4872** | **12.8** | **1** | **18** | **0.21** | **1** |  | **KSQGLQSK** |
|  | 1420 | **555.7953** | **1109.5761** | **1109.5829** | **-6.11** | **0** | **18** | **0.21** | **1** |  | **GNSGPPGIVGQK** |
|  | 1513 | **565.8124** | **1129.6102** | **1129.6131** | **-2.60** | **1** | **18** | **0.3** | **1** |  | **EYKDHVLVK** |
|  | 347 | **422.7298** | **843.4451** | **843.4563** | **-13.21** | **0** | **18** | **0.3** | **1** |  | **AVNQSIGR** |
|  | 47 | **366.7198** | **731.4251** | **731.4177** | **10.1** | **0** | **18** | **0.27** | **1** |  | **INSSAIK** |
|  | 3063 | 379.6925 | 1514.7408 | 1514.7486 | -5.17 | 0 | 18 | 0.2 | 1 |  | MMQTILHFPQNR |
|  | 2145 | **637.3178** | **1272.6209** | **1272.6422** | **-16.68** | **1** | **18** | **0.22** | **1** |  | **IEEARSQANQK** |
|  | 2761 | **712.3691** | **1422.7236** | **1422.7215** | **1.47** | **0** | **18** | **0.24** | **1** |  | **QENQNNLLSHVK** |
|  | 1011 | **515.7805** | **1029.5464** | **1029.5454** | **0.92** | **1** | **18** | **0.3** | **1** |  | **LEEKENIR** |
|  | 38 | **363.6705** | **725.3264** | **725.3166** | **13.4** | **0** | **18** | **0.1** | **1** |  | **YEVCR** |
|  | 3606 | **585.6046** | **1753.7920** | **1753.7829** | **5.22** | **0** | **18** | **0.15** | **1** |  | **ACASEEVPTLTYEER** |
|  | 935 | **506.7932** | **1011.5718** | **1011.5825** | **-10.59** | **1** | **18** | **0.15** | **1** |  | **VSPTSPRLR** |
|  | 420 | 435.7233 | 869.4321 | 869.4467 | -16.82 | 1 | 18 | 0.2 | 1 |  | AANREGPR |
|  | 1933 | **612.8008** | **1223.5870** | **1223.6047** | **-14.48** | **0** | **18** | **0.2** | **1** |  | **EGLGPHGPSFAR** |
|  | 2043 | 417.2111 | 1248.6115 | 1248.6251 | -10.87 | 0 | 18 | 0.29 | 1 |  | FTFRPPNNEK |
|  | 505 | **442.7636** | **883.5127** | **883.5127** | **-0.03** | **0** | **17** | **0.13** | **1** |  | **LADILSPR** |
|  | 4448 | **786.0647** | **2355.1723** | **2355.1707** | **0.69** | **0** | **17** | **0.2** | **1** |  | **SSDSSVIVQPFSKPGYITNSSR** |
|  | 1982 | **616.8341** | **1231.6535** | **1231.6561** | **-2.06** | **0** | **17** | **0.32** | **1** |  | **LWTLVSEQTR** |
|  | 4139 | **676.9826** | **2027.9260** | **2027.8928** | **16.4** | **0** | **17** | **0.13** | **1** |  | **MPAISDQDMSAYLAEQSR + Oxidation (M)** |
|  | 1697 | **585.7900** | **1169.5655** | **1169.5499** | **13.4** | **0** | **17** | **0.22** | **1** |  | **RPSSMYSTGGK** |
|  | 3358 | **819.9523** | **1637.8900** | **1637.9042** | **-8.66** | **1** | **17** | **0.14** | **1** |  | **LPWWRESSPLVLR** |
|  | 2284 | **436.8900** | **1307.6480** | **1307.6656** | **-13.45** | **1** | **17** | **0.31** | **1** |  | **MLRADGDFLVR + Oxidation (M)** |
|  | 512 | **445.2598** | **888.5050** | **888.5029** | **2.47** | **1** | **17** | **0.33** | **1** |  | **KSVESALR** |
|  | 3288 | **804.9009** | **1607.7873** | **1607.7647** | **14.1** | **1** | **17** | **0.34** | **1** |  | **MCRALEDQLSEIK + Oxidation (M)** |
|  | 3787 | **913.9039** | **1825.7932** | **1825.8022** | **-4.93** | **1** | **17** | **0.1** | **1** |  | **HSADLPHKCSDCLMR** |
|  | 2208 | 431.2384 | 1290.6933 | 1290.6932 | 0.08 | 0 | 17 | 0.28 | 1 |  | YEGILANGVLSR |
|  | 731 | **478.7828** | **955.5510** | **955.5603** | **-9.76** | **1** | **17** | **0.22** | **1** |  | **KVTPAWVR** |
|  | 3420 | **838.4777** | **1674.9409** | **1674.9165** | **14.5** | **1** | **17** | **0.096** | **1** |  | **QRAEAAVLSGPGPPLGR** |
|  | 3450 | **846.4039** | **1690.7933** | **1690.8162** | **-13.54** | **1** | **17** | **0.24** | **1** |  | **KTGQGDYPLNNELDK** |
|  | 475 | **438.7286** | **875.4427** | **875.4501** | **-8.48** | **0** | **17** | **0.48** | **1** |  | **QPYNNLK** |
|  | 211 | **409.7426** | **817.4707** | **817.4592** | **14.0** | **1** | **17** | **0.35** | **1** |  | **MRGGLLR + Oxidation (M)** |
|  | 3793 | **913.9063** | **1825.7979** | **1825.8200** | **-12.06** | **0** | **17** | **0.12** | **1** |  | **DLGTHMMHLSGNDSGVR** |
|  | 1890 | **604.7855** | **1207.5564** | **1207.5577** | **-1.08** | **0** | **16** | **0.26** | **1** |  | **MDDDLMLALR + Oxidation (M)** |
|  | 1531 | **568.2699** | **1134.5252** | **1134.5305** | **-4.67** | **0** | **16** | **0.27** | **1** |  | **EGVLDFNADR** |
|  | 845 | 495.2309 | 988.4473 | 988.4648 | -17.65 | 0 | 16 | 0.18 | 1 |  | NNMEASVPK |
|  | 1721 | **588.3206** | **1174.6267** | **1174.6267** | **-0.04** | **0** | **16** | **0.42** | **1** |  | **SPAMSTLLDIK** |
|  | 2419 | **671.3420** | **1340.6694** | **1340.6870** | **-13.14** | **0** | **16** | **0.35** | **1** |  | **TPAAAAAMNLASPR** |
|  | 729 | **478.7808** | **955.5470** | **955.5603** | **-13.92** | **1** | **16** | **0.27** | **1** |  | **KVTPAWVR** |
|  | 1182 | **530.7904** | **1059.5663** | **1059.5461** | **19.0** | **1** | **16** | **0.49** | **1** |  | **RIAADFDPR** |
|  | 2935 | **734.3499** | **1466.6853** | **1466.6790** | **4.27** | **0** | **16** | **0.25** | **1** |  | **VVEDNHSYYVSR** |
|  | 2200 | **644.8231** | **1287.6316** | **1287.6241** | **5.79** | **1** | **16** | **0.34** | **1** |  | **AHDMITTERAK + Oxidation (M)** |
|  | 787 | 487.2339 | 972.4532 | 972.4624 | -9.48 | 0 | 16 | 0.2 | 1 |  | ENQNNQVK |
|  | 2951 | **737.8744** | **1473.7342** | **1473.7324** | **1.22** | **0** | **16** | **0.4** | **1** |  | **NQGTSDFLPSRPR** |
|  | 76 | **379.2393** | **756.4641** | **756.4494** | **19.5** | **0** | **16** | **0.41** | **1** |  | **EGIVALR** |
|  | 1696 | **585.7891** | **1169.5636** | **1169.5499** | **11.7** | **0** | **16** | **0.3** | **1** |  | **RPSSMYSTGGK** |
|  | 4080 | **497.2248** | **1984.8702** | **1984.8870** | **-8.48** | **1** | **16** | **0.12** | **1** |  | **FPSSQELEDASCCSLKK** |
|  | 2202 | **645.3428** | **1288.6711** | **1288.6697** | **1.12** | **0** | **16** | **0.49** | **1** |  | **GCLEAGLESLLK** |
|  | 1863 | 601.8121 | 1201.6097 | 1201.6125 | -2.33 | 0 | 16 | 0.51 | 1 |  | LSVSNMVHTAK + Oxidation (M) |
|  | 679 | **471.2273** | **940.4400** | **940.4363** | **4.00** | **0** | **16** | **0.24** | **1** |  | **DGHTLQDR** |
|  | 1226 | **357.1780** | **1068.5123** | **1068.5022** | **9.42** | **0** | **15** | **0.36** | **1** |  | **AVVDHMEPR + Oxidation (M)** |
|  | 2811 | **716.8486** | **1431.6827** | **1431.6841** | **-0.99** | **0** | **15** | **0.36** | **1** |  | **SNLNSLDEQEGVK** |
|  | 1802 | 596.8079 | 1191.6012 | 1191.6248 | -19.81 | 1 | 15 | 0.5 | 1 |  | IGDSLFGAREK |
|  | 3390 | **828.9446** | **1655.8747** | **1655.8777** | **-1.79** | **1** | **15** | **0.34** | **1** |  | **MAADQRPKADTLALR** |
|  | 2702 | 705.8356 | 1409.6567 | 1409.6422 | 10.3 | 1 | 15 | 0.31 | 1 |  | ADLAEEYSNDRK |
|  | 3850 | **926.9949** | **1851.9752** | **1851.9479** | **14.7** | **1** | **15** | **0.27** | **1** |  | **YLQLTQSELSSYHRK** |
|  | 513 | 445.2602 | 888.5058 | 888.4963 | 10.6 | 1 | 15 | 0.49 | 1 |  | RMGSLGLR |
|  | 2501 | **453.5725** | **1357.6957** | **1357.6772** | **13.6** | **1** | **15** | **0.53** | **1** |  | **LRAQMDTSPAPR + Oxidation (M)** |
|  | 4277 | **722.7064** | **2165.0974** | **2165.0613** | **16.7** | **1** | **15** | **0.32** | **1** |  | **SGEKPEADRNPPVLYHNSR** |
|  | 4252 | **1066.0276** | **2130.0406** | **2130.0317** | **4.20** | **0** | **15** | **0.31** | **1** |  | **LGFSKPQAFMDHTQSHGVK + Oxidation (M)** |
|  | 2442 | **449.5765** | **1345.7078** | **1345.6878** | **14.9** | **0** | **15** | **0.56** | **1** |  | **LDPAGSFVPTNTK** |
|  | 2526 | **455.8967** | **1364.6684** | **1364.6792** | **-7.92** | **1** | **15** | **0.52** | **1** |  | **KNVIMELSEMR + Oxidation (M)** |
|  | 260 | **417.2024** | **832.3902** | **832.4039** | **-16.46** | **0** | **15** | **0.47** | **1** |  | **TSAAAEQR** |
|  | 504 | **442.7634** | **883.5123** | **883.5127** | **-0.44** | **0** | **15** | **0.25** | **1** |  | **LADILSPR** |
|  | 2179 | **642.8782** | **1283.7419** | **1283.7449** | **-2.35** | **0** | **15** | **0.22** | **1** |  | **VLATVTKPVGGDK** |
|  | 104 | **384.7326** | **767.4507** | **767.4402** | **13.7** | **1** | **15** | **0.21** | **1** |  | **HKSQLR** |
|  | 3840 | **617.9504** | **1850.8293** | **1850.8534** | **-13.01** | **0** | **15** | **0.22** | **1** |  | **SGEGQEDAGELDFSGLLK** |
|  | 281 | **421.7577** | **841.5009** | **841.5021** | **-1.41** | **1** | **15** | **0.36** | **1** |  | **VAGAATPKK** |
|  | 2632 | 464.5648 | 1390.6727 | 1390.6616 | 7.99 | 0 | 15 | 0.5 | 1 |  | VEESSWLIEDGK |
|  | 3820 | **615.2859** | **1842.8360** | **1842.8569** | **-11.33** | **1** | **15** | **0.34** | **1** |  | **KQSAGPNSPTGGGGGGGSGGTR** |
|  | 1772 | **592.8661** | **1183.7177** | **1183.7289** | **-9.38** | **1** | **15** | **0.15** | **1** |  | **GLVAQLQKTVK** |
|  | 4333 | **737.7067** | **2210.0984** | **2210.1113** | **-5.87** | **0** | **15** | **0.37** | **1** |  | **MNPASGALSPLPRPNGTANTTK + Oxidation (M)** |
|  | 2408 | **669.9144** | **1337.8143** | **1337.8295** | **-11.39** | **1** | **15** | **0.056** | **1** |  | **RAIPPPLPPKPR** |
|  | 3528 | **862.9445** | **1723.8745** | **1723.8676** | **4.02** | **0** | **15** | **0.49** | **1** |  | **KPSIAAVVGSMDGHPSR + Oxidation (M)** |
|  | 3754 | **604.6180** | **1810.8323** | **1810.8068** | **14.1** | **0** | **15** | **0.29** | **1** |  | **GTNSSDSEELSAGESITK** |
|  | 2579 | **691.8630** | **1381.7115** | **1381.7131** | **-1.14** | **1** | **15** | **0.52** | **1** |  | **KMALNSLMSLMK + Oxidation (M)** |
|  | 3033 | **750.3705** | **1498.7265** | **1498.7450** | **-12.30** | **0** | **15** | **0.42** | **1** |  | **ADEICIAGSPLTPR** |
|  | 1797 | 595.8425 | 1189.6705 | 1189.6707 | -0.12 | 0 | 15 | 0.34 | 1 |  | ILQFATLEQK |
|  | 2293 | **655.3774** | **1308.7403** | **1308.7401** | **0.17** | **0** | **15** | **0.24** | **1** |  | **IALLPNNAADLGK** |
|  | 3794 | **913.9064** | **1825.7982** | **1825.8200** | **-11.93** | **0** | **15** | **0.19** | **1** |  | **DLGTHMMHLSGNDSGVR** |
|  | 3445 | **562.2888** | **1683.8446** | **1683.8726** | **-16.62** | **0** | **15** | **0.42** | **1** |  | **QSLNCHGSSLLSLLR** |
|  | 200 | **408.2342** | **814.4538** | **814.4548** | **-1.33** | **0** | **15** | **0.82** | **1** |  | **ELIIGDR** |
|  | 421 | **435.7559** | **869.4972** | **869.5083** | **-12.72** | **0** | **15** | **0.27** | **1** |  | **VVQANALR** |
|  | 1115 | 523.2911 | 1044.5677 | 1044.5829 | -14.50 | 1 | 15 | 0.57 | 1 |  | GVGSRGFPLR |
|  | 2502 | 679.8686 | 1357.7226 | 1357.7388 | -11.89 | 1 | 15 | 0.5 | 1 |  | ITVNPDGKIMVR + Oxidation (M) |
|  | 3060 | **505.2475** | **1512.7207** | **1512.7494** | **-18.97** | **1** | **15** | **0.43** | **1** |  | **DYTSGAMLTGELKK** |
|  | 2290 | **655.3229** | **1308.6313** | **1308.6211** | **7.83** | **0** | **15** | **0.46** | **1** |  | **QNFEIGNNFAR** |
|  | 4099 | **1007.9629** | **2013.9112** | **2013.8810** | **15.0** | **1** | **15** | **0.27** | **1** |  | **SSGQSNNNSDTCAEFRIK** |
|  | 606 | **460.7453** | **919.4760** | **919.4797** | **-4.03** | **0** | **15** | **0.59** | **1** |  | **AVSSQMIGK** |
|  | 680 | **471.2273** | **940.4400** | **940.4363** | **4.00** | **0** | **14** | **0.31** | **1** |  | **DGHTLQDR** |
|  | 2275 | **654.3154** | **1306.6162** | **1306.6010** | **11.7** | **0** | **14** | **0.48** | **1** |  | **MAEEGAVAVCVR + Oxidation (M)** |
|  | 2997 | 746.3511 | 1490.6877 | 1490.7001 | -8.32 | 0 | 14 | 0.31 | 1 |  | DVPESPQHAADTPK |
|  | 5 | **353.6974** | **705.3802** | **705.3843** | **-5.83** | **0** | **14** | **0.65** | **1** |  | **TMLAVR + Oxidation (M)** |
|  | 1088 | 523.2854 | 1044.5562 | 1044.5386 | 16.9 | 0 | 14 | 0.69 | 1 |  | LGVNDCVLR |
|  | 3978 | **479.5092** | **1914.0075** | **1914.0145** | **-3.64** | **1** | **14** | **0.34** | **1** |  | **IYSGEGEVLLMAHALRR** |
|  | 1876 | 603.7804 | 1205.5462 | 1205.5313 | 12.4 | 0 | 14 | 0.3 | 1 |  | ENPDNLSDFR |
|  | 2793 | **476.9017** | **1427.6834** | **1427.7117** | **-19.82** | **1** | **14** | **0.44** | **1** |  | **QQLEAQRAEAER** |
|  | 242 | 414.7133 | 827.4120 | 827.4151 | -3.66 | 1 | 14 | 0.4 | 1 |  | HSWRSR |
|  | 566 | **453.2574** | **904.5002** | **904.5018** | **-1.74** | **0** | **14** | **0.51** | **1** |  | **APTNIVYK** |
|  | 3108 | **383.9506** | **1531.7731** | **1531.7552** | **11.7** | **0** | **14** | **0.6** | **1** |  | **EQMVAVTEANEALK** |
|  | 1712 | 587.2945 | 1172.5744 | 1172.5682 | 5.34 | 1 | 14 | 0.56 | 1 |  | HGKIMEAMEK |
|  | 2812 | **716.8690** | **1431.7235** | **1431.7504** | **-18.76** | **1** | **14** | **0.62** | **1** |  | **AQMLLAELSRER + Oxidation (M)** |
|  | 4317 | **737.7056** | **2210.0949** | **2210.0637** | **14.1** | **1** | **14** | **0.43** | **1** |  | **DDPDGKQEAKPQQAAGMLSPK** |
|  | 3989 | **642.3022** | **1923.8847** | **1923.9214** | **-19.08** | **0** | **14** | **0.36** | **1** |  | **IGPGDVLTFYDGDDLTAR** |
|  | 2418 | **671.3413** | **1340.6681** | **1340.6507** | **13.0** | **0** | **14** | **0.58** | **1** |  | **NPPAMSPAGQLSR + Oxidation (M)** |
|  | 3710 | **598.9430** | **1793.8073** | **1793.8028** | **2.53** | **0** | **14** | **0.3** | **1** |  | **TGTTGQSGAESGTTEPSAR** |
|  | 3823 | **922.9109** | **1843.8072** | **1843.8015** | **3.09** | **1** | **14** | **0.18** | **1** |  | **CIAFGMKQDSVQCER + Oxidation (M)** |
|  | 312 | **421.7582** | **841.5018** | **841.5021** | **-0.39** | **1** | **14** | **0.4** | **1** |  | **VAGAATPKK** |
|  | 2198 | **644.3127** | **1286.6108** | **1286.6143** | **-2.72** | **0** | **14** | **0.43** | **1** |  | **FSGSGAGTDFTLK** |
|  | 856 | **495.8065** | **989.5985** | **989.5943** | **4.20** | **1** | **14** | **0.13** | **1** |  | **IGVMSLVKK + Oxidation (M)** |
|  | 3480 | **850.4147** | **1698.8149** | **1698.8222** | **-4.29** | **1** | **14** | **0.46** | **1** |  | **MAATFFGEVVKAPCR + Oxidation (M)** |
|  | 540 | **450.2692** | **898.5238** | **898.5348** | **-12.30** | **1** | **14** | **0.44** | **1** |  | **VATISPRR** |
|  | 1223 | **534.7675** | **1067.5205** | **1067.5070** | **12.6** | **0** | **14** | **0.5** | **1** |  | **AGVLFGMSDR + Oxidation (M)** |
|  | 1070 | **523.2836** | **1044.5527** | **1044.5451** | **7.26** | **0** | **14** | **0.77** | **1** |  | **LETELDGLR** |
|  | 4680 | **779.8414** | **3115.3364** | **3115.3458** | **-3.03** | **1** | **14** | **0.069** | **1** |  | **LKETEETPSELSFQDFEYPDYDDYR** |
|  | 2852 | **720.8152** | **1439.6158** | **1439.6133** | **1.75** | **0** | **14** | **0.15** | **1** |  | **MDGASNVTCINSR + Oxidation (M)** |
|  | 2926 | **732.3625** | **1462.7104** | **1462.7353** | **-16.99** | **0** | **14** | **0.62** | **1** |  | **AMFFSMGFIVAVK + Oxidation (M)** |
|  | 685 | **472.2297** | **942.4449** | **942.4447** | **0.21** | **0** | **14** | **0.34** | **1** |  | **YISFEER** |
|  | 20 | **360.1845** | **718.3543** | **718.3432** | **15.5** | **0** | **14** | **1.1** | **1** |  | **ALACER** |
|  | 536 | 449.7770 | 897.5394 | 897.5508 | -12.69 | 1 | 14 | 0.25 | 1 |  | AVIAAAARR |
|  | 1076 | **523.2847** | **1044.5548** | **1044.5386** | **15.5** | **0** | **14** | **0.89** | **1** |  | **LGVNDCVLR** |
|  | 2441 | **673.8532** | **1345.6917** | **1345.6725** | **14.3** | **1** | **14** | **0.8** | **1** |  | **NEKEQELDTLK** |
|  | 1068 | **523.2694** | **1044.5243** | **1044.5386** | **-13.74** | **0** | **14** | **0.81** | **1** |  | **LGVNDCVLR** |
|  | 912 | 503.2720 | 1004.5295 | 1004.5291 | 0.41 | 0 | 14 | 0.75 | 1 |  | FDVATSLPR |
|  | 3759 | **605.3187** | **1812.9342** | **1812.9118** | **12.3** | **1** | **14** | **0.43** | **1** |  | **QIQEDWELAERLQR** |
|  | 2201 | **644.8516** | **1287.6887** | **1287.6969** | **-6.36** | **1** | **14** | **0.65** | **1** |  | **ISAMGKALVDQR** |
|  | 2444 | **673.8824** | **1345.7502** | **1345.7466** | **2.68** | **0** | **14** | **0.49** | **1** |  | **GIIDQGHIQLPR** |
|  | 1296 | **543.8126** | **1085.6106** | **1085.6080** | **2.32** | **0** | **14** | **0.58** | **1** |  | **QLSGQLAELK** |
|  | 1720 | **588.2864** | **1174.5582** | **1174.5764** | **-15.51** | **0** | **14** | **0.52** | **1** |  | **AVMEQANLQR + Oxidation (M)** |
|  | 2653 | **465.5770** | **1393.7093** | **1393.7313** | **-15.84** | **1** | **14** | **0.67** | **1** |  | **EQIHNLEDRLK** |
|  | 4679 | 779.8409 | 3115.3347 | 3115.3458 | -3.58 | 1 | 14 | 0.075 | 1 |  | LKETEETPSELSFQDFEYPDYDDYR |
|  | 4526 | **847.7279** | **2540.1619** | **2540.1676** | **-2.23** | **1** | **14** | **0.3** | **1** |  | **YAGEVYGMIRFSGTTGQMSDLNK + Oxidation (M)** |
|  | 664 | **468.7502** | **935.4859** | **935.4932** | **-7.86** | **0** | **14** | **0.63** | **1** |  | **IGMSMLIR + Oxidation (M)** |
|  | 3239 | **790.8987** | **1579.7829** | **1579.7738** | **5.77** | **1** | **13** | **0.66** | **1** |  | **MKGPEVMAFIEQGK + Oxidation (M)** |
|  | 4250 | **1064.5350** | **2127.0555** | **2127.0742** | **-8.80** | **1** | **13** | **0.47** | **1** |  | **NHIRIVESMQSTLDAEIR + Oxidation (M)** |
|  | 445 | **435.7741** | **869.5337** | **869.5447** | **-12.59** | **1** | **13** | **0.32** | **1** |  | **VVVERLR** |
|  | 2842 | **480.2316** | **1437.6730** | **1437.6996** | **-18.52** | **0** | **13** | **0.49** | **1** |  | **QAMEMVGFLPATK + Oxidation (M)** |
|  | 1222 | **534.7665** | **1067.5185** | **1067.5288** | **-9.59** | **0** | **13** | **0.55** | **1** |  | **DNFFSLTPK** |
|  | 2099 | 631.3443 | 1260.6740 | 1260.6561 | 14.2 | 1 | 13 | 0.69 | 1 |  | ALSETLEEKNK |
|  | 1137 | **526.2639** | **1050.5133** | **1050.5202** | **-6.55** | **0** | **13** | **0.65** | **1** |  | **ITGNMGLAMK + Oxidation (M)** |
|  | 1361 | **550.8320** | **1099.6494** | **1099.6349** | **13.1** | **1** | **13** | **0.39** | **1** |  | **ITRGIEAVGGK** |
|  | 238 | **414.2229** | **826.4313** | **826.4297** | **1.94** | **0** | **13** | **0.58** | **1** |  | **SPAPAQTR** |
|  | 2001 | **619.7899** | **1237.5652** | **1237.5584** | **5.51** | **1** | **13** | **0.48** | **1** |  | **WMITECRDK** |
|  | 1724 | **588.8127** | **1175.6108** | **1175.6186** | **-6.66** | **0** | **13** | **0.91** | **1** |  | **FLDDQGQLIK** |
|  | 3175 | **517.5903** | **1549.7490** | **1549.7518** | **-1.84** | **1** | **13** | **0.67** | **1** |  | **LRGSSMASSVASEPR + Oxidation (M)** |
|  | 2647 | **697.3699** | **1392.7253** | **1392.7401** | **-10.65** | **1** | **13** | **0.73** | **1** |  | **QGLFPNNYVTKI** |
|  | 3789 | **913.9052** | **1825.7959** | **1825.8013** | **-2.98** | **1** | **13** | **0.25** | **1** |  | **KEQSHEDQGHIMDTR + Oxidation (M)** |
|  | 2508 | **681.3460** | **1360.6775** | **1360.7021** | **-18.07** | **0** | **13** | **0.86** | **1** |  | **VTMDAPVSSVALR + Oxidation (M)** |
|  | 119 | **392.7376** | **783.4607** | **783.4464** | **18.3** | **1** | **13** | **0.39** | **1** |  | **GRKPGGGR** |
|  | 1336 | **547.2801** | **1092.5456** | **1092.5299** | **14.4** | **0** | **13** | **0.78** | **1** |  | **TTNIAEETSK** |
|  | 2753 | **711.3561** | **1420.6976** | **1420.7085** | **-7.70** | **0** | **13** | **0.77** | **1** |  | **LLPEESDLYQSK** |
|  | 3755 | **604.6190** | **1810.8352** | **1810.8068** | **15.7** | **0** | **13** | **0.43** | **1** |  | **GTNSSDSEELSAGESITK** |
|  | 854 | **495.7645** | **989.5145** | **989.5215** | **-7.11** | **0** | **13** | **1.2** | **1** |  | **AVLAAMDATK** |
|  | 409 | **433.7372** | **865.4599** | **865.4545** | **6.24** | **0** | **13** | **0.77** | **1** |  | **AVFDLSSK** |
|  | 772 | **484.7616** | **967.5086** | **967.5199** | **-11.71** | **0** | **13** | **0.62** | **1** |  | **IGARPSDPR** |
|  | 1717 | **587.8137** | **1173.6129** | **1173.6129** | **0.03** | **0** | **13** | **0.93** | **1** |  | **VSEEIEDIIK** |
|  | 4166 | **511.7389** | **2042.9263** | **2042.9644** | **-18.62** | **1** | **13** | **0.32** | **1** |  | **VKAEDEALLSEEDDPIDR** |
|  | 2697 | **704.3838** | **1406.7530** | **1406.7630** | **-7.06** | **1** | **13** | **0.56** | **1** |  | **AEHQAASKAAAKPK** |
|  | 2681 | **468.5783** | **1402.7132** | **1402.7303** | **-12.23** | **0** | **13** | **0.81** | **1** |  | **EGSTAQLIINTEK** |
|  | 2481 | 453.2451 | 1356.7135 | 1356.6885 | 18.5 | 0 | 13 | 0.84 | 1 |  | EQSNLLPDNSLK |
|  | 1319 | **546.2712** | **1090.5279** | **1090.5380** | **-9.27** | **1** | **13** | **0.86** | **1** |  | **IHHGDRGGSR** |
|  | 174 | **403.2349** | **804.4552** | **804.4415** | **17.0** | **0** | **13** | **0.58** | **1** |  | **LVESVMK** |
|  | 3537 | **576.5895** | **1726.7466** | **1726.7780** | **-18.19** | **1** | **13** | **0.19** | **1** |  | **HTGAKPFKCNHCDR** |
|  | 8 | **353.6978** | **705.3811** | **705.3843** | **-4.64** | **0** | **13** | **0.93** | **1** |  | **TMLAVR + Oxidation (M)** |
|  | 295 | **421.7581** | **841.5016** | **841.5022** | **-0.69** | **0** | **13** | **0.52** | **1** |  | **AVSVTPIR** |
|  | 3644 | **883.8964** | **1765.7782** | **1765.7723** | **3.31** | **1** | **13** | **0.29** | **1** |  | **DAVQNCCGISKTEER** |
|  | 839 | **494.2954** | **986.5763** | **986.5648** | **11.7** | **0** | **13** | **0.64** | **1** |  | **LLETPISSK** |
|  | 756 | **482.2449** | **962.4752** | **962.4855** | **-10.66** | **1** | **13** | **0.93** | **1** |  | **VAGMDKQAK + Oxidation (M)** |
|  | 2856 | **721.3817** | **1440.7489** | **1440.7321** | **11.6** | **0** | **13** | **0.71** | **1** |  | **QEVLDQALQQNR** |
|  | 2669 | **467.2333** | **1398.6782** | **1398.6793** | **-0.79** | **0** | **13** | **0.63** | **1** |  | **AVVGDAQYHHFR** |
|  | 1736 | **590.3230** | **1178.6314** | **1178.6183** | **11.2** | **1** | **13** | **0.7** | **1** |  | **ALFVSEEEKK** |
|  | 2891 | **725.8806** | **1449.7467** | **1449.7537** | **-4.84** | **1** | **13** | **0.82** | **1** |  | **EMTQAPYLEIKK** |
|  | 431 | **435.7738** | **869.5331** | **869.5447** | **-13.31** | **1** | **13** | **0.37** | **1** |  | **VVVERLR** |
|  | 1718 | 587.8248 | 1173.6350 | 1173.6353 | -0.29 | 1 | 13 | 0.9 | 1 |  | KVISSLQEDR |
|  | 99 | **384.2351** | **766.4557** | **766.4449** | **14.0** | **0** | **13** | **0.43** | **1** |  | **ASLAIHR** |
|  | 1794 | 595.8129 | 1189.6112 | 1189.5914 | 16.7 | 1 | 13 | 0.96 | 1 |  | WVGEGEKMVR |
|  | 4671 | **1018.1707** | **3051.4901** | **3051.5232** | **-10.83** | **1** | **13** | **0.39** | **1** |  | **NFMLHLVSMHDFNLISIMSIDKAVTK + 3 Oxidation (M)** |
|  | 1857 | **601.8046** | **1201.5947** | **1201.6051** | **-8.64** | **1** | **13** | **0.74** | **1** |  | **TNLADDIRER** |
|  | 2492 | **679.8433** | **1357.6721** | **1357.6950** | **-16.86** | **1** | **13** | **0.85** | **1** |  | **VQATDADAGLNRK** |
|  | 3582 | **582.6346** | **1744.8821** | **1744.9148** | **-18.76** | **0** | **13** | **0.87** | **1** |  | **VIFKPPDPDNTFLSR** |
|  | 1187 | **531.2757** | **1060.5368** | **1060.5288** | **7.58** | **0** | **13** | **0.99** | **1** |  | **LEVTDDLEK** |
|  | 543 | **450.2694** | **898.5242** | **898.5097** | **16.2** | **1** | **13** | **0.57** | **1** |  | **AQGAQRLR** |
|  | 3790 | **913.9053** | **1825.7960** | **1825.8013** | **-2.92** | **1** | **13** | **0.28** | **1** |  | **KEQSHEDQGHIMDTR + Oxidation (M)** |
|  | 659 | **468.2448** | **934.4750** | **934.4654** | **10.3** | **1** | **13** | **0.99** | **1** |  | **LTENMRR + Oxidation (M)** |
|  | 3300 | **538.9534** | **1613.8383** | **1613.8414** | **-1.90** | **0** | **13** | **0.7** | **1** |  | **IFHTVTTTDDPVIR** |
|  | 32 | **362.7572** | **723.4999** | **723.5007** | **-1.05** | **0** | **13** | **0.055** | **1** |  | **IILPLR** |
|  | 1731 | **589.8244** | **1177.6342** | **1177.6125** | **18.5** | **0** | **13** | **0.81** | **1** |  | **AATSLMSALAAR + Oxidation (M)** |
|  | 4141 | **507.9892** | **2027.9276** | **2027.8928** | **17.1** | **0** | **13** | **0.39** | **1** |  | **MPAISDQDMSAYLAEQSR + Oxidation (M)** |
|  | 828 | **492.8083** | **983.6020** | **983.6127** | **-10.94** | **0** | **13** | **0.42** | **1** |  | **LLGSALALAR** |
|  | 525 | **448.2351** | **894.4557** | **894.4382** | **19.6** | **0** | **13** | **0.76** | **1** |  | **ATFCALGR** |
|  | 2847 | **720.3628** | **1438.7110** | **1438.7390** | **-19.41** | **1** | **13** | **0.84** | **1** |  | **SVRLGGPGGGAGGAGGGR** |
|  | 3144 | **770.9454** | **1539.8763** | **1539.8660** | **6.67** | **1** | **13** | **0.26** | **1** |  | **KLAIWEQLSPDIK** |
|  | 539 | **450.2691** | **898.5236** | **898.5097** | **15.5** | **1** | **13** | **0.62** | **1** |  | **NLNGGLRR** |
|  | 2440 | **673.8528** | **1345.6910** | **1345.6837** | **5.40** | **0** | **13** | **1.1** | **1** |  | **LGLGDSNNQSTLK** |
|  | 3643 | **883.8958** | **1765.7771** | **1765.7723** | **2.69** | **1** | **13** | **0.3** | **1** |  | **DAVQNCCGISKTEER** |
|  | 1390 | **553.7666** | **1105.5186** | **1105.5377** | **-17.26** | **1** | **12** | **0.85** | **1** |  | **GAGGFGGGGGTRR** |
|  | 3194 | 778.3286 | 1554.6427 | 1554.6694 | -17.21 | 0 | 12 | 0.25 | 1 |  | QEDICMGPLSDYK |
|  | 328 | **421.7582** | **841.5019** | **841.5021** | **-0.25** | **1** | **12** | **0.57** | **1** |  | **AAGGPKLTK** |
|  | 1960 | **410.2351** | **1227.6834** | **1227.6935** | **-8.21** | **1** | **12** | **1.2** | **1** |  | **AQIRNSIIGEK** |
|  | 3810 | **921.9014** | **1841.7882** | **1841.8149** | **-14.49** | **0** | **12** | **0.23** | **1** |  | **DLGTHMMHLSGNDSGVR + Oxidation (M)** |
|  | 3193 | **777.8967** | **1553.7789** | **1553.7548** | **15.5** | **0** | **12** | **0.83** | **1** |  | **ITLDCQNIYTWK** |
|  | 48 | 366.7200 | 731.4255 | 731.4177 | 10.6 | 0 | 12 | 0.97 | 1 |  | SNIGTLK |
|  | 386 | **428.7658** | **855.5171** | **855.5178** | **-0.76** | **0** | **12** | **0.6** | **1** |  | **SPLSLIAR** |
|  | 2535 | **456.5956** | **1366.7649** | **1366.7568** | **5.91** | **1** | **12** | **0.36** | **1** |  | **QPPERIASGAITK** |
|  | 3698 | **897.9080** | **1793.8014** | **1793.8028** | **-0.79** | **0** | **12** | **0.47** | **1** |  | **TGTTGQSGAESGTTEPSAR** |
|  | 1605 | **575.7903** | **1149.5660** | **1149.5852** | **-16.71** | **0** | **12** | **0.86** | **1** |  | **MAVFADLDLR** |
|  | 491 | 441.2632 | 880.5117 | 880.5130 | -1.47 | 0 | 12 | 0.3 | 1 |  | AALPGTVPR |
|  | 3150 | **514.6312** | **1540.8717** | **1540.8573** | **9.31** | **0** | **12** | **0.33** | **1** |  | **NQLTSVPSLISGLGR** |
|  | 1879 | **603.8093** | **1205.6040** | **1205.6148** | **-8.96** | **0** | **12** | **1** | **1** |  | **QDMLALQMIK + Oxidation (M)** |
|  | 759 | **482.7421** | **963.4696** | **963.4556** | **14.6** | **1** | **12** | **1.1** | **1** |  | **GMSRSSSPR** |
|  | 3366 | **822.4451** | **1642.8756** | **1642.8800** | **-2.66** | **0** | **12** | **0.62** | **1** |  | **MVINLCLPQFRPR** |
|  | 1292 | **543.3010** | **1084.5875** | **1084.6063** | **-17.32** | **0** | **12** | **0.82** | **1** |  | **CLVAGLLSPR** |
|  | 303 | **421.7581** | **841.5017** | **841.5022** | **-0.55** | **0** | **12** | **0.6** | **1** |  | **AVSVTPIR** |
|  | 1722 | **588.3207** | **1174.6268** | **1174.6445** | **-15.06** | **0** | **12** | **1.1** | **1** |  | **SGEVTISIELK** |
|  | 1136 | **526.2634** | **1050.5123** | **1050.5202** | **-7.49** | **0** | **12** | **0.87** | **1** |  | **ITGNMGLAMK + Oxidation (M)** |
|  | 2405 | **446.9452** | **1337.8138** | **1337.8104** | **2.54** | **0** | **12** | **0.097** | **1** |  | **LPNALLMLVNLK** |
|  | 760 | **482.7424** | **963.4702** | **963.4556** | **15.2** | **1** | **12** | **1.1** | **1** |  | **GMSRSSSPR** |
|  | 464 | **436.7763** | **871.5379** | **871.5491** | **-12.82** | **0** | **12** | **0.57** | **1** |  | **VVVTVISR** |
|  | 3785 | **913.8946** | **1825.7747** | **1825.7757** | **-0.54** | **0** | **12** | **0.23** | **1** |  | **QQGGGMMMGPGGGGAASLSK + 3 Oxidation (M)** |
|  | 1666 | **582.7979** | **1163.5813** | **1163.5935** | **-10.50** | **0** | **12** | **1** | **1** |  | **TSAEVFQVQR** |
|  | 2068 | **418.9012** | **1253.6817** | **1253.6802** | **1.23** | **0** | **12** | **0.54** | **1** |  | **VNLVALETMHK** |
|  | 2656 | 698.3229 | 1394.6313 | 1394.6170 | 10.3 | 1 | 12 | 0.46 | 1 |  | DGAVKAMEEMNGK + Oxidation (M) |
|  | 2178 | **642.8458** | **1283.6770** | **1283.6721** | **3.77** | **0** | **12** | **0.73** | **1** |  | **VSPAGGTLDDKPK** |
|  | 3182 | 776.9294 | 1551.8443 | 1551.8549 | -6.79 | 0 | 12 | 0.54 | 1 |  | FLQDIFTTLVDLK |
|  | 2002 | **619.8090** | **1237.6034** | **1237.5873** | **13.0** | **1** | **12** | **0.9** | **1** |  | **AGENAGMGRFTK** |
|  | 857 | **496.2425** | **990.4704** | **990.4804** | **-10.14** | **0** | **12** | **0.92** | **1** |  | **GEASLEVMR** |
|  | 365 | **424.2408** | **846.4671** | **846.4811** | **-16.55** | **0** | **12** | **0.99** | **1** |  | **SAVASSVVK** |
|  | 855 | **495.7668** | **989.5191** | **989.5215** | **-2.42** | **0** | **12** | **1.3** | **1** |  | **AVLAAMDATK** |
|  | 2762 | **712.3698** | **1422.7251** | **1422.7177** | **5.21** | **1** | **12** | **0.88** | **1** |  | **NNFAAEMEKLIK + Oxidation (M)** |
|  | 3744 | **905.4161** | **1808.8177** | **1808.8332** | **-8.53** | **1** | **12** | **0.55** | **1** |  | **MDAMLLRQCPTQGTR + 2 Oxidation (M)** |
|  | 2966 | **493.5793** | **1477.7159** | **1477.6871** | **19.5** | **0** | **12** | **0.91** | **1** |  | **SMLEEVISNWDR** |
|  | 307 | **421.7582** | **841.5017** | **841.5022** | **-0.48** | **0** | **12** | **0.63** | **1** |  | **AVSVTPIR** |
|  | 1132 | **525.7771** | **1049.5396** | **1049.5506** | **-10.39** | **0** | **12** | **0.95** | **1** |  | **TVINYDVAR** |
|  | 874 | 498.2602 | 994.5059 | 994.5117 | -5.80 | 1 | 12 | 0.9 | 1 |  | KTMELTTR + Oxidation (M) |
|  | 3718 | **897.9139** | **1793.8133** | **1793.8028** | **5.88** | **0** | **12** | **0.51** | **1** |  | **TGTTGQSGAESGTTEPSAR** |
|  | 302 | **421.7581** | **841.5017** | **841.5022** | **-0.55** | **0** | **12** | **0.64** | **1** |  | **AVSVTPIR** |
|  | 2455 | **676.3331** | **1350.6516** | **1350.6680** | **-12.18** | **0** | **12** | **0.91** | **1** |  | **QVNNFLTSSWR** |
|  | 4324 | **737.7061** | **2210.0963** | **2210.1259** | **-13.40** | **1** | **12** | **0.72** | **1** |  | **QLQPSVVWIEDTEKTFYK** |
|  | 1343 | **547.7780** | **1093.5413** | **1093.5511** | **-8.96** | **0** | **12** | **0.95** | **1** |  | **VLTAVEMGMK + Oxidation (M)** |
|  | 299 | **421.7581** | **841.5016** | **841.5022** | **-0.62** | **0** | **12** | **0.64** | **1** |  | **AVSVTPIR** |
|  | 450 | **435.7746** | **869.5347** | **869.5487** | **-16.10** | **1** | **12** | **0.45** | **1** |  | **VVKFLHK** |
|  | 2692 | **703.8137** | **1405.6129** | **1405.6118** | **0.75** | **0** | **12** | **0.31** | **1** |  | **DLAAEPGNMWMR + Oxidation (M)** |
|  | 4224 | **700.0107** | **2097.0102** | **2097.0491** | **-18.52** | **0** | **12** | **0.69** | **1** |  | **ATYIQTIEEGINTHTHAAK** |
|  | 2315 | **438.9297** | **1313.7672** | **1313.7554** | **8.91** | **0** | **12** | **0.35** | **1** |  | **EAGLLAAVTLTQK** |
|  | 2651 | **697.8084** | **1393.6023** | **1393.6006** | **1.19** | **1** | **12** | **0.34** | **1** |  | **MKDTHMEDFPK + Oxidation (M)** |
|  | 3907 | **627.6453** | **1879.9140** | **1879.9019** | **6.39** | **1** | **12** | **0.89** | **1** |  | **TKMQTEEVCDASAIVAK** |
|  | 1488 | **563.2741** | **1124.5337** | **1124.5206** | **11.6** | **0** | **12** | **0.77** | **1** |  | **MSQEICTLK + Oxidation (M)** |
|  | 3547 | **867.9092** | **1733.8038** | **1733.7791** | **14.2** | **1** | **12** | **0.58** | **1** |  | **HSMREEDFIQPSSR + Oxidation (M)** |
|  | 804 | **489.2475** | **976.4805** | **976.4872** | **-6.88** | **1** | **12** | **1.2** | **1** |  | **VASMNQRR + Oxidation (M)** |
|  | 2218 | **647.8018** | **1293.5890** | **1293.5772** | **9.10** | **0** | **12** | **0.65** | **1** |  | **CSQNFGPEVTR** |
|  | 508 | **443.2613** | **884.5080** | **884.5080** | **0.02** | **0** | **12** | **0.71** | **1** |  | **AALSATVPR** |
|  | 2565 | **689.8556** | **1377.6966** | **1377.6922** | **3.23** | **1** | **12** | **0.99** | **1** |  | **ISGLKTSMAEGER** |
|  | 342 | **421.7587** | **841.5028** | **841.5022** | **0.83** | **0** | **12** | **0.66** | **1** |  | **AVSVTPIR** |
|  | 336 | **421.7584** | **841.5022** | **841.5022** | **0.09** | **0** | **12** | **0.66** | **1** |  | **AVSVTPIR** |
|  | 627 | **465.2744** | **928.5341** | **928.5202** | **15.0** | **1** | **12** | **0.92** | **1** |  | **QVSRAAAAR** |
|  | 3549 | **579.2875** | **1734.8406** | **1734.8134** | **15.7** | **1** | **12** | **0.85** | **1** |  | **KGDSSAEMSVYASLFK + Oxidation (M)** |
|  | 213 | **410.7158** | **819.4171** | **819.4273** | **-12.41** | **0** | **12** | **1.4** | **1** |  | **VATNIMR + Oxidation (M)** |
|  | 330 | **421.7583** | **841.5020** | **841.5022** | **-0.19** | **0** | **12** | **0.66** | **1** |  | **AVSVTPIR** |
|  | 3344 | 546.2534 | 1635.7383 | 1635.7675 | -17.89 | 1 | 12 | 0.53 | 1 |  | GVTSFGLENKCGDPR |
|  | 322 | **421.7582** | **841.5019** | **841.5022** | **-0.34** | **0** | **12** | **0.67** | **1** |  | **AVSVTPIR** |
|  | 4357 | **746.3782** | **2236.1129** | **2236.1014** | **5.13** | **1** | **12** | **0.74** | **1** |  | **AIVNPMDMQTSGALLRTCVK + 2 Oxidation (M)** |
|  | 4247 | **707.3231** | **2118.9475** | **2118.9892** | **-19.66** | **0** | **12** | **0.37** | **1** |  | **SPVHMGSPYTDVPSLSTSNK + Oxidation (M)** |
|  | 2530 | **456.2892** | **1365.8456** | **1365.8344** | **8.25** | **0** | **12** | **0.066** | **1** |  | **AQLGLGEIILAIR** |
|  | 1776 | **396.1991** | **1185.5755** | **1185.5890** | **-11.44** | **1** | **12** | **0.9** | **1** |  | **EAEFRHLER** |
|  | 2698 | **704.3853** | **1406.7561** | **1406.7454** | **7.57** | **1** | **12** | **0.68** | **1** |  | **KMIIWFPDMVK** |
|  | 2569 | **690.7996** | **1379.5847** | **1379.5672** | **12.7** | **0** | **12** | **0.22** | **1** |  | **CAYCVMGFTQK + Oxidation (M)** |
|  | 3642 | **883.8953** | **1765.7760** | **1765.7723** | **2.06** | **1** | **12** | **0.35** | **1** |  | **DAVQNCCGISKTEER** |
|  | 4049 | **652.9621** | **1955.8645** | **1955.8782** | **-7.03** | **0** | **12** | **0.29** | **1** |  | **SIMEDSTILSDWTNSNK + Oxidation (M)** |
|  | 3914 | **941.9827** | **1881.9509** | **1881.9506** | **0.16** | **0** | **12** | **0.75** | **1** |  | **STVGLSLISPNNMSFATK + Oxidation (M)** |
|  | 3538 | **864.3808** | **1726.7470** | **1726.7596** | **-7.26** | **1** | **12** | **0.26** | **1** |  | **KYDSTGMFHWCAPK** |
|  | 3460 | **424.4570** | **1693.7990** | **1693.7981** | **0.52** | **0** | **12** | **0.79** | **1** |  | **TSYHTPGDMVSITAAK + Oxidation (M)** |
|  | 2350 | **661.8327** | **1321.6508** | **1321.6588** | **-5.98** | **0** | **12** | **0.96** | **1** |  | **EMTQAPYLEIK** |
|  | 277 | **421.7572** | **841.4999** | **841.5022** | **-2.67** | **0** | **12** | **0.72** | **1** |  | **AVSVTPIR** |
|  | 3640 | **883.8952** | **1765.7758** | **1765.7723** | **2.00** | **1** | **12** | **0.36** | **1** |  | **DAVQNCCGISKTEER** |
|  | 2630 | **696.3334** | **1390.6522** | **1390.6398** | **8.90** | **1** | **12** | **0.86** | **1** |  | **AMAEDLGDQDKAK** |
|  | 1027 | **517.2834** | **1032.5522** | **1032.5617** | **-9.19** | **1** | **12** | **1.1** | **1** |  | **HFLSAFRR** |
|  | 290 | **421.7580** | **841.5015** | **841.5022** | **-0.76** | **0** | **12** | **0.71** | **1** |  | **AVSVTPIR** |
|  | 2447 | **674.3444** | **1346.6742** | **1346.6751** | **-0.72** | **0** | **12** | **1.1** | **1** |  | **IMELDSNDGLLK** |
|  | 3389 | **552.9377** | **1655.7914** | **1655.8123** | **-12.66** | **1** | **12** | **0.88** | **1** |  | **QLARGGTMVTYGGMAK + Oxidation (M)** |
|  | 332 | **421.7583** | **841.5020** | **841.5022** | **-0.19** | **0** | **12** | **0.71** | **1** |  | **AVSVTPIR** |
|  | 596 | **458.7881** | **915.5617** | **915.5575** | **4.60** | **0** | **12** | **0.25** | **1** |  | **VLLTMIAR** |
|  | 4499 | **825.0690** | **2472.1851** | **2472.2034** | **-7.40** | **1** | **12** | **0.55** | **1** |  | **GLPGLAGDHGEFGEKGDPGIPGNPGK** |
|  | 191 | **406.7459** | **811.4772** | **811.4664** | **13.3** | **1** | **12** | **0.44** | **1** |  | **ALAPERR** |
|  | 345 | **421.7595** | **841.5044** | **841.5021** | **2.72** | **1** | **12** | **0.71** | **1** |  | **VAGAATPKK** |
|  | 1082 | **523.2852** | **1044.5559** | **1044.5716** | **-15.05** | **0** | **12** | **1.5** | **1** |  | **LSSAHVYLR** |
|  | 287 | **421.7580** | **841.5014** | **841.5022** | **-0.84** | **0** | **12** | **0.72** | **1** |  | **AVSVTPIR** |
|  | 300 | **421.7581** | **841.5016** | **841.5022** | **-0.62** | **0** | **11** | **0.72** | **1** |  | **AVSVTPIR** |
|  | 2536 | **456.5956** | **1366.7649** | **1366.7568** | **5.91** | **1** | **11** | **0.44** | **1** |  | **QPPERIASGAITK** |
|  | 542 | **450.2694** | **898.5242** | **898.5097** | **16.2** | **1** | **11** | **0.74** | **1** |  | **AQGAQRLR** |
|  | 1561 | **570.7824** | **1139.5503** | **1139.5533** | **-2.62** | **0** | **11** | **1** | **1** |  | **FPLTTESAMK + Oxidation (M)** |
|  | 1340 | **547.3144** | **1092.6142** | **1092.6152** | **-0.89** | **1** | **11** | **0.6** | **1** |  | **APTLATAHRR** |
|  | 285 | **421.7579** | **841.5012** | **841.5021** | **-1.04** | **1** | **11** | **0.73** | **1** |  | **ELKSIPR** |
|  | 359 | 423.7175 | 845.4205 | 845.4131 | 8.82 | 0 | 11 | 1.3 | 1 |  | DQLLDDK |
|  | 534 | **449.7770** | **897.5393** | **897.5396** | **-0.26** | **1** | **11** | **0.43** | **1** |  | **AKITNVPR** |
|  | 3000 | **746.3994** | **1490.7843** | **1490.7816** | **1.78** | **1** | **11** | **1** | **1** |  | **MAAPILRSFSWGR** |
|  | 3110 | **511.5986** | **1531.7740** | **1531.7565** | **11.4** | **1** | **11** | **1.2** | **1** |  | **GAQLCFEANAKAPR** |
|  | 1557 | **570.2974** | **1138.5802** | **1138.6022** | **-19.39** | **0** | **11** | **0.95** | **1** |  | **EFSFLDILR** |
|  | 4338 | **1106.5491** | **2211.0836** | **2211.0949** | **-5.11** | **1** | **11** | **0.83** | **1** |  | **LTMELTGDSMEVKPIMTRK + 2 Oxidation (M)** |
|  | 326 | **421.7582** | **841.5019** | **841.5022** | **-0.34** | **0** | **11** | **0.74** | **1** |  | **AVSVTPIR** |
|  | 509 | **443.2617** | **884.5088** | **884.5080** | **0.92** | **0** | **11** | **0.79** | **1** |  | **AALSATVPR** |
|  | 597 | **458.7883** | **915.5620** | **915.5575** | **4.92** | **0** | **11** | **0.26** | **1** |  | **VLLTMIAR** |
|  | 591 | **458.7642** | **915.5138** | **915.5025** | **12.3** | **0** | **11** | **1.5** | **1** |  | **LVLTEGER** |
|  | 2199 | **644.3513** | **1286.6880** | **1286.7095** | **-16.75** | **1** | **11** | **1.2** | **1** |  | **DLRFQPVSIGR** |
|  | 1827 | **598.8580** | **1195.7015** | **1195.7077** | **-5.18** | **1** | **11** | **0.35** | **1** |  | **IKFPNSLHLK** |
|  | 1774 | 395.9029 | 1184.6868 | 1184.6666 | 17.1 | 1 | 11 | 0.71 | 1 |  | LGSLFVKHER |
|  | 325 | **421.7582** | **841.5019** | **841.5022** | **-0.34** | **0** | **11** | **0.76** | **1** |  | **AVSVTPIR** |
|  | 470 | **438.2514** | **874.4882** | **874.4872** | **1.10** | **0** | **11** | **1.1** | **1** |  | **ITVNSVSR** |
|  | 2114 | **633.8138** | **1265.6131** | **1265.5995** | **10.7** | **0** | **11** | **1.1** | **1** |  | **NTMALMDLDVK + Oxidation (M)** |
|  | 1952 | **614.2787** | **1226.5429** | **1226.5390** | **3.22** | **0** | **11** | **0.35** | **1** |  | **EVCGFAPYER** |
|  | 3859 | **621.9705** | **1862.8896** | **1862.8581** | **16.9** | **0** | **11** | **0.85** | **1** |  | **GEAGPAGPTGPAGECSVPPR** |
|  | 2219 | **647.8166** | **1293.6187** | **1293.6169** | **1.41** | **1** | **11** | **1.1** | **1** |  | **VVAAMNRMDQK + 2 Oxidation (M)** |
|  | 254 | **416.1827** | **830.3507** | **830.3559** | **-6.18** | **0** | **11** | **0.24** | **1** |  | **DSYGSFR** |
|  | 274 | **421.2421** | **840.4697** | **840.4817** | **-14.31** | **0** | **11** | **0.78** | **1** |  | **AVQALPSR** |
|  | 2636 | 696.8470 | 1391.6794 | 1391.7045 | -18.01 | 0 | 11 | 1.1 | 1 |  | QLSESEYVGKPR |
|  | 1022 | **516.7981** | **1031.5816** | **1031.5737** | **7.72** | **1** | **11** | **1.2** | **1** |  | **RSLVHHQR** |
|  | 1095 | **523.2856** | **1044.5566** | **1044.5386** | **17.2** | **0** | **11** | **1.5** | **1** |  | **LGVNDCVLR** |
|  | 2635 | **696.3805** | **1390.7464** | **1390.7456** | **0.59** | **1** | **11** | **0.94** | **1** |  | **EGLYLSDTLPRK** |
|  | 3096 | 763.8792 | 1525.7439 | 1525.7420 | 1.25 | 1 | 11 | 0.91 | 1 |  | QPGALTQGHSCSRK |
|  | 3123 | **768.8327** | **1535.6508** | **1535.6675** | **-10.82** | **0** | **11** | **0.26** | **1** |  | **EGGPAFEAGLCTGDR** |
|  | 269 | **418.7242** | **835.4337** | **835.4300** | **4.46** | **1** | **11** | **1.5** | **1** |  | **YRNEVR** |
|  | 391 | **428.7668** | **855.5189** | **855.5290** | **-11.76** | **1** | **11** | **0.79** | **1** |  | **RDILALR** |
|  | 730 | **478.7808** | **955.5471** | **955.5451** | **2.19** | **1** | **11** | **0.87** | **1** |  | **AKVSSLSHK** |
|  | 1381 | **552.2880** | **1102.5614** | **1102.5618** | **-0.42** | **1** | **11** | **1.6** | **1** |  | **EKSQDEVLR** |
|  | 1102 | **523.2858** | **1044.5570** | **1044.5716** | **-14.00** | **0** | **11** | **1.5** | **1** |  | **LSSAHVYLR** |
|  | 2795 | **714.8710** | **1427.7274** | **1427.7481** | **-14.49** | **1** | **11** | **1.1** | **1** |  | **LSGAAARGDVQEVR** |
|  | 827 | **492.7949** | **983.5753** | **983.5625** | **13.1** | **1** | **11** | **0.71** | **1** |  | **QIAGRIGGGR** |
|  | 350 | **423.2263** | **844.4381** | **844.4515** | **-15.86** | **1** | **11** | **1.7** | **1** |  | **AIRENSR** |
|  | 314 | **421.7582** | **841.5018** | **841.5021** | **-0.37** | **1** | **11** | **0.81** | **1** |  | **ELKSIPR** |
|  | 681 | **471.2273** | **940.4401** | **940.4362** | **4.12** | **1** | **11** | **0.7** | **1** |  | **EQKHEDR** |
|  | 3132 | **769.3461** | **1536.6777** | **1536.6699** | **5.07** | **1** | **11** | **0.49** | **1** |  | **SDGSACNSGISGGRGR** |
|  | 327 | **421.7582** | **841.5019** | **841.5022** | **-0.34** | **0** | **11** | **0.82** | **1** |  | **AVSVTPIR** |
|  | 1791 | 595.8053 | 1189.5960 | 1189.6051 | -7.61 | 1 | 11 | 1.5 | 1 |  | KVDTAAQTNSR |
|  | 1358 | **366.8415** | **1097.5026** | **1097.4884** | **13.0** | **1** | **11** | **0.72** | **1** |  | **TRSCSSASSR** |
|  | 339 | **421.7584** | **841.5023** | **841.5021** | **0.20** | **1** | **11** | **0.82** | **1** |  | **ELKSIPR** |
|  | 1107 | **523.2859** | **1044.5573** | **1044.5716** | **-13.65** | **0** | **11** | **1.6** | **1** |  | **LSSAHVYLR** |
|  | 3224 | **394.2046** | **1572.7893** | **1572.7896** | **-0.23** | **1** | **11** | **1.3** | **1** |  | **LSEGFSIHTRDSPK** |
|  | 2729 | **473.2345** | **1416.6817** | **1416.6732** | **5.96** | **1** | **11** | **1.1** | **1** |  | **ITRDDDPESEIK** |
|  | 471 | **438.2563** | **874.4980** | **874.4872** | **12.4** | **0** | **11** | **1.2** | **1** |  | **TASIGTLGR** |
|  | 2791 | **357.9277** | **1427.6818** | **1427.6827** | **-0.63** | **0** | **11** | **0.97** | **1** |  | **QTGAAADMPQPAVR + Oxidation (M)** |
|  | 535 | 449.7770 | 897.5393 | 897.5396 | -0.26 | 1 | 11 | 0.5 | 1 |  | AKITNVPR |
|  | 1371 | **551.7949** | **1101.5752** | **1101.5778** | **-2.39** | **1** | **11** | **1.7** | **1** |  | **AADLKDLNSR** |
|  | 777 | **486.3109** | **970.6073** | **970.6063** | **1.08** | **0** | **11** | **0.37** | **1** |  | **GITVVELIK** |
|  | 1092 | **523.2855** | **1044.5565** | **1044.5386** | **17.1** | **0** | **11** | **1.6** | **1** |  | **LGVNDCVLR** |
|  | 2497 | **453.5720** | **1357.6943** | **1357.7201** | **-19.00** | **1** | **11** | **1.4** | **1** |  | **QIQEKTDIIDR** |
|  | 588 | **457.2770** | **912.5394** | **912.5392** | **0.18** | **1** | **11** | **0.94** | **1** |  | **KLTVPAER** |
|  | 2587 | 692.3835 | 1382.7524 | 1382.7446 | 5.68 | 0 | 11 | 0.83 | 1 |  | LPSGFDLIPPAEK |
|  | 3284 | **803.4456** | **1604.8767** | **1604.8774** | **-0.42** | **0** | **11** | **0.71** | **1** |  | **QLLQQLVTSYPSTK** |
|  | 4353 | **744.0740** | **2229.2001** | **2229.1754** | **11.1** | **1** | **11** | **0.53** | **1** |  | **TDLTDLQGAIVDVFSRAGPVR** |
|  | 640 | **467.2357** | **932.4568** | **932.4498** | **7.49** | **1** | **11** | **1.7** | **1** |  | **MADRTAPR + Oxidation (M)** |
|  | 3285 | **803.4460** | **1604.8774** | **1604.8774** | **0.03** | **0** | **11** | **0.7** | **1** |  | **QLLQQLVTSYPSTK** |
|  | 2313 | **438.9292** | **1313.7657** | **1313.7554** | **7.79** | **0** | **11** | **0.45** | **1** |  | **EAGLLAAVTLTQK** |
|  | 1385 | **552.7820** | **1103.5495** | **1103.5458** | **3.34** | **0** | **11** | **1.6** | **1** |  | **ENSGAIEASVK** |
|  | 1867 | **602.3356** | **1202.6567** | **1202.6441** | **10.5** | **1** | **11** | **1.3** | **1** |  | **SLKDINNMIR** |
|  | 2652 | **697.8619** | **1393.7092** | **1393.6945** | **10.6** | **1** | **11** | **1.3** | **1** |  | **NTMALMDLDVKK + Oxidation (M)** |
|  | 441 | **435.7740** | **869.5334** | **869.5447** | **-12.94** | **1** | **11** | **0.61** | **1** |  | **VVVERLR** |
|  | 1073 | **523.2844** | **1044.5543** | **1044.5716** | **-16.58** | **0** | **11** | **1.8** | **1** |  | **LSSAHVYLR** |
|  | 2334 | **659.8890** | **1317.7635** | **1317.7479** | **11.9** | **1** | **11** | **0.36** | **1** |  | **LITAVVMKNWK + Oxidation (M)** |
|  | 842 | **494.7655** | **987.5164** | **987.5236** | **-7.26** | **1** | **11** | **2.2** | **1** |  | **IAEEEAKAK** |
|  | 1293 | **543.3012** | **1084.5879** | **1084.5950** | **-6.62** | **1** | **11** | **1.2** | **1** |  | **EPCKTPILK** |
|  | 2449 | **674.3455** | **1346.6765** | **1346.6943** | **-13.20** | **1** | **11** | **1.5** | **1** |  | **QGSVPDYGQRLK** |
[truncated: 2,438,708 more chars]
