# Supplementary figures and images for: Downregulation of circLIFR exerts cancer-promoting effects on hepatocellular carcinoma in vitro (part 1 of 4)
Source: Front Genet. 2022 Sep 12;13:986322. doi: 10.3389/fgene.2022.986322 (PMC9513674; doi:10.3389/fgene.2022.986322)

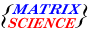

Supplement: Supplementary file 1 [file DataSheet3.ZIP › Original dataú¿1ú⌐/Mass spectrometric detection/Control group no.6/Peptide Summary Report (.._data_20191122_F024829.dat)_files/88x31_logo_white.gif]

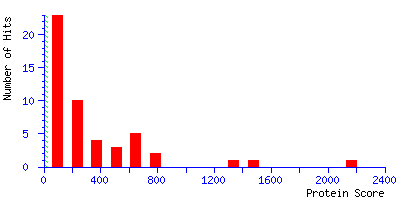

Supplement: Supplementary file 1 [file DataSheet3.ZIP › Original dataú¿1ú⌐/Mass spectrometric detection/Control group no.6/Peptide Summary Report (.._data_20191122_F024829.dat)_files/score_gif.pl.╧┬╘╪]

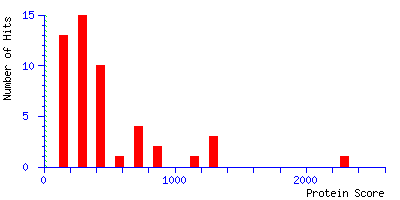

Supplement: Supplementary file 1 [file DataSheet3.ZIP › Original dataú¿1ú⌐/Mass spectrometric detection/Enrichment group no. 5/Peptide Summary Report (.._data_20191122_F024828.dat)_files/score_gif.pl.╧┬╘╪]

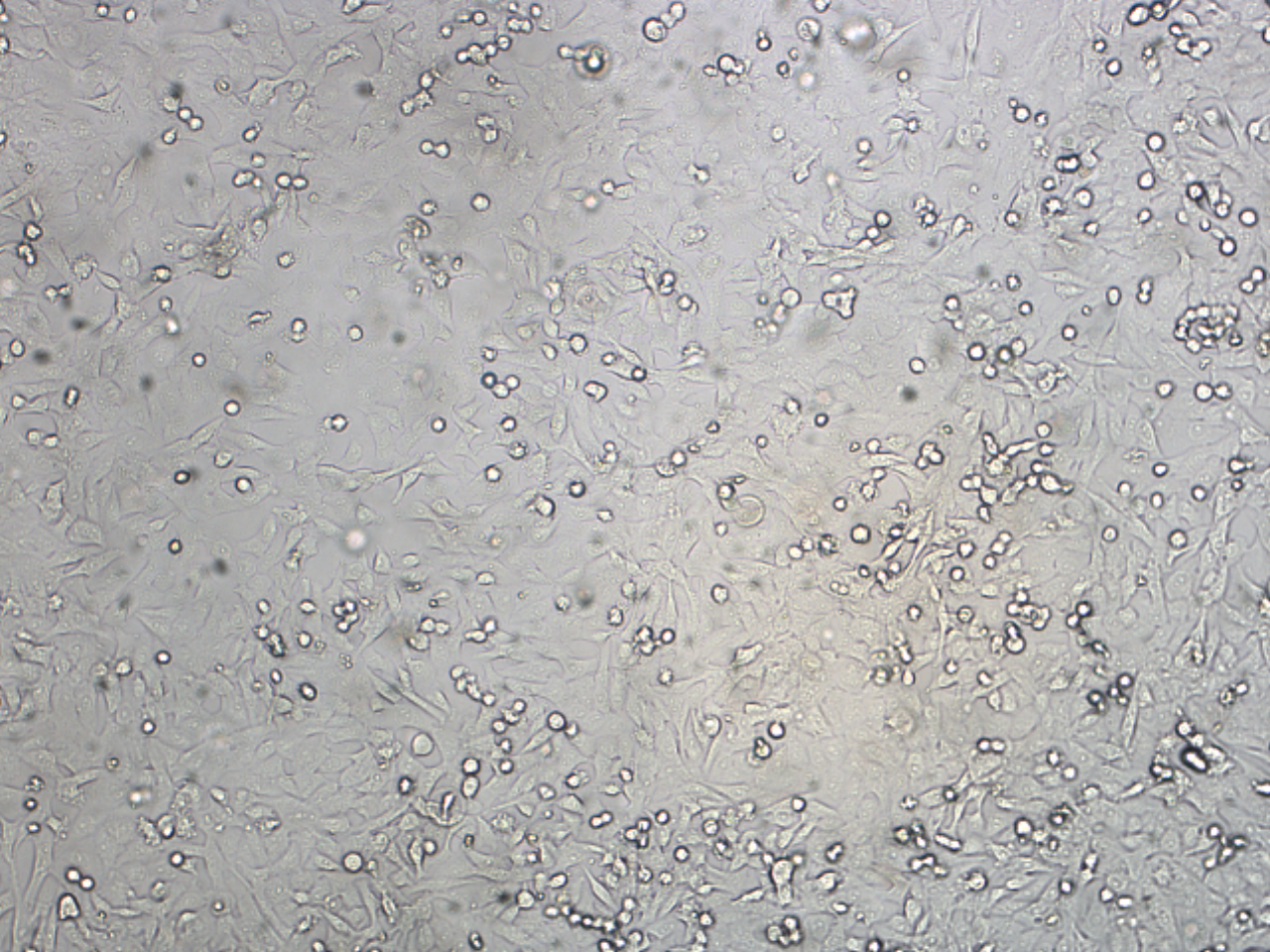

Supplement: Supplementary file 1 [file DataSheet3.ZIP › Original dataú¿1ú⌐/Pull down/Cell transients and capture protein induction/sk-hep-1-b2.tif]

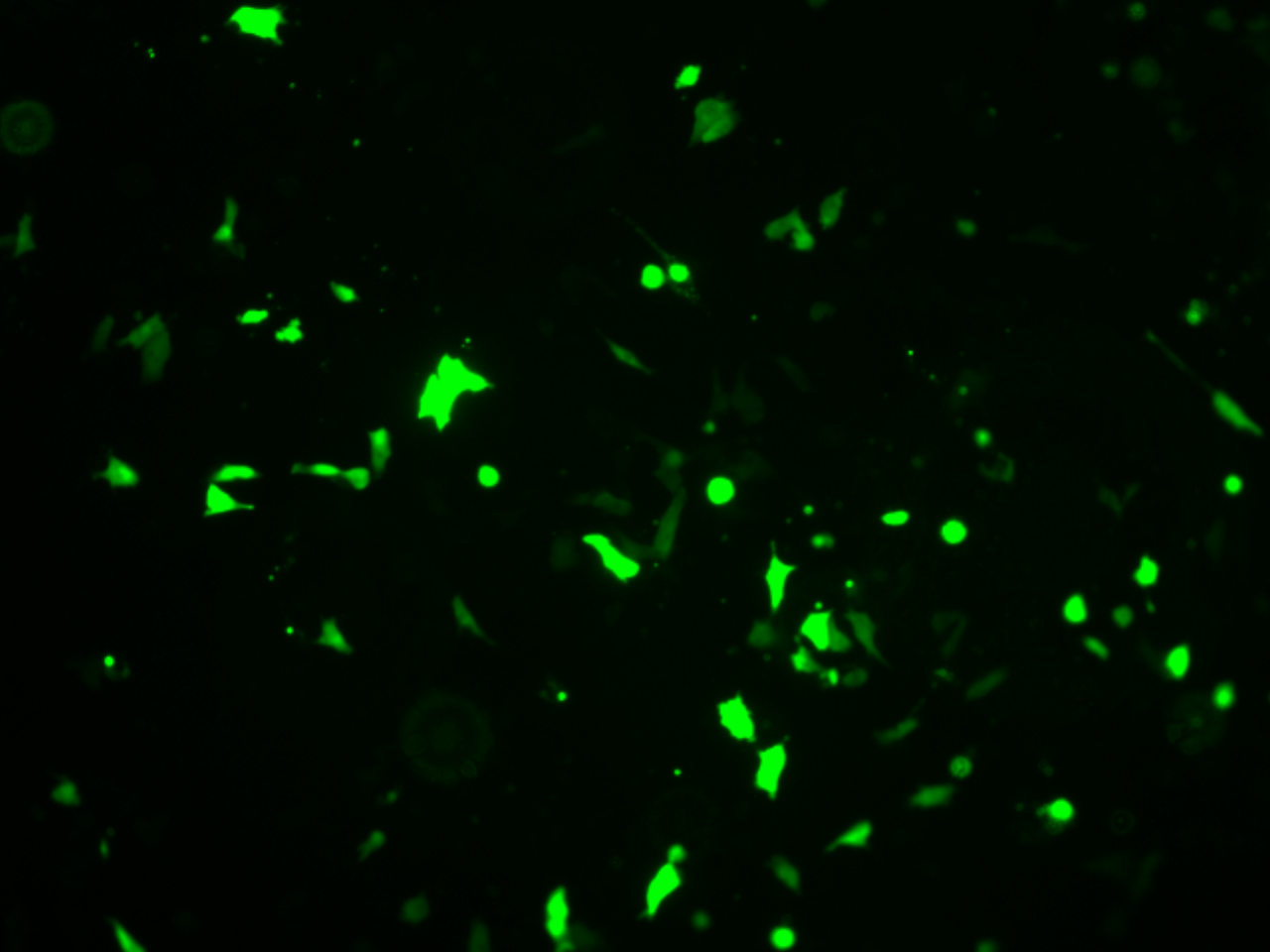

Supplement: Supplementary file 1 [file DataSheet3.ZIP › Original dataú¿1ú⌐/Pull down/Cell transients and capture protein induction/sk-hep-1-g2.tif]

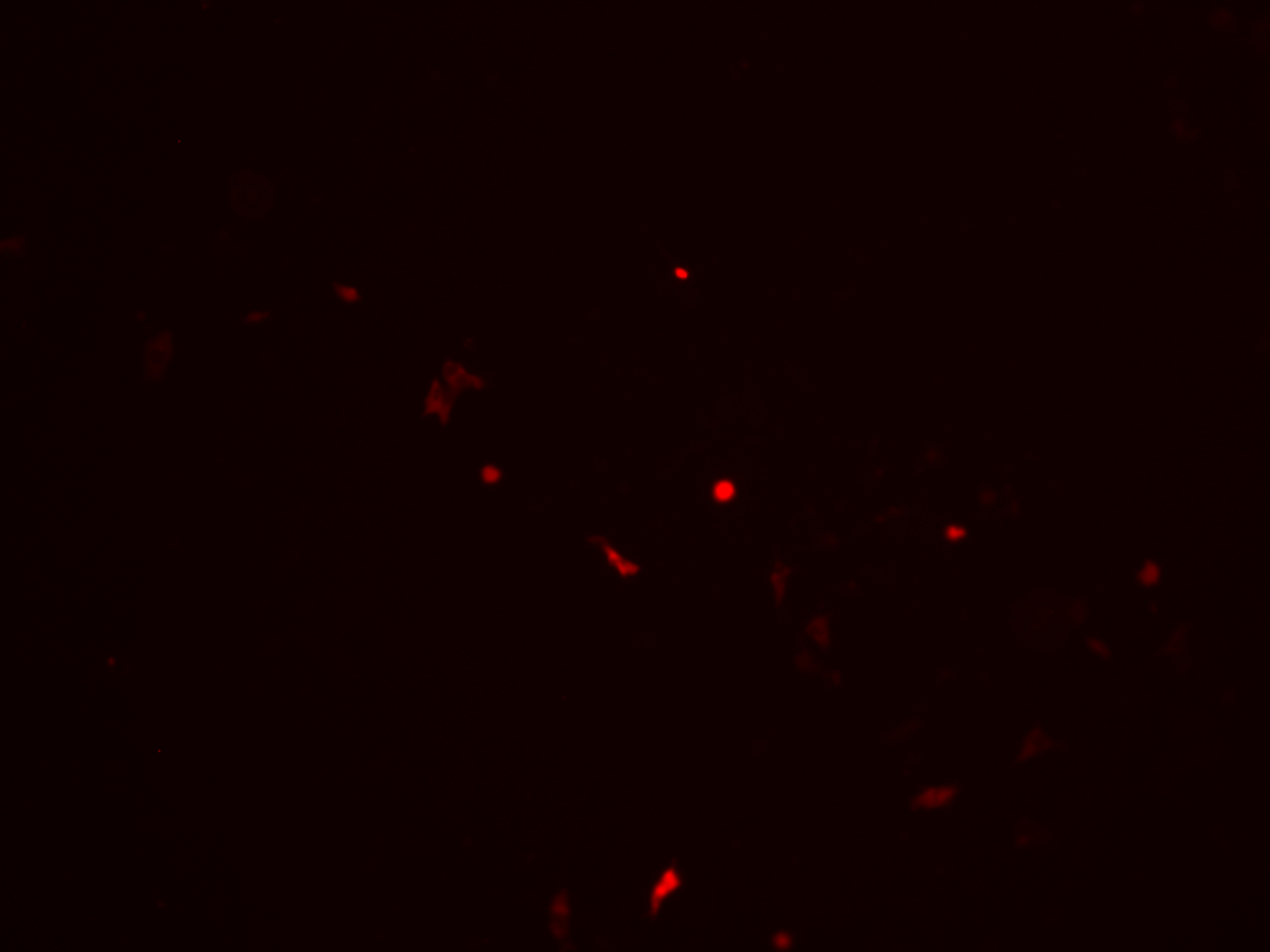

Supplement: Supplementary file 1 [file DataSheet3.ZIP › Original dataú¿1ú⌐/Pull down/Cell transients and capture protein induction/sk-hep-1-r2.tif]

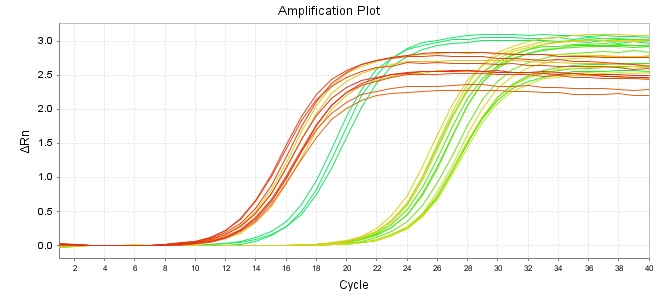

Supplement: Supplementary file 1 [file DataSheet3.ZIP › Original dataú¿1ú⌐/Pull down/Pull-down and enrichment detection/Dissolve curve and Amplification curve/Amplification Plot-GAPDH.jpg]

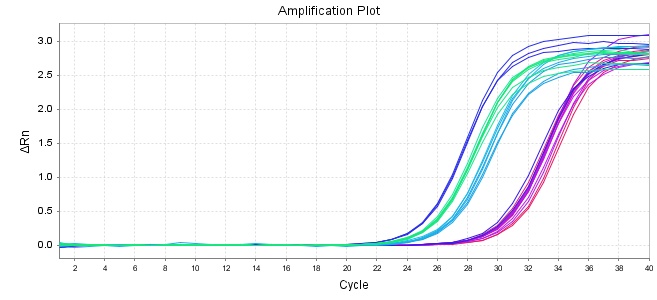

Supplement: Supplementary file 1 [file DataSheet3.ZIP › Original dataú¿1ú⌐/Pull down/Pull-down and enrichment detection/Dissolve curve and Amplification curve/Amplification Plot-hsa_circ_0072309.jpg]

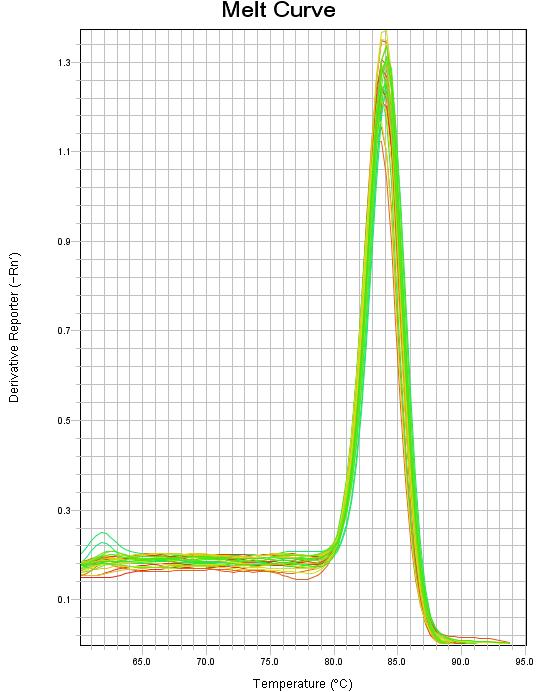

Supplement: Supplementary file 1 [file DataSheet3.ZIP › Original dataú¿1ú⌐/Pull down/Pull-down and enrichment detection/Dissolve curve and Amplification curve/Melt Curve-GAPDH.jpg]

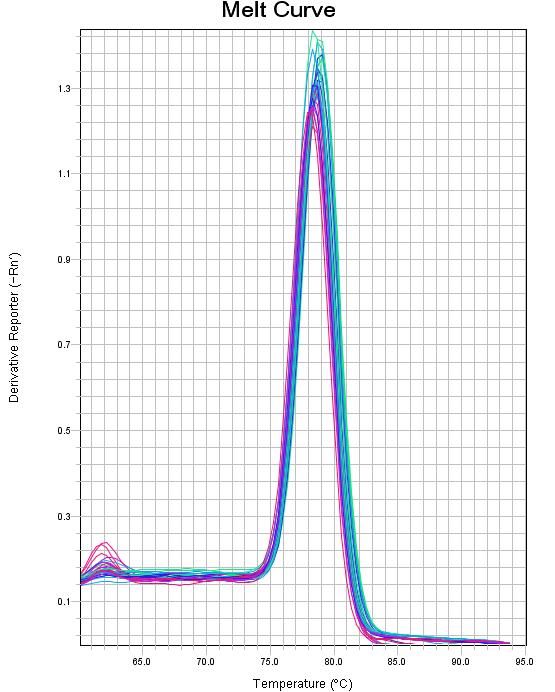

Supplement: Supplementary file 1 [file DataSheet3.ZIP › Original dataú¿1ú⌐/Pull down/Pull-down and enrichment detection/Dissolve curve and Amplification curve/Melt Curve-hsa_circ_0072309.jpg]

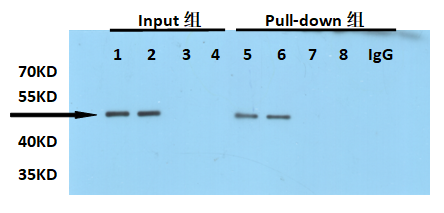

Supplement: Supplementary file 1 [file DataSheet3.ZIP › Original dataú¿1ú⌐/Pull down/Pull-down and enrichment detection/wb.tif]

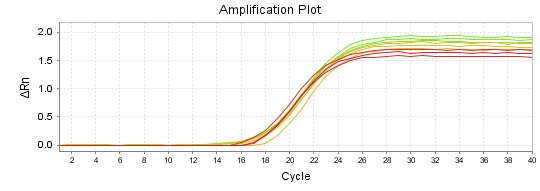

Supplement: Supplementary file 1 [file DataSheet3.ZIP › Original dataú¿1ú⌐/Pull down/Vector construction and validation/qPCR/Dissolve curve and Amplification curve/Amplification Plot-GAPDH(SK-Hep-1-0072309).jpg]

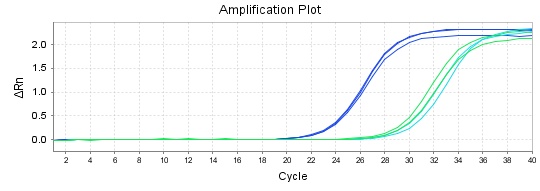

Supplement: Supplementary file 1 [file DataSheet3.ZIP › Original dataú¿1ú⌐/Pull down/Vector construction and validation/qPCR/Dissolve curve and Amplification curve/Amplification Plot-hsa_circ_0072309(SK-Hep-1).jpg]

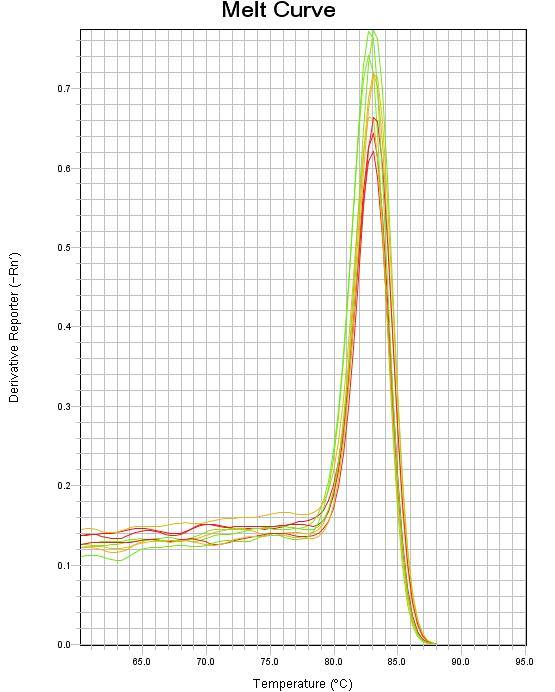

Supplement: Supplementary file 1 [file DataSheet3.ZIP › Original dataú¿1ú⌐/Pull down/Vector construction and validation/qPCR/Dissolve curve and Amplification curve/Melt Curve-GAPDH(SK-Hep-1-0072309).jpg]

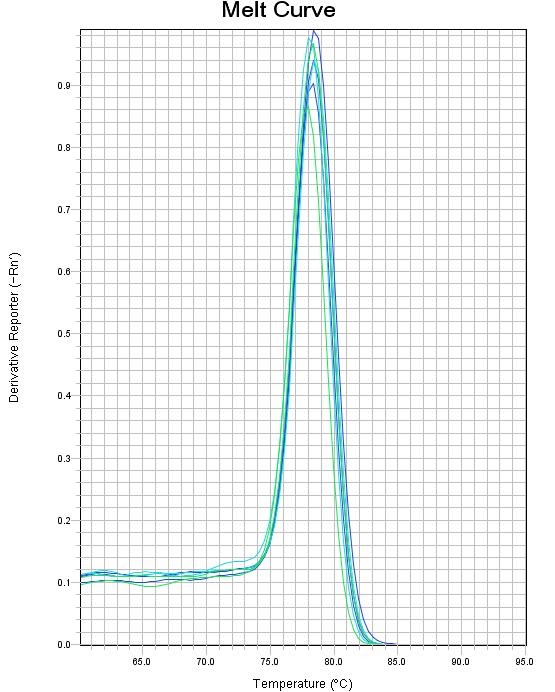

Supplement: Supplementary file 1 [file DataSheet3.ZIP › Original dataú¿1ú⌐/Pull down/Vector construction and validation/qPCR/Dissolve curve and Amplification curve/Melt Curve-hsa_circ_0072309(SK-Hep-1).jpg]

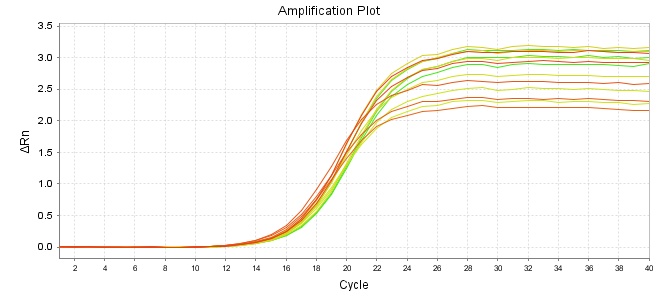

Supplement: Supplementary file 1 [file DataSheet3.ZIP › Original dataú¿1ú⌐/qPCR/Dissolve curve and Amplification curve/Amplification Plot-GAPDH.jpg]

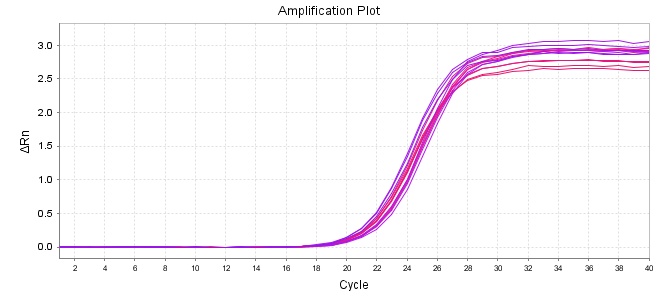

Supplement: Supplementary file 1 [file DataSheet3.ZIP › Original dataú¿1ú⌐/qPCR/Dissolve curve and Amplification curve/Amplification Plot-MAPK1.jpg]

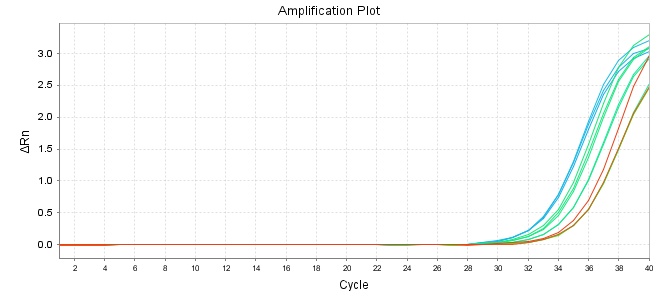

Supplement: Supplementary file 1 [file DataSheet3.ZIP › Original dataú¿1ú⌐/qPCR/Dissolve curve and Amplification curve/Amplification Plot-MMP13.jpg]

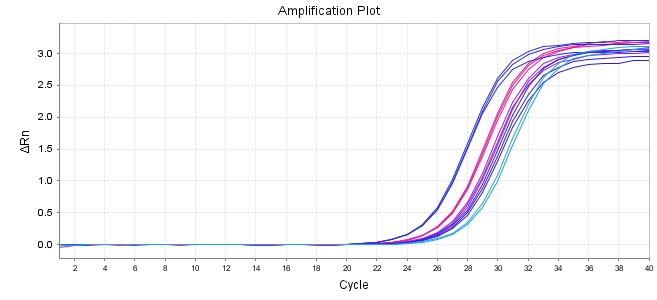

Supplement: Supplementary file 1 [file DataSheet3.ZIP › Original dataú¿1ú⌐/qPCR/Dissolve curve and Amplification curve/Amplification Plot-MMP3.jpg]

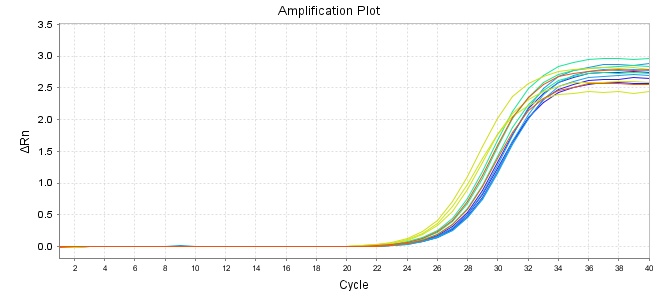

Supplement: Supplementary file 1 [file DataSheet3.ZIP › Original dataú¿1ú⌐/qPCR/Dissolve curve and Amplification curve/Amplification Plot-VEGF.jpg]

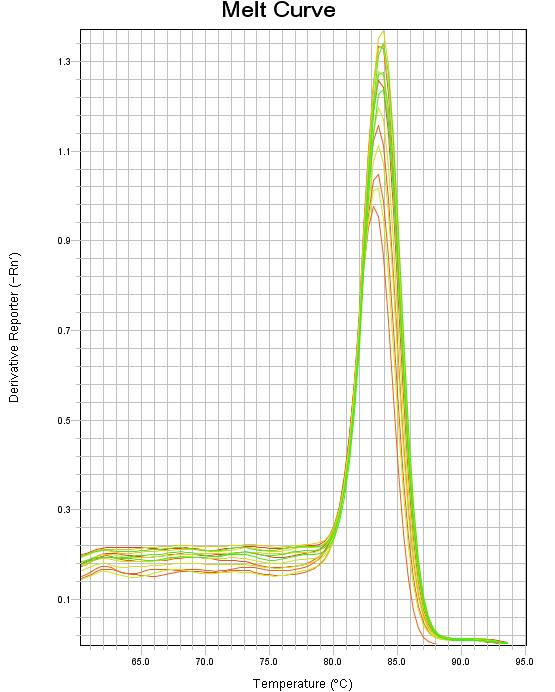

Supplement: Supplementary file 1 [file DataSheet3.ZIP › Original dataú¿1ú⌐/qPCR/Dissolve curve and Amplification curve/Melt Curve-GAPDH.jpg]

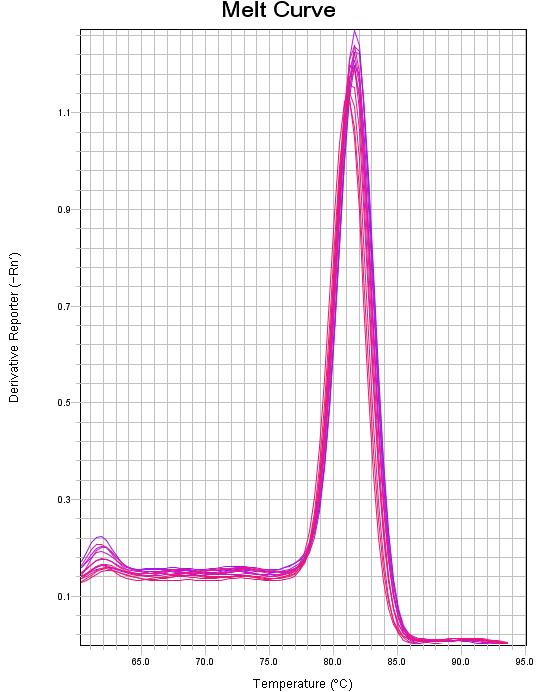

Supplement: Supplementary file 1 [file DataSheet3.ZIP › Original dataú¿1ú⌐/qPCR/Dissolve curve and Amplification curve/Melt Curve-MAPK1.jpg]

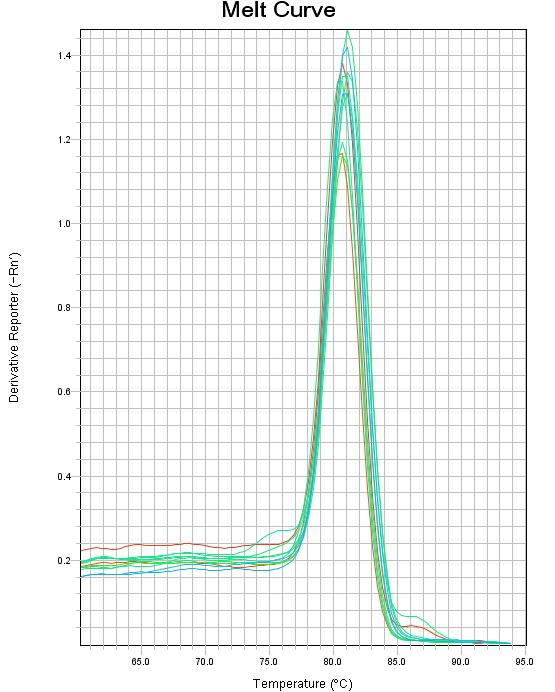

Supplement: Supplementary file 1 [file DataSheet3.ZIP › Original dataú¿1ú⌐/qPCR/Dissolve curve and Amplification curve/Melt Curve-MMP13.jpg]

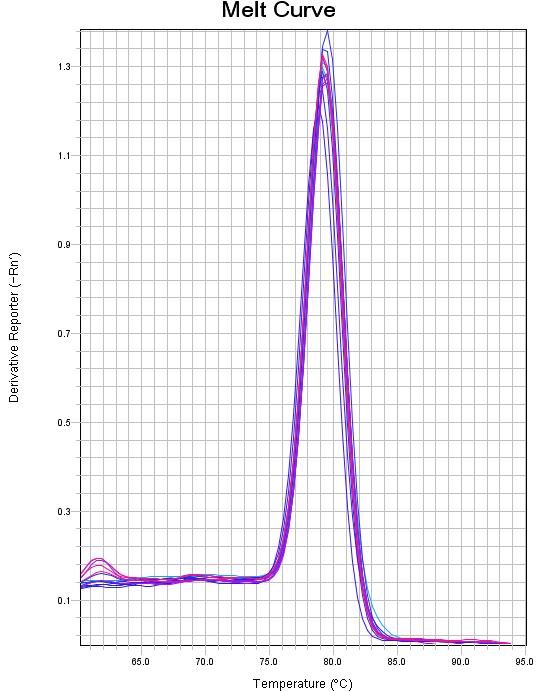

Supplement: Supplementary file 1 [file DataSheet3.ZIP › Original dataú¿1ú⌐/qPCR/Dissolve curve and Amplification curve/Melt Curve-MMP3.jpg]

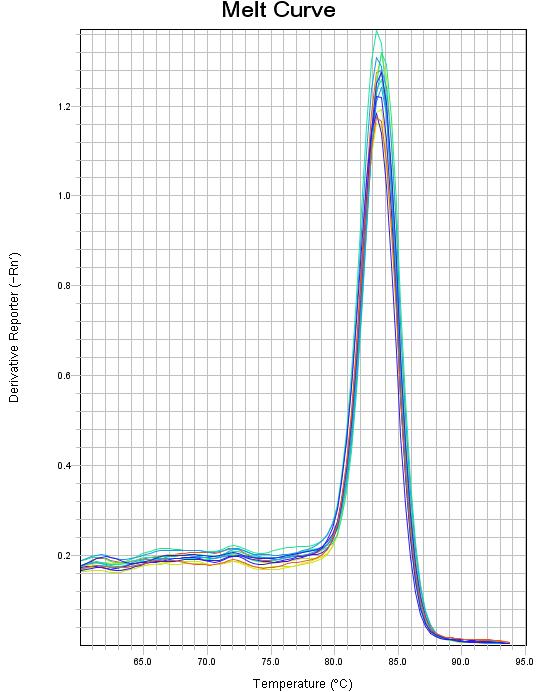

Supplement: Supplementary file 1 [file DataSheet3.ZIP › Original dataú¿1ú⌐/qPCR/Dissolve curve and Amplification curve/Melt Curve-VEGF.jpg]

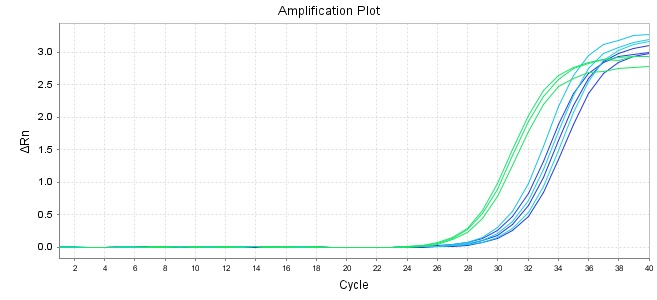

Supplement: Supplementary file 1 [file DataSheet3.ZIP › Original dataú¿1ú⌐/RIP/Dissolve curve and Amplification curve/Amplification Plot-hsa_circ_0072309.jpg]

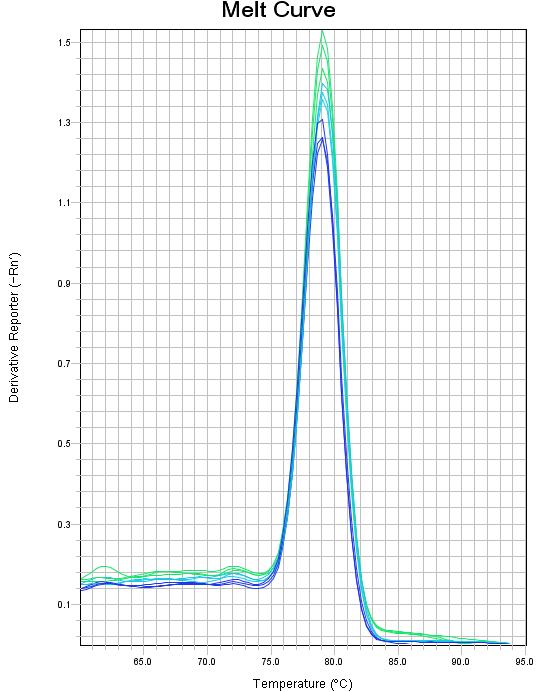

Supplement: Supplementary file 1 [file DataSheet3.ZIP › Original dataú¿1ú⌐/RIP/Dissolve curve and Amplification curve/Melt Curve-hsa_circ_0072309.jpg]

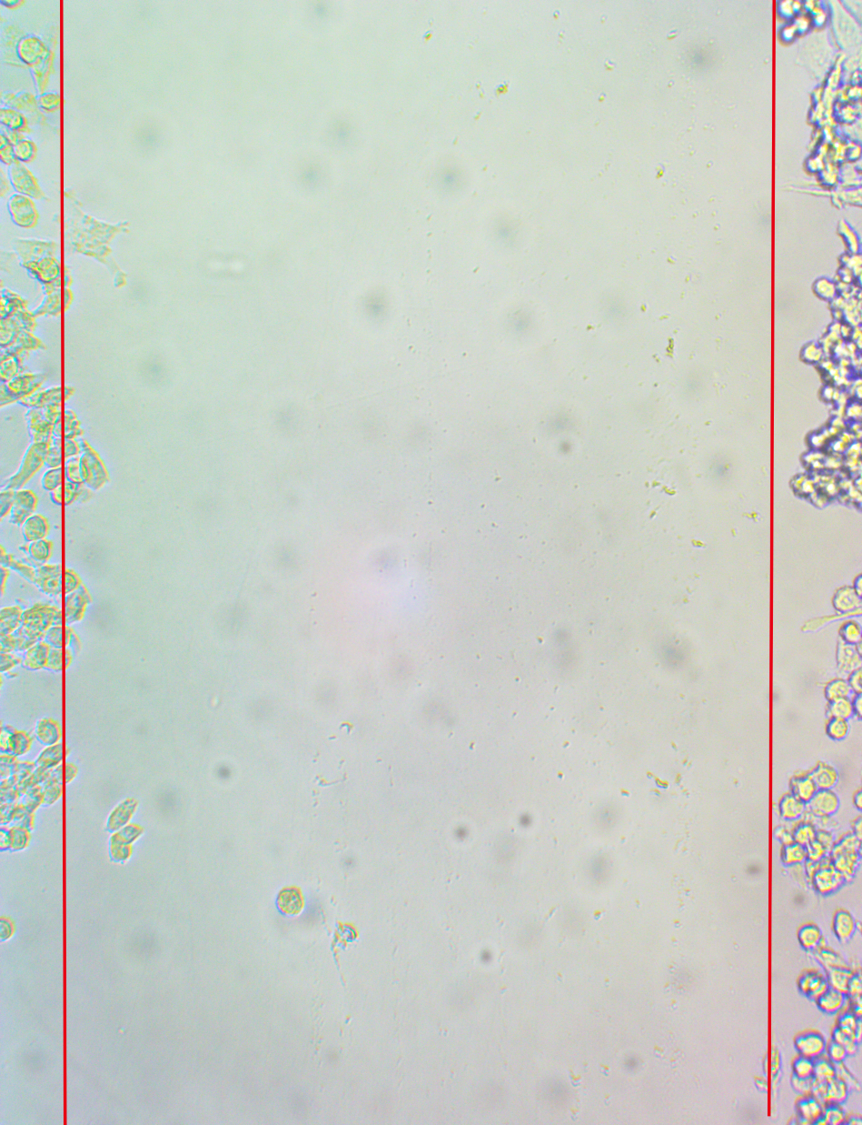

Supplement: Supplementary file 2 [file DataSheet11.ZIP › Fig 2-B-Wound-healing assay/C72309 0h.tif]

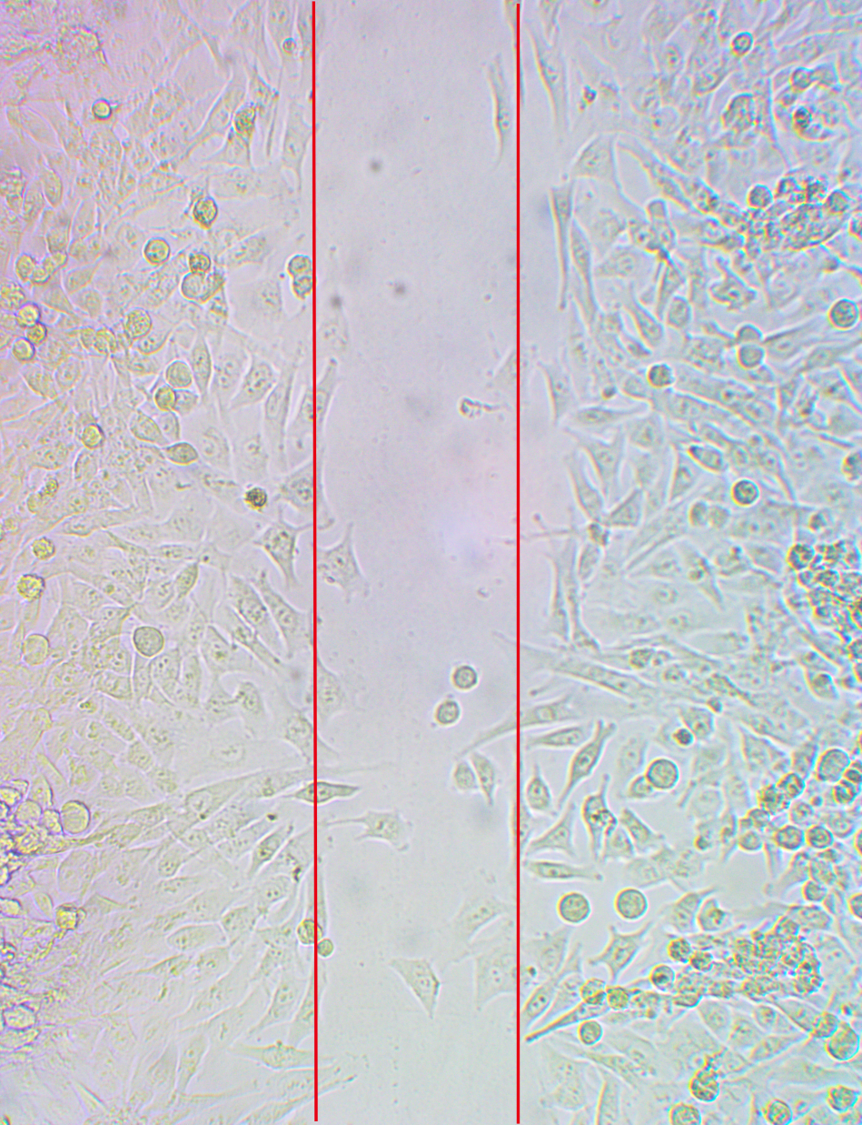

Supplement: Supplementary file 2 [file DataSheet11.ZIP › Fig 2-B-Wound-healing assay/C72309 24h.tif]

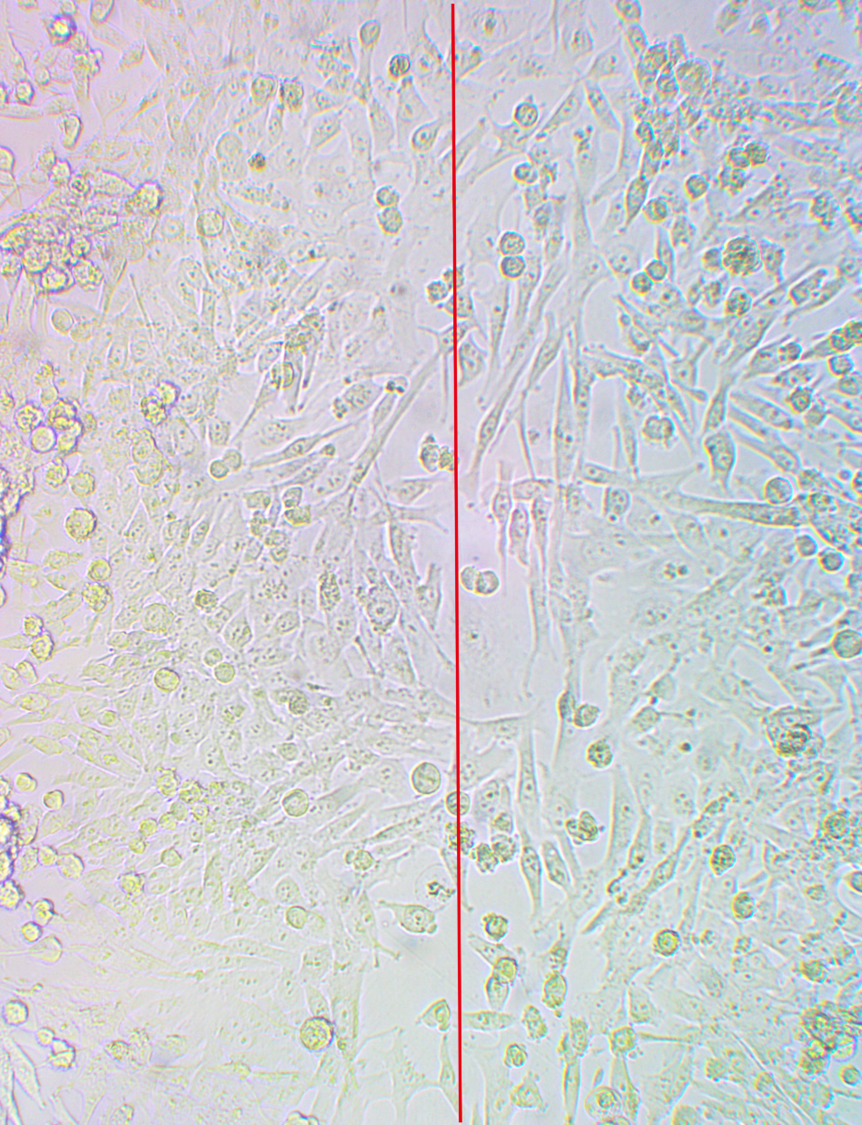

Supplement: Supplementary file 2 [file DataSheet11.ZIP › Fig 2-B-Wound-healing assay/C72309 48h.tif]

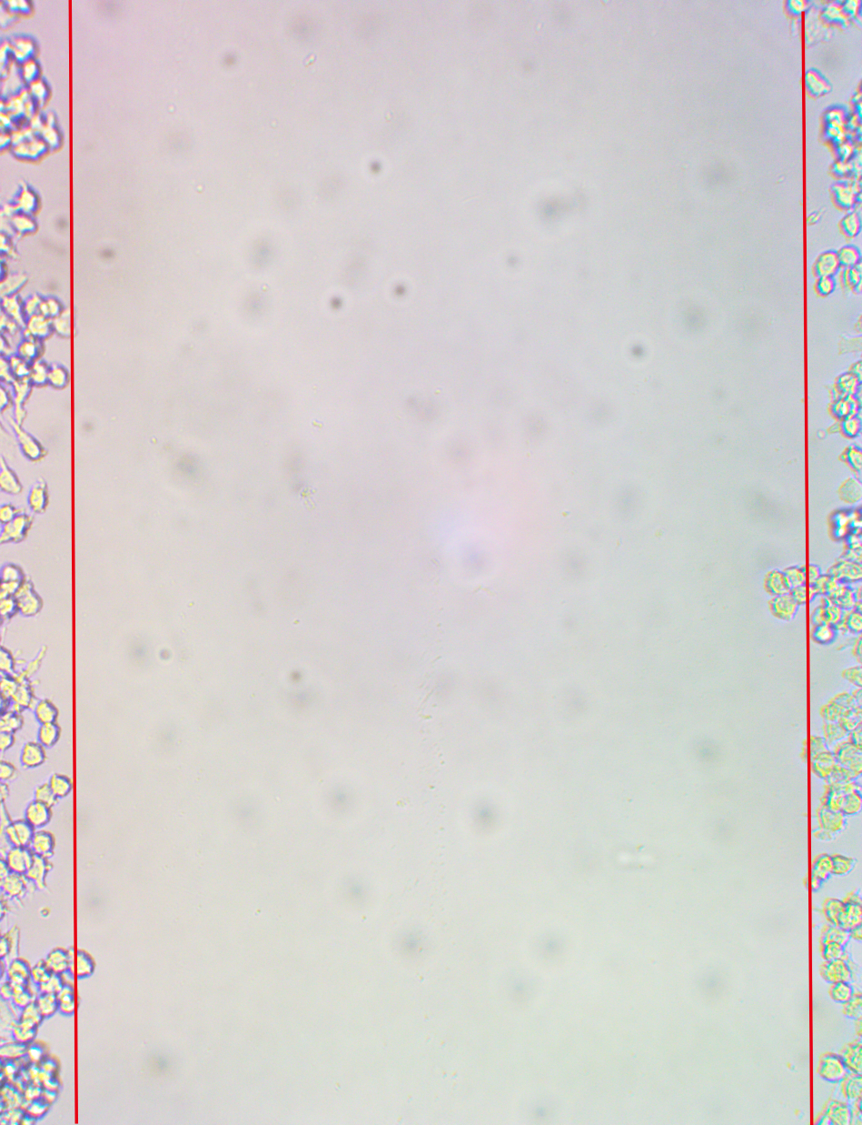

Supplement: Supplementary file 2 [file DataSheet11.ZIP › Fig 2-B-Wound-healing assay/control 0h.tif]

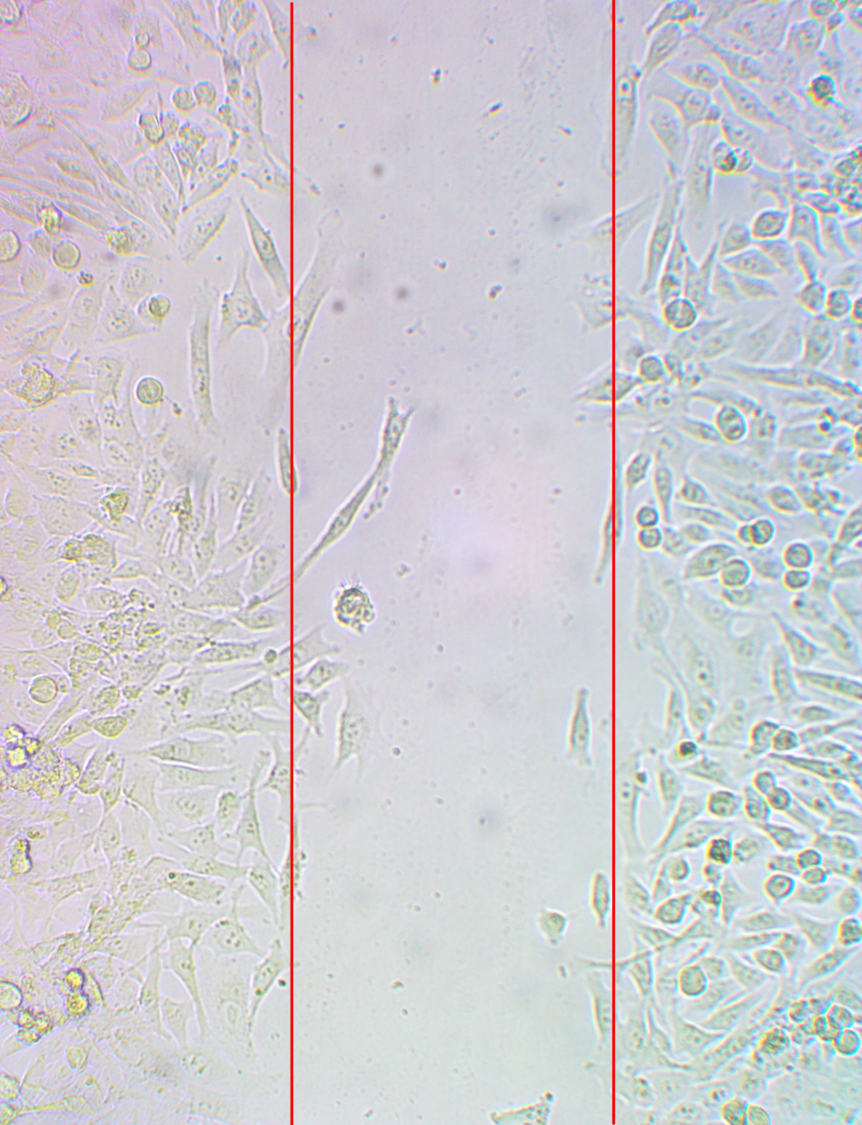

Supplement: Supplementary file 2 [file DataSheet11.ZIP › Fig 2-B-Wound-healing assay/control 24h.tif]

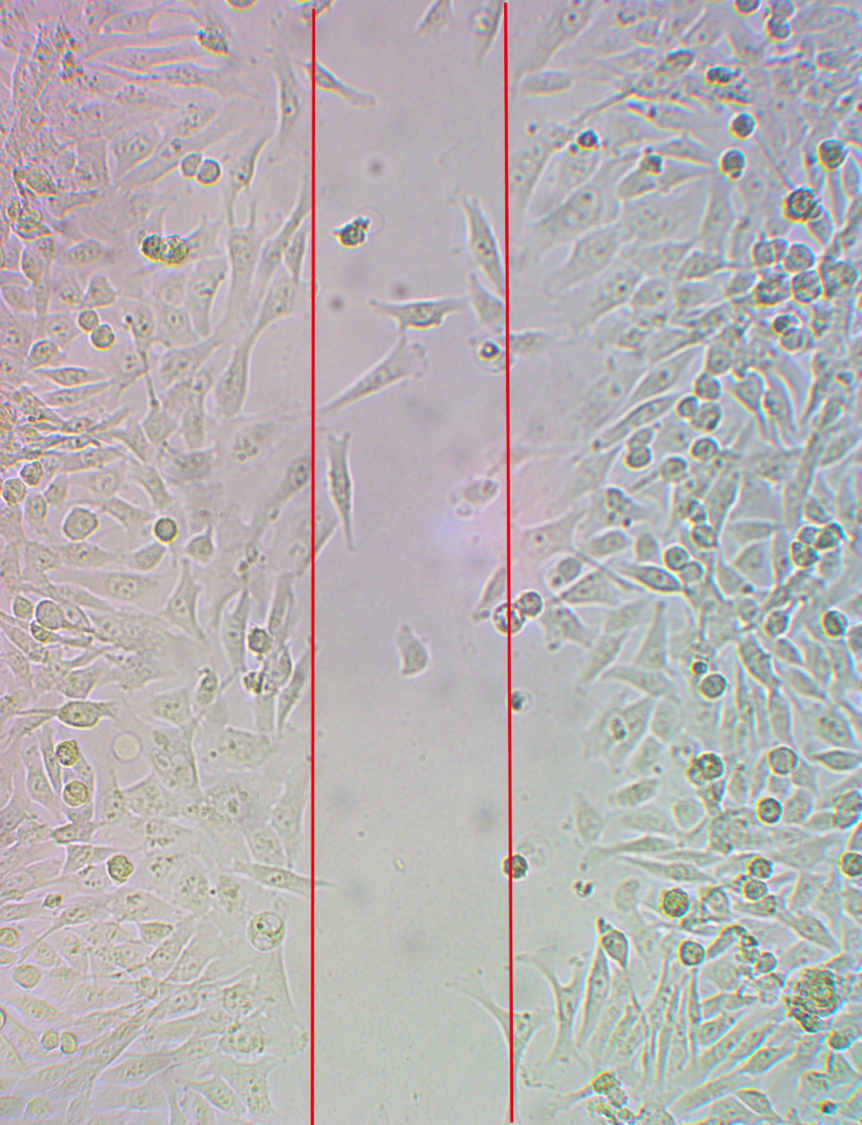

Supplement: Supplementary file 2 [file DataSheet11.ZIP › Fig 2-B-Wound-healing assay/control 48h.tif]

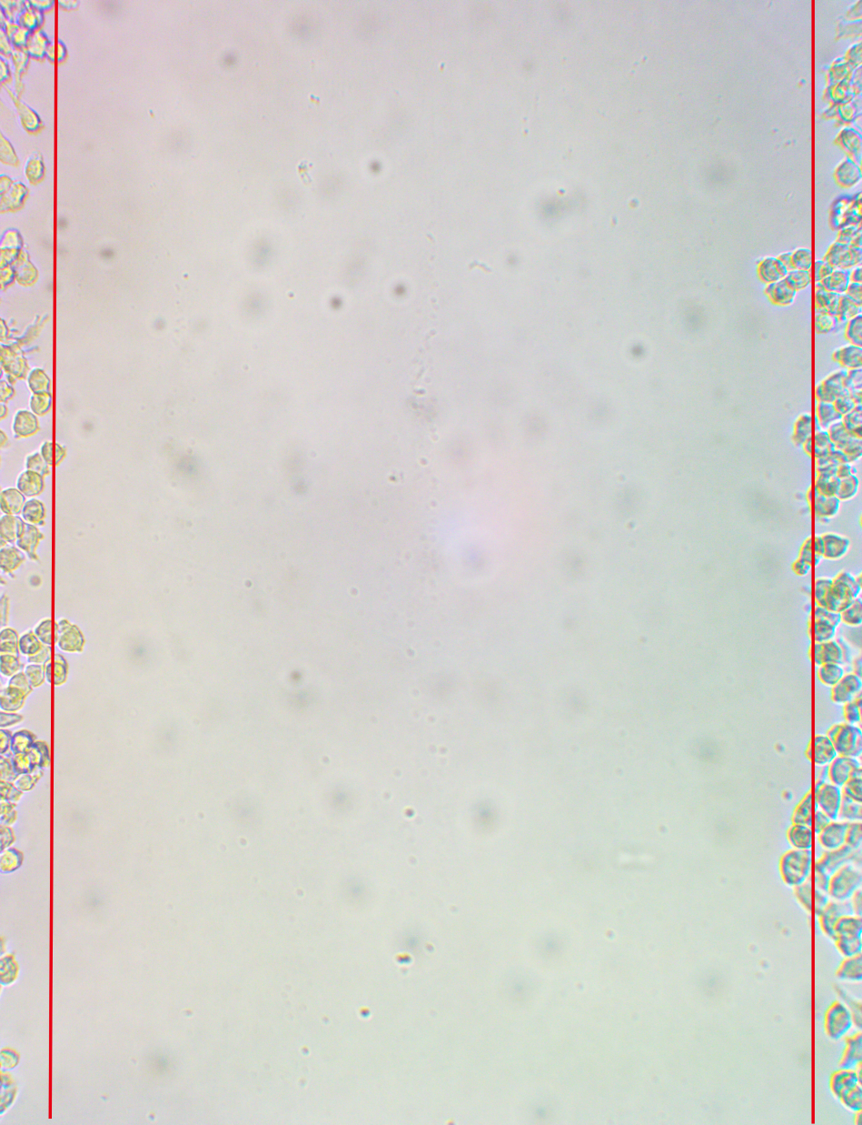

Supplement: Supplementary file 2 [file DataSheet11.ZIP › Fig 2-B-Wound-healing assay/pLC5 0h.tif]

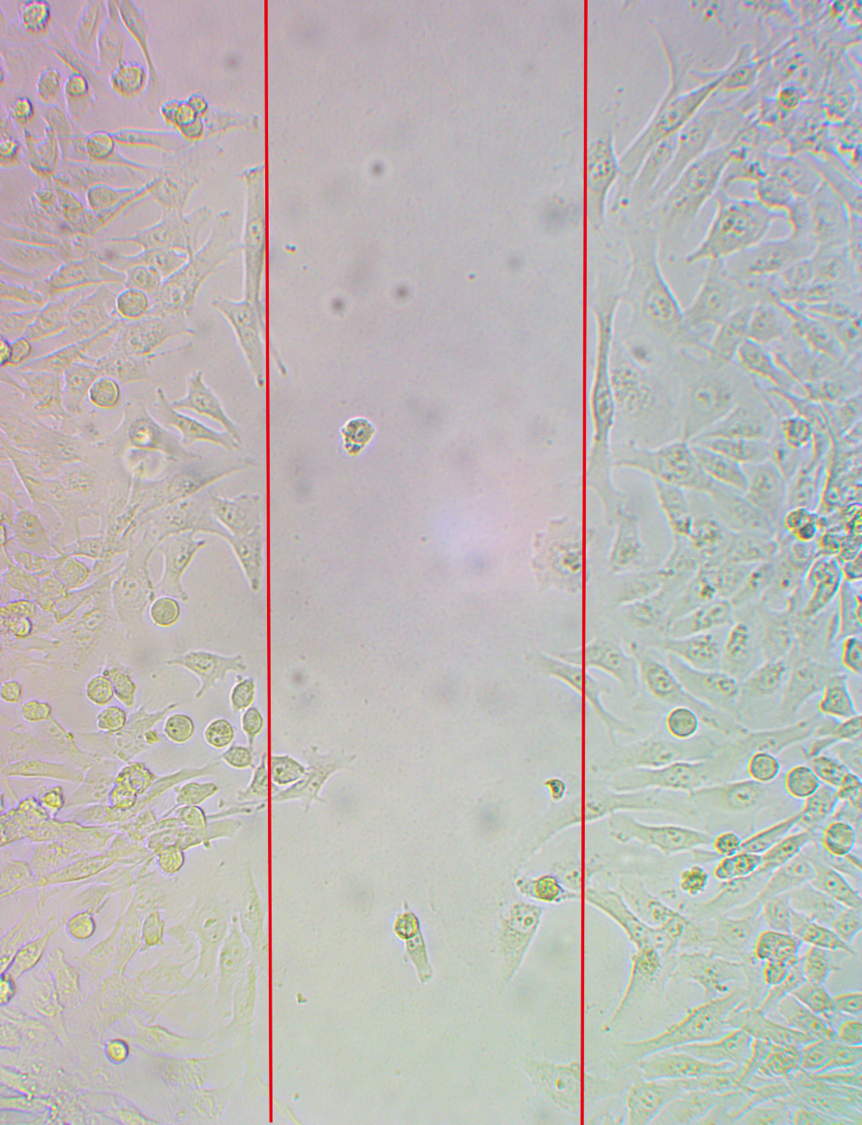

Supplement: Supplementary file 2 [file DataSheet11.ZIP › Fig 2-B-Wound-healing assay/pLC5 24h.tif]

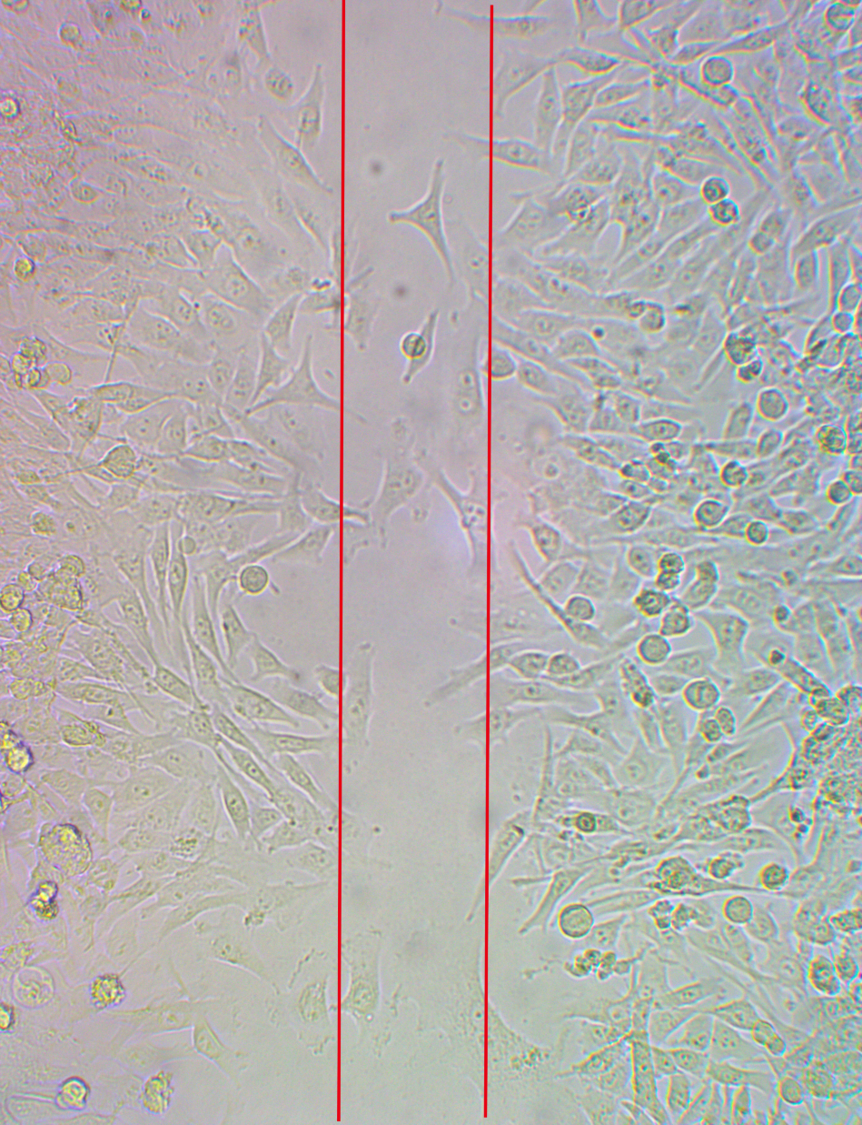

Supplement: Supplementary file 2 [file DataSheet11.ZIP › Fig 2-B-Wound-healing assay/pLC5 48h.tif]

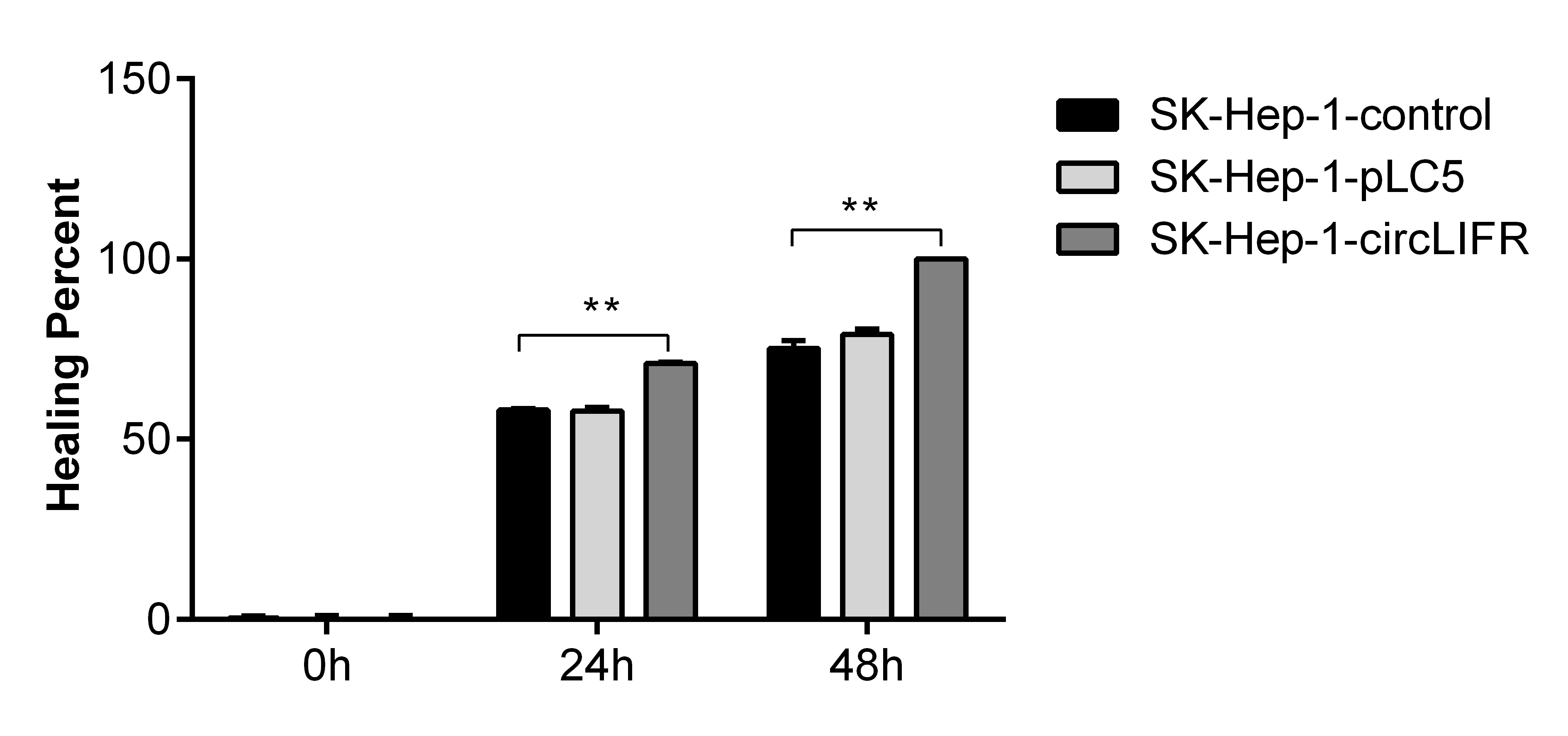

Supplement: Supplementary file 2 [file DataSheet11.ZIP › Fig 2-B-Wound-healing assay/sk-hep-1╗«║█.jpg]

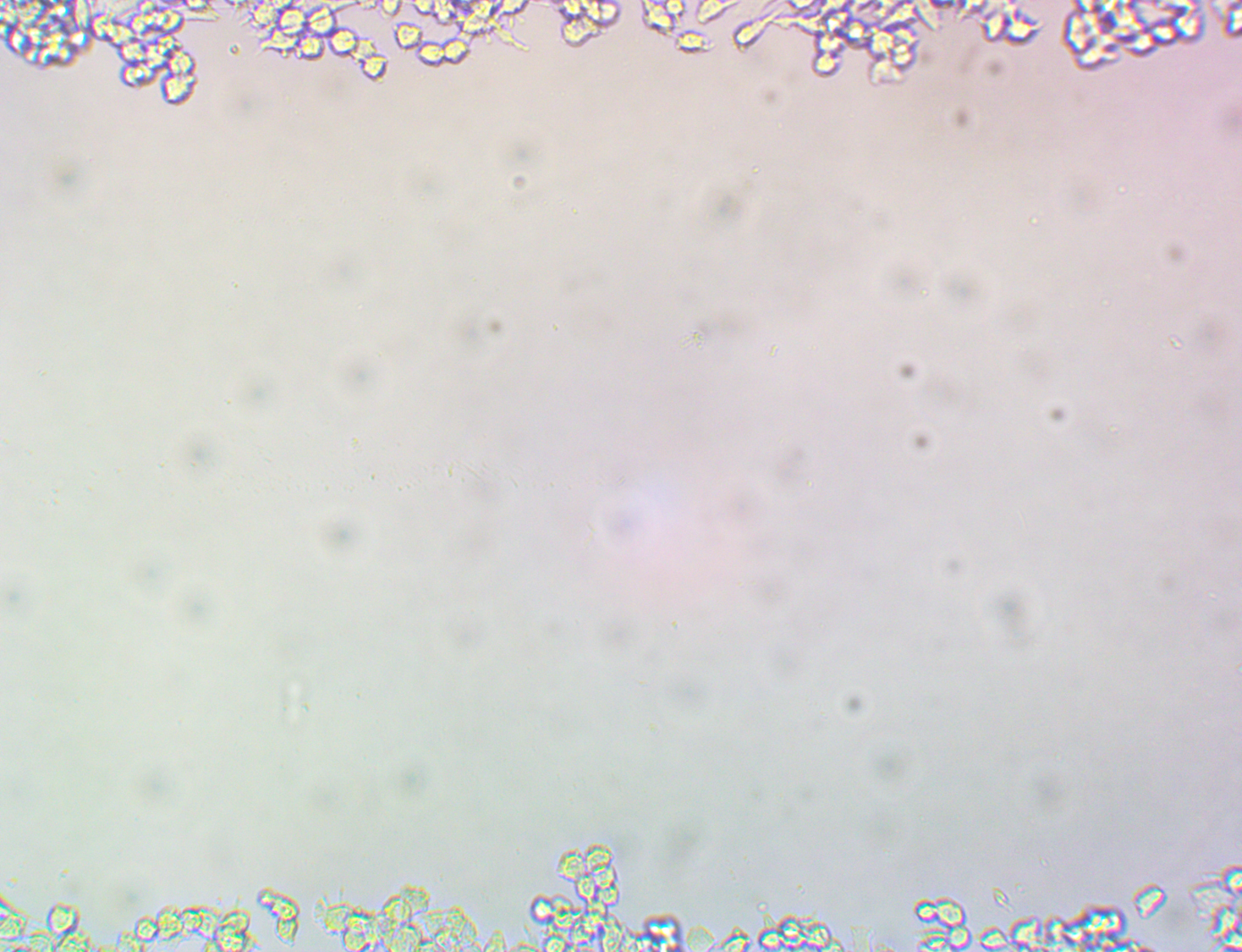

Supplement: Supplementary file 3 [file DataSheet8.ZIP › 0H/control-0h-1 raw.jpg]

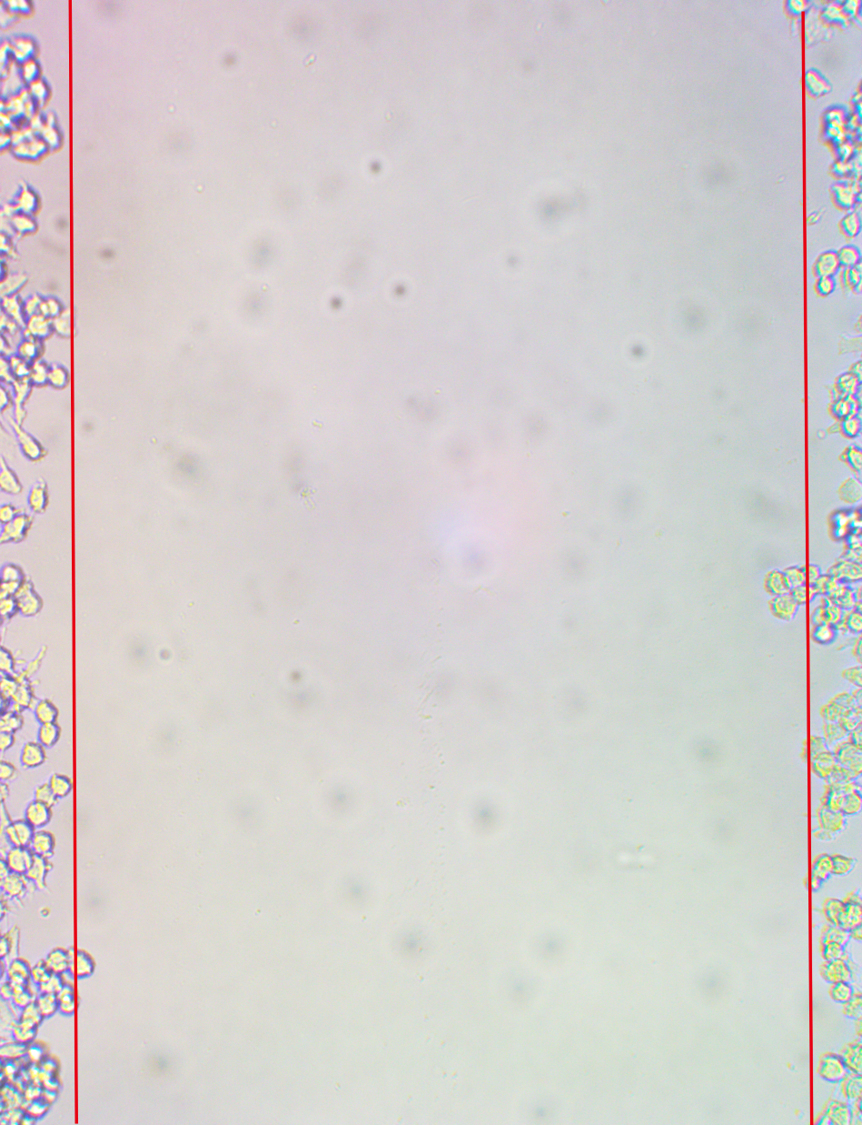

Supplement: Supplementary file 3 [file DataSheet8.ZIP › 0H/control-0h-1.jpg]

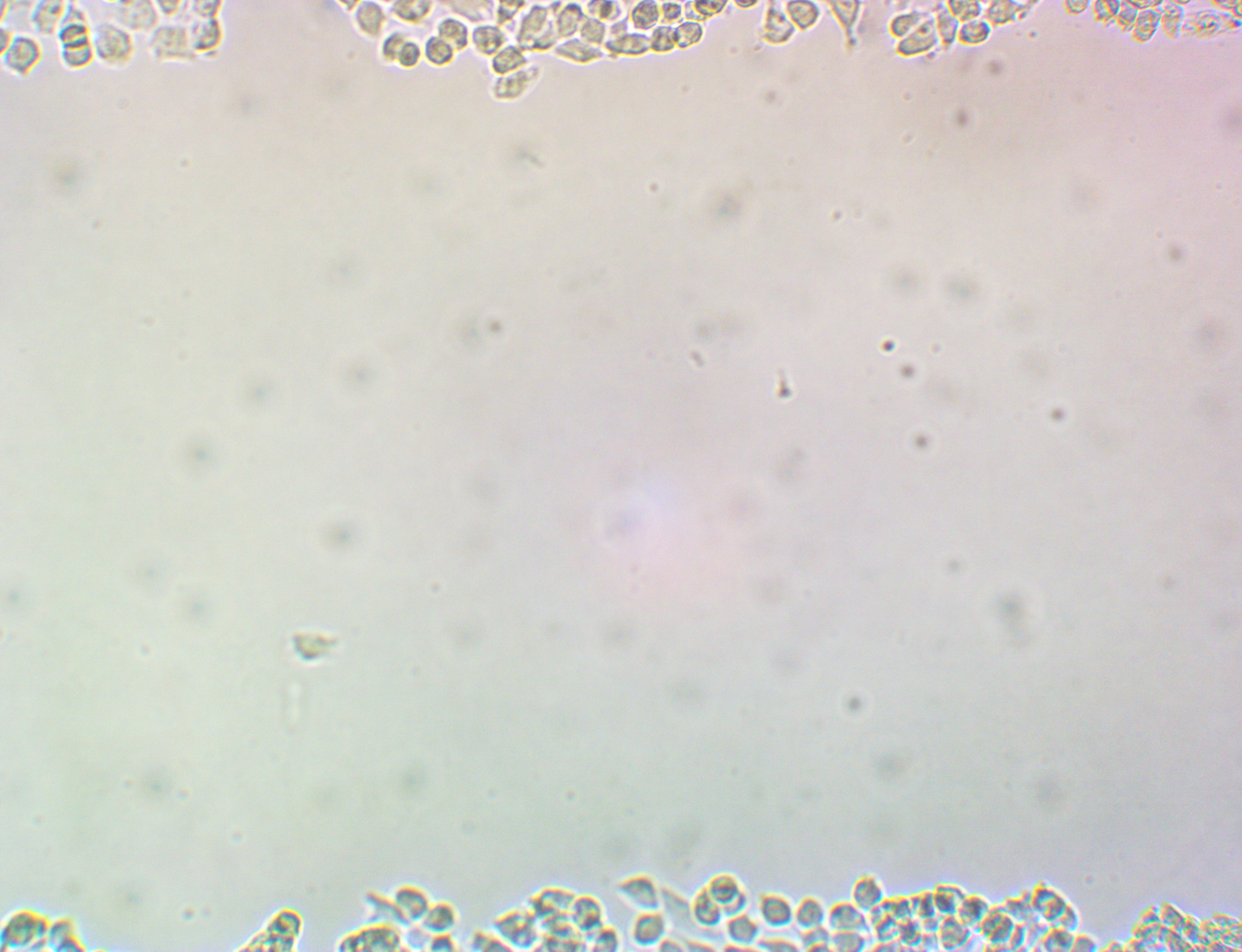

Supplement: Supplementary file 3 [file DataSheet8.ZIP › 0H/control-0h-2 raw.jpg]

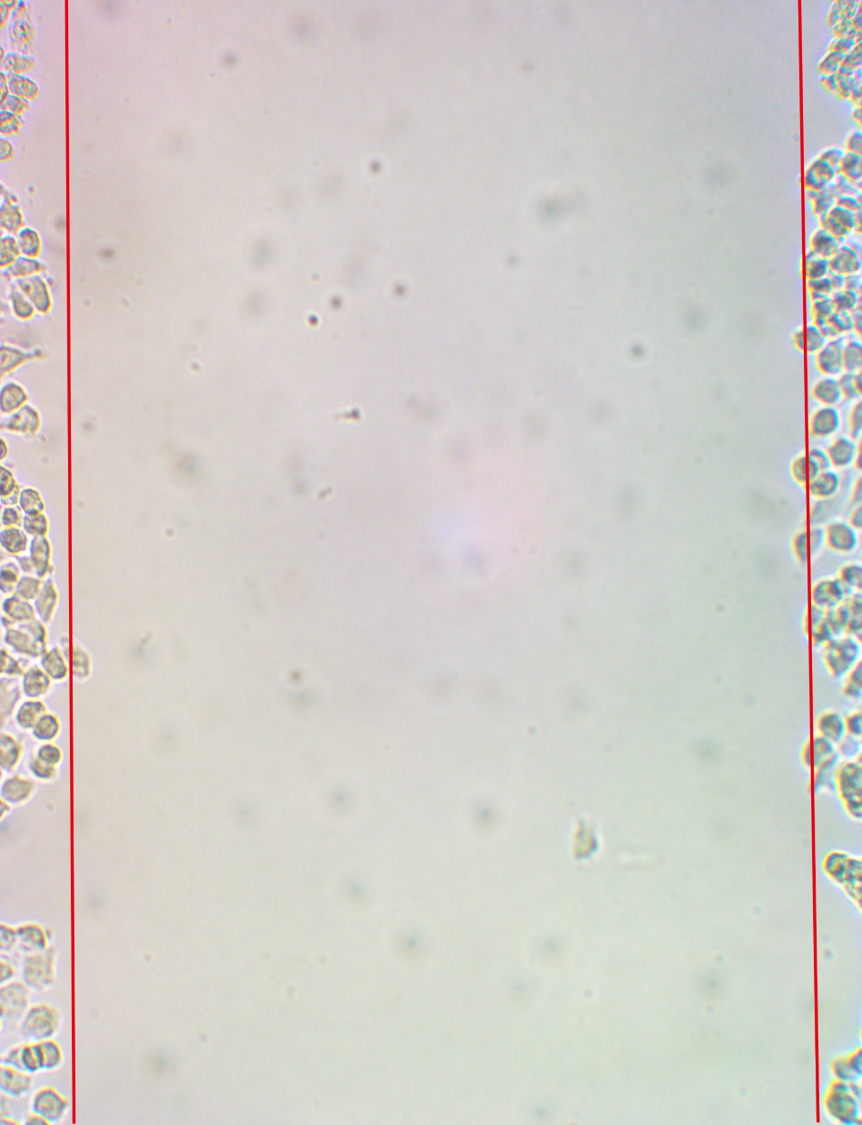

Supplement: Supplementary file 3 [file DataSheet8.ZIP › 0H/control-0h-2.jpg]

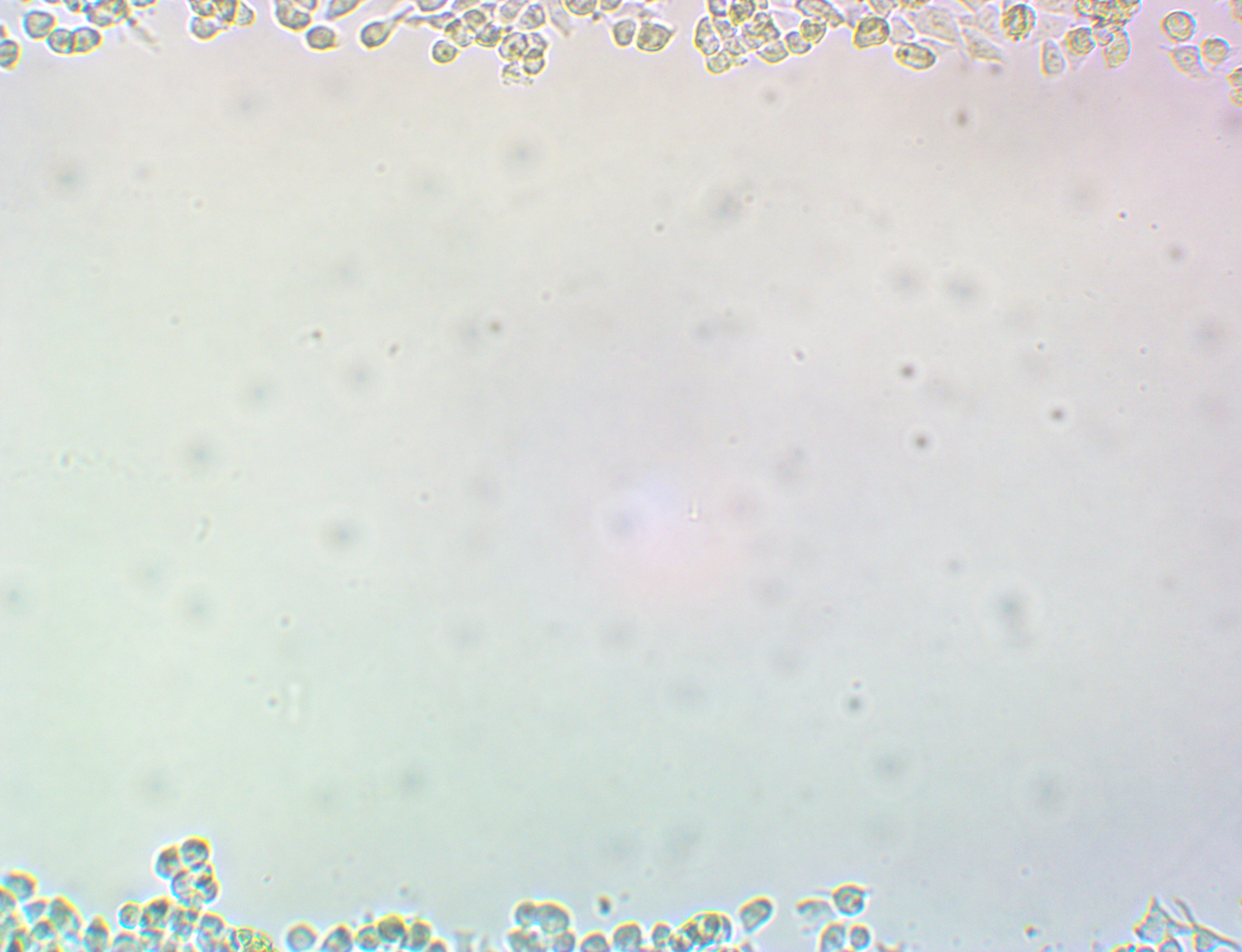

Supplement: Supplementary file 3 [file DataSheet8.ZIP › 0H/control-0h-4 raw.jpg]

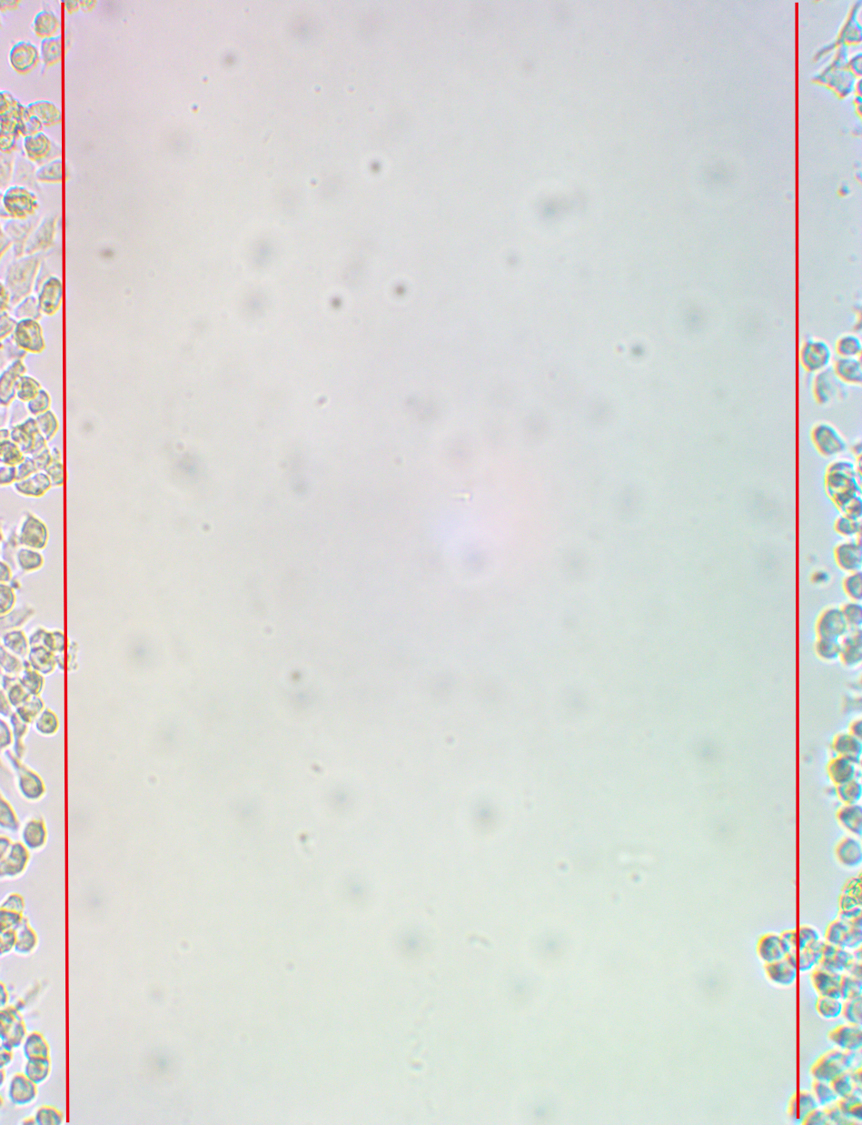

Supplement: Supplementary file 3 [file DataSheet8.ZIP › 0H/control-0h-4.jpg]

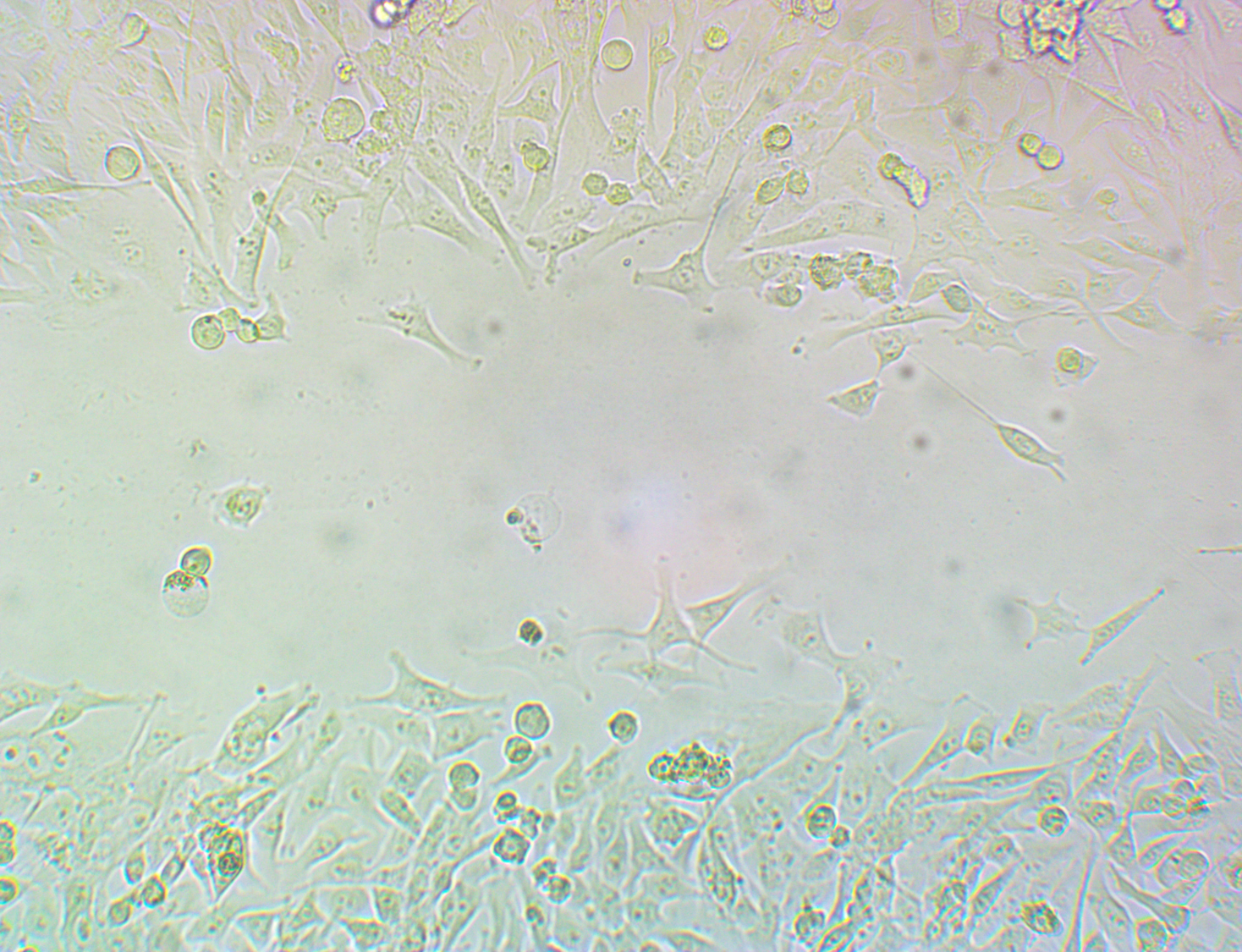

Supplement: Supplementary file 3 [file DataSheet8.ZIP › 24H/control-24h-14 raw.jpg]

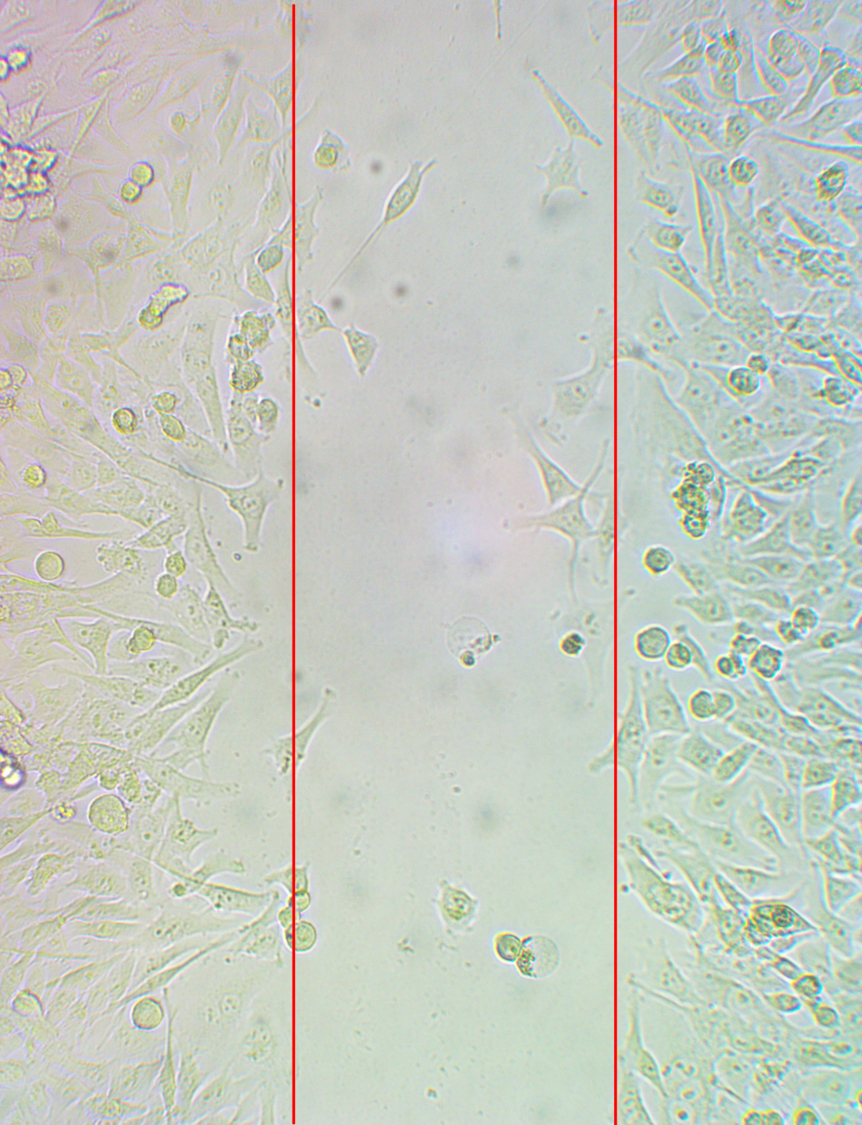

Supplement: Supplementary file 3 [file DataSheet8.ZIP › 24H/control-24h-14.jpg]

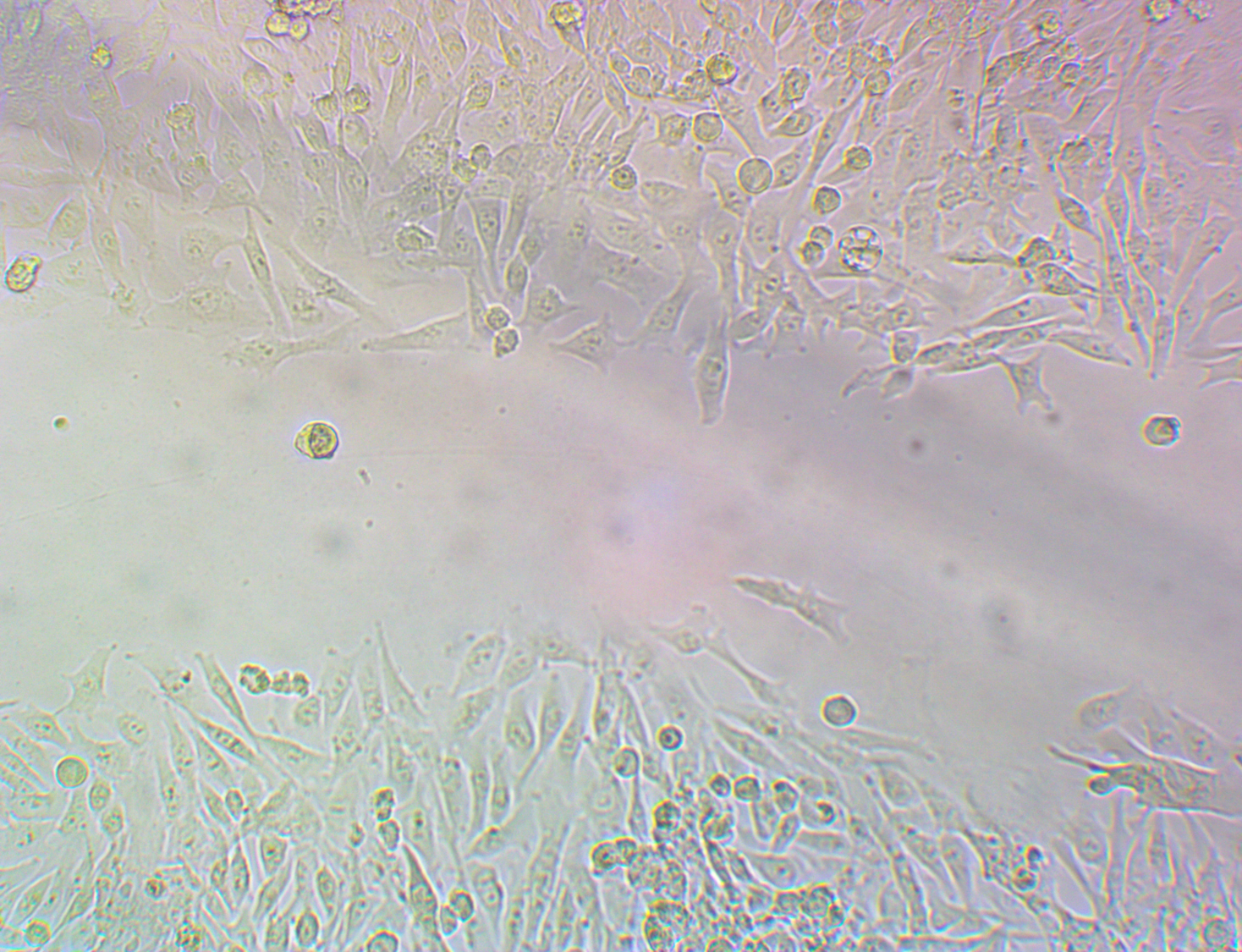

Supplement: Supplementary file 3 [file DataSheet8.ZIP › 24H/control-24h-22 raw.jpg]

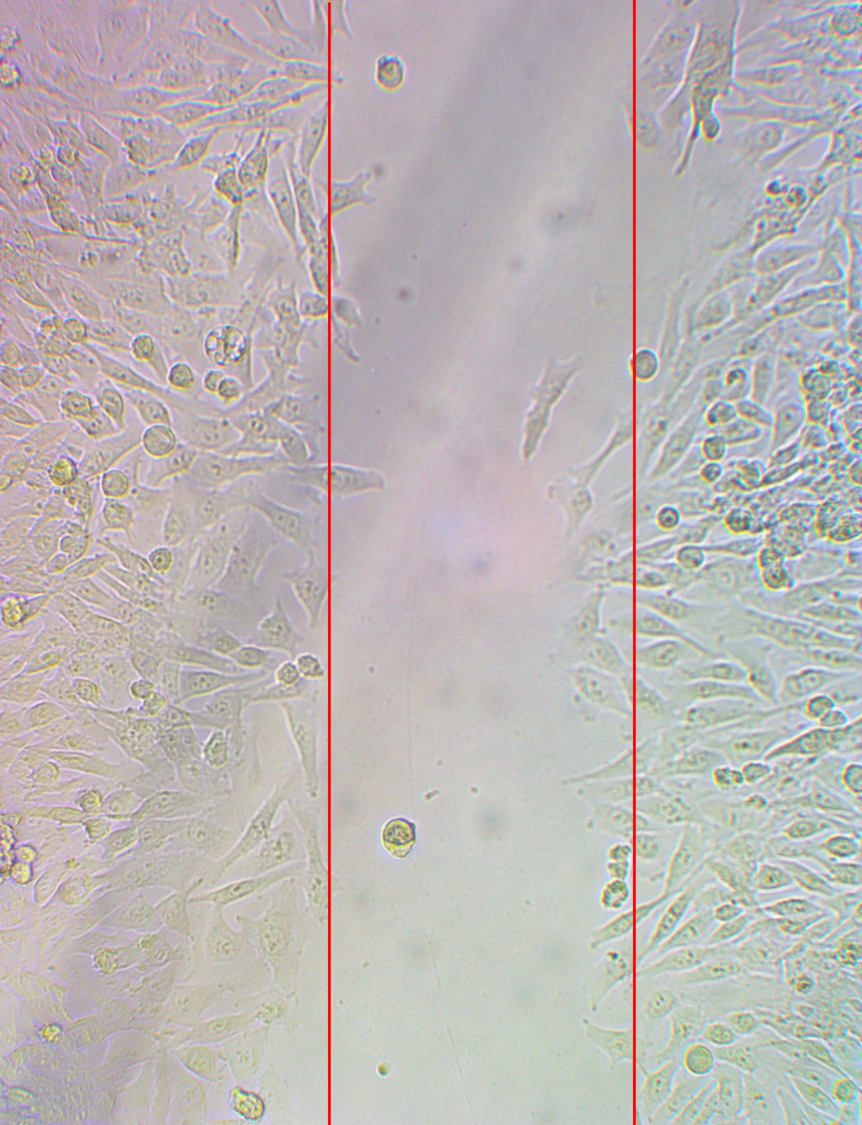

Supplement: Supplementary file 3 [file DataSheet8.ZIP › 24H/control-24h-22.jpg]

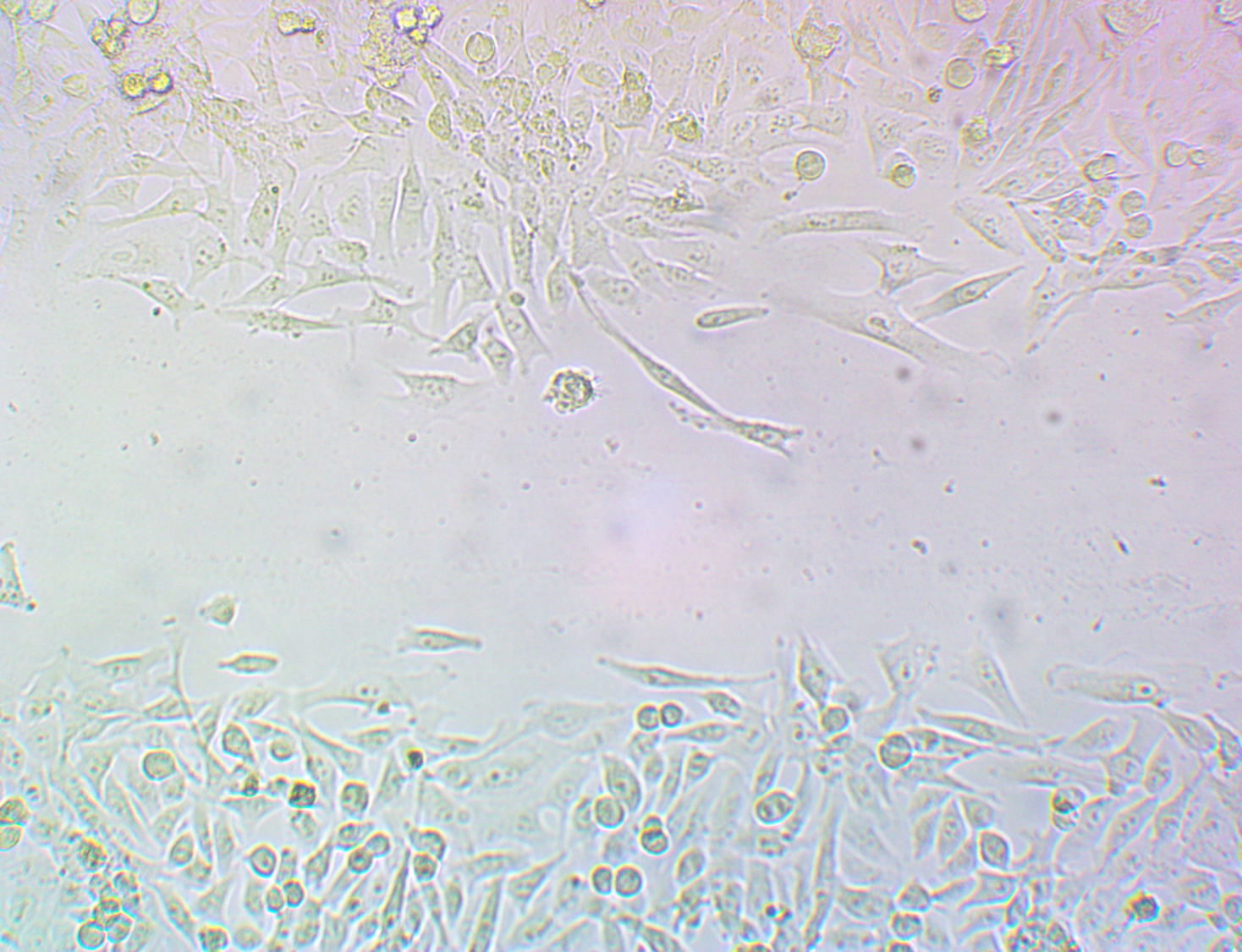

Supplement: Supplementary file 3 [file DataSheet8.ZIP › 24H/control-24h-4 raw.jpg]

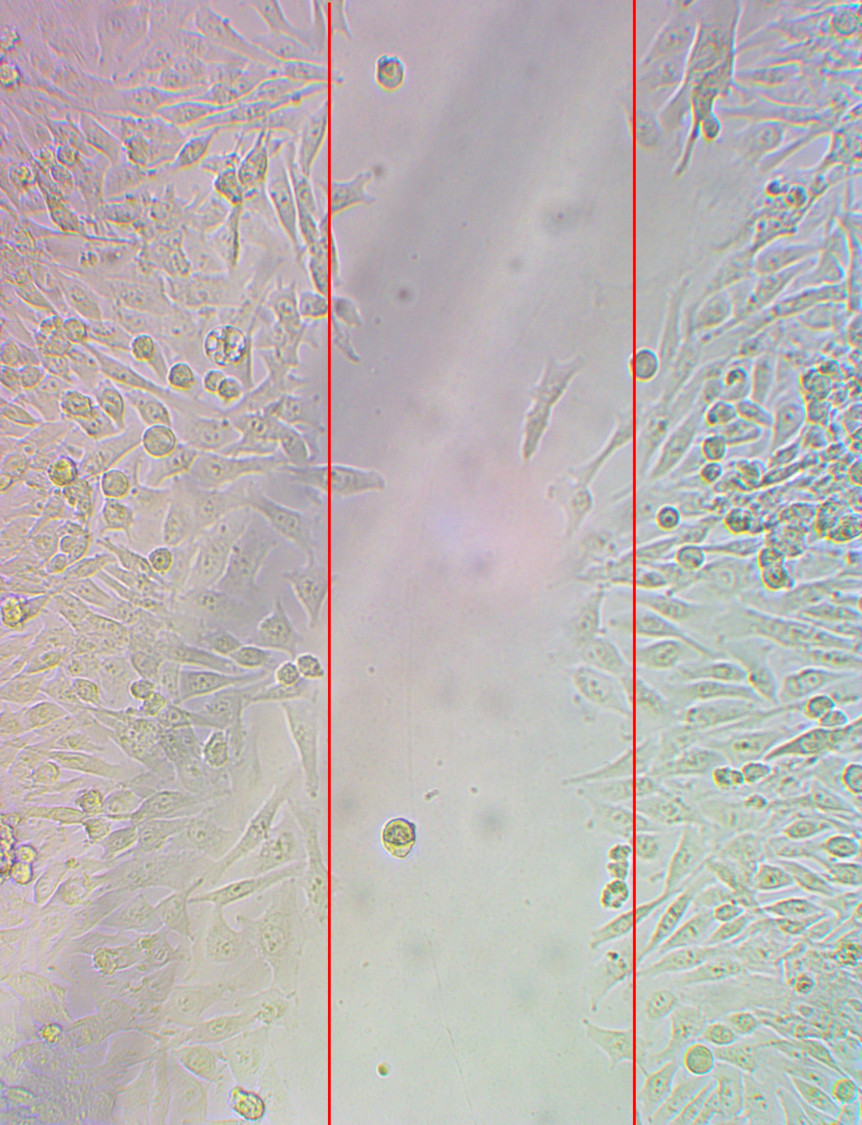

Supplement: Supplementary file 3 [file DataSheet8.ZIP › 24H/control-24h-4.jpg]

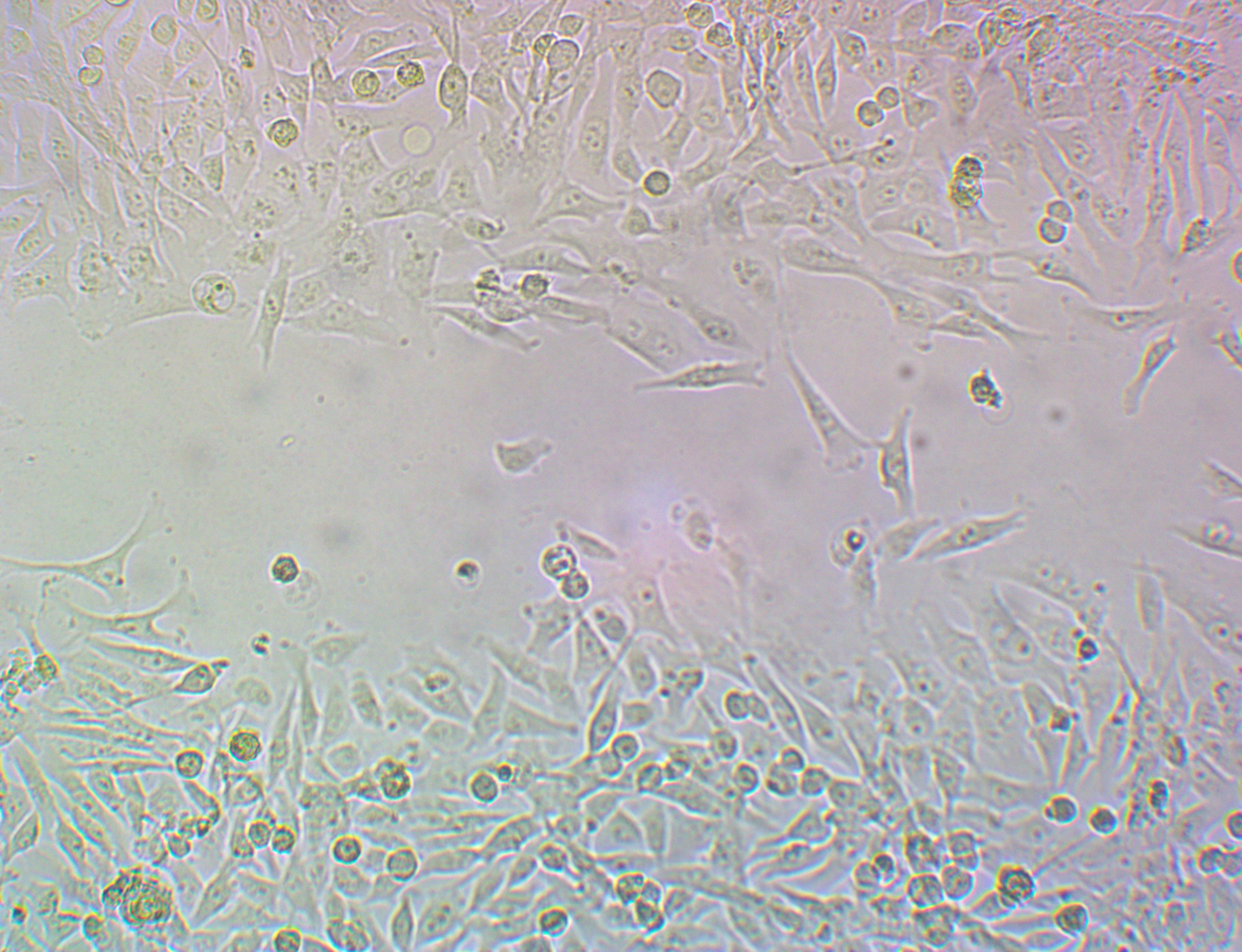

Supplement: Supplementary file 3 [file DataSheet8.ZIP › 48H/control-48h-14 raw.jpg]

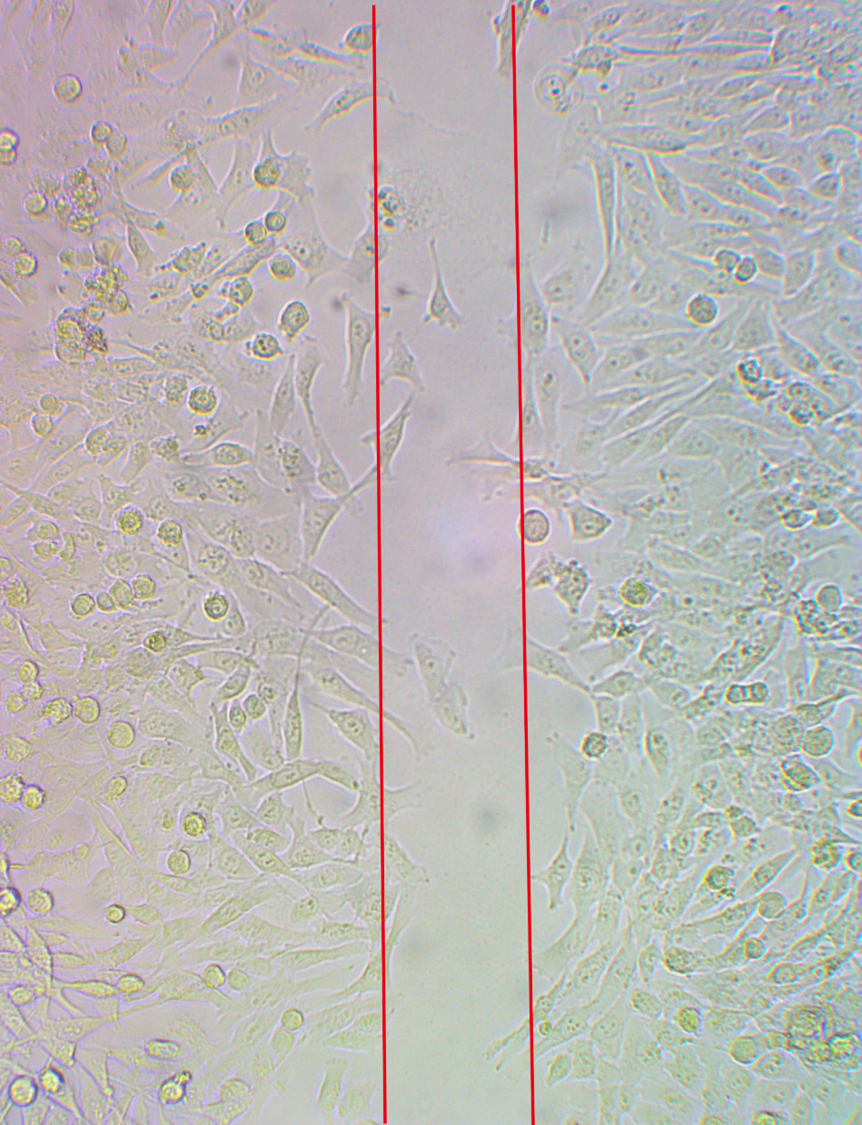

Supplement: Supplementary file 3 [file DataSheet8.ZIP › 48H/control-48h-14.jpg]

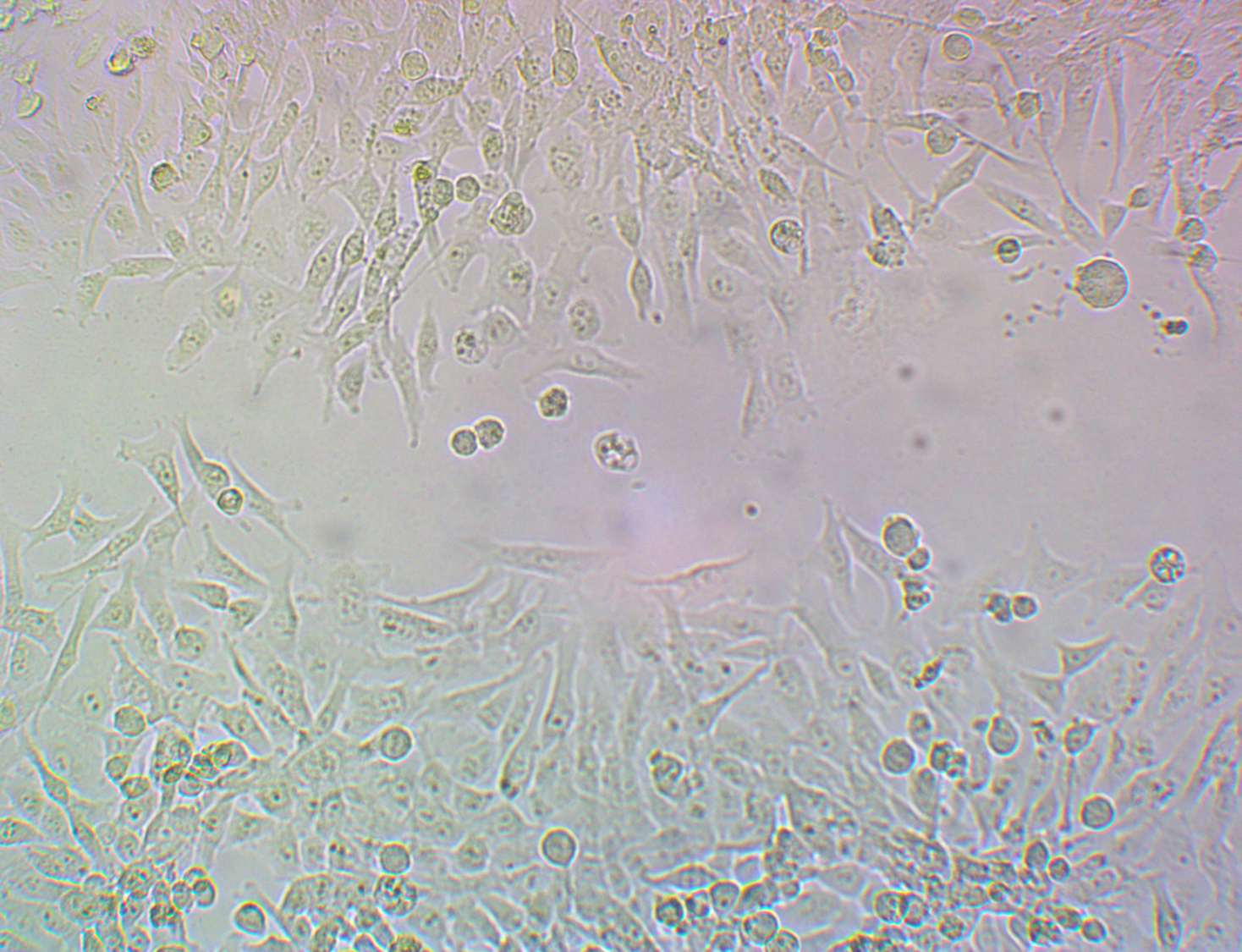

Supplement: Supplementary file 3 [file DataSheet8.ZIP › 48H/control-48h-15 raw.jpg]

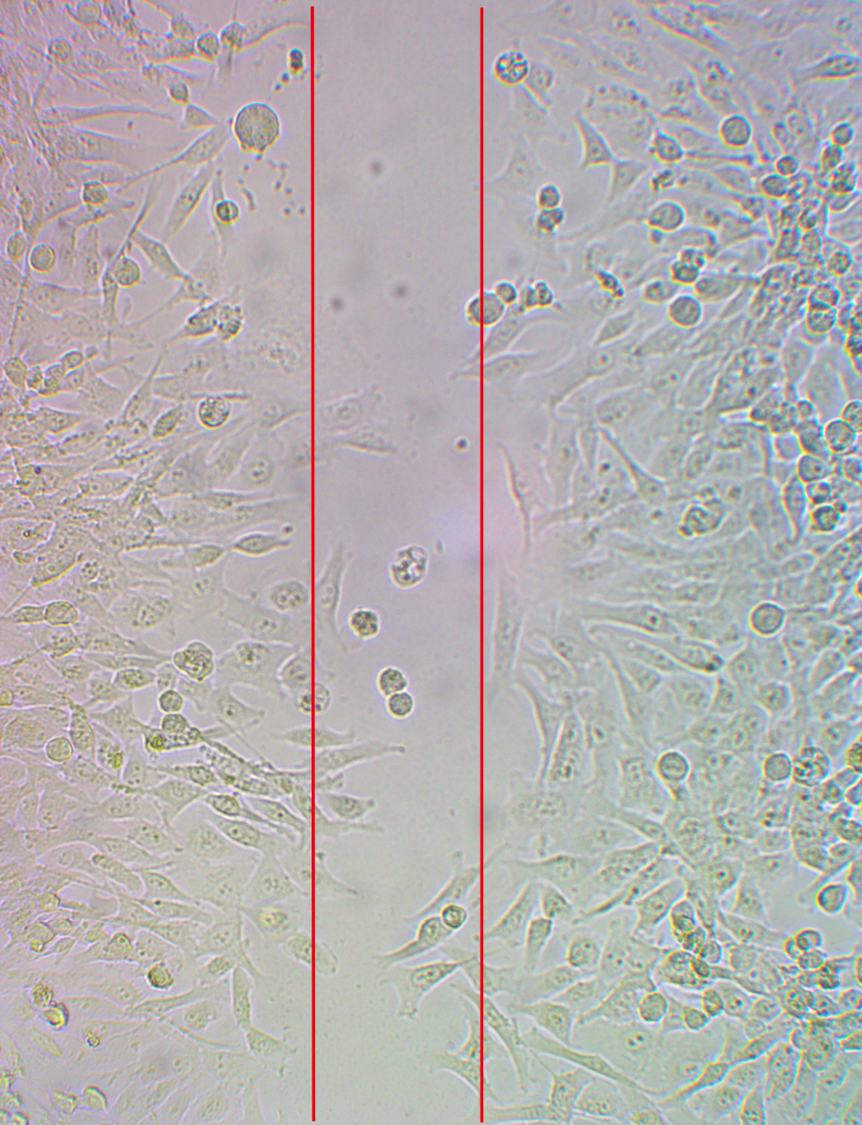

Supplement: Supplementary file 3 [file DataSheet8.ZIP › 48H/control-48h-15.jpg]

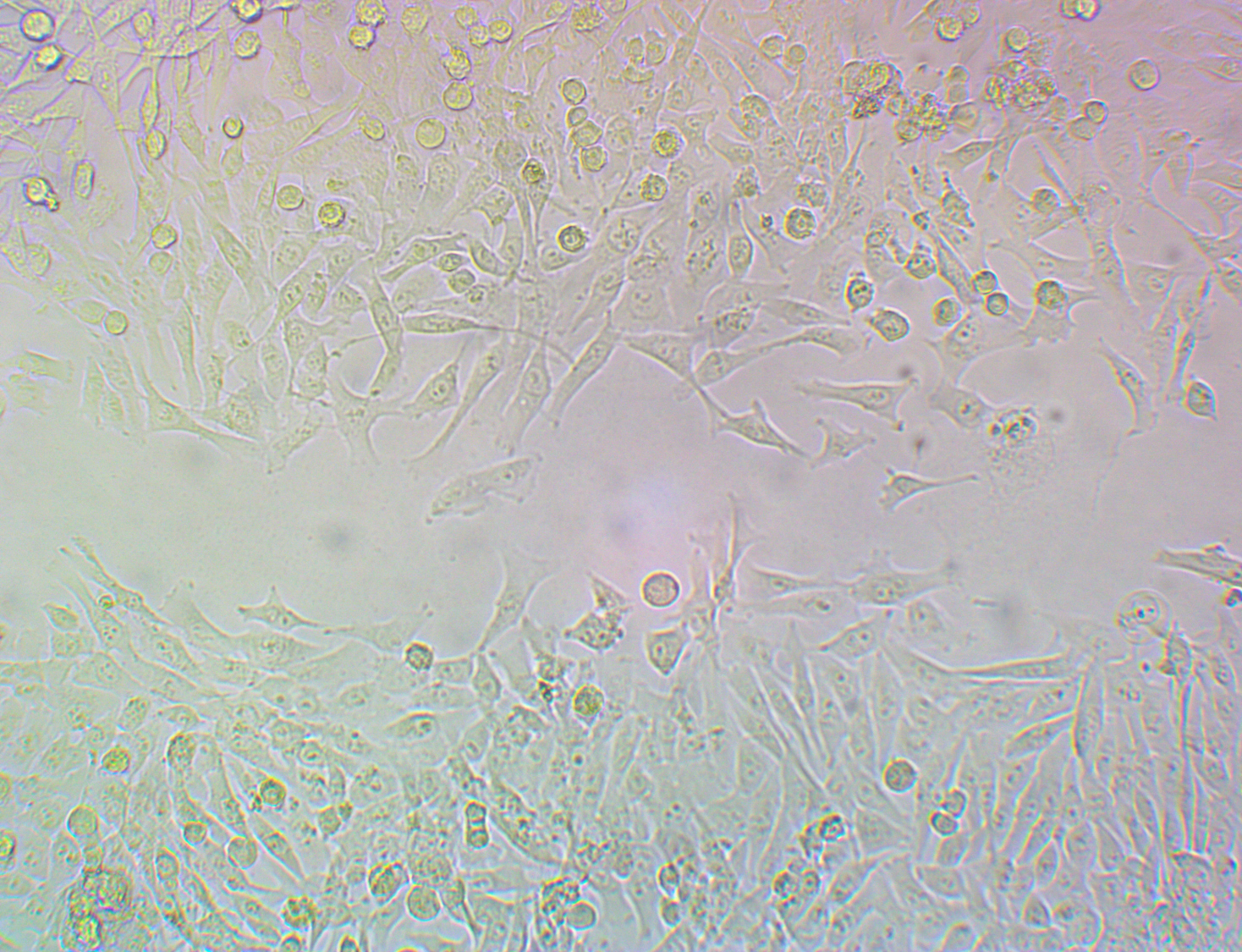

Supplement: Supplementary file 3 [file DataSheet8.ZIP › 48H/control-48h-19 raw.jpg]

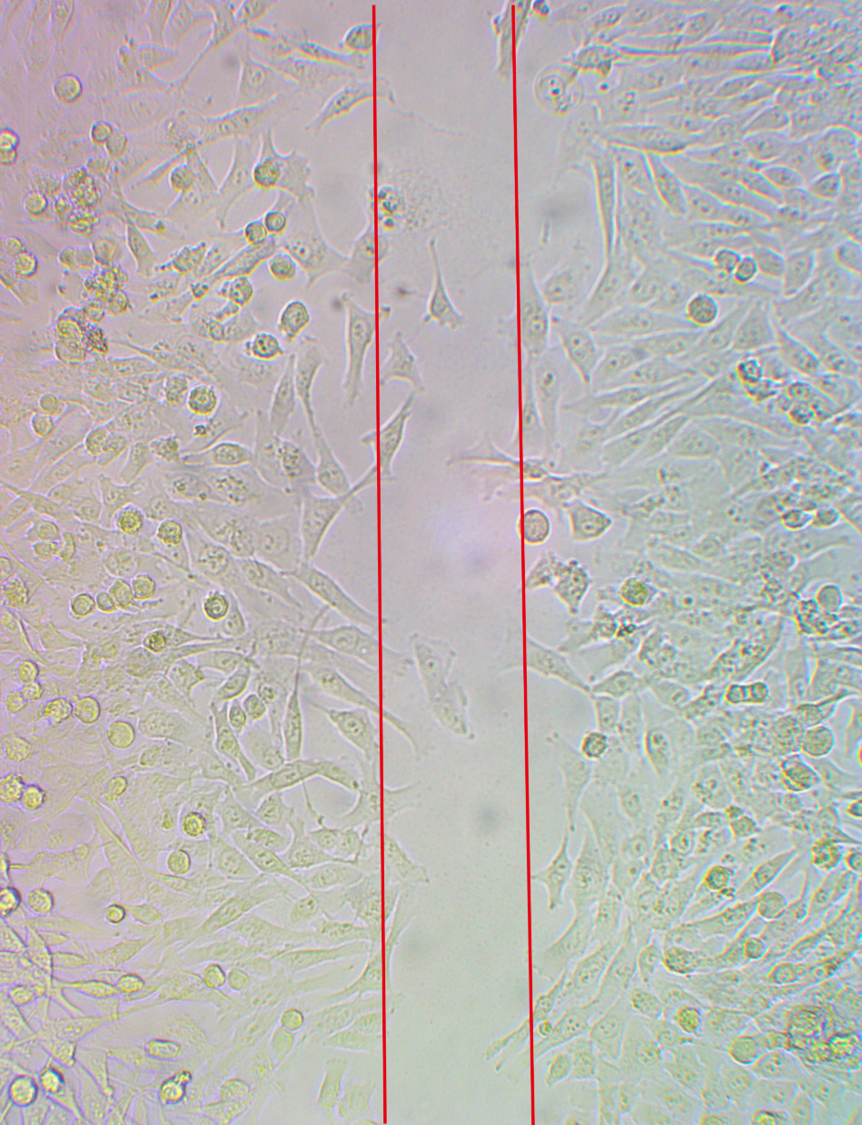

Supplement: Supplementary file 3 [file DataSheet8.ZIP › 48H/control-48h-19.jpg]

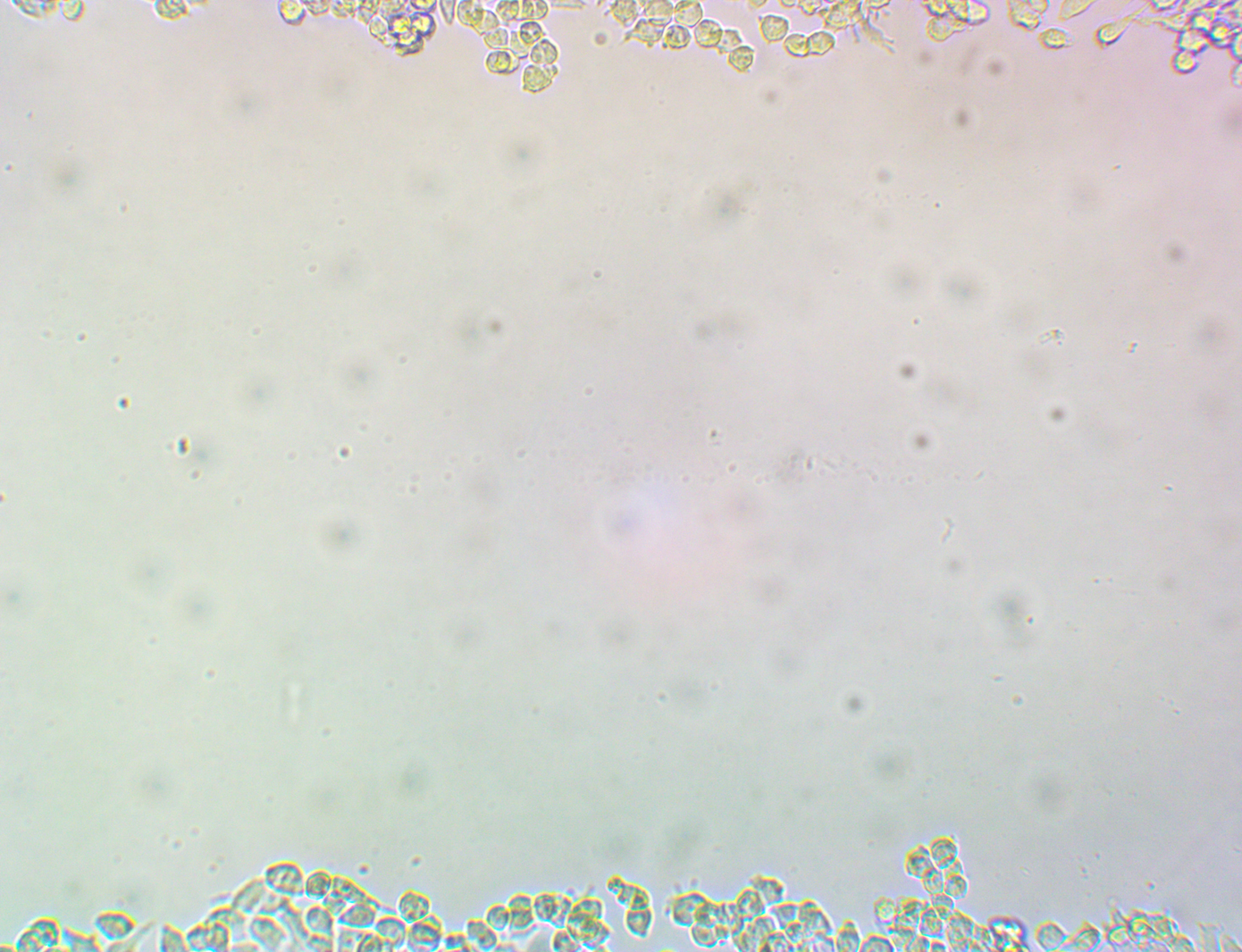

Supplement: Supplementary file 4 [file DataSheet9.ZIP › Fig2-Wound-healing assay-sk hep1-plc5/0H/plc5-0h-3 raw.jpg]

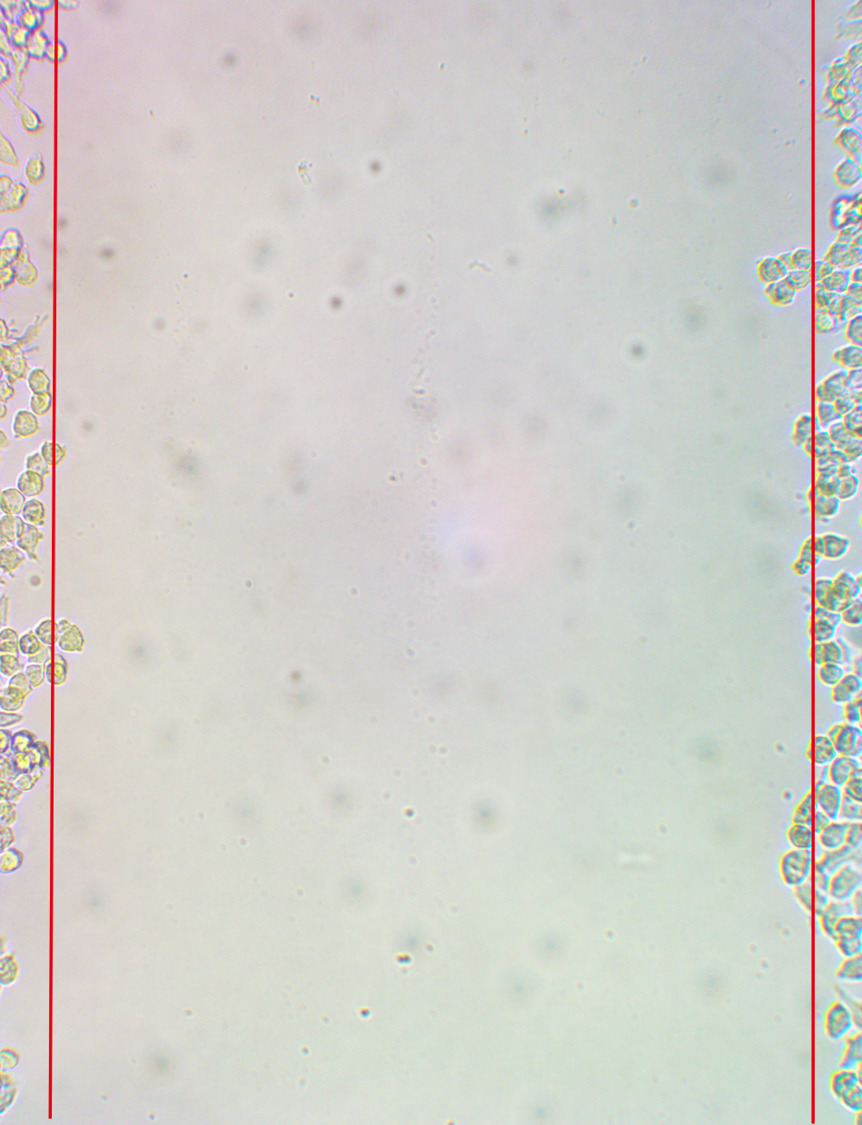

Supplement: Supplementary file 4 [file DataSheet9.ZIP › Fig2-Wound-healing assay-sk hep1-plc5/0H/plc5-0h-3.jpg]

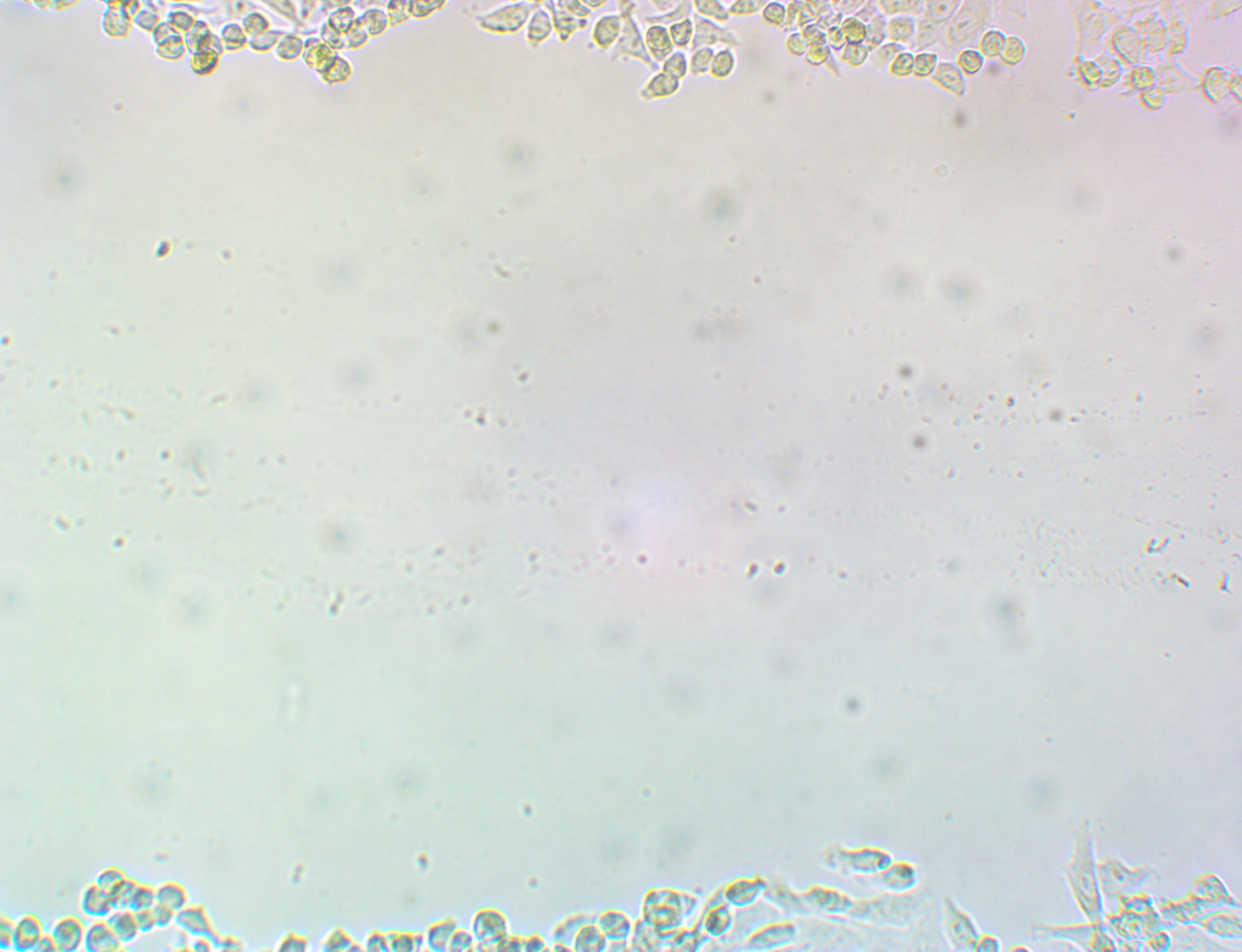

Supplement: Supplementary file 4 [file DataSheet9.ZIP › Fig2-Wound-healing assay-sk hep1-plc5/0H/plc5-0h-5 raw.jpg]

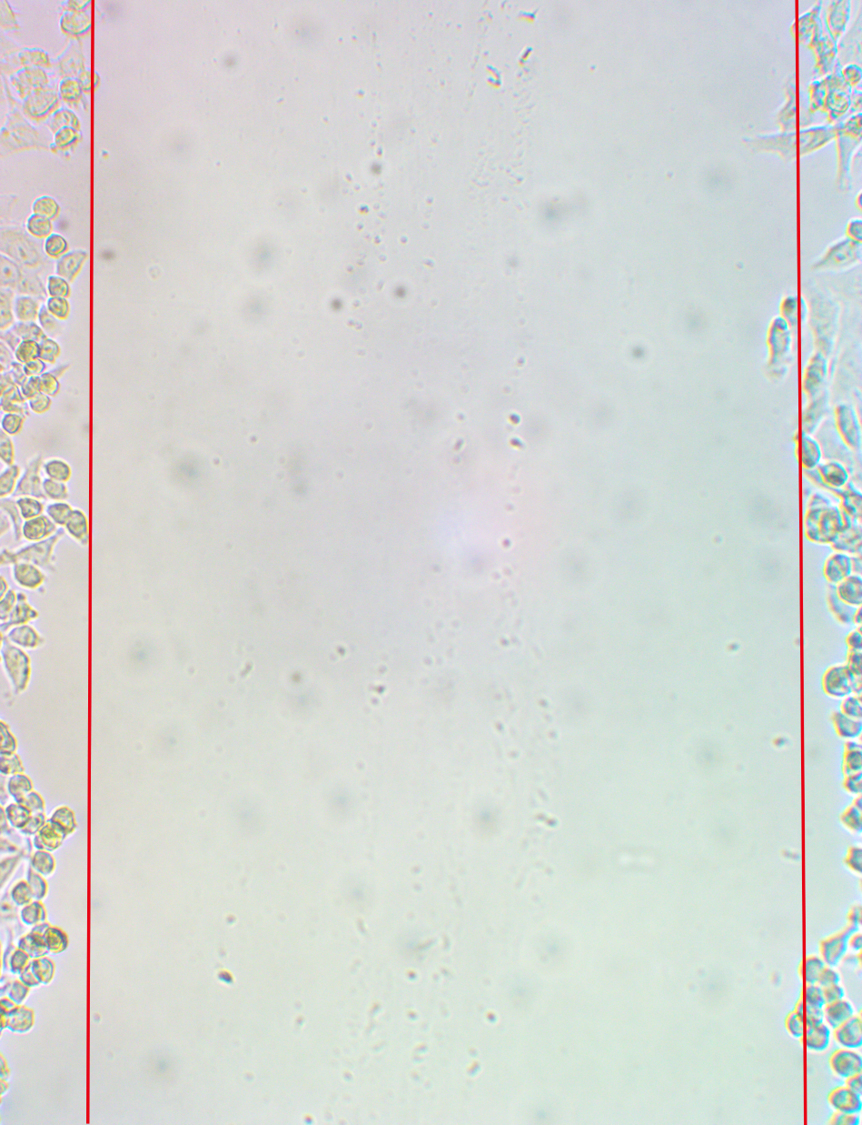

Supplement: Supplementary file 4 [file DataSheet9.ZIP › Fig2-Wound-healing assay-sk hep1-plc5/0H/plc5-0h-5.jpg]

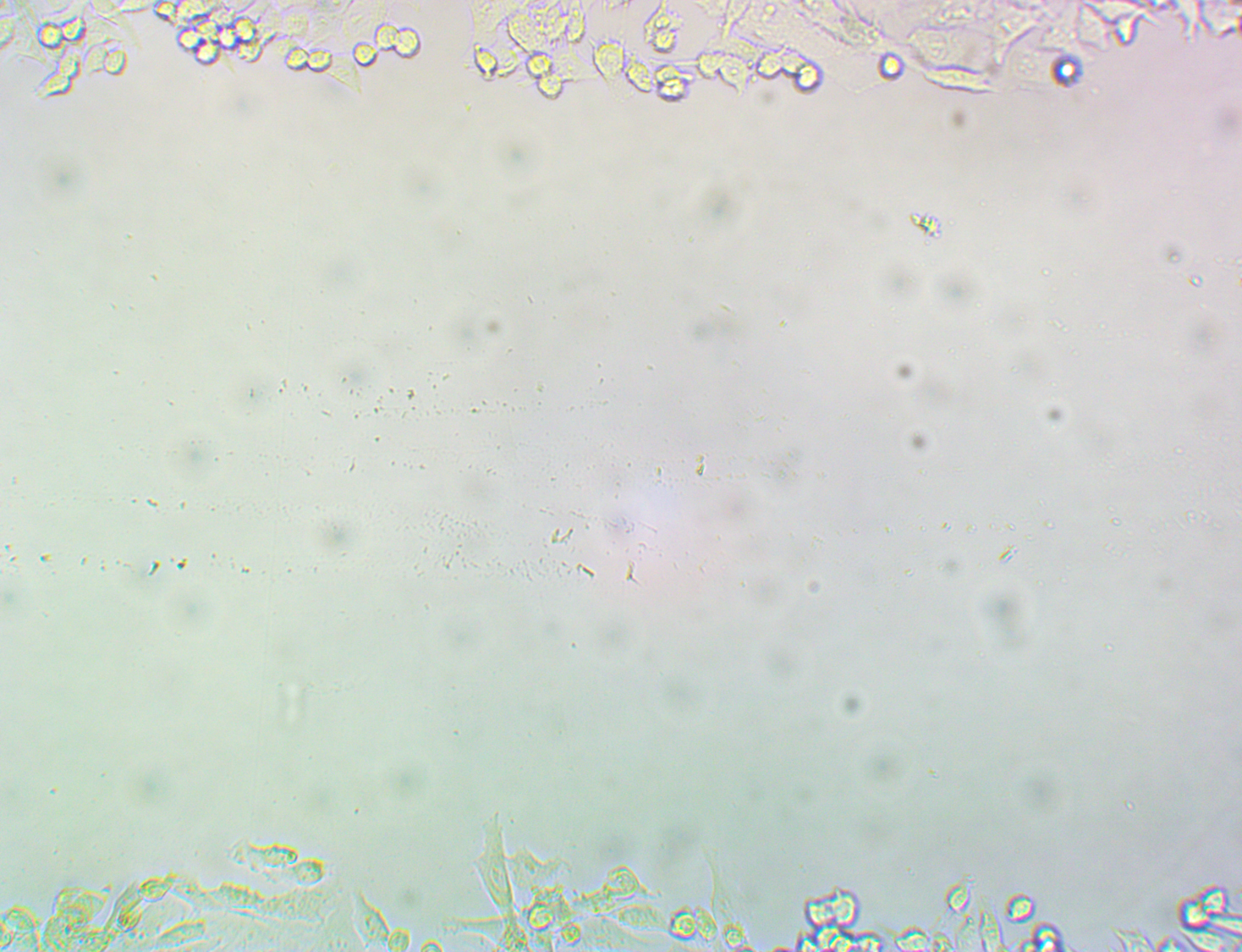

Supplement: Supplementary file 4 [file DataSheet9.ZIP › Fig2-Wound-healing assay-sk hep1-plc5/0H/plc5-0h-6 raw.jpg]

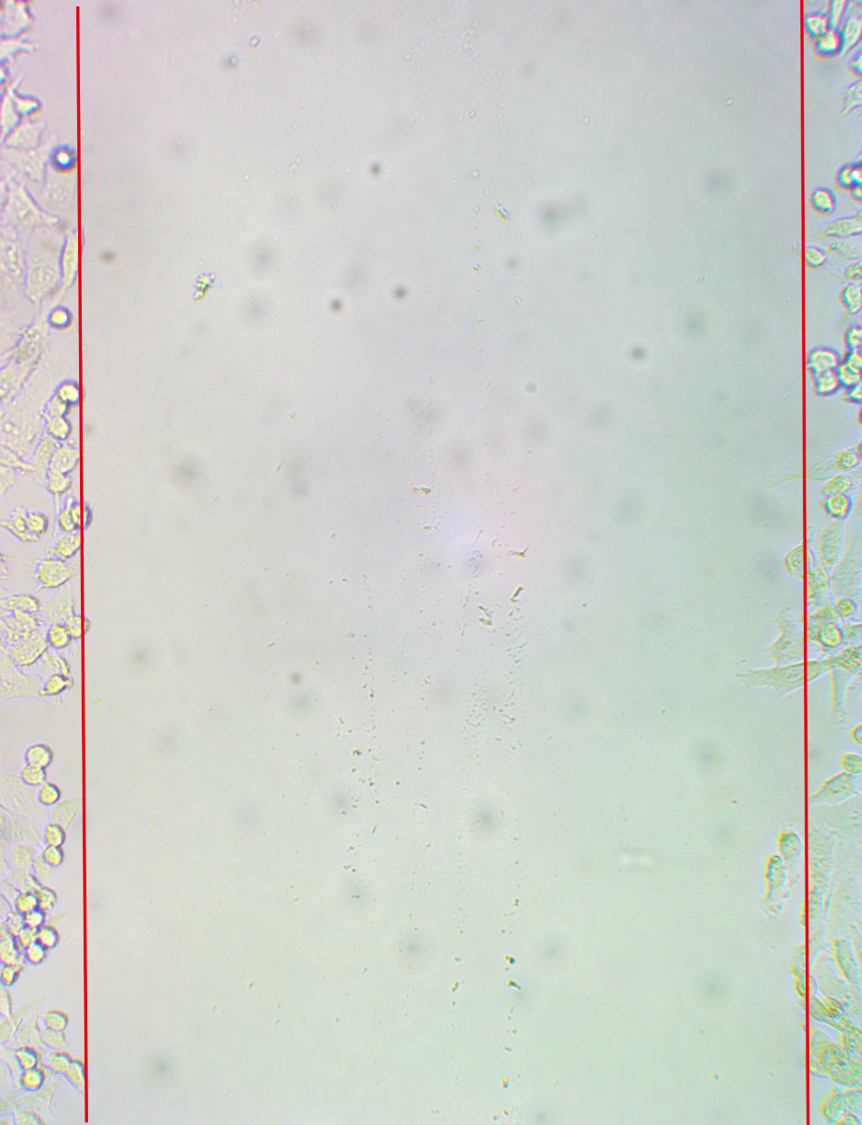

Supplement: Supplementary file 4 [file DataSheet9.ZIP › Fig2-Wound-healing assay-sk hep1-plc5/0H/plc5-0h-6.jpg]

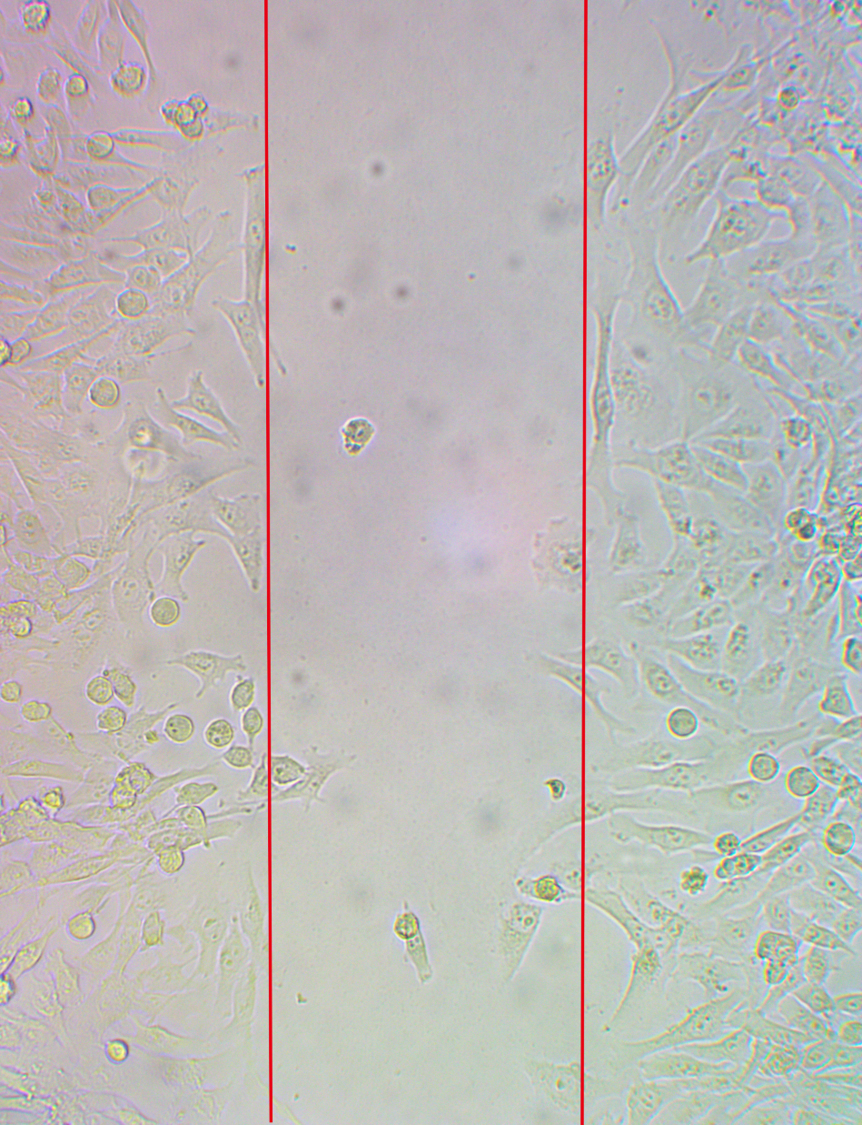

Supplement: Supplementary file 4 [file DataSheet9.ZIP › Fig2-Wound-healing assay-sk hep1-plc5/24H/plc5-24h-20 .jpg]

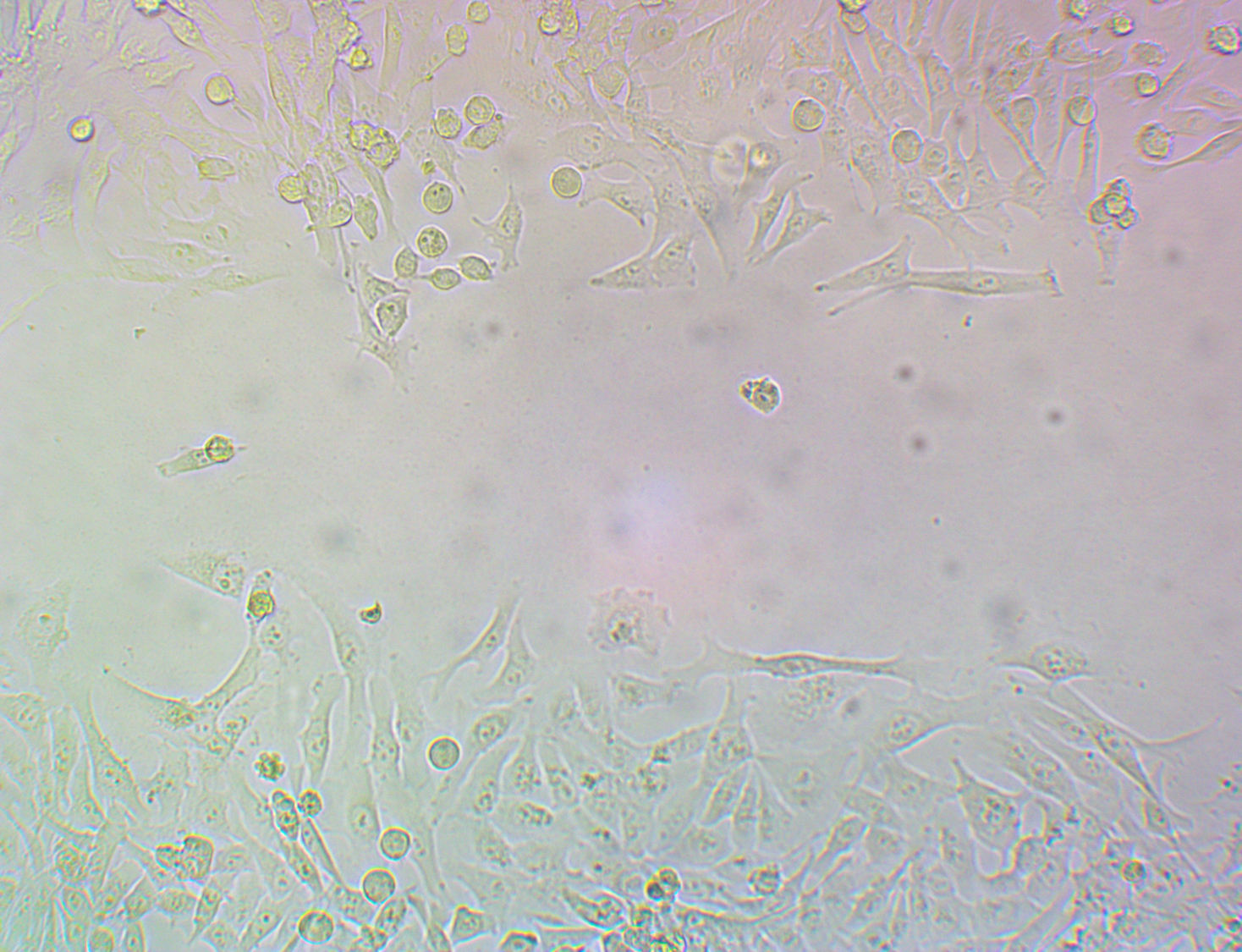

Supplement: Supplementary file 4 [file DataSheet9.ZIP › Fig2-Wound-healing assay-sk hep1-plc5/24H/plc5-24h-20 raw.jpg]

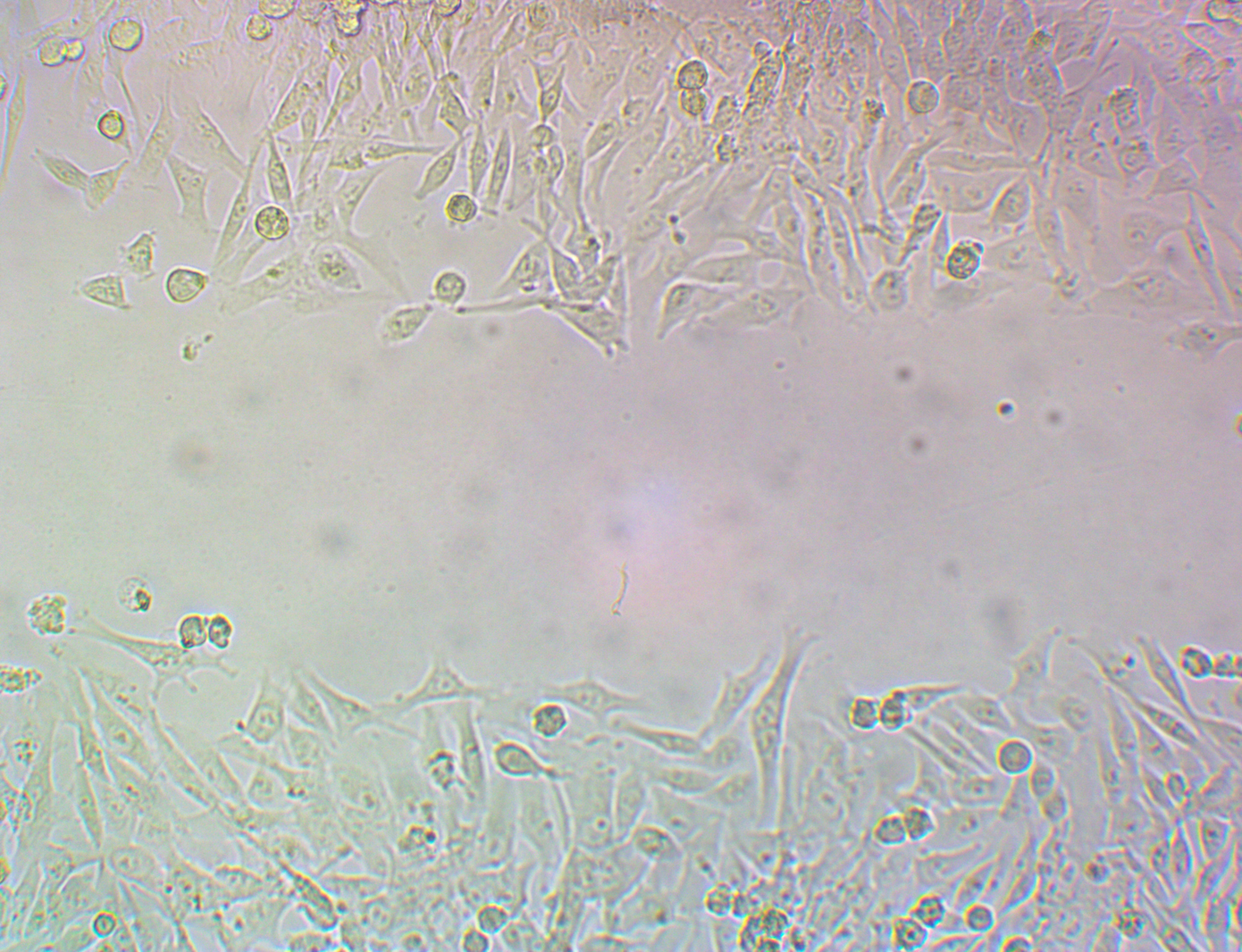

Supplement: Supplementary file 4 [file DataSheet9.ZIP › Fig2-Wound-healing assay-sk hep1-plc5/24H/plc5-24h-21 raw.jpg]

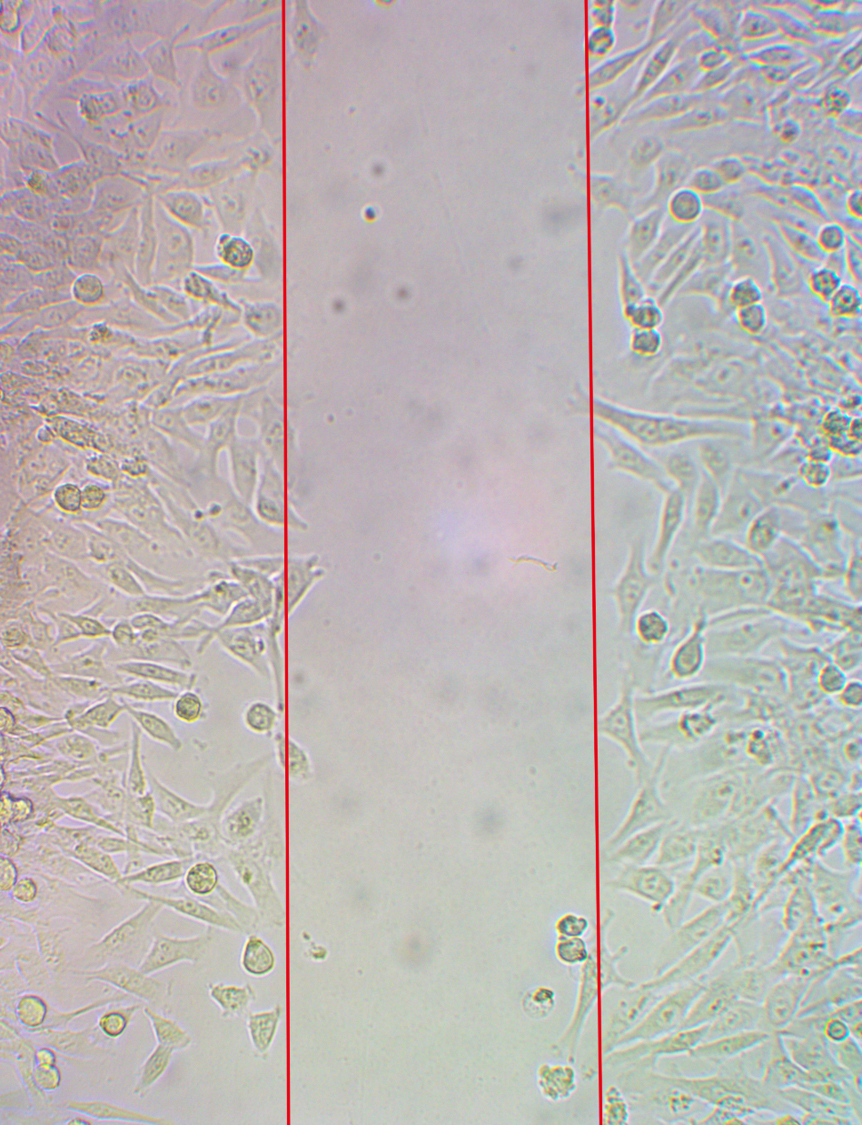

Supplement: Supplementary file 4 [file DataSheet9.ZIP › Fig2-Wound-healing assay-sk hep1-plc5/24H/plc5-24h-21.jpg]

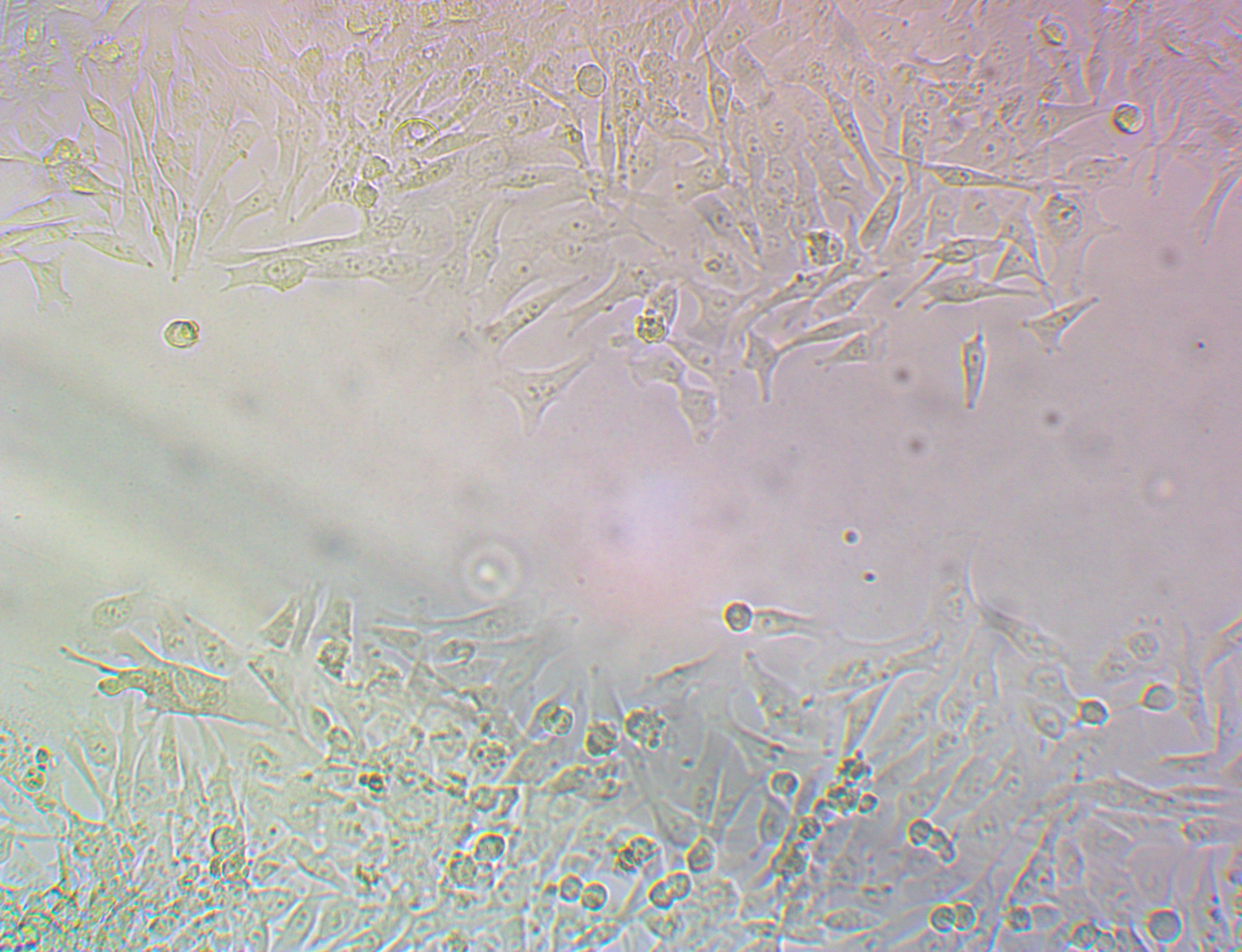

Supplement: Supplementary file 4 [file DataSheet9.ZIP › Fig2-Wound-healing assay-sk hep1-plc5/24H/plc5-24h-23 raw.jpg]

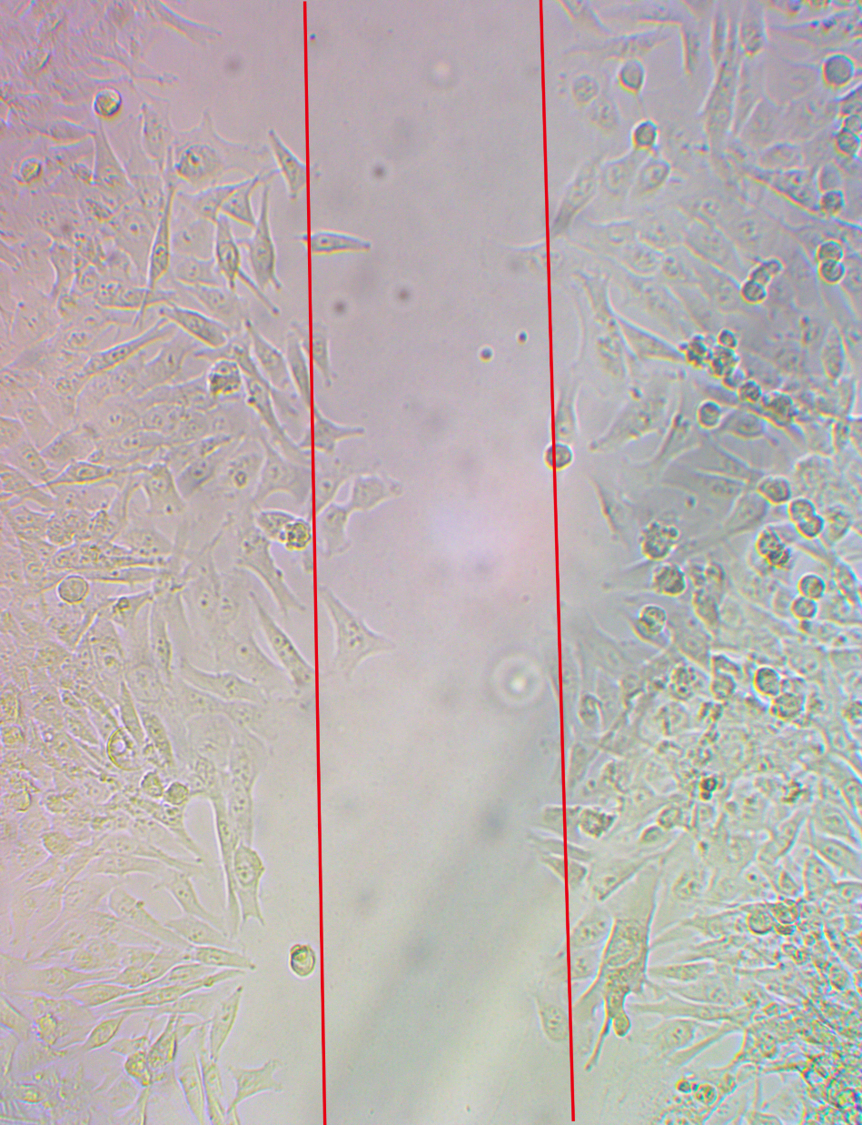

Supplement: Supplementary file 4 [file DataSheet9.ZIP › Fig2-Wound-healing assay-sk hep1-plc5/24H/plc5-24h-23.jpg]

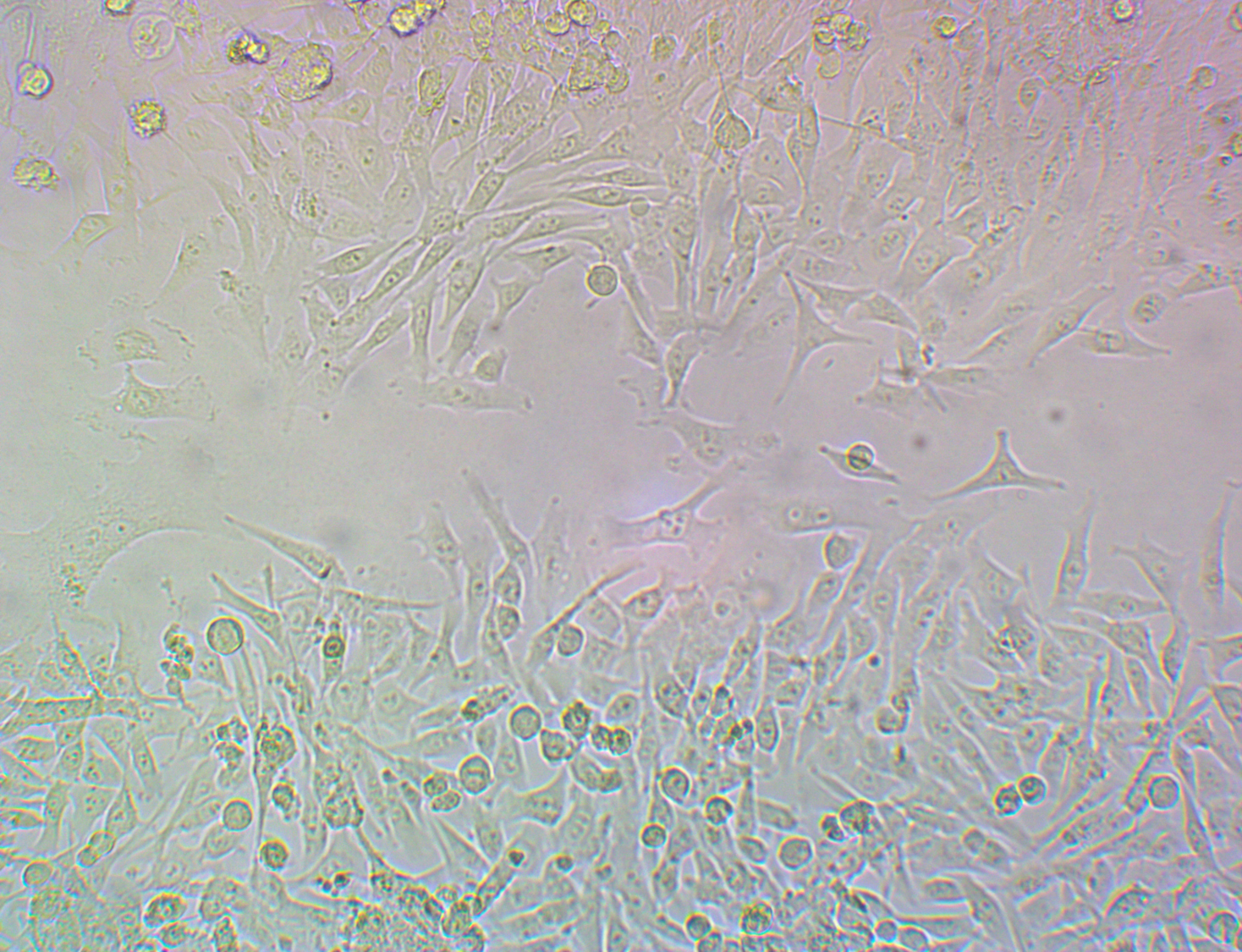

Supplement: Supplementary file 4 [file DataSheet9.ZIP › Fig2-Wound-healing assay-sk hep1-plc5/48H/plc5-48h-16 raw.jpg]

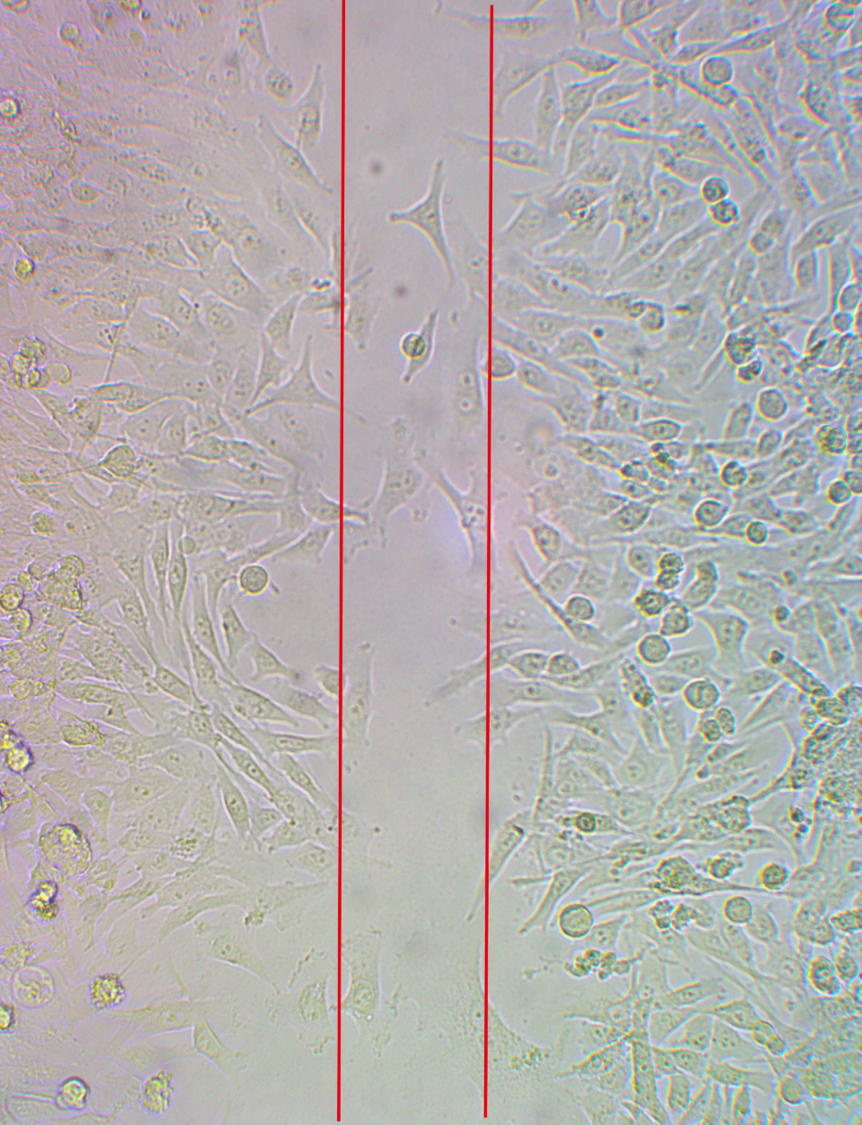

Supplement: Supplementary file 4 [file DataSheet9.ZIP › Fig2-Wound-healing assay-sk hep1-plc5/48H/plc5-48h-16.jpg]

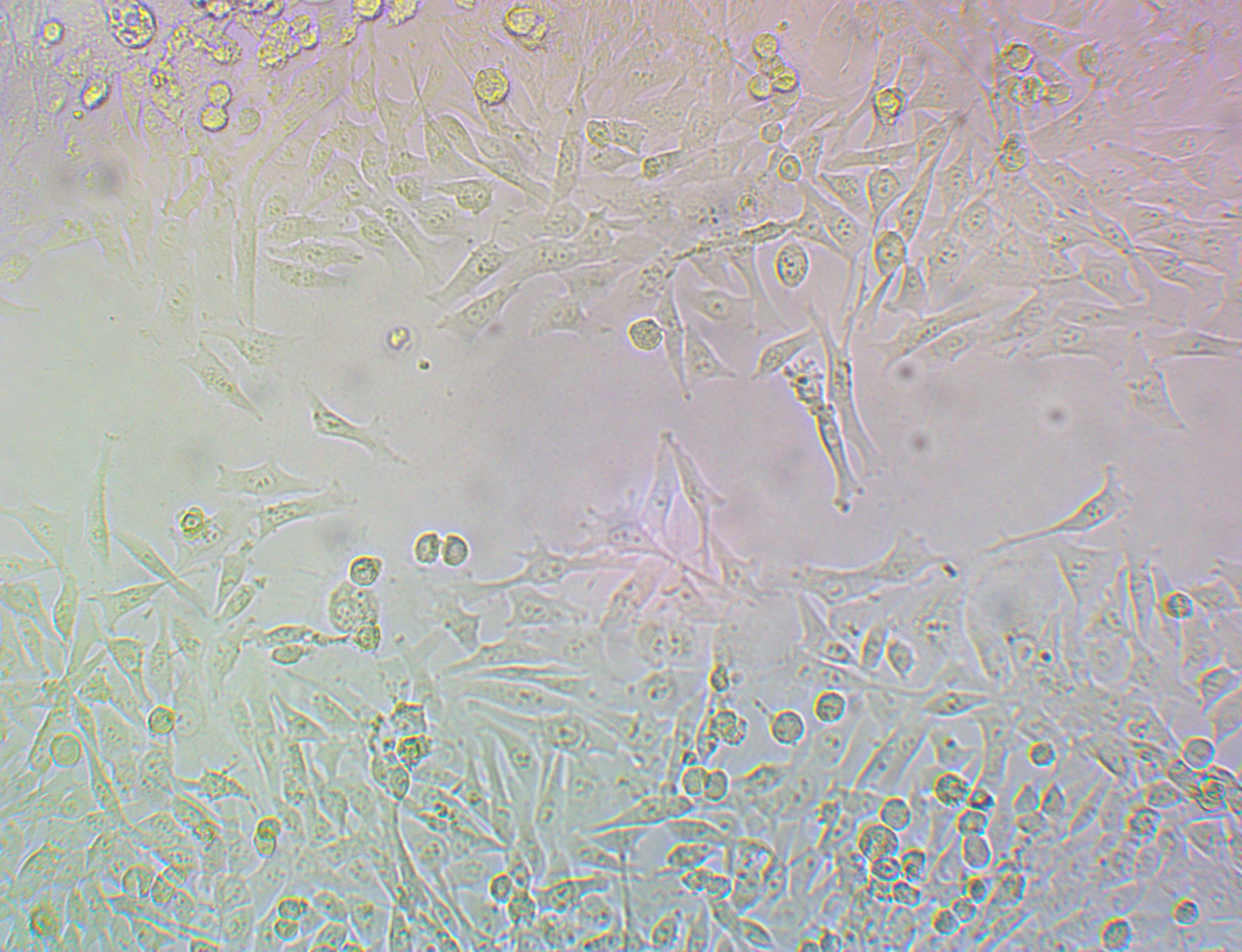

Supplement: Supplementary file 4 [file DataSheet9.ZIP › Fig2-Wound-healing assay-sk hep1-plc5/48H/plc5-48h-17 raw.jpg]

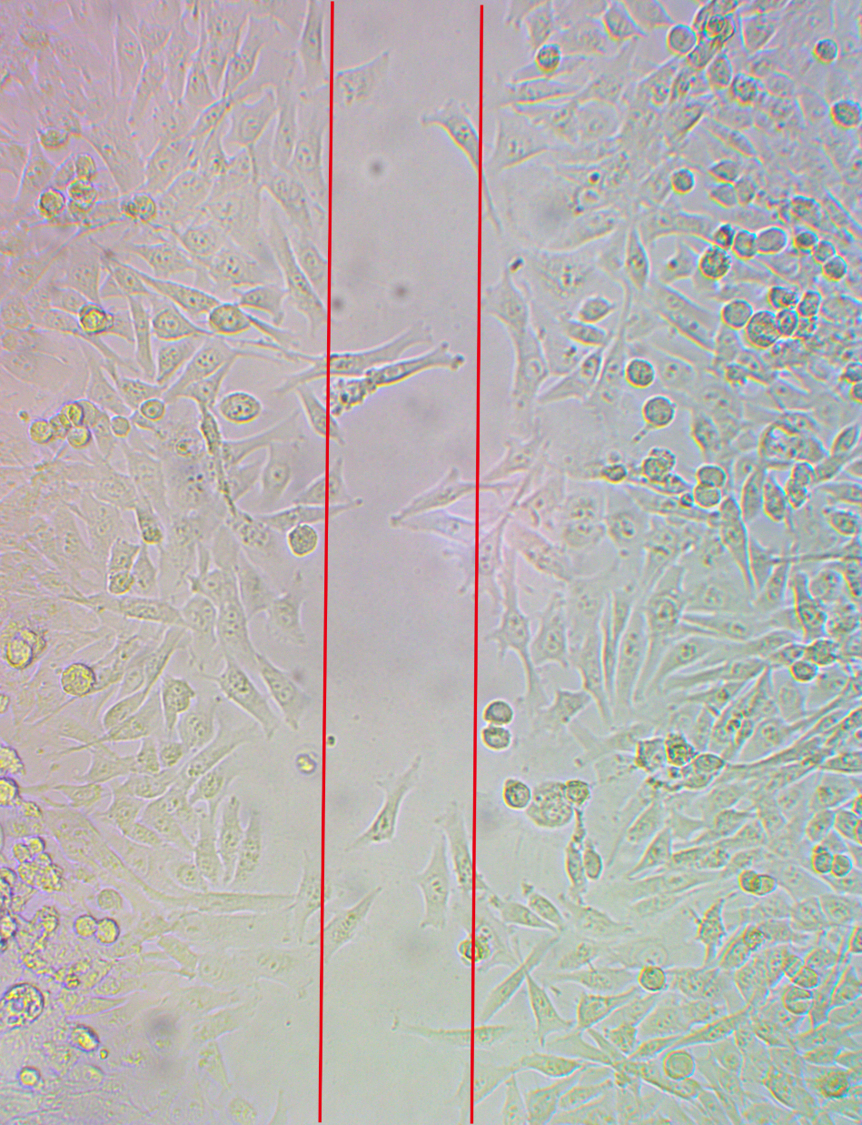

Supplement: Supplementary file 4 [file DataSheet9.ZIP › Fig2-Wound-healing assay-sk hep1-plc5/48H/plc5-48h-17.jpg]

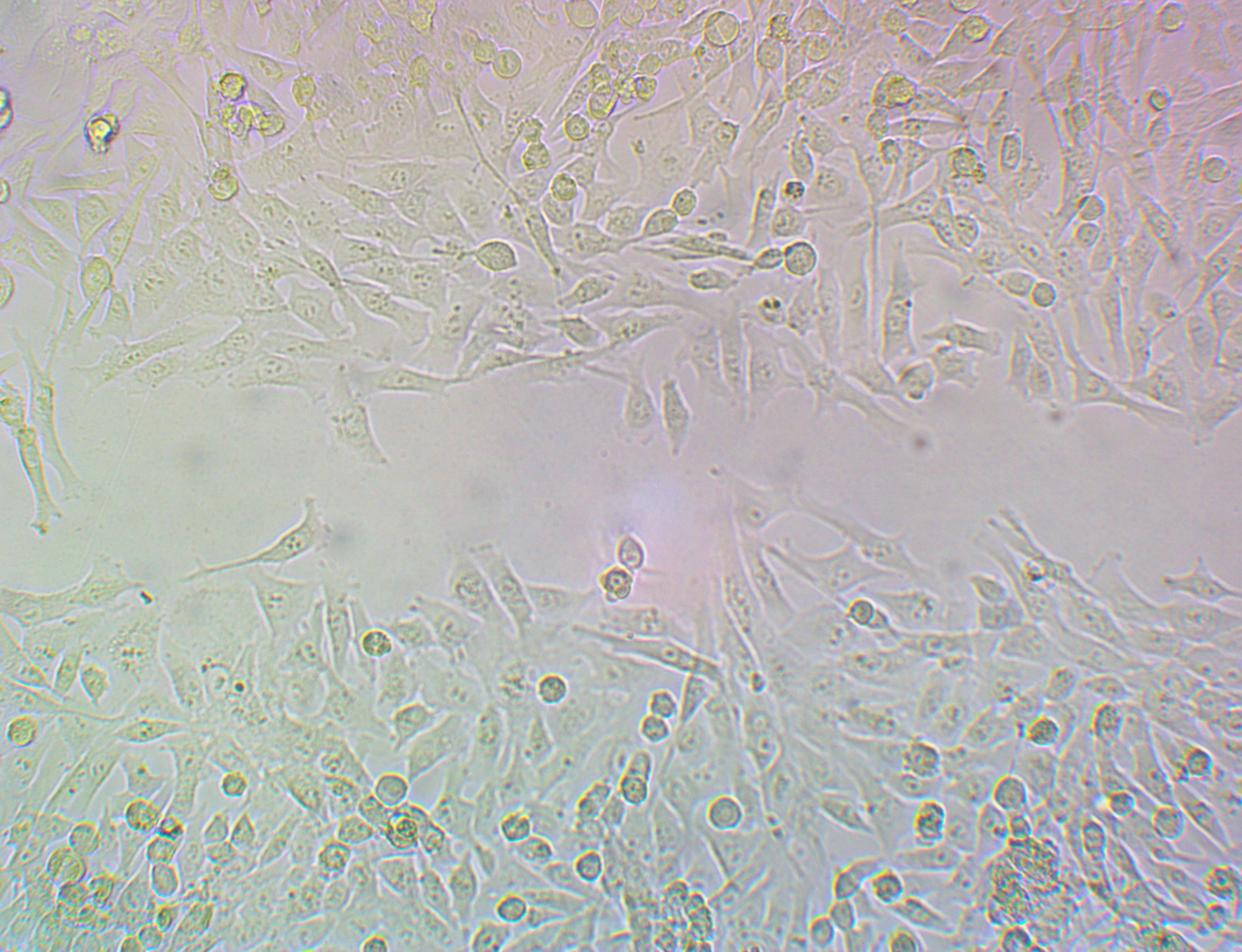

Supplement: Supplementary file 4 [file DataSheet9.ZIP › Fig2-Wound-healing assay-sk hep1-plc5/48H/plc5-48h-18 raw.jpg]

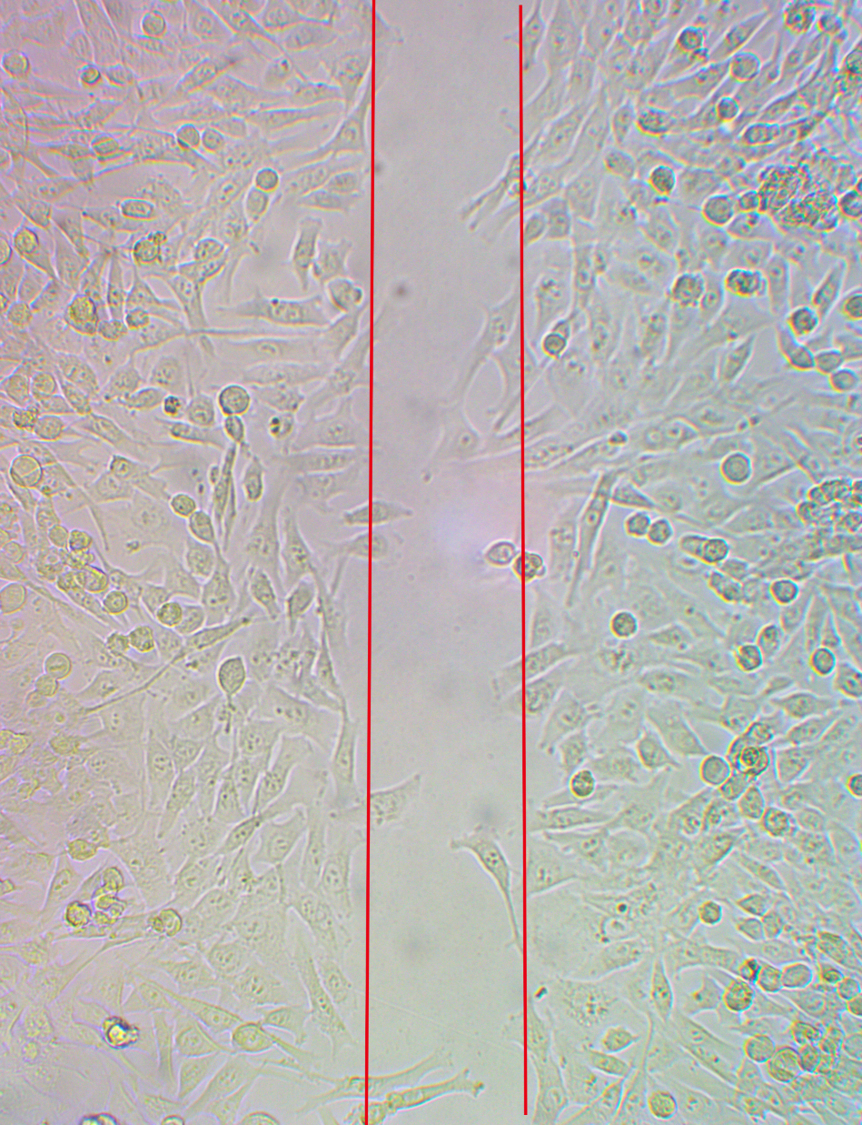

Supplement: Supplementary file 4 [file DataSheet9.ZIP › Fig2-Wound-healing assay-sk hep1-plc5/48H/plc5-48h-18.jpg]

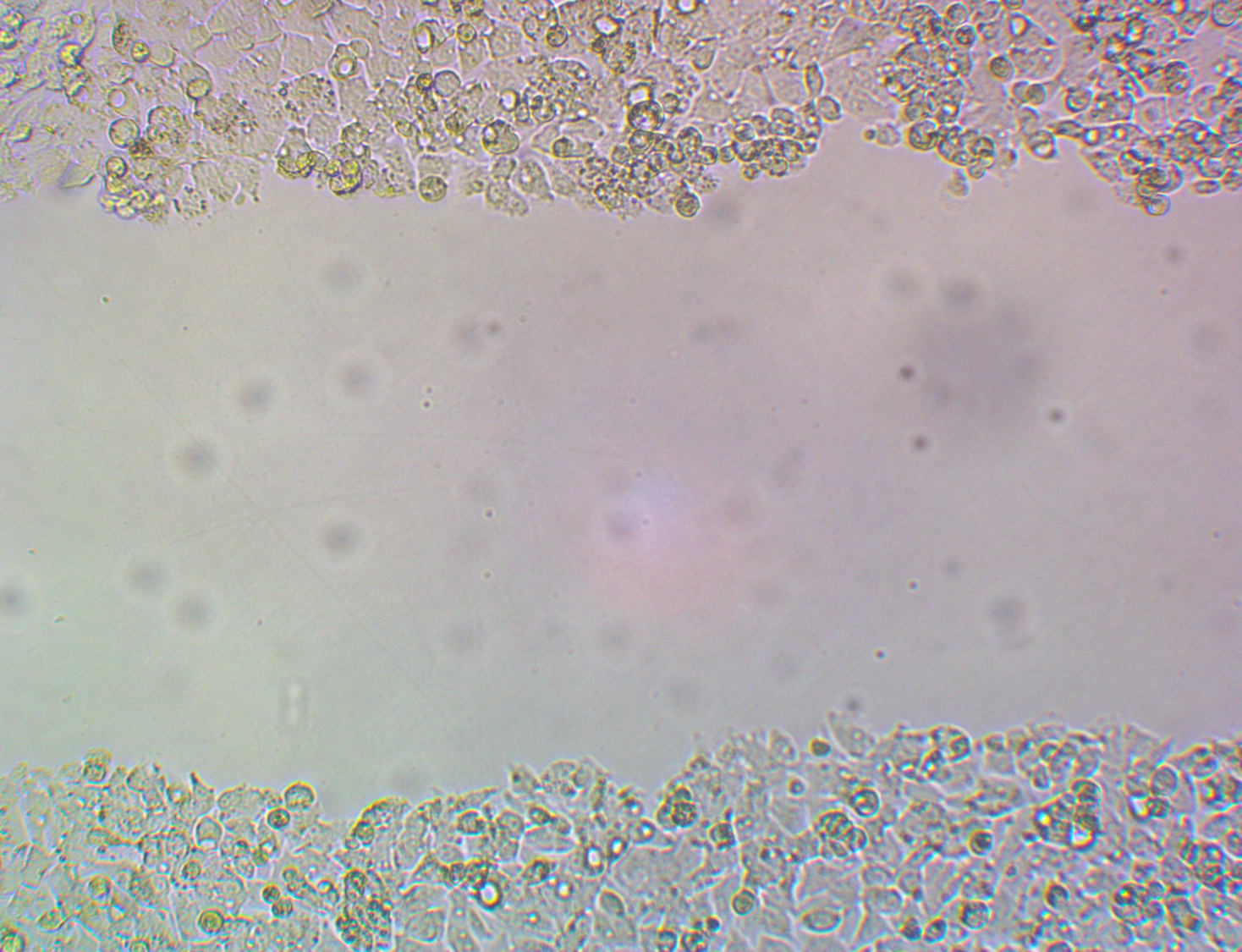

Supplement: Supplementary file 6 [file DataSheet4.ZIP › 0H/72309-0h-1 raw.jpg]

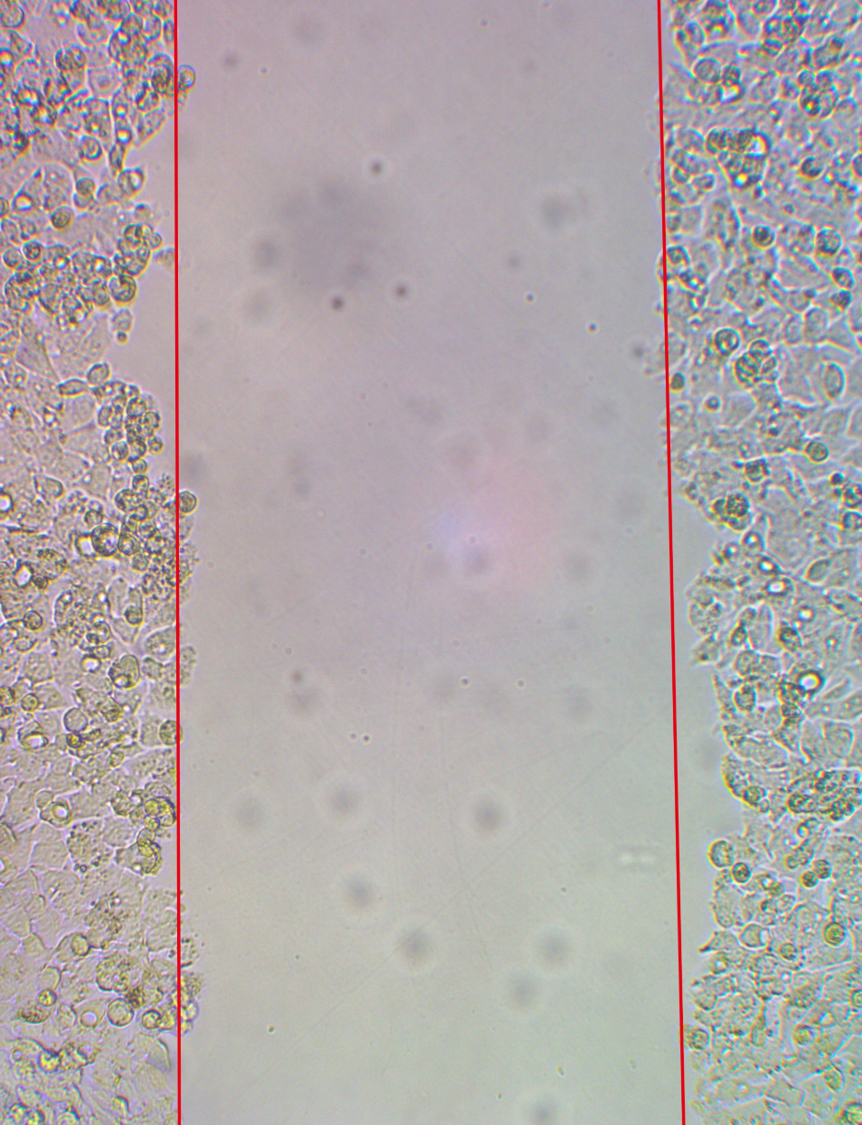

Supplement: Supplementary file 6 [file DataSheet4.ZIP › 0H/72309-0h-1-5.jpg]

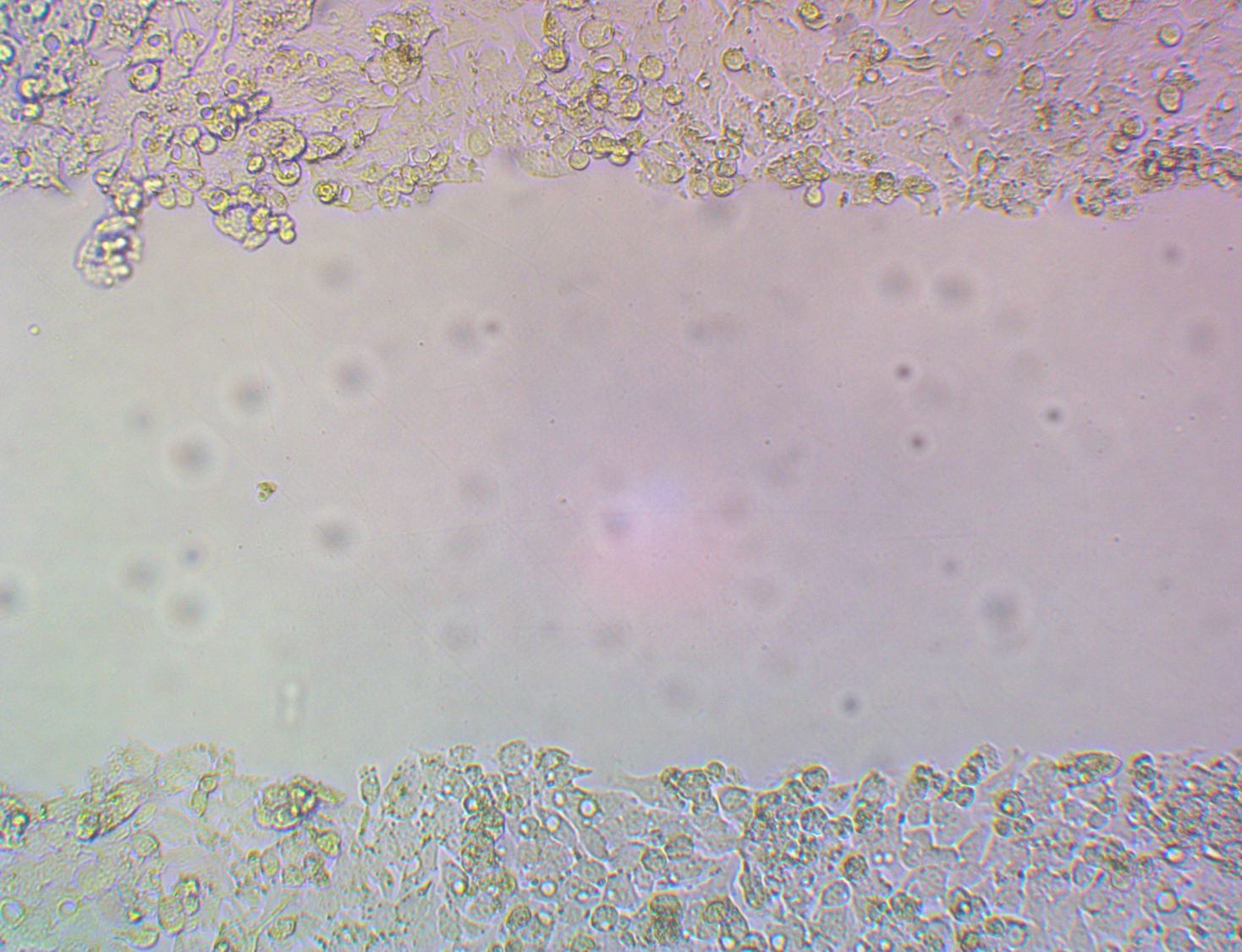

Supplement: Supplementary file 6 [file DataSheet4.ZIP › 0H/72309-0h-4 raw.jpg]

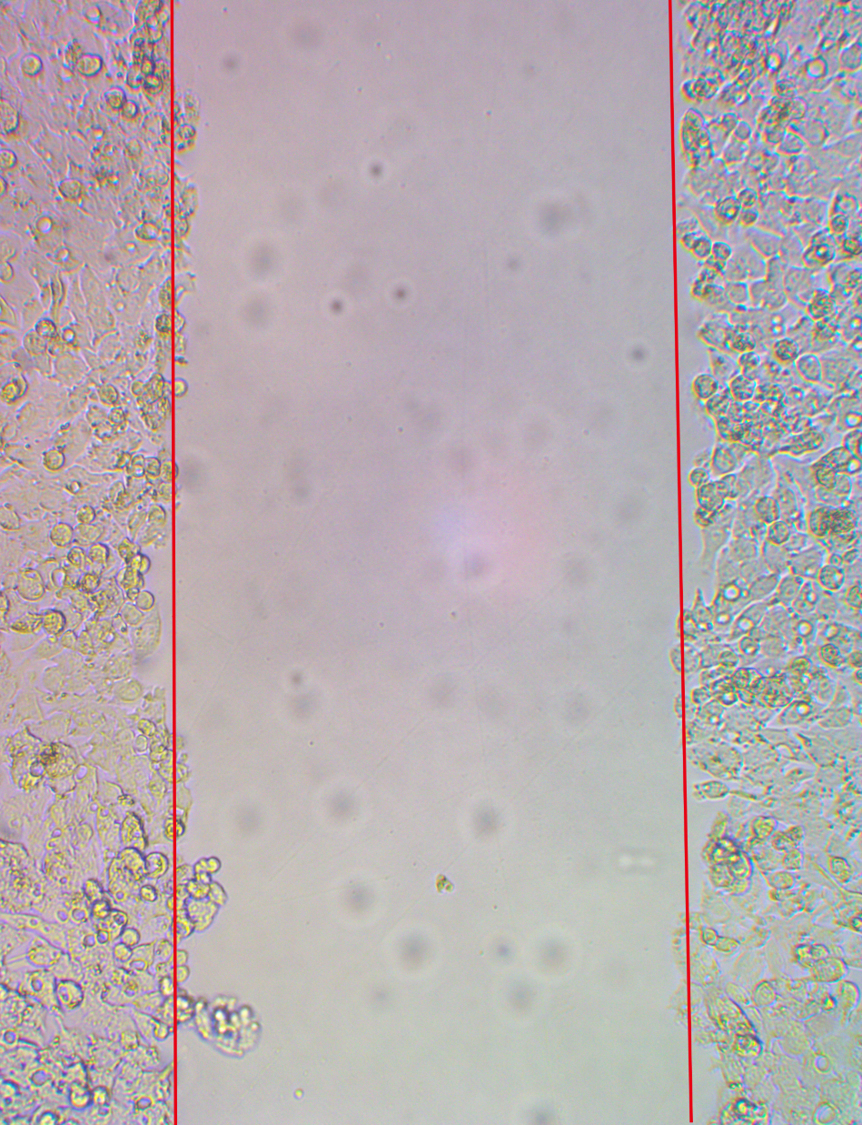

Supplement: Supplementary file 6 [file DataSheet4.ZIP › 0H/72309-0h-4-56.2.jpg]

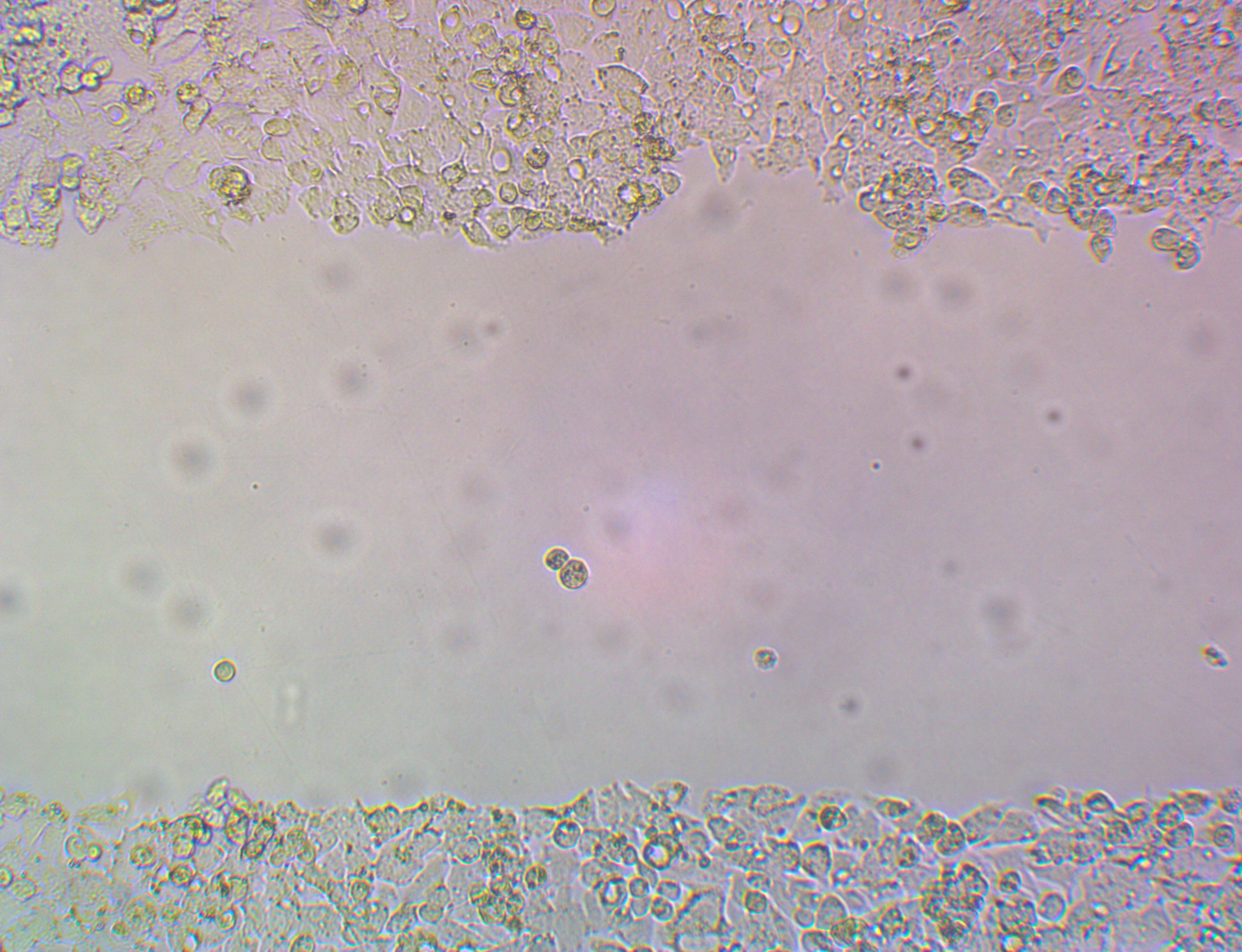

Supplement: Supplementary file 6 [file DataSheet4.ZIP › 0H/72309-0h-5 raw.jpg]

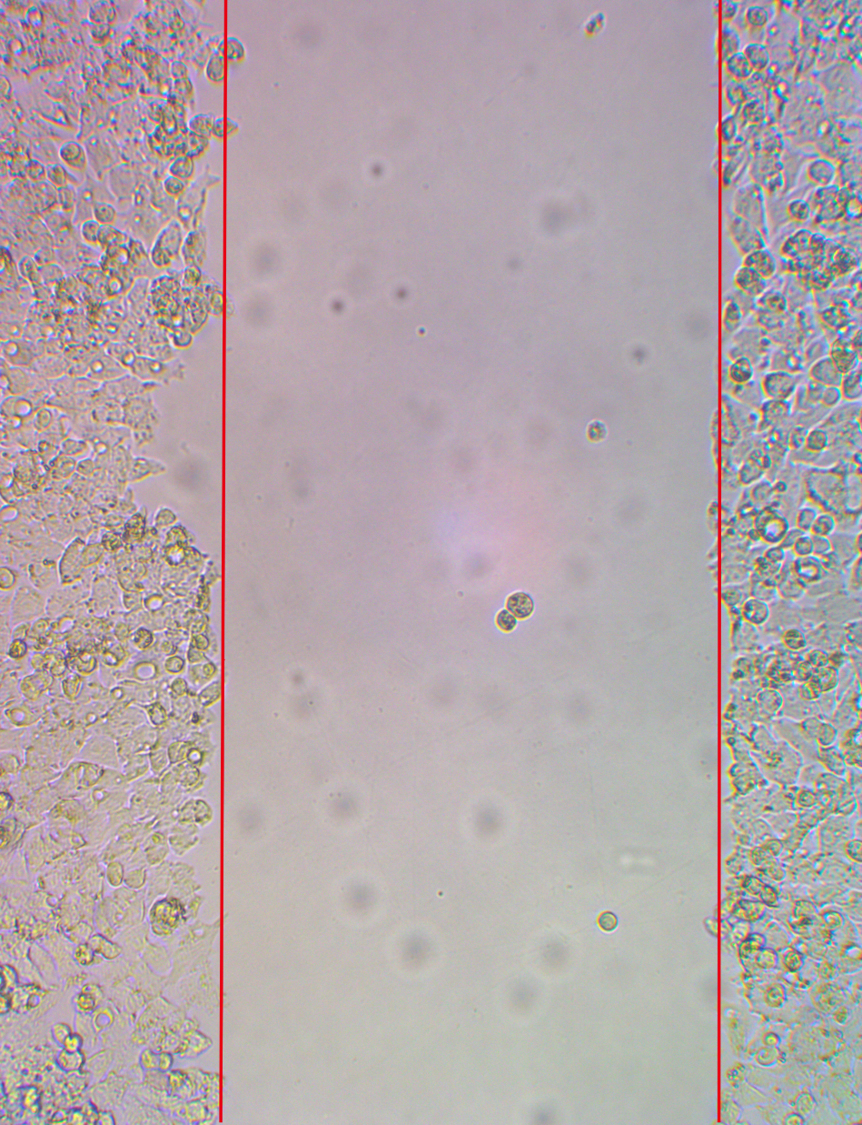

Supplement: Supplementary file 6 [file DataSheet4.ZIP › 0H/72309-0h-5-55.jpg]

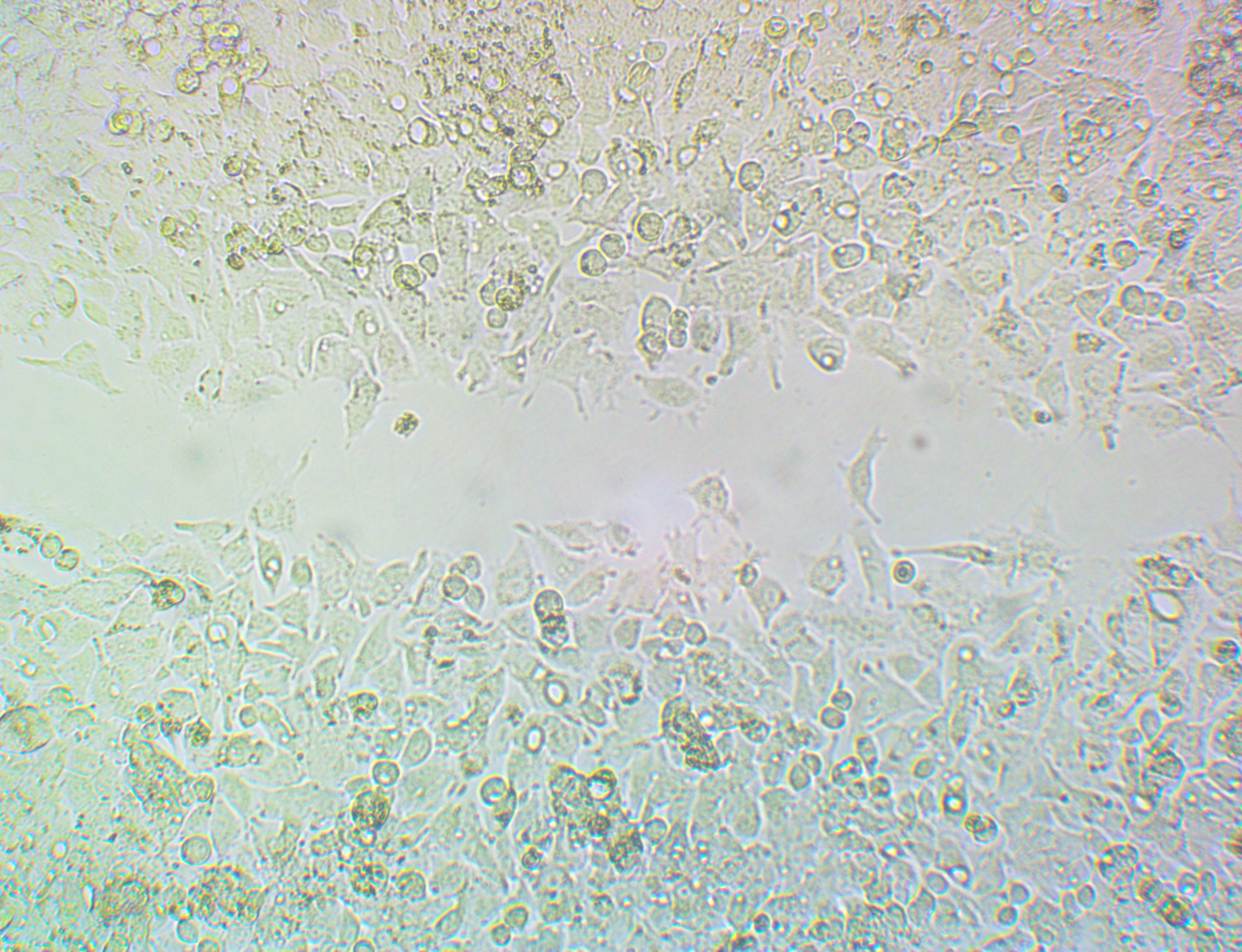

Supplement: Supplementary file 6 [file DataSheet4.ZIP › 24H/72309-24h-3 raw.jpg]

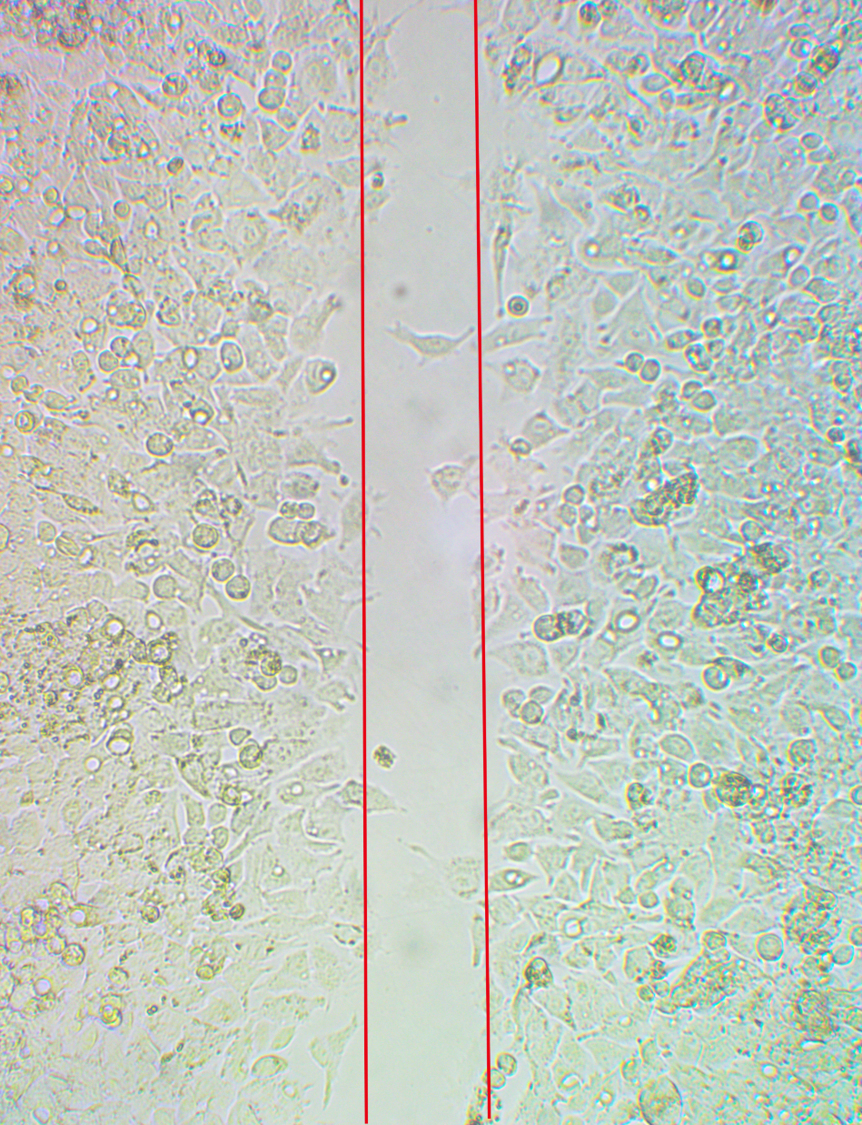

Supplement: Supplementary file 6 [file DataSheet4.ZIP › 24H/72309-24h-3.jpg]

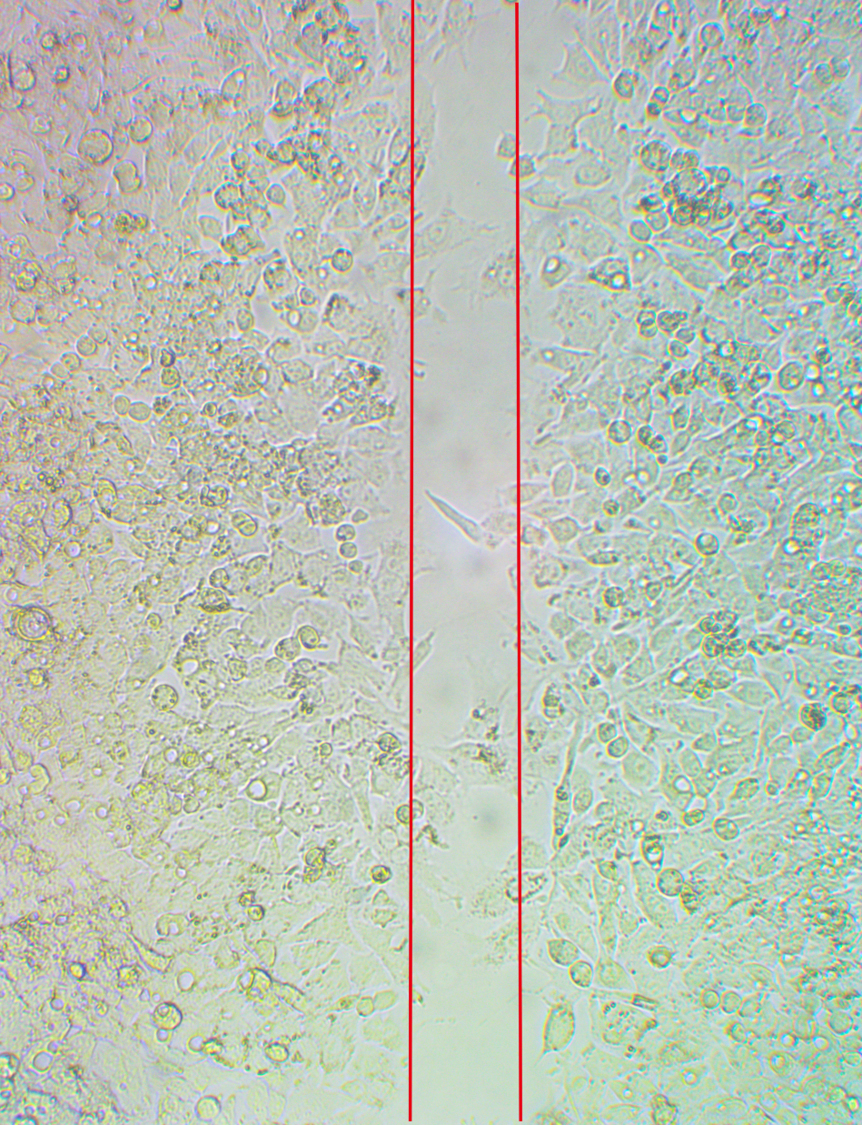

Supplement: Supplementary file 6 [file DataSheet4.ZIP › 24H/72309-24h-5 .jpg]

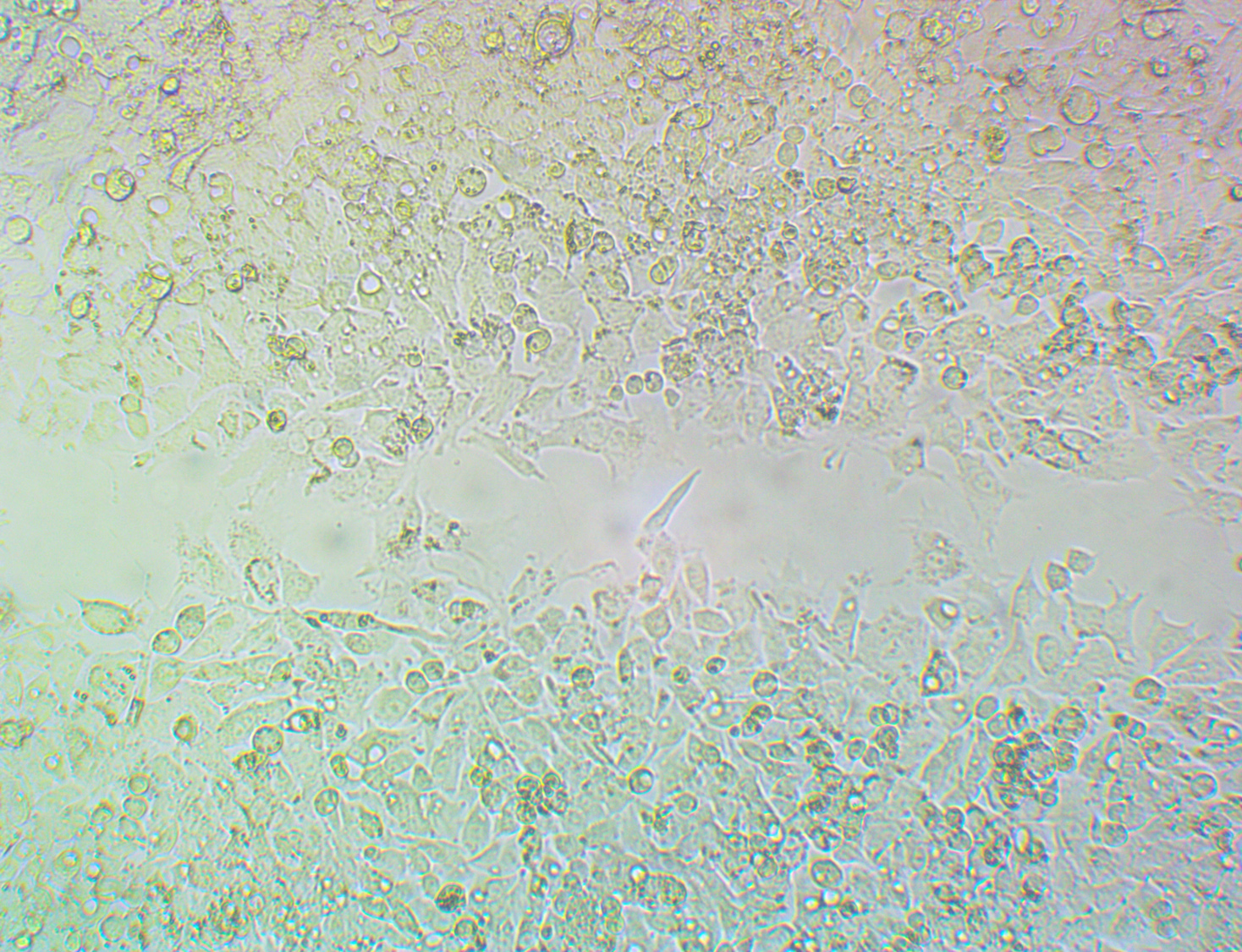

Supplement: Supplementary file 6 [file DataSheet4.ZIP › 24H/72309-24h-5 raw.jpg]

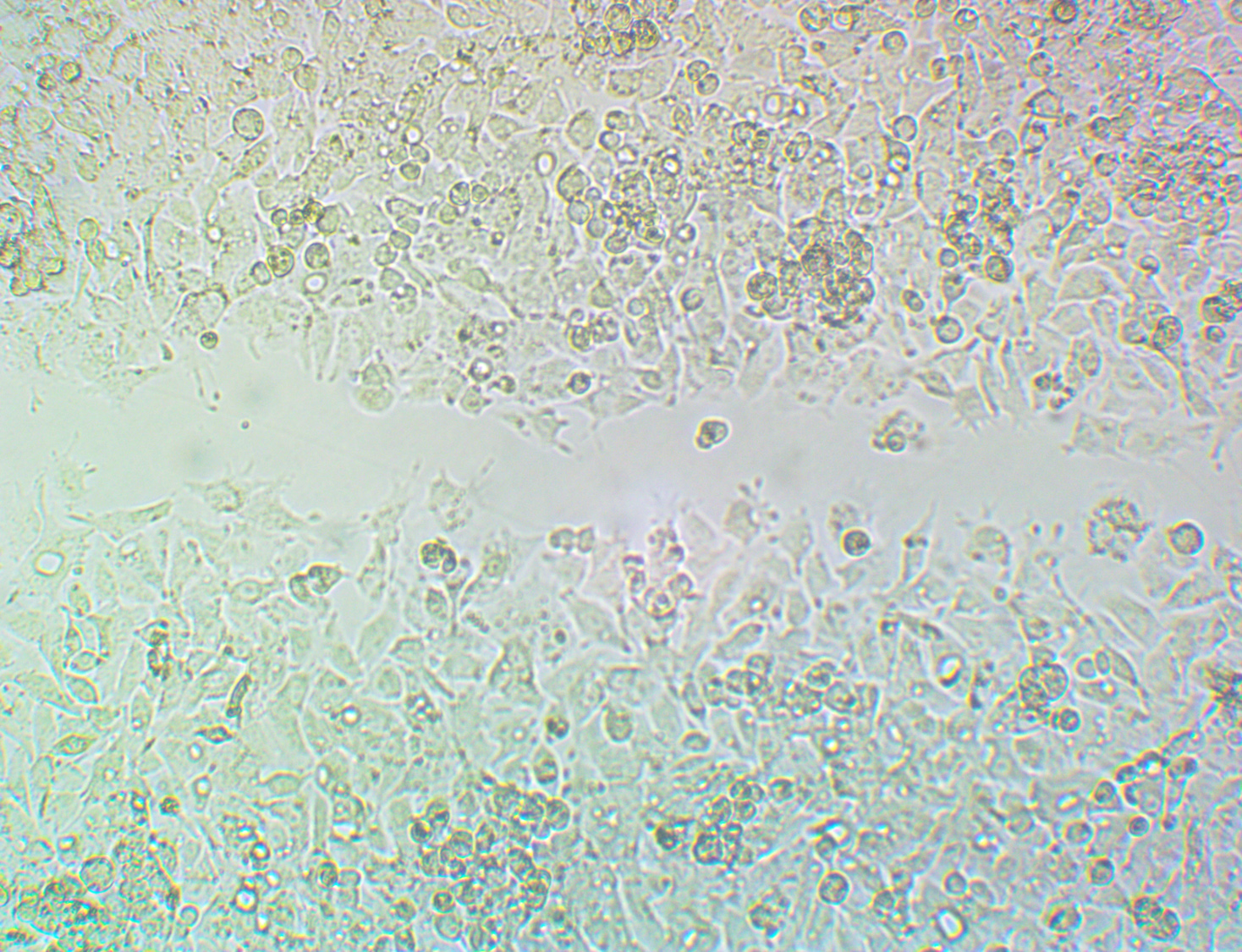

Supplement: Supplementary file 6 [file DataSheet4.ZIP › 24H/72309-24h-8 raw.jpg]

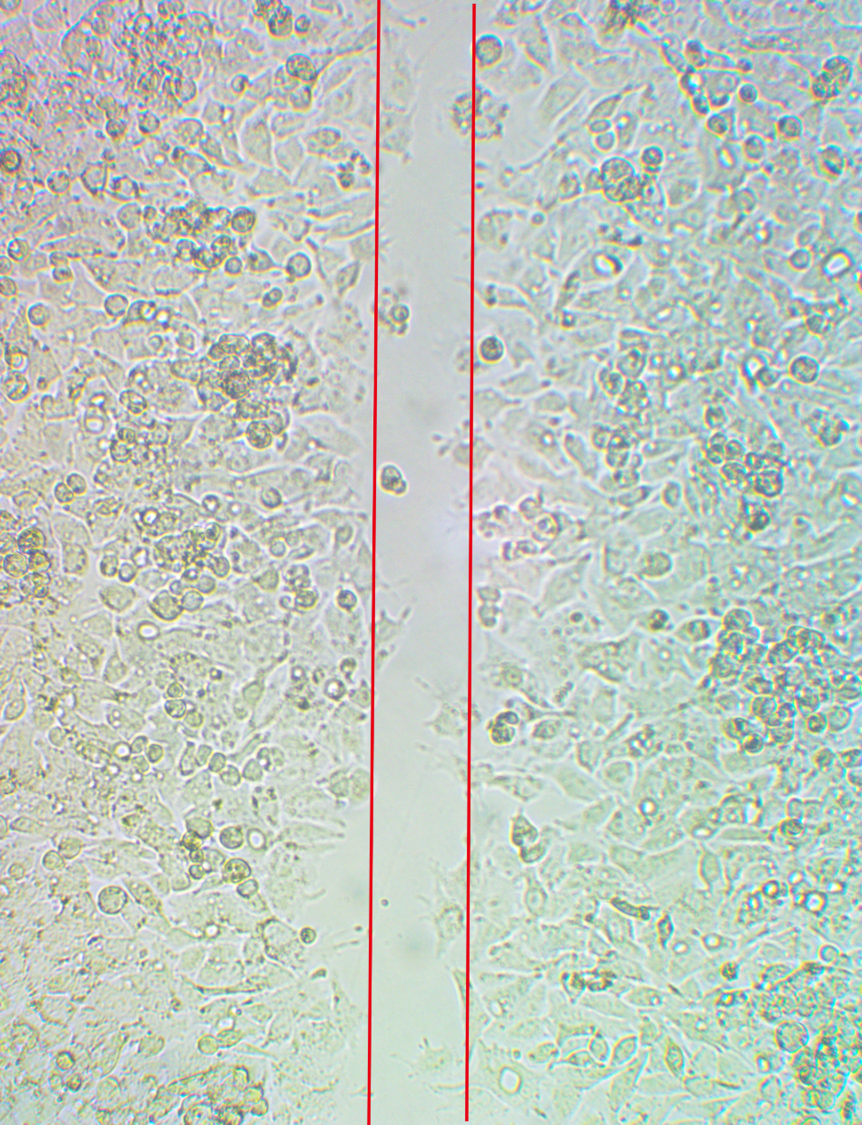

Supplement: Supplementary file 6 [file DataSheet4.ZIP › 24H/72309-24h-8-12.jpg]

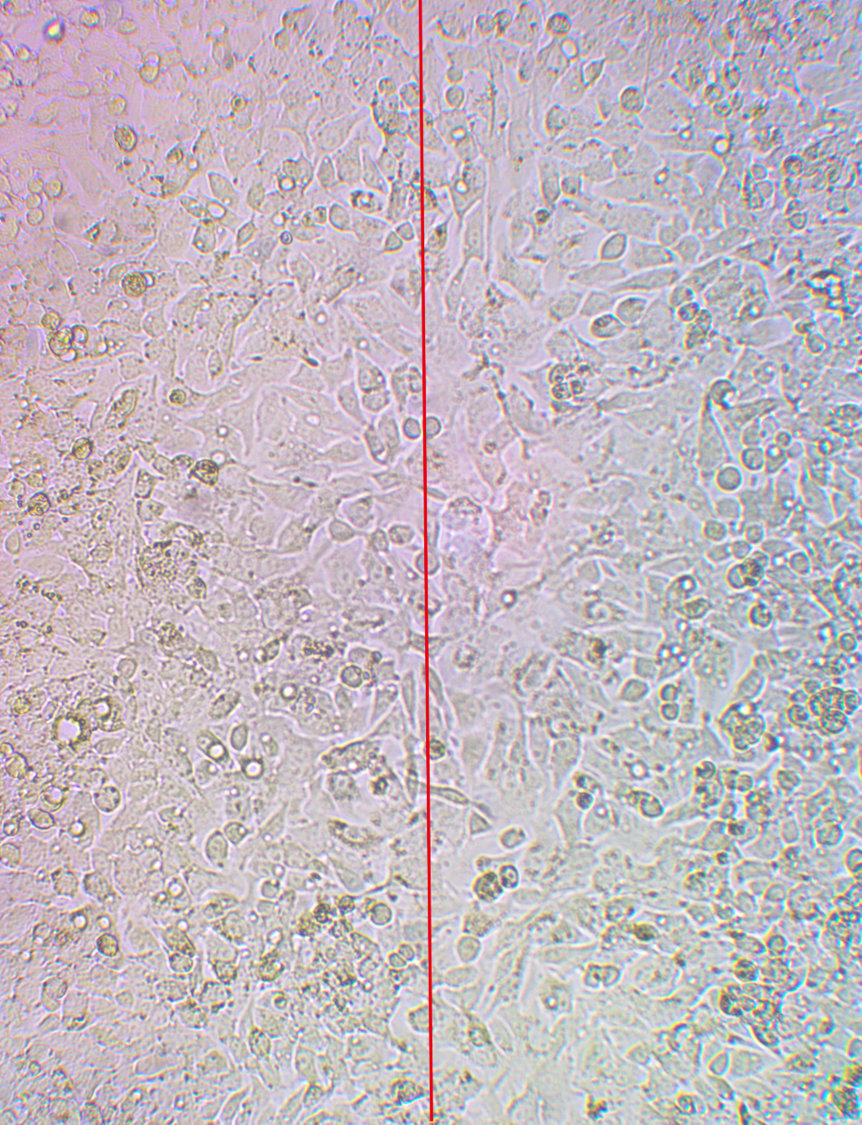

Supplement: Supplementary file 6 [file DataSheet4.ZIP › 48H/72309-48h-6 .jpg]

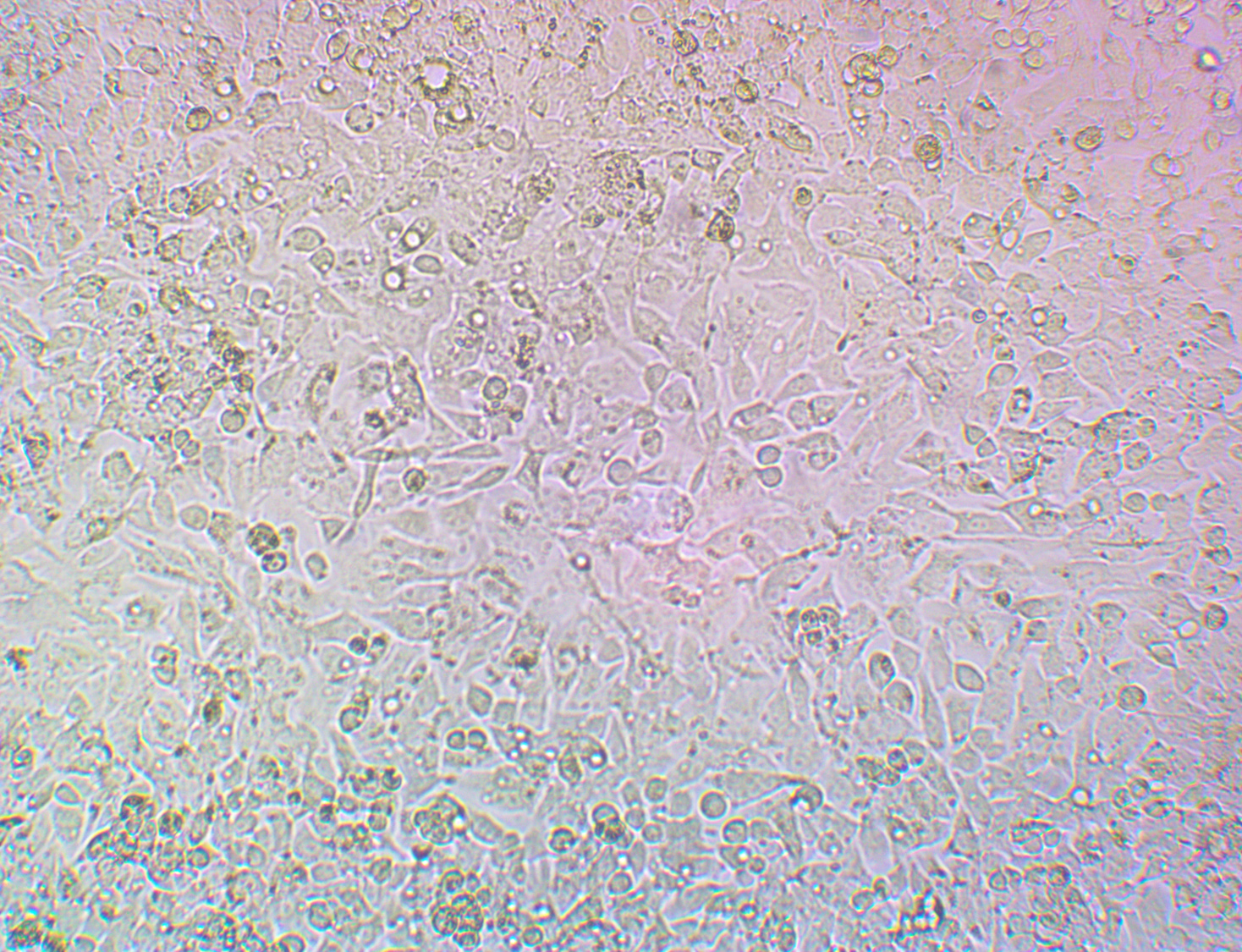

Supplement: Supplementary file 6 [file DataSheet4.ZIP › 48H/72309-48h-6 raw.jpg]

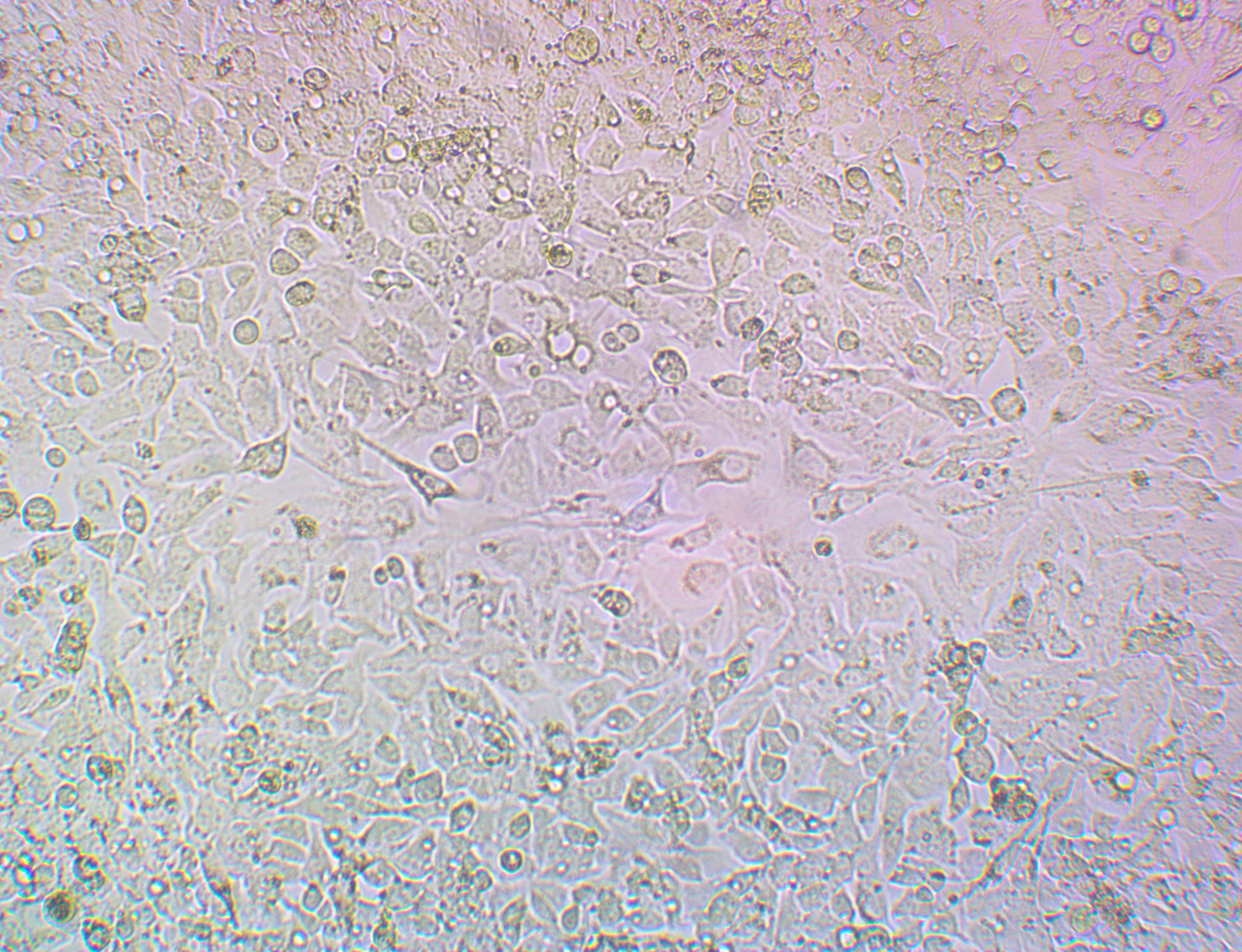

Supplement: Supplementary file 6 [file DataSheet4.ZIP › 48H/72309-48h-7 raw.jpg]

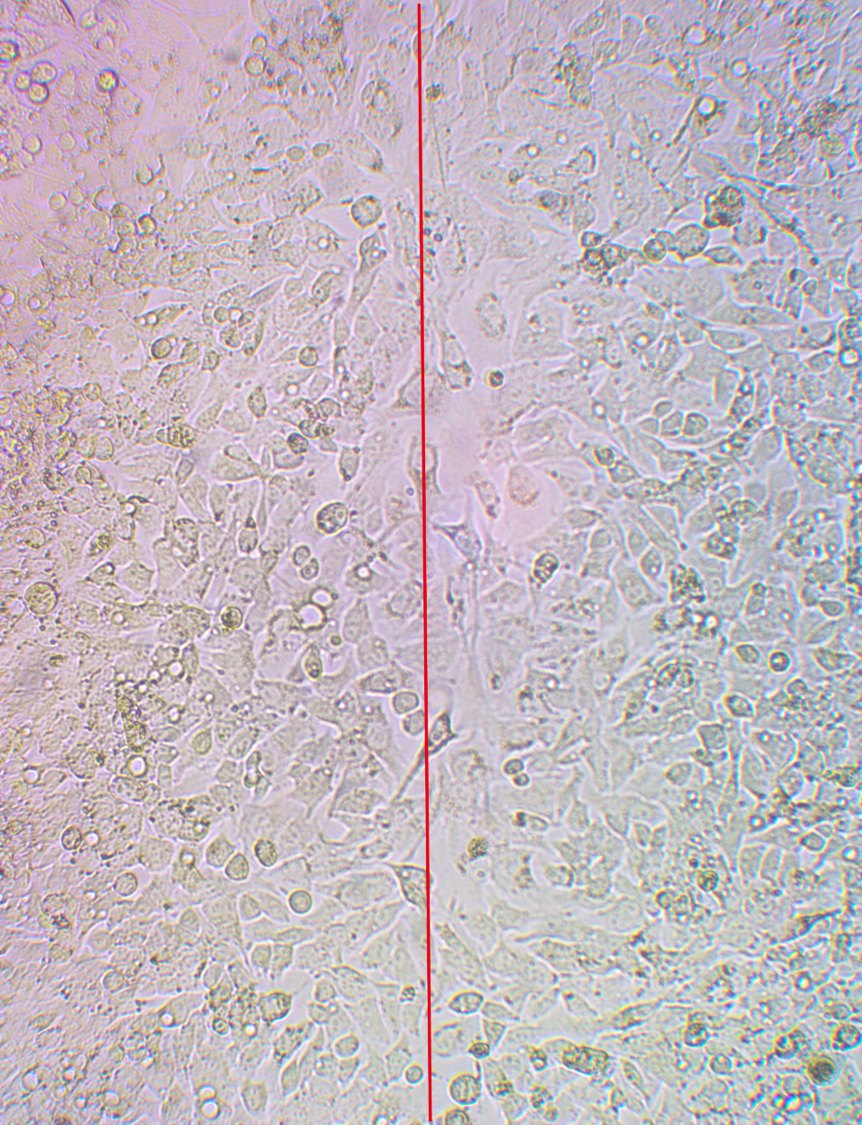

Supplement: Supplementary file 6 [file DataSheet4.ZIP › 48H/72309-48h-7.jpg]

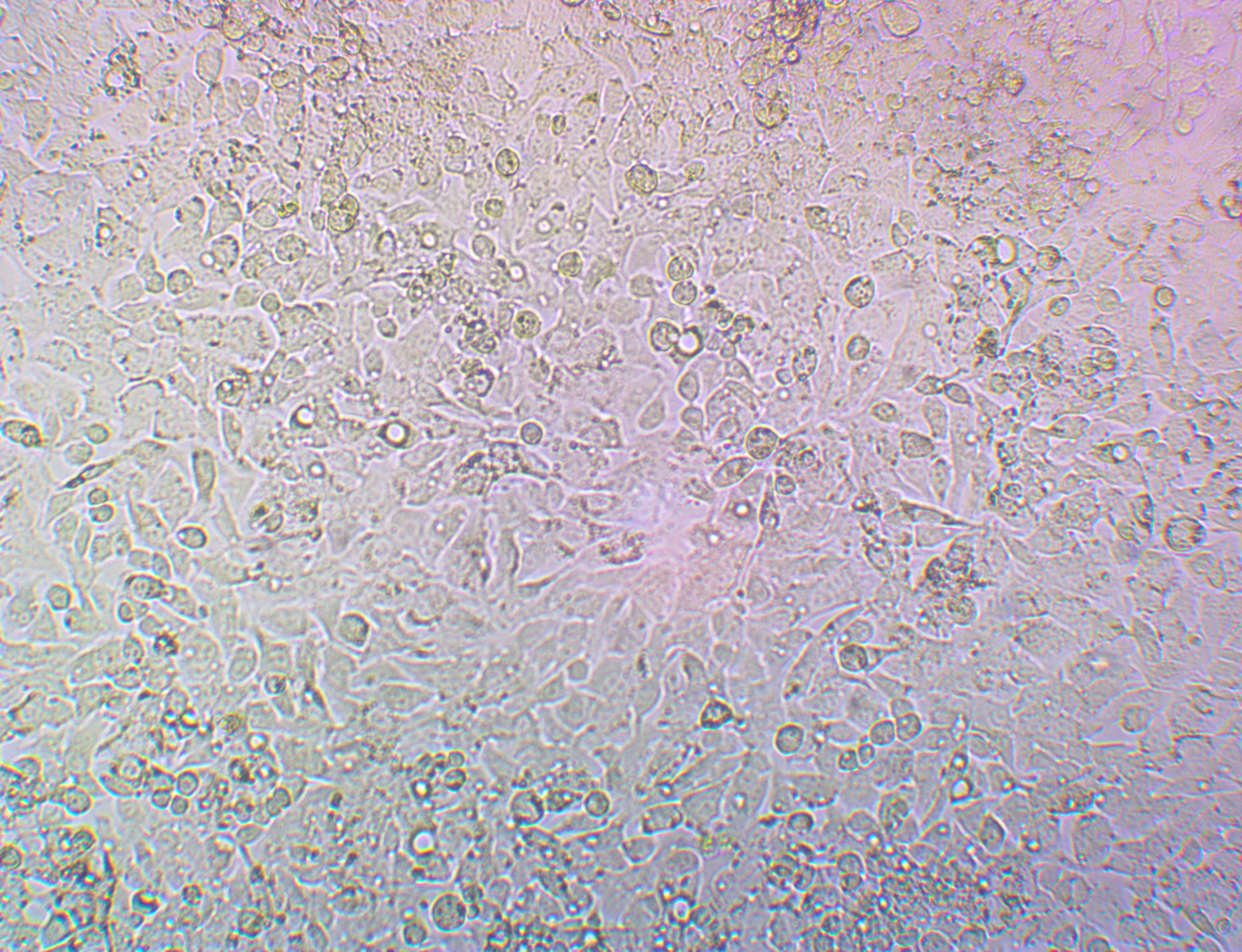

Supplement: Supplementary file 6 [file DataSheet4.ZIP › 48H/72309-48h-9 raw.jpg]

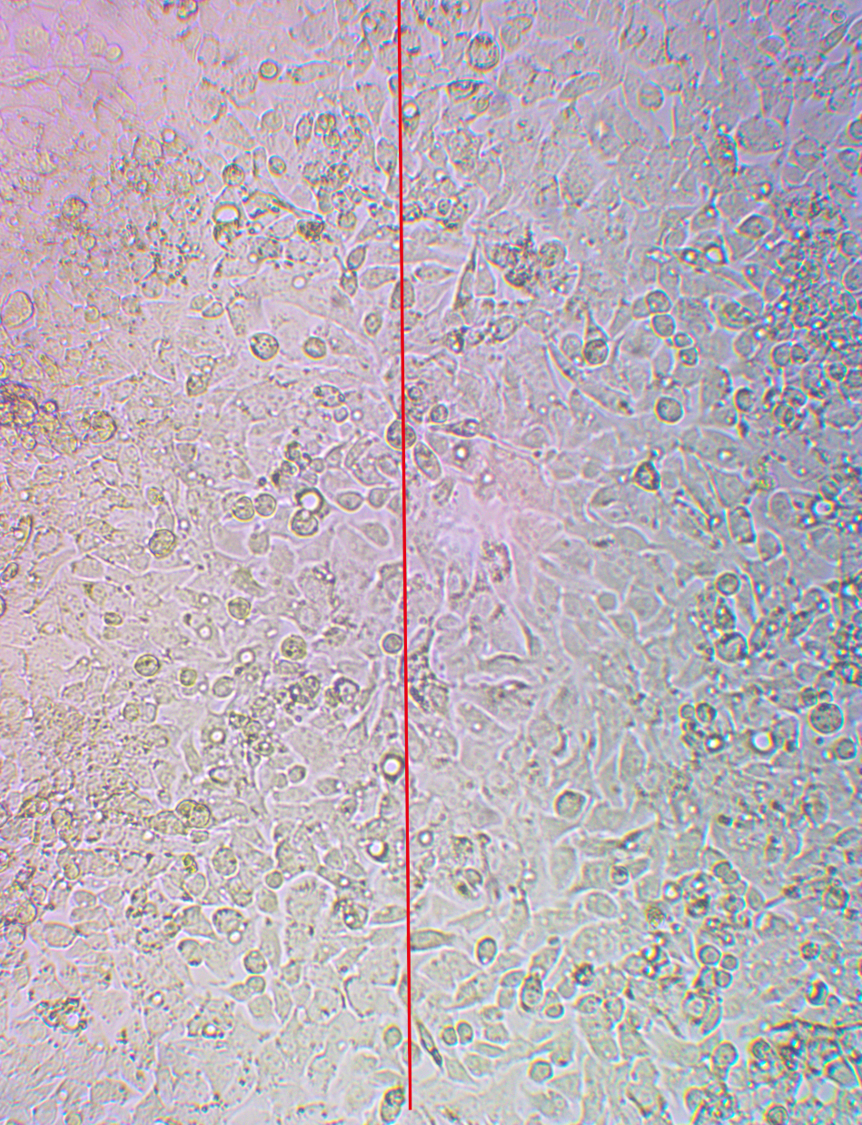

Supplement: Supplementary file 6 [file DataSheet4.ZIP › 48H/72309-48h-9.jpg]

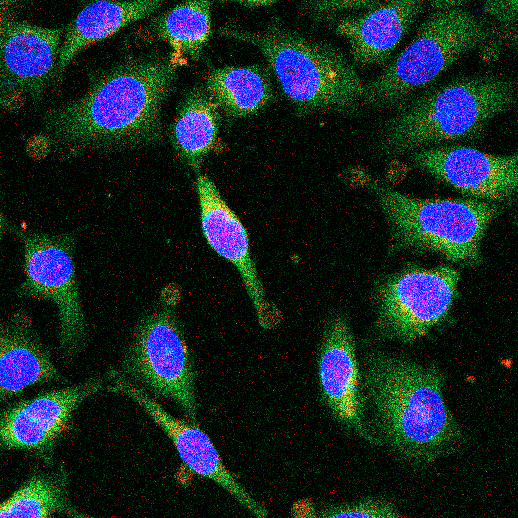

Supplement: Supplementary file 7 [file DataSheet13.ZIP › No Scale/LO2 (No Scale)/l10-40-单个文件导出-04_c1+2+3.tif]

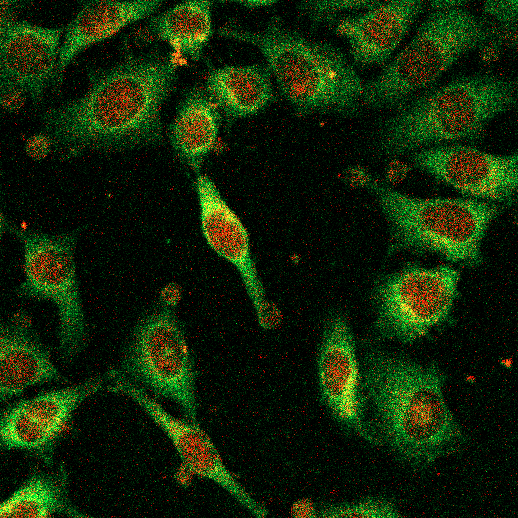

Supplement: Supplementary file 7 [file DataSheet13.ZIP › No Scale/LO2 (No Scale)/l10-40-单个文件导出-04_c1+2.tif]

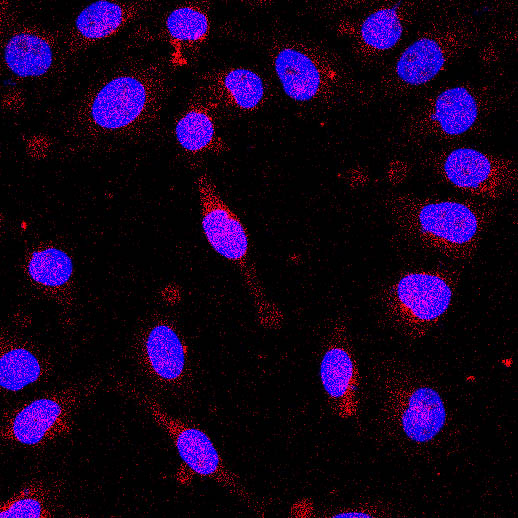

Supplement: Supplementary file 7 [file DataSheet13.ZIP › No Scale/LO2 (No Scale)/l10-40-单个文件导出-04_c1+3.jpg]

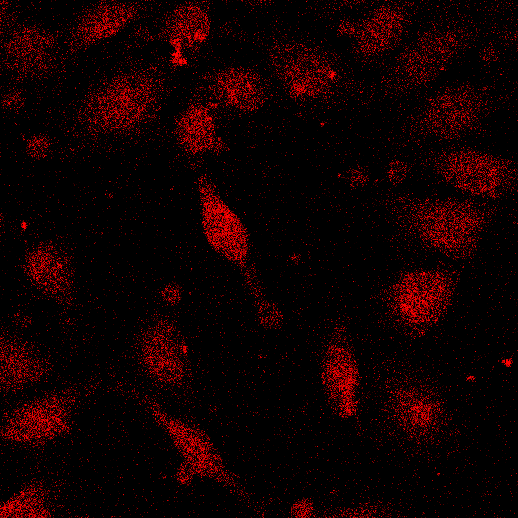

Supplement: Supplementary file 7 [file DataSheet13.ZIP › No Scale/LO2 (No Scale)/l10-40-单个文件导出-04_c1.tif]

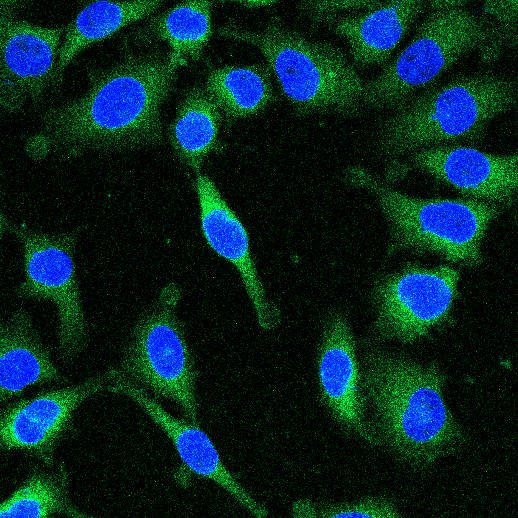

Supplement: Supplementary file 7 [file DataSheet13.ZIP › No Scale/LO2 (No Scale)/l10-40-单个文件导出-04_c2+3.tif]

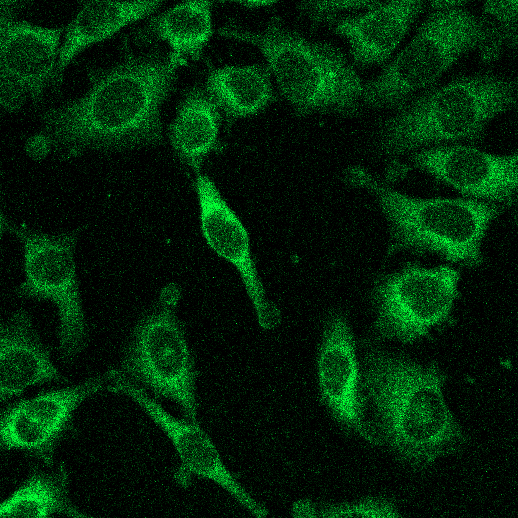

Supplement: Supplementary file 7 [file DataSheet13.ZIP › No Scale/LO2 (No Scale)/l10-40-单个文件导出-04_c2.tif]

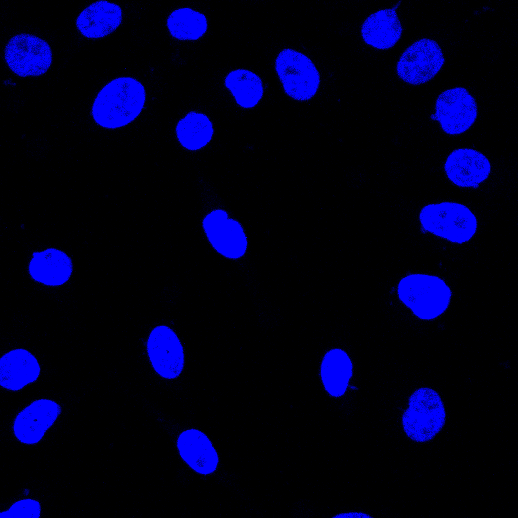

Supplement: Supplementary file 7 [file DataSheet13.ZIP › No Scale/LO2 (No Scale)/l10-40-单个文件导出-04_c3.tif]

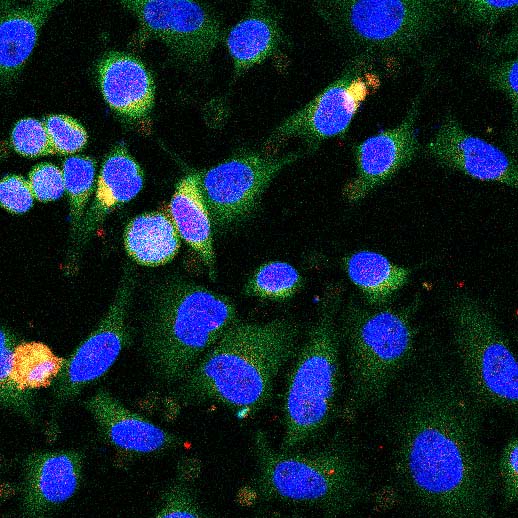

Supplement: Supplementary file 7 [file DataSheet13.ZIP › No Scale/LO2 (No Scale)/l11-40-单个文件导出-05_c1+2+3.jpg]

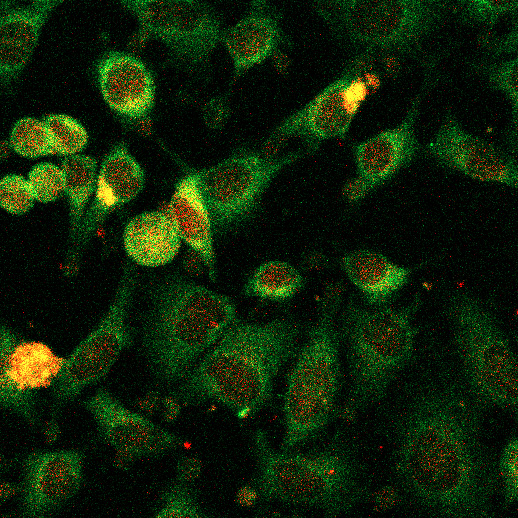

Supplement: Supplementary file 7 [file DataSheet13.ZIP › No Scale/LO2 (No Scale)/l11-40-单个文件导出-05_c1+2.jpg]
