# Supplementary material for: Downregulation of circLIFR exerts cancer-promoting effects on hepatocellular carcinoma in vitro
Source: Front Genet. 2022 Sep 12;13:986322. doi: 10.3389/fgene.2022.986322 (PMC9513674; doi:10.3389/fgene.2022.986322)

Supplementary Material

**Fig S1.** Cell Proliferation and migration assays in Hep G2 cell. (A)The proliferation of HepG2 cells transfected with control overexpression circLIFR detected by CCK-8 assay; (B)Wound-healing assay for migration ability of HepG2 cells after overexpression of circLIFR; (C) Transwell assay showed that the overexpression circLIFR promoted the matastasis and invasion of HepG2 cells; (D)The colony forming ability of HepG2 cells transfected with circLIFR in control group. *P<0.05,**P<0.01 (Student’s t-test); (E) The proliferation of HCC cells transfected with control overexpression circLIFR detected by cell cycle assay; (F) Flow cytometry used to detect the effect of overexpression of circLIFR in control group on apoptosis and renewal of HepG2 cells.


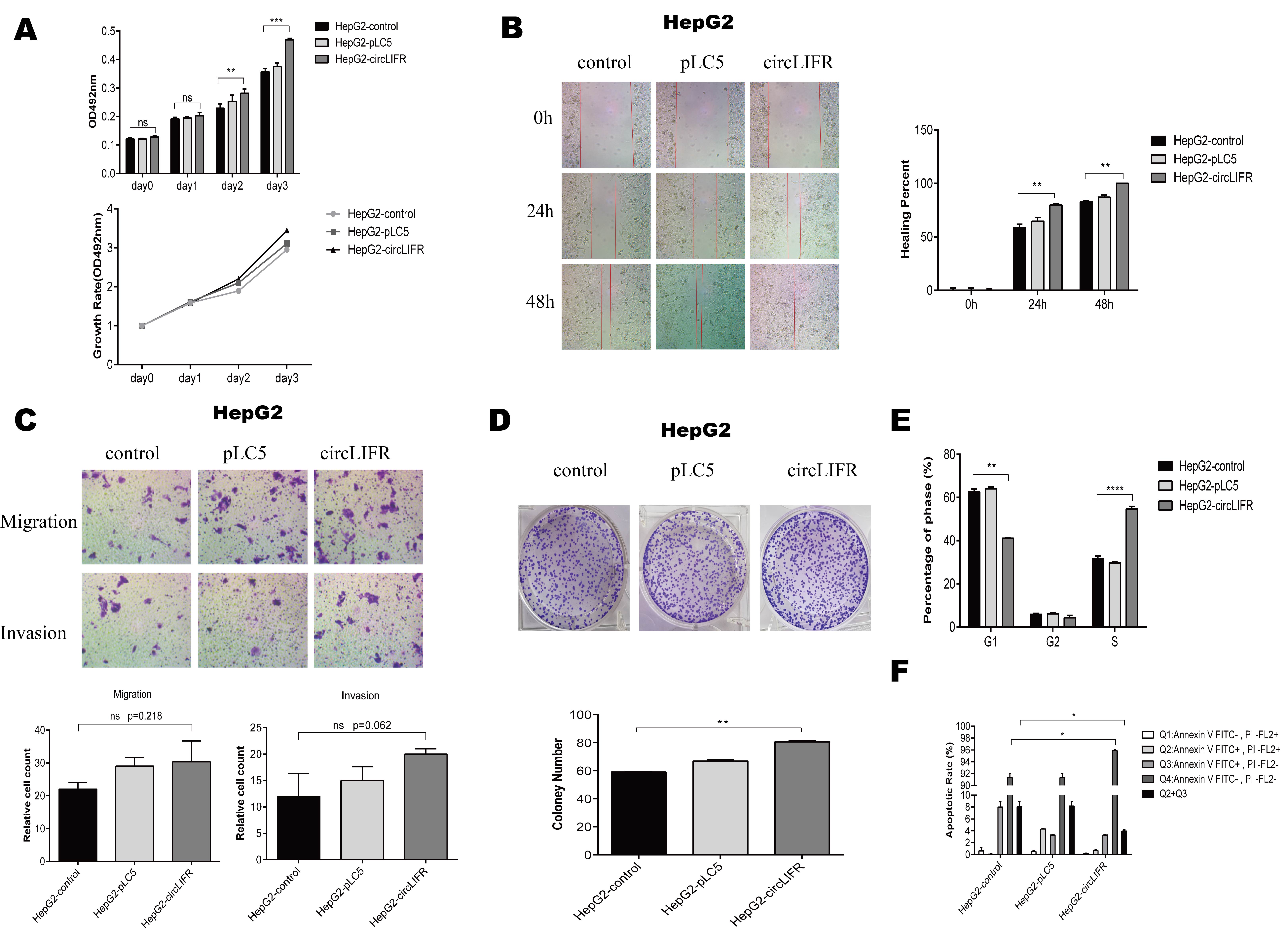


**Fig S2.** Cell cycle and Apoptosis assays in HCC cells.

(A) Cell cycle analysis in SK-Hep-1 and HepG2 cells; (B) Apoptosis assay in SK-Hep-1 and HepG2 cells.


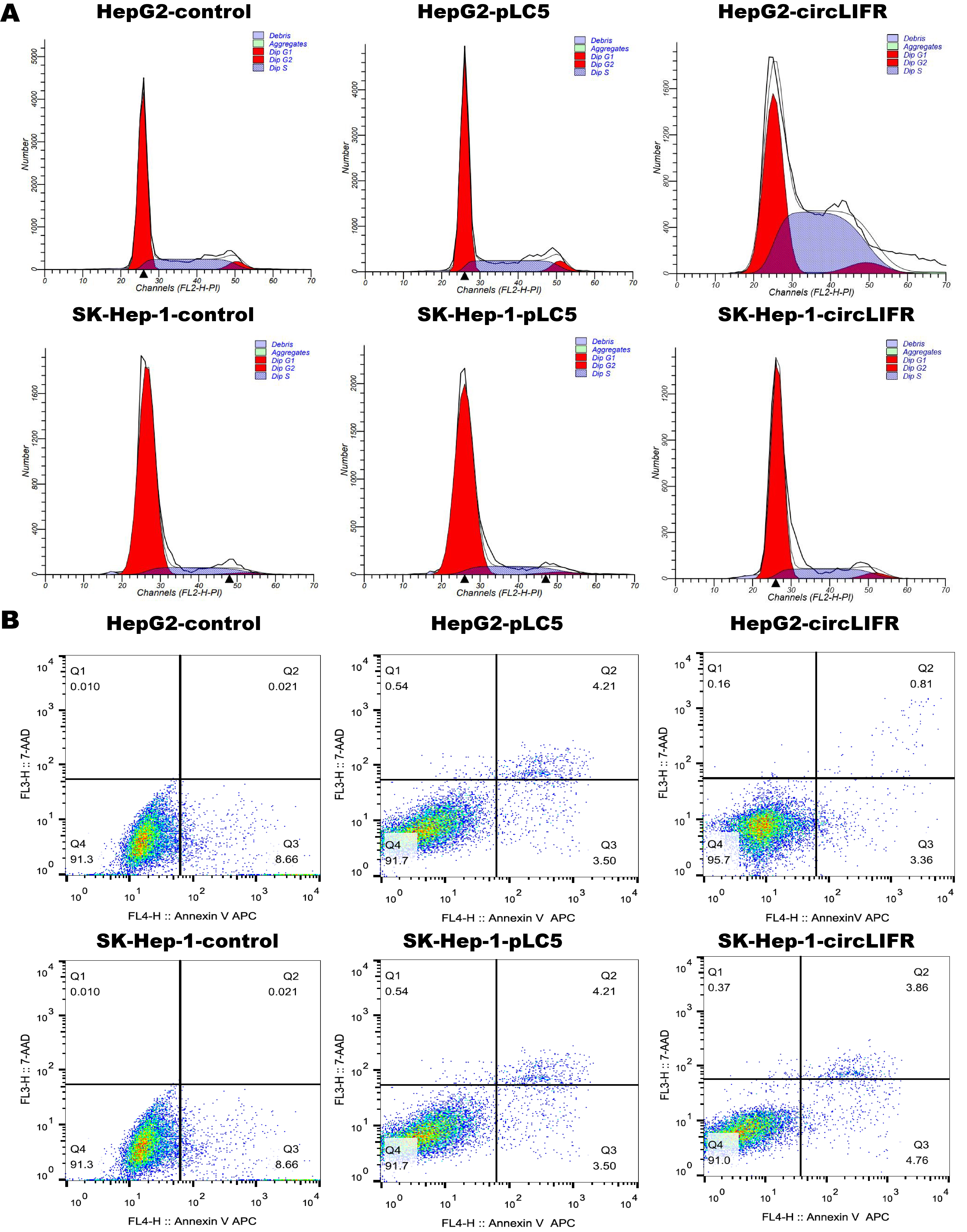

Supplement: Supplementary file 5 [file Table1.DOCX]
