# Supplementary figures and images for: Downregulation of circLIFR exerts cancer-promoting effects on hepatocellular carcinoma in vitro (part 3 of 4)
Source: Front Genet. 2022 Sep 12;13:986322. doi: 10.3389/fgene.2022.986322 (PMC9513674; doi:10.3389/fgene.2022.986322)

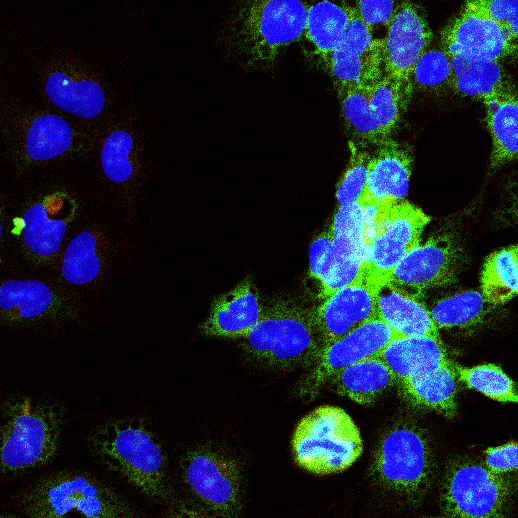

Supplement: Supplementary file 7 [file DataSheet13.ZIP › No Scale/sk-hep-1 (No Scale)/pLO5 (6)-单个文件导出-12_c1+2+3.tif]

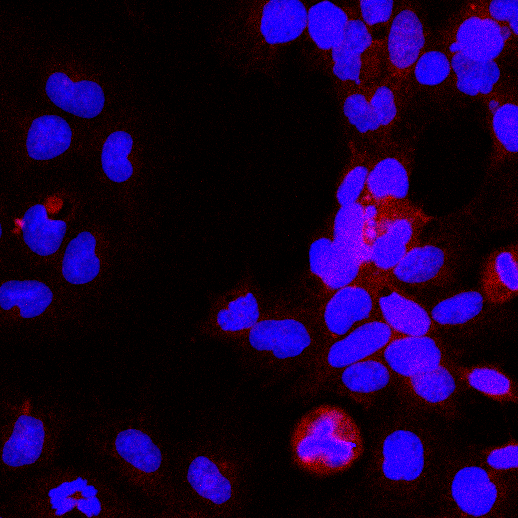

Supplement: Supplementary file 7 [file DataSheet13.ZIP › No Scale/sk-hep-1 (No Scale)/pLO5 (6)-单个文件导出-12_c1+3.tif]

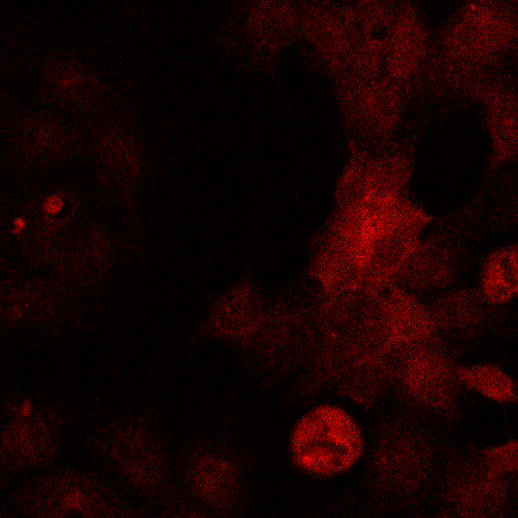

Supplement: Supplementary file 7 [file DataSheet13.ZIP › No Scale/sk-hep-1 (No Scale)/pLO5 (6)-单个文件导出-12_c1.tif]

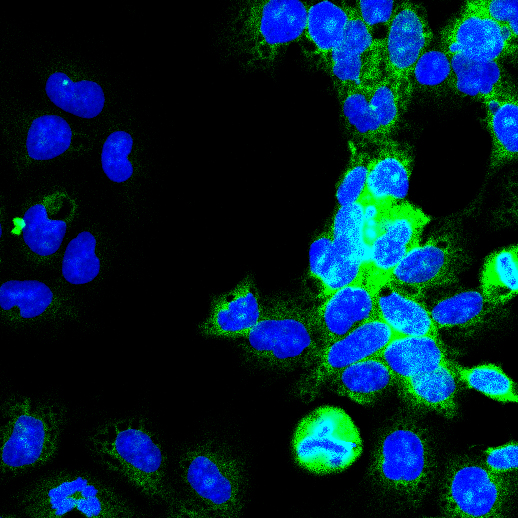

Supplement: Supplementary file 7 [file DataSheet13.ZIP › No Scale/sk-hep-1 (No Scale)/pLO5 (6)-单个文件导出-12_c2+3.jpg]

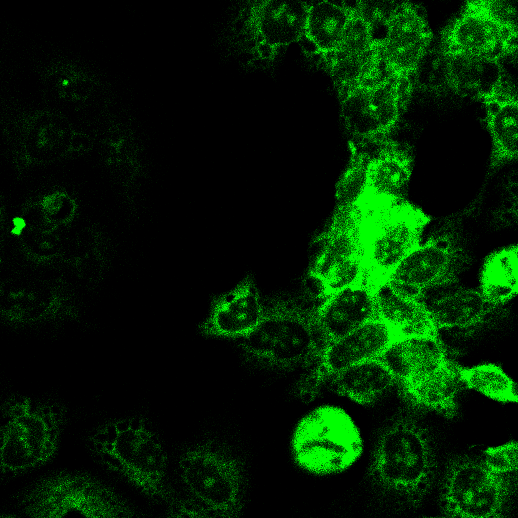

Supplement: Supplementary file 7 [file DataSheet13.ZIP › No Scale/sk-hep-1 (No Scale)/pLO5 (6)-单个文件导出-12_c2.tif]

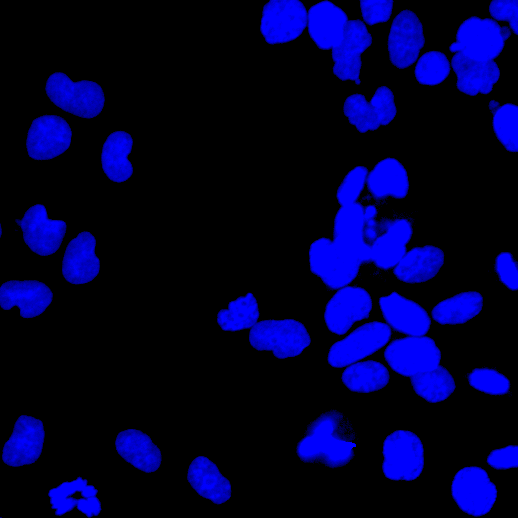

Supplement: Supplementary file 7 [file DataSheet13.ZIP › No Scale/sk-hep-1 (No Scale)/pLO5 (6)-单个文件导出-12_c3.tif]

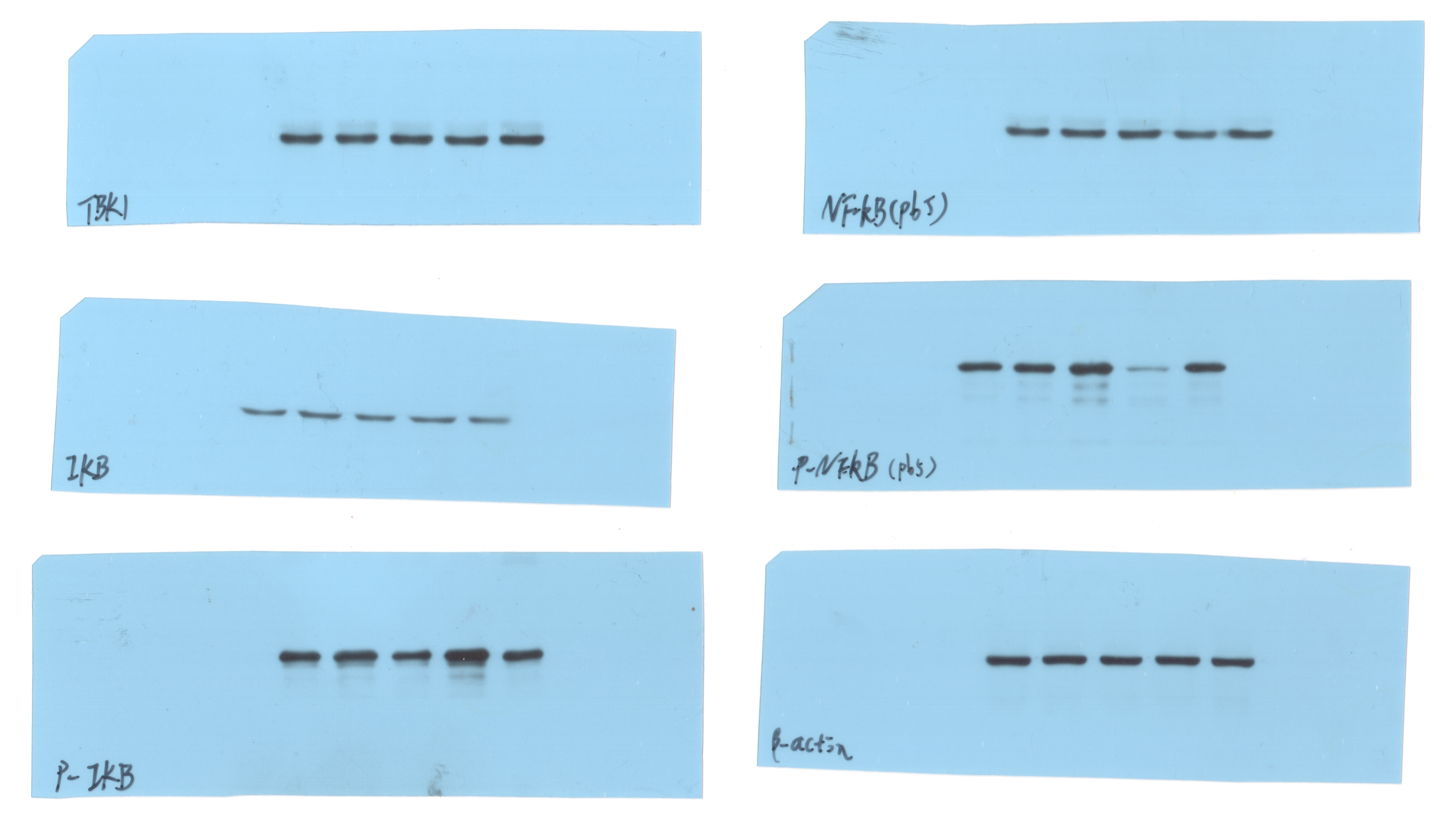

Supplement: Supplementary file 8 [file DataSheet1.ZIP › Original data(3)/WB/The original image.TIF]

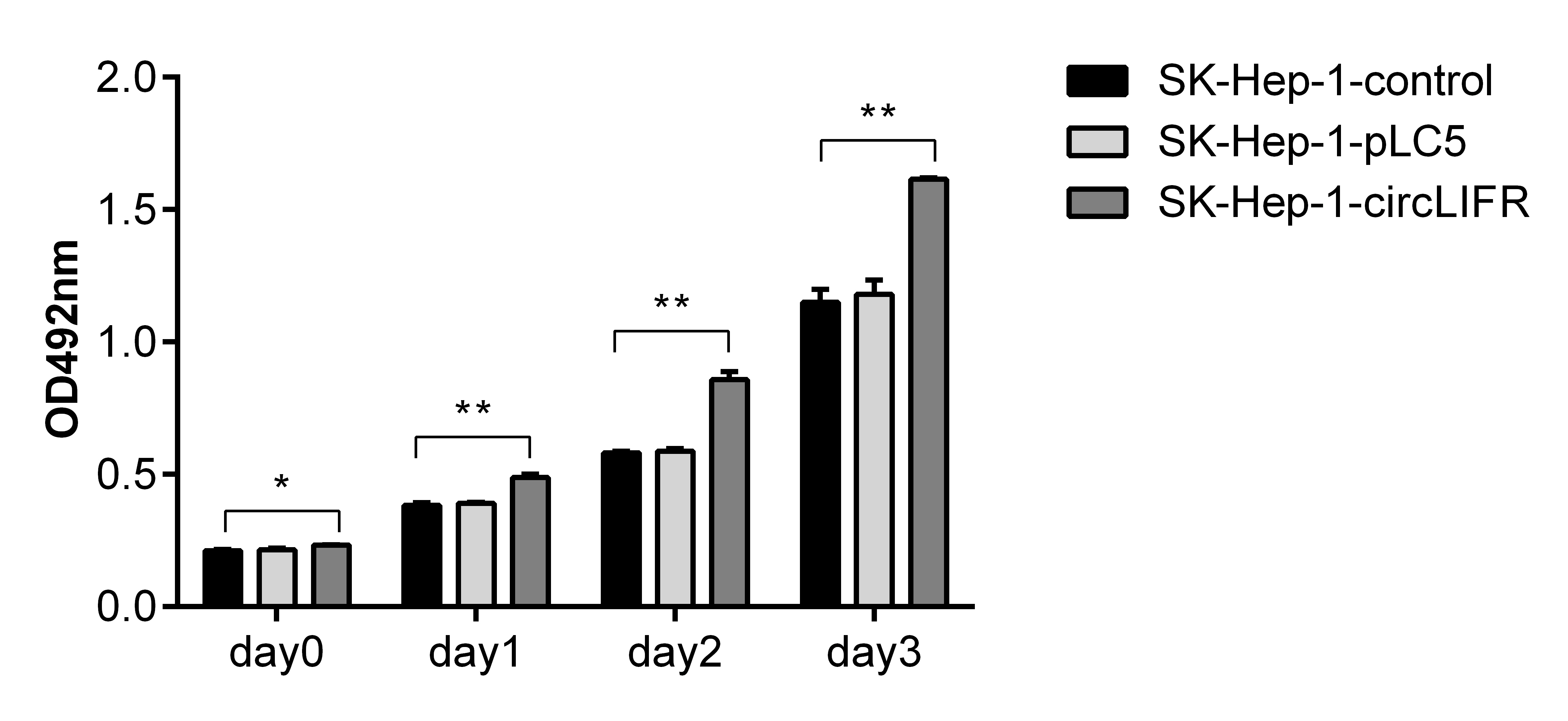

Supplement: Supplementary file 9 [file DataSheet10.ZIP › CCK8/sk-hep-1-1 Proliferation detection.jpg]

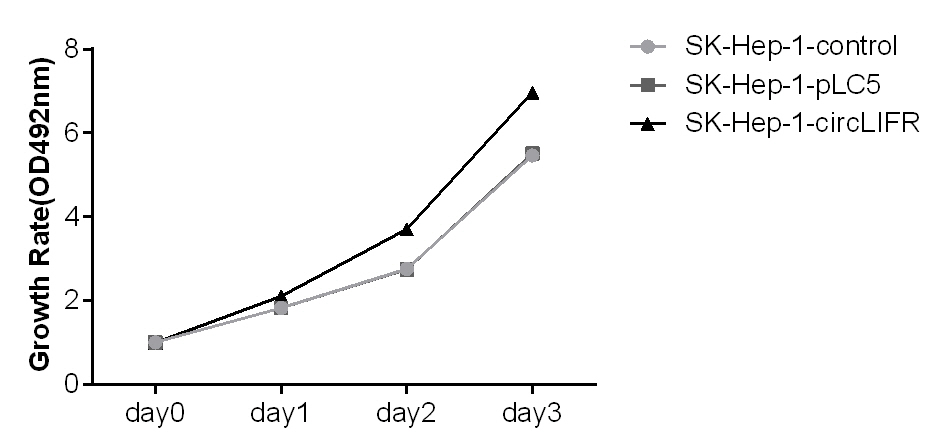

Supplement: Supplementary file 9 [file DataSheet10.ZIP › CCK8/sk-hep-1-Growth curve.jpg]

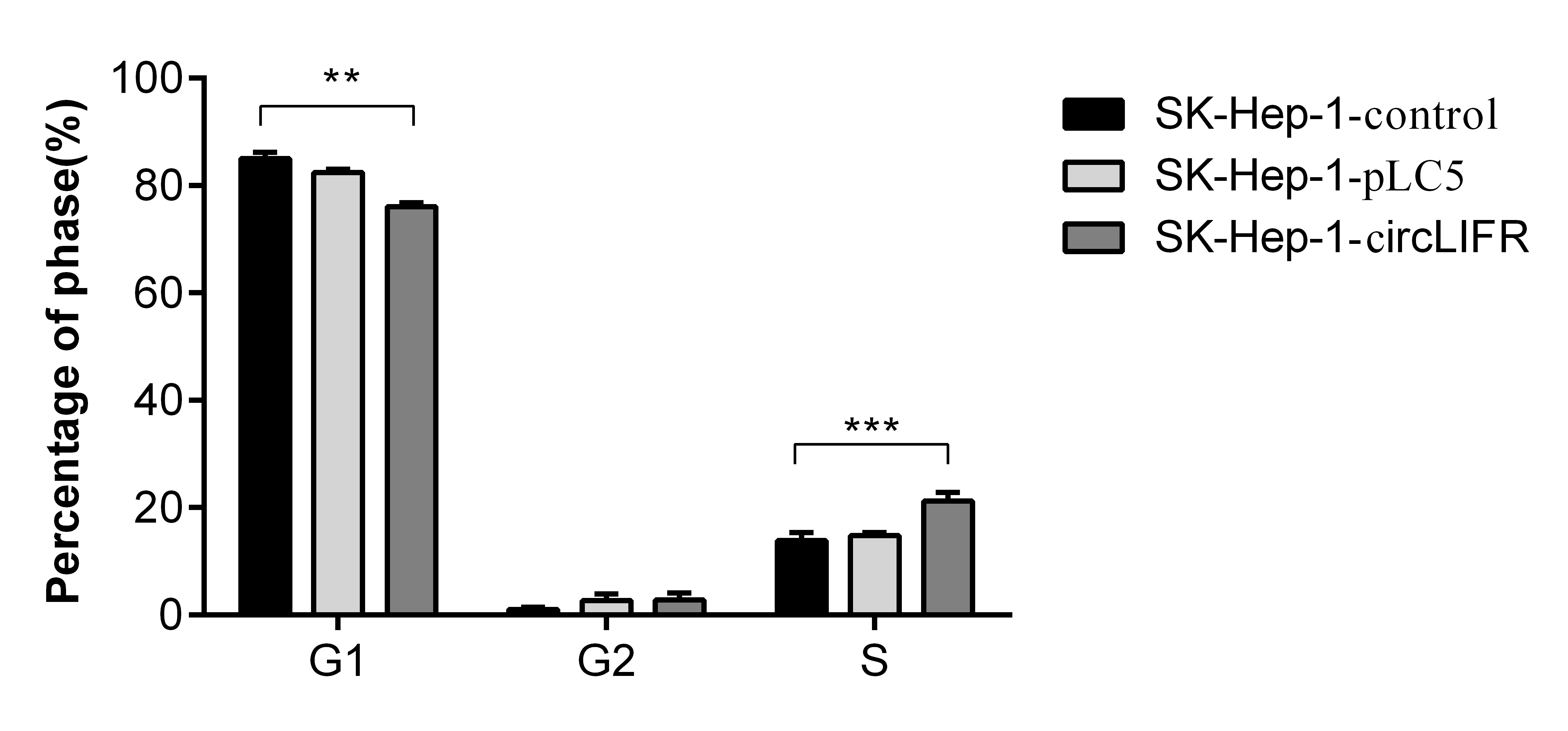

Supplement: Supplementary file 9 [file DataSheet10.ZIP › Cell cycle assay/sk-hep-1.jpg]

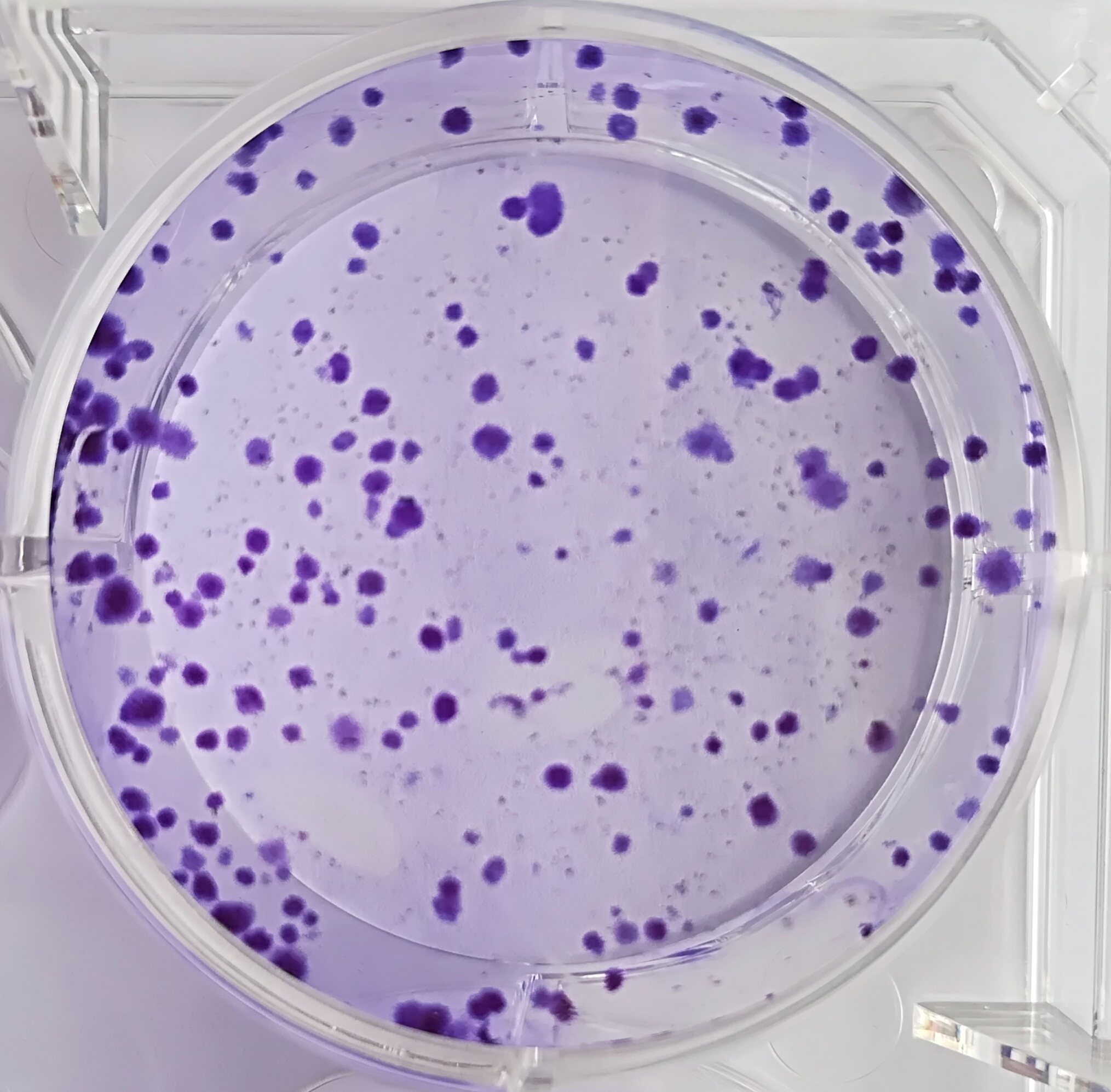

Supplement: Supplementary file 9 [file DataSheet10.ZIP › Colony forming/C72309-183.jpg]

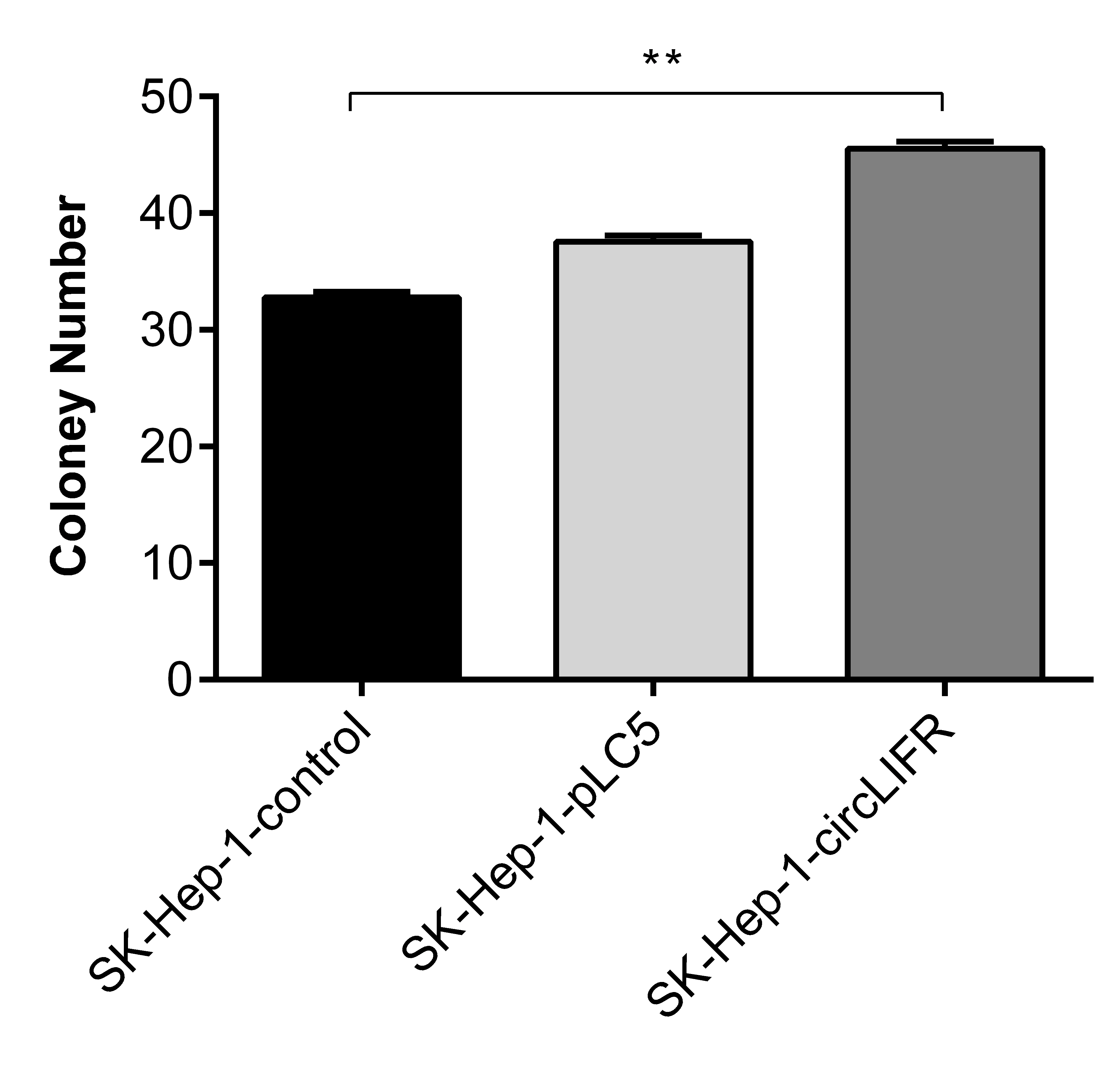

Supplement: Supplementary file 9 [file DataSheet10.ZIP › Colony forming/Clone formation rate.jpg]

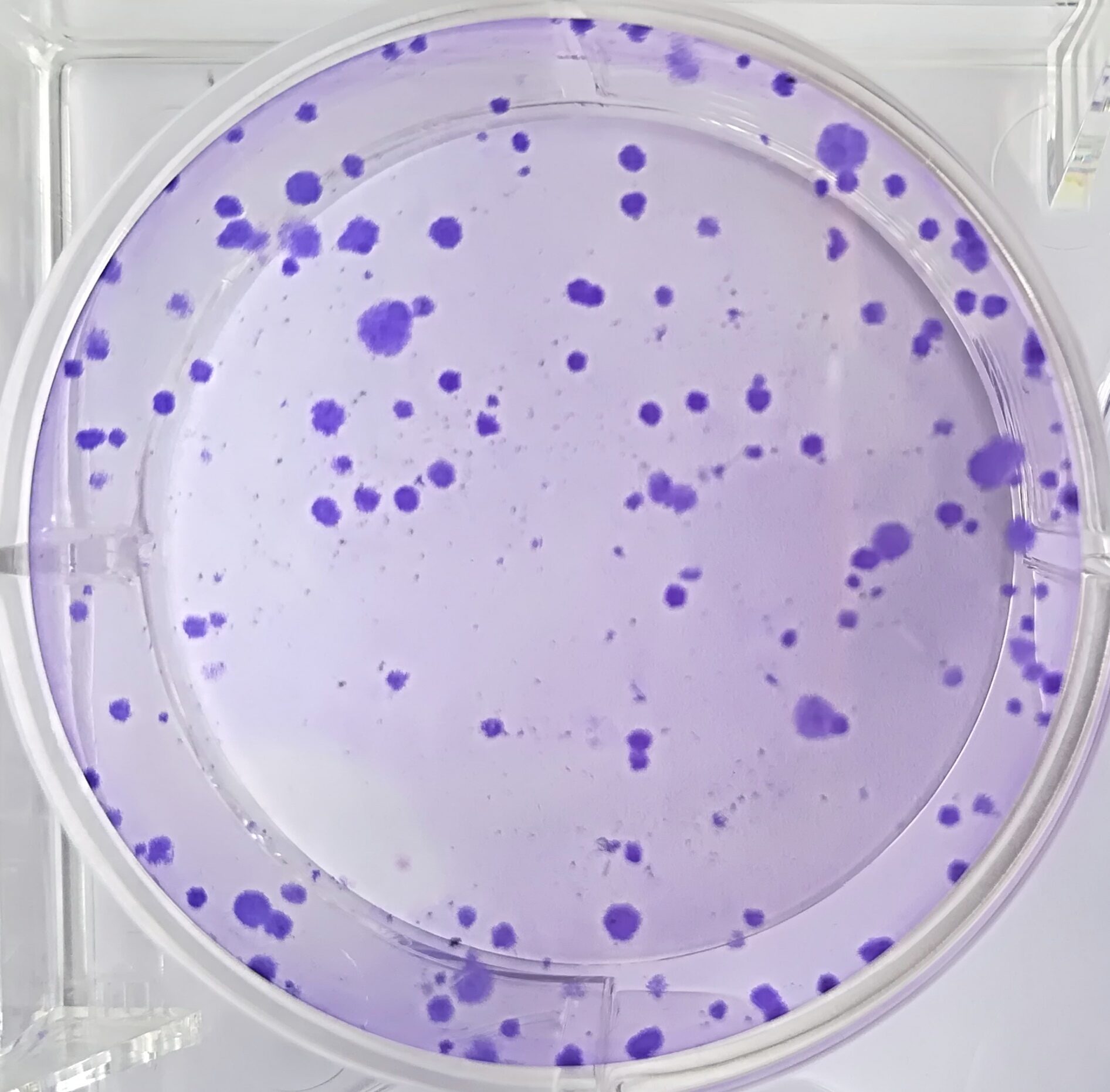

Supplement: Supplementary file 9 [file DataSheet10.ZIP › Colony forming/control-131.jpg]

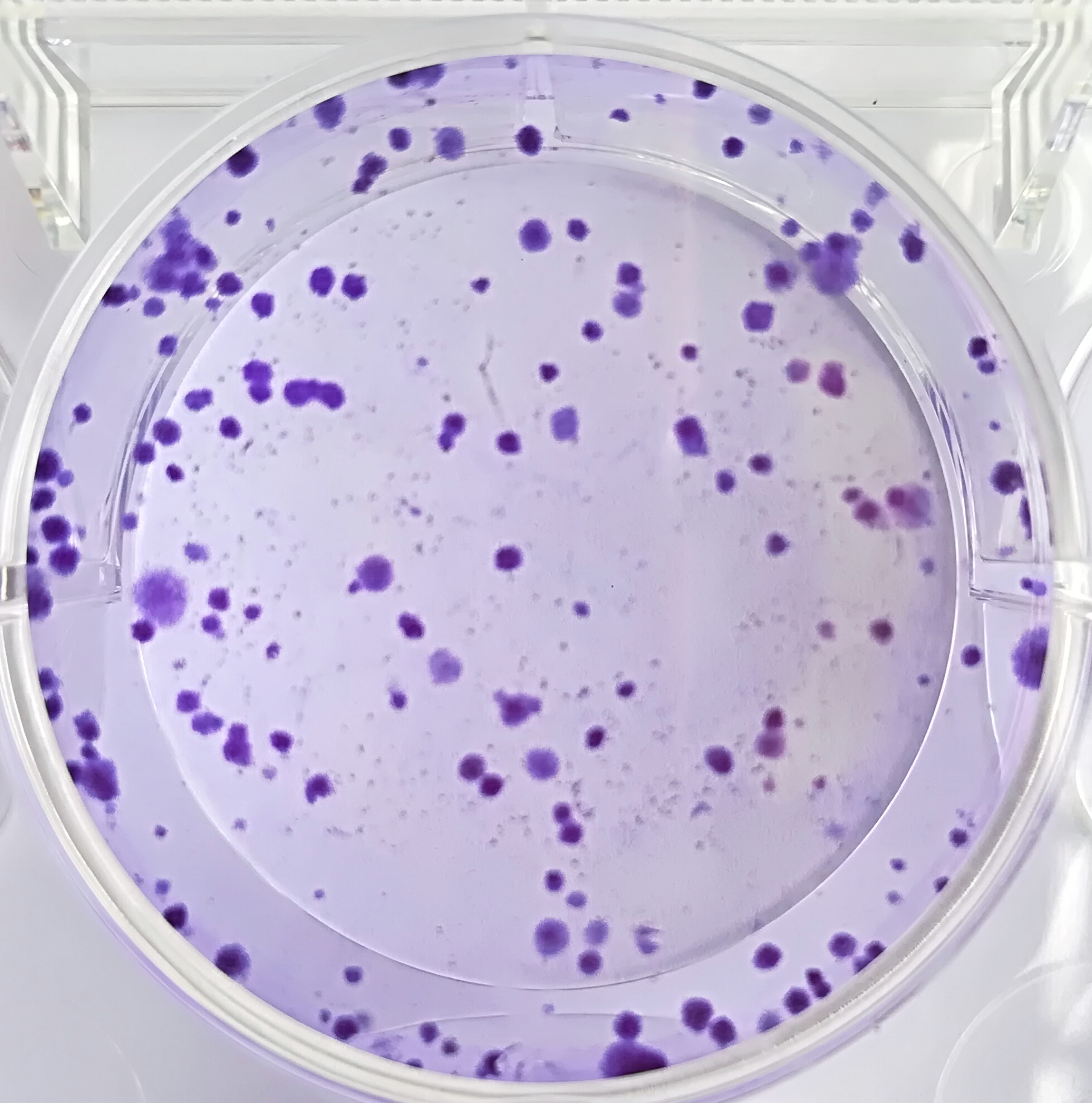

Supplement: Supplementary file 9 [file DataSheet10.ZIP › Colony forming/pLC5-151.jpg]

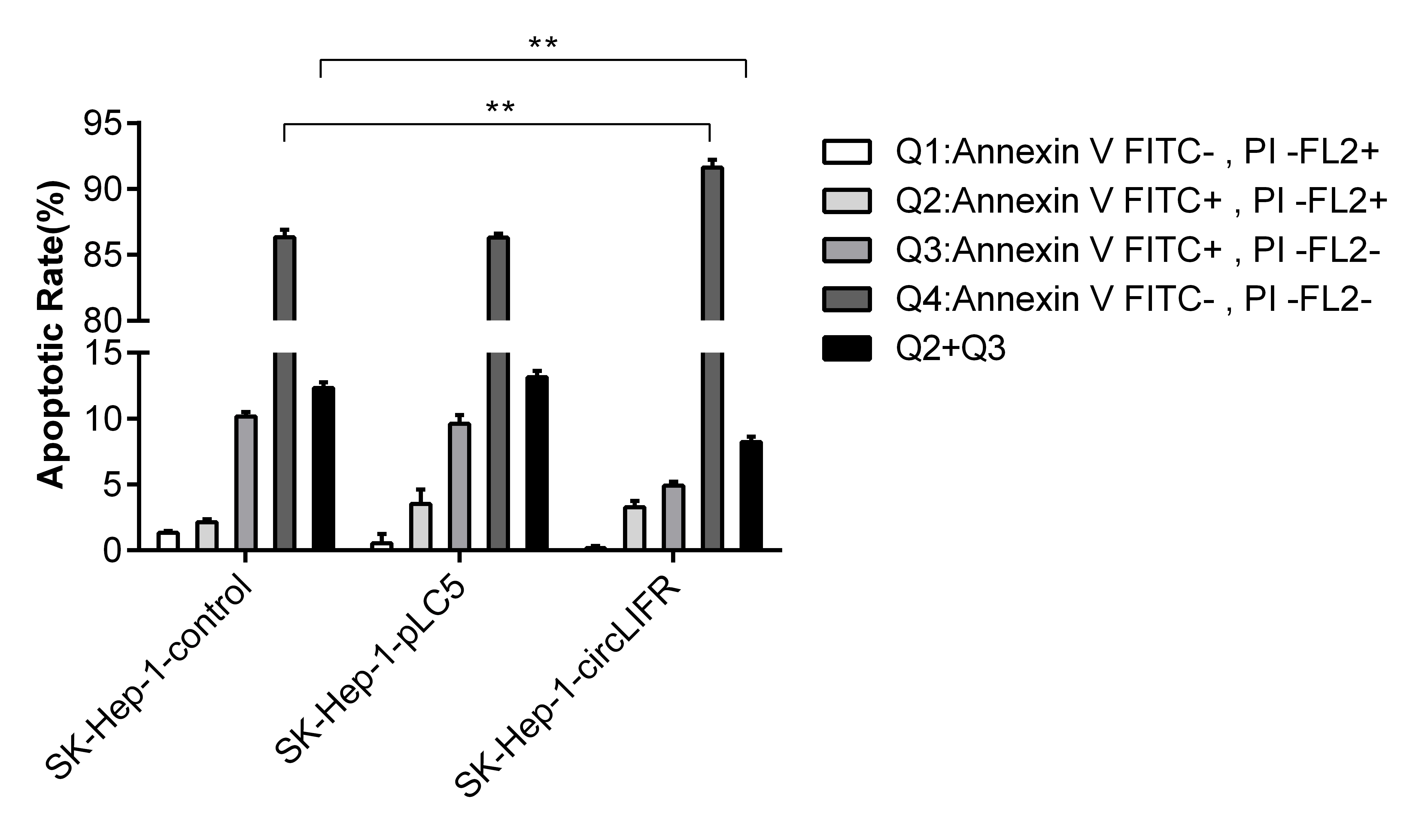

Supplement: Supplementary file 9 [file DataSheet10.ZIP › Flow cytometry/sk-hep-1-Apoptosis detection.jpg]

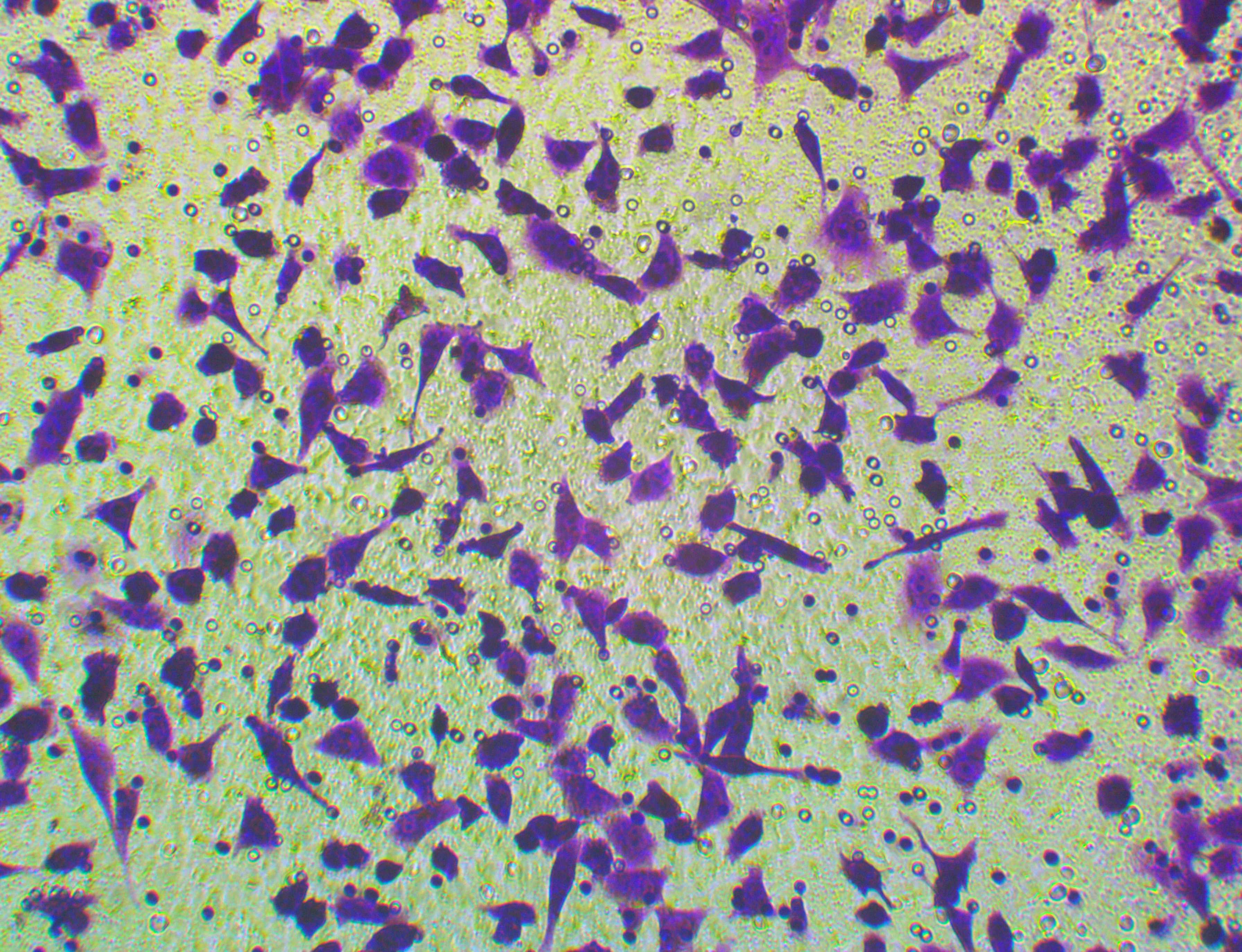

Supplement: Supplementary file 9 [file DataSheet10.ZIP › Transwell/2-211-invasion-control.jpg]

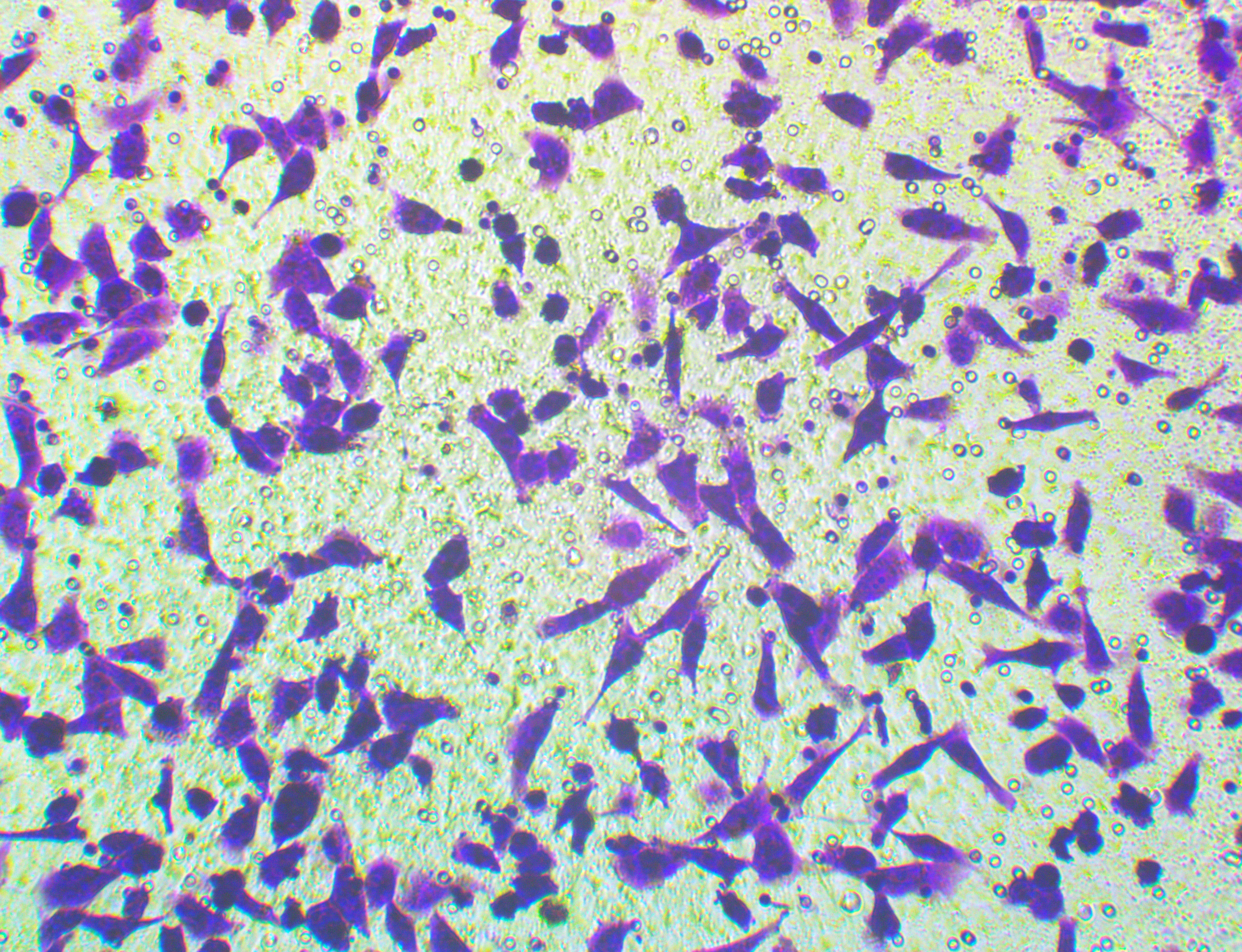

Supplement: Supplementary file 9 [file DataSheet10.ZIP › Transwell/2-258-invasion-plc5.jpg]

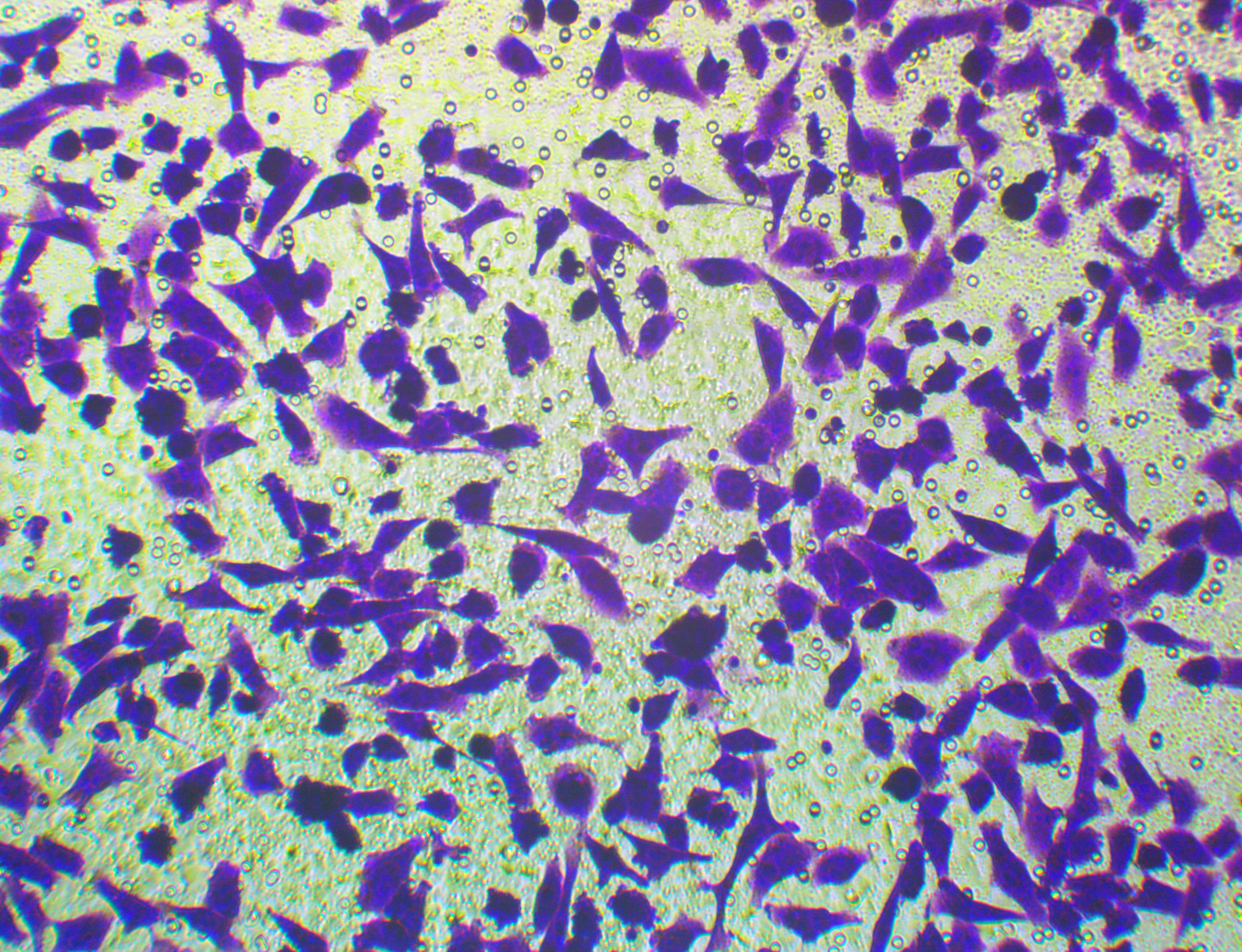

Supplement: Supplementary file 9 [file DataSheet10.ZIP › Transwell/2-2840-Migration-control.jpg]

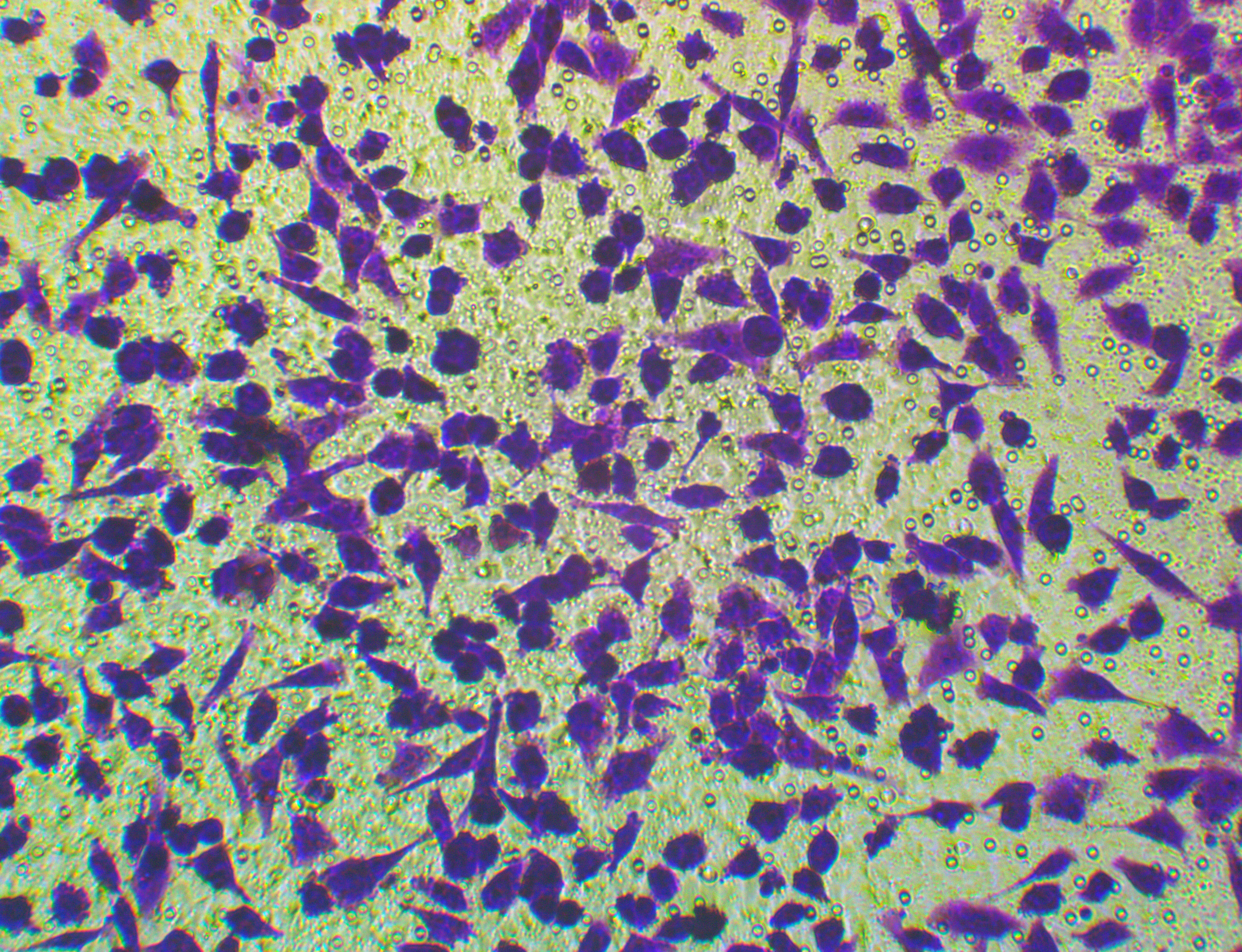

Supplement: Supplementary file 9 [file DataSheet10.ZIP › Transwell/2-333-Migration-plc5.jpg]

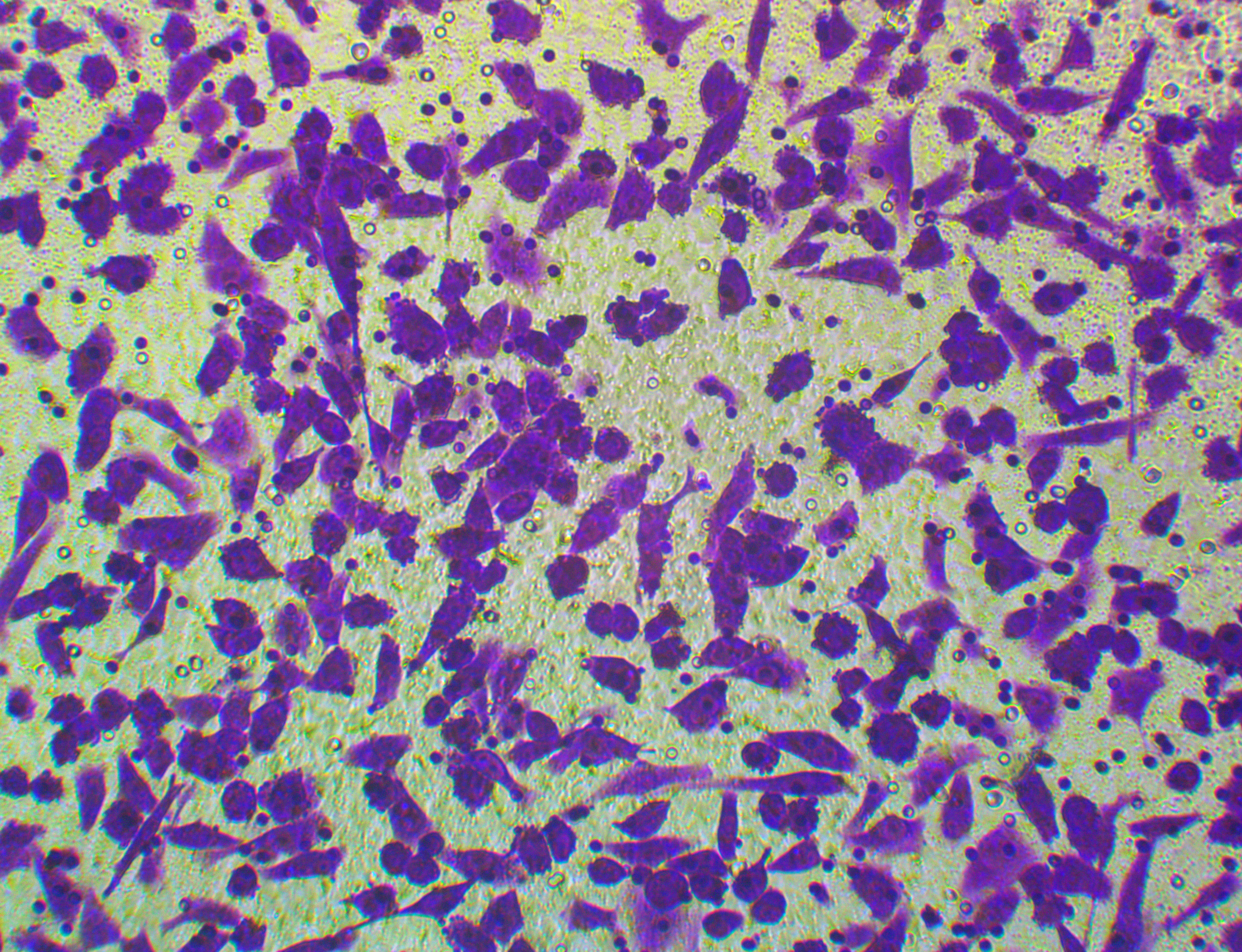

Supplement: Supplementary file 9 [file DataSheet10.ZIP › Transwell/2-345-invasion-circLIFR.jpg]

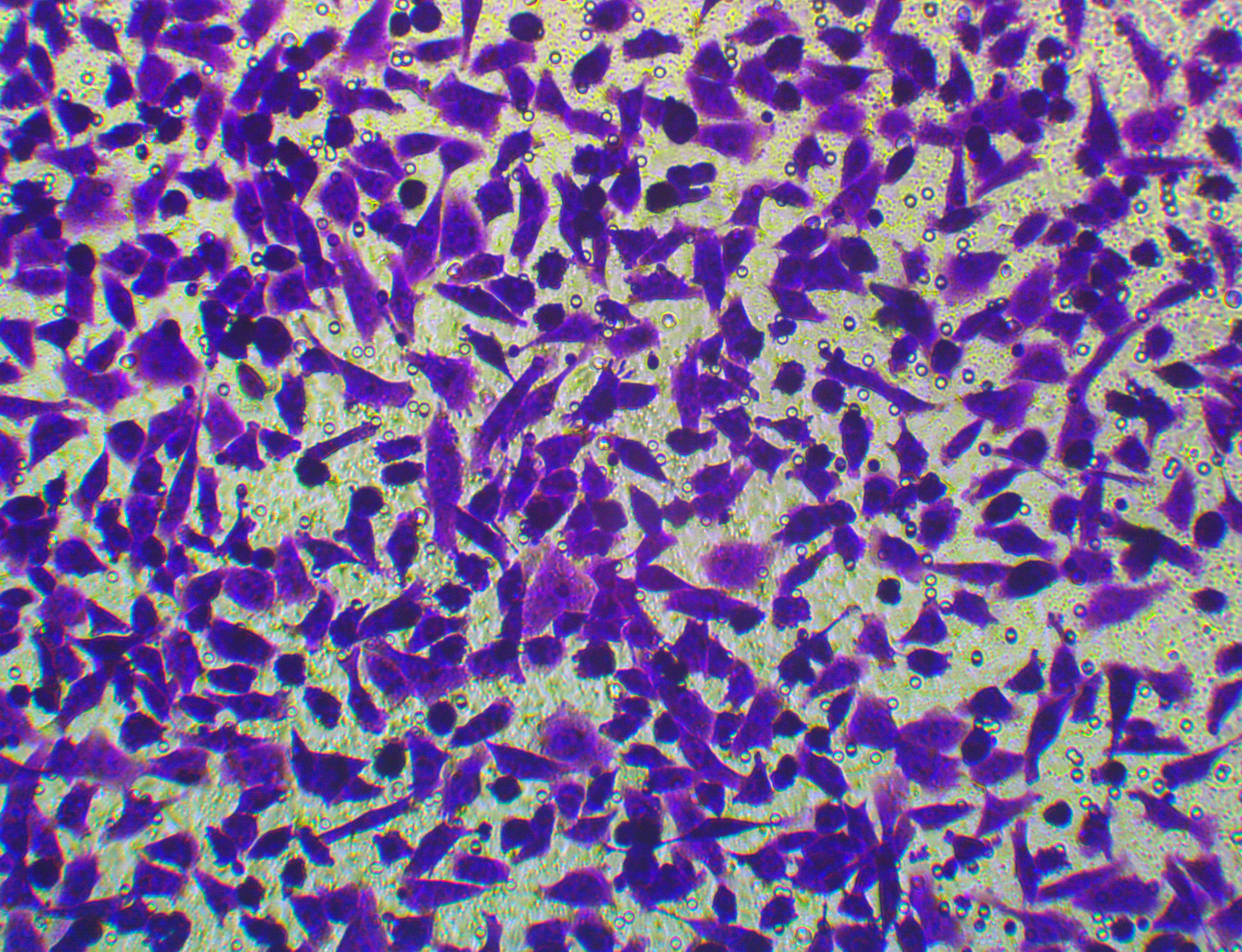

Supplement: Supplementary file 9 [file DataSheet10.ZIP › Transwell/2-487-Migration-circLIFR.jpg]

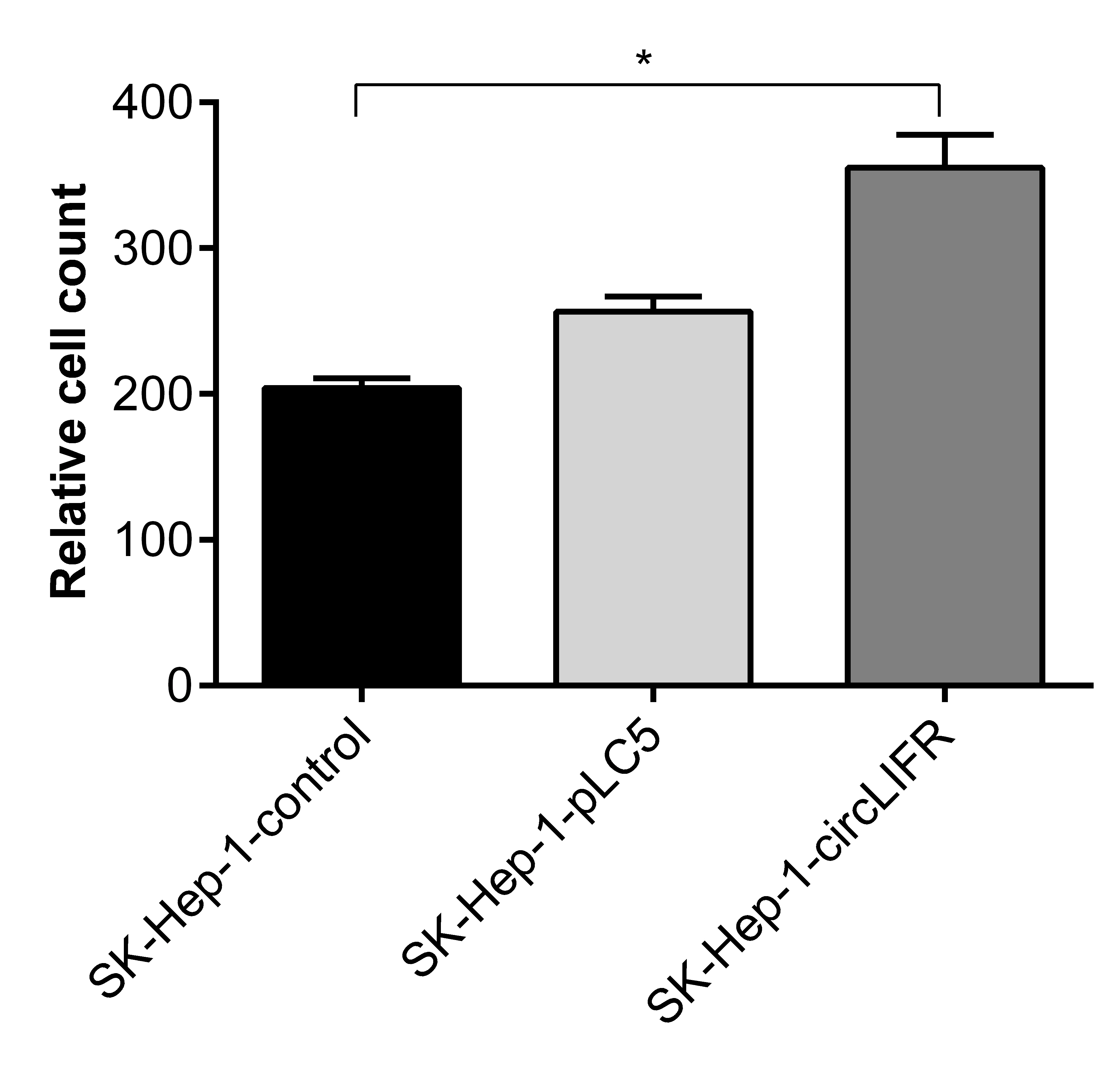

Supplement: Supplementary file 9 [file DataSheet10.ZIP › Transwell/sk-hep-1-invasion.jpg]

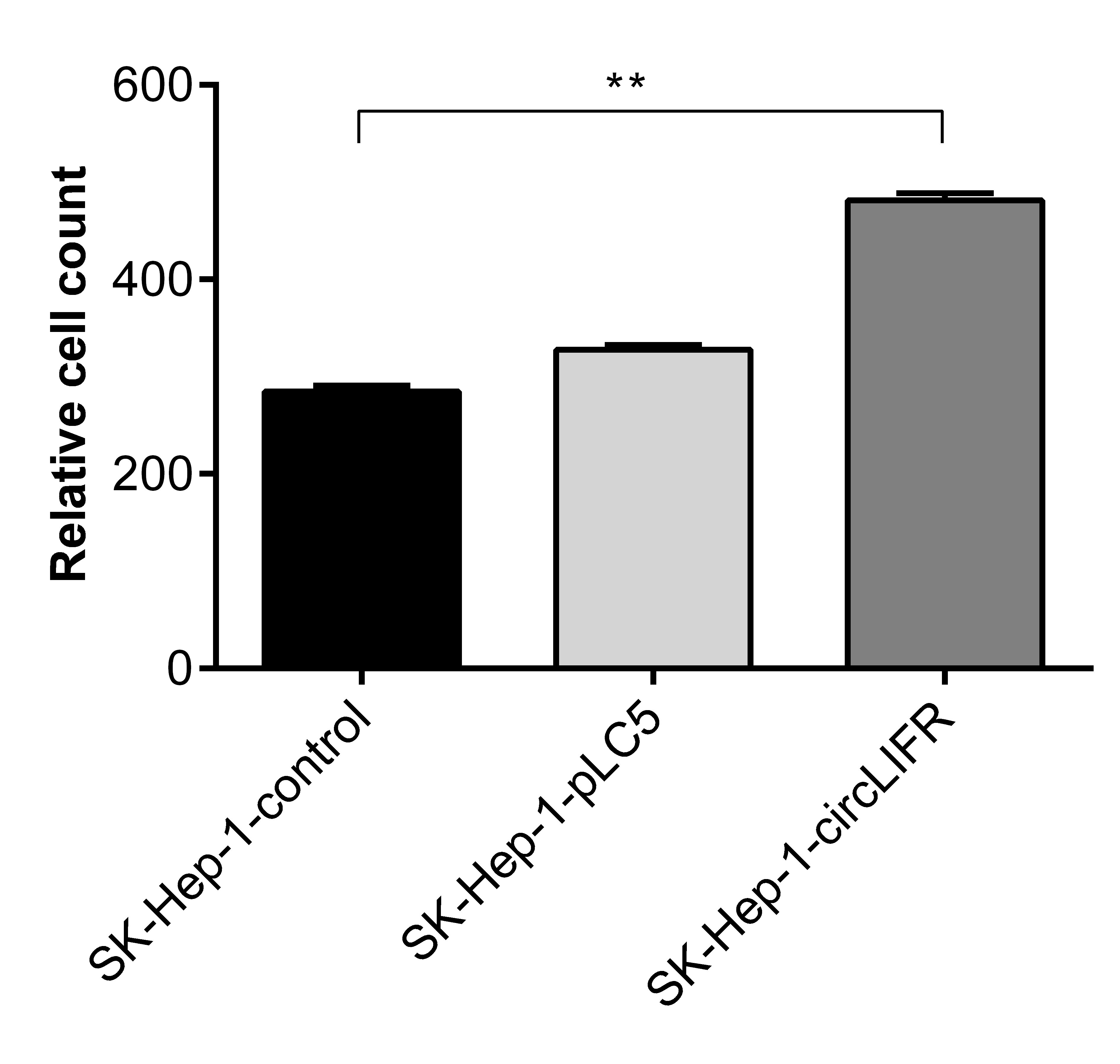

Supplement: Supplementary file 9 [file DataSheet10.ZIP › Transwell/sk-hep-1-Migration.jpg]

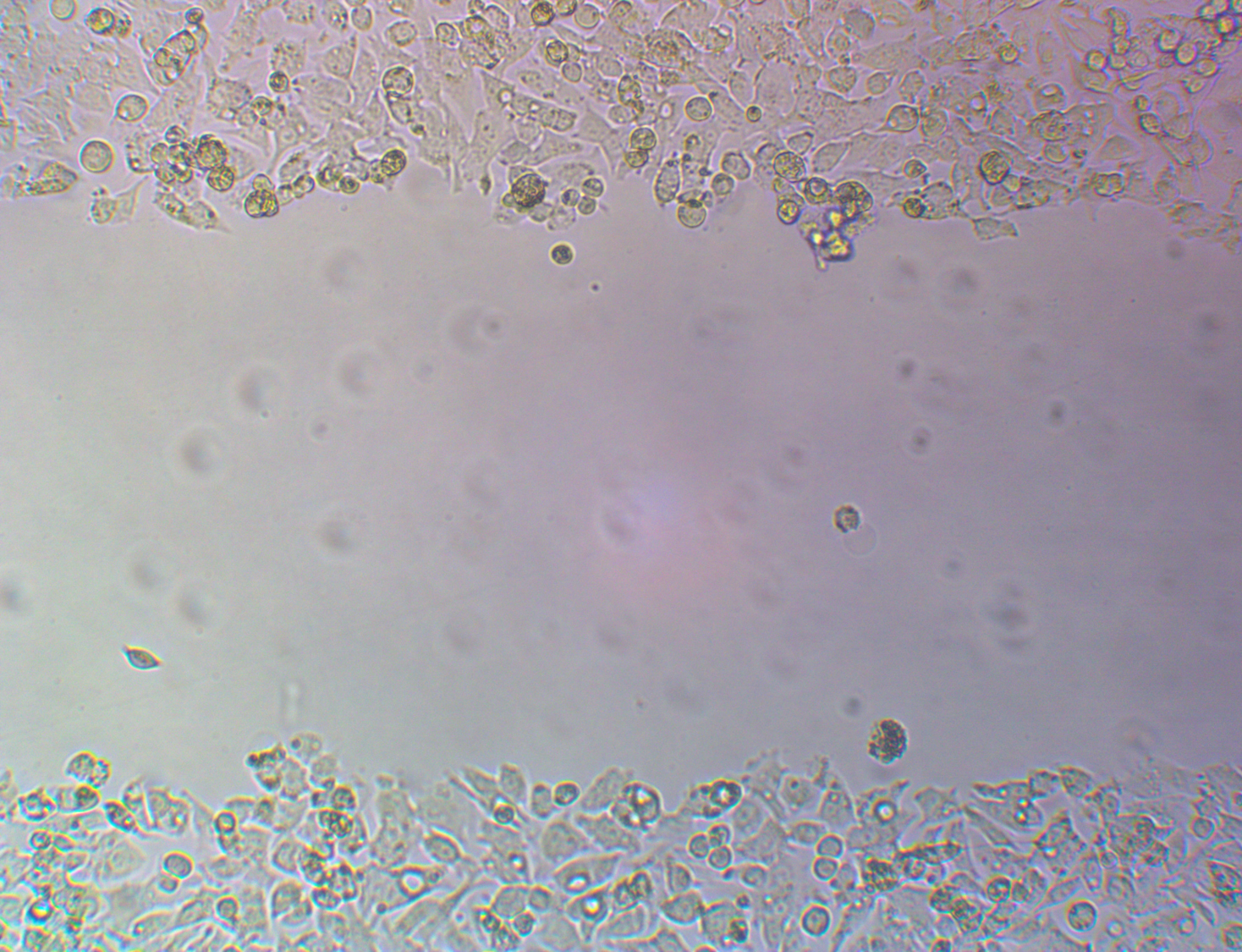

Supplement: Supplementary file 10 [file DataSheet6.ZIP › 0H/plc5-0h-3 raw.jpg]

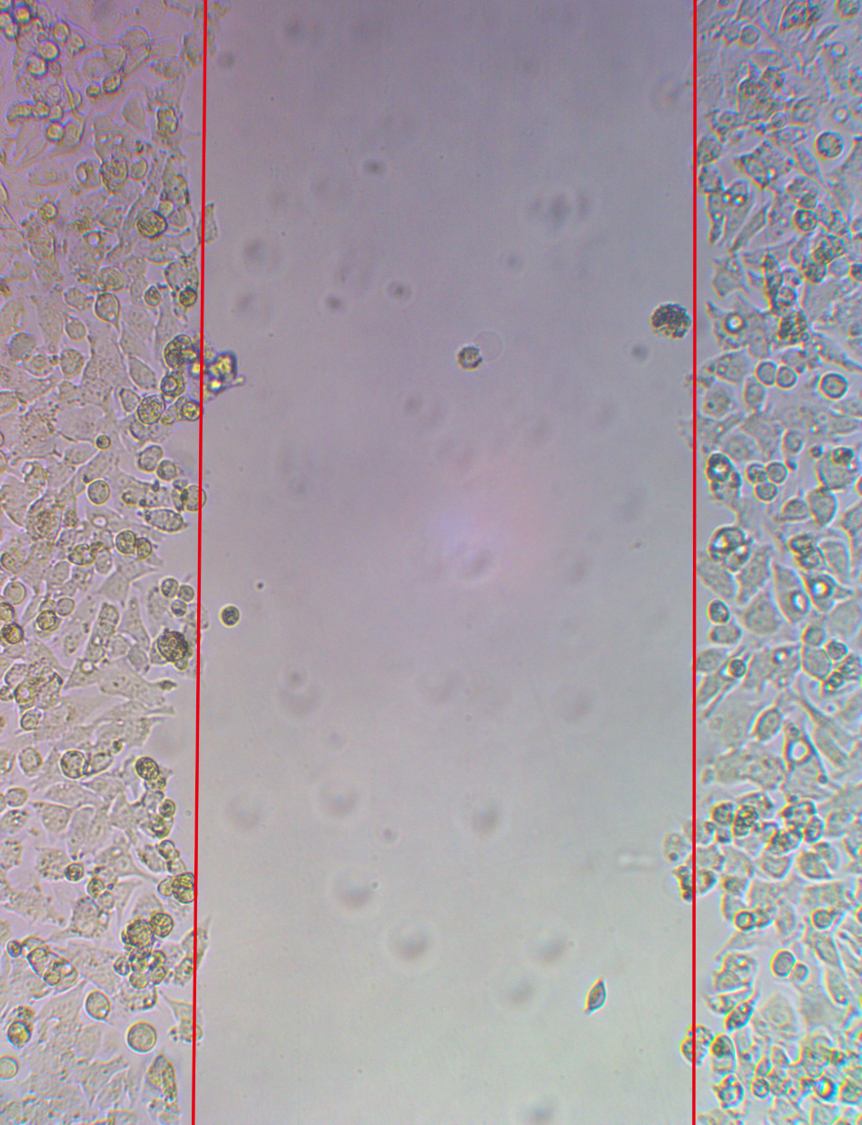

Supplement: Supplementary file 10 [file DataSheet6.ZIP › 0H/plc5-0h-3.jpg]

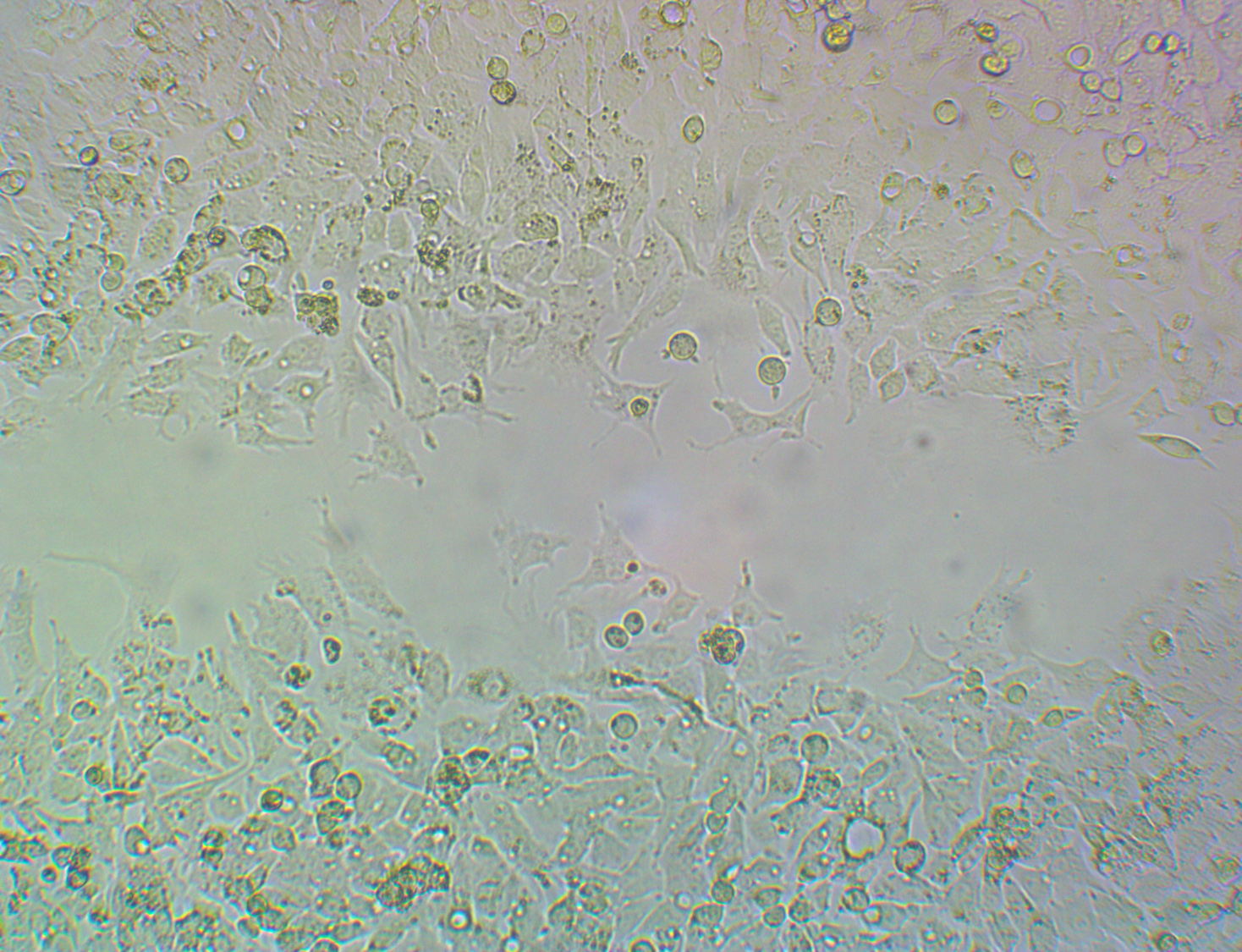

Supplement: Supplementary file 10 [file DataSheet6.ZIP › 0H/plc5-0h-5 raw.jpg]

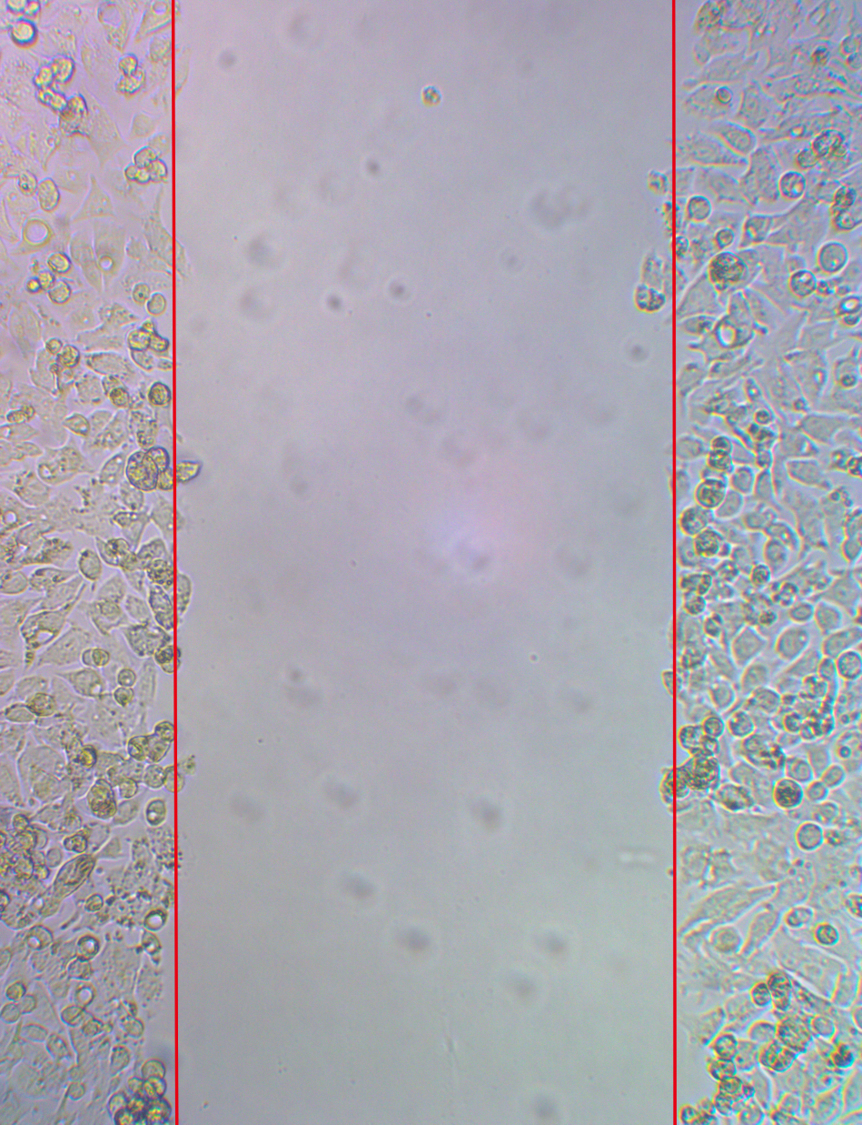

Supplement: Supplementary file 10 [file DataSheet6.ZIP › 0H/plc5-0h-5.jpg]

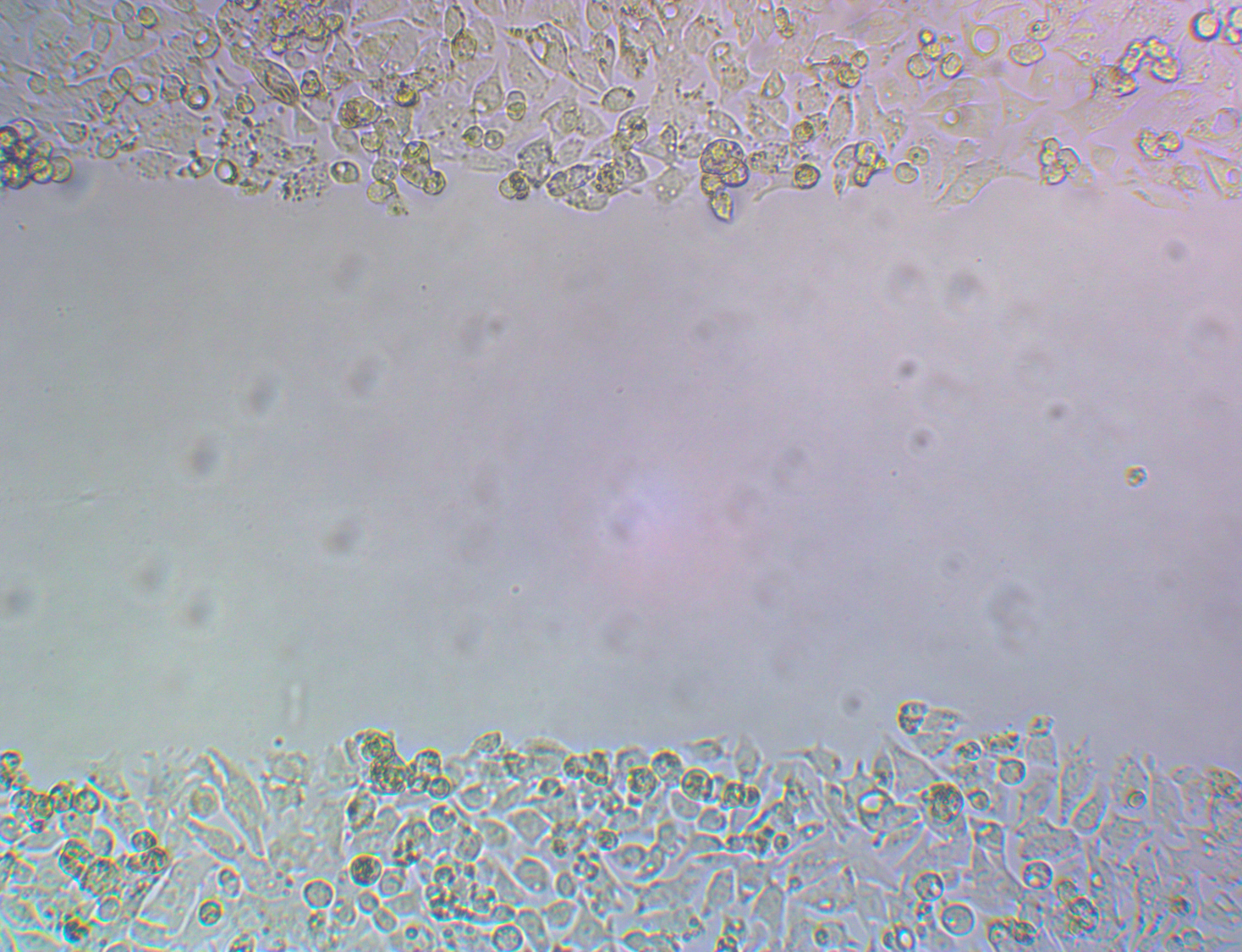

Supplement: Supplementary file 10 [file DataSheet6.ZIP › 0H/plc5-0h-5raw.jpg]

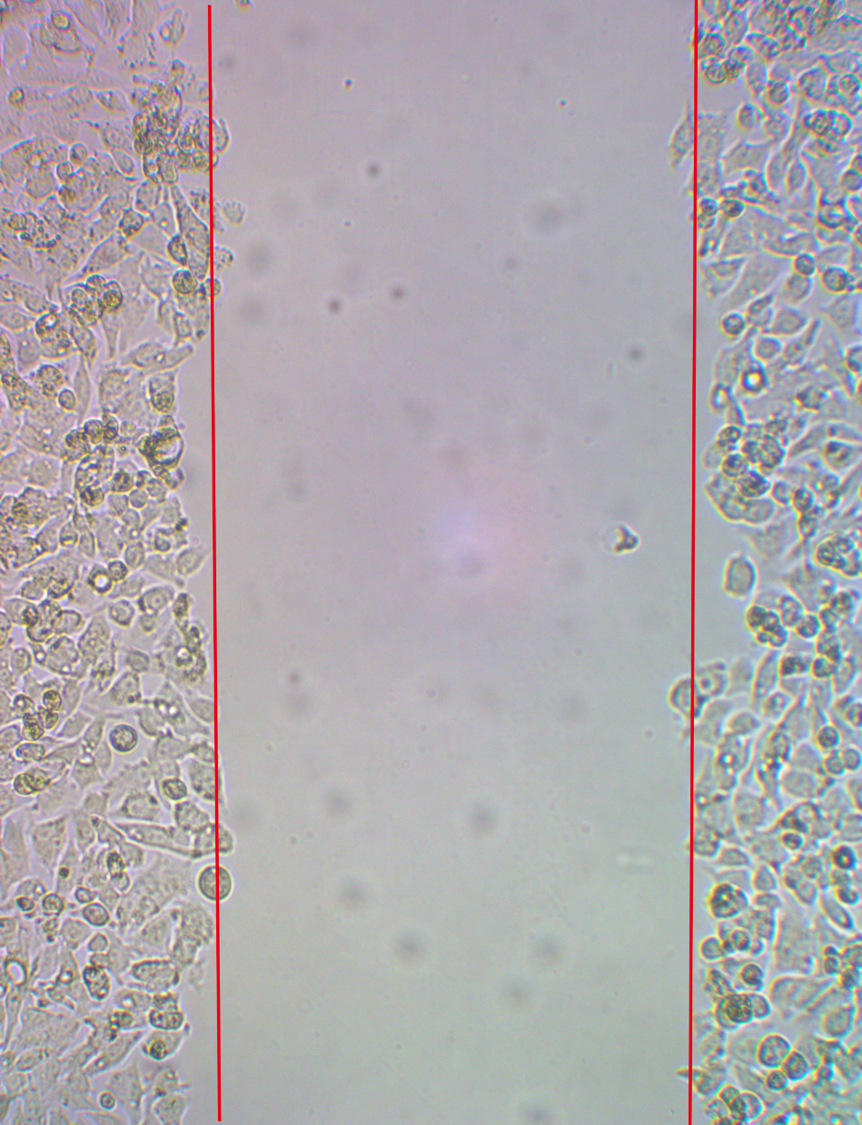

Supplement: Supplementary file 10 [file DataSheet6.ZIP › 0H/plc5-0h-9.jpg]

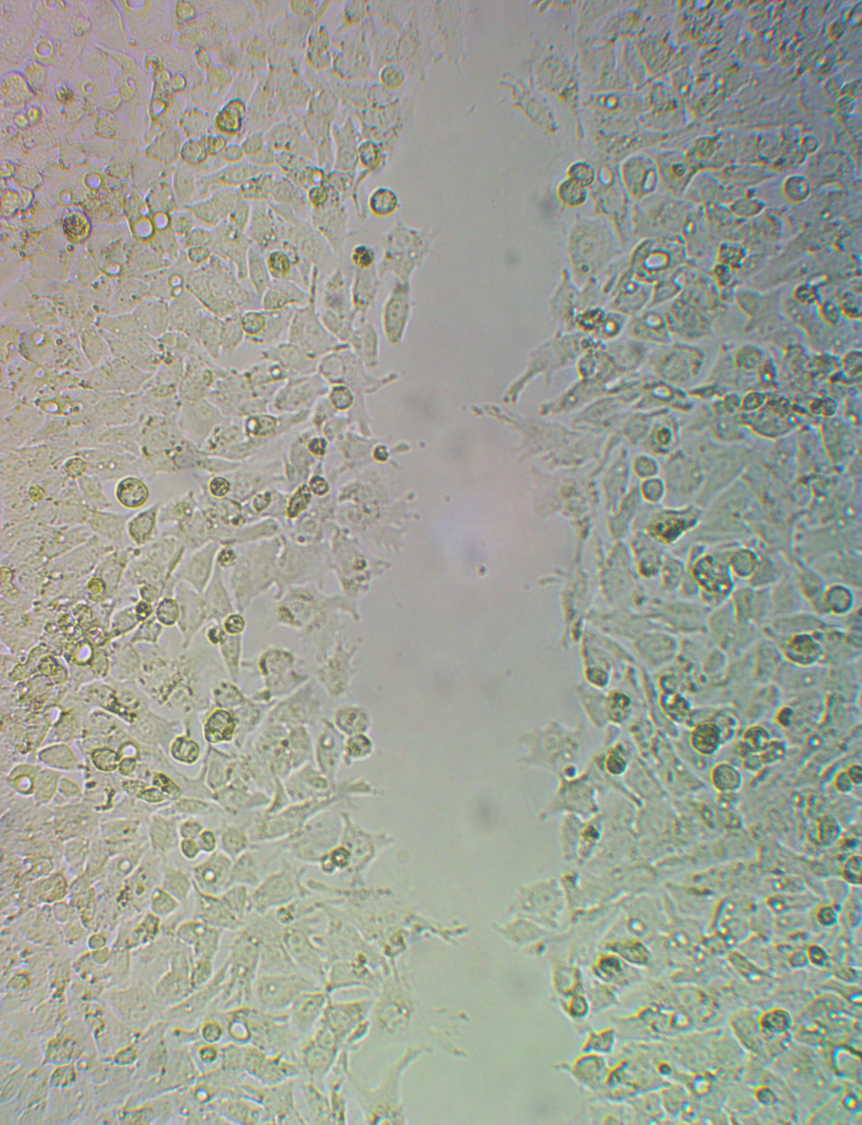

Supplement: Supplementary file 10 [file DataSheet6.ZIP › 24H/plc5-24h-11 raw.jpg]

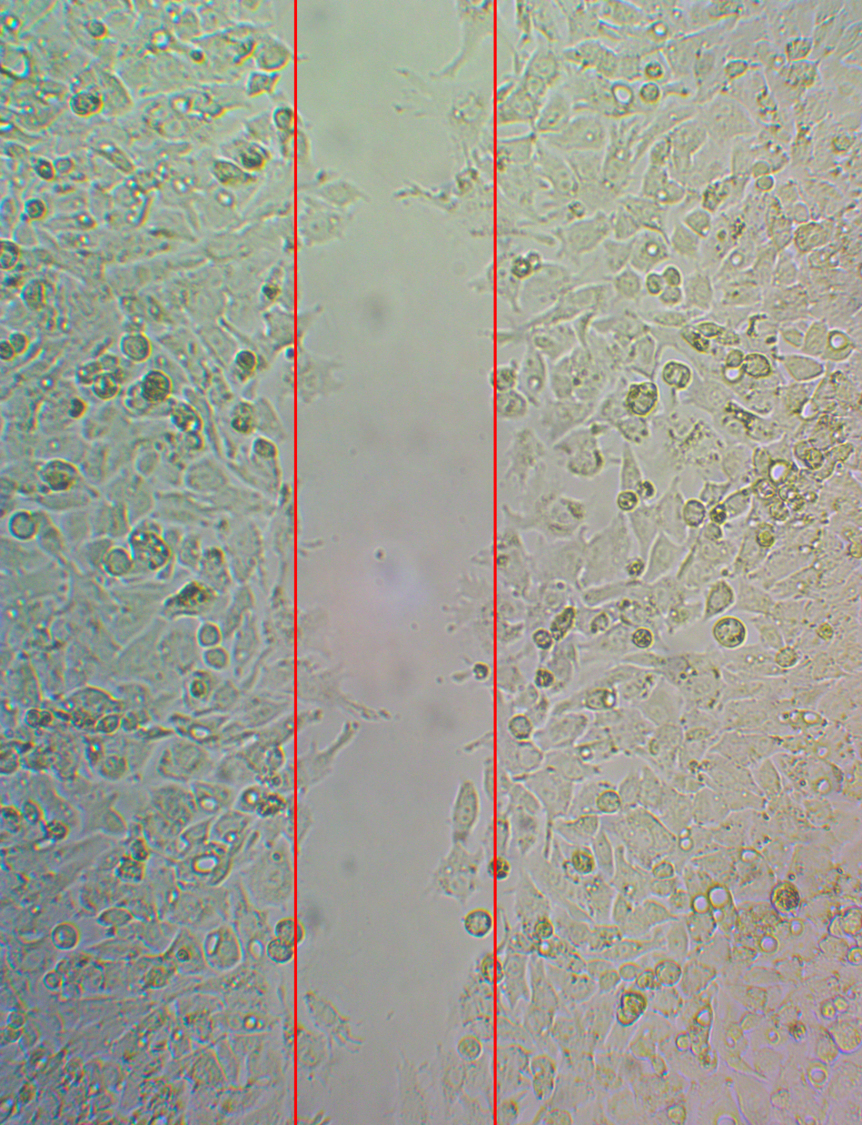

Supplement: Supplementary file 10 [file DataSheet6.ZIP › 24H/plc5-24h-11.jpg]

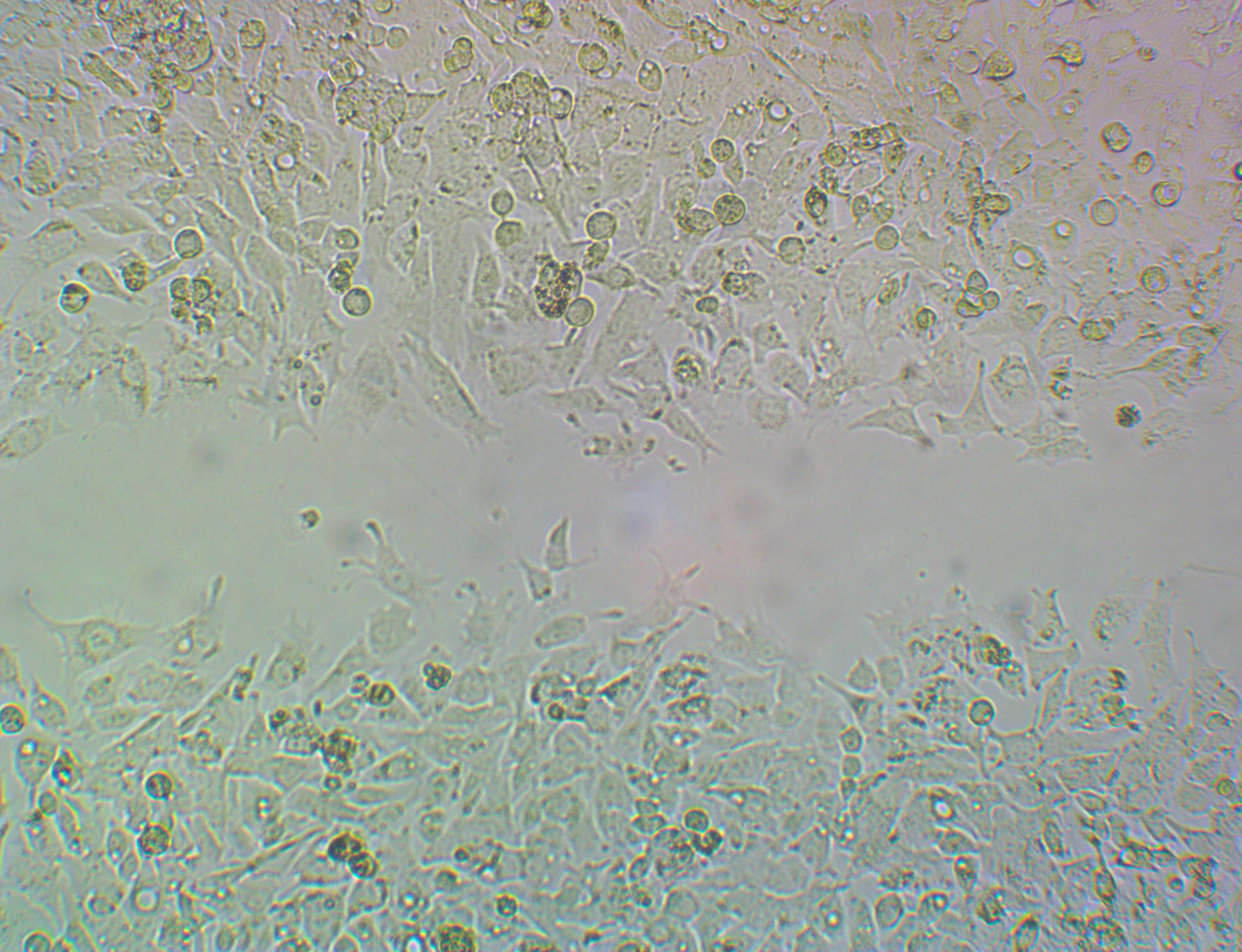

Supplement: Supplementary file 10 [file DataSheet6.ZIP › 24H/plc5-24h-8 raw.jpg]

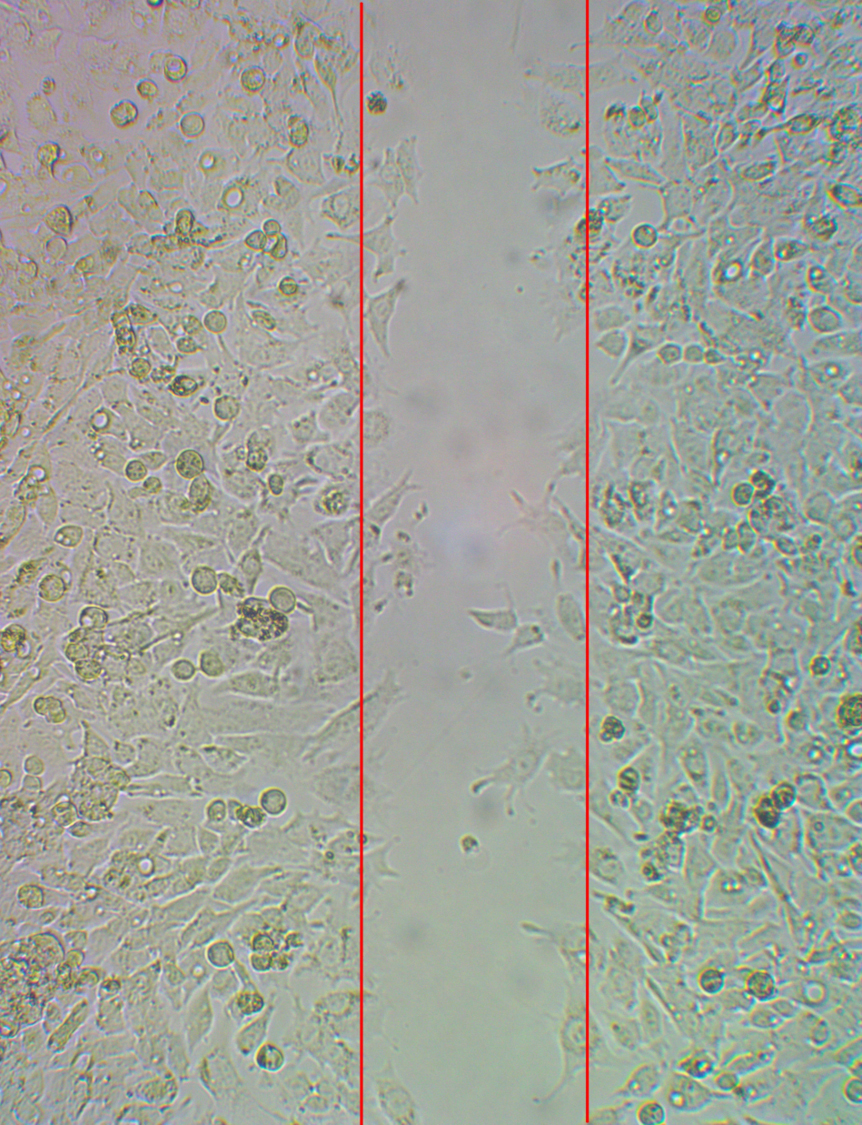

Supplement: Supplementary file 10 [file DataSheet6.ZIP › 24H/plc5-24h-8.jpg]

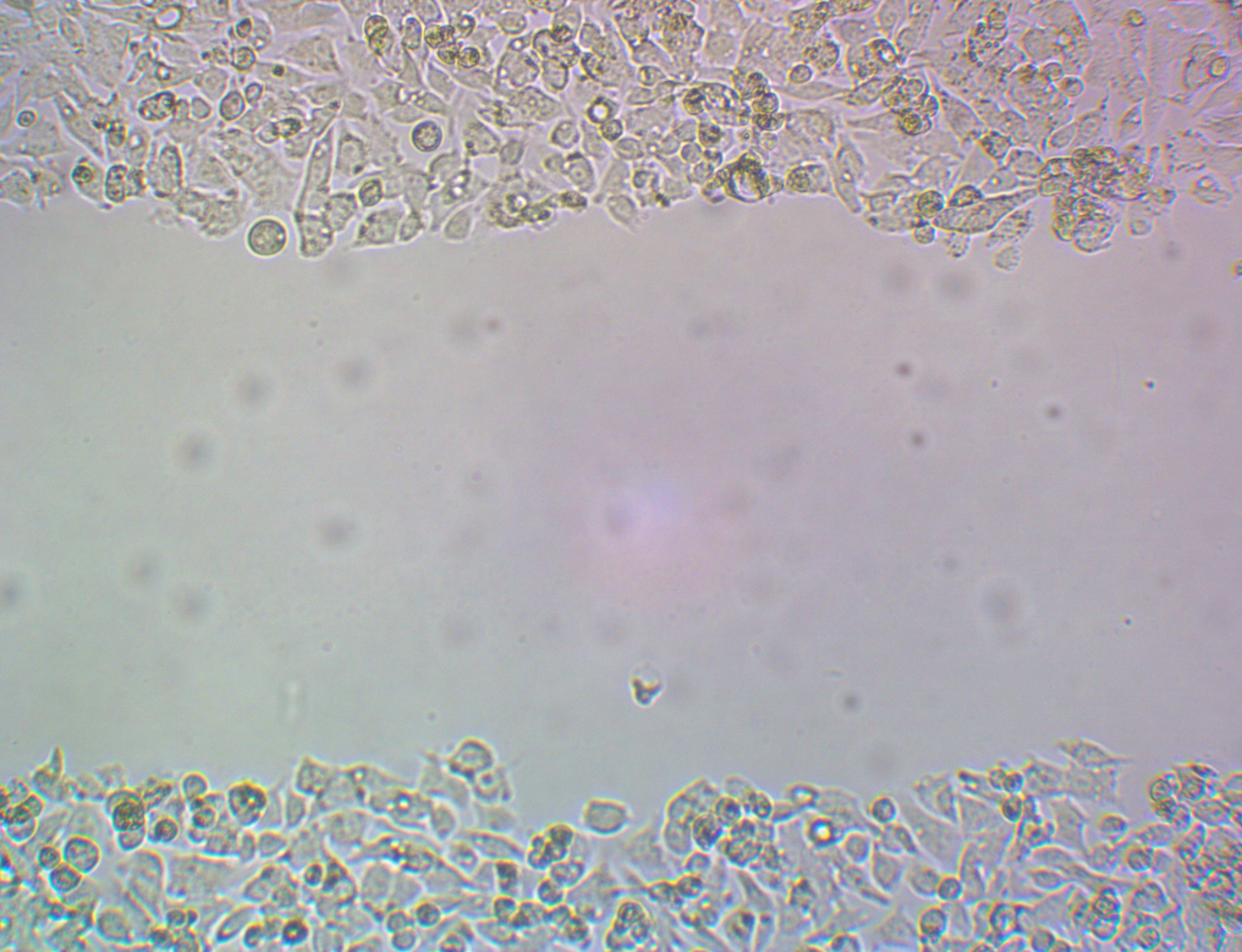

Supplement: Supplementary file 10 [file DataSheet6.ZIP › 24H/plc5-24h-9 raw.jpg]

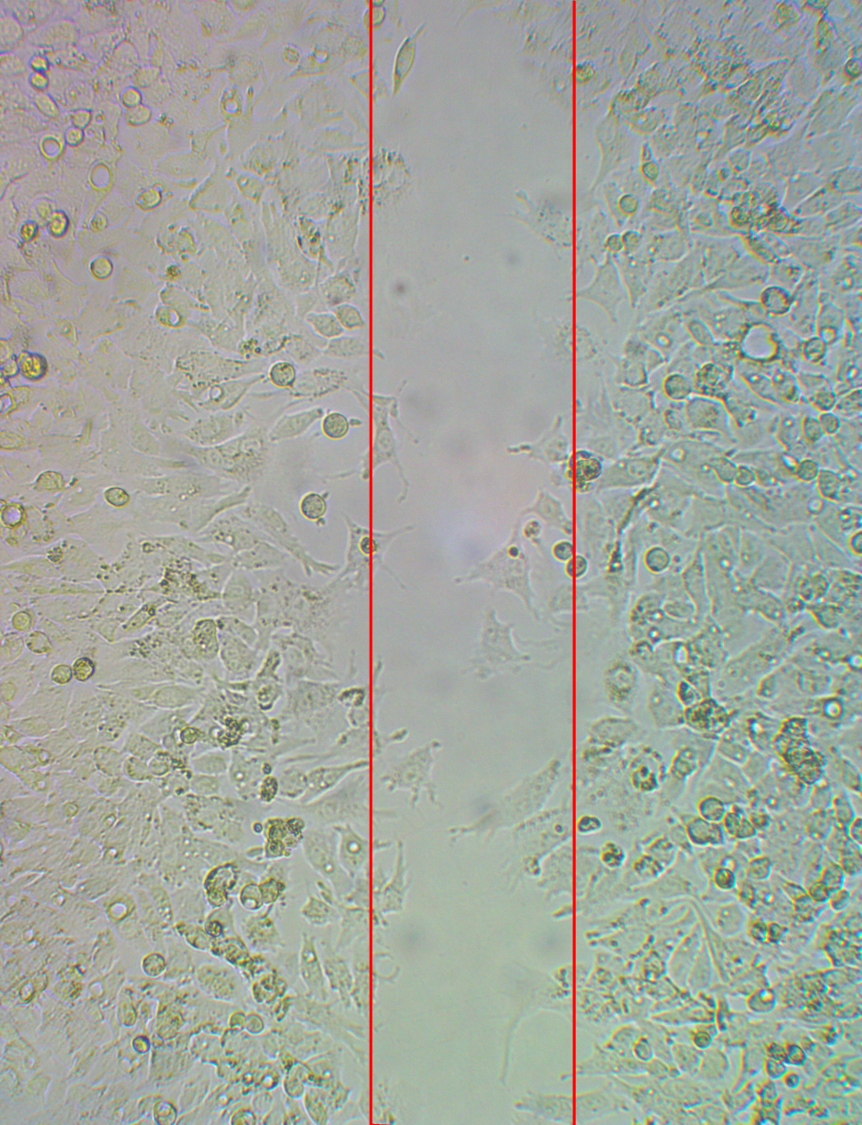

Supplement: Supplementary file 10 [file DataSheet6.ZIP › 24H/plc5-24h-9.jpg]

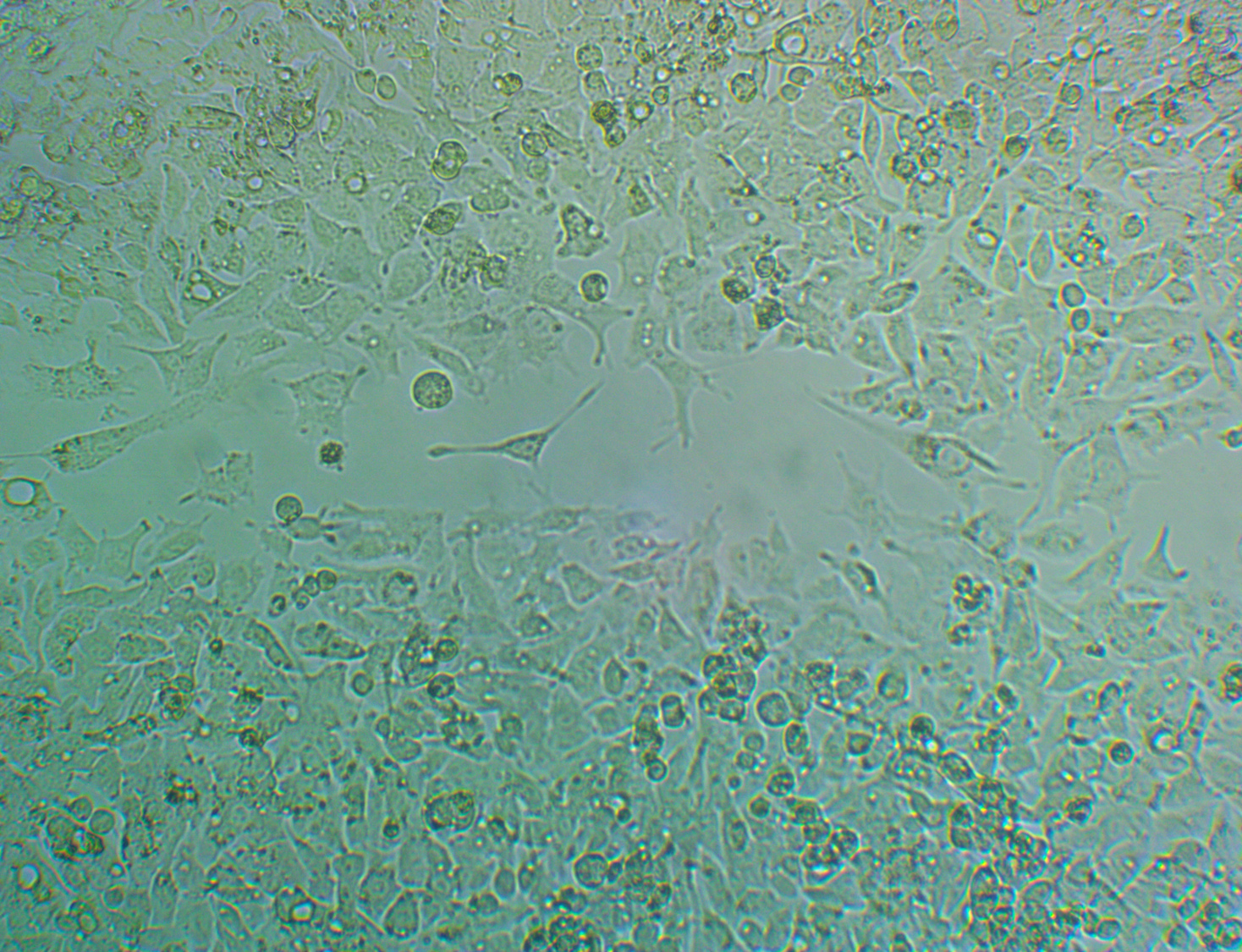

Supplement: Supplementary file 10 [file DataSheet6.ZIP › 48H/plc5-48h-2 raw.jpg]

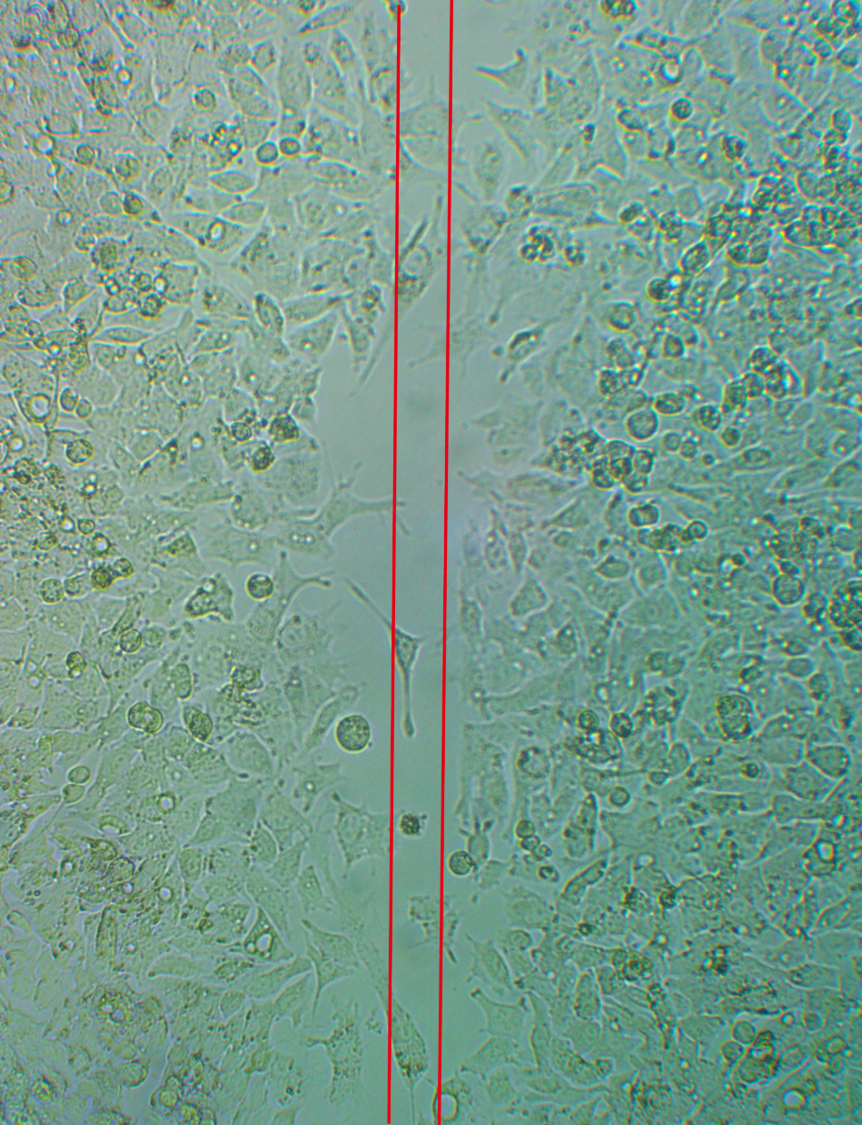

Supplement: Supplementary file 10 [file DataSheet6.ZIP › 48H/plc5-48h-2.jpg]

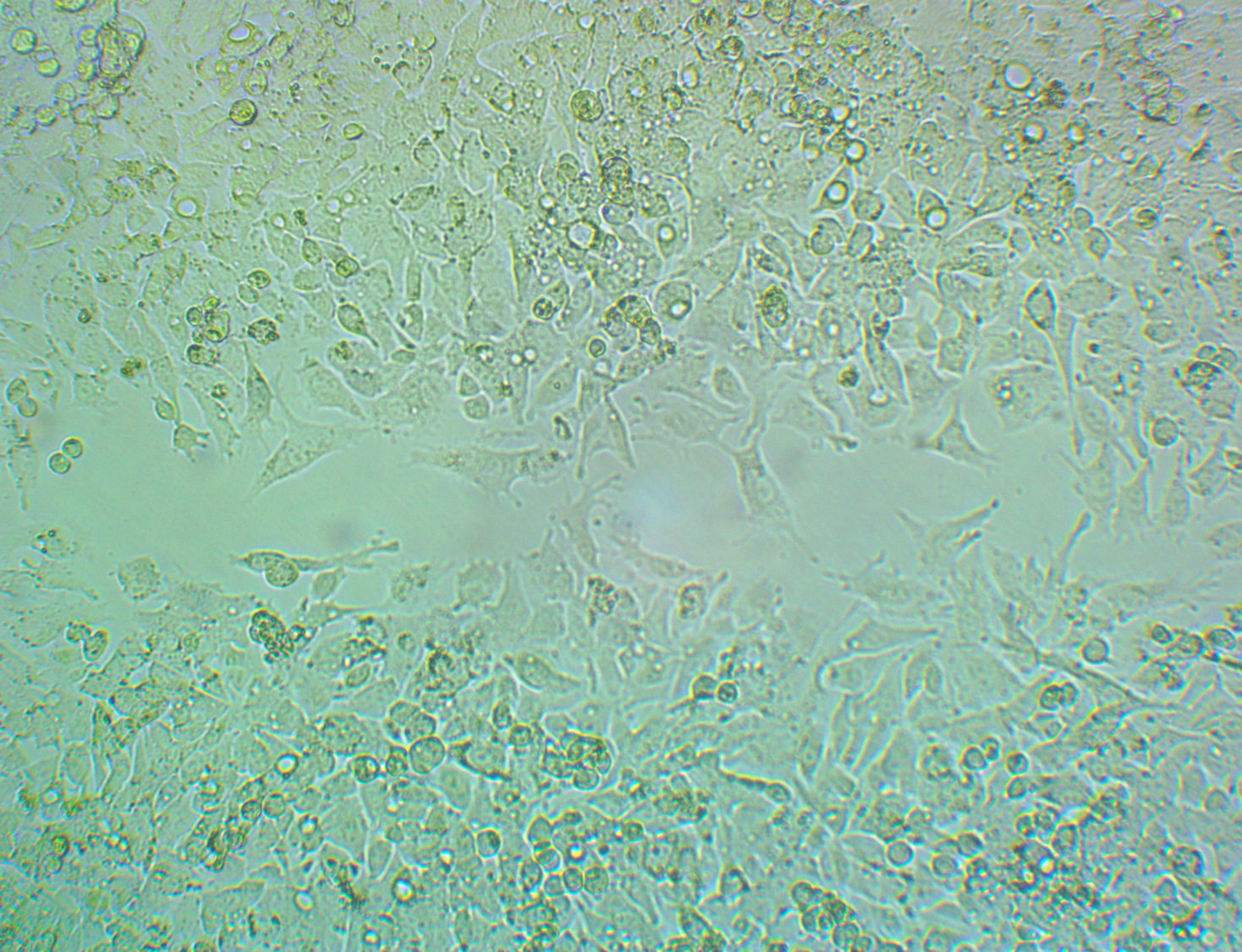

Supplement: Supplementary file 10 [file DataSheet6.ZIP › 48H/plc5-48h-3 raw.jpg]

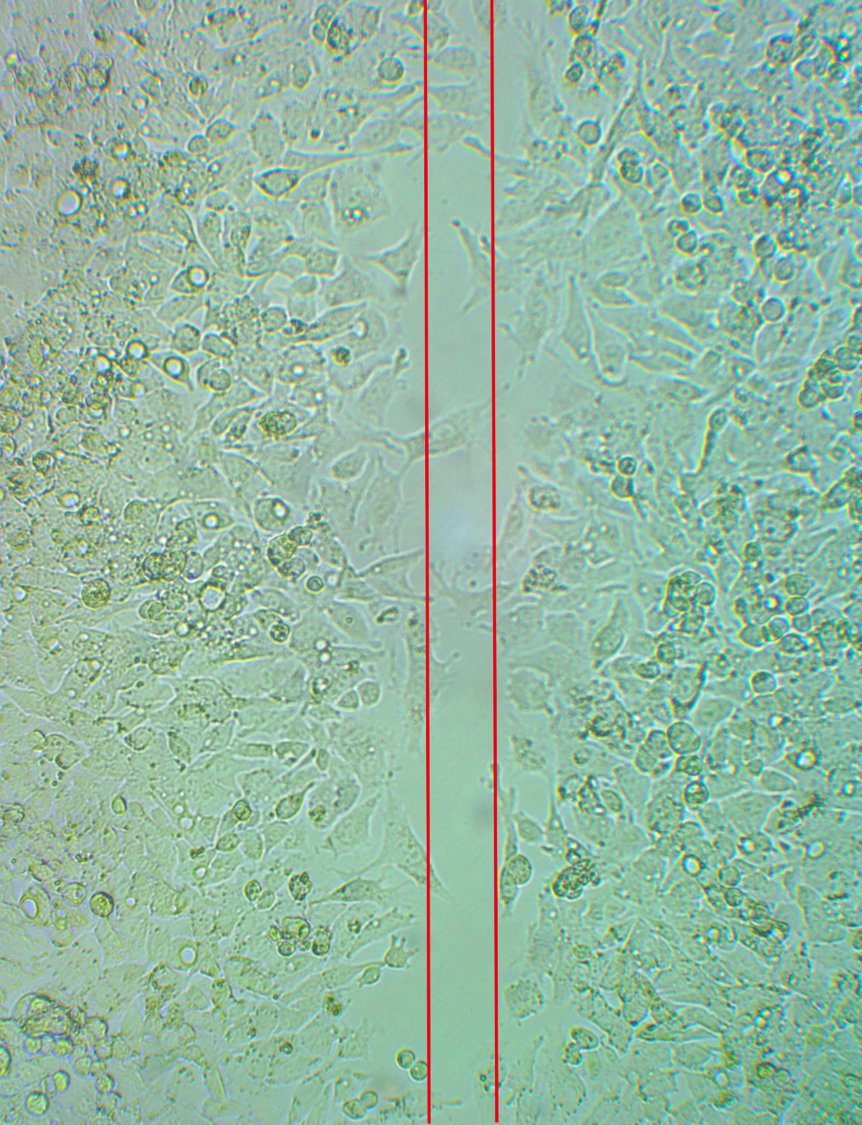

Supplement: Supplementary file 10 [file DataSheet6.ZIP › 48H/plc5-48h-3.jpg]

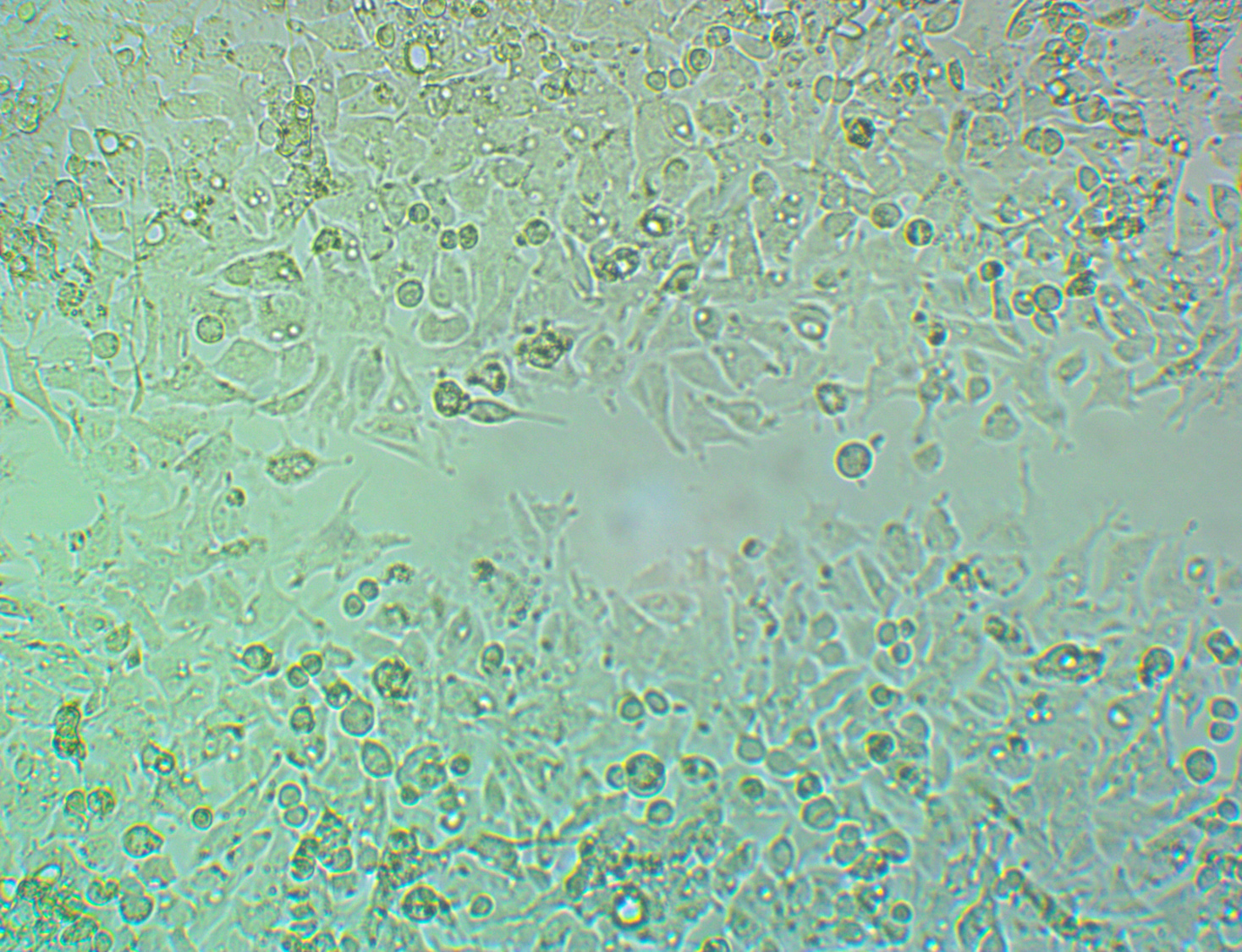

Supplement: Supplementary file 10 [file DataSheet6.ZIP › 48H/plc5-48h-4 raw.jpg]

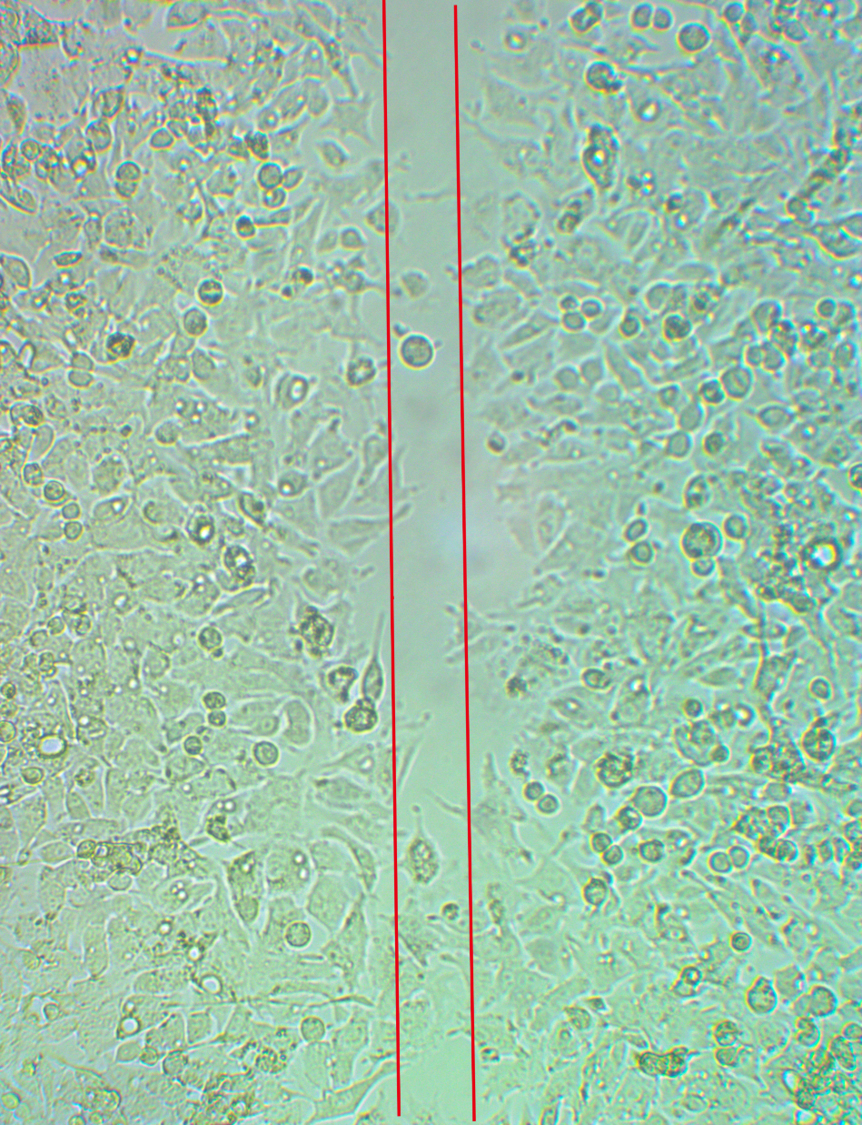

Supplement: Supplementary file 10 [file DataSheet6.ZIP › 48H/plc5-48h-4.jpg]

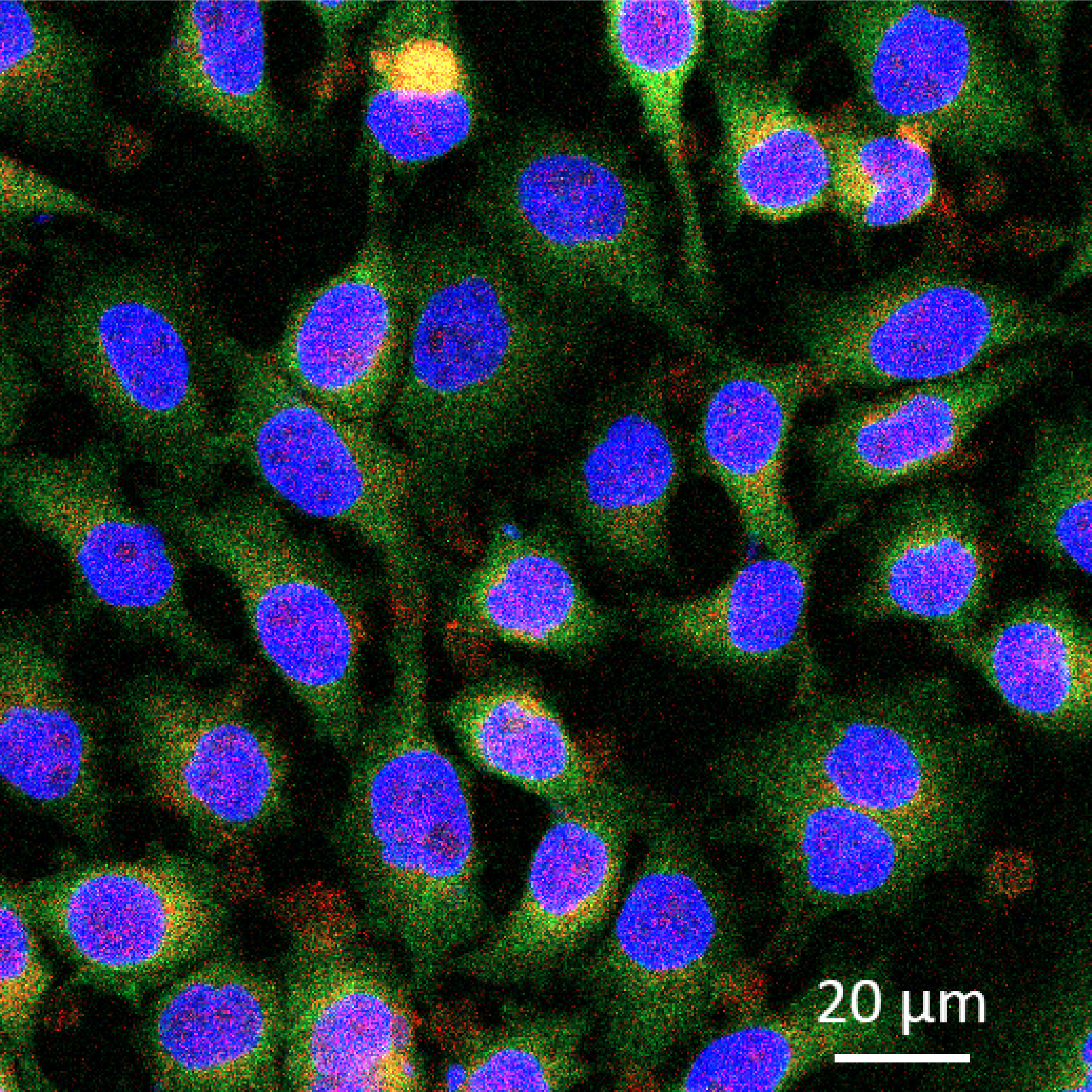

Supplement: Supplementary file 11 [file DataSheet12.ZIP › FISH/LO2/l9-40_c1+2+3.jpg]

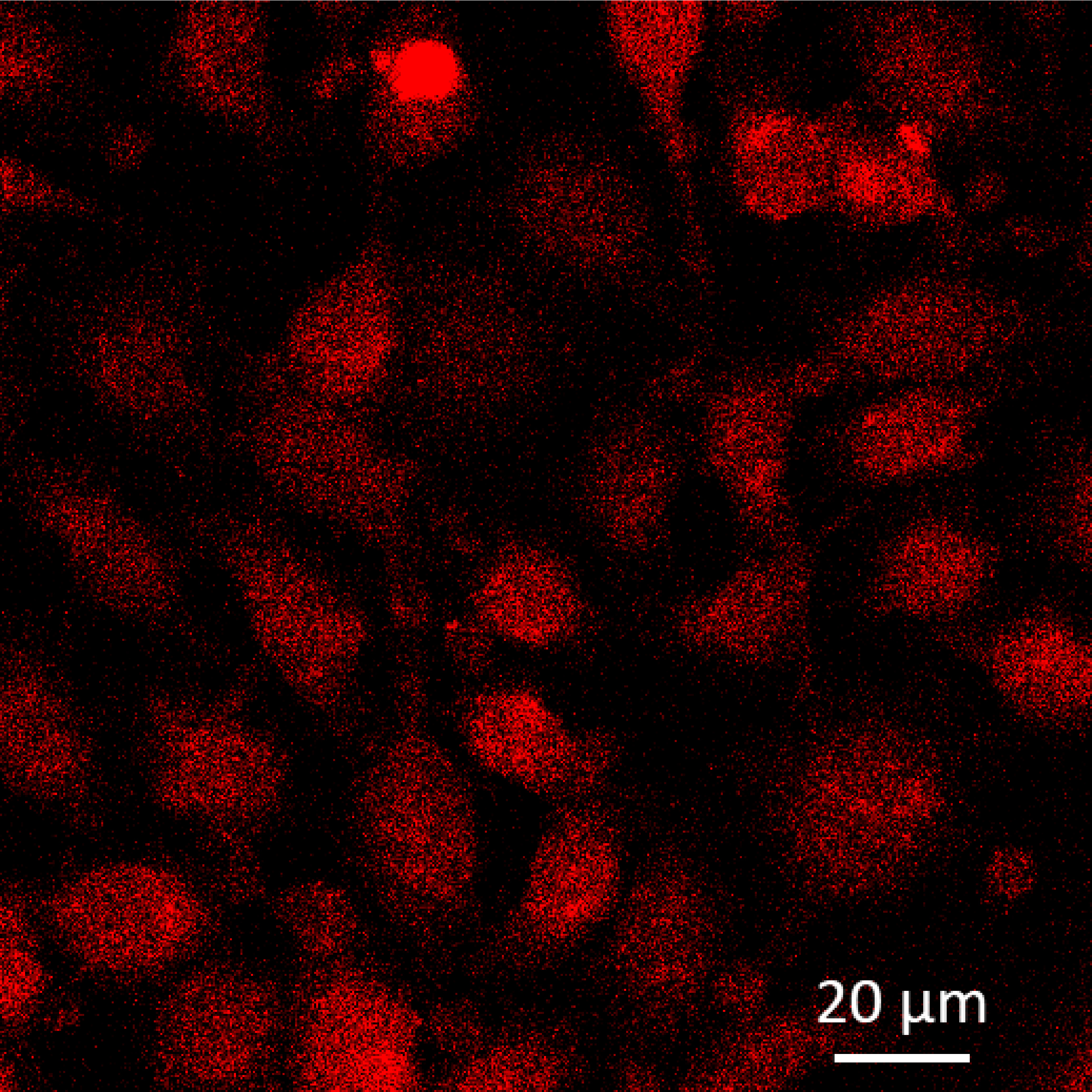

Supplement: Supplementary file 11 [file DataSheet12.ZIP › FISH/LO2/l9-40_c1.jpg]

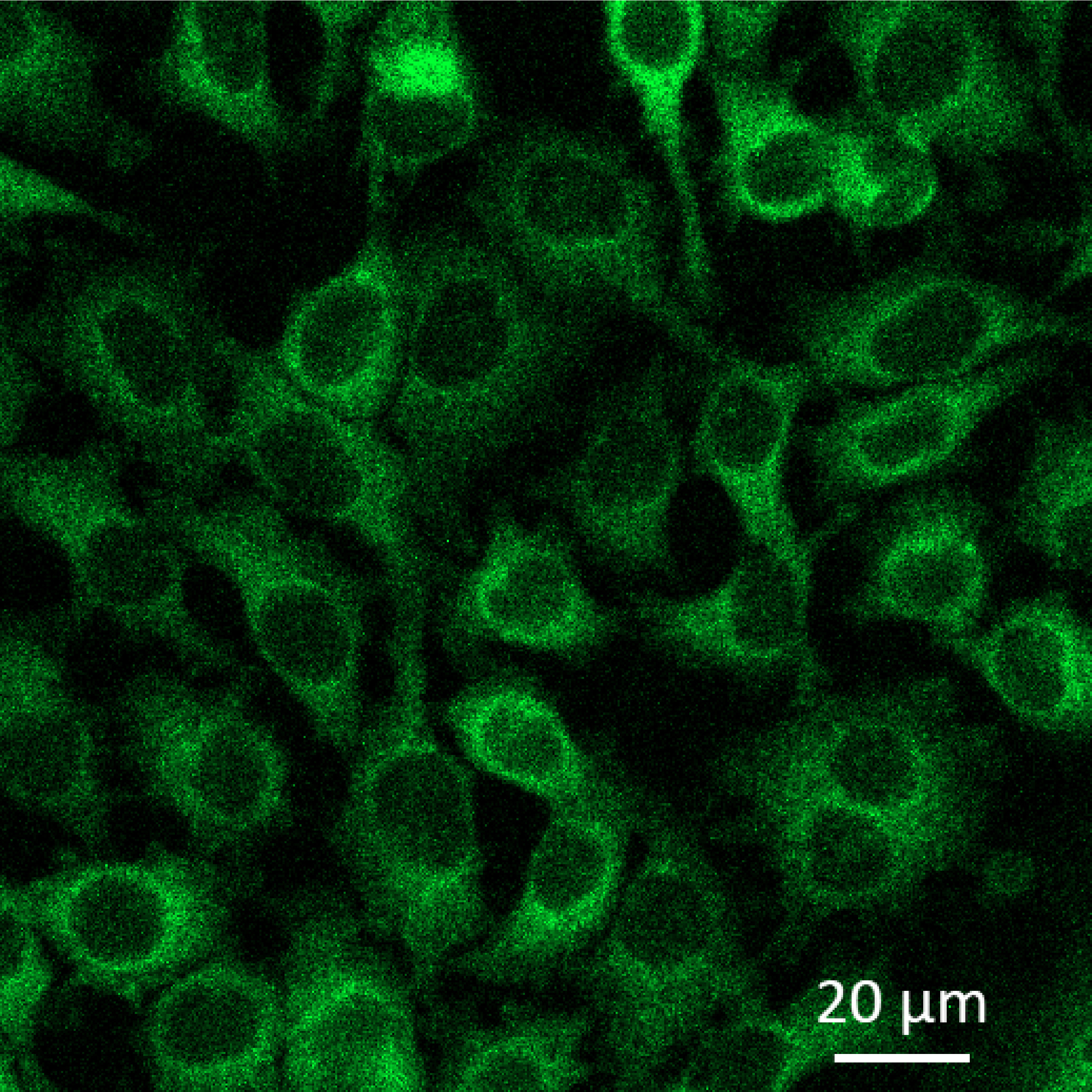

Supplement: Supplementary file 11 [file DataSheet12.ZIP › FISH/LO2/l9-40_c2.jpg]

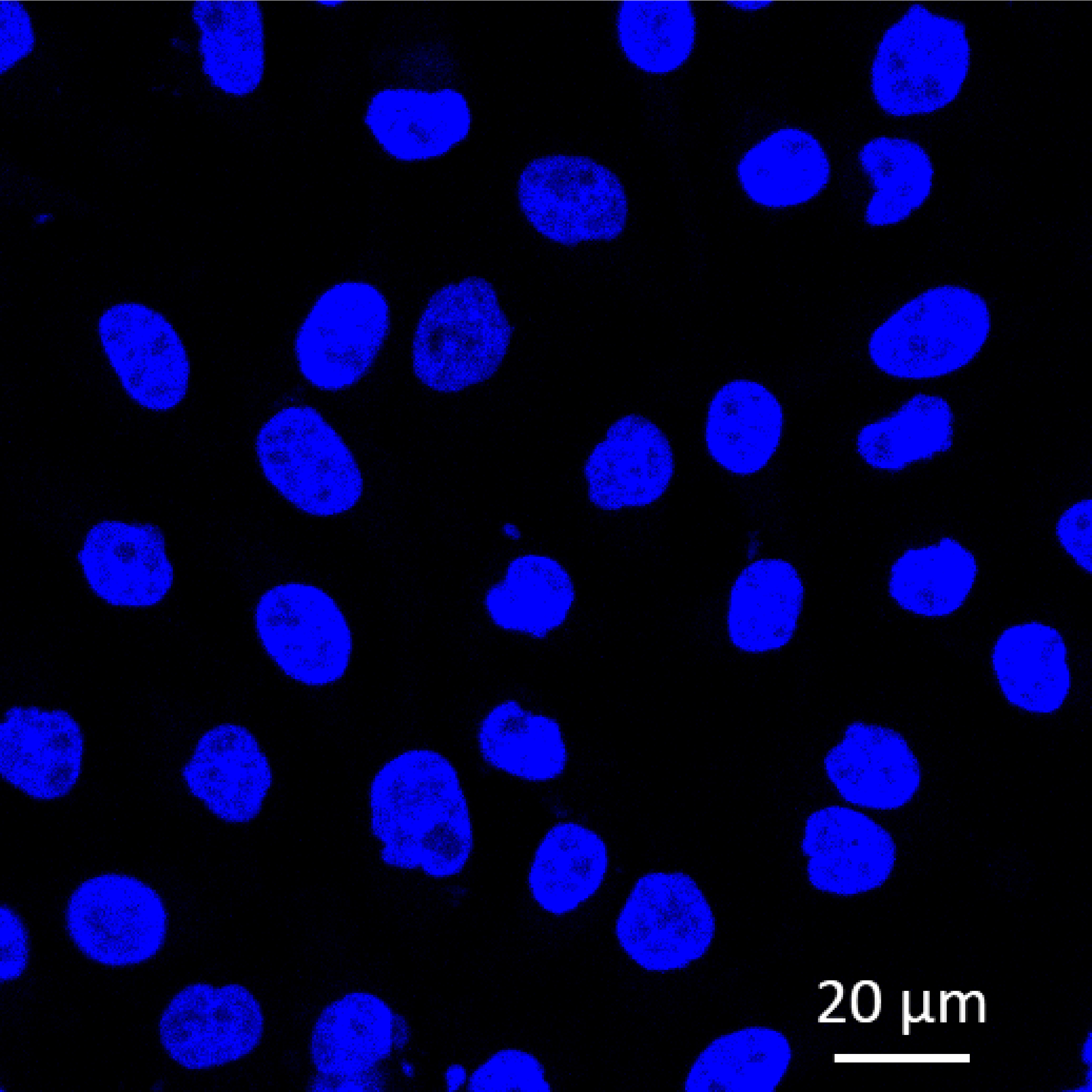

Supplement: Supplementary file 11 [file DataSheet12.ZIP › FISH/LO2/l9-40_c3.jpg]

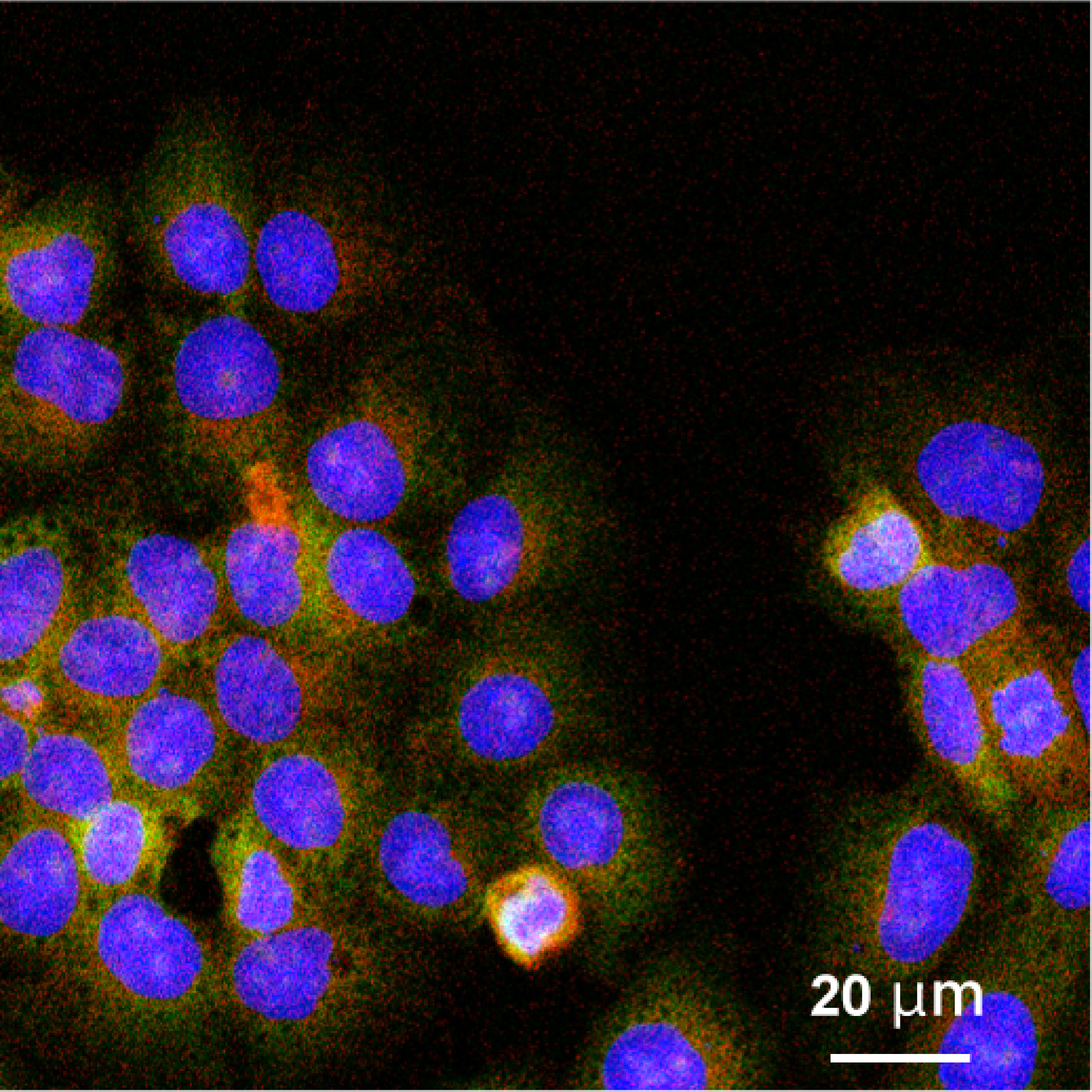

Supplement: Supplementary file 11 [file DataSheet12.ZIP › FISH/SK-Hep-1-circLIFR/C2309 (2)-单个文件导出-02_c1+2+3.jpg]

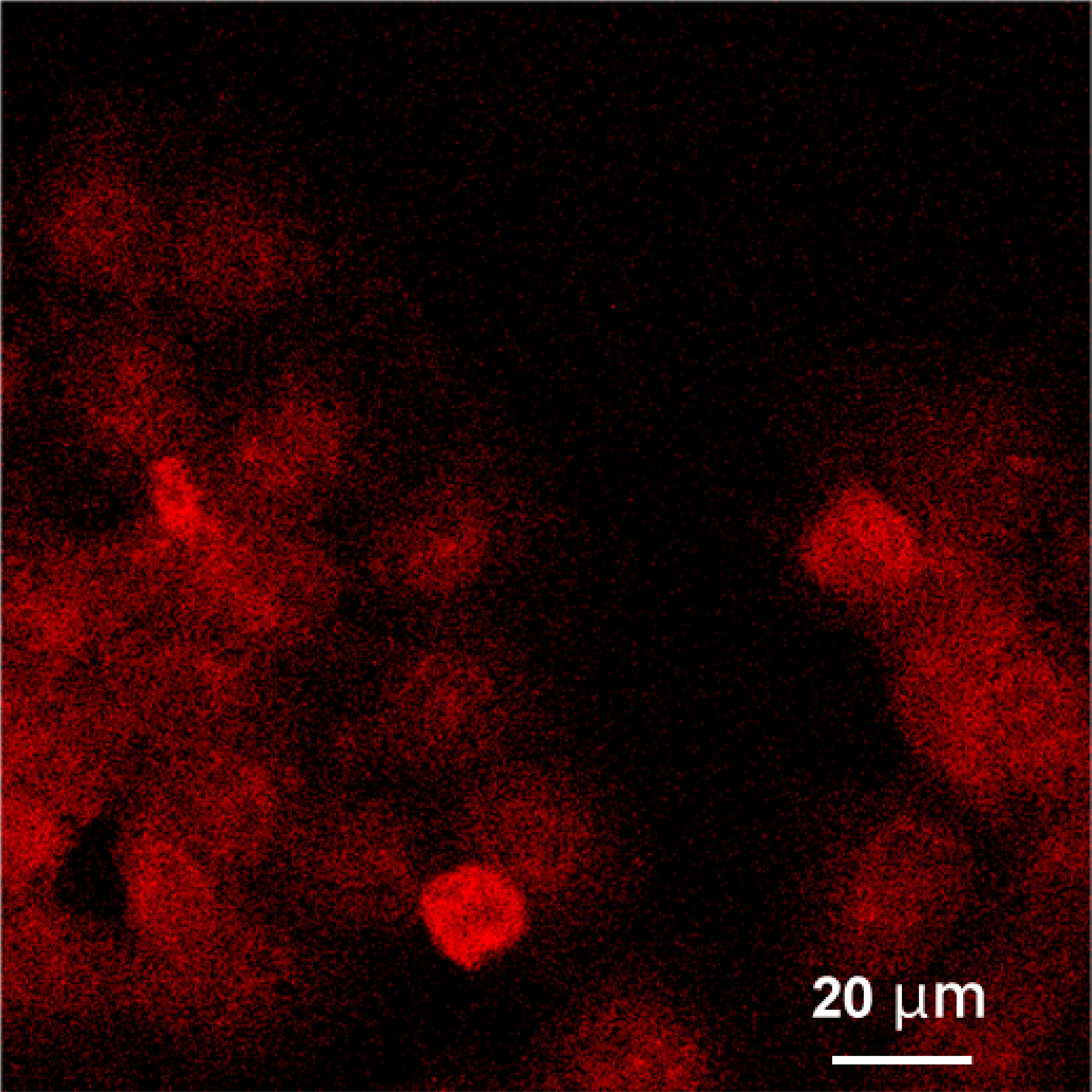

Supplement: Supplementary file 11 [file DataSheet12.ZIP › FISH/SK-Hep-1-circLIFR/C2309 (2)-单个文件导出-02_c1.jpg]

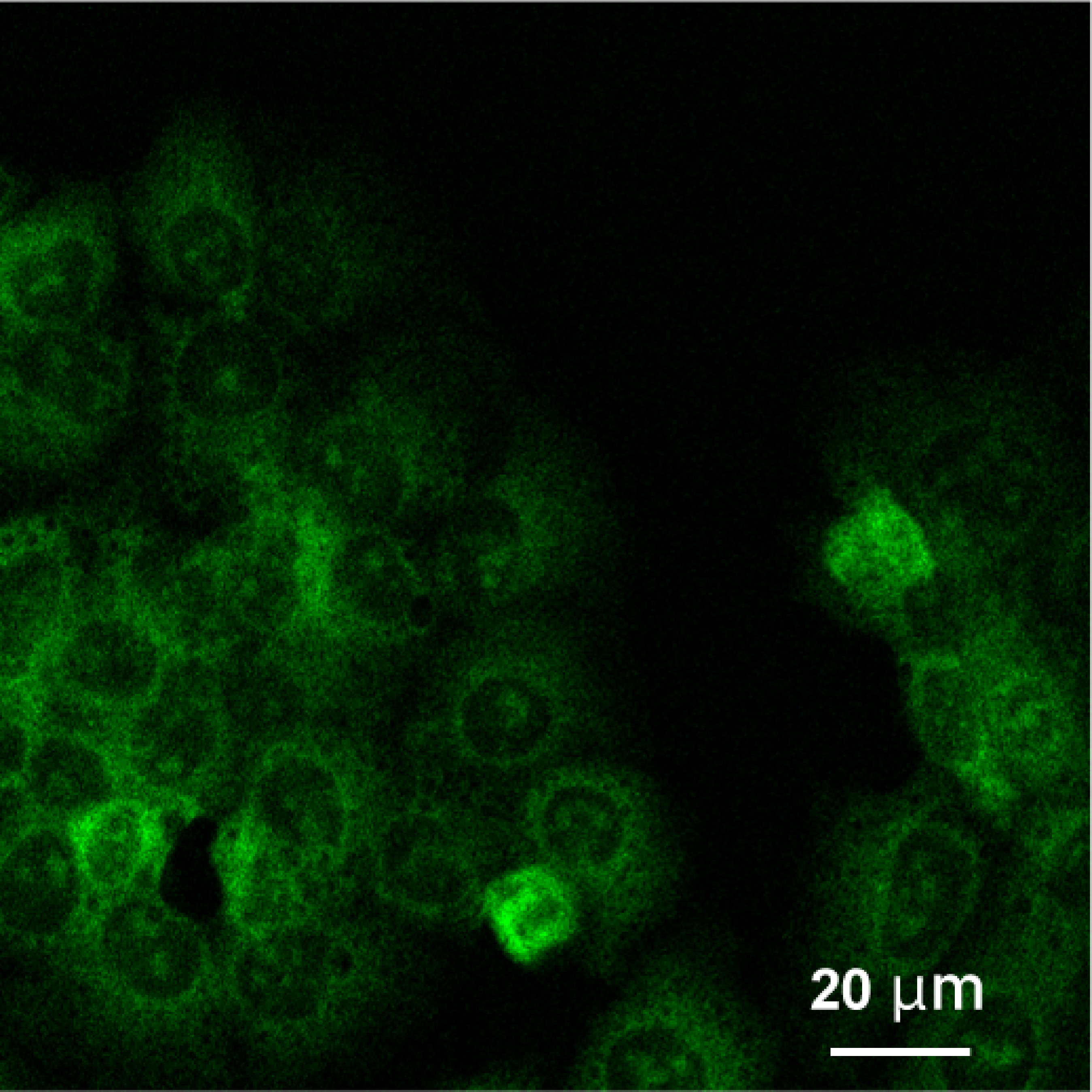

Supplement: Supplementary file 11 [file DataSheet12.ZIP › FISH/SK-Hep-1-circLIFR/C2309 (2)-单个文件导出-02_c2.jpg]

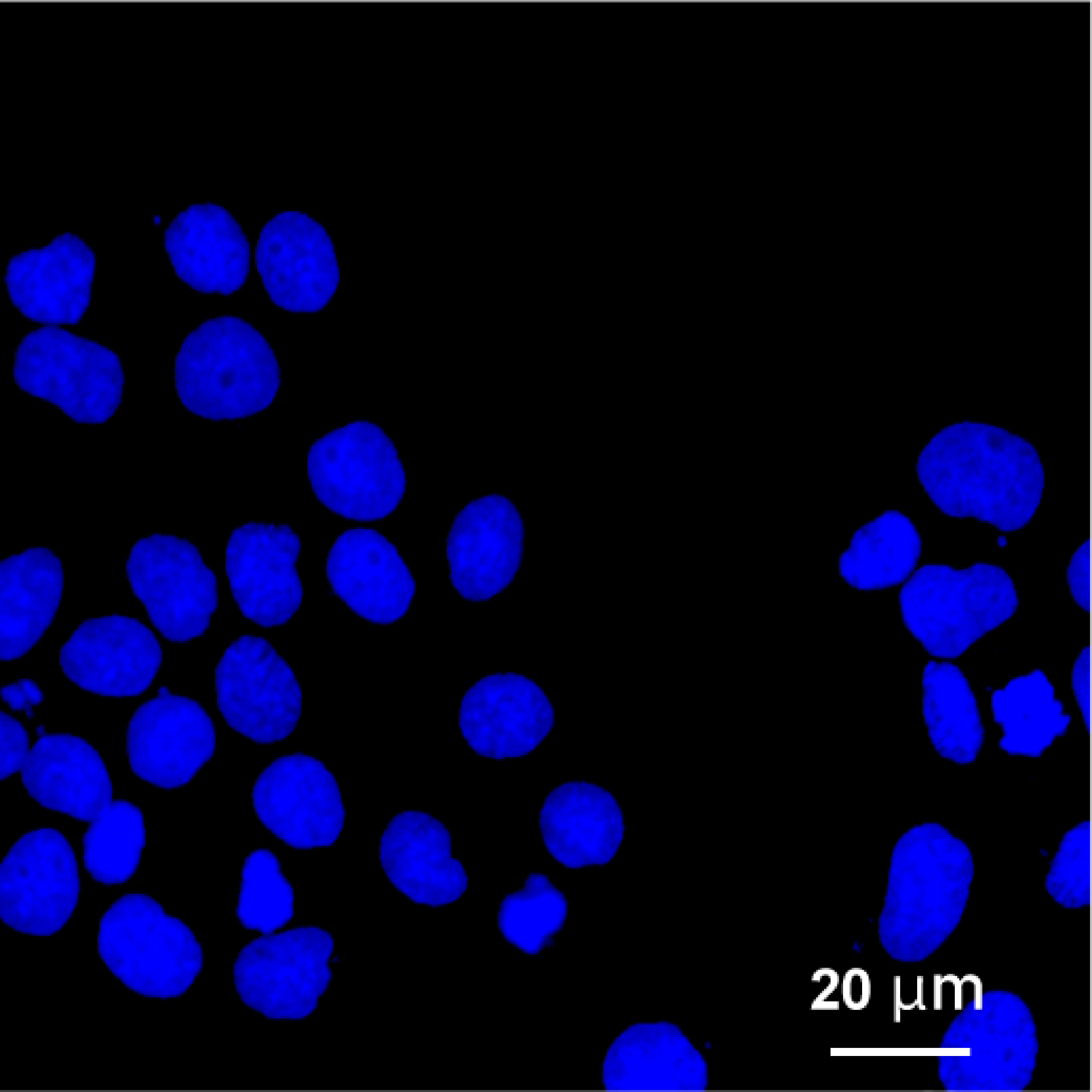

Supplement: Supplementary file 11 [file DataSheet12.ZIP › FISH/SK-Hep-1-circLIFR/C2309 (2)-单个文件导出-02_c3.jpg]

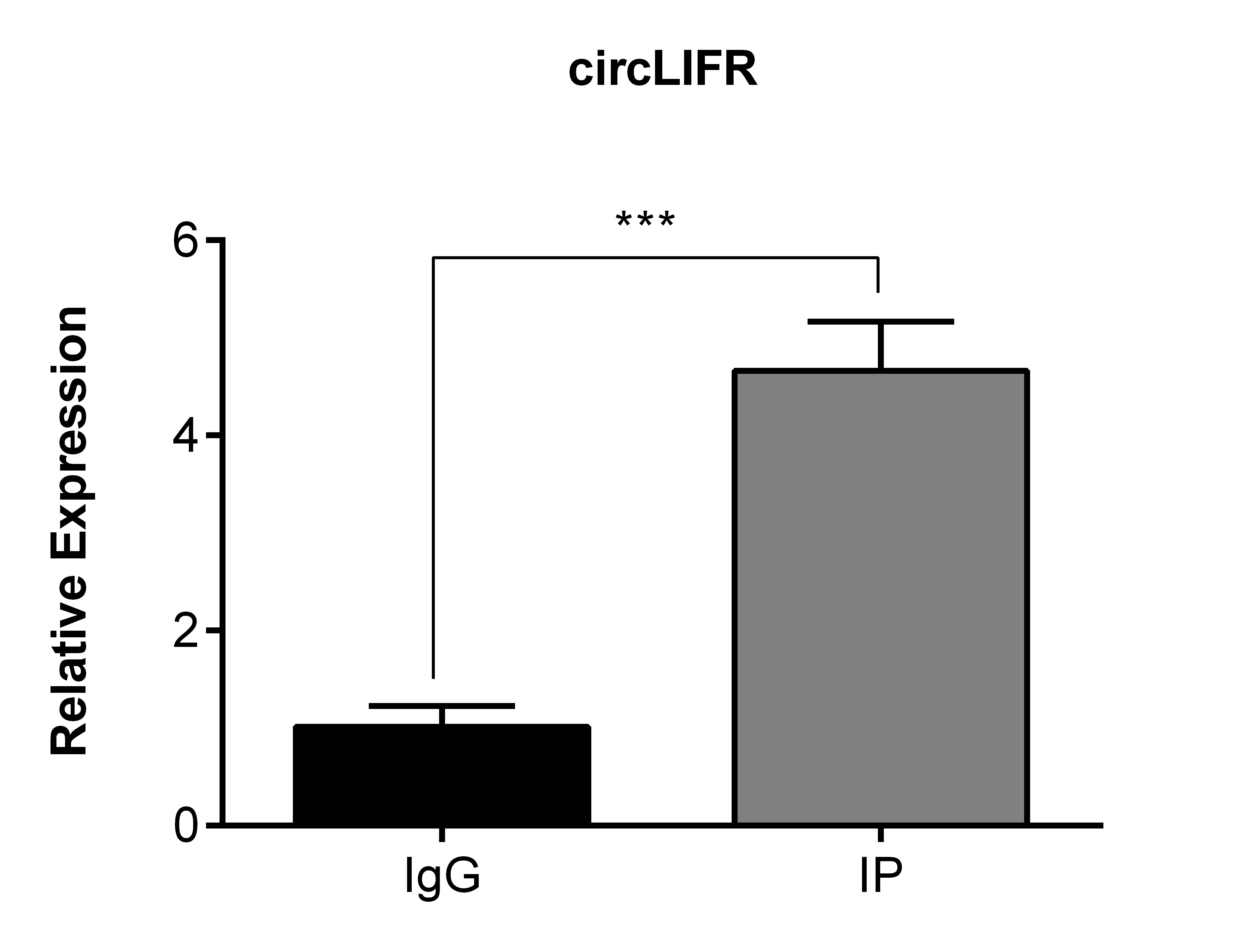

Supplement: Supplementary file 11 [file DataSheet12.ZIP › RIP.jpg]

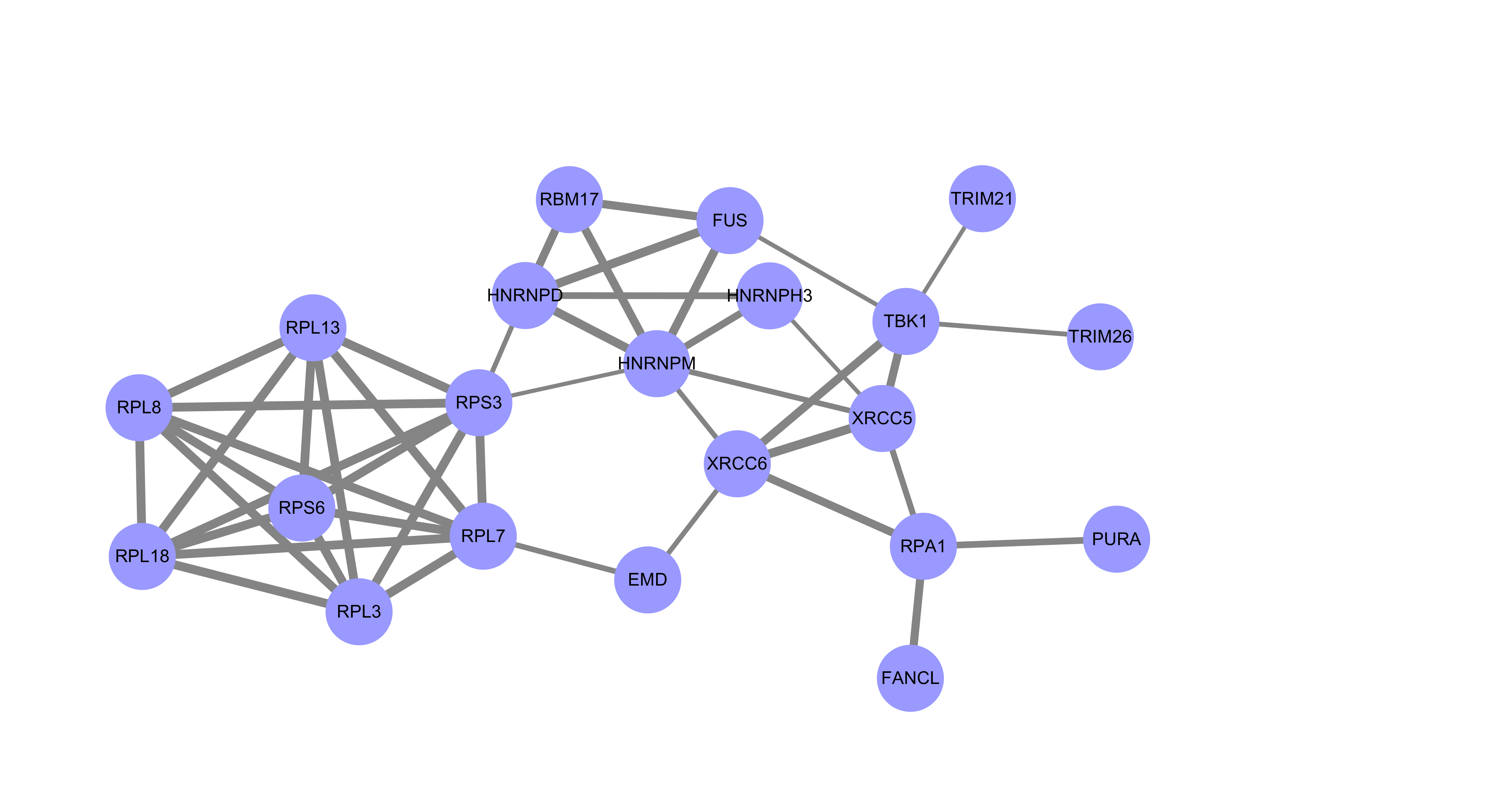

Supplement: Supplementary file 11 [file DataSheet12.ZIP › string_interactions_ppi.png]

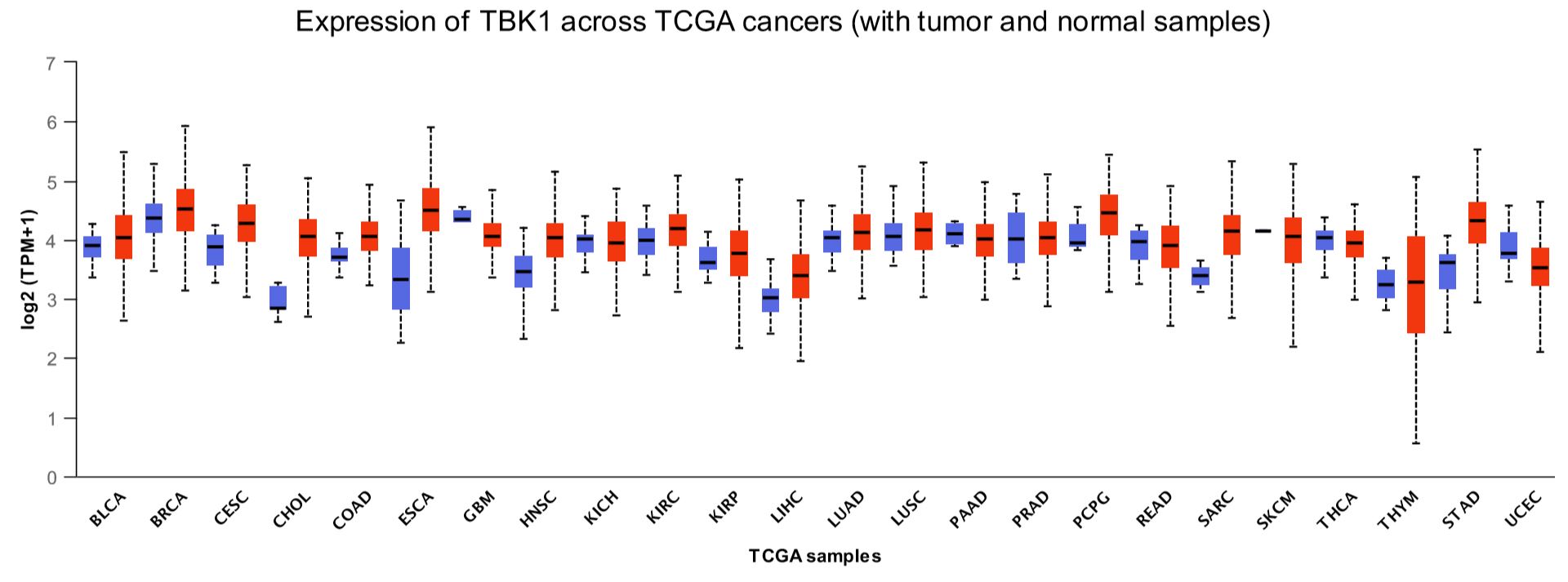

Supplement: Supplementary file 11 [file DataSheet12.ZIP › tbk1.JPG]

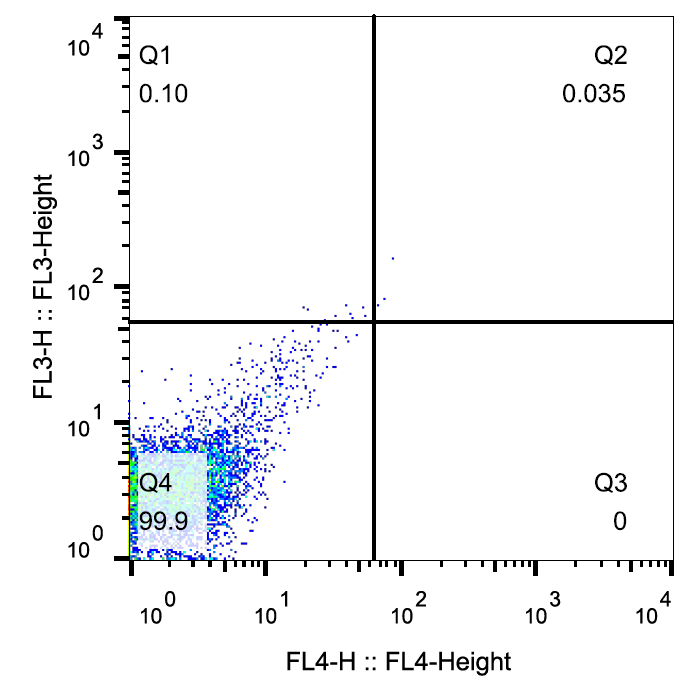

Supplement: Supplementary file 12 [file DataSheet2.ZIP › Cell function experiment/cell apoptosis assay/hep-G2 cell/hepg2 0.tif]

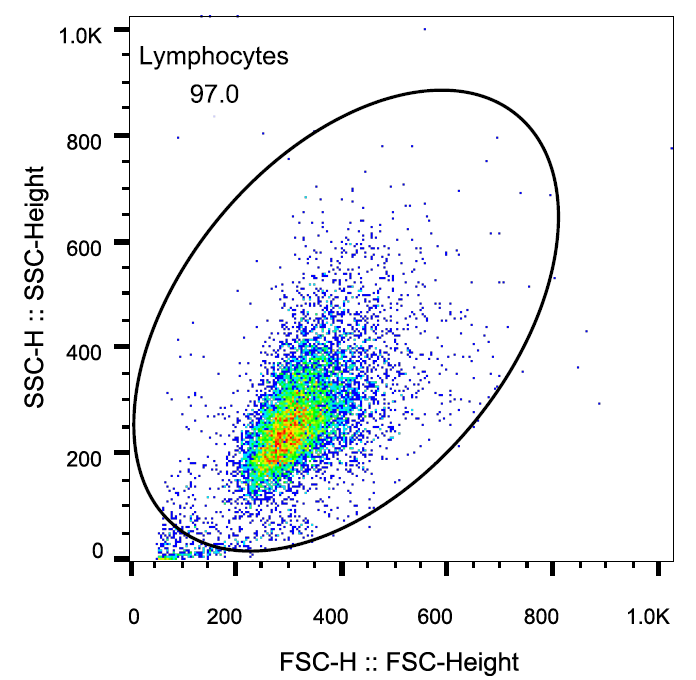

Supplement: Supplementary file 12 [file DataSheet2.ZIP › Cell function experiment/cell apoptosis assay/hep-G2 cell/hepg2 C.tif]

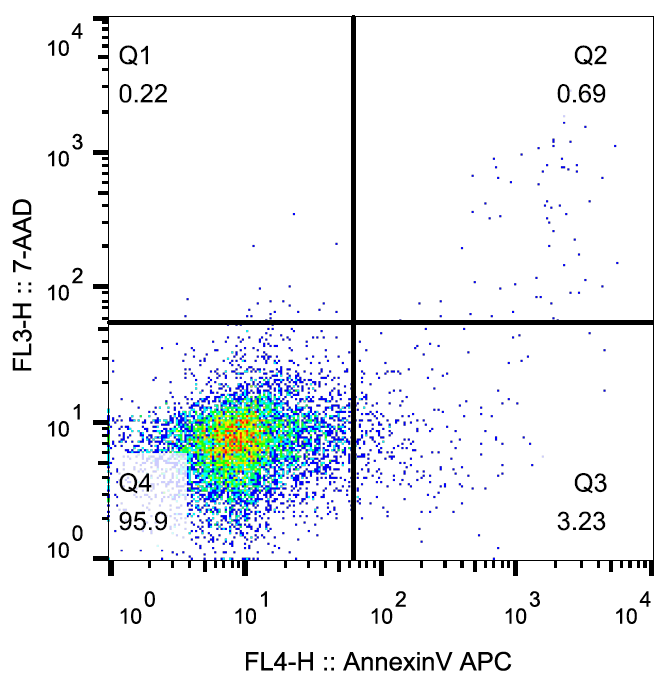

Supplement: Supplementary file 12 [file DataSheet2.ZIP › Cell function experiment/cell apoptosis assay/hep-G2 cell/hepg2 C-1.tif]

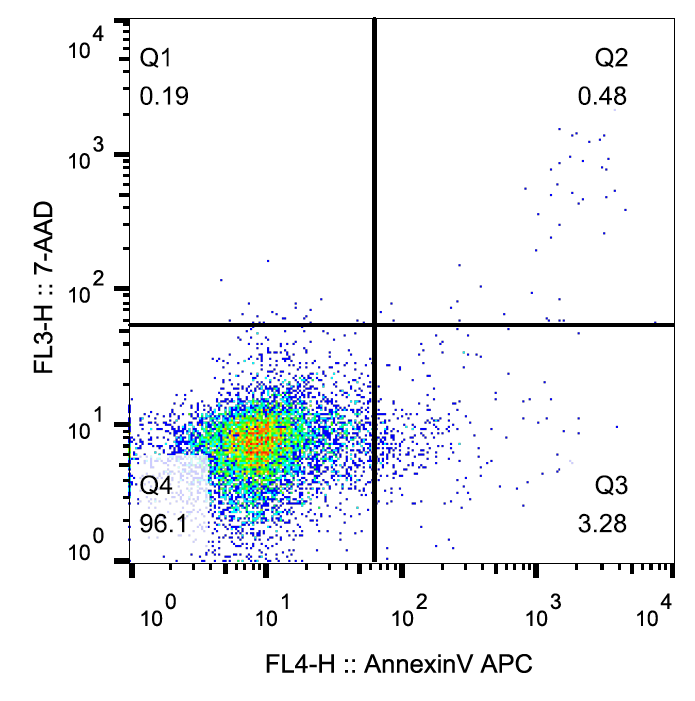

Supplement: Supplementary file 12 [file DataSheet2.ZIP › Cell function experiment/cell apoptosis assay/hep-G2 cell/hepg2 C-2.tif]

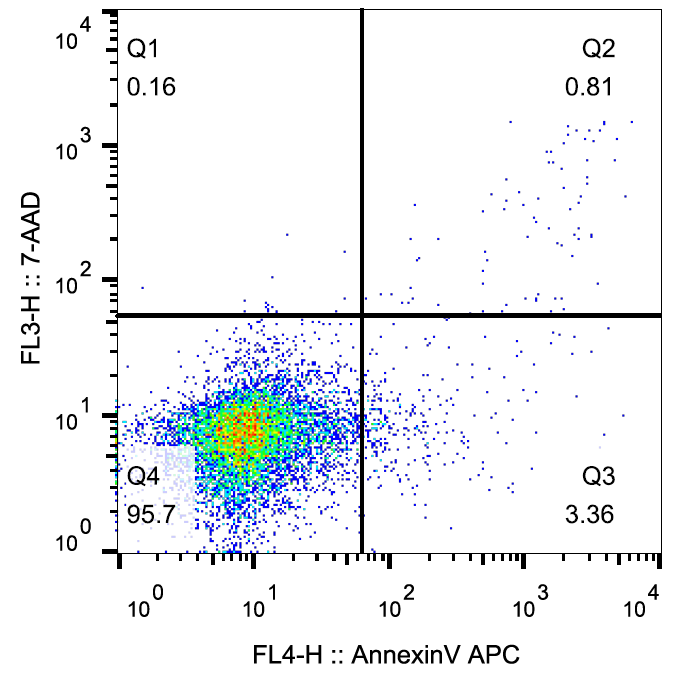

Supplement: Supplementary file 12 [file DataSheet2.ZIP › Cell function experiment/cell apoptosis assay/hep-G2 cell/hepg2 C-3.tif]

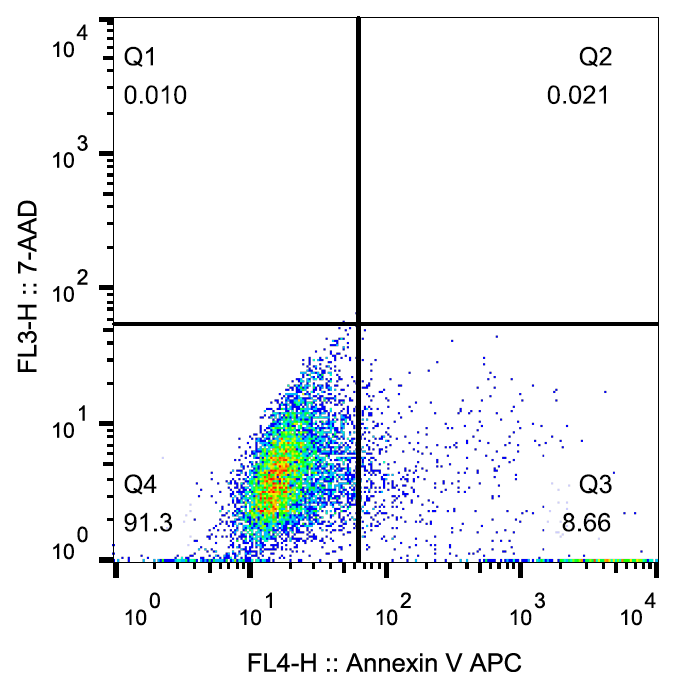

Supplement: Supplementary file 12 [file DataSheet2.ZIP › Cell function experiment/cell apoptosis assay/hep-G2 cell/hepg2 cell-1.tif]

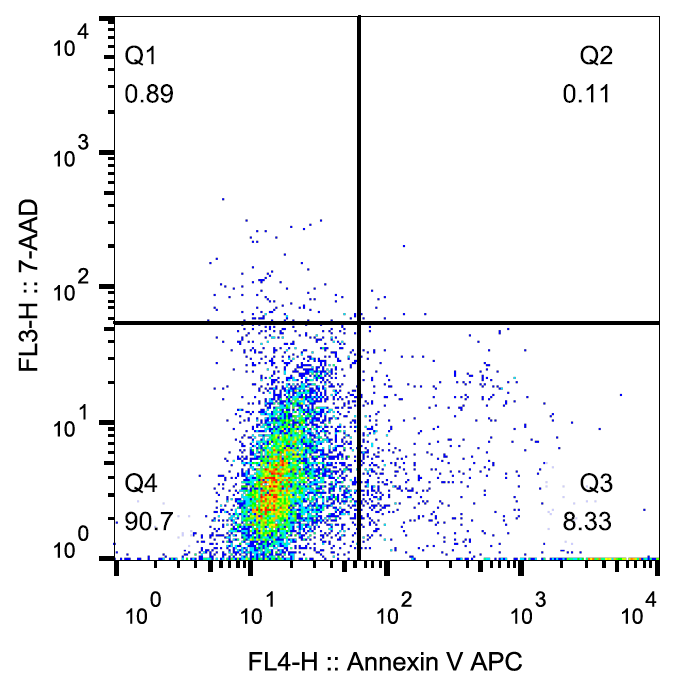

Supplement: Supplementary file 12 [file DataSheet2.ZIP › Cell function experiment/cell apoptosis assay/hep-G2 cell/hepg2 cell-2.tif]

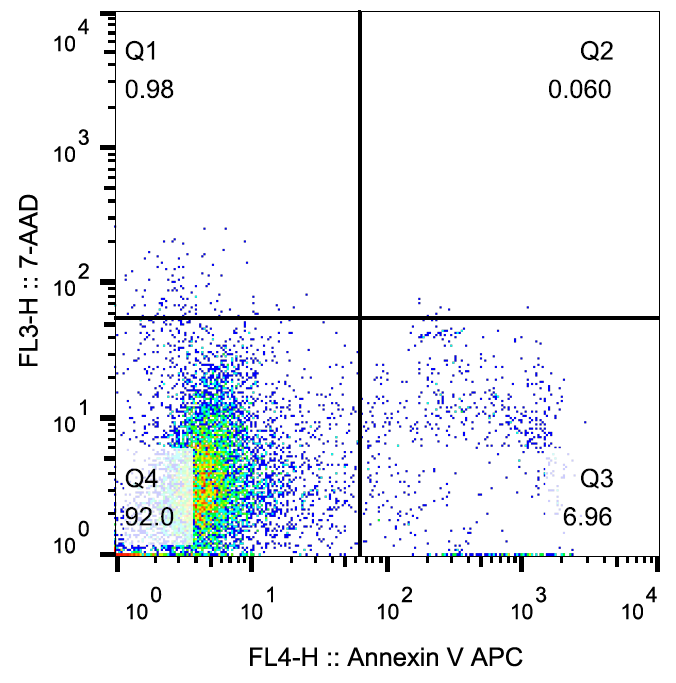

Supplement: Supplementary file 12 [file DataSheet2.ZIP › Cell function experiment/cell apoptosis assay/hep-G2 cell/hepg2 cell-3.tif]

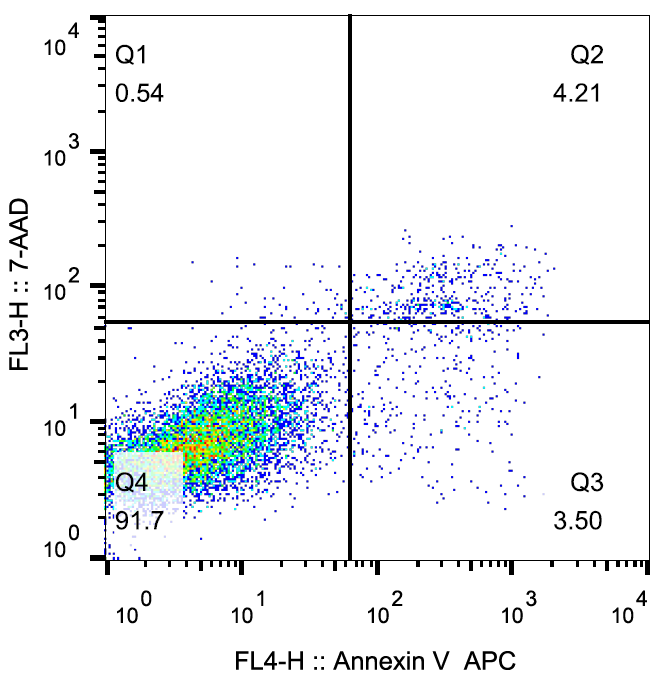

Supplement: Supplementary file 12 [file DataSheet2.ZIP › Cell function experiment/cell apoptosis assay/hep-G2 cell/hepg2 nc-1.tif]

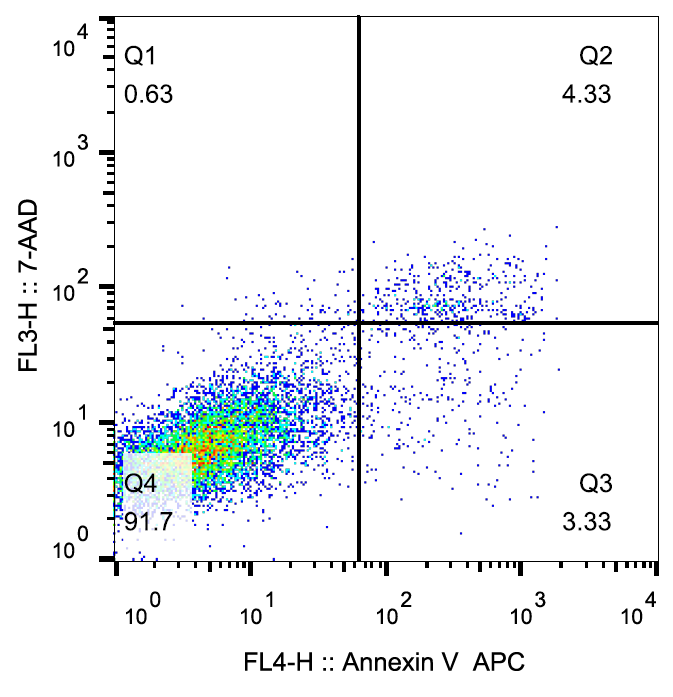

Supplement: Supplementary file 12 [file DataSheet2.ZIP › Cell function experiment/cell apoptosis assay/hep-G2 cell/hepg2 nc-2.tif]

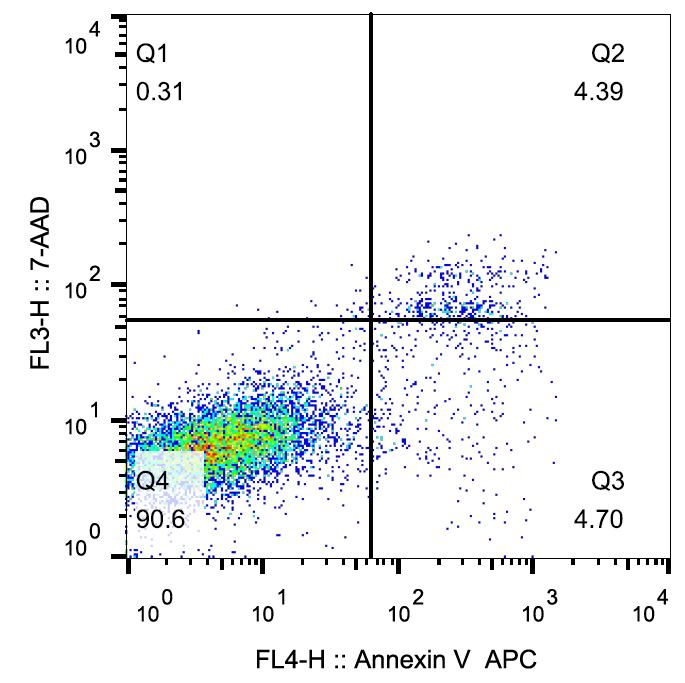

Supplement: Supplementary file 12 [file DataSheet2.ZIP › Cell function experiment/cell apoptosis assay/hep-G2 cell/hepg2 nc-3.tif]

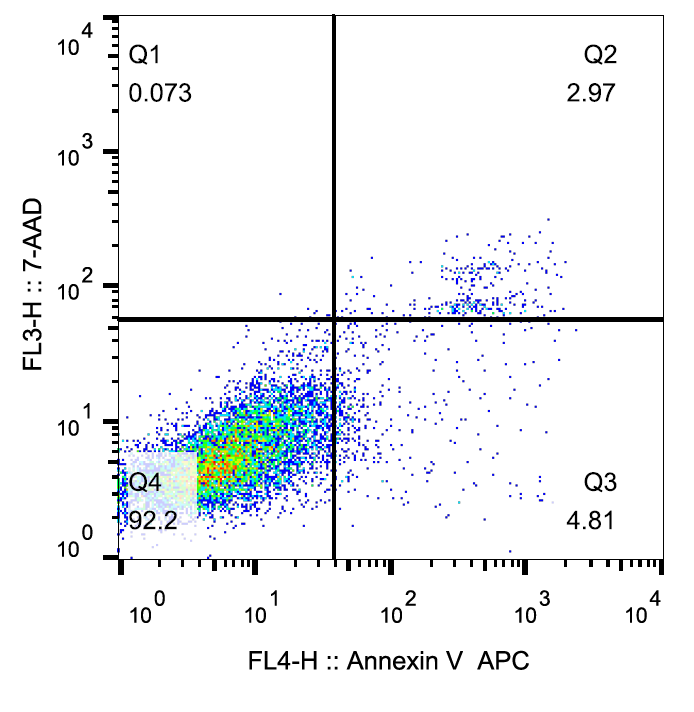

Supplement: Supplementary file 12 [file DataSheet2.ZIP › Cell function experiment/cell apoptosis assay/SK-Hep-1 cell/SK C-1.tif]

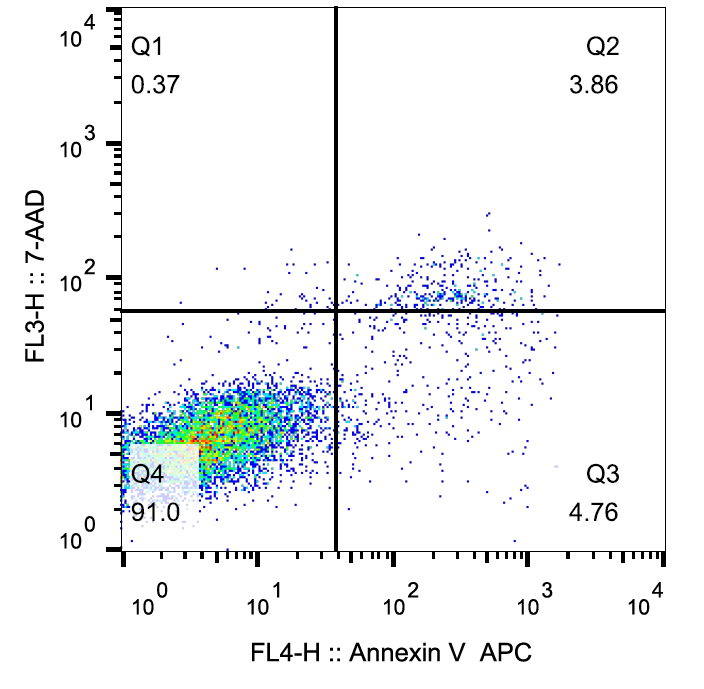

Supplement: Supplementary file 12 [file DataSheet2.ZIP › Cell function experiment/cell apoptosis assay/SK-Hep-1 cell/SK C-2.tif]

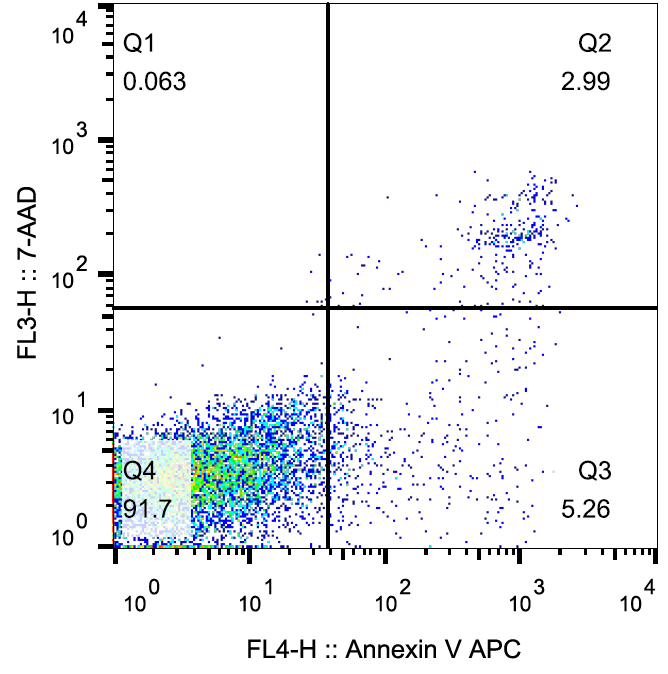

Supplement: Supplementary file 12 [file DataSheet2.ZIP › Cell function experiment/cell apoptosis assay/SK-Hep-1 cell/SK C-3.tif]

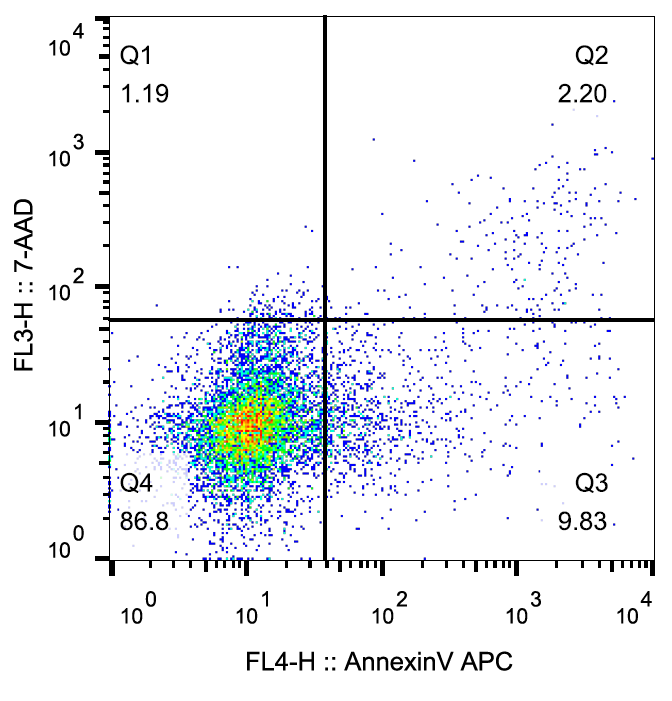

Supplement: Supplementary file 12 [file DataSheet2.ZIP › Cell function experiment/cell apoptosis assay/SK-Hep-1 cell/SK cell-1.tif]

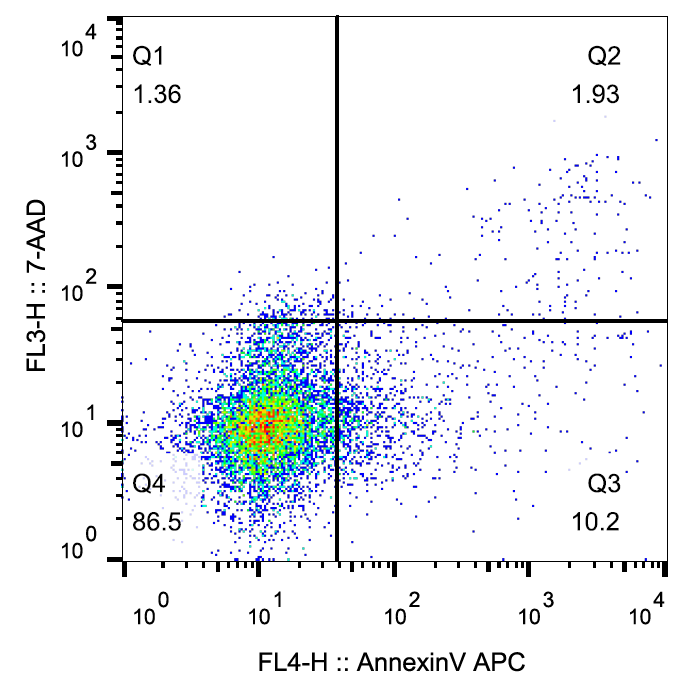

Supplement: Supplementary file 12 [file DataSheet2.ZIP › Cell function experiment/cell apoptosis assay/SK-Hep-1 cell/SK cell-2.tif]

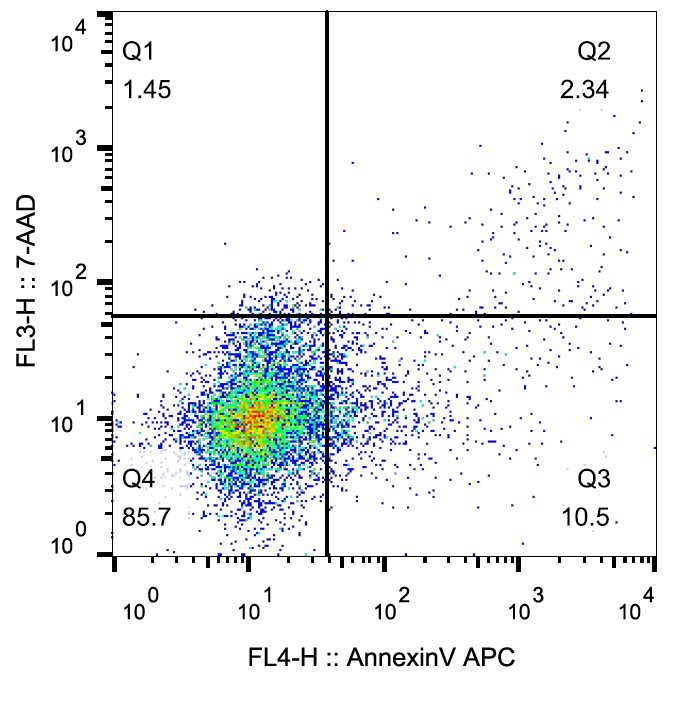

Supplement: Supplementary file 12 [file DataSheet2.ZIP › Cell function experiment/cell apoptosis assay/SK-Hep-1 cell/SK cell-3.tif]

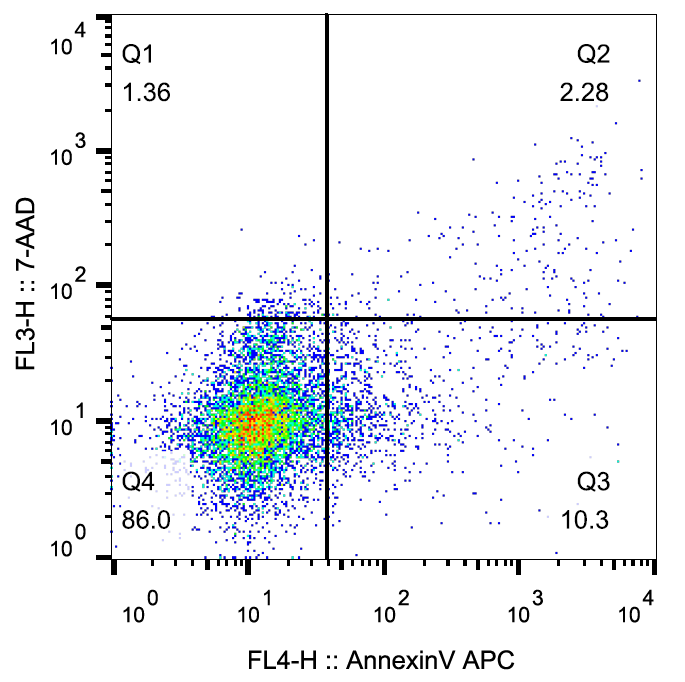

Supplement: Supplementary file 12 [file DataSheet2.ZIP › Cell function experiment/cell apoptosis assay/SK-Hep-1 cell/SK NC-1.tif]

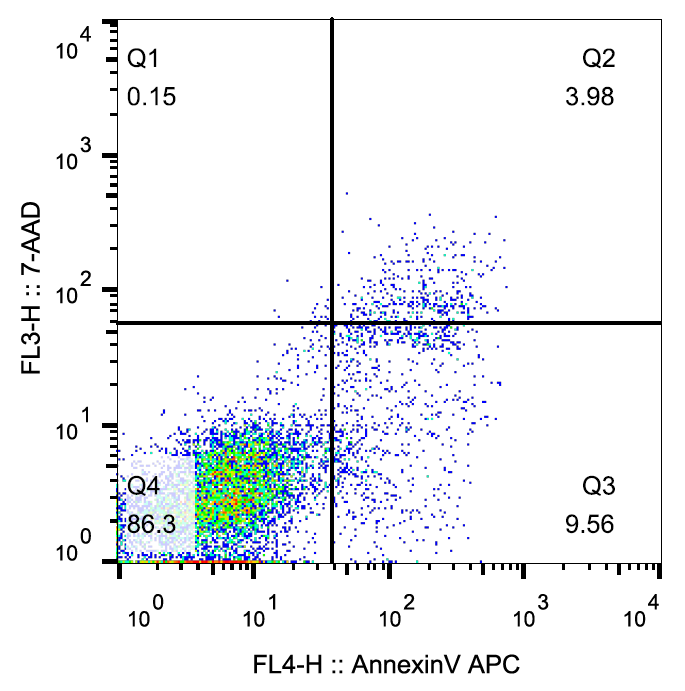

Supplement: Supplementary file 12 [file DataSheet2.ZIP › Cell function experiment/cell apoptosis assay/SK-Hep-1 cell/SK NC-2.tif]

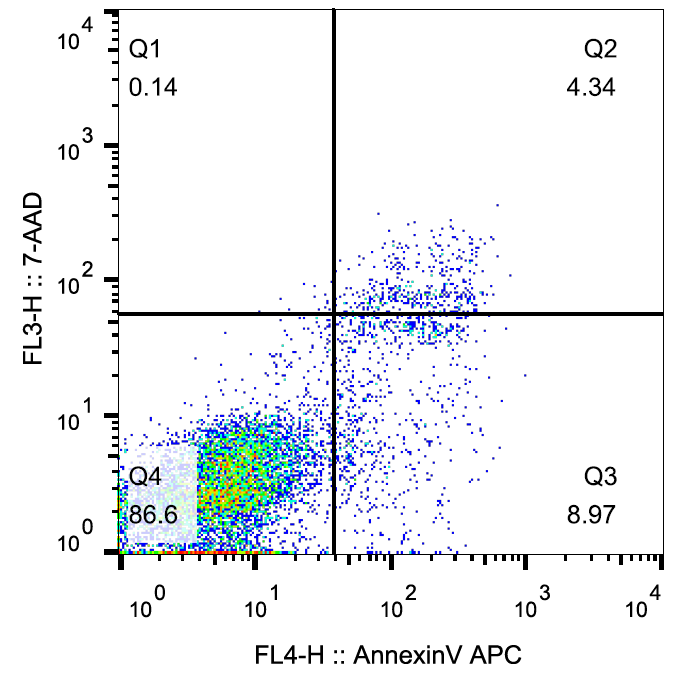

Supplement: Supplementary file 12 [file DataSheet2.ZIP › Cell function experiment/cell apoptosis assay/SK-Hep-1 cell/SK NC-3.tif]

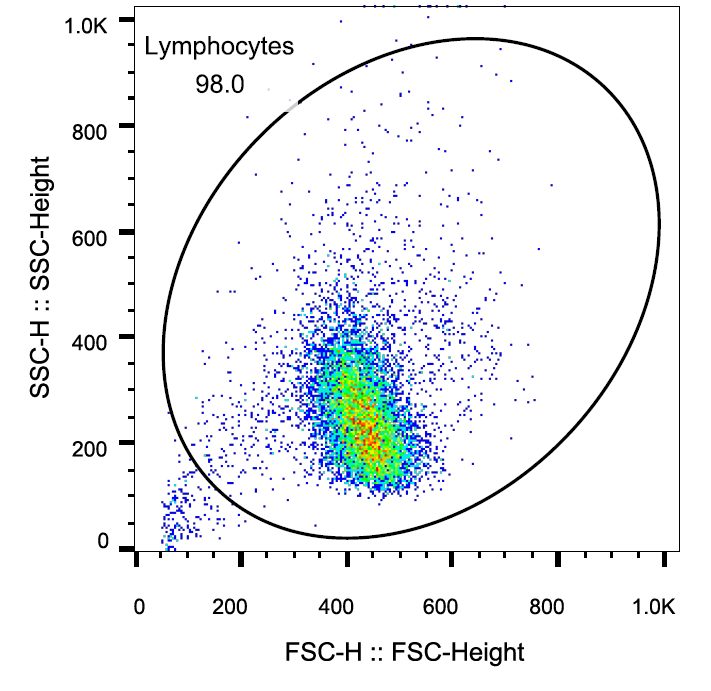

Supplement: Supplementary file 12 [file DataSheet2.ZIP › Cell function experiment/cell apoptosis assay/SK-Hep-1 cell/SK-0.tif]

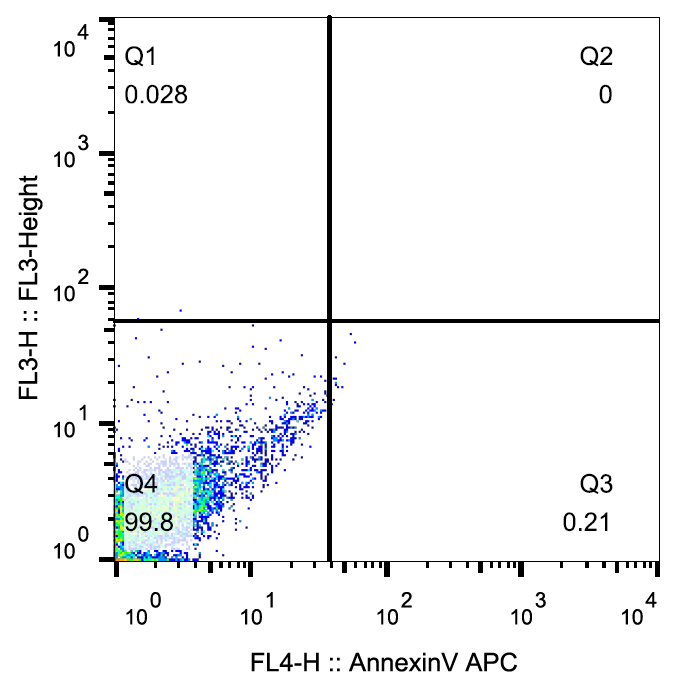

Supplement: Supplementary file 12 [file DataSheet2.ZIP › Cell function experiment/cell apoptosis assay/SK-Hep-1 cell/SK-C.tif]

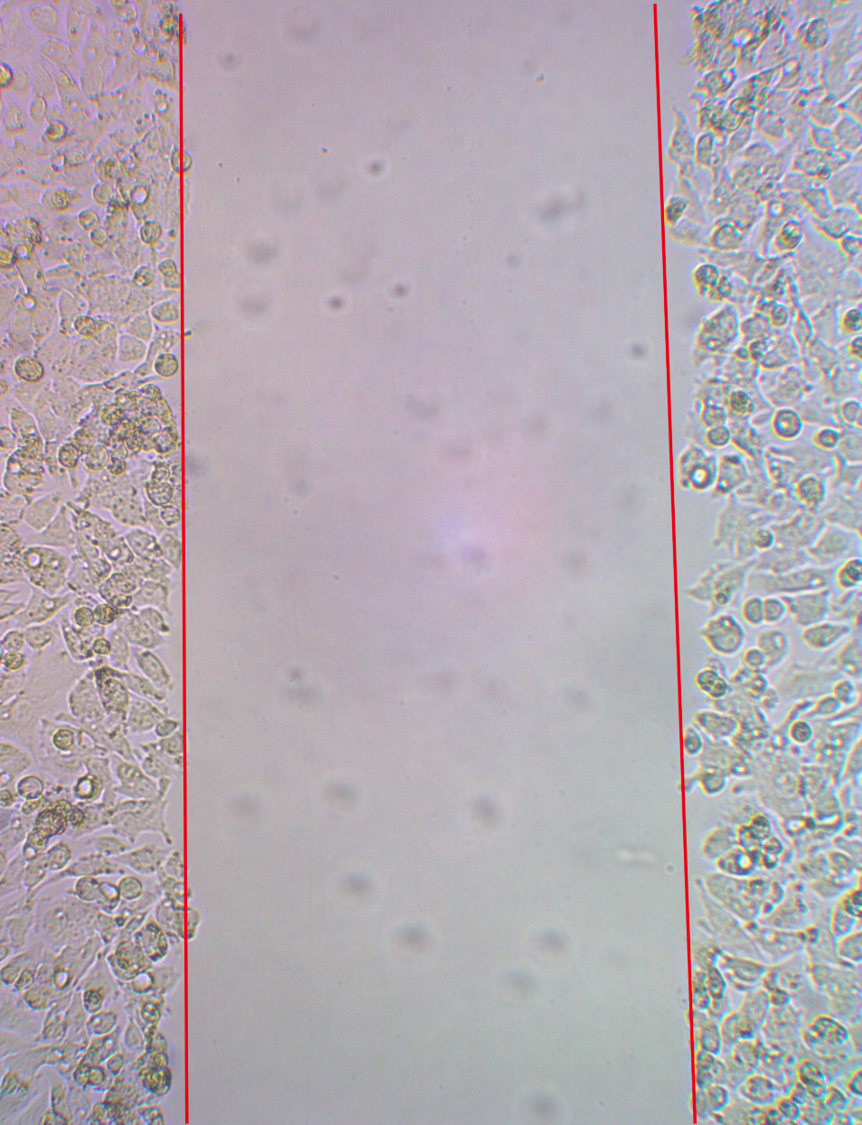

Supplement: Supplementary file 13 [file DataSheet5.ZIP › 0H/control-0h-1.jpg]

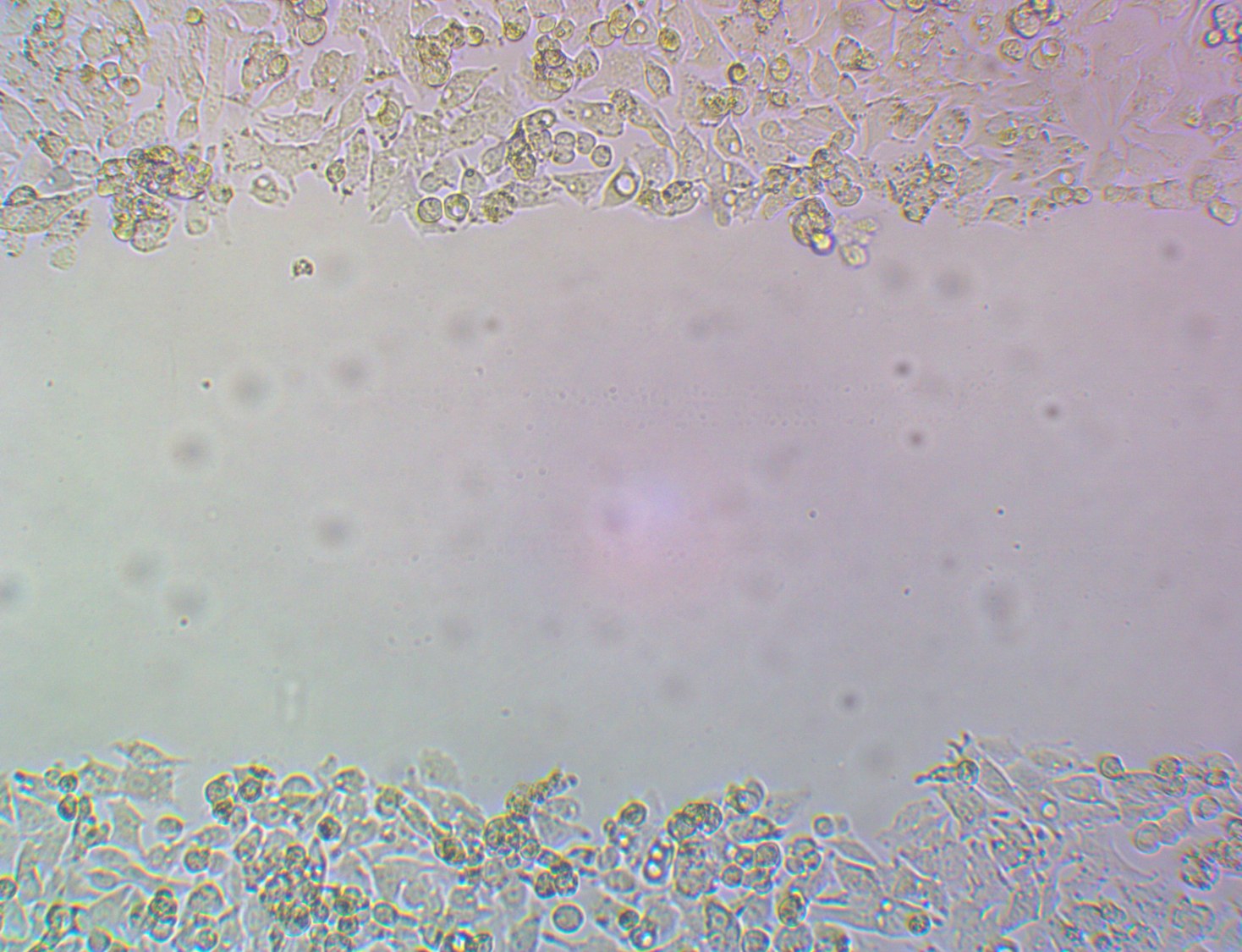

Supplement: Supplementary file 13 [file DataSheet5.ZIP › 0H/control-0h-10 raw.jpg]

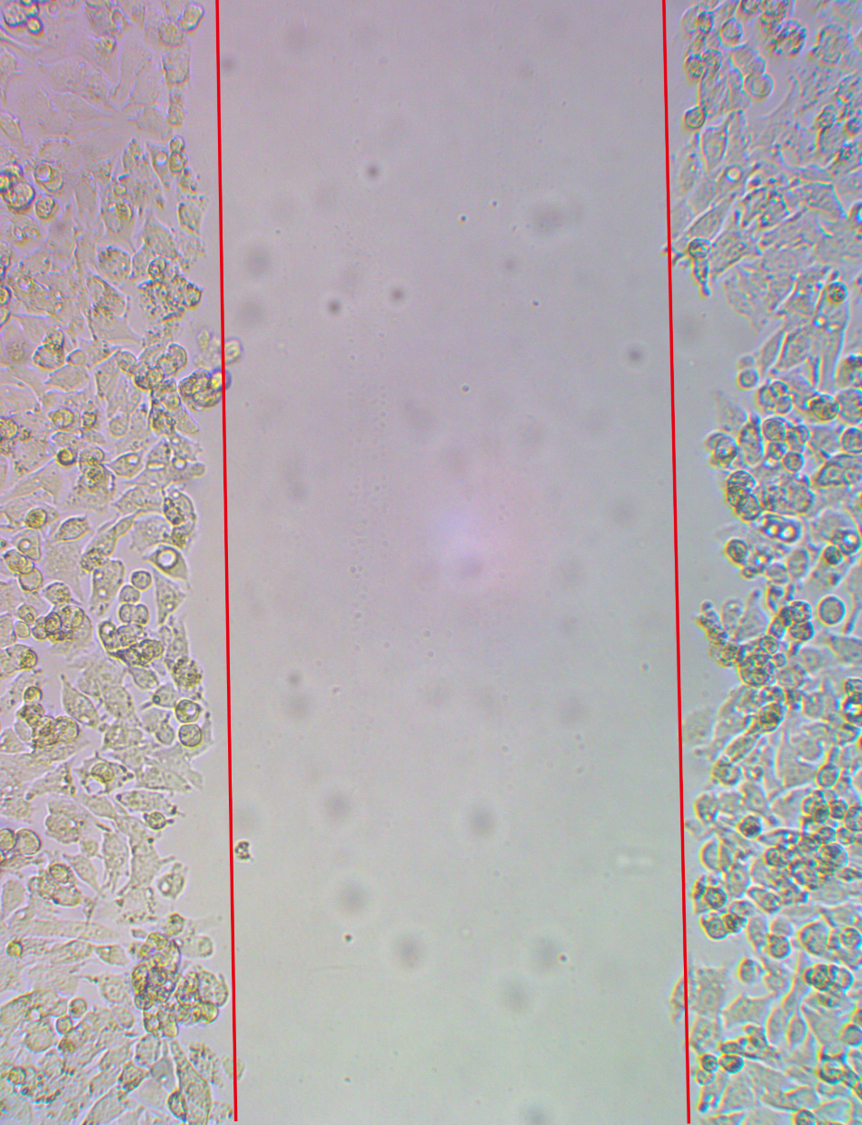

Supplement: Supplementary file 13 [file DataSheet5.ZIP › 0H/control-0h-10.jpg]

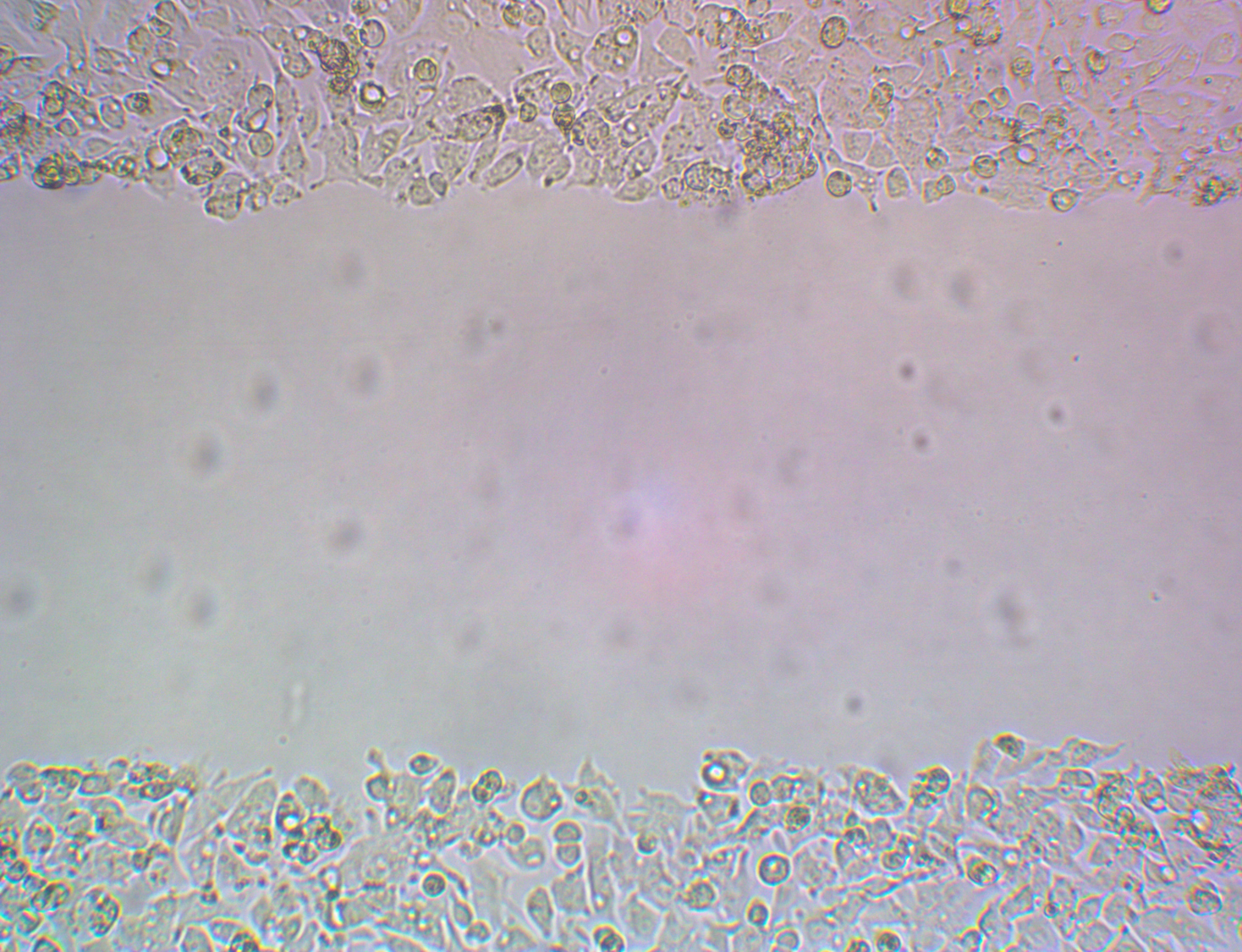

Supplement: Supplementary file 13 [file DataSheet5.ZIP › 0H/control-0h-1raw.jpg]

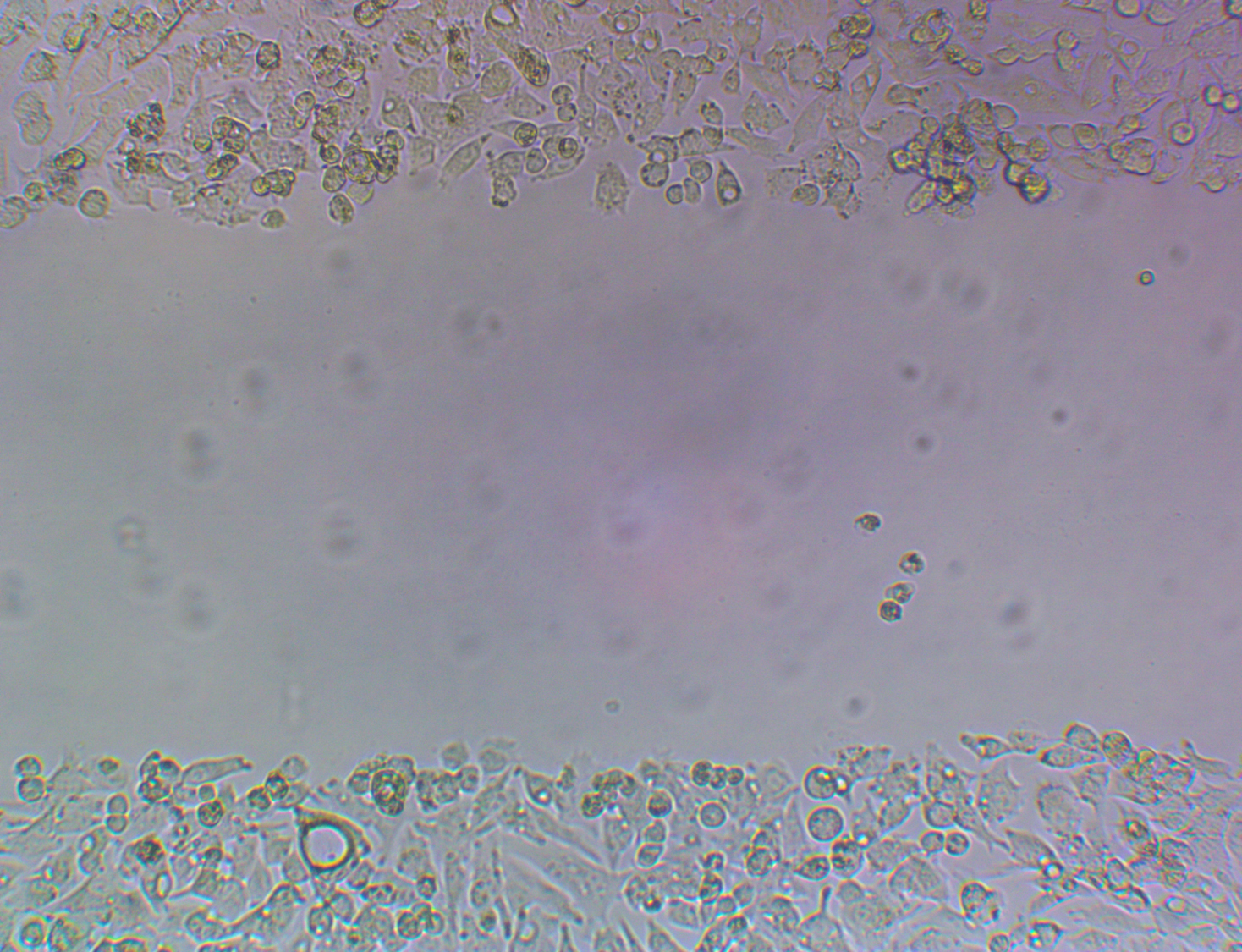

Supplement: Supplementary file 13 [file DataSheet5.ZIP › 0H/control-0h-4 raw.jpg]

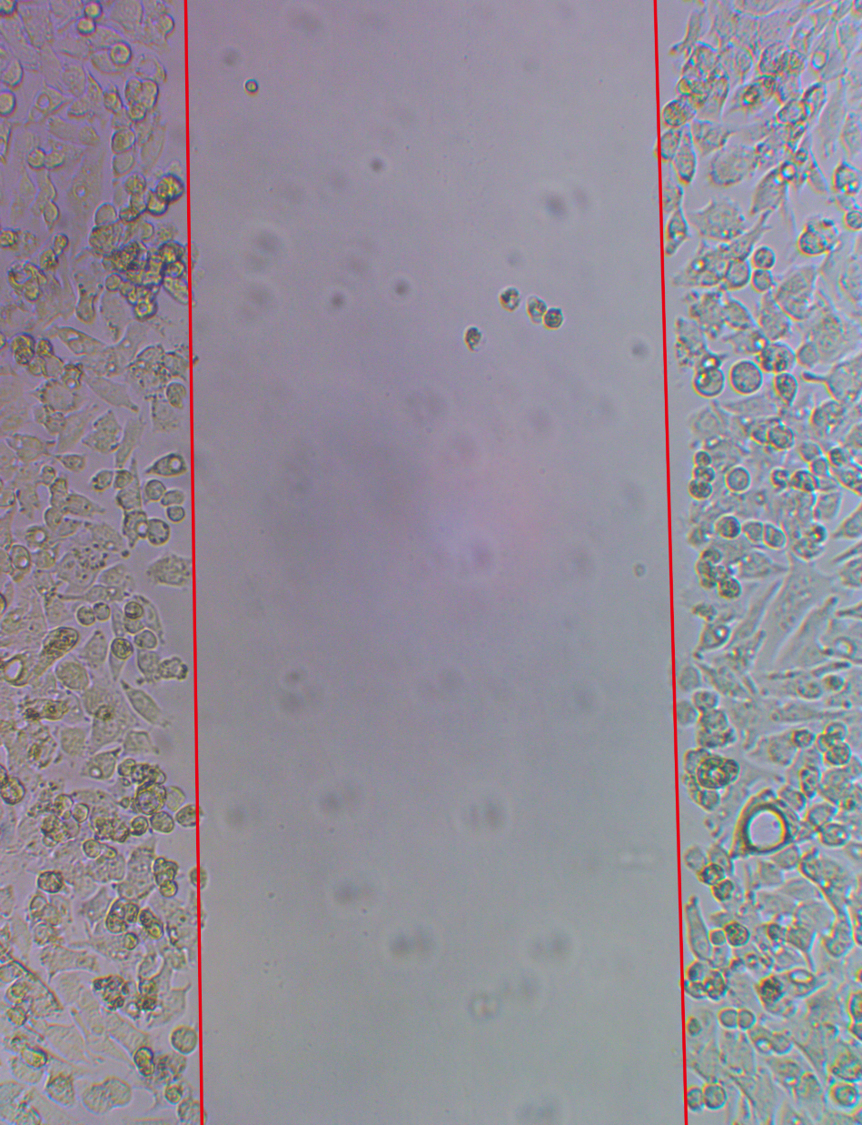

Supplement: Supplementary file 13 [file DataSheet5.ZIP › 0H/control-0h-4.jpg]

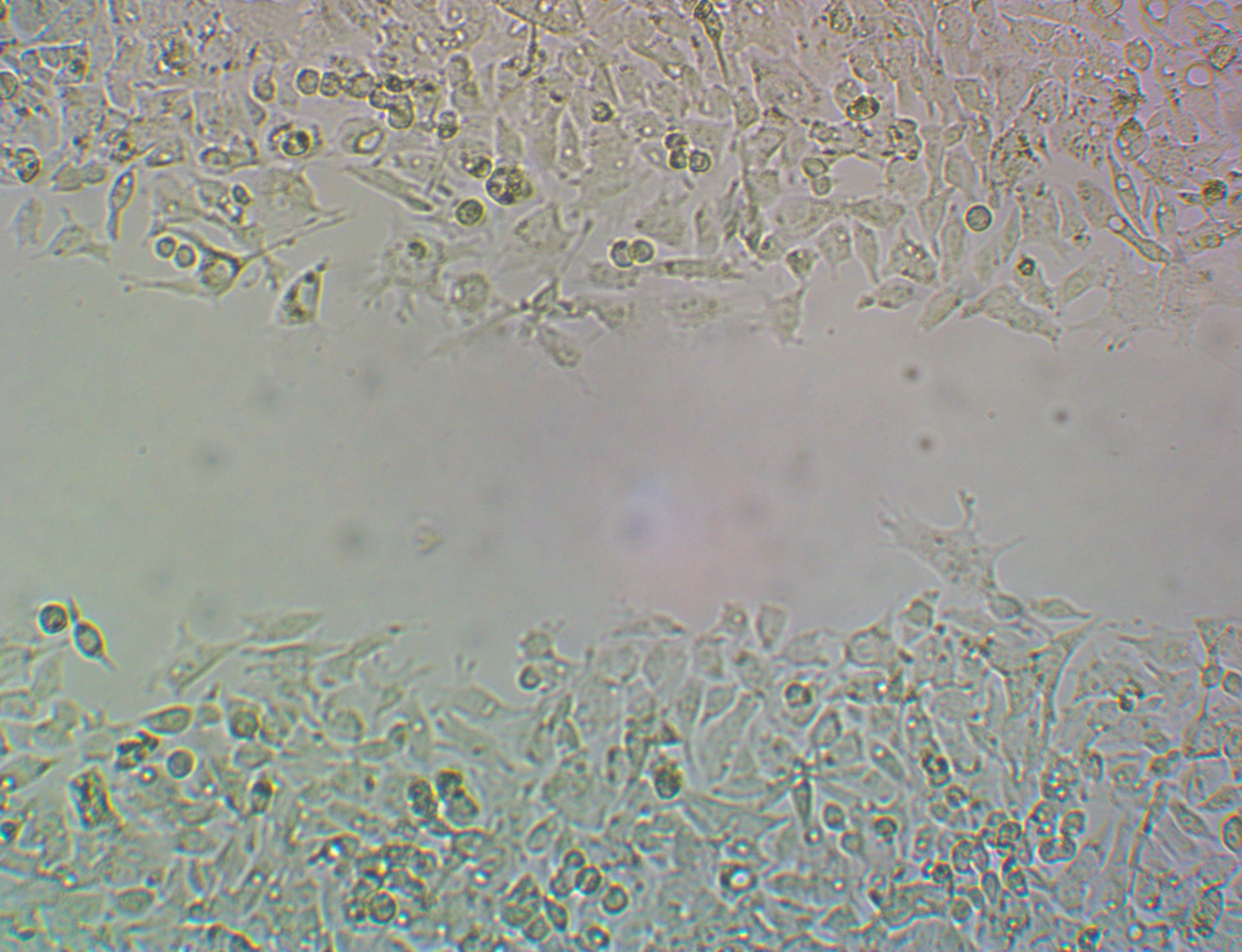

Supplement: Supplementary file 13 [file DataSheet5.ZIP › 24H/control-24h-5 raw.jpg]

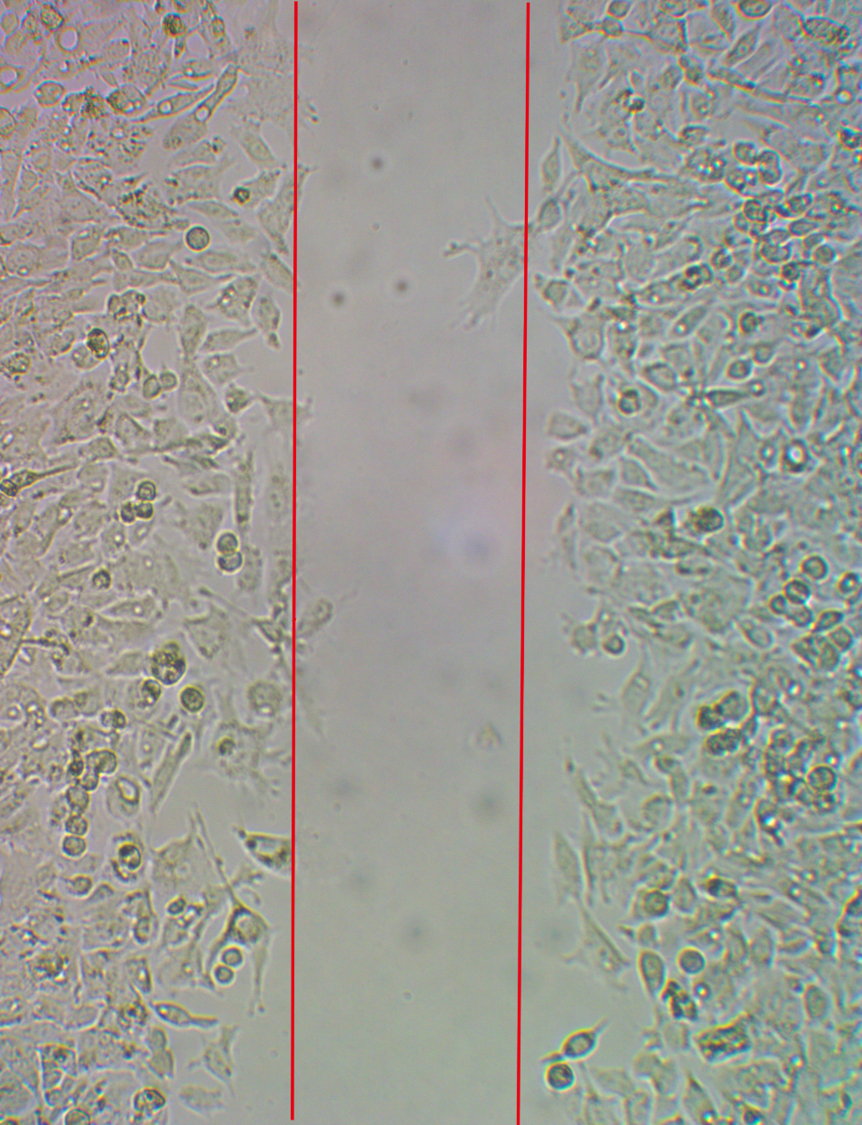

Supplement: Supplementary file 13 [file DataSheet5.ZIP › 24H/control-24h-5.jpg]

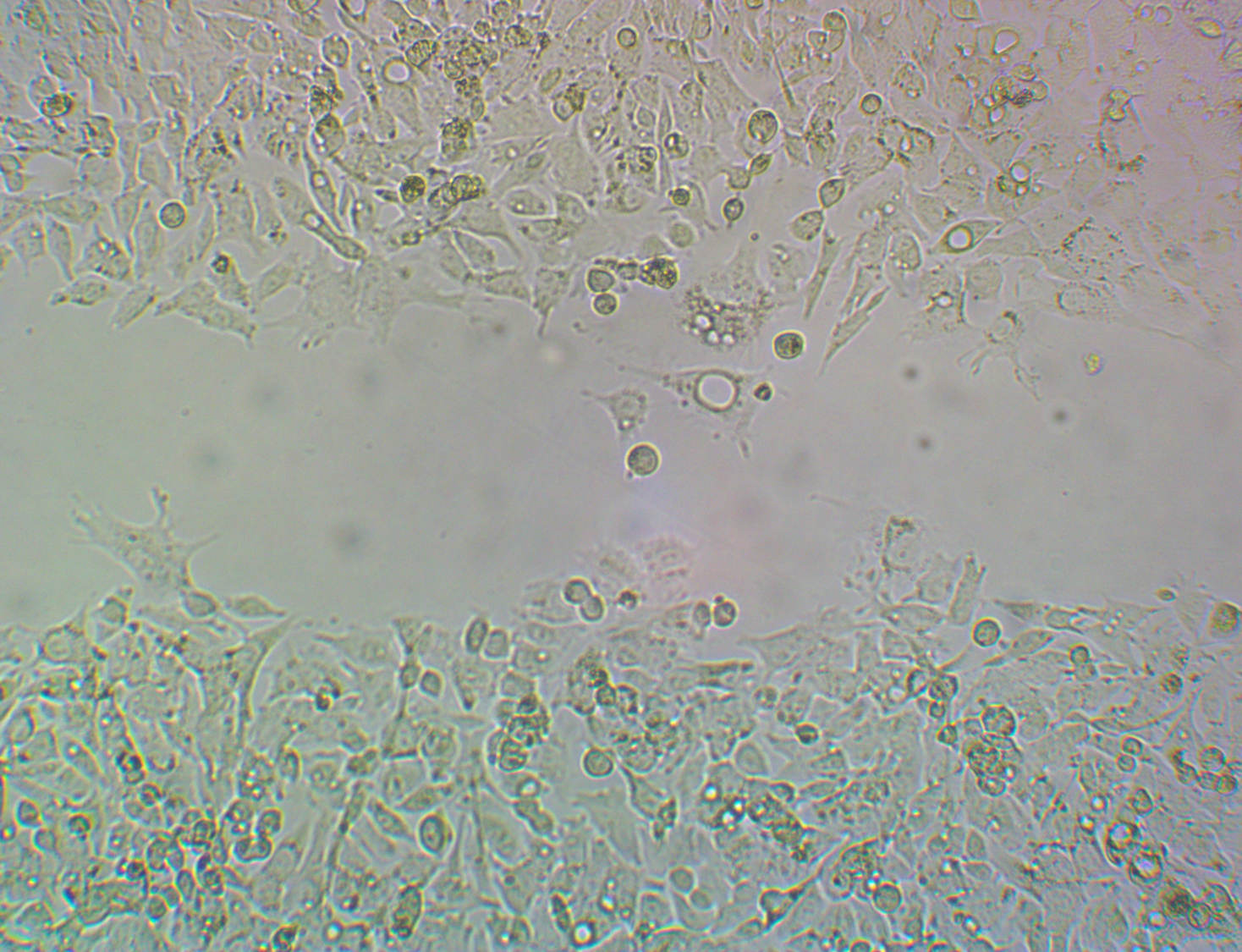

Supplement: Supplementary file 13 [file DataSheet5.ZIP › 24H/control-24h-6 raw.jpg]

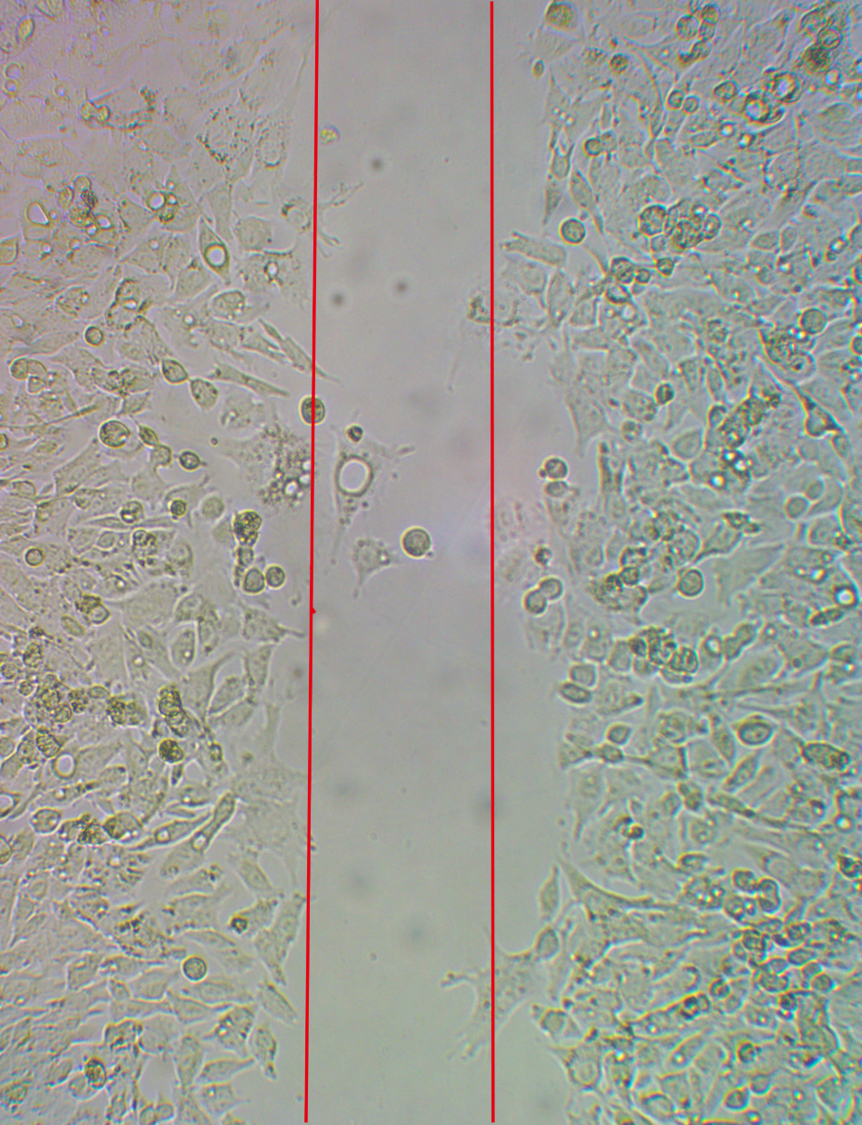

Supplement: Supplementary file 13 [file DataSheet5.ZIP › 24H/control-24h-6.jpg]

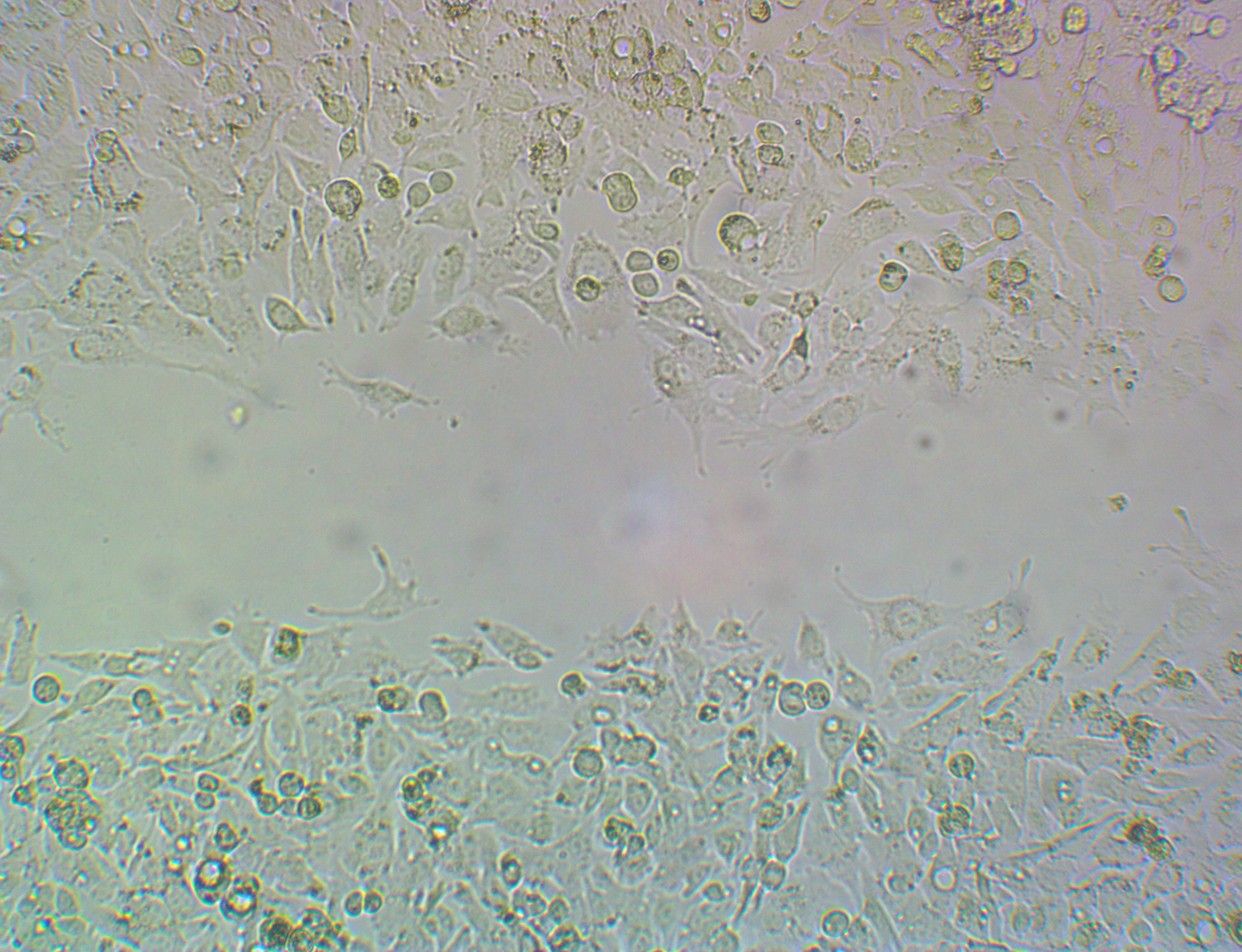

Supplement: Supplementary file 13 [file DataSheet5.ZIP › 24H/control-24h-7 raw.jpg]

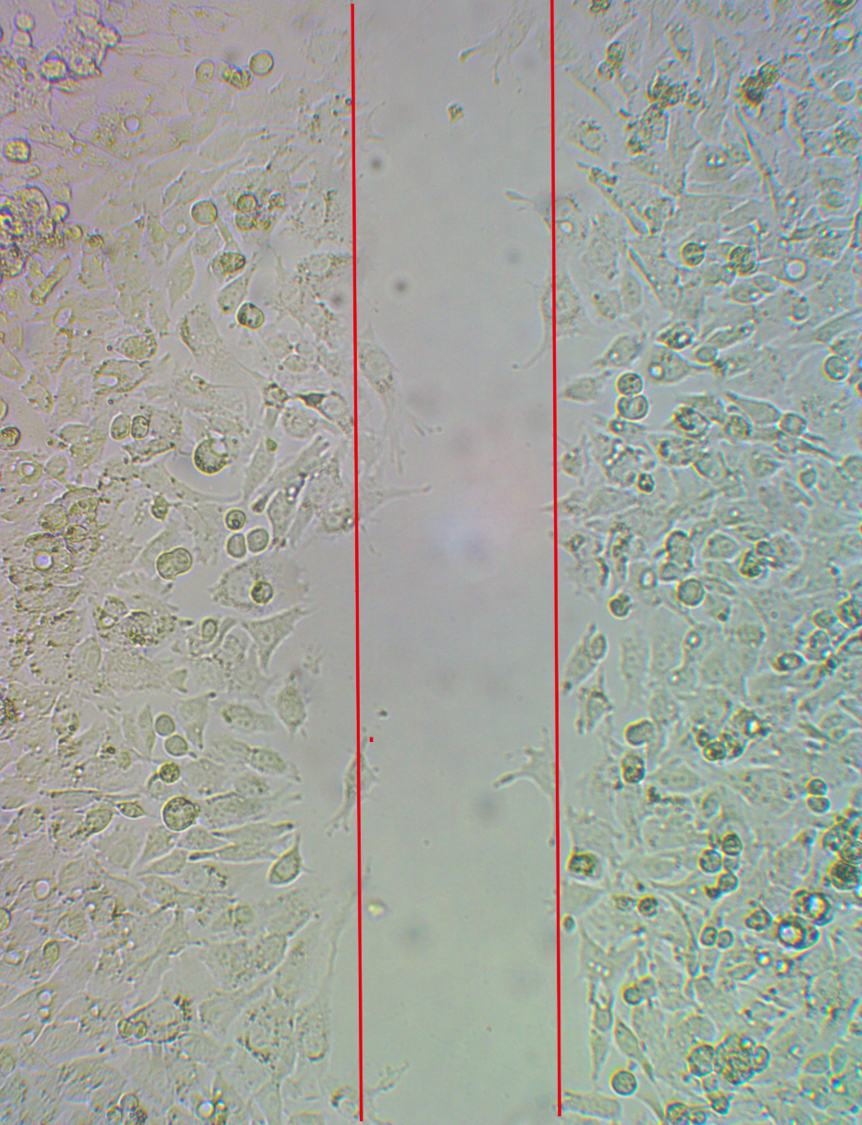

Supplement: Supplementary file 13 [file DataSheet5.ZIP › 24H/control-24h-7-22.jpg]

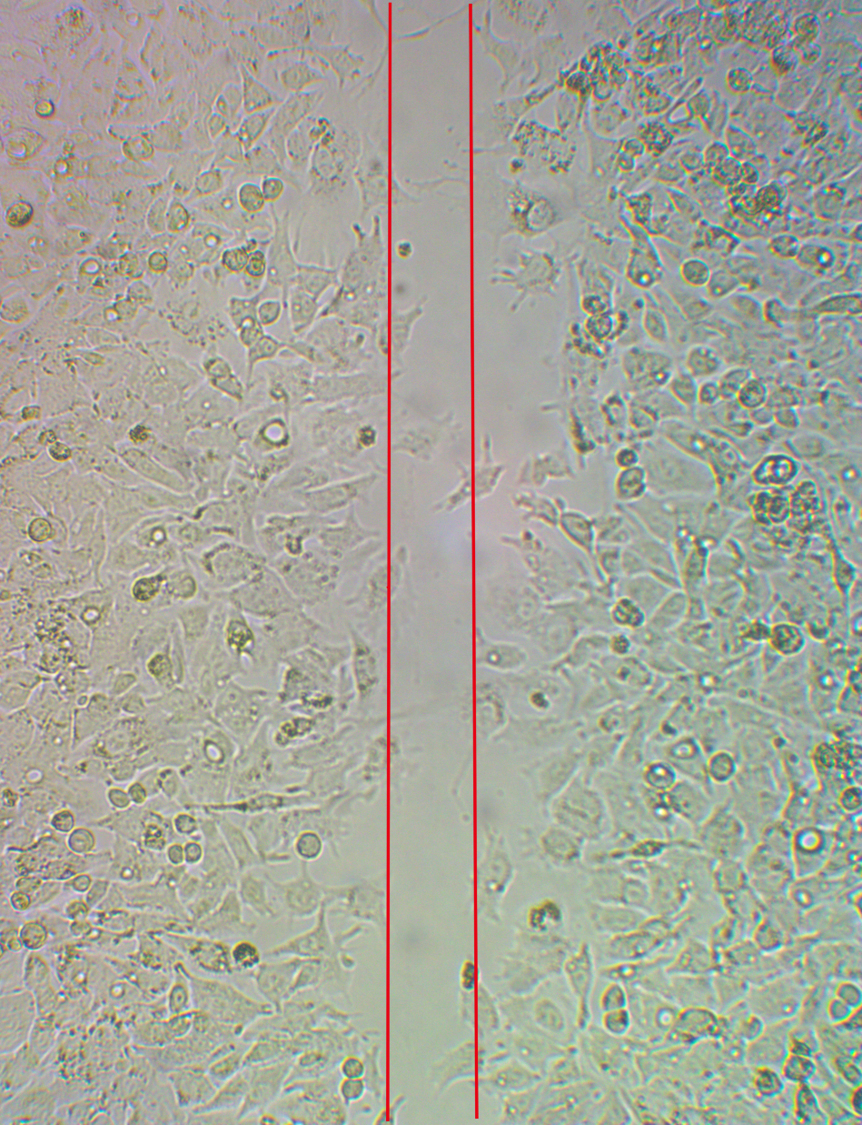

Supplement: Supplementary file 13 [file DataSheet5.ZIP › 48H/control-48h-10 .jpg]

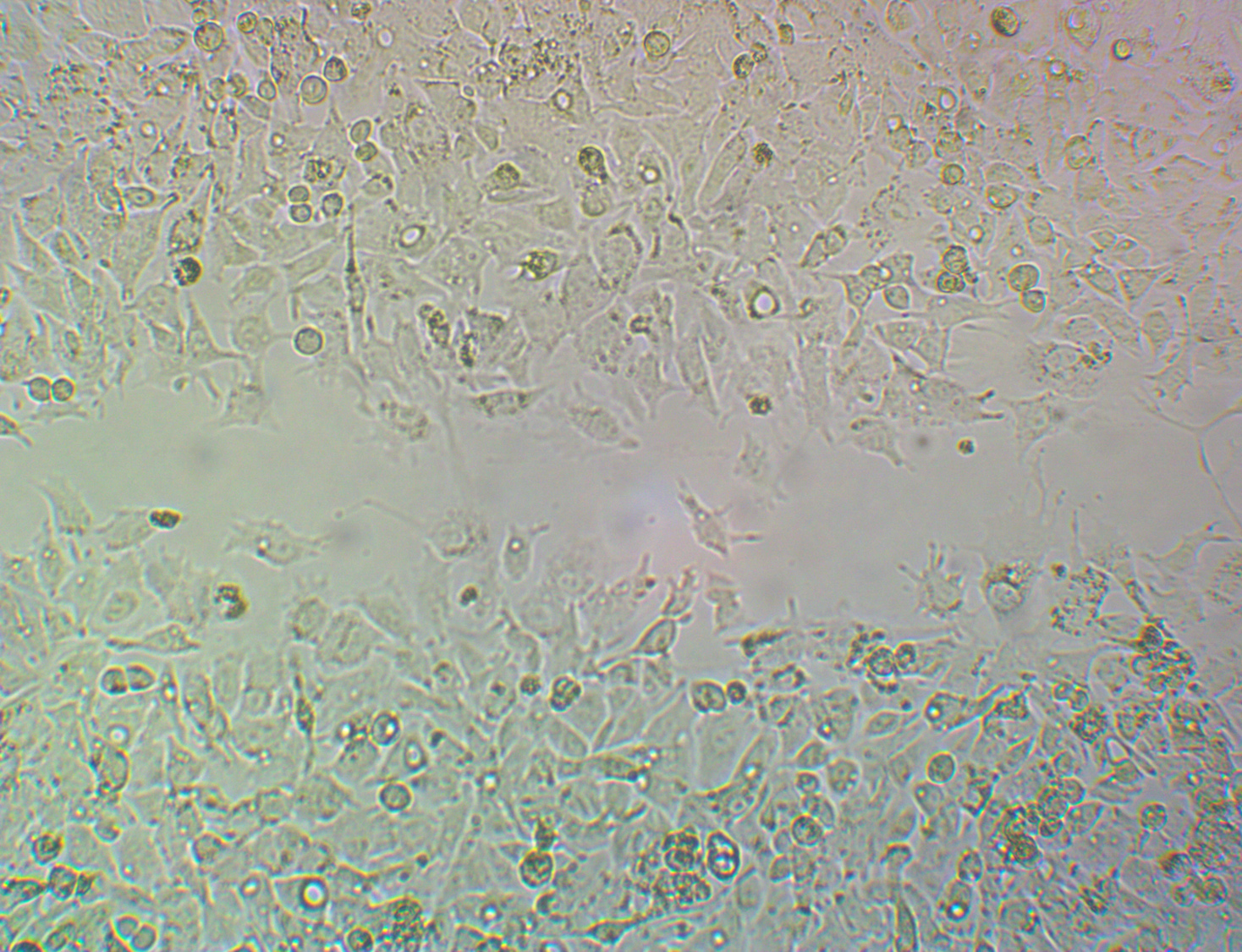

Supplement: Supplementary file 13 [file DataSheet5.ZIP › 48H/control-48h-10 raw.jpg]

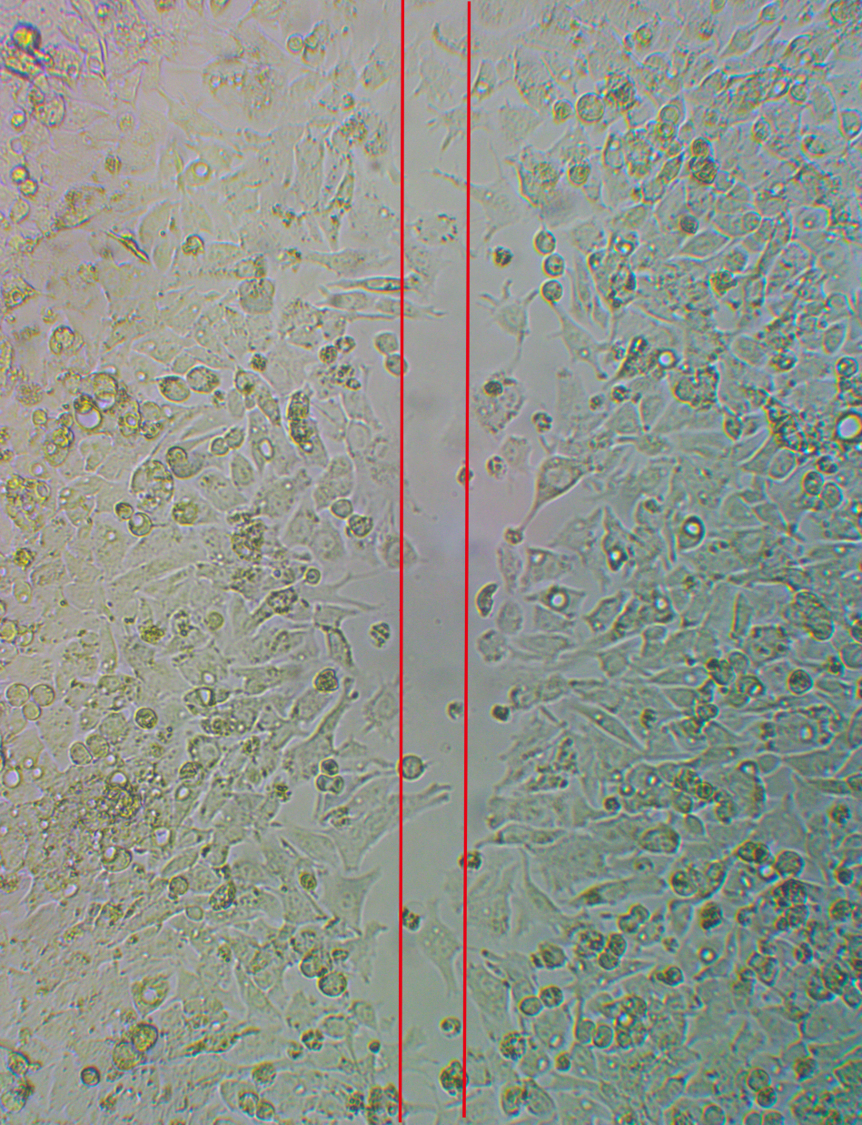

Supplement: Supplementary file 13 [file DataSheet5.ZIP › 48H/control-48h-12 .jpg]

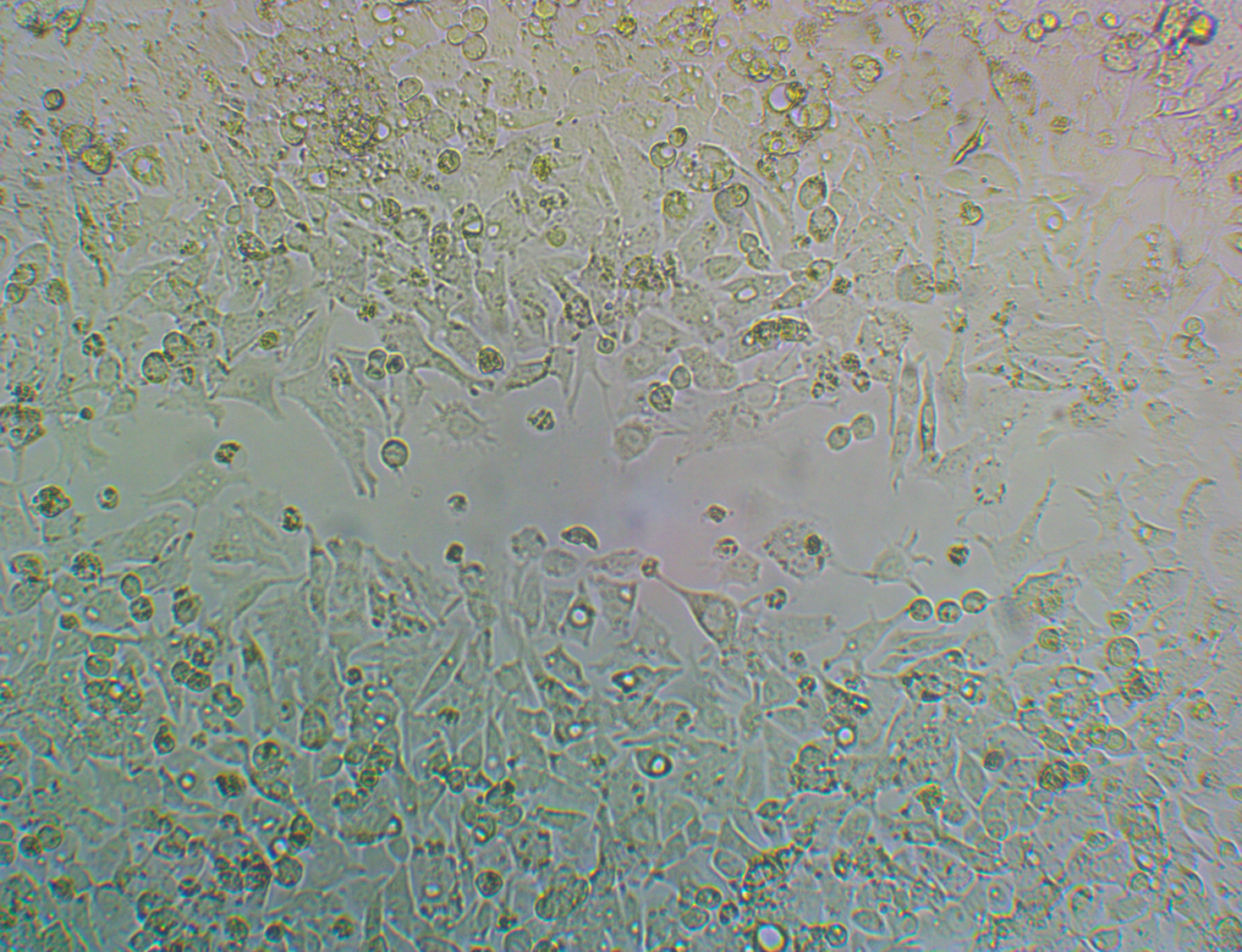

Supplement: Supplementary file 13 [file DataSheet5.ZIP › 48H/control-48h-12 raw.jpg]

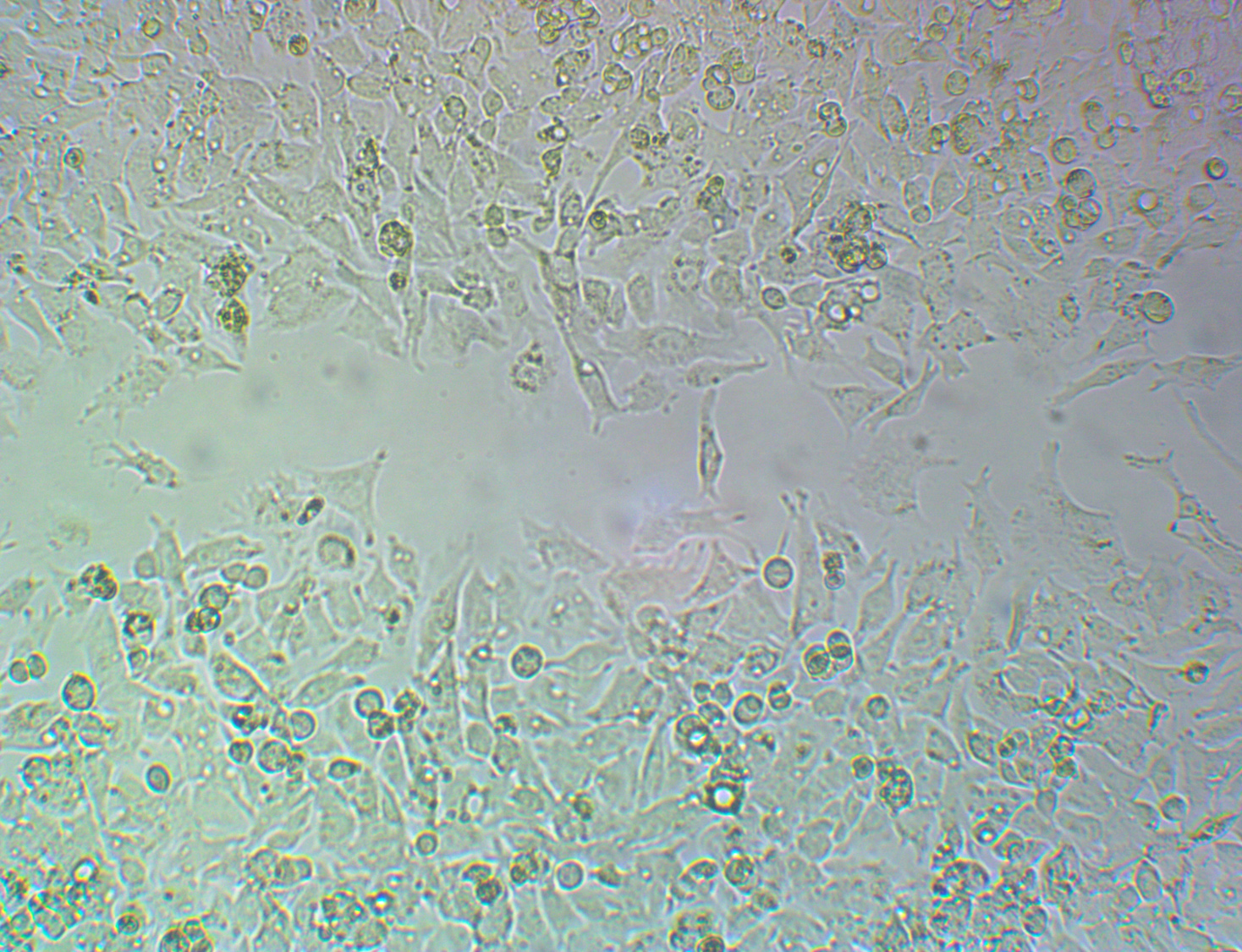

Supplement: Supplementary file 13 [file DataSheet5.ZIP › 48H/control-48h-14 raw.jpg]

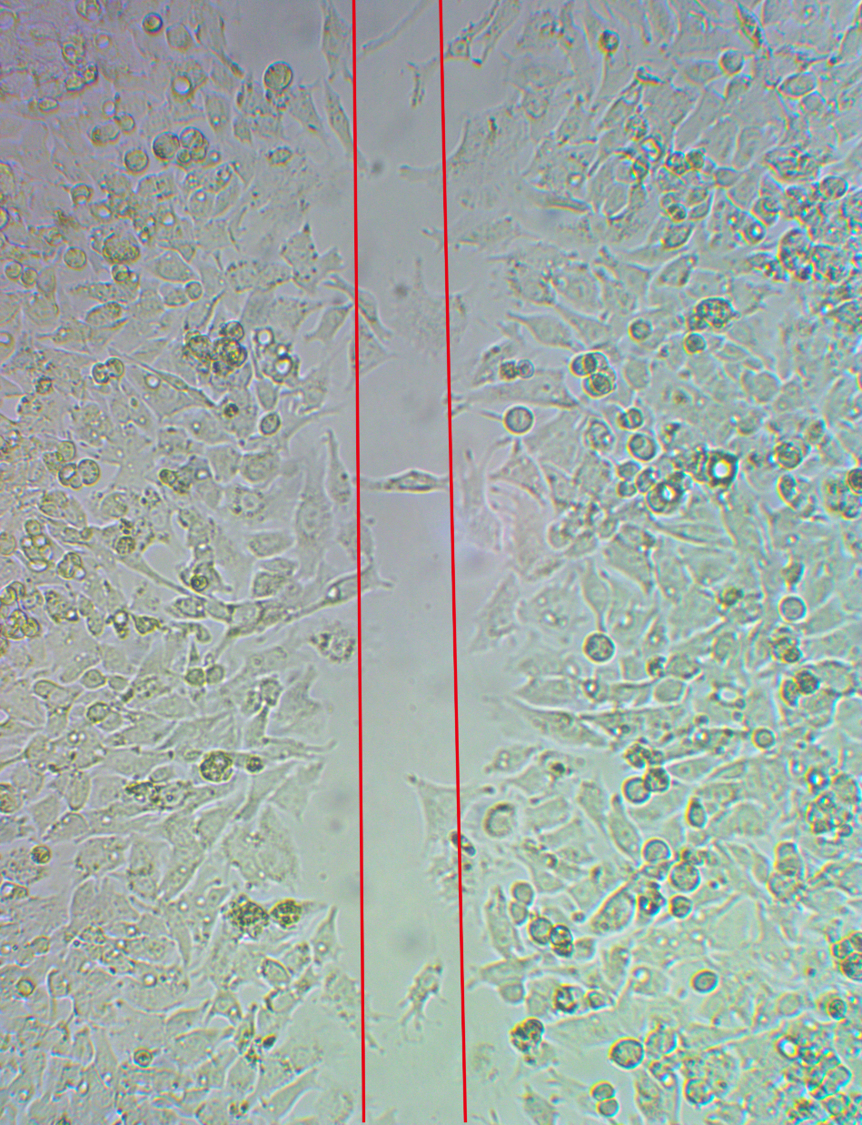

Supplement: Supplementary file 13 [file DataSheet5.ZIP › 48H/control-48h-14.jpg]

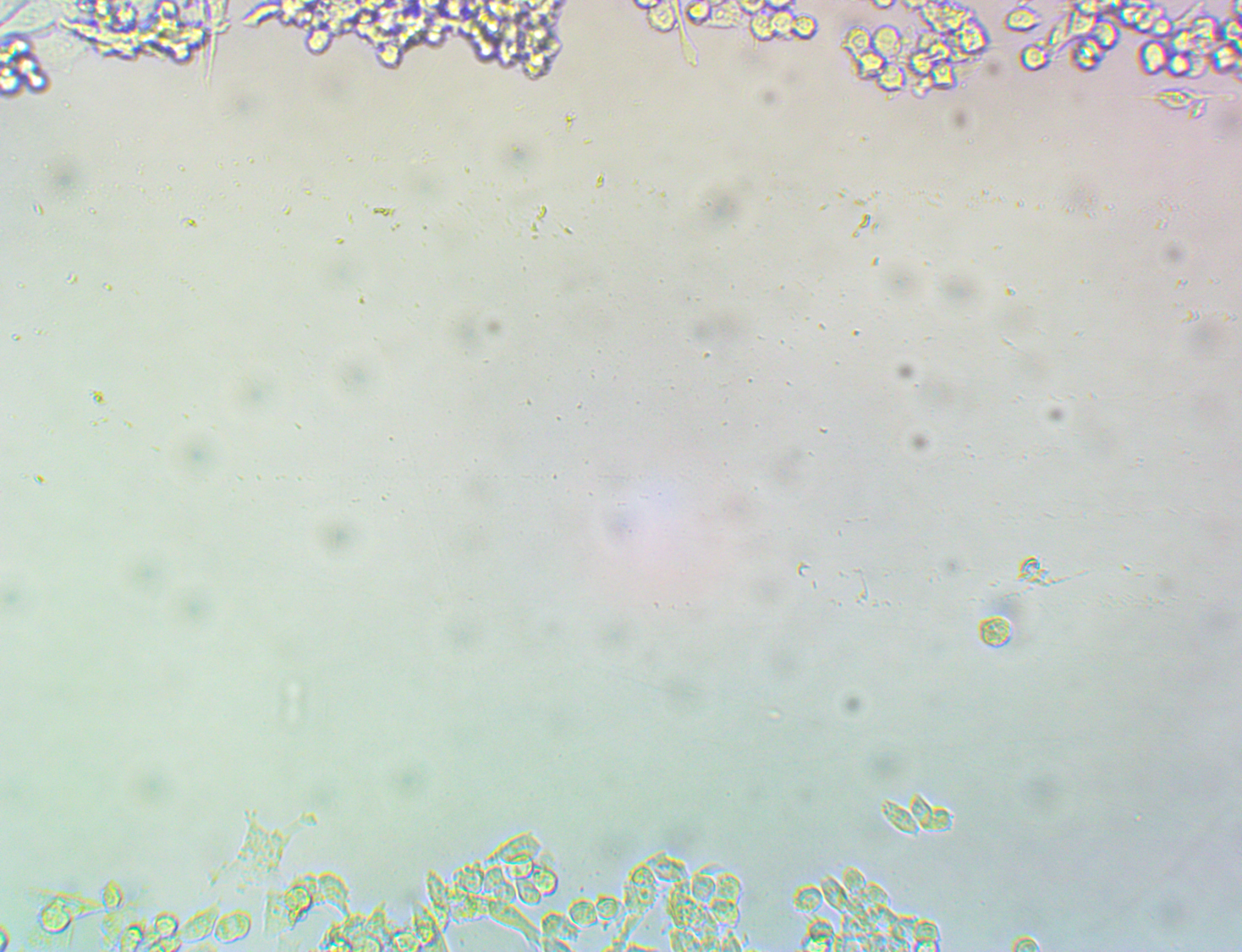

Supplement: Supplementary file 14 [file DataSheet7.ZIP › 0H/72309-0h-1 raw.jpg]

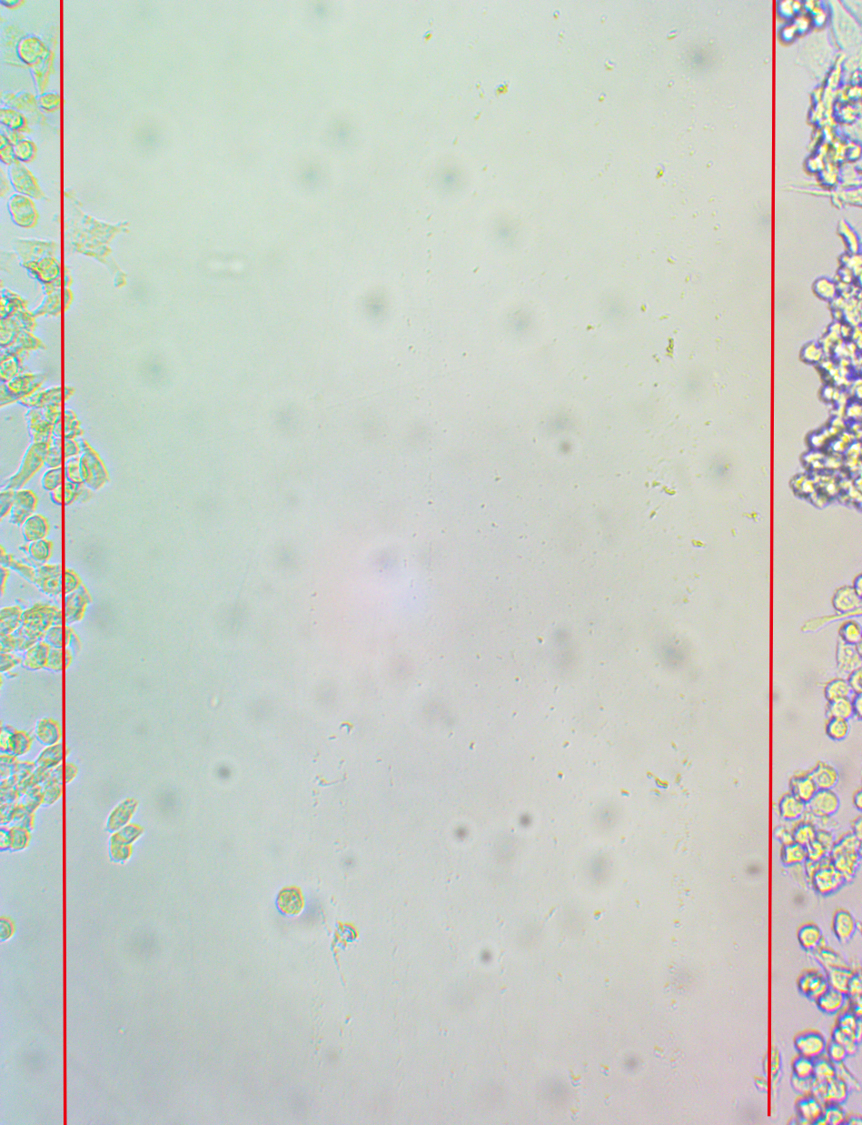

Supplement: Supplementary file 14 [file DataSheet7.ZIP › 0H/72309-0h-1.jpg]

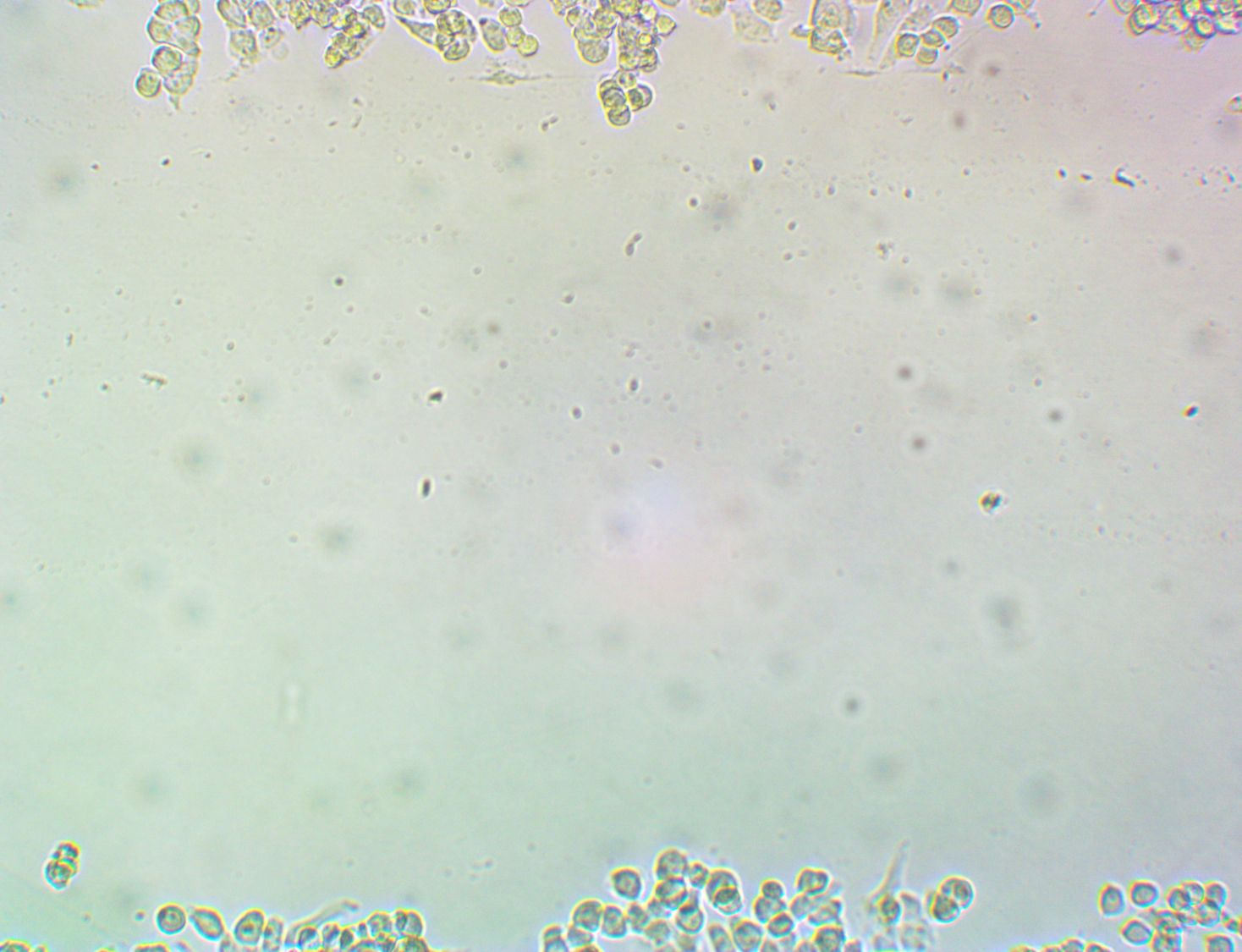

Supplement: Supplementary file 14 [file DataSheet7.ZIP › 0H/72309-0h-3 raw.jpg]

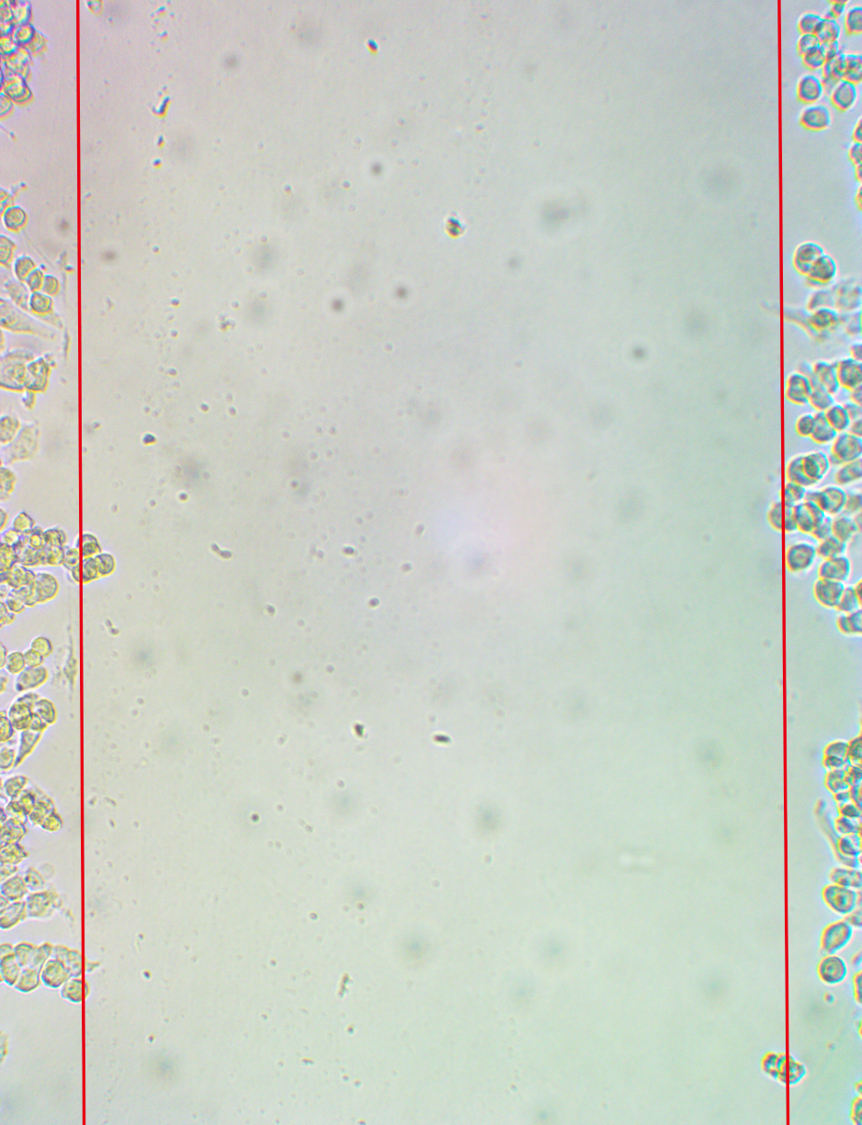

Supplement: Supplementary file 14 [file DataSheet7.ZIP › 0H/72309-0h-3.jpg]

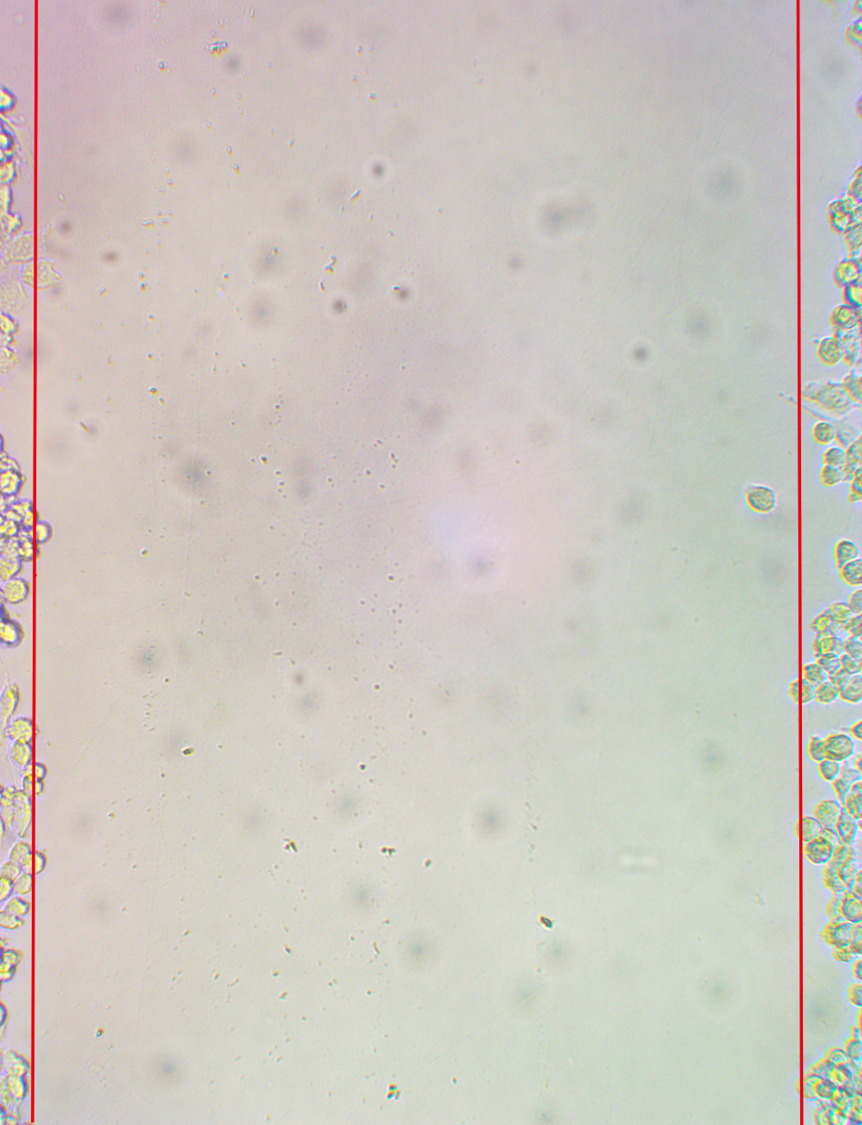

Supplement: Supplementary file 14 [file DataSheet7.ZIP › 0H/72309-0h-4 .jpg]

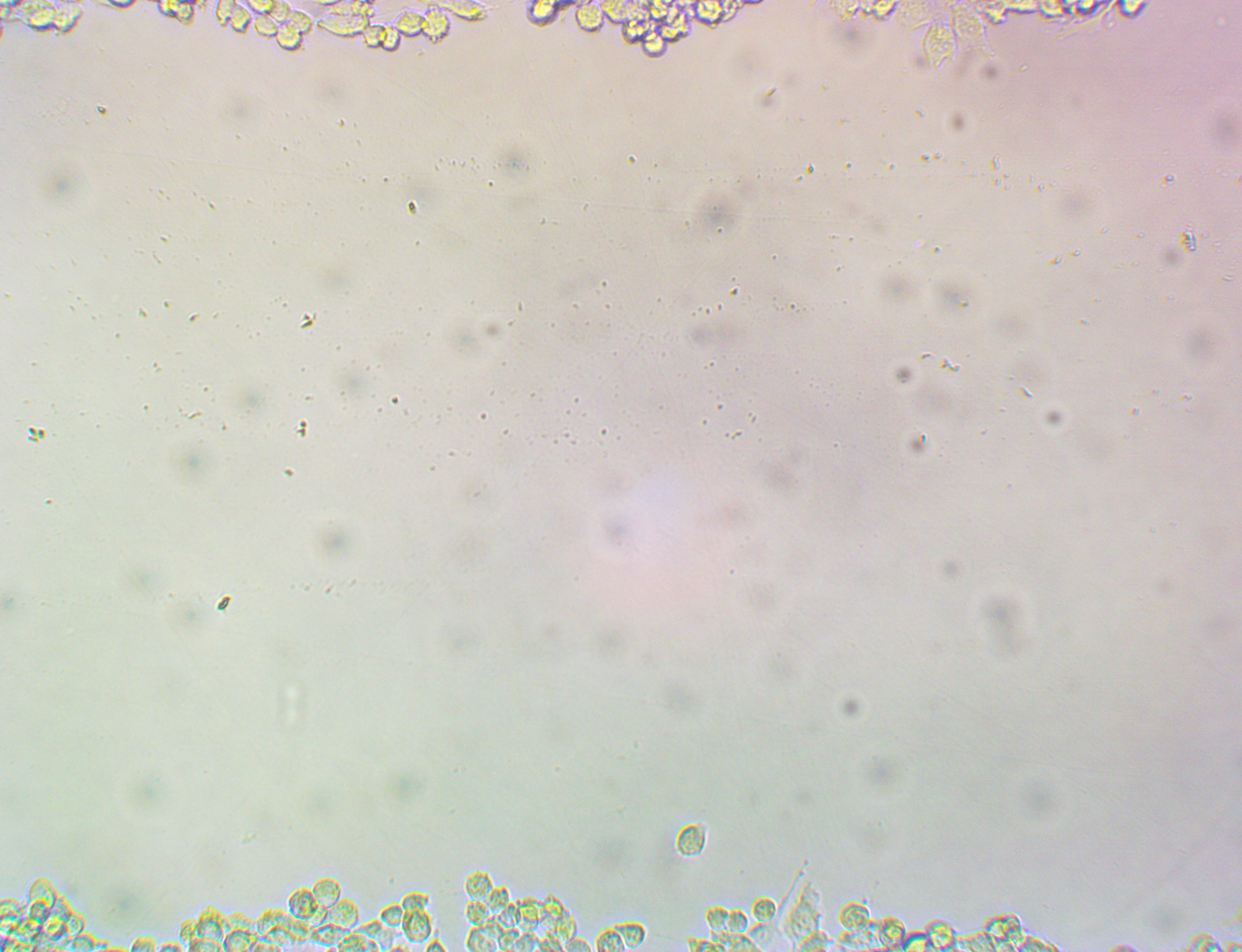

Supplement: Supplementary file 14 [file DataSheet7.ZIP › 0H/72309-0h-4 raw.jpg]

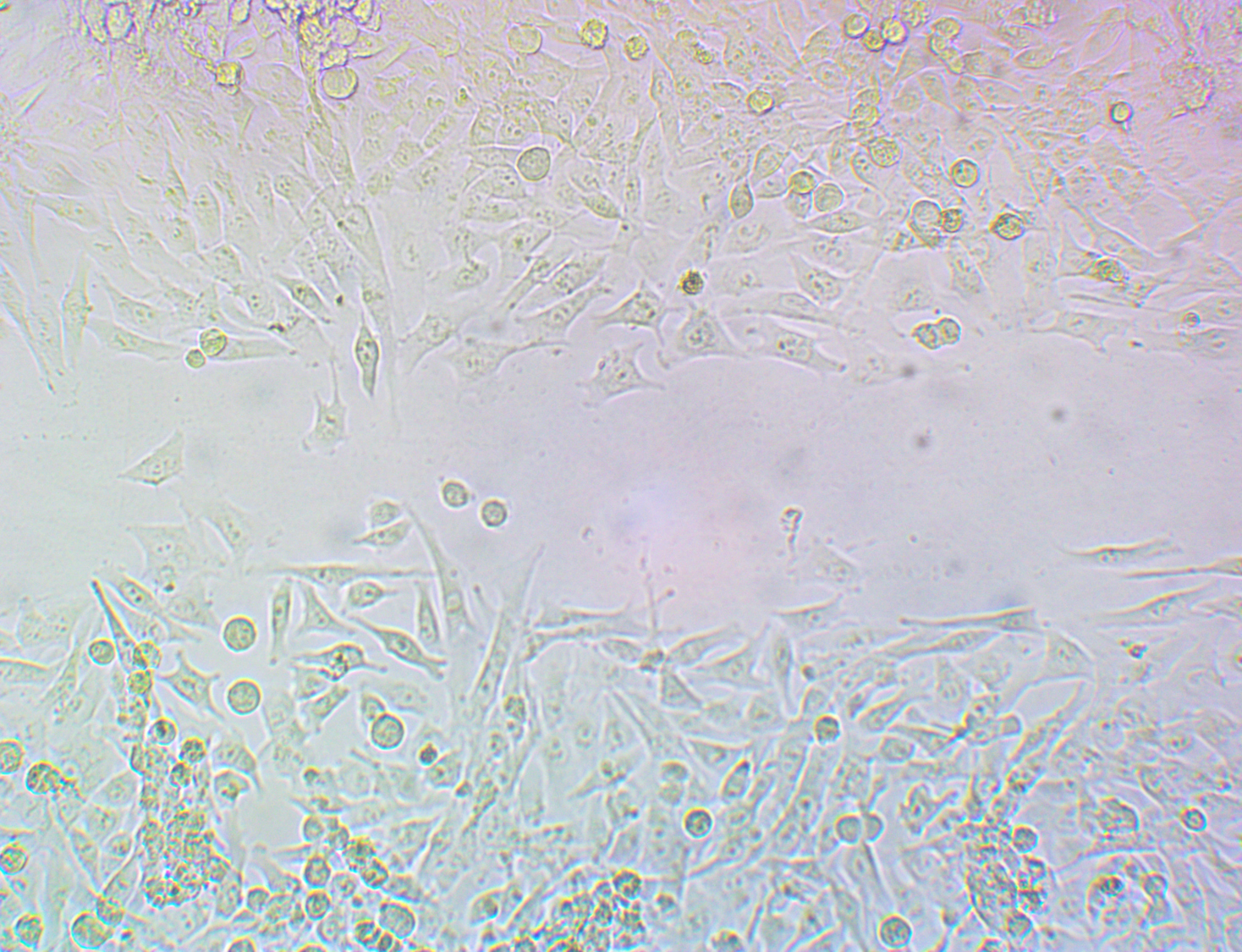

Supplement: Supplementary file 14 [file DataSheet7.ZIP › 24H/72309-24h-7 raw.jpg]

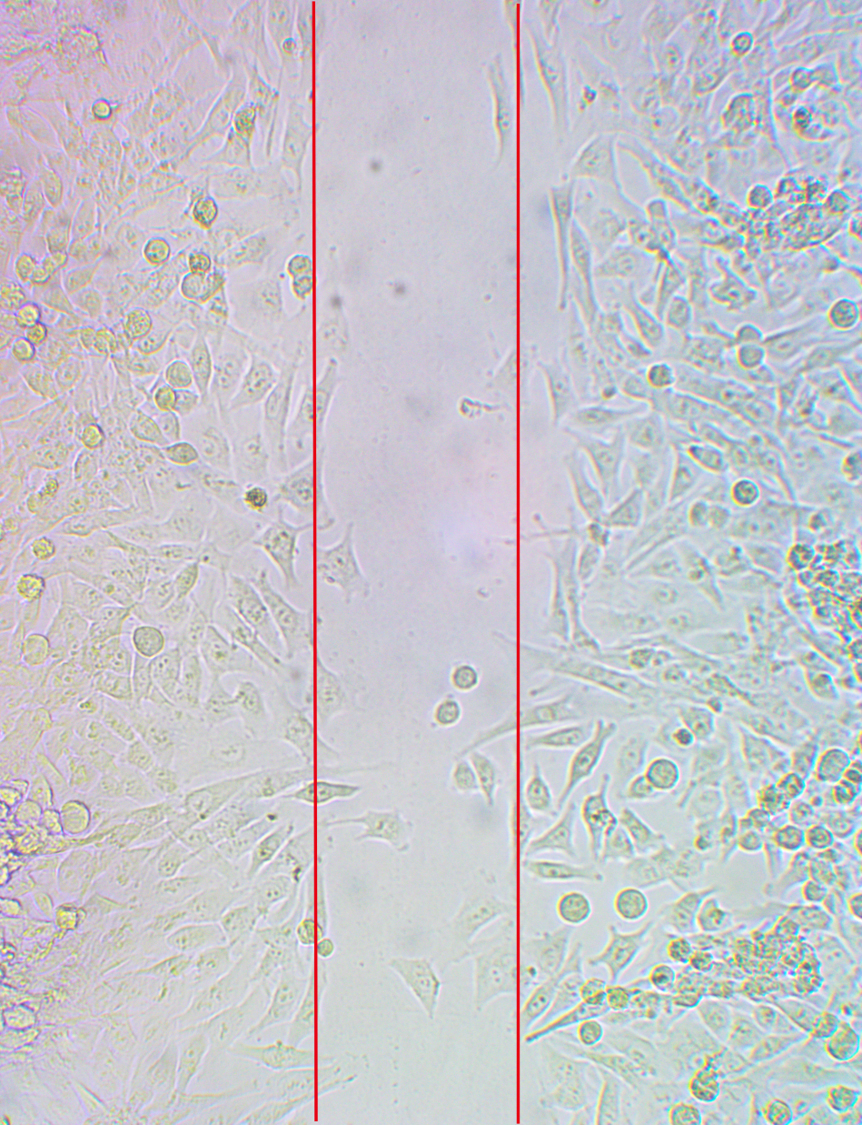

Supplement: Supplementary file 14 [file DataSheet7.ZIP › 24H/72309-24h-7.jpg]
