# Supplementary figures and images for: Downregulation of circLIFR exerts cancer-promoting effects on hepatocellular carcinoma in vitro (part 2 of 4)
Source: Front Genet. 2022 Sep 12;13:986322. doi: 10.3389/fgene.2022.986322 (PMC9513674; doi:10.3389/fgene.2022.986322)

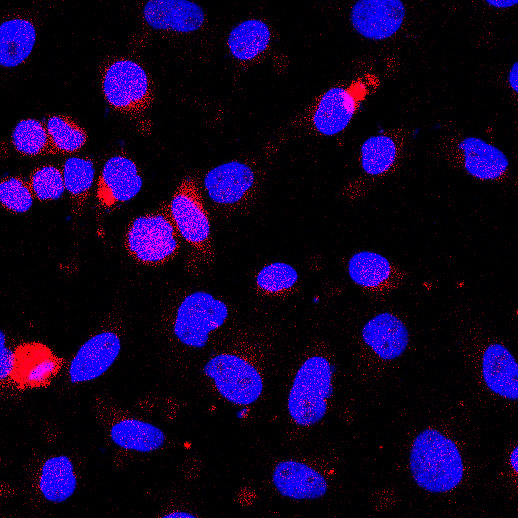

Supplement: Supplementary file 7 [file DataSheet13.ZIP › No Scale/LO2 (No Scale)/l11-40-单个文件导出-05_c1+3.jpg]

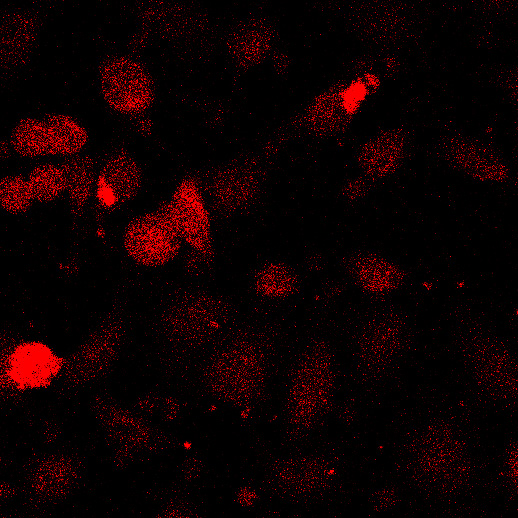

Supplement: Supplementary file 7 [file DataSheet13.ZIP › No Scale/LO2 (No Scale)/l11-40-单个文件导出-05_c1.jpg]

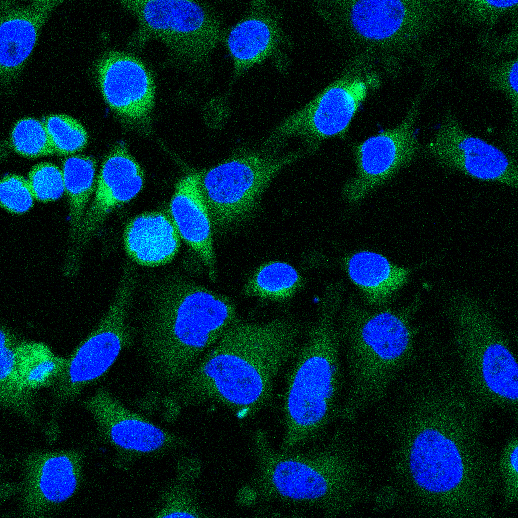

Supplement: Supplementary file 7 [file DataSheet13.ZIP › No Scale/LO2 (No Scale)/l11-40-单个文件导出-05_c2+3.tif]

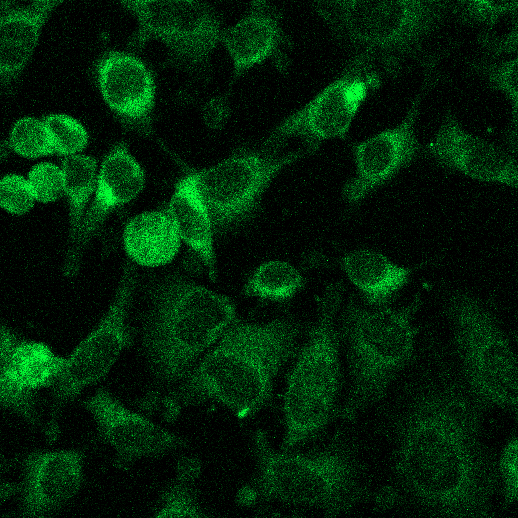

Supplement: Supplementary file 7 [file DataSheet13.ZIP › No Scale/LO2 (No Scale)/l11-40-单个文件导出-05_c2.tif]

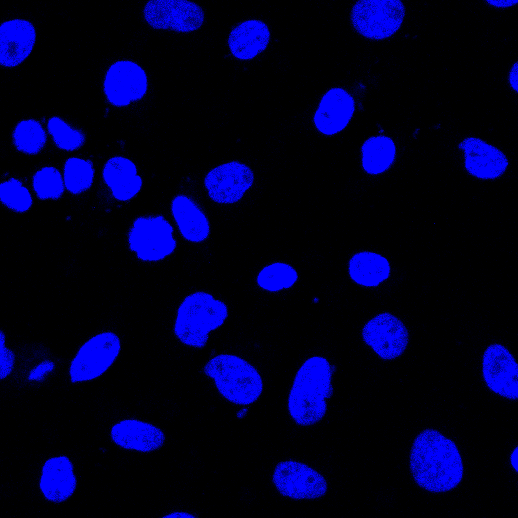

Supplement: Supplementary file 7 [file DataSheet13.ZIP › No Scale/LO2 (No Scale)/l11-40-单个文件导出-05_c3.tif]

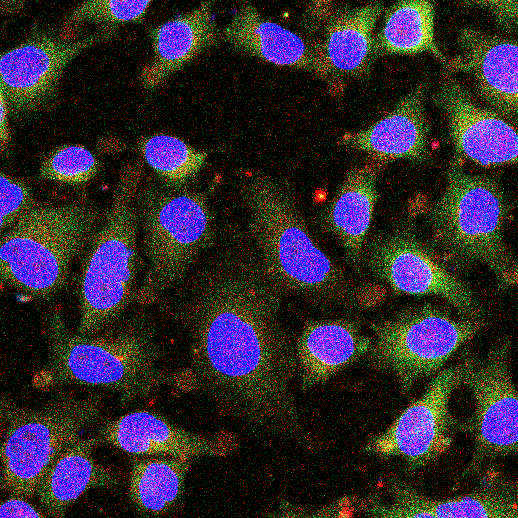

Supplement: Supplementary file 7 [file DataSheet13.ZIP › No Scale/LO2 (No Scale)/l12-10-单个文件导出-06_c1+2+3.tif]

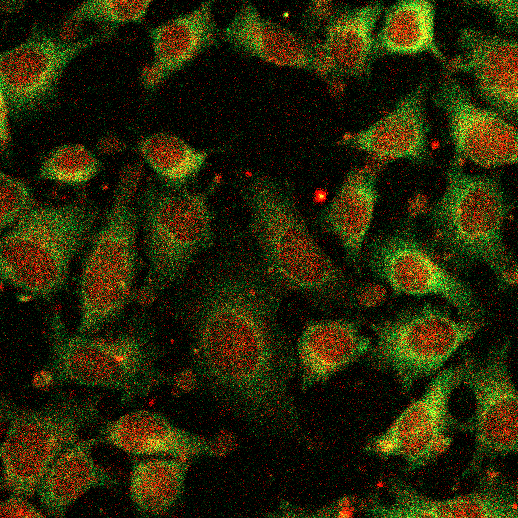

Supplement: Supplementary file 7 [file DataSheet13.ZIP › No Scale/LO2 (No Scale)/l12-10-单个文件导出-06_c1+2.tif]

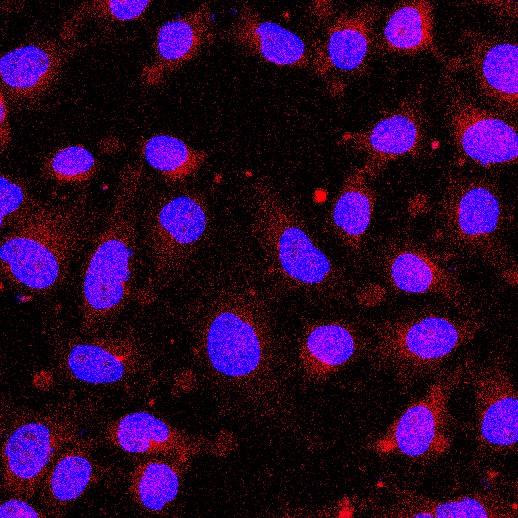

Supplement: Supplementary file 7 [file DataSheet13.ZIP › No Scale/LO2 (No Scale)/l12-10-单个文件导出-06_c1+3.tif]

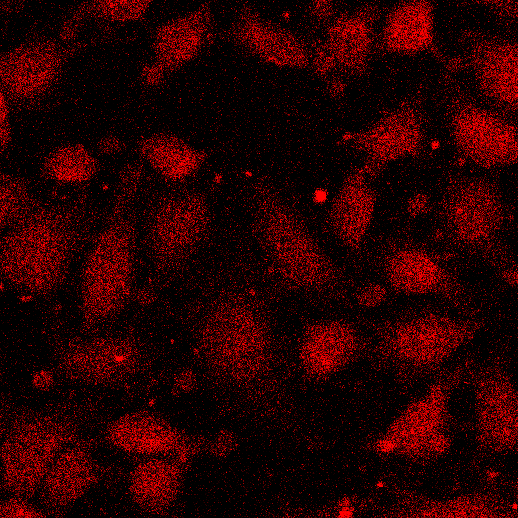

Supplement: Supplementary file 7 [file DataSheet13.ZIP › No Scale/LO2 (No Scale)/l12-10-单个文件导出-06_c1.tif]

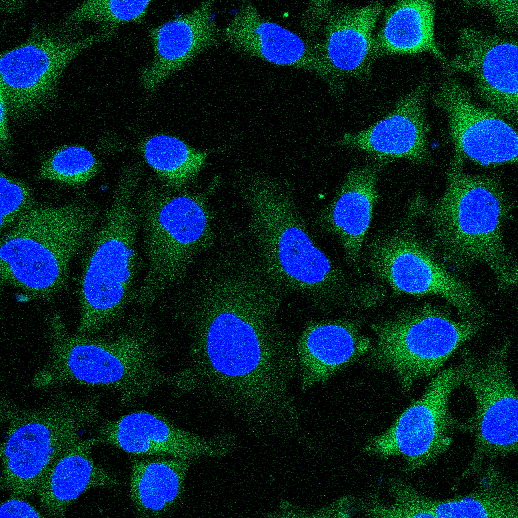

Supplement: Supplementary file 7 [file DataSheet13.ZIP › No Scale/LO2 (No Scale)/l12-10-单个文件导出-06_c2+3.tif]

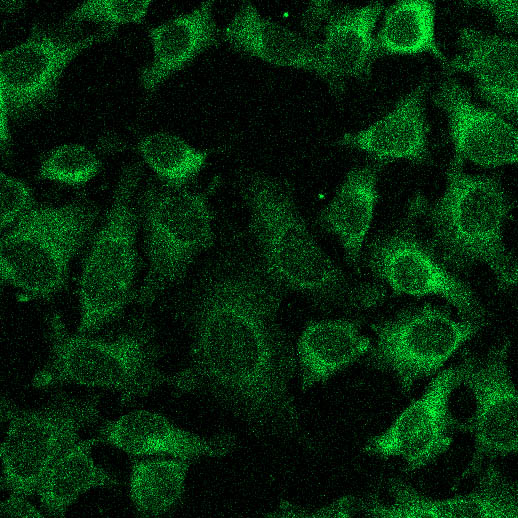

Supplement: Supplementary file 7 [file DataSheet13.ZIP › No Scale/LO2 (No Scale)/l12-10-单个文件导出-06_c2.jpg]

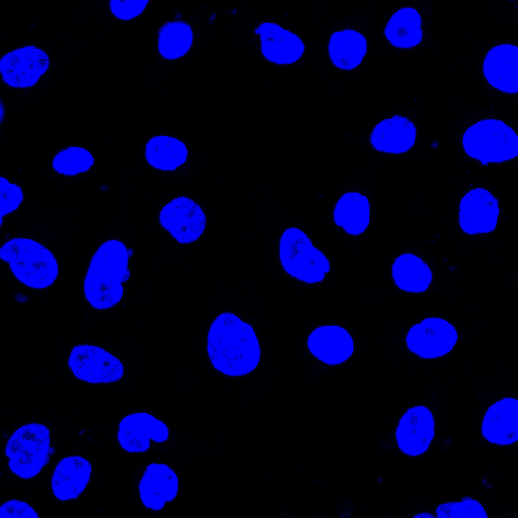

Supplement: Supplementary file 7 [file DataSheet13.ZIP › No Scale/LO2 (No Scale)/l12-10-单个文件导出-06_c3.tif]

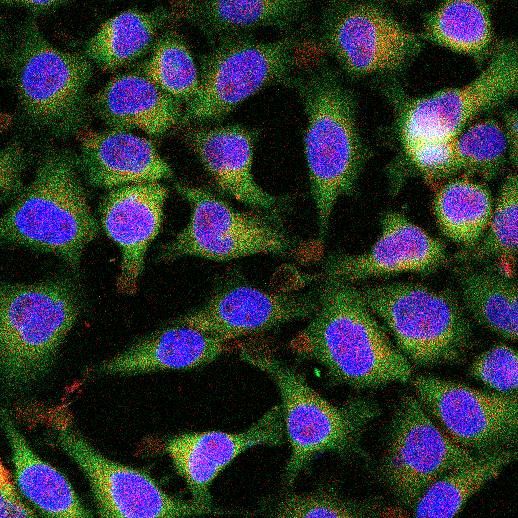

Supplement: Supplementary file 7 [file DataSheet13.ZIP › No Scale/LO2 (No Scale)/l7-40-单个文件导出-01_c1+2+3.tif]

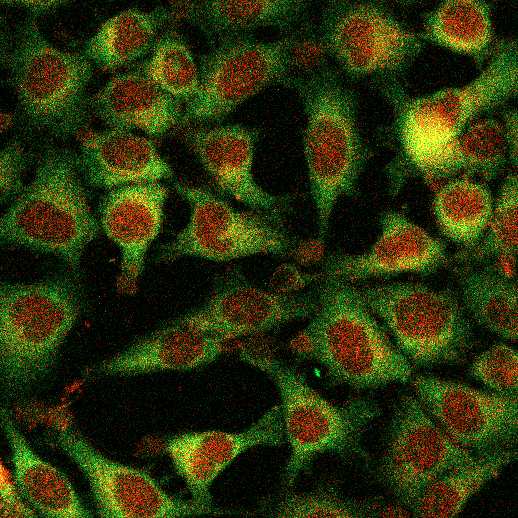

Supplement: Supplementary file 7 [file DataSheet13.ZIP › No Scale/LO2 (No Scale)/l7-40-单个文件导出-01_c1+2.tif]

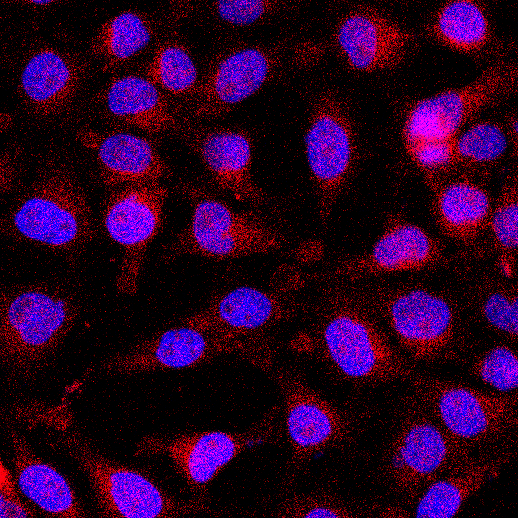

Supplement: Supplementary file 7 [file DataSheet13.ZIP › No Scale/LO2 (No Scale)/l7-40-单个文件导出-01_c1+3.tif]

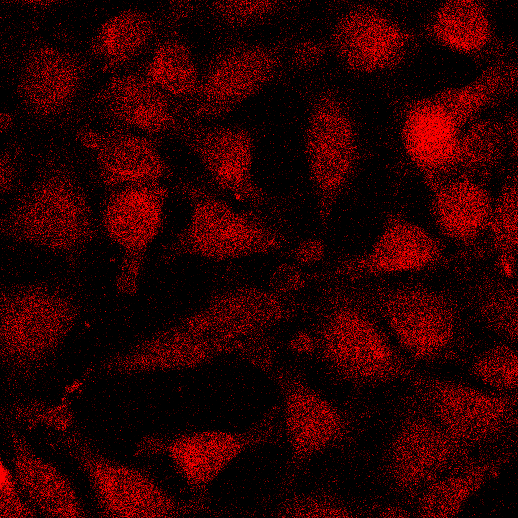

Supplement: Supplementary file 7 [file DataSheet13.ZIP › No Scale/LO2 (No Scale)/l7-40-单个文件导出-01_c1.tif]

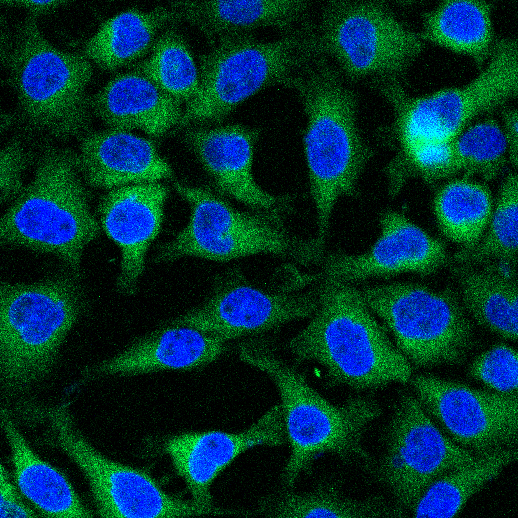

Supplement: Supplementary file 7 [file DataSheet13.ZIP › No Scale/LO2 (No Scale)/l7-40-单个文件导出-01_c2+3.tif]

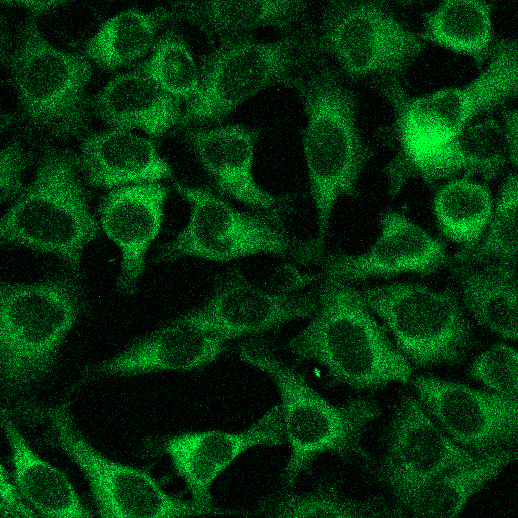

Supplement: Supplementary file 7 [file DataSheet13.ZIP › No Scale/LO2 (No Scale)/l7-40-单个文件导出-01_c2.jpg]

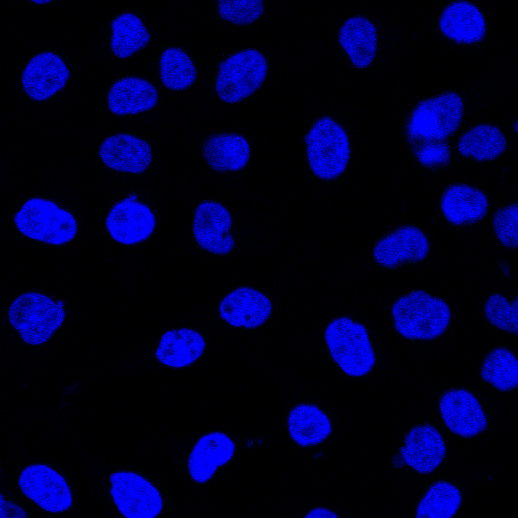

Supplement: Supplementary file 7 [file DataSheet13.ZIP › No Scale/LO2 (No Scale)/l7-40-单个文件导出-01_c3.jpg]

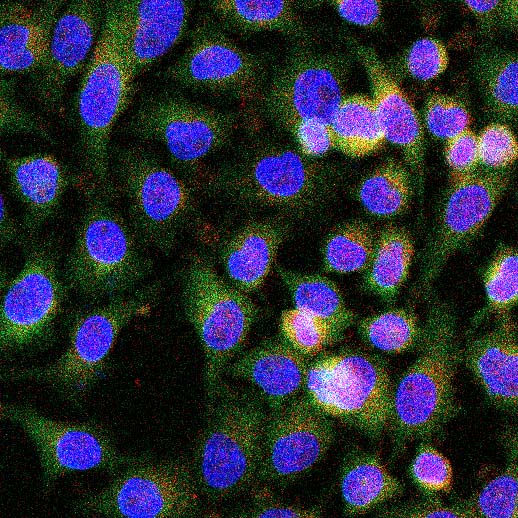

Supplement: Supplementary file 7 [file DataSheet13.ZIP › No Scale/LO2 (No Scale)/l8-40-单个文件导出-02_c1+2+3.jpg]

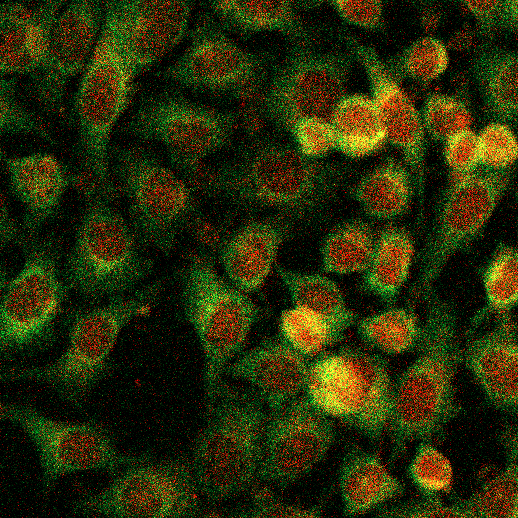

Supplement: Supplementary file 7 [file DataSheet13.ZIP › No Scale/LO2 (No Scale)/l8-40-单个文件导出-02_c1+2.tif]

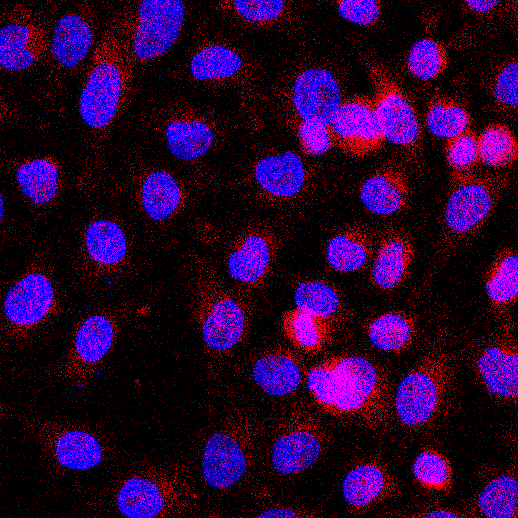

Supplement: Supplementary file 7 [file DataSheet13.ZIP › No Scale/LO2 (No Scale)/l8-40-单个文件导出-02_c1+3.tif]

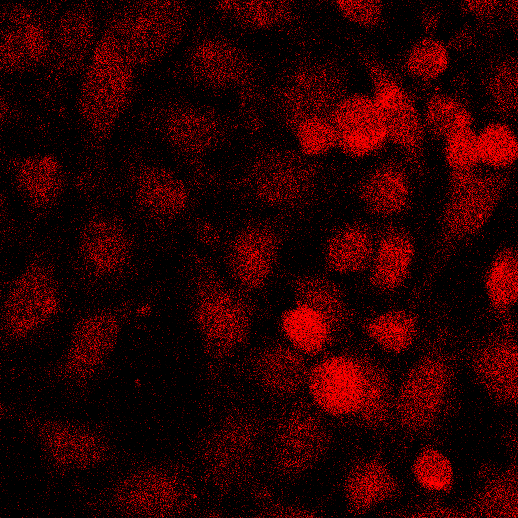

Supplement: Supplementary file 7 [file DataSheet13.ZIP › No Scale/LO2 (No Scale)/l8-40-单个文件导出-02_c1.tif]

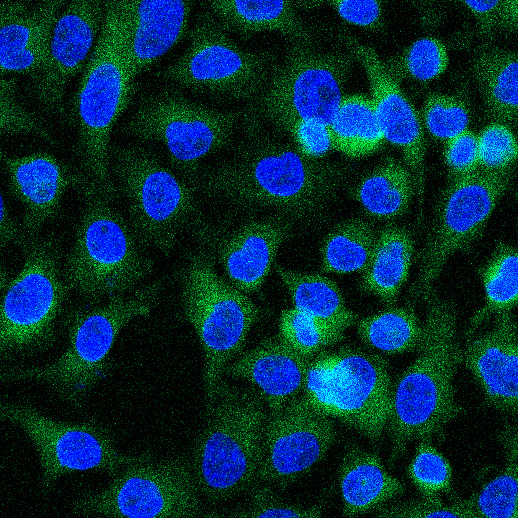

Supplement: Supplementary file 7 [file DataSheet13.ZIP › No Scale/LO2 (No Scale)/l8-40-单个文件导出-02_c2+3.jpg]

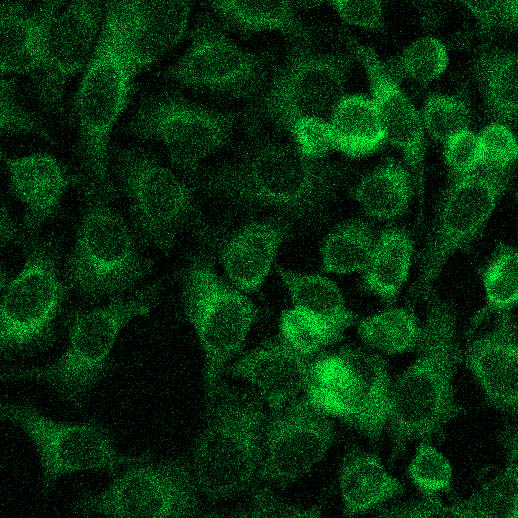

Supplement: Supplementary file 7 [file DataSheet13.ZIP › No Scale/LO2 (No Scale)/l8-40-单个文件导出-02_c2.jpg]

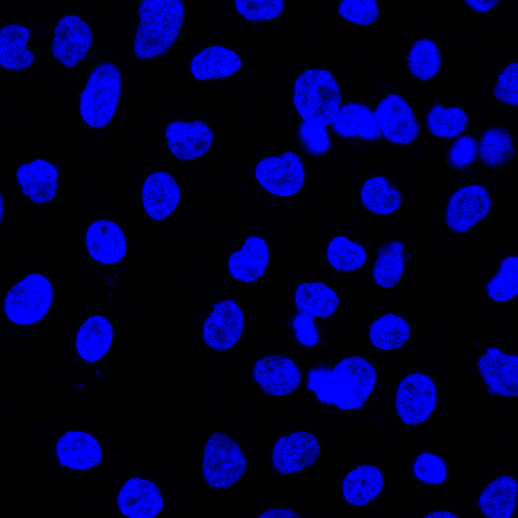

Supplement: Supplementary file 7 [file DataSheet13.ZIP › No Scale/LO2 (No Scale)/l8-40-单个文件导出-02_c3.tif]

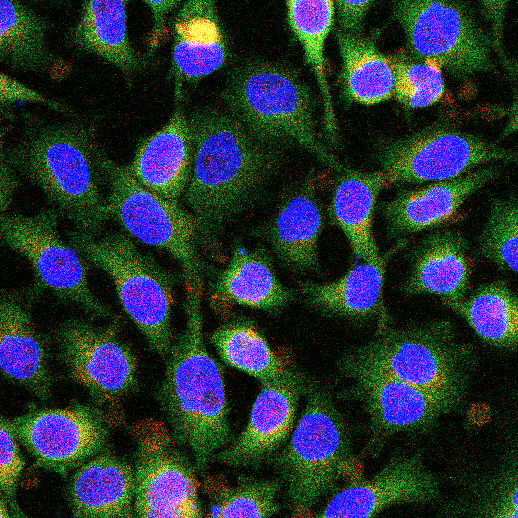

Supplement: Supplementary file 7 [file DataSheet13.ZIP › No Scale/LO2 (No Scale)/l9-40-单个文件导出-03_c1+2+3.tif]

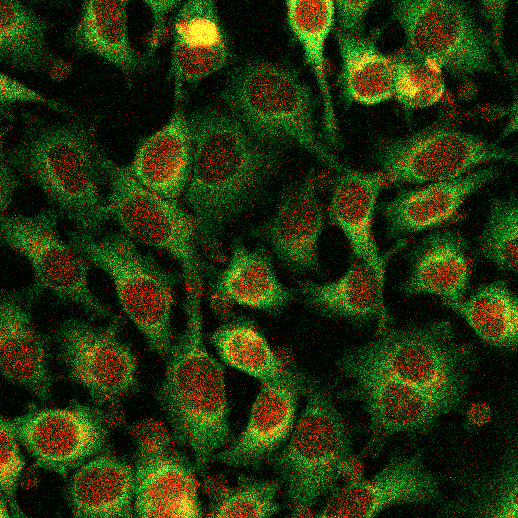

Supplement: Supplementary file 7 [file DataSheet13.ZIP › No Scale/LO2 (No Scale)/l9-40-单个文件导出-03_c1+2.tif]

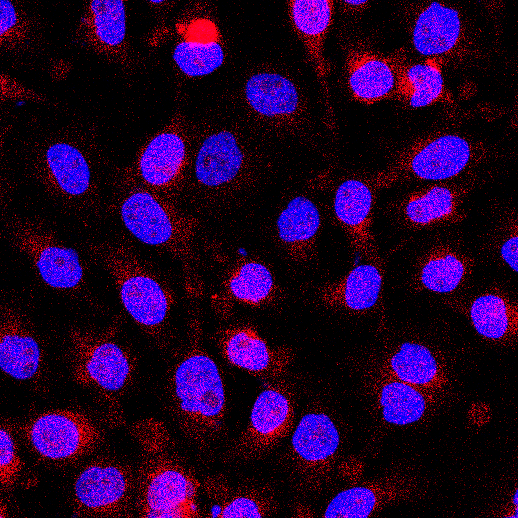

Supplement: Supplementary file 7 [file DataSheet13.ZIP › No Scale/LO2 (No Scale)/l9-40-单个文件导出-03_c1+3.tif]

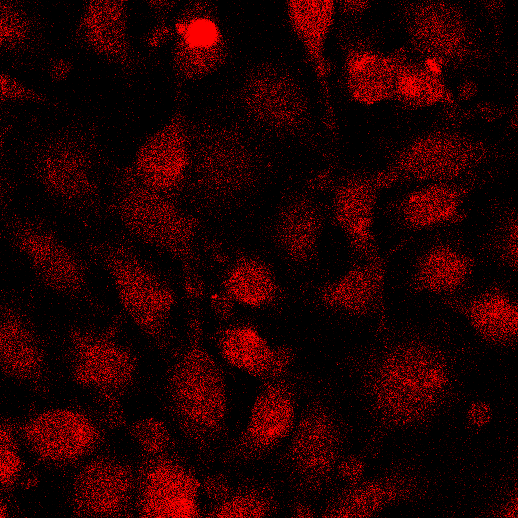

Supplement: Supplementary file 7 [file DataSheet13.ZIP › No Scale/LO2 (No Scale)/l9-40-单个文件导出-03_c1.tif]

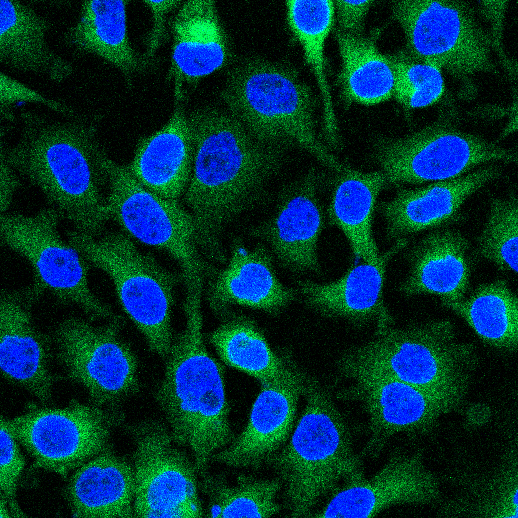

Supplement: Supplementary file 7 [file DataSheet13.ZIP › No Scale/LO2 (No Scale)/l9-40-单个文件导出-03_c2+3.tif]

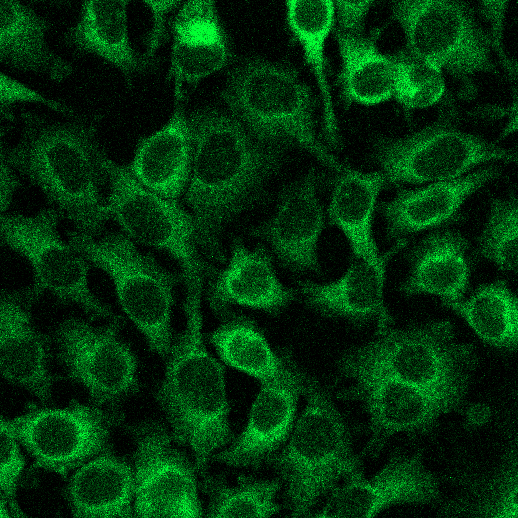

Supplement: Supplementary file 7 [file DataSheet13.ZIP › No Scale/LO2 (No Scale)/l9-40-单个文件导出-03_c2.tif]

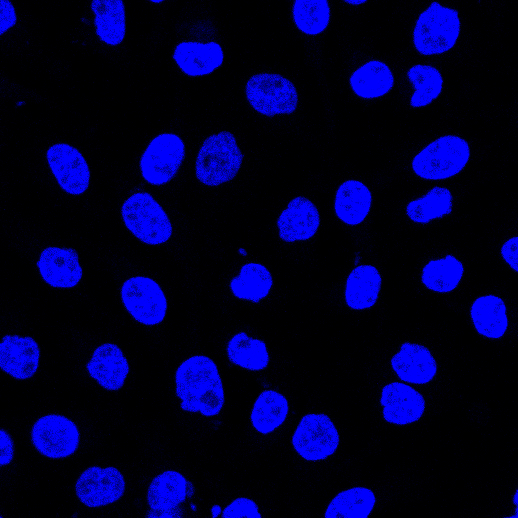

Supplement: Supplementary file 7 [file DataSheet13.ZIP › No Scale/LO2 (No Scale)/l9-40-单个文件导出-03_c3.jpg]

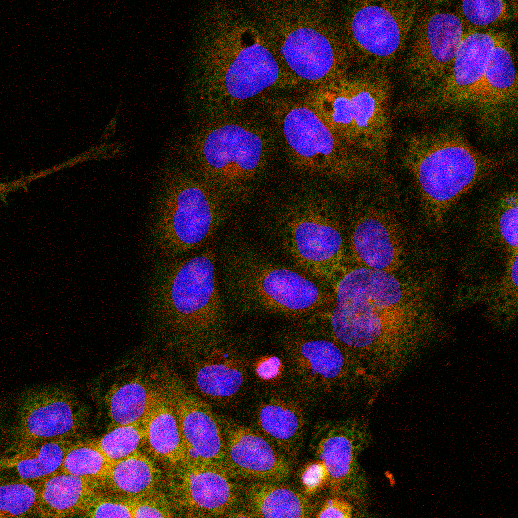

Supplement: Supplementary file 7 [file DataSheet13.ZIP › No Scale/sk-hep-1 (No Scale)/C2309 (1)-单个文件导出-01_c1+2+3.tif]

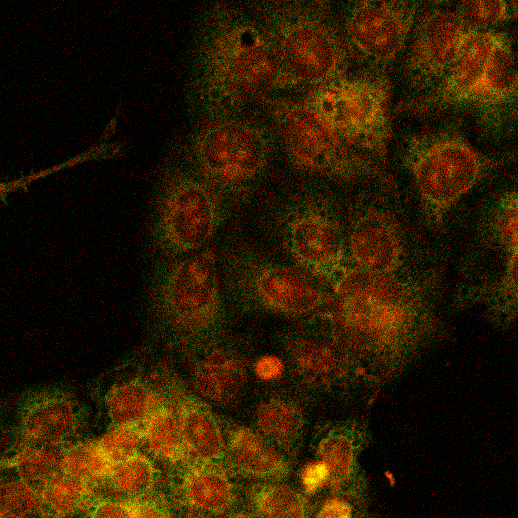

Supplement: Supplementary file 7 [file DataSheet13.ZIP › No Scale/sk-hep-1 (No Scale)/C2309 (1)-单个文件导出-01_c1+2.tif]

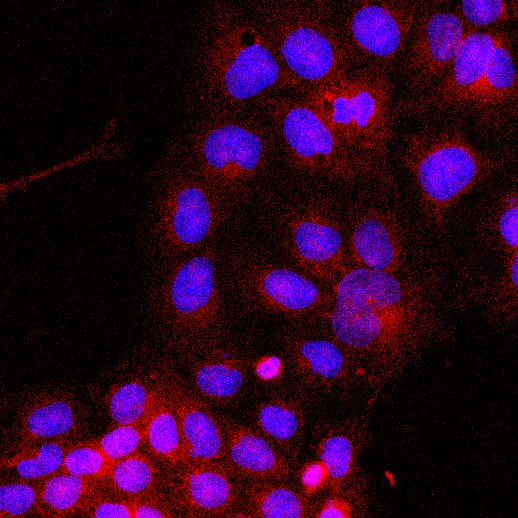

Supplement: Supplementary file 7 [file DataSheet13.ZIP › No Scale/sk-hep-1 (No Scale)/C2309 (1)-单个文件导出-01_c1+3.tif]

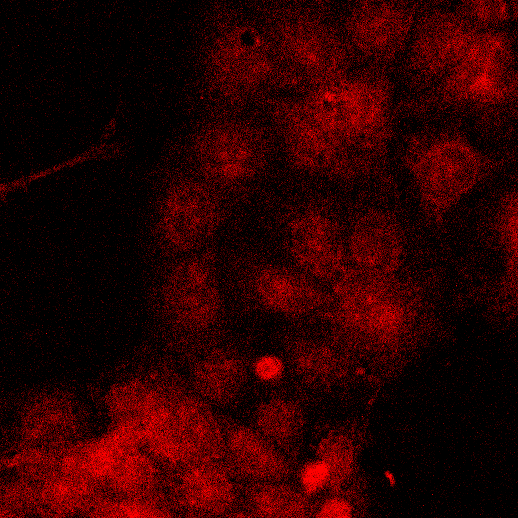

Supplement: Supplementary file 7 [file DataSheet13.ZIP › No Scale/sk-hep-1 (No Scale)/C2309 (1)-单个文件导出-01_c1.tif]

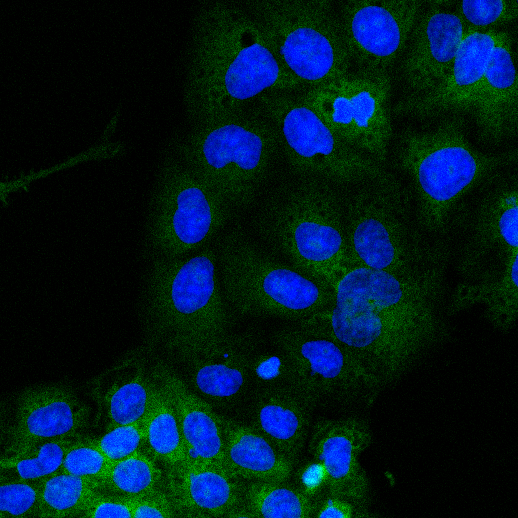

Supplement: Supplementary file 7 [file DataSheet13.ZIP › No Scale/sk-hep-1 (No Scale)/C2309 (1)-单个文件导出-01_c2+3.tif]

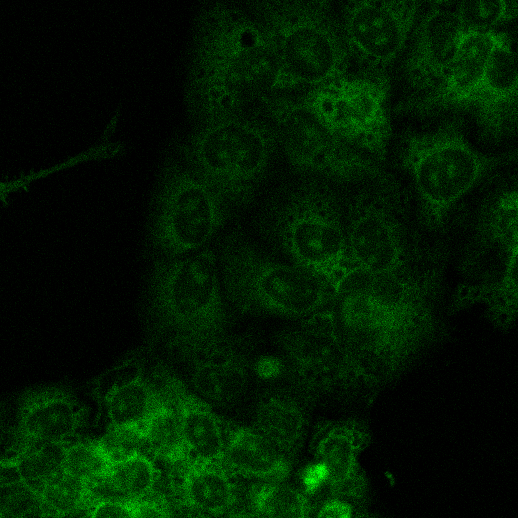

Supplement: Supplementary file 7 [file DataSheet13.ZIP › No Scale/sk-hep-1 (No Scale)/C2309 (1)-单个文件导出-01_c2.tif]

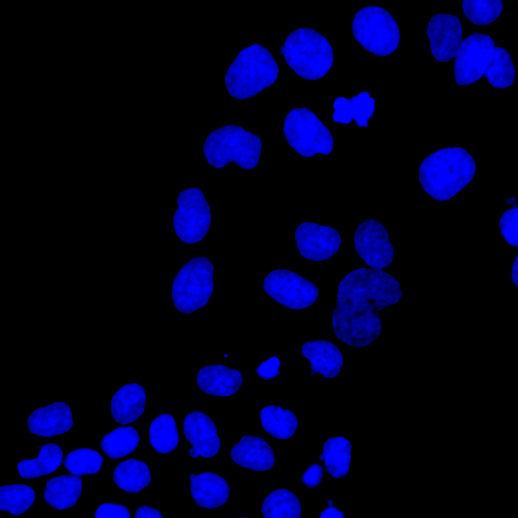

Supplement: Supplementary file 7 [file DataSheet13.ZIP › No Scale/sk-hep-1 (No Scale)/C2309 (1)-单个文件导出-01_c3.tif]

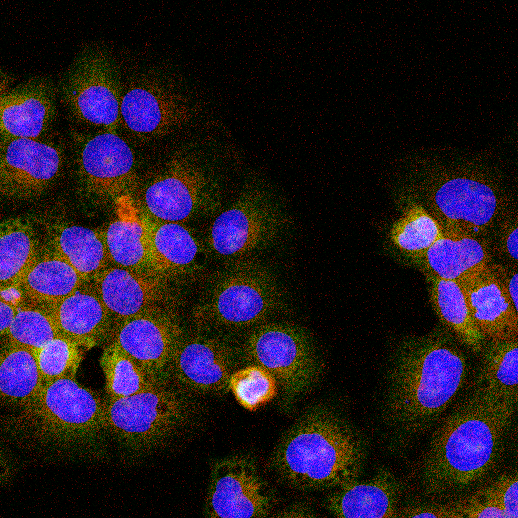

Supplement: Supplementary file 7 [file DataSheet13.ZIP › No Scale/sk-hep-1 (No Scale)/C2309 (2)-单个文件导出-02_c1+2+3.tif]

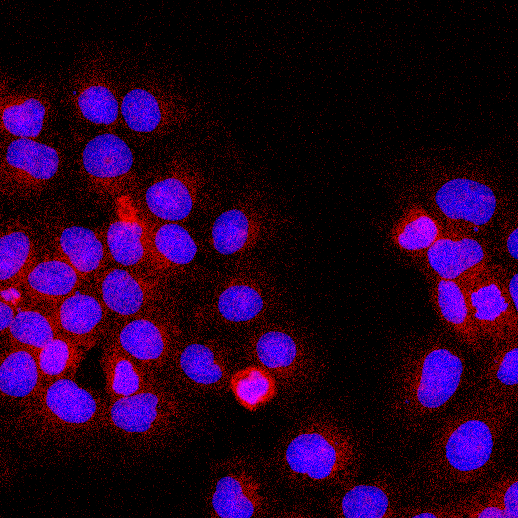

Supplement: Supplementary file 7 [file DataSheet13.ZIP › No Scale/sk-hep-1 (No Scale)/C2309 (2)-单个文件导出-02_c1+3.tif]

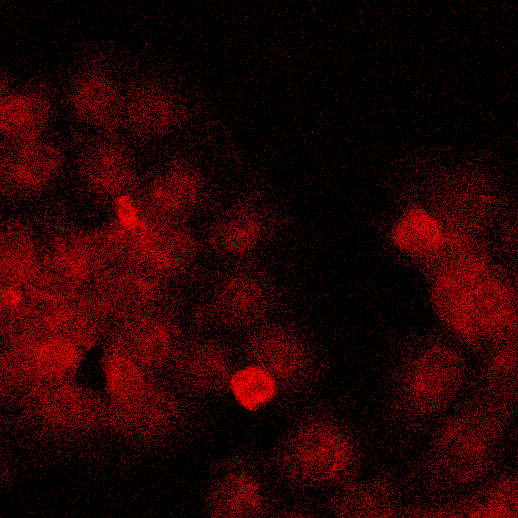

Supplement: Supplementary file 7 [file DataSheet13.ZIP › No Scale/sk-hep-1 (No Scale)/C2309 (2)-单个文件导出-02_c1.jpg]

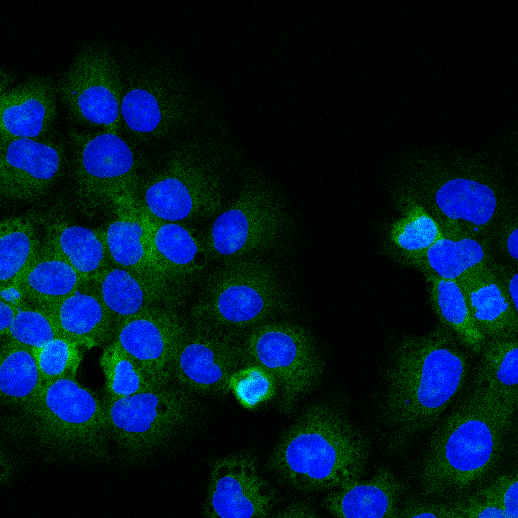

Supplement: Supplementary file 7 [file DataSheet13.ZIP › No Scale/sk-hep-1 (No Scale)/C2309 (2)-单个文件导出-02_c2+3.tif]

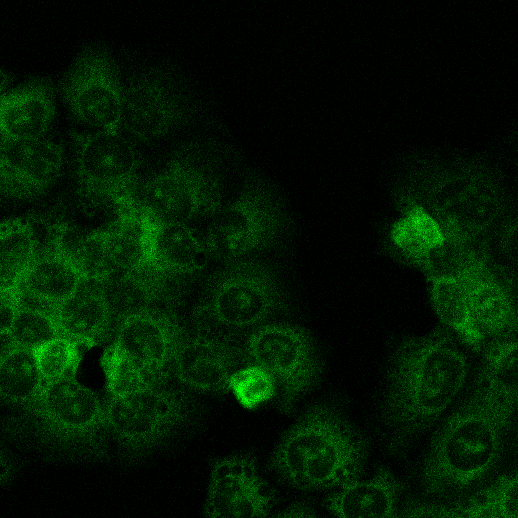

Supplement: Supplementary file 7 [file DataSheet13.ZIP › No Scale/sk-hep-1 (No Scale)/C2309 (2)-单个文件导出-02_c2.tif]

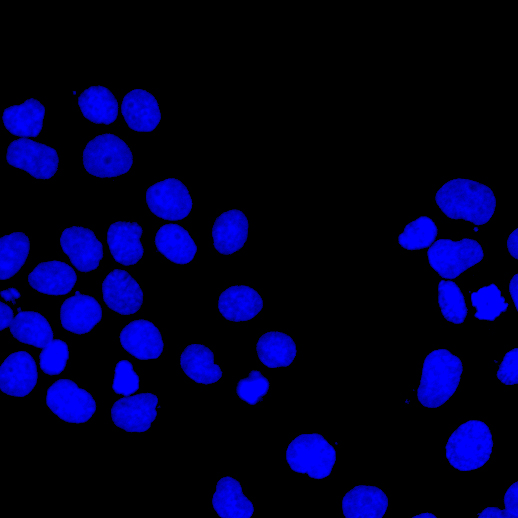

Supplement: Supplementary file 7 [file DataSheet13.ZIP › No Scale/sk-hep-1 (No Scale)/C2309 (2)-单个文件导出-02_c3.jpg]

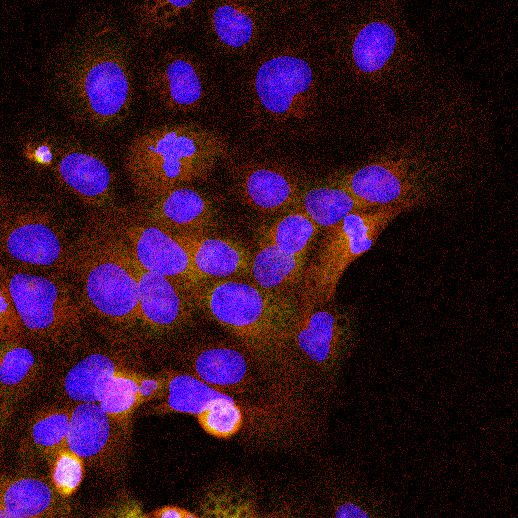

Supplement: Supplementary file 7 [file DataSheet13.ZIP › No Scale/sk-hep-1 (No Scale)/C2309 (3)-单个文件导出-03_c1+2+3.tif]

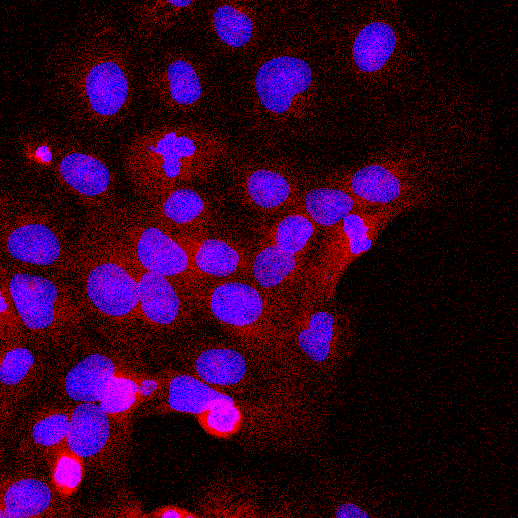

Supplement: Supplementary file 7 [file DataSheet13.ZIP › No Scale/sk-hep-1 (No Scale)/C2309 (3)-单个文件导出-03_c1+3.tif]

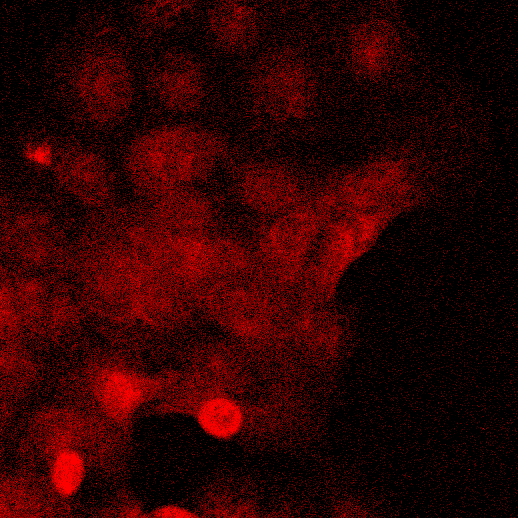

Supplement: Supplementary file 7 [file DataSheet13.ZIP › No Scale/sk-hep-1 (No Scale)/C2309 (3)-单个文件导出-03_c1.tif]

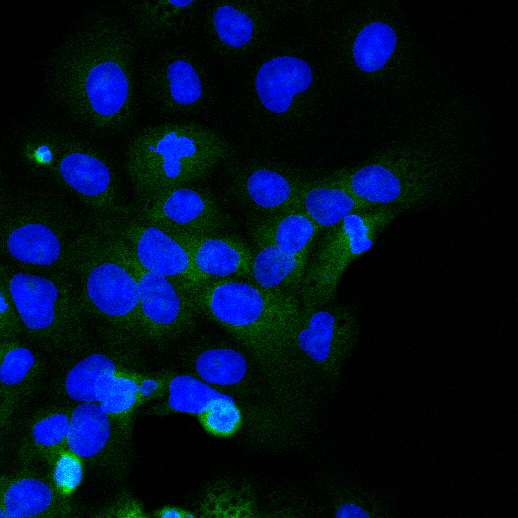

Supplement: Supplementary file 7 [file DataSheet13.ZIP › No Scale/sk-hep-1 (No Scale)/C2309 (3)-单个文件导出-03_c2+3.jpg]

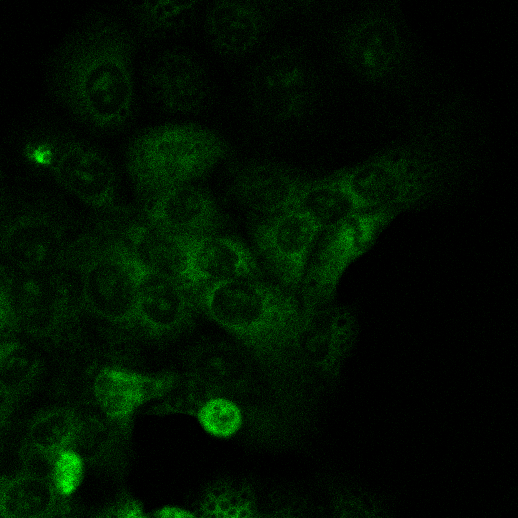

Supplement: Supplementary file 7 [file DataSheet13.ZIP › No Scale/sk-hep-1 (No Scale)/C2309 (3)-单个文件导出-03_c2.tif]

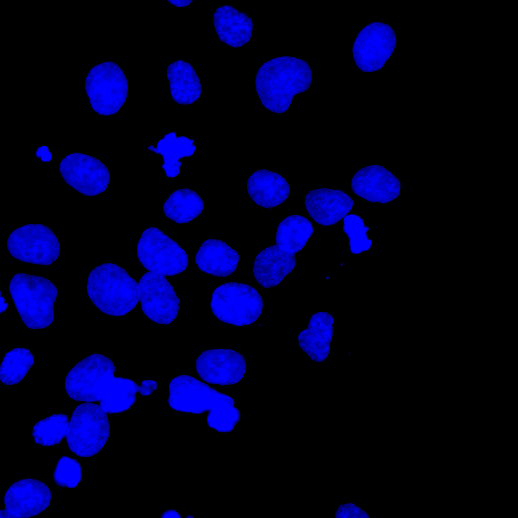

Supplement: Supplementary file 7 [file DataSheet13.ZIP › No Scale/sk-hep-1 (No Scale)/C2309 (3)-单个文件导出-03_c3.tif]

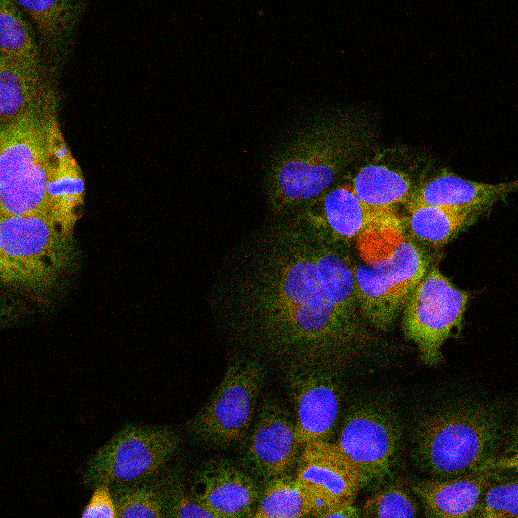

Supplement: Supplementary file 7 [file DataSheet13.ZIP › No Scale/sk-hep-1 (No Scale)/C2309 (4)-单个文件导出-04_c1+2+3.jpg]

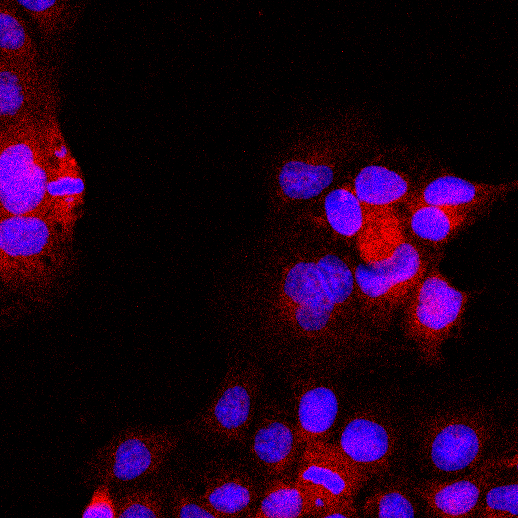

Supplement: Supplementary file 7 [file DataSheet13.ZIP › No Scale/sk-hep-1 (No Scale)/C2309 (4)-单个文件导出-04_c1+3.jpg]

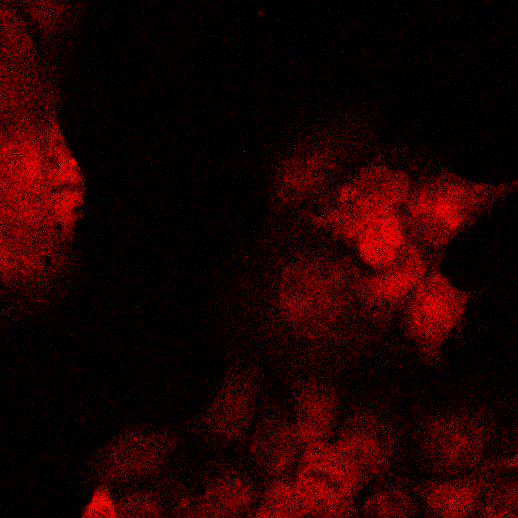

Supplement: Supplementary file 7 [file DataSheet13.ZIP › No Scale/sk-hep-1 (No Scale)/C2309 (4)-单个文件导出-04_c1.tif]

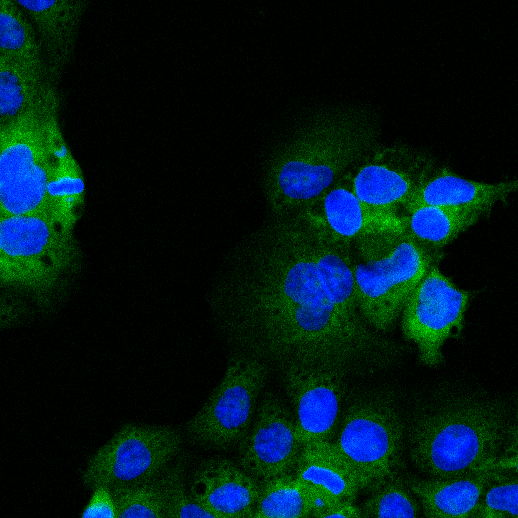

Supplement: Supplementary file 7 [file DataSheet13.ZIP › No Scale/sk-hep-1 (No Scale)/C2309 (4)-单个文件导出-04_c2+3.tif]

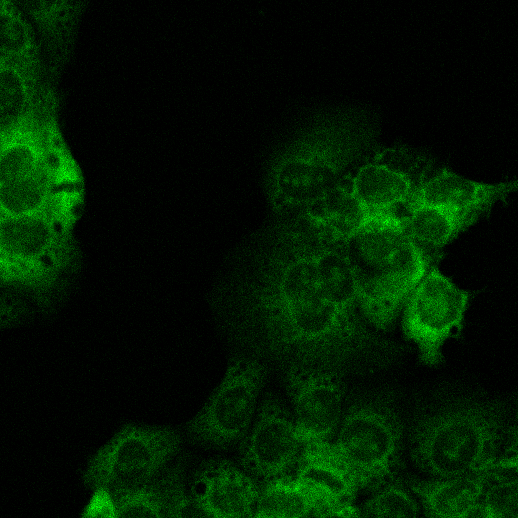

Supplement: Supplementary file 7 [file DataSheet13.ZIP › No Scale/sk-hep-1 (No Scale)/C2309 (4)-单个文件导出-04_c2.tif]

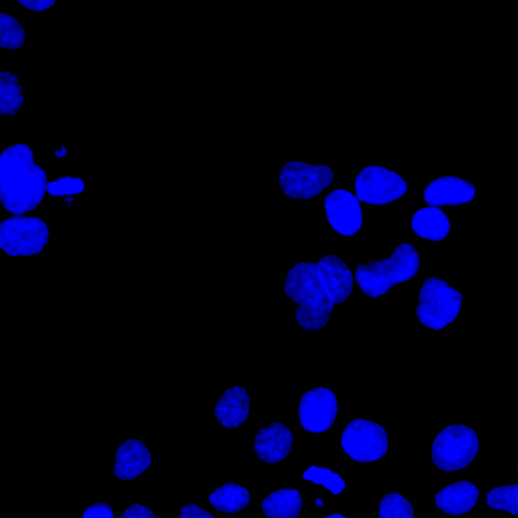

Supplement: Supplementary file 7 [file DataSheet13.ZIP › No Scale/sk-hep-1 (No Scale)/C2309 (4)-单个文件导出-04_c3.tif]

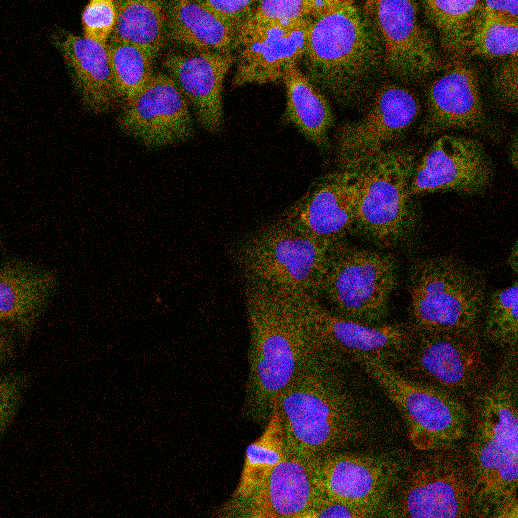

Supplement: Supplementary file 7 [file DataSheet13.ZIP › No Scale/sk-hep-1 (No Scale)/C2309 (5)-单个文件导出-05_c1+2+3.tif]

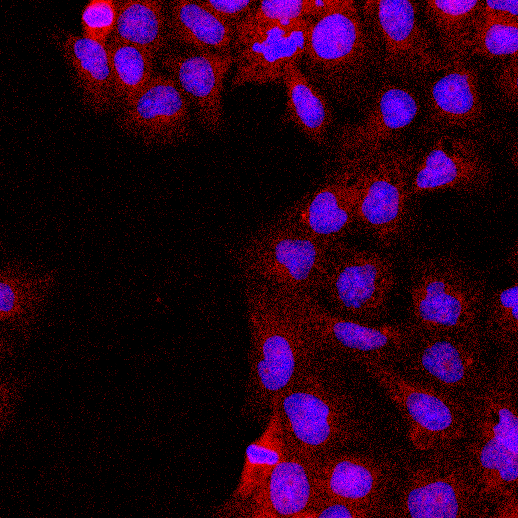

Supplement: Supplementary file 7 [file DataSheet13.ZIP › No Scale/sk-hep-1 (No Scale)/C2309 (5)-单个文件导出-05_c1+3.tif]

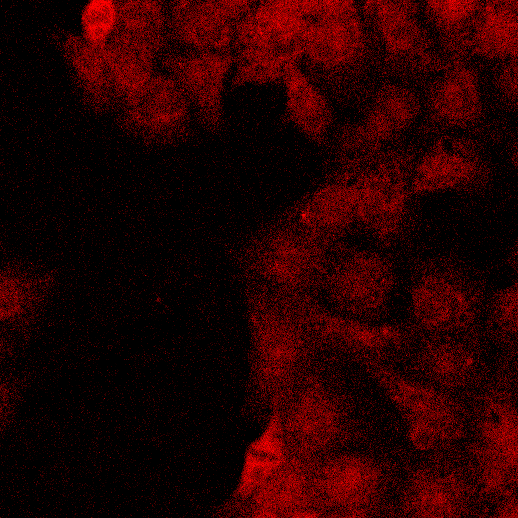

Supplement: Supplementary file 7 [file DataSheet13.ZIP › No Scale/sk-hep-1 (No Scale)/C2309 (5)-单个文件导出-05_c1.tif]

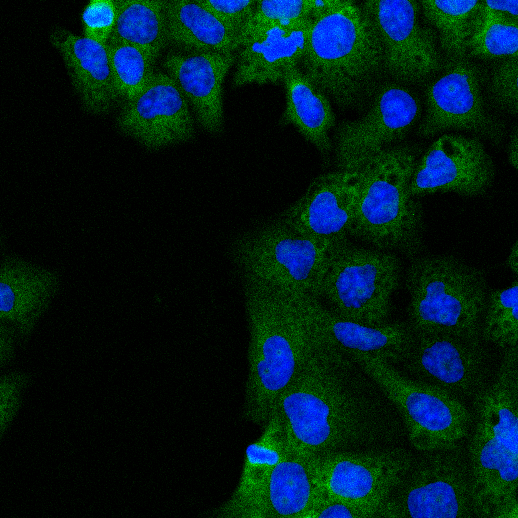

Supplement: Supplementary file 7 [file DataSheet13.ZIP › No Scale/sk-hep-1 (No Scale)/C2309 (5)-单个文件导出-05_c2+3.tif]

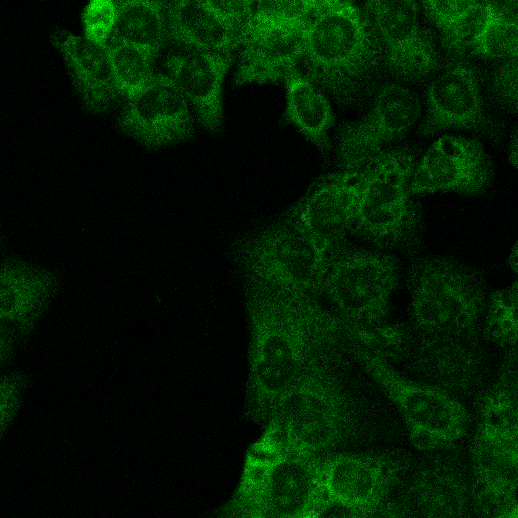

Supplement: Supplementary file 7 [file DataSheet13.ZIP › No Scale/sk-hep-1 (No Scale)/C2309 (5)-单个文件导出-05_c2.tif]

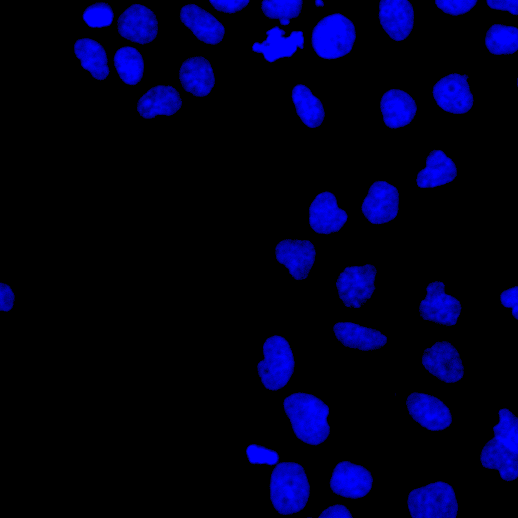

Supplement: Supplementary file 7 [file DataSheet13.ZIP › No Scale/sk-hep-1 (No Scale)/C2309 (5)-单个文件导出-05_c3.tif]

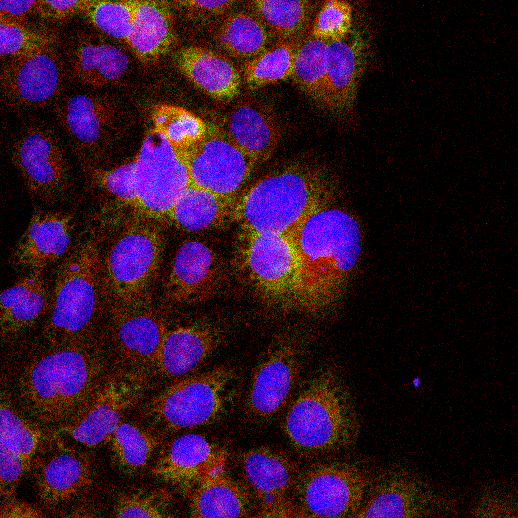

Supplement: Supplementary file 7 [file DataSheet13.ZIP › No Scale/sk-hep-1 (No Scale)/C2309 (6)-单个文件导出-06_c1+2+3.tif]

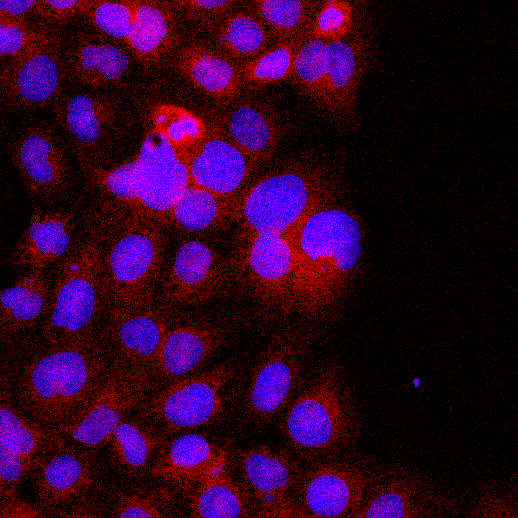

Supplement: Supplementary file 7 [file DataSheet13.ZIP › No Scale/sk-hep-1 (No Scale)/C2309 (6)-单个文件导出-06_c1+3.tif]

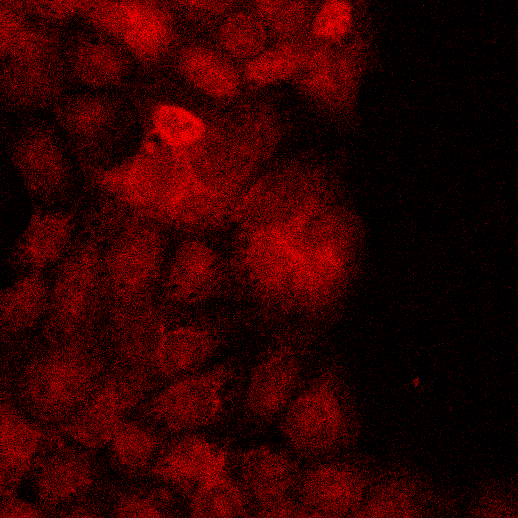

Supplement: Supplementary file 7 [file DataSheet13.ZIP › No Scale/sk-hep-1 (No Scale)/C2309 (6)-单个文件导出-06_c1.tif]

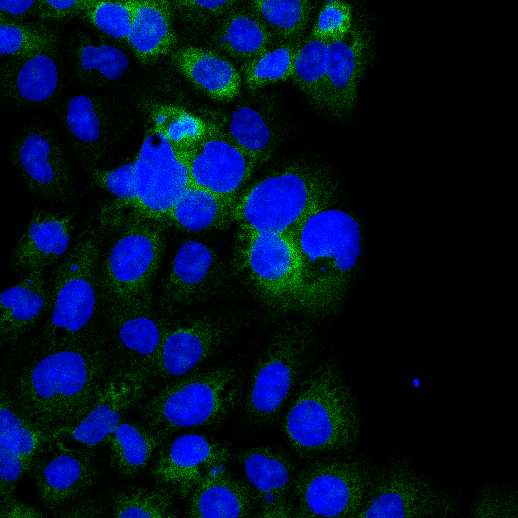

Supplement: Supplementary file 7 [file DataSheet13.ZIP › No Scale/sk-hep-1 (No Scale)/C2309 (6)-单个文件导出-06_c2+3.tif]

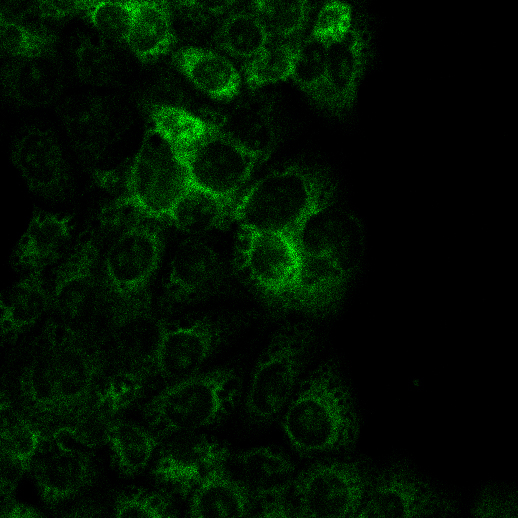

Supplement: Supplementary file 7 [file DataSheet13.ZIP › No Scale/sk-hep-1 (No Scale)/C2309 (6)-单个文件导出-06_c2.jpg]

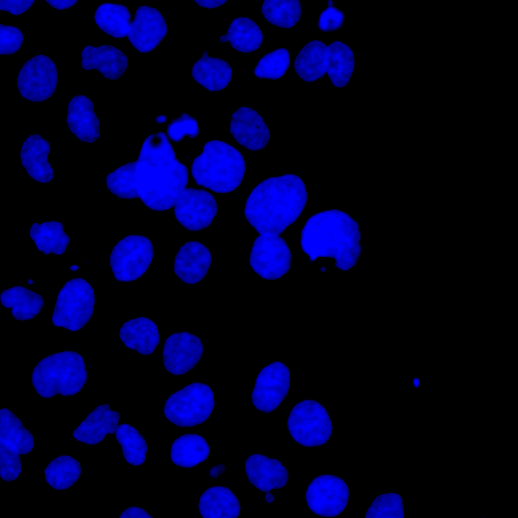

Supplement: Supplementary file 7 [file DataSheet13.ZIP › No Scale/sk-hep-1 (No Scale)/C2309 (6)-单个文件导出-06_c3.tif]

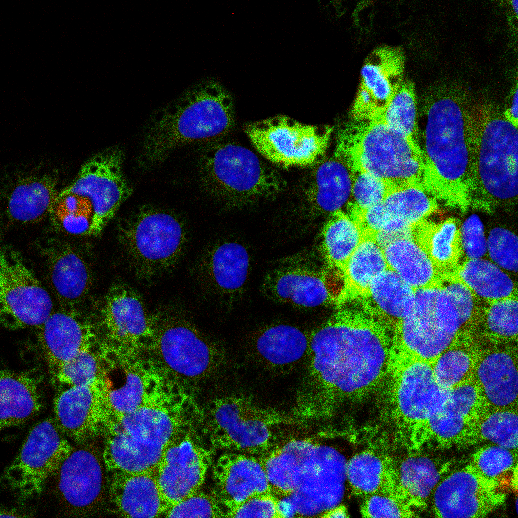

Supplement: Supplementary file 7 [file DataSheet13.ZIP › No Scale/sk-hep-1 (No Scale)/pLO5 (1)-单个文件导出-07_c1+2+3.tif]

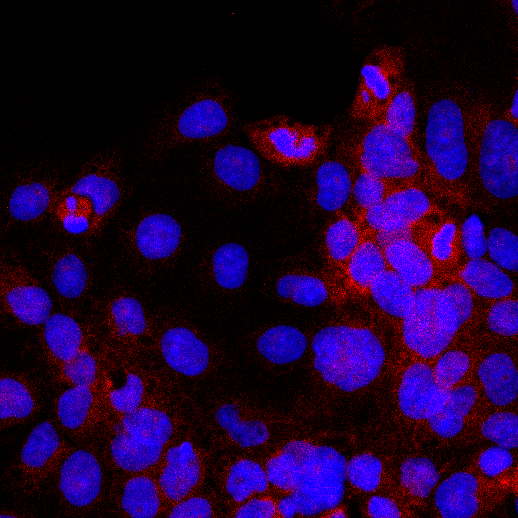

Supplement: Supplementary file 7 [file DataSheet13.ZIP › No Scale/sk-hep-1 (No Scale)/pLO5 (1)-单个文件导出-07_c1+3.tif]

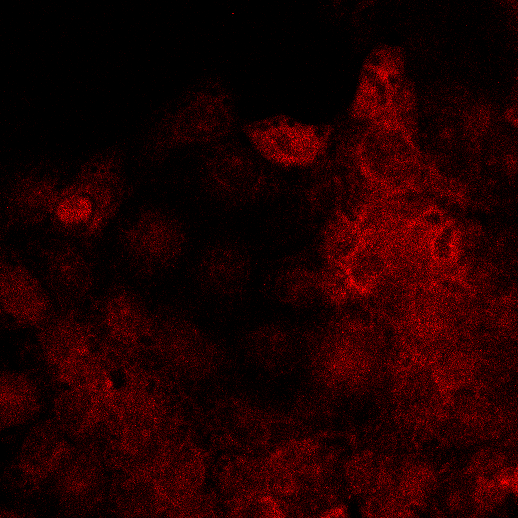

Supplement: Supplementary file 7 [file DataSheet13.ZIP › No Scale/sk-hep-1 (No Scale)/pLO5 (1)-单个文件导出-07_c1.tif]

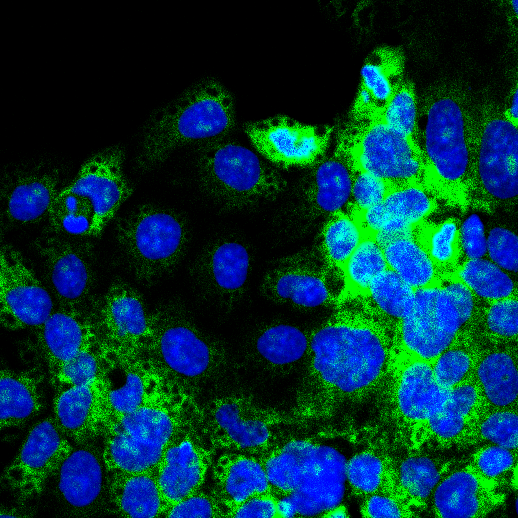

Supplement: Supplementary file 7 [file DataSheet13.ZIP › No Scale/sk-hep-1 (No Scale)/pLO5 (1)-单个文件导出-07_c2+3.tif]

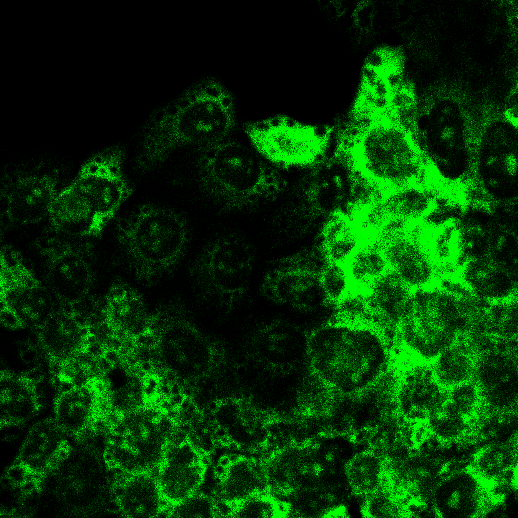

Supplement: Supplementary file 7 [file DataSheet13.ZIP › No Scale/sk-hep-1 (No Scale)/pLO5 (1)-单个文件导出-07_c2.tif]

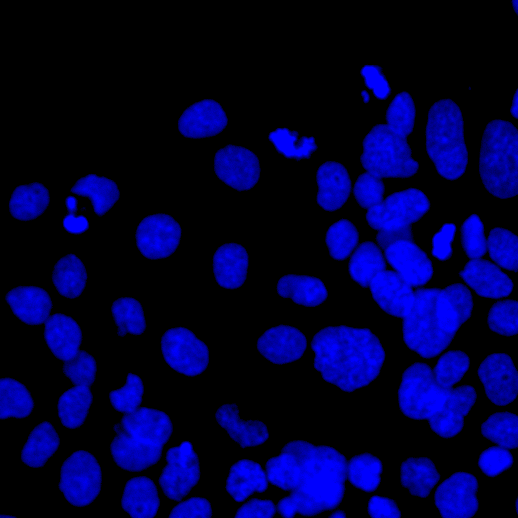

Supplement: Supplementary file 7 [file DataSheet13.ZIP › No Scale/sk-hep-1 (No Scale)/pLO5 (1)-单个文件导出-07_c3.tif]

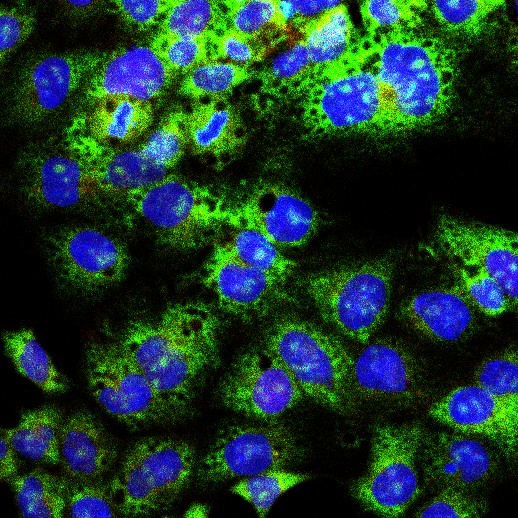

Supplement: Supplementary file 7 [file DataSheet13.ZIP › No Scale/sk-hep-1 (No Scale)/pLO5 (2)-单个文件导出-08_c1+2+3.tif]

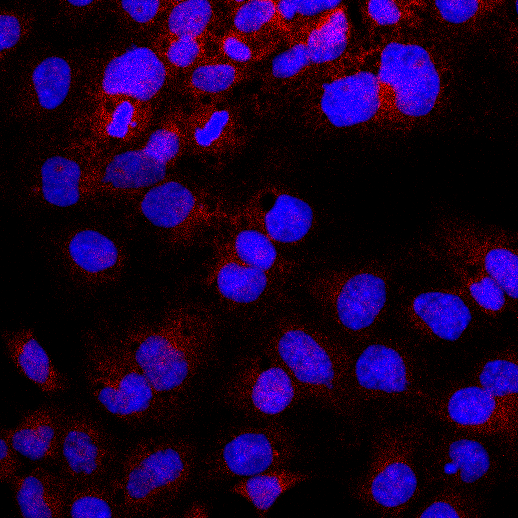

Supplement: Supplementary file 7 [file DataSheet13.ZIP › No Scale/sk-hep-1 (No Scale)/pLO5 (2)-单个文件导出-08_c1+3.tif]

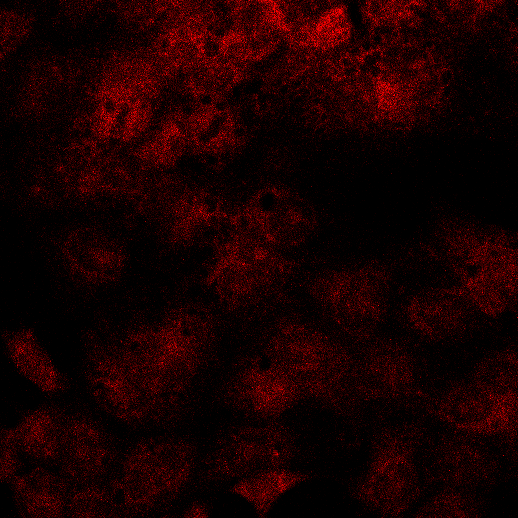

Supplement: Supplementary file 7 [file DataSheet13.ZIP › No Scale/sk-hep-1 (No Scale)/pLO5 (2)-单个文件导出-08_c1.tif]

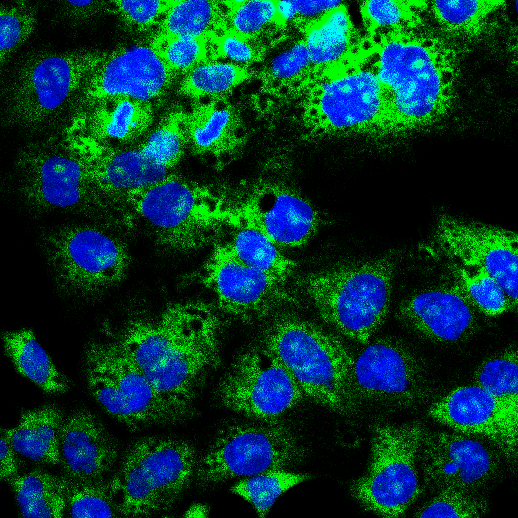

Supplement: Supplementary file 7 [file DataSheet13.ZIP › No Scale/sk-hep-1 (No Scale)/pLO5 (2)-单个文件导出-08_c2+3.tif]

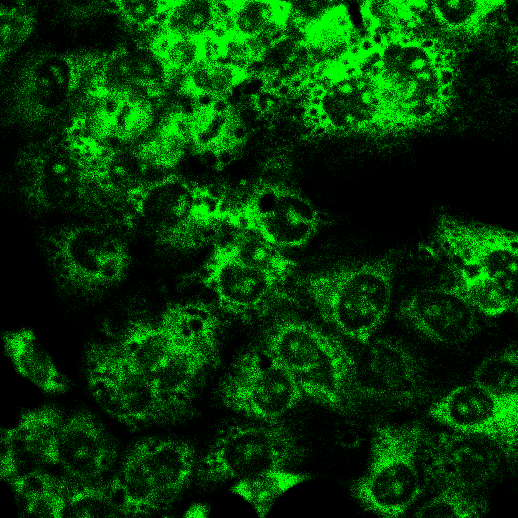

Supplement: Supplementary file 7 [file DataSheet13.ZIP › No Scale/sk-hep-1 (No Scale)/pLO5 (2)-单个文件导出-08_c2.tif]

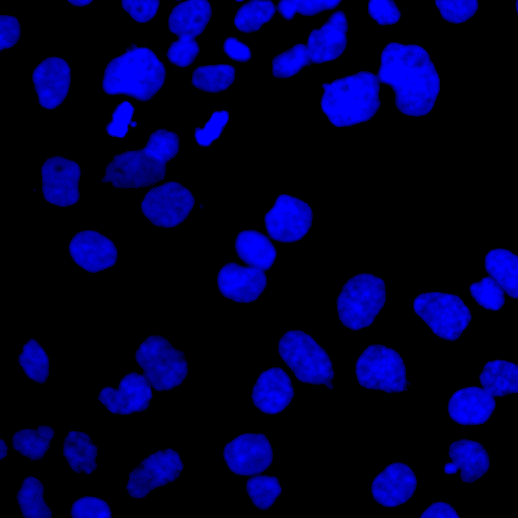

Supplement: Supplementary file 7 [file DataSheet13.ZIP › No Scale/sk-hep-1 (No Scale)/pLO5 (2)-单个文件导出-08_c3.tif]

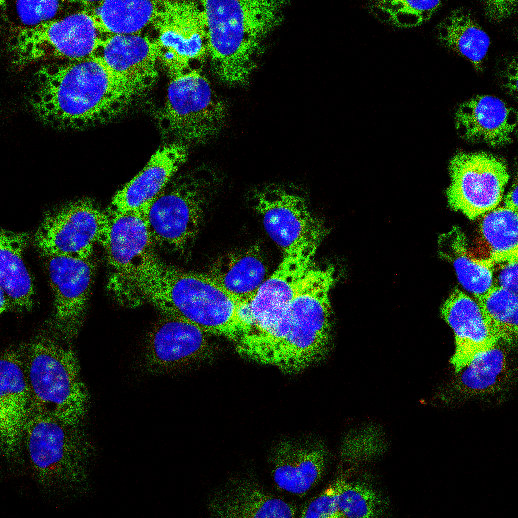

Supplement: Supplementary file 7 [file DataSheet13.ZIP › No Scale/sk-hep-1 (No Scale)/pLO5 (3)-单个文件导出-09_c1+2+3.tif]

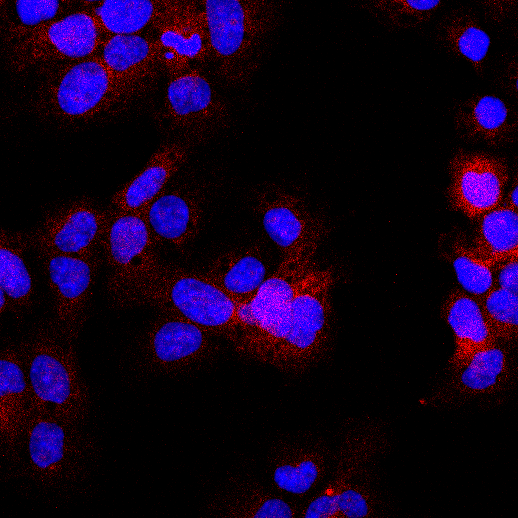

Supplement: Supplementary file 7 [file DataSheet13.ZIP › No Scale/sk-hep-1 (No Scale)/pLO5 (3)-单个文件导出-09_c1+3.tif]

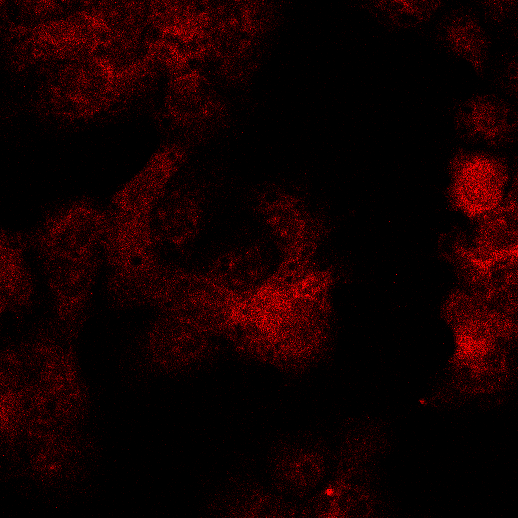

Supplement: Supplementary file 7 [file DataSheet13.ZIP › No Scale/sk-hep-1 (No Scale)/pLO5 (3)-单个文件导出-09_c1.tif]

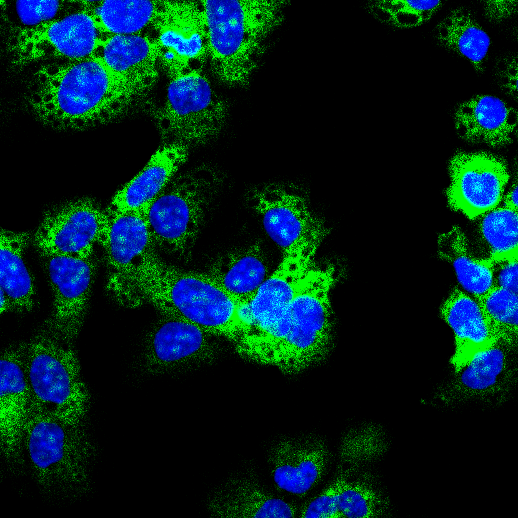

Supplement: Supplementary file 7 [file DataSheet13.ZIP › No Scale/sk-hep-1 (No Scale)/pLO5 (3)-单个文件导出-09_c2+3.tif]

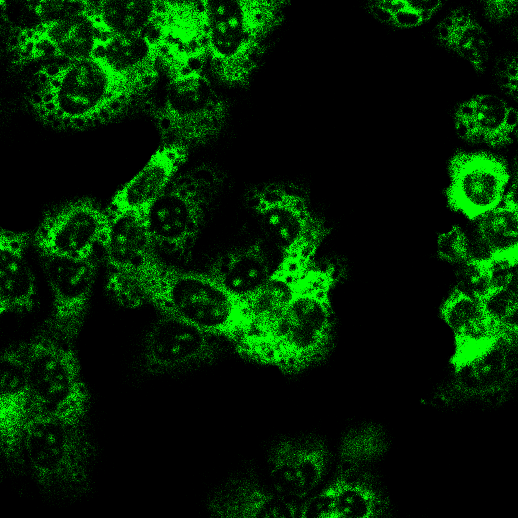

Supplement: Supplementary file 7 [file DataSheet13.ZIP › No Scale/sk-hep-1 (No Scale)/pLO5 (3)-单个文件导出-09_c2.tif]

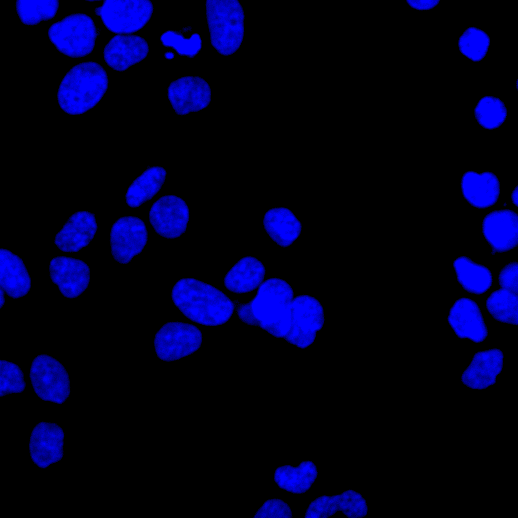

Supplement: Supplementary file 7 [file DataSheet13.ZIP › No Scale/sk-hep-1 (No Scale)/pLO5 (3)-单个文件导出-09_c3.tif]

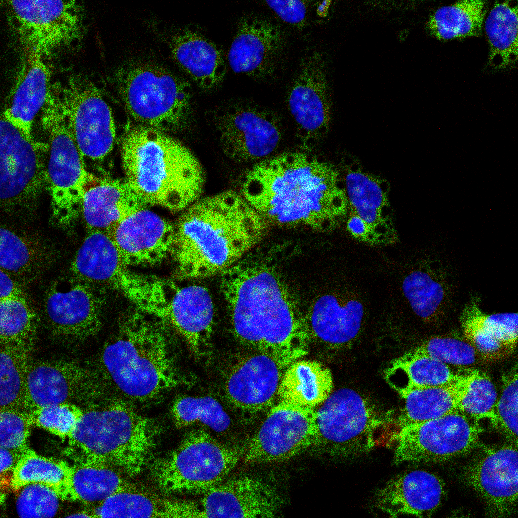

Supplement: Supplementary file 7 [file DataSheet13.ZIP › No Scale/sk-hep-1 (No Scale)/pLO5 (4)-单个文件导出-10_c1+2+3.tif]

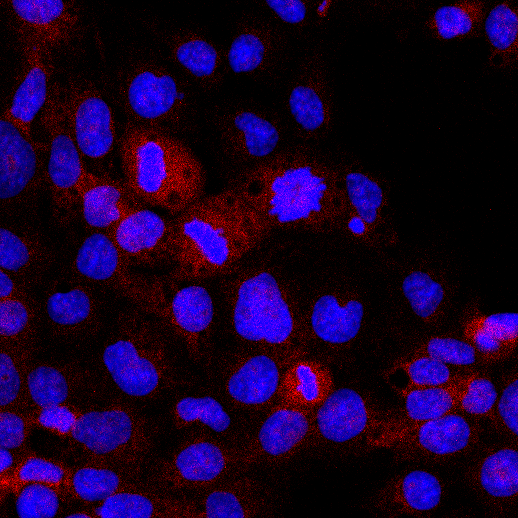

Supplement: Supplementary file 7 [file DataSheet13.ZIP › No Scale/sk-hep-1 (No Scale)/pLO5 (4)-单个文件导出-10_c1+3.tif]

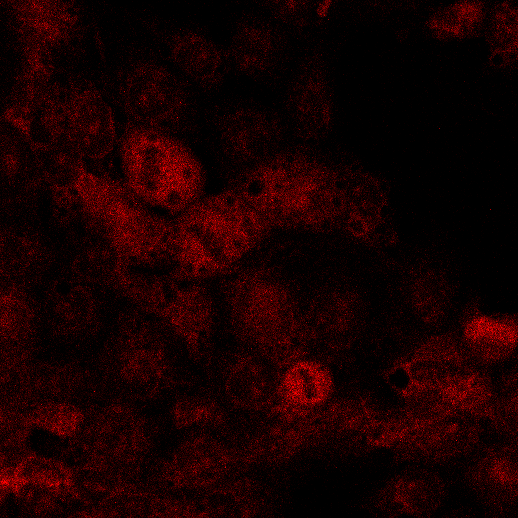

Supplement: Supplementary file 7 [file DataSheet13.ZIP › No Scale/sk-hep-1 (No Scale)/pLO5 (4)-单个文件导出-10_c1.tif]

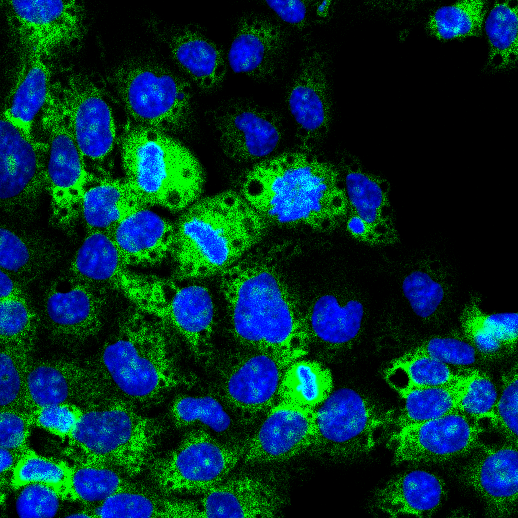

Supplement: Supplementary file 7 [file DataSheet13.ZIP › No Scale/sk-hep-1 (No Scale)/pLO5 (4)-单个文件导出-10_c2+3.tif]

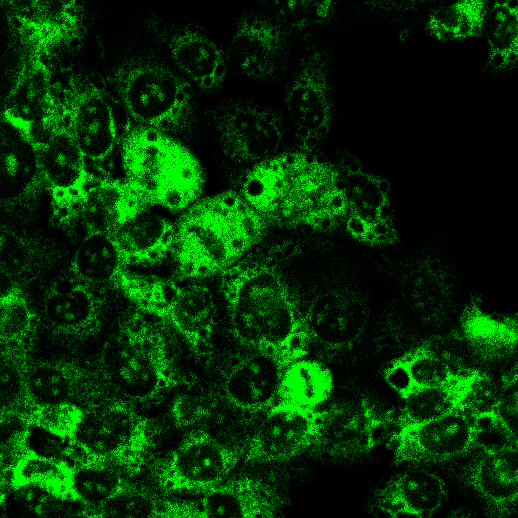

Supplement: Supplementary file 7 [file DataSheet13.ZIP › No Scale/sk-hep-1 (No Scale)/pLO5 (4)-单个文件导出-10_c2.tif]

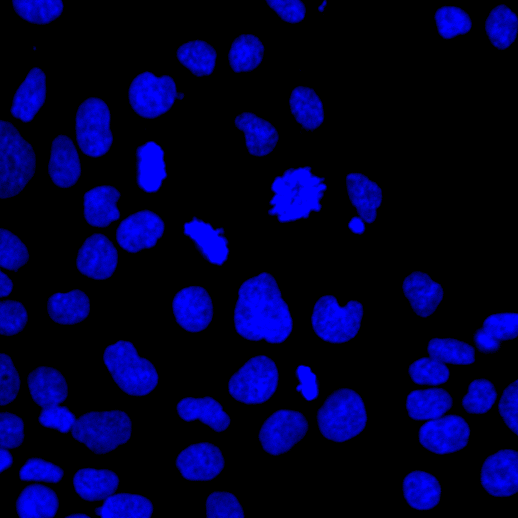

Supplement: Supplementary file 7 [file DataSheet13.ZIP › No Scale/sk-hep-1 (No Scale)/pLO5 (4)-单个文件导出-10_c3.tif]

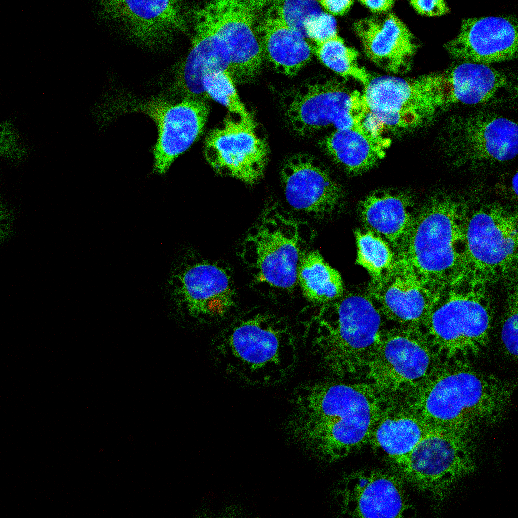

Supplement: Supplementary file 7 [file DataSheet13.ZIP › No Scale/sk-hep-1 (No Scale)/pLO5 (5)-单个文件导出-11_c1+2+3.tif]

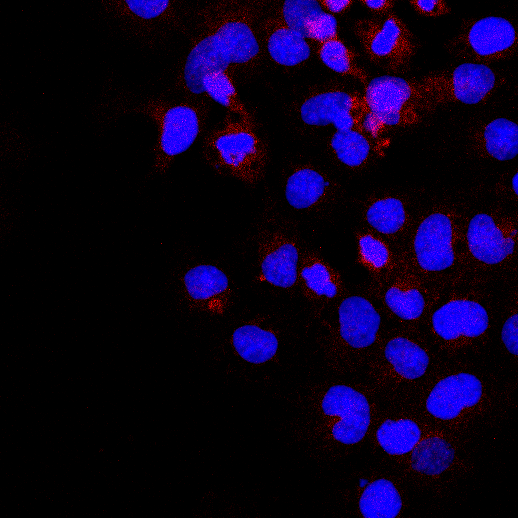

Supplement: Supplementary file 7 [file DataSheet13.ZIP › No Scale/sk-hep-1 (No Scale)/pLO5 (5)-单个文件导出-11_c1+3.tif]

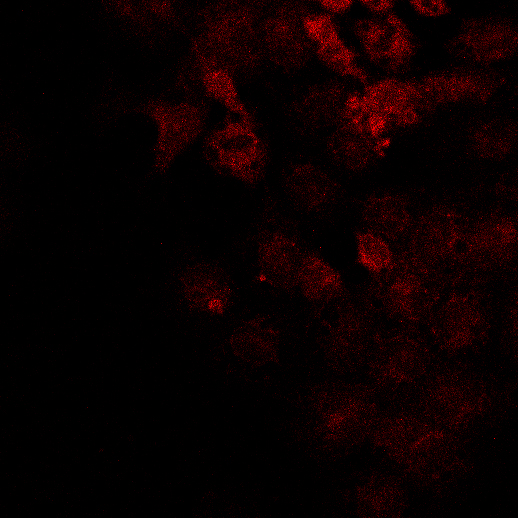

Supplement: Supplementary file 7 [file DataSheet13.ZIP › No Scale/sk-hep-1 (No Scale)/pLO5 (5)-单个文件导出-11_c1.jpg]

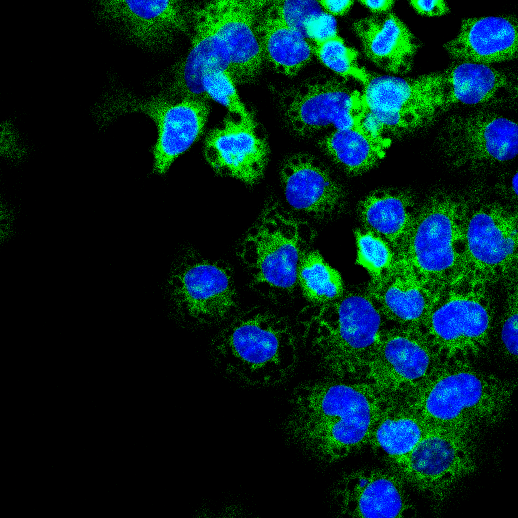

Supplement: Supplementary file 7 [file DataSheet13.ZIP › No Scale/sk-hep-1 (No Scale)/pLO5 (5)-单个文件导出-11_c2+3.tif]

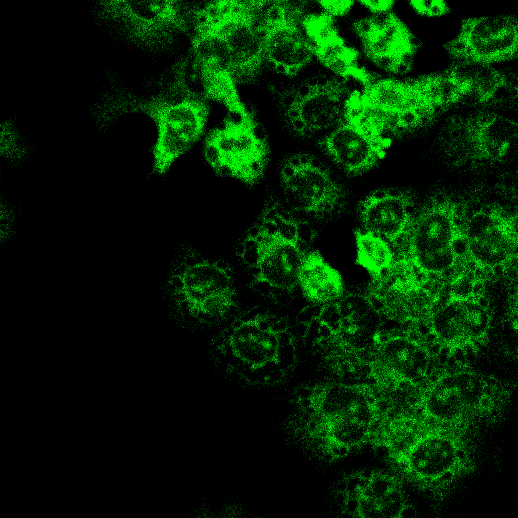

Supplement: Supplementary file 7 [file DataSheet13.ZIP › No Scale/sk-hep-1 (No Scale)/pLO5 (5)-单个文件导出-11_c2.tif]

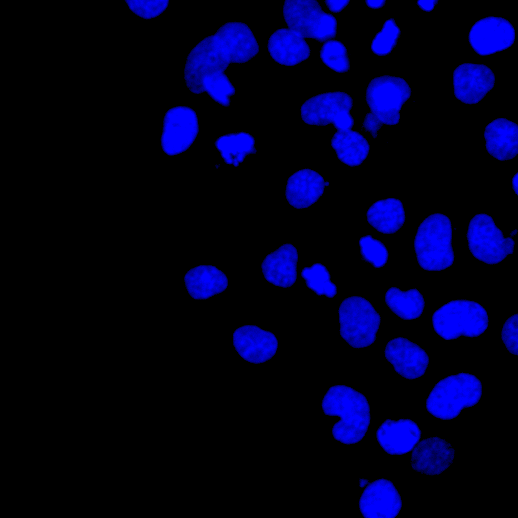

Supplement: Supplementary file 7 [file DataSheet13.ZIP › No Scale/sk-hep-1 (No Scale)/pLO5 (5)-单个文件导出-11_c3.tif]
