# Supplementary material for: Downregulation of circLIFR exerts cancer-promoting effects on hepatocellular carcinoma in vitro
Source: Front Genet. 2022 Sep 12;13:986322. doi: 10.3389/fgene.2022.986322 (PMC9513674; doi:10.3389/fgene.2022.986322)
Supplement: Supplementary file 12 [file DataSheet2.ZIP › Cell function experiment/Cell cycle assay/hep-G2 cell/G2 C-1.pdf]

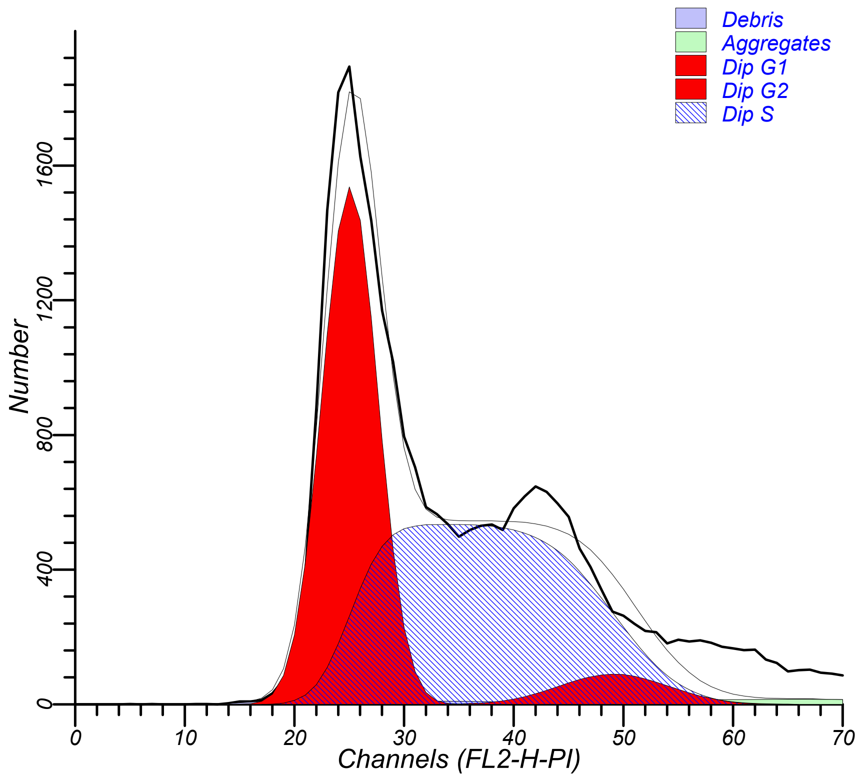

File analyzed: 20200711C.006
Date analyzed: 11-Jul-2020
Model: 1DA0n_DSD
Analysis type: Manual analysis

Ploidy Mode: First cycle is diploid

Diploid: 100.00 %
Dip G1: 41.04 % at 25.07
Dip G2: 4.70 % at 49.13
Dip S: 54.25 % G2/G1: 1.96
%CV: 10.02

Total S-Phase: 54.25 %
Total B.A.D.: 1.71 %

Debris: 1.71 %
Aggregates: 2.65 %
Modeled events: 24829
All cycle events: 23747
Cycle events per channel: 947
RCS: 15.521

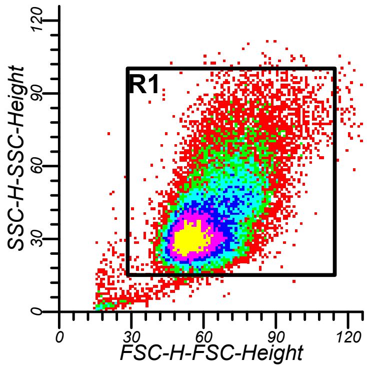

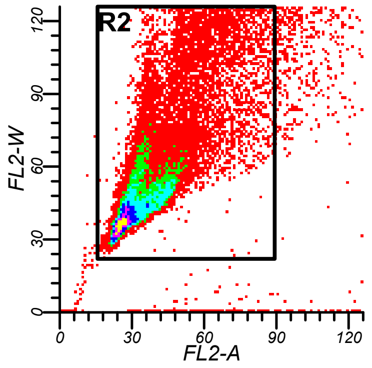

ModFitLT V3.1(Win32)
