# Supplementary material for: Downregulation of circLIFR exerts cancer-promoting effects on hepatocellular carcinoma in vitro
Source: Front Genet. 2022 Sep 12;13:986322. doi: 10.3389/fgene.2022.986322 (PMC9513674; doi:10.3389/fgene.2022.986322)

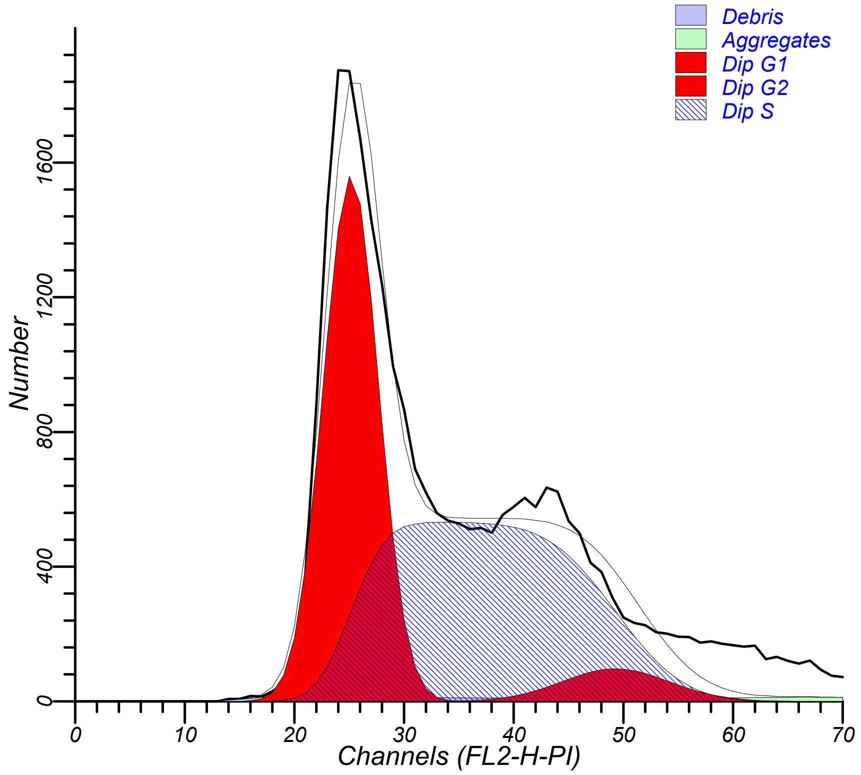

File analyzed: 20200711C.007
Date analyzed: 11-Jul-2020
Model: 1DA0n_DSD
Analysis type: Manual analysis

Ploidy Mode: First cycle is diploid

Diploid: 100.00 %
Dip G1: 41.16 % at 25.16
Dip G2: 4.98 % at 49.31
Dip S: 53.85 %   G2/G1: 1.96
%CV: 9.91

Total S-Phase: 53.85 %
Total B.A.D.: 1.88 %

Debris: 2.11 %
Aggregates: 2.16 %
Modeled events: 24951
All cycle events: 23887
Cycle events per channel: 950
RCS: 16.838

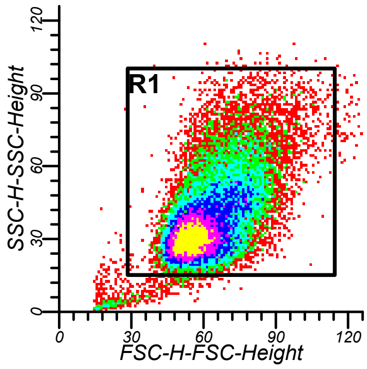

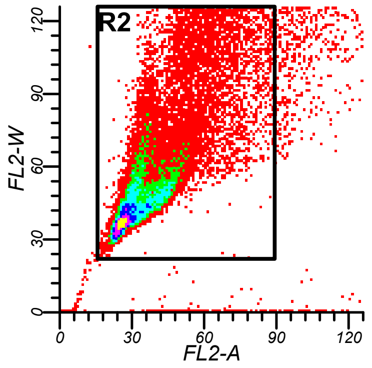

Supplement: Supplementary file 12 [file DataSheet2.ZIP › Cell function experiment/Cell cycle assay/hep-G2 cell/G2 C-2.pdf]
