# Supplementary material for: Downregulation of circLIFR exerts cancer-promoting effects on hepatocellular carcinoma in vitro
Source: Front Genet. 2022 Sep 12;13:986322. doi: 10.3389/fgene.2022.986322 (PMC9513674; doi:10.3389/fgene.2022.986322)
Supplement: Supplementary file 12 [file DataSheet2.ZIP › Cell function experiment/Cell cycle assay/hep-G2 cell/G2 C-3.pdf]

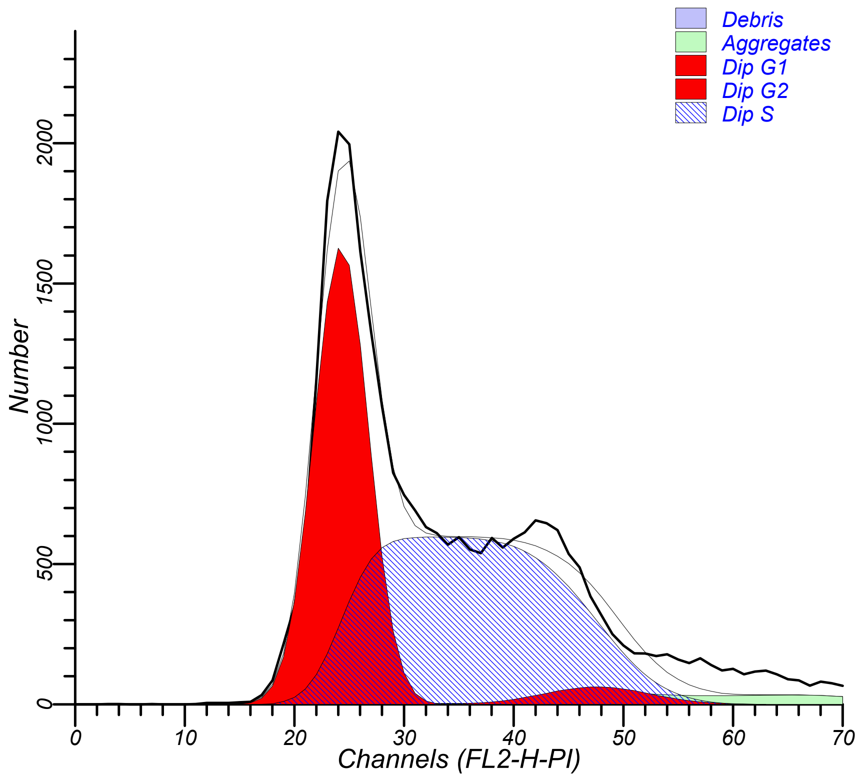

File analyzed: 20200711C.008
Date analyzed: 11-Jul-2020
Model: 1DA0n_DSD
Analysis type: Manual analysis

Ploidy Mode: First cycle is diploid

Diploid: 100.00 %
Dip G1: 40.87 % at 24.27
Dip G2: 3.05 % at 47.57
Dip S: 56.09 %   G2/G1: 1.96
%CV: 10.12

Total S-Phase: 56.09 %
Total B.A.D.: 0.95 %

Debris: 0.28 %
Aggregates: 5.36 %
Modeled events: 26301
All cycle events: 24818
Cycle events per channel: 1021
RCS: 12.098

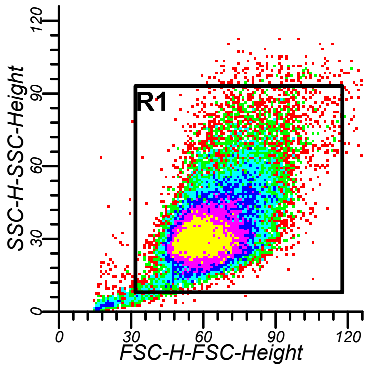

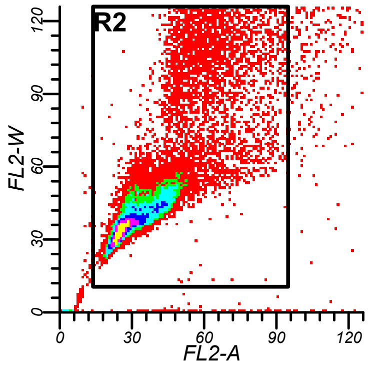

ModFitLT V3.1(Win32)
