# Supplementary material for: Downregulation of circLIFR exerts cancer-promoting effects on hepatocellular carcinoma in vitro
Source: Front Genet. 2022 Sep 12;13:986322. doi: 10.3389/fgene.2022.986322 (PMC9513674; doi:10.3389/fgene.2022.986322)
Supplement: Supplementary file 12 [file DataSheet2.ZIP › Cell function experiment/Cell cycle assay/hep-G2 cell/G2 cell-2.pdf]

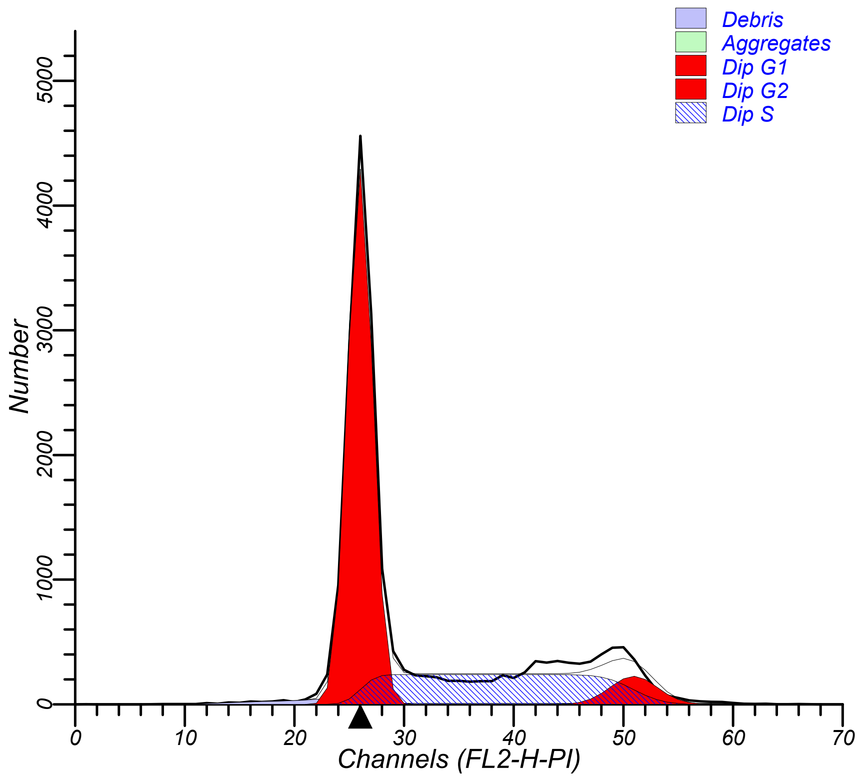

File analyzed: 20200711C.017
Date analyzed: 11-Jul-2020
Model: 1DA0n_DSD
Analysis type: Manual analysis

Ploidy Mode: First cycle is diploid

Diploid: 100.00 %
Dip G1: 62.94 % at 25.99
Dip G2: 6.32 % at 50.94
Dip S: 30.74 % G2/G1: 1.96
%CV: 4.21

Total S-Phase: 30.74 %
Total B.A.D.: 1.57 %

Debris: 3.28 %
Aggregates: 0.07 %
Modeled events: 20046
All cycle events: 19374
Cycle events per channel: 747
RCS: 4.648

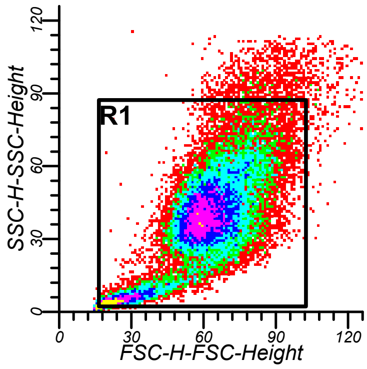

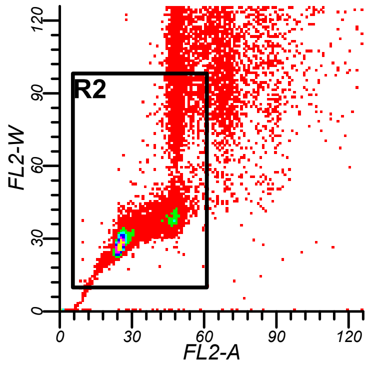

ModFitLT V3.1(Win32)
