# Supplementary material for: Downregulation of circLIFR exerts cancer-promoting effects on hepatocellular carcinoma in vitro
Source: Front Genet. 2022 Sep 12;13:986322. doi: 10.3389/fgene.2022.986322 (PMC9513674; doi:10.3389/fgene.2022.986322)

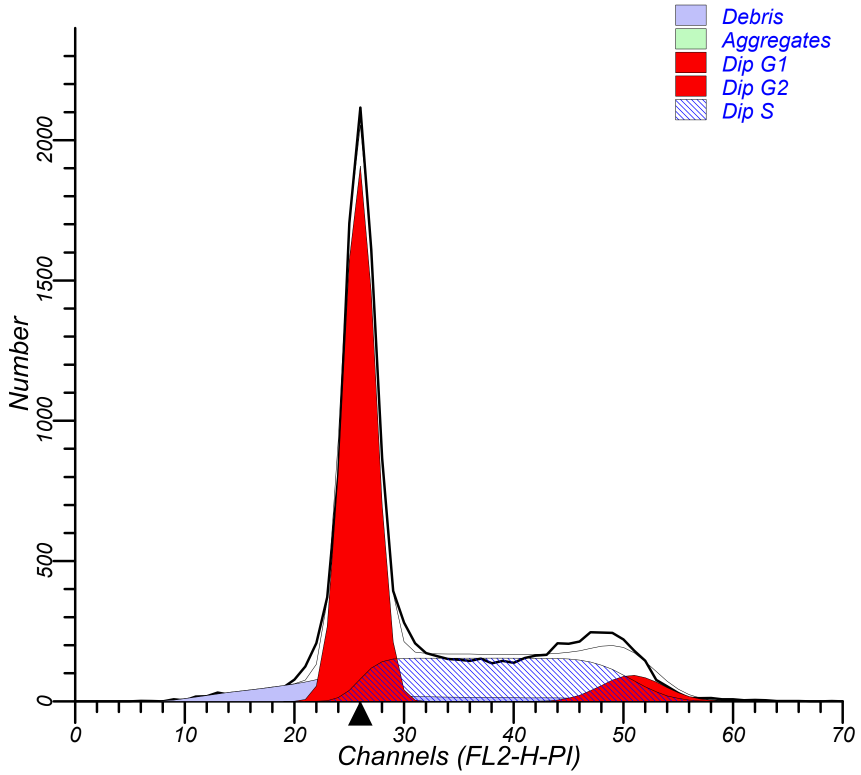

File analyzed: 20200711C.018
Date analyzed: 11-Jul-2020
Model: 1DA0n_DSD
Analysis type: Manual analysis

Ploidy Mode: First cycle is diploid

Diploid: 100.00 %
Dip G1: 61.09 % at 25.92
Dip G2: 5.74 % at 50.80
Dip S: 33.17 % G2/G1: 1.96
%CV: 5.57

Total S-Phase: 33.17 %
Total B.A.D.: 5.00 %

Debris: 9.88 %
Aggregates: 0.30 %
Modeled events: 12851
All cycle events: 11542
Cycle events per channel: 446
RCS: 4.412

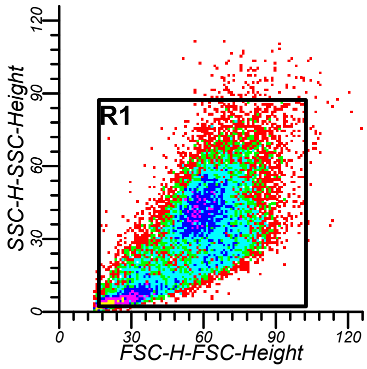

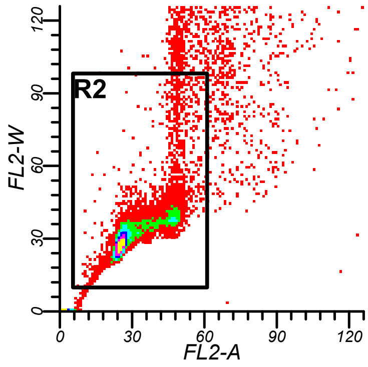

Supplement: Supplementary file 12 [file DataSheet2.ZIP › Cell function experiment/Cell cycle assay/hep-G2 cell/G2 cell-3.pdf]
