# Supplementary material for: Downregulation of circLIFR exerts cancer-promoting effects on hepatocellular carcinoma in vitro
Source: Front Genet. 2022 Sep 12;13:986322. doi: 10.3389/fgene.2022.986322 (PMC9513674; doi:10.3389/fgene.2022.986322)
Supplement: Supplementary file 12 [file DataSheet2.ZIP › Cell function experiment/Cell cycle assay/hep-G2 cell/G2 EP-3.pdf]

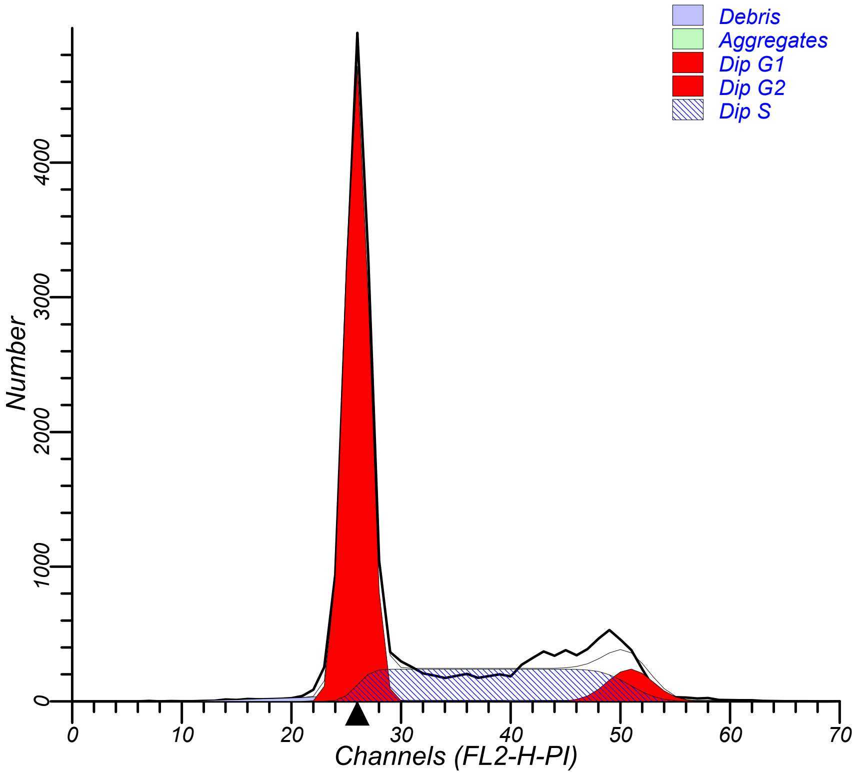

File analyzed: 20200711C.023
Date analyzed: 11-Jul-2020
Model: 1DA0n_DSD
Analysis type: Manual analysis

Ploidy Mode: First cycle is diploid

Diploid: 100.00 %
Dip G1: 63.92 % at 25.97
Dip G2: 6.24 % at 50.90
Dip S: 29.84 %   G2/G1: 1.96
%CV: 4.03

Total S-Phase: 29.84 %
Total B.A.D.: 1.21 %

Debris: 2.56 %
Aggregates: 0.09 %
Modeled events: 20637
All cycle events: 20091
Cycle events per channel: 775
RCS: 10.654

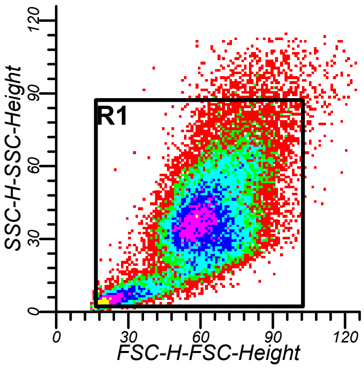

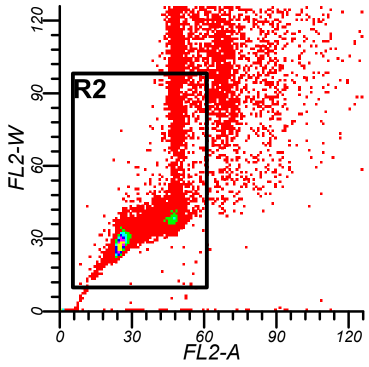

ModFitLT V3.1(Win32)
