# Supplementary material for: Downregulation of circLIFR exerts cancer-promoting effects on hepatocellular carcinoma in vitro
Source: Front Genet. 2022 Sep 12;13:986322. doi: 10.3389/fgene.2022.986322 (PMC9513674; doi:10.3389/fgene.2022.986322)
Supplement: Supplementary file 12 [file DataSheet2.ZIP › Cell function experiment/Cell cycle assay/SK-hep-1 cell/SK C 1.pdf]

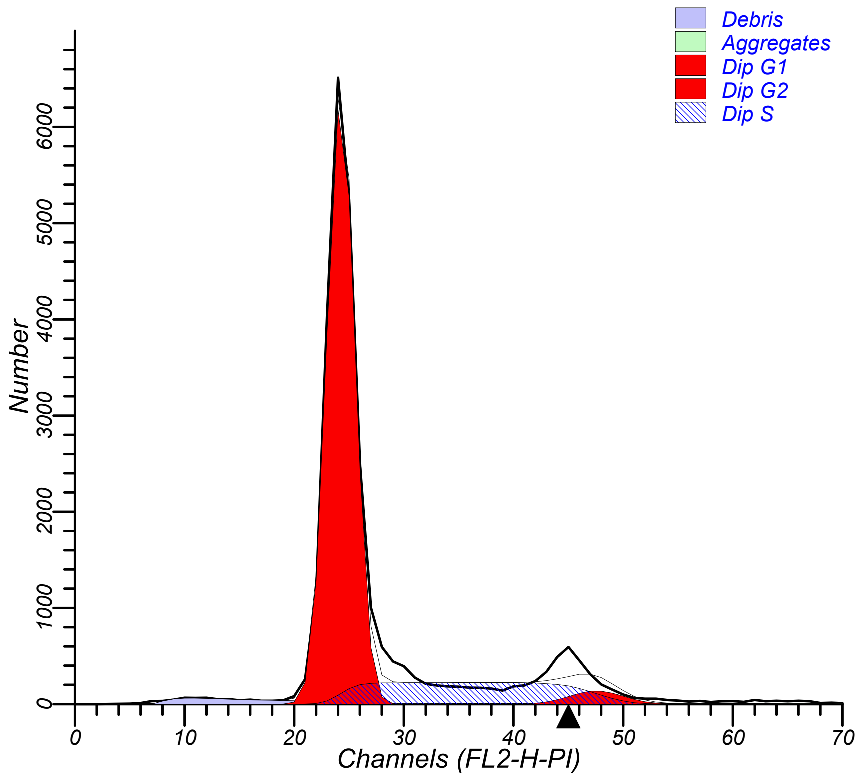

File analyzed: 20200711C.011
Date analyzed: 11-Jul-2020
Model: 1DA0n_DSD
Analysis type: Manual analysis

Ploidy Mode: First cycle is diploid

Diploid: 100.00 %
Dip G1: 76.89 % at 24.25
Dip G2: 3.25 % at 47.53
Dip S: 19.85 %   G2/G1: 1.96
%CV: 5.06

Total S-Phase: 19.85 %
Total B.A.D.: 1.29 %

Debris: 3.38 %
Aggregates: 1.40 %
Modeled events: 27204
All cycle events: 25902
Cycle events per channel: 1067
RCS: 14.933

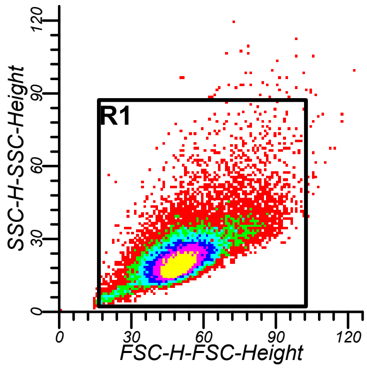

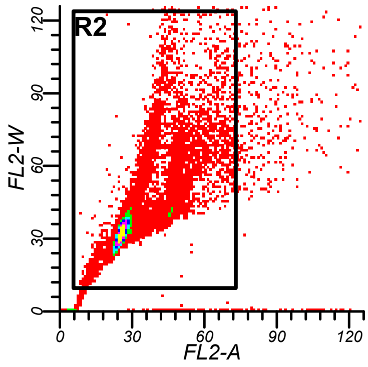

ModFitLT V3.1(Win32)
