# Supplementary material for: Downregulation of circLIFR exerts cancer-promoting effects on hepatocellular carcinoma in vitro
Source: Front Genet. 2022 Sep 12;13:986322. doi: 10.3389/fgene.2022.986322 (PMC9513674; doi:10.3389/fgene.2022.986322)
Supplement: Supplementary file 12 [file DataSheet2.ZIP › Cell function experiment/Cell cycle assay/SK-hep-1 cell/SK C 2.pdf]

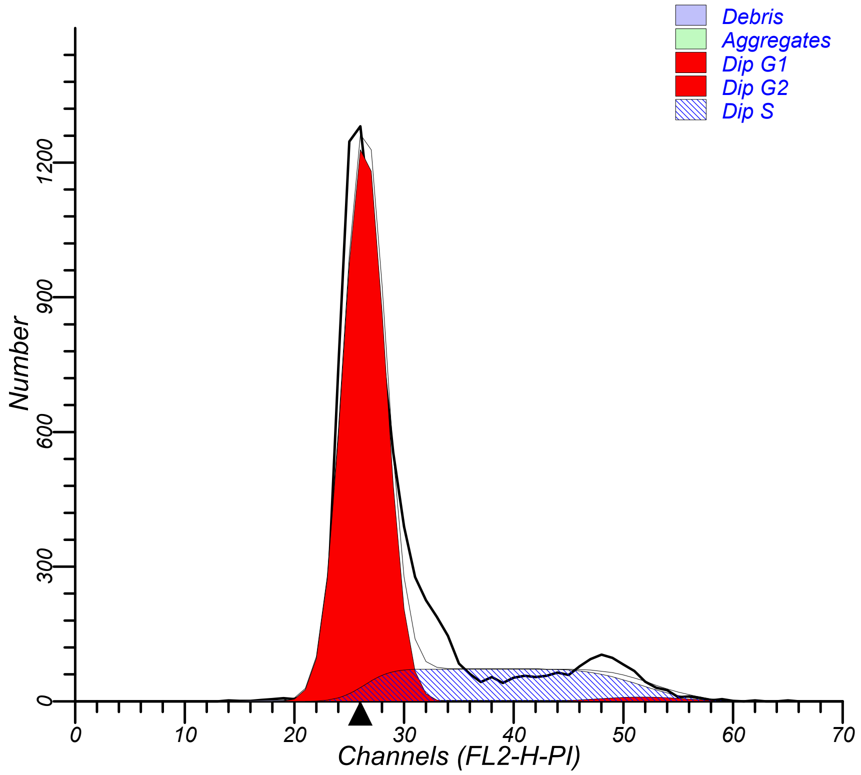

File analyzed: 20200711C.027
Date analyzed: 11-Jul-2020
Model: 1DA0n_DSD
Analysis type: Manual analysis

Ploidy Mode: First cycle is diploid

Diploid: 100.00 %
Dip G1: 75.79 % at 26.35
Dip G2: 1.20 % at 51.65
Dip S: 23.01 %   G2/G1: 1.96
%CV: 7.21

Total S-Phase: 23.01 %
Total B.A.D.: 0.21 %

Debris: 0.58 %
Aggregates: 0.15 %
Modeled events: 7999
All cycle events: 7940
Cycle events per channel: 302
RCS: 8.319

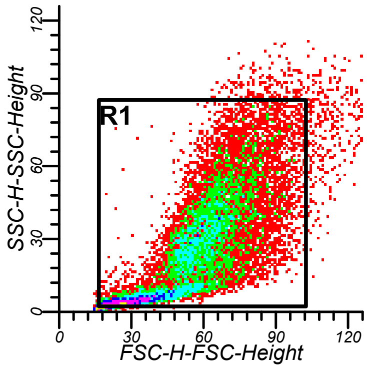

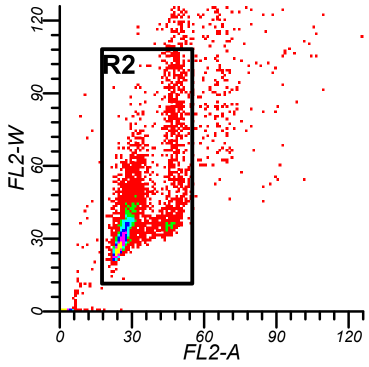

ModFitLT V3.1(Win32)
