# Supplementary material for: Downregulation of circLIFR exerts cancer-promoting effects on hepatocellular carcinoma in vitro
Source: Front Genet. 2022 Sep 12;13:986322. doi: 10.3389/fgene.2022.986322 (PMC9513674; doi:10.3389/fgene.2022.986322)
Supplement: Supplementary file 12 [file DataSheet2.ZIP › Cell function experiment/Cell cycle assay/SK-hep-1 cell/SK cell 2.pdf]

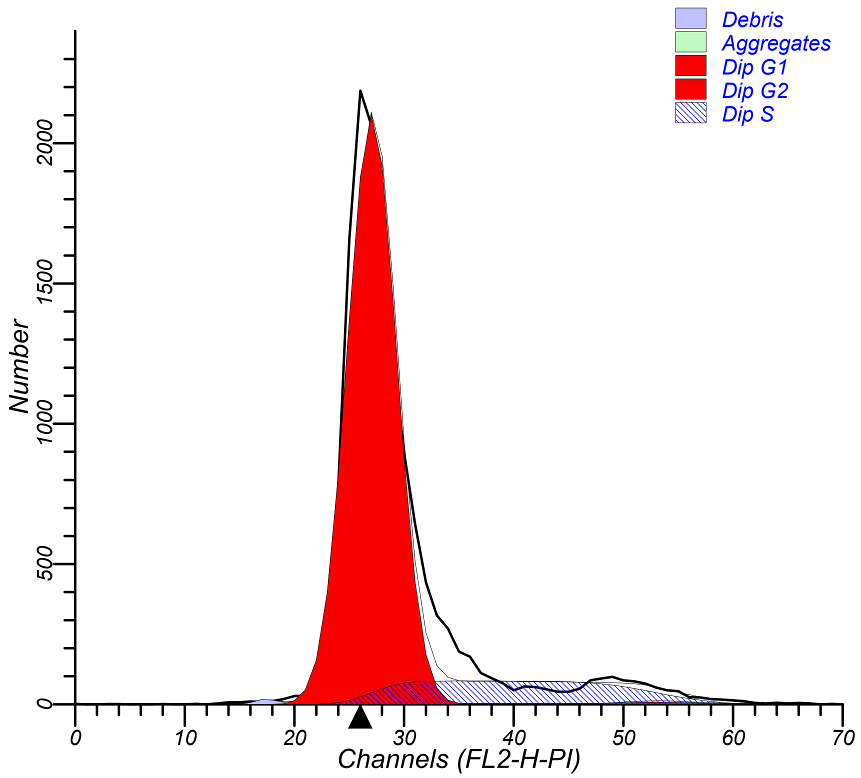

File analyzed: 20200711C.034
Date analyzed: 11-Jul-2020
Model: 1DA0n_DSD
Analysis type: Manual analysis

Ploidy Mode: First cycle is diploid

Diploid: 100.00 %
Dip G1: 83.89 % at 27.05
Dip G2: 0.60 % at 53.03
Dip S: 15.51 % G2/G1: 1.96
%CV: 8.16

Total S-Phase: 15.51 %
Total B.A.D.: 0.62 %

Debris: 0.00 %
Aggregates: 1.05 %
Modeled events: 14133
All cycle events: 13985
Cycle events per channel: 518
RCS: 10.295

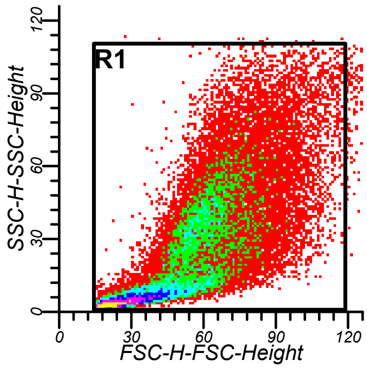

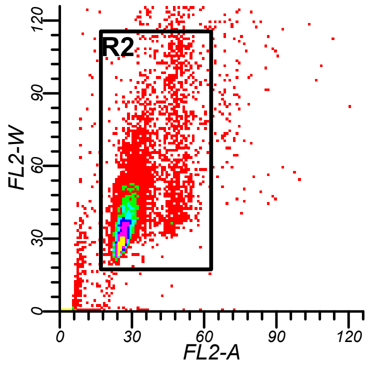

ModFitLT V3.1(Win32)
