# Supplementary material for: Downregulation of circLIFR exerts cancer-promoting effects on hepatocellular carcinoma in vitro
Source: Front Genet. 2022 Sep 12;13:986322. doi: 10.3389/fgene.2022.986322 (PMC9513674; doi:10.3389/fgene.2022.986322)
Supplement: Supplementary file 12 [file DataSheet2.ZIP › Cell function experiment/Cell cycle assay/SK-hep-1 cell/SK cell 3.pdf]

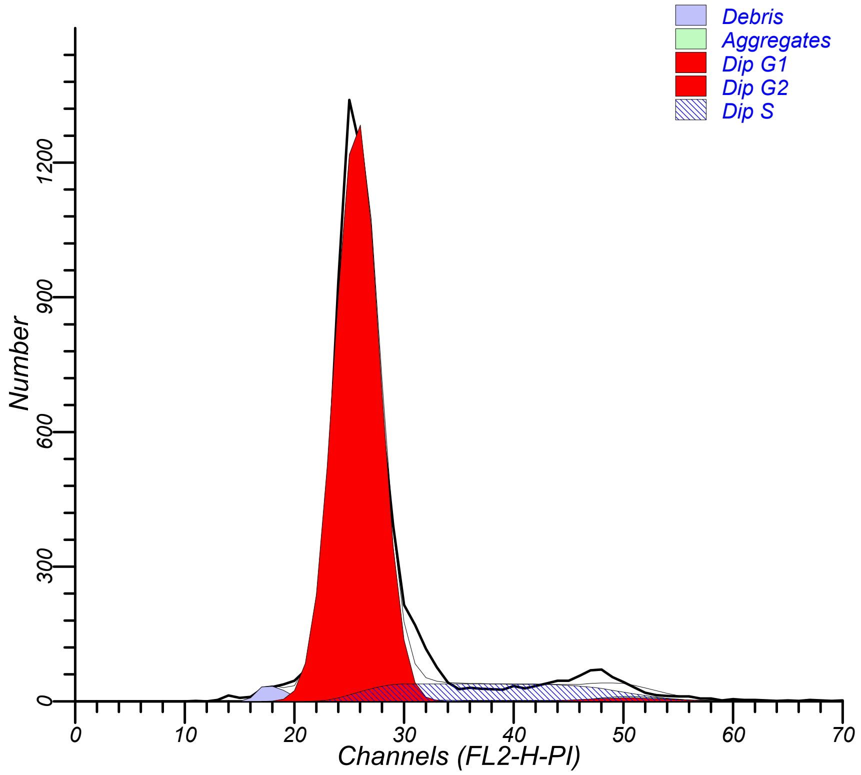

File analyzed: 20200711C.037
Date analyzed: 11-Jul-2020
Model: 1DA0n_DSD
Analysis type: Manual analysis

Ploidy Mode: First cycle is diploid

Diploid: 100.00 %
Dip G1: 86.26 % at 25.72
Dip G2: 1.03 % at 50.41
Dip S: 12.72 %   G2/G1: 1.96
%CV: 7.77

Total S-Phase: 12.72 %
Total B.A.D.: 0.99 %

Debris: 0.00 %
Aggregates: 1.44 %
Modeled events: 7724
All cycle events: 7613
Cycle events per channel: 296
RCS: 3.048

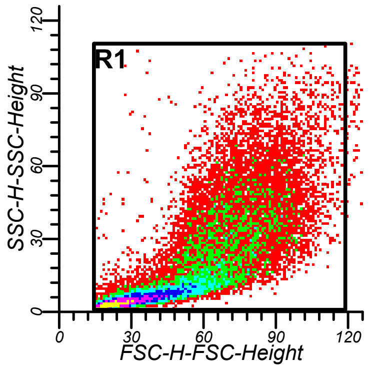

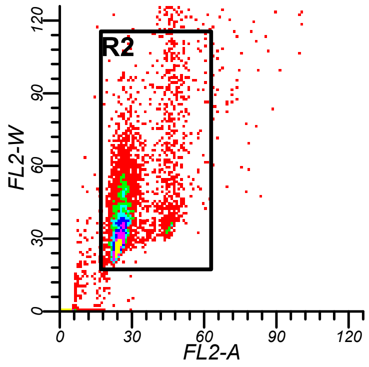

ModFitLT V3.1(Win32)
