# Supplementary figures and images for: Downregulation of circLIFR exerts cancer-promoting effects on hepatocellular carcinoma in vitro (part 4 of 4)
Source: Front Genet. 2022 Sep 12;13:986322. doi: 10.3389/fgene.2022.986322 (PMC9513674; doi:10.3389/fgene.2022.986322)

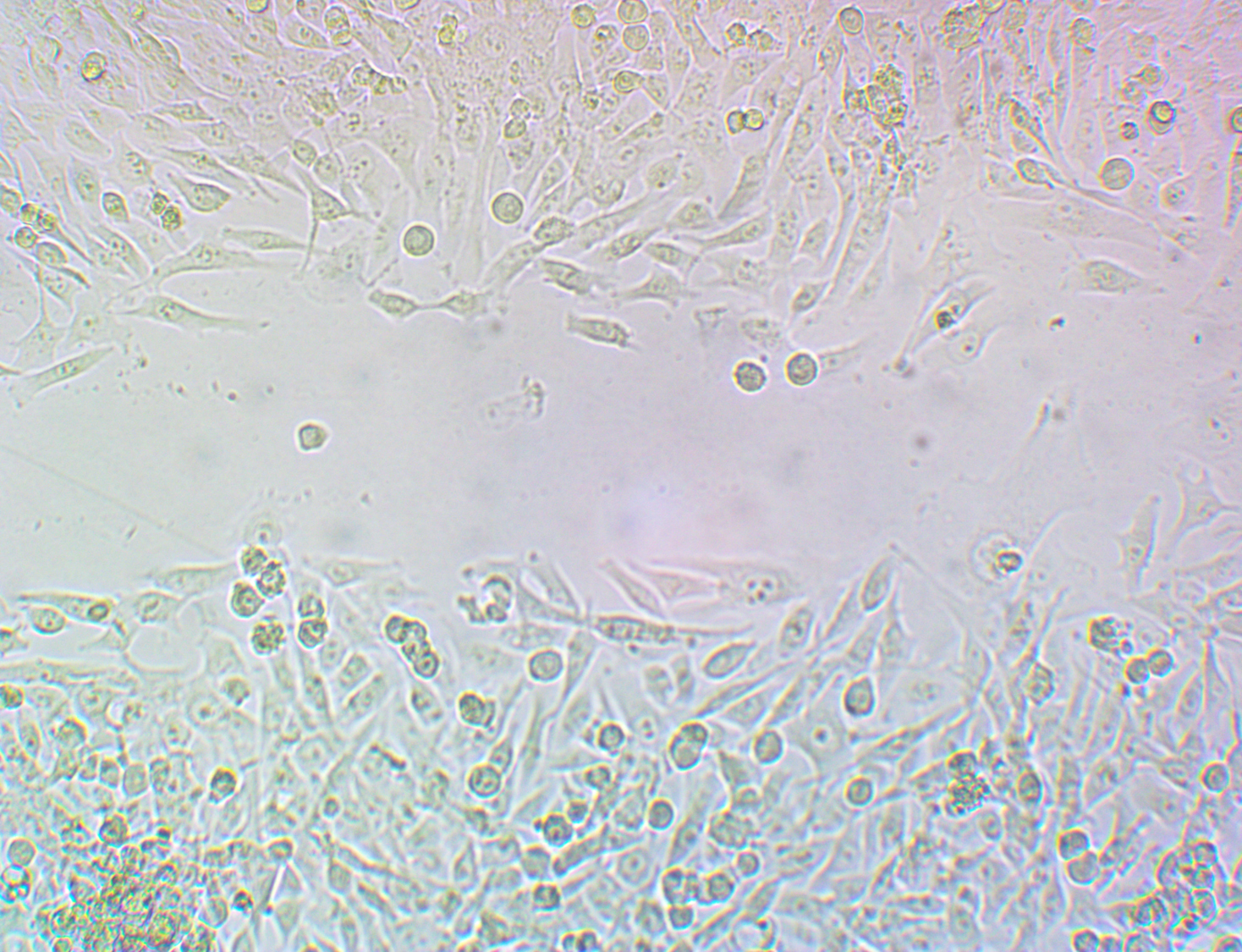

Supplement: Supplementary file 14 [file DataSheet7.ZIP › 24H/72309-24h-8 raw.jpg]

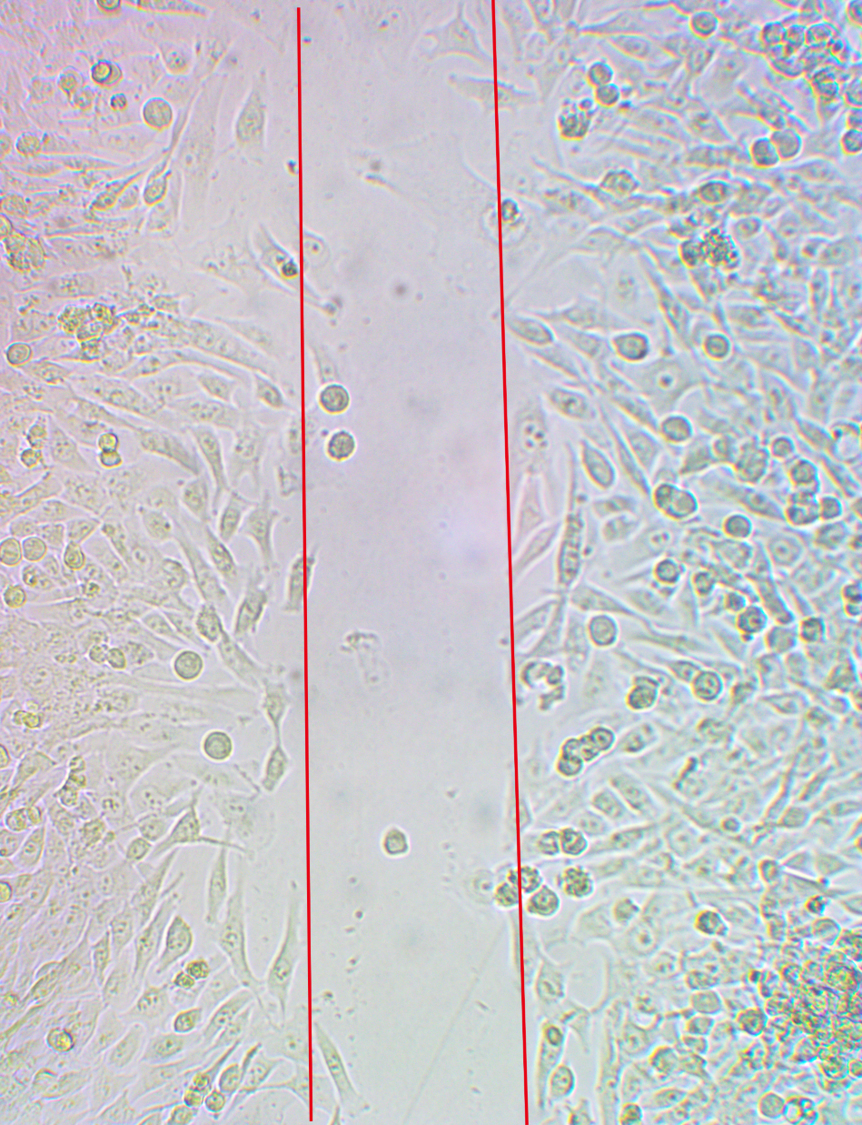

Supplement: Supplementary file 14 [file DataSheet7.ZIP › 24H/72309-24h-8.jpg]

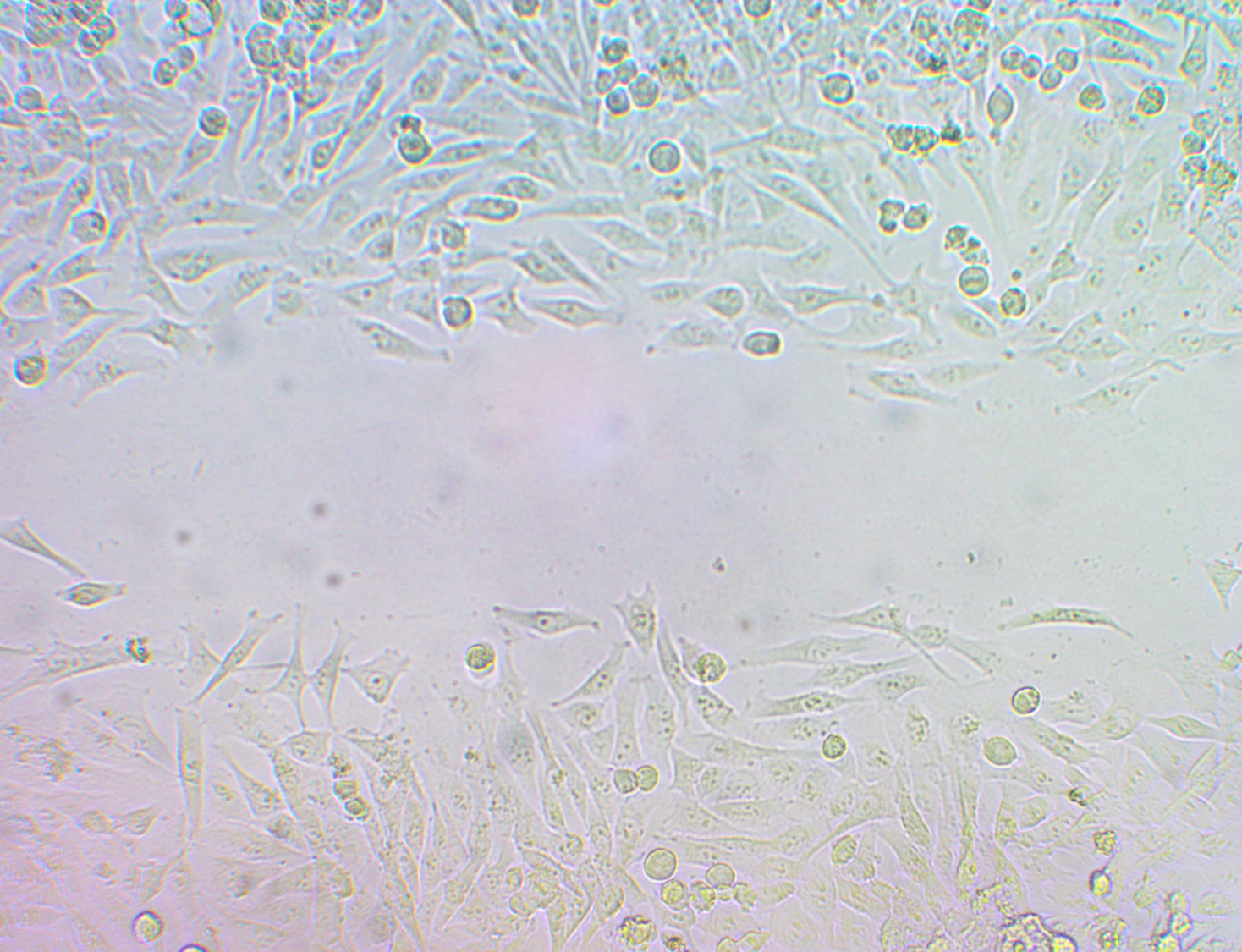

Supplement: Supplementary file 14 [file DataSheet7.ZIP › 24H/72309-24h-9 raw.jpg]

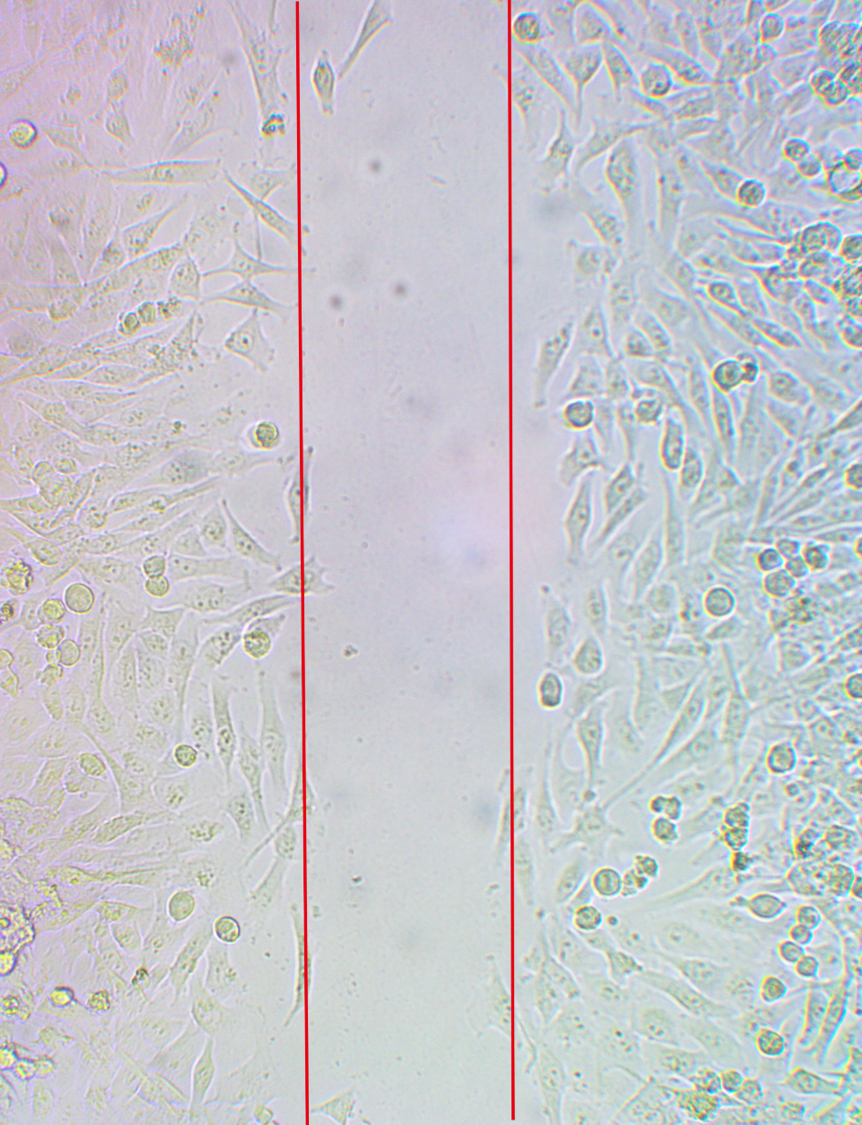

Supplement: Supplementary file 14 [file DataSheet7.ZIP › 24H/72309-24h-9.jpg]

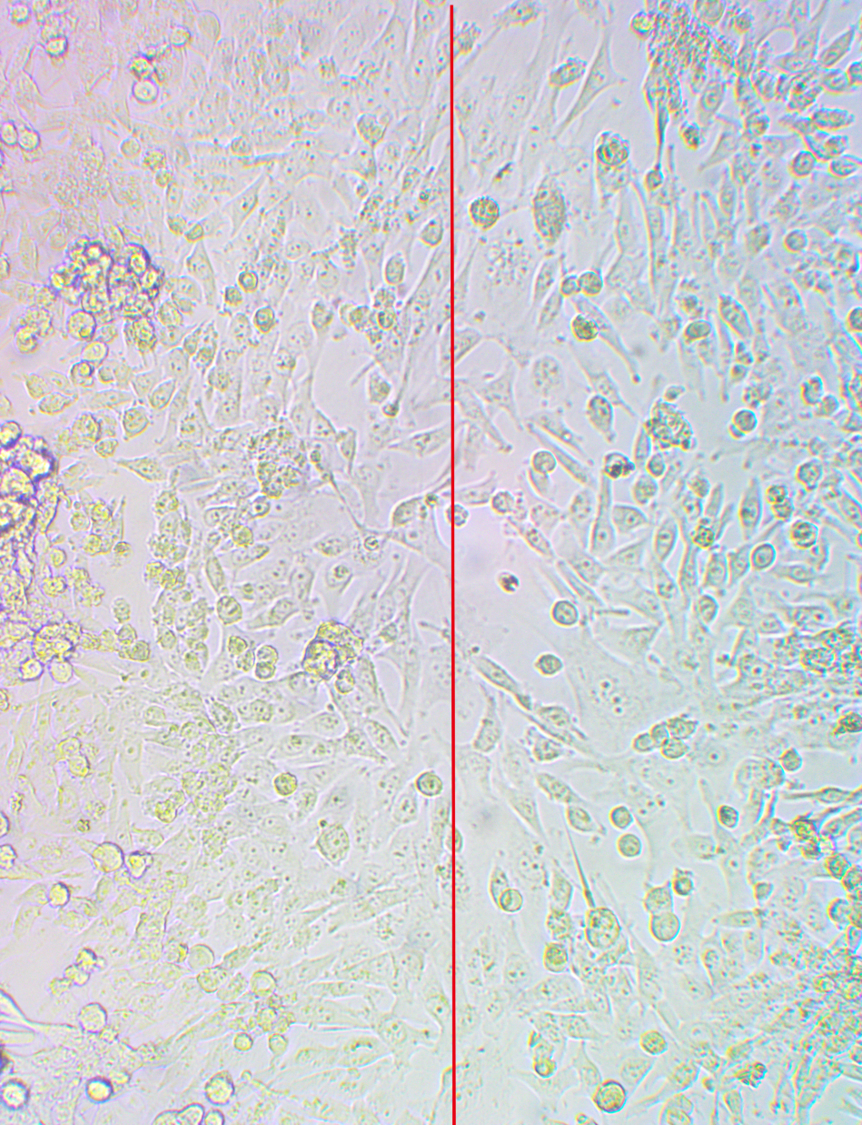

Supplement: Supplementary file 14 [file DataSheet7.ZIP › 48H/72309-48h 8.jpg]

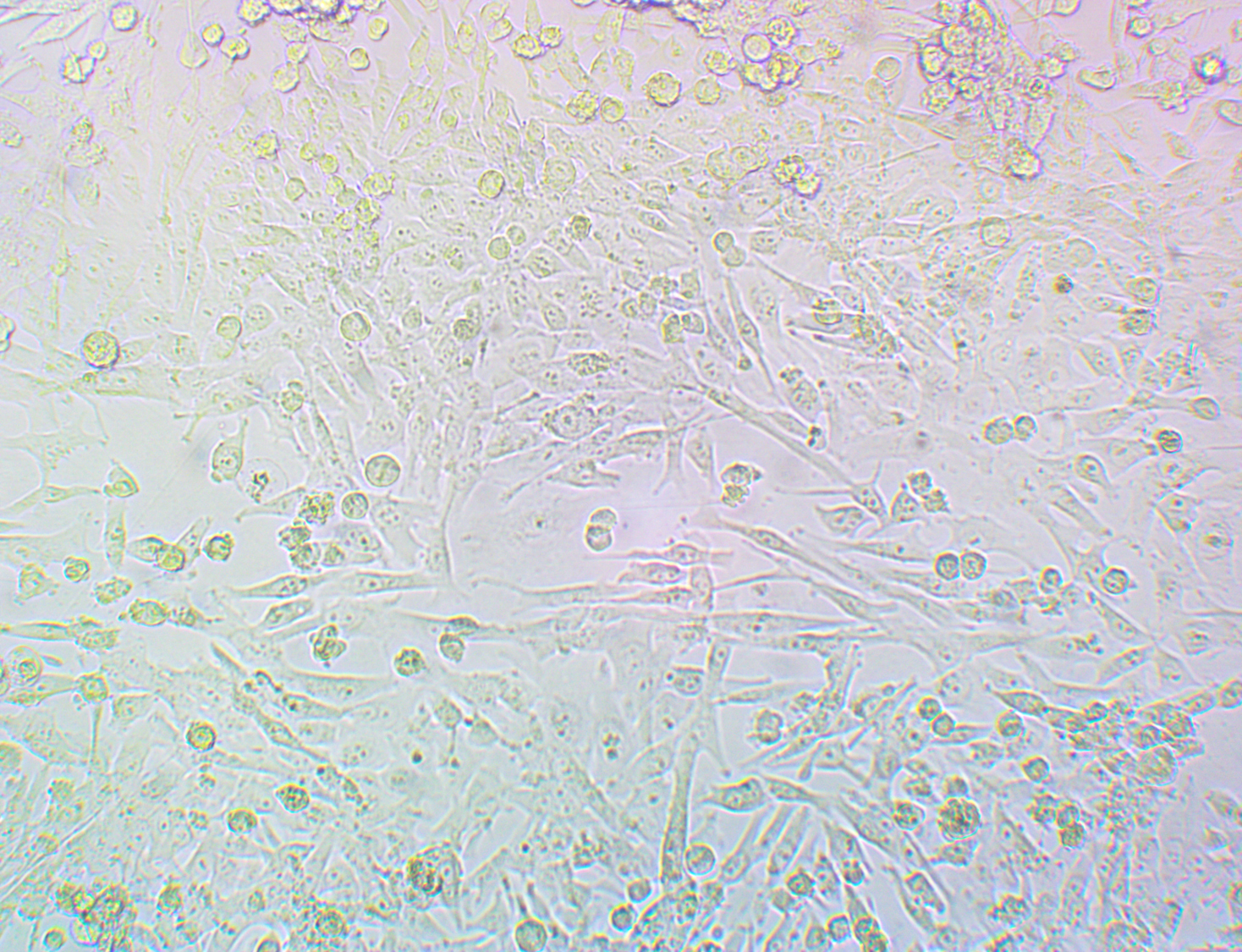

Supplement: Supplementary file 14 [file DataSheet7.ZIP › 48H/72309-48h-7 raw.jpg]

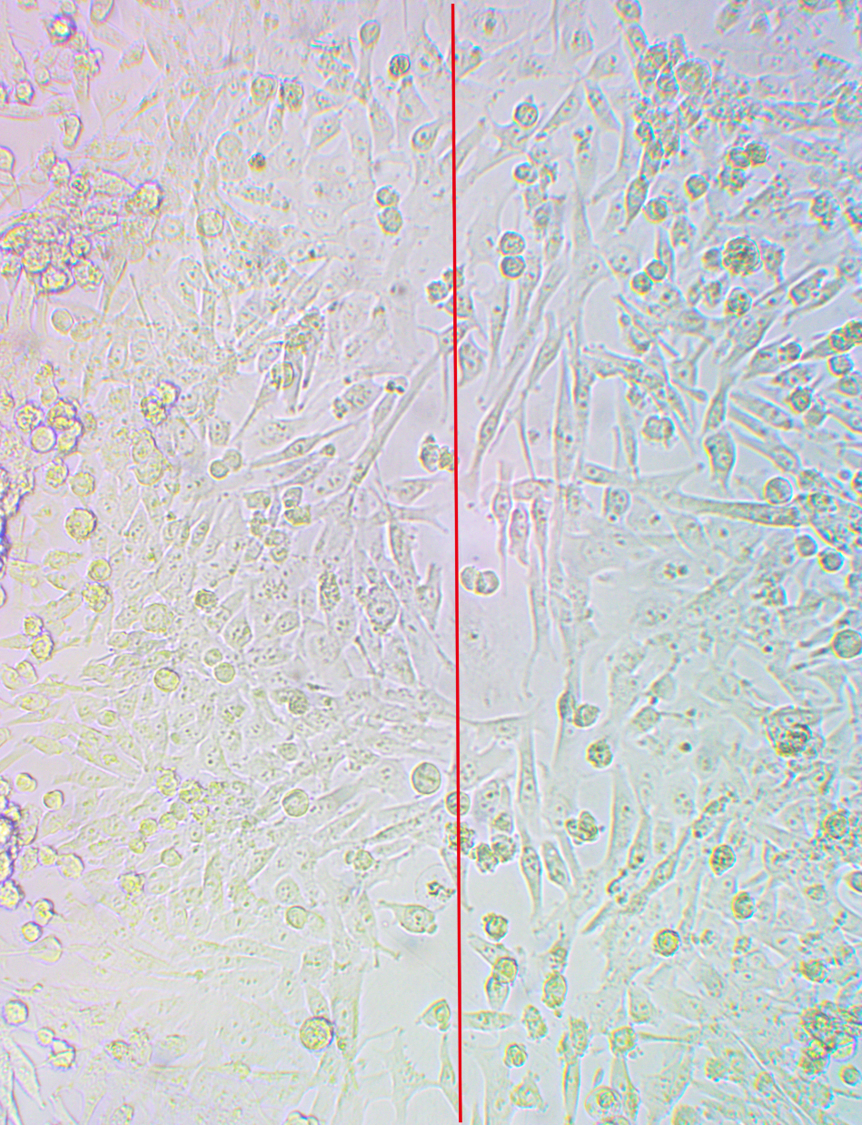

Supplement: Supplementary file 14 [file DataSheet7.ZIP › 48H/72309-48h-7.jpg]

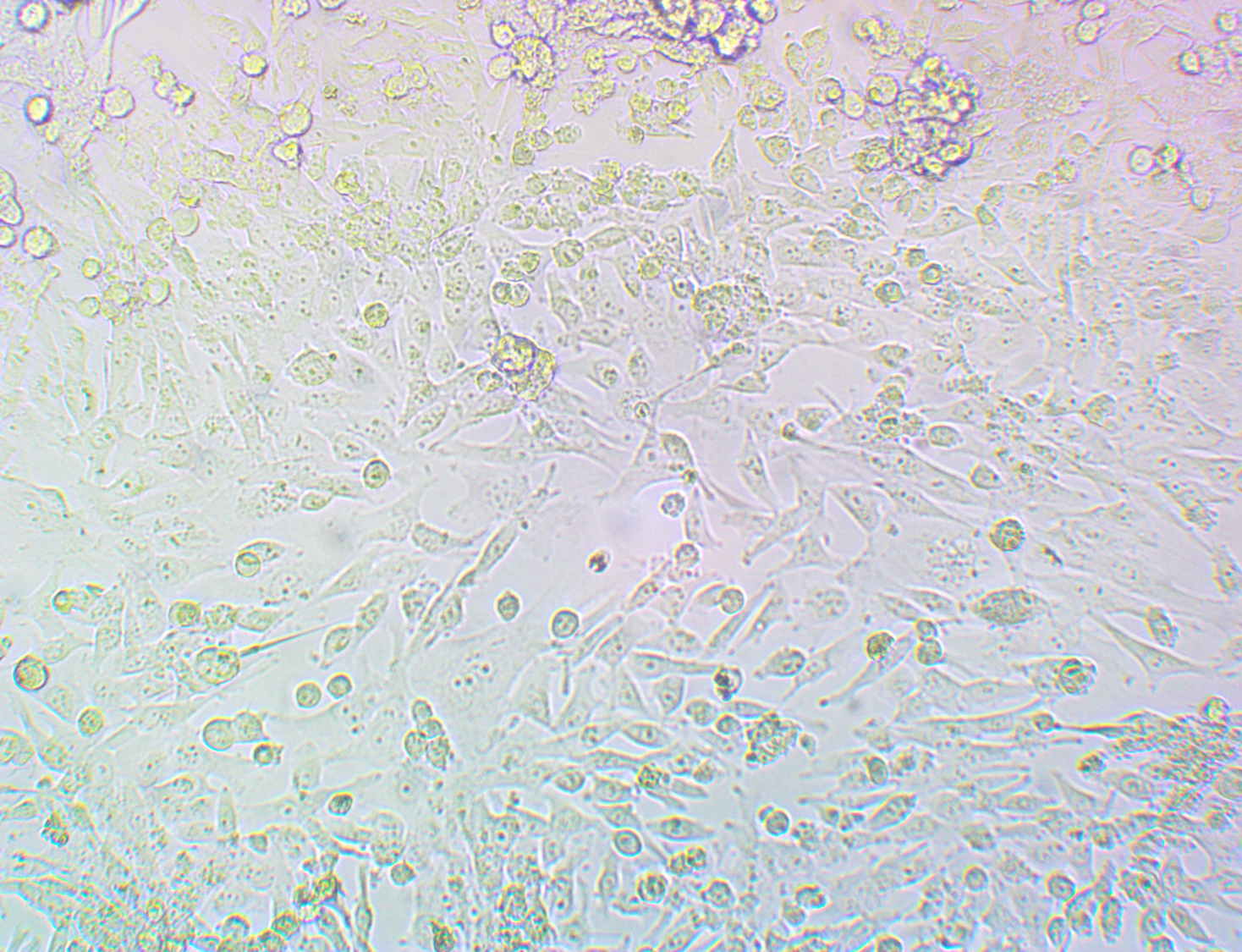

Supplement: Supplementary file 14 [file DataSheet7.ZIP › 48H/72309-48h-8 raw.jpg]

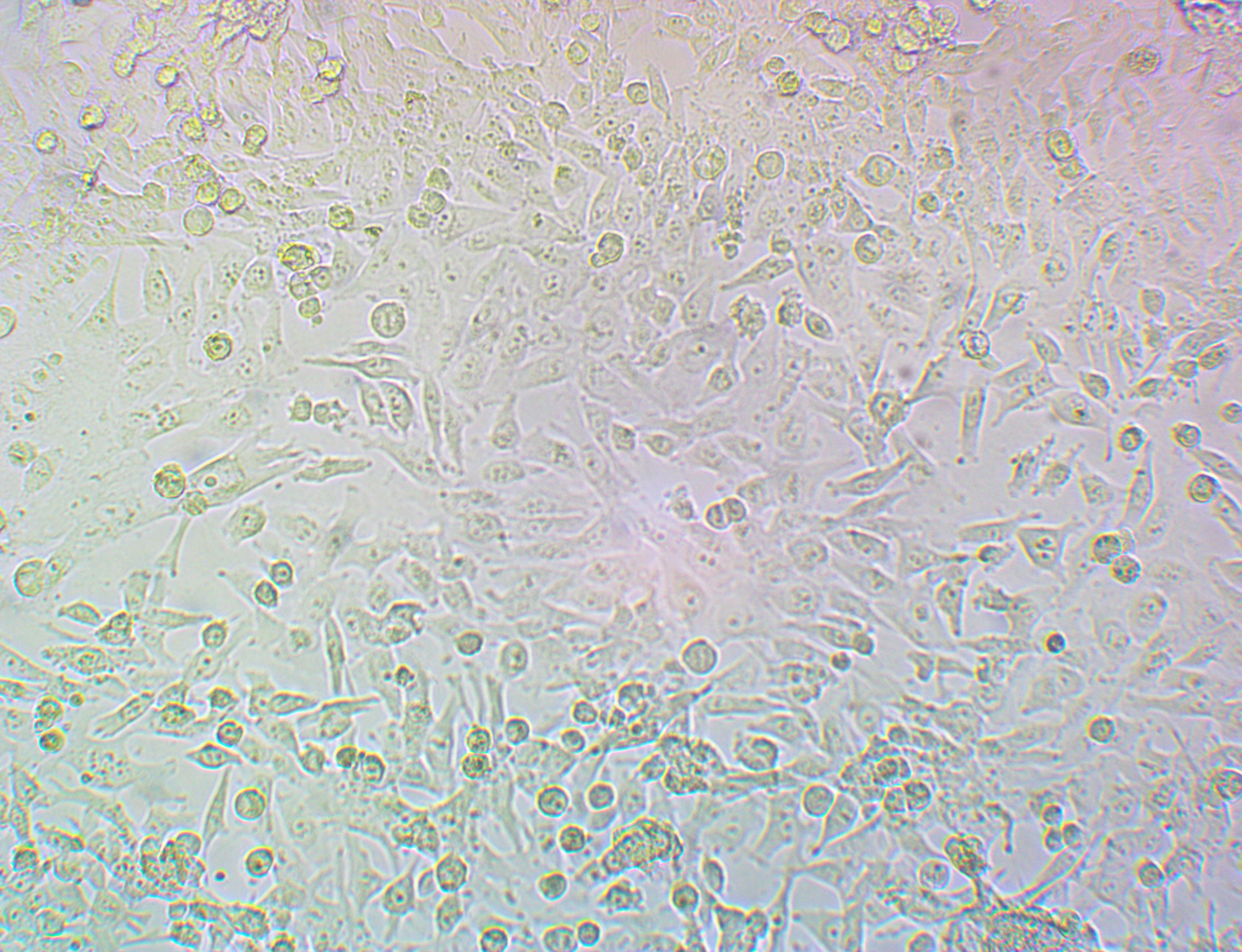

Supplement: Supplementary file 14 [file DataSheet7.ZIP › 48H/72309-48h-9 raw.jpg]

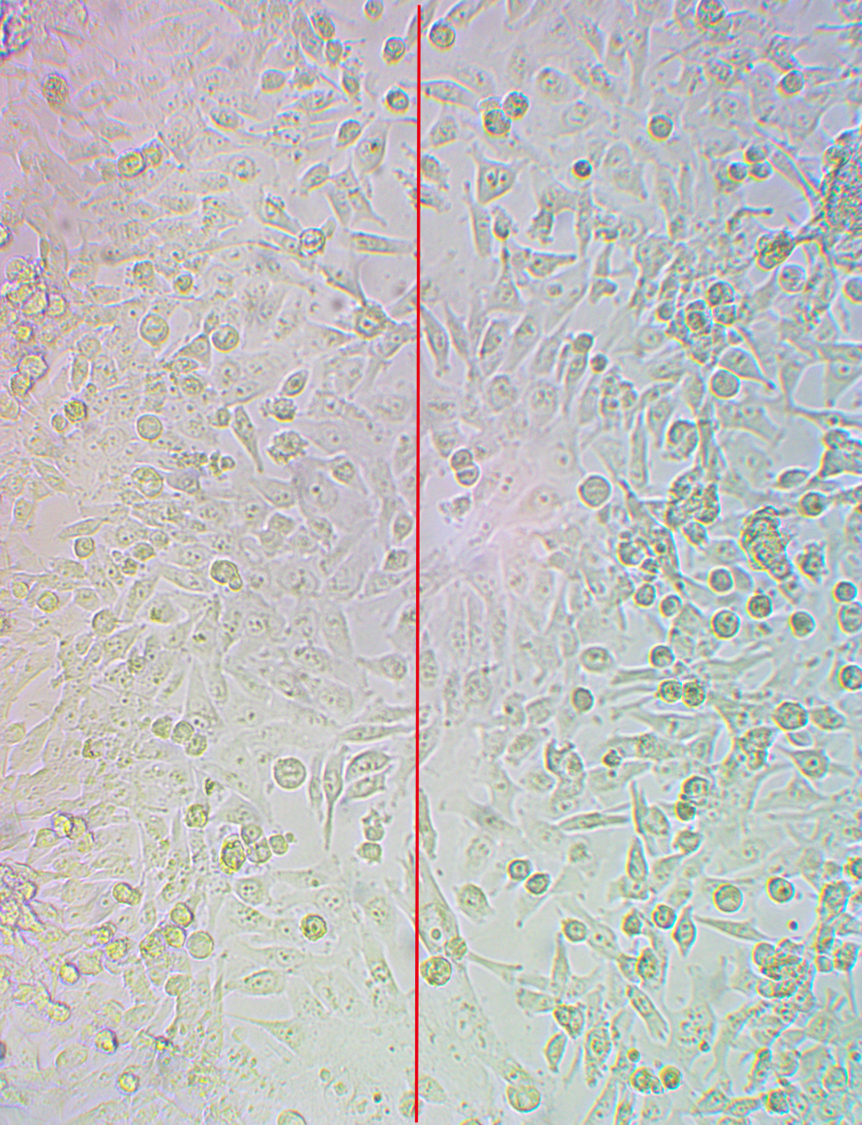

Supplement: Supplementary file 14 [file DataSheet7.ZIP › 48H/72309-48h-9.jpg]
